# Mutations Affecting HVO_1357 or HVO_2248 Cause Hypermotility in *Haloferax volcanii*, Suggesting Roles in Motility Regulation

**DOI:** 10.3390/genes12010058

**Published:** 2020-12-31

**Authors:** Michiyah Collins, Simisola Afolayan, Aime B. Igiraneza, Heather Schiller, Elise Krespan, Daniel P. Beiting, Mike Dyall-Smith, Friedhelm Pfeiffer, Mechthild Pohlschroder

**Affiliations:** 1 Department of Biology, School of Arts and Sciences, University of Pennsylvania, Philadelphia, PA 19104, USA; michiyah@sas.upenn.edu (M.C.); cmceearemu@yahoo.co.uk (S.A.); iaime@sas.upenn.edu (A.B.I.); hsrs@sas.upenn.edu (H.S.); 2 Department of Pathobiology, School of Veterinary Medicine, University of Pennsylvania, Philadelphia, PA 19104, USA; ekrespan@vet.upenn.edu (E.K.); beiting@vet.upenn.edu (D.P.B.); 3 Veterinary Biosciences, Faculty of Veterinary and Agricultural Sciences, University of Melbourne, Parkville 3010, Australia; mike.dyallsmith@gmail.com; 4 Computational Biology Group, Max-Planck-Institute of Biochemistry, 82152 Martinsried, Germany; fpf@biochem.mpg.de

**Keywords:** *Haloferax volcanii*, archaea, swimming motility, archaella, chemotaxis, hypermotility selection, transposon mutagenesis, two-component regulatory system, extremophiles

## Abstract

Motility regulation plays a key role in prokaryotic responses to environmental stimuli. Here, we used a motility screen and selection to isolate hypermotile *Haloferax volcanii* mutants from a transposon insertion library. Whole genome sequencing revealed that hypermotile mutants were predominantly affected in two genes that encode HVO_1357 and HVO_2248. Alterations of these genes comprised not only transposon insertions but also secondary genome alterations. HVO_1357 contains a domain that was previously identified in the regulation of bacteriorhodopsin transcription, as well as other domains frequently found in two-component regulatory systems. The genes adjacent to *hvo_1357* encode a sensor box histidine kinase and a response regulator, key players of a two-component regulatory system. None of the homologues of HVO_2248 have been characterized, nor does it contain any of the assigned InterPro domains. However, in a significant number of *Haloferax* species, the adjacent gene codes for a chemotaxis receptor/transducer. Our results provide a foundation for characterizing the root causes underlying *Hfx. volcanii* hypermotility.

## 1. Introduction

Although best known for species that thrive in extreme environments, archaea are actually ubiquitous, playing vital roles in a variety of ecological processes of global significance, including the carbon and nitrogen cycles [1,2,3]. The human microbiome also includes archaeal species, although their importance to human health is largely unknown [4,5].

Despite this importance and ubiquity of archaea, our understanding of key aspects of archaeal cell biology, including the regulation of swimming motility, remains superficial so far. Swimming motility allows cells to reach optimal conditions. Cells may swim toward energy sources (e.g., nutrients, light) but may also escape from repellents. Additionally, they may reach surfaces where they can form biofilms but can also quickly disperse from biofilms to reestablish a planktonic state. The swimming speed might depend on the need to escape an unfavorable condition.

Prokaryotic motility is frequently facilitated by a rotating filament, the flagellum in bacteria and the archaellum (archaeal flagellum) in archaea [6,7,8]. Although functionally equivalent, flagella and archaella are unrelated at the molecular level. While flagellar rotation is driven by an ion gradient [9], archaellar rotation is driven by ATP hydrolysis [10]. The archaellum is distantly related to type IV pili, including a similar pathway of N-terminal protein maturation by prepilin/prearchaellin peptidase [11,12,13,14,15]. A protein pair involved in archaella biogenesis (ArlI/ArlJ, previously FlaI/FlaJ) is distantly related to the type IV pili biogenesis proteins (PilB/PilC) [16,17,18]. ArlI, however, is bifunctional, as it is not only involved in archaellar biogenesis but also is an integral part of the archaellar motor [19,20]. The kinetics of the archaellar motor of *Halobacterium salinarum* have been analyzed in detail [21,22], and its step-wise rotation has been directly observed [23].

Directed prokaryotic motility depends on interaction with a (chemo)tactic system, which detects environmental signals by a set of receptors/transducers (Htr: Halobacterial transducer; also known as MCP, methyl-accepting chemotaxis protein) [24,25]. At the core of the signaling cascade is a two-component signal transduction system consisting of the histidine kinase CheA and the response regulator CheY [26,27,28,29,30,31]. The docking of the bacterial-type chemotactic system to the archaea-specific archaellar motor involves CheF and ArlCDE, as was recently resolved in the halophilic archaeon *Haloferax volcanii* [32,33,34]. Cells of *Hfx. volcanii* vary in shape depending upon growth conditions and can form pleomorphic disk-like forms as well as uniform rods. The tubulin homolog CetZ1 is required for rod formation and is essential for swimming motility [35]. To assist genetic analysis, a *Hfx. volcanii* transposon insertion library (Tn-library) has been constructed [36] that contains >4100 randomly introduced mutants in a genome with ca. 4000 protein-coding genes. The utility of this library in investigating motility has been previously demonstrated by the isolation of non-motile mutants, which revealed transposon insertions (Tn-insertions) between *cheW* and *cheB* as well as in *pibD*, *pilB1*, and genes not previously suggested to be involved in swimming motility [37,38].

Some archaeal mutant strains were also reported to be hypermotile, providing some additional molecular insights into the archaella biosynthesis, function, and regulation [19,39,40,41,42]. To obtain more insight into the phenomenon of hypermotility, we applied several screens to obtain hypermotile *Hfx. volcanii* isolates with a Tn-insertion. A large set of isolates was subjected to whole-genome sequencing to identify transposon insertion locations along with single nucleotide polymorphisms (SNPs), deletions, and other secondary genomic variations. Two genes, *hvo_1357* and *hvo_2248*, which do not show any obvious interconnection, were mutated in the majority of isolates.

## 2. Materials and Methods 

### 2.1. Strains and Chemicals

*Hfx. volcanii* transposon library mutants [36], as well as the wild-type strain H26 (Δ*pyrE* derivative of DS2) [43], were grown at 45 °C in a semi-defined *Hfx. volcanii* Casamino Acids (Hv-Cab) medium supplemented with uracil (50 μg mL^−1^ final concentration) [44]. Cells were cultivated in either a liquid medium (orbital shaker at 250 rpm) or on solid (1.5% agar) or semi-solid (0.35% agar) plates. Difco agar was purchased from Becton, Dickinson, and Company (Becton, Dickinson, and Company, Franklin Lakes, NJ, USA). Casamino Acids were purchased from Sigma (Sigma-Aldrich, Saint Louis, MO, USA). To ensure equal agar concentrations in all plates, the agar was completely dissolved in the media prior to autoclaving, and the autoclaved media were stirred before plates were poured. For genomic DNA extraction, the Thermo Scientific GeneJET Genomic DNA Purification kit (Thermo Fisher Scientific, Waltham, MA, USA) was used.

### 2.2. Screens for Hfx. volcanii Hypermotility Mutants by Stabbing Individual Tn-Mutants 

Single colonies of a *Hfx. volcanii* Tn-library were stabbed into 0.35% motility agar plates with toothpicks (24 or 50 stabs per plate). Plates were then placed on top of a damp paper towel in a plastic container with a loosely closed lid and incubated for two to four days at 45 °C. Cells from the edge of motility halos that were at least 1.3 times the radius of H26 (wild-type) were picked with a toothpick and streaked onto solid agar plates. Cells from single colonies of the streak were re-stabbed into new motility agar plates. Each motility agar plate was stabbed with two mutants and two wild-type samples. Mutants whose halos still formed a radius at least 1.3 times the size of that of the wild-type control (average of at least three replicates) were grown in a liquid medium for DNA isolation and sequencing.

### 2.3. Hypermotility Mutant Selection by Streaking Cells of the Tn-Library across the Center of a Motility Agar Plate and Isolation of Cells That Migrated the Farthest

The *Hfx. volcanii* Tn-library was amplified and the strains were pooled. A total of 15 μL of this pool (10^6^ cells/ml) was streaked in a straight line across a 0.35% motility agar plate. Motility agar plates were placed on top of a damp paper towel in a plastic container with a loosely closed lid and incubated at 45 °C for seven to eleven days. *Hfx. volcanii* Tn-insertion mutants from the outer edge of motility halos farthest away from the streak were picked with a toothpick and streaked onto solid agar plates. Cells from single colonies of the streak were re-stabbed into new motility agar plates to confirm hypermotility. Each motility agar plate was stabbed with two mutants and two wild-type samples. Mutants whose halos formed a radius at least 1.3 times the size of that of the wild-type control (average of at least four replicates) were grown in a liquid medium for DNA isolation and sequencing.

### 2.4. Genome Sequencing

Sequencing libraries were prepared from DNA extractions with the Nextera XT DNA Library Preparation Kit (Illumina, Catalog #FC-131-1096, San Diego, CA, USA). After quantification on Qubit, 1 ng of input was used for library preparation. Completed libraries were quantified on Qubit and assessed for quality on an Agilent 4200 Tapestation. Sequencing was performed on an Illumina MiniSeq with a Mid Output Kit and paired-end, 150 cycle reads.

### 2.5. Detection of Primary and Secondary Genome Alterations

For the detection of genome alterations, a reference genome sequence is required. This was created in silico in a multistep process based on genomic alterations to the available genome sequence for the wild-type strain DS2^T^ [45] (Figure S1). The type strain was cured for plasmid pHV2 [46], resulting in strain DS70. During curing, plasmid pHV4 was inadvertently integrated into the chromosome [47]. Strain DS70 was converted to the Δ*pyrE2* strain H26 [43]. This strain was subjected to deep sequencing, which led to the detection of a few additional SNPs [47]. The genome sequence of strain H26 was kindly provided by Thorsten Allers (University of Nottingham, UK). Strain H26 is the parent of strain H295 (Δ*rad50* Δ*mre11* Δ*trpA*) [48], and its construction is described in Figure S1. The additional mutations (Δ*rad50* Δ*mre11* and Δ*trpA*) introduced into the H26 genome sequence to obtain a reference sequence of H295 were as described in [48]. 

Illumina reads were imported into Geneious Prime, trimmed using BBDuk (default settings) from the BBTools package [49] and mapped to the genome sequence of the parental strain (strain H295) using the Bowtie2 mapper available within Geneious Prime. Tn-insertions were identified by searching for transposon-specific terminal sequences and the insertion point was noted as the first base differing from that of the genome. Other changes were identified by manual scanning of the assembly within Geneious.

During an initial analysis, we detected genome alterations which occurred in all strains: (a) An A>T mutation at pos 521,827 of strain H295 (357 nt from start of HVO_A0011 on the integrated plasmid pHV4; corresponds to pos 12,162 on pHV4). (b) For pHV3 (GenBank accession CP001953), we detected six distinct mutations, a G>A at pos 26,672 and five others which all occur in the gene encoding HVO_B0311 (G>C at pos 359,036, G insertion after pos 359,039, G insertion after pos 359,057, G insertion after pos 359,101, C insertion after pos 359,134). The regions affected by mutations match to those from resequencing of the *Hfx. volcanii* type strain genome (GenBank accession AOHU01000021) [50]. These general mutations were assumed to exist in the parental strain. They may have been introduced either during generation of the Tn-library or might be attributed to mutations that had occurred during serial passage of the parent strains in the laboratory, while generating strain H295. We updated the reference genome sequence (H295mod1) and analyzed for mutations compared to that genome version. FastA files with these reference sequences (chromosome and plasmid pHV3) are provided as Files S1 and S2, respectively.

Mutants were of two principle types: (a) Every mutant in a Tn-library needs to show a transposon integration site, which we refer to as primary mutation. (b) There may be additional genomic alterations, which are unpredicted and which we refer to as secondary mutations (Table 1). All primary and secondary mutations are listed in Table S1.

### 2.6. Bioinformatic Analyses

Ortholog analysis was performed using OrthoDB [51,52]. Gene synteny was analyzed with SyntTax [53], and by the “conserved neighborhood” option available at the DOE Joint Genome Institute (JGI) IMG/MER database [54,55]. The 3D structure prediction was attempted with Phyre2 [56].

## 3. Results and Discussion

The isolation of non-motile mutants from a *Hfx. volcanii* transposon insertion library has revealed that genes outside the core archaellum cluster may have a major impact on motility. The molecular basis of such regulatory components is still under study. In a complementary approach, using two distinct strategies, we set out to isolate hypermotile Tn-mutants, aiming to identify additional components required for archaella function or regulation. 

### 3.1. Screening for Hypermotility by Stabbing Individual Strains

**Figure 1:**
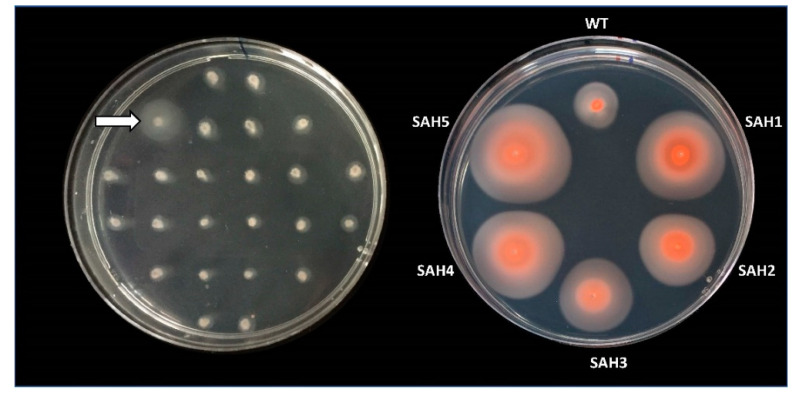
Hypermotility screen. Cells from *Hfx. volcanii* mutants of a transposon insertion library (Tn-library) were stabbed into a motility agar plate (0.35% agar) and incubated for 48 hours at 45 °C (left). To confirm the hypermotile phenotype of Tn-mutants with larger halos (e.g., marked by arrow), cells from the outer edge of the motility halo were re-streaked onto solid agar plates. Cells from individual colonies were then re-stabbed into motility agar plates and incubated for 90 hours at 45 °C (right). Isolates with a halo size radius at least 1.3 times that of the wild-type strain were considered hypermotile and were subjected to Illumina sequencing. Right image was taken after 10 days at room temperature. Halo size did not increase but they did become darker due to an increase in pigment production.

As a first screening method, we stabbed individual mutants from the Tn-library onto motility plates, incubated these for two to four days, and looked for colonies that had formed unusually large motility halos (Figure 1). 

Out of approximately 1250 stabbed Tn-mutants, five were hypermotile (SAH1-5). Whole genome Illumina sequencing revealed that each mutant had a distinct Tn-insertion site. Two mutants (SAH4, SAH5) had insertions in the same gene, *hvo_2248*, albeit at different positions (Table 1). Whole genome Illumina sequencing also uncovered that several mutants were affected by unanticipated, secondary genome alterations. Such secondary alterations were encountered in two of the five isolates (SAH3, SAH5) (Table 1 and Table S1). SAH5 lost a few spacers from a CRISPR array, which is unlikely to affect motility. In contrast, SAH3 lost two long sections of plasmid pHV4, totaling 230 kb, making it difficult to determine whether the hypermotility phenotype is due to the original transposon insertion (into the gene encoding HVO_0430) or due to the secondary genome alterations, which eliminate the genes for many proteins (HVO_A0014 to HVO_A0119 and HVO_A0279A to HVO_A0412).

HVO_1726 contains a helix-turn-helix (HTH) domain at the N-terminus and thus may function as a transcriptional regulator. The adjacent genes are one of the Orc1-type DNA replication protein paralogs and one of the TATA-binding transcription initiation factor paralogs. 

HVO_2176 has an assigned HalOD1 domain according to InterPro. This domain may be found in combination with a response regulator receiver domain [57]. HVO_2175, annotated as *sph3*, encodes a structural maintenance of chromosomes (SMC)-like protein. The SMC-like protein Sph1 from *Hbt. salinarum* (OE5212F, the ortholog of HVO_A0180) resulted in elongated rods when overproduced in its native host. When heterologously expressed in *Hfx. volcanii*, it leads to rod formation compared to predominantly pleomorphic disks in the non-transformed strain [58]. The other adjacent gene is *samp3*, which encodes a ubiquitin-like protein that is covalently attached to target proteins [59]. 

HVO_2248, which was also identified dominantly in an alternative screen (see below), belongs to the genomic dark matter as it lacks characterized homologs and has no assigned InterPro domains. It is predicted to be a cytoplasmic protein. As it was identified in all seven whole proteome datasets, which were reanalyzed within the Archaeal Proteome project, it is considered to be crucial under a variety of experimental conditions [60]. Homologs of HVO_2248 are only found in the taxonomic class Halobacteria and neither in other Euryarchaeota nor in other Archaea. It is encoded in only about one-third of the Halobacteria (66 of 166) according to orthoDB [51]. The genes in the vicinity of the *Hfx. volcanii* DS2 genome have no obvious relation to motility, and conservation of gene synteny is restricted to closely-related species. However, we note that the gene for a methyl-accepting chemotaxis protein (MCP, commonly referred to as Htr, halobacterial transducer protein) is intercalated right next to that encoding HVO_2248 in several *Haloferax* species, such as the ARA6 strain of *Haloferax gibbonsii*. We analyzed 25 genomes from the genus *Haloferax* and in half of them (12 of 25), an *htr* gene is present while no such gene is found in the other half (Figure S2). Outside the genus *Haloferax*, only a single genome was found to contain a *htr* gene in a similar arrangement, that of *Halogeometricum borinquense*, which, however, shows additional differences. The Phyre2 server could not predict a 3D structure model of high confidence. Thus, although over 1200 Tn-insertion mutants were screened, only three candidate genes (or gene regions) for hypermotility were identified: *hvo_1726* (SAH2), the region between *hvo_2175* and *hvo_2176* (SAH1), and *hvo_2248* (SAH4 and SAH5).

### 3.2. Selection for Hypermotility by Picking Cells That Moved Farthest from a Central Streak

**Figure 2:**
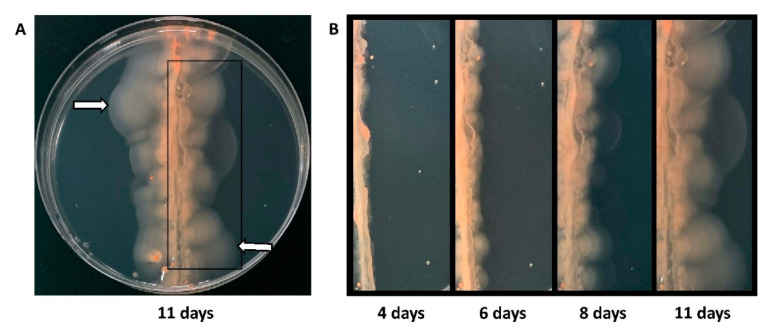
Selection of hypermotile isolates. (**A**) Pooled cells (15 μL, 10^6^ cells/ml) from the *Hfx. volcanii* Tn-library were streaked across a motility agar plate and incubated at 45 °C for eleven days. The white arrows mark examples of positions of halos farthest away from the streak from which cells were re-streaked onto solid agar plates. Cells from individual colonies were re-stabbed into motility agar plates. Isolates with a halo size radius at least 1.3 times that of the wild-type strain were considered hypermotile and were subjected to Illumina sequencing. (**B**) The progression of the motility halos over eleven days can be seen in the panels going from left to right: four days, six days, eight days, and eleven days after streaking.

In order to screen a significantly larger number of Tn-mutants in a short period of time, we designed a different method, which involves auto-selection of hypermotile cells. We streaked a pool of strains from the amplified Tn-library across the motility agar plate and picked cells that had moved farthest from the initial streak (Figure 2). Upon re-streaking cells from the edge of the halos on solid agar plates, single colonies were isolated and analyzed for hypermotility.

It should be noted that each Tn-mutant will be represented multiple times in the pool that is streaked, and thus such a mutant may be isolated multiple times during this screen. However, multiple isolations of the same Tn-mutant, taken from well-separated positions of the agar plate, should occur only if this specific mutation leads to an especially strong manifestation of the phenotype, which is selected for (Figure S3). Of 54 samples isolated from the screen, 49 retained hypermotility after clonal isolation. The sequence data returned for three strains were insufficient for analysis, and the genetic changes of the remaining 46 mutants were determined and are summarized in Table 1 and Table S1. They represent 13 distinct transposon insertion sites, some of which have been isolated several times (one Tn-mutant 13 times, one Tn-mutant 10 times, one Tn-mutant eight times). However, even in those cases where the transposon was found inserted at the same position and thus is likely to represent the same original mutant, distinct secondary genome alterations were encountered in some cases, which confirms the independence of their isolation, especially when picked from distinct plates. The secondary alterations might have occurred during the growth from the initial streak position to the final location where the isolate was picked. Alternatively, the streaked transposon library might have already been heterogeneous due to its amplification. Several isolates had not only the same Tn-insertion site, but also identical secondary genome alterations, as shown in Table 1 and Table S1.

HVO_2248 was very prominently identified in this screen, with three distinct Tn-insertion sites within the gene or immediately upstream and a total of 20 isolates. For several Tn-insertion sites, the mutant was independently selected several times (10 times; eight times; twice) (Table 1). The two transposon insertion sites within *hvo_2248* that had already been identified in the stabbing screen (SAH4, SAH5) were re-isolated in this independent screen.

Many of the isolates carried additional secondary genome alterations, among those being mutations in *hvo_1357* (see below). On the other hand, many mutants that had a Tn-insertion into other genes also carried a mutation of *hvo_2248* as a secondary genome alteration. However, because several of the isolates with the Tn-insertion into *hvo_2248* lack any secondary genome alteration, we consider this gene to be directly responsible for the hypermotility phenotype. 

**Figure 3:**
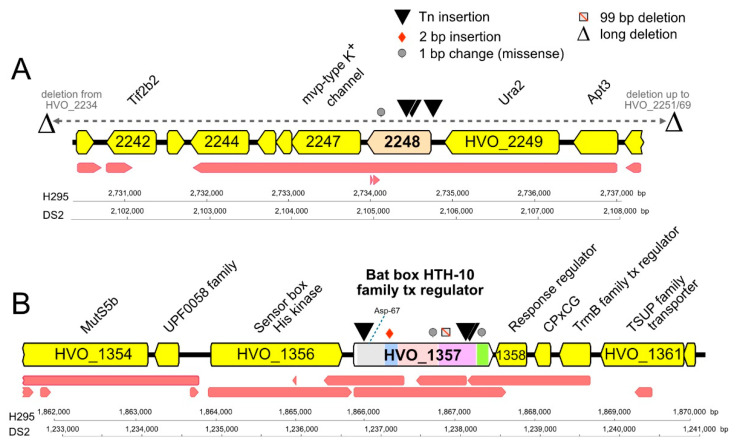
Mutations, domains, and genomic neighborhood of HVO_2248 and HVO_1357. Tn-insertions and other mutations detected in the chromosomal genes coding for HVO_2248 (**A**) and HVO_1357 (**B**) of hypermotile *Hfx. volcanii* mutants. The types and positions of mutations are indicated by symbols above HVO_2248 and HVO_1357 (key at top right). Protein product names are given above gene arrows, and locus_tag numbers are shown within arrows (often without prefix HVO for space reasons). The transcript map reported by [63] is represented by red arrows below each gene map. Scale bars and nucleotide numbering for strains H295 and DS2 are shown below each map. Panel A. HVO_2248 and its immediate gene neighborhood. Ura2, xanthine/uracil permease family transport protein; Apt3, purine phosphoribosyltransferase (adenine phosphoribosyltransferase, xanthine-guanine phosphoribosyltransferase); Tif2b2, translation initiation factor aIF-2 beta subunit/probable RNA-binding protein. Panel B. HVO_1357 and its immediate gene neighborhood. MutS5b, DNA mismatch repair protein MutS; response regulator, receiver box response regulator; CPxCG, small CPxCG-related zinc finger protein; tx, transcription. Colored regions within HVO_1357 denote conserved protein domains: grey, signal transduction response regulator (IPR001789); light blue, HalX (IPR013971); pink, GAF (IPR029016); purple, BAT (IPR031803); green, HTH-10 (IPR007050). The position of the conserved phosphorylatable Asp-67 within the receiver domain is indicated by a blue dotted line.

The other gene prominently retrieved was *hvo_1357*, with a total of 19 isolates representing three Tn-insertion sites that were isolated multiple times (13 times; four times; twice) (Table 1). HVO_1357 and the proteins encoded by its genomic neighbors (HVO_1356 and HVO_1358) belong to an extended two-component regulatory system that probably regulates genomic gene expression (Figure 3). HVO_1356 is a sensor box histidine kinase that also has a GAF and a PAS domain. HVO_1358 is a receiver box response regulator. HVO_1357 itself is a multidomain protein; it has a receiver domain at the N-terminus, with the phosphorylatable aspartate (Asp) residue being conserved (Asp-67). It also has a Bat-type HTH domain (HTH-10) at its C-terminus. This HTH domain subtype is known from the *Hbt. salinarum bat* gene (OE3101R), which activates the *bop* gene that encodes bacteriorhodopsin [61,62]. HVO_1357 carries an additional Bat-specific domain (IPR031803), a GAF domain (IPR029016), and a HalX domain (IPR013971). Thus, HVO_1357 is likely part of a signal-dependent gene regulation cascade and may be directly involved in the transcriptional regulation of target genes. As several of the isolates with the Tn-insertion into *hvo_1357* lack any secondary genome alteration, we consider this gene to be directly responsible for the hypermotility phenotype.

Six other isolates had a Tn-insertion into distinct genes, but in each case, *hvo_2248* was affected by a secondary genome alteration. Only one isolate was completely independent of these two genes and had a Tn-insertion into *hvo_2269* but additionally lacked two long regions, totaling to 230 kb, from the integrated plasmid pHV4 (the same regions deleted as in SAH3), and thus was not further analyzed. Thus, in summary, 45 of the 46 mutants had either a mutation associated with HVO_1357 (19) or HVO_2248 (25), and five had both. Of the five mutants that had mutations in both HVO_1357 and HVO_2248, none showed a further increased hypermotility phenotype compared to mutants with just one of the genes mutated. HVO_2248 and HVO_1357 appear to be critical in direct or indirect motility regulation and it is very possible that they interact with each other in this process.

## 4. Conclusions

Using distinct screening and selection procedures, we identified a large number of hypermotile isolates derived from a transposon insertion library. The selection especially allowed for the rapid isolation of many hypermotile isolates. Whole-genome Illiumina sequencing uncovered that many, but not all, of the isolates carried secondary genome alterations, underscoring the importance of extending mutant analysis to whole-genome sequencing. Our results reveal that the genes encoding HVO_1357 and HVO_2248 were affected in nearly all hypermotile isolates, either alone or in combination. While HVO_1357 is likely participating in a two-component regulatory system, probably acting as a transcriptional regulator, HVO_2248 is part of the genomic dark matter, with as yet no InterPro domain assigned and no clues from 3D structure prediction nor gene synteny analysis, but a subtle link to chemotaxis due to an *htr* gene in the immediate vicinity of about half of the genomes from the genus *Haloferax*. Nevertheless, the strong overrepresentation of these two genes in a large set of independently obtained mutants highlights the likely importance of both genes in *Hfx. volcanii* hypermotility. Thus, we have opened the road to detailed experimental analyses and a deep understanding of this process.

## Figures and Tables

**Table 1:** Genome alterations in hypermotile *Hfx. volcanii* Tn-isolates.

		Affected Genes ^1^			
Tn-Insertion Position ^2^	Total Count	Primary	Secondary	Count	Isolates ^3^
2,734,510	3	HVO_2248	HVO_1357	1	MC3
		HVO_2248	-	1	MC50
		HVO_2248	yes	1	SAH4
2,734,434	9	HVO_2248	HVO_1357	1	MC14
		HVO_2248	-	6	SAH5, MC9, 15, 16, 17, 26
		HVO_2248	yes	2	MC27, 49
2,734,755	10	HVO_2248 (Near)	HVO_1357, +	3	MC1, 28, 31
		HVO_2248 (Near)	-	4	MC8, 23, 30, 35
		HVO_2248 (Near)	yes	3	MC4, 6, 7
1,147,136	2	HVO_0576	HVO_2248, +	2	MC36, 45
2,404,062	1	HVO_1926	HVO_2248, +	1	MC34
2,723,422	1	HVO_2229	HVO_2248	1	MC54
2,871,511	1	HVO_2377	HVO_2248, +	1	MC44
621,497	1	HVO_A0546	HVO_2248, +	1	MC52
1,867,146	4	HVO_1357	HVO_2248, +	1	MC11
		HVO_1357	-	1	MC19
		HVO_1357	yes	2	MC24, 37
1,867,189	13	HVO_1357	-	7	MC5, 10, 22, 29, 40, 41, 48
		HVO_1357	yes	6	MC2, 12, 20, 21, 25, 33
1,865,880	2	HVO_1357	-	2	MC42, 46
2,678,278	1	HVO_2176 (Near)	-	1	SAH1
2,225,641	1	HVO_1726	-	1	SAH2
1,020,227	1	HVO_0430	extensive	1	SAH3
3,126,642	1	HVO_2649	extensive	1	MC47

**^1^** Mutations are classified according to the genes which they affect (“HVO_2248 or HVO_1357” or “Other genes”) and according to primary (Tn-insertion) or secondary (secondary genome alterations). Mutations affecting HVO_2248 are highlighted blue, those affecting HVO_1357 are green. Other genes are highlighted yellow if they are considered relevant. Grey background color (indicating not to be relevant) is used for primary mutations in other genes if HVO_2248 is affected by a secondary genome alteration. Grey background color is also the case when secondary genome alterations are extensive. **^2^** Tn-insertion position refers to the first change compared to the chromosomal sequence of strain H295 (see Methods). For each Tn-insertion position, the total number of isolates is specified (Total Count). Subclassification is used when some isolates are affected only by Tn-insertion, some have secondary genome alterations affecting either HVO_2248 or HVO_1357, possibly also affecting other genes (indicated by +), and/or some have only other genes affected by secondary genome alterations. The number of isolates for each subcategory is given (Count). **^3^** Isolate names starting with SAH indicate the 1st screen (stabbing individual colonies on hypermotility plates). Isolate names starting with MC indicate the 2nd screen (central streak of the amplified library, picking strains that had moved farthest). If multiple MC isolates are listed, MC is omitted except for the first isolate. Full details for each isolate are provided in Table S1.

## Data Availability

Data are contained within the article or supplementary material.

## Supplementary Materials

The following are available online at https://www.mdpi.com/2073-4425/12/1/58/s1, Figure S1: Generation of *Hfx. volcanii* reference genome for Tn-insertion mutant genome analysis, Figure S2: Gene Ortholog Neighborhoods output using HVO_2248 of *Hfx. volcanii* DS2 as the reference, Figure S3: Motility plates used to select for hypermotile mutants MC1-54, Table S1: Primary and secondary mutations in hypermotile *Hfx. volcanii* Tn-isolates, File S1: *Hfx. volcanii* H295mod1_CHR reference sequence H295mod1 of the chromosome (with integrated plasmid pHV4), File S2: *Hfx. volcanii* H295mod1 pHV3 reference sequence H295mod1 of plasmid pHV3.
