# Supplementary material for: Mutations Affecting HVO_1357 or HVO_2248 Cause Hypermotility in Haloferax volcanii, Suggesting Roles in Motility Regulation
Source: Genes (Basel). 2020 Dec 31;12(1):58. doi: 10.3390/genes12010058 (PMC7824242; doi:10.3390/genes12010058)
Supplement: Supplementary file 1 [file genes-12-00058-s001.zip › genes-12-00058-s001/genes-1028798-supplementary/Collins et al. 2020 Supplementary Figures 2/Figure S1_Generation of the Hfx. volcanii reference genome for Tn-insertion mutant genome analysis.docx]

Figure S1: Generation of *Hfx. volcanii* reference genome for Tn-insertion mutant genome analysis

**
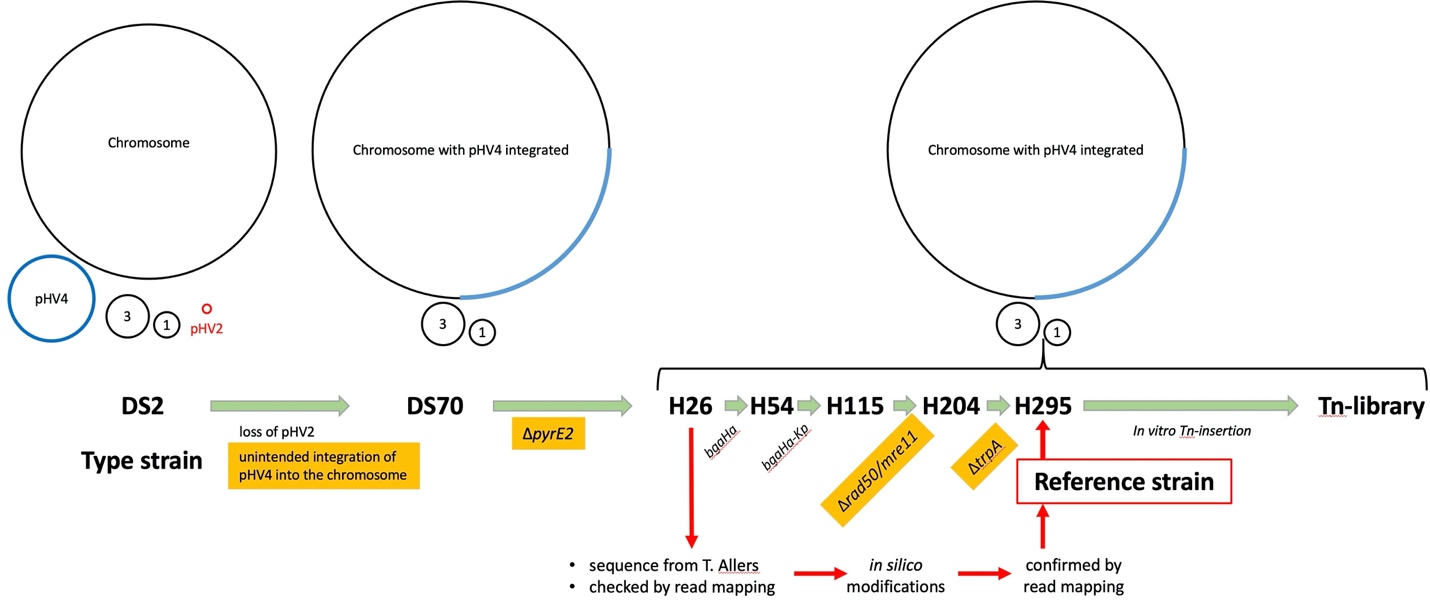
**

**Supplemental Figure 1. Generation of the *Hfx. volcanii* reference genome for Tn-insertion mutant genome analysis**. A reference genome was created *in silico* based on the type strain DS2^T^ ^[^[^45^](https://www.zotero.org/google-docs/?FkatNG)^]^. Strain DS70 is a derivative of DS2 that was cured of plasmid pHV2 but was found to have pHV4 inadvertently integrated into the chromosome ^[^[^46^](https://www.zotero.org/google-docs/?PI08vi)^]^. The DS70 strain was used to construct the Δ*pyrE2* strain H26 ^[^[^43^](https://www.zotero.org/google-docs/?q1ERwh)^]^, which was subjected to deep sequencing, with detection of a few additional SNPs ^[^[^47^](https://www.zotero.org/google-docs/?PI08vi)^]^. The green arrows describe the experimental strain genealogy between DS2 and H295, with the more important mutations affecting the chromosome highlighted in dark yellow. The red arrows denote the derivation of the strain H295 genome sequence from the sequence of strain H26 (kindly provided by Thorsten Allers), which was checked by mapping H26 reads (SRA accession SRX3581369) directly to the provided sequence. Next, modifications were introduced into this sequence *in silico* following the changes described in ^[^[^48^](https://www.zotero.org/google-docs/?z9dFUm)^]^, in order to obtain the strain H295 sequence. The changes made *in silico* to generate the H295 sequence were confirmed by mapping H295 reads (generated in the current study) to the *in silico* derived sequence. Strain H295 was used for in vitro Tn-library construction and as the reference genome.
