# Supplementary material for: Mutations Affecting HVO_1357 or HVO_2248 Cause Hypermotility in Haloferax volcanii, Suggesting Roles in Motility Regulation
Source: Genes (Basel). 2020 Dec 31;12(1):58. doi: 10.3390/genes12010058 (PMC7824242; doi:10.3390/genes12010058)
Supplement: Supplementary file 1 [file genes-12-00058-s001.zip › genes-12-00058-s001/genes-1028798-supplementary/Collins et al. 2020 Supplementary Figures 2/Figure S2_ Gene Ortholog Neighbourhoods output using HVO_2248 of Hfx. volcanii DS2 as the reference (1).docx]

Figure S2: Gene Ortholog Neighbourhoods output using HVO_2248 of *Hfx. volcanii* DS2 as the reference


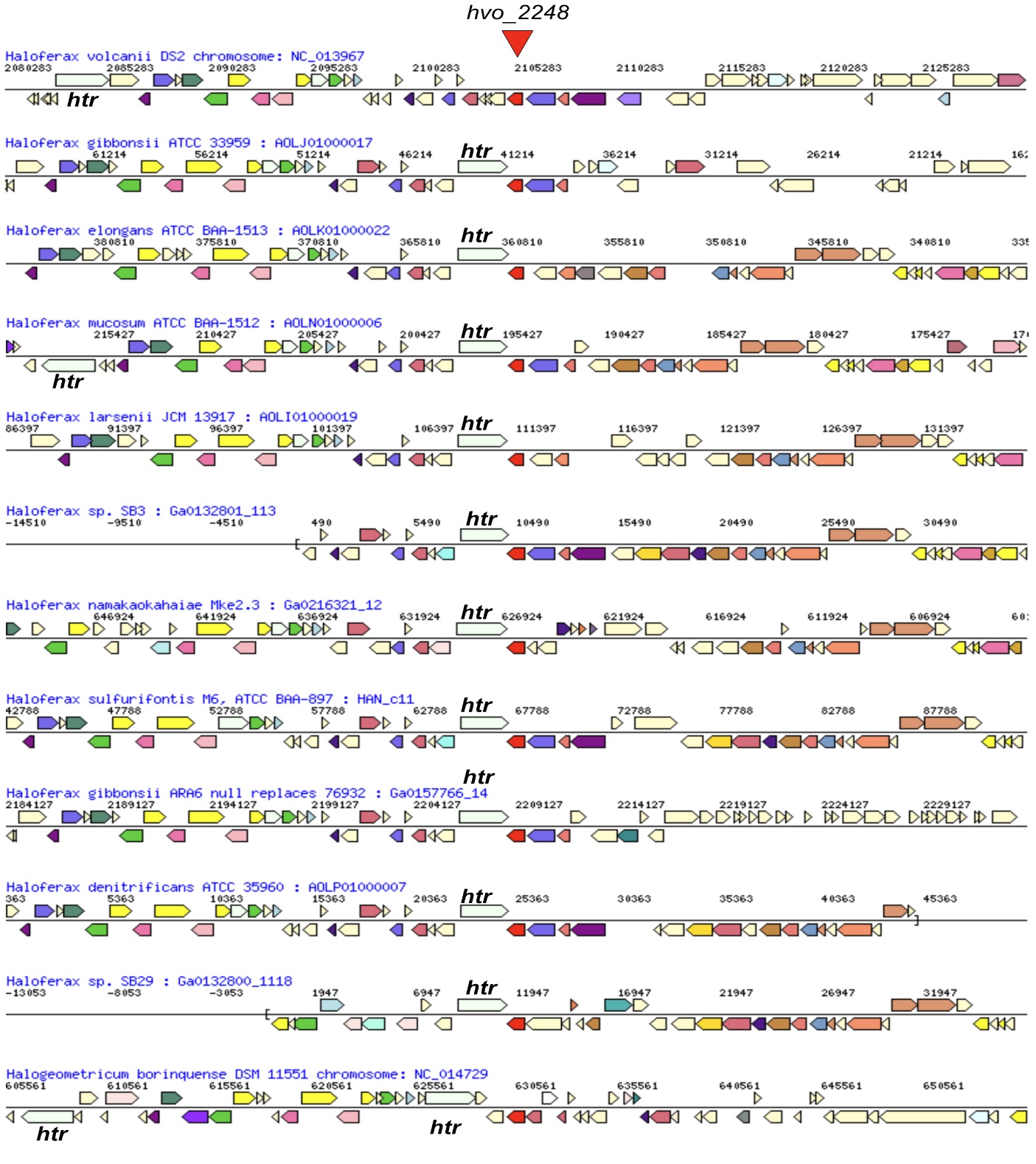


**Supplemental Figure 2.** **Gene Ortholog Neighbourhoods output using HVO_2248 of *Hfx. volcanii* DS2 as the reference.** This output was generated using the “Show neighborhood regions with this gene's bidirectional best hits” option available at the DOE JGI IMG/MER website ^[^[^54,55^](https://www.zotero.org/google-docs/?352bUU)^]^. All gene maps have been aligned so that their *hvo_2248*-like genes (red) are aligned with *hvo_2248* of *Hfx. volcanii* strain DS2 (top line), which is labelled above the line and indicated by a downward pointing arrow. The ten *Haloferax* spp. selected for comparison to *Hfx. volcanii* DS2 are those in which an *htr* gene (pale blue, labelled *htr*) is found adjacent to the *hvo_2248*-related gene. In all cases, the two genes are inwardly directed (convergent). The lower most gene map (*Hgm. borinquense*) shows a similar arrangement but in this genome, the gene for the Htr is separated from the *hvo_2248*-related gene by two intervening genes. Htr refers to a halobacterial transducer, which is also known as a methyl-accepting chemotaxis protein. Nucleotide numbers are shown above each gene map. Overall, the left half seems to show a higher level of gene synteny among genomes, while the gene sets differ much more in the right half.
