# Supplementary material for: Mutations Affecting HVO_1357 or HVO_2248 Cause Hypermotility in Haloferax volcanii, Suggesting Roles in Motility Regulation
Source: Genes (Basel). 2020 Dec 31;12(1):58. doi: 10.3390/genes12010058 (PMC7824242; doi:10.3390/genes12010058)
Supplement: Supplementary file 1 [file genes-12-00058-s001.zip › genes-12-00058-s001/genes-1028798-supplementary/Collins et al. 2020 Supplementary Figures 2/Figure S3_ Motility plates used to select for hypermotile mutants MC1-54 (1).docx]

Figure S3: Motility plates used to select for hypermotile mutants MC1-54


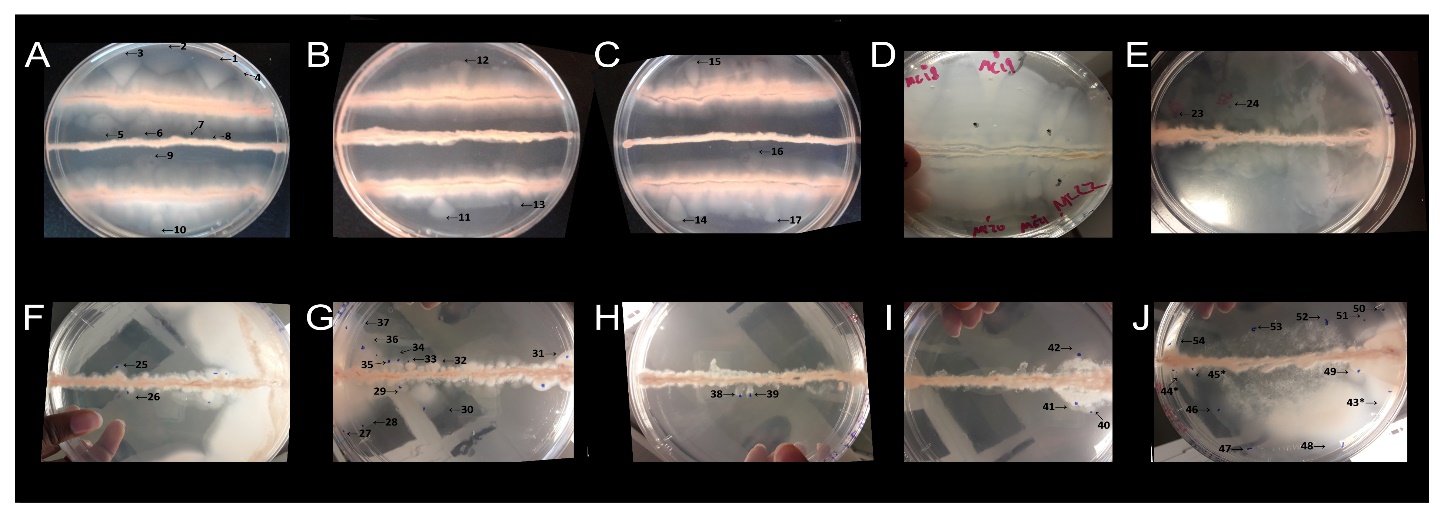


**Supplemental Figure 3**. **Motility plates used to select for hypermotile mutants MC1-54.** Pooled cells from the *Hfx.* *volcanii* TN-library were streaked across a motility agar plate and incubated at 45℃ for seven to eleven days. Plates A-C contain one central streak of a nonmotile mutant (*pibD*) and at some distance on each side, a parallel streak was made with pooled strains from the TN-library. Cells from these streaks do not merge and thus we were able to isolate cells that had moved farthest from the side streak toward the central streak. Additionally, some of the isolates were picked close to the edge from the side away from the central streak. Plates D-J contained a central streak of the pooled TN-library. Numbers correspond to MC mutant names. *not certain from which of the three halos MC43, 44, and 45 originate. Arrow indicates sample site.
