# Supplementary material for: Mutations Affecting HVO_1357 or HVO_2248 Cause Hypermotility in Haloferax volcanii, Suggesting Roles in Motility Regulation
Source: Genes (Basel). 2020 Dec 31;12(1):58. doi: 10.3390/genes12010058 (PMC7824242; doi:10.3390/genes12010058)
Supplement: Supplementary file 1 [file genes-12-00058-s001.zip › genes-12-00058-s001/genes-1028798-supplementary/Collins et al. 2020 Supplementary Figures 2/File S1 Hfx. volcanii H295mod1_CHR reference sequence H295mod1 of the chromosome (with integrated plasmid pHV4).docx]

**Supplemental File 1: *Hfx. volcanii* H295mod1_CHR reference sequence H295mod1 of the chromosome (with integrated plasmid pHV4)**

>Hfx_volcanii_H295mod1_CHR reference sequence H295mod1 of the chromosome (with integrated plasmid pHV4) (3476827 bp)

GACGACATCGCCGGGGGAGCGAAACTGATACGCGCGGGTTCTCGTGTTGA

AACGGTCCCGCCCGGCCGAGTTCCGGTCGAACGGCGGCGGACGGGTCGGA

GAAGTAAGGTTTTTGTACCAACTGTTCGTTTGGTTCCTTTGCATCATCTG

GAATCGCCTTCTTCGGCGTGATGCGTAAATGCCGACTATCACCGGCTTCA

CGCACCCTATACGCCGCCGCGGGCGACTATTCCACTTGAAACAAAGGGGT

ATTCACGATGGACGAGGAAGAGAATCCGCGAGGCGGCGCACGAGGGGAAG

CCGGGAACAGCACGCCGGACGACGGCGGCGCGGACGCCGACGCCGATGCT

GGCACCGACACCGACGACACACCCAGTAACGACGCGTCCGGCGGTGACGG

TTCCGCCGACAGTTCCGCCGTCGAGGACGACGTGTCGCCCGAAGACATCG

ACATTCAGGCGAGCATCGGTCCCGACGCCGACGCGCCCGACGCGTCCGAC

GAGGACCTCGACCAGCACAAAGACCCCGACGTCGGCCTCGACAAAGTCGT

TCTCAACGACGACGAGGGGTCGAAGGGGCTGTTCGACGACCTTCTCGCCG

GCGAGCCGATTTTCGAGAACAAGGAAGTCCTCCGCCCGTCGTACACGCCA

CACGAACTCCCGCACCGGACCGACCAGATAAACCAGATGGCGACGATTCT

CGTCTCCGCCCTGCGCGGAGAGACGCCGTCGAACATCCTCATCTACGGGA

AGACGGGGACCGGTAAGACCGCGAGCGCGAAGTTCGTCAGCCAGGAGCTC

GAATCCACCTCCCAGAAGTACGACGTGCCCTGCGAGGTCGAGTACATCAA

CTGCGAGGTGACGGACACGCAGTACCGCGTGCTCGCCCAACTCGCGAACA

AGTTCATCGAGAAGAACGTCGAGCGCATCGAGGCCGAACAGGAGCGCCTC

GACGAGATGCGCACGCGGGCGACCGAAGACCCCAACGCCCTCGAAGAGAC

GCCGTACGACTCCATCGCCGAAATCGACGAGCGCGCGGCGGAACTCGACG

ACGACGCCGACGAGATGGAGACGGTGCCGATGACCGGGTGGCCGACCGAC

CGCGTCTACACGACGTTCTTCGACGCCGTCGACTACAAAGAGCGGGTGGT

CGTCATCATGCTCGACGAAATCGACAAACTGGTCGAGAAGTCGGGCGACG

ACACGCTGTACAACCTCTCGCGGATGAACTCCGAACTCGATAACTCCCGC

ATCTCCATTATGGGTATCTCGAACGACCTGAAGTTCACCGACTTCCTCGA

CCCCCGGGTCAAGTCGAGCCTCGGCGAGGAGGAAATCGTCTTCCCGCCGT

ACGACGCCAACCAGCTCCGCGACATCCTCCAGCACCGCGCCGACGTGGCG

TTCAAGCCCGGCGCGCTCACCGACGACGTGATTCCGCTGTGCGCCGCGTT

CGCCGCGCAGGAACACGGCGACGCCCGGCGCGCGCTCGACTTGCTCCGCA

CGGCCGGCGAACTCGCGGAGCGCGGGCAGGCCGACACCGTCGAGGAGGCC

CACGTCCGGCAGGCGCAGGACAAAATCGAACTCGACCGGGTGGTCGAGGT

CGTCCGCACCCTGCCCACGCAGTCGAAAATCGTCCTCTTCGCCATCATCC

TCCTGGAGAAAAACGGCGTCCGCAACATCAACACCGGCGAGGTGTTCAAC

ATCTACAAGCGCCTCTGCGAGGAGATCGACGCCGACGTGCTGACCCAGCG

CCGCGTCACCGACCTCATCTCGGAACTCGACATGCTCGGCATCGTCAACG

CCGTCGTCGTCTCGAAGGGTCGCTACGGCCGGACCAAGGAAATCAGCCTG

TCCGTCCCCATCGACGAGACCGAGGCCGTCCTGCTGACGGACTCCCGACT

CGGCGACATCGAGAGCGCACAGCCGTTCGTGCAGGCGCGGTTCGACAACT

GAAACTGAAGAACGAAGTCCCGAAGTCCGACGCTTTTAGTGCACTCGCGT

CACGCCGCGAGCGCGGCGGTCCGCATCGGTGTGTTCCCCGCTTCGACCCG

AAGCGGTGCCGTCGTCGCCGCCGCGTCGCTTCCCGGCGCGTCGCCTGCCG

GCGCGTTTGCCGTCGACGCGTCGGGAACCGCCGTTTCCATCGTCGGCACG

TCGACGAACTCGACAGGGGCGGGCGCGACCGGCGTCGTCGTCGCTATCGA

GTTGGCGAGCGTCAGCCGAACCCACCCGAGAAGCGGGATACGGATTCGCG

CGACGCCTTGGACCCACTCGGGGCGGACCGGGTCGGCGATGCCGCTTACT

TGGTCGTACCGGGGGTTGTTGTCGCCTTTCGTGATGAAGCCGCTGTACTC

GGCGGGGCAGTTGGCGAGTTCTCGGCAGTTGTCGGCCGACATGTACTCGG

GGTTCGCCCGGTCGTACCAGTTCTCTCCGTCGTCGACCCAGAACATCGCC

CGGTGGATAATCGGGGGACCTGCCGACCCGGGGTCGTCGTAGACGATGAC

GCTCCCGGGGCCGCCGAACTTCCGGTAGTCGACCTCCGCGCCCGTCTCGG

CCGTGACGACGGCGGTCCCCTCGACCGCCGAGTCGGGGGCGTATCGCTCC

GGTCCCGTGATGAACACGAGGTCGCCTTTGTGCATGTGCGGTTCCATGCT

CCCGCTTTCGACGGCGACCATCGGCGGCCACACGCCGCTCAGCGCGAACA

GGAGCAACCCGACGGCCAACACGGCGAGCGCGCTCGTCAGGAGTTCGCGG

ACGAACAGAAGCGGCCCCTCCTCTGCCGTCCGAAGCCGAGTTAGCACGCC

TTCGTCGGAGGTTGGACCGCGCCGCGAGTCGGGGGAGGGCGGGCGGCCAT

CGTCGTCACTCATCACTCCCAGTTTACCCGGACTGGGTTTCAACGTTCTG

GGTTTCGGCATCCTTTTTGCCGCGTGTCGCGCACTCCGGGTGTGCCACTG

GAGACGCCGGCGCGCATCGTCCGGACGCTCGTCGGTCGCGGCTACAACGC

CGAACGCGAGGCCGTGACCCTCATCGCCGGCTCCGACGACCCCGGCCGAA

CCCTCGCCCGCGTCGTCGAAGCCGCCCCGGACGACGCGCTTCGCATCACC

GCCGACCACGTCCGCGAGGTGCTCGCGTCCGCCCCCGCCGCGGACCCGTC

GGGAACCGAACCGACGCCCCCGGCCGCTGCGGACGCTGCGAACGCCGCGG

ATGCCGACCCAGCCGCCTCCGCCGCATCAGACCCCTCCGTTTCCGCTGCA

ACATCGCACGACAACGCGGCCCACACCGACCAGTCTGCTCCAGCCGAAGC

GCAGGGGACACCGGGCGGCCGGAACGTCGACCCGGCGCTTCGGTCCCTCG

AAATCGCCAACGACATGACCGGCCAGTCGACGGGTACCGGCGAGTACGAC

GACTTCGTGAAGGTGTTCCGCGACCGCTACGAGAAGCTCTCGAAACTGCT

CCGCGGGCGCGTCAACCACCGCCCCGCGAAGGCCATCTCGAACATGTCCG

GCGGCGAGGACGCCGAACTCATCGGCCTCGTCGACGACGTGCGCTCCACC

AAGAGCGGCCACTGGATTATCGACCTTGAGGACACGACGGGGACGTTCCC

CTGTCTCGTGATGAAGGACAAGGACATCGCGGGCTACGTCGACGAACTCC

TCATGGACGAGTGCATCGCGGTGCAGGGGACGCTGTCGGGCGACGCGGGC

ATCCTCTTCGTCGACTCGATGCACTTCCCCGACGTGCCGCGGAGCCACCG

CTCCAACACCGCCGACAGGGACGTGCAGGCGGCGCTCATCTCCGACGTGC

ACGTCGGCAGTCAGGAGTTCATGGCCGACGCGTGGAACCGCTTTGCCGAC

TGGCTCCACACCGAGGAGGCAGCGCGCATCGAGTACCTCCTCATCGCCGG

CGACATGGTCGAAGGCGTCGGCGTCTACCCGAATCAGGACGAAGAACTCG

ACGTCATCGACATCTACGAGCAGTACGAGGCGTTCTCCGAGCACCTCAAA

TCGGTGCCCGGCGACCTCGAAATCGTGATGATTCCGGGCAACCACGACGC

GGTCCGACTCGCGGAGCCCCAACCCGGCTTCGACGACGAACTCCGCGACA

TCATGTCGGCTCACGACGCCCGCATCACGAGCAATCCCTCGATGGTCACG

CTCGAAGGCGTCAACGTCCTCATGTACCACGGTGTCTCGCTTGACGAGGT

CATCGCCGAACTGCCGGCCGACAAGGCGAGCTACGACGAGCCGCACAAGG

CGATGTACCAGCTTTTGAAAAAGCGTCACGTCGCGCCGCAGTTCGGGGGC

CACACCCGCCTCGCGCCCGAGGAACTCGACTACCTCGTCATGGAGGACGT

GCCCGACATCTTCCACACCGGCCACGTCCACAAGCTCGGTTGGGGCAAGT

ACCACAACGTCCTCGCGGTCAACTCCGGCTGCTGGCAGGCCCAGACCGAC

TTCCAGAAATCGGTCAACATCGACCCCGACGCCGGCTACGCGCCCATCGT

CGACCTCGACACGCTGAACATGACGGTCCGGAAGTTCTCCTGAGGCGGCG

ACCGTTTCACTACTGACGACCGTTCGACTCAGCGACCGCTCTCCTACCAG

TATGCTCCCTGATAGCACGCCGGATGTGCGGCGACCGAGACCATTCCACC

GTCGCTTTTTATCCGCGACGCCCACAGAATCGGTGATGACCGACGACGCG

CTGTTCGACGCGACGAGCCTGAACGACCTGTCGCTTTCCAACCGAGTCGG

GCTGGCACCGATGACGCGCATCAGCGCCACCGACGAAGGACTGGCGACCA

ACGAGATGGCCCACTACTACCGGAAGTTCGCCGACGGCGGCTTCGGCTTT

CTCGTCACCGAGGGCGTCTACACGGACGACGCGTACAGTCAGGGATACCT

GAACCAGCCGGGACTCGTCACCGACGACCACGTCGAGGCGTGGACGAACG

TCACGGACGCCGTCCACGAGGTCGACACACCGATTTTCGCGCAGTTGATG

CACGCCGGGGCGCAGTCGCAGGGCAACCCCCACCTCGACGGCGACCGGAC

GCTCGCGCCCTCCGCGGTCCAGCCCGACGGCCAGAAGGCCGAGGCCTACG

GCGGCAGCGGCGAGTTCGCGGTCCCGAAGGCGGCCGACGAGACCGACCTC

GAAACCGCCCGCGAGGGGTTCGTGCGGGCGGCGACGAACGCGGTCGAAGC

CGGCTTCGACGGCGTGGAACTCCACGGCGCGAACGGCTACCTACTCAACG

AGTTCCTCGCGGCGAACGCGAACCGACGCGACGACGAGTACGGCGGCGGT

CCCGAGTCCCGCGTGAAATTCCCCGCCGAGGTGCTTTCGGCGGTCACCGA

CGCGGTTCCCGAGGAGTTCGTCGTCGGCATCCGCGTCTCGCAGGAGAAAG

TGACCGACGGCGACTACGAGTGGCCCGAGGGCGAGGACGCGGCCGCGGTC

TTCTTCGAGGAGCTGTCGGCGGCCGGAGCCGACTACGTCCACACGACCGA

GACCGACGCGACGACTCCGACCTTCGGCGCGGACGGTCCGACACTCGCTG

AGGCCGCCGCCGAGTACGTCACCGACGACACGGTCGTCATCGCCAACGGC

GGGCTCGGGGACCCGGACGCGGCGCGGGCCGCCGTCGACGCCGGCGCGGA

CCTGTTCACGCTGGGCACGAGCGCGCTCGCCAACCCGGACTGGCCGGCGC

GCGTGGCCGCCGGCGACGACCTCGACGCGTTCGACCCGGCGAAGTACCTC

GCGCCGACCGCCGGGCTCTCGGACCACGAGGTCCCGTCCGAGCCGCCGCT

CGCGGACGACTGACGCGCGCTCGACGGCGGCTTCTCCGCCTGCGAACTAC

CCACCGAACCCCTTCGCTTCCACTCGAACTCCCGTTTCACAGTTCCTCGA

CGGTCGCCGTATTCGGACGCGCTAACTCGATTCGACTACCCGAGCGTCAC

GTCGAGGTAGAGCATCACGACGACGCCGACCATCGTCCCGAACGTCGCCA

CGCGCTCGTGGCCGTTCGAGTGCGTCTCGGGGACGATCTCGTCGGAGATG

ACGAACAGCATCGCGCCGGCGGCGAAGCCCATCGCGTAGGGAAGCAGCGC

CGCGGCGTACTGGATGGCCCACGCGCCGAAGACGGCGAGCGGAATCTCCA

CGAGGCCGGCGCGGATACCCGCGAACGTCGCGTAGGTCGTGTTCCGAAGC

CCGGCGTTGACGGCGGCGATGGAGACGGCCAGCCCCTCGGGGATGTTCTG

GATGCCGATGGCGAGCATCAGCGGGACGGCGGTCCCGAGGTCGCCGGAGC

CGAAACCGACCCCGACGGCGAGGCCCTCGGGCATGTTATGGATGGTGATG

GCGACGATAAACAGGACGACAGAGGCCATCTTTTTGTCCGTCTCCGGGGC

GTCCGCTCGGGTCTTGCCGGTCACGAGGATGTGGACGTGCGGAATCCACA

GGTCGGCTTGGTCGAGGACGACGACGCCGATGACGAAGCCGACGAGGACG

GGAATCGGACTCCCGCCCGCCGCCTCGATGCCGGGGAGGATGAGGCTCGT

GAAACTCGCGGCGAGCATGACGCCGGCGGCGAAGCCGAGGAGCGTGTCGA

GCGAGCGCTTCGAGGGGTCGCGCCAGACGAGAATGAGGAGCGCGCCGAGC

ATGTTCATCCCGGCGATGACGATGCCGCCGAGGAGGCCCTGCATCACCGG

GTTCGAGCCGGCGAGCGAGACGAACAGCTCTTCGACCGCCGTCACGGCCG

TCTCACTTCCGGCGGCCGTCTCTCGCGTCGTTCGGTCATGGACCACCTGA

CGAGCGGGAGGTATGTATTCCCTCCGTCGGTTCTCGCCGGCCGCGAGGCT

CGACTATCCCGTCTCGTCGAGGACGTACCGAAGCGTCGGGGACCCTTCGA

ACCGCGGGCCGTGGCCGACGCGGTCGAAGCCGACGTGCTCGACCGCCTCG

CACAGCGGGGCCTCGGATTCGAGGACGAGCACCTCGACCGGCAGGTCTTC

GGCGCTGGCGAAGCGCAGCGGCTCTTCGAGCAGTCGCCGCACCGCCGCCT

CGGTGCCGCCGAGGCGGGTGACGTGGAGGACGCCGTCGCGGACGTCGAAG

CCGACGAAGCCGAGCACCGGTTCGTCGTCGGCCGGCGCGTCGGCGTGGGG

GTCCGAACTCGTCTCGGCGGTGGCGAGGCGGACCGTCCGGTCGCGTATCA

GCCGACGCATCGCCCGTATCGGCGCGTCGGCGACGGCGGCGAGCGCCTCG

GCGTCGGTGTCAACGGCGTCTCTGACATCCATGCCCGAACGTTCCGCGGC

TTCGGTGATAAATCTCCGCCCGCGAGGCGTCGGTACGGGCCGTCTCAGCG

TCGGATTCGCGGGTGACGGCAACGGTTGAATCGGAGGACATAGTTATACG

TCCCCCCGCGTTAGCCCCTCGCATGTTTACCAACGAAGCCCCGAAGCGAC

GCGACGCACCGGAGGAACGATGCGCGTAGTCGCGAAGTTCGGCGGCACCT

CGCTCGGTAGCGGCGACCGAATCAACCGCGCCGCCGACTCCATCGCCGCG

GCCGTCGAACACGGCCACGAAATCGCCGTCGTCGCCTCCGCGATGGGCTC

GACGACCGACGACCTCCTCGACGAAATCAAGTTCGAGGCCGACGACCGCG

ACCGCGCCGAAATCGTCTCGATGGGCGAGCGGACCAGCGTGCGCATGCTC

AAGGCGGCGCTGGCCGCCCGCGGCGTCAACGCCCTGTTCGTCGAGCCCGG

CACCGACGAGTGGCCGGTCATCACGAACGACCTCGGCGAGGTCGACGTCG

AGGCGACCCGAGAGCGGGCCGCGAAGCTCGCCGCCGAACTCGACGGCGTC

GTCCCGGTCATCACCGGCTTCCTCGCGCAGAACCACGACGGCGAAATCAC

GACGCTCGGCCGCGGCGGCTCCGACACCTCCGCCGTGATGCTCGGCAACT

ACATGGACGCCGACGAGGTCGTCATCGTGACCGACGTGGAGGGCGTCATG

ACCGGCGACCCGCGCGTGGTTGAAGGCGCGCGCAACGTCGGCCGCATCAC

CGTCGACGAGCTTCGGAACCTCTCGTTCCGCGGGGCCGAGGTCGTCGCGC

CGTCGGCGCTGTCGTACAAGGACGCGGCGCTGGACGTTCGCGTCGTCCAC

TACCAGCACGGCGACCTGCTCACCGGCGGGACGCTCATCGAAGGCGAGTT

CCACAACCTCATCGACATGCAGGAAGAGCCCCTCGCGTGTCTCACGGTGG

CCGGGCGAGCGATTCGCAACCGACCGGGAATCCTCGCGGACCTCTCGGCA

GCCCTCCGAGAAGAGGACATCAACGTCGACTCGGTCGCCTCCGGGATGGA

CTCTATCACGTTCTACGTCCTCGAAGACGACTCCGACCGGGCCGAGGCCG

TCCTCCACGACCGCGTCGTCGCCGACGACGCGCTGTCGTCGGTCACCGTC

GAAGACGACATCGCCGTCGTCCGCGTCACCGGCGGCGAACTCCCGAACCG

CCCCGGCGTCATCCTCGACATCGTCGAGCCGCTGTCCGAGGCCGGCATCA

ACATCCACGACGCCATCACCTCCGCGACCTCCGTCGCCATCTTCGTGGCG

TGGGACGACCGCGAGGAGACGCTCGGCATCATCCAAGACGAGTTCTGAGC

CGGTTCTCGTCGCGCTCTCGAAGCTGTTTCTCGCGCGCTCGCGTCCTCGA

AAGTGACATCGCTCGACCGGTGGTCGTCGGCGGTCGCTGAAGTCGGCTCG

TGGCGAGAACGGAACAGCCGGCGACACCGATGCACACACCAGTCCACGAG

CGCCGAAAACCGGGCGTAGCCCCTCGATTTTCCGCCTGCCGATTACTTCA

CATTCGCGGACCTATTGCGGGCATGAAATCGTACAAGGCGAAGATGGTCG

AGCCCATCGAACTCCCCTCGCGCGAGGAGCGCGAGGCCGCCCTCGAACGC

GCCGGCTACAACGCGTTCAACCTCGACGCTCGCGACGTGTACATCGACCT

CCTGACCGACTCGGGGACGGGAACGATGTCCGCAGAGCAGTGGGCCGCGA

TGATTCGCGGCGACGAGGCCTACGCCGGCAGCGAGTCGTTCGCCCGACTC

GCGGAGTCGGTCCGCGACGTGATGGGATTCGAGCACGTCGTGCCGACCCA

TCAGGGCCGCGGCGCTGAGAACGTCCTCTACGGCGTCCTCTTGGAGGACG

GCGACGTGGTGCCGAACAACTCCCATTTCGACACGACCCGCGCCCACGTC

GTCAATCAGGGCGCGGAGCCGGTCGACTGCCCGTCGCCCGCCGCTCGCGA

CCCCAACTCGACGGAGACGTTCAAGGGCAACTTCGACATCGACGCGGGCT

ACGCGCTCGTCGAGGAGGTCGGGGCCGACGCGATTCCCGTCGTCGCCCTC

ACCATCACGAACAACTCCGTCGCCGGCCAGCCGGTCTCGATGGCGAACAT

CCGCGCCACCGCCGAGTTCGCCCGCGACATCGACGCCATGTTCGTCATCG

ACGCCTGTCGGTTCGCGGAGAACGCCCACTTCATCAAGACCCACGAGGCG

GGCTACGAGAACCACTCGGTCGCCGAAATCGCCCGCGCGCAGTTCGAACA

CGCAGACGCCATCACGATGTCCGGGAAGAAGGACGCCCTCGTCAACATCG

GCGGCTTCGCCGCGATGCGCGACGAGACGGTGTTCGAACACGCGAAACAG

CGCGCCATCCTCTACGAGGGCTTTCCCACCTACGGCGGCCTCTCGGGCCG

CGACATCGAGGCGATGGCCGTCGGCCTCCGCGAGGCCGTCACACCGCCGT

ACGTGACCGACCGCGTCGAGCAGGTGGCCGAACTGGGCGACCTGCTGGTC

GAGGCCGGCGTCCCCGTCTACCAACCGACCGGCGGGCACGCGGTCTACCT

CGACGCCGGCGAGGTGTTCCCGCACATCCCGAAAGAGGAGTTCCCCGGAC

AGGAACTCGTCTGCGCGCTCTACCTCGAAGGCGGCGTTCGCGGCGTCGAA

CTCGGCGGCTTCGCGTTCCCCGGCACCGACCGGCCAGACCTCGTGCGCCT

CGCGCTCCCGCGGCGCACATACAGCCGCGAACACCTCGAACACGTCGCCG

AGACGGCCGCGAAGGTGATGGCCTCGGCCGGCGAATACGGCGGCCTCGAA

ATCGTCGCGGAGCCGCCGATGAAGGAGCTTCGGCATTTCTCGTCGCGGCT

CGAACCGGTGTCGAACTGATGGCTTCAGGACGGCCACTCGCGTTCCCCTA

TTTTCCCTCGTACAGCCGACTCGCCAGTCGCTCCGGCAGGCCGGCCTTGC

GTACGCGCTCCGCGACGCGGTCGATGTCGTAGTTGACGCGTTCCGCCTCG

ATTTCCATCGCGTCGAGGTCGACGACCGCGTAGGCCGCCCGCGGGTCGCC

GTCGCGGGGCTGGCCGACGCTACCGGGGTTCAGCACGATTCCGTCGTCGT

AGATTCGGTAGCCCTGCACGTGGGTGTGGCCCAACACCAGCAGGTCCTCG

TCGTCGAGGAGCGACGGCGAGAAGTCGTCGGGATAGGTGTAGCGGTCGGG

GTGGTTCGGGTGGCCGTGGGCGAGTTTGACTCGGCCGTCGGCGAGCGTCC

GGTTCTTCGGAAGCGCCGAGAGCCAATCCATCGACGCCGGCGACAGCGCG

TCGCGGGCGTAGTCGACGCCCGCGGCCGCCATGCTGTTGAAGCGGAAGCC

GGTCCCCGACGTGACCGCGCGGTCGTGGTTCCCCGAGACGGTCGGCACGT

CCCGTTCTGCGAGTTCGGACACGCACTCCTCGGGCCACGGGTTGTAGCCG

ACCACGTCGCCGGCGCAGACCAGTCGGTCGACCGAGGGCATCGATTCGAG

CACCGCTTCGAGTGCCGGGAGGTTCCCGTGCACGTCCGAGATGACGCCCA

GACGCATGGCTCGGTGTACGGTGTCGCGGGTGAAAAATCTCGGGCCGCGG

CGGCGTTATTCGCCGTTGTAGAAGGAGGTTTCGACGGCGTCGCCGACGGT

CGCGGCGGCGGCGCAGACGGCGTGTTCGAACTCGTGGTCGTACAGTTCGC

GGGCCGCGTCGGCGGCGGTCTCGGCGTCGAGGTCGAACGCGCGCGGGTCG

TCTTCCTCGTAGGTCGCCACGAGCGTCGGTTCGGTGACAGCTTCGACGAC

GAGGGCGTCCTTGCGGACGATGCCGATGTAGGCCTCGTCGTCGCCGACGA

CGCCAGCGATGCGGGGCGTGTCGTAGTCGTCTTTCTCGTAGTCGAGCGCG

AGCAGAATCTCCGCGAGCGCGTCGCGCGGCGGGTAGCCGAGGTCGAGTTT

CTCCGCGACGGGGTCGACCTGCGAGCCGTTGCCGACGACGACGTAGTCGC

CGCCCTCGCGGACGCAGTTGTAGGCGATGTAGGGGTTGTCGGTCTCGGGG

GCGTCGGGCGTCGGCCCGACGGTCAGGGCGTCGTCCCGGTCGACGACCCG

TCGGTTCGGGAAGGACCGAGAGGAGACGCGATACGCACCGATTCCGGGAC

TAACGACGACGAAACGTCCGACGTACATACACGACAGTGGATAGAATAGG

GGGAAATAGTTGGCGGTTTATGCACGTCCGTCCGTCACCGTGCGACGAAA

TCCCACGGCGGAATCGACACGCAACGTTTACAAATACCCGCGAAGTACGA

ACTGATGCGAAGTCCCATGGGGTAGTGGCCAATCCTGTTGCCTTCTGGGG

GCAACGACCCAGGTTCGAATCCTGGTGGGACTATTTCTCCGATTCTCACG

CGCTTCGAGGCTACGCTGTCCCCCCTGATCTCCAGTTGAAACGAAGGGGT

GGGATTCGAAAACCCAAGGGGTTTGTTTCGAGTGGAGTTCTTGAGTACGG

ATTCGCGGGACCACCGGATACGGCCGATACGGGCCGCGAAAACCGCTTAG

CGGTCGACCGCGAGGAGCGCCACTCGCTCTCTGACCAGCGCGGGGAGGTC

GCCGTCGACGACGTGCAACTCCGTCTCGCCCACGTCGAACACGTCGCGGA

CGAGGCTCTCGTCGTAATCACCGAGGGTCTCTGCGGGTTCGAAGTCGAGG

CGGTCGAACAGCGCGGCCTCGGCGGCGGCCTCGTCGCCGCCGTCGACGAG

AACCACGACGGGGAGCGTCCCTTCCGAGACGCCGATTTCGAACGCGCGGT

TAATCTGCCGGCGGCCGCTGGCGTAGAGGAGGATTTCGACGGCGCGGTCG

CGGGCGATTTCGTTGCCGCGGGCGATGGCCCTGTCGGCGAGCTCGACCGC

TCGTTCGAGGTGCTCGCGGTCCACGACGTACCGGGCGTCGAACGCCTGCA

CCGTCGCGCCCGTCTCGTCGGCCACGTCGCCGACGGCCGCGATGAACGAA

TCGAGGTCCGAGACGGTCGCCTCGGCTTCGAGGAGCCTCATTCGAAATCA

CCCAGACTGGCCTGCTGGTCGGCGCGCTCTTTGGCCGTCTCGAAGCCGGC

GTCGTCGGGGACGGCGTCGTCCGGCGCGTCGTCTTCGTCCACCGCGTCCA

TCGACGGGTCCTTTCTCCCCGCGGCTTCGAGGATGTTCTCCGCGGTCTTC

CTGCGACCCCGGAGGGCGGCGAGGACGCGCGGTTTGTCGGCCTCGCGGAG

GTCGGCCCGAGTTTCGACGCCGGCCTCGAACAGCCGGCGGGCGCGCTTTC

GGCCGACGCCGCGGACGCCGGCGAGGTCGAGCAGTTCCTCGCGGACGCCG

TACTCGACGCGCTTTTTCGCCTCGCGGACCGCGTACACCGAATCGAGGTC

GAGTTCGGTGGCGAGTCGCTCGGCCGCGCCGAGGAGCCACTCGGAGGTCT

CGACTTTCCCGCGGATGTCGCCGGGGCCGACCCCGTAGCGCTCGGTGATG

CGGTCTTCGTCCACCTCGCCGACCCAGTCTTCGAGGAGTTTCGCGGTCTT

CAGCGCCGACAGCCAGTCTTCGAACGCCACGTCCTCGTACTCCGAGGGAA

CGCGCCCGAGGAACTCCGGTTCGCGCTCGTAGCACAGTTCCGTGTAGGTC

TCGCGGTCGCCGGACTTCAGGTAGAGCTGATACATATCGGGCGTCCGGCA

GACGAGGTGATAGAGACCGAGCGGCGTCGGATACGTCCGGTCCGTCTCGA

CCCCGTCGGCGTCGTCGCTGTCGCCGTCGCCGTTCGCCCCCACCCCGTCT

TCGCCGCCTCCACCGTCGCCGTCGTCAGCGACCATCTCGCTTGCCCGCTG

GAAGCCGCCGGATTCGTCGGATTCGCTTCGGTCTCGCGTCGGCTTCTCGG

GCGTCTCGCCCGCGAGCGCCCGGAGCTTCTCGGTCCGGTGGTCCGCGGCC

CATTCGAGGCCGTCGATGATTTCGGCGGCGCTCATCGGGTCGAGGTAGAG

CCGCGAGACGGTGTGGCCGACGGGCGTCGCCTGTATGGTCTCTCCTTCGA

ACTCGACGAACCCGTTTACTTCGAGGTAGTCGAGCACGCGGTCCGTCACC

TGTCCGAGTCGCTCGGGGTCGTCGGTCTGGGTCGCATACAGTGTCTGGTC

TAAGAATTCGAGCAGTCCCTCACGGGTGTGGGCGAAGCCGGAGGCGACCG

TGGCGAGCAGGTGCGTCCGCAGGGCCGGCTCGGCCGCGAGCTTCGACCGC

ACGTCCTCGGCGTCGGCCCAGATGTACCGCTCGAACAGTTCGTCGCGGGC

GTCCGCGTCCTTCGCCAACAGCACCGCCTCGCCGTAGGGGTCGAGGCCGG

GGCGGCCGGCCCGACCCATCATCTGGTGGACTTCGAGCACGTCCAGCGGT

TTCATGCCGCCGTAGTCGCCGTCGTAGCGTTGCCAGTCGCGGACGACGAC

GCGGCGGCTCGGGGTGTTGACGCCGGCGGCGAGCGTCGGCGTCGCGCAGA

TGCATTTGATGAGTCTGTCGCGGAAGGCGTCTTCCACGAGCGTTCGGTGT

TCCGCGGCGAGTCCCGCGTGGTGGAACGCCGCGCCTTTGGCGACCGCGTT

GGCGAGGTCGTCGGAGGTCTCGGTGTCGGACACGTCGCGTATCTCGGCGG

CGAGCTCCGCGAGGTCGCTCCGCTCGTCGCCGGTGACGTACCGCTCCGTC

ACGTCGGCCATGCGGCGGGCCGCGGACTCCGCGTTGCGCCGCGAGTTCAC

GAAGACGAGCGAGGAGCCTTGGTCGCCCTCGCCGTCGCCCTCCAGCGCGT

CGGCGACGAGCGCGGGCGTCTGTCGTTCCCCGCGGCCGACCGGCACCTCG

CGTTGGCTCCCGTCGGCGAAGGAGACGGCGTTGCCGTAGTGGACGCCCAT

CTTGAGGTCGATGGGTCGCCAGTCGGACTTGACGAGCTCCGCGTCGAGCC

AGTCGGAGACGACGCCCGCGTTGCCGACGGTCGCCGAGAGTGCGACGACT

TGCAGGTTCGTGTTGAGTCGGCGGAGCTTCGCCAGCGTGACCTCCAGCGT

CGGCCCGCGGTGGCGGTCGTCGACGAGGTGGACCTCGTCGGCGACGACGC

AGGTGAGTTGGTCCATCCACGCCGCGTTGTTCCGGACGAGCGAGTCGACC

TTCTCGCTCGTGGCGACGATGATGTCGCGCGAGGAGAGCCACTCGCCGTC

GGACTCGTAGTTGCCGGTCGAGACGCCCACGTCGATGCCGTACTCCTCCC

AGCGCTCGAACTCGGCTTTCTTCTCGGAGGCGAGCGCCCGGAGCGGGACG

ATGTACAGCGCCTTCCCGCCGCGCGCGACGCTCGACAGCATCGCCAGTTC

GGCGATGAGCGTCTTTCCGCTCGCCGTCGGGACGGCGGCCACGAGGCTCT

CGCCGTCCGTCAGGCCGGCCTCGACGGCCTCGGCCTGCGGCGGGTACAGC

TCCTCGATACCCTCGTCGCGGAGGGCCTCGGGAATCCCCGTCGGCAGGCC

CGTCAGGTCCGCAGTTCGCATTGCATCTCCCTTGGACCGTCCCCCGGTTT

AAGCCATCGGGTCGGTTCGGTCGCCGACCCACGTCCCCGCGTCGGCAGGG

CTTTGTACGGCCCGGCGGTACGATGGCGCATGAGAGTCGAGTACGACCGC

GACACCTGCATCGGCATGTTCCAGTGTGTCGACGAGTGGGAGGGCTTCGA

GAAGAACGTCGACGACGGCAAGGCCGACCTCGTCGACGGCGAGGAGACCG

ACGACGGCGTCTTCGTCCGCGAGGTCCCCGAGGACGCCGAGTTCGACGCG

AAGTTCGCCGCCCGCGTCTGCCCCGTCGAGGCCATCCGCATCCTCGACGA

CGACGGCGAACAGCTCGTTCCCTGACCGCGACGCCGACGACGCCACCGGC

TTCTCTCGCTTCTTCGACCTGACCGGCCCGTGTGACCGCCGACCGTGGGC

CGCGTCCCCGAACCCCTCCGTTTCGACTGGAGATTCGACCGACGATAGAG

TGTAACAGTTAAGCGGCGGGCCCCTAATCGTGCCACTATGAGCCAAGCGA

CGAAAATCGTGCTCGGTACCGTCGGCGTGTCGGCCCTGCTGGCCGTCGTG

TTCGTCGGGATGAGCATCGCCTGAACGGATGTTCGAGACGCGCAGCCTGC

CGTCGGACCTCGAATCGGTCCGCGACGACTACGCCCCCGGCGCGCTCGTG

TTGGACGTGGCCGGCGACTTCGACACCATCCCCCCCGAGGCCGCCGAAAA

CCTCGGGCTGGTGGTCGACTCGCTCTCCCCGGCGGCGTACCCCGCCGAGT

GGCTACCGGACGACGCGCCCCAACAGCTTCGGCGCTACGCGTCGTCGGAT

TTCACCATCGGGATGCCCGGCGACGGCACCGTGTCGTGGTCCCGACAGAC

CGACCCGCCGGTCGTGCTGGTGAAGTACCGCGCGAAGGGGACGCCGGACG

ACTTTCTGGATTTCCTCATCGCCGAGGCGTTCGTGCAGGCCGGGAACGAC

GAGATTCCCGAACACTTCCTCCCCTTCTTCGGTGAGCAGTACGCCGACCT

CGCGGCGGCGACGCCGCTCGGCCCGAGCGAGACGTATCAGGTCGCCGCCG

CCCTCTACGAGGGTTGGGTCGGCCTCCACACCCGCGAGGCGTTCGCGTCG

TGGGAGGGCGACCACGACCGCCTCCACGAGGCGTGGGTCGACGCCGGCGG

CCGCCTCGACAACCGCCTCGAAAATCTCCCGCGGCTCGTCGCGCTCGGTA

GACTCTCGTTCGCCGAGGCGACCGAGTTCGCGTGTTCGGCCGTCAAACAC

GGCCGCGATCTCCCCGCGCCGTTTTCGGCGCTGGACACCGCGGCGTACCG

CGACCACGGCCCGAGCTACGCGGTCAAGTGGGCCGAAAAGACGTTCGAGC

AGCTCGCGGCCGACGACGATGGCGCTGACGCGGAATCGAGCGACGACGCC

GACCCGACCGCGGACGACGCCGCCGACTCGGCGTAGTCGGTCGCGTCGCG

TTTCGCCTCAGAACTCGGTCGTGACGGTGCCGTCCTCGTTGAGGTCGACG

ACGCCGTCGAACAGTTCGCGGAAGCGGTCGAGCGTCTCCTGGTCGTGGAC

CTCCTTCGAGAGGTGGAACAGCCCCACCGCGTCGAACTCGTCGAGCAGTT

CGAGAATCTGTTTCGTCGCCTCGAAGGCGCGGTCCTCGTCGGCGTAGTAG

GCCATCTCCGTGACCGAATCGACGCTGAGCCGGAGCTTCCCGTCGTGGCG

TTCGAGGAACTGCCGGGTCTTCTCGACGATGGCGTCGAGGTCGTCGGGGG

CCGCGACGTAGTGGATGTTCTCCGAACTGCGCCGGGAGTAGCCGCGCTCG

ATAGACAGCGTGTCGAGGATGGTGGCGCTGGACTCGTCGACCTCGTAGTG

TTCCAGCTTCTGTTCGACCTCGCGCGCGGTCGTCCGCGTCGAGATGACGA

GGAAGCGGTCGGTGTCGACCTTGAAGAAGTCGGTGTCGATACGGTCGGTC

TCGCCGATGCTCGGATGAAGGAGCAAGATACCTGTGCCGCCCGGAATCGT

CGCGGGCGCGTCTTCGATGGCGAGCTCGTAATCCATACCCCAAGCAGTTA

CCCTGCCGACTTAATCCTTCATGCCGCTCGGGCGGCGTCGTCCCGGAATC

GTAACGTATGTTCACGCCGCCGGGCGAGGTCAGAACAGCGAGTCGGCCGT

GGCCGCGCCGATGACGCTAAACACCGCGCCGACGCTCGTCGCCTTGAGCG

TGATGCGGAGCACCGCGAGGTCGAGTTCGTACCCGAGCACGGAGACGAGG

ACTTCTCCCGACACGTCCGAGAGGAACGTCCCCGGCGCGTCGAACGCGAG

CGCGAGGATGAACACGGAGAGATACGAGACGGAGATGAGCGAGATGAATC

GGACGGGAATCCCGCCGACCTCGCGCTCTCTGTCGGGGTCGCGGTCGTCG

GCCTTGTAGAGCGCGCCGTAGCCGACCGCGAGCACGATGATGAGCGTGAG

AAGCGCCTGCGCGAACGACATGCTCCGCGCGAGCACCCACACCTCCTCGG

TGACGACGAACGGCCCCGCGAGGAGGAAGCCGCCGACGACCTGCTGGGCG

GTGTCGGCGAGCGCGAACCGACGGCGGACTCCGACCATACCCCGTCGTCG

TGACGGCGGCAATTAAAACGCGCGACGGGGGGGCGCGACCGGGTCGTCGT

CGGACTCGCCCGTCTGTGCGGTCCGGTCCGAACGCATTTTCACCCCGGCG

CGCGAGGCTCCGATATGAGCGTCCGCGACGAGTTCGACGCCTGGGCGGCC

GACGGCCGCGACAAGGGCATGGAGGACCGACACTGGCACACCGCGAAGCA

CGCGCTCGCACGGATGCCCGTCGAGGAAGGCGACACCGTCGTCGACCTCG

GGACCGGAAGCGGCTACGCCCTCCGCGCGCTCCGCGACACGAAGGGTATC

GGCCGCGGGTTCGGCCTCGACGGCTCGCCCGAGATGGTCCAGAACGCCCG

CGCGTACACCGACACCGACGACCTCTCGTTTCTCGTCGGCGACTTCGACG

ACCTCCCGTTCGACGACGACAGCGTCGACCACGTCTGGTCGATGGAGGCG

TTCTACTACGCCGCCGACCCGCACCACACCCTCGAAGAAATCGCTCGTAT

CCTCAAGCCGGGCGGCACGTTCTACTGCGCGGTCAATTATTACGAGGAGA

ACGTCCACTCCCACGAGTGGCAGGAGCACATCTCCATCGACATGACCCGC

TGGTCCCACGCGGAGTACCGCGAGGCGTTCCGCGACGCCGGCCTCCACGT

CGCGGAACAGGACTCCATCGCCGACCTCGACATCGACATCCCGGCGGCGA

CCGAGTTCCCGACCGACGACTGGGAGACCCGCGAGGCGATGGTCGAGCGC

TACCGGACGTTCGGCACGCTCCTGACCGTCGGCGTCGCGCCCTGAGACGG

CGGAAAAACCGACCGTCTCGACCTATTCGACGACGTGAGCCGACGCCGGA

GCGAATCCGACTTCCAGCGTCGCCCCGACCTCGAACCCCGCGTCCGACTG

TTCGCCGCCGTCTTCGACCGCCACGACGAGTTCCGTTCCGCCCCAGTCGA

GTCTGACCCGCGTCGTCGCCCCCTGAAACTCGGTGTCGACGACGGTCCCC

CGAATTTTGTTCGTCCCCGCGCCGGCCCGGAGTTGTTCGGGGCGGACGCA

GAAGGTGAGTTCGTCGCCGGCTCCCGCTCTCGACCCCGCCTCCCCGAGCA

CCCGCGGCCCCTCGGCCAGCGTGAACACCGCGTCGCCGACGCCGACGCGA

ATCCCCCCCGTTTCCGGTCGAGACTCGACGACCCCGTCGAGGACGTTGTT

CTCGCCGAGGAACTCCGCGACGAATCGCGTCCGCGGGCGGTGGTAGAGGT

CGCGGGGGTCGCCGACCTGCTCGACGCGGCCGCCGTTGAGGACGGCCACC

CGGTCCGAGACCGCCAGCGCCTCCGACTGGTCGTGGGTGACGTAGACGGT

CGTCACGCCGAGGTCCGACTGGATGCGCTTTACCTGCCGGCGGAGTCGGT

CCCTGAGTCGGGCGTCCAGCGCCGACATCGGCTCGTCGAGGAGCAGCAGG

TCCGGGCCGGGCGCGAGAGCGCGGGCGAGCGCGACGCGCTGTTGTTGGCC

GCCAGATAGCGAGTCGGGGTCGCGGTCTTCGAACCCCGCGAGGTCGACGA

GGTCAAGCAGTTCCGCGACGCGGTCGTCGCGGGAGCCGCCGCCCGGCGGG

TCGGTAAAGCGGAGGCCGTAGGCGACGTTCTCGCCGACGCTGAGGTGCGG

GAACAGCGCGTAGTTCTGGAACACGACGCCGACGCCCCGCGACTCGGGCG

CGACGCCGACCATCGACTCGCCGTCGAAGCGGACGGTCCCCTCGGTCGGG

GACTCGAAGCCGGCGATACAGCGGAGCGTGGTCGTCTTCCCGCAGCCGGA

GGGGCCGACGAGCGTGAAGAACTCGCCGTCGTCGACCGACAGCGAGACGG

AATCGAGCGCCGTCGCCGACCCGTAGCGTTTGGACACCGCGTCGAGTTCG

AGTCTCACGGGTTCGCCTCCGAAGTCACGTGCTCCCCTCGCCGGCGGACG

GGCATAATTCTCACTTCCCGCGGTTCGTCTCCGTCTTGTTCTCCGGGCTC

CCTCATCTCCCTCATAGCTCGCCCCACCGGCCGCCGAAGCGGTCGACGAC

GAGGAAGCTGGCGGACGTGACGAGAAGCAGGAGACAGCCCATCGCCGTCG

CGGGGCCGAGCCGTCGACCGAGGAATCGCTCGACGGCGACGGGCATCGTG

TAGCTTCCCGCGCCCTCCGCGAGGATGATGGTCGAGTCGAACTCGCCGAT

GCTAATGGCGACGGCGAACGCCGCGCCCGCGACGACGCCGGTCCAGACGA

GCGGGAGTTCGATATCCACGAGCGCGCGGGTTCTGGTCGCGCCGAGCGAG

CGCGCGGACTCCACGAGCCGGCCGTCGAGCCGGGCGAAAAGCGGCGCGAC

GTTCCGGGTGACGAAGGGGTACGCGCCGACGGCGTGGGCCGCGACGATGG

CGAGCGCGCCGGTGACGCGGATTCGCGTGCCCAACACGTCGACGCCGAAC

ACCAGCCCGCGGAGCAGGCCGAGGCCGACGACGATGCCCGAGACGGCGAA

CGGAGCCATCGAGAGCACGTCGATGAGCCCGCGCCCGCGGTACCGGCGGG

TCGTGAGGACGGCCATCGTCACGCCCATCGGGACGGCCACGACGAGCGTC

CCCGCGGCGAAGCCCAGCGAGTTCAGGATGGCCGGCAGGGGCTTGACCTG

AAAGCTCGCGCCGGTCGCCTGCCGCTCGGCGAGGAAGGCGTAGTTGGCGA

GGGTGAGGCCGCCGTCGCCGCCGGTGACGCTCGCCAGCACCATGCTGGCG

ACGGGGACGACGAACACCAGCCCGACGACGAGGGCGTAACCGCCGATGCC

GACCGTCCGGAGCGTCGATTTCGGCGTCCAGTCAGCGGGAAGGACGGACT

GACGGGGAAGCGGGTTCGCTGCGCCGCCGGCCGAGCGCTGGCTGGCCTCG

TACCGGAGGTAGACTGCGGTCAGCGTAATCGAAACGGCCGTCTCGACGAC

CGCGAGGCTGGCGGCCTCGGCGTACGCGAGGTCCCGCACCCGCGAGTAGA

CGAACACCTCGATGGTCGCCAGTTGGAAGCCGCCGAGCGCGAGGACGATG

GGGAACGACGCGAAAGTGAAGATGAACGTCAGGGTCGCGCCGACGCCGAT

AGACGGCAGGAGTTGGGGGAGGACCACGTCGCGGAAGGCCCGCCGCGGGT

TCGCGCCGAGCGAGCGGGCGGTCTCGACGGTCCGGGCGTCCACACTCTCC

CACGCGGCGGTGACGACGCGGGCGACGAGCGGGGCGTTGTAGAACGCGTG

GGCGACGACGATGGCTTCGAGCGTGAACAGCAGTTCGACCGGCGGGAGGC

CGACGAGAGAGAGCGCGCGGTTCAGCGTCCCGTTGCGGCCGAACGTCGCC

ACGAAGCCGATGGCGACCATGATGGAGGGCATGACGAACGGGAGGATGGT

GAGCGAGCGGAGCGTCTCGCGGCCGCGGAACTCGAAGCGGGCGAACAGCC

ACGCGGCGGGGAGGCCGAGCGCGAGGCTGGCGAGCGTCGAGTAGAACGCC

TGCTTCGCCGTGAACCAGATGATGTCGACGAGGTAGAACTCGCTCGTCAG

CACCGCCGCGATGGGCTCGACGGTGAGCCGGCCGTCGGCGAGGACGGCGT

CCGCGAAGACGGTGGCGACGGGGTAGTAAAACAGGACCAGAAGGACGACG

GCGGTGAGCGCCGCGACGAGGGTGAGCAGCCGCTGTTCGAGGGCGCGGGC

GACCCGGCGAACTGAGGCCGAGGCGCGGCTCACCGGCTCACCTCACTTAC

TGGCGAACTCGCGGGCCCACGCGTCGGTCCAGTCACTCAGGTTGTTCTGA

AGCCGGTCGTACGAAAACGTGACGGCTTCCGGCGGCTCTTGGGCGTACTG

CGCGAAGTCCTCGGGGAGTTCGGCGGTGGTCGTCGCCGGGAACTGGACGT

TGCGGACGGCGATTTCGGCCTGCACCTCGGGCCGGAGCATGAAGTCCATG

AACTCGACCGCGAGGTCGGGGTTCGACGCGTCCGCGAAGGGGGCCATCCC

CTCGGGGTTGGCGTACGCCTGGTCGTTCAGGAAGCGAATCTGGTGTCTCG

CCATGTCCTCGCCGGACTCGGCGGCGAACACCTGGTCGGTCGAGTACGAG

ACGACCATCGGGGCCTCCCCGTTCGAGTAGGCGTTGTAGGTGTCCTCCCA

GTTGCCGAGGACGCGCACGTCGTTTTCCTTCAGGCTCGCCCAGTAGTCGA

GATAGCTGTCCTCGCCCTTGGCGTCGATGGTGTGGAGCAGGAACGCCTGC

CCCGTCGCCGACGACGTGGGGTTCTGCGCGAGGAGCGCGCCCGCGTATTC

GGAGTCCAGCAGGCCGTCGAACGTCTCGGGGGCGACGAAGTCGCCGTCGC

CGTAGGTCTCGTCGTAGACGAGCGAGATGTAGCCCGTGTCGTAGGGGACC

GCTCGGCCCTGCGGGTCGAAGTTCAACCCCTCCTTTACGTCGTCGATGCG

GGACAGCCCCTCGGCGGGCGAAAACAGCGCCTCGTCGAGCTCCCGGTCGA

TGCGGACGAGCATCTGCGCGTCGAGGCCGACGTAGAGGTCCGCGCCGGAC

TCGACGCCCCGGAGGCCGCGCTCGATGTAGTAGTTCACGCCCGAGTCGGG

CGTCTGCCACTCCAGCGTCGCGTCGAACTCCGACTCGAACGTCTCTTTCA

GCCACGGGCCGGGGCTCGAACTCGGCGCGTCGACGAACGAGCCGTAGGTG

CCGACGGTGAGCGTCCGCGACTCGCCGCCCGCGGTCGTCCCTTCGGTCGT

CGCTGTCGTCTCGGTCGCGTCGCCCGAGGCCTCGGTCGTCGTCTGCTCGC

CGCCGGTCCCGGTACAGCCGGCGAGGAGCGCGGAGACGCCTCCCGCCCCG

GCGGCCCTGAGGAAGCTACGTCGTCTCATTACTCGGTTGTTGTACTCTGT

GGTACTTAACGGGCGTGATGTTCGCGGGGCCGGGTAAACGGCCGAATCAG

TCGGTTTCGAGGGTTGTCACGGGAGAACGGCTTTACCGTCAGCGAGAGTA

TCACGGGCGATGACTGCTTCTCGCCCGTACGTCCTCGGCGGCGTGTTCGC

CCTCTTTGCGCTCCTCGCGGCGGTCCTGCTCGTAGACGTGCTGGCGACGG

TGTTTTTCGCCATCACCGTCGCCTACCTGCTCGTCCCGCTCAGGCGACGC

CTCGAAGCGTGCGGGGCCTCCCGGTGGGTCGCCAGCCTCGCCGCCACCGT

CGTCGCGGCGGTCGGCGTCGGCGTCGTGCTCGCGCCGCTCGTCGTCATCC

TCTTTCTCCGCCTGAGTGACATCCTCGAACTCGCGGCGCTCCTCCCCGAC

GTGGTGACTATCGAGTTCCTCGGGATGGTCGAGACGGTGACGCTGGACGA

CGTGGTCGCGGTCGGTCTCGGTCTGCTCCGGTCGGTCGGCCGCACCGCCG

CCACCGCCGCGCCCGTGGTCCTCATCAAACTCACGCTGTTCGGCTTCCTC

GTGTTCGCGCTGCTCCTCAGCGGCGACGCGGTCGGGCGGACGCTCCCCGC

GCTGGTCCCGGCCGACTACCGGGGCGCGGCGACGGCGCTCAACGAGCGGG

CTCGCGAGACGCTGTTCGCTATCTACGTCTTGCAGGCGGCGACCGCCGTC

GGCACGTTCGCCATCGGCCTCGTCGTCTTCTGGGCGCTCGGCTACGACTA

CGTCGTCACGCTCGCGACAGTCGCGGCCGTTCTCCAGTTCATCCCCATCG

TCGGCCCGAGCGTCCTGCTCGCGGCGATGGCCGCCTACCACGCCGCCGTC

GGCGACGTCGTCGCCGCCGCGCTCGTCGTCGCGCTCCTCGCGGAGTCGGT

CGACCTGCTCGGCGCGGAGTTACGCGTCGACGACGTGGGTGGTGACGGCG

ACGAGCGCGACGAGTCCGCGGACGACTCGGTCGACCCGAGTCAGCCGTAA

CCTCGCTCGTCAGCCCTCGACGACCGCGCCGTCGCGCTCCTCCCACTCGG

TGAGGCTGCCCTCGTAGAACGCCACGTCGTCGTAGCCGAGGTGAGAGAGC

ACGACGTAGGTGTGGCTGATGCGCCGGGCGGTGTTGCAGTAGAGGACGAC

CCGCCGGTCCGGGGTGACGCCCACGGCGTCCAGAATCGCGTCGAGTTCGT

CCCGCGGTTTCAGCCCGCGCGTCTCGTCGTCGACGAGTTCGCGCCAGTCG

AGGTTGACCGCGCCGGGGAGGTGCCCCTCGTCATACTCCGCGGGGTCGCG

GGTGTCCACGATGACGGTCTCGGGGTCGTCGAGCGCGGCCTCGACGGCCT

CGAAATCCACGAGCGGCGTCTCGGCCGGCTCGGTTATCTCGTAGACCGTT

CCCGCGACCTCGCTCGCCTCGGTCGTCGTCTCGCGCTCGCGGTTCCACGC

GCTGAAGTCGCCGTCGAGGAGGTGGAGCCTGTCGGGGTCGTGGCCGTAGA

GGAGCGCGGTCACGAGAAAGCGCGCGGCGAAGACGCCGTGGGTGTCGTCG

TAGGCGACCACGTCGTCGTCGGCCGCGACGCCCGCGCCCGACAGGAGGTC

GGTCCACGCGTCGCGGCCGGGAAGCATCCCCACGTCGCCGTCGGCGCTTC

GGAACTCGTCGAAGGGTATCGACACCGCGCCGGGGAGGTGTCCGATGCCG

TCGAACTCCCAGCCGTCGCGCACGTCCACCACGCGCACGTCGTCGAGTCG

GTCCGCGAGCCACGTCGGAGAGACCACGTCTACCATGCCCCCACGTTTCC

GGGCCGCGGATTTAGGTCCGCCCCTCCTCGCACCCCTCGCCCCATGTCCG

GGGTAGCGGATGTTGTTGCTGGTTCCGTTGGATACCGGACTGAAACCCAC

CGAGACGGCCGTTCTCTGAATCCCTTTCGTCGTCTCTCGGGGCATACTCA

GGCTAATATTGCCGCATCACCCGAGGTCAGGCCGTACATGCCGTCGCTGT

GAGAGTTATGGGCCTTCCTGTTGTACGGTGAAACGCGATGTCAAACTCCG

ATTACGCGAAGGACGTACTCGTCTCTGCGGACTGGGTGGAGAGCCACCTC

GACGAGTTCCAGAGCGACGACCCGGCGTACCGACTCGTCGAAGTGGACGT

GGACACCGAGGCGTACGACGAGAGTCACGCCCCCGGTGCCATCGGGTTCA

ACTGGGAGTCCCAGCTGCAGGACCAGACGACCCGTGACGTGCTGACCAAG

GAGGACTTCGAGGACCTCCTCGGCTCCCACGGCATCTCCGAGGACTCTAC

GGTCGTCCTCTACGGCGACAACTCCAACTGGTTCGCCGCCTACACCTATT

GGCAGTTCAAGTACTACGGCCACGAGAACGTTCACCTGATGAACGGCGGC

CGCGACTACTGGGTCGACAACGACTATCCGACGACCGACGAAATCCCGTC

CTTCCCGGAGCAAGACTACAGCGCCAAGGGTCCCTTCGAGGACATCCGCG

CGTACCGCGACGACGTCGAGAAGGCGGTCGACAAGGGTCTTCCCCTCGTC

GACGTTCGCTCGCCCGAAGAGTTCTCCGGCGAGATTCTCGCGCCCCCGGG

ACTGCAGGAGACCGCCCAGCGCGGCGGCCACATCCCCGGCGCGAGCAACA

TCTCGTGGGCCGCGACCGTCAACGACGACGGCACCTTCAAGTCTGCCGAC

GAACTCCGCGACCTCTACGCGGACCAGGGCATCGAAGGCGACGAGTCCAC

CATCGCCTACTGCCGCATCGGCGAGCGCTCGTCCATCGCGTGGTTCGCCC

TTCACGAACTCCTCGGCTACGAGAACGTCACCAACTACGACGGCTCGTGG

ACGGAGTGGGGCAACCTCGTCGGCGCGCCCGTCGAGAAGGGTAACTGAGC

CGCCTCGGCCTCAGTTCGTTTCGATACCGACCGACCGGTTCTTTTTGGGG

TTCACATCGGCAGGACGCTGATGCCGACGGCGACGAGTACGCCCGCGCCG

AGAAGCACCATCACGCAGTAGCCCATGATGTCGCGGACCGACAGGCCGCT

GATGGAGAGCAGCGGGATGGCCCAGAAGGGCTGAATCATGTTGGTCCACG

CGTCGCCCCACGAGGCGGCGACGGCGACCCGCGGGATGGACTCGCCGGAC

GCCTTCGCGGCCGTGACGAGCGTCTCGCCGATGACGGCCCACTCGCCCCC

GCCGGAGGGGACGAAGAAGTTCACGAGGCCGGCGGTGAAGAAGGCAAAGG

CCGGGAGCGTCCCGTCGGGCGCGACGGCGACCATGCCCTGTGCGATTTGC

GTGGCGAGGCTGACGGAGCCCTCCGGCGCGTAGGCCATGATGCCCATGAT

GCCCGCGTAGAAGGGGAACTGGAGGATGATTCCCCAGACGTTCTCGACGG

CCTCGACGACGGCCTCGATGTACGCCTTGGGCGTCCCGTGGAACAGCACG

CCGAGAAAGAGGAAACCGAAGTTGACGATGTTCAGGTTGAGATTGTTCCA

CGGCATCGTCCCGTTCTGGACGCCCTCCCAGAAGTACAGGGCGACCGCGA

GCAGGCCGACGACGCCGATTGCCACGCCGATGCCGAGCGAGTGCTCGATG

CGCGTCGCGAGCGAGGCGTCGTCGGGGACGGCCGTGGAGGCCCACCCGCC

GGTCGCCTGCTCGCCGCCGTCGGTCGCCGTCTCGAACGCCGCGGGGTCGA

TGGGCGTCTTCTTCGCGTCGTCGGTCGGGTACATGAGCGCGAACAGCGCC

GGCAGGAAGAGGAAGCCGACCGCGACGACCAACACGAGGTTGGCGACCGT

GAAGATGGTCCCGCCGGTGCCGAACGTCGTGTCGAGGATGCCCGCCTCGA

TGAGGAAGTTCCCCTCGGTGTTCAACAGGAGCGGAATCGACCCCGCGAGA

CCGCCGTGCCAGACGACGAACCCGGAGTACGCCCCGGCGACGACGATGGG

GAAGTCGATGCCGCGCATCTCGGTCGCTATCTTCCGGGCGAACAGCGCCC

CGACGACGAGGCCGAGGCCCCAGTGGACGAACGACGCCCCGGCGGCGACG

ACCGGAACCATCGCGGCCGCGCCCCGTTCGGTGTTCGGGACGCCCGCAAG

TCGTGTCAAGAGCCAGTCCACGGGCTTCGTCTGCGCGAGCGCGTAGCCCG

TCATCAGGATGAGCGTCATCTGCATCCCGAACGAAAGCAGGTTCCAGAAG

CCCCCGTACCAGCCGTCGAGAAGCAGGTTTCCGGCGTGCCCCACCATGCC

GACCCCCTCGGCCGGCGCGACCGACACCAGCGCCAGCACGAACGCCACTC

CGGTCAGGATGATGGCGAACAGGAACGCGTCGGGGAGGTACTGTTCGACC

AGTTTCGAACTGCGCTCCGCCGCCTGCCTGATAGCGTTTGTCATAGCTCG

GATAACCTCATGATAATACAATATATAATGTTTAATATTCCTGCTAGTTC

GATTCGGTAGAATGTCACGAACCGGCCGACACGCCGGTCGTCGTCTGGGG

TCGGGTCGTCACAGACCCGCGCCGGACTCGCCGCCGCCGATGGGTACAAA

CCCGAGACGGACATACGGGGCGTATGTCCGAACTCCTCGACACCCTCCGC

GACGACCACGAGACGCCGCTTTCCCGACTCGGCTCCTCGAAGGCGCTGTA

CGCCGTCACCGGCGGCGAGATGGACGGCGACGCGGTCCGCGCCGCGGCCG

CCGCCGAGGCCGCGGCCGCCGCCGACCTGTTCGACTGGTGGGCAGACGAC

GAACCGAACGACGAGGCCGCCGCGCTGTTTTCGGACCTCGCCGACACCGC

CCGGGAGCACGCCGAAACCGTCGGCGCGGAGTCCGACGGCTCGAAGCCGA

ACGTCTACGACGTGCTCGCCGAGTTCGAGACGACCGACGGCCGCCTCGGC

GGCGCGCTCGCCCGCGCGCTCGTGTCGCTGAAGACCGTCGAGCAGATGGT

CGGCTTCTTCGTCGGCGACGCGGACCCGATGGCCGCCAACGACTTCCGCA

CGCTCAAATCCGATCTCAACGACCAACTCGACACGCTCGAAGCCGCCGTC

TCCGACCTCGTCGACGACGACGCAGTCGCCCGCGAGGCCGCCGACGCGGT

GGTCGAAGCCGCCTACGACGAGTACGTCGAGACGCTGGAAGGCATGGGCG

TCAAGCCGAAGAACGTCTGCTAAGCCCCCTTTTCACTCACAGAGGGTCGC

CGTTGGCGACCCACCCGGAGTAAACCGTCTCCGTGAACGGTAGCGGAACG

ACGGCTCGTGAGACGCAGCGCGCCTCACGGTGGGGAAAACAGCCGCGAGA

CTCGACTTCGGCCTGGCTCGCTGTGCGTTATGCTCGGAACTTGTATCTTC

GTTCGCCCAGTGCTGACAGCACCGACGCGCGGCGTATGTGAGTGAGTTCC

AGTAGAAACGAGGGGGTATGTCCGCCGGTCGTCAGACGCCGGGGACGCCC

TTGCGGTATTCGAGGACTTCGCTTCGGGCCTCGCGGACCGTCTCGGCCGC

GTCGCCGTCGAGGTCCTCGGCGAGGTCGTGAAGGACCGTCATGTGCCGGG

CGAGTCGCCCGTGGTCCGGCCCCTGCTCGCGCGTCGCCAGCGTCGCCAGC

TGGTCGGACTGTTCGTAGAGTCGCTCCTGCACGTCGCCCTCGGTCGCCTC

GGCGGCGTCCTTGAGCAGGTTGCTCGCGCGTTCGAGTTCGGCTCTCGTCA

TACGGGAACGTTCGATAGCCCGCCACAAAACCGTTCCTCCGGCGACAAGC

ACCGCCTCGCTCAAGTACGCCGCGACCCAACGACGGGCAATGAGCGAGTC

CGGTCCCCTGTCCGCCGACAGACCCGACGCAGACCGCGAATTCCGCGTCG

ACGCCCCCTTCGACCCCGCGGGCGACCAACCCGAGGCAATCGAGGCGCTG

GCCCGCGGCTTCCGCGAGGGTGCCGACGTACAGACCCTGCTCGGTGTCAC

CGGCTCCGGCAAGACCAACACCGTCTCGTGGGTGGTCGAGGAGATTCAGA

AGCCGACGCTCGTCCTCGCGCACAACAAGACGCTCGCCGCCCAGTTGTAC

GAGGAGTTCAAGGGCCTCTTTCCCGACAACGCGGTCGAGTACTTCGTCTC

CTACTACGACTACTACCAGCCCGAGGCGTACATCGAACAGACCGACACCT

ACATCGACAAGGACATGTCGATAAACGAGGAAATCGACCGGCTCCGCCAC

TCCGCGACCCGGTCGCTTCTCACCCGCGACGACGTCATCGTCGTCGCCTC

GGTGTCGGCCATCTACGGCCTCGGTGACCCGAAAAACTACACCGACATGT

CCCTCCGGCTGGAGGTCGGCCAGGGGATGGACCGCGACGAACTGCTCCGC

GCCCTCGTCGACCTCAACTACGAGCGCAACGACGTGGACTTCCGGCAGGG

AACGTTCCGCGTCCGCGGCGACACTGTCGAGGTGTTCCCGATGTACGGCC

GCTACGCCGTCCGCATCGAGTTCTGGGGCGACGAAATCGACCGCATGCTG

AAGCTCGACCCGCTGGAGGGCGAGGTGAAGTCGTCGGAGCCGGCGGTGCT

CGTCCACCCGGCGGAGCACTACTCCATCCCCGAAGAGCAGCTCGAAGGCG

CAATCTCGGAAATCGAGGAACTGATGGAACAGCGGGTGAAGCACTTCCAG

CGACAGGGCGACCTCGTGGCCGCCCAGCGCATCGAAGAGCGCACCACCTT

CGACATCGAGATGCTCCGCGAGACGGGTCACTGCTCCGGCATCGAGAACT

ACTCGGTCCACCTCTCGGACCGCGAACCCGGCGACGCGCCCTACACGCTT

CTGGACTACTTCCCCGACGACTTCCTCACCGTCATCGACGAGTCGCACGT

CACGCTCCCGCAGATTAAGGGCCAGTACGCCGGCGACAAGTCCCGAAAGG

ACTCGCTCGTCGAAAACGGCTTCCGCCTGCCGACGGCCTACGACAACCGC

CCGCTCACCTTCGAGGAGTTCGAAGAGACCGTCGGGCAGACGCTTTTCGC

CTCCGCGACGCCCGGCGACTACGAGCGCGAGCACTCCGACCAAATCGTCG

AGCAAATCGTCCGGCCGACCCACCTCGTGGACCCGAAAGTCGAGGTGACG

GAGGCGACCGGACAGGTCGACGACCTGATGGCCCGCATCGACGAGCGCAT

CGACCGCGACGAGCGCGTGCTCGTCACGACGCTCACGAAGCGGATGGCCG

AAGACCTCACCGAGTACCTCGAAGAGGCCGGCGTCGACGTGGCCTACATG

CACGACGAGACCGACACGCTCGAACGCCACGAACTCATCCGGTCGCTCCG

CCTCGGCGACATCGACGTGCTCGTCGGCATCAACCTCCTCCGGGAGGGCC

TCGACATCCCGGAGGTCTCGCTCGTCGCCATCCTCGACGCCGACCAGCAG

GGCTTCCTGCGCTCCGAGACCACGCTCGTCCAGACGATGGGTCGCGCCGC

CCGCAACGTCAACGGCGAGGTCGTGCTCTACGCCGACGAGATGACGGACG

CGATGGAGGCAGCCATTTCCGAGACGCAGCGCCGCCGCCGCATCCAACAG

GAGTTCAACGAGGAACACGGCTACACGCCGACGACCATCGAAAAGGAGGT

CGGCGAGACGAACCTGCCGGGGAGCAAGACCGACACCCGCGGCGTCTCCG

GCGACGAACCCGCCGACGCCGACGAGGCGGTGGAACAGATAGCCTTCCTC

GAAGACCGGATGCAGGAGGCCGCCGACAACCTCGAGTTCGAACTCGCCGC

CGACATCCGCGACCGCATCCAGAACCTCCGCCGCGAGTTCGACGTGGACG

CGCTCGAAGACGGCGTCGCGCCCGAGTACCCCGACGAACACGACGGCGAG

GCCGACGACGGCCTGACGCCCCCGGACGAGTTCTGATCGGTCGTGTCCTC

ACCACCGACCCTGACGTTCGCGGCCGGCGGGCTTCTCCGCCGCGACGACG

GCCGACTCTGTCTCGTCCACCGGCCGCGCTACGACGACTGGTCGCTCCCG

AAGGGGAAACTCGAACCCGGCGAGACGCTGGTCGAGACCGCCGTCAGAGA

GGTCCGCGAGGAGACGAGATGCGAGGTCGACTGCGGCCGGTTCGCCGGGC

GCTACGAGTACCGCGTTCCCGACGACGCGGGGACACGGAGCGGGCCGAAG

GGCGTGTTCGTCTGGCACATGCGCGTCGTCGACGAGCACCAGTTCGAGCC

CGACGCCGAGGTGGACGCGCGCCAGTGGGTTACCCCGGTCGAAGCCCTCC

AACGGCTTACCTATGAGACCGAACGGGCGCTGGTCAGACGGGCGTTCGAA

CTCAACGAGTGAGCGCAGATTTTGGATAGAAACACATGTTACTGGTTTTT

CGACGCGCGTCGGTGTGTGGTCCGTACCCAAATCAGTGGGTCGACATCAA

GACTTATTACTCCGACTTCGCTATGCCCGTTCCACCGTGATCCGCACCTC

GGCGGACGAGCCTGCAACCCACACCGTTCACCACGCCTACCCCAACGGAG

GCATTACCTGAATGGTCCGCGGATTCGAGCACGGAGTGCTCGATAGTTCG

GGCGACCACCGCACGCCGGCCCATCCGGCGTGCGACGCGGTCGCATCCTC

GGGAGACGGTGAACACGCGCGGGTCGGTGCCGCTGATGACTCGGCGAACG

GGCTGTTTTCGACGTGCCACTCCACGCGAGTGCAAGCGAACGCTACTGCG

TACTGATAACAACGAGAACCATGAGCTCTCAGTCTCTCAGTCACGTCCAG

AAGTCGTTCCTGAAGTACCAACACATCCTCGTGTTCATCGCTCCCCTGCT

GTTCCTCGCCTCGGTGTTCACCATGGCACCGACGCCGTCGGGTGTAGGCA

TGGAGTACTGGCTCCAGTACTGGTGGCTGTTCCCGGTGTTCCTCACCGGC

GCGACCATCGTGAACACGGTCGGCATCAGCGGGTCGGCGCTCTTCGTCCC

GTTCCTCATCTTCATCTTCCCCATCTTCGCGCACCCGCTCGACTCGTCGA

CGCTGGTGAAAGTGGGCCTCATCAGCGAGGCGTTCGGCCTCTCCAGTTCG

GCCGTCGCTTTCATCCAGTACGGCCTCGTCGACCGGCGACTGGCGCTGAC

GCTCGTCGGCGGCTCGATTCCGTTCGTCGTCGGCGGCGCGCTCCTGTCGT

TCGTCATCCCCGACGTGGTGTTCCACGCGCTGCTCGGAATCGCGCTGCTC

GCCGCGTCCTACCTGCTGTTCAACGCCGACCTCGGCCACGACGAACCCGG

CTCGTCGGACACCGACCACGCCGCGGCCACCGACGGCGGCACCGTCTCCG

CGAGCCTCCCCAACGACCCTGGTAAGCTCGGTCCCGCGGGCGTCAACACG

GCCGACGACGGGACCGTCACCCGCGTCGACCGCGACGGTGACGACTACAC

CTACACCCGCGGCGGCTACCTGCGTCGCTTCGCCAATTACAGCGTCGGTG

GGATGTTCCAGGGCCTCGCCGGCTTCGGTATCGGTGAACTCGGCATCATC

TCGATGCTCGGCACGAAAGTCCCCGTTCGCGTCGCCATCGGCACGAACCA

CATCGTGGTCGCGCTGACCGCCATCTTGGCGTCGCTCGTCCACGTCTTCG

GCGGCGGCCTCGTCGGCGGCCACTCGCTGAGCCTCGCGACGACGCCGTGG

AACATGGTCGTCTTCACCGTCCCCGCGACGGTCCTCGGCGGCCAAATCGC

GCCCTACGTGTCGAACGCGCTGGAGACCAGCGTCATCAAGAACTTCGTCG

GCGTCCTCTTCGCCGTCATCTCGCTGGCGCTGTTCCTGATGGCGCTGGGT

ATCTAACGACTCACGACCACCCGAACCACCCCCACTGGCACCACTTTCAC

AGTCCTCGATTCTGAACGCTCACCCATGTACGACCACATCCTGCTTCCGA

CCGACGGCAGCGACGCGACCGACGCGACCATCGAACACGCGGCGACCTTC

GCGGAGACCTACGGCGCGACGGTCCACGTGCTGTCGGTGGCCGATTCGCG

CAACCGCTTCGAGTCGCCCTCGGCCGGCATCGCGCCGGACGTGTGGGAAA

AATCCGAACTCGACCGCGCGGAGTCGGCGGCCGACGCCGCAATCGAGGCG

CTCCCCGAGGGCGTCGAGACCGAACGCATCGTCGTGGAGGGCGTGCCGCA

CTCGACTATCGTCGACTACGCCGCCGACGGCGACATCGACCTCGTCGTGA

TGGCGACCCACGGCCGCACCGGTCTCGACCACTACCTCGTCGGCTCCGTC

ACCGAGCGCGTCGTCCGGCAGTCGGACGCGCCGGTGTTGACCGTCCGCGC

GGCGGACGAAGAATAAACACCCGCGGCTCGCTCGCGGGCCGCGTTACTCG

AACTCTTCTGCCAGCGCGGCGCGGTGTTCGCGGCGCTTCTGGAGCGCCGC

GTCGCGCAGTTCGCGCTCCTCTTGGGAGTCGCAGACGAGGTCCGGGACCG

GAACCGGCTTCTCGTCGTCGTCGAGCGCGACGAAGGTGAAAAAGGAGGTG

AGCGTCTCGCGGCGGTCGCCCTCGCTCGGGCGCTCGGCGGTCACGTCCAC

CTTGATGTCCATGCTGGTGCGGCCGGTGTCGAAGACGTACGCCTCGACGG

TCACCACGTCGCCCACGTCGATGGGCGCGAGGAAGTCCACGTGGTCCATC

GACGCCGTGACCACCTGCCGCTCGGCGAAGCGCCGCCCGGCGATGGCCCC

GCAGACGTCCATCCACTCTAAAATCCGCCCGCCGAGGGCGCGTCCGAGGT

TGTTCGTGTCGTTGGGCATCAGAATCTCGCTCATCTCGGCGCGAGACGCC

GACAACGACCGCGTCTCGACCGACGATTGCTGTTGCATGCCACACCGTCA

GAGTCGGACGTGGTTAAATTATTCTACCTCTTGAAAAATAGAAATAAAGG

CGATTAATCGCTCGGGTTGTCGATTGGTCGGTGAACGCCCGCACGTTTCG

ACAACCTAGACAATTAATAGTGTTTATTGCGTATCGAATGGTGTCTGCGC

GAACCGCAGACCGGTGGTGGGACGCCGACGCCCGTCTCTCGTCCGCGTTC

GGGAGGACGTTCCCGGAGGGTTCCGACTCTCGTTCGACCGGACGGGCGGG

AAATCAACCGCGACCCGAGAGTCGACGCACCGAGCCGCTCCCGTCTCACG

TCCGCCTCGCGCCTCACCGAACCGCGACGGTCGGACGACGGGACTCGCGC

CGGTCGCCCCCGCAGACCGGACACGACGCTGCACCGGCCCCCGGTGCGTC

GGACGGGTCTCCGACTGATTCGCGCCTTCGATTTTCGCTCACTCGACGCC

GCCGTCTTCGATTTCGATGTCGACCGCGTCCGCGGAGATATCGGCCGTCT

CGACGCCGTCCAACTCCCCGAGTATCTCGCCGATGTCGTCGAGGCCGAGG

AGTTCGCGGGTCTCCTCGTCGAACGCCTTGCTTTCGAGTCCCGCCGCCTG

CTGTACGTCCGAGCCGGACAGCCCCTTGCCGTAGCGGCCGAGCAGCGACG

TGAGCTCCTGCGGCAGGACGTACGTCGTCGACGGCGAGGTGCCGATGTTC

GCCAGCGTCTCCATGCCCTTGTCGATGATGGCGCGCTCGCCCATCGACTC

CGCCGCGCGGGCCCGAAGCACCGTCGAGATGGCGTCGCCCTGCGCTTCGA

GGATTTGCGACTGCTTTTCGCCCTGAGCGCGGATGATGTTCGACTGCTTG

TCGCCCTCCGCCTTCTCGACGGCGGAGCGACGTTCACCCTGCGCTTCGAG

AATCATGGCCCGGCGGCGGCGCTCGGCGGAGGTCTGCTGTTCCATCGCGT

TCTCGACGTCCTTCGAGGGCTTCACCTCGCGGACCTCGACGGACTCCACG

CGGACGCCCCACTCGTCGGTCGGCTCGTCGAGTTCCCGGCGGATGCGCGC

GTTGATGTGGTCGCGGCGCGCCAGCGTGTCGTCGAGTTCCATGTCGCCGA

GGGCGGCTCGGAGCGTCGTCTGGGCGAGCAGCGAGACGGCCCGGCGGTAG

TTGTCGACTTGCAGGAAGGCGCGTTCGGGGTCCATCACGCGGATGTAGAC

CACCGCGTCGGCCGTGACCGGCGAGTTGTCCTCCGTGATGGCTTCTTGGG

AGGGCACGTCGAGCGTCTGGGTCCGCATGTCGAAGCGGTAGGTCTTCGAG

ACGAACGGCGGGACGACGTTCAGCCCCGGTTCGAGGATGCCCTTGTAGTC

CCCGAACACGGTGAGCGTCCGCTTTTCGTACGCCTGCACGATTTCGACCG

CATCGTACACCGCGGCGACGGCGAGCGCGAGGACGACGTAGCCCGCGAGC

GTCAGCGGCGTGACGGGGAAGACGACCACCGCGAGGCCGAAGACGACCAC

CGCGACCGCGAGGGGGGCGAGCCACCGCGGGAGTCGGCCGGGGTCGCTGT

CGACCCGCTCCGCGCCCGACGACGAAGACGACGAGGAGGACGCCGAGAGC

TTCCCGAGCTGGTAGAACAGGCTGTCCATGGTGGAGAGTGACACCGAGAC

GGAATAAAGCTAGCCGGCTACTCACCTACCCACTCACCCGGCCACCAACT

ATTTGTCCGCGAACGCCCCACGCCCCGGTATGCGAACGAACCGCGGCCGA

ATCGACGTGGAGGACCTGCTGAAAATCATCCTCCTGCTCGTCCTCGTCTG

GCTCGTCCTCGAAATCATCGGCGAGGTGCTCGGGCTGTTCGGTGCGCTGC

TCGGCCCACTCCAGCCGCTTTTGGGACTCGTCGTCGCGGCGCTCATCGTC

CTCTGGCTGCTCGACCGAATCTGACGTTCCCGCGGCGACGATACGCCTTT

GTGTCTCTCGCGTGAGACACGGGCGTGTACAGTCTGAACGTCCCGGTTCC

GGGGCGGGTCGCCCGCCTCGCGTCCGACCTGTTTCCGTATCTCGCCCCGT

TCGACCGGGTTCGCGACCGGCACACGCTCGTCTGCAAGCGGTTCGAAGAC

GCCGACCTCGACCGCCTCCGCGAACAGTTGCGGCGCGCGCTCGCCGGCCA

ACCGGCGTTCGAAGCCCGCGTCACTGACGTCCGATTCTTCGAGGAGCCGC

CCCGCGGAGCCGCGCCGGTCGTCTACCTCGCGGTCGACAGCCCCGGCCTT

CTCGACCTCCACCGGACGCTCACCGACGATTTCGGCGCTATCGAGGGAAT

CGAGGGCGACGACTACGTCCCGCACGTCACGCTCGCTCGCGGCGGCAGCG

TCGCGGACGCCCGCGCGGTCGCGAGCCAACCGCTCGACCCCATCGAGTGG

ACCGTCTCGCAGCTCGATATCTACGACTCGTCGTTCCGCGAGACCACGGC

CTCGATTTCGCTCCCGGCCTGACGGCGAGGGTTCGACCGGCGTCGGCGGT

GCGCCCGTGTCCGCTGGTCGCGCCGTCAGTCGCGCGCCCGGAGCCGCATC

CCCATCGGCTCCTCCGGATGCATCGTCAGCGACCCGCGGAGCGAGAACGG

CTCGTCGCGGACGTAGTCGAGTTCGTACCGCTGGGCGACGGTGCCGAGGA

TGAGCCGCCCTTCGAGCAGCGAGAGGTGCTTGCCGATGCAGTGGCGCGGC

CCGCCGCCGAACGGGAAGTACGCGAAGCGCGGGCGGTCGCCGGCCCGCTC

GGGTGCCCAGCGGTCCGGGTCGAACTCCAACGGGTCGTCCCACCAGCGCT

CCGAGCGGTGGACGACCCACTGCGGGAGCATGATGGCCGACCCCGCGGGA

ACGCGGTAGCCGCCGAGGCGAACGTCCACCTTCGGCTCGCGGAACATGAC

GTACACCGGCGGATACAGCCGCATCGCTTCGTTCAGCACGCGCTCTGTGT

ACTCCAGCTGTCGCACGTCCTCGAACGTCGGCGTCCGCCCGCCGAGCACC

TCGTCCAGTTCGCGGTGGAGCTTCGCCTCCGCCTCGGGGTGTTGCGAGAG

CAGATACCACGCGTACGTCAGCGTCAGCGCCGTCGTGTCGTGGCCCGCGA

GGAGCATCGTCATCAGCTCGTCGCGGAGGTTCTCTTCCGTCTGCTCGCCC

TCGTCGTAGGCGCGGAGCAGAATCGAAAGCAGGTCCATCGGCCGCTCCTC

GCCCGTCGCGTCGGCCGGCACTGACGACGCCGGCGTCTCGCCGTACTCGG

TCCCGCGGCGCTCCTCGACGATGTCCCAGACGAGCGATTCGAGTTCGGAC

AGCGCCTGCTTGTACTCGCGGTTCTCCCGCGTCGGTGCCCAGTCGGGCGT

CAGGAACCGAAGCGGGTCGGGTTCGAACCGCGCGCCGAGCGGTTCGAGGT

TCTCCTGTACCCGCCGGATGCGCTCGTCGTCGAGGTCGGTGCCGAACATG

GCGTCGACGATTATCTCGACGGTCAGCCGCGCCATCTCCAGTTGCACGTC

CACCACGTCGCCGTCGCCCCACGACGACAGCATCGACTCCGTCCGGTCGG

TCATCATGCCGGCCATCGTCGAGATGCGCCGCACGTCGAACGCCGGCTGG

GCGAGCTGGCGCTGTCTCTTCCACGTCGCGCCCTCGCTCATCAGCAGGCC

GTCGCCCAGCAGGTCGCCGATGGCGCGGTCTTGGAACTGCGGCTTTCTGA

ACTTCGACGCCTCGCTCACGAGCACCGTCTCGACGTCCGCCGGGTTCGTG

AGCATGTACGTGTCGAGCGGGCCGAGGTCGAAGTGGACCACGTCGCCGTA

GGCGTCCGCGACGGCCGTTAGGAACGTAAACGGGTCGCGGGCGTACTGTC

TGCTCGCGCCGAACAACGGGAGGCCCTTCGGGCCGGGAGGGGTCGCACTC

ATCACCCTGTGTTGGTTCGAACAGGTAAGAATCGCACGGCGGTCGCGACC

GGCTTTTCCGCGCCGAGCGCGACCCGCTCAGGCTCAGGGCCGCGGCGGGT

CGCCGTCGTAGGCGTCGTGGTCGGTGTAGAAGTTCAGCATCGCGAACTTG

AGTTTGTTCGGCGCGATATCGACCATCTCGGAGCGTTCCTCGACCGGGAA

CGACCCGCGGACGCCGTACTTCCCCATCGAGGTGCTCTCGCGGGTGTACC

GCTTGTCGGCCAGCACGCGGACACCGAAGTCGTCGGGCGCGCGGATGACC

CGGCCGAGCGCCTGTCGGGTCTTTCGAATCGTCGGAATCTCGACGGCGTA

GCGCCAGCCGGCGTCCTTTTTCCGACGGTAGACGCGGTCGTAGGCGTCCT

GTACCGCCTCCAATCGCTCGGAGAGATGCGGGTAGGGTACGCCGACGACC

ACGACCGTTCGGGCGTCGTCGCCGTCGAAGCTCACCCCCTCCGCGAGCGT

CCCCCACAGCGAGGTGAGAAGCACCGCGCCGTCCTCGGCGACGAACTCCC

GGCGCATCTCCTCGGCGCGGACGCCCGGTTCGTCTAAAAACAGCTCCGCG

TCGACGTTCGGGTTCGCCCGCAGGCGCTCGTGGTAGCGCTCGGCCTCGGC

GTACGACGGGAAGAACGCGAGCGTGTTCCCCGGCGTGAAGCCGGCGGCGT

CCGCGAGCACCTCCTCGACCGTCTCCTGTGTGCCGGGGTCGTCGCGCTCG

GAGGAGAACAGCGCGGGCAGCGAGACCGAAAACGTCCGGCGGTTCTCCTC

GGGGTATTCGAGGCCGTAGGCCATCGTCACCGGGTCGTCGAGGCCGAGCG

TGCTTTCCGTCACGTCGAACGGGCGGAGCGTCGCCGACATCAGGATGCTC

GCGTGAACCTCCTCGAACAGCTCCTTCGTGACCTCCCGCGGGATACAGGT

GTACAGTTCCGCGCGGCCGTAAATCTCGTCGGTGCCGCCGTCGCGGCGGA

CCGACAGCACCGGGTGGCGGCCGAGTTCGTCGCCCAGCTCCGTCCAGTCG

GCGATGAAGTTCGCCGCCTGGAGCGTCTGGCACTCCTTGCGCGTCGTCGC

CTCGCCGTTCTTGTACGCGTCTTCGTACTGCTCGTCTAACTGCTTGCCCA

ACTGGAGCGCGAGTTCGACCTCCACGTCGATGCCGCGGCCCTCGTAGGAT

TGTAGGAACTCCATCGTCAGGTCGTCGCGACGACCCTGACTGGCGATAGA

GAGGTCGTACCAGTTCTCACCGACCTGCTCGCGCTCGCCGAAGCCGAACG

CCGATTCGTAGCTGTCGCGCAGCGCGTCGAGGAACGTTCCGATGACGTTG

CGGGCGCTCTCGGCCCGCGAGTCGTCTTCGTCTTCCAACTCGGTCATCGC

GCTTTCGAGCGTGTTCTCGGTGAGCGCGCGACTCGCGTGGTCGCGGGCCG

CGCCCTCGATGTTGTGCGCCTCGTCGAACACCGTAATCACGTCGTCGGGG

TCGCGGTCCAACCACCGGAAGAACTGCTCGCGTATCATCGGGTCGAGCAG

GTGGTGGTAGTTGCAGACGACGAGGTCGATGCCCTCCATGCCCTCCTTGA

GCAGTTCGTAGCCGCAGAGGTTCTGCTTGCCCGCGTACTCGAAAATCTCA

TCGGGCGTCCGCACGTCGTCGAACAGCCACTGGAAGAACTCGTCTGTGTT

CCGCGTGAGGTTGTTGTAGTAGTGCTCGCAGTAGTTGCCCTCCTTCAACT

CCGCCAACTCGTCGTCGATGGCGTCGAGTTCGTCCGTGACGGCGCTTCGC

GCGTCGGCCGCGCCGGACTCACCCTCTCGGATCCCGTCGAGGAGCGACTG

CGTCTGCTCGGAGAGTTCGGCCTTGTCGGACTCCTTTTCGACGATGCTCC

GGGTCGTATCGCGGAGGGTCTGACACTCCTGAAAACCCACGTCGATGTGG

CACATCGAGGACTTCCCGCGGAACACCACCGCGCGGATGGCCTCCTCTCT

GGTGATGGCGCGGGCGTCCTCGACGAACTGGCGCATCTGCTGGTGGACGT

TCGTCGTGATGACGACCGTCTTGTCGTGCTCGCGGGCGTACGAGAGCGCG

GGCACGAGCGCGGAGATGGTCTTCCCGGTCCCGGTCGCCCCTTCGAGGAG

GACGTTCCGCTCGTCGTCGAGCGCGTCGGCGATGCCGGACATCGCCGCCT

CCTGATTCGGGTACGGCTCGTCGTACGGGAAGAACCGCCACGCTCCGTCC

GCGTCGCCGTCGTCGGGTGCGCCGTTGGCGTGGGCCACGGGAGAATCTCG

GCCGCCATCCGATTAAAAGGCTCGGACCCCCCGACACCGCCGGACTCGAC

AGGTCCGGTATAATGGTACCCCTGCGGTGCGGCGGTGCCGCCCGTCCGAG

TGACCGTTATCGTGCCAGACGTTGGCACGCTAACCATGGCCTCTGAAACA

GCCACGACACGACTCGAATCGACGACGGCCACCTCTTGGCGCACCGGCGT

CGCCGCCGGCTCGCTCGCCGCCGTCGTCATGGGAGCGATGATGGTCGTCC

AGATGCGGCCGGTCCTCGAAGTCGCCATTCCCTCGATGTACGGCTTGACG

GGCGGGGCCGCCGGCTTCACAATCCACGTCGCCCACGGCGCGATTCTCGG

CGTCGCGTTCGCCGGCCTCGTGAGCGCCCTCGACTTCGACCTCGACGGTT

CGCTCAGGTCGCTCGGAGCGGGCGTCGGCTACGGCGTCGTCCTCTGGGTC

GTCCTCGCCGTCTTGGTAATGCCCGTGTGGCTCGGTGCGGTCGGCTCGCC

CGCGAACCCGCCGCTCCCGAACGTGAACGTGACGAGCCTCGTCGGCCACG

TCGTCTACGGGGCCGTCCTCGGGGCGTCGTACCCCGCGCTCGACGGCGTC

TTCTGAGACGCTGAAAAGAGCGGCGACCGCTGCAGACCCGGACTATTCTT

CGAGCAGTTCGACGATGAGCCCTTTCTGGGCGTGCAGGCGGTTCTCCGCC

TGGTCCCAGACGAGCGAGCGCTCGTCTTCGAGCACGTCGCCGGTAATCTC

CTCGCCGCGGTGGGCCGGCAGGCAGTGCATGACCTTCGCGTCGGTGCTCG

AAAGCAGGTCCTCGTTGAGTTGGAATCCCTCGAACGCCTGCAGTTTCTCG

TGGCGCTGGTCCTCCTGACCCATCGAAATCCAGACGTCGGTGTAGACCAC

GTCCGCGTCCGCGACGGCCTCCTCGGGGTCGGTCGTAATCGTCGGCGTCG

AGCCGAGTTCCGCCGCCTTCTCGAGCACGTCGTCGTCGACGCCGTACGCC

GGCGGCGTCGCCACGGTGAGGTCGATCCCGGCCATCGCGCAGCCGAGGAC

GAACGACTGGCCGACGTTGTTGCCGTCGCCGACCCACGCGGCCTGCACCT

CGTCGAAGTCGCCGACGTGCTCGCGGATGGTGAGCAGGTCGGCGAGCGTC

TGGCAGGGATGGGCGTCGTCGGTCAGACCGTTGATGACCGGCGCGTCGGA

GTGTTCGGCGATTTCGAGCAGGTCCTCGTGGTCGAATAGGCGGACCATGA

TGGCGTCGCCGTAGCGACCCAGCACGCGCGCGGTGTCCGAAAGCGGCTCG

CCGTGGCCGAGCTGGATGTCCTCGGGGCCGAGGAACAGCGCGTGGCCGCC

CAGTTCGGTCATCCCCGTCTCGAAGGAGACACGCGTCCGGGTACTCGGCT

TCTCGAAGAGCATCGCCAGCGTCGCTCGGGTGAGCTGCGTCTCGTCGTCG

CCGGACTTGATGTCGGCGGCGCGGGTGAGGACGCGGTCTAACTCCGATGC

GCTGATGTCGTCGATGTCGGTGAAGTGGGTGGTTTCGAGCATTGTGTCGT

GTGTCGTGTCTCCGTGTCGGTTCGTCGTCTCGGGTGTCTGGTCGTCGTCG

CCGTTCGCCTCCGTCGCCGTCCGCCTCAGTCGTCCGCGAGGCGCTCGCAG

ACGTCGACGAGCACGTCGATTGCGCTGTCGAACTCGGCGAGGTCGAGATG

TTCGTTCGGAGCGTGGTCGAGGTCGGAGTCGCCGGGGCCGTAGGTCGCCA

TCGGGCAGTCCCACGTCCCGGCGAAGATGTTCATGTCGCTGGTCCCGGTC

TTGCGGAGGAGCCGGGGCTTCACGCCGCCGACGTTGCGGATGGCGACGCG

GAACGCCCGCGCCACGTCGGTGCGGGGGCTCATCATGACCGGCGGGATGG

GCTTGTTCCAGTGGACGCCGCCGCGGGTGAGTTCGCTTTCCGCGACCTCG

CGCACGTCGTCGATGGAGAGCCGTGGCGGGACGCGGAACTGCACGTCCAC

CGTCGCCTCGACCGCGAGGCCGTCTTCCGTCGGGCCGCCGTCGAACGTCA

CGGGCTTGGTCGTCACCGTGTCGAAGACGCCGTCGCGGTCCTCGTCGAAG

AAGTCGGCCACGCGGGACCACCACGCGACCGCGGACTGGATGGCGTTCTC

CTCGGGGCGCGAGGAGTGACCCAGTTCGCTCGTCGAGATGTACGTCCCGG

AGAGGAACCCGCGGTAGCCGAGCGTGACGCCGTCCCAGCCCGAGGGCTCG

CCGTTGACGACCGCGTCGGGCTCCTCGCGGTCCTCCACGAGGTGCCACGC

GCCGCGCGAGGAGGTCTCCTCGCCGACGACGCCGACGAACGAGACGCCCG

TCTCGACGGCCGCCGCGGCCATCGTACAGAGCGGGCCGGTCGCGTCGACG

CTGCCGCGACCCCAGAGCACGCCGTCTTCTACCTTCACCGGCACGTCGCC

GGGGACGGTGTCGACGTGGGAGGTGAGAAGCACCGCGTCGTCGGCGGGCG

CGCGGACGTTGCCGACCTCGTCTATCCAGACCTCGCGGTCGTGCGCCTCG

AAGAACGCCTTCAGGACCTCGGCGGCCGCCTCCTCGTCGCCCGACACCGA

GGGCGTCGAGACCATGTCGTAGAGGAGGTGGCGCGCGTCGACCCACTCGC

CCTCGTCGAGAGCGTCGGTGTCGGCGTCCGCGTCGACCTCGGTGTCGGCC

GCCGTCTCGGCCGCCGACTCCGCTTCGGTCTCCGACTCGTCGTGTCCCTC

GCGGTCGATTTCGGCGTTCATGATAACACGTTCGTCATGGCGTCGACCGC

GCGGTCGGCGTGTTCCTCCTCGATGACGAGCGGCGGCAGGAACCGGACGA

CCGTCCGGCCCGCGGGGAGCGCGAGCAACTGCTCGGACAGCGCGAGGTGT

TTCAGCGTGCGGTTCGCGCCGCGTTTGACCTCGACGCCGACCATGAGGCC

GTCGCCGCGGACCTCGCGGACCGGCAGGTCGTGTTCCTCGACGGCGGCTT

CGAGCTCCGTCGTGAGGTAGTCGCCCACCGCGGCCGCGTGGCCGGGCAGG

TCCTCTTCGACGATGGTGTCGAGGGTGGCGTTCGCCGCCGCACAGACTAC

GGGACCGCCGGAGAACGTCGAGCCGTGAGAGGCCGCGCCGTCCGCAATCC

AGTCGGCACAGAGCGTCGCGCCGAGCGGCAGGCCGTTGGCGATGCCCTTC

GCGCTCGTGAGGATGTCGGGGACGACGCCGGCGTTCTCGCAGGCCCACAG

CGCCCCGGTGCGACCGATACCGGTCTGAATCTCGTCGAAGACGAGCGCCG

CGCCGGCGTCGTCGGTCAGGTCGCGGGCGGTCTGGAGGTACTCCGCCGCG

GCGGGGTTGATGCCGCCTTCGCCCTGAATCGGCTCTAAGAACACGGCGGC

CGTCTCGTCGTCGACGGCCTCGGCGAGTTCCGCTTCGTCGCCGTAGCTGA

CGAACTCCACGCCGCCGGCGACCGGTTCGTAGGGCTTCTTGTACTTCTGT

TTCCACGTGAGCGCGAGGCTCCCGAGGGTGCGACCGTGGAAGGCGCGCTT

CGTGGCGACGATTTTCTGGCGGCCGGTCGCGGAGCGGGCGAACTTCATCG

CCGCCTCGTTGGCCTCGGTGCCGGAGTTACAGAGCCAGACGTTCGAGATG

TCGCCGGGCGCGAGCGTCGCCAGCTTCTCGTACAGTTCCGTGCGGACCTC

GACGGGGTACGACGCCTGCACGTAGGTCAGCTTCGCGGCCTGCTCCTGAA

TCGCGGAGGTGACAGCGGGATGGGAGTGGCCGAGCGCCGCGACGGCGTAG

CTCGCGCCGAAGTCGAGGTACTCGGTGCCGTCGTCCGAGTAGAGATACGA

CCCCTCGCCGGACTCGATGGCGATGGGTTTCTCGTTGAAGACGAAGCCGC

TCATTGTTCGGCTCCCTCGGCTTCGACCAGCGCGCCGGGCGTCACGTGTG

TGCCGCCGCCGTTGAGCGCCGTCACGATGGGGTCGTTCAGGTTCGCGTCG

GCGACGACGACCTCGGCGGCCCCGCCGTCGAGCGCCTCCTTCGCGGCCAT

GACCTTCTTGGTCATGAACCCTTCAGCGGCCGATTCGAGCGCCGAGAACT

CCTCGGGCGTGTCGGCCGTCTCGATGAGCGTGGACTCGTCGTCGGGGTCG

GCGTAGACGCCTTTCACGTCGGTGAGGACAACGAGTTTCGCGCCGAGCGC

GCCCGCGACCGCGGCGGCGGCGCGGTCGGCGTCGGCGTTGACCGGCACGC

CGTCGTCGGCGAGCATCGGAACGGTCACGATGGGCGTGTAGCCGCCGTCG

AGGAGGGTTTCGAGGAGCGTCGCGTTCACCGAGGTAATCTTCCCGGAGTG

GTCGCCGCGCTTGATTTTCTTCTTGCCGTCTTCGACGACGCGGACGGCCG

ATTTGCGCGGCCCGGTCAGGAGGCCGCCGTCGACGCCCGAGAGGCCGAGC

GCGTCGACGCCCGCCTCGCGGAACAGCGCGGTCAGGTCGGTGTTGAGCTT

GCCGGGCATCACCATCGAGAAGACCTCCATGGCGCGCTCGTCGGTGAAGC

GGCCGGAGACGCCCGAGGGCGACTCGACGTACGTCGGCTCCTCACCGAGT

TCTTCGAGCGTCTCGTCGACGGCGGTCGAGCCGCCGTGGACGACGACCAC

GTCGGTGCCGTTGGCGACGAGGTGGGCCACGTCGGAGACGGCTCCCTTCG

GGTCGACGGCCTTCGCGCCGCCGATTTTCACGACGACCGGCGGCTCCTTG

CCCCCGTCAGCGCGGAGCGCTGACGAGGTACCGGAATCTCCGATTCCGAT

ACCGCCGTCGGCGATGAGGTTGTCTTCGTTGTCGACGAGCTGTTCGTGTG

CCGCGAGCAGTTCCTCGCGTGTGTATCCTGTCATAGTCGAAATGGTGCGT

GAATCGTGGGTCGGGGCGAGCGTTCGGGGGCGGTGCGGTCAGGGCGAGCC

GACGGGGTGGAAGCCGGTGAAGTCGAGTCCGGCGGTCTCGTCCAACCCGA

GCGCGATGTTGGCGGCGTGGACCGCCTGCCCCGCGGAGCCTTTCATCATG

TTGTCGATGGCCGAGAAGACGACGAGTCGTCGGTTTCCGGGGTCGACTTC

GAAGCCGACCTCGCCGTAGTTCGTCCCGGCGACCGACTTCGGTTCCGGGT

AGCGGTAGACGCCGCCGCCGCCGGCGACGGTGCGCACGAACGGTTCGTCG

CCGTAGGAGCCGCGGAACGCTTTCCACATGTCGCCCTTCGAGACGGGGCC

GTCGGGGAAGACGTGACAGGTCGCCGCCGCGCCGCGGACCATGTCGACCG

CGTGGACGGTAAACGAGACCGAGAGGCCGAGGTACTCCTCGATTTCGGCC

TCGTGGCGGTGGCCGGTCGGCGCGTAGGGGCGGACGATGCCCGAGCGCTC

GGCATGCGACGACGCCTTGCTCGCGCCCGCGCCGCCCTCCGACGAGCCGA

CTTTCACGTCCACGACGACCTGCTCGTCGCCGGCGAGGATGCCGGCGTCG

AAAAGCGGCTTGAGGCCGAGAATCGTCGCGGTCGCGTTACAGCCGCCCGC

GGCGATGAGGTCCGCGCCGGGGAGGTTCTCGCGGTTCAGTTCGGGGAGCG

CGTACTCCGATTTCTCCAGATACTCGGGGCAGACGTGGCCGTCGTACCAC

TCGTCGTACTGCGCCGCCTCGGAGAGGCGGAAGTCCGCAGAGAGGTCGAC

GACGGTGTCCGCCGCGTCCTGAAAGGCGTCGATGTGCTCCATCGAGACGC

CGTGGGGCGTCGCCGTGAACAGCACGTCGACTGATTCGAGGTCCTCCGGC

GAGGTGAAACGGAGGTCGAGGTGACGAAGGTTCGGGTGGACGTGACCGAC

GGTCTTGCGCTCGTAGGAGCGGCTCGTCGCCTGTTCGACCTCGAAGTTCG

GGTGGCCGTCGAGCAGGCGGAGCAGTTCGCCGCCGGTGAAGCCGGAGCCG

CCGACGACGCCCGCGGTGAGTTGGTCGCTCACGCCGAGACCTCCGCCAGC

GACTGCGCGCCGTCGGCCTTCGCTTCGAGCCAGTCGACGACCGTCGCGGG

AACGTCCGCCTCGACGGCGTCGTTGAGCGCCTTGAACTCGACGGTGTGGT

TGACCTCGTGGACGGTGTAGTCGTCACCCGTCTCCATGAGGTCGACGCCG

AGCAGGCCGCCGCCGACCGCGTCGGACGCCTGCTTGACGAGTTCCTTCGC

GCGGTCGTCGAGTTCGAACTCGTCGACGCTCGCGCCCTTCGCGGCGTTCG

TGAGCCAGTGGTCCGACGAGCGGACCATCGCGGCCACGGGTTCGCCGTCG

GTGGCGAGCACGCGGATGTCGCGGCCCGGCTTCTCGACGAACTCCTGGAT

GTAGAACACCTTGTGCTCGTAGTTGCCGAGCGTCGACTTGTGTTCGAGGA

TGGCCTCGGCGGCCGACTCGGAGTCTATTTTCGCCATCAGGCGACCCCAC

GAGCCGACGACCGGCTTGAGGACGCAGGGGTAGCCGAACTCCTCGACGAT

GTCCATCGCCGAGTCGACCGTGAACGCCACTTTCGTGTTCGGCGTGGGTA

CGCCCGCGTCCGCGAGCGCGAGGCTGTTTTTCGCCTTGTCGGCGCAGATG

TCGGCGGTCTCGTGGGAGTTGACGACCGGGATTCCGTACGACTTCAGGAA

GCGCGTGATGTAGATGCTCCTGCTCGTCGCTAGACAGCGGTCGACCACCA

CGTCGAGTCCGTCGAACGATTCCGGCGGCTCCGTGAGGTCGAACTGCTCT

TTCCGAACGTCTATCTTCGTCACCTCGTGGCCGCGGTCGCGAAGCTCGTT

GAGAAGCAGCTTCTCGTCGCGGCGAATCCGGGAATAGAGCAGTCCAACGT

GCAATTTACTCTCCCCAGTCCTCTTCGAGTTCGGGCGCTTCTTCGAGTGT

CACGGGGTCGAGAGAGACGACTTCCAACTCTGCGCCCGTGGCGGGGCTGT

CGATGATTTCACCGACTTCGACGTCGGCCGGCAGCTCGATTTCCTCTCCG

CTCAGCGGGTCTTCCGCGGTGATGGTGTCGCTCATTACATCCGTGGCTGG

TCGACGGACGGTATTAAAGCCGTCGAAAATTACGCGATGAAAATTAATGC

AAAGAAGTCTCTAACGGGTGTTATCTGTCGAATCGGCCGACGATACGGGT

CGACCGAACGACGCGTCGAGCTTGGGTGCCACGCTCACTCGACGCCGGAG

ACGGGACCGGTCGTCGGACCGGTCGACCCGTCCCCCGCGTCGGTCGTGGT

CGTCGTCGCCGCGGTTCCGGTCGGGGTCGTCGGAGCCCCGCCGGTCGTCG

CGGCGCGACCGCTCGGGGTCGTCGGAGCGGGCCCGCGGGCGTCTCGCGTT

CGGACTCAGACATAGCTCGAAACCTCCTCGTCGAGACCTTCGGCGGCCGC

CGCGAGCGAGTCGCGGGCGTCGGCGACGGCCGCGGCGTCGTCCGAGAGCT

CGTCGCGGGCCGTCGAGAGCGTCGCCTCCACGGCGGCGGGTGCGGGCCCG

CCGACCGAATCGCGACTCGCCACGCTCTCGGTCGGGTCGAGCGCGGCTTC

GACGGCCTCGGCTGTCACGTGCGTAAAGAGTGACTCACCTAATACGTCCG

TAGCGACGGCGTCAAGTGTTGCCACGTCGGGCGTTCCCTCGGAGCGGGCG

GCCGCCTCCGCGACGACCTCGTGGGCCGTGCGGAACGGCAGGCCGGCCAT

CGCCAGCAGGTCCGCCACGCCGGTCGCGGTCGAAAAGCCGTCGCCCGCGG

CCGCGGCGAGTTCGTCTTCGGGCCACGTCGCCGACGCCACCGCGCCGGCG

GCGACCGCGGCCGCCTCGCCCACGTCGTCGACGGTGCGGAACGCGTGTTT

GTGGGCGCGCTGGAGGTCGCGGTTGTACGCGCGCGGCAGCCCCTTCAGCG

TCGTCAGGAGGCCGGTGAGTTCTCCGACCGCGTCGCCCGCGACGGCGCGG

ACGAGCTCCATCGTATCGGGGTTCTTCTTCTGCGGCATGATGGAGGAGGT

CGACGAGTAGTCGTCCGACAGCTCGACGAGCCCCTTGTTCGAGAATATCA

CGAGGTCCGCGGCGAGGCCGGAGAGCGTGACCGCGTGGGTCGTGAGCGCC

GACAACGTCTCCGCGAGGAAGTCGCGGGTCGCGGAGGCGTCCATCGAGTT

TTCCATCACGCGGTCGAAGCCGAGCAGGTCGGCGGCGAGTTCGCGGTTCA

CGTCGAAGGGCGTGCCCGCGAACGCCGCCGACCCGAGCGGCGACTGGTTG

ATGCGCTCGTACGCGCACATGAGGCGGGCGCAGTCGCGGGCGACCGCCCG

TTCGTACGAGAGAAGGAAGTGCGCGACCGTCGTCGGTTGCGCGGGCTGGA

GGTGCGTGTAGCCGGGCATCACCGTCTCGGTGTGTGCCGCGGCCGTCTCG

AGTAACGACTCGCGCAGAGCGAGCGCGGCGTCGAGGGCCGAAAGCACGTC

CTCGCGCAGACGGTAGCGGATGCAGGTCGCCACCTCGTCGTTGCGCGAGC

GGGCGGTGTGCATCTTCCCGCCGTCGGGGCCGACGATGTCGATGACCGCG

GCCTCGATGGCCTCGTGGACGTCTTCGCCGCCCGAAAGCGCGTCGTGGCC

CGCGGCCTCGACCTCGTCCAGCGCGGCGAGAATCTCGCTCGCGACCTCGG

ACTCGATGATGTCCTGCGACGCGAGCATCACGACGTGCGCCCGGTCGACG

GCGAGGTCGGCCTCGAAGATGCGTTCGTCGGCCGCGAGGCTCGACATGAA

CCCGCGGGCGGGGCCGCCGCTGAAGCGGTTCCGGCGGATGACGCCCTCGG

AGTCGCCGTCCTCGCCTGCCATCTTACTCGTCGCTACCGCCGTCTGCGGC

GAGTTCGGGCTTCCGCTTTTTCGCGCTCTGGTTGGCGAGGCGCGCCTGGA

AGCCGTGGTACTTGGCGACGCCCGTCGCGTCGGCCTGTTCGATGCCGTCG

ACCGTCTCCGTGTTGAAGGAGGCGGCGGACTCGGAGTAGGCGGCGTACTC

GGACTCGCGGCCGACCGGGCGGGCCTGTCCGCCCTCGAACTTGATGGTCA

CGGTGCCCGTGACGCGCTCTTGGGTCTTCTCGAGGAAGGCGTTCAGCGCG

CCCACGAGCGGGGCGTCGATGAGGCCCTCGTAGGCCTTCTGGGACCACTC

GTTGTCCACGGTGGCCTTGAAGTCGCGTTCCTCCTTCGTGAGGACGAGCC

CTTCGAGCGCCTCGTGGGCGTTCAGGAGTGTCGTCGCGGCCGGGTGCTCG

TAGTTCTCGCGCACCTTGAGGCCGAGCATGCGGTCTTCCATCATGTCGGT

GCGGCCGACGCCGTGCTTGCCGGCCTTCTCGTTGAGGAACGAGATGAGTT

CGAGGGGTTCGAACTCCTCGTCGTCGACGGCGACCGGGTAGCCGTCTTCG

AACGCGATTTCGATGAGTTCCGTCTCGTCGGACGGCTGGTCGGTCCACTC

GTAGATGTCCTCCGGCGGGACGTAGCCGGGGTCTTCGAGGTTGCCGCCCT

CGATGGAACGGCTCCAGAGGTTCGTGTCGATGGACCAGACGCCCTCGTTG

CCGCCCTGCACCGGGAGGTCTTTCTCGGCGGCGTACTCGATTTCCCACTC

GCGGGTCATGCCCATCTCGCGGACGGGCGCGATGACTTCGAGGTCGGAGG

CGCGCCAGACCGCCTCGAAGCGGAGTTGGTCGTTACCCTTGCCCGTACAG

CCGTGGGCGATGCCGTCGCAGTCCTGCTCCTCTGCGAGTTCGAGGATGGC

CTTGGCGATGACCGGGCGCGCGAGCGCGGTGCCGAGCGGGTAGCCCTGAT

AGTCCGCGTTGGCGCACACCGAGTCGAGACAGAGCTGTGCGAACTCCTGT

TTCGCGTCGACTACGTAGTGTTCCAGTCCGAGGGCCTCGGCCGTCTCGTA

GGCCTCCTCGAACTCCTCTTCGGGCTGGCCGACGTCGACCGTGACGCCGA

CGACTTCGTCGTATCCGTACTCCTCTTCGAGAATCGGGACGCAGACTGTC

GTGTCGAGTCCGCCCGAGAACGCGAGTGCGACTTTCTTCATGTACTCGTC

TGTGGCGGGGACACACAGATAAATTCTTTGCATTCAGTTTATGAAATTAA

AAGGTAGAGACGCCTATGGGCACGAAAACAGGAGATGAGCGATTTCGACG

TGTCCCGAAGGAGATGATGATTGTGGCCCTAAAGGGCCCGTCGTCGGGGT

CGGACAGCCGCGGCTCGCAGCGGGGCAAAAGTGGCGCAAGTGCTGCGAGT

CGCCATGGTTGTGAGACGCTACGACGGCTTCCCTATTAAACGCTTCCGGC

GACGCAAACGTTTCCTCAGTCGAGCAACTCCCCGGCGAGTTCGACCAGCA

GTTCGTTGTCGACGAGCGCGAGGACGAGCGCGATAACCGCGACCACGACG

ACTGCGATGATGAGCCACTCGATAAGGGTCATAGCACCCGCAACGTGACG

GCTCCTCCGGTAAGTGTTCTGTGGCCGTTCGGATCGGTCGGCGTCGACGG

GTCGAAAACGCCGCGCCGGGACGAATCAGGCGCTCTCGGTCGAGGCGTCT

CCGTCTGCCGCGCCGCCGCGGAACACGAGTCGCTCCACGAGGTCGGATCG

CTGCATCAACACCGTGCCGAGGGCGCTCATGACGAACACGTAGCCGACGG

CGAACGCCGGGATGATGTCGCGCATCACCGGCGTCGTGCCCGCGGCGGCC

AGCGCCGCGATGACGAGCGAGAACTCACCGCGGGGGACCATCCCGACGCC

GACCCGGAGCGACCGGTGCGCCGAGAGGTCGTACGCCCGACCGCCGAGAT

AGCCCGAGAGAACCTTCGACGGCGTCGAGAGGACGACCGCGATGGCGAGC

GGGACGGCCGCCGCCGCCAACAGCGTCGGGTCGGTGCCGAGCCCAATCCA

GAAGAAGAACACCGCGGCGAACACGTCGCGCACGGAGACGAGCAGGTGTT

CGAGTCGTTCCCGGTGGCCGCTCGTCGAAAAGCCCATGCCGACGAAGAAC

GCCGCGACGGCCTCGCTCACCCCGAGCGCGAGCGCCGCGCCCGAGATGGG

GACGACGACCGCGAGCGCCCGCAGGACGAACGCCTCCTGGTTCTCCACGT

CGAGGACGCGGGCGAAAAGCGCCGTCCCGTACTGGACGGCCACGAACAGC

AGGCCGAGGAAGCCGAAGGCGATGGCGAGCGACCGCCCGATGGCCGCGAC

GCCGCCGTCGCCGCCGAGCACGAGCGAGGTGACGACCGCGAGGTAGACCG

CGATGGCGAGGTCCTCGAAGACGAGCGTGCCCAAAATCGGCTCCGACTCG

GGGTCGGCAATCCACCCGAGGTCGATGAGCGTCTTCGTCACGATGGCCGA

CGACGAGATGTAGACGATGCCGCCGAGCAGGAGCGCCTCGACCGGCGACC

AGCCGAGGACGAGACCGATGGCGACGCCGATGGGGAGGTTCACCGCGAGG

TCGATGACGCCCGCGCGGCCGATTTTCGCCCCCGACGACCGGAGCCGGTC

GAGGCTGAACTCAAGGCCGAGGAAAAACAGCAGGAGGACGATACCGAGTT

CCGCCAGAACCGTCACGACCTCGCCGTTCGGGACGTACGGGAGGCCGAAC

CGCCCGGCGACGTACGGCCCGGCGACGACGCCGCCGACGACGTAGAGGGG

AATCACCGACAGACCGAGGCGGAGCGCGACCGCTCCGACGACCGCGAGGA

CGGCGAACAGGTAGCCGAATTCGAGTAGCGCCGCCGCCATCAGGCCCCTT

TGACGAGCGCGACGAAGTCGCGGCAGGCCGTCTTGGGACCGATGACGACG

AGCGTGTCGCCGGCTTCGACCATGGCGTCGCCGCCGGGCGAGGTGACGAC

CTCGTCGCCCCGCTCGATGGCGATGACGGACGCGCCGGTCGCCTGCCGGA

GGTCGGACTCGCCGAGCGTCTTGCCCGCGATGTCGGAGTTCGCGCCGACT

TCGACCCACTCGATGAGCGTGTTGTCGCCCAGAAGCGTCTCGATGGTCTC

CGTCTGGACCGGCTGGAAGTACGCGCCTTCGAGGAGCGTCCCCACCTGTC

GGGCGAGTTTGTCGGTGAGTTCGAACAGCTTCTCGGAGTCGCCGTCGGCG

CTCGCGCGCCGGAACACCTCCCGCTTGCCGCTGTTGTGCGTCACGATGAC

GAGCCGGGACCCGTCGCCGAGGTCCACCTCGTGTTTCTTCCCGACGCCCG

GAAGGTCGCGCTCGTAGACAGTCATGGCTCGACGATTGGCGAGCGGGCAA

AATAAAGCCGCCTTCCGCTCAGGTGAGCAGCCAGAGCGTCGCCGCGATGG

CGGTGCAGGCGACCGGCGGAATCGAGAGGTTGTCGTCGATGACGTAGCCG

GCGACGACCGGCTTCGCGCCGTCGGCCAGCGTCGCCCCGGCAGCGCCGGC

GGCCGCGGCGAGGCCGCCCGCGACGCCCCCCGAGACGGGAATCACGAACG

GCGCGGCGAGGGCGAAACAGACCGCGAACATCGCCGCGAGCGTCCGCGCC

GACTTCAACTCACCCACCGGTGCGGAGCCCATCAGGCCGCTTATCGGGTC

ACCGATGGTGAGCATGAGCATCCCCGGAACCGCGATGTGGGGGCCGAACA

CCAGCGCGACGGCCGTCTGGCTGTAAACGTACAGCGCGTAGCCGGCGACG

TTGTCCTGTTCGTACTCGCGGGTGAGTTCGTCGTAGACCGCCCATTCGAG

ACCGCCGAACAGCCGGAGCAGTTCGAGGACGCTGACGACCGCCGCGAGGA

AGACGAACAGATACCCGAGGGTACGCCACTCGACCAGCCCGAGGAGGTAC

AAAAGCGGCATCCCCGAACCGCTCGCGTGGACCAGTCGCCGTTTCACCTC

GGCGGTGCTCGGTCGCCCCACGGCGGGTTACGCCTCGACGACCTCGTTGT

CGTCGAGCACGTCGTCGAACGAGGCGGTGCCGGCGCGCAGGCCGACGAGC

GTTCCGACGAGGTCGTCGACGGGCAGCCGGACCTGCTTGCCGCTGTCGCG

CTCGCGGATAGTGACGGTATCCGCGCCGTCGCCTTCCAGCCCGTCGCGGT

CGATGGTGATGCAGAACGGCGTGCCCACCTCGTCCTGTCGGCGGTAGCGC

CGACCGATGCTGCCGGAGTCGTCGTAGACGACCGCGAAGCCGGCCGCGCG

GAGCTCTTCGGCCACGTCGTCCGCGAGGTCGACCAGCCCGTCGACGTTGC

TCACGAGCGGGAAGACGCCGACGTTCGTCGGCGCGACCGACGGCGAAAGC

GAGAGGTAGCTCCGCGCCTCGCCGTCGACCTCGTCGGTCTCGTACGCGTG

CGCGAGAAGCGTGTACACCGTGCGGTCGATACCGAACGACGGCTCGACCA

CGTGCGGCGTGATGTGCTCGCCCGCTTCGGTCTGCGTCTCGACCGCGAAG

TTGGCCACGTCGGTGTCGACGGTGATTTCCTCGCCGTCGACGTCGAGGGT

GACCTCGTCGGCGTCGAAGGCGTCGGGGTCGCGCTCGGCGAGCGTCTCCA

GCGCCTCGGCCACGTCGGCGGCCTGCGCGCCGAACTCGGGACCGAGCGTT

GCCATGTCGGGGTCGACGACGGCGCGCTCGACCGTCTTCGGCTCGTCGTA

CTGCTGGAAGACGGTGAAGTCGTCGTCGCCGTACTCGCCGTGCTTCGAGA

GGTCGTAGTCGCTCCGGTAGGAGAAGCCCGCAATCTCTATCCAGTCGCCG

TCGACCTCGCTTTCGGCGTCCCAGCAGTCCGAGGAGTAGTGGGCGCGCTC

GCCCGCGAGGTGCTGGCGGAAGCGGAAGCGGTCCATGTCGACGCCGACCG

TCTCGTACCACTCTTGGGCGATGCCGAGGAAGTAGCCGAGCCACGCGTTG

CCGATAATGCCGTCATCGACGGCCTCGCCGATGGTCGTCTCGACGTAGTC

GCCGTCGTCGGCCTCCTGCTCGGTCGCGGGGTACAGAAGCACCTCCACGT

CCTCGACCGCCGAAAGGTCGGCCTCGTCGCGCTCGGGGTCGATGAAGTGT

TCCAGTTCGGCCTGCGTGAACTCGCGGGTGCGGACGATGCTCTTCCGCGG

CGAAATCTCGTTGCGGTAGGCGCGGCCGACCTGCGTCACGCCGAACGGGA

GGCTGTTGCGCGCGTACTCCTTGATGCGCGGGAACTCCACGAAGATGCCC

TGCGCCGTCTCGGGGCGGAGGTAGCCCGGCGTCGACGAGCCGGGGCCGAT

GTTCGTCTCGAACATGAGGTTGAAGTCCTCGACCGACTGGCCGGCGAGGT

CCGCGCCGCAGGTCGGACAGACGAGGCCGAGGTCGGCGATTTTCTCGGCC

GCCGCCTCAGGGGATAGCGTCTCGGCGTCCTCCAGTTCGGAGTTGTCCTC

GATGAGGTGGTCCGCACGGTGGGACTCGCCGCATTCGGCGCACTCGACGA

GCATGTCGTCGAAGCCGTCGAGGTGGCCCGACGCCTCGAAGACGGGCTCG

GGCATGATGGTCGGCGCTTCGATTTCGAGGTTGCCCTCCTGGACGGCGAA

GCGCTCGCGCCACGCCTCCTCGACGTTGGACTTCAGGGCCGCGCCCTGGG

GGCCGTAGGTGTAGAACCCGCCGACGCCGCCGTAGGCCTCCGAAGAGCCG

AAGAAGAAGCCCCGGCGCTTGGCGAGTTCGGTGAGCGCGGCGGACTCGTC

GCCCTCACTCATACAGCGCCTCCAGCAGGTTGATGTCGCGGACGACGCCG

ATGAGTTCGTCGCCGGAGACGAGCGGAATCTGCTCGATGTCCTCGCGAAT

CATCGTCTGGGCGGCGTCCTTGGCGGTGGCGCGCGTGGCGACGCTCACGA

GGTCGTCGGTCATGAACTTCGACACCGGTTCGGCCGGAATTTCGACGTTT

CGGGTCGGGATGTAGCGATTGCCGACCGCCTTGATGCCCTCCCACATCCA

CTCGTCGTCCTGATTGGCGACCGAGTCGCCGGTGCCGGCCTCGCCTTCGA

CGACGCGGGCGACCTCGATGATGTCGACTTCGGTCACGATGCCGGCCAGC

GAGGCCTCGTCGTCGAGGACGACCGCGTAGGGCACGTTCGCGTAGAATAT

CTCGCGCTCGACGATGTGGAGCGGCACGTCGACGTACGTGGTGTTCACGT

CGCGGGCGGCGACGTCACCGACCTCGGTCTCGCCGTCGATGTCGCCGCGG

GCGATGGCCCGGACCACGTCGGTAACGGTGAGGATGCCCTCGATTGCGTC

GCCGTCGACGACGGGGATTCGGCGCGCGCCCTCCGAGACCATCGTCGCGG

CCACCGCTTCGAGGTCGTCGTCGGCGCTCGCGGTCGGCACCTCGCGGACG

AGCACCGCCAGCTGGTCTTCGTCGGGGTGTTCGATGAGGTCCTCCCGAGA

GATGAGCCCTCGGTACTTCGTCCCCTCGTCGGTCTCTTTCACCACAGGAA

CAGAAGAGAACCCACGCTCTTGGAGGTATTCCAGCACGTCGTCGCGCGTG

CCCGGCAGGGACACCGTCACGACCTCCTCACCGCGGGTCATCGCGTCGGC

TACTTTCATACACCCGCTTTCTTCCCCCCGCCTAATGTACTCTGCGAACG

CTTTCGCCTCGCTGACAGGCGTCCGCCGCCGCTCGCGAGGGTCGGAGGAC

AGACGATGCGCACGCGGCGCAGAAGCGGAACGGTCCTCGGCTCAGTTCTC

GTCGCCTTCGCCCTCGCCGTCGCTGTTCGGCGCGTACCGCCGAATCGCGT

CGAGGTTGGGGAAGAACTTCTCGGGGTCGTCGTGTTCGACGACGCCGACG

ACGGTGCCGTCGAGCTTCTGGAGGTAGGAGTACATCCGCCCCTCCTCGAC

GGCGACGGTCGCACACTCGAGCGCCTCGCGGAGGTCCCACGCGCCCGCGA

TGAAGACCGCGGTCTCGGTCTCCGGCGAGAACAGCGCCGTCACGATGTAG

ACGCGGCCGTCGTTCTCCGCGCGGTACACCTCGTACTCGGGGAACGAGGT

CGCTTCGAACAGGTCGGCGACGGTCTCGGCCTTGTCGCCGGGGATGACGT

ACGCGAAGCCGAACGCCCCCTCGCCGCCTTTGCCGCTGGAGGGGCCGAAC

GGGCCGCTGTCACCCGCCGGGATTCGGACCGTCTCCCACCCCTCGTCCTC

GAACTCGTCGGCCATCGCTGTCATGTCGTCGAGCGTCGCGCCCCACGCCT

CGCGGCGACGGTCCGCATCGCTCGCGATCCGTTCGGCCTCGGGCGTCTCG

TCGTCTCCGAGCTCGGCCATACCCGTTCTGGACGACGTATATGGTAAAAG

GCATCGAAGGTCACGGGTGGTCCCGTGCCGGTCAGTAGTTGAGTTCGAAG

ACGACTTCCATCGCGTACGAAGTCGCGAGGATACAGACCGACGCGAGGAG

GAGCTTCACCCCGCTCGACGGCCGCTCGTCGCTGTTCGCCGGTCGCAGTC

GCGGCGGGAGTATCGCGTCGAGCGCGTCCCCGACGAGCTTTTCGACGCCG

ACCGGTTCGTCGCTCCCCGAGAACAGGCCGCCGTCGCTGCCGCCGTCACC

GTCGTTTCCGTCCCCGTGTTTCCACGCCGCCTTCGGGCGGACTTTGAGCG

ACGCGTAGCCGAACATGGCGAACCCGAGAAAGAACATGAGCCACTTCGCG

CCGGGCAGTCCGTTGCCGGCGAGCGTGCTCACGACGGCGGTCACGACGAA

CACCACGCCGGTGAGGGAGACGGCGTAGACCACGGCGTCGATGGCCTTGA

CGCCGAATCGGCGTGGGTCGAACTCCATGGCTATTGGACGAGCGTCTCGA

AGACGGTCGCCGGCTCCTCGTACTCGGCCTCGTGGCGGTGACAGAGGCTC

AGTCGCCCCCGGTTGCCGACCGCGAGCTGTTCGGGTCGCTCGTGGTCACA

GACGCTGCCGAACTCCTCGTGGAGGTAGGTCCGTGCGGCGTCCTCGTCGT

CGTTTTCGGCGTAGTCGGCGGCCTCGTGGATGTGGTTCATCACGTCCGAG

GGCGCGTCGAAGTCGCCGAACAGTTCGGGGATGATTTCGTCCATCCCGGC

GCGGTGGCTCTCGCGGCCGAGCAGTTTCCGGACGCGGTCGCTCAACGAGG

GGTCGGCGCGCGAGCGCTCCCGCAGAACTTCGCGGAAGACCTCGATGCGC

TCCCAGAGCTCTGGGTCCATGTCTCTGTACGCCTCCGGGCGAATCTTCAC

CGGACAGCGCGTCGAGAACGGACAGCCCGCCGGCGGGTCGCGCGGGCTCG

GCGGCGTCCCGCGGAGGGTGATGCGGTCGCGCTCGATAGTCGGATCCGGC

TCCGGAATCGACGACAACAGCGCGTGCGTGTACGGGTTCGCCGGTTGGTC

GAACATCTCGTCCGCCGGTCCGATTTCCATCATGTTGCCGAGGTACATCA

CGGCGACGCGGTCGCAGATGTGGCGGACGACCGCGAGGTCGTGGGCGATA

AACAGGTACGTCAGCCCGAACTCGTTTTGCAGGTCTTCGAGCAGGTTGAG

AATCTTCGCCTGCACGGACACGTCGAGCGCCGACACCGGCTCGTCGAGGA

TGATGAACTCCGGTTCGAGCGCGAGTGCGCGGGCGATGCCGATGCGCTGG

CGCTGGCCGCCGGAGAACTGGTGCGGGTAGCGGTAGTAGTGTTGTTCCTG

CAGGCCGACGGTTTCGAGCAGTTCGCGGACGCGCTCGCGGCGCTCCGACG

GCGTCTTCCAGTCGTGCACGTCGAGCGGCTCGCGGACGATTTCGCCCACG

GTCATCCGCTCGTTGAGGCTCGAATCGGGGTCCTGGAACACCATCTGTAC

GTCCTTGCGGAACTGCTTGAGGTCCGAGCCGGACAGCGTGGTGATGTCCG

TGCCGTTGAGTTTGACCTCGCCCGCGGTGGCCTCTTCGAGGCGCATCAAC

GTGCGCCCGAGCGTGGACTTCCCGCAGCCGGACTCACCGACGAGTCCGAG

CGTCTCGCCGCGCTGGATGTCGAACGAGACGCCGTCGACCGCTTTCACGG

GGTTGCTGCCGAGGAAGCCGCCGTCCTCGTAGTAGGTTTTGAGATCGTTG

ACTTCGAGGATGGTGTCTCCGCGCGCCTCGGTGCTTCGCTCTTCTTCAGT

GGATGTCGTACTCATCGCGTGTCACCTCCGCGCTGTCCGCCCTCGCGGTG

GACGGCGACCGCCTCCTCCGTGGGAAGGTCGTCGGGGTACAGCAGGCACG

ACGCGCGGTGTCGGCCCGTGCCGTCCCCGACTTCGACGGGCTCTGGGTGG

ACCGTGTCGCACTCGGTGAACGCCTTCGGGCACCGGGGGGCGAACCGACA

GTACGTCGGGTCCTCGTTCGGTGTCGGCACGTCGCCCTCGATGGTCGCGA

GGCGTTCGTCTTCGTCGAGCCCCTGTCCGGGAATGGAGTTGAGAAGCCCC

TGCGTGTAGGGGTGTTTCGGCGACTCGAACAGTTCGACGACGGGTGCGCT

CTCGACGATTTCGCCCGCGTACATCACGTTGACGCGGTCCGCTATTTCGG

CGATGACGCCCATGTCGTGGGTGATGAACATGATGCCGAGGTCGCGTTCC

TCTTGGAGCTCTTCGAGGAGTTCCAGAATCTGCGCCTGGATGGTCACGTC

GAGCGCCGTCGTCGGCTCGTCGCAGATGAGCAGTTCGGGGTCGCACGCGA

GCGCCATCGCGATGACGGCGCGCTGGCGCATCCCGCCGGAGAACTGGTGG

GGGTACTCGCGAACGCGGCGGTGCGCGTCCGGAATCCCGACCGCTTCGAG

CAGTTCGATGGCCTCTTTCGTCGCCTTCGACCCGCTCAGTCCGCGGTGGA

GGCGAAGCGCCTCCTTAATCTGGTTGCCGACGGTGTAGACGGGGTTCAGA

CTCGTCAGCGGGTCCTGGAACACCATCGCGATGCTGCCGCCGCGGATGGA

GCGCATCTGCTTTGCCGACTTGTCGAGGAGGTCCTCGCCGTCGTACATGA

TGCGGCCGTTCTCGATGCGGCCGGGCTTTTCGACCAGCCGCATGATGGAG

CGGGCCGTGACAGACTTGCCCGACCCGGACTCGCCGACGATACCCACGGT

CTCGCCGCGGAAGATGTCGAACGAGATGCCGTCGACCGCGCGGATGACCT

CCTTGTCGGTGTAGAACGACGTTCGGAGGTTCTCGACGGAGAGCAGCGGC

TCACCCTCGGTGCGGGCCGAGCGCGCCTGTTCGGTGCTCATGCGCCACCT

CCGGTGGCCGCGGCCTCGTCACCGGTGTCCGCCTGCGGGTCAATGGCGTC

GCGGATGCCGTCGCCGAAGGCGTTCAGGCCGGTCACGACGAGCGTGATGA

GGAGGCCGGGAAGCAGCGAGATGTGCCACGAGGGGCTCGCGACGTACTGC

TGTCCGTTCGCGATGAGCCGGCCCCACTCGGGGGTCGGCGCGGTGATACC

GAGCCCGAGGTAGGAGAGCCCGGCGACGCTGATGATGATACCACCGAGAC

TGAGCGAGGCGTAGATGAGCATGTAGCTGAACACGTAGGGGGCCATGTGC

TTGCGCATGACCTGCGACGGCGACTGACCGAACGACTTCGCGGCGTCAAT

CCAGTCCTGCTCGGAGACCTGGAGTGCTGGCCCACGTATCGACCGCCAGA

TGAACGGCCAGTAGACGATACTGAAGATGAGGATGATGAGCACCGCGCCG

TCGTACAACTCCCTCACCCAGGTGTTCTGGAAGATGACGAGCATCAAGAT

GAGGACCATGATGACCGGCAACGCCTGTATCGAGTCGGAGACTAACACCG

TTGCCAAGTCTGCCAGCCCCTTGTAGTACGCCGTTATCATGGCCAAGCCG

AGTCCGATGAGGCCGGCGATACCCATGGCGACGACGGCGATGGTCAGGGA

TATCCTGGCCCCGAACACCACCTGTGTGAACAAGTCCTTCCCGTTTGTCG

CCGTGCCGAACGGATGGAATCTACCGAAGTCGTCGTACGTCAGCGGCGCG

ACGTTCTCGTCACCCGCTCCCTGCGACCCGGAGCCGAGGTTGGCCTCGCC

GACGAGGACGGTCTCGACGCTCTGGGTGTCTTCGCTCCAGTAGCTGACCT

CGTAGTCGTACGGCTGGAGGATGTTCCGCTCCATCGTGGTCGGACCGAGC

GCGGGCGCGAATACCGCCATGACCACAAACGTGATGACGATAATGAGCCC

GAACTTGCCCCAGGAGTGACCCCGGAAGCGGTTGACCACGTCGTCCCGCG

GCGTCCAGTCGACGCGGCGGTAGTGGCGGCGGAACCGCTTATAGCCGTTC

CACGCCCACGCCAGCACCGCGAAGGCGTACGCGTAGACGACCGCGACGCG

GATGGCCCACGCGACCGCGGGCGAGAGCCCGAGGAACGTGTTCTCGTAGC

TCGTGCCGTCCCAGTAGCCCTGGTTCGGAATCACGTCGCGGGAGACGAGC

GTCGGAATCGCGTCGAACGCCGCCTGCAGCGACTGGACGGCGGACGCGCC

CGGGTCACCGGGAAGGAGGTTGACGACCACGCCGGCGACGGCGCCGACGA

CTTGGAGGATAGCGCCCAGCTCGGCGCCGATGAGGACTGCACCGACGGCG

GCCCAAATCAGCGCGGGCTGGGGGTTCTCAGCGACTCGTTGTCGGAGCGG

TATTTCGGTTTCGGTTGTCGTGCTCACTGTGGTTCACCCGTCGTACCCGA

CGCGCGGGTCGATAATCGTGTACAGGAAGTCCTGTAGGATGTTCGTGCCG

ACCAGGATGAGGATGAAGATAAACATCAGCGTCCCGATGAGCGGCAGGTC

CCCGTTGATGGCTGCGTTGAAGAACAGCCAGCCGAGGCCGTTAATGGCGA

AGACCGTCTCGACGAACACGGACCCGCCGAGAAGCAGGAACGCCTCGCCC

GTGATGATGGGCACGAGCGGGATGAGCGCGTTCCGGAAGATGTGCTTCCA

GACGAGCGAGCGCCCGGAGACGCCCTTCGCGCGGGCGGTCTCGACGTAGT

TCGAGTTGATGGTCTCGAGGACGGCGGTGCGGCCGATGCGCATCTCGTTC

CCCATCGACGCCGACCCGAGGACCAGCGCGGCCGGGGCGATCTGCTTCGT

CGCCCGGACGAACGACTCGGTCCACCCGCCGGGGTCTTCGAGGAACTGTT

CGACCGGCGACTGGAAGAAGTCGAGCGCGGGCGGCGTCACGACGTTCGTC

CTGACGATCCACGTCTGCCACGCGAACAGGCCGTTGGTCCACGTCCCGAG

CTGCGAGAGGGCCGTGACGAGCATGATGGCCAGCCAGAAGTTCGGCATCG

CGCGCCAGACGATTCCGCCGAAGGAGGCGGCGTAGTCGGAGGGCGTGTTC

GGGTTCAGCCCGGCGTAGAAACCGAGCGGAATCCCGACGAACAGCGCGAT

GACGACCGACCAGAAGCCGAGCCAAATCGTCCGGGGCGCGTAGATTTCGA

TGAGCTCGTACGCGGTCGTCCCGGGTGCGATGACCCACGACTGGCCGAGT

TGGAAGGTGAACAACCGATACATGAAGTCGAGGTACTGACTCCAGAGCGG

CTGGTTCAACCCGAGGTTCGTCCGAATCTGTTCTGCCGCCTGTGGGTTGT

ATTGCGTCCCCAGAATCGCCGAAACGGGGTCGAGTGGCCCCAGGCGGATG

AGGGCAAACGTAATCGTCGTCCCGAAGATGACGACAGGAATCGACATCAG

TACCCTGCGGAGGAAGTACTGCCATCGACTCATGGCATCACCTCGCAGAC

CGTTCGAGCGGACATTGTTGTAAGCTTTTACTGAGTGGTATGGATATGTC

TTGCGCTTTGAGGTCCAAATATCGCAGTTTCAGCAACTCTGCTGCCAGTT

TATTTCATATAGTGGCCACTAATTGTACGCTTGTCAAAAATATCTGGCCG

TTTCGATTGAGACGGGACGCGAAAGCGTTCCCGTGTTCCTCGGTTTTATC

GGTTGAGGGTGACGGTGTTGAGCTTCTGGCGGCTCGGACCCATCCCACCG

AACGGTTCGATGTCGACGGTGTCGTACCAGAACATCTCTTCTTTCTGGTT

GTAGACCGGCAGCATCGCGACGTCTTCCCAGTTGGCCATCTCGATGTCGA

CGTAGGCCTCGTCGCGGACCTGCTGGGCCTCGTCGGTCGGCGCGGGGTTG

TTGACGACCTGCTGATAGGCGTCGGTCGCCTGCTGGGCGGCGTCGCCGTT

TTCGGCCGTCCAGTTGACGTACGAGATGGGGCCCTGTTCGGACGTATCAG

TCTGCGGCGGATTCAGGAGCTGCAGGAAGTTGTCCGGTGCCGGCCAGTCG

GCGATCCAGCCGAGGGTGTACGCTTCGAGCTGACCGTTGCGGCCGCGTTC

GAGAAGCGTCGAGAAGTCGGCCTTCTGAATCTGCATGTTGATGTGGGCGG

ATGCGAGCTGGTCGCGGAGGATGCTCGCCATCTCCTCCCACGCGTTGTTG

TTGTACTGGGTCCACTGGACCTCGAACTGGTTGTCCGGACCGTAGCCGGC

CTCCTCCATGACCTGGCGGGCGGCCTCGATGTCCGTCTCGCCCGCGCTGT

ACGGGTAGTCGGAGCTGACGAGGTCGCTCGCGGCCTGCGCACCGCCGGGG

AAGATGGTCGGCGGGGTAAAGAACTCCGCCGCGGAGCCGCGGCCCTTGAA

CACTTCCGAGACCATCTGGTCTTGGTTCAGGACGTAGGCGAACGCCTGAC

GGACCGCCTTCGGGACCTTGTTCATGTTGAAGCCGACGTAGTAGATGGAG

AGGGTCGGAACGCCGACGTAGTTGAGCGTCTTCCCGTTACGGACGGGACC

GTACGTCCCGATTTCGCGACCGTACTCGTCGGTTGACTCCACCTGAGCCA

GTCCGGGGTCGTACTGCGCGGTGGGCAGTCCGAAGTAGTCCGCGTTCTCG

TTCATCGCGTAGTTGTAGCGAGCCGTGTCGTCCTCGATGATCTGCCAGCG

GACGTTGTCGACCTGCGCGACCTGCCCGTAGTAGTCGTCGTACTTGGAGA

CGGCCGCGGCGGTGCCCTGCTCCCAGAACGCGAACTCGAACGGACCCGCA

CCGATGGGGTTGTTCGACGCGAACTCGGTGTACTCCATCTCGCCGTCGTG

ACCCTCGATGTCGCCGAGGATGCCCTCGGGAAGGGCGGCGAACGAGGTGT

AGGCGAACATCTCGAGGGTGTCGTGGAACGGCTCGGAGAGGTTGACGACG

AGTTCGGTCTCGGTCTCGCCGACTTCGACGCCGAGCGAACCGGGCACGTA

GTTGCCTTCGTCGTCCGTCTCGTGTTCCACGCCGATGGAATCGAGGATGA

AGTAGGCGCGGCGGCTGTTCGAGGAGGCCGCGAGGCGCTCCCACGCGTAG

ATGAAGTCGGACGCAGTGACCGTCTCACCGTTGTGGTAGGTCGCGTCGGC

GAGCTGGAACGTGTAGGTCGTGAAGTCGTCGGAGACCGTGTAGTCCGCGG

CGAGTTCGTTTTCGACCGTCGGGAGGGCGTTCTGGTAGGACATCAAGCAG

TCGAAGACCTGCTGGATGATGATGCCCGACGACGTGTCGGTCGCGGCGAC

GGGGTCCATCGTCGTGATGGTGCCCGAGAGGATACGGTTGAACACCGAAC

CGGACAGCTCCGTGCCCGTGTCCTCTTCGGTCGTCGTCGCGGTGGTGGTC

TCGTCGCCACCGGATTCGGTCGTCGTCGTCTCTTCACCGTCACCGCCGGT

ACAACCGGCGAGAGCGGCGGCCGTGGCAGCCCCACCCGTTGCCTTCAGGA

AGCGGCGACGCGAGAGTTTGTTAGTGTCTGGCATTCAGAACGAAATTTTC

CCGGCGCGTGGATAAACTTACCGTATTCGATTTGTTTGATATAGTGTAGC

GTGGAAGGGAGGTAGTTCCATACCCAAGCGGCGAAAACTGCCACGAGAAT

CCCTACAAAACTCGTTTTACATTTTAAACCCGCTTTCGAGGCGACAGAAT

CAGTCAACGGGACACTTCTCGGCCCGCAGTTTCGGTCCACTTATATAGGG

GTGTCACTATGTATCATACATGACGCCGGATGCGACGGCTTCCGTCGACG

ACTCGGGCGCCGAGGTCATGGCCTCGGTGGATCGCTCTCAGACGGGCCAG

CGTCTTATTATCGCGGATATCTCCCGCGACGACGCGTGGCTCGCTGCTGA

CGTAGCCGACGCGCTTGCGCTCGACGAGTGGCAGTAATCGCACCGGCCCC

GTAAGCCCGCCGCTGGACGCCCGGCGGACAGACCATTCACATTTTAGTCC

GACTCGCTCGTGGTACGCTGCATGAACGCTCGAGCGGTGACGGTGAGCGA

GGAGTACCTCGCCCGTCTCGAACACGGTGCGGACTGGCGCGAGGAGATCG

AGGAGTTCTGCGCGCGCAAGGACATCGAGTCCGCGTGGTTCAACGCCATG

GGTGCCGTCCAGGACGCCGAACTCTGGTTTTACGACCAGACGGACCAGGA

GTACCAGTCCGTGACGTTCGACGAGCCGCTCGAAGTCGCCGCCTGCGTGG

GTAACGTCGCGCTCCTCGACGGCGAACCCTTCGCACACACGCACGCAATC

CTTTCTCGGCGTAGTGGGCAGGCGCTCGCCGGACACCTCGACTCTGCGAC

CGTCTTCGCCGGCGAACTAAACCTCCGCGCCTTCGAGGAACCGCTCGAAC

GCGACCACGACGCGGTGACCGACCTCGACCTCTGGCTGTAACCGTGCGCG

AGGAGGAAACCCGGTACTTCCGTCGCATCGAAGCCCGCCTCGACGAGGCG

TTCGACCTCGCGGAGGCCGCGAAGGCGACGGGGTACGACCCGAAGACCGA

AGTCGAGATTCCGGTCGCCAAGGACATGGCCGACCGCGTCGAGAACATCC

TCGGCATCGACGGCGTCGCAGAGCGCGTCCGCGAACTCGAAGGCGAGATG

TCCCGCGAGGAAGCCGCGCTCGAACTCGTGACCGACTTCGTCGACGGCAA

CGTCGGCGACTACGACTCCCGCGAGGGCAAAGTCGAGGGCGCGGTCCGCA

CCGCCGTCGCCCTCCTCACCGAGGGGGTCGTCGCCGCCCCCATCGAGGGC

ATCGACCGCGTCGAAATCCTCGAAAACGACGACGGCACGGAGTTCGTCAA

CGTCTACTACGCCGGCCCGATTCGCTCGGCGGGCGGCACCGCACAGGCGC

TCTCCGTGCTCGTCGCCGACTACGCCCGCTCGCTGCTCGACATCGACGAG

TACAAAGCCCGAACCGACGAGGTCGAGCGCTACGTCGAGGAGATAAACCT

CTACGACAAGGAGACCGGCCTCCAGTACTCGCCGAAGGACAAGGAGTCGC

GCTTCATCGCGGAGAACATGCCCATCATGCTCGACGGCGAAGCCACGGGC

GACGAGGAGGTCTCGGGCTACCGCGACCTCGAACGCGTCGACACCAACTC

CGCCCGCGGCGGCATGTGTCTCGTCATGGCCGAAGGCATCGCGCTCAAGG

CCCCGAAGATTCAGCGCTACACCCGCCAACTCGACGAAGTCGACTGGCCG

TGGCTCCAAGACCTCATCGACGGCACCATCGGCAAGGACGACGACAACGC

CGCGAACGCCGACGACGCGGGCGACGACGGCGACGAAGCCGAGGCCGAGA

CCGACCCGGACGCAGAGGCCGACGACGCCGAGAGCGACGCCCCCGACGGC

CCGACCCGCGTCGAACCCGCGACCAAGTTCCTCCGCGACCTCATCGCGGG

CCGCCCGGTGTTCGGCCACCCCTCCGCGCCCGGCGGCTTCCGCCTGCGCT

ACGGCCGCGCTCGCAACCACGGCTTCGCGACCGCGGGCGTCCACCCCGCG

ACGATGCACATCGTGGACGACTTCATCGCCACCGGCACCCAAATCAAGAC

CGAGCGTCCCGGCAAGGCCGGCGGCGTCGTCCCCGTCGATTCCATCGAGG

GACCGACGGTCAGACTCGCTAACGGCGACGTGCGCCGCATCGACGACCCC

GAGGAGGCGAAGGAACTCCAGAACGGCGTCGAGAAGATTCTCGACCTCGG

CGAGTATCTCGTCAACTTCGGGGAGTTCGTCGAGAACAACCACCCGCTCG

CGCCCGCCTCGTACGTCTTCGAGTGGTGGATTCAGGAGTTCGAGGCGACC

GAGGCCAACGTGCAGGCGCTCCGCGACGACCCCGCCGTCGACCTCGAAGA

GCCGTCGGTCGAACAGGCCCTCTCGTGGGCGACCGAGTTCGACGCCCCAC

TCCATCCCGTCTACACCTACCTCTGGCACGACATCTCTGTCGAGCGGTTC

GACGCGCTCGCCGACGCCGTCGCGGCCGGTGAAATCGTCGCCGCCGAAGC

CGACGGCGGCACGACCGCCGCGCTCGAACACGACAACGAACCCGAGCACG

GACTGGAGGGGACGCTCGTCCTCGACAACGCGCCCGAGATTCGGGAGGCG

CTCGAACACCTGCTCGTCGCTCACCGACAGACCGACGAGGCGCTCCGCGT

CCCCGTCTGGCGACCGCTCGCCCGGAGCCTCGGCCTCACCGACGACCGCG

AGCGGACGTGGGAACTCGATGACCTCTCCGAACGCGCCCGCACGTGGGAC

GACGGCGACAACGCCGTCGAGGCCGTCAACGAGGTCGCGCCGTTCAACGT

CCGCGAGCGCGCGCCCACCCGCATCGGCAACCGGATGGGTCGCCCGGAGA

AATCCGAGCGCCGCGACCTCTCGCCCGCGGTCCACACGCTGTTTCCCATC

GGCGAGGCCGGCGGGAGCCAACGCGACGTTGGCGACGCCGCCAGACACCG

CGGCGAGTCCGGCAAGCGCGGCCAGATTTCGGTCCGCCTCGGTCAGCGCA

AGTGCCCCGACTGCGGCGCGTTCGGCTTCAAATCGAAATGTCCCGACTGC

GGCGGTCACACCGAACCCCACTACGAGTGCGACGACTGCGGGAGCGTCAT

CGAACCCGACGAGTCGGGCCGCGTCTACTGCGAGCGCTGCGAGTGGGACG

TCGAGAGCGCCGAGTGGCAGGACGTCGACCTGAACAGCGAGTACCGCGAC

GCCCTCGAACGCGTCGGCGAGCGCGAGTCGTCGTTCCAGATTCTCAAGGG

TGTCAAAGGTCTCACCTCCGCGAACAAGACGCCCGAACCCATCGAGAAAG

GCGTCCTGCGGGCGAAACACGACGTGTCGTCGTTCAAAGACGGCACCGTC

CGCTACGACATGACCGACCTGCCCGTCACCGCGGTCCGCCCCGAGGAACT

CGACGTGACCGCCGACCACTTCCGCGAACTCGGCTACGAGACCGACATCG

ACGGCGAACCCCTCCGGTTCGACGACCAACTGGTCGAACTCAAAGTCCAG

GACATCGTCCTCTCGAACGGCGCGGCGCAACACATGATGCAGACCGCCGA

CTTCGTCGACGACCTCCTCGACCAGTTCTACGGCCTCGACCGGTTCTACG

AAATCGAAGAGCGCGACGACCTCATCGGCGAACTCGTCTTCGGGATGGCC

CCCCACACCTCCGCCGCGGTCGTCGGCAGAGTTGTCGGATTCACGACAGC

AGCAGTTGGATACGCGCATCCGTACTTTCACGCCGCGAAACGCCGGAATT

GCGACGGAGACGAAGACTGCGTCATGCTGCTCATGGACGGTCTTCTCAAC

TTCTCGAAAAAATATCTCCCCGACAAGCGCGGCGGGCAGATGGACGCGCC

GCTCGTCATGTCCTCGCGCATCGACCCCTCGGAGATCGACGACGAGGCGC

ACAACATGGACATCGTGCGGCAGTACCCCCGCGAGTTCTACGAGGCGACC

CTGCGGATGGAAGACCCCGACGACTGGGAAGACGAGGTCACCATCGCCGA

GGAGTACCTCGGAACCGACCGCGAGTACACCGGGTTCGACCACACCCACG

ACACCACGGACATCGCCGCCGGCCCGGACCTCTCTGCGTACAAGACCCTC

GGGTCGATGATGGACAAGATGGACGCCCAACTGTTCCTCGCGCGGAAGCT

CCGGGCGGTCGACGAGACGGACGTGGCCGAGCGCGTCATCGAGTACCACT

TCCTGCCGGACCTCATCGGCAACCTCCGCGCCTTCTCCCGACAGGAGACG

CGGTGTCTCGACTGCGGCGAGAAGTACCGCCGGATGCCGCTTTCGGGCGA

CTGCCGGGAGTGCGGCGGCCGGGTGAACCTCACGGTCCACCAGGGCTCCG

TGAACAAGTACATGGACACCGCGATTCAGGTGGCAGAGGAGTTCGACTGC

CGCGACTACACGAAACAGCGCCTCGAAGTGCTCGAAAAGAGCCTCGAATC

GGTCTTCGAGAACGACAAGAACAAACAGTCGGGCATCGCGGACTTCATGT

AGTTTAGCGGTCGGTTTTTCCTCCAGGTTTTGCGAGTCGAGCGGAGCGAG

ACTCGGAAAAAGTGGAGAACGAGCAGTCGGGCATCGCGGACTTCATGTAG

TTTAGCGGTCGGTTTTTCCTCCAGGTTTTGCGAGTCGAGCGACTCCGCCG

CTCGGCGTCTCAGTCCTGCGCCGCCCCTAACCCGTGGAAGCCGTCGGCGG

CGACGACGTACACCGCGCCGTCGACGGGGACTGCGGCGGCGACGCCCTGT

GAAACCACGTCCGACACCTGCACTTCCGGGGTCTGGTGTGTCCATAACTG

ACGCTCTTCATCAGTGTTGTACGCTGCTAGTCGTCCCTGCTCATCGGAAA

CCAACACTACTTCTCGTGCGCGCGCGTGCGGAGCGCCGACGAATCGCGTC

GCCACTTCTTTCCCGTCGTCGCGAGACAGCGTCGCGGTCTCCCCGTCGAC

CGCGACGTGGAGCACGTCGTCGGTGACGACCGGCGCGCCGCTTCCCGGCG

ACCGCCATCGTTCGGTGCCGTCGCTCGGGGCGAGCGAGAGGAGCTCTCCG

TCGCGGGTGGACAGATACGCGGCCTCGGGCGTCGCCACCGGCGTCGTCCA

TTCGCTCGTTCGCACCTCGACGCGGAAGCGCCGCTCGCCGGTCGCGAGGT

CGACGGCGTGGACGACGTCGCTCGTCACGACGAACACCGCCCCGTCGGCG

ACGGCGGGCGCGCCCTTCGCGTCGCTTTCGAGGTCGTAGCGCCAGCGTTC

GCTCCCGTCCGCGAGATCGAACGCCCGAAGCTGGGTCGGGACGCCGTCGA

ACCCGCCGCCTGAGACGACGGCGACGCCGTCGCCCACCGCGGGCGGGCCG

TGGATGCCGTCCATCTCGGGGCCGCGCCAGAGCACTTCGTCGCGGTCGGC

GGCGAGACAGAACACCTCGATGAAGGTCGTGACGACGACTCGGTCGCCCG

CGACGACGGGCGGCGAGTTCACACCATAGCCGACAAGCGGGGTTACCGCT

CGCTCACCCATCGTCGAGGGCTCGCGTCGGACGAACGCTCGCTCGTCACG

CCCGCGCCCATGGAGGTTGTAGAGGCGGTCGCCGTCGAGGACGGGTGAGC

CGCCGGCGTTTAGCCGCCAGTACTCCTCGGCGTCGGTCGGAATCGAAACG

TCGGGGTTGTAGCCGGTGTTTCCCGCGTCGAAGCGGTACTGCGGCCACGC

CCCCGACGGCGAGAGCGCGGGGTCGCCCGGCGGCGTGGCCGTCACGCGTT

CGTCCGGCCCGGAAGTCGGACCGTAAGTCGTAGACGGTTCGGCGGTGGTG

GTCGTCGCCTCGGTCGTCGTAGGACCGGACGACCCGCCGTCGCGGCCGAG

ACAGCCGGCGACACCGAACGACGCGCTTCCGAGCAGTTGGAGGAATCTGC

GGCGGCTCCGAAGACGGGGCATACTGTCGGATCTATGACGTGGCGGCAAG

TGCTTTGTCGAGCCACGAGATTCTATATCGCGGTATTTGATTTATTATTT

ATATGCGGACATCACTGCCGGCCTATTGCTGGGCTCGCTCGACCTCGACG

CGGATTCGGCCGTTCCTTTTTATCCGCTCGCGCGACGACTAGTCGCGTAT

GGCTGACGAGACACAGCCAACGGTGGGAATGACGGTGTACGCGGCGGACG

GAACCGAACTCGGGAGCGTCCGCGGTTTCGACGACGACGGCTTCTTCGTG

ACGACCCGCGAGGGACTCGCGGGGATGTCCGTCGAACACGAGCGCGCCGG

CCACGAGTTCGGTGAGGCCGAGTTGATGTGGCGGTGTAGCGACTGCGGCG

AAATGGGCGACCTCGACGAACTGCCCGACGCCTGCCCGAACTGCGGTGCC

GAGCGCGAGCAACTCTACTACTGGACCGAGGACTGAGGGGTCGCCCCGAT

TCGACCGCTGCGCCGCGAAACTCGCTATTTTTGTCGCCCGCCGAGTACGG

GTAGCCATGCGTATTCTCGTCTTCGGCGCGGGCAGCCTCGGCACGCTCGT

CGGCGGTCTCCTCGCGTCGGTCCACGACGTGACGCTCGTCGCCCGCGACC

CGCACGCGGCCCGGGTGTCGGCGGCCGGGCTCGATATCATCGGTGCCGGG

TCGGCGCACATCTCGCCGGCCGCGACGACGACCGACACGGGCCATTCGGC

GGACCTCGCGCTCGTCACGGTCAAGTCGTTCGACACCGCGGCCGCTGCCG

ACGCGCTCGCTGACTGCGACGTGGACGCGGTGCTGTCGCTCCAGAACGGA

CTCACCGAGGAGACGCTCGCCTCTCGCCTCGACGCGCCGGTCCTCGCGGG

AACGGCGACCTACGGAGCGCGATTAGTCGAGCCGGGCCGCGTCGAGTGCA

CCGGCGTCGGTCGGATCGTCTTGGGCGCGCTCGACGGCGGGCCCGACCCC

CTCGCGGAGCGCGTCGGGAAGGCGTTCCGCGATGCGGGGCTCAACACGCT

CGTCGCGACCGACATGCCGCGACGGCGCTGGGAGAAACTCGCCGTCAACG

CCGGCATCAACGCGGTCACCGCGCTCGCGCGGGTCGAAAACGGCGCGCTC

GCGGGCGACGACGCCGGCGAACTCGCACACCGAGCGGCCCGCGAAACGGC

CCGCGTCGCCCGATTAGAGCGCGTGTCGCTCCCGAACCGCGTCGCCCGCG

AGGCGGTCGACCGCGTGGTCGAAAAGACCGCCGCGAACCGCTCGTCGATG

TTGCAGGACGTGGCGGCCGAAAAGCGGACCGAGGTGGACGCTATCAACGG

TGCGGTAGTGGATATCGCCGCCGACCACGACTTCGAGGTGCCGACGAACC

GGACGCTCGCGGCGCTCCTCCGGGCGTGGGAGCGGGGAGCAGGGCTTCGG

TAGTCGCGTCGGACCCGAGACGCCCGTGTGCGAGCTATTCGAGGTAGCCG

AGGTCGCGGAGTCGGTCCTGCGCCTCGTCGTCCATCTGGTCGACCGAGTC

GTCCGACACGTCGGTGTCGAGCGCGTCGGTCCACGCGCCGCCGATTGCGT

CTTCGAACTCCGCGAGGGCGGCCTCGGTCTCCGCGATGGCCTCGTCGCCC

GTGTCGGCGTCGACGACGTTCTCGGTCTCCTCGGGGTCGTCGTCGATGCG

GTAGGCTTCGTCGGGGATGCGGTCGATTCGGACGTACTTGGCGTCGGTCC

GCCGCGCCGCCCGCATCCGGGAGTAGAACCGCGAGTCTTCAGGTAGGGTG

ATGCCGGCGCTCGACGCCTTTTCTTCTAGCTGCTTGAGTTCGACCACGGG

ACGGGAGTACTCGACGAAGGCGTACTGACCGTCGCGGCGCTGACCGGGGT

CGTCGTTCGACGACTGCGCGAACTCCCGGTAATCGGCCGACAGGAGCGAG

CGCGTGCGGTCGAGGCCGACCGCGTCGTCGCCGGGGGTCGCGGGCTCGCC

GCCTTCGACGCCGAGCGAGTCGAGCACCGTGTGGTAGAGGTCGACCAGTT

CGACCTGGTCGCCGCGTCGCTCCGCGTCGAGGTCGGGGTGTTTGACCATC

AGCGGGACGTTGATGAGCGGGTCGTACAGGCAGAACTCGTGGCCGTAGAG

GCCGTGTTCGCCGTGGAGTTCGCCGTGGTCGGCGCAGACGACGACCATCG

TGTCGTCCCAGCGGTCGGTCTCTTTGAGGTGGTCGAACAGCCGGGTGAGC

TGGTCGTCGATGTGGGCGATTTCGGCGTCGTAGAGCCCGCGGATGTCATC

CCACTCGTCGTCGTCAATCTCGTAGGCACCGGCGTTGTACTCCTTGGAGT

TCTGGCAGACCTCGGTCGAATCGACACCCGGCGCGAAGCGCTCTTTGTAC

TCCTCGGGCGGGTGGTACGGCAGGTGCGCGTCCATGAGGTTGACGAACGC

GAAGAACTCCTCGGAGTCGTCGATGAAGTCGATGGTCTGGTCGACGACCG

CGGGCGTCTTCGAGTCCGCGCCCTCGCCGCCGGCGAGATACTCGTGGGCG

GTGTTGCCGAGGCTGACGAGTTTGTCGGCGAGCGTCCGCAGCGCGTCGCT

GTCGTTCATCGTCTTCCAGGCCTTCGCCAGCGGCCCCGACAGGAACTCGC

CGGGCATGACCTCGAAGAAGTTGTCCTGCTCCGCGAAGCCGTCGGTCAGG

TGCGTATAGGGCGTTATCCACGCGTTCGAGGAGTAACAGGCGGTGTCGTA

GCCGGCCGCCGAGAGCGTCTCGGCGAGCGTCGTCGCGCCTTCGAGGTAGG

GGTTCTCCTGGTTGGCCCCGTGCTGGCTGGGGTACATCCCGGTGAAAAGC

GAGGCGTGGACCGGCAACGTCCACGGCGCGGGCGCGACGGCCTGCTCGAA

GACGGTCGCCTCGTCGGCGAACCGGTCGAGGCCCGGCGTCGTCGGGCGGT

CGTAGCCGTACGGCGTGAGATGGTCCTTTCGGACGGTGTCCATGACGACG

AAGAGGACGTTCTCGGGCGACTCAGCAGTCATACCCGAGGGTGGGGAGTA

GTTGCCGATAAAACTCCTGTTCTCGGCCGCCGCCGCGACGAAAACGTCCG

AACCGCCGAGTTAGAACGGGGCTTGCGGGCCCTGGTCGTCCTCGATATCG

GTCGTGTCGTCGCTGGTGTCGCCGGGGAACGACGGACTGCCGCCGCGGCT

GTCGCCGCCCATCCCGCCGTCGCCGCCCATGCCGGTGTCGGCGTGGACCT

CGTTGATCTCGGGAATCTCCTTGACCATGCGGGTCTTGATGGCCTGGATG

GTCATCGGGGAGATACCGCACCCGGAGCAAGCGCCACCGAGGAGAACGGT

GACCTCGCCGGTCTCGCGGTCGAGGTCTCTGATGGCCGCGCTGCCGCCGT

GCATCTGGATCTGCGGGAAGTTCCGACGGAGGAAGTTCACGACACGCTCT

TTGAGGTCGTCCTCGCCGTCCTGCGTCTCCGTGCTCATGCACGCGGCTTC

GAGGTGAACGTGCTTAGGCCTTTGGTTCGGGGTTACGCCTCGACGCCGAA

GACCCGCCGAAGCTCGCTTTCTATCTCGTCGACGTAGCGGTCGAGGACGG

CGTCGAGCTCGTCGTCGTCGATTTCGACCGCCGTCAGCCGCTCCGCGGGG

TCGTCGGGGAGCTGCACGGAGAAGTCGCCGTCATCGTAGAACGGCTCGGA

CTCGTTGAGAATCTGCTGGTCGACCCCGTGGATGAGCGACGAGTCGAACT

CGTCGTTCATCGTCTCGAAGGCGTTCTTGTACGCGCGCTGGAGTTCGGGG

AAGTAGTTGGCGTACTTGTCCTCGAACTTCTCGGGGTCGAACTCGGTCAT

ACTCGGAGCTTTCGCGCTCCCGGCAAAAAATCGGACGGTTAGTTCGTCGG

AATTGTGGATTTTGTATAGCAACGTAGCATTTATCGGCTGTTCGTCCTCT

GAACGGCGATTCCTCTCTTAGCGCGTCTCAAATACGGAAAATCGAGCGAC

GCGGGGCGTCACTCGATGAGCGGGCTGTCCACGCGCGCGGCGGAGAGTTC

GCTCGCCGCGAAGATGGACGCGAGCTCGGTTATCAGTTCGTCGTACGACT

CGTCGTCCTCGCGCAGCGCGTCGAGTCGCTCGACGGTTCGACGGTCCAGT

TCGACCGTCTCTGTTTGTACCTCCGGTTCGTAGCCGACCGGGTAGTCGTA

TTCGACCTCGTTCGAGTTGGCAGTCGCCATCGTTCACCCCACTCCGGCAT

GCGTGCCGGTGCCATGACATACACAAGCATCCGATATGAGGATTTCGCGG

GCTTCGACCCCGAGGGCGAACGCGAACGCGGTCTACACTCGGCCGTTTCT

TCCCCCACCTCGGCCATAATAAATCATATTCAGCTATATCGAATTGCTCC

CGTATCCGAATCGAGGGAGTCATAGCCAGTATGGGTTGTTTGAGGACAAC

CGATTTTATACTCGATAGGTTGTCGGACTCTAAATATATGCGTAAATATA

TTCCCAGACACGAGTCAATTCCTCTAGAAGTGAGTACTTTATCACGTTCT

ACCCGTCGAGCAGTTCGGTCTGTCGCGATTGCGGGCCGCACGACTCCGAC

AGGCGCTCTCCCGAGGTCGCGCCCCCTGATGTACGACCGTCGCATTCTCA

TCGACGCGCTGGCACACAGGCCGTTGTTCCGGCGGATGGTCAGACCGCTC

GTCGCCCGCGGCGTTCGTCGTCGCCGGCGTCGAGCGCGCCGCCCGCCCCC

GCTTGCTATTTGGTCACAGCGCTCGCAGTTCTCGTATGTCCGCCGACGAG

CCCCGCTACGACGACGAGGGGGTCCTGAAGAAGCAACAGTACAAACGGGA

GTTGCACCGCCTTCAGGAGGAACTGGTGAAACTCCAGTACTGGATAAAGG

AACGCGACCTCCGGGTCTGCGTCGTCTTCGAGGGGCGCGACGCCGCGGGG

AAAGGCGGCGTCATCAAGCGCATCACCCGCCGGCTCAACCCGCGGGTCGC

GCGGGTCGTCGCGCTCGGCAAACCCACGGAGCGCGAACAGGGCCAGTGGT

ACTTCCAGCGGTACGTCGAACAGCTTCCGACAGAGGGCGAGATGGTGCTT

TTCGACCGGAGTTGGTACAACCGCGCGGGCGTCGAGCGGGTGATGGGTTT

CTGCACCGACGAGGAGTACGAGGAGTTCCTGCGGACCTGCCCCGAGTTCG

AGCGGATGCTCGTCCGCTCGGGCGTTATCCTCGTCAAGTACTGGTTTTCC

ATCAGCGACGAGGAACAGGAACGGCGGTTCCAGAAACGAACAGACGACCC

GAAGCGACGGTGGAAGCTGAGTCTGATGGACCTCGAAGCGCGCTCTCGGT

GGGTCGAGTACTCGAAGGCGAAAGACCGGATGTTCGAACACACCGACATC

GACGAGGCCCCGTGGCACGTCGTCCACGCCGACGTGAAGCGCCACGCGCG

GCTCAACTGCATCTCGCACCTGCTGGACCAAATCGACTACGAGGACCTCA

CGCCCGACCCCATCGAACTCCCGCCGCGACAGGACGACACCGGATACGAG

CGGCCGCCAATCGACAGCCAAAACTGGGTGCCCGCGCGGTTCGGGGAGAA

CCCGGTCGACGATTAGTCCGCGTTTTCAGCCAACTGCTCGCCGACGATGC

GGTCTGCCTCGGCGATGAACGCCTCGACTTCGCCCTTCGGAATCGTCGCG

CCCGCGGCCACGTCGTGGCCGCCGCCGTCGCCGCCGACTGCACGGGACGC

CTCGCGCATGACCGCCGAGAGGTCGAGCCCGTCTCTGACCATCACGTACG

ACCCGCGCGAGGAGACTTTCACTTCGCCGTCTTCTTTCTCGGCGAACGCG

AGGACGGGAATCCCGTTTCGGGTCGCGTCGGTGCCGACGGCCATCCCGGC

GACGATGCCGACGATGGTCTCGCGTATCTCGTCGCCCGCGTCGAACCACT

GGAGGTTGTCCTCGACGCGGACGCCGTGTTCTTTCACCCACTGGAGGCCG

TTCGAGAGGTTCTTGCGGTGGTTCCGAAGGAGCCTGCGCGCCCGGTCGAG

CGCCGCGTCGCGCTCGCCGAGACAGACCGCGAGGCCCACGTCGGCGCGGT

CGTAGCGCGCGGTCGCGTTCAGGAGCGTCGAGAACTCGCTCACGTCGCGG

AGTTCGGTCCCCTCCCGCTCTCGAGAGAGCACGTAGGTCGTCCCCACGAG

GTCGTCGATGCGGGAGGCGGGAACGCCGCTGGCGATGGCGCGGCGCATTA

GGGCGCTGGCGAGCGTCTGGCGCTCGTCGCCGGTGAGGTCGACCCAGCGC

TTCCACTCGCCGTCGTCGTCGCGGCAGGGCACGTCGAGTTCGGTCAGGAA

CTCGATGGCTCCGGCCTCGTTGTTCGAGATGCCGGGGATGCGTACGTCCG

TCGCGTACTGGAGCAGTTTCGGGAGCGCGCGGGTCTGGCGGCCGTAGATG

CGGAGGTCGGTGCCCTCCTCGACGACGCCGACTTCGACGCCCTCGTCGAC

GATGCCCGCGTTGGCCCCGCGGAGTTCGCCGTTCGTGTCCTGCATGTCCC

CGACCGCGCCGACGACGGCGAGGGCGGCGAGGTCGCGGTTGTCGCCAGCG

TCGGGTTCGAGCGCCCGCGCGAGGACGTAGCTCGCGCCCGCGCCGGAGAG

TTCGCTCGCGCCGTTGAGACCGAACCGGAGCGGGTTGAGGTGGTGGTCGG

TCTCGACGCCCTCGGCGGGCTGGTGGTGGTCGGCGATGACGGGGTGGAAG

TCGCCCGCCGCCTCGTGGTCGGCGATGATGTCGAGTTGGCCGCTGCCGAA

GTCCGTGAACAGCACGGTGTCGTAGTCGGTCGCCGCGATGTCGGCCACGG

CGGCCTCGTCGAGTTGGCGGCAGAACACCGTCTCGAAGGGGATACCGGCG

CGTTCGAGCGCGGTCGAGGCGATGCCGGCGCTCGTCAACCCGTCGGCGTC

GATGTGCGAGGCGAGGAGCACCCGATCGGCGTCGCGGAGGCGGGCCGCGC

AGGCCGCCGCGTGCTCTGTGAGTTCGGGGACGGGTCCGTCCATTGGTGTG

GTACTCACGCCCTCTCCGGCATAAACGCTGGTAGTCCGGCCACGCGACCC

CTTCGTTTCCACTCCAGTTCGACGGCGAACACGTGTCGCTCTTGCCGCGG

CTTCGCGGATAAAGACTCGTCTCCCGCCGTCAGATTCGCCGTCTGACGAC

GAACAGGAGGACGGTCGCCAGCACCGTCGAGCCGGCGACGAGCGCCCAGC

CGGCCCCGTAGCCCGTCGATTCCACGACGAAGCCGAAAAGCGGCGGGGCG

ACGAGGCCGCCGACGTTGAGCGCGGTCTGGCCGCCGGCGGTCGCCGCCCC

GATGTCGCCGTCGTCGACGACGCCGCTCAGACAGGAGTAGAAGACGCCGG

TCGAGCCGTGGATGGTGAGGCCGAGCGTCACGAAGACGGCGATGGTGAGC

GCGAACGAGCCGCCGGTTCCGACGAGGAGCGAAAAGAGGGCAACCGCGCC

GGCCAGTTGGACGAGCGCGACGGTGGCCGCGCCGCGGGGCCCGCCGAGGC

GGTCCGCGAGGCTTCCCGCGCCGATGCGGCCGACGCTTCCGGTCACCTGC

GTCGCCGCGAGGACGCCCCCGGCGAGCGCGGGACCGGTTCCGACCACGTC

CTGCACGTAGAGGACGGTGTAGCCGAGCATCGAGAAGATGGACGCGCCGA

TGAACAGTCCGGCGGCGACGAGCGCGACGTAGGCGCGGTTGCCGCCGAGG

CCGGCGAGTCGGGGGCGTTCGAGCCGTCCTGTCCCGAGGTTTCCGCGGTA

GCGCGTCGCGAAGACGAGCGCGTAGCCGGCGGCGAAGACGGCGATGGCCC

AGAAGCCCACCTGCCACGCGGCGACGACGGCGACGCCGGTGACGACGAGC

GACGACGCCCCGCTGCCGACGGTGACGCCGACCTGCTTCAGCCCCATCGC

GAGGTTCTTGCTCCCGGCGGGCGCGGCCGCGACGATGCCGCGGTTCGACG

CCGGCATCGCGGTCGAGTAGGCCGCCCCGAGAAGCGCCACCGTCACCAAC

AACAGGAGGTACGACGGCGGCGCGAACGTGACGCCGACGAGCGCGACCGA

CAGCGCCAGCAGGCCGACGACCATCACGGGCTTTTCGCCGTAGCCGTCGA

CGGCCGCGCCGCTCGGAAAGAGAAACACCGTGTAACCGAGCAGGCCGGCG

GTCAGGAACAGTCCGACCAGCGACTCGGAGACCGAAAACGCGTCGCGGAC

GAAGCCGGTCGCCGCGAAGATGGCGTAGTAACAGAGGCTCGCGGCGGTCT

GCCAGCCGGCGACCGAGCCGACGGCGCGCCACGAGCCGTCCCCCGAGTCA

TCTCTCGTCTCGACTCTCGCCTCCTCTCCCGCTTCGTTTCCCGTCACGTC

ACTCGTGATACGTCGGCGCGGCGGTCTCGACCGCGGCGACCGCGAGCGCT

TCGAACGTCGCCTCGTCGGGGACGACCGTCACGTCGATGCCGTGGGATTC

CGCGGTGTCGCGGGTCGGCGGCCCGATTGCGCCGACGACCGCGTCGTTCA

GGCCGGCGATGGCCTCCTCGCGGATACCGCGCTCGGCGGCGGCGTCGAGG

AAGTGTTCGACCGTGAGCGACGAGGTGAACAGGGCGGCTTCGAGGTCGCC

GGCGGCGGCCAGCTCGGCCGACTCGCCGGCTCCCTCGGGGCGGACGAGCC

GGTAGAGCACCGTCTCGTGAACGTCCGCGCCCGCGTCGCGCAGGCCGTCC

GTGAGGACGGCACTGCCGTGGTCGCTCCGGGCGACTTCGACCGTCGCGCC

GTCCACGTCGGGCGCGAGGTGGTCGACCAGTCCGGCCGAGGTGTACTCGT

CGGGGACGCGGTCGACCGTCCAGCCGGCGTCGCGGGCGGCGTCGGCGGTC

GCGGGGCCGATACAGCACAGCACCGCGTCGCCGGGGTTCCAGCCGGCCTC

GTCGAGGAGTTCGACGCCGGTCTTACTCGTCAGGACGACGAACGCGCCGG

GTTCGGGCGTCGCGCCGGTCGGCTCGATTGCGAGCATCGGGTCGGGGACG

GGGGTCGCGCCGAGCGAGTCGAGGAGTTCGACGGCGCGCTCGATGCGGTC

GTCGTCGGGGCGGAAGACGGCCGCGCGGACCTGCTGGCTCATTCGTCCCC

TCCCGCACCTCCGGAGGTCGGGTTCGCGTCCGCGCCCATCTCGATGGCGT

CCGCGCCGCCGCCTTGCAGGAACCCCTTCACGCGGTCGCGGGTCGCCGCG

ACCTCGCCGATGACGGTAATCGCGGGCGGCTCGATGTCGGTCTCGTCGCG

CACGTCGACGATGGTGTCGAGCGTGCCGGTGGCGACGCGCATATCGGGCC

ACGTCGCCCGCTCGATGAGGGCGACCGGCGTGTCGCCGGCCAGCCCCGCC

TCGCGGAGTTCGGCGGTGTAGAGCGGGAGCTTGCCGACGCCCATGAGGAC

GACGAGCGTCCCGCCGGTCGCGGCGAGCGCGTCCCAGTCGACCGCGGACT

CGTCTTTCGTCGGGTCCTCGTGGCCGGTGACGAACGACACCGACGAGACG

TGGTCGCGGTGGGTGACGGGGATGCCCGCGGCACCGGCCCCGGCGACCGC

CGAGGTGATGCCCGGCACGACCTCGAAGGGAATCCCGTTGTCTGCGAGGT

GTTCCATCTCCTCGCCGCCGCGGCCGAACACGAACGGGTCGCCGCCCTTC

AGGCGGACCACGTCCTTGCCCTCGCGGGCGAGTTCGACCAGTCGGGTGTT

CGTGTACTCCTGCGGGGTCCACTCGCCGCCGGCGCGCTTGCCCACGTCCT

CGCGCTTCTCGACCGGAATCATCTCGAGAATCTCCGGGCCGGGGAGCTTG

TCGTGGAGCACCACGTCCGCCTCGTCGATGAGGCGGGCGGCCTTCATCGT

CAGCAGTTCCGGGTCGCCCGGGCCGCTGCCGACGAGGTGGACCGTGCCGA

CGCCGTCGTCGTCGGTCGCGTCGTCGTTCGAGTGCTCGTCGGCTCCCGCG

CGCTGGTCGTCGCCCGTCATCTCACTCCTCGGCCTCCTCGCGGGCGGCGT

CGACTAACTGACCCGCACCGCGGTCGCACAGGGCCGCCGCGAACTCGCGG

GCGGCGTTGGCGTGGTTGCCGACGGGGAGGTCGCGAGAGGTCTTGATGGA

CTCCGAACCGTCGGTCGCCAGCACCTGCACGTCGACGTGGACGTGTTCGC

CCTGCAGGAGGGCGTGGACGCCGACGGGGGCGATACAGCCGCCGCCGAGT

TCCGCGAGGATGGTCCGCTCGACGGTCGTCTCGACGCGGGTCCGCGGGTG

GTCGAGTTTGTCGCGGATGAGTTCGATGACCACCGAGTCCGCGGCGGTCA

CGGCGATGGCCCCCTGTCCGGGTGCGGGGACGAACGTCGTCCGCGGCAGG

CGCTCGTAGTTCACCTTCTCGGTGAGCCCGCTGCGCTTGAGCCCGGCCTC

CGCGAGGACGATGGCGTCGTACTCGGTTTCGACCTGCCGGCCGAGCGCCT

GCCGTTCGAGTTCGGTGAGCCCCTCGAACCACTCGTCGGCGGTCTCGTCG

AACGCCTCGTCGTGGTCGGTCTTGCCCTTCTTGCCCTGTCGCTCTTTGTC

GTTTTCGACGCGGGCCTCGTGTTCGCGCTGGAGGTGCGTCGCCAGCAGTT

TCTCGATGCGGGTGTCGACGTTGCCCCGGAGCGGCTCGACTTCGAGGTCG

TCGCGGTAGTTCAGCAGTTGGGCCTGTCGGCGGAGCGACGACGTGCCGAC

GACGGCTCCCTCCGGGAGGTCGTCGATGTCGTGGCCCTCGGCCGTCACGA

GCACGTCGCCCGCGGGCGCGCGCTCGGGCACGCCGGCGACGACGAGGTCG

GCGGGCTTCTCGGTCGGCATGTCCTTCATCGAGTGGACCGCGGCGTCGAC

CTCGCCGTCGAGGACGCGCTCGTCGAGCGCGCGGACGAACGCGCCGGTCT

TGCCCAGTCGGTGGATGAGTTCGTCCTGAATCCGGTCGCCCGTGGTCTCG

ACTTCGACGAGTTCGACGTCGAGTCGGCGGCCCGCGAGCGCCCCCTGCAC

GCTCGCGGCCTGCGCGCGAGCGAGCGCCGACCCTCGCGTCGCCAAGCGAA

GTGTTGTAGTCATACGCGTCAGTCCGGCCCCCGCGTTGAAAAGCCCACCA

GTTCGCTCCCGCGCGCCGGGTCGAATCCGAACGGAAGACCGATACGGTCC

CCGCGCGACGACCCGGACGATGCCCGCCCTCGATTCGGCCGTCCGGCAGG

TCGGTGACTTCGTGGTCGTCGCCCTCCTCCTGTTCGGCCTGACCTCGGTC

GTCGCGCCCCTCGACCTGTTTCTCTCGTCGGTCGGCGTCGAACCGCCGTG

GTTCGCCGGCCTCGTCGCGGCCGCGCTCGTCGCGCTCGCGCTCCTCCTCG

CGCGACCCCTCCGCCTCCGACTCGTCGCCTGCGTCTGGGGCGTCGGCCTC

GTCGTGACCGCCGTCTGGATTCCGCTGTTGGTCTTCCTCGAACTCCAAGG

AGACCCGGTCGGTATCCTCGTCTCGTGGGCGGCCGCGCTCGGCGTCGGCG

TCGCCCTGACCTACCCGCCGCTGTGGCGGGCGGCCGAGGCGCGACTGCGG

GTCGAGTGAGGCGCGTTCGGCCCGGGCCGGGCCGGGTCGAAGTCGTGGCG

GACGGTCCTCGCTCGCCTCGGCCGCCTCGCAGTCGTGTCGAAGTGCCGAA

CAGTTATCGGCCTCCGCGGCGACGACTCCGACGTGAACAGGTCCGCCCGT

CTCGCCCTCACCTTCGGCGTCGCGTTCGTCGTCGCGCTCCCGCTCGGCTT

CATCTTCGCGCCGGACCCGACCGGCATCGCCCCGCTGTTCCTCACCGCCG

GACTGGCCGCCGTCTTCGGACTGCCGGTGTACTTCGGGCTCTCTCGGGCG

AGCGGGTCGGAGTCGTGAAAAAGCGCGCGACCGGCGAATCGTCGATTCTG

GTTATAGCGCCGCTTTGTACGCTTCCAGCGTCTTCTCGATGTCCTCGTCG

GTGTGGGCGTAGGAGACAAACTGCGATTCGAACTGGTTCGCCGTGAGGAA

CACGCCCCGGTCTTTCATCTCCTGCCAGAAGACGCGCTCCCAGCGGTCGG

TCTCGGTGTTCGACACGTCCGCGCCGGTCTTCGGACAGGCGTCGTAGTTG

GGACACGACTCGACCTGCGAACAGCCGTCGACGCAGGCGTCGGCGCGCTC

GGCCGCGCCGTGGCGCGTGAACACCGTCTTGAACATCGAGTCCGTGCCGA

CGACGGTGTACTCGGGTGCTTGGTCTTCGAGAATATCCGTGATACCCGCC

CGAAGCTTCTCGCCGAGGCGGTCGACGTGCCCGTACACGTCGTTTTCGGC

GGCGTACTTCAGGTACTCGTGGCCCGCGGCCATCGTGACCGGGTGGCCCG

AGAACGTGCCCGACTGGAACACGTCGCCGCCGGGGGTGAACTGCTCGACG

AGTTCGGCCTTCCCGCCGACCGCGCCGACGGGGAAGCCGCCGCCGATAAT

CTTCCCGAACGTGGTGAGGTCGGGCGTGACGCCGAACTTGCCCTGCGCGC

ACTGGAGGCCGCCGACGCGGAAGCCGGTGATGACCTCGTCGAAGATGAGG

AGCGCGCCGTGTGCCTCGGTCACGTCGCGGAGGGCTTCGAGGTAGCCGTC

GACCGGGTGGACGATGCCCGTGTTCGCCAGAATCGGCTCGGTCAGCACCG

CCGCGATGTCCTCGCCGTGTTCCGCGAACACCTCGCGGACGGTCTCCTCG

TCGTTGAACGGGACGGGGATGGTGTGGTCAGCGAACGACGAGGGGATGCC

GGGCGTCGAGGGCTTCGCGCCGTCGGGGCCGCCCTCGACGAGGGTGGACT

CCTGTGCGCCGTGGTAGCCGCCCTGCATGACGACGATTTTGTCGCGGCCG

GTGACGCCGCGGGCGAGGCGGACGGCCGAGACGGTCGCCTCCGTGCCGGA

GTTGACGAACCGCAGCATCTCGACGGAGGGGACGTGCCGCGCGACGAACT

CGGCGTGTTCGACCTCGATTTCGGTCGGCGCGCCGTACATCGGCCCCGCG

GCGGCGTGCTTCTGGACCGCCGACTGGACGGGTTCGGGCGCGTCGTGGCC

GTACAGCAGCGGCCCGTAGCCCATCACGTAGTCGACGTAGCGGTTCCCGT

CGGCGTCGATGACGTGCGCGCCGTCGCCGCGCTCGACGAAGAACGGGTAC

GGTTGGGTCGCCCGGACGGAGGAGTTCACGCCGCCCGCGAGCACGGACAG

CGCGCGGTCGTACAGCGACCGTGACTCGTCGTGGTTCATGCGCGGCGGTT

CGGCCGCTCGCGGGAAAGACCTTACTGGGTCGGCCGCACCTGCCGCCGTC

GAACTGGCGGACGAGGCGATGCCCGGCCGACCGGCGTCGCGGGACTGCGG

CGACCTATCGGTTCGCTCCGGCGGCGGCGACCTACCGCGCCGTTCCGTCC

GCGGCGGCGTCAGCACCGTCGGCGTCGCCGGCGTCCTCGACCGACCCGGC

CAACAGCAGTCCGACCGCGTCGTTGAGCGCGTGGATACAGACGAGCGCGA

CCACGTCGCGCACGAGGAGGTACATAATCGTCGTCAGGAGCGCGGGGACG

GCGATTCTGGCGACCGCGCCGCGCTCCCAGCCGACGGCGTGGCTCGCGGT

GAACGCGGCGAACGAGACGCCGCCCGCGAGAAGCGGGCTGCCGGTGAGTT

CGAGCAGTCGCTCGATGGCGTAGCCGTGGAACAGCACTTCCTCCGTAATC

CCGGCGGTTCCGGCCACGAACAGTCGGTGTCTGACCGACAGCGAACCCAA

CCCGGACATCCCCTCGTCGAGGCCGCCGACGCCGAGTCGGTCGAAGACGG

GCGTCGTCACCGCGTTCGCGGCGAACAGGACGAAGACGCCGCCGCCGACC

ACCGCGACGAACGCGAGGGGGGCAAGCGAGCGGCCGGTCATCGAGGACAG

CGGCCGCCCCTCGACGACGAGCACGTAGCCGCACAGGAGGGCGAAGACGG

CCCACTTGTTCGCGTCGCTGACGAGGAGGTCGGCGCGGAGGTCGTCGGGG

TCCGAGAGATATCGGTCGGCGAGCAGGCCGACGGCGGCCATCCCGAACAG

CGCGACGGCGAGTCCGCCGAGGACAGCGACAGTCACCACGTGGAGCCACC

GCTCATCGTCACGACGCTCGGTGCGGAGGGGAAAAACGGTTTCGCGTGGG

TTCGGCTCTCGCCCCGTGGGGGGTCACCGGTACGTTCGGCCGAGCGCGTA

TAACCCCGTCGCCCGTCAGGCTGACGACGCCGGGGACGGCTCGGCGCGAC

CGAACCGAACGAAAACAGCGGTCGGCGGCGTTCAGACCGCGTCGCGGCCG

GTCTTGCCGGTGCGGACCTGCACCGCGCTCTCGACGGGGAGGGTGAACAC

CTTCCCGTCGCCTTTCTCGCCGGTCTGGGCGGCGTCGGCGATGGCGTCGA

CCACGTCGTCGGCGGGGATGTCGGCGACGACGCACTCGATTTTGACCTTC

TGGTGGAGGTCGACGGTGTACTCCTCGCCGCGCCACTGACTCTTTTTCGC

GGGCTGAGAGCCGCGGCCGGAGACGTTGGTGACGGTGAGCGACGGCGCGC

CGATTTCGGCCAGCGCGGTCTTCACGTCCGAGAGCTTGTCGGGGCGGATG

ACCGCCATCACCATCTTGATTTCGCCGTCGTTCGGAGCGTCTGCATCACT

CATGGGTTAGTCCTCCCGTCCCGTCGTCATGAATCCATCCCCGCTCGGGA

CGCCGGAGCCGTCGGTGCGGATGCCGGTGTCGTGGTCGGGCGAGCCGAAC

TCGGGGTAGGTGTCCACGCCGTGTTCGGCCGTGTCGAGGCCCTCGCGCTC

GTGGTCCGAGGAGACGCGGACCTGTCCGAGGGCGCGGAAGCCGTCGAAGA

CGACTGCGGTGGCGACGAACGTCCAGATGGCGATGACGCCGACGCCGATG

ACCTGCGGGACGGCCAGCGAGACGAACGACGCGCCGTCGTGCCACAGCGG

GACGGCGAACACGGGGTAGAGCAGCGTCCCGAGGACGCCCGCGGAGCCGT

GGACGGGGAAGACCGCGCACACGTCGTCGATGCGGAGGCGCTTTTCGACG

AACTCGAAGACGACCGGGAGCTGCGCGCCGGCGAGGAGGCCGACGACCAG

CGCGCCCGGCCAGACGATGTCGTCGGCGATGGCGGTGATGCCGACCAGGC

CGGCGAGGACGCCGTTGGCGACGTAGAGCGTGTCGACCTTGCCGGTCTTG

TACATGGCGACGCCGCCAGCGCCGATTGCGCCCGCGGCCATGCCGAGGGT

CGTCACGAGGGCGACCCGGCCGACGTAGGCGAACGAGCCGAGCGTGACCG

CGCCGTCAGCGTACGCGAGCGGCGCGGCGGCGGTGCCGACGTTGAAGCCG

TACCAGCCGAACGCGAGGATGAGCGTACCGAGGACGGCGAACGTGATGGA

GTGACCGGGAATGACGTTCGCGGTGCCGTCGGGCTTGAAGCGGTCCATGC

GCGGGCCGATAATCCACGCCGCGGTGAGGCCGGCGATGCCGCCCATGCCG

TGGACGATCATGCCGCCGGCGAAGTCGTGGAAGCCGAGGGCGTCGAGGAA

GCCGCCGCCCCACGTGAAGCCGACCACGACGGGGTAGATGACGCCCGCGA

TGAGGATGGTGTACGTGAGGTACGCGCGGAGCCGGGCGCGACCCGCCACG

GCACCGGAGACGATGGTGGCGGCGGTCATGGCGAAGACGGCCCCGAACAG

CCAGTCGACCCACGCCGTCGTCGCCGAGGCGTCGGGCGCGTAGAGGCTCA

TGAACGCCCCGGTGATGTCCAGCGCGGAGCCGCTGGTGAGGGCGGCGACG

ATGGACGACACGGCCGCGCCGAGCAGGAAGAACACGATGACGCCGATACT

CCACGTCAGCAGGTTCTTGGTCAGTTGGTTGGCGACGTTCTTCGCGCGGA

CCTGCCCGGCTTCGAGCATGGCGAAGCCGGCGTGCATGAAGAAGATGAGG

AACGTGACCGTGAGGACCCACACGAGGTTCACGCCCTCGACGACCGAGGC

GAGGTCGGTCTGCAAGGGTGTCAACATACGATGCCTCCGGCGAGCGGTTC

GAGCTGGGAAGTTGGACAGTCGTTCATATTGGTGCTCGATTCGCGACCGA

ATGTCTCGCGAACCATGAGAGATGCTATCTCAAGACGTTCATATAACGGT

TCGCGTTGACAAACGTCTACAAAATGGTGTTCTGAGAGACGTATGTCCGA

TATAAGATGAAATATGTCTTGTATAAGGTCGTAAGGTTGGCACTCAGTGA

ACAAATGTTGCCACACAACCCTACGAACGTAGTGTCGGAAAAGTAAACAG

AACGACGTGGGGCGTTCGTCGAACCCCGGTCAGGTGCGCACTCGCGCGGA

CCACGACGAGAGCGGCGAAAGGAGACTTCCAGTCGGCTTCGGTCGCAGTT

GCGCGCTCAGACCGCCGTCTCGCCCTCCTTGCCGGTACGGACCTGCACCG

CGCTCTCGACGGGGAGCACGAATATCTTGCCGTCGCCTTTCTCGCCGGTC

TGGGCGGCGTCGGCGATGGCGTCGACCACGTCGTCGGCGGGGATGTCGGC

GACGACGCACTCGACTTTGACCTTCTGGTGGAGGTCGACGGTGTACTCCT

CGCCGCGCCACTGCCCCTTCTTCGCGGGCTGGGAGCCGCGGCCGGAGACG

TTGGTGACGGTGAGCGACGGCGCGCCGACCTCTGCGAGCGCGGTCTTCAC

GTCCGAGAGCTTGTCGGGGCGGATGATGGCCATCACCATCTTGATGCCCT

GTTCTCCGTCGCTCATTGGCTGTCACCTCCGTTGGCTTCGACGGTCTCGT

CTTGCTCGACGCCACCGTCGGTGCGGACGTCGCTGCCGCCGTCGGTCGCC

GTGGGCGAGCCGAGCGCGCCGTCGGGACCGGCGTCGCCGACGAATTCGGG

GTAGACCGAGACGCCGTGTTCGCCGGCGTCGAGGCCCTCTTCTTCCTCTT

CTTCCGAGACGCGGAGGCCGAAGACGGCGTCCGCGATGGCGAAGACGACC

GCCGAGGCGATGATGGTCCACGCGGCGATGACGACCACGCCGACGACCTG

CATCACGAGCTGGGTGCCGGAGAAGCCGCTCACCGCGAAGACCGGGATGA

GCGCCGTGCCGACCGCGCCCGCGACGCCGTGGACGGCGAAGACGCCACAC

ACGTCGTCGATTTTGAGCGAGTCGACGGTCCAGCGGTACGCCGGTAGGAC

GATTGCGCCGCCGAGCGCGCCGAGGATGAGGCCGCCCCACCACGTGACGT

GGGGGACAGCGCCGGTGACTGCGACGAGTCCGGCGAGCAGGCCGTTCGCC

ATCCAGAGGGGGTCGGGCTTGCCTTGGTAGCTCGTCGAGACGATCATCGC

GGCGACCGCGCCGGCGCCCATGCCGAGGGTCGTCACGAGGGCGACGCGAC

CGAGCGCGGCACCCATGAACTCGAGTCCGCCGCTGTCGGTGGCCGCGAGG

ACGGTCGCCTGCGTGCCGACGTTGAAGCCGTACCAGCCGAACGCGAGGAT

GAGCGTGCCGAGGACGGCGAGCAGCATCGAGTGGCCCGGGATGGGCTGGC

TGTTGCCGTTCGAGTCGAAGCGGCCCTTACGCGGGCCGACCATCTTCGCG

CCGACGAGGCCGGCGACGCCGCCGCACATGTGGACGACGGTCGCACCGGC

GAAGTCGAGGTAGCCGACGCCGAGCGCGGCACCGATGTAGCCGCCACTCG

AGAGGAGACCACCGGACCACGTCAGACCCTGCACGACGGGGTAGATGAAC

CCCGTGATGGTCGCCGCGAAGACGATGTACGCGCGGAAGTCCATGCGCTC

TGCGACCGCGCCGGAGACGATGGTGGCGGCGGTCATGGCGAAGACGGCTC

CGAAGAGCCAGTCAATCCACGCGCCGGAGTCGCCGATGTACGAGAACGCG

GCGGCGACGTCGAAGCCGCCGGGCGACGTGAGGCCGCCGACGATTGTCGC

GACCCCCGCGCCGACGAGGAAGTAGACGAGCACGCCGAGCGCCCAGTCGG

TCATGTTCTTCATCAGTACGTTGCCCACGTTCTTCGCGCGTACCTGCCCT

GCCTCTAAGAGCGCGAAGCCCGGCTGCATGAAGAAGATGAGGAACGAGAC

GACGAGGATCCAGACGTAGTTGACCCCCTGCGCGATAACGCTCGGGTCGA

CTTGGAGCGGTATCACGACTGCCCACCCCATGCGTCCACGAGTGACCGTT

CAGCTACTTTTGCTCCGTATCCGTGTGGATTCTGGTGCTTCATCACGGTT

CGGCTTCATGGTGAGTTAGTACATAAAGTGCGGGGTTTATTTTGTCAAAT

TTAGTGGGACGTAATACCACAACTGCACAAAATAGGCTAGAATATTACGC

ATATAAGGTTGTATATCGTCCAAGGATTTTGAGATTATCGGGTCTTCTGT

GGACGAACGTTCAGGAAAATGTTGCCTGACAAGTGTGTTTTGGCCGTGCG

CACCGCGGTATCGTGTCACGTAGTTCGGAGTTCGAATTGGCGGGTTCGGG

GCGTTCGACTCGGCCGTCGACGCCTCAGTCGAGCTGCCGCGCCACGCGCT

CTGCGAAGTACGTGATGATGAGGTCCGCGCCCGCCCGTTTCATCGACAGG

AGCGACTCGTGAGCGACCCCGTCGAGGTCCAACCAGCCCTTCTCGGCGGC

GGCGTGGAGCATCGCGTACTCGCCGGAGACGTTGTAGGCGGCGACCGGGT

GGTCGTAGCCCTCGCGGATGGCGCGGACGATGTCGAGGTACGGCAGGCCC

GGCTTGACCATCAGCACGTCCGCGCCCTGTTCGACGTCGAGGGCGACCTC

GCGCAGCGCCTCCCGGGCGTTCGCGGGGTCCATCTGGTAGTGCCGTCGGT

CACCGAACGCCGGCGCGCCGTCGGCCGCGTCGCGGAAGGGGCCGTAGAAG

GCGCTCTCGTACTTCGCGGCGTAGGACATGATGGGTATCTCGTCGTGACC

GGCGTCGTCGAGCGCCGTGCGAATCGCGCCGACCATCCCGTCCATCGAGG

CCGAGGGGGCGACCATGTCCGCGCCCGCCTCGGCGTGGGAGACGGCCGTC

TCCGCGAGCAGGTCCAGCGTCGGCCCGTTTTCGACCGTCAGCGTCGGGTC

GTCTTCGGCGTGGTCCTCCAGCACGCCGCAGTGGCCGTGGTCGGTGTACT

CACAGAGACAGACGTCGGTGATGACGTAGGCGTCCGTCTCGGCGGTGATG

GCGCGGACGGCCTCCTGTACGACCCCGTTTCGCGCGTACGCTCGGGACCC

CTCGGGGTCTTTCGACTCGGGGATACCGAAGACGATGACGGCCTTGACGC

CGGCCTCGCGCACCTCGGCGACGCGGTCGGCCGCCTCCGCGACCGGCACG

CGCTCGTGGCCCGGGATGGATTCGATGGGGACGCGCTCGTCGGTCGTCGC

GTCCACGAAGACGGGCGCGACGAGGTCGGTCGCGTCGAGGGTCGTCTCCG

AGACGAGCGGCCGAATCCCGTCGGTTCGAAGCCTGCGGGGGCGGTCGGTG

AACTTCATACGCCGGGTTCGGTCGGCGGCGGCAAAATGCCGTCGCTCCCG

TCGGTTCGCGCCGCGTTATCCAAACCATAATGAACATGATTCCGCTCGAA

ACACGCATACGACATGAGCGCCTTCACCGCCCAACTCCCCGACTTCCTCC

GCGAACTCCTCGCGTCCGACCTCGCCCTCGTCGCCCTCTTTTTCGTGTTC

GTGCTGGAGGGTGCGATGCTCCTCTACGTCGCGCCGAGCGAACTCCTCGT

CCCCGGTGCGCTGGTGCTGGTCGGCGAGCGACTGCTGCTTCCGATTCTCG

CCGTCGCCGTGCTCGGCGCGACCGTCGGTCAGGTCGGACTGTTTCTCGTC

GCCAAGCGGGGCGGCCGCGAGTATCTCCTGTCGCGGCCGTGGTTCCGCGT

CAGCGAAGACTCCCTCGACCGGTTCGACGGCTAGTTCGACCGCTGGGGTC

CCGTCGTCGTTCCGCTGAGCAACGCGATGCTGTTCACCCGCGGGATGCTC

ACCGTGCCGGCGGGGCTGTCCGGGATGTCGGTAAAGCGGTTCCTCGCGCT

CTCGGCGCTCGGGACCGTCGTGTTCGAGAGCGCGCTCGCGGCGCTGTACG

TGTTCGGCGGCCAAGTGCTCGCGTGAGCGGACGCGCCGCCGACCGAGGCG

GGTCGGCGCGGCGGCGGTAGGCTGACGTTAGTGGAGGGACTGCTGTGGGG

TGGCCGTCACTTTGTTCGGTTAATATCGAGTTTTTCCTTCAGGGCGGCGA

GTTCCTCGGATTCGGCGAGTTCCTCGACCCGCTTGCGGAAGTGTCGCTCG

CAGAGGCCGACCTTAATGTGGTCTTTCTCCGCGGCGTATGCGGCCTCACG

GTCGCAGTAGTGACACTGCATACCGCCCCTTCAGTTCCGATAGGATTTAA

CTCTACGCCCCACGGCACGTTCCGGGTTATTCACGGACTAACACGGTTAG

AGACCGTCTACCGCCGCGGCGAACCGGGCGAAGTCGGTCTCCGGCGGCCC

GGCGCGGCCGAGCGTCTCGTCGTGGGCGTCGCTCCCGCCCGTGGCGAGCA

GGCCGTAGCGCTCGACGGCCTCCTCGACGAGGTCCGCCTCGACATCGAAG

CCGTAGGGATAGTAGCGTTCGACGGCGTCGAGGTCGGCACAGAGTTCGAG

CGCGCCGGCGGGGTCGCGGTAGCGAAACGGGTGTGCGAGGCCGACCACGT

CGCAGGCGTCTCGCAGGGCCGCCACGCCGTCGTCGAACGTCGGGAGTTCG

CGCGCGACGTAGCAGGGACCGTCCGCGCCGATGAGTTCGTCGAACGCGCC

CTGGTAGTCGTAGGGTGCCTCGCTGGCGTCGATTGCGCGGGCGACGTGCG

GCCGGCCGACGCCGTCGTGGATGTCGATCTCGAGGCGAACTCCGGTTTCT

TCCTCGATCGCCGCGACGATTTCGCGCGCTCGTTCGACGCGGTTCCGCTG

GAGTCGTTCGACCGTCTCGACGAGAGCGGGCCGCTCCCTGAGACCGTACC

CGAGGAGGTCGACCCGGCGGTCGCCGGCGTCGACGCGCAGTTCGATGCCG

CGGACGACTGTCACGCCGTCGCGTTCGACCACCGGCGCGTCGAGCTCCGG

GTGATAGCGGTCGTGGTCCGTCACGGCCACCACGTCCACCCCGCCCGCGC

GGGCCGCGTCCGGGAGCGCGTCGATAGTGAGCCGGCCGTCCGAGGCGGTC

GTGTGGAGATGGAGGTCCGCGACCGGCGGGCTTTCGTCCATGTCTCGTGG

TCGGGGCGAGGGGGAGACAAACGTTGCGCCCAGTACGTCATTAAGGACGA

TTAAGGACTCCAATGCCACTAATCAAATACATGGAAAATCATTAAGAATG

ATGGCGGTCCAGTTGTGGGTGTAATGCGCCTGAACGGACTCGGCGACGCG

ATTGATCGCCACGAATACCCGATTAGCTCGACCGACTTCGCCCAACGTCA

CGGCGACGAAGTAATCGAGCTCCAGAACGGTCAGGAGACCGTCGCACAAA

TCCTGGCCCGTCTCGGCGACGAGACGTACACGTGCCCGCAGGACGTGCGG

GACGCCCTCTTGACCGGTGTCGGACACGAGGCAATCGGTCGCCGCTACTA

CAGTGACCGCGACCCGTCCCCGCTCGGTGAGAACGGACCAGAGATGGTCT

CGTTCTGAGGTCGGCTCTCGTCTCGCTCCGATTTTTCTTTCTCCCCGCGA

TTCCCGAGCCGTCGGCTCGGCGTCCCGCTCGTTGATTCGTTCATTCGCTG

ATTCGCCCGTGTCACCGCCGTTCACGGGCCGAAGCGGCCGACAGCCGACG

GCCGACAGGAGTCCGCCCCGTCACTCGGTGAGCCACGGCAGGTCCACCGG

GACCGACCCCTTGGAGTAGCCTTCGACCGCTCCCGACGGCCGGAGGCGGA

AGACGACGGCCGGTCGGTGCCGAACGTCGGGCTTCTCGCCCATGATGGCG

GCCCAGTCGTCGTCGGGGAACGACGAACAGTCGACGAACAGCGTCACGCC

GCCGCCGTGTTCCGCGAGTTGGCCTTCACCGTTGGTCTTGGTCTGAGCCG

TGTCGCGGATGGCGGCGACGGGGTTGCTCACGGAGCGTCGGTTCGGCGGC

AGCGGGCGCGTCACCTCGATGAGCGCGCCGCCCGCGCGGGTGTTCTCGGC

GCGGAAGTCGATGGAGTGGCCGGTCGCCACGGCGATTTCGGGCGTGATGT

CGTAGCCCGCGTCGTCGAGGAGGTACGCCACGTCGAACTCGCCCATCGCC

GCGGCCATCCGTATCGGGTCGAAGTACTCCGAGGTGCCGAGCTTTCCGGC

CATGACCTCGCGGTACTCGTCGAGGACGCCCGTGGCGAAAAACGACTCGT

AGAACTCTAGGGCTTCCTTGCGGGTCGCGTCCGGGAAGCCCGCGGCGTGG

TCGTGGAAGAACTCGCGGGTCGTCTCGCGGCCGTCCTTCGAGAGGAACAC

CGGGAGGAAGAACCACGAGAGGTGCGAATAGGGCTTCAGCCACGGCGACT

GGTCGAACAGCTCGGCGATGAGCTCGCGCTGGGCCCACCGGGAGACCTCG

AAGGGGGCCTCGTCGAAGCCCTCCTTGTCGGTCCGCCACAGCGAACTCGG

CGTCTCGGTGTTGCCGATCCAGTAGGCGTGGTCGTCGGTCCACGCGAACA

GCGCGGTGTCGCCGTTGTCCATATCGAACCGTCGGGCCTCGTAGCCGTCG

GGCGGTGCGTACCACGGCGCACCCATCTCGGCACCGAGGTTGGCGTCGAG

CGGCTGGAAGATATCGCGGCCGATGCGCGACTCGTCCCAGCGACCGGGGG

CGTAACGAAAGCGAAGCGGGCGTGCCACACACGGTGTAGTTCGGCGATTG

TTTTTACGCTTTCCTGCGACAGCCCCGAATTTCGGCTCCATCCCCGCTCG

GTTCGGCGTTCCGGTCCCCCGTCTCCAGAATATCACGATTGTGAACGTCT

CCGATAGTTATATGTGGTACTATACCACATGGTAAGTCGAACCATGTCAA

TGGGGGCATACGACGAAGCCGAACACGAACGCCGCGAGCGGATGACGAGC

CAGGTCGAACCCGGTACCGACGACGACCGGAAGACGTACCACGGCAGCGT

CGAGTACGACTCCGGCGACTCGACCGACGCGCTGCTCGAACAGTTCAAGC

GGATGAAGTCCGACGACTGACCGTCCCCTCTCCTGTTCCTGCGTCCCTCA

CGTCCTCGTCAGCGCCGCCGCCGTCACCGAGACGACGGTCACGGCGACGA

CGTGCCCGCCGACTGCGAGCAACAGCACCGGCTGGTCCGCGACGAACGGG

AGCAACAGCACCTGCGCGACCGCGAAGCCGACGTTCAGAAGGTTCACTCG

CCTGACCGTCTCGGCGGTCGGACCGTCGAATCGGTAGCGAATGGCCCCCC

AGAGGCCGACGACGGCCGCCCACCACGACGTGCCGATTCGGTAACAGAGG

TCCCAGAGGACGAGCAGCGTCAGGTAGACGACCACGACCGGCGGCTCGGG

GCCGAACAGCGTCGTCATGAGCGGCGTCCCCGGCTGTCGCGGGTCGAAGA

CGAACAGATGCGTCACGAGCGCGACGAACGCGAGCACACCCAGCACGACT

TCGATGCTCGACCCGAACAGCAGGCGTCGATAGGGAGCGGGCACTCTCGC

GGCGCGGGTCCACTCGCCCATGCGGAGCATCACGACACTCCCCGCGGCCG

CGACGACGATGGCCGCGGTGCCCGCGACCGCCGCGGCCCACAGGTCGTAC

ACCCACGCGAAGACGAGCACCGCGACCTCGAACACGGCGATTTGGAGGGC

GATAGCGCCCGCCTCGCCGATTCTCACGCCCGGGAGCGCGCCGACGATGC

TCTCGTACACCCACGTCTCGCCGTACTCCGGGGCGTCGTTCATGTCCCGC

TCCTACGGCCCCGCGGTATATCAATCGCCGCCGCGCTCGGGATGCTCGGG

GCGGTCGGGGCGGTCGAGGCGCGCGGACGGGCGATCCGGGTCCCGTTCAC

GTCGGAGTCGTCGACGCCGGTTCGACGGTTTTCTCCCGCTGCTCGCGCTC

CTCGCGGAGTGCCCGCTCGACCGCGGCGTCGAACGGCGTCTCGTCCACCG

CGACGATGTCGCGGATGCGGTCGTCGCCCACGACGACGGGGTTTTTCAGC

CCCTCGATGAGCGGGCGGGCGACGCCCGTGTCCACGTCGGTCACGAGGCC

GATCCAGTACGACGAGAGCCGCGGCGTCAACACGGGCACGGGGACGATTC

GCGGCGCGTGGCCCAACTGCGCGCCGACGCGCTGGAGCATCTCCCGGTAG

GTGAGCACGTCGGGGCCGCCGATTTCGTAGGTCTCGCCCGCCGTCTCGGG

GTGGTCGAGGACGCCCGCGAGGTAGGCGACCACGTCCGCGATGGCGACGG

GCTGACACCGCGTCTCGACCCACTGCGGCGTCACCATGACCGGCAGGCGC

GTGGCGAGCTGGCGGACCATCTCGAAGCTCGCGCTCCCCGCGCCGACGAT

GATGGCCGCCCGGAGCGTCGTGAGTGCGGGCGCGCCCGACGCGAGAATGT

GTTCGACCTCGCGGCGCGACCGCAGATGCGCCGAGAGTTGGTCGCGGTCC

TCGCCGAGGCCGCCGAGGTAGACGAGTCGTTCGACGCCGGCGGACCCGGC

GGCGTCCACGAAGTTGCGGGCGGCGAGCCGGTCGCGCGCCTCGAAGTCGC

CGCCGGCGTGCATCGAGTGGATGAGGTAGTACGCCGCGTCCACCCCCGCC

ATCGCGGGGTCGAGCGTCGCCGGTTCGAAGATGTCGCCCTCGACCACCTC

CACGCCGGGCGGACCGTCGTAGCGCGCCGCGTCGCGGACGAAGACGACCA

CGTCGTGGTCGGCGTCGAGGAGGAGGGGTACGAGGTGCCGGCCGACGAAC

CCGGTCGCACCGGTCACGAGAACTTTCATGCCATACGGTAGCGCCGCTCA

CGATGTTAACGTATCGCCCGTCCGACCGACCACCGACCGACCACCGACTG

CCCGCTGACCTGACCGCGGACCGCCGTCTCCCCCGCCGCTCGCCGCCCGC

GACTCAGCCGTCGTCGCGAACCGCGTCCCAGCCGGGCGTTTCGGGCGCGC

CGCGAGCGACTTCGACCTCGTCGGGGTCGGGCGCGTCGCCGGCCGCGCTC

CCGAGGCGACCGGCGACCGCGTCCCACGCGGCGACCGCTTCGCCCGCCCA

CGACTCGCTTTCGAGCGTCTTCGCACGCGCCCGCGAGGCGCGGTCGCCGT

CGACCTCGGTCGAACCGGGGTGCGACCCGACCGCGACGCCGACGAACTCC

GCGCGGTCTTCGGGCGTCAGTTCGCGGCGTTTGAGGTGGTCGATAGTCCC

CGCCAACCGCTCGGGCTCCGGGTGGACGAACAGTTTCGCGCCGAGGGCGT

GGGGGCCGAGGAGCAGGCCGTGAAGGTCGTCCGGGGCCGCGTCGTCGAGC

AGGCGCTCGCCGCCGTGGTGTCGCTCGACGCGCTCGCACTCGGTCTCCTG

TTCGAGGACGAGGTTGTGCCCGGGGTACTCGGCGCACTCGCCGGGATAGA

ACTCGGTGTCGTGGATGCGGCACTGGAGGGTTTCGGGGTCGAGAAACGCG

CAGGCGCGGAGCCACGTCCGTTCCAGTCCGAACGGCGCGACGGGCTTCGG

CGGCTTGCGCATGCCGACGAAAAAGGCCGGCTTCCCGTCGACGGCCGCGA

TTTCGACGCCGTCGATTTCGACGCCCTCGCCCGGCGGCGCTTCCCACAGC

CGCGGGGAGAGAGCATTGCCGAACCCGGCCTCGACGAAGTCGCGAACCTC

GTCGCGGGTCAGCGGGACGAGGTTGTAGGTGTCGTCGAGCGGCGCGCGCG

GCCCGCGACGCTCGTGGTCGAGCGCGGCCGGCGCGACCGGTCGCCAGTCG

ATACAGCAGCCGGCACAGCCTTCGCAGTCGACGCGCATGGAACGAAAGTT

ATACGCCAACCGACAAGAACCTCTCGCCGGGTCGGCGGCGCCGGCCGATT

ACGTTTCCGCAGACGCGGCGGGGCGCTTTTGCCGCCTCGGCTCTTCGGTT

CCCTCATGACCTCCGAACCGTGCGACGCCTGCGGGAAGGGCGTCCGCATC

GCCGGCGGCATCGGCGACCTCTGGAACTTCCCCACCTCCTCGTCCGGCGG

GATGACGCTGGAACTCGTCGACGGCTCCGAACACTTCCTCTGTTTCGACT

GCATGGAGCGACTCCCGGGCGACCGCGAACCGACGGCCGAGGACGTGGCG

GCGCTTTAGGCTCCGATTTCTCGGCCTTGTCGATGCCGCGACCGCACCCC

GGAGCGGTGGATTTAGGCCCGTCCGAGGACTCCATTGACCGTGGACCCGT

CGCGCATCATCGACTCGTTTCCGGCCCCCAGTTTCCGCGGCGCGCAAGAG

CGGGCCCTGCGCGACATTCGCGACGCCTTCGCCGACGGCAACGACGTGGT

CTTGGTCCGCGCGCCGACCGGGAGCGGCAAGTCGCTTCTCGCCCGCGCCA

TCGCGGGGTCGGCGGCGACCGTGGACGAGACTTCGCCCGCGCAGGCGACC

GACGCCTACTACACGACTCCGCAGGTGTCGCAACTGGACGACGTGGCCGA

AGACGACCTGCTTTCGGACCTCAAAATAATCCGCGGCAAGTCCAACTACA

ACTGCATCATCCGCGGCGAGGAAGACACGCCGGTCGACCGCGCCCCCTGC

GCCCGCAAGCGCGGCTTCGACTGCTCGGTTCGGCACCGCTGTCCGTACTT

CTCCGACCGCGCCATCGCCTCGAACCGCCAAATCGCGGCGATGACGCTCG

CGTACTTCATGCAGACCGCCGGCTCCGACGTGTTCCGAAAGCGCGACGTG

GTCGTCGTCGACGAGGCCCACGGACTGGCCGAGTGGGCCGAGATGTACGC

GACTATCGACCTGAAACCCCGGACCGTCCCGGTGTGGGACGACATCGGCG

TCCCCGACGTGGCGGCCGCGGGCGACCCCGTCGAGCGCGCCTCGCGCTTC

GCGGAGACGCTCGTCGGCGTCTGCAAGCGTGAGAAAGACGAGCTGCTGAC

GAAGTCCGAACTGATGCCCGAGGAGGCCGCCCGCCGCGACCGCTTACAGG

AGCTCATCGGCGAGCTGAACTGGTTCGTGGAGGACTACCGCGACCCCCAG

AGCCCGACGACGTGGGTCGTCGACCAGCACGACGGCGAGGGGTCGCCCAT

CGCCATCAAGCCGCTCGACCCCGCGAAGTACCTCAAACACACCGTCTGGG

ACCGCGGAAACAAGTTCGCGCTCCTGTCTGCGACCATCCTCAACAAGGCG

GCGTTCTGCCGCTCGGTCGGCCTCGACCCCTCGAAGGTCGCGCTCGTGGA

CGTCGAACACACCTTCCCCGTCGAGAACCGGCCGCTGTACGACGTGACGC

AGGGGAAGATGACCTACGAGCACCGCGACGAGACGCTGCCCAAAATCGCC

CGCCTCGTCGTTCGCCTGATGGCGAAACATCCCGACGAGAAGGGGTTGAT

TCACTGCCACTCGTATGCGATTCAGGCGGAGCTTCGCCGCCGACTCGCCG

AGATGGGCCTCGGCAACCGCGTCCGCGGCCACGACCGCGACGACCGAAAC

GCGGAACTGGAGACGTGGAAGGCGACCGACCGCCCGGAGGTGTTCCTCTC

GGTGAAGATGGAGGAGGCGCTGGACCTGAACGGCGACCTCTGTCGCTGGC

AGGTGCTCTGCAAAGCGCCGTATCTCAACACGAACGACTCCAGAGTCGCC

CGCCGCCTCGAAGACGGCCAGTGGGCGTGGTACCGCCGCGCCGCCCTCCG

AACCGTGATTCAGGCCTGCGGCCGGGTCGTGCGCGCGCCCGACGACTACG

GCGACACCTACGTCGCCGACAGCAGTCTGCTGCAACTGTTCGACAAGACG

CGGACCGACATGCCCGACTGGTTCGCGGCGCAGGTCGACCGGATGACGAA

ACCCGACCTCCCCGAGTTCGACCCCGTCGTGGCGGGCGGCCGCGGGAGCA

ACGCGGGCAACGCAACCGGCGGGACCGCGAATCTCGGCGGTTCGACGGGT

CGGTCGCGAGGAGCGTCGGGTTCCGGCTCCGACTCGCGGCGCGACTCGAC

TCGCGGTTCGAGGTCCGCTGGGAGTCGCGCGGGCGGCTCCGGCGGTACGT

CGTCCGGCGGTTCCGACGGCCGCGAGACCGGTGGCTCGGACGGGAGCGCT

GACGACAGCGGCCGGAGCAGTCACCCGCTTTCCGACGTGTGGGGTGAGTG

ATTACAGAATGACACTGGCCGCGGAGAACAGCGTCGACCCGATCATGAGC

GCGACGAGCGTCAACGCGAGGAACTGCTGTCTATCCATGGGTGTGGGTCG

GTCGGCCGGTAATTAACGTTTCTGAACCGGTTCCCACGAGTGATAACCCC

GCCCGACAGTCGGTGCCATCCTCACGATGTCATGCCATCACATCTCGTTT

GTCCGGCAAAAATCGCCATAGTGTCCAGGAGAGTCGCCGCCTCCGCCACG

TTTCCTCAATGATTGTTAATGCGTTTAACCGTTTAGAGAGCGTAGATGGA

GAATAGCGCAACGTTTGTAAATCTATATTTCTACTAACTTTGTCTTTGAT

ATGCTTTCTCACGAAATAGTTAACACCAATGCCCCTGAATGGTGGAGTAT

CGGTCATGCACACCAACGCACTCGCCACGGCGATGACACTGTATCGTAGT

GGGGCACTCACACTCTCGCAGGCGGCCGCTCGCTCCGGCTACTCCGAAGA

CGACCTGCTCCTCGCCCTCCAGCGCCACGGTGTGCCCGTGCACGAAGACG

ACGCTCCGGCCGCATCCCTCTCGGCTGACCGGCCGGCCAGCGCCGACTGA

TTCTGCTTCGTCGTTCGATTATCGCCCGTCTCTTAGCGACCGCTCTCTGG

GCGATCTCGCTGGCTCGGACCGCGAGTCGTACAAACCGAAAACGACGATC

TCGTCGTGTTCTGCCTCTCTCCTCGCCGCTTCTCGCTTACTCCTCGTCGT

CGTCGACGACTTCCTCGGGGAAGATGTGCAGCCCCGACGGACTCTTGAGG

ACCGGCACCATCGACGCGTCGGGGTCGAAGTAGTCCGGCCGCGGGATGGT

CTTCGCCTCGTACGTCTCGGGGTCGAGCACCTGTACGGCGTGTTCGTCCT

CGACGGTGACGACGGTGGTCTCCGCCGCGTCCTCGATGGAGCCGAGGTCT

TCGGCCTCGGGTCGTTCGCCCTCCTCGAACTCCGATTCGTAGAGGTCGCC

GGAAGTGAGCCGCGTTCCTTTGAGTCGGCCGCGGTTGCTCCGCACGAGGA

CCGGCCCGTCGCCGTCCTCGGGGTCGATGACGTCGCCGGCGGTGTACCGC

GGCAGGCGGGCGACGAACGTGACGCGGTAGACCTCGTTGCCGTCGCCGTC

CTCCGTGACGAGCGTCGGGTGCTCCTCGATGGTGCCGCCGAAGCGCTCGC

GAATCTGCTTCGCGACGCCGCTTCCCATCTGGTTGGTCGATATCTTGATG

TTCGGGCCGTCGGGCGTCCGGTTCACCTCCGTGATGAAGGCGTTGCGGTC

GCCTTTGTCCTCGCGTTCCGCGATGTAGGACTCGGCGATTTCGATGGCCG

TCTCGGCCTCCTCGGTCGTCGGCGTCCGGTCGTCGGCGCGGACCTGCACG

AGGCTGGCGAAGTAGCCGCCGGCGATGCGGCCGCAGCGCTGGCACGTCTC

GCGGGCGATGCTCACCGGGACGACGATGGACTCCTCCAGCGGCGTGCCGC

GGACGACGCCCGAGAAGTGGCAGTGCATCCGGATGTTGTTCTCGTCGACC

TGCTCGGGTTCGACGCCCCAGCGGACCTCCTCGGCGTTGAGGTGGACGCC

CAGGGAGTTCGATACCTCCTCGATGGCGATGTCGGTGTAGTCGCGCGCGC

CGACGTCCACCCACCGGTTGCCCCGGTGAATCGCACCGCACTGCGCGCAG

ACGCGGACCTGCACCTCGTCGGGCGCGTCCACGAGGTCGAAGTCCTCGAA

GTAACACGAGTCGCAGAGCACGTCGTCGCGCTCGCGCGGCATCCCGGGGA

GCGGTTCCGGTCGCTCCGGAACCGGGTCGCCGCACCGAGGGCAGAAGTCG

CGGGATTCGCTCATTGACGGCTCTAGTCCGTTCGACCGCTTAAGTTCGGC

GTCACGGCGCGCCGGTGAGCCTCGGCCGTCAGCAACTCGCCTCAGTGCTC

GCCCGCCCCGTCGCGGAGGTACGTCGCGGCCTCCTCGTCGGAGACCTGCG

CGAAGTCGCGGTAGAACGCGCCGACGGCCCCGAACGCCTCCGGCGACTCG

ACCGCGACGACCGCGTCGCACTCGGCTTCGAGTTCTGAGAGGGTGTTCGG

CGGGCCGACCGGGACCGCGAGAACGACGCGCTCCGCGCCGCCGGCGACGA

CCTGCCGCAGGCACGCCCGGGCGGTCGCGCCCGTGGCGACGCCGTCGTCG

ACGACGACCACGCGCTTTTCGGCCACGTCGGGAAGCGGGTCGCCGCCGCG

GTACAGCGACACCTTGTCTCTGGCCGCCGCGGCCTCGCGCTCGCGCTCGC

GGTCGAGGTAGTCCTCGCCCACGTCGAGGTACGAAAGCAGGTCCTCGTTC

CACCACGCGCTCCCGTCGCCGGCGACCGCCCCCACGGCGAGTTCGGGGTT

GTCCGGCGCGCCGACCTTGGAGGCGACCACCACGTCGAGCGGCGCTCTGA

GGCGGTCTGCGACCTCCCTGCCGACGGGGAGGCCGCCCCGCGGGATGGCC

AACACGAGGTCGGCCGTCTCATCTCGCTCGTCGAGCAGGTCCGCCAGCCG

GCGGCCCGCGTCTTCCCTATCGGCGAACATGTCACCCGGAGCTACGCGCT

CCCGCGGCAAAACGGTATATTCCGATTCTTCACGACTGGGGCGGTCGCGT

GCGAGGGCTTCGCACTCCAACAGCGTTTATTAGCCGGATTACTAATCACG

TGGTATGAACTGGAAACCGGACTGGGGACTTCGCGGGCGGATGGCGCTCA

CGATGTTCCTCCTGTTCGCCCTCTACATCGTCTTTGCAGGCCTGTTGGCA

TGGTACTTCGACGGCCTGTTCATCATGGCCGGGTTCATGGGCGTCTTCCT

CTTCGCGCAGTTCTTCTTCAGCGACAAAATCGCGCTGTACAGCATGGGCG

CGAGCGTCGTCGACGAGGACGACGGCCCGCAGGCGCGGAAGCTCCACGCG

ATGGTCGGCCGGCTCTCTCAGCAGGCGGACCTCCCGAAACCGAAGGTCGC

CATCGCCGACACGCGCGTCCCGAACGCCTTCGCCACGGGGCGCTCCCAGA

AGAGTTCGGCCGTCTGCGTCACGACCGGCCTGATGGAGACCCTCGACGAC

GACGAACTGGAGGGCGTCATCGCCCACGAGCTCGCGCACGTGAAGAACCG

CGACGTGATGGTCATGACCATCGCGTCGTTCCTCTCCAGTATCGCCTTCC

TCATCGTCCGCTGGGGCTGGCTCTTCGGCGGCGACGACAACCGCCAGAAC

GCGCCGGTCATCGTCGCCATCATCGCGTCGCTCGTCGTCTGGATTATCTC

GTACCTCCTGATTCGGGCGCTCTCGCGCTACCGCGAGTACGCCGCCGACC

GCGGGGCCGCCGTCATCACCGGCCGCCCCTCGGCGCTGGCGTCCGCGCTC

CTCAAGATTTCGGGCCGGATGGACAACGTCCCGAAGCGCGACATGCGCGA

CACCTCGGAGATGAACGCGTTCTTCATCATCCCCATCAAGTCCGACTTCA

TCGGTCGCCTGTTCAGTACCCACCCCTCCACCGAGAACCGCGTCGAGCGC

CTCCGCGACATGGAGCGCGAGATGGAGACCGCCTGAGTTCCGATGGGGCT

GTTCGATTCATTCCGCGCCATGCTCGGCATCAGCGCCGAGTCCGACGCGA

CCACGAAAGCGGACCCCGAGGACCTCTTCGGGATGAGCACGGCGTACCTC

ACGATGGAGGCCGACCTCCGGTTCGACTCGGCCGACGAGGCGGCGCTGTG

TTTCTCCTCCGTCGACAGCACCGACTTCGCCGACACCGTCGACGCCGTCG

AAGACATCCTCACCGCCGGGAGCGAAGAGACCGGCACCGAGTTCAGCCGC

CACACCGACGGCCACGGCTACAACTGGGTCGTCCTCGCCGACGACGACCC

CGAGGACCTCGTGACGAGCGTCCACTTCGCGGCCGACGAGTTCATCGAGC

GCGGCTACGGGTCGCGCCTGCTCGCCGCCCTGTTCGGCTTCGAGCGCGAC

GGCGACCGCGCCTACTGGATTTACTCGTTCCGCCGCGGGGCGTACTACCC

CTTCGCCCCGCAGGGCACCTCGACGCGGAATCAGAGTCTGGAGTTCAAAC

TCGAATCCGTCCTCGACGGCGAACTGGAAATCGAGGGCGACGAGAGCTAC

TGGTACCCGCTGTGGCCGTCGACGCCGAACGGCCATCCGTGGGACTAACC

GCGCTCGCCCGTCGTGCCTGACGCTCTCGTCGTCAGCCCCCGACCCCTTT

CCTTTCTGTTCGATTCTACTTCCGCGCCAGCCCGCGCTCTCAGCCGAGTT

CGGAGCCGACCCAGTCTCTCCCGTCCGCCCCCGTCCTCGCGATATCGCCC

TTGAATCTCCGCACCGGCCCGATTGCCGACCCCACCACGTCTCCCGTTTT

CACTTTCATCTTGCTGTTAACGAGGGTTTATGAGGGTATAGGCGGAACGA

CTGGGTATGGCAGAAGACGACCTCGAAAGTCTCCCCGGCGTCGGCCCGGC

GACGGCCGACAAACTCGTCGAGAGCGGTTACGACAGCTACCAGTCCATCG

CGGTCGCCAGCCCCGGCGAACTGTCGAACAAGGCCGACATCGGCTCCAGC

ACGGCCTCCGACATCATCAACGCGGCCCGCGACGCGGCCGACGTGGGCGG

CTTCGAGACCGGTTCCATGGTTCTCGAACGACGCCAACAGATCGGCAAGC

TGAGTTGGCAAATCGACGAAGTGGACGAACTCCTCGGCGGCGGTCTCGAA

ACGCAGTCCATCACCGAGGTGTACGGCGAGTTCGGTGCCGGGAAGTCCCA

GATAACCCACCAGCTCGCGGTCAACGTCCAGCTTCCGCCCGAACAGGGCG

GCCTCGGCGGCGGCTGTATCTTCATCGACTCCGAGGACACGTTCCGCCCC

GAGCGTATCGACGACATGGTCCGCGGGCTCGAAGACGAGGCGCTCGAAGC

GACGCTCGACGACCGCGAGATGGAGGGCTCCATCGACGACGAGGAGACCA

TAAAGGCGCTCGTCGACGACTTCCTCGACAAGATTCACGTCGCGAAGGCG

TTCAACTCCAACCACCAGATTCTTCTCGCCGAGAAGGCCAAGGAGCTCGC

CGGCGAGCACGAGGACACCGAGTGGCCGGTCCGCCTCCTCTGTGTTGACT

CGCTCACAGCTCACTTCCGCGCCGAGTACGTCGGCCGCGGCGAACTCGCA

GAGCGCCAGCAGAAGCTCAACAAGCACCTCCACGACCTGATGCGCATCGG

TGACCTGTTCAACACGGGCATCCTCGTGACGAATCAGGTCTCGTCGAACC

CCGACTCCTACTTCGGCGACCCGACCCAGCCCATCGGTGGCAACATCCTC

GGCCACACCTCGACGTTCCGCATCTACCTCCGCAAGTCCAAGGGCGACAA

GCGTATCGTCCGCCTCGTGGACGCGCCGAACCTCGCCGACGGCGAGGCCA

TCATGCGCGTACAGGACGCCGGTCTCAAGCCCGAGTAAGCCGTCGCTCGG

TCGGCCGCTCAATCACTCGCTCGGCCGGCCGACTTCACACGACTCCGCGG

TCCCCGTTCCCCCCACTCCCTCCGTCTCACTCCACTCCACTCCACTCCAC

TCCACCCTCCCCACCCCACCACATTCTCCCCTTCGTCTCGTCCCCCGAGC

GGCCGCACCGCCGCTTGGTTCTCATAATGTTGGAAATGGCTTGCAATACT

ATCTCGTCGGCGGCCTACCGTTTAGGTAGGTGAACTCCATGACGCACCGA

ATCGTAGTCGTTGGCGGTGGCACGGGTGGGACGGTCGTTTCGAACCGTCT

CGCCGAGGAACTCGAATCGGAGATAGACGACGGCGACGTGGGGGTAACGC

TGGTCTCCGACGATACGAAACACGTCTACAAGCCGGTGTTCTTGTACGTC

CCGTTCGGAGTCGCGGAGCCGGACGACGGCGTCCGCGACCTGCGGGACCT

CGTCGACGAGCGGGTGGACATCGTCACCAACCGCGTTCGCCGGGTCGACA

CCGACGAAAAGTGCCTCTACTGTCAGGACGGCGACGAGGAACTCGACTAC

GACACGCTCGTCCTCGCGACCGGCGCGAAGCTCGACCCCGACGCGGTTCC

CGGCTTCCGGGAGGGCGCACACCACTTCTACAACGCCGATGGGGCCGAAC

GGCTCCGCGACGCGCTCGCGGAGTTCGAGGGCGGTCGCCTCGTGTTGAGC

GTCGTCGGGACGCCCCACATGTGCCCGGCCGCGCCGCTCGAATTCACGTT

CATCGTGGACGACTGGCTCCGGAGCCGCGGGCTGCGCGAGGACACGGACC

TCGTCTACACCTACCCGATGGAGCGGAGCCACGGGAAGCCCGAGGTCGCG

GAGTGGGCCGACCCGATTCTGGCCGCACGGGGCGTCCGCGTCGAGACCGA

CTTCGTCGTCGACGCCGTCGACCCGGACGAACGGGTCGTCTCGGCGGAAA

GCGGCGCGGAGGTGGACTACGACCTGTTGGTCGGCATCCCGCCGCACCGC

GGTGACGAACTCATCACTGACTCCGGCCTCGGTGACGCCGGCTGGGTCGA

CGTGGACCAGCGGACCCTCCACGCGACCGCCGCCGAGGACGTGTACGCCA

TCGGTGACACGGCGGCGCTGCCCATCCCGAAAGCCGGGAGCGCGGCGCAC

TTCCAGGCCTTTGCGGTCGCGGAGCGCATCGCCGCGGAGGTCCGGGGTCG

CACGCCGACGAAGCGCTACGACGGCAAGACCGTCTGCTTCGTCGAGACCG

GCCTCGACGAGGCGTCGTTCGTCTCGTTCGACTACGAGACGCCCGCGACG

ATGCGCCAGCCGTCGAAGCCCGTCCACTGGGCGAAACACGCGTACAACGA

ATCGTACTGGCTGACCGCCAGGGGGATGCTCTGAGGTGAGACCAATGCAG

GACCACGAACCAACCACGACGACAGAACAGCAGGTGCCCGAGGAACTCGT

ACGGGCCATCGAGAACAACCCCGAGGAGGTCGCGCTCCTCGTCGAGCGGA

TGGGTCTCGTCAACGACCTCATCGACGTGCTCGAACTCGGCGTCGGCGCG

CTCGACGACGAGATGGTACGGTCGCTCGCGCGAACCGGCACCTCGCTCGC

GGAAGTCGCAGACGACGCTTCCGACCCCGACACTGTCGCCGGGATGAAGC

GCCTGCTCCGCGCCGTCGGCGACGCGGAGGAGGCGGAGGCGACGCCCGTG

GGCGCGGTCGGCCTCCTGCGGGCCACTCGCGACCCCGAGGTCAAAGCGGG

GCTCGGCTACCTCGTCGCGCTGGCGGCGGCGCTCGGCGCGGGGACGGACG

AAGAATAACTCGAATCGCGTTCGGTTCGCCGTGCCGCTGCCGGCCGAAGC

CGGCTCAGTCGTCGCCGTCTTCGGTCTTCGTTTTCTCGATTTCTATCTCG

CCGTCGTGAATCTTCGCGCCGTCCTGTGCGACCTGTCGCGCGAGGACGGC

GCACTTGATTCGCATCGGCGAGATGTCGACGCCGAGCATCTCGGTGATGT

CGTCGGTGTCCATCGCTTCCAGCTCCGAGAGCGTCGTGCCCGGCAGTCGC

TGGGTGAGCATGCTCGCGGACGCCTGACTGATGGCGCAGCCATCGCCGGA

GAACGCGACGCGCTCTATGGTCTCGCCGTCGTCGGCGAGCTGCACGTCCA

TCGTAATCGTATCGCCGCAGGAGGGGTTCTCCCCGGTGTGGGTGAACGTC

GGCGACTCCAGCCGACCGTGGTTCCGGGGGTTCTTGTAGTGGTCCATTAT

CTGCTGACGATACATGTCTGAGCCAATGCCCATTGGTGTTCGTCGGGAGT

ACGCCCGAGCGCCGTAAAAGGATTCCGGGGCCGGCGATTCGGCCGAATTC

GTCGCTTACGTCGCGTCGGCCGTTGACGCGTCTTCGGCCGCCGGCGCGTC

GTACTTCGTCCACTTCTTTTCGACGTCGCGGTTGGCCACGACGACGGTGT

CGCCCTCGTCGCTCTCGAAGCGCGTCTTTCGGAGTTCGATGGAGCTGACC

GTCCCCGTCACCGGGTCCGATTTCACGCGGTCGCCGGGGTTGAAATCGGG

GTCTCGAAGCAGGTAGACGCCGGCCACCGTGTCGGCTATCATGTTCGACA

GCGCGTAGGAGACGCCCAGCGCGATGAAGCCGGTCGCGGTACCGAGGCTC

GCCGCGATTTCCGTCATCCCGACGATGTTCAACAGCGCGAGCGCCGCGCC

GAACCAGAGGAACACGCCCGCGACGGCGACCCCGAGTTCGACCACGAGGT

CCTGCTCTTCGGGGTACAGCCCGTCGAGCACGCCGCGGACGACGAACAGT

ATCGCCTTGATGCCGACGTAGGCGAGCGCGAGGAAGACGACCGCGGTGAG

CACCTTCGGCGCGGCGTCCTGCACCGCGGTCCCGAACTCCGCGATGGTTT

CGCGGAGCGTCTCGACGAGAAAGCCGGTCAGCGATTGGAGCATACGCTCC

GACGACGTGTCAGCGGGACAAATGTATTGGGACGGTCGCGCTCCGACACC

GCGAAAAACCGGGTACTCAGGCGAACAGTTCGCGAGCGGCGTCGATAGCG

TCGACCAGTTTGTCGACTTCCTCTCTCGCGTTGTAGATGTAGAACGACGC

CCGCGTCGAGGCGGCGACGCCGAGTTTGTCGTGGAGCGGCTGGGTACAGT

GGTCGCCGGCGCGGATGGCGACGCCCTGTTCGTTGAGGATGCTGGAGAGG

TCGTGGGCGTGGACGCTGTCGAGGTTGAACGAGACGAGGCCGCCGCGGTC

GTCGCCCGGCGGGCCGTAAATCTCGATGTCGTCGAACTCGGTGAGGCGGT

CGTAGGCGTACTCCGCGAGCAGTTCCTCGTGGGCTTGGACGTTCTCCATG

CCGATGTCGTCGAGGTAGTCCACGGCCGCGGCGAAGCCGACGCCCTGCGC

GATGGGCGGCGTGCCCGCCTCGAACTTCCACGGGAGGTCCTCCCACGTGG

AGTCCTCGTAGGTGACGCTCCGAATCATCTCGCCGCCGTAGAGGTACGGC

CCCATCTCGTCGAGGATGTCGCGCTTGCCGTAGAGCGCGCCGATGCCGGT

CGGGCCGCACATCTTGTGGCCCGAGAAGGCGAAGAAGTCGGCGTCGATGT

CCTCCACGTCGACCGGGCGGGTCGGGACCGACTGCGCACCGTCGACGAAG

ATGTACGAACCGACCTCGTGAGCCATGTCCGCGAGTTCGGAAACCGGGTT

GACGGTTCCGAGGGTGTTCGAGACGTGGACGACCGAGACCATCTTCGTGG

AGTCGTCGATGAGTTCCTTCGCGTGCTCCATGTCGAGGCGGCCGTCGTCG

TCGACGCGGATGTAGCGCACCTCGGCACCGGTCTTCTTGGCTATCTGCTG

CCACGTGACCAGCGAGGCGTGGTGTTCCATCTCGGTCATGACGACCGAGT

CGCCCGGCCCGAGTTCGTCGAGGCCCCACGCGTAGGCGACGAGGTTCATC

GACTCGGTGGTGTTCTTCGTGAAGACGACCTCCTCGCGGCCGCCGGACGC

GCCGATGAACTCCGCGACGCGGTCGTGGGCGTCCTCGTAGGCGACCGAGG

CCTCCTGGCTCAGATGGTGGATGCCGCGGTGGACGTTCGAGTTGTAGCCG

TGGTAGTAGTCGACGATGGCGTCGACGACCTGTTCCGGCGTGTGGCTCGT

CGCGGCGTTGTCGAGGTAGACCAGCGGCGTGGTGTCGTCGTCGTGTTCAC

CCGGCGTCGAGATGTCGCCGCCGACCTTCCGCTCGAGGATGGGGAAATCC

GCGCGGATAGCGTCGACGTCGACGGGGTACGACTCCTGCACTCTCATTGG

GTGTGAGTTACCGCCGTAGGCACAACACGTCTTCGGTCCGCACGCGGCTC

TGCAACCGATTTCGGTTTCGTCAGCCGGCAACTGTGGCCGGTGGCGGTGA

CGCAATCGCGATGCGGTGGCGACGTGTCGCTCACTCGCCCAAACGAATCA

AGTAGCTCACGCCCGTTCATCCACCAATGTCGAACGCCACCTCGTCCTCG

CTCGCGTTTTCGAGCCTCGACGCGCTTCCGACGCAACTCGCGATTCTGGA

CGAGGACGGCGTCATCCTCTACACCAACCGCGCGTGGCGGGAGTTCGGAA

CCGAACACGACTATCAGGGCGACAGCAGTTCGGTCGGCACGAACTACCTC

GGGGTCTGCGACCTCAGCGCCGACGCCGACGCGACGACGGCCAGCGAGGG

CATCCGCGCCGTCATCGACGACGACAGAGACGAGTTTTCCTTCGAATACC

CCTGCGAGACGCCCGAAGAGCGCCTCTGGTTCACCATGCGGGCGACTCGA

TTCACCGACGACGGCGAGACGTACGTTCAAATCGCACACCTCGACATCAC

CGACCGAAAGCGCGCGGAACTGGAGGCCGAAGAGAAGGCCGAGCGCCTCC

GGAATCTCGCGCGGATGCTCTCGCACGACCTCAGAAACCCGCTTTCGGTC

GCCGTCGGCTACGTCGAATCGCTCCTCGACGAGACGGCCGCCTCCGACCG

ACTCGAACGCGTCGCGGGCGCGCTCGACCGGATGGACGACATCATCACCG

ACGCGCTGGTGTTGGCCCGTCACGACACGGTCGAAGGGCTGTCGACTGTC

GACCTCGAAACCCGCGCGGCGGCCGCGTGGGAACACGTCGAAACCGGCAC

GGCCACGCTCGTCGTCGCCGACTCCGTCGAGTTCCGGGCGGACTCGAACC

TGCTGGGACACGTGTTCGAGAACCTGTTTCGGAACTCGGCCGAGCACGGC

GCGAGCGACGACGACCTGACCGTGACGGTCGGCGTCCTCGATGGCGACGC

GGCCGAAGCGGGCTTCTACGTCGAGGACGACGGCGTCGGCATCCCCACGG

AGACGCGCGACGTGGTGTTCGAAGCCGGTTACTCCACGGGTGAGGAGGGG

ACGGGTCTCGGCCTGTCAATCGTCGCGCAGGCGGTCCGCACCCACGACTG

GGACGTCGCCGTCACCGAGGCCGAGGGCGGCGGCGCTCGCTTCGAGATAA

CGGGCGTCGAGCGCCCGGCGTAACGTACCGGCGCGGAAGCGCGGAGCACA

CCGATTTTCGACGGCGGCTCAGCGGAGCCAGAGCAGTTGCGCGTGGAGCG

TGCCGTCGACTTCGAGGACGGTTTCCTCGTCCACGAGGCCGGCGTCGATG

GCGACCGAGACGGACTCCTCGCCGACGATGTTGGCGACGGTCGCGCGGGT

GAGGCTGTCCACGACGGCGTCTTCGTCGGCGGTCTCGGCCTCGCCGCCGC

CGTAGAAGTCCTCCGTCACGTCCAGCGAGACGCCGTCGTCGGTGTAGGTC

TCGCCGATGCAGTCGGGGTCGCAGACCGAGACGAGGAGTCCCTCGGGGGT

CTCCCGCTCGCGGAGGAGCATCTAATACTCCTCGACGAGTTCCGCTTCGG

CCTGCTCGCGGAGGTCGTCGGCCTGCTGCTGGGCCTGTTCTGCTTCCTCG

TCGCGCCCGAGTTCTTCGAGCGCGCGCGCCTTCTCCTCGTGGACGCCCGG

AGTGCGCATCCCGAGGCGGATGGCGTTGTCGAAGGCGTTGACGGCGTCCT

CGTTGAGGCCGCGCTCGTGGAGGAAGAAGCCGCGGTTGTACCACGCCTGC

GGGAAGCGCGGGTCGAGTTCGACGGCGCGCTCCGCGTGGTCGAGCGCGTC

GGCCGTCTGGCCGGCCTCCCAGAGCGCGTACGCGAGGTTGGTGTGGGCCG

TCGCGGCGTGCTCGCTGTCGTCGTCGAGTTTGAGCGCCTCTCGGTAGGAG

CCGATGGCCTCGTCCCACTCTTCGAGTTGGCCGTGCGCCGCGCCCTTGTT

GACCCACGCCTCCTGGGCGATGTTGTCGTCGTCGCCGGCGTACCGAGCGA

CGCGTTCGAACGCCCCCGTGGCCTCCTCGAAGCGGTTTATCTGCATGTAC

GAGAGCGCCACGTCGAGCAGTTTCTCGACGTCGACGTCCTCGGGGTCGAT

GTTCCGCTGGTCGAGGATGTCGGTCAGGACGCGCGAGTCGACCGGGTCGA

CCTTCGTCGGGTCCACCGAGAGTTCGGGCGGGTCGAGCGAGAAGTCGGAG

TAGTCCTCGTCGAACCCCTGTCCCTCGGAGAAGCGGTGGGGTCGATTGCC

GTCGTCGGTCATATAGCCGCGCTTGGGCGTCACGGCGGTTAAACGCTGCG

TCAGCGGGACGACACCGACCGCTTTTTAGCCTCCCGCGGGCGAGCGTCTC

CGAATGTCCGCGCCCCCGACGACGGTCGTGTCGTTCGTCCGGCACGCCCA

CGTCCCGTCCGTCTCCGACGCCGAGCGCACCCCCGGCCTCTCGCGTCACG

GCCGCCGCGACGCCGCGCGCGTCACCGCCCGCCTCGCGGACCTCGCGGAC

GTGGTCGCCACCAGCCCCGCCGAGCGCGCCCGCGCGACCGTCGAGGGCGT

CGCCGACGCGGCGGACGCCCCGCTCATCGTCGACGACGATCTCAGAGAAC

GCGAACTCGCTGACTCACCCATCGAAGACTTCGACGAGGCCGTCGAACAT

CTCTGGGCGAACCCGAACGCGTCGCACCCCGGCGGCGAGTCCCACGCCGA

GGCGCAGGCCCGCGGCGTCGCCGCCGTGGACCGCCTCGTCGAGGCGTACC

CCGACCGCCACGTCGTCGTCGGCACCCACGCCACGCTCATGGCGCTCGTG

TTCAACGCCTACGACCCCCGCTACGGCCGCGAGTTCTGGACGGGGCTGAC

GACGCCCGACGTGTACGAGGTGACGTTCGTGGACAGCGAGGCGTTCAGCA

TCGCCCGGACGTGGACGCCCGAGGAGGCCGCCCGCGAGGGTGACGCCGAC

CGCGACCCGGCGGAACGTTAACGTCCGGTTCGGGTCAACCACCTGCCATG

CGTTGCTTTCTCGCCGTCGACCTCCCCGACTCGCTCGCGGCGGGCGTCGC

CGCGGTACAAGACCGGCTCTCGGACGCGGACGGGCTTCGGTTCACCGACC

CCGAGAGTGCGCACGTCACCCTGAAGTTCCTCGGCGAGGTGTCGCCGGAC

CGCGTCGGAGCGGTGGAGGATGCGGTCGAATCAGCCGTCGAAGCCGCGGG

CGTCGGCCCCTTCGACGCCTCGGTCGGCGGACTCGGCGTCTTCCCCTCGC

TGGACTACATTCGAGTCGTTTGGGTCGGCGTCGACGACGGCGCGGCCGAG

TTGACGCGGCTCCACGAGGCCATCGAGCGCGAGACGACGGCACTCGGCTT

CGACCCCGAAGACCACGAGTTCACGCCGCACGTCACGCTCGCCCGCATGG

ACGACGCCCGCGGGAAGGTCCTCGTGCGGCGCGTCGTCGGGGGCGAGTCA

CCGACGGTCGGGTCGTTTCGCGTGCGCGAGGTACGCCTGAAGAAGAGCGA

CCTCGGCCCCGACGGCCCCGAGTACGAGACGGTGGCGCGGGTCTCGCTGT

GAGGCGCGTCAGTCGTCGGTCGGTCGGCCGCCCGCGACGCCGACCCCCGC

GGAGAGCCGCGTTTCAGCCGATTCGGGCGTGCGGACGCCCCTCACACGCC

CGAAACTCGAAGGAACAAGATTTTATGCGGAGGATGGAAAGTACAGCCCA

CTATGGGTAAGAAGTCGAAGTCCAAGAAGAAGCGTCTGGCGAAGCTCGAA

CGCCAGAACAGTCGCGTCCCCGCGTGGGTCATGCTCAAGACGGACATGGA

AGTCACGCGAAACCCCAAGCGTCGCAACTGGCGGCGGAGCAACACGGACG

AGTAAGCAATGAGCGCAAACGACTTCGAGGAGCGCGTCGTCACCGTCCCG

CTCCGAGACGTGCAGGCGGTTCCGGCACACGAACGCGCGGGCCGCTCGAT

GACGCTCATCCGCGAGCACCTCGCGAAGCACTTCAAGGTCGACGCCGAGA

ACGTGCGTCTCGACACGCAGATCAACGAGGACATCTGGGCGCACGGTCGG

CAGAGCCCGCCGAGCAAGTTCCGCGTCCGCGCCGCGCGTTTCGACGAGGA

CGGCGAGTCGGTCGTCGAAGCGGAACCGGCCGAGTAAACGGTGCTTCGCG

CCTCCTTCGCCGGTTCGTCCTACATCGGCGTCTTCGCTCGCGCGACCGAC

GACGCGCTGTTGGTCCGCCCGGACGCCGACGACTCGCTCGCCGAGCAGAT

GGCCGAGGAACTCGACGTGCCGCTGGTCAAGACGACCGTCGGCGGGTCGG

GAACGGTCGGTGCGTTAGCGACGGGTAACGAGAACGGTCTTCTCGTGTCG

AGCCGCGCCACGTCCCGCGAGAAGGAGGCTCTCACCGACGCCGTGGACCT

CCCGGTCTACGAGCTTCCCGGTCGCATCAACGCCGCCGGCAACGTCGTGC

TTGCGAACGACTACGGCGCGTACGTCCACGCGGAGCTCTCCGACGAGGCC

GTCGCGGCCGTCGAGGAGGCGCTGGAGGTCCCCGTCGAACGCGGCGACCT

CGCCGACGTTCGAACCGTCGGGACGGCGGCCGTCGCCAACAACCGCGGCG

TCCTCTGTCACCCGAAGTCGCGCGAACCCGAACTCGAAGCGCTCGAAGCG

CTGCTCGACGTTCGCGCCGACATCGGCACCATCAACTACGGCGGACCGCT

CGTCGGCTCCGGCCTCGTCGCCAACGACGAGAGCTACGTCGTCGGCGAGG

ACACGACCGGCCCGGAGCTGGGTCGCATCGAAGACGCCCTCGATTACATC

GACTGAGGGCCTCGCGTCCCCCCGTTTCTCGCTTCAGTTTCGCTCCGAGT

AGGAAGACCTTTCCCGCTCGCCACCCGACCGACGTGCATGAGCCAGTTCA

TTATCACGGGCAGCTTCACGAGCCGTGGCGTCGTCCACGAGTTCACGAAG

ACTGTCGAAGCGCCGAACGAGAACGTCGCACAGGAACGCGCCTTTTCGCT

CATCGGGAGCGAGCACGGAATCAAGCGCACGAAGGTCGAGCTCAACGAGG

TGACCGCGGCATGATGGGCGGCGGTCAGCAGCAACTCCAGCAGCTCTCCC

AGGAGCTTCAGGCGCTCGACGAGGAAATCGAATCGCTCGAAGCCGAAGTC

TCCGACCTGAACGACGAGAAAGACGAGATCGACGAGGCGGTCGAGGCCAT

CGAGACGCTCGAAACGGGCTCGACGGTGCAGGTGCCCCTCGGCGGCGACG

CGTATCTCCGCGCCGAAGTCCAGGACATCGACGAGGTCGTCGTCTCCCTC

GGCGCGAACTACGCCGCCGAGCAGGAACAGAGCACGGCCATCGAGACCCT

GCGACGCAAGCAGGACGCGCTCGACGAGGAAATCGCGAGCGTTCGCGGCC

AGATCGAGGAGCTCGAAGAGGAGAGCGACGAGATCGAAGAGCAGGCGATG

CAGGCCCAACAGCAGATGCAGCAACAGCAGATGCAGCAGATGCAGCAGAT

GCAGGGCGAGGGCGACGACGGCGACGACGAGTAAGGTAACCCCTACCCCC

ACCTCCTACTATTCAGATGTTCGACGGACTGAAGAAGAAACTCAACCGCT

TCCGTAACGACGTTGAAGAGACCGCCGAAGAGAAGGCCGAGGCGGCCGCC

GACGAGGCCGAATCCGACGCCGACGCGGAAGCGGAATCAGCACCAGCAGA

CACGGACAACGCGGCGGTCGAACCCGAGGCGTCCGAACCCGCTGCTGCCG

ACCCGGACGCCGACGCCGTCGGCGACGCGGACGCCGGGTCCGAAGCCGAC

GCGGTCGACGCGGCCGACGCTCCCGCGGACGCCGAGTCGTCGTCTGCGGC

GGTCGAAGCCGATGCCGAATCCGAATCCGAATCCGCGGCGACCCCGGAGC

CGGATTCCGAGGTCGACGCCGGCGCGGACACGGGCGACGAGCCGAGCGGC

GAGCCGACGGCCGACGAGGTCGAACCGCGCGAGTCGCTCGCCTCCGACGC

CGCGAAGGCGGCGCTGACCGAGGAAGACGAAGACGATTCGTCCGGTCCGG

GGCGGCTCCGCCGCGCCGCCGCGTTCGCCACGGGGAAGGTCGTCATCGAG

GAGGAAGACCTCGAAGACCCCCTCTGGGAGCTCGAGATGGCGCTCCTCCA

GAGCGACGTCGAGATGCAGGTCGCAGAGGAGATTCTCGAGACCATCCGCG

AGAAGCTCATCGGAGAGACGCGAAAGCAGGTCGAGAGCACCGGCCAACTC

GTCTCCGAGGCGCTCCACGACGCGCTGTACGAGGTCATCAGCGTCGGGCA

GTTCGACTTCGATCAGCGCATCGCCGAGGCCGACAAGCCGGTCACGCTCA

TCTTCACCGGCATCAACGGCGTCGGGAAGACGACGACCATCGCCAAACTC

GCGAAGTACTTCGAGAAGCAGGGCTATTCGACGGTGCTCGCCAACGGCGA

CACCTACCGCGCCGGCGCGAACGAGCAGATTCGCGAGCACGCTGAGGCGC

TCGACAAGAAGCTCATCGCCCACGAGCAGGGCGGCGACCCGGCGGCGGTC

ATCTACGACGGCGTCGAGTACGCCGAGGCCCACGACATCGACATCGTCCT

CGGCGACACGGCCGGTCGGCTCCACACCTCGAACGACCTGATGGCGCAGT

TGGAGAAGATAGACCGCGTCGTCGGCCCCGACCTCACCCTCTTCGTGGAC

GAGGCGGTCGCCGGGCAGGACGCGGTCGAACGCGCCCGGCAGTTCAACGA

CGCGGCCGCCATCGACGGCGCGATTCTGACGAAGGCCGACGCCGACTCCA

ACGGCGGCGCGGCCATCTCCATCGCGTACGTGACGGGCAAGCCCATCCTG

TTCCTCGGCGTGGGACAGGGCTACGACCACATCGAGAAGTTCGACCCCGA

GCAGATGGTCGAACGCCTGCTCGGTGAAGACGAGTAATCGGACTCGGTCC

GTCGTCCGTTTCTTTCGGTCGCCTCACGCTCACTCCAGTGCGAGCACGAC

TCCGAGCAGTAGCATCGTCCCCGCGCCGACGTAGTTCAGCGCGCTCGCGA

CCCGCGTCTCCGCGAGTCTCGACCCGAGTCGGTTCGCGCCGAGCGCGACG

ACCGAGAGGTACAGGGCCGTGAGGGCGGCGTAGGTCGCGCCCAGACCCGC

CATCCTGACGCCGGCTCCCGCGCCCTGTCCGGCGAAGCCGGGGAGGAACG

CGAGGAAGAACAGCGCGACTTTCGGGTTGAGCGCGTTGACGAGGACGCCG

CGCCGAAAACTCGCCCCGGCGTCGGTCTCGACCGAGGGGTCGAACTCGTC

GTTCCGGAGGGAGGTGACGCCGAGGTAGCCCAGATACGCCGCGCCGACGT

ACTTGAGCGCCGCGGCGGCCTCGGGGACGGTTCGAAAGAGCGCCGCCGCG

CCGACCGTCGCGAGGAGCGTGTGAAAGAGGACGCCCGTGGCGATGCCGAG

CGCCGACCGGACGCCCGGCCCGCGACCGTCGAGGCCGCGGGCGAGGACGT

ACATCGTGTCGGGACCGGGCGCGAGGATGAGCGCGACCGCCGCGCCGCAG

AACGCGAGGTAGGTCGCGGCGTCGAGTCCGAACGCGAGCGTCGTCATCGC

GCGAGCCTATGATTCGTCGCGGGAAGTAGCTCAGGGATTCGGACTCGGAA

CGACCTGATTGCGGAGCGTTGGCGTTTCGCCGTCCGACGCCGACGCCAGT

CGCCCGACGTTCTCGGCGAGGATGTCGGCGACCCGCGTCCAGTAGTGCGG

CGTGTGGCCGGAGACGTGCGGAGTGATGTAGACGTTCTCGAAGCCCCAAA

GCGGGTGGTCTTCGGGCAACGGCTCCGGGTCGGTCACGTCGAGCGCGGCG

GCGCGGAGTCGGTTGCTTCGGAGGTGCGAGACGAGGGCGTCGGTGTCGAC

GATACCGCCGCGGGCGACGTTGACGAGGATGGCGTGTTCGGGGAGCGCGC

TCAGCGCCCGCCGGTCGATGAGATCGCGCGTCTCGTCGGTCAGCGGGCAC

GCGACCACGAGGAAGTCGGTGCGGACGAGCGCCGACTCGAGGTCGTCGAA

CCCGAGTACCTCGTCGGTCGGACCGCCCTTTTCGGGCGTGTAGCGGACGC

CGACGGTCTCGACACCGAACGCGTCGAGTCGCTCGACGACCGCCTGTCCG

ATGGCACCGAGGCCGACCACGGTGGCCGTCGACCCCTGCAACTCGCCGTA

CGACTGGAAGCGCCGCCACTCGCGGCGTCGCTGGCGGCGGAGTCCTTCGT

CGAGTCGGCGGGTAATCATGAGGAGCCAGCCGATGACGTGCTCGGCGATG

TTGGGGCCGTGGACGCCGGAGGCGTTGGTGACGCGGACGCCGCGCTCGCG

GAGCGTTTCCAGCGGGAGATGGTCGTAGCCGGCGGCCGCCCCGGCGAACA

GTCGGAGTTCGTCGGCGGCGTCGAGCACCTCGTCGGGGAGCGACGTACTC

GTGATGATGGTCGCGTCCGTCGCGGCGTCGAGCGTTTCGGCGGTCGTCTT

CGGGTGGCGCACGGTCGCGTCGGGAAGGCGCTCGCGGAGGGCCGCGGCGT

ACTCCGACGCCGGGATGCCGTGCGTGGGGCGGTCGAGGACGAGAATCGTC

GTCATCGAGTGCGCTGTCGGGGGCCGAGCCGATAGGTGTTGGCCCCGAAC

GCGACGGATAAAGCCTTTACCGGCGCGCCGGCGTAGGAAGGCACAATGGT

ACTCGACAATCTCGGGAGTTCCCTCCGCGGCAGTCTCGATAAACTGCGCG

GGAAGTCCCGCCTCGACGAGGACGACGTTCAGGAGATCGTCAAGGAGATT

CAGCGCTCCCTGCTCTCCGCCGACGTCGACGTCAGCCTCGTCATGGACCT

CTCGGACTCTATCAAGACCCGCGCGCTGGAAGAGGAGCCGCCGGGCGGCA

CGAGCGCGCGCGACCACGTCCTGAAAATCGTCTACGAGGAACTCGTCGAC

CTCATCGGTGAGTCGACGGAGATTCCGCTCGAATCGCAGACCATCATGCT

CGCCGGCCTCCAGGGGTCGGGGAAGACGACCACCTCCGCCAAGATGGCGT

GGTGGTTCTCGAAGAAGGGGCTTCGCCCCGCGGTCATCCAGACCGACACC

TTCCGCCCCGGCGCGTACGACCAGGCCAAGCAGATGTGCGAGCGCGCCGA

GGTCGACTTCTACGGCGACCCCGACTGCGACGACCCGGTCCAAATCGCCC

GCGAGGGCCTCGAAGCGACCGAGGACGCCGACGTGCACATCGTCGACACG

GCCGGTCGCCACGCGCTCGAAGACGACCTCATCGACGAAATCGAGGAGAT

AGAGGGCGTCGTTCAGCCCGACCTGAACCTGCTCGTCCTCGACGCGGCAA

TCGGGCAAGGGGCGAAGGAACAGGCCCAGCAGTTCGACGAGTCCATCGGC

ATCGGCGGCGTCGTCATCACCAAACTCGACGGGACGGCGAAGGGTGGCGG

CGCGCTGACCGCCGTCAACGAGACCGACTCGTCTATCGCCTTCCTCGGCA

TGGGCGAGACGGTCCAGGACATCGAGCGCTTCGAGCCGAACGGCTTCATC

TCTCGGCTGCTCGGCATGGGCGACCTGAAACAGCTCTCCGAGCGCGTCGA

ACGCGCCATGTCCGAGACCCAGGCCGAAGACGAGGACTGGGATCCCGAGG

AGATGATGAAGGGGAACTTCACCCTCAAGGACATGCAAAAACAGATGGAG

GCGATGGACAAGATGGGGCCGCTCGACCAGGTGCTCGACATGATTCCGGG

CTTCGGCGGCGGCATCAAAGACCAGCTTCCGGACGACGCGATGGACGTGA

CCAAAGACCGGATGCGTTCGTTCGAGGTCATCATGGACTCGATGACTGAG

GAGGAACTGGAGAACCCCCGGAAGGTCGGCGCGTCCCGCGTCAGGCGCAT

CGCGCAGGGGTCGGGACAGGACGAGGAGACGATTCAGGAACTGCTCGAAC

AGCACCGCATGATGGAACAGACCATCAAGCAGTTCCAGAACATGGGCGAC

GGCGACATGCAGCGGATGATGAAGAAGCTCCAGAACCAGGGTGGCGGCGG

CGGTGGCGGCATGGGCGGTCTCGGCGGCATGGGGCCGTTCTGAACGCGCG

CGATTTCTTCGCCGCGGTTTCTGTGCCTGCCCGCGCGAGTTCCTCGCCTC

GCTGCGCCGCGATACCGCCGACTTTTAACGCTCGGGTCCGTCGCACCCCA

CAATGACCGTGCGAGACGTGGCGGTCGAGGCCTACAAGGAAGCCTTGCCC

GCGCTCGCGGCCAGCCTCGTCGGCGGCCTCGTCGCCGGCGTCGTCCTCGG

GGGGATGCGGGCCGAACTCCGGGCCGTCCCGGGGCTGCTCGTCCTCGTTC

CCGCGCTGTTGGCGACCCGCGGCAACGTCTACGGCTCGCTCGGCGCTCGC

ATCGCCACCGCGCTCCACCAGGGGCTCATCGAACCCCGCGTCACCGGCGG

CGACGAGCGCCTTCGGGCGGCCGCGACGGCCGCGCTCGCAAACGGCGTGC

TCACGAGCACGTTCGCCGCCGTCGTCGCGTACTTCCTCCTCACGTTCCTC

GGCAGCCGGGTCGCGCCGCTTCCGATTCTCGTCGGCGTCGCCATCGTCGC

CGGCCTGCTCTCCGGTATCGTCCTGACCGTCGTCGTCGTCAGCGTCGTCT

TCGCGGGCTACCGCCGCGGGCGCAACCCCGACACCATCGTCGGTCCGGTC

GTGACGACTACCGGCGACGTGTTCGGCGTCCTCTTTTTACTCATCGCCGT

CAGAACCGTCCTCGCCGTCGCGGGGGTGTTCTGAGTGCCGACCGAGTGGA

CCGTCCGCGCCATCACGCGGGCGATGCTCCCGGTGTTGCTCGTGCTCACG

CTCGTCGAACTCGGGAGCGGCCTCGTTCTCGGGAGCTTCGAGGCGCAACT

GTTCCGCTACCCGTCGCTGCTCGTGCTCGTCCCCGTCACCATCGGGACCG

CGGGGAACCTCGGGAGCGTCCTCGCGGCGCGACTGTCCACGTCGTTCCAC

CTCGGGACGCTCTCGTTTTCGCCCCGCGACGACGAACTCGCGGGCAACGC

CGTCGCCACGCTCGCCCTCGCGGTGACGGTCTTCCCAGTCATCGGCGCGG

GCGCGTGGGTCGCCACGCTCCTCGTCTCCGGCGACACGTCGCTGGCCCTC

CAGAAGGTCGTCCTCGTCGCCTTATCGAGCGGGATCTCGCTGGCCGTCCT

CGCGGTCGTCGTGACGTTTTCCGCGACGTACCTCGCCTATCGGTTCGGTC

TCGACCCCGACGACGTGGTCATCCCCGTCGTCACCAACCTCTGCGACGTG

CTGGGGGTCGTCGTCCTGTTCGGGGTGGCGCAGGTGCTCGTCTGACCCCG

CTGCCCCCGACGTTCGAACGCCTCCCGCCGACCGACGCCGATTAGTTCGC

TCGGCACCGACCGTCGGTCGTGCAGCCGTCGGCCGTCCTCCCGGTGCTGT

TCGAGGTGCTTCCGCGAGTCGCGCGCATCGCCGCCTACATCGCCGTGGGC

GTCTTCGCCGCGAACCTCGTCGTCGCCTTCGGCCTCGTCGAGCGCATCGC

CGGTCTCTCGCGGTATCTGACGAGCCCCGCGAACCTCCCCGACGAGGTGG

GGACGGCCATCGTCACCACCGCCGCCTCGACGACGGCGGGCTACGGGATG

CTCGCGGAGTTCCGCGAGTCGGGCGTCCTCGACGACCGGGCGACGCTCGT

CGCCGTCACCATCAACACGTTCTTCGGCTTCGTCCAGCACATCTTCACGT

TCTACTGGCCGGTGCTCATCCCCATCCTCGGCCGCGAGGTCGGCTTCATG

TACGTCGGCGCGCGGGCGGCCATCGCGCTCGCCATCACCGCGACCGGCGT

CGTCGCCGGCGCGGTGCTCCTCGCGGACCGGAACACGACCCCGGTCGCGG

TCACCGAGACCGACGGCTCGGGCGGCGCGGTGGATGCGGCCGATTCGGCC

GCCGACGCGGGCGACTCCGGCGACGCGGCCGAAAGCGACGACTCGCTCCG

CGAGACGGTCGACGACGCCGCCCGGAGCACCTGGAAGAAACTTCGGCGCA

TCGTCCCGCGGCTGGCGGTCGTTTACGTGGCCGTCACGCTCCTGCTTCGG

ACGACCGACCTCGAATCGTTCGCGGCGCTCGCCAGCCCGCTGACGAACCT

CGTCGGCCTGCCGGGCGCGGCCGTCCCCGTCGTCGTCGCCTTCGCGTTCG

ACACGACGACCGGCGCGGCGACCATCGCGCCCGCCATCGGCGAGACGTTT

ACGCCGAGACAGGCCGTGGCGACGATGCTCATCGGCGGCATCATCTCCTT

CGCCGTCTCGACGTTCAAGCGGTCGATTCCCTTCCAGTACGGCATCTGGG

GCCCCGAGTTCGGGTCCAAGGTCATCGCCGTCAACACGGGGCTGAAAATC

GTCTTCATCGCCGTCGCGGTGGCGCTGTTGGTCGCCTGAGTCGCAAGAAG

CGTCCCCGCCGCAGTCGGCCGAGTTAGCGGTCGTTCGGGTCGACCGGCCG

CCCCTCTTCGGTCGGCGGCGCGATGTAGTCGATGACCTCGTCGACGGAGG

GGTCGCGGACGGTCACGTGCACTTCGATGTCACCGAGTTCGTCGGGGTTA

CCGACGGAGAAGTTGACGACGCCCTTGAACGCGGCCTGCTTTTTCAGCGC

GAACGAGAAGCCGTCGTCGTCGCGGCGCTTTGCGAACTCGCGGCGGGCGG

TGTCGAGGATGGCCTGCTCGTGGAGGCGTTCGGAGAAGCGCTCCAGCTCG

TGGGCCTCGCCGACGAGCTGTCCGGGTTCGTGGTCGAGTTCGACGCCGGG

GAAGATGTTCTCGACGGCGTCGCCGACGCGGTCGGTGACCTCGGTGTCGC

GGACGGGCACCTCGATGCGGACGTCGACGCTGTAAATCATGCTCACGCGT

CGGCCTCCGCGAGCGCTTCCGGCCCCTCTTCGAACAGCTTCGTGATGGTT

TCGCGGTACTCAGCCAGCGTGCCGGTGTTCTGGATGACCACGTCGGCGCG

GTCCATCGCGTCGCCCATCCCGAAGTCGAGTTCGCGGCGTTCGCGCTTTT

TGAGCGCCTCTACGTCGGTGTCGCTGTCGTCGCGGTCGCGGTCGAGCAGG

CGCTCGGCGCGCGTCTCGAACGGCGCTTCGACGCTCACGAGGACGAAGTC

GTCGCCGAACGCCTCGCGGAAACGGTCGAGTTCGACGCCCGACCGAAGCC

CGTCGACGAGCACCACGTCGTTCGATTCGAGGTGCTGTTCGACCATCGGC

AGCGAGCGCGCCGCGATGGCGTCGTCGCCCTCCTCCTCGCGGAGCGCGGT

CGCTATCTTCCCGTGGTCCGTCGCGGGGTCGAGGCCGCGCTTGCGACACT

CCTCGCGGATGACGTCGCCCATCGTCACGACGGGTACGCCCGCGTTGCGG

GCCACCTCGGCGAGCTCGCCCTTGCCGCTTCCCGGCAGGCCGACCGTCCC

GATGACTCTCATAGCCGGTGTTACCGCGTTGGCGCTGTTAAAGCCTCCCT

TCACGTCGCTCCGGGTGGCGGGTTTCGCCGGTTCCCTTCACCCCGCTCCA

CCTCACCTCGACCACCCCGCTGCTTTTCGCCTCGGCCCGCGTACTACGCC

TATGTCTCAGCGCTCGTTCGTCGTCCGCGCCCTCTGGTTCGTCTTCGTCG

GCTGGTGGGCGACGCCCGCCGTCGTCAACGTCGCGTGGTTCCTGAACGCG

ACCGTCATCGGCATCCCGCTCGGCGTCGCGCTCATCAACCTCGTGCCGAC

GGTGCTCTCGTTGAAGGAGCCGAAAACCCGGCTCGACCCCGACTCGGGCC

GCGGCCAGCGGTCGGTCGTCGTCCGCGCCGTCTACTTCGTGTTCGTCGGC

TGGTGGCTGAGCTGGGTCTGGGCGAACGTGGCGGTGCTGTTCACGCTCAC

CATCGTCGGCCTCCCGGTCGGCATCTGGATGCTCAACCGGCTCCCCGCGG

TCACGTCGCTGTATCGGTTCGACGGGTAGAACCCGATTCGTTTTTCTTGC

AGGGACGGTTTGTGGGCATCGAGGGCGCGTAGCTCAGTCGGACAGAGCGT

CGGACTTCTAATCCGATGGTCGCGGGTTCGAATCCCGTCGCGCTCGCTCG

GCCGGAGGCCTCGCTCGCGCGACGGACATCGCAAACGCTTCGCGTTTGCT

CAATCCCGTCGCGCTCTTTTCGTTGCTGAAACCCCCGAGTGGTAGCGAGA

CGTGTGGGAACGCCACGCGCGGGATTCGAATCAGGGAGCAACTTGTTGCG

ACTGGGGTTCGAATCCCGCCGCGCTCGCTCGGCCGGAGGCCTCGCTCGCG

CGACGGACATCGCAAACGCTTCGCGTTTGCTCAATCCCGTCGCGCTCTTT

TCCTTGCGGTAGACCGCCGAGCGACGGCATCGGCGGCCCCACCTACTCCT

GTCCTGAGCGGGTTCTGTTCTCGACGACTATGGGGCTTGACCTCGGATAC

GTGTGACCTACAAGTTAGGATGACAACATTTTTCGGTCGAGCGGCGCGAA

ACGCCCCGCATGGACGTTCTCGGCATCGTCGCCGCGGCGGTCGTCTTCGG

CGTCGCCGCCTACAATCTCGTGACGAGCCTCGACGGCCGATTCGCGAGAC

TCCTGTTCGGGTGTCTGGGCGTCGTCGCCGCGACGCTCTACGTCATCGAA

CAGTTCGGACTCGCGGCGTCGCTCGGACTGAACGTCGCTCACGGCCTCCA

ATTCGTCGCGGCCCTGCTCGTGGTCTCGGCGCTGGTCGATTTCCTCGCCC

GGCGCGCCGAGGCGGGCGATTCCGGGTCGTCGTGAACGCCGCGACTCCCC

GTTCGGGGGACGGTTCGAACAGACGATAGACGACGCGGTCGTCCACGGCG

ACGCCGACATGGAGTCCGCGCTCCACGAGGGGCCGGTCCGGGTGCTCGTC

AACGGCTGGGTCGAGGTGCCGGGCGGCCGGTATCTCTCCCCGCACGCGGT

CCACCATATCGACGTGCGAGTTCCCGAGTGATGTCGAGTCTCAGACGACG

GTCAACTCCCGACCGCAGTCGGGGCACTCGCCGTCGACGATAGTCACCGG

CGCGTCGCAGTCCGGACAGAACTCGCGGTAACACGCCCTGTCTCCCATAC

GTACCGCTATCACGGGAACGGTGGTGAACGTTGGGGCGAACCGGAGCGCC

GAGTGTCGGCCGGCCCGTCACTCGTCATCGACGTCTTTTCCCCGACCGGC

GAAGTTCGCGCCGAGTTCGGCACCGTAGCGTCGGGCGTAGACGCCGGGGA

CCTCGAGGAGGTCGCTCGGCGCGCCGGATTCGACCACGACGCCGCCGTCG

ACGACGTGGACGCGGTCGGCGTCGCGGACGGCGGCCGCGCTATCGGTCGC

CACGACGAGCGGGGCGTCCGGTCGCCGCATCGCGGCGAGGGTGGCGCGAA

GCTGTGACGGGGCGGAACTGTGCGGCGCGCGGACGAGCGGGCCGGCTCCG

TGGAGGAGCGGTCCCGCGAGCGCCGCCGCCTCGGAGATTCGGGGCGCGAC

GACGGCCAGAAGCGGCGGCCGATTCGCGGGCGTGGTCGCGTCCGAGTCGG

CGTTGGTCGCCGGGCTCTCGGGTGGGGACATACGATGTGAGATTCGCTGG

CACGGGAGATAAGTATAGAGCGGACACTGGCGTTTCACGAATCACATCGG

ATGATTCATAGTGCTTTGTCAACTTCTGTCACGAATCAGTTGTCGATACG

CCCGCCGCCTCGGGGCGGTAGCCGACGGCGCGGCACGGGCGCTCGATTAA

CGGCGTCCAAACCGGCGCGTCCGCGGGCAAAGCAGCTGCTAACGGGTGTA

AAGCCGCAAAAAACGTCGTGAATTTGGCCGAGCGAGTCGAGTCGATTCGA

ATCAGGGTGTGGGTGTATGGTGGCCTACTGGGACGCTCACATTGCGCCGC

CCATGCCGCCCATACCGCCCATGCCACCCATGCCGCCGCCCATGCCGCCG

GGTGCGCCGCCGTCGTCGTCGTCGCTGCCGGTCTGGCCACCGGAGAGGTC

GCCAGCCGCGATGACGTCGTCGATGCGGAGAATCATGACTGCGGCTTCGG

TCGCGGACTCGATAGCCTGGGTCTTGACGCGGAGGGGCTCCACGACGCCC

TCTTCTTCCATGTCGATGACCTCGCCCGTGTAGGCGTCGAGGCCGGCTGC

GAACTCGCCGCCGTCGTGGCGCGAGCGCAGGTCGACGAGGGAGTCGATGG

GGTCGAGACCGGCGTTCTCGGCGAGGGTGCGCGGGATGATGTCCAGCGCC

TCGGCGAACGCCTCGACGGCGAGCTGCTCGCGGCCGCCGACGGAGTCAGC

GAACTCGCGGAGCTGCAGGGAGAGCTCCGTCTCGGGAGCGCCGCCGCCGG

GGAGGACCTTGCCGTCTTCGAGCGTCGTGCGGACGACGCCGAGGGAGTCC

TCGATGGCGCGTTCGAGCTCGTCGACGACGTGTTCCGTGCCACCGCGGAG

GATGAGCGTGACGGACTTGGCGTCCTCGACGTCCTCGACGAAGATGCGCT

CGTCGCCGCCGACGTCCTTCTGTCCGACGGAGCCGGCGAAGCCGAGGTCG

TCGGCCTCGATGTCGTCGAGACTGCTGACGACGCGGCCGCCCGTCGCGCG

GGCGAGACGCTTGAGGTCGGAGGACTTGGCGCGGCGGACCGCGAGGATGC

CCTCCTTGGCGAGGTAGTGCTGCGCCATGTCGTCGATGCCGTCACCGACG

AAGACGGCGTCAGCGCCGACCTCGACGAGTTGGTCGACCATCTCTTTCAG

CTGCTTTTCTTCCTGGTCGAGGAACTGCTGAAGCTGGTCGGGGTCCGTGA

CGTTGACTTCCGCGTCGATTTCGGTCTCGCGGACTTCCAGCGCGTCGTCG

AGGATGGCGATGTTCGCGTCCTCGACGGCGTAGGGCATGTTCTCGTCGAC

GCGTTCCTTGTCGACGATGACGCCCTCGACGAGTTCGGAGTTGTCGATGG

TGCCGCCGACGACCTTCTCGATGGAGACGTTGTTCGTGTCGATGCCGTCG

TCGTCCTTGACGGCCAGCACGGCGTCGACGACGAGTTCGGAGAGCAGGTC

CTTCGCGGACTCCGCGCCCTTACCGGTCATCGCCGTTGCGGCGATCTTCG

TGAGGGTCTCGCGGTCGTCCTCCGTGACCTCGATGGCGTTGTCCTCGAGG

ACTTCCTTGGCCTTCTCGGCGGCCTGGCGGTAGCCCTGCGCGATGGTCGT

CGCGTGGACGTCGGAGTCGAGGAGGTCCTCGGCCTGGTCGAGGAGTTCAC

CGGCGTTGATGACGGCCGTCGTCGTGCCGTCTCCGACCTCGTCCTCCTGG

GTCTCGGAGACTTCGACGATCATGTTGGCCGCGGGGTGGTCGATGTCCAT

CTCCTTGAGGATGGTGACGCCGTCGTTCGTGACGACGACCTGCCCGCCGG

AGTCGACGAGCATCTTGTCCATCCCTTTGGGGCCGAGCGTCGTGCGTACG

GCCTCTGCGACGGCCTTCCCGGCCGTGATGTTCATCGACTGCGCATCCTG

TCCGGATGTGCGCTGGGAGTCTTCGCCCAGAATGATCATGGGCTGACCCT

GCTGCATTCGCTGGCTCATAGTCATCGCCTGATTGTTTGTGATTCTATAA

AAAAGCTTCGTTTATTGGTATGCCAAAACGCAACACTGTGGGGTGGTCGA

GACCCGGAGACGGCGGTCGATTTCGGCGGTTTATCTGTGGCTTTCGCCGG

GCGGTCTCGCCAGTCCCGAAGCGGGTGCGGTGAAAAGACGATGCGGGCGA

GTGCCGTGTCGCGTCGGGGTGGACTCGTCGGCGGTGGCGTCTCAGTTGTT

CTGTCGGACGTTGAAGCCCTGGGTCAGTTCGTTGTGCTTCCGTTCGAGGA

AGGAGTACACCGCGCCGTGGGGGGCACCGTCGAGAATCATCTCGACGGCC

CGGCGGACGACCTCGACCTCTTCGGGCTGGCCGATGATGCCGAGCGTCGT

GCCGTAGATGACTACGTCGGCCCCCGTCAGCTCCTCCATGAGCTCGCGGG

TGCGGCCGTTCTCGCCGATGAGGCGGCCCTTCTGCCGCCGCATGTCGTTT

TTGTTTCGGGTGTGCCGGTCGATATCGATGAGTTCGAACATCATCATGTC

GTCGTCGAGGAGCGCCATCGCGTCCTCCGGGGCGAAGCCGCGGCCGACCG

CTCGAACCACGTCGGGAGCGACCATGCCGAGTACGGGGTCGCCGACGCTG

TCGATGGCGACGCTGCCGTTCTCCGAGTCCACGTCGAGTCGGACTTCCGC

GCGACTCTCTATCTCTCGGAGCGTCGAGCCACCTTCGCCGATGAGAACAC

CGATACGGTCCTGCGGCACCTTGACGTGCTGCATATCACCCGATACCCGT

CGGAGTGGTTTAAGCGTTCGCTCGGCGGGTCGCCGCGTGTCTCGGTCGCA

CACCCGTCTCGCGGCGGGTCTGGGTTGCTACTCGTCTCCGTCTCCGTCCT

CGCCCTCGTCGCCCGTGACGAACTCGTACAGCGAGTCGCCGTCGGCGTCG

GCCCCCTGCCGCCGGAAGAAGTTGGCCACGTTGCGGCAGTCCCGCCGGAG

GAACTCCTCGGCGTTGGGGTGGTGGACCGTGACGGCCTGTCCGAGGTCGA

TGACGACGAGTTCGCCGTCGTGGATGATGAGGTTGTACTCCGAGAGGTCG

CCGTGGACGAGGCCGGCGCGGTGGAGCCGACGCATGTACTCGCGGACGAC

TTCGTAGGCTGTCTGGGGGTTCTCGACGCGCACCTCCGAGAGCCGCCGCG

CCCGGTCGTCGACGACGCCGACGAGCTCCATCACGAGGACGTTCCGCTGG

ACCGCGATGGGCTTCGGCACGCGGACGCCGGCGCGCTGGGCGCGTTCGAG

GTTGGCGAACTCCTTTCTGACCCACGCGCGGACGACCTGTCCCTTGTCGT

GGCCGATGTTCTCGAAGCGCGGGTCGCCTTCGAGATAGTCGCGCATGTGC

CGGAAGTCGGAGGCGTTGATGCGGTATATCTTGACCGCCACGTCCGCGTC

GTCGCCGCCGAGCGCCTCGAAGACGTTGGCCTCCTTGCCCGTCGAAATCG

GCCCGCCGAACGCGTCGATGTGCCCGTCTTGGACGAGTTTGTAGATGGCG

GCGAACGTCGCGTCGTCGAACACCGACTGCTCGACTTTGAACTGGTCGGC

GTCTTTCAACCGCATCCGGAACTCGTCGAAGTCGCGGTCCTTGCGGCGCG

CGATGCGGTCCGCCTCGTCGTCGGAGACGTCTATCTCCTCCCACTCGTCG

CCGAACGCTTCGCCCTCCTGGGGTTCGACCATGCCGAACTCGTCAGTCAT

TGGACTAGGGCTACGAGAGGCGAGATGAAAAGGGCACAGGCTCCGGGTTC

GGCGTCTTCGGGGAACAAACAACAAACGGCGCGCCGTCTCGGACGTGCGC

GTTACTGGATGTGACCTTCGCGGCGGAGCTGGTCGGCTTCGGACTTCTCG

TAGCGCCAGCTCACGTCGGCCTTCTCGTCCTGCCAGTCCCACGGCTCGAC

GAGCACCACGTCGTCCTCTCGAATCCAGATTCGCTTCTGCATCCGTCCGG

GAATGCGAGCGGTCCGCTCGACGCCGTCGGCACAGCGGACGCGAATACGA

TTGGCCCCGAGCATGTTCGTCACGACGGCGAACACTTCGTCGTCGTTCGG

CATTCGAAGGTCTCGGCGGCTCTCGTTCTCGTCGTCGCTCATGCGCGAGC

GTTCGACGTGGGCATGGTTAAATCCGCCGACGTTCGGCCGGTAGGAAGCG

TCCGACGGAACGACGCGTTTAGGGCGGTCCCGAAACGCCGTCGTGGTATG

CTCGACAAACTCGGTACGAAGGGTATCGCCGGCGTCGTCTCGCTGCTCTT

GGGTATCGGTATCGTCGCCTCTCAGGCCCCCGTCGTCGCGGCGGGCCTCG

CGTTCGTCGTCGCCGGGCTCGGCCTCGTCGCCGGCGGCCTCGCCGAGGGC

GTCATGAAGATGTTCGGGATGGCCTGACGGGCCTTCGACTCCGGCCCTCG

GCTCACAGCGATTCGCGGAGGAACGAGAACACCCGTTTTCGCACCGCGGG

ATAGTGGTACGACGAGTGCAAGAAGACGTGGTCGGCCGCGTCGACGGCGT

CGTACTCGACCTGTGTTCCGGCCCCTTCGAGCGCGTCCGCCAACCGGGCG

CTCTGGTCCGGTGCGACGACCGAGTCCTCGCTCCCGTGGAGCAACAGCGT

CGGCGGCGCGTCGGCATCGGCGTCGGTGTCGTCATCCACGTAGGTAATCG

GCGAACACCGGCGCAACTCGGCGGCCGACGGCCGGTTCTCGAACAGCGGC

AGCGACTCGCCGGCGTGCTCGTGGTCGTCGGCTCGGAGGTCATAGACGCC

GGAGACCCCGACCGCGGCGTCGATATCTGTCCGGCGACCGAACTCGCCGT

CCGAAAGCGCCGCCAGTAACGCGAGGTGCGCCCCCGCGGAGTGACCGACG

ACGGCGACGGCGTCCGTGTCGATTCCGAACTCGTCGGCGCGGTCCGTCAG

CCAGTCGATGCCCTCGCACACGTCTTCGATTTGCGCGGGGAACGGCGCTT

CGTGCCCGAGTCGGTACGACACCTCGGCCGCGACGAACCCCCCGTCGGCG

GCGTCGAGCGCCCAGCGGGCGAACTGGCCGGTCGAACCGGTCTCCCACGC

GCCGCCGTAGACGTAGACGACGAGCGGCCGGGTCGGTTCGGCCGCCGGCG

ACGCTGTCGTTTCGGCTATCTCGGTCGCTGCGGGCGATTCGGGGCAGTAC

ACGTCCGCGGTCAGCGTGCGCTCCTCGCGGTCTGCGACGGTTCGGTTTCG

ATGAACGAGAACGTCGGAAGGGCGGGGCATACCGAGTCGTCGGGTCGGCA

GCTTCTATCGGTTTGGGAGACGGCGAGTAGCGGGTCGTCGAGAAGGGGTG

AACTACCGGCGGGGCGCGGCGATTACTCGGCTATCTTCGCGCGACCCGGC

TCGATGAGTTCGTCGAGGTAGTCCGCGAGCGCGCCCTTCGCGTCGGCCGG

GTGGAGTTCGCCGGATTCGAGATCGGCCTCCAGCGTCTCGTAGTCGTCGT

AGTCGAGGTTCCCGCCGTACTGTTCGGGGCGCTCGACGACGACCCGCTCG

AAGCGCGGGAAGACGTGGTACTCGAAAATCTGGAGGACGGGGTTCTCGCG

CTCCTCGCCCTCGTCGGTCGGCTCGGGGTCGGCCGTCGGCGGGCAGAACG

CGCCGTTGACCTTGTCTTCGATGTCCTCCGTGGAGTCCTCCATCGAGATG

GTGACGCCCTCGGAGGAGGACATCTTGCCGATGCCCGTCCCGAGGTCCGC

GATGAGCGGCGTGTGCATGCAGGTCGGCGACTCCTCGCCGATGCTCGGGA

GCGTGTCGCGGGCGAGCATGTGGACCTTGCGCTGTTCCATCCCGCCGATG

GCGAGGTCCACGTCGAGGTAGACGATGTCGAGCGCCTGCATGATGGGGTA

GACCGCCTGCGCGACGGTGACGCCGTCGCCGCTTTTGATTTCGGCCATGG

CGCGCTCGGCGCGCGAGAGCGTCGTCTCCAGTTCGACCGCGTGCAGGTCG

AGGACGTAGCTCTCGTCGAGCTGGTACTCCGAGCCGAGGACGAACTCCGT

CTGGCTCTCGTCGAGGCCGTAGGCGATGAACTGCGCTTTCATCCGCTCGG

CCGTCTCGCGAATCTCCTCGAACGTGCCCTTCCCGTTGAGGTAGGCGTGT

ACGTCCGCCAGCAGCACGACGACTTCGAAGCCCGCCGCTTGGAGGTCGAT

GAGCTTGTTGGCGGTGAGCATGTGACCGATGTGGAGGACGCCGGAGGGCT

CGTAGCCCACGTACGCTCGCTTGCCCTCGGGGTCGTCGGCCAGCGCGCGC

ACTTCGTCCTCCGTGACCACTTCGGAGGCGTTCCGAGTGATCAGGTCGTA

TGCGTCCATACACGTCCGGTGTCGGGGACGTGGCATATGACTTCTGGAAG

CCGCCCGCGGGCGAAACGGACAACACCCACCGGTCCCAACTCCGGGTGAT

GTCCGACTCAGGAACCACGACGCGTGCGCCCGTCATCATCTTCGCCGCGC

TCATCCTCGTCGTCTTCGGCCTGCTCGCGGTCATGTGGGCGTCGGTCCGC

GGCGGCCACCTCCTCCCGCACATCCTCGGCTTCGCCGTCTACTTTCTCGC

GTTCCACGTCTACCTGCCGTACCGAGTCCACAAGGACGCGACGCTCAAGG

GGCGAAACGCGACGTTCTGGGCCGTCATCGCCTTCTTACTGCCGCTCGTC

GGCGCGGCGCTGTACTTCGTGGTCGCCGTCGTCGGCCACGACGCGACCGA

CTAATCCCTCGCTTCGACGCTATCCGCGCCGGTTCTCGGCGCGGTGTTCG

GAGATAATCTGGTCGACCATCTCCTCGCGCTGTTCCTTCTGCCGTTCTTC

CGCCCGGTCTTCCCAGTCGGAAATTGCAGATTCGACTTCGGACTCCTTCG

CCACGGCGAGTTCGTCTATCTCCTGAATCGTCACGCCGCCGGCGGGTGCG

ACCGGCACGTCGGCCTCGAACAGCACCTCGTCTGCGGCCTCCGAGAGGTT

GCCCGACCGGAGGACGACCTTGGGGTCGAACTCGGCGAGGCGCTCGGCGG

TCGTCCGCCCCGCCCCGCTGGCGTCGCGGAGGTAGACCACGTCGCCCGCG

GCGATGCCGTACTCCTCGACGGTGTGGTCGAGCGCGCCGTTGGTGAACTG

CTCGACGATTTTGACGGGCACGAGATTTCCGCCGGATTCGTTCGCTTCGC

TCTCTCTTCCGGCGACTTCCCACTCGCTGTCGCTCGTGAAAATCCCGTTC

GTGTTCACGTCCGCGAAGTTCGAGTGGTCGAGCTTCCACAGCGACTTCAG

CTGGTCGAGCTTGTCTGCGAGCTCCGCCTTGTCGGATTCGAGCGATTCGA

CTTTCCGCTCTAACCGACCGTTCTCGCGTTCCAGTCGGGTGACTTCGCGG

CGCTCGCGGGCCTCGCGACGCTCCTCGCGGCGGGCGTCGGAGAGTTCCTC

CTTGTACTCCTCGATGGTCTCGTCTTTCTCCTCGATGGTCGACTTCAGGT

CCCCGACGTGCGATTCGAGGCGCTCGACCCGCGACCGCAACTCCCGAATC

TCGCGTTCTTCGGGCGTGAGTTCGGGTTCCTCGTGAGCCTCCTCGTCGGC

GTCGTCGGGGTCGCCCCCGTCGCCGTCGTCGGCCATCTCGCGGAGAACGG

CCTCGACCGACTCCTCGCTGACGAGGACGCGGGCGATGACCTCCTCGCGG

TCCACGTCGGCGGGCACCTTCCGGGAGATGCGCTCGAACTGGTCTTCGTG

GTCGTCGAAGGCGAACAGCGCCGCGGCGAGGGCGTCGCGCTCGTGGTCGT

TCTCGTAGTCGACCTCGCGGGTTCGGTGGAGCTTCTCGTCCACGGGGATG

TCGCTCGCGGGGGCCCAGCCGGCGGCGTCGAAGCTCCGGCGGAACTTCTC

GACCGTCTCGGGCATCGGCTGCACGTCGGCGGCGACGAGACTCGGTCGCC

CGCGCTCGATGAGCCACTCGATGACGGCGGCGGTGTCGGCGGTGCGCGTC

GAGTGCACGTCGAGGACGCGCCCGTCGAGGCCGACGACGGCGACGGCGGT

GGTCGTGCCGGGGTCGATGCCGACGATGACGCGGTCGCGGCGCTTCACGA

GCGGTTCGAACTCGATGCCGTCGCGGCGCTCGCGCTCGATTTCGACGCGG

GTGTCGCCCGAGCGGTGGCTGGACACGGGGATGTCCTCGGGCCGCCCTTC

GACGGTAAACAGCGCCTGCGAGTAGCCGCCGTACTTCTCGGTCACGTCCC

GTTCGTAGTCGAGGTTGGCCTCCTTCAGCGAGGACTCGACCTCGCGGGAC

TGCTGTTTGACCGACCCGTGGATGCGGCGCGTGTAGCGGTCCTGACTCCA

CCCGCCCTTACCCGTCGAGCGGCCGCGAGAGACCTTCACGGTCGTCGTGT

TCTCGAATGCGGCGACCTCGTAGCCGACGTTGGCGAGGGCGAGCCGGGCC

GCGGCCTCGGCCTCCTTCATCGGGTCTTTGCCGTAGGGGACGCCGTGGCG

CGAGGCGACCCGCGAGAGCGGTTCCGGTCGCTCCGCGCCGGTCACTTGCA

CCAACTGCGTCCCGTGGGGGAGCCACCGCAGGAAGCGGACTAAATCGTCC

TTGTCGGTCGCCAACTCGTACATGTTGTCCGTGGCGACGACGCGCGGTTC

GTCGCGCTCGATGAGGCGGCGTAACTTGCGGAAGGAGACCACGTCTCGTT

CGATTCGGACCTCGTCTGACTCGGTGTCTCGGGTGTCCAACACGACCAGC

GCGTACGACGGCGAGTCGCCGCGGATATCGCCGCTCTGGATGTCGACGCC

GAAGACGACCGAGTCGAGCGCACTCGTCCGGTCGTTCACGACGGCGACTA

GGGTCGCGCCGAATATATACTCCACGCCGGTTGCGGATACCGACTCGCCT

CGGGTGTCGCGTTTGCCCGTCTCGGCCGACGACGCCGTTGCCACCGACGA

CACCGCCGCCGGGACCCGCGATTGACACGAAACAAACAAGTTTCGGCTAT

TTGAAAGTAGAGTGATAAGTTCTGACATCCCCGACCCGCCGTCGGTGACC

TCCCGGTCGCTGACGCGCATCGCCCTCATCGGTGTCGTCGCCCTCCTCCT

TATCGCCGCCCCAATCGCCGGGGTCCTCGCGTGGGAACCCGTCGAGGAAG

GGAACGTCAAGGTGGTCAAGAAGTGGGGCGCGACCACCGGCACCGTCTTC

GAACCCGGCGCGCACTTCGTCAACCCCGTCTCGCAGTCGACCTCGTCGCT

GTCGGTGCGACCGCAGTCGTACACGATGTCGTCGTCGACGAGCGAAGGCG

ACAGGCGCGGCGACGACGCCATCACCGTCCTGAGCGAAGACGGCCTCCGG

ACGGACATCGATGTGACCGTCCGCTACCGAATCGACGCCGGACAGGCGGT

CGAGTTCTACCGCAACTACCGGACGCTCGCCACCGCCGAAGAGCGCCTGA

TTCGCCCGTCGATTCGGTCGGTGCTCCGGACCGAAGCGGGCCGACTGCCC

GTCACCGTCATCTACACCGGCGAGAGTCAGACCCAGTTGAAGGCCGCCGC

CGAGCGTGAACTCGCCGAGGAGTTCGCCGACGACGGCCTCATCCTCGAAG

CGGTGCAGGTCCGCAACGTCGAACTCCCCGCCGAGTACGCGCAGGCGGTC

GAACAGAAGGAAATCACCGAACAGCGCCGCCAGCAGAAGCAGGACGAACT

CGCCGTCGAGGAACTCGAAGCCGAGCGCAAGCGCATCGAGGCGCAGGGGC

AGGCGGACGCCAACCGCATCCTCGCGGAGTCGCTGTCCGACGAGGTGCTG

GCCCAGAAGTACATCGGGAAACTCGACGAGACGGACACCGTCTACATCCC

GGTCGGCGACGGCGGCTACCCGCAGTTCGTCCGGTCGCTCGATAGTGACG

GGTCGTCCTCGTCGGGTTCGTCCGCCGGTTCGTCGTCGGACACTACCTCG

GCGTCGTCGAGGTCGTCGACCTCGTCCGGCTCCGAAAACGAGACGAGCAA

CTGACGCCGTCGATGCGTCTCCTCCGTGAACTGGCCGTCGCGGTCGCGTT

GCTGGTCATCGTCGGCGTCCTCGCGCGCTCCGGTGTCGGCCGGTTCGTCC

TGCCGGTCGTCGGCCTCGCGGTCGCCGCGGCGCTCGTGGCGCTCCTCGCA

ACACAGTCGGCGTACCCACGGACGGCCGTCGGCCCGCGGACGAGAATCAT

CGAATCGGCCGCCCAGTCCGCGGACGCCGCCTGCGTCGAGTGCGGGTCGC

CCGCGACGGCGCGCCGGCGCTACGTCCGCGAGTGGGTCGTCCTCGGGGTT

CCCGTCGTCCTCCTCGACGACGGCGAGAACCCGGTCTGCGACGCCCACCG

CGACTGACCCGACCTCGTCTTTCGGCTCGATGGCCCGTTCAGCGGCCGCT

CAGACGAGCCTGCCCGTCGAGACGAACTCGACGCGCCCGCCGACGCGGAC

CGCCACCTCGTCGCCGCGGTCCTCGGCTTCGAGGTGGAGTTGGGACGGTC

GGCCCATCTCGTAGCCCTGCTCGACCGTGACCTCGATTTCGTCGTCTCCG

AAGTAGCGATGGCGGGCGAGGTAGCCAGCGAAACAGCCGTTCGCGCTCCC

GGTTGCGGGGTCCTCGGTGACGCCGTGGCCGGGCGCGTACATCCGCGCCG

CGAAGTCGTTCGCGTCGTCGCGGGGGTCCGGACAGAACAGAAAGAGGTTC

TCGACGCCCACGTCGTCGAAGAACGACCGGTACGCCGGGGTAAACACCTC

GCTCCGGGCGAGCGCGTCGCGGTCGCGGAGCGGAATCATCACCGCCGGGA

GGCCGGTCGAGACGACCTGTATCGGCCAGTCGGCGTCGAAGTCGTTCGCG

TCGAGCGACAGCACCTCCGCGAGCGTCTCGCGGTCGAGTTCCGCGCCGAA

CCTCGGCGCGTTCTGCGTCATCCAGTACTGCTCGCTGTCGCTTCCGTCGC

CCCGGACCTCGACCGAAATCGGACCGACGCCGAGGTTCAGCGTCACCTCG

TCGCCCGCGCCGAAGTGGTCGCGGAGGACGGCGGCCGTCCCGAGCGTCGG

GTGTCCGGCGAACGGAATCTCGCCGTCCGGCGTGAAGATGCGAACGTCGT

AGCCATCGTCGGGCGACCCGCCCTCGATGAACGTCGTCTCCGAGTAGTTC

ATCTCGTTGGCGAGCGCGGCCATCTGCTCGGAACGCAGCGCGTCCGCGTC

CTCGAAGACGGCGAGTTGGTTGCCGGCGTACTTCGCCGTGGCGAACGCGT

CGACGGTGTGGAAGGCGTTCTGCGGCACATCGACGGGTGCGCGCGGGCGA

GGGAAAAGCGTACCCGTACCGCGGCCCCCGGACTACTCCCCGGTCTCCGC

CCCGCCGCCGTCGCTGTCGGCGTACGGGACCTCCAGTTTCTGGCCGTCCT

CGGCGACGAACGCCTCGCCGTCGTACACCTCGCGGGCCTGTTCGAGAAGC

GGGCTCGGGTCGGCGGCGTACCGGGCGGAGATGTGCGTCAGCGCGAACCG

CCGCACGTCTGCGTCGCGGGCGACGCGGGCGGCCTCCCGCGCGGTCGAGT

GCGCGGTCTGTTTCGCCCGCTCGGCCTCCTCGTCGGTGAACGTCGCGTCG

TGGACGAGCAGGTCGGCGTCGCAGGCGACCTCGACGGTCGAGTTGAGCGG

GCGGGTGTCGCCGGTGTAGACGACGGTTCTGCCGGGTCGGGGGTCACCGA

CCACCTGCTCGGAGCGCACGACGGTTCCGTCTTCGAGTTCCACGTCCTCG

CCGGCGTGGAGGCGGCCGAACGCCGGGCCGACGGGGACGCCGAGTTCCTC

GGCCTTCTCGCGGTCGAACCGGCCGGGGCGGTCGTCCTCGACGAGGGCGT

AGCCGACCGAGGCGGTCCGGTGTTCGGTGTCGAACGCGCGGACCTCGTAG

TCGTCGGCGCGGTAGGCGACGTTCCCCGGTCGGACCTCGTGGACGGAGAC

GTGGAAACCGGGCTGGTAGCCGCCGGCGTGGACGAGTTGTTCGAGGTGAC

CTTTGCTCCCCGGCGGGCCGTGGATGGCAAGCGAGTCGTCGCGGTCGTTG

AAGTCGAGCGTCTGAATCAGCCCGGGGATGCCGAGGATGTGGTCGCCGTG

GAGGTGGGTGACGAACAGGTGGCTGACGCCGAACCCGGTCCCGTAGCGCA

TCATCTGGCGTTGGGTGCCCTCCCCGCAATCGAACAACAGGCGCTCGCCG

TCGCGGTTCACGAGGAACGCGCTCGGCGCACGCGCCGTGGTGGGGACGGC

CCCGCCGGTCCCGAGGAAGGTCGCGCGCATGGACATGCACCACGATGATA

GCGGCGGGACTAAACGTGCGTCGAATCCGTCGCGGCCGCTCGCCGCCCCG

AGGCGGTGCGGCCCCCCGACGCCCCGGACCGACCGATTCTTACCCGACCG

TCTGCTACCGGAGTCCAATGGACGCGCCGCTGTGGACCGAGACGCACGCG

CCGGGGCTCGACGACCTCCCACAGCCGGAGGTCCGCGACCGCCTCCGACG

CGCCGTGGACGAACCGATGAACCTCGTCGTGCAGGGCCCGCCGGGCGTCG

GCAAGACCGCCGCCGTCCGCGCGCTGGCCCGTGAGGCGCACGCGGACCCC

GAAAACGACCTCATCGAACTCAACGTCGCGGACTTCTTCAACCGGACGAA

AAAGCAGATTCGCGCCGACCCGCGGTTCGAGCAGTTCCTCGACGGCCGCA

GTCGCATGGCGAAACGCGACATGATAAACCGCGTGCTCAAGGAGTCCGCC

GGCTACGCCCCGATGTCAGGCGAGTACAAGACCATCGTCCTCGACAACGC

CGAATCAATCCGCGAGGACTTCCAGCAGGCGCTCCGCCGCGTGATGGAAC

AGCACCACCGGACCACGCAGTTCGTCATCACCACGCGCCAGCCCTCGAAG

CTCATCCCGCCGATTCGCTCGCGGTGTTTCCCGGTTCCCATGCCCGCGCC

CGACGACGAGGCGCTCGAAGCGCTTCTCGCCGACATCCTCGACGCCGAGG

GCGTCGACTACGACGCCGGCGGCCTCCAGTTCCTCACCGACGCCTCGAAC

GGCAACGTCCGAAAAGCCGTGCTTTCGGCCCAGCGGACGGCCACCGAGGC

CGACGAGGTCACCATGTCCACGGTCCACGCCGCCCTCGGCGACGTGGGCT

TCGACGACGAGCTGAAGACGCTCCTCATCAACGCCCGCGAGGGCGACATC

AAGGACGCCCGCAAGACCCTCACGACGCTCCTCGACGACGAGGGCTACGA

GGGACAGGAGCTCCTGGCTGACATCCTCCGCGTCGCCGACTCGACCCCCG

AGCGCTTCGCCGACGGCGAACTCGCCCGCCTGCACGAACTCGCGGGGCAG

GTCGACCTCGACATCTCGACGGGCATCGACGACCGCCTGCACATAACCCA

CCTCCTCACGAGTTGGGGCGCGGACGTCCGCGGCGAGGCATAATGCGGTT

CGCACCCGGCTACCGACGGTTCGCACTCCCCGCCTTCGTCGGCGCGGCGG

TCGCGGCGGTCGTCTTCCCGCCCCTCGGGGCCGTGCTGCTCGCGGTCGGC

GCGTTCGTCCTCTGGTTCTTCCGGGACCCCGAGCGCTCGCCGCCCGACGA

ACCGGGCGTCGTCGCCCCCGCCGACGGGCGCGTCTCCGTGATTCGCGTCG

AGGACGGGCGCGTCCGCGTGGGCGTGTTCATGAACGTCACCGACGTGCAC

GTCAACCGCGCGCCCGTCTCCGGTTCGGTCCGAACCGTCACCCACCGCCC

CGGCGCGCACAAACCGGCGTTCTCGAAGGACTCCGACCGCAACGAGCGCG

TCGATATCGAACTCGACACCGACGCGGGCGACCACGAAGTGTCGCTCATC

GCCGGCGCGTTCGCCCGGCGGATTCACTCGTACGTCGCGCCCGGCGACGA

ACTCGTCCGCGGCCAGAAACTCGGCCACATCGACTTCGGCTCGCGGGCCG

ACGTGCTGTTACCACCGGAGTTCGGCTCCGAAGACGTGGTCGTCGAGAAG

GGTGAGTCGGTGCGGGCGGGCGAGACGGTGTTGGCGCGGCGGGAGTGAGC

GTCGCTGTCGGCGTTGGCCGTCTCGCCCACCTTGCTGTTCTGTTCGACGG

TGAGCCTCGGGAACGCCTCGCAGCCCATCGGTGATAGATACACAAACCAG

TAGCACCTCGGCCCTCGACTGGGCAAATATTGTTCCCCTGGCCATATTTA

TCCTAAATGTCTCGAATATGTCGCTAAAGATGTGTGAATACCCACTTGGT

CATTCCGACACGAACGCGTCCGTTCGGTGTGTGTCGCTATGACCGGCGAG

TTCGTTCCCGGTGAGGTCATCACCGCCGACGGGACCATCACGCTGAACGA

GGGGCGAGAGACGGCCGAGGTGACGGTGGGGAACACCGGCGACCGGCCGA

TACAGGTCGGCTCTCACTTCCATTTCTTCGAGGCGAACGCCGCGCTGTCG

TTCGACCGCGAGGCGGCCTACGGGATGCGGCTGAACATCCCCGCGGGGAC

CGCGGTCCGGTTCGAACCCGGCGACCAAACGTCGGTCGAACTGGTCGCTA

TCGGCGGCAAGCGCCGAGCCCACGGGATGAACGGGATGGTCAACGGCAGC

GTCGACACCGACCCGAGCGCCGCCGTCGAGCGCCTGCGCGCGGCCGGGTT

CGCTGACACCGGCGCTCCCGACGACGAACACGGCACCGCGGAGGGGTCGA

GCGAATGACGAAGGACATCGACCGCGAGGCCTACGCCGACCTGTACGGCC

CCTCGGAGGGCGACCGCGTCCGACTGGCCGACACCGAACTGTTCGCCGAG

GTCGAGACGGACCTCCGGACGCACGGCGACGAGGCCGTCTTCGGCGGCGG

GAAGACGCTCCGCGACGGTCTGGGGATGGCCCCCGACGTGACCCAAGCAG

ACGGGGCGCTGGACTGGGTCATCACGAACGCGACCGTCATCGACCCGGTT

CTCGGCGTCGTCGCCGGCGACATCGGCATCCGCGACGGCGACATCGTCGG

CGTCGGCAAGGCGGGCAACCCCGACACGATGGACGGCGTGGACATGGTCG

TCGGCCCGGCGACCGACGTCTACCCGGCCGAGGGGAAGATTGCGACCGCC

GGCGCGCTCGACATCCACGTTCACTGGAACTCCGCGCAACTGCACGAACA

CGGGCTGGCGTCCGGCATCACGACGATGCTCGGCGGCGGCTACGGCGGCG

GCGCGACGACCTGCACGACCGGCCCGCAGAACATCAAGCGGTTCCTCCAA

GCCGCCGAGGCGTGGCCCGTCAACGTCGGCTTCTACGGCAAGGGGAACGC

GTCCACGCCCGAACCCCTCATCGAGCAGGTCGAAGCCGGCGCGTGCGCGC

TCAAACTGCACGAGGACTGGGGGTCGATGCCCGCGGCAATCGACACCTGC

CTCGACGTGGCCGAAGACGAGGACGTACAGGTGTGTATGCACACGGACAC

GCTGAACGAGGCCGGCTTCGTCGAGAACACGTTCGCGGCCGTCGACGGCC

GGACGATGCACCTGTTCCACATCGAGGGCGCGGGCGGCGGTCACGCCCCC

GACATCATGGAACTGGTTGGCGAGCCGAACATGCTCCCGTCGTCGACGAA

CCCCTCGATGCCGTACACGGACAACACGTTCGACGAGCACCTCGACATGG

TGATGGTGTGTCACCATCTCAACCCCGACGTGCCCGAGGACGTGGCGTTC

GCGGAATCGCGGGTGCGCGCCGAGACCATCGCCGCCGAGGACGTGCTCCA

CGACATGGGCGCGATTTCGATGATGACGACCGACTCGCAGGCGATGGGTC

GGCAGGCCGAACTCGTCTCGCGGACGTGGCAGGCCGCCTCGAAGATGAAG

TCCCAACGCGGGCCGCTCCCCGAAGACGAGGGGACCGGCGCGGACAACCA

CCGCATCAAGCGCTACCTGGCGAAGTACACCATCAATCCCGCGATATCCG

CGGGTATCGACGACTACGTCGGCACGCTCGAACCCGGCAAACTCGCGGAC

GTGGTGCTGTGGGACCCCGCGTTCTTCGGCGTCAAGCCGGCGATGATATT

CAAAGGCGGCTTCCCCGTCCACTCGGAGATGGGCGAGGCCAACGGCTCGT

TGATGACGTGCGAACCGATTCTCCAGCGCCAGCGGGCCGGCGCGGACGGC

AAGGCCAAACACGGCCTCTCGCTGTCGTTCGTCTCCCCGGCCGCCGCCGA

CAACGACGTCGGCGAGGCGTACGGCCTCGACTCGCGGGTCGTCCCGGTGG

GCGGCACGCGCACGCCCGGCAAGGAGGACATGCTGTACAACGACTACTGC

CCCGACGACATCGACGTCGACCCCGAGACGTTCGAGGTCGAAGTCGACGG

CGAACTCGTCACCTGCGAACCGGCTTCCGAACTCCCCCTCGCACAGCGGT

ACACGCTATGAAACTCACCCCGAAAGAACAGGAACGGCTCACGGTCTTCA

CCGCCGCAGAGGTCGCTCGACGCCGCAAGGAGCGCGGCGTCCCGCTGAAC

CACCCCGAAGCGGTCGCGTACATCACCGACTGGTGTATCGAGCGCGGCCG

GGACGGACAGTCCGTCGCCGAGATTCGCTCCGGGGCGTCGCAGTTGCTCG

GCCGCGAGGATGTCATGGACGGCGTCCCCGAGATGATTGACATGATTCAG

GTCGAACCGATGTTCCCCGACGGCACCAAGCTCGTCACCGTCCACGACCC

CATCAGGTCCGACAGCGTCGGCACCGCCGATTCGACGGCGGACGCGGCGA

CCGACGGGGGCGACACAGACGACATCGACACGAGCGGGGGTGACGACGCG

TGAGCCTCACGCACCGCGACGTGGCGACGGTCGGTATCGGCGGGCCCGTG

GGATCGGGCAAGACGTCGCTTCTGACCGAACTCGTGCCGCAGCTTCGCGA

ACGGGGGTTGAACGTCGGCGTCATCGCCAACGACATCCTCACGCAGGAAG

ACGCCGACGTGTTGCGCGAGCGGTTCGCCGGGGTCGTCCCCGAGAGCCTC

GTCGCGGGCGTCGAGACCGGCGCGTGCCCGCACACGGGCATCCGGGAGGA

CCCGTCGATGAACCTCCAGCAGATAGACGACTTCCTCGCGGACCACCCCG

AGCTAGATATCGTGCTGATAGAGAGCGGCGGCGACAACCTCGCGGCGACG

TTCAACCCCGAGCTCGCGGACTACTCGCTGTACGTCATCAGCGTCGCGGA

GGGCGACGACATCCCGCGGAAGCGCGGCCCCGGCGTCGTCGACTGCGACC

TGCTCGTCATCAACAAGACCGACCTCGCGCCGCACGTCGACGCCGACCTC

GACGTGATGGAGCGGGACGCGAAGGCGGTCCGAAGCGGGCCGTTCGTCTT

CACCGACTGTCGTTCTCAGACGGGCATCGAGGAGGTGCTGACGCACGTTC

GCGAAGGGGTGCTGTTCGCCTGATGGCCGCCGACTCGCCCCCGCCGTCGT

TCGAACGGTACGCGGCGGAGCCCGTCCCGCAGGCGGCGGTCGGCGCACCG

GGAAAAGACGGCGTCCTCGAACTGACGTTCGAGCGGACCAGCCGCGGGAC

GGCGCTCGTCCACGACTACGCGACCGTGCCGTTCCATATTTCGGGAACGC

TCGGCCACGACCCGCACCCCGAGGCAGAGACGGTGTTCGTCCAGTCGCCG

ACCGGCGGCGTCGCCCAAGGCGACCGCCACGACCTGACCATCGCGGTCGA

AGCCGACGCAATCGCGCACGTCTCGACGCAGAGTTCGACGAAGGTGCAGA

CGATGGAGCGCAACTACGCCGGCGTCGACACCGCGCTGTCGGTCGCCGCC

GGCGGCCACCTCGACTACGTGCCGGAGCCGACGATTCTCCACGCGGACGC

CCGGTACACGCAAGACCTGCGTCTCGACGTCGCAGACGGCGCGACGGCTA

TCGTCGGCGACGTGGTCGTCTCGGGGCGACTCGCCCGCGGCGAGCGGTTC

GAGTTCGACCGCTATCGCTCTCGACTGCGCGCGACCGGCCCGGACGGCCT

CCTGTTCGAGGACGCCACGCATCTCGCGCCCGACGAGTCGGACCCGAGCG

CGCCGGGACTCCTCGGTGAGCACGCGGTCTACGGGACCCTGTTCGTCGTC

GCGCCGGGCCGAGACGCGGCCTCGTTGAGCGACGACCTCCACGAGGCGGT

CGCGGCTACCGACGCCCGCGCCGGCGCGACCGAACTGCCGAACGACGCCG

GTGTCGCGGTTCGCGCGCTCGGTGACTGCGCGGACACCGTCCGGACCGCG

TGCGACGCCGCCTGGGACCGTGCGCGGCGGGCGTTACTCGACGCCTCCGC

GCCGTCCGGGAGGAAGTACTGATGCTCGTCGCCGACACCTACCTCGGCCA

CCGGGACGACCCGGCGGTCCGAGGTAGACTGGACGAGGCCGACGCCGCCC

GCGTGGTGCTGTCCGACGACGACCGACGGCGCTCGCGCGTCAGAACCGAG

ACCGAAGACGGCCGGGATCTCGGCGTCGTCGTCGGGCGCGAACTCGACGA

CGGCGACGTGCTCGAAACGGAGACTGGCGACCTCGTCGTGGTCGAACTCG

CGGCTATCGACGTGCTCGTCGTCGACGTGGGCGCCGCCGACGTGTCCACG

ACCGCCGCGGTGGAACTGGGTCACGCGCTCGGCAACCGCCACTGGAACCT

CGCCATCCGTGACGGCGAGGCGCTGTTCCCGGTTCCCGACACCTACGAGC

GGATGGAAGCGACCGTCGCGGACCACCTCCCGCCGGGCGTGACGACCCGA

CGCGAGACCGTCTCGCCGGCTCTCTTCGACGGCGACGAACCCGACCACGG

CCACGGCGGGCACGGTCACGGTGGACACGACCACAGCCACGAGCATGGCG

GACACGGCCACAGCCACGGCGAGCACAGTCACGACCATGACGACGGGCAC

AGTCATGGCCACGACCACGATGATGGACACGACCACAGCCACGACGGCCT

GCTCCGGTCCGCTCGGGGGGACGAGCGATGACCGACGGCGCTCGGCTCGA

ATCCTTTCGGTTGGCCGATTCGTTCCTCCCGGTCGGGACGTACACCGCCT

CGTACGGCCTCGAGCAGTTCGTTCAGGAAGAGCTGATCTCCGACGCCGAC

GACCTCCGGGCGTTGCTATCGACGTATCTCAAACAGCAGGTCGGCCCGTG

CGAACTGGTCGCGCTTCGGGCGGCTCACACCGCCGCCCGCGACGGCGACT

TCGACGCGGTCTGTCGCGCCGACCGCCGCCTGACCGCGGCGACGCTGTCC

AAGGAGTTCAGAAAGAGCGCCCGACAGTCCGGCAACCGCCTCCTCTCGCT

CCAGCGGGAGCTCCGCGAGTCGCCCCTCCTCGACGAGTACGCCGAGGCCG

TCGACGCCGGCGACGCGACCGGGAACTACGCCGTCGTCCTCGGCGTCGCC

GCCGCGAGCGCGGACATCGACGCCTCGGAGGCGTGTCTGCTCTGTTGTCA

CGGCTTCGTGACCGGACTCGTCGGGGCCGCCCAGCGGGTGCTCTCGCTCG

GACACACCGACGCCCAGCGGATTCTCCACGACCTCCAGCCGGTGATGACC

GAGGCCGCCGCCGACAGCGCCGACCGAGGGATAGACGAGATGTCGCCGTT

CGCGCCACTCATCGACGTTCTCTCCGCGAATCACGAGCGCGCCGACCGGC

GGCTGTTCATGAGCTAAGTCGGGCCGGGCGACCGCCGCCAGCGCGCCGTC

CCGGCGAGGGATGTCGAGCACACAAGGCACTTAACGTGACAGATGAACAA

CCTCTCATGCAGCGCAGACAGGTCCTCGCGTCTCTCGGCTCGCTCGCCCT

CCTCTCGGGATGTCTCGGCGGCCCCGCCGAATCCCCGACCGAGACGACGA

CCACGACCGGAACCAACACCTCCGAGTCGGAGTCGACCGAATCCGGACGG

GCCGCGACCACCACGAGCGACTGCGGTCGGACGGAGTTCTGCGAGGGGTC

GGAGCTCGTTCGCGTCCGGGTCGACGGTGGCTTCTCCGGCAAAGTCGTGT

TGAACCCCGCGTGCCGCGAGAGCGACATCGAACTCTCGCCCGGCGAGACG

GAGACGCTCATCCGACAGACCGACGCCGAGACCTGCGACGTGGAACTCCT

CGTCGACGGCGAAGTGGCCTACGACGGGCGGATTCAGGACTACGAGTACG

TGATGGTGCGCGTCGGCTCGGACGGGGAAATCTCACTGCAGAAGGAAGTC

CTCTAACGGTCTCAGTCGCCTCCAGTCATCTCCGGTCGCCTCGGCTACCG

GCGCGTCTCGACGGCCAGCACCACGACCGAGACGACGAGCGAGAGGACCG

CCACCACGAGACCGAACAGGTAAGACGGGTTCGAACTGGGGTTCGACAGG

AGCGACACCAGCAGGAACGACGACCCCATCGCGACGATAGCCATCGCCCG

CGGGTCGGGGTCGTACGCCTCGGCGAGGGTCTCTCGAACCGTGCGCGACA

TGCCTCCGCTTCGACGCGCTCGCTCATAGCCTTGTCGCGCGGTTGAACAA

ACCCGTCTCGGCTGCGGCCGTGTCGGAGCCGCCGCACCGCCGCCGATTCC

GACTACCGATGCAGCAGATGCACGTACCGCACCAGCGACCGGTGGACCCG

TCGCTCGAACACTCGTTCGACCCCCCAGCCGGCGTCCTCGGCCGCCTCAG

CCCACGACCGGTCGGCAACCATCACGCACTCGGGGGCGACCCGGCGGACC

TCCGCCAGCGCGCCGCCGACGAGGTCCGCAAGCGAGTGGCGGGCGATTTT

TGACTGCCGGCCGTAGGGGGCGTCGAACACCGCGCCGTCCATCGAATCGT

CGCGGAGGGGGAGCGCGGTGGCGTCGCCGCGGACCACGTCGCCGGAGCCG

ACGTAGTGACGCAGGTTCTCCCGCGCGCCGCCGACCATCTTCGCTTGGGC

GTCGACGCCGACTACGTCGGCACCGACGAGGCCGGCTTCGAGGAGGACGC

CGCCGGTGCCGCACATCGGGTCCAGAATTCGGGCACCGGGCTCCGCGCCG

GCCATGTTGACGAAGGCGCGGGCGTCCACGGGGGCCATGCTGCCCGGCTG

GAAAAACGGCCGGTCGGTCGGTTTCCGGTCGGTGAAGTCGCGGACGGCCT

CGACGGCGGTCCAGCCGACGAGACAGGCGTCCTCGGAGAAGCAGACCCGT

AGCTCGTGGTCGGGGTCGTCCAAGTCGACGCCGAAGCCGCGGTCGACGAG

CGCCGACCCGAGTTCGCGCTCGGCGTCGGTCGTGCTGATACCGGTGAGTC

CTCGCACGTCGCGGGCGCGGACGGCGACGGTCCCCTCGCGGTCGACGTTC

GCGGCCGCGACGACGGCGCGGGCGGCCTCGATGTCGGGGGCGCATCGCCC

GACCAGTTCGACGACGCGCCGGGTGTAGGCGAGTCGCCTCGCGCGCTCCG

GTTCGACGCCGCGGGCGGTGGCGAGGCCGGGGGCGACGACCGTCACGCCC

GTCGCGGCCGCCTCGGCCTCGCGGGCGGCGAAGGCGTCTTCCTCGCCGGC

GAGTTCCAATCCGTACACGCTCGGACCTGACGGGCCGCGGAAATGAGGGT

ATCGGTCGTCGTCGCGCCCGCGGGGGCGTCGGCCGGCCCGGACCGACCGC

GGCTCCGCTGATCCTGACACCTCGACGACGGCGAGACTTATATTGGGCGC

TCCGAATCCACTCGCATGACCTCTCACGACGCCATCCTCTGGGGCGCGGC

GGCGCTCACCTGCGGGCTCGGTGACTACGTCACGACCGTCCTCGGAGTCA

GGACGGCGGGCGTGCAGGAAGGCAATCCCCTCGTGCGACGCCTCTCCGGC

GGCGACCCCGGTCCCGGCTCGTTCGCGGTGTTGAAGCTCGTCTCGGTCGC

GCTGTTCTTCGCGGCCTACTGGGCGCTCAAGCCGGCGGTCGCCCGACTCG

CAGTTCCACTCTCGCTGACAGTTCTCGGCGCGGTCGTCACCGCTCGAAAC

GCCCGGATTATTCACCGCCGCGCGTAAGCTGTCATTCCCTCGCACCAATC

TTTTTAACCCTTAAATACGTGACTTAAATCGTTGTATGACCGACCCCAAG

GACACAATCAACATCGAAAACGTCGTCGCCTCCACGGGAATCGGCCAAGA

GCTCGACCTCCAGAGCGTGGCGATGGACCTCGAGGGTGCCGACTACGACC

CCGAGCAGTTTCCGGGACTCGTCTACCGCACGCAGGAGCCGAAGTCCGCG

GCGCTCATCTTCCGGTCCGGGAAAATCGTCTGCACCGGCGCGAAGTCGAC

CGACGACGTCCACGAGAGCCTCGAAATCGTCTTCGACAAGCTCCGCGAGC

TTCAGATTCCGGTCGACGACGACCCCGAAATCACCGTGCAGAACATCGTC

ACGAGCGCGGACCTCGGCGAGAACCTGAACCTCAACGCCATCGCCATCGG

GCTCGGTCTCGAAAACATCGAGTACGAACCGGAGCAGTTCCCCGGCCTCG

TCTATCGCCTCGACGAACCGAGCGTCGTCGCGCTTCTCTTCGGGTCGGGC

AAACTCGTTATCACGGGCGGCAAACAGCCGACAGACGCCGAGGCGGCGGT

CGACGTCATCATCTCGCGGCTCTCCGAACTCGGTCTCCTCAACGGGTCGT

TCTAACGCCACCGAACCTCGACTGCGTCGCGTAACCGGGACTCGAATCGA

CCGAATCTAAGTCAGTCGCGTCCTTCGGGCCGGTATGGACGCGCTCACCT

TGCAGACGGCTACCGGTGCGCCGGTCACGGCCGTCGCCGGAACGTTCGCG

CTGTTCGCGCTGTTCCTCTCTCTCACCGCCCACATCGCCGCGCGGAACGT

CCTCGGCGACGTGGAACTCAAGAAGGCGTTCGCCGTCGGCCCGGTCCCGG

CGGCCATCGCCGTCGTCTTCACCTCCTTCGGCTGGAACTCCTTCGTCGCG

CTCGCGCTCGCACTCGGCCTCGACTTCGGCTTCGTCAAGTACCTCTACGG

CCGGTCGACCCGCCTGTCGGCGTACGTGATCGTCATCCACTTCGTCGTCT

CGGTGCTTCTGGGTCTCGTCCTGTTCGGTCTCTCCGCCATCCTGCTGACC

GCGCCATTCTGAGGTTCCCGCCGTACTTTCTTGCCTGAGGCCGCCGAACC

CCCGTTCGTGTCTCCCCGCCGACTCCTCTGGAACGACGCGGAACGCCGGC

CGCGCGCCCCGTTTCGCCTGCTTTTGACGCCCGTCGTCTTCGTGGCCGTC

TCGCTGGTGGTCGGATTCTCGCTTTCGACCGCGCTCGGCGGCCTCGCCGG

GTCGAGCCGCCTCGCGTCGCTCGTCGTCTCGCTCGTCTCGGTCGGCGCGA

GCGGTCTCGCCGCGTTCGTCGTCGCGCGGTACGTCGATAGGCGGACCGTC

GCGGACCTCGGACTCGGACTCGACCGCGAGTGGGCGGTCGACCTCGGGTT

CGGCCTCGCGCTCGGAACGGGCCTGATGACCGCCGTCTTCGTCGTCGGCG

TCGCGGCCGGTTGGATTGCGGTCTCTCCCGCCCCGCTCGGCGTCGACCGA

CTGCTCGGCGTCGGGTCGCTTCTGGCGTTCTTCGTCGTCGTCGGCATCGC

GGAGGAACTCCTGCTTCGGGGAATCGTCCTCACCGACGTCGCCGAGGGCC

TCCGCTGGCGGTTCGGCCCGGACGCGGCGGTCGCCGGCGGCCTCGCCGTC

TCCTCGGCGGTGTTCGGCGTCGCCCACTACACGAACCCGAACGCGGGGTT

CGCCAGCACGACGAGCATCACGCTCGCGGGCGTCATGCTCGGCCTCGGCT

ACGCGCTCACCGGCGACCTCGCCATCCCGACGGGCATCCACATCTCGTGG

AACTTCGTGCAGGGCGGCGTCTTCGGCTTCGCGGTCAGCGGCCTCGACTT

CGGCACGTCGCTCGTCGAGACGACCGAGCGCGGCCCCGACGTGATTACCG

GCGGCGCGTTCGGCCCGGAGGCCGGTCTGCTCGGCGTCGGCGCAATCGTC

CTCGGCGCGGTCTGCATCGCGGCGTACGTCCGGGTGCGCCGCGGCGACCT

CGGGTTCGAGCCGTCGGTCACGGTGCCGGACCTGCGCTGGAAGGAATAAC

GGGCGCGACGGGCGCGTCGATTACTCGACTAACCGCTCGATTTCGGTGAC

GAGGATGCCGCTCGCGCCGACGTTCTTCAGGTCGCTGACGACCTCGAACA

CGTCGCGTTCGTTCACGACGGCGTGGACGGCGACCGTCGAGTCGCCGGCG

ACGTCCATCACGGTCGGGCCGCCGAGGCCGGGGATGACCTCCTTCACCTC

GTCGAGCTTCTCGCGCGGCGCGTTCATCATCAGATAGCGCTTGCCCTCCG

CGGACCGGACGGATTCGAGCGCCATGAGCAACTGCTGGACCTTCGGGTCG

TCGACCACGTCGGGGCGGGCGAACAGCCGGACCGACGACGACAGCACCTC

GTCGATGACTGCGAGGCGGTTCACCCGGAGGGTCGTCCCGGTCGACGTGA

TATCGATGATGGCGTCGGCCATCTCGACGTGCGGCGTCAGTTCGGTCGCG

CCCGTGACCTCGACGACCTCGGCGTCGATGCCCTCCTCCTCGAAGTAGGT

CCGCGCGATGTGCGGGAACTCGGTGGCGACGACCTTGCCCGACACGTCCG

CGACGGTCTCGATGTCGCCGTCCTCGGGCGCGGCGAGGACGAGCCGACAC

TTCCCGTACTCCAAATCGAGCAGGTCTTCGAGTTCGTGGCCCGACTCGCG

GACCTGGTCGAGTCCCGTGATGCCGACCTCGGCGGCCCCGTCGCGGACGT

ACTCGGGGATGTCGGCGGCGCGGGCGAACAGCACGGTCACGTCTGGGTCG

ACGGTCCCGGCGTAGAGTTTGCGCTCGGCACCGTTGTCGAGGTGCAGCCC

CGCACGCTCCAGAAGTTCTATCGTCGGTTCGTGCAGGCGGCCCTTGTTGG

GCACGGCGATTCGCATACCTCTCCGTCACGTCCCGGAGGTAATTGACTTT

CGCAAGCCGGAACGGTTTTTGACACGTCTACGAAGTGTCACCTATGGCTC

CCGTCACCTTCGCCGGCGCGCTCGTCGGCGTCTCCCTCCTCGGTGCCGGC

TGGTCGCGCCTCAGCGCCGGCCGCCTCGAACGCCGAGACGTGTCCGTCCA

GCGGTTCAGACAGCGAGTGCGAAGCAACTGGCTCTACGCCGCCGTCCTGT

TCGTCGGCGTCGTCTGGTGGACGAACGCGGACCGAGCGCTCCTCGCGGCC

GTCGGCCTCCAGACCGACTGGCCGTGGGCGGCTCTCGGCTGGGCGCTCAC

CGGCGTCGGTGCCGCGGTCGTGGCGACCGTCTCGTACATGGGCGCGTTCC

CGGTGGCGAGACGCGTGCGAGACGCCGATATGGGCGCGGGGACGGTCGCC

GCGAAGATGTTCCGCTACCACCTCGTCGTCGCGGCGCTCGTCTTCTGCGT

GGTGACCGTGCTACACGTCGAATTCCGGGTCTTGGAGACCAGCGGCCTGA

TCACCCTGCTCGCGTTCGCAGCCGCCGCGTACGCCTTTTCGGCACCGCTC

GTGGGCGTCTCGCAGACGACGGCGGTCCCCGACGACGCGACGCGCGAGCG

ACTCGACCGCCTGTGCGACCGCGCCAGTCTCTCGGTCTCACGGATTCGAC

TCCTCGACGGCGGCGGCCACCGCTCGGACCATCTCGTCCGCGGCCCGGTC

GGTCGGAAGACGCTCTTTCTCACCGACTCGCTGCTCGACCGCTACGACGA

CGAGACGGTTTCGGCACTCTTGGCGGTCGACGCCGCGCGGCTCGGTCGGT

TCGTCTACGAACTTCGACTGTTCACCGTGACCGCGGTCGCCGGACTCGTC

GTCTGGGGTGTCGGTCGGTCGCCGTTCGGGCTCCTCCCGTCACTTCTGGC

CTTCGCCGGAACCGGCGTGATACTCCTCGTGACCGGCGAGTACGCGAGTA

AGCGGCTCATCTATCGGGCCGACGAGGCGGCGGCAGAACGGGTCGGACGC

GCGGCCGTCGCCGACGCGCTCGCCGCGACTGCCGACGAGGTCGACCCCGG

CCGCGCGGGGTCGCGTCTCTCGTTCGAGCCGTCGCTGGCGTCGCGCATCG

AACGGCTCCGAGACGGCTCCGGCGCGGGCGAAACGGACGCGTAGGACCGC

TCCGGGGACCCGACGCTATCCTTCGACGAGGTGGTCGAGCAGGCGGTCGA

ACCGGTCGACGAACCGCTCGGGGAGCGACGGCGACACGTCGTCCAACAGC

CGGGTCGTCCGCTCGGGGTGGGTGAGGACGAGCGTCACGCGGTTGTGCAG

GTCGCGCTCCTTGCGGACCACGTCGCGTTCGACGAGGTGGTCGAGGTGCC

ACTCCAGCGTGCTGCGGGCGACGCCGATGTCGTCGGCCACGTCGTCGGGG

CGGGCGTCGCCGTGTTCGAGGAGGTGGCCGAGGATGTCGCGGGCGGTCTC

CCGGCGGACGAGCGCGAGCGCGCCGCGCTCCCACGCGTCGAACTCGGGGG

GGAAGTAGTGCGTCCGTCCGTAGAACTCGGAGCGGCGGACCGTCTCGTCG

GAGAGCAGTCTGCGGACGTGGTGTTGTACCTGGCCGGGAGCGAGGTCGAG

CGCGCGAACGAGCTCGTTGAAGTGGACTCCCGGGTTGTGCTTGATGTGGT

CGGCGACGCGGGTTCGTGTCTCACTCATGGGTCGTTGACCCCCGCCTCGC

GCTCGATGCTCCGGGCGTAGTAGATGGCCGCGAGGACGAGGCCGGCCATC

GCGAGGTCCAAGAGGTGTTCGACGACGTGGTGGTCGAACTCGGAGAGGAC

GCCGCCGAGCGTCAGCGCCGCGACGGCCGCCCGCGACGCGAACGCCAACA

GCGCCAGCGCGACGAGCAGGTGCGACCGCGTCCGCCGCCGGAGGAAGACG

GCGACGCCGAGCGCGGCCAGCGTGACCGACGAGACGCCCGCGAGAACGAC

GACAGCGGTCATCCACGGACCGCCCGCTTGGACGTGCCCCGGAACGAGCG

CGAGGTTCGACGGGGGCTCCATGTCCCCCGATACTGGGGGTTCGGACCTA

AGGGGGCGTGCTCGTTCTCGATTTTCGGGAATCGGACTACCGCCGGTATT

CGGCCCGCTCAGCGCCGAAACGGGCGGTCATCTCTCGCGGCCGCGAGACG

GTGGATTCACCACCCCGCCGCCCCGAACGAGCGACATGAACACGCTCCGA

ATCGCGGGCGGACAGGTGCTCCGCCCCGACGCGACGGTCGAGGACGCGGA

CGTACTCGTGGACCGCGACGAGGGAACCATCCTCGACATCGGCGCGGACC

TCGACGCCGACGCCGACGAGACGCTCGACGCCGCGGGCTGTCTCGTCACG

CCCGGTCTGGTGAACGCCCACTGCCACGTGGCGATGACGCTCCTGCGCGG

CTACGCCGACGACAAGCCGCTCGACGCGTGGCTCCGCGAGGACATCTGGC

CCGCGGAGGGCGCGCTCACGCCCGAGGACGTGCGCGCCGGCGCGGAACTC

GGCCTCGTGGAGATGATTAAGTCGGGGACCACCGGGTTCGCGGATATGTA

CTTCCACGTCCCCGAAATCGCCGCCGCGGTCGAGGAGGCCGGCCTCCGCG

CCCGCCTCGGCCACGGCGTCGTCACGCTCGGGAAGGACGACGCGGACGCG

CGGGCCGACATCGACGAGAGCCTCGACGTGGCCCGCGAGTTCGACGGCGC

GGCGGACGGCCGGATTCGGACCGCCGCGATGCCGCACTCGCTGACGACGG

TCGCGGAGGAGTACCTCCACGAGTTCGTCGCCGACGCCCACGACGAGGGC

ATCCCGGTCCACTACCACGCCAACGAGACGACCGACGAGGTCGACCCCAT

CGTCGACGAGCGCGGCGAGCGCCCGCTGTCGTACGCGAAAGACCTCGGCA

TGCTCACCGCCGACGACTTCCTCGCTCACGGCGTCCACGTCGACGACGCG

GAAATCGACCTGCTCGCGGACGCGGGCACCGGCGTCGTCCACTGCCCGGC

GTCGAACATGAAGCTCGCCTCCGGCATGGCTCCCGTCCAGAAACTGCTCG

ACGCGGGCGTCACGGTCGGCCTCGGCACCGACGGCGCGGCCTCGAACAAC

GACCTCGACATGTTCGACGAGATGCGCGACGCCGCGATGCTCGGCAAACT

CGCCGCCGAGGACGCCAGCGCCGTCGCCGCGCCCGACGTGGTACGGATGG

CGACCGCCGGCTCCGCCGCCGCCGTCGACCTCCCCGGCGGCGCGCTCGAA

GTCGGCGGCGCGGCCGACCTCGCCGTCGTCGACCTCGACGCGCCGCATCT

CACGCCCGCGAACGACCTCGTGAGCCACCTCGCGTACGCCGCCCGCGGCT

CCGACGTTCGCCACACGGTCTGTGACGGGCGGGTGCTGATGCGGGACCGC

GAGGTGCTGACGCTCGACGAGGACGCCGTGATGGCCCGCGCCCGCGAGGC

GGTCGCGTCGCTCCGCGAGCGCGTCTGAACCCCCCGTCCGGCCCGCCGCC

GCCTCCGACTCGCCGCCGTCTGGTCCGGTCGCACCGCCCTCCAGCCTGCT

ACCCGGCCAGCTCGATGCGCAGTCCGTCGTCGGTCACGCTGACCTCCATG

CGCTCGAACGTCCGGTGGTACTGCGCGACGTGTCGGCCGTCGCTGTGGTC

GACCATGATGGACTTCTCGAACAGCGACGCCTCGTGAAGTCGCTTGACCT

CCCGGTAGGCCGTCGACAGCGGCACGTCGGCCCGACAGCTCAGCTCCTTG

ACCGTCAGCGGCTCGTCGGCGACGCGGAGGAGGTCCGGAACTGCGGGGGC

GCTCATCAGGGCGAACAACTCCTGTTCGGCGAGCGTCTTGCCGTTATCTA

TGACGAGCATCGGCCCTCGGCGGTACAATCTCACGGTTGACACTTGTAGA

TTCGGGGGCGGCGACCGCCTTCGGTAGGGTTTTCGGACCCCTCTCCGAAC

CCACGGGTATGAGCGAACACTACGCTCCCGTCTCCGAGCACCTCGACGAC

GTCGAGGCCGCCCGGACGGAGGGACGACGCAAGATGGACTGGGCGCTCCA

GCACATGCCCATCCTGCAGGAGCTCCGCGAGCAGTTCGAGTCGGAACAGC

CGCTCGCCGGCGAGGTCGTCGGCATGGCGATGCACGTCGAGGCGAAGACG

GCGAACCTCGTCGAACTGCTGGCGCTCGGCGGCGCGGAAGTCGCCATCAC

CGGCTGTAACCCGCTTTCGACCCACGACGACGTGTCGGCGGCGCTCGACG

CCAACGACGACATCACCTCCTACGCCGTCCGCGGCGTCGACGAGGAGGGC

TACTACGCGGCCATCGACGCCGTCATCGCCCACGAACCGACCGTCACGGT

CGACGACGGCATGGACATGGTCTTCACCATCCACGAGGAGTACCCCGAAC

TCATCGAGACCATCGTCGGCGGGGCCGAAGAGACCACGACCGGCGTCCAC

CGACTCCGCGCGATGGACGAGGACGGCGAACTGAACTACCCCGTCTTCGC

CGTCAACGACACGCCGATGAAGCGCCTGTTCGACAACGTCCACGGCACGG

GCGAGTCGTCTTTGGCCACCATCGCCATGACGACGAACCTCTCGTACGCC

GGCAAGAACGTCGTCGTCGGCGGCTACGGCTACTGCGGCAAGGGCGTCGC

CAAGAAGGCGTCCGGCCAGAACGCGAACGTCATCGTCACCGAGGTCGACC

CCCGGCGCGCGCTCGAAGCCCACATGGAGGGCTACGACGTGATGCCGATG

GAGGAGGCCGCGAAGGTCGGCGACGTGTTCATCACGACCACCGGCAACCG

CGACGTCATCACCCGCGAGGACTTCGAGAACATGAAAGACGGCGTCCTGC

TCGCCAACGCCGGCCACTTCGACATCGAAATCGACCTCGACGCGCTGTCG

GACCTCGCGGTCGACGAGTACGAGGCCCGCGACGGCGTCGACGCCTACGA

ACTCGAAGACGGCCGCCGCCTGAACGTCCTCGCCGAGGGCCGCCTCGTCA

ACCTCGCGTCGCCCATCGCGCTCGGCCACCCGGTCGAGGTCATGGACCAG

TCGTTCGGCGTGCAGGCCGTCGTCGTCCGCGAACTCGTCGAAAACGGCGA

CGACTACGACGCCGGCGTCCACGACGTGCCCGACGAACTGGACCGCGAAG

TCGCCGAAATCAAGCTCGACGCCGAGGGCATCGAGTTCGACTCGATGACC

GACGAACAGCGCGAGTACATGGGTAGCTGGGCCCACGGGACGTGAGCCGA

CCCGCGCTCTCTCTCGTTTCTCACAGCCGCCGCGCGTCAGCGACCGCGTT

CGGCCCACTCGCCGGCGTTAGTGCGCGGCCATCTCCGAGAGGACGTTGAC

GCAGTAAATCCCGGCGAGGATGAGAAGCAGGCCGACGACGCCCGTCAGGT

CGACCGGTTCGTCGAACGCGACGACGCCGATGGCCGCGACGCCGACGATG

CCCAGCGCGGCCCACGTCCCGTACACCACGCCGATGGGGAGTTCTTCCAG

CGTCAGCGAGACGAGGTAGAACGCGAGGCCGTAGCCGGCCACCACGCCGA

GGCTCGGGAGAGGCTTCGAAAACCCTGCGGACAGTTTGAGCGCGGTCGTT

CCGACGAGTTCCGACGCGATTGCGCCGGCGAGTAACACGTACGGGTTCAT

GTCCGTCGGTCCCGCCACGACCCGGATGAGCGTTCTGCAATCGCCCGCCA

CTTGCGGTCCGTTGCTCCCCGGTCAACAGGAACTATCAACTTACTAACCA

CCGGGCGACGGACACCTCCCCATGACCGAACTCGGGAACTAGGCCTGTCG

ACCTACGAGGAGAAGGCGTACCGGACGCTCCTCGCCACGGGAGCCGCGAC

GGCCGCGACCGTCTCGGACGCGAGCGGCGTCCCGAACGGCCGGGTCTACG

ACGTGCTCAACGGGCTTCGGTCGCGCCGACTGGTCCGCGCGCAGTCGACC

CAGCCGACGCGGTACGCCGCCGTCGACCCGGGCGCGGCGGTCGAGCGACT

GCTGGCCGAGCGCGCGGCGGAACTGCGCGAGGAGTGGACGCGATACCGCG

ACGTGGCCGACGCGGTCCGGTCGAACCTCCTGCCGACGCCGCCGGCGGAC

GGGAGCGTCTGGCTCGGCCGCCTCGGCGGCGACGAGATGCGGACGGCGAT

GCACGAACACGTCCGGGCGGCGACCGAGTCCGTCTCCGCCGCGGTCGGCC

CGCCGTACGAGCGGGCGTCGTGGGAGACGCTCAGAACCGAGTTCGACGCG

TTCTTCGAGGGCGCTCGGGACGACCTCGGCGTGTCGCTGCTCCTCAGCGA

CCCCGTGCTCGACTCGCTTCCCGACGAGTTTCGGGGCCTCGTGGCGTCGA

AGCCCCAGACGGTTCGAATCCGCGTCCTGCCGCACCTGCCGGTCTCGTTC

GACGTCGTCGACGAATCGGTCGCGTCGGTCGACATCCCGCACCCGCAGTC

CGCCGCCGACCGCCTCGGCGTCGTCGTGGTGACGGACGCGGGCGTCGTCG

ACGAGTTCGACCGCCAGTTCCGGGCGCTGTGGGGGGACGCCGTCCCGCTT

TTCGAGTGACGCCGCGCATCTCGTCTGCGCTCCCGGACGCCGGTCGCCCC

ATTTATCACCGCCCGCGGCCGACTCCCGACCATGTCTTCGAACAGAAAGG

GAGACCGACGCGAGCGCGAACTCGTCAACGCTCTCGACGAGGCCGGCTTC

GCCGTCATGCGCGCGCCCGCCTCCGGGAGCGCGACGACGCGCGAACTCCC

CGACGTGCTCGCGGGCAACGGCGAGGTGTTCTACGCCATCGAGGCGAAGG

CCTCCAGCGGCCGCCCCATCTACCTGAGCGGCGAGGAGGTCGAAGCGCTC

GTCTACTTCTCGCGGAACTTCGGCGCGAAGGCTCGCATCGCCGTCCGGTT

CGACCGCGAGGACTGGTACTTCTTCCACCCCGGCGACCTCTACGTGACCG

ACGGCGGGAACTACCGCGTCAAAAAGGAGACGGCGCTGGCGGAGGGCGAG

GACTTCGAGTCGTTCACCGGCGGCCCGACGCAGACCAAACTCGGCGGCGA

CTGAGGTCGCGACGCGATTTTCCGCGCTCGCTCACGCCCGAGCGAGCCGC

TCGCCGAGCGAGTTCGCGCCCACGAAGCCGCGCTCGACGCGGCTGAGCCG

CGAGGCGGGGAACCGGTAGGCGTGCAGTTCACCGAGGTCGGAGAGACCGG

CGTTCACCGGCAGCGAGTCGAGATAGACCGCCTCGAACGTCGGTTCGTGG

TCGCCGTAGTCGGCGTTCGTCTCGTACGCCGAGACGAGGGTTCCCGTCTC

CGTCGGCGTCTCGTCGGCGCGGGTGCCGAGCGCCCGCGTGACGACGAGGA

GGCCGCCGCTCTCGCGGTCGCGGACGAGGTCGCCGGGGCGGTGGTCGTGG

TCGCGACAGAGCTGCGGCCCCGTCGCGTCCATCGGAACGAACGTCTCGTC

GCCGCAGACGGCGCAGGTGGCGCGCCGGCGGCCCGGCGGCGGGACCTCGC

CGCGGGTGATTTCGATGGCGACCCGGCGGAGGTGCTTACAGCGGGCGTGC

CGGATGGCGTTGTCGGGACAGGTGCAGGTGCGCGCCTCCACGTCGACGAC

GTAGGTGCCGCCGTCGGTCTCGACGACGTAGCGACCGTCTCGGAGCGGCC

GGACGGCCATCGGTTCGACGCGGGCGCGGCGGGCGCGCCCGGACAGTCCG

TCCGACGGAAGCGTTGTCTTGCGGCGCACCGGGCGGCGAGTCGACGCGGG

TGTGTGTGCGATGTGCGTCATGGTGGTGCGGGATTCCGACCCTCCCAGCG

GCCGTTCGGGGTCGGTTTCCGTCACCTGCGTGTAGGTTCTCGAACGACCT

AAACCCTCGTTTCGTGTGGAAAATCGGGCGCGTGAGTGGCTCTCGTCGGG

CATCCGCTCATCGAAGACCTTTTAGAACGGCCGCGGATACGTCGTCGCAT

GGCTATCGAAGCGGAGATGCGGCGGAAGATTGCCGTCTCCATCGTCGCGG

TCGGGGTGTTTATCGCCCTCATCGTCGGCATCGGTGCGACCTACAACCAG

AGTGGACTCGTCTCCACCGGCGGGCTCGCACTCGTCGGCGCTATCACGGC

GTTCGTCCTCGTGATGGCTGGTATCGGCGTTTGGCTGTCGCGTTCTTCCT

AATCCGGCTGGTCGGTTTCGACGACGGGTTCGCCGTCGCGCGGGCGCTCG

ACACGCAGCGGCGGCGCTCGCGGGTCGGCTACGTCGTCAGCAAGGACTCC

GTCAGTCGGCGGCTTCGTCCGCGGCGTCCGCGTCCGCGTCGTTCGTCGCC

GTCTCGAAGTAGTTCATCGGGTGCTTGATGGTGTCACAGCGGTCGTCGCG

GTTCACACAGAGGTCGTACGCCTGCATCGTCTCGCAGGTCGGCGGCGCGT

ACTGCGACCCGCTCGAATCCGCGAGGACGGTCGCCCGCTTCTGCAACGCC

TCGGCGGTTTCGTCGCCGACGACGCCCCCGACCTCATCGGGGTCGAGGCC

GACGCCGACGAGGAACGCCGCGAGCGCGAACGCCGCCTCGTTCGGGAGCG

ACTCGCCCTCTCGCGCCCGCGCCAACAGCGCCTCGACGCAGGGCGGGAAG

TGGTCGGGGTCCACCGCGTCGACGTAGACGACGTTGACCGCGCCGCGGTC

CGAAAGCAGGTTCCGCAGGCGCTCGACGTGGGGTTTGAGCGCGTCGGCGA

GTCGGTCGCCCGCGTCGCTGCCGCGGACGGCGAAGGGAAGCCCCTCTGCG

ACCCGGCGGCGGACCGCCTCGCGGAGCAGTTCCGAGAGCTCCTCGCGGGT

GACGCGGACCGCGCCGTCGGCCAGCGAGCGGTTGACGAGCCGCCAGTCCG

CGCCCCAGTCGGTGTCGGAGTAGGTGAGGTACGGTCCCACGTCGACCCAG

TAGTTGTCGGGCGCGCGTCGGCTGTTCCGGTTCCGGTTCGACTCGGCCCG

CACCGCGCCGTCGAGGTCGAACTCGCGGAGGAAGTCGTCGAGGCTCGCGC

GGGCCTCACCGTGGACGTCGTCGTCGCCGGCGTCGAAGTCGGCGAGCATG

CGCTCGTAGGACGTGTCGGCCTCGGCGCGGGCGTACTTGTCCACCGCCGC

GGGCGTCTCGACGAGCGAGACGATGATGCGCGAGATGGGGTACGAAAGCA

GTTCCGTCTTGGTCTCCCGCGGGTAGGCCTCGCCGGGGTCGGGTTCGACG

GTGCCCTCCATGAGGGCGCGTTCGACGCGCTCGACGCCACGCTCGACGGC

CGGGGCGTCCTCTGCGACGAGCGTCGCGATGGCGACGTCCGCCGATTCGA

CCGCCTCTCTGGCCGCGGCGAAAAACGGGTATCGGGCGTGGAGCGGGCGC

ATCCGCATACCGCCACGTTCCGGGTGCCCACCGATAAACGCGACGGATTC

GGCGGTGGCGGCGTGGGCCGTGCGGCCGAGTCTCGCACCCGCTCCGCCGC

GACGACGGCGCATCACACAACCTATTACCGACCACTTCCTCAGGCTCTCA

CGTGCCGCACTTCGATTACCCGTGTCCGGACTGCCGCGCCACGACCAGTC

TCCACGACGCCGACTGCCGCTTCGAGGGGACCCCGTGGGTCGAAGTCGAG

CGGGCCTACGTCGACATCGTCTCCGTTCTCGCGGGCGGCCCGTGCGACGA

GGAGACGCTCCGCCGCGAAGCGCCGGGCGAGTGGGGGCCGCTCCAACAGG

CCGCGCTCCGCCGACTCAAGCGCGACGAGCGGGTGTCGGACGCGAATTCG

GGCGTCCTCCGACTGCGCACCGCCGAGGAGTTCAGAGAGGAGGTCTCGGA

GCCGACCCGGGAGCCGATGCGGACGCTCCACCAGTACGGGAGCGTCCCCG

GCTGTCACGACAACGCCGTCTTCGCCATGATAGCGTGGTACGAGATGGTC

GGCCTGTCGTGGCCCGAGACGCGCGAAAACGTCGTCAACTGGCTCCGCGA

CACCGGCGCGTGGGACCGCGGCGGCTTCGAGGAGGCGACGCCCGCCGAAC

TCGTCGAGAAGAAGCGCCACGTCTACGAGGCGGGCTACGGCTGGAAGGAA

AAGGCCGTCTCGGCCAAGCGCGTCATCGACCGCTACCGCTCTTGAGTCGA

CGCCGCGACCGAACCGCGTCATCGCGGCCGACGAGTCGGGCGGCGCGGTC

GCTGACCGGTGAGAATCGAGAGAAACGAGTGGGACGGCTCGGTGGTCGGA

CAACGACGCGGCCGCCGAAACGTCCGCTCAGTCGCTCTGGATGCGCGGGG

CGAGCATGTACGTGATGGTGCCCATCCCCTCAGCGATTTGGTAGTGAAGC

TTGACCGGGAACTCCTCGCCGAGTTCGACGGTGACCTCGGCGTCGGTCGG

AATCGCCTTGTTCATGTCTTTGAGATAATCGAGCGAGAACAGCGAGTCGG

CCGCGCCGGCTTCGATGCTGATGAGGTCCGCCGGGGGAAGCGACAGGTCC

ACGTCGTCGGTGTCGCCTTCGGCCTCGATGTGGAACGTCTCTTCGGCGCC

GTCGACGCGGAGGCGGATGTGGTCCGAAACCATGTCGGCCGCCTTGATAC

CGCGGTCGAGGTGCGTCCCTTCGAGGACGATGTTCGCTGCGAGGTCGAGG

TCCGGAATGTCGGGCTCCTGGCGAATCGAGTCGGGGTCGATGAGCGCGAG

CGTGTACGACAGCCCGTCGATGCGGATGTTGAGCTTGCGCGTCTCCTCGT

CGAGCGTGAGGTGGATGAGGTCGCCCGCGCCCGCCATCCCGGCGACCTCT

TCGAGGCGCGAGAGGTTGACACCGATGACGCCGCCGTGGGCTTCGTAGGA

TTCGAACGCCGCCGCGTCGAGCGTGAGGTCCACCATGCCGACGTTCGCGG

GGTCGACGGCGCGGATGGAGAGGCTCTCCTCGTTGAGTCGAATCTTACAC

TCGTCGACGAGGACGCTCACGGAGTCGAGCGCGTCCCGGAGCGTCGCGGC

GCTCACGATGGCCTTGAACATATGTGCCGTCGTACGACAGCGGTCTTAAA

AATACTCCTGATTTGGACTCATCGCAATATGATGTTCGCACGCTCTCCCG

CGTGCGCGCTCCCGCGAGCACCCCACCGTTCCCCTCGGCTCTCCATGGGT

GTTGTTGTCACTGTCTTCGTACATAGCTAACCCGACGATTTCGAGGGTTT

CGGGCTTCAAAGCACTCAAATAGCTGATTGTGTAACTCCAGGCCATGGCG

AACGAGGAGACGAACCCAGCCGCCCGCCGACGAGGTGCCGAGCGATGACC

GACGCCGCCGATGTCGATGTTGACGCCGACACCGACGCGACTACGACCCG

CGTCGCCTTCGTCTGCGTCCAAAACGCCGGCCGGAGCCAGATGGCGACGG

CCTTCGCCCGCCGGGAGCGCGACGAGCGCGGCGTCGGCGACCGCATCGAG

GTCGTCACCGGCGGCACCGACCCCGCCGACCACGTCCACGACGAGGTCGT

CGAGGTCATGGGCGAGAAGGGGTTCGACCTCGCCGACGAGACGCCCCGCG

CAATCGAGCAGGACGAGATTATGAACGTGGATATCGTCGTCACGATGGGT

TGTTCGGCCGAGGGCATCTGTCCCATGACGTGGCGCGGCGACGCCCGCGA

CTGGGACCTCGACGACCCGGACGGCCGGGACCTCGATGCGGTCCGGGCGA

TTCGCGACGACATCGAGGGGCGCGTTTCGGCGCTGTTCGACGAACTCGCG

GGTTAGCCGAACAGCCGCTCTCGGAGCGACCGTCGCCGGGGGCGCTCGGC

CAGCAACACGGTCGTCTCGAGGTCGTCGAGCACGGACAGCGCCAGCGACC

CGCCGACGAGTCGGGACAGCAGGCCGGTCTCCGTCGCGCCGACGAGGACG

AGCGACTTGTCCGCCGCCGCCTCGCCGATGACGGTCTCCACGTCGCCCGT

CTCGACCAGTTGCTCCACTCCGTGTAGGTCGTGTTCCGCGACCCACTGTT

CGAGGAACCCCCGACCGGCCGCGACCTCGTCAGCGACGTGCAGCACCGAG

ATGTGCGCGTCGGTCGTCGCTTGCAGCGCTCGTGCGACCTCCGCGGAGAG

GTCAGACGAGTGCCCGCCCGCCGTCGGGAGCAGCACCTCGCTCGGGTCGA

ACTCCTCCCCGTCGAGGACGACCACGTCGCACGGGAGGTCGTGCGTCAGT

TCGTCGAGCGCGCCCTCGACCCGTCCGCCGGCGAGTTTCGCGCCGCTGTG

GCCCATGATGACCCGGTCGACGCCGTGTTGGCGCGCCGCGTCGAACACCT

CCGCGAGCCCCTCGTGGGAGAGAATCGTCCGCGTGTCGACCGGCACGTCC

AACTGTTCGACGTCGGCTCGCGCGTCGGCCATGAGCCGTTCCGACGCCTT

CGAGATGCGGTCGCGTTGGTCGTCGGCGGCCGCCAGCGAGGTTTGGTCCG

GGACCTGAATGACGTGGGTCGCGAGGACCCGCCCGCCGTCGAACTTCGCG

AGCGCGGCCGCGAGCGTCACGAGCGCCCGCTCGGTTCGGGGGTTCGAAAC

CGCGACCATCGTCGTCGTCGCGGGCGCGTCGTCGATGCGACCCGACCCGT

TCGGCGCGACCGTCGCCGCGGCGTCGACGACCGTGTTCGGTAGCTCGTCG

CCCCGAGAGAGGATGTGCCGACCGAGAAGCCCCTGTCTGTCGGCGTTGTT

GCGTGCGTACACGAAGTACCAGACCACGGCACCGACGACGAACAGCCCCG

AAAGCAGGAGCTCTTCCCCGCCGACGAACGCCAGCAGTCCGAGCGAGAGC

GCCATGCCGACGAGCGGCGTAACCGGGTACAGCGGCACGGTGAACGCCGG

GTCGTACTCCGGGTCAGTCTCCCGGAAGACGATGAGCGCCGCGTTCATGA

GCGCGTAGACGACGAGGTGGAGGACGCTCGCGGCTTTCGCCAACACCTCG

ATGTCTCGGCCGAGCGCCGCGATGAAGAGGATGATGACGGCACCCGTGAC

GAGAATCGACCGGTAGGGCGTCGCGTAGTTCGGGTGAATCTCGTTGAGCC

AGTTGGTGACGACTCGGTCCCGGCCCATCGCGAAGTTGATTCGGGCCGAC

GCCAGAATCGACGCGTTCGCGCTGGAGACGGTCGCGAGGAGCGCGCCCAG

CGTCACGACGGTGACGGCGAGGCCGGCCAGCCCGCCGGGGAACGCGAGTT

GCGTCGCCTGCGTCAGCGGCGCGCTCTGGCTCAGGTCCGGCCACGGCACC

ACGCCGAGCATGATGCTCACGAGAATCGCGTAGAGGACGGTGACGATGGC

GACGCTCCCGATAACCGCCAGCGGGAGGTTCCGGCCGGGGTTCTTCAGCT

CCTCGGCGACGGTCGCAATCTTCGCGTAGCCGAGAAACGAGACGAACACG

AGCGCCGTCCCCGGCAGGATGGCACCGTAGCCGAACGGCGCGATACCGCC

GTCACCGACGAGCGTTGCGACGTCGAACGACAGCCAGCCCTGGACCGCGA

ACAGGGCCAGAATCGCCAACAGGACGGTGACGATGACCGTCTGTACGCCG

CCGGTCTCCTTGGCACCGATGTAGTTGACGCCCACGAAGACGGCCCCGGC

GACGAGCGCGCCGACTTGCACCTCGTTGAGAAAGAGGAACTCCGGCATCG

GCACGAGCGTCGCGAGGTACTGCCCGAAGCCGATGCTGTAAAACGCGGAG

GCGAACGCCAGCCCCATCCAGTCGCCGAGGCCGGCGATAGAGCCGAACAG

CGGCCCGAGCGCGCGGTTGACGTAGTAGCCGCCGCCGGCTTTCGGCATCG

CGGTGCCGAGTTCGGAGACCGAAAGCGCGTTCACCAGCGCGATGAGCCCC

CCGACGACGAACGAGGCGACGACGACCGGGCCGGCGGTGCTGGCCGCGAC

GCCGGGTAGGACGAAGATGCCCGCGCCGATCATCGTCCCGATGCCGATAG

TCAGCGCCGACAGGAGGCCGAGGTCCTTCGCGAGTTCCTCGTCGCTCATC

CGTTGGCCTCCGTGCTGGGACCCTCGCTCCGTCGCTTCGGAACCCGCTGT

TCGGGTCGGAGCGCCGCGCTGTCGGCCGTCTCGGTGACGTTCGTCTCGTG

CATAGTCGCTACCCGGTTGTGTAACGATATATGTTCTACCCTTTCACTTC

GAATTCGAAAATAGAAATGAATATAATTTCTAAGAACGAAATCCTCGTGA

GTCATCCGTCTCCGGGCTGGCTCGTCTTCGCGGTCCCTCACGGGCCGGTA

CGGGGCCGTCCTCACCTCGCGGCTCCTCGGTCGAGCAGTTCCGTCTCGCA

AGACGGGCGTCGTCCAGACAAGCCTTATATGAAAACGCGGGGTAGCTAAC

TTCGCAAACCAAAACGCGACACAGTCACCGATGCCATATCGCAACACCGA

CCACCGGCTCGACGAGATTGACCGCCGCATCCTCTACGCCCTGATGGACG

ATGCGCGTAACACGTCCGCCAGCACGCTCGCGGCGGAGGCCGGCGTCTCC

GGAGCCACTATCCGGAACCGAATCCACAAGCTCGAAGACTCCGGCATCAT

CCGCGGCAGCACCGCGCAGGTCGATTTCGAACTCGCCGGCGGGAAGCTCA

CGAACCTCTATCTCTGCGACGTTCCCGTGACCGAACGAGAGGCGCTCGCC

CACGAGGCGCGGGCGATTCCGGGCGTCATCAACGTCCGGACGCTGATGAC

CGGTCGCCGCAACCTCCACGTCCTCGCGGTCGGCGAGAGCACCGGCGACC

TCCGGCGCGTCGCGCGGTTGCTCACCGACATCGGCATCCACATCGAAGAC

GAAGACCTGCTCGAAGAGGAGCTGTTCGCCCCCTACGGCCCGTTCGACCC

CGACGACGGCGGTCACGCGCCCGAAGCCAACGATTTCATCAGCCTCACCG

GCGACGCGAGCGTCGTCGAGGTCCCCGTGCAGTCCGACGCGCCGATAGCC

AGCCTCACGCTCGAAGACGCCTCCGAGCGGGGGATTCTCGACGACGAGAC

GCTCGTCATCGCCATCGAGCGGGGCGACCGCGAACTCACCCCGCACGGGG

ACACGGTCATCGAGCCGGACGACATCGTGACGGTTCTCTCCCGCGCCGGC

GGGGACGCGGATGCGGACGCGCTGTCGGCGTTCCGCGGTCCACAGACGGA

GTCGTCTCCGGGCTGACTTCGGCGGTGTCGCGTCCGCGGTCCCCGCGCTG

GCAGCCGCGCCGCCCCGTCTCCGGGCGCTATCCGATGTGCCTTTCCGGGC

TGACCACGTCACACCCGATATGAACGCGAATTCGCCGGCTGTTCCCACGC

GGAACATCACGGGCAGTTCAACGAGGTAAGCATGGCACGGAAAGACCACT

ACTACAACAAGGCCAAACAGGAGGGCTACCGCGCCCGCTCGGCCTACAAG

CTGAAGCAACTCGACGGCGACGCGGGTCTGTTCGGCCCCGGCAACACCGT

CGTCGACCTCGGTGCCGCCCCCGGCGGGTGGCTCCAGGTCGTCTCCGAAG

AAGTCGGTGACCACGGCAAGGTCGTCGGCGTCGACCTCCAGCGAATCCGC

GGCATCGACCGCGACAACGTCCAGACGATTCGCGGCGACATGACCGAAGA

CGAGACGAAGGAGGAACTGACGGCCGTCATCGGTGAGCGCGGAGCCGACG

CCGTCGTCTCCGACATGGCACCGAACATGACCGGCGAGTACTCGCTGGAC

CACGCCCGCTCCGTCTACCTCGCCCGACAGGCGTTCGAGGTCGCACAGGA

GCTGTTGGCGACCGGCGGCGACTTCGCCGTGAAGGTGTTCGACGGCCCCG

ACGTGGCGGACCTCCGCGCCGACATGGAACGGGAGTTCCAGTACGTTCGG

TCGATGCGCCCCGACGCCTCCCGCGATAGCTCCTCCGAGCAGTACCTCGT

CGGCAAGCACTTCCTCACCGCCCCGGTTCGGAAGGGCGACGAACTCGACG

TGGAAATCGTCGACGTGGGGAGCGAGGGCGACGGCATCGCCAAAGTCGAG

GAGTTCACGCTGTTCGTCTCCGGGACGGAGGCGGGCGACATGCCGACGGT

CCGCGTCACCGACGTGAAACCGCGGTTCGCGTTCGCTGAACCAATCGACG

ACGAAGACTGAGCGCGCCGACCGCACGCCGATTCCCGCGTCGAGTCCGGC

GCGTCCCGTGCCCGCCCGCGACCGCGCCGACGGCTCCGCGCCCGCCGCAA

TTTGTGTCTAACTTCGGGCAACCATTATCCGTGTTTGACACGACTCCGCT

CGTGTCACCCATGTCAGCAGATACGACGACCACCGAGTCCCAACCGTTGT

TCACCGGCCTCCCGTCGGGTATCGTTCCGTACGTCGCCATCCTCGGGGCA

TTGGCGTCCGTGTACGCCCACCTCTCTCTCGCGCCGGTGTTGATGCAGTT

CGACCAGACGCAGGCGATTCTGTTCGTCCTCGCGGGCGTCGGCTTCCTCG

CGGGCATCGCCGTCTACCTGAGCAAGTTCTGGCGGCGCGAGTTCTACCTC

GTCGCCATCGCGTTCGCGCTCGCTCAAATCGCGGCGTGGGCCGTCATGAG

CGGGCGAGTCAGCGAGATGGCGATTCTCTCGAAGGGCGGCGAGGCCGTCT

TCAGCGCCGCGGCGGCGTACCTCTATCTGAGCGACTCGCCCGAGACCGAC

GGGGCCGCCTGAGCCACGTCCCGCGTTCGACCCGTCGCCCCGCCGTCTCG

ACGTTCTGGCGGTCCGCCGCCCCGTCGCATCTTTTCACTCAACGACCACG

AGGTCGAGTCGGAGCCCCGTCCGGCAGCCCTTCCAACCGCCGCGACAGGC

CCCGGTGGCGAGGATGTCGCCGAACGACGTGTAGGTCCCGCAGTTCGGAC

AGCGAATCCCCATCTCCATCCCGTCTCGCTCGGTGAGCCTGTGGTGGTGC

TCGTCGGCCATACATCGTGTGGTATGCTACCAAAAGAGATAAGCCGTCCG

TCGCCCCGGCCGGCGTCTCAGTCCGCGACCCAGCCGTCGGTCGAGGACTC

GTCGTCGCCGCGGAGGAGCGCGACGGTCCCGTAGACGAACGGGGTGTCCA

CGATTGCGATGAGGAGCTTCAGGAGGTACTGGCCGACGGCGAGGCCGACG

AGGACGTTCCACGGGAGCACGTCTCCGATGCCGAGGACCGTCGGCGCGGC

GTAGAACCCGACGCCGACGAAGATGACCGTGTCGATGGCCTGACTCGTCG

CCGTCGAGACGATGTTGCGGAGCCAGAGAAGCGAGCCGTCGGTCAGCTCG

CGGATGCCGTGGAAGACGACGACGTCCCAGTTCTGACTCACGAGGTACGC

GAGCAGGCTCCCGGCGACGACGTTCGTCCCCGACGAGAGCACCGTGGCGA

ACTGCGCGGCGAACTCGGGGTTCGCGGCGGGCGCGGCGATGGTGCTCCAG

ACGAGGCCGAGGAGGACGAAGTTCATGGCGAAGCCGACGTTCACCATGAC

CTGCGCCGCCCGGCGGCCGTACAGTTCGGAGTAACAGTCGGACGCGAAGA

AGGTGAGCGCGTACGCCAGCGCCGCTCCCGGCATGAGGATGGCCGCGCCG

ACGAACGGGAGTTCGCCCACCGCCGACGGCAGGGGAATCGAGAGCAGTTT

CGACGCCGTCAACTGCGCCGTCGTCAGCGCGGTGACGAACAGGCCGACGA

GGGCGACTTGTCCGATGGCCGAGCGCCCCTCACTCATCTTCGACGAGGCG

GTTGTGTCGGTCGTCGATTTCGTCGAGGATGTCGAGCGTCTTCCGGATGG

ACGCCCGAACCGCATCGCTTCGGTTCACGAACTTCTTGTCGTCGCCGACG

TGCTCGTCTAAGTCCGCGAGCAGTTCGTCGGGTATCTCGACGCTTATCTT

GGCCATACTCGGGGATTCTCATGAGAATACTTAACGCCTTTTCAACGGCC

TCAGCGGCGATATCTGGCGCGTCTTCGCGTCGTCGAGTCGACGGGTTCGT

GACGCCCCTCGCGGGTGCGCCGTGCTCACTCCGCCGCGCCGCGGGCGGGC

GAGAGGTCGCGCCGGCCGCCGACCGTCAGCACGAACAGGACGCCGAGACC

GACGCCGTAGGCCGCCATCAGCGGGAGCGTCACCATGAACATCGTCGTGA

TGCTCGCGGGGGTGAACACCGCCGAGATGGCGAGGATGAACACCGTGACC

TCGCGCCAGCGATTCCGCATCATCCGGTAGCTGATGCCGGCGGTGTTGAG

AAGCACCATCAGGATGGGCACGTCGGCGAGGAGGCCGATGCCCGCGGTGG

TGAAGAAGATGAGCCAGAAGAAGTTGGTGATGCGGTAGGTGATTATCATG

TTCGCCGCGAGCGCGTCTTCGACGAGGAACGTGATGACCGTCGGCGCGAC

GTAGGTGTAGCCGAGCGCGAAGCCGCCGAGGAGGCCGCCGGCGAGCGCGC

CGGTCCACACGAAGACCGTCCGGCGACGCTTGCGGATGATGTTGCGCTCG

CGGAGCGCGGGCCACACGAAGTACGCGACGAGCGGGAGCGTCGCCAGCAC

GGCCAGAATCGTCGAGAACTTCACCTCGAAGATGAGCGCCTCCATCGGGT

GGAGCGCGACGACGTTGAGGACTTCCTCGGGTCGCACGGCGGCTGGCAGT

CGGCCGAGGAAGTCGTCGTACACGTCCCTGATACCGCCGGTGTAGAGCCA

GCCGAACGTCGTCGCGAGGACGAGCATGAACCAGCCGACGACCCAGAACG

CCCGCGAGGTGAGCGAGTCGACGATGAACGCGATGTCGGTGTAGTAGCCG

CCGATGTCGTCCTCGTCGGTCTCGCCTTCGGTCAGTTCGGAGACGAACGC

GCCGCCCGCGCGGGTCGTCCGGTCTTCGAGTTCGCCCGGTTCGTCTTCGG

CGTCGGCCGCTTCGGCCTCGCGGTCCGCCTCGGCCTCGTCGAATCGGTCG

ATGAGCGCCTGCGCTTTGGCCTTGTCGCCGTCGTCGATGGCGGCCGACGC

GAGGGCCATCACCTCGTCTTCTTCGAGGTCTGCGAACGCCTCCGGCGGCG

CGGCGCGGACGCCCGCCACGTCGAGCGCCGAGAGGTCGAGTTTCGTCGGG

TCACCGACGCCGATTTCGGTCCGTTCGAGTCGCTCGATGTCGCGGTAGAC

GAGGTAGGCGAGTCCGAACGCGAGGCCGACGAAGCCGCCGGCGACGACGA

ACGCGCCCAACGCGACCCCCGAACCGGGAGCGAGGAACACGTAGTCGCTG

CCGATTGCGGCGAGGCCGTCGTTCGCGAGTTCGACGCCGCCGTACTCGTA

GAAGGCGTAGACGAGGAGGCCGACGACGACGCCGACGCCCGCGAGGAGGT

TCCAGTGGGTTGTCGCGGTCGATTTGACGTCTATCTTCTCGCTGCCGCGT

TTGGCCGTGACGACCACGCGAGCGAGATAGAGGCTGAAGGCGTACAGCAA

GATGACGGGCACCGCCCACATGATTTGGGTGAACGGGTCCGGCGGGGTGA

ACAGTGCGCCGAAGGCGAAGATGCCGACGATGGCGTGCCGCCACTTGTCG

CGGAACAGCTCGTAGGGGACGACTTCGGCGTACGAGAGCCCGGTCATCGC

GAGCGGGAGTTGGCTCGCGAGGCCGAACGAAAGCGTGAGCAGGAAGATGA

ACTGCGCCCACTTCACGATGGAGTAACTCGGCGTGAAGCCGGCGGAAATC

GCGTTCTGCGCGAGGAACGCGAACGTGAACGGGAAGAAGACGAAGTAGCC

GTAGGCGACCCCCGCGGCGAACAGCGCGACCATCGTCAGTCCGATGAGCG

CGAGTTTCCACGGCGCGACGGGCGATTTAGGCCACGCGTCCCGTGCTTTC

AGCGCGCCCCGCGAGACGTAGATAAACGGCGGAAGCGCGAACAGCACCCC

CACGACGAGACCGATTTTGGCCTGCAAGAGAATCACGTCGAAGGGGGTCT

GGGCGATGATGCTGACGTTGCCGGAGACGCTGGCGTCCATCTGCGCCTTG

GTCACGCCACGGAAGAACTCCCAGACGTAGAGTCGGAGCGCGTAGAACGT

GCCGAGGAACCCGACGAGGAAGACGATAAAGACCTTCTGGAGGTCTTTTT

GCGCCGCTCGGAGCATCGCACCGGCGGTCTCGCGGCCCGCCGCGATGGTC

TGTTGGGTGTCCTCGTCGAGGGCGCTGGACATACGTACGTGGCGAAAGCA

GTCTGGTGGTTATCAATCTTTTTCACCGCGGGCGTGAGTTCGGCTCCCTC

GGCGCGCCTCCTCAGGGCGATGCGAAAAGACCTATAACTGGCCGGAAGCG

TAGGACACCTGAATGGCGGACGAGGAGCGTGACGCAGGGCTCTCTGCGGC

CGACGACGAGACGGACGCCTCGGACGACACCGACCAGCGCTCGTCCGACG

GCGACGCCGACGACGCCGACGACGCGTCTTCGTCGTCTGACGGTCCGGTT

TACGGTCGAGTCACCCCGCGAGACGAGACCGTCACCCATGGGTCTGACGA

CGACGCTTCTGCCGACGTTGCTGCCGAGACGGGCGACAACGGCGACGACT

CCGATTCCGACACCGACGCGGCTCCCGACGACGCGGATGATTCAGCTACC

GACTCCGACGCCGACTCTGACGACGAACCGCGTCTCCTCGCGGACGACGA

ACACACGTCGCACGTTCCCGAGGGGACATACGACGATTCGAGCGACGAGT

CGGCTGACGACGTCGACCCGGACGCGGCGGCCGACGGCGCGAGCCCGGCG

CTGACCGGCGAGGACGAGATGGGCGGTGTGGCCCCGTCGTCCGTCTCCGC

CGAGGACGCCGACTTCGACGACGAGGACGTCGGCGGCCTCGTCGGCGAGG

CCCCCGAGAGCGACCAGGAGATGCCGCTGACCGCGCACATCGAGGAGATG

ATTCGCCGGTTGGCGGTCGTCCTCGGCGTCGCCGGCGCGATTACGCTCGT

GCTGTTTCCCGGCGCGGACATCCTCAACGCGCTCGTGGACACGCAGGCCG

CCTTCGGCGTCCACATCCCGAGCGCGACCGACGTCATCAACTTCCTCTGG

AACTCCCACATCCCCGGCGCGGAGACCATCGTCGACCGCCGGCCGCGGCT

CTACGGCCCGCTCGAACTCATCCTCACCAAACTGAAGGTCGCCGGCCTCG

CCGGGACCGTCATCGGCCTCCCCGTGTTCGTCTACGAGACGTACCTGTTC

ATGCGCCCCGGTCTCTACCCCAAAGAGCGCAAGTACTACCTCGCGGCCGT

CCCGACGAGCCTCGTCCTCGCGCTCGTCGGCGTCCTCTTTGCCCACTTCG

TGGTGCTGCCGGCCATCTTCGCGTACTTCACCTCCTACACCGAGGGGACC

GCGGTCGTCGCGTTCGGCCTCAAGGAGACGTTCAACCTCATTCTCATCCT

GATGGGCTACATGGCGGTCGTCTTCCAGATTCCGCTGTTCGTGGAACTGG

CCATCATGATGAACCTCGTCACCCGACGGTGGCTCGAAGACCGCCGCCTG

CTGTTCTGGGGCGCGTTCCTCGGCCTCGCGTTCCTCGTCAGCCCCGACCC

GACCGGGATGGCCCCCATCATCATCGGCGCGACGATGATTACCCTGTTCG

AGGGGACGCTCGCGGCCTTACGCTGGACCGGGAACTGACCGGCGTCTCGT

TTTCCTCCCGTCGCTCCCTCGGCCGGGTCGTTCTGCCTCGCTTTCACCGA

CGGCCGCCCGTTCAGAAGACCTATGTCATAATAGTTGGAATCGTGACACG

ATGGCCGACCTCGTCGCCCTGTCTGCGGTCGCCCTCCCGCTCTGGTTCGC

CGTGAGCGTCTGGGCCGGCATCGACGCGACCAGACACAGTTCGCACAACG

CCTTCCTCTGGAGCCTCTCGGTGTTCGTCGGCGGGATTCTCGGCTTGGCG

CTGTACCTCAACCCCGGGCGCGACGAACCCGGGGTGGGCCGCCGCGCGGG

TCAGTCGCGGTCGCCGGACGGCATCTCTTCCGCCCGTACCGAGACCGTCT

CCTGTCCGAACTGCCACTCGCTGGAGGAAGCCGACCGGGATACCTGCCGT

TTCTGCGGCGAGTCGCTGTAACGCGCCGAATCTCCCCGAGACCGCGGGGA

ATCACACTGCGTCCGACGACTCCGTCGCCTTCCGATTCTCGGCCGCGACC

ACTCCCTCGACGCGCGTCTTCAGCCCCTCGTTCATGGCGACGAATCCCGC

CCGGGTTTGCTCGCCGACGAACCGCAACAGGAGTCCGGCGAGGACGCCCG

AGAACGTCTCCGCGTGTTCGAACCGGGTGCGCTCGCCGCCGTCGAGCGGT

TCGAGGCGGAACCGGTGTTCGCCGTCGAACAGTCCCGGAACCACGAGGTG

GCCGACCCAGCGCAGTTCGCGGTGCTTCTCGCAGTAGACTACGTCGGGTT

CGAACTCCGACTCGCGGCCGCCCGGCGGTCGCAGGTGGACGGTGAGCGTC

GCGCCCTCGTTCGGTCGCCCGGCGATTCGCATGAACGGGTTCCATTCGGG

GTAGCGGTCGAAGTCGACGAGCGCCTCCCACACCCGCTCGGGCGGGGCGT

CTATCTCGATGCTCGTGACGAGTTCGTGTGCCATACCTAACAGTTGGGCG

GCCGAGACCGTGAATCTCGCGCGGAACGTTCATTGCCGGTGAGGAGAGAG

ACGGACGTATGGCAACCCTCCTTCTCGCCAGACACGGCGAGACGACGTGG

AACCGCGCCGGGCGCGTACAGGGGTGGGTCCCTGTGTCGCTCACAGAGCG

CGGCCGCGAGCAGGCCGACGCCCTCGCGCGCCACGTCGCGGACAGCTACG

AGGTCGACCGCCTCGTCTCCTCCGACATCGAACGCGCCCAAGAGACCGCC

CGCCCCGTCGCCCGCGAACTCGGCCTCGAACCCGTCCTCGACTCGGCGTG

GCGCGAGCGCGACGTGGGGTCGTTTCAGGGGCTCGAATTCGACGAACTCA

CCGACCGGTACCCGCAGTACTTCCTCTCGGCGGTCGGCGCGCCCGCCGCC

CGCGAGCGCCCTCCGAGCGGCGAGAGCCTCGTCGAGGTCCGCCGCCGCGT

CCTCAACGCTCACGAGGGGCTCGCCGACTCGCTCGACGCCGACGAGACGG

TGCTCGTCGTCAGCCACGGCGCGCCGATTCGGCTGTCGCTCGGTGAGGTG

AAGGGCCTCGACATCGTCGAGACGATGCTGTCGCAACCGCTCGACAACGG

CGGCATCTGCGAGTTCGAAGTCGAACTGAGCGACGACGGCGACGAACCGC

TCGTCCACGTCGTCGCCGAGAACGAGACGCGATTTCTGACGACGTAGGTC

GGTCCGGCGGTCTGGTCCGAGTTTCTGACTGTTATCTCTCAGCTACTTCC

GCATCGGATAGTCCTGCGCGAACACGTCGTCCACGAGGAGGTCGTCGTCG

CCCGCGTCGGCGTCCGTCTCCGCCTCGGCCCCGGGGCTGACGCTCCCGGT

CCGGTAGCCGTGGAGGTCGAGCGTGACGTGGTCGAAGCCGGCGTCCTTCA

GGTGGTCGCGGGCGGCGCGGACGAAGTCGGCGTCGAGGGCGCGGTCGAGT

TCCTCCGGCGCGACCTCGATGCGGGCGAGGCCGTCGTGGTCGCGGACGCG

GAACTGCTCGAAGCCCCACGTCCGGAGAATCGTCTCCGCGCGCTCGACCC

GCGAGAGTCGTTCCTCGGTGACTTCGAGGCCGGTCGGAATCCGCGAGGAG

AGACAGGCCATCGAGGGCTTGTCTGCGACCGACAGGTCGTAGTCGTCGGC

GATGGCGCGGACCTCCTCTTTCGTGATGCCGTGGGCCAAAAGCGGCGACA

GCACCTCTAACTCCTCGACGGCGCGGAGGCCGGGGCGGTGACCCTCGCCG

GGGTCCGAGGCGTTGGTTCCGTCGCAGACGGCCTCGATGCCGCGGTCGCG

GGCGGCCTCGTACATCCGGCCGAGGCGCATCGTCCGGCAGTGGTAACACC

GGTCGTCGTCGTTGGCGACGAAGTCGGGGTTGTCCAACTCGGAGAACTCG

ACGGTCAGGTGTTCGATGCCGATTTCCTCGGCGACGCGCTTCGCGTCGTC

CAACTCGGCGTCCGGCAGGGTCTCGCTTTTCGCGGTGCAGGCGACCGCGT

CGTCACCGAGCGCCTCGTGGGCGAGTGCGGCGACCACGCTGGAGTCCACG

CCGCCCGAAAAGGCGATGAGGACGCTCTCGCGCTCCGCGAGCGCCTCGCG

TACTGCGTCGGCCTTCGCCGCGACGTCTCGCTCGCTCATGGCCTCCTTTC

CCGGCCGACGGGCAAAAGCCCCTCGCCCCCGGCGCGAACGACCGCGAAGG

GCGCACGCGCCGTCGAATCGGCCGCGACGGGCGCTCCCGCGTCGACGCGA

CCCGCCCGTCGTCCCGGAGTCACCACCCCGCGACTTTTTGTCCTATGCCG

CGATAGGTCCGCCCGAATGGACTTCGAGACCATCCCGGGCGTCGGCGAGA

AGACGGCCGCCGCGCTCGCGGAACTCGACGACGCCGAGCGAGCGCTCACC

GACGGCGACGTGGCGGCGCTCGCGCGGGCACCGGGACTCACGGAGGGGAG

GGCGGCGGCCATCGCCCGCGGCGCGATTCGCCGCGACCACGACGACCCCG

GCGGCTTCCTCGCCACCGACCGCGCCTCGGAAATCTACCGCGACGCGCTC

GGTCTCTTGCAGGCGCGGACGGTCACGACGTACGCCGAGAAGCGACTGGA

GACGCTGTTTCCCACCGGGTCGGCCCCGCGCATCGAGGAGGTCCGGGCGT

TCGCCGCCGACGCGACCGACCTCGACCCCGACCCGGACGTGCTCGCGGCG

CTGTCGGGCGTCGAACCCCTCGCGGACCCGACGCCGCGTCGCGTCCGCGA

GCGCTGTATCGCCACCGCCGACGCGGAGCGCTACGCCGCCGCCAAGGAGG

CGTTTCCCGAACTCTCAATCGAGGTCGTCGAAGACGGCCGCGACATCGCC

GAACTCGCGCGGTCGTACTCGACGGTGGTCGTGTTGGACGAGTCGTTCGC

CGGCATCGACGTGGACGGTGACGTGCGCGTCCTCCCCGACGCCGCCGAGC

GGACCGACGAGGTCGTCCCCGAGCGACTGCTGGCCTTCTTCGCCGCGAAC

CGCGAGGCCTTGGAGGCGGCCGCCGCGGTCCACGAAGTGGCCGAACTCGA

CCCGCCCTGCGACCTCGCCGCCCTCCGCGACGTCCTGTCCCGACTGGCGG

ACGACGGGACCGTCCTCGGAGACGACGAACTCGAACGCCTCTCGAACGCG

GTGGACGACCTCGACACCGCCGTCTCGACCGCCGAATCGGTCGCCAACGA

CCGCCTCCGCGACGTGATTCGCGAGCGCGACGTGACCATCGAGGGGACCG

ACTTCCTGTCGCTCGTCGAGCAGGGCGCGCGCGTCGACTCCCTGCTGTCG

CGCGAACTCGAAGACGAGTTCGACGAGGCGCTTTCGGCCGCCCGCGACCA

CCTCGTGGAGTCGCTGTCGCTGCGCCCCGGCGAGGCCGACCTCACGGAGC

GGGTGTTTCCCGACGACCCGTCGTTCCCGCTCGGCCACGACGAGGAGGCG

GTCGGCCGCCTCCGAACCGAACTGAAGGCCGCCCGCGACCGCCGCGCCGC

GAAGTTGAAAGCCGGCCTCGCCGCCGACCTCGGCGACCTGCGCGACCCCG

TCGAGACGCTGGTCCGCGACGCGCTCGAACTCGACGTGCGCCTCGCCGTC

TCGCGGTTCGCCCGCGATTTCGACTGCGTCTTCCCCGAGTTCACCGGCGC

GGCCGGGGACGACGGTGCCGGCTCGTTCGCCATCGAGGCCGGCCGCTCGC

CCCTCCTCGACGTGGACTTCTCGGACGTCGACCCCGTGGACTACGAGGTG

TCGGGCGTCACGCTCCTCTCGGGCGTCAACAGCGGGGGCAAGACCTCGAC

GCTCGACCTCGTGGGGCTCGTCGTCGTCCTCGCGCACATGGGGCTTCCCG

TCCCCGCCGAGTCGGTCCGCCTCTCGCGGTTTTCCGAACTCCACTACTAC

GCCAAGTCGCAGGGAACGCTCGACGCCGGCGCGTTCGAGAGCACCCTCCG

CGACTTCGCGGCGCTCACCGACGGCGCGGCCGACCGCCTCGTCCTCGTGG

ACGAACTGGAGAGCATCACCGAACCCGGCGCGTCGGCCAAAATCATCGCC

GGCATCCTCGAAGAACTCGCGGAACAGGGCGCGGCGGCCGTGTTCGTCTC

CCACCTCGCCGGCGGCATCCGCGAGGCGGCGGACGTGTCGGTCGCGGTCG

ACGGCATCGAGGCCGTCGGACTGGAAGACGGCGAACTCGTCGTCAACCGC

TCGCCCGTGAAGGACCATCTGGCGCGTTCGACCCCGGAACTCATCGTGGA

GAAACTGGCCGGCGAGAACGAGACGAGTTTCTACGGGCGACTGCTGGAGA

AGTTCGACTGACGCGGCCGCCAGCGCCAACACACGATTTATCTTAACACG

CCGACAACTCCGCCCGTGCCCTCCGACTCGCTCTCCCCCGAGGAACGACA

GCAGTACGACCTCGTCTACCACGCCACGAAGAACGCCATCTGGGACGTGC

TCGGGACCGCGGTCTACGTGTTATTTCTGGTGTTCGGCGGGTTCCTCGTG

CTGTTCGTCTTCGTACTTCCGGCGCTTAGTGCGCTCTCGCAGACCGGCGG

AACGCCGGTCGTCCTCGGCGTCGGCGCGGTCGGACTGATTCTGTTCGTCG

CCATCGGCTACCGAATCGTCCGGCTCCTTCAGTAAGTGACGTATCGGCTC

GCGAGTTCCCACCGAACCGTGGCGACGACGCCGACCGCGCCGAGCAGTAC

GGCGACGAGCGAGCCGACCGTCGTCGGGGCGGTGTAGTCGGTGGTCGCGT

CGAACTCGAACGCGTAGTAGTTCCCGTCACGCTCGACGAGTCCCGGGAGA

TTTCCGGGGTCGTCTTCGAACCGAAGCACTTCGCCGGCGAGGGCGCGGTC

CACGGCCCGCTGCTCAGACTGCGAGAGTTCGCTGTACAGCGGGGGAACCG

TCTCCGGCGTTTCCGACAGTTGCTGCTCGTAGCGATAGCTGTAGTCCTCG

TCGGCGGCGAAACCGAACCCGAGCACGCCCCCCCCGACGAGCGCGAGCGA

GACGAGCAGCGCGGTGAGCTGTGCGCGAGTTGCCATGGCTCCGCATCTAT

TGTTGTGAAATTAACTATTGTGGCTCAACTCCTGATGGGCTGTCGGTCCA

CGGGGCACACAGAAACGACGACGCCCGTCCGCCCCCCGTCGGACGCCGCC

GCGGTAAACGATTAAGTCCCCCGCGCGTGGTACTTTACTCGATGAGGGAG

GACCCCGAAGAGGGGATGCTGTCGTGGGACGAGACGGTGTTCCGCGACGA

ACACGTCTTCGAAATCGACCACGTTCCGGAGACGTTCAACCACCGCGAGA

GCCAACTGCGGAGCCTGAAGTACGCGCTTCGGCCCGCGGTCCGCGGCTCT

CGCCCCCTGAACACGATGGTGCGTGGGCCGCCGGGGACGGGCAAGACCAC

CGCGGTCCAGAAGCTGTTCGGCGAGTTGGGCACGCAGTCGGGCGTCCGGA

CCGTCCGGGTGAACTGCCAGGTCGACTCGACGCGCTACGCGGTGTTCTCG

CGCGTCTTCGAGCACATCTTCGAGTACGAACCCCCGTCGTCGGGCATCTC

GTTCAAGAAACTGTTCGGCCAGATAACCGACCGCCTCGTCGAGGACGACG

AGGTGCTCGTCGTCGCGCTCGACGACGTGAACTACCTGTTCTACGAGAAC

GAGGCCTCCGACACGCTCTACTCGCTGCTGCGCGCCCACGAGGCGCACTC

CGGCGCGCGCATCGGCGTCATCATCATCTCGTCTGACCTCTCGCTCGACG

TCATCGACGAACTCGACGGGCGCGTCCAGAGCGTCTTCCGGCCCGAGGAG

GTGTTCTTCCCCCGCTACGACGTGGACGAAATCGTCGATATCCTCCGCGG

GCGGTCGAAACGCGGCTTCCACGAGGACGTTATCGGCGCGCCCGAACTCG

ACCAGGTCGCGGAGTTCACCGCCGATAGTGGCGACCTCCGGGTCGGCATC

GACCTGCTCCGTCGCGCGGGCCTCCACGCCGAGATGCGCGCCTCCCGAAC

CGTCGACATGGAGGACGTGGAGGCCGCCTACGACAAGTCGAAGTACGTCC

ACCTCTCGCGGTGTCTGCAAGGGCTTTCGGACCCCGAGCGCGAACTCGTC

CGGGTCGTCGCCGAGTACGACGGCGAGCGCGCCGGCGCGGTGTACGACGC

GTTCAACGAGGCGACCGGCCTCGGCTACACGCGCTACTCCGAACTCGTGA

ACAAACTGGACCAACTGGGCGTCATCGAGGCCCGATACACCGAAATCGAG

GGACGCGGTCGCACCCGCGCCATCTCGCTCGCCTACGACGCGGACGCGGT

CTTGGACCGCCTCTGAGGGCGTCTCGCCCGCCCCGTTTCAGGACAATTTT

TACTCGCCGGCCGAGTCGGATGAGCCATGAAGACTACCGGCGGAACCGAC

GCGCAGAAGCGCCGCGCCGCGGCGGCGGCGGTCGAAGCGGTCGAAGACGG

GGCCGTCGTCGGGCTCGGCACCGGCAGCACCACCGCGTTCGCCATCCGCG

CCATCGGCGACCGCGTCGCCGACGGCCTCGACGTCCGGGGCGTCCCGACC

TCCTTCGCGGCCCGCGAACTCGCCCGCGAGTGCGGCATCCCCGTCGTCGA

CCTCGACGAGGTGGACGCTGTCGACCTCGCAATCGACGGGGCCGACCAGG

TTGCGCTCGCGGACAGCGCGCTCGTCAAGGGCGGCGGCGCGGCCCACGCC

CGCGAGAAAGTGGTCGACGCCTTCGCCGACCGGTTCCTCGTCGTCGCCGA

CCCCTCGAAAATCGCGGAAACGCTCTCGCACGCCGTCCCCGTCGAGGTGC

TCCCGTCGGCGCGAACGACCGTCGCGGCGGGCGTCTCGGACCTCGGCGGC

GAGGCGACGCTCCGCCGCGCCGAGCGGAAGGACGGCCCCGTCGTCACCGA

CAACGGAAATCTCGTCCTCGACTGCGAGTTCGGCGCGATTCCGAACCCCG

AGTCGCTCGCGACCGATCTCGCGTCGCTCCCCGGCGCGGTCGAACACGGT

CTGTTCGTCGGCCTCGCGGACGCGGTCTACGTCGGCACCGACGACGGCGT

GGAAGTCACTGAACTCTGACTCGATTGGTCCGGCGCTCGAACGGAATCCG

GCAGACCGAGTGGTCCGACGCTCGAACGTCCCACCTCGACCGCCCCGGCT

CACGGGGCGGGTCGCTTTCTCTGAGAGACACAACCGGCGCTCTTCGGTCG

GCGATAAAAGAACGAGAGGTCTAAAGACCGAGTTTACAGGTCGCGGGGCT

GGACCGTCTTACGGTCGTTCTGCTCAGCGCGGCGAGCAGCGTCTTCGAGA

AGCTCCTTGACTTCCTCGTCGAGAGCGTCGTAGAAGTCCGAGGCCACGTT

CTTGTCCTTGAGCGCTTCCTTGACGGCTGCCTTGACAATGAGGTCTGCCA

TACAGGATGTCCTACCGATTCTCCTTTAATAAACTTTCCTATATTGGACG

TTAATACCGCCGGATTTGGGGGTTATGGCGCTTCTATGAGGACGGATTTA

CCGGGAACAATCTCCACTTATATATGCTTTATCCCCGAGAGCTCGGAACG

ACGGGTATGAGTACGCCTCGGGCCAACGTGCGGCCATGCGCGACATCTTG

GAGGCGGTCGCGGACGGTTCGCTCTCGCCCGCCGAGGCCGAGGTACGACT

CTCCGGGTACGCGACGACCGACGCCGGGCGGTTCGACGCCGCCAGGGAGA

CGCGGCGCGGCGTCCCCGAAGCGATTCTCGCGGAGGGAAAAACGCCCGAC

GAGACGGCCACGCTGGCCGTCGCGGCGGTCGAGACCAGCGGTCGTGCGCT

CGTCACCCGCGCCGACACCGCCCACGCCGAGGCCGTCGCCGACGCGCTCC

CGGACGCCGACATCGACCGCGACGCCCGGGCGAAGACGGTCGTCGCGGCC

GCGCCGGAGTTCGAGCGCCCCGACCTCGACGCGACCGTCGCCATCGCCAC

CGGCGGCACCTCCGACGCCGAGGCCGCGGGCGAGGCGGCCGCGGTCCTCC

GCGAGATGGGCGTGGACGTCGAGCGAATCGAAGACGTGGGCGTCGCCCAC

CTCGGGCGCGTGCTCGACAACCTCGACACGCTCCGCGAGGCCGACGTGGT

CGTCGTCGCCGCCGGCCGCGAGGGCGCGCTGCCGACCGTCGTCGCCGGCC

TCGTCGACACGCCCGTCATCGGCCTGCCCGTCTCGACGGGCTACGGCCAC

GGCGGCGACGGCGAGGCGGCCGTCCTCGGGATGCTCCAGTCGTGTACCGT

CCTCTCCGCGGTGAACGTCGACGCCGGCTACATCGCCGGCGCGCAGGCCG

GACTCATCGCCCGCGCGGTCGCTGACGCCCGCGACGACGACGCCTGACCG

GGCGCAGTCTGTCCCCCGTCGTCGCCCACGTCTGTCCCCTCGTGGTCGTG

CGCGATTTCGCGTGCGTACGCACACGCGCGAAGCCCTCATATGGGTGGGG

GACTGTGTATCACACACCGGCTTGGTGGCTGCCGGACCGCAACAATGCCA

GTCTGTGACCACTGCGGGTCACACGTCTCCGAGCGCTTCGCACGCGTGTT

CGCCGACAAGAACGGGCAGGTTCTCGCCTGTCCCAACTGCTCGGCGAACG

CGGGTATCGCGGAGGTCGCTCGACAGCGTGCCCGCACTGCATGACCACTG

AAGCGACAAAAGACCACCACGCGAAGCCAACTCCTCGTGCGCGAGGTCGG

GCGCAGGGTTCATACCCGCGCCCCTGAACCATTCTCTCGTGCACTTCGTC

TACGTCATCGAGTGTAACGACGGCTCGCTGTACACCGGCTACACGACCGA

CGTGGAGCGACGCGTCGCGGAACACGACGCGGGCGAGGGAGCGAAGTACA

CCCGCGGTCGGACCCCGGTCGAACTCGTCCACGTCGAGGAGTTCGACTCG

AAGTCGGCCGCGATGTCCCGCGAGTACGAGATTAAACAGCTCCGCCGCCG

GGAGAAACAGCGACTCGTCGAGTCGTGAGCGCTTCTCCTCCCCGTCGGCT

CGTAACGTAGTTTTTTGCTCCACCGCGGAGTGGTGCGCGTATGGACGAAA

TCTCATTCGGGACCGACGGGTGGCGCGCCACGCTGGACACGTTCACCGAC

GACCGGGTCCGCGTCGTCGGACAGGCGGTCGCGGACTACCTCGCCGACGA

GGGCTTCACCGCGCCGGTGCTGGTCGGCTACGACGCCCGCGAGACCTCCC

CCGGCTTCGCGGAGTCGCTCGCGGAGGTCGTCGCCGGCAACGGCTTCGAC

GTGCTCCTGCCGGAGCGCGACTGCCCGACCCCGCTCGTCGCCTACGCCAT

CGCCGACCGCGGCCTCTCGGGGGCGCTCATGGTCACGGCCTCGCACAACC

CGCCGGAGTACAACGGCGTGAAGTTCATCCCCTCGGACGGCGCGCCGGCG

CTCCCCGACGTGACCGACGCGGTCGCCGAGCGACTCGCTGAACCGGACCT

CCTCCCCGAGTCCGAGCGCGGCACCATCGAGCGCGTCGACGTGGTCTCGC

CGCACGCCGACCACGCGATGGACCTCGTCGGCGACGACCTCTCGGACCTG

ACGGTCGTCTACGACGCCATGCACGGCAGCGGCCGCGGCGTCACCGACGC

GCTCCTCGAAGCCGCCGGCGCGGAGGTCGTCCGCCTGCGCTGTGAGCGCG

ACGCCGACTTCGGCGGCATCTCGCCGGAGCCCTCCGCGGAGAACCTCGGC

GGCCTCGCCGAGGCGATGGCCGAACACGACGCGGACCTCGGGGTCGCCAA

CGACGGCGACGCCGACCGCATCGGCATCGCCACGCCCGACCGCGGCGTCC

TCGACGAGAACCTCTTTTTCGCCGCCGTCTACGACTACCTGCTCGAATCC

GACTCCGGTCCGGCCATCCGGACCGTCTCGACCACGTTCCTCATCGACCG

CATCGCCGAGGCGCACGGCGAGGAGGTGTTCGAGACCGCGGTCGGCTTCA

AGTGGGTCGCAGACGGCATGCGCGAACACGACGCGCTCATGGGCGGCGAG

GAGTCCGGCGGCTTCTCGGTCCGCGGGCACGTCCGCGAGAAGGACGGCGT

CCTCATGGGCCTGCTCGCGGCCGCCGCGACCGCCGAGGAGGACTTCGACG

CCCGCCTCGACCGCATCGAGGCCGAACACGGCGACATCGTCGCCGACAAG

ATAAGCGTCGCCTGCCCCGACGCCGAGAAGGCGCGCGTCATCGACGACCT

CGAAGACGTACTTCCGGAGACGGTCGCCGGCCGCGATATCGCCAAGGTCG

TCACGCTCGACGGCTTCAAACTCCTCTTAGACGACGGTTCGTGGCTCCTC

GTCCGCCCCTCGGGGACGGAGCCGAAGATGCGCGTCTACGCCGAAGCCGG

GAGCGAAGACGCGGTCTCGACGCTCCTCGAAGCCGGCCGCGAACTCGTCG

AACCGCTCGTCTGAGCGGGCGGTTCAGGGCTCTCGTCTCTCTTCTTCGTC

GCCGACCGGTCTGACGACCCCGCGGTACGCCGGCGTGAAAAGCGGGAGCG

TCTCCGCGTGCTCGGTCAGCGAGACGCCGTCGCCCCCGGACTCGACGAGG

TCCGCGGCCTGAAGCTTCGGCAGGTGGACGTGAACGATGCCGACCTCCGC

CTGCTGGACGACCTGCACGTCGACCTCGTCGGGCGGGAGGTCGGTCGTCC

GAACCGCGAGGGCGACCGCGAGGTCCCAGACCGACGCCGGCTCCGACCGG

TCGTGGAGGTACTCCAGCACGTAGCGGCGCGACGGGTCCGCGAGCGCGTC

GAACACGCGGTCCCAGTCGGTCAGGATACGGTCGCTCCGGTCTCGGTGTG

CCATGGGCCTTGACTCCCTAGCGCTTGACGACCGGCGGCTAAATCTGTGT

CGATTGTTAACTCTCAACATCCGCGGCGGCGCGGCCGTCACTCGTCGCGC

AGGACGCCGCGAAACCGCCCCTCGTCGTCGCTTCGGTCCTCGATGTCGAA

TCCGAGCGACTCGTAGAACGGCTTGACGCCCCCGTCGAAGTCGACGACGA

GTCGGCCCCGGCGGTCGAGCGCCGCACGGACGAGTTGGGTTCCGACCCCC

CGGCCGCGGCGACTCGGGGAGACGGCTACGGCCTCGATATAGTCGCCGTC

GAGCGCCAGCGCGCCGACGACCCGGCCGGACGCCTCCGCGACGAGGCAGT

CGCCGGCGGCCAGTCGGTCGCGGATTTCGCCCGCGTCGGCTTCGAGCAGT

CCGGCGTCGAGGACGCGCATCACGGCCACCAGTTCGTCGGGGTCGCCCTC

GCGGACGCGGGCTCCGCCGGTCTCACGGTCTCTCTCGGTGTCTTCCATAC

CGCCGACTACCCGCCTTTGATGAGGCGGAGCACCTTCACGCGGTCGACTT

CGACGGACTGGTCTTCGGGGACGGGGCGGCCGTCGACGAGGACGGTCACC

TCGTGGGGGCTGAGGTCCACCGCCCGCACGAGGTCCGCGTAGGTCCCGTC

GTCGTCGACGGCGACCTCGCTGGTCTCCTCGCCGACGACCTCGACGGTCA

CGTTCATGGTCGCTCGTGGGTCGGGCGCGGGTTTCAGTCCGTCGGCTCGG

CGTCGGCCGGGGGCGGTCTCCGTCTCGCACGGTTTATGTGCGGGGCGGTC

GGTAGAACGGATATGAGCGAGGCCGCGGAGTCGGGCGACGCCCCGGCGGG

TCGTGAAATCTGGATCGAGAAGTACCGACCGCAGACCTTCGACGACGTCT

ACGGGCAGGACGACATCGTCGAGCGCCTGCGCAGCTACATCGAGCGCGAC

GACCTGCCGCACCTCCTGTTCGCGGGGCCGGCGGGCGTCGGCAAGACCAC

CTCCGCGACGGCCATCGCCCGCGCCATCTACGGCGACGACTGGCGCGGCA

ACTTCCTCGAACTCAACGCCTCCGACGAGCGCGGTATCGACGTGGTCCGC

GACCGCATCAAGAACTTCGCGCGCTCGTCGTTCGGCGGTCACGACTACCG

CGTCATCTTCCTCGACGAGGCCGACTCGCTGACGAACGACGCGCAGTCGG

CGCTCCGCCGGACGATGGAGCAGTTCTCCGACAACACGCGCTTCATCCTC

TCGTGTAACTACTCCTCGAAGATTATCGACCCCATCCAGTCGCGCTGCGC

GGTGTTCCGGTTTTCCCCGCTCGGCGACGACGCCATCGCGGAGCAGGTCC

GCGACATCGCCGCCGCCGAGGACATCGAGGTCACGGAAGACGGCCTCGAC

GCGCTGGTCTACGCCGCCGGCGGCGACATGCGCCGCGCCATCAACTCCCT

TCAGGCCGCCGCGACGACCGGCGAGGTCGTCGACGAGGAGGCCGTCTACA

TGATTACCTCGACGGCCCGCCCCGAGGACATCGAGGAGATGGTCCGGGCC

GCCATCGACGGCGAGTTCACGGCCGCGCGCAAGCAGTTAGAGACGCTCAT

CGTCGACACCGGGATGGCCGGCGGCGACATCATCGACCAGCTTCACCGCT

CGGTCTGGGAGTTCGACCTCGACGAGCGCGACGCCGTCCGCCTCATGGAG

CGCATCGGCGAGGCCGACTACCGCATCTCCGAGGGCGCGAACGAGCAGGT

CCAACTCGAAGCGCTCCTCGCGTCGCTCGCGCTCTCGCAGAACTGAACGC

AGGACACTGCACCGGTCGTTTCGACGCCGCGGTCTCCGGTCTCTCCACTC

GTTTTCGCGTCTGTCACGCCCGGAACTCGAAGCGCGCGCCGCCCGCCGTT

CCGGAGGTCACGATGGCCGACCAGCCGTGGGCCTCGGCGATGGATTGGAC

GATGGCCAGCCCGTAGCCGATGCCGTCCTCGCCGGTCGTCTCGCCCGGTT

CGAACACCTCGTCGCGGCGCTCGGGCTCGATGCCGGGGCCGTCGTCTTCG

ACGTAGAAGCCGCCGTCGACGCCGCCGACCTCGACGCGCACGTCGCGGCC

GTCGTGTTCGACGGCGTCGTCGGGAGTATCCCGACTGCCCGTCGAGCCGT

GCTCGACGGCGTCACCAGACGCAGTCTGGTTGCCCGTGGAACCATGTTCG

ACGGCGTTCCGAAACAGATTGGAAAGCAGTTCGGCGAGGCGGTCCTCGTC

GGCGTCGACGACGACCGACTGGCTGGTGACCAACTCGGCGTCGCCGGTAT

CGGTGGTCGCCCACGCCTGCCGGGCGACCGCCCCGAGGTCGACGCGCTCC

GTCTCGTCGACCAGTTGGCCGCGGCGAGCGAGCGACAGCAGGTCGTCGAT

GAGTTCTTCCATCTGTTCGAGCGCCCGCTCGGCGGTGTCGAACCGCACCT

CGTCGCCGGTCTCGCGGGCCAGTTCGAGGTTCCCGCGGGCGACCGAAAGC

GGGTTGCGAAGGTCGTGGCTCACGATGTTCGCGAACTCTTCGAGGCGCTC

GTTCTGTCGCTCTAGCTCGCTTTCGCGGGTCTTCCGCTCCGTGATGTCGG

TGTAGATGGCGTACCCCGCCCCGCGCTCCTCGCCGGGCGACAGCGGGACG

ACGTTCAGGATGAACTCGCGGACGCCGCCGTCGGTCCGGCGGCGGACCTC

CCCGCGGTAGCTCTCGCCGGTGGCGACGAGGAGTTCGGGGATGGTCTCGT

CGGCCCCCGGTTCCGGCACCGGGTCCGGGTCCGGCTCCGGCGGGACGATT

CGCCCGAGGAGGTCGCTGCCGACCACGTCGTCGGCGTCGTAGCCGAACGC

CGACTGGAACGCCCGATTCACGTCGCGAACGGTGACCGCGCCGGCTTCCA

CGTCGTAGGCGACGGCGGCGTCGGAGACGTGTTCGAACAGCGCCGTCGAG

CGGTCGCGCTCCTCGCGGAGCCGCGACTCGGCGCGGATGCGCTCGGCCGT

GACCGCGACGTGCGCCATGAGGAGTTCGGCCAGTTCGGCGTCGCGCTCGT

CGAACGCGCCGGGGGTGAACGTGGTCGCCTGAAAGACGCCGAGGTCCCCG

ATGGGGACGCTCAGGACCGCGCGGTACATCCGCTTGACCGGCCTGGCCTC

GGGGTGGTTGCGCACGTCGTCGGTGATGTCCGTGTTCCCCGACAGGTACA

CCTCGGCCGCGATGCCGCCGTCGTCGAGCGGGACGCGCTCCGCGCCGTCG

GCCGGCGCGTCCGACGAGATAGCCGCCGGAACGATGTAGCCGTCCTCGAC

GACGCCCACGTAGCTGATGTCGAACTCGAGGATGTCGTCGGTGATTTCGA

CGGTCTGTTCGAACAGTTCGTCGAGGCTCGTCGCCGCGGCCAGTTCGGTG

GCTCCTCGGTGGAGTCGCTCTATCTGCTCGCGGCTCTTTCTGACGGCGGC

TTCGGACTCGATGCGGCTCCGCGTCTCGGAGACGTAGGACACGAGGAGTT

CGGCCAGCGTGAGGTCGGTCTCGTCGTACGCGCCGACCTCGTTGGAGATG

GCCTGAAACACGCCGTCGTCGCCCAGCGGGACGCTCACCGCCGAGCGGTA

CGCGGGGTCGTCGGGGTCGGCCACGGGGTGGTCGCGCACGTCGTCCACGA

GAAACGAGCGTTTGGTCCGGTGCGTCTCCCCGAGCAGGCCCGTGTCCGAG

GGAATCGGCCCCGTCCCGGCCATATCGTCGTTGTTGGCGGCGACGTAGAA

GCTGTCCCCGTCGTGGACGAAAAACGAGGTGTTGTCGAACTCGAGGATGT

GCTCCGTGAGGCCGATGGCGCGCTCGTAGAGTTCGTCGACCGTCCCGGCC

GTGATGAGGTCCGTCGTCCCCTCGTAGAGCCGCTTGAGCCGGTCCCACTC

GGCGTCGTTTCGGGCCAGCGCCACCTCCTCGGCGTCTCTGTCGGCGGTCG

ATGCGAGCACCCATCGAACCTGCGATTCGAGCCCGGAGCGGTCGCCGCGG

CTGACGTAGGCGTCGAATCGGGCCGGCCGCTCGACGGCCTCGGGCGACAC

CTCGGTGTACAGAATCGTCGGCACGTCGCGGGGTATCTCGGCGTCCGGGT

CCGTGACGACGACGCACGCCGCGTCGCTCGGGTCGTTCTCGTCACTCGCT

GACGCCACCGTGACCCCGTCGCCGAGGGCGATATCGTCGAAGTCGTCGTC

CACGAAAACCGTTGGGTCCGTCGCGTGCACGGCAGTCCCCCTACTGGAGG

GCGACGAGAACTATAATCGTTGCGGCATGTTCTCAATGAGCAAGAACACG

CCGCGAGCGGTCCGATTCGGCGGCGCGGCGCTTTGACCTCGCCGGATTCC

TCAGTTTAGCGACCAGAACCCGTAGTTGAGCGCGGCGGCGAAGCACGTCC

ACAGGAGGTAGGGGACGAGGAGGGCGGCGGCGCGCCGATTGACGGCGGCG

AACTCGCGCGTCGTCGCCAGAATCGCCGCGAGGAGGACGACGATGACGGC

GAGGCCGACCGCGGGCGACCGCAGTCCGAAGAACGCCGCGGACCACGCGA

CGTTGACGACGAGGTGGCCGACGAAGACGGCGAGCGCCCGCTCGCGGCGG

GGGTGGTCGCGCCGGGAGACGAGCCACGCGGCGACGCCCATGAGGGTAAA

CAGCGTCACCCAGACCGGGCCGAACACCCAGTTCGGCGGGGCGAACCACG

GGCGGGCGAGCGTCGCGTACCACGACCCCGCGTCGGTGACGGTGAAGACA

CTCCCGAGCGCGCCTGCGAGTTGCGCGAGGGCGACCCACGCGAGCAGTTC

GACCGCCGGTCGCCGCCGGAGCGAAGCGAGTTTCATACTCTTCGTTCGCT

CGGGTGGGAAATGAACGACGCGGCCCGTGCGGGCGAAATTGTGGGGAATC

GGGCGTCTGCGTCCATCTCCCGGAACTGTTTTACCCGACGGTAGACCAGA

GTTTGGCAATGAGCGAACTCGAAGCGGAGTACCGCCTCGACTACTTCGAG

GAGGAGGGCTTCTACCGGAAGCAGTGCCCCGTGACGGGGGTCCACTTCTG

GACACGAGACCCCGACCGCGAGACTTGCGGCGAACCGCCCGCCGACGACT

ACACCTTCATCGACAACCCCGGCTTCGACGAGGAGTACACCCTCGAAGAG

ATGCGCGAGAAGTTCCTCTCCTTCTTCGAGGAACACGACCACGAGCGCAT

CGACCCCTACCCGGTCGCCGCGAACCGCTGGCGCGACGACGTGCTCTTGA

CGCAGGCCTCTATCTACGACTTCCAGCCACTCGTCACGTCCGGCGAGACG

CCGCCGCCGGCCAACCCGCTGACCGTCTCCCAACCCTGCATCCGGATGCA

GGACATCGACAACGTGGGCAAGACCGGTCGCCACACGATGGCGTTCGAGA

TGATGGCCCACCACGCGTTCAACGCCCGCGAGGAGGCCGGCGACAAGTAC

GCTTACGAGGGCGAGGTCTACTGGAAGGACGAGACGGTCCGCCTCTGCGA

CGAGTTCTTCGAGTCGCTCGGCGCTGACATCTCCGAAATCACCTACATCG

AGGACCCGTGGGTCGGCGGCGGCAACGCCGGTCCGGCCTTCGAGGTGCTC

TACCGCGGTGCCGAACTCGCCACCCTCGTCTTCATGTCGATGAAGCAGGA

CCCCGACGGCGACTACGAACTCAAGGACGGCAACACCTACTCGCCGATGG

ACACCTACATCGTCGACACGGGCTACGGGCTCGAACGGTGGACGTGGGTG

TCGCAGGGGACGCCGACGGTGTACGAGGCCGTCTACCCCGACATGATCGA

GTTCCTCAAGGACAACGTCGGCATCGAACACACCGACGAGGAGGAGGAAC

TCATCCACCGCGCGGCGAAGCTCTCGGGCCACCTCGACATCGACGAAATC

GACGACCTCGCCACCGCGCGCGCCGAAGTCGCGGGCGAACTCGGCGTCGA

CGAGGCCGAACTCACTTCGCTGCTCCGCCCGCTCGAAGACATCTACGCCA

TCGCGGACCACTGCCGAACCCTCGCGTACATGTTCGGCGACGGCATCGTC

CCCTCGAACGTCGGGACGGGCTACCTCGCCCGTATGGTCCTCCGGCGCAC

CAAGCGCCTCGTGGACAACCTCGGCGTGGACGCCCCGCTCGACGAACTCG

TGGACATGCAGGCCGAGCGCCTCGACTACCAGAACCGCGACACCATCCGC

GACATCGTCCGCAGCGAGGAGCGCAAGTACAGAAAGACGCTCGAACGCGG

CTCCCGTAAGGTCCAGCAGCTCGCCGACGACTACGCCGACACGGACGAGC

CCATCCCGCTCGACGAGCTCATCGAGCTGTACGACTCCCACGGCATCCAG

CCCGACATGGTGCAGGACATCGCGGAAGAGCGCGGCGCGACCGTCGACAT

CCCCGACGACTTCTACTCGCTCGTCGCCGACCGCCACGAGCAGGTCGACG

GCGAGGGCGAGGCCGACGAGCGCGACGACCGCCTCGGCGACCTGCCGGCG

ACGGACAAGCTCTACTACGACGACCAGGAACGCACCGAGTTCGAGGCCGT

CGTCCTCGACGTGTTCGAGCGCGAGGAGGGCTACGACGTGGTCCTCGACC

AGACGATGTTCTACCCCGAAGGCGGCGGTCAGCCCGCCGACCACGGCTCG

CTCGCCACCGACGACACGACGGTCGAAGTGACCGACGTGCAAATCGTGGG

CGACGTGGTGCTCCACCGAACCGACGAGGACCCCGGTAAGGGCGAGTTCG

TCCGCGGCCAGCTCGACGCCGAGCGCCGCCGTCGGCTCATGCGCCACCAC

ACGGCGACCCACGTCATCGGCCACGCCATCCGGACGGTCCTCGGCGACCA

CATCCGGCAGGCCGGCGCGTCGAAGGGCGTCGACCGCTCGCGGCTCGACG

TGACCCACTACGACCGCATCACCCGCGAGGAGGTCACGCAGATAGAGCGC

GTCGCCAACGAAATCGTGATGCGGAACATCCCGGTCAAACAGGAGTGGCC

GGACCGCCGCGACGCCGAGAAGAAGTACGGCTTCGACCTCTATCAGGGCG

GTATCCCGCCGGGCGAGCAGATTCGCCTCATCCACGTCGGCAGCGACGTG

CAGGCCTGCGGCGGCACCCACGTCAAGCGCACCGGCGACATCGGGGCCGT

CAAGGTGCTGACGACGGAGCCCGTGCAGGACGGCGTCGAGCGCGTCGTCT

TCGCGGCGGGCGACGCGGCCGTCGAGGCCACCCAGCGCACCGAGGACGCG

CTGTACGGCGCGGCCGACGTGCTCGACGTGAACCCCGCGGACGTGCCCGA

GACGGCAGAGCGCTTCTTCACCGAGTGGAAGGAGCGCGGCAAGACCATCG

ACCGCCTGAAGACGGAACTCGCCGAGGCGCGCGCCGCGGCGGGGGCCGAC

GAAATCGACATCGACGGCACGCCCGCCGTCGTCCAGCGCCTCGACGGCGA

CGCGGACGAACTCCGCGCGACCGCGAACGCGCTGGTCGAAGAGGGGAAGG

TCGCCGTTCTCGGCTCCGCCGCGGGCGGCAGCGCCCAGTTCGTCGTCGGC

GTCCCCGACGACGTGGGCATCAACGCCGGGCAGGTCGTCGGTCGACTCGC

CGGCAAGGTCGGCGGCGGCGGCGGCGGTCCCGCGGACTTCGCGCAGGGCG

GCGGCCCCGACGTGGACGCGCTCGACGGCGCGCTCGACGAGGCTCCCGAC

GTGCTTCGGACGGTCCTGAACGCCTGAGTCGGTCGCGCGACGCCGACGAG

TTCGCGTTCGCTTTCGCGGTTTCGCTTGCTTCGCGTCCGCGAGACGGACG

CCCTAAGTCGCTCGCCCGCCGAGCCCCCGGTATGCCCACGGCAGTCGCGG

ACGGGGTCGAACTGTACTACGACAGCGAGGGCGACGGCGAGACGGTCGCC

TTCGTCGGCGACGCGGGCTACGGCGCGTGGCAGTGGGGGTGGCAGCACGC

CGCCGTCGCCGGCCGCTACGAGAGCCTCGTGACCGACCTCCGCGGGGCCG

GCCGCTCCGACGCGCCCGCGGGGCCGTACTCGGTCGCGACGCTCGTCTCC

GACCTCGTGGCCGTCCTCGCGGACGCGAACGTCAGGAAGGCCCACCTCGT

CGGATTCGGCCTCGGCGGGCTGGTCGCGCTGGAGGCCGCGCGGACGACGA

ACCGCGCCCGGAGCCTCACGCTCGTCGGCACCGCCGCCTCCGGGGATGGC

ATCGACCCCGACCCGCTCTCCGCCGCGCCCGACGACCCGGCCGCGCTCCG

CGAGTCGCTCGCGCCGGCGCTCTCCGAAGAATTTCGCGCGGAACAGCCTG

ACGTGGTCGAGCAACTCGTCTCGTGGCGCAAACGGGAGGACGCCGACCCC

GAGGCGTGGGCCGCGCAGGCCGCCGCCGTCGCCGACTACGACGCCGGCCC

GCTGTACGAGGTGACGGTCCCGACGCTCGTCCTCCACGGCGGCGACGACC

CGGTCTGGCCGGTCGAGGGTGGCCGCGCGCTGGCCGAGGGTCTCCCCCGC

GGCGAGTTCGAGTCGTTCGCGGGCGCGCGCCACCTCGTCACCGTCGAGCG

GTCGCGGCTCGTCAACGACGCGCTCGTCTCCTTTCTCGAATCGCTCGACG

ACGACTGAGCGTCGCTGTCGGGCGCTCTCCCCGCGGTCGCCGCCGACGCG

CCCCGTCGCAGATGCCGGCAGTGAGCGTTCCGGGCGCAACTTCTTTAGAG

CCGCACCGATTGTCTCTCCACGATGACTCGCCCGCGACTCGCGTTCTTGG

ACGCCTCCCACGGCGATTCGAGCACGTCACGAAACTTCAGGCGCGAACTC

GACGCCGACCTCGTCGAGTTCGACGTGACCGACGGCCGTCTCCCCGACCA

CTTCGACTACGACGGCGTCGTTATCAGCGGCTCGTCGTCCTCGGTCTACT

GGGACGAGCCGTGGATTCGCAACCTCGTGTCGTGGGTCGCCGACGCCGAC

GAGCGCGGCGTCCCCCTCTTGGGCGTCTGTTTCGGCCACCAGGTCGTCGC

GGCGGCGCTCGGCGGCACCGTCGAGGACATGGGCGCGTTCGAACTCGGCT

ACAACGAAATCGAGCGGACTCGCCCGGACGACGGGAACGACATCCTCGCC

GGCATCGGTGAGCGCTTCACCGTCTTCACCTCCCACGGCGACCGGGTGAC

GGAACTCCCGTCTGGAGCGGACCTCCTCGCCGAAAACGAGTTCGGCGTCC

ACGCGTTCCGCCGGAATCACGCCTTCGGCGTCCAGTTCCACCCCGAGTAC

GACACCGACACCGCAGAAGCCATCGCCCGCCAGAAGGACTTCCTCCCCGA

CGAGCGGATTCGCTCGGTCGTCGACGGTATCACGCCGGAGAACTACGCCG

CCGCCTGCGAGGCAAAGCGCCTGTTCGACAACTTCGTCACCTACGTGAAC

CGGACGAACGCGGGCTCGGTCGAATCGGCGGCCTGAGCGTTCCGACGGTC

GCGAGCCGTCTTCGAGTCGCATCCGAGCCGCCGTCGAGTCGTGCCCCTCT

CGCTGCCGAGCCGCGCCTGCCCCGCTTTCGTCACCGACTTTTCGGTCGTC

CGTCTTCCCCGAATCTCGACCGAGCGAATCGCTTTTCACCGCGCCGAGCG

CAGGGTCTATCATGCTCGATTATCTGGAATTGGAGGGCGACCTGACCGGC

GAAGAGAAACTCGTCCGCGACGAGGCCCGCCGCTTCGTCGACGAGCAGGT

CAAACCCGACATCGGCGAGCACTACGAGGCGGGGACGTTCCCGACCGACC

TCATCCCGAAGATGGGCGACCTCGGCTTTTACGCGCCCAACCTCGACGGC

TACGGCCTCCCCGGTCTCGGCGAGCGCGCCTACGGCCTCTTGATGCAGGA

ACTCGAAGCCGGCGACTCCGGCCTCCGGTCGATGGCGAGCGTGCAGGGGT

CGCTCGTCATCTACCCTATCCACGCCTACGGCTCCGACGAGCAGAAACAG

CGGTGGCTTCCGAAACTCGGGTCGGGCGAGGCGGTCGGCTGTTTCGGCCT

GACTGAACCGCAACACGGCTCGGACCCGTCGGGGATGGAAACCCGCGCCG

AGCGCGACGCCGACGGCTACGTCCTCAACGGCTCGAAGACGTGGATTACG

AACTCGCCCATCGCGGACGTGGCGGTCGTCTGGGCGCGCGACGTGTCGGC

GGAGGGGTCGCCGGTCCGCGGGTTCCTCGTCGAGACCGACCGCGACGGCG

TGACGACGAACAAAATCGAGGGCAAACTCTCGATGCGCGCGTCGGTCACG

GGCGAAATCGGCCTCGACGACGTGCGCGTGCCCGAGGAAGACGTGCTCCC

GGGCGTCGAGGGGATGAACGGGCCGCTGTCGTGTCTCACGCAGGCGCGGT

TCGGCATCGCGTGGGGCGTCGTCGGCGCGGCGCGCGACGCCTTCGAGGAC

GCCCGTCAGTACGCGAAGGACCGCGACCAGTTCGGCGGCCCCATCGCGCG

GTTCCAACTCCAGCAGGGCAAACTCGCGGAGATGGCGACCCAAATCACGA

ACGCGCAACTGATGGCCCACCGCCTCACCGACTTGAAAGAGCGCGGCGAC

CTGCGACACCAGCAGGTGTCGATGGCGAAGCGCCACAACGTCCGCGTCGC

CCGCGAGGTGACCCGAACCGCCCGCGAGATGCTCGGCGGCAACGGCATCA

CGACCGACTACTCGCCGATGCGCCACATGGAGAACATCGAGACCGTCTAC

ACCTACGAGGGGACCGACGACATCCACACGCTCGTCCTCGGCGCGGACCT

GACGGGAATCGAAGCATTCCACTAGGGCTCGGGAGCGGACGCCCCCGTGG

CTTTCGCTGTTGTGCGTGTCTCATGACAACAGTACACATACATTTATATC

TTCCCGCCCATAGGTTACGATAGAGGCCCCACAATGACCACATCGGCCCA

GGACCGTCCGACGGCGCTCGACCTGTCTCGCGAAGAGACGTGGATCGTCC

ACGCCGCCTTGCTCGACGCCATCGAGCGGGCGGTCGATGCAGACGAAGAG

CCGACGCCGGCCCCCGGCCTGCTCGCGCGGGTCGAGGCCGGCGACGAAGA

TTTCGACAGCGCCGAACTCGACTATCTCGTAGACGCCCTCCGAACCTATC

GGACACAGGCTCCCGCCCGGGACGACCACCACATCTCGCGCGTCCTCGAA

CACATCGAGGCCGCGCGGGCTTAGTCGCCCGCCCGACGCTCGCCCGCTGG

CTTTTTCCGCTTCCGAGGTCCTCGAGCGGCCGCGTTCTCCGGTGATGCCG

CCGACGCGTCCCGGTCGTCTCGGTGACCGACCCCGCCCCAACCACCGACT

TCGACCCCGACTCCGTCTCCGAGTTCTCGCGTCTCGATGCGGACGTTTTA

GTACGCGGTACGGCCAACGCCCGGCGATGGCTATCGACTCGAACTTCGAC

CAGAACCGGGAACGCGCGGGCGAGGAAAACGGCGTCGCCGTCTGGGGACC

TGTCGAACCGCCGGAGAAACTCGGCATCCACGGCACCCACGTCGCCGTCG

ACTACGACATCTGTCTCGCGGACGGCGCGTGCCTCGAAAACTGCCCCGTC

GACGTGTTCACGTGGGTCGACACGCCGGACCACCCGGTGAGCGAGAAAAA

GGTCGAACCGACCAACGAAGACCAGTGTATCGACTGCATGCTCTGTGTCG

ACATCTGTCCCGTCGACGCCATCGACGTGGACGCCTCGCGGCAGGCCTGA

CGCGAAGACCAGACGTTATCTCTCCCCGCCGCGTCGCGTCGGCCATGGGT

CTCATCCACACCGCCCTCGTCGTCTCGGACATCGACGCCACGCTCGATTT

CTACGCCGACCTCGGCCTCGAACGGACCAACGAGTTCGAACTCGACGGCG

TCCGGAACGTCTACGTCGGCAGCGACGACACCGACATGGAACTGCAGTTC

AAGTACGACCCGACCTCGGCGGCGCGCGTCGAACCCGCCGGCATCGACCA

CGTCGCGGTCGAAGTCGCCGACGCCGACCGCGCCTTCGAGGACATCGTCG

AGGCGCAGTCGCCCGACATCGTGAAACCGCCGGTGGACATCGACCCGGTG

AACGCCCGCGCGGCGTTCGTGAAGGACCCCGACGGCTACGTCGTCGAACT

CGTCTCCATCGAAGACTGAACTCACCCGCGGTACCGACTCGCCTCGGCCG

CTGATTTTCGCCGACGCCGACGCTTCACTCACCCGACGGGCTCGCCGCGG

CGAACAGGTATTTGTTGCCAGAGCGTCAATACGAAGTCGCTATGTCTGAG

GACGTAACCGCGCTGTTGAAGCGCGCCTATCAGGACGAGATCGAGACCGT

GATGAACTACATGACCAACTCCATCGTCCTCGACGGCGTCCGGGCCGAGG

AGATTAAGGAGTCCCTCCAGACCGACATTCAGGAGGAACTCACCCACGCC

GAACAGCTCGGGAACCGGCTGAAGCAGCTCGACGAGCAGCCGCCGGGCTC

GGCGTCATTCGAGGCGCGCCAGCACGACCTCCAGCCCCCGGAGGACAGCA

CGGACGTGCTCGCCGTCATCAACGGCGTCCTCACGGCCGAGGAGGACGCC

ATCGAGACCTACCGCGCGCTCATCGACGCCGCGGAGGAGGCAAACGACCC

CGTCACCGAGGACCTCGCCGTGACCATCCTCGCCGACGAGGAGGCCCACC

GGACCGAGTTCCGCGGTTTCAAGAAAGAGTACGACCGCGAATAGGTCCTC

CGAGCACCGAGCGCCGACCGACGGGCGACTGCACGAGGGTTCGATCGCGC

TCCGTATTCACTACTTTTCGAAAAATGTGTCATTATCGACCCGTATCTAA

CAAATTATCATCTGTCACAAAAGTAGTATTCCCAGTTCTTCGAAACAGGG

GTTTATTTTATCAATCCGTCGCTCTAACGACCGGTATGAGCACTGAACGC

GGCAAAGGTACGGTCGCGGTGGTCGGCGGCGCCGGCGCAGTCGGGTCGAG

CACGGCGTTCGCGCTGATGGAGAGCAGTCTCGTGGGCGAAATCATCCTCG

TCGATATCGCCGAAGAGCGCGTCGAGGGCGAGGCGATGGACCTCAACCAC

GGCGCGTACTTCACGTCGCCGGTCCGGGTGCGGACGGGCGACTACGAGGA

CTGCTGGGACGCCGACGTGGTCATCGTCACCGCCGGAGCCAGCCAGAAGC

CCGACGAGACCCGCCTCGACCTGATGGAGCGGAACGCCGACATCTTCGCC

GACATGATTCCGCAGATTACCGAGGGGCTGAACGACGACGCCGTGATGCT

CATCGTCACCAACCCCGTCGACGTGCTGTCGTACGTCACGTGGAAGGTGT

CGGACCTGCCGGCCGAGCGCGTCATCGGGTCGGGGACCGTCCTCGACACG

TCGCGGTTCCGCCACTCGCTCAGCCGCGAGTTCGACCTCGACCCGGCGAA

CGTCCACGCCTACGTCGTCGGCGAGCACGGCGACAGCGAGGTACTCGTCT

GGAGTTCGGCGAACCTCGGCGGCATCCCGTTCGAGTCGTACGCCACCAGC

CACGGCGTCGACGACATCGACGCGCTCAAAGCCCGCGTCGAAGAGGAGGT

CCGCGAGGCCGCCTACGAGATTATCGAGCGTAAGGAGCGGACGAACTACG

GCGTCGCCCGCTCGGTGGCCGCGACCACCGAGCGCATCCTCGGCGGCGAC

AACTCCATCCTCACCGTCTCGACGCTCGTCTCGGGGGAACACGGCATCGA

CGACGTCTACATGAGCCTCCCGTGTACCGTCAACCAAAGCGGCGTCCGCG

ACGTCCACGAGTTCGACCTCTCGGCGGACGAGACGGCGGCGCTCCGGGAG

TCGGCGGGCGTCATCCGCGAGTCTATCGACCGCTTGGACCTACAGTAAAA

TAGCCGCGCCGCCGACTCAGTCGGCAGACGCGGGCGTCGTGTCCGCGCCC

GTCCCCGTCGCGGCGGCCTTCGCGTCGAGCGCCTCCACGACGAGTTCAGA

GATGTCCACGACGTCGATGGCTTCCTCGTAGTCACCGGTCTTACGGCCGT

CTTCGTACATCGTCGCGCACATCGGGCAGGCGACGACGAAGCGCTCGACG

CCGCCGGTCGTGTCGTCGAGCGCCTCGCGCAGGCGTTCTTCGGACGGCTT

CGACTCCTCATCGTGGTCCATCCAGAGGCCGCCGCCGCCGCCGCCGCAAC

AGAACGACTGGTCGCGGTTGCGCGGCATCTCGGCGAGTTCGACGCCGGTC

GCGCGGATGACCTCGCGGGGCGCTTCGTACTCGCCGTTGTACCGGCCGAG

GTGACACGGGTCGTGGTACGTCACCGTCTGGGCGGCGAGTTCGTCACCCG

CGAGGCCGAGGCGGTCGTCGCCGACGAGGCGCTCGACCAGTTGGGTGTAG

TGGTACACCGGGTAGTCGAACGAGTCGTCCATCTCCGGGTACTCGTTTTT

GAACGTGTTGTACGAGTGCGGGTCGGTGCAGACGATTTTGTCGAACTCGC

AGTCGGCCATCGCGTCGACGTTGTCCTCGACGAGCATCTCGTAGAGGCCC

TCCTCGCCGACGCGGCGCACGTCGTTGCCGTCGTGCCCTTCGTCCTCGTA

GAGGATGCCGTAGGAGACGCCGGCTTCCTCGAAGATCTGGGCCAAAGAGC

GGGCGACGGCGCGGTTACGCTCGTCGTAGGAGGGGTAGTCACCGACGTAC

CAGAGGAACTCGACCGATTCGTCGCGGGCGTCGGGCACCTCGAAGTCGAG

GTCGTCGGTCCAGTCGGGGCGCTTGCGCTGGGGGTCCCCGAAGGCGTTGC

CGTTCTGGAAGATGTTCATCATCGCCTCCTGGACCGGCTCTTGCTGTTGG

CCCGTCTCGGTGAGCCGGCGGTTCATCTCGGTGAAGTGCGTGAGGTGTTC

GATATCGACCGGGCAGGCGTCCATGCAGGCCATGCAGGCCATGCAGGACT

CCATCGACTCGGCGTCGACGACGGAGGTGCCGCCGTCCGCGACGATGTCG

ATGGAGTCGCCGCCGGCGTCGACCGACTCGCGGTAGGCCTTCAGGTCGAG

AATCACGTCCCTCGGGTCGAGGTTGCGGCCGGAGGCCTTCGCCGGGCAGG

CGTCGGTACAGCGGCCGCACTTCGTGCAGGCGTCCATGTCGAGCATCGCC

TTCCACGAGAAGTCGTCCTCGGAGACGTAGCCGATTTCGTCCGGCGGCGT

GTCCGCGGGCACCTTCGGGAGGCGCGTGCCCGCCTTCTCGTCGGCGGTGA

CGACGTTGGCGAAGGAGGTGAGCATGTGCAGGGGCTTGGCGTAGGGGACG

GCCGCGACGAACGCCAAGGCGAGGAGCGCGTGGGACCACCAGCCGAACCA

GTAGAGCGTCTGCGCGAGGCCCTGTCCCATGCCCGCGGCGTCGAACACGA

GGGCGACGAAGTAGCCGACGAAGCTCACCGTCTCGAAGTCGGGAAAGTCG

GTGCCGACGATGCGGATGCCTTCGAGGACGTAGCCGCCGACGCCGAGGAA

AAAGAGCGTCCAGACGAACGCGGCGTCCTCGAAGCCGGTGTGTTTGCCCC

ACAGCCGCGGGTGTCGGACGCCGTAGCGCCGCCACAGCGCCATCCCGACG

CCGACGACGAACAACAGCCCCATGAGGTCCATGACGAACGAGTACGAGAG

GTAGAAGTCGCCGACGAAGAAGGAACTGCCCGTCACCCGCCGCCACACGT

CGATGTCGATGGCGAGGATGGTCGTCCCGATGAGGAGCGTCAGGAAGCCC

CACATGATAAAGGCGTGCATCACGCCGGCGAACCGGTCGCGCTTGAACTG

CTTCTCGTTGGAGAACACCACTTTCGCCGCCCGAACCGTCCGTCCCGGCA

GGTCGGACAGCCGGTCGAACGGGTCTTCACCACCTTTCGCGTATCGCGCG

AACCGCTCGTACACCCCGTAGAGGAACACGAGCGACGCGATGGCGGCGAG

ACCGTAGAAGAGCGCCTCGCCGACCGGACTAATCGTCCAGAACGTCTCCC

GCGTAACATCCGCCTGTGCCGCAAAGTCCATAATCAATCACCGGAGAACG

GAGGGTTAAATCTTGCCACCCGAGGCGTGTCGGTCGGCGGATTACGAGCG

CCCCGGGCGACCTCAGAGCGCGAGGCTGACGAGGAGCGCGGCGGCGAGGA

CGAGTCCGGGCGCGTCGCGCCGGCCGAACCGAATCTCCGGAAGCGTCGGG

TTCCACGCGAAACACCGCGCGCGGAGCGCGAGCGCGAGGGCGTCGGCCCG

CGCGAACGACCGTCGGAGGCCGGCGGCGGCGACGAGCGAGAGCCGTTCGT

CCAGCCGTCTTTCGCTCCCGAGGCGGGCCGCCTGCGCCTCGCGGACGCGG

CGCAGGTCGCGGCGGACCAAGGGGAGAAACCGGAAGACGAGGGCGACGCC

GACGCCGAGGGCGACGCCGAACCGACCCGGCACGAGCAGCTGAATCGCCG

CGCGGGACTCGCGTACGGGCGTCGTCCTGACGTACGCCGCCGCGACGAAG

AAGACGAGTAGCACGCGGACGCTGGCGAGCGCGGGTTCGACGGCCGCCTC

GAGGTCGAACCACGGCGCGCCGAGGCGGGCGGCCTCGACGACCGGGCCGA

GCGCGAGGAGCGGAATCGCGTAGCGGAGGTCGCGGAGGGCCGAAACGGGG

GACGCGTCGGCGGCGCGGAGACAGCCCGCGGCGACGACGCCGAGCGCGAC

GAGTCCGGCGGGCGTCGTGTGCGCGAAGCCGGCCGCCGCGAAGGCGACCT

GCACCGCAAGCTTGGTCCGCGGGTCGAGTCGGTGGGCGAGAGTGTCGCCC

GGTTCGTACGTCAGCACGGCGGGCGAACGTCGAGGTCGGGGAGGCGGTCT

GCGGCCTCGTCGGGCGCGGCGTCGAGGGCGACGTCGCCGTCGGCGAGGAC

GACCACGCGGTCGGCCAGTTCGGCCACGTCGCGCAGGTCGTGGGTGACGA

CGACGAGGCTCGTCCCCTCGGCGCGGAGGTCGCGGAGGCGGGCGAGCACG

GAGCGCCGGGCGGGTTCGTCGAGGCCGGTGAACGGTTCGTCCAAGACGAG

GTGGTCGGGGCGCATCGCCAGCGCGCCGGCGATGGCGAGTCGCTCGACCT

CGCCGCCGGAGAGAGAGTCGACGCGCTCGTCTTCCCGGCCGTCGAGGTTG

ACCGCGGCGAGCGACTCGCGGACGCGGCGGTCGATGTCGCCGCGGTCGAG

GCCGAGGTTCTCCGGGCCGAACGCCACGTCAGCGCCGACGGTCGCGGCGA

CGAGTTGGTCTTCGGGGTGCTGGAACACCATCGCCACCGCGGTCCGGGCG

GCGACGAGGTGGTCCGAGACGGTCCGGCCGTTGACCGACACCGTTCCCTC

GTCGGGGTCGAGCAGGCCGTTGAAGTGGCGGACGAGCGTGGTCTTGCCGG

AGCCGTTCGCGCCCGCGACGACGACGCACTCGCCGTCGTCGATCGCGAGG

TCGACCCCGTTGACCGCGACCGTGTCGCCGTACCGGTGGACGAGTCCCTC

GACGCGAATCATCTACTGGGCGGCGATGGCGTCGGAGCGGACGATGCCGA

CCGCGGCGGCGATTTTGAACGCCTCGGCGGGGATGAACGCGGCGGAGGCC

GTCCAGAAGGCGGATTCGAGGCCGACGCGATTGACGTACGCGAAGCCGAG

CGTGCCGAAGGCGTAGACGACGACGGTCCCGGCGACCATCGCGCCGACGA

GCCGGGGAGTGCTCACGCGGTAGTCACCGGCGCTGATACCGCCGTGGACG

ACGAGGCCGACGACGACGGCGGCGACGGGGTACGACCAGAGGTAGCCGGC

GGTCGGGCCGACGAGCGGCGCGAGCCCTGCCGAGCCCCCGGCGAACACCG

GCGCGCCGAGACCGCCGGCGGCGAGGTAGAGCGCGATTGAGCCGCCGCCC

CAGACGGGGCCGAGGAAGATGCCCGCGAGGAAGACGACGAGCACCTGCAT

CGTCACCGGAATCGGTGAGATGGGGTTCGGGAACGAGACGTACCCACTCG

CGCCCATGAGCGCCGCGAACAGGGCCGCGCGGGCGATGTTGCCGACGAGT

TCGTCGCCGACGAGTTCGACTGACTCGCGTTCGGTTGCCATGCTCGTCGC

TGTCTCGTAAACCCAGTTTAACTTATCGGTTTACAGACTCGGGGCGGGAG

GTCGGTTCGATGCTCGTCGGCCGATTCGGGGGTCTCTCCGGCGCTCGGGC

GGCTCTCCGGTTAGTCGGTCAGCATCATTAAAGACGCTGGGCGTGCCACG

TTCCGGGTATGGACGAGAAGACCGCCGAACTCCGCGATATCTTCATCGAG

GCGACCGGCTCGGACACCGTGACCGAACAGCAGTCCGAGTCGCGCGGGTC

GCTCGCCGACGTGGACGACCCCGAAGCGGTCCGCGAGCGCGTCCGCGACA

TCGTGGCGACGATGCGCGAGCGCTACGCGTTCGACACCGAGTTCGACGAC

GACGCGCTCGTCGCGCTCGTCGTCGGCTTCTTCGACGGCGACTCCGACGC

CGACCTCGCGGTCGAACTCGACGCGTCGCCCGAGGCGGTTCGCACCGCCC

GGTTCGACCTCCACCTCGTCCGCGACGCCGACCGCGAGTTCCCGTTCGAC

GCCGACCGCTTCGACACCCTGCTCGTCGACGGCGCGGACGACGAGGCCAT

CGTCGAGGCGCTCGACGTGCCCGTCGAGGTCGTCTCCGAGTGCCGCCCCG

TCGCCGAGGCGGACATCCGTTCGAGCCGGGCCAACTACCGCTTCCGCGAC

GACTTCGCGGAGCTTCTGACCGACGACGACCTCTCGAACCGGATGGCCGA

GGACGCCCGCCGCGACGGCCTCCGCGAGGCGACGGAAGATATCGAGACCG

ACGTGTCGCTGTAACGGTCGGCCGAACGACGAAGGTTCTTAACCGATTGG

AGACATACCCCTGTGCGATGGCGCAGGCCCCCCAGAACCGAGACCTGACG

GAACGGTTCATCGAGTTCTACCGAAACTACTACCGAGAGGAGATCGGGAC

GCTCGCACAGCAGTACCCGAAGGAAAAGCGGTCGCTGCACATCGATTACG

ACGACCTCTATCGGTTCGATTCGGAACTCGCGGACGACTACATCACGAAG

CCCGGCCAGTTCCAGGAGTACGCGGAGGAGGCGCTGCGCCTGTTCGACCT

CCCCGCGGACGTGAAACTCGGGCAGGCGCACGTCCGGATGCGAAACCTCC

CCGAGACGGTCGATATCCGGAACCTCAGGGTCAACGACGACCACATCGGG

ACGCTCATCTCGGTGCAGGGCATCGTCCGGAAGGCGACCGACGTGCGTCC

GAAAATCACGGAAGCCGCCTTCGAGTGCCAGCGCTGCGGGACGATGAGCT

ACATCCCGCAGGGCGACGGCGGCTTTCAGGAACCCCACGAGTGTCAGGGA

TGCGAGCGCCAGGGGCCGTTCCGCATCGACTTCGACCAGTCGAACTTCGT

CGACTCACAGAAACTGCGCGTCCAGGAGTCCCCCGAGGGCCTGCGCGGGG

GCGAGACGCCGCAGAGCATCGACATCAACCTCTCCGACGACGTGACCGGC

AAGGTCACCGCCGGCGACCACGTCACCGTCGTCGGCGTCCTCCACATCGA

ACAGCAGACATCGGGCAACGAGAAGACGCCCGTCTTCGACTACTACATGG

AAGGCATCTCGCTCACCATCGAGGACGAGGAGTTCGAGGACATGGAGATA

TCCGACGAGGACGTGGCCGAAATCGTCGAACTCTCGAACGACCCGGCCAT

CTACGAGAAGATGGTCGAGTCCGTCGCGCCCGCCATCTACGGCTACGAGC

AGGAGAAGATTGCCATGATTCTCCAACTGTTCTCGGGCGTCACGAAACAC

CTGCCCGACGGCTCGCGGATTCGCGGCGACCTGCACATGCTGTTGATAGG

GGACCCCGGAACTGGCAAATCGCAGATGTTATCATATATCAGACATATTG

CGCCCCGCTCGGTCTACACCTCCGGCAAGGGTTCGTCCTCGGCAGGTCTC

ACCGCCGCCGCCGTCCGCGACGACTTCGGCGACGGCCAGCAGTGGACGCT

CGAAGCCGGCGCGCTCGTCCTCGCGGACAAGGGTATCGCGGCGGTCGACG

AACTGGACAAGATGCGGCCCGAAGACCGCTCTGCGATGCACGAGGGGCTC

GAACAACAGCAGATTTCGGTCTCCAAGGCCGGCATCAACGCGACGCTCAA

GTCGCGGTGTTCGCTCCTCGGCGCGGCGAACCCGAAGTACGGCCGCTTCG

ACCAGTACGAACCCATCGGCGAGCAGATAGACCTCGAACCCGCGCTCATC

TCGCGGTTCGACCTCATCTTCACGGTCACGGACGACCCCGACCCCGACGA

GGACTCCAAGCTCGCGGACCACATCCTCAAGACCAACTACGCGGGCGAGC

TGAACACCCAGCGGACGAACGTCGCCAACTCCGAGTTCACGGAACAGCAG

GTCGACGCCGTCACCGACGAGGTCGCGCCCACCATCGACGCGGACCTGCT

CCGCAAGTACATCGCCTACGCGAAGCGCACCTGCTACCCGACGATGACCG

ACGAGGCGAAGGAGGTCATCCGCGACTTCTACGTCGACTTCCGCGCCCGC

GGTGCCGACGAGGACGCGCCCGTTCCCGTGACCGCCCGGAAGCTCGAAGC

GCTCGTCCGCCTCGGCGAGGCCTCGGCCCGCGTCCGCCTCTCGGACAAGG

TGACTCGCGAGGACGCAGAGCGCGTCACGGGCATCGTCGAGTCCTGCCTG

CGAGACATCGGCATGGACCCAGAGACCGGCGAGTTCGACGCCGACATCGT

CGAGACGGGCCGTTCGAAGACCCAGCGCGACCGCATCAAGAACCTGCTCG

AACTCATCCGCACCATGCAGGAGGAGTACGAGGAGGGCGCGCCCCACGAG

GAGGTCCTCGAACGCGCCAACAGCGAACTGAACATGGACGAAAAGACGGT

CAACGACCAGCTTGACAAGCTGAAGATGAAAGGCGACATCTACGAGCCCC

GCGGCGACGTGTACCGCGCGACTTGAGCCGTCCTCGGCTGCTGCGCCCGC

CCGTCAGCGACTCGGCTTCGATTCCGCGCGGAACCGACGAACTTAGTGGA

GCGTGCGCTGAGGGGACAGACGACCGATGGACCGAATCTCCGCCATCCGC

AACATCGAAGACGCCATCCGCGACCTCGAATCGGGGGACGCGGACCTCGC

CAGCACCGAGCAGCGCGTCGTGACCGTGCTCCGGACGTTCGCCACGGAGT

TCGAAGACGACGCCGGCGACGGGACGCTCGACGCGTGGACGGCCGTCGGC

GACGACCGCGCCGAGGGACTTGTCGTCCTCGCCGCCGACGCCGACGACGC

GCGGGCGCGGGTCAGAGACTTACTCGACGAGGCGACCGGCGACGCCGACG

ACGTGACGTTCTCGGTCGAGCGCGTCTGAGACGACCGCCGCGGGGATTCG

GAATCGGATTCGACGACCGCACGCTTCTGCCGTCGCCGTCACCGTCTCCG

CCACCCATTGGTGTCATATAGCTGACCGGAGGGCGACGCGGTCGAACGAT

TTTTCTTTCGACTCCGTGACCGGTCGTGCGTGTTGTTGGTCGTGACCTAT

TCGCAGGCGGCGCGGACGACGCTTCGGAACATCTGCCGGACCCACGACGA

GGTCGTAGTCCGTCAGTTGGGTCGCGCCGCGCTGTTCGACGAGACGGAGT

TGGCCGCCTTCCTCGCGCTTCGGCTTCGGGAGAAACACGACGAAGACGTT

CAAATCGAGCAAACCCAGCCGTTCAACGAGTTCGCGGCCGTCCCCGATGC

GGTCCGCGAGGCCGCGGCCGCCTACGAGGACCGCGAGTCGCCGGCGACGC

CGTACAGCAAGTTCGCCTCGGGGACCGACCACCCGTCGGCCGCCGAGATG

CAGCGCCGCGAGCTATGAGGCTCCGATTGCCCGACGGAACCGCCGTCGAG

GGGCGAGCTATCGACCTCGCCAGCGACTCGGTCGCTGTCGACGCCGACGA

GGTCGTCGCCGCGATACGAGCGGACGGGAGAGCCGACGCGCCGGGCGCGG

TCCGAATCGACTGCTCGTCGCCCGGCCCCGGCCACGACCGACTCGGCAGA

GTTCCCGTTTCGGACCCGGCCCCGTCCCGCGGGTGGCTCCTCGCGGCAGT

CGGGCGGTCCCGCGGCCGCCGCGCTCCCGTCGCCGACGACCTCGACCGAC

TCGAAGCGAAACTCGCGGAGCACCGCGACCGTGCGGCCGGGGAGTCGAAT

GCGGACGACCCCGAGCGCGTCGACACGTCCGCGGCCGCGCTCCGCCAGGC

CCGCCACCGCGTCGCCGCCGCCGGAGAAGACGAGTCTCGGCTCAGAGAGC

GCGTCGCGACCCTCCGCGGACGGGTGAACGCCCACCGCGACGCCGGCGAC

GACGAGGCGACCGCGGCGGCCCGACAGGAGCTCGTGGAGGCGGCGACGGA

ACTCTCGGAAGTCGAAACAGAACGCGTCGCGGCCGAACAGCGGCTGTCGG

CGCTCGAACAGGCGAGCCGAGCCGAACGAGACAGGCGGGACGCGAGACTC

AAACTCGAAGACGCGGTCGAGAACCGCCGCCGAGACGCGCGGGCGTACTT

CGCGGCCGAACTCGGAGCGGAGTTCGAAGCGGCGCTCCGAGACCTCGCGT

CGCTCCCGGCTGCGGCGACTCCGGCGGTCGACTCGCCACCTGTGCCGCCC

GCCCCCGAATCAGACCCCGTCGCGCGCTCGCTGGCCGCCGTTCGCCTCGC

CGACCTGTCCGCCCCGGTCGTCCTCTCGTGCGGCCGGTTTTCGGACGCCG

AGGCCGCGGCGGACTGGCTCGGCGCGCCGGTCGTTCGGTGTTCTCCCTCC

GATTGACGGGGCCGCTCCGGGCGTTGATTTATATACGAAGCGGCGGCCAA

CCCCGCCATGCACTCCGATATCGACGTGAGCGCGGACGACGGCGTCGCCC

TCGTCTCGGTCCGAGTCGACAACGACGCGCCGGTCGACCGACGCGTCAGG

CTTCGCAACCGCCTCGACGGCCCGGTGCTTCCGCCGCGTCGCGCCGGCGT

CCCCGAACCCGGCTGGGACAACGAGGGCTTCGAAGGCGTCGTTCCCGCGG

GTTCGACGGTCGCGCTCGGCTACGCCGTTCCCCTATCGCCGCCGGGCGAC

CGTGACGCCGAGGCTGTCGACGCCGACGGCCTCCCGGAGCACGCGGTCGA

TGTCGAGACGCTCGGCCGCGCCGACGAGTCGGACGAGGCCGCCGATTCGA

CGCCCGAGGGCGCGATTCGGTCGCTCGGCTCGGCGCGGCCGCCGGCCGAC

GCGGTTCCCGTTTCTGCTGACCCGCCGCCGACGGTCTCCGCGCCGGAGAC

GCCCGAGTCAGCTCCCGACACCGCCGGCGACGAACGCCCCGACCCGCTTC

CGCGACCCGACCCCGACGCCGCCGGCGGCGAACGTTCCACTCCGTCCGAG

CAGCCGACGGACGCCGACGTGTCGGCGGCGGTCGAGACGCGCGAGCCGCG

CGACCACGAGACCCAGCGGCCATCGGAGCCGGCGTCTCTCGCGGCCGTCG

AGCGTCGTATCGACCTCGCTGAGCGGCTGGACGGCGCGTCGGTCGCGGAC

GCCGCGGCGGCGCTCTCGGAGGCCGAACGCGGCGTTGAGGCGCTCGAATC

GCTCGACGCGGACCGCGAACGCCTTCGTCGATTGGCGGCGCGCGCGACCG

CGCTCGCGGACCGCGCGGCCGACGCCGACCCCGACGTGGACGCGCTCCGG

AGGCTCGCGTGATTCTCGCGGTCGTCTCCGGGAAGGGCGGCGTCGGGAAG

TCGACGCTGTCGTTCGAACTCGCGGCGGAACTGGGGTCGGTCGTCGTGGA

CGCGGACCTCGGAATGGCGAACCTTCCGTACGCGCCCGGTCCCGACCTCC

ACGACGTGCTCGCGGGTCGGGCCGACCCGCTCGAAGCCGTCCGCTCGGGC

GGGCCGGTCTCGATACTCCCGTGCGGGCGGACGCTCGCCGGCGCGCGGGC

GGCCGACACGCGCCTCCTCGCCGACGCGCTTCACGCGGTCGAAGCGGCCT

ACGGCGACGTGGTCGTGGACTGCCCCGCCGGGATGCGCGCCGATGCGGGC

GTCCCGCTCGCCGTCGCCGACGCCTGCGTCGTGGTCGCGTCACCGAAGGG

CTACGCGCTGGCCGACGCGGTTCGAACCCGCGAGCTGGCGCGCGAACTCG

GCTGCGGGCTCGCCGCCGTCGCGGTCAACCGCGTCGTCGACGCGGTGCCG

CTGGACGCCTTCGCCGACGTGCTCGGCGCGCCGGCGACGGGGATTCCCGC

GGACTCGCGGCTGGCCGAGGCGGTCGAGCGGTTCGAGCCGGTCGTCGCGT

CGTCGCCGGAGAGCCTTCCCAGTGCTCGGATTCGGGAACTCGCGGACGCG

GTCCGGGTGAGCGGTCGGTAGAAGCGTCGTCTCGTCTTCGGTTCGTTCGC

GAAACAGCGGTTCAGCTCTGGCGTCGTCGTCGCCGTCGGTCAGTGACGGT

CGCAGTCTCGCGGCTCACTCGCGGAGCGCCACGTACGTCTTGCGGAGCGT

CACGGGCGTCACGTCGGCTACGTCGGCGGCCTCGGCCTGCGTGCAGTCCT

CGCCGCGGTCGGACGCGGCGGTGTAGAGACACGCCGCGGCGACGCCGCAG

GGGTTCTTGCCGCCCACGAGCCCCTCGTCGCGGGCCTCGGCGACGTACTC

GCGCGCCCGGCGTTCGGTGTCGCTGTCGAGGTCGAGCTTGGTCGCGAACC

GCGGCAGGTACTCGGTCGGGTCGGCGGGCGCGACGGGGAGGCCGAGGTCG

CGGTTGAGCGCGCCGTAGGCCGCTTGGTGCTCCGCCTCCGTAGCCTTCGA

CACGTCGCAGACCTCCTCGACGCTCCGGGAGACGCCGGCGACGCGGCAGG

CGGCGTAGACGCAGGCCGCGGCGAACCCTTCGAGCGAGCGGCCGCGCAGG

AGGTCTTCGTTCTGGGCGGACTCGAACAGCGAACACGCCTGGTCGCGGAT

GTGGTTCGGCAGCGACAGCGCGCTCGTCACGCGGCGAATCTCGGTGAAGG

CGTACACCTGATTGCGCTCGCGCTTGGTCGAGATGCGGGCGCGGTTGTGC

TGGCGGCGCATGCGGGCGAACTGCCGGCGCTTGCGGCCCTTGATACGCGT

CGAGCGGCCGATTTCGGTCGACAGGCCGCGGTCGTGCCGCGAGCGGGTGA

GCGGCGCGCCGGTCCGAGCGGGGTTGCGGTCGTCGTCGGCGAACGAGCGC

CACTCGGGGCCGTGGTCGATGCGGTACTCCGAGACGACGAGACCGCAGTC

GGTACAGACCGTCTCGCCGGCTTCGAAACCGAGGTGTCCATCGCATTCGG

GGCAACCCTGGGGGGAGCGTGCTTCACTCATCACAATTGAAAATCGGTCC

CGAACGGGTATTTAAACACGGGTTGTAACGGGGTCGAACACCCGACTCGG

CGCGGTGAAGCCTACCGGTTACCGTTACGCTTGGATATCAGTACTGATAC

TATCTGAGAACACTTTTACCCGATAGGCGCGGAGCACCGAGCGATGGGGT

TGTCAGAAATCGCCGCCGCGTTAGAGACGACGACGACTGAACAGCGTTCG

CGCTGTGTCCCGACCGTCGATGACACCGATGTTTCCCTCCGCGAGCGCTT

CGAGGCGCACGCAGACGCGCTCCCCTGTGCGCCTGCGGCAGCGGAACGGG

TTGTCGAAGCACACGGGTCTGGCACGTCGGTCGGAGCCAGCGCGCGCGAC

GCAGGCGTCGCTCCCGTCACGGCGGCGAAGCTCTTGCACCGTTGCGGCGA

AGCGGGCGTGACCCCCCTCGCACCCGAGGCGCGACGCATCGTCCGCGATT

GGATCGACGGGCGCATCTCGCGCGCCGACGCGCTCGCGCTCACCGGCGCG

GAGCCGTCGGAGTTCGCCCTCGCAACGTACGTCGAGACACACGACCCCAT

CGACCCCCTCGTCGAAGCCGTCGAGGGCGCGCTGGCCGACCGCACCGACG

CGGCCGTCGCCAAGCGCGACTCGCTGGCCGAGACCATGAGCTCGGTCACC

GATATGCAGTTCTGACGCGGGAAACGCCTCCCCACCTCCCGTTTTCGGCT

CGGATTCCCCGCTCGCGAGCGGCGGCGCTCGTCGGTCAGCTATCGCTCCG

TCCGAAACGCGGCTGGTGTCCCGTGCGCCTCAGCGACGGACGTACTCGCT

GACCGAGCCGAGGACGCCGTCGGTGGCAGCCACGGCGTCGATTTCGATGT

CGAGAAGCACCTCCGCGACCGCGGCGACGCCCGCCGCGAGCTCGGCGTCT

TCGCGGCACGCCGGCGCGTCGGCGAGGCCGGTCGCCTCGGTCTCGGGCAC

GACCACGTCCGCGTCGTCGAGCGCGCCGAACGTGGAGACGACGGCGTCGA

TATCGGTGTCGATGTCGGCGGCCCGCGCACTGAGCCGACCGACCGCGTCG

GCACCGCGGTCCGTGGCGGGCGCGACGAGCGCGACCCGGTCGCAGGCCGC

GGCGGCGGCGACCGTCTGGTTCGACGCGAGGAGCGGCGCGTCGACGAGGA

CGTGGTCGAAGCCGTCTGCGGCCTCGGCGATACGACCTTCGAACGCCCGC

GCGGCGTCGGGCGTCATCGCCCGCGCGAGCCGCTCGAAGGGGGCCGCGGC

CGGGCAGACGGCGACGCGTCCCTCGGCGTCGGTCTCGAACGCGACCAGCC

CCGACTCCAGCGGCACGTCGGCGGCGTCGGTGACAAGCGCCGTCGCGTCG

GGGTCGAGCGTCCCCGAAACGTAGTCTGAGAGGCCCTGCGTGGCGAACGC

CGCGTCGAGGACGGCCACGTCGCGGCCGTCGGTCGCGAGCGCTGCGGCGA

GTTCGAGCGACAGTCGGGTCGTGCCCGCGCCGCCGGTGGCCCCGACGAGC

GCGATGGTGGTGGGAATGGACATACCGCGAGTGGTCGGGTGCTCAGATTA

ATAGCTTCGGTCGCCCGCCGCCGGCGTCGCCGGACGCGCGACTACGCCGT

CCACTCGCCGTCACCCGATTCCGGGTCCGCTCCCGGCGCGGCGTCCGCGC

AGGCCCGGTAGAAACAGACCTTGTACGTTCGGCCGACGAGCGTCGTGACC

GTCGAACCGACGGCCACGACGGCGATATCGACCGTCGACATCGGCGCTTG

GAACGCCGTCACCGCGCCGGCGACGGTGGTCGCCCCGTCGAGGGTGACGA

ACCAGAGCACCGGCAGGTCGACGACCGAGGTCACTGCGGTCGCGACGACC

GCGTAGCCGACGACGGCCCGGAGGTTGCTGAGGACGAAGTCGACGCTTCT

ACTGAGCGACCCGCCGGCGGTCTGTCCGTCGAGGACGACGGCGGTATCGA

AGAAGATGAAGACGTAGACGACGGCCATCAGGACCACGAGCGCCACCCCG

GACGCGGCGAGTTCGGCGGGCGTGAGGCTCGCGGAGCCGTTCAGCACCGC

GTTGAGTCGGTCGAACCCGACGTAGCCGAGGACGCTGCCGCCGACGACCA

TCGCGACGACGAACACGACCAATCCGGCGAGGAGCGTCGCGCCGAGCAGG

CTGACGTAGTAACGCTGTGCCGCGGTGGACGCGCGCAGGTCGGTGTCGGA

GCCGGGGCGCGCGCCGTCGGCGACGGTGTAGAACGCGCCGGTGAGCGCCG

GCACGGCGAGGAGTTGGAGGAGCGAGCCGACGGCCCCGGCCGGACCGGAG

GCGTTGTTCAACGCGCCGACGAGCCCGAACGCGAGGGCGAACGCGACGAG

CACCGGCGTCTCGGTGACGATTCGGAGGGCCGTTCGAAACGATTGGAGGA

CTGCCACGACAGTCGGCACTCGCGTCTCCCTTATATGTTTTTACATCGAC

ACGTTCGGCGGGAGATGTAGCGCCGGCTGGAGCGACAGCGTGCCCTCAGC

CCGAGATGGCCGCCTCGATGTCGGCCAGTTCCTCGTCGGAGAGGTCGGGG

CGCTCGCCCGCGACGGCGTGGATGGGACTGCCGCCGTCGCCCTCGAACCG

CGGGACGATGTGGACGTGGACGTGGTCGACCTCCTGGCCGGCGGCGGGGC

CGTTGTTGATGCCGACGTTCGTCGCGTCGGCGTCGACCGCCGCCTCGACG

CGCGGCACGAGGTCGTTCACGGCGGCGAACAGGTCGGCCGCGGCGTCCGC

CGGCACCTCGTCGAGGCGGGCGTGGTGCTCTTTCGGGACGACGAGCGTGT

GACCGGGCGCGAGCGGGTTGGCGTCGAGGAACGCAAGCGATTGGTCGGTC

TCGTGGACGATTCGGCCGGGGATGTCGCCGTCGACGATAGCGCAGAAGAT

GCAGTCGGACATACGTCGGATGTCGATTGACGAGGAAAAGAAAGTTCGGA

CCGGTCCGTGCGTGCCGTCTCGTCTGTCTCGCTGTCGGCACAGGTGGAGC

GTGTCCACCGAACCTCCTCGGCCGATTGTGGAGGGTCTCGTCTGTGAGAG

ACGCAACTGCGCCAATTGCACGCAATCGCAACTGCAATCGACCCTCGATT

GCGCAACCTCCCGCTGTTCCCTCGGCGAACTAGTTGATATTGCGCGCAAT

CCATCTCAGATTGCGTAATACATCTATTGAATTGCGCAATAATCGGTGGG

TTATCGCTCGGGAAGCAACGCCTCCAAGTCGGCCAGATACTCCTCTAACT

CGTAGCCGAGACGCGACAGCCACACGTCGCGGTACGAGGGGTGTAAAAGC

GGGACCACGGTGTAGCCGAACCGCTCGCTCTCGACGGGGTCGAGGACGGT

ATCGAGGAAGCCGTCCAGCGAGGCGTCGTCGAGCGCGAGGACGCTTTCGG

TCGCGTGGCGGCCGGTCGGGACCACCACGTCGGGGTCGACGGCTTCGAGT

TCGGAAACGAGGTGGTCGCGGCAGTTCGCCTTTTCGGCGGCCGTGGGCGC

GCGGTTCGACCCTTCGCCGTCCGAAGGGAAGCACTTGACGGCGTTCGTGT

AGAAGACGCCCGATTCGTAGCCGACGGCGGCCATCGTCTCGCGGATGCGG

CGGCCCGAGTGCCGGGCCGTGTAGGACATCCCGGTCCAGTTGCCGCCCCG

CCAGCGCTCGGCGTCGGGGTTGCCCGCGCCCGGCGCTTCCCCGACGACCA

TCACGGTCGCGTCGTCGGGGCCGACGCCCCACGAGATGCGGTTGCGGCAG

TCGACGAGCGCCGGACAGCGCGCGCAGTCGGGTTCGAGGACGAGCGTCTC

GTCGGCGTCGGGGAACCGCGGGTCGGCGTCGACCATCTACTCCGTCAGCG

GGAGCACGATGCCGAAGATAATCGGCGAGAACAGCACCAGCGCGATGCCG

AGCCACTCGGCGTCGAAAAACAGCGTTCGCTCCCAGCCGGGGATGGCACC

GCCGCTCACGGCGAGGGCGACTTTCGCGCCGATAGCGCCGAACAGGAACA

GCGCGAGCCCCAGTCCGAGGCCGGTCTTGGTCATCCGCGGGTAGTCGATG

TCTCCGTAGCGTCCCATACTATCCCGAGATGCGGGGACTGGCAAAACCGT

TTCGAGAGAGTGTGTGATACGTTATCGTTCGATTTCGCCGGCGAGAACGG

GCGCGACGGACACCTCGCTCACGTCGCGTTCGATGGTGTCGGTACCGACG

ACGCGCTCGATACCGGCGCGCGCGAGTTTCGTGCGGGCGTTGCGGGCGAG

AAGCGGGTGGACGGTCGCACAGAAGACGCGCTTCGCGCCGCCGTCGGCGA

GGACGCCGACCGCCTCGCTCATGGTCGACCCGGTGGCGATGATGTCGTCG

GTGATGACCACGTCGCGGCCCTCGAAGTCGGCGTCGCTCGGCGTGATGTC

CACGTCGGTCCCGGAGTGGCGCACCTTCTCGAAGTAGTCGGTCTCGCCCT

CGCCGTAGGCGTCGCGGACGGTCTCCGCGATGTCGATGGCTCCCGAGTCG

GGCGACAGAAACAGCGGGGCGTCGAGTTCGGGGAGCGGCTCCGCGAGTCG

CCCGGCGGCGTCGACGATGTCGACGGGCACGTCGAAGAAGTCGGCGACGG

CGTCCTCGTGGGGGTTGACGAGCACGACGCGGTCGGTCGTCGTGCTGACG

GCGCGGGCGACCGCGCGGGCCGAAATCGGGTGGCCGGCCTCGAACGCCTT

GTCCTGTCGGGCGTAGCCCATGTACGGGATGACCGTCGTGACCGCCGATG

CGCCGGCCTCGCGGACGGCGTCTTGCAGTTGGAGCAGTTCGACGTAGGCG

TCGTTGCTGGCGGTGCTGGCGACGATGGTGGCGGCGTCGGCGTCGAAACC

GGGGACCGCGGCCAGCGTCTCGCCGTCGGGGAAGCGGTCGTACTCGACGG

CCGCGAGCGACTCGCCGAGTTCGTCGGCGAGCGCGGCCGCGAGTGCCTGC

GAGGATGCGCCGGGTACGAGCATACCCGTTCGTCTCTCGTGGGGGGTAAA

ACCGCTTTTCGTTGCGTTCAGCCGCGTCGCGTGTCAGCGAACCGCGACGC

CGACCGTCTCTAACCCGAGCACCTGCGTCCGCCAGTCGCCCCCGGTCAGC

CGAACGCAGAAGCTCCCCGCGTCGGTGACGGCGTAGGCGGCCCGCGCGTC

GTGGGCGAGCGCGACGACCGACTCGTCCAGCGGAATCGGCGCGGCCTCCC

ACGCGCCCGACTCCTCGTCGCGCGCGACGACGCCGTCCGGGCCGACCGCG

TGGGCGCGGCCGTCCGGCATCGACGCGACCGCCGAAAACGCCCCGTCGAG

CGCGTCCATCCAGCCGTTGCCGAGCGCGTACAGTCCCGAGTCGGTCGCGG

CGAGCGGGGTTCCCGAGTCGCCGCCGAGGCGGCCCGCGGGGTCGGACACG

TCGGTCACGTCGTTCAAGCCGACGTGCGAGAGGCCGCCGTCACCGATTCG

GTAGACGCCGTCGTCGGCCGCGACGAGGCTCCCGTCGAGGGCGCGGACCG

CCCCGACCGCGCCGAGGTCGGTCCACCCGGACTCGTCGAAGCGGGCGACC

CGACCCGAGTCGTCGGCGGCGACGACGCGATTGCGGTGGAAGCCGACGGC

CGCCGCGGGGCCGAAGTCGAGTTCGTGGTAGTCGTCGCCCTGCACGAGCA

GCACGTCCTCGTCGGTGGCGACGACGACTTCTCCGGGCGACCCGGCGGCG

TCCAGCGCGGTACACCGGCGGTCGATGCGGAAGCCCCCGACGAGGTCGTC

GGAGACGTCGACGGTCACGACCCCGACGCCGGCGGCGACGTACGCCGTCT

GCTTGCCGGACTTGTCCGCGTACACCCGCTTTTCGTCGATAGAGAGGTCG

AAATCAGCCATCCGTTGGCCCGAGGTTCACGGCGGGGTTCGGAAAGGATT

GTGGTCGTTCCCGCGGCGCGGCGACGGGCCGAACGGGCGCGCGAGTGAGC

CGTGTGCTTACAGAAACTCCCGCACGTCGGAGTACCACATGTCGTGGTGG

TCGAGTTCGTCGAGGGCCTCGGCCACGTCGACCGCGAGGACGTGCCAACA

GCGCTCGCCCTCGTCGGGGTCGAGGTTGTACTGGGAGTCCTTGCAGGTAC

AGCCCCGGTCTTCGACCACGTACTCGTCGTCGTAGCCGACGACCACGTCG

AAGTCGCGGTAGCGCTTCACCCGCCGCTCGGAGACGGCCTCGATGGCCGT

GACGCCCCGGTCGCCGTGGGCGTCGACGATGGCCTGCACGATTTGCGGCG

AGAGTCGCCCGGTCTCCGCGAGCGCCGCTCGCCAGTCGTCGGCCGAATCC

ACGCTCCCGAGTTCGTCGGTGGCGGTGAAAACGCATTCGATGCCGCGGGG

TGGACCGACCGCCGCAATCGCGTCGTACAACCGCATCGCACAATCGCACC

GCACAATCGCATCGCAACCGGTTTCCGCTCGCGCGGGCGACTGCGGGTAT

GCACGTGAGCGAGGGAGGCGTCGAAATCGAGGTTCCCGACGCCCGCGACG

GGGCGTCAGCGGGGACCGGCGACGATGTCTTCTACAACCCCACACAGGAG

TTGAACCGGGACATCACCGCGGCCGTCCTCCGCGCCTTCCGCGAGCGCGA

ACCCCGCGCCGAGAGCTACCTCGACGCGATGGCCGCCTCGGGCATCCGGG

GCGTCCGCGCCGCCGCCGAGGGCTACGACGTGACCTGCGCCGACCTCGAC

GCCGACGCCGTCGAACTCGCCCAGTCGAACCTCGACCGCGCCGGCAACGG

CGGCGAGGCGGTCCACCGCAACGCCAACGCCCTCATGTACGACGAGGTCT

TCGACGTGGTCGACCTCGACCCCTTCGGCACGCCGATGCCCTTCGCCGAC

CCCACGTTCGCCAACACCCGCGACCTCGTCTGCGTCACCGCCACCGACAC

CGCCCCGCTGTGCGGCGCACACCAGAAAAGCGGCATCCGAAAGTACGGCT

GTCTCCCGCAGAACACCGACTACCACCCCGAGATGGGGCTTCGAACCCTC

CTCGGCGCGATGGTCCGCACCGCCGCCCGCTACGACAAGGCGGCCGTCCC

GATTCTCTCGCACGCGACGCGTCACTACGCGCGGGCGTATCTCGAACTCG

ACGAGCGGGCGACGAAGGCCGACGAACTCCTCGAATCGACCGGCTTCGTC

TACCACTGCGAGGACTGCCTCCACCGCGAGGCCGAGCGCTCGCTGGTCGC

CCACCCGCCCGAGGCGTGCGCTAACTGCGACTCGACCCGGATTCTCGAAG

CCGGCCCCATCTGGCTCGGTCCCGTCTCCGAACCGGCGTTCGCCGGGGCC

GTCCGCGACGAGGTGACAGAGGACTTCGGCACCGCGAAACGCGCCCGAAA

GCTCCTCGACACGCTCGCGGTCGAACTCGACACGCCGACCCACTTCGACC

AGCACCGCCTCTGTAAGCTCTGGGGCCGGTCGGCCTCGAAGATGGAGACG

TTCCTCGAAACCCTCCGCGACGCCGGCTACGACGCCACCCCCGCCCACTA

CCACGGCACCGCGTTCAAGACCGACGCGACGGTCGCTGAGATACGCGAGG

TGACCGCCGCGCTCGACCCCGAGGCCTGAGAACGACCCCGCGTAACCGAC

CTACGGCGTCACTTCGGCGGGTAGCTTCTTTGCCTCCCGTGTCGTTTCGG

TGATACGTGAGCGCCAACATCCGAACCGCCGTTCCCGTCGCGCGGGCGGT

CGTCGACACGATTCGGGAGAAGGAAATCTCGTTTCTCGCGGCCAGCATCT

CGTACTACGCGCTCGTCTCGCTGATTCCGCTTCTCGTCCTCGGCGTCGTC

GTCGCCACCGCCGTCGGCGGCGCGGCGTTGCAGGCACAACTCCAGACGCT

CGTCGAACAGTACCTCGTTCCGACGGGCCAGGGCCTCGTCGAGGAGGCGC

TTTCCGACCGAACCGGGCAGGGAAGCGTCGGGGCGGTGGGCCTCGGGCTG

ACGGTGTGGGGCGCGCTGAAGCTCTTTCGCGGCCTCGACATCGCCTTCTC

GCGCATCTACGGCTCGGAGGCGGGCGGCCTCCTCGACCAGCTCCGCGACG

GCGCAATCGCCCTCGCCAGCATCGGCGTCGGCACCATCGGCGTCGCGGTG

CTGACCGCGCTGCTCGGCCTCCTTGACGTGCCGTTTCTCCAACTGCTCTC

GCCGCTTTTGCTGTTGCTCACGCTCTGTGCGGCGTTCTTCCCGCTGTACT

ACGTCTTCCCCGACGCCGACCTCTCGCCGCGACAGGTCGTGCCGGGGACC

GTCTTCGCGGCCGTCGGCTGGACCCTCCTCGGGGTCGGCTTCGGCATCTA

CGCGTCGGTGGCCGGCGCGTCGGTCGCGGGCGCGCTCGGGACGCTCCTGT

TGCTCGTGACGTGGTTTTACTTCTCGGGGCTCATCCTGATTTCCGGCGCG

GTCGTCAACGCGGTCCTCGCGGGGGTCGGGGGCGACGCGTCGCCCGCAGA

ACCGCGTGACCCGCCGGGGGGACCGCGCGGAGTGGACCGGCAGGTACAAC

ATACGGGAGGCCGACATGACGCTCGTACGGATATGAGCGACGACCGAGGT

GACACCGATGGGCCGCGGACGGAGGACGCCGACGACGCGGAGACCGGCGC

GTCCGTCTCCCCGCGCGGCGCTCCCGACATCGAGGAGCTGGAATCGCAGG

TCGAGGAACTTCGGGCCGACCTCGACGCGTTCGAGGACGACGTGACCGAC

CGGACGGTCGAAAAGCCCAAGCTCGAAGCCGAGTTGAAGCGCTACGTCCG

CCGCCGGATGCGCCGCGGCAAGGCCCGCGGGTGGGGTCCCTACCTCGTGC

TCGGCTACGGCGTCGTCCTCACGCTCGGCGCGTTTCACTACCTCCAGTCG

GACCTCATCGCGGTCGTCGCCATGCTCATCATCTTCCTATCGACGCTCGG

ACTCTACGTCCTATTCGTCATCTTCGGCATCGGCCTGAGCGCCCTCGGCG

TCCCCGGGCGCGCCATCGACGCGGTCCGCAAACGCCGCGGCTGAGATGTT

CCGGTCCGCGCTTTCGCTTCAGACCCGCGGGTTCGGGGAGTTGGTGCTCG

CGGACGCCCCGGACGCGCTCGTCTTCCTGTTCGGTCTCGTCACCCAACTC

GGAGACCAGTGGTTCTTCTTCGTCGCGTTCACCTCGCTGTACTGGCTCTG

TCGGCCCCGAATCACGGCGCGCCCGCGGCGAACCGCGGCCTCCTTCGTCG

GCCTCGCGCTCGGGTCGCTGGCGCTCGTCACCGCGCTGAAAGTCGGATTC

GCCCTGCCGCGACCGCCGACCGCGGCCGTCGCCGCCGCGCCCGCGTGGCC

GTCGCCGCTTTCGGACCTGTTCGTCTCGTTCACGACGGACGACGGCTTCG

GGTTCCCGAGCGGCCACGCCCTCGGGACGACCGTCGTCTACGGGGCCGCG

GTCTCGCTTCTCGACGTGTGGGACCGTCGCCGCCGGCTCGTCGCCGCGGC

GGTCGTCGTCGGCATCGTCTCGCTCTCGCGGGTCTTCCTCGGCGTCCACT

ACGGTGTCGACATCGTCGTCGGCGTCCTCCTTGGCCTCGGCTTCCTGAAG

GCGGTGTCCGTGGTCGCCGCCGCGGACGACCCCGACGCGACCGGCCATCT

CGACCCGGCGCGCCTGTTCGCCATCGCGGCCGGCCTCAGCGTCCTCGCGC

TCGCGGTGGTGTTCGCCACGGGTCTCTCCGGGCACACCGAAAACGCCGCG

GCGGCGCTCGGCGGGTCGCTCGGCGGTCTCTTCGGCTGGACGCGACTCGC

CGGCCACGAGTCGCTCCCGACGCTGTCGCCGCCGGTCGCGCTCGTCGCGT

TCCTCGGCGCGGGCGGCCTCTGGGTCGGCGTCGACGTTGCCGACGCCTCG

GTTCCGGTGACGGTGCTCGTCACCGCGGCGGTGGTCGCGTTCATCCTCGT

CGCGCCGCGGATACAGGGCCGACTCGGGCTCGGAAACGGAGCGACCCGGC

GCGCCGACTGAGAGCCTGTTTCGGTGTGTTGCTCAGGTTCTAGGTAGTGT

TGTCTCTGGTTTCTCGCCGACTGCGATACCGGTGGCTGTCGCTCTCCGGC

GAGCGGGCGCGAAAGAAAAATCGGTGTTGGGCGTGCGCGACGCTTAGAAC

GTCTCGAGGTACCGGTCGAGCTCCCACTGGGAGACGTCGACGAGGTAGTC

CTTGAACTCGGAGCGCTTGGCTTCGACGAACTTCTCGAAGACGTGGTCGC

CCAGGGCCTCCTGGATGACTTCGTCCTCTTCGAGGGCGTCGACGGCGCCG

CCGAGGTCCTTCGGGAGCGTCTCGATGCCGTACTCTTCGCGCTTTGCCTC

GTCGAACTCGTAGATGTTCTCGCGGACCGGGTCGGGGCAGTCGAGGCCCT

TCTCGACGCCGTCGAGACCGGCGTGGATGAGCGCGGCGAACGCGAGGTAC

GGGTTACAGGACGGGTCGGGGAAGCGGGCCTCGATGCGCGAGGCGGCCGG

CGTGCGGGCGGCCGGCTTGCGGATGAGCGCCGAGCGGTTGCGGTCGGACC

ACGCGATGTAGACCGGCGCTTCGTAGCCGGGGACGAGGCGCTTGTAGGAG

TTGACCGTCGGGTCAGCGACCGCCGTGATGGCGGGCGCGTGGTCGAGGAT

GCCCGCGACGAAGCTCTTGGCCGTGTCGCTCAGGTCGAACTCGTCGTCGC

CGTCGTGGAACGCGTTCTCGCCGTCCTTGAACAGCGAGATGTGCGTGTGC

ATGCCGGAGCCGTTGATGCGCGGGATTGGCTTGGGCATGAACGTCGCGTG

GAGGTCGTGTTCGGCCGCGATGGCGCGGACGACGGACCGGAACGTTGCGA

CGTTGTCGGCCGTCGAGAGGGCGTCGTCGTACGTGAAGTTAATCTCGTGC

TGACCCTCGGCGACCTCGTGGTGCGAGGCTTCGATGTCGAAGCCCATGCT

TTCGAGGCCGTAGATGATGTCGCGGCGCACGTCGGACGCGAGGTCCTTCG

GGGCGAGGTCGAAGTAGCCGCCGGCGTCGTTCGTGACGGTCGTCGCGCGG

CCGTCTTCGTCTTCTTCGAACAGGAAGAACTCCGGTTCGGGAGCGACGTT

CACGTCATAGCCGAGTTCCTCGGCGCGCTCGATAGCGCGCTTGAGGACGC

CACGCGGGTCGCCGCTGAAGGGCTCGCCGGTGGACGTGTTGAACACGTCA

CAGATGAGACGGCCGGCGGCGCTGTTTTCCTTCTTCCGCCACGGGAGGAC

GGCGAACGTCGACGGGTCGGGTTCGAGGCGCATGTCGGACTCCTGGATGC

GCACGAAGCCGTCGATGGAGGAACCGTCGAAGTAGATGCCCTCGGTGAAC

GCCTTCTCGGCCTGCGAAGCCGGGATGGAGACGTTCTTCACCGTTCCGAG

AATGTCGGTGAACTGGAGTCGGAGGAAGTCGACGTTCTTCTCTTCGATTT

CGTCGATGACTGCCTGTGCCTCGTCGCTCAGGCCGCCGTCGGTGAGTGCG

TTGTCTTCCGTCATTTTCCTGAACACGCGATTCGAACACCCGCACTATAA

AGACATTTCCGCTTAATGCAATTCCCCGCACCTCGCCACAGAATTGGATA

TTCGTAAAATTCTAATGCCCCCGGACGGTCTTTGGATGTAATGACGTACG

AAAACCTCGACGCGAAGCTCATCAATGCGCTCCTCGGCGACGGTCGAGCG

AGTCTGCGAAGCCTCGCCGAAGAGCTCGACGTGTCGGTGACGACCGTCTC

GAACCACCTGCGAGACCTCGAAGACGAAGGCGTCATCGAGGGCTACACGC

CGCGGGTGAACTACGACGCGCTCGGCTACGACGTGACCGCGGTCATCCAA

CTCAAAGTCGAGGGCTCCGCGCTCCCCGAGATTACAGAGCGCCTCCGCGC

CGAAAAGCAGATGATTTCGGTGTACGAGGTCACCGGCGACTACGACATCA

TCGCCATCGGCAAGTTCCGCGACACCGACGGCATGAACACGCAGATCAAG

AAACTGCTCACCGACGCGGACATCCGCGAGTCCAACACCTCGGTCGTGCT

CAACGCCGTCACCGAAAACGAGCAGTTCGACCTCGACCTCGAAGAGTAAC

GCCGTCAGACGATTCGCGTTTTTACGTCGCGCATCTCCGACTTGCAGTAG

GGACACCGATATCGCGTGTCGAAGCCGTCGGGCGTCGTCTTCGTCGTCGT

CTCTATCTCGTGGACCGCCACGTCGCGTCCACACTCCCGGCACTGAACCG

CTGGCATCGGTTCGTGTTCGTCGACTGGGCGGAATAAATCTACTGGCGTG

TGTGTATAGTTATCATCAAATTGTCTGTATCCGGACGGGAACCGCTTCGA

CTGGAGAATCTACGGTTTTCGGTCGATACGCTCCGTCCGAACCCCGCGTT

TCCGGGTCGATTTCGGCGGCCGTTCGAAGGCTACTGTTTCGACTGGAAAA

CCCGATGACGTGGCGCGATTCCTCGGTGGATAAACTGGAGACGAAATGTT

GCGAGTTTTCGCTGCCCGGCGGAGCCGGCGGTCCGGTCAGAACCCGTCTT

CGAAGACGAACGTGCCGTCTCGCTGGACGACCTCGCCGTCGACCTCGATG

ACCGAGTCCTCGGACATATCGACGATCATGTCGACGTGCTCGGCGCTCTC

GTTGAGTTCGTTGTCCGCGCCGACCGTCTCGGGGTACGCCGAGCCGACGG

CCATGTGGACGGTGTCGCCCATCTTCTCGTCGAACAGCATGTTGTAGGTG

AACTGGTCGATGCCGCGGTTCATGCCGATGCCGAGTTCGCCGAGATAGCG

CGCGCCCTCGTCGGTGTCGAACACGCCGTCTAAGACCTCCTCGTTGCCCT

CGGCGGAGTAGTCGACGACCTCGCCGTCCTCGAAGCGGACGCGGACGCCG

GAGACCTCGCGGCCCTGCCGGTACAGCGGCAGGTCGAAGTGGACCTCGCC

GTTCACGGAGTCCCTCACGGGCGCGGTGAACACCTCGCCGCCGGGGAGGT

TGTGTTCGGCCGTGTCGTTGATGGCGGTGTTGCCGGCGATGCTCATCGTC

ACGTCGGTCTCGTCGCCCGACGTGATGCGGACCTCGTCGCCGTCGTTGAG

GATGTCGACCAACTGCTGTTGGAGTTCGCCCTGCGCGTCCCAGTCGAGGG

TGACGGCGTCGTAGACGAAGTTCTCGTAGGCTTCGGTGCTCATGCCCGCG

AGCTGGGCGCTCCCCTTCGCGGGGTACTGGGTGAGACACCAGCGCTTCGA

GAGCATCTCGCCCTGCACGGGTTTGTAGGCGCGCTTGTACGCGGCGTTCG

TCTCCGGCGGCACGTCGCTCTGTTCGGTCGCGTTCGTCTCGCCGCGGACG

CGGATGATGACGTCGGTCTCCTCGTAAAGCGCCAGCACGTGCTCGGGCGT

CTCGTAGTCGTCGTCGCCGGCGCGGAGGAACGCGCGGGTGAACCGCGGGT

TCGCGTCCATCGCGACGACGTTCGCGCCGCGGTCGCCGAGCAGTTCGTGG

ATGGCCGTGACGAGGTCCTCGGCCACCGAGGGCGCGGAGACGACGACGTT

GTCGCCGGCCTCGACGCTCGTGGAGTGGTCGACGATGGTTCGTGCGTGCT

CGCGGATGCGCGGGTCCATACCGCGTCGTGTGTGCCCCCGGGGCAAACAC

CTTTCGAAGCGCAAATATTGCGGCCGCGGCCGAGAACGCCCCGCGGACCA

CCGATTATTTCTCCGTCGGCGCGGTCTCTCCGCGCATGATTGACCTCCGA

AGCGACACCGTCACGCTCCCGTCAGACGAGATGCGCGAGGCCGCCCGCGA

CGCCGCCGTCGGTGACGACGTGTACGGCGACGACCCGACCGTGAACGAAC

TCGAAGCGCGCGCCGCCGAACTCGTCGGCAAGGAGGCCGCGCTGTTCGTG

CCGTCGGGGACGATGGGCAACCAAATCGCCGCCCGCGTCCACGCCGACCC

CGGGCAAGAGGCGCTCGTGGACGAGAAGGCGCACGTCTACGAGTGGGAGG

TCGGCGGCTTCGCCCAACTGTCGGGCTTGCAGGTCCGCGCCTACGACGCC

GGCGAGCGCGCCGCGCCGACGCCCGCGCAGGTCCGCGACCACGCCCGCGA

GGAGTCGCTTCACGTCGCCGGCACGGGCGTCCTGTGTCTCGAAAACACCC

ACAACGCCCGCGGCGGCGTCGCGGTTCCGAAGGCCGATATCGACGCCGCG

GCCGACGCCGCCCGCGACCTCGGGATTCCCGTCCACCTCGACGGCGCGCG

CCTGTTCAACGCCTGCGTCGCCCTCGACGAGGACCCGACGGCGATGGTCG

AGCGCGTCGACACGGTGATGTGCTGTCTCTCGAAGGGCCTCGGCGCGCCC

GTCGGCTCGATGCTCGCCGGTCCCGAGGCGTTCATCGAGGAGGCCGTCCG

CGTCAGAAAGCAGTTCGGCGGCGGTATGCGTCAGGCCGGGCTCATCGCCG

CGCCGGGGCTCGTCGCGCTCGACAACGTCGACCGCCTCGCGGACGACCAC

GCGAACGCGACGGTCCTCGCCGAGGGTCTCGACGCCGTTTCCGGACTGTC

GGTGCCGACGCCCGACACGAACATCGTCGTCGTCGACTCCGAAGGTGCCG

GGCTGACGGCCGAGGAGTTTGTCGAACTGTGCGACGATGTTGGCGTCCTC

GGCGGCACGTTCGGCCAGTACCACACCCGCTTTACGACGAATCTGAACGT

CTCGCGTGCGGACGTGGAGCGCGCGGTCGACCTCGTCGCCGACGCGGTCG

AATCGCGGTAGGTCGGGGGAAGCCGCCGCGAGTCGCGGTCGGCCGGTGGC

GGCCCACTCGGTTCGTTCGGATTACAACTCGGGTTCGATGTAGACGAAGT

GCACCCGGTCGTCGGCCGCCTTGAGTTCGTCCTCGATTTCGGTGATGTGG

TCGTCCAGTTCCTCGGTGTCGAGTTCGGGGTCGAAGCTCACGTCGAGGGC

GACGAGCAGCTTCTCGGGGCCGACGTAGCTCGCGCGGAAGTGGTCGATGT

GGACGACTCGCGGGTCGTTTTTGACGATGTCGCGGAGCGCCTGTTCTTCG

TCCACGGGGACGCTCTCACCGATGAGCAGGCGTTTGTTCTCCCACGCGAG

CGCGATGGCGAAGCCCATGAGCAGTATCCCGATGAGGAGCGCCGCGCCGG

CGTCGAACAGCTCGTTGCCCGTGAGCTCGGTGAGGCCGATGCCGGCGAGC

GCGAGCACCGCGCCGACGAGGGCGACGGTGTCCTCCGTGAACGCCGTGAG

CGTCGTCACGTCGCTCGTCTCTCGGAAGGCCTCGACGAGGCCGCTCCACT

CGTATTCTTCTATCTGCCGGCGGAGTTCCGCGTTCGCCTTCAGGAAGGCG

TAGCTCTCGAAGGCAATCGCCCCGAGGAGGATGGCGACGTTGACCAACAC

GGGCTCGATTTCGAATCCGAGCAGGACGAGCGCCTCGCCGCCCCCGCCGT

GGCCGGGGTGCATCAGGGCGTCGTAGCCGTGTTTGGCCGACTCCCAGCCG

GCGATGCCGAACAGCATCACGGAGACGAGAAACGAGTAGAAGAACTGCGA

CTTCCCGTGGCCGAAGGGGTGCGACTGCGTCTCGCCGCGCTTGCTGTCGC

GGATGCCGATGAGGAGAAACACCTGGTTGCCGGTGTCGGAGATGGAGTGA

TACGTTTCGGAGAGCATGGAGGGACTCCCGGTCGCGAGATAGCCGAGGAA

CTTGAGGACCGCAATCACCCCGTTCGCGAAGAGCGCGGCGACGACGACGG

ACGTACTGCCTGCCATGTCGTGGGCCACTCCCCCCGACCGGATAGAGCTA

CCGACGGCGAATCGAGAGACGGACCATCCTCGACGGGCCGACGGGCGGAC

CGACGCCGCAGTCGGTAGGTTTCAATCCCTCCGGTCCCTAGCCCGCGCAT

GCTCGTCGTCGTCTCCGACACCCACAGCACGGACGGTCATCGCCTGACCG

ACAGAACCCTCGACGCGGTCCGCGAGGCCGACCTCGTCGTCCACGCGGGC

GACTTCATGCGCGAGTCCGTCCTCGACGCGTTCGTCGACGAGGCCGACAG

CTTCCTCGCGGTGTACGGCAACAACGACGGCTCCGAGATTCGCGACCGGA

TTCCCGCGGCGCGGAACGTCATCTACGGCGGCGTCGAGTTCGCGGTGACG

CACACCCGCCGCGGCGGGAACACCGCGCTGTCGATGTTCGGCCGCGAGCG

CGGGGCCGACGCGGTCGTCTTCGGGCACAGCCACCGGCCGGGTTTCCACG

GCACCGGGCCGATTCCGCTCCTCAACCCGGGGAGCCACGCCCAACCGCGG

GGCAACCGCAAGGCGCACGCGGAACTGGAGGAACTGCCGGACGGCGGCCT

CCGCGGGCGACTCGTCACGGTCGACGGCGAGACGTTCGAGACGTTCCGTA

TCGTCCCCGACGAATAGGGGAGGTCCGGTGGAGGGCCGGAAGAAGCAGCG

GGAACAGACCGGAGCGGGTCTGTTGGGGGGCAGTCCGAGGCCGCGCGAGA

CGACGGGGAGAGGGGCAGGGGGCACGGATGGCAGCCCCGTCGTCGTCGGG

GGCCACGCGCGACCTCGCACTCACTCCTACGGGTGACGGTATCAAATACG

CACCGTAAGCTCAAACAGCCGTTTTAGTCACGCAGCACGCCCACCGCGAC

CCACGCGAGAAGCACGGTCAGCCCGCCGAGGAAGAACAGCGTCCCCGGCG

ATAGCGTCCCGATGGTCTCGGCGAGTCCGCCCGACCCGGACGTGACCCGC

GCCGCGGTCTCCGTGGCCGTCTGCGCGGCCGTCGTGTCCGGAACCGGCGT

CTGCGTCGGCGTCGCGGTCACGGTCTCCGTCGCGGTCTGCGCGGCTTCCG

TCACGGTCGGCGTCGACTCGACCGCGGTTCGGGTCGCTTCCGCGGTCTGC

GTCGTCGAATCCGCGGTCGTGGTCGCCTCGGCGATGCTGAACCCACCGCC

GCCGTCGGTCGTCGCCGTCTCTGTGGCCGTCCTCGTCGGGGTCGATGCCG

TCTCGGCGGTCGCGGGAGTGGCGTTCGCCGAATCGGCTGTCCGGGTCGCG

TTGAGGTCGGCTCCCGAGCCGCCGCTCTGGCCGGCACCGGAGTCGCCCGC

GCCGCCGGTCGAGCCTCCGAGACCGAACGGAAGCATCGACCCGGCACGGG

CGAGCCGGTTCACGACGGCCGCGCCGATGCCGAGGACGCCGACGCCGCCG

ATGAGGCGCGTGAGCGCGCCCTTCAGACCCTTCGTCTCGTCTTCGCGCCC

GGCGACGACGACGAGCGCCCGGTCAGCGGGCGTGTAGACGTTCATCTCGC

GGCCCTTCTCGGAGTACCTCGTGTCCGCGACTTCGATGAGGCCCGCTTCC

GAGAGGTTGCCGAGGTGGTACTGGACGTTCTGGAGGGACGTGTCGATTTC

GGCGGCGAGGTCGGAGGGCGTCGCCGGCGACTCGTGGAGCGACGCGAGGA

CCGTCCGGGCGGTCGACGAGGAGATTGCGCCGAGGAGGTCGTCCGCCTCG

TCGCTGTCGAGGCCGATGACCTTGGGGTCTCGGTCGTCCCGGTCGTCGTC

CGGGTCGCCCGGGCTGGAGGGGATGAGGTCGGCCATCGAATCGGCGTTAC

TCACCGCTTCCCATGAGTGTTCGTATTCTTTCACGACACGTCGCCGAAAT

TGCTATACCTCGTGCCCGCCGAACCCACCCACATGACCGACCCTTTGGTC

GTAGACGGCCTCGTCGACTTTCTCGCGGGGAATCCCGTGTTCGTCGGGTT

GCTCGCCGCGTTATTGCTTTTCGTCTTCTTCGGCTATCTCCTCGTCCGGC

GGACGCTCCTCGGGCTGAGTGAGGGCTACGACGAGGCGCGCCGCCGCTGA

CATGGACCGGACAACTGCCGACGACCGCCGAGGTGTCGCCCCGTGGTAGC

CGCCGGAACGCTCGCAACGTTCGTCGTCGCCGCCCTCGCCAGCCTGTTCA

TGGCGTGGGCCATCGGCGCGGGGTCGTCGGGGTCGACGCCGTTCGCGCCC

GCCGTCGGAGCCAACGCCATCTCGGTGATGCGCGCGGGCTTCATCGTCGG

CCTGCTCGGCTTCGCCGGAGCGTTTCTGCAAGGTGCGAACGTCACCAACG

CGGTCGGGACGGAACTCATCGGCGGCGTCACGCTGACCGCGGGGGCCGCG

GTCGTCGCGCTCCTCACCGCGGCGGTGCTCGTCGCAATCGGCGTCTTCGC

CGGCTACCCCATCGCGACCGCCTTCACCGTCACCGGCGCGGTCGTCGGCG

TCGGCCTCGCCATGGGCGGCGACCCCGCGTGGTCGAAGTACCAACAAATC

GTCTCGCTGTGGGTGCTCACGCCCTTCGTCGGCGGCGGCGCGTCGTACGC

GACCGCCCGGCTCCTCCGCGCCGACGGCGTCTCCGAGCGGTGGACCGTCC

CCGCGCTCGCCGGCCTCGTCGGTCTGCTCGTCGCCAACATGGAGTTCGCG

GGACTCGGCGGCGCGTCGGGGTCGGCCTCGGTCGCCCGGGCCGCCGCGGT

CGAACTGACCGGTCTCGGCGGCCTCGGCGAGACGGGCTGGTTCGTCGCCG

CCTCGCTTCTCGTCGCGCTCGCCGCCGCCGCCGTGGTCGGCCGCTGGGTG

ACGACCGACAAGACTCGCGGCCAGCGGCGCTTCCTGCTCGTTCTCGGCGG

CCTCGTCGCCTTCTCTGCCGGCGGCAGTCAGGTCGGCCTCGCCATCGGGC

CGCTCGTTCCCCTCCTCGACGTGGTCGCGGTCCCGCTCCCGGCGCTCCTC

GTCGGCGGCGGACTGGGCCTCCTCGCGGGGTCGTGGACCGGCGCGCCGCG

CATGATTAAGGCGCTCGCGCAGGACTACTCCTCGCTCGGGCCGCGGCGCT

CCATCGCGGCGATGATTCCCTCGTTCGCCATCGCCCAGACCGCCGTCGCG

CTCGGCGTCCCCGTCTCCTTTAACGAGATTATCGTCAGCGCCATCATCGG

TTCGGGCTACGCCGCCGGCGGCTCGGGGGTCTCCGGGCGGAAGATGCTTT

ACACCGTTCTCGCGTGGATTGGCTCGCTCGCCCTCGCGCTCGGACTGGGC

TACGGCGTCTTTACGCTCGCGTCGAGCGTACTCGGCGTGTGAACGTCCGT

GCGCTCCGCGTCGCGCTTACCACCCTCGTGTCCGCGCTCTGGGCCGCCTT

CGGCGTCTACCACGTCGCGATGGCGGTCGTGACCGGCATCTCGCTCCGCG

GGTTCGCCTTCGGGACCGTCGGCCTCGGCGCGTTCGCGCTGGCGATTCGC

TTCCGCGGACGGGCGCGACGGCTCGGTGACGGCACCGCCGACGCGACCGG

ACTGGTGGTTCTCGCGCTGGCGTCGCTGGTGCTCGTGGTGCTGTCGGTTA

CGCTCCTCGAATAACTCGCGGTCGGAAAACGGGTTCGACCGGGTGTCGGC

GGTGGCGACTCCGTCGCGTCGTCAGGTCAGGTCAGGTGTCGGGTTCGACT

TCCGCTTCCTCGTCGCCGCCGTTCTCCTCGTCGTCGCCGTCGCCGTTGCC

GTCGGCGGCTTCGATGCCCGCGTTGGTCCGGATGCGGGCTTTCATGATGC

GCGTGTTGTCGACCTGCTCGATGCGGATGACGATGTCGTTGTAGCTGATC

TCCTCGCCCTCCTCGACGAGGCGGCCGGCGCGGTTGAAGATGAAGCCGGC

GAGCGTCTCGAACTCCTCGCCCTCGGGGAGTTCGATGTCGAGCATCTCGT

TGACCTCGTCGATGTTGACCTCGCCGCGGACGAGGTAGGTGTTCTCGTCG

ACCGACTCGAACGGCTCTTCCTCGTCGCCTTCGAGGATGTCGCCGACGAT

TTCCTCGACCATGTCTTCGAGGGTGATGAGCCCCTCGGTGGTCCCGAACT

CGTCGATGACGACGACCATCTGCATCCGGGTGTCCTGCATCTCGGCCATC

AGTTCGTCGACGTTCTTCGACTCGGGGACGTGGAGCGTCGGCGAGACGAT

GTCGGCGATGCCGACGCCGCGTTCGCCGTAGTACTTCTCGCGGACGAGGT

TTCGGATGTTGACGATGCCGATGATGTTGTCGAGGTTCCCGTCGTAGACG

GGGACGCGCTCGTGGTCGGCCTGCACGCACGTCTCGATGGCCTCGTCGAT

GGTCGCGTCCTTCGGGACGGCGGTCATGTCGAGGCGGGGCGTCATGACCT

CCTTGGCGATAGTCGAGTTGAAGCGGAAGATGCGGTCGAGCATCTCGCGT

TCTTCCTCCTCGATGACGCCCTCGCGCTCGCCCGTCTCGATGAGGTTCTG

AATCTCGTCGCGGGTGACGTACGTCGTCTCGATGGCGGTCTGCCCGCCGG

TGACGCGGTTGACGAGCCGCGTCAGGTGGTCGAACAGGACGACGAGCGGG

AGCAACAGTAGCTCGGCGTACTTCAGGGGGCGTGCGATACGGAGCGACCA

CGATTCCGTGTTCTCGACGGCGTATGACTTCGGTGCGCTCTCACCGAACA

GGAGGACGAGCGTGGTGATACCGAACGTCGAGATGAGGACCGACTCGCCC

GCGCCGAAGTCGAGGACGGCGAGGAGCCCGGTCGCCAGCGACGACATCGC

GATGTTGACGATGTTGTTTCCGACGAGGATGGTCACGAGCAGGCGGTGCG

GGTCGTCTTTGAGCGACTTGACCCGCTCCGCGCCCGGAATCCCCTCTTCG

ACCATCGAGTCGACGCGGTGCTTCGCGAGCGAGAACATCGCGATCTCGGA

CGAGGAGAAGAACGCCGAGAGACCGACGAGAACGATAATCGCAGCCACGC

CGAGGACTGTGATCGTCGTGTCGTCCAGCGGAATATTCGAGACCTGTGCG

GCCGCGAGTGTCGAGGCTGGTGGTAACTGTGTCGGCGGCAAACCCATGTA

ACTACACTGTCTTGCGCGGGACCGGAATTAAGAGTTGCCCTCGACACTGC

CTCGGCCGGCGCGTCGTTCGTTCTCCCCGGTCCGGCCGTCGAACCGATGT

TGCGGCGCGGATGCCCGCGAGCGAAGGGCTTAGGCGACGCTCTGCTCAAC

ACCCGCCCATGGCAGACGAGCAACCGGCGATTACGCTGTACCGACTGCAA

GCGTGCCCGTTCTGCGAGCGGGTCGTCCGCACGCTCGACGAACAGGGTCT

CGCCTACCAGTCGCGGTTCGTCGAGCCGATGCACTCCGACCGGAACGTCG

TCAAGCGCGTCTCGGGCAAGCGCTCGGTCCCGGCCATCGTCGACGACAAC

ACCGGCGTCACGATGTCCGAGTCGGCGAACATCGTCGACTACCTCGAACA

CACCTACGGGGAGGGAGCTTGATGCCGGAGTTCGACGTCGTCTCCCTGCC

CGACCACGACGCCCCGTCCGTGGGCGACACCGCCCCGGACTTCACCCGCC

CGCTCGTCAACGCGGAGTTCTGGGAGGACGCCGCCCTCTCGGACCTCACC

GACGACGGGCCAGTCCTCCTCGTGTTTCACACGATGGCGGGCGCGTTCCC

CGCGACGTACGTCTGGAACGAACTCCGCGACCGCGGCGTCCCAGACGAGG

TACAGACGGTCGGCGTCTCCATCTCGTCGCCGTACGAGCACAAGTCGTTC

CTCGCGGAGCGCGGCGTGGACGCCCGCCTGTTTTCGGACCCCGCGGCCGG

CGTCGCCGCCGACTACGACCTCGAACACGACCTCGACGGCATGGCCGGCG

TCACCGAACACCGCCCCGCCGTGTTCCTCCTCGACGAGGAGCGGACGGTC

CAGTACCGCTGGGTCGCCGCGGAGTGGCCGGACTTCCCGGACTACGAGGG

CATCGCCGACGCCCTCGACGACCTGTAAGCCGCTTCCCGACGCTCGCCGC

GTCGACCGCATTCGCCCGCGCGGAATCGCCGTTTCGTCACGCTTTTTCGC

CCGTTGCGCGTCGGTTCGTACATGAGCGACGACGTACAGCGTGCCGCCGC

CGCCATCCGCCGCGGCGAGGCGGTCGTCTACCCGACCGAGACGGTCTACG

GCATGGGCGCGGACGCGACGAACGCCGACGCGGTCGAACGCGTCTTCGAC

ATCAAGGGCCGCGACCGAGACAACCCGCTCTCCGCCGGCTTCCCCGACGT

GGACGCCGCGCTGGAGCTCGTCGAGGTCGACGACCGCGAGGAGGCCTTTA

TGCGCCGGTTCCTCCCCGGTCCGGTGACGGTCGTCGTGGAGCGACGCGAC

AGCCTGCCGGACGCGCTCGTCGCCGGGAAAGACCGCATCGGCGTCCGCAT

CCCCGACCACGAACTCGCGCTCGCCCTCTTTCGCGCGTCCGGGACGCCCG

TCACCGCGACCAGCGCCAACCGCTCCGGCACCGGGAGCATCACGCACCCC

TCGCAGCTCTCCGACGAGATTCGGGAGGCGGTCGCCGTCGTCCTCGACGG

CGGGACGACACCCGGAACCGAGAGCACCGTCGTCGACCCCGGCCGCGGCG

TCGTCCACCGCCGCGGCGCGATGGCCGACGACATCGAGGCGTGGTTAGAC

GAGCACGACGACGACGCGTAACCCGACGGGCGCGACGACGAGGCAACCCT

GAGCCACGCGGACGCGACCCGGAGTCACCGCGGACGCCGCCCTCATCGAC

CGAGTAACGACGACAGCGACCACGTTTTCACGCCGCAGCGCTCCCGATAG

GTACAGGACTCGCATTTCGCGGAGTCGCGGAGCCGCGACGGGACGAAATC

GAGGTCGCGGGCGACGCGGAGCGCCCGCCGATAGGCCGCCTTGTTCCCGG

TCGTGAGCCCGACCGTCCGGACGACGCCGACCGCCGGGTACTCGACCAGC

GCCCGCGGCACCGGTGCCTCGCGCTCCCATGCGAGCGCCTTCGCCAGCGC

AACGGCCCTGACGCGCTGGGGCTCCCAGACGCCGCGCTCGGGCGGTTCGC

CCGGCGAGACGAGCGAGGGAACCGGCACTGGCGGTCGGTCCCCCTCGCCG

TCGGGCGCGCCGACGAGTTTGTGCGCGACGCCGCGGCAGTCCTTGCCCGC

GAGGAGTTCGTCGCGGGCGACGGGGTCGGTGAGCGCGTCCCAGTCGTCGC

GCTCGCGGAGTCGGGTGAGGCGCTCGCGGTACGCCGCGGGCGACACCGAA

ATCGGCCGCGACGCGAGTTCGTCGTCGGTCGCGTCGAGCAGGTCGGGGTA

GTCGAAGGCGAGCGCGCGAACCTCGCGGACCGCGGGCGGCGGCGTTCGGT

CGTCGTCGCGGCGGGCGTAGTAGAGCTGTCGCGGGCAGTACGCGGCGCAA

GCGAGGTCGGAGGCGGCGACGAGAGCGGACACGGGAGGGCTGGACCCGGT

ATCGGACAAGAACGTTCGCGCGAGCGCGCCGTGGAGGCGGCGGGTTCCGA

ACCGGACTCGCGTCGTCTGATTCGCCGCCGTGCGTGAATTAATACGGTCG

TGTCCCTTGGCTTACTGACATGGCATCGACCGCCACCGGCTCGTGGCTCC

GGATGACGCGGCCCGAACTCGCCGTGTTCGTCTCCGGGGTCACGAGCATG

GGTCTGGAGATTCTCGCCGGGCGAATGGTCGCTCCCGAGTTCGGCAGCAG

CATCTACACGTGGGGGAGCATCATCGGCGTCTTCCTCGCGGCGCTGAGCC

TCGGCTACCACCGGGGCGGACAGCAGGCCGAACAGGCGTCGAACGGCGCG

CTCGCCCGCGTCTTCATCGGCACGGCGGCGTACATCGCGGGCGTCATTCT

CCTCGGCGACCTGTTCATCCAGTCGACCGCCGGCCTCCCCGTCCCGAGCC

GATTCGCGTCGCTCCCCGCCATCACGCTCCTGTTCGGGCCGCCGACCTAC

TTCCTCGGCTTCATCAGTCCCTACGCGGCGGAACTCTCCGGGCGGACCGA

CGTGGGGTCGGCCTCGGGGCACGTCTACGCCGTCGGCACCGTCGGGAGCA

TCATCGGCGCGTTCGCCACCACGTACGTTCTCATCCCGTCCCTGAGCGTC

ACCAACATCGGACTCGTCTTCGGCCTCGTCTCGGTCGTGACGGCGCTCGC

GCTCGTCCGACCCGAAATCGGTCGAGAGGAAGCGGTCTGGAGCGTCGGCA

TCGCCGCCGTCCTCGTCCTCGCGTCCGCGACCGGGGGCGTCGGTCTCGAC

TCCCGCGGCAGCACCGTCTACCACACCCAGACTGCCTACCAAGAGCTTCG

CGTCGCCGACGCGGGCGGGACGCGGACGCTCTACCTCGACGGCCAGCCCC

ACAGCGCGATGGACCTCGACGACCCGACGCGGCACGTCTTCGACTACACG

TCGTACTTTCACGTCCCCTTCCTGTTGTCCGACGATATCGACCGCGTGCT

GTTCGTCGGCGGCGGCGGGTTCACCGGCCCGCGCGTGTTCTTGGAGAAGT

ACCCGAACGTGACCGTCGACGTGGTCGAACTTGACCCCGAAGTCGTCGAC

ATCGCCGAGGAGTACTTCCGCGTCGAGGAGTCGCCGCGACTCAACGTCCA

CACGATGGACGGCCGGCAGTATCTCCGGGAGACGAACCGGACGTACGACC

TCATCGTCCTCGACGCCTACCAGAAAGACAAGGTCCCGTTCCAGCTGACG

ACACAGGAGTTCATGCAGCTCACGTCGGACCGCCTCGACGACGACGGCGT

CCTGTTCGCCAACGTCATCTCCGCGCCCAGCGGCCCGGCCTCGCAGTTCT

ACCGCACGGAGTACAAGACGATACAGTCGGTCTACCCGCAGGTGTACAGC

TTCCCCACCGCCGGGCGCGTCGTCGTCCAGAACATCGAGGTCGTGGCGAC

GAAAGACGAGACGCTCGTGACCGCCGAGGAGCTTCAACGTCGGAACCGGA

ACCGGAATATCGGCATCGACCTCGCGGCCGAACTCACATCGTACCGGAAC

GACGAACCCACCGACGACGTGCCGCTCCTCACCGACGACCGCGCCCCGGT

CGATAGCCTCTTGGACCCGATGGTCGGCCAACGCTACGTCGTCCAGCGGA

CGAACGAGACCGAACAGCGCGCGGCCGACAGGAACGCCACTCCCCGGAGC

GCGGTCGCCGGCACGGCCGTGAGGGCGGCGTAGGCGATGGTTCACGACAG

AATCCACGCCAGAGAGCCGACGCACGACCGCGACCGCTGGACCGAGGGCG

TCGTCGAGTCGACCGTCGAACGCGACGGCCATTGGGTGGCCCGCGCGCGC

CCGGCCGGCAGCGACCGACCGGCTGACGAGACGGTCGAACGTGCGGGCGA

CGGCTCGGTCGAACTGGTCGTCACCTTCGCGGTCCGCGACCTGTTTCTCC

GCCGACTCGATGTCGACGCCGGCGAGTCGCCCGTCGGCTGTCGGTTCTGG

TACCGAAAGAAGCGACGGTAACTCCGCGGCCGCGACCGCAAACTCGACCC

GACGGCCTGAAACACGGGTCGGCCGGGGGTCGATGCCGGTTAGTTCGTCG

TTTCGAGGGCTGTTTGATACATGCGCGGCCCGGCGACGGCGGCCGTTCCG

AGCACCATGAGCGAGGCACCGACGCCGAGGCGCTGCCCCGGGGCGACCGC

GGACGCGACCACGCCGGTCACGACCGTGAGGCCGGCGAGGAACATGACCC

CGCCGAGAACATCGAGCGGCTGGATTGACATCTCAGTCGAGACGTTTCCC

GCTCGTTGCAAAAGTCTTCCCGCCGCCGTCTCGTGGTTCTCGTTCCTGCG

GCCCTCTCCCGAACACGACATGCGGCACTCACGTATCAGGTCAGAGTCAA

AATGGGACCGCCCGGGCTGTCCTTACCTATCAGTAGCCGACTCTCCACTA

CCTCTATCGGTCGAACTGAGATATTCCTACTCATGACTCATTCAATCCGG

AGTTTGTCCAGGTGAGGACGGCGGCGCAGCTCCATCCGTTCGCGCTGGGA

CGCTTTGTCGTAATGCTGTTCAATAACCTCTTGAGACGCATTCACGCGCT

CCGCGGTCACCTCCGCAGGGAACCCGCGATCTCTGTGCCACGTAATCGAC

CCCGTTCGAATGTGGTGTGGTGATCGAGAAGACGGACACTTGCTACCCTG

AGTGTATGACCCCGCGAACTCGCAGGAATCTTTCTCGTACTCGTGCGGGC

ACGGACCAGCAACACACGGCTGCGTCGCCTGATACATCCACCCCCGAAAG

CCGTTCTTCGAGATACGAGTTCCGTGGTGCGTCACGAACAGCGGAGAGCG

ACCAAAGTCGTCGTGGCCATCCGGCCGGAACTTCTCGATGTAGGTGTTCA

GCGACTCGACGACCTCCGGCAAGAGCGAAACCGTACGTTCGCCATTCATC

TGATTCTTCAGCGGCGTCTCGGACTCCGGCCGGTGGACGAACTCGACGTA

CTGCTCATCGGGGTAGTAGTCGCGCAGATCGAGCCCGCGAAGGGCACCGA

GACGAGCGCCCGTGTGCCACGCGAGTTCGAGAACCGCGTGGAACCGAGTC

CCGCGGACGCTACTGTGTGACCGGTAGTACCGAAGCAATCGAAGCGCGTC

GTCGGGAACCAACATCGTGTCTCGCGACCGCTCCGACATCGGGACACTCG

GTACGTGGACCTTCTTCGAGAGACCGTCCTCGACCGCCTCGATGCGCTCG

AGGTACTCGATGAACCCGAGCAGCGTCTCCATCTCGCCGTGGAGAGTGGG

TGAGGCGATACCCTGACCAGCGCGAAAGGCCTCGAACTGCTCGAACGTCC

ATCCGTTCAGGTCAGAGACGCGCTCGATATTCTGGTCCTCGCACCATTCG

ACCCAGAGTTTCAGACGGTATCTGTAGGTCTTGATGCTCGACTCCGAGAG

TTCCGTCCCTCGACGGTCAAGATAGCGGCGAACGGCCTCGCGGGGCTTGA

GTTCTGAGGGTCCGTTCATGGCTCACCCCACCCGTCGAAGACAGACATGG

CGCATCGCCAATGCAGGTAGTAGTCGTCGACATCGTTCCGGTCGGCGGTG

AACCTGTACTCCGCTTCGACAGAGACGTAGTCTTCAGACGGGTCGACTTC

CATCCGACAGACCGCACACGTCGGGTAGTACGTCATCTGCATCCTCCGTC

GTGCTCGGGGCAGACCGGAACCATTCGCGGATAATACTCGTCTTCGGTCG

AGAGATCGAAGCCGTCGCACTCCGTCGGCTCCAGAACTCGCCCGCAGAAG

TGACACTCAACCCACTCGGTCAGCGGATCGATGAACGCAGGACGAGTAGA

ATCGACTTGCCCGCCGTCGGTCACGAGCTGGCGTTCGGCGCGACGACACT

CAGAACAATGACCGTCGTCGTCGAGACCGCGGAGTCTGTCACACGCGCGG

CAGGGACGCCTGCACGCGGGGTGACTCATCTGTCGACCTCCAGCGCCGCG

CACTTCCGGCAGGCGCTCAGGCGAGTCTCGGCATCGACGCCGACCGCCGG

GCGGAGGAGACAGACCTCACACGTGGACTCGTCGCCCTTATCAAACTGTG

CTGCATCGGTCGCTATGCGGGTGGTAACACCCGTGGTTTTGGCTTCCGTC

ATGGCTATTGACGGACCCTTCCTGAGAGCGGTGCTGGACCCACCGCGCTC

AGGGCCTACAAAAATCATCTCCAGCCGACGGTGTCTCTCAGGGTTCCGTG

AGTCTACCATAACACTAACTGTGCATAAATGCACCGTTAGTTTGAGAACA

GATGTTTGGATTGCTAAAGCACGATTCGTCGGCAATAGACCAAACCAGCG

TATACGTACACGTAATGAGAAAGTCTGCATCGTGGATGACGATATGGGAC

GACCGAATCCTCGAGTGGGCACGGGAACACGAGTCGGCGAGCGCCGCCGC

GATGAAAGACACCGAATACTTCCAGGTATCTCGTTCGTCGCTCTCACGGC

GGCTCTCGAAGCTCAACGAGAAAGGGCTACTCCAGCACCTCGGCAATGGT

GTCTACGTCATCACGGCTACCGGCGAAAGCTATCTCGAAGGTGAACTCAG

CGCTGAAGAACTGAACGGCGAGAATGGTGAAGAGGGCTCTGCGTCCGCGT

GACTCACTCTGAGATGCGGAAGTCAGGTGACTGGATGACGATCTGGGATG

ACCGGATTCTCGAGTATCTCCTCGAGCACGGCTGGGGGTCTCCGGAGACG

ATCCATTGGGAAATCGGCCGCGAGACGACGCTTCACCAGATTCGAGAACG

ATGTCGCGTGCTGTGTCACGCTGGGCTGGCGTCGCCGTTCATCGACGAGC

GGAGTGCAGACATGTTCGAGATCACGATCTGGGGGCAGTTGTATCTCGAG

GGGAAGGTGAATGCGGGGTTGATTCGACCGCTTCCGAAGCCAAGGCCGCC

AGACAAAGTACGACCCAGATATTGGTCACAGATTGCGTGACTGTTCATAA

ATTATAACATTCACCTATACCAATAGGATAGGAAATGTCTGGAACAAAGT

TCTACTTTGCTACCTTTGAGGTAGAGGGTAATTTCGGTTTTGATTACCCG

GCGGATATGGAATACGATCGAAAGGTGCGTGAATCGTTGACTGATTTTGT

TGAATTGGAGGGTGCTGTTGCCCAAGGAGACTCTGAAGAATGGTACTTTG

GACGGCCTGAGTTCGATGCCAACATGATTTATGGGAAATTTGGAAAAGTG

TATGCAGATGAACCATTAACCTACGATGATGAAATAGGTGACTTCGTGGA

GGGTGATGAACCAAACAAAGAGGCAGACTACTCTCTATTTGCCATTGATC

TAGAAAATAATCTCGTCATTTTCAGCTCAACCTATCGCGTTAGACACCGA

AACTTCACCAAGTATCTCAAAAAAGGATATGACAGCTTTACAGGTGGTGA

TGCCTCAATTCATTTAGAGCTTGTTAGAAATAAAGAGGGTGTTGAGACAG

TAATTCAAAACTATCCTGTTCACAAAATCCATGCAGAATTAGTCCCATCT

AACCCGTCTCCTGATGAAGAATGGGAAGAGTTAGACGAGAGCCTCAGAGA

GATGCTAGCCGATAAACTAGGAATTACTGCTGAACAGTACAATGATGGTG

GATTAGATTTCTCAGAGAGTTTTCTGTCACAAGTGGCCAGTATGTCACAG

TCAAAATATGGTGAATCTTGGGAGATTACCTATAGTGACGATGACGGAGA

ATTCAAGGTCATTTCATCGGATGACGATCCGGCCTCAAAGGTTATTGATG

AAGAGCCGACCACTACTGGCGGTCTGAAAGCGCATATCGAAGGGATACTG

AGTTACGGTCTCACGTTCTTAGACTCAGTACCTCACAAAGCATCCTCGGC

TAGCTGTTTCTGAAGCCTGAGTTCTGTGGCGGAGCGGTTGGACTGGCGGT

TTCGACGAGAGAATTATGGACGGATCGGCGGAGAGCCGTCGCTGTCGGCG

GCGGCTGCCGCCGACGCGACAGCGTCGCCACTCCCACCGTTGGCTAGCCC

GGTCGGGAGTTACCGGTGGAACCGGTCGTCTGGTGGTCGGTTCGCGGGGA

CGGCCCGACGCACCGCGAGGGCCGTCCACGCTGCTCGACGGACCATATTG

ATGAACTCCTTGAACGACCACTCCCAGAGGCGACGCCCCCCACGGCGGGG

CGTCGCCACGTACTCCCAGTGCAAATACCGCCAGACGTTCTGTAACAGCA

GGCTCACCACGACGTACAACAGCCGTACGACCGGATTTTGTGTCGAGGTC

GTCGCGATACTTTGCTCGGAGAGTCGATAGCTTGCCTCGATACCGAAGCG

TTTCGCGTAGTGGTATCGAGCGTCCCGTGGTGAGTCGATGAACGGCGCGT

CAGCGGCGTAGCCGTGACGCGCCACGCCATGTTCGTCGTACCGTCCGTTC

TGGTAGGTACAGTCGATGTAGACGGGAAACTCGACGGTCCAGCTGTGACC

GTCGAGTTTCGCTGTCAGACTGTGCTGAATCACGCGACTCCACCCTTCTG

AGAGTTCTCGCTTGATCGTCTGTCCCCAGCGGACGATCGGCATGACGTAG

GCGTGGTTGTGCGCCTGAAGCAGCGTCAAACACTTGCTGTCGTAGAATTC

GCGGTCAAGATAGACGGCCTTGACACCGAGGTCAAGGCCGTCGAGAATAC

CGAGGAACTCTGCGAGGACACTGCTGGCGGTGTCGCCGTCTTCGAGACGG

CGCACCGCCAGCGTGTAGCGTTTGTTCTTCACGCGTGCGTACAGTGTCGC

GTACGCGTGAAACGCGGTGGTTCCACGCTTCGCTTGTGAGTGATACAGGC

CGTCTGTATCGTCTTCGTCGCCGTAGTAGGGCCGCAGGTGGAGGTCTGCG

CAGACCTCCACCTGCTGGGGAAGGACGTTGAGAATGTCCTTCTGGAGGAG

CGTGTTCCCGATTTGTTCGAGCGTCTCGAGGTCGAACTTGGTGCGGAGAT

GGTAGAGAACCGAGTTTTCGTGAGGTGCATCTTCGCTTCTCTTGCAGAGT

GTAGAGACCGAGGTCCCGTCGGCGCAGGCGCCGACGAGGACCTCGTAGAT

GTCTTCAGCATCGAGTTCAGCGTTTTCAGCGAGTGAGAGAGCAACTTCCT

CGTCAAGAGAGTTGACGAGGAAGTTAAGGAGCTGGTCCTCGTGGATTTCA

TCGTCTGTTTGCTGGTTGTTGGACACATCTTCAGCAAGCAGACGTCTCAA

CTAACCGGCTTTGTGATGTACTGAGACTAATTATGGTCAACCGTGGTACC

GTTCAGCGAATACGTAGTTTTGGTCCTATCCGGATTGTCACTTCATTTGA

GGTTATCGGGGCAGCTCTACTTACAGCGATTGCTCGGTGTTCTTTTTCAG

GTAGTTTTCAACCGGATGGGATGTCTAGTTTTGTAGCTGCAGCAACTGGT

ATATCTTCATCCCTCATTGCAGTTGTCCTCACGGGAATCGCAATTCTAGT

TTCCTTGTCTAGTGAACAGTTCCTATCCTTTCTACGGGAACAAGATATTT

ATGATAGAATTATGTTCGTGTTTGAATATACCGTCGCACTCGCGATTATA

ACGTCATTATTGGGGTCAATACTGCAGACTGTAGAATATTCTGAGTACAT

GTTTTGGATATTTGTATTCTTCTTCATCTATCTACTTCTAAGTACTATGC

AAATCGTATCTACTATAATTGAGTATGGGGACAAGATTGGTGAGTTCAGC

AAGGCTAATAATTTTGAACCAAATGAAGATCTGAAAAAAGATATGCAAGA

TCTGATTCAGGAGCATGGTAATTTCGACGAGGACGATAATCAGACACGGA

ATCGTGGCGAGAACGAAAGTGAGAGTGAGTAAATCACCTCCACTGACTCT

CCAAACTCGTCCGATATCGCATCCCCAACACGGACGTATTGCAACAGTCC

TTCACAGTCTGAGGCGGATACGCATCGCGAACTTCCTCGAGATTTTCGTA

GCTCTCGATCAAGTGCGTGAGCGCCGCGTCGATCACATCACTTCGCGGCG

GATCGTCCGACACATCTCGAGCCACGATCTCGCTTGCTTTGTCGAAGAGC

AGCTGCCGTTCGTCGGTCAACTTCAGGCTGGTTCGTTTGGTCATTTATGA

ATCACCGTATGCACCGGGGGTCGATTTGCGTGCATCTCGAGTGAACGAGC

CTCGGTTCTTCTTATACTGGGGGTGACCCATGACCGCGACCGTGCCGGCC

ACTTGGTCGAATCGGCCTGAATCTATGCACGGTACTCGCCGTCGACGGCG

GTGAAGGTGCATACATCGAGGATGGCGTATACATTCACGCCTGACCCCAT

GGGGGCGAGATTGAGAAGGGGTCTGCACGGGGCTACTCGCCGTCCGAACG

CTTATCCTCTGAGACCGATTCGAATTCAGTATGGCTAGTGCGACCCGTGA

TGATAACGGCGAGGAGGGCGTCGAGTTCATCCACGAGGATGACGGCAGTA

TCACCGCCCGAGACATCGAGACGGGGATCGCATCCTTCGGCGAGACGAAG

TCTGAGGCGCTTCGGATGCTCTCGGAGGCGCTTCTGCTGCACGAAGGGGG

TGGCGAACCCGTCACGGACGAAGATCTCGAGGAGTTGGGACTCGATACTG

ACGAGTTCGACGATCGAGAGCTTCCGGACTTCATGCAGTAACGTGGCGCG

AACGACCTTCTCCGGCCGGGAAGTCGTGAAGGCGTTAACCCGTCATGACT

TCGAACCCGCTGGCCGAACCGGGAGCCACGTTCAGCTGCGGTACGAACAT

CCAGAGACTGACGAAGTCCGAACGGTAACCGTACCGATGAAGTCAGACAT

CCCGACCGGAACGCTTCAGTCCATCGCCAAGCAGTCCGGCGCGAAGGATT

TCCACTCGTGGTGCGAGTGGATCGAACAGACGCTGTAACCGTCATCGGTG

TGGAATCGATTCCGGGAGTTCTGCGATTCCGCCGTCCGTCACATTTCGAA

CCACGTACACGCCTTCGTCGAGACGCTGTACGTGCATCTGTTTCCCCTCC

GGTGCTGTACCGTTCTCGAGAACACCATCCATCTCTAACTCATTTTTATC

CAGCGCGATCAGCGGCGTCCCATCGTGATTTCGAAGTTTTCTGAACATCG

CTACCGCGGTCGGCCACCCGCCTGTACTTAATCCTTCGGACGGGGTGAGA

ATCTCAATAGGACCACGCACCAAATCCTCCGGAATTCCTGAATGTTATTG

CACAACGGGTAGCCGCCGTCTTCGTATCATGTCCGTAATGGAGTTCGACC

CGGAGGACGCCGCGGCGCTCGTCGCGACGTTCATCGGGACGACGGCAATG

GCAGGAATCGCATCGTGGTCGCTGTTCGACGTGACGCTCTCAGACGTGGC

GTTCCAACTCGCGGGCAACGACGTGACGCTGGCGACGCTCCTCACGTTGG

GCGCGCTCGGCGTCACCATCCTCACGAACGACAACGCCGAGTTATCCACC

CTGCACCAGCAGGCGAAGGACCTCGACAACTACTACTACTACAGCATCGT

CGCCTCGGTCGGCCTGTTGGTCGGCTGGGTCTTCTTCGGTGACGTGAGTT

CGTTCGTGCAGTCGCAGGACCTCTGGGGCGTCGGCTACGTCGCTGTGAGT

CTCACCGCGCAGATGGCGATCGGGTGGATGCTATGAACCTCGATCTCGAC

GACCTCGATCTCGAAACGGTTGCAATCATCGGTGCGGGCGTCGGTTCGCT

CGATGCCGGACTGTCGGCCGCGACTGACATCACGCTCCTGTCTGATCTCC

TCGGTGGAAGCCTCGAAACCGGACTTATCGCTGTCGGCGCGGCTGGTGGT

GTCGTGATCGCGGACAAGCTCGGACTCGTTGAGGTGTTCGAGTCGTGAGG

CGCGGCCGCGCAACCCTCGCGCTCGTCGGTCTTTTGGTCGCAGCTGTTGC

CGCAGTCGCCTTCGTCGGTCTCGGTGCGGCGACCGTGACGACCGACTCGG

CCAACTTCACGAGCGTCGGCAACGACTCGCTCGCGAACCAGACCATCGAC

GTGACGAACGACACGCGGAGTCTCTACGTTGAGTTGGACAACGACACCGC

GACTGCCTCCGAACCCGTCGAAGTCGCGGTCTTCGAAGTCGATGCTGACG

GTAACGAGACCGAAGTCGACCGCGTGCAGATCTCTGCAGCGGACGGGACA

ACCGAACTCTACGAGTTCGATGCCCTCGACCCGACGAACGTCTCGACCTA

CCGCGTCGAAGTGCTCGGGACTGCGTCGGCGATTGACTCGTCTGCCCTCG

ACGTGGGGACGGTCGCGGCAGTCTCGTCCGGTGGTGGCTTCCTCGGCGGC

TCGTCCGGTGGTATCGGAATCGGTGCGATTGCAGTCGTCGGAATCGGCGG

CTATCTCGCGCTCAAGGAGGACTGACCCCTGATGCTCCGACTTGCATCGA

CCTACGCCGCCGTTGGTCTCTCGGTGGCCGCCGCCTACCTCGCCGGGGTC

CACGGCGACCACCATGCCGCCGCCATCGTCGCCGCGGTCGCTGTCCTCTT

CGTCGCGCTTCGACTCTCGTTCGGTGATGTGCTTCGGCTCGGCGCGCTCG

ATGCCGACCGGGTCAAGGTCAAGGCTCGACAACTCGGCACGCTCGCCGCC

GTCAGCCTCGTCGTGACAACCGTGGCCACGAGTGGCGTTGTTCCTGGTGC

GTCGCCGGTTGGTGACGCTTCTGCGGAGTGGGTCGACTGCTCGCTGTCCG

ACCCGCTACTCGGCGCGGCGTTCAACACCTTGACCGGCACAGACACCGGC

TGTCGCTGGGAGTCCGGCGAGCAAATCGACTATGAGAACGTCTCGAAGAC

TGACGCGTACGCCTCCGCCCTCGGGATTGCTGACGCCTCCGAATCGTACA

CGACGACGACGAGTAACTTCCTCGAAGACACGCGGTCGGTCGCGTGGTCG

AAGGCGAAAATCACCATCGTTAACGAGCTCAACAACGGCTCGACGGTCAG

TCAGGCGAAAGTCGCGGCCAATCAGACAGTCGCGGAGTACTACACCGGCG

TCCAGCGGAACGTGATTGCTGATTGGAACGCGAAGACGTACACACTCGGG

TACCTCCACACGGAAAGCGGACTTGCCCTCACTCCAGACGGTTTCTCGCA

CATCACAGAGTGGGACAACAAGACGTACACGCTTTCGGATGGGTCGACGC

AGACCCTGCGAGCGATGGACTACTCAACGAATGGAGACTACCACGTGGCC

CCTGAGACGACTATCATGCCACGCTCGACGGCCGACCCCGGTATCACGAC

TATGGGGTCCATCCAAGCCGAAGACCCGTCGGACGGATCTACGACTCACG

TTCTTAGTGCGGTGGCCTACGCCGAGATTCTCGACAACACCTACAATCAG

TCAAATCAGATTCAGGCGAACGTCGACGAGTACGCTGATGATGCGTACGC

GCAGTACCAAGCGGGCGACATCAACACGAGCGACCTGCTCGACCCGACAA

CGATTGCCTCGCAAGCGGCGACTGATTACAACTCGACGGGGTACTACTCG

TTTGCCGCTGTGCAACTCGCGTCGCTCGGTGCGTCGGGCGACCTCAACGC

CTCGCACACTATCGAGACGGGCGACGGTACGACGCTCAACGGGACGCTCT

ACTACACAGGCGACGACGCGCCTGCCGGTGGTTGGGTGACTAATGAGACG

TACACCGTTTCGAACTTTAGCGGGACGTTCTACGTGGCCGCCCAACAGCA

GGACGGAAACGGGAGTATCGTCGACCTCTCGACCTACGAGAATTTCACAA

TCGTCGACGCGGTCAACACCCGGACAGGTGAGTCGCTGAACGCGACGCAA

CCCGAGACGTACATCTACGACTCGACGAACGCAAGCGCCCTCGCGGACGA

AATTGATCGGCTTCGTGATCTTCGCGCAGAGTACGAGCAGGCCGGCGGTT

CCGGTGGCGGCGGTGGAATCGGAATCGGAACTGAGGACCGCCTTATCATC

GCCGGTGCGATCGTTGCGCTCATCCTCGTCGCTACCCGGAACTGACCGTT

TCGACCACTCTACCAACCTTTCACACTCATGCGCAGAACCATCATCACGC

TCGTGATCCTCATCGCAGTCGTATCGCCCGCGCTCGTCGGCACGGTCGCC

GCGCAAGAATCGACGAACTCCACGACGACCACCGCGCCGTCGACCACGCC

CGAAGCAACTCAGACTATCGAACTCTCTCCGACGACGCGAATCAAATCGT

GGTCGTTCTCGAATGGGACGTTCTCGCTCGTCGTCGAGGCTGACATCCCG

ACACGGATCGCCATCACCGACGCTGGTGAACTCTCTCGGATTCTTTCCGA

GGGAGACGGTGCAGCTGCCGGGAAAGCCCGCGTCCGGCGCATGACCTTGA

CCCCCGGAACGACCGTCGTCAAGTTCCGCGCCGAATCGGTCGGTGACGCG

TCGGCAATCACCGTCTCCTCGTCGAACGCAGACGGTATCGTTGCTATCCG

CTCCGACGCGATCAAGACTGGAAACCCGCCCGTCGAGTACGGAACGGTCC

AGACGCTTCTCGCGTCGACGGCGGTCGCCGCGGCCGGTGGGACCTTCCTG

TGGGTTCGCAAGAAGCGCAACGAGAAGCAGCTGGAGGTTGAGCGGGAATG

GTGAACGACTGGGTGAAGCTCGGCGCTGTGGGCGCTGTTTTCGCCTACTT

CGGCGGGTACCAATTCCCCGAGTGGGCACCACTCGCCGCGTCCGGTCTCG

TCATCGCTGGAATCGGTGCGCTGTTCGCGACGGGGAAGATCAACGATCTC

CTCCCCGACCCACCGCGGGTGCGTCTCGTGCAGGTGAACGCGAACAGCGA

CGACCCGCTGGCGTGCTGGTCTCTGTCTCCGGACAAGTTCGCAGAGATGC

AGGTCGAGTGGGGCCCGCTGTATCCACACGAGCAGGCTCACGGCGATGTG

TACGAGTGCTACGCGTATGATCCGTCCTCGAACGTCGCAGTTGGAACGTG

GCGGCGTTCTCTCCCCGGTTCGTCACTCGTGGGGCGACACGATACAGACG

ACGTTCTCGACGTAGTTGGAGAGCTTCGCGGCGACCTCGAACCGGCGGCC

CGGAGAGGACAGGAACTCAGACAGGCGCTTCCGGCGATTGTGAGAAGAGT

GAAGCACGATACGATGGAGGCACAGAACGCCGCGCTCGATCCATCGGCAC

CGATGATCACCGACCAGCCGACGGTCGACGAGATCATCACGTCCGAACTC

CCCGAAGAACTCCGACCCGGCCGGCTGCAGAACGGTGACCTCCGTGATCT

CCTCGAGGCTGCGGGCGACCAGGACGACAATCGGGGCGACTGGGGCGATG

GTCTCGGGATGGTCTTCGACGACGACCTCGCCGATGATGCGCTCGAACCT

GTTCCCACCGACCCGCTCGTTAACGATGGTGGTAACCGATGAGCGGACGC

AAGCAGGGTGACTCGGCTGTTTACGCAGCTGCACAGGGACGCGAGTTCCT

ACGGGGTGCTCTCCGTGACAAGAGCAACGAGTACATTCGTGAGTTCGCCG

GGATGATCGACGACCCCGACGTGCTCGACTTTCTGAATCGGTACTGCTCG

ATCTATGAGGAGCGCGGCAAGAACTTTCTCGAGACCCACGTCGGTCGGCA

CGTCGTCCGGTCGGCCGCAACGTCGATGGCTGACCGCGCGTACCGCGAGG

GGAACGTCTCGCAACTGCAGGGCATGGTCGGGCTCACTAACCAGAAGCGG

GACGGCTCCGAGGCTATCGTCGAGGCGGCGAAACGACTCGCCGACGAAGG

TGCGATCTATCTCGTTCTCGGTCCACCGGGTGCGGGAAAGACTGCATTCG

CGCTCGACGTGGCGCGCGTCTTCGGTTCTCTCACCGGCGGCACCGTCCTC

GCGAACGTCGCGTGGGACGGTGCAGACCGAGTCACGACATCTTCCGGCTC

GATGCTCGACGCGATGGGTTCGACTGATGGACAAGTGCTACAGCTCATCG

ACGAGGCCGGACAGTCGCTCACCTCTCGAGGAGCTGAGGCAGCGATCACC

GACGAGTTCGTGAAATCGCTGAAGTACGTCCGCAAGAAGGAGGACGGCGA

TACCTACGCGAAGCGCGGCTCGGTTCTGCTCATCGGACACACGAGAAAGG

ACACCGCCGCCGAGATTCGCCGACTCGCCTCGGGCGCGTTCGTCAAACCG

ACTCGGAACGACCCCGGCCGCGTTGTCTTCCTCGACTCCGAGGGTGGTGC

AGACTCGTTCGAGGAAGCCGCCGAGTTCACGGGCGTCACGGATACGCGTG

AGAAGTACGATGAGCACGAGGCGTCGCATTTCTCGGTGACGCTCGACGGC

GGTGACGACGACCAGGACGACTCGCCAGACCCGAACCAGATACGATGGGA

GTCCGCCGTGGCGACCGTGATCAAAGCGTGCAAGCCGTGGGATGAAGAAA

ACGGGATGAGCTATCCTGACGCGGCCGAACTCGTCATTTTCGGAGATTCA

TGGGTTGGGAATCGCGTCCGCGAGTGGAAACGTGGCGATCATCGAGATAT

CGTAAGCAGTCCGGAAGGTGATTTCGAGTGGCGGCGGTAACTTCCAGCAG

TCGCCACCACTATCGACCCCCCGGACGGCGTATAATTACTTATCTACTGA

TGCGCCGCTGGCTGCCCCCGGTGGTGGTCCGACCCCGACCGAACCGGGAT

TTTCGACGAAAGTCGCGTTTCGAGCGTCGAATCGAGGCGGTGGGTCCATC

CGTTCTAGTTTAACGAAGAATCCAGTTGCTGTCTGTTGATTTGATATCGT

CGCAACTACCTGATTGAGAAACCCGGTGGGAAGTATGACCTACAGCCCAC

CGGATTCGGTCATAGTTGACCGGATTCAAAGAGCGTTTCCCTCTGATGAG

TTGCGCGAGCGCGCTCGCGCAACGAATCTCGTCCAACGAGAGCGGAAATT

CGACATCGTTGCGCTGTTCTACACACTCTCGTTTGGCTTCGCTGCTGGCT

CAGACCGCTCTCTCCAAGCATTTCTCGAACGCTACGTCGAGATGGCTGAC

TGTGACGAACTCTCCTACGCATCGTTCCACGACTGGTTCGAACCAGGATT

CGTTGCACTCCTTCGAGAGATTCTCGATGACGCAATCGAGAATCTCGATA

CCGGACGAGAAGATTTGAACGGCCGTCTCGAACGCTTTCGAGACGTCCTC

ATTGCTGACGCAACCATCGTTTCGCTGTACCAGGACGCCGCTGATATCTA

CACAGCAACCGGCGACCATCAAGCCGAACTGAAACTTCACCTCACCGAAT

CTCTCTCGACTGGGCTCCCGACACGATTCCGGACAACCGATGGGACGACT

CATGAACGGAGTCAGCTACCCACCGGTGAGTGGGTAGCTGACGCCCTCAT

CTTGCTCGATTTAGGCTTCTACGACTTCTGGTTGTTCGACCGAATCGACC

AGAACGGCGGGTGGTTCGTCTCCCGGGTGAAGGACAACGCGAACTTCGAG

ATCGTCGAAGAACTGCGAACGTGGCGAGGCAACAGCATTCCGCTGGAAGG

AGAGTCGCTGCAGGCCGTCCTTGAGGACCTGCAGCGACAGGAAATCGACG

TACGCATCACGCTTTCATTCGAGCGCAAACGAGGGTCGGGCGCCAGCGCG

ACCCGGACGTTCCGACTGGTCGGCCTGCGCAACGAGGAGACCGACGAGTA

CCATCTGTATCTGACGAATCTGGCGAGAGAGGACTACAGCGCGCCCGATA

TCGCGCAGCTCTATCGGGCGCGCTGGGAGGTCGAACTGCTGTTCAAGGAG

CTGAAGTCGCGGTTCGGCTTGGACGAGATCAAGACGACCGACGGCTACAT

CATCGAGGCGCTGATCATCATGGCCGCAATTTCGTTGATGATGAGTCGTG

TAATCGTGGATGAGTTACGGTCGCTCGAGGCAAGACAGCGAGAGGGCGAA

GCCGCCGCAGACGCCGACTCGTCGGCGTCGCGGCTCCCTCGGCGTCGCTG

TTCGCTCGCCGTGGAACGCCACGGTCATCTGATCCAGTTGTATCTCATGA

TCGAGTTGGGCTACGAACTGCCGGATTTGGACGAGCTGTTGCTGTGGGCG

TCACGAAATCCAAATCCACACAGAGATCGGTTACGTGAGCAGGTTGAACG

AGGTGAGTTCGGCTTTGATCGCTACTAAACTAGAACGCATGGCGGTGGGT

CCATCCGTTCTAGTTTAACGAAGAATCCAGTTGCTGTCTGTTGATTTGAT

ATCGTCGCAACTACCTGATTGAGAAACCCGGTGGGAAGTATGACCTACAG

CCCACCGGATTCGGTCATAGTTGACCGGATTCAAAGAGCGTTTCCCTCTG

ATGAGTTGCGCGAGCGCGCTCGCGCAACGAATCTCGTCCAACGAGAGCGG

AAATTCGACATCGTTGCGCTGTTCTACACACTCTCGTTTGGCTTCGCTGC

TGGCTCAGACCGCTCTCTCCAAGCATTTCTCGAACGCTACGTCGAGATGG

CTGACTGTGACGAACTCTCCTACGCATCGTTCCACGACTGGTTCGAACCA

GGATTCGTTGCACTCCTTCGAGAGATTCTCGATGACGCAATCGAGAATCT

CGATACCGGACGAGAAGATTTGAACGGCCGTCTCGAACGCTTTCGAGACG

TCCTCATTGCTGACGCAACCATCGTTTCGCTGTACCAGGACGCCGCTGAT

ATCTACACAGCAACCGGCGACCATCAAGCCGAACTGAAACTTCACCTCAC

CGAATCTCTCTCGACTGGGCTCCCGACACGATTCCGGACAACCGATGGGA

CGACTCATGAACGGAGTCAGCTACCCACCGGCGAGTGGGTAGCTGACGCC

CTCATTCTCCTTGATTTGGGCTTTTACGACTTCTGGCTGTTCGACCGCAT

CGACAAGAACGACGGTTGGTTCGTCTCTCGTGTCAAAGACGACGCGAACT

TCGAGATTGTCGAAGAACTGCGAACGTGGCGAGGCAACAGCATTCCGCTG

GAAGGAGAGTCGCTGCAGGCCGTCCTCGACGACCTGCAGCGACAGGAGAT

CGACGTCCGCATCACGCTCTCGTTCGAGCGCAAACGAGGGTCGGGCGCCA

GCGCGACCCGGACGTTTCGACTGGTCGGACTACGTAACGAGAAGACCGAC

GAGTATCATCTGTATCTGACGAATCTGGCGAGAGAAAGCTACAGCGCGCC

CGATATCGCGCAGCTCTATCGGGCGCGCTGGGAGGTCGAACTGCTGTTCA

AGGAGTTGAAGTCGCGGTTCGGCTTGGACGAGATCAAGACGACCGACGGC

TACATCATCGAGGCGCTGATCATCATGGCCGCAATTTCGTTGATGATGAG

TCGTGTAATCGTGGATGAGTTGCGGTCACTTGAGGCAAGACAGCGAGAGG

GCGAAGCCGCCGCAGACGCCGACTCGTCGGCGTCGCGGCTCCCTCGGCGT

CGCTGTTCGCTCGCCGTGGAACGCCACGGTCATCTGATCCAACTGTATCT

CATGGTTGAGCTGGGCTACGAACTGCCGGATTTGGACGAGCTGTTGCTGT

GGGCGTCACGAAATCCAAATCCACACAGAGATCGGTTACGTGAGCAGGTT

GAACGAGGTGAGTTCGGCTTTGATCGCTACTAAACTAGAACGCATGGCGG

TGGGTCAAAGAAAATGAATCGCTCGCGGAAAGCAGCACGCTACGGTCGGC

TCGCGGAAGAAGCGGCGCGGCGAATCTATGACCTCGACGCCGACCACGCG

AGTTGGCACGACGCCCACACGTCGGACGGTAGGCCCGTCGAGACGAAAGC

GGCGATGCTGAACCGTGCTGATGGGACAGAGGGCCGATTTCGGATTTTCG

AGGACTACCACGAGAGACTGGTGCGGCACGATGGCCTGTACGTCTTCATA

CCCTACCGCGCCCGTGGACGCGGTATACAGGTCGTGGACGCCCGTTCGGT

GGAAGCATCGAAACTACGGTTCAAATTCTACGGCGCTGGTGGGCACCGAG

AGTCACAGCAAGTGAAAATCCATCCGCGTCGGGTCTTTGGTTAGTCAGGG

TCTGTTGAACTGTTCAAAATATATCTGAAGTTGTGTGTGGTTGGAAATTA

TATTCACAAAACACCTGAAAGGAAGACATGATATGACTAAAATTAAAATC

GAGACACACTTTCAAGGCGAGGCCCATCTTCGATACTAATTACAAATTGA

GTTATGGACGCAGACTTTACTATTATCCCAATCGTTGAAAATCCAGTTGT

TAACAAAGGCGAAACCGTTCGGATACAATTATTCGTCTCTGGCTCTGGTT

CACACTCAAAGCACAAGTTATACATCAATTATTCGTATGAAAATTTACTT

GACACAAGTGAACCGAAAAATGGGAGTATTGGCTTCTTCAAGATGCCAAT

CACTACGGACAGTGGCGGAATTAATATCTCAAGTCAAGAATTGCCTCCTA

ACGCAAGCTATTTTTATGAACCCTCTCGGTTCGGAACCGCAACTGGAAAA

ATGACTGGTGAAACACCTGATGGTCAGGTTCCAGTAAGAGTATCGGAGGC

AACTGCCGACGGTTATCCACCAATGTGGGTTGAGTTAAATATCTCCTCTG

AAGCCACTCCCGGCGACTATGGCATTCCAATAACGTTTACGCACTATGAT

GAATCAACGGTCGCTGTAACCAAAGAAACCCCGACATTCCACGTGAACAC

GTGGGCAGAAAAACACAGAACCAAAATTGAATTGGTTGGTGTCATCGCTG

CCTTAATTGCCTTTTTCGCGTTTCTAATTCAAGCACTAGATGTGATTGGT

TGGATTTAATCTTCCTATCCCACTCATCCCCCGGTTCAGTGAAGCTCTCG

TGAACTATTTCACATACGTCAATCATCTTCTCTCCTGAATGGTTATTGAC

TGGTGCTGACTTGATGGAAATTCTGCTATCCCATCGGAGCGTGAATCAAT

TATGGGACGCAAGAGACCGAGCTGTATTCCGTGCGCTAGAGCACGTGGTG

CTGACTGGTCCGAGTGCGAAAGAAGCCGAGTTATGGGCGTCACAGCCCGG

ATGCCGCTCCTGGCGGCATCCGGGAAAGCCCGCATAGGTGAATTTCTGAT

TCACGCACCGCCGTCCTATCCCGGAGGAACGAACCATGTACCTCGGAATC

GACCTACACAAACGATACGCACAGGTGGCAGTAATGGACCTCGAGGGCGA

GATTGTCGAAGAGGTTCGCGTCAAAAACGCGAACCTCGACGACCTCGCTC

AGCGATACGCTGGCTCTCGTGCAGTAATCGAGGCGACCAGCAATTACTAC

CACGTCTACGATACTCTCGCGGAGTACTTGGACGTAACCGTCGCTCATCC

AGGCAAACTGACGCTCATCGCTCGGTCAGACAAGAAAACCGACCGCGTCG

ACGCGAAAGAACTCGCGCGGCTGCTCCGGCTGAACTCTGTTCCGCAGAGT

TACGTTCCTACCGACGAGATTCGGGAAGCCCGCGCACTCGTGCGCGGGCG

ACAGACCTTGGTCGAAGATCGGACCAAGTTCGCCAACAAAATCCACGGCT

TGCTCGCCGATAACGGCATCACTCAGACGGTAAAACCGCTGAGTGTCGAG

GGACGAGAGTTCCTCCGGGAACTCTCGCTCCCGTCTCCGTGGGACAGTCT

CTTGGACTCGTATCTCGACGTGGTCGAGACGCTAACCGAGCAGATGACTC

AGTTGGAAGCGGCGATTGAGGAGCGCGCTGGGTCTCTGAAGGAGACCCAG

CTGCTGATGACCATCCCCGGTGTCAGTTACTACTCCGCGTTGCTGGTCTA

CGCTGAACTGGGTGAGATCGATCGGTTCGACGGCCACAAAGAGGTCGTGA

GTTACATGGGACTGAACCCGACGATCCGCGAGTCCGGTGACTCGCGGATC

GAGGGTGGTATCTCGAAACGCGGCTCAGGACGAGTTCGCTGGATCTTGGT

TCAGAGTGCGTACTCAGCCGTGTACACGTGTAAAGACGCGTATCTAAGCA

CGTTCTTCCACCGGTTGAATCGTCGAATGAACTCGAAAAAAGCGATCGTC

GCGACGGCACGAAAACTGCTCGTCTCGATGTACTACATGCTCGTCCGAGA

GGAAGTCTACGATCCACCGGGGGTGAGCGCCTGAAGAAGGGAATCTGAGG

AACGCCGGACCGGCGTTCCTCAGTGGCCGGATGAGGCCAGCGTAAGCGAC

AGCTCTAGCAAGACTCGTTCCGACCGCCTACCGGTTGTGGAGGTCTCAAT

CACCACGTTGTCTAGTGGTTTCTCGTCGTTGAGGGACTAGAGAAGCTAGA

TCGTGCGAACAACTTTATCGCCAGCGGTGGTTAGTTGAGCCAGCAGAATT

TCCATAGGTGAATATAGAGGGGGAGTTCAGCAAGACTCAGATATTCATTT

CACGCGACCAGTGACAAAAATACACCCAAACAAGGGCCTTCTGTAATACA

GAACTCAGGATATAGGTACAAAATGAACACTGTTCGAGCACCGAGCGTAC

CGCTCGTCATCCCATGTCGACTGGCAGGTTGTCGTTCCGAAGTGAGTAGT

GAGGCGGGTCGAACGAGGTCGTAATCGCGTGTACGCCCACCCCGATCTCC

TGTACACGGGCGAGTAAGTCGACGAACTCCGGGTCGACAGCTCGGTAGGG

GCGGAAACGTTCGACATCAGGTCGCTGCACGACGAACACGACGTGTGTCT

CGTGGCCGTCTTCAGAGAGCGCTTCGAGGCTCCGGAGGTGGCGCCGACCG

CGCTCGGTTTGGCGGTCGGGAAACATGGCGACGCCGTCCTCGACGTGCGT

GCAAGATTTGATTTCGACGAAGGCGGTCTCGTCGGCCGGCGTCTCGACGA

GAAAATCTGTCCGGCCGTGCTCCGGTAGTACAGGCTCGCGTTTCAGGCAA

CTGTACCCGGCGAACGCGGGGATTGTATCGCGGGCGAGCACGCGCTCGAA

GAGGTCGTTCGCAAACGCCGGGCGGACGCTCACGTACACGCCGTCGACGC

GGACGGTGATGGCGTCGTAGTCCGTCGCACGCTCCGGGTCGTCAACCGGC

GAACAGAGAATCTCGTTTCCCGGCTCGACCGTGCCTTCGAGGGCACCGGG

GTCTCCGAGAAAGACGCGCTCAGGGGCGTCCTCGAACCGAACACGGACGA

CGAACCGATTCGGTCGGTCGACGATGATTCCGGTGAGTAACTCGCCGTCG

ATTGTGAGTAGTGGGTCCATGTGTTGTGGATACGATGCGGCCGAGGGGAT

ACCAATCGTTCGCTTCGAGTCTGTGAAAGCTCTCGGGTCGTCGGGGCGTG

TCTCTCTTCCGGCCCTTATCAGGCTCCGAGATTTGCGATATCGGACCCGA

CCGTCGCCAGCGCGTCGGCAGGTTCGGGTCGCTGTCAGCCAGATGCTCGT

ACCAGTGGTTCGGAACACGAGCCAGCGCCGTGGCGGTGGCCTTGCCGTAC

GTCTCGACGCCGGGCTCTGCCGAGTCGTTACTATCCCAGACATCGCGGAT

GACGGTCATCGACTCGATTCAAACCGCTACGTTCTGTGGCTCGTAGCGAG

CTATAGTTTGAAGAGGCCAAGTTGGAGAGCGACGTACTCCGCGGTGTTGT

TCCCCGCCCGAGATCCGACGGGGCGATCGAGACGAGCCACCAACGTACAG

GACGAACGAGCGGTCGGTCGGCTCCGGGACGGATAGACGGCTGCGATCTG

AGGACAGGAGATCTTCGAGTGCCTGACGGAACTCCGCCGGGGTGGTTGCG

GGGTCGAGGAGGCTGTTGTATCCGGGGACCGCGTCGTCGATGAGTTCGGT

GGCAACTGCCATCTTGTATCCGACGCCTGCGAGCACCTCGTCGACGAGCA

TGGCGAGCGATGAGAGGTGTTCGAAGGGGAGCGGTTCTTCGCTCACGCCA

GCACCACCTCGTTCCGGTCTCCGTTGCCGAATCTCCTATCCTCGCTTGTG

GGTGAGTTCGTCGGTTCGTCGAACATCTGCATACCTCACCTCCGTCGTCG

AACGAGATAATTCCTTCAATGCGCTAGCCGTGGCAGTGAGGTATCGATTG

AAGCCCGCCCACCTGTCCCGACGATGCCCGACGCGAGCACCCCCATAACC

GGAGAGAACGTCAAGTTGGTGGTCCAGCCATCTCTACCTCTCGGCGGTCA

TCCCATTAGTCATGGGCTATCTCGGGTTCAAACGCGCGGACCTGAACTGG

CCGACTACTCCAACCACCCCCGAACAGCACTAACAGCGATGGTCCAGCGT

GCGATACTACCGAGTGCGAGCGAGTCGGAGCGGGAGCCCGATGTCGGACA

AAGATTACGCGAATAGCTCTGTAATTCGATCGCACACCATCGGTTCCGCT

GAGCTGGTGCGTGTTTCTTCATACTGAAGCGCGTTGCTCTCGAACCGCTG

GTTTTCGCCCGGTGGTTCCGAACCGCCGTGTCCGGGTTCGTCGCCGAGAG

CCCCTACGTTGAGGTGTTTCGGCGGCCCCCACGGTCGATTCGGAAATAGT

GAATGCTGTGCCGACACCGAGTACGACCGTGAGAAGACTAAACGCGGCGT

CCGCCCGCCCACAATTCTACAGCAATCCAAACCTATATTAGAATGATAAT

GATAATGGTCTATTATGGCGCAAGTCATTATCGGCGTAGACGCCGGTACG

ACAGTGATTAAATCGGTCGCCTTCTCGCTCAACGGTGAGGAGCTACACAA

GAGCAGTGTCGAAAACGCGGTCGACCGGCCCGAATCCGGCTGGGCAGAAC

AGTCCATGGTGACGACGTGGAAGAAGACCGCACAGACGCTCAGCGAGGTC

GAAGACCAGTTGGACGACGACGACGAGGTCCTCGCGCTCGGCGTCACCGG

GCAGGGCGACGGCTGTTGGCTCGTCGACGAGGACGGTGACCCGGTCCGCC

CCGCGATTCTCTGGTCCGACGGCCGTACGAGTTCCGTCGTGCAGGCGTGG

CAGCAGTCAGGGGTCTCCGAACAGGTCTACGACATCTGCGGCGGCACCCA

GTTCCCGGGGAGCAGCCTCGTCATCCTCCGGTGGCTCAAGGAGAACGAAC

CCGAGCGCTACGAGGAGGCCGACACGGTCTTCTACTGCAAGGACTGGCTG

AAGTACAAGCTCACCGACGAACTCACCACGGACCCGAGCGACGCGTCGCT

CCCATACGTCGAATCGGAGTCCGCCGAGTACTCCGACGAGGTTCTGGACG

TCGTCGGTATGCCGGAGGTCGGCGAGATGCGCCCCGATCTCGTCCCGGGC

ACCGACGTCGTCGGCGGCCTCACCCGGAACGCGGCGGTCGAGACGGGGCT

CCCTGAGGGGACGCCCGTTGTTTCCGGCTTCATCGATATCGCCGCGTCAG

CCTTCGGAAGCGGGGCCGCAAACCCGGGCGACGGCTCTTCTATCGTCGGG

ACGACATCAGTGAATCAGACAGTTCTCGACGAGGCCCCCGACGACGACGA

GCAGACGGGTATCCTGCTGACGCTCGGCGTCGAAGGCGGACTCTGGACAA

AGTTCATGTCCTCGATGACCGGCACGCCGAATCTCGATTGGGCCATCGAG

GAGATTATGGACAAGAGCAGTTTCAACCTCGTCGAAGAGGAGGTCGAGTC

GATTCCCGTCGGCTCCGACGGCCTCATCTACCACCCGTTCCTCAGTTCTG

CAGGCGAGCGTGCCCCCTTCCTCAACCCCAACGCGCGAGCGCAATTCATG

GGGCTGAACCAAGAACACACGCAGGCACACCTCGTCCGCGCGGTCTACGA

GGGCATCTCGCTTGCCATGCGTGACTGTTTCACGCACCTTCCACAGAACG

CCGACGAGGTGTATCTCAGCGGCGGCGGCGCGAACTCGGACTTCTGGTGT

CAGATGTTCGCCGACTGCCTGAACGCGACCATCATCATCCCGGAGGGAAC

CGAGTTCGGCGCGAAGGGAGCCGCGCTCCTCGCCGGCGTCGGCACGGGTG

CCTACGACGACCTCCCCAGCGCCGCGGCCACCACGTCGTCCGTCGCGCAG

TCGTTCGAACCGCGGCCGAAGAAGGTCCAGCAGTACAGCCGCTGGTACGA

CGTGTACACCGATGCCTACGAGGCGACGTTCGCGGTCTGGGACGAGCGCG

TCGAGGCGTTGGAGGACCTCCGCTACATGTCGCAGTCCGCGGGCGCGAGA

CCGAACAAGAAGACCGTCGTCGGCAAGGACACAGTGAAAGGGGGATACGA

TGATTGACCTGTCGTTCCTCTCTTTCGGTATGCTCGTCGCCGCCTTCGCC

GGCGGCGCGTTCGGCGCCGCAATCGGCGGTCAACCCGCCTTCATCTTCAC

CGGCTTCCTCGTCATCGCGGGCGAGGCCGGCAACCTCGCGACCGAGGCCG

TCGCCGCGGCCACGAACGCCGACCCGGCGCAACTGAGCGCGCTCGGCCTC

ACCGGGTCGGTCGCGTTCGGTCCCGTCTTCGGCCCGCACATCAGCTTCGC

CGCGGGGGCCGCCGCCTCCGCCTACGCCGCCAAGCGAGGGTACATGGACG

CGGAGTTCGATTACCACCCCGCGAAGAACATCAACTACGCGTTCGGCACG

AAACCGGACGTGCTCCTCGTCGGCGGGCTGTTCGGTATCGCCGGCTACCT

GATGGCGCAGGTCATGGTTGCGTTCGCCGCGCCGTGGGACCCCGTCGCGA

TGGCCGTCGTCCTCTCGGCGGCGCTCCACCGCGTCGCGTTCGGCTACGAC

CTCGTCGGTCAGTCGGCGGAGGGACGGCTCAACATGCGTCCGTTCGTCGA

AGAACGGTTCCGCGACGCGCCGCAGGCCACCACCGACGGCGGACAACCGC

AGTCCGGGGCGGCGACCCGGTACCTCGTCGAGCCGTGGCTGCCGCAGCAG

TACAAGTGGCTGAACGTCTCCCTGCTCGGACTCGCCGTCGGGGTGCTCGG

GGCGTACCTCGGGTACGCGACGGGCAGCGTCTACCTCGGGTTCGGCATCA

GCGCCGTCTCGCTCATCTTCCTGAGCATCGGCTTGGAGCACTTCCCTGTC

ACCCACCACATCACCCTCCCCGCGAGCACGGCGGTTTTCGCGATGGCCCC

CGAGGGCGCGGCGGTGAGCTCGCTGCCGCTCGCCGAGGTGGTCCTCGTCG

GCGCGGCCATGGGCATCGTCTGCGCGCTGTTCGGCGAACTCTTCCAGCGC

GTCTTCTACGCGCACGGCGACACGCACTTCGACCCGCCGGCCGCGGCCAT

CGTCTTCGGGACGTTCCTCGTCGCGGTCCTCGGAGCGCTCGGCGTGTTCC

CGCACACCTCGTGGGTGCCGCCACTGTGACTGACACTCGCCCGCAGACCA

CGAGACTCGACTGAGACGACGACTCGACCGACGCACCAGACCCGACCGCT

CTCGACCGACGGGCACCGATGGACTACCGTTCCCAGTCCGCGCACCGCGA

ACGGCTCCCCGCATAAACGACCGCTTCGCCGCTAGTGAATCGCTTCGAAC

GCCGCCTGCGCCGTTTCGAGCACCTCCTCGTGGGCCCGACCGTTCGACGC

GACCAGCCCGGGGCGTCCAGGCAACCACTCGTTTCCGTGAATGTCCGTCA

CGACACCGCCAGCCTGCCGAACGTGGTGCACGCCGGCGACCGTGTCCCAC

GCGTTGGGGTGCTCGTCGAGGCCAACGACCGCATCTATCGAACCGTCAGC

GACCCGCGAGAGCGTTAGTTGCGTGGTTCCGATGCGGCGGAGTTCTCCGA

ACCGGCCGAATATCTCGCCGGCAAGCGTCTCGACCGCGGGGCGGTCCTCA

GGTCCGAACCGGAGCGTCGAGGCGACGATGAACGCCTCGATGTCGGGTTC

GTCGTTCACGGTTATCGGCCGTCCGTTGCGCTCGATTTCCCCGCCGGTCG

TGACGTACGTGTCGCCCGTCGCCGGCGCGACGTTGACCGCGGCCAGCGGT

TCCATGTCCTCGACGATGGCGACGCTCGTCACCCACGCGTTCGTCCCGCG

GGTGAAGTTCTGCGTCCCGTCGATGGGGTCGATGACCCACGCGTAGCCCG

ATTCCGGCACGGTCTTTCGCTCGTCTTCCTCCTCGCCGACGACGGCGTCG

TCCGGGAACCGCTCCCGAATCGTCGAGATGACCCGACGCTGGGTCTCGCG

GTCGATTTCGGTCACGAGGTCGGTCTTGTCGCCCTTCTGTTCGATATCGA

AGTCCGTTCTGAAGTGTTCGAAGGCGTACTCCGCCCCGGTCTCTGCGGCC

TCTACCGCGACTGCTTCGCGCGAGTGCTTCTCCATACTCGGCTATCCGTT

CACGGCGAGAAAAAACCTCGGACGGCGGTCGTCCCTTCCCGAATTCGGTT

CGTCCCGTTCCGTCTCGTTCCATCCTGTCTCGTCCCGTCCCGCAACGCCC

CGAGCACCCTCGGCCGCGCCGCTCACTCGACCGGGTCGACCGCGCGGTTC

GCCGTCGGAACCGCCTCGTCACCGCCCGCGGCCGCCCATCTGACCGCGTT

GTCGAGCACCTCTCGCACGACGGGAACCTCGTACACCGGATAGGTCTCGT

GGCCGGGGCGGAAGTAGAATATCCGACCGCTACCGCGCCGGTAGCAACAG

CCGGAGCGGAACACCTCGCCGCCCTCGAACCACGAGGCGAACACTAGACG

GTCGGGCTCGGGGACCGCGAACGGTTCGCCGTACATCTCCGTCTCCGGGA

CGACAAAAGACTCGTCGAGACCGTCGGCGATGGGGTGGCCGGGGTCGACC

ACCCACACTCGCTCTCGCTCGCCGGACTCGCGGTACCGGAGCGAACAGGG

CGTGCCCATCAGTCGCTTGAACACCTTCGAGAAGTGCGCGGAGTGCAACG

GGACGAACCCCATGCCGTCGTAGACGTGTTCGCAGACCCGCTCGACGACC

GCGTCTTCGACCGCCTCGTGGGCCTTGTGACCCCACCAGATCAACGCGTC

GGTGTCGTCGAGCACCGCCTCGGTGAGGCCGTGGTCAGGTTCGTCGAACG

TCGCGACACGCACGTCGTGACCCCGCGATTCGAGGGCCTCGGCGAGCACC

GCGTGGATGCCGTCGGGGTAGACCGCGGCGACTTCCTCGTCCGTTCGCTC

CTGTACGTACTCGTTCCATACGGTCACGGATGCCATACGTCCACAACGCG

CGCACCCGGTCTAAAACTACCGAAATATCGACATGTGGGCGGTGACCGAC

GCCGCGGCGGTTGCGAAAAGCGAGGCGGGACAGACGCCCCGTGGGTGCCT

GCGTGAGCGTTCTCGAACCGCGGTCACGGCTCGGACCGGTCGCGGTCTCG

CCGCCGGCGTCGTCGACCTCCGAGTCCGGTCGCTCCCGCGGTCAGTCGTC

AGTCGCTCGCGTTTCCTCGGAACGGCTCAGTCGTCGCTCGGAACCGCGTC

CGCTTCGACGAGTTTGGTCCAGCGCTCGACACCGGCCTCGGCGACGTCGC

GGTCCTGCGTGACAGCGCCGCCGGAGACGCCGATAGCGCCGACGACTTCG

CCGTCGCGTTCGAGCGGGTAGCCGCCGCCGAAGACGACCATCCGGCCGTC

GTCTCGCGTGTCCAGCCCGTACAGCGAGTTCCCCGGCTCGGACGCCTCGG

CGAGTTCGTGCGTCGGCATGTCGAGAGCGGCGGACGTGTAGGCCTTGTTA

CGTGAGATGTTGACGGAGGCGAGCCACGCGCCGTCCATCCGGTGCTGCGC

GATGAGGTTCCCCTCGCTATTGGCGACCGTGACGACCATGGGGTTCTCGA

TCTCGTCGGCCCGGGTCTCGGCCGCTTCGATCAACTGCGTTGCTACGTCT

AGCGGGATTGCTTCCACCATGCATGCGTAATGCCCGTACGCGGACACATA

AATCCGGTCATTTTTCGTCGGAGTTCTCCCCCGGCCGTCACTCGACGAGC

GCGTCTTCGAGCAGTTCGAGCGGGTGGCGTATCTCGTAGCCCGTGCCGTG

TTCCATCTGCATCGCGCAGGTGGGACACTCGGTGATGCCCGCGTCGCCCT

CGGCGTGGCGCATGTGGTCGAACATCTCCTCGCCGATTTTCATCGACTTG

TCGTACTTCTCTTCCTTCCAGCCGTAGGTCCCCGAGATGCCGGAACAGGA

GTCACCCACGTCCTCTATTTCGACGCCGTCGACGTCGCGGAACAGTTCGA

CGACCTGCCGGTGGAGCCCCTGATTGCGAGCGTGGCACGGTCCGTGGTAG

CCGAACGACTCCCCGTCGAGTTCGGCGTCGGCCAGCGCGCCCCGCAGGTT

CTCGTGGATGCGGAGGTACTCCAGCGCCTCGAACGTGTTGTCCGAGACGT

CCTCGATACCGTCGATGTCGAACAGCTCGGGATACTCCTGCCGGAGCGAC

AGCGAACAGGAGGTACACGAGGCGATGACGTCCGCGCCGTCCGCCAGCGC

GGCCGCGAGGTGTTCGACGTTCGTCTCGGCGTGTCGTCGCGCGTCGTCGA

GCATGCCGTTCGCGAACATCGGCGTCCCCGAGCACCCCTGCGGCGGGACG

AGGACCTCGTAACCGAACTGCTCGAAGACGCGGACCATCGCCTTCGCCAC

CTCCGGCGTGTTGTAGTTGGCGTAACAGCCGTGGAAGTAGGCGACCCGCT

TTTCGGGGTTCTCCACCCGCGCCCCGCCGCGTTCGCGCCACCACTCCCGG

AACGTCTGCGACGCGAACTCGGGGAAGTCGCGTTCGGCGGTGACGCCCAG

CAGTTTCTCCATCGCCCAGCGCGCCGGGCCGAAGTTCATGGCGAAGTTCG

CGAGTCGCGGAACCGCCGACGCGACCGCGGCCGAGGTCCGGTAGTTGGCG

AGGATGCGGTTGCGGACGTACTCCCGCGAGAGCGTGCTCATCTGTTCACT

CACGTACTCGCCGCGGGCCTCGTTGTGCATCTGGCTGAGCGGGACGCTCG

ACGGGCACGCCGAGTCACAGCGCATGCAGTTCGAACACGACATGACGGAG

TCGTCAATGTCGGCGTCATCTTTGCGCTTGAGTCGCCACTGCTCCGGCCC

TTGGAACTTCGGTCCGGGAAACTCCTCGTCCACCTCAGCGACCGGGCAGG

ACGTGTCGCAGGCAGTGCATTTGTAGCAGTCGTCCGCGCCCGCTCGGAGG

TCGAGGTCGCCCTCCTCGAACACGTCGGTCAGTTCCGAGCCGGTATCGTA

CTCGGCACTCTCGGAGGCTGACGCGGCCGCAACCCCGCCGTCAGTGACTC

GTCGTTCCCCGTCGGTTCGTCGCTGTTTTCCCCCGCTGGTCGTCCTGTCT

CTCTCAATCGTCTGGTCGTGGCTGTTCGTCCGATTCGATTCGGTCGTTCG

GTCTCTGTCGTCGCTCGCTGTCATAGTCTCGTGTGTGGGTCGTGTCTGTT

GTCGTTCGGTCGTCCGTGTCGCCGGCCGTCTCGCCGTCAGTCTCCGAGTT

CCTTCGCGGCGTTGCGTCCCGCGACAACACCCGTTGCGAGAGAGACGCCC

GCAGCGGACTTCTCCCGGGCGGCGTCCGCGCCGCCGACCACCGCGCCGGC

GGCGAAGAGGTTCTCGAACTCCGGTGTTCCGTCGGCGGCGAGCGGCCGCA

TCTTAGCGTCCGGTTCGACGCCGAAGCGGGCGTAGGGATGCGCCCCGAAC

GCGTCGTCAACGAACCAGTCGTAGCGGTCCGACGGTTGGGGGACGCGGCA

GTCGAACACGGGCTCTCTGACCGCCGTCCGGTCGGAGTCGAGGCCCTTGC

CGACGAGGCCGCCAGTCGCGAGGACGAACGAGTCGGCCGCGTACGGCACC

TCTCGGCCTTTCCGGTCAACGAGCACCGCATCGACGTGCCCCGACTCGCC

ATCGGTCTCGTAGCCGACGACGGGGTTCCCGGTCTCGTACAGGACGCCCT

CGGCGTCGAGCGCGTCGTAGAGACGGTCCTCCAGCCGGAGGCCGGGGAGG

CTCGGCGGCCCCATCGGAATCTCGAACACGTCGACGCCGAGTCGCTCCGA

GAGCGCCGCCCGAACCGCGTCGGCGCGGTCGTCGCCGAGCACGGCCGGAA

AGCCCACGCGCTCCGCGCGGTCGACGTACGGCTTCACGGCGTCCACGAGC

GCCCGCCTCACCGGAGTCCCGTCGAGGCGCTCGTCGTGGTCCAGCGCCTT

CGCGATTCGCGTCACCGGTGCGTCCGCCCGGAACTCCTCGGCGAACTCGA

TTTCGACGCCGGCGACGGGAAACGGGACGCCCGCGGCCTCAAGCGAGTCC

GCGAGCATGCGGGCGTCGAAGTCCGTGAACGATCGGAAGCCGACGACGAG

CATCGGTCGGTCGTCGCTCGCCAGCCCTTCGGCCGCGGCGCGCGGGTATC

GTGCGGTCGGCTTCACCGCGCCGCCGAACGTCGGCAGGAGCGCGTTACGG

TCCGTGTGGCCGCCGTGATACGCGTCACCAGTGAGGTCGTCAAACAGCGC

GAGGCCGTCCCGCAGGGCGGACTCGCCGACGAGCGAGTAGGGGTGGTCCG

CCGGGAGGTCGTCGAATCCGTCGGTCGGTCTGACGAGCGGTCCCTGGTAC

GCGTCCCGGTTCGACCGCACCGCTCGGAAGTCTGCGCGCCCTTGGGTGAC

GTAATCCGCCCCCTCTGCCGCCGAGTCCCGCGACGGCACGTAGCCGAGGG

CGTCGACCAGCCCCGACGCCTGTCGAAGCGTCGTCTTCTTGTGGGAGACC

AATCTGACCGTCACGCCCTCGCGGGCGGCCGCGACCGCGCTTGCCATCCC

GGCGAGTCCGCCGCCGACGACGAGCACGTCGCTCTCAATCGCCATGGCTG

CCCTCCGGAACTGCGGTCCGTCCCCCGTCGAAGGCGCGGTACTCGATGTT

CTCGTCGCCGGCGACGTGATTCGCGTCGTGATTCATCGTCGTCGCGTGAA

GCATGGCGTTGAGCATAGCCTGCGAGAGCTGTTCGCCCCAGAGGGCGTGA

CGCTGACCCTTCCAGCGCTCCTGATACAGTTCGTCGACGGCGTCGCGGGC

GACCTCGGCTCCGTGGTCGGGATAGAGTTCGGCTCCCAGCCGATGACTAC

AGAAGCCGCCCTGACAGTTCCCCATCGACGCACGGGTTCGGAGGCGGACC

CCGTTGAGGTCCGCGCCGACTTGGTCGATGGCGTCTCTGACCTCGGCGCG

GGTGACTGCCTCGCACTCGCAGAGCGTCGGGTTCGGCTCGTCGATGTCGA

GCACCTCGGGAGCCCTGTCGCCGAGTCGCTGACCGCTTCTGCGGGCGATG

GGCGAGCGCAGGTCGAACTCGTCCATGTACTCGTCGAGCGCCGAGGGGTC

GGCGCTCCCGGGGAGCGGAACCTCGTCCGTCCGGCAGGGTTCCTCGACGC

CCAGTACCTCGCAGACGTGGTCGCTGACGGACTCCGCCATCTCTCGGTAG

GTGGTGAGCTTGCCGCCGACGACGGAGGCGAACCCCGCGACTCCGTCGCG

CTCGGCGTGGTCGAGCACGAAGTAGTTCCGCGTCACGTCGCCGGGGTCGG

TCGTCGACTTCGGGTTCGGGTCGTACAGCGGTCTGACGCCCCAGTAGGCG

CGTATCATGCGGGCGTCGGCGACCACCGGAACCATCTCGGAGGCGATGTC

AATCATCATGTCCACTTCCCACTGCTCTTCGGGGTAGTCGTCTGGGTCGT

CAACCGGGTCGTCGTTTGCGCCGAGCAGGACCGTCGTCTCGTGGGGGATG

ATGGTATCGCCCTCGCCCTTCGGGAGACAGCGGTTGATGACCGTGTCCAA

CTGCCGGACGTTGGTGACGACCATCGCGCCCTTCGAAATCGCCATTTCGA

GGTCGACGCCGGCCATCGCCGCCAACTGTCCCGCCCACGCGCCGGTCGCA

CTGACAACGTAGTCCGCCTCGAACGTCTCGGTGTCGCCCGCCGCGCCCTC

GCTGTGGTGGTTCGGCCCCTGTCGCTTGACTTCGACGCCGGCCACCCGTC

CGCCCTCGACGACAAGGTCGACGACTTCCGCGTGCGTCTCGATGCGCGCG

CCGTGTTCGACGGCGCTCGCGGCGTTCGCCACGCAGAGGCGGAACGGGTC

GACCGCGCCGTCGGGAACCCATATGGCACGCTCGACCGCGTCGGTGAGGT

AGGGTTCGCGCCGTCTCGCCTCCTCGCCCGAGATGACCTCGGTCGGGATG

TCGCACTCCGCACACCCGGCCAACTTCCGTTCGAAGTAGTCGTCGGAGTC

GCCCTCCAACTGGACGAACAGCCCGCCGGTGTCCTCGATACAGTGGCCCG

CGATGCGGTGGAGCACCCGGTTTTCCCGCATGCAGTCGACGGCGCTCTCT

TTGTCCGACACCGCGTAGCGGGCCCCGCTGTGGAGGTGGCCGTGGGTGCG

CCCGGTCGTCCCCTCTGTGAGATTCCCCCGCTCCACGAGCGTCACGTCGA

AGCCGCGCATCGCGAGGTCGCGGGCGGTCCCCGTACCGGTGGCTCCACCG

CCGATAACGACGACTGAGTAGCTCATATAATTATGAAGAACCATGGGTAG

CGACGTAATAATTGTTGTGCTCACCGAATTTTCCGCGGTCGCGGGTCGGA

CGCGGCGGCGGCACCGCTCACGCGCCTTCCGTGACACGGGTCTCGATTCG

GCGCTTCTCCGAGCCGTGACCGGCGATTCGCCGGTGTGCCGACTCGACGG

TCGCTCCGAGGACGGCCCGTTTAGTGGTCCTGTCCGTAGTCGGCGAAGCG

GTCGAGGAGGGTATCGATTTCCTCGTCGGGGAGTAGCGAGGGCTCGCCGA

TGTTGAGCGCCTGGTAGTGGATGCGGGCGCAGTACTCGACCATGAGCGCC

ACCTCGTACGCCTCCGCGACGGAGTCGCCGACGGCGACGACGCCGTGGTT

TTCGAGGAGACAGGCGTTGTAGTCGTCGCCCAGTGCCTCCAGAGCGAGGT

CGGCCAGTCCCTGCGTCCCGTAAGTCTCGTACCCCGCGACGGGAATCTCG

TCGCCGGCAAAGGCGATGAGGTAGTGCGAGGCCGGAATCGGCTCGCCGAG

GCTCGCGAAGGTGCTGGCGTACGGGGAGTGATTGTGGACTACCGCGCCCG

CGTCCTCGCGCTCGCGGAGGATGCCGGTATGCATACGGACCTCGCTCGAC

GGCTTGCGGTCGCCGGCGACCAACTCGCCGTCCAGCGTGACGACGGGCAC

GTCCTCGGATTCGATCTCGTCGTAGGGCATCCCCGAGGGACTGATGGCGA

CGAGGTCGTCGGCCTGCGCGCTGATGTTTCCGCCGGTCCCCTCGGTCAGT

CCCTGATGGAGCATCTCACGGCCGAACTCGCTGACCGCCGAGCGGTGCGA

CGCGAGGTCGGATAGTCGGTCGGCGGGGACGCGATCCCCGGTCGGGTCCG

GCCCGTCCTCGTCGGCCGACTGCGTCTGCCCGTAGTCGGCGAAGCGGTCG

AGGAGAGTGTCGATTTCCTCGTCGGGGAGCAGCGAGGGCTCGCCGACGCT

GCGCGCCTGGTAGTGGATGCGGGCACAGTACTCGACCATGAGCGCCACCT

CGTACGCCTCCTCGGCGGTCGCACCGGTAGCGAGGACGCCGTGTTTTTCG

AGGAGACAGGCGTTGTAGTCGTCGCCTAACGTCTCCACCGCCACGTCGGC

GAGTTCAGCGGTCCCGTAGGTTTCGTAACTCGCCACGGGAATCTCGTCGC

CGATGAACGCGATGAGGTAGTGCGAGGGGCCGACTGGTTCGTCGATACTG

GCGAAAGTGCTGGCGTACGGGGAGTGGTTGTGGACCACCGCGCCCACGTC

CTCGCGCTGGCGGAGCACGTCCGTGTGCATCCGGAACTCGCTCGATGGCT

TGCGGTCGCCGGCGACTCGTTCTCCGTTGATGTCGAGTATCGGTACGTCC

TCGGGTTCGATTTCTTCGTAGGGCATCCCCGAGGGGCTGATGGCGACAGT

TCCGTCGTCCGCCCTGACGCTGATGTTTCCGCCGGTTCCTTTCGTGAGGC

CGTCTGCCAGCATGCGCTTGCCGAGCGTACTGACTAGACGCTGAGGCTCG

TTCACCGTCATGATTGTTCTATCCAGAGGGGGGGTGATAATTCTAGGGGT

TGCAACAGCGACGGTCCGACGCGACCCGTGTTTTTCTCGGCGTTCGTCGC

GGGCGGGCCGCCGCAGTCGGACAAACTACTTTACGACCGCGGATCGTCGG

TTCGCCCATGGAAATAACCGGGTACGAACTGTTCGAAGTCCCGCCCCGGT

GGGTCTTCCTCAAGCTCGAAACCGACGCGGGCGTCTGCGGCTGGGGGGAG

CCAATCGTGGAGGGCTACGCGAAGACGACGAAGGCCGCCGTCGAGGAGAT

GGTCGACAACTACCTCCTCGGGACCGACCCGCTCGAAATCGAGCGCCACT

GGCAGGCGATGTATCGTGGGCGGCACTTCCGGGGCGGCCCGGTTCTCATG

AGCGCTATCGGCGGTATCGACCAAGCGCTGTGGGACATCAAGGGGAAACA

CTACGGCGCACCCGTCTACGACCTCCTCGGCGGAAAGGCCCGCGACCGGA

TTCGGGTCTACCAGTGGGTCGGCGGCGAGACGCCCGAGGCCATCGCGCGC

GCCGCGGCCGAGGAGGTCGACCGCGGCTACGACTGCCTGAAGCTCTCCGC

CGTCTCGCAGTTAGACCGCATCGACTCCCCGGCCGCGGTGGCCCGGGTCC

GCGAGCGCCTCGAAGCCGTCCGATCCGAGGTCGGGGACGACGTGGACATC

GTCGTCGACTTCCGTGGGCGCATCACGACCGGGATGGCGAAATGGGTCGC

CGACGAACTCGACCCGCTCGATCCGATGTTCTACGAGGAGCCTGTCCTCC

CCGAACACGCGCGCTCCCTGCCGCGCATCGAGAACCGAACCAAGGTCCCT

CTCGCGACCGGCGAACGCCTGTACTCCCGCTGGGACTTCGAGCGGACGCT

GGAACTCGACGCCATCGACGTGCTTCAACCCTCCCCGTCCCACGCCGGGG

GTATCTCGGAGGTCCGCAAGATAGCCACGGCGGCCGAGGCGAAAGATATG

CTCGTCTCGCTTCACTGCCCGCTGGGGCCGATTTCGTTCGCCTCCTGTCT

CCACCTCGATATGGTGCTCCCGAACGCCATCGCACAGGCGCAGAACCTCG

AAATCCACCGCGCCGACGGGAACGACCTCCTCGGGTACCTCGACGACCCC

GCCGTCTTCGGGTTCGACGACGGCTTCGTCTCCGCTCCCGACGCCCCCGG

CCTCGGCGTGAAGATAGATGAGGAGGCCGTCCGCGAGCACGCGCAGTCCC

GCGTCGACTGGCAGAGCCCGATATGGTACCATGAGGACGGGAGCGTCGCC

GAGTGGTGAACCACCGACGCGGCCGATTCGGCGATAGTGAATACTTTTAT

GGTAAACTCCGTTCTTCGTTTGGCCATGAACGAGCGCGGAGACCCACCGG

TGAAGGCGACGACGACGAGCGGGCGCGTGCTGGACGCGCTCTCGAAACTC

GGCGACGCCAACCTGACCGAGGTCGAGACGGAGGTCGGCCTCTCGAAGAG

TTCGGTGCACAACCATCTCGAAACGCTCCGACAACTGGGGTTCGTCGTCA

AGGAGAACCAACGGTATCGACGCAGTCTTCGGTTTTTCGAACTCGGCTGT

TCGGTCCGGTCCGAAGCCCCCTTCTACGGGGTCGGGAAGTCGGAGGTCGA

CCGACTCGCGCGGATTTCGGGACTCGCCGCGGGCCTCACCGTATTCGAGC

GCGGACGGGGCGTCTGCATCTACGAGCGGACCGGCCAGAACGTTGAGGCC

CCGCCCGTGACCGAAGGACAGACGGTCCCACTCTACTGCACGGCCCCGGG

GAAGGCGATGCTCGCACAACTCCCGGCGGACGACCGCCGCGACGCCGTCG

ATGCGACGTCACTTGACCGCCACACGGACCAGACGATTACGAGCGCGGCC

GCACTCCGTAAGGAACTCGACACCGCGGAGACTCGCGGCCTCTTGTCGGA

CCGCGAGGAGTGGCAGACCGACCTCCGCGGTCTCGCCGCTGGCGTCGTCG

ACCCCGAGGACGGTCGGGTCGGGTCGATATTCGTGTTGAGTTCCTCGGAG

AGCATGTCCGGAAAGCGTTTCCAACAGGACGTGCCCGGACTCATCATCAG

TTCCGCCAACCAGATTCGGAAGGCGCTCCGCGAGTCGGTCTGAGTCGCGG

CGCTCCGTGCCTCGCGGCCGCTCAACGCCTACTTCTGTTCTCTCTTCGTC

CGATAGCAGCTTCGACCACCGAGCGAGTGCGCACCCTACACGGAGCATTA

GTGGACGACTCGTCCCCTTCGAATTGTGTTCACTATATCCGAATACTGCG

TTGGGACACCCATTACTGTCGTGCCTGTGTCATTACGTGTCTTCACCGTC

GGTCAAGACATCCGTCTCATGCCGCACAGTAGCGATACGATAAGGATTGG

TGACTGTATCATTTGTTCATACCTCGATCGGAGGTGGTAAGCGCACGGTC

TCAGCCCCGGCACCAGACAGTCGCCCCGACGACTGGGTGGGAGGACCCTC

AACTGGATGTTCATGTCAAAAATAGAATATACAATCACGAGGGCGGTGTC

GCTTTGAGTCGGTGTGCGACTCGTAGATCGTCCATACATGACAGACGCAC

CGCTGTAACGTTATAAATTACAGATGTACATGTGTAATTGAGGACCACCT

CATCCACTGTTACGAGGCGTGGAGCGAACGGTCATTAGGTCGCCAGTCAA

GAAGGGGGTCTGCCCGTGTGGGCCACAACTCTACTTACCACCAAGAGAGT

CATAAGAGTGTTCAGTTTTGTGAGCCACTAATTTTAGAGAGATTGAATGA

AACTACTGTATATCGTAGAATGAATGGAGATGTGTGAATTATATGACTTA

TTCAGATACTCAATCAACAGGAAGTCTAGTTAAGCCAGTTTGAGGCACGA

GCAGGTCGTTGAGTATGTTTGGGATGAAGCCCGCAGACCTGCTCAGTGAG

TACTTTGCGACAGATTTAAAAGAAACGTGGGAGCGTGAGCGGACGGCGAT

ACTCGTCAGGTCGTTCGCCGTCCAGCTCCACACGACCGGTTGTTCACTCA

GAGAGATAAAACAGATTCTTCGATATCTCGGCATTGAACGCTCTCACCAA

GCTGTTTAGCAGTGAATATATCGTATTTCTGATAGCTGTCACAACCCGCC

TGAGGCGAAACCGAAGCGGGTTGCGGTCGACGAGACTGCTGTCACAATCA

ACGGTGAGTGTTCTTGGTTGTACGCTGCAATATACTTCGACATAAAAGTG

ATTCTTGACGTTGCGCTGTTCAAGCGTCATGGAACCGATCCGGCGGTTGC

GTTTCTCCACGGAGTCTGTAAGAAACACGACGGTTCAGAGACGGTGTTTC

TGGCTGATGTCTTCGGATATCAGACTGCCTTCTCTCGATTAGGGTTGAAC

AGTCGGGTTGATTACACAGACCGAAACCTGATTGAAAAATGGTTTCACAC

ATTCAAAATGAGAGTTGACCGTTTCCACAATTCATGGGTGGAAGTCGGCT

GAGCGTCCGCCAATGGCTTGCAGTGTTCATCCATTACTGTAATTTTCAGC

GACCGCATCAGTCGCTCGACGGAAGAACGCCAGCTCAGGAGGTTAACTAG

ACAGTGCCCTGGCTCAGATTGAGTCCACAACCTAAGTTAGGAACAACAAA

CCACCGGCCTCACGGTGAGTCAGACTCCTGTTTCGACCGACACCTAGCTC

GTCGAACGACGAATGTACCAACCAACATAGCGATTACTGTAAGTACAACG

CCGAATCCCGGTGAGTCACTAGCCGTCGTGGCTGGCTCGTCAGTTGTGTC

TATCGTGTCCGTTATGTCCGATTCGGTTGTCGCGGCTGACTCGTCAGTTG

TGATTGTCGTGTCGGTCGTGTTTGCAACTGGAGTCGAGCCGATTACGAAC

GTCGACAGTCCTGGCGATGTGGCTTCCAGCGTATACCGCGCATCAGTCTC

CTCAACGATAGTGGTTGGTAGTCGATTCCACTGAGTCGTCTCGGCGCGGT

AAAGCGCGACGGAGTCGGAATTGAGGCCCGAGTCGGCGAACTGTGACTTG

CGGAGACTAACGGTGAATATCACGTCTTCGATATCCTCGTCGGCAATCGT

ATGGTCGACGGTAATCGTCCCCATCGACTGCGCTTCCGTCCCAGAGGTAA

ACGACGGCTCGGTTTCGCCACTTCCGGCGTCACTGGTCGTGATGTCGAGT

TCGAAATCCGAATCGTCGTCGATATTCATCTGCAGGCCGGTGAGTGACAG

ATTGCGTTCGGTATCGTCAGAACTGGTGTCGCTGAGTAGGATCTCGACTG

GTTCGTCAGTCGAGGGGTCCGAGACGTCGACGCGCCACGTACCCTCTCCC

TTATCTCCGGCGGTTACACGAACTTCGGGATCATTACTGGACGACGAAGA

TGACCCCAAATCATGGGGCGTTGGTTCGTCAACGCCGATGATCGTTACCG

TAATCGTCCCACGTGCAGTATCACCGTCGACATCCGAAACAGTGTAGGTA

AAGTTGTCTGTCGTATTTTCCCCATCCTCAAGGGACTCGAACTGCCCGTT

CGGGTCGTACGTCCAACTTCCGTTCCCATAGACGGTGAGGTTCGCCCCCG

AGCCGAGGGTGAGGACTCTACCGCCGCTGAACATCGTGCCGTTGACCGCC

GTCAGTGAGTCGCCAGCGCTGGTAGCGGTCCCTAGTTGTATGAGGTTGAC

GCTCTCGCCGTCGGCGATGGAAAGCACGGTGTCCTCGCGAGTTGTTTGTG

AGCTGTTGTCGGGTGCGACCCGGGGTTCGGCAGCATCGATCACGGTAACG

GTGATTGTCTCGGTGGTGTTGATGCCGAACTCATCAGCCGCCTGCACGTC

GACCACATACTCGTTGTCCCCGTCGGCATCGGTCGCGTTCTCGAAGTCCG

GTGCGCTGTCGAACGAGAGGGTGCCGGTGGTTGCGTTGGTGCTGAATGCT

GATCGATCCACCCCACCGACAAGCGAGTAGGTGATGTTGGTATCGTTGCC

GTTTCCGTCGGTAGCGTTCACGTCGGCTACAATGACGGTGTTTTCCGCGA

CGGCCCAGTTCTCACTAGTGGTGAACGTCGGCCCTGTGTTGTCGACGGTG

AACGACTCGCCGCCCGTATAGCTCCCGTCGGTACTCCCGGCTGCTCCGGA

TTCGACCAGCGCGTTACCGTCGGCGTCGGTAATACTGTCGTCGTCGACGA

GGTCGACTCCGAGGTCGCCCGTGCCCGAGACGGTGTCGACGGTGACGATG

TACCGGCTGTCAGCACCGGTAACCGAGATGTCGTCGTCTCCATTGGCTGT

TGTCGCAGTACCAGTCGTTGCAAGCGTGAAATCGTCAGCGTCCACGCCGG

TGACCGGCTCCGAGAATGTCACGGTGAGGTTCACGCGTTCGGCGGTGGTT

GCCTGAACGTCTGGGTCATTCCGGGTAATAGACTCGACGACTGGCTTTGC

AATACCCTGCGCTTGAAGCTGTTCCGTCCGGTCGATTCCCCGCAGTATCG

GATAACCGTCCATCGCCGCGTCCGTATCCTCGCGTTCGACCATTCCCCAC

GTGTTGGTGAAGTCGAGACCGGTCATGTTCGTCTCCGCACTCGCCCCCTG

CATCTCGGGAGCGGGAGCGGTGTCGGCCGTCGCTCCGAAGCCGCTGACGT

CGGTGAAGGTTATCGGCTCCCACCCATCTCCCGTACTCACACTGTCCTCG

TTCGTCGTTCCTACGTCCCAGTAAGCCTGCTCCACGGTAGTCGGTTCAAC

AGCGGAGCCACCGACGAGGCCACCAATCCCCTGCGTCTCGTCGTCGATAC

TGAGAGACCCAGTGGCGTACGAACGCTGAACCGTTCCTCCAGGGTCCGCT

TGGAACGACCCGAGTACTCCACCGGCGTACCAGCTCGCAGTAACCGTGCC

AGTAGCGTAGGAATCCGCTATCGTTCCCGTGGAGACGCCGACCAGTCCGC

CAGCAGACGTGCTGCCTCCCCCTCCGTCGACGGCTCTTGCAGCGAAGGAG

CGATTGACCGTCCCCTGGTTGAAACCGACCAGTCCGCCAAACCAATCGCT

TCCACGAGTCGGTTCTCCAATCGCGTAGGAGCGACGAACCGTCCCATCGT

TACGGCCGACTAGGCCGCCAACTCGATAAGTCCCGGTAACCTCACCAGTG

ACGTGAGAGTTCTCGACGGTCCCATCGTTATACCCGGCGAGCTGTCCGGT

TCTTCTCGCGCCGGTGACGGATCCGCCCTCGAGACCGATGTTGGTGATCG

TTCCTCCCGACGCAACCTCACTAAACAGGCCAACGGAGTATGTCCCTCCT

CGGTTGATAGTCAGGCCAGAAATCGTGTGGTCGTCGCCATCGAAGGTGCC

CGTAAACTCGTTGTTTTTGTCCCCGATCGGGTCAAACCCATTGTTGCTGT

CCCACGTCGATGTCTCGCTCGCGTTGATATCTCGGGTTAGAACGTAGTTA

TTCCCCAGTCCCTTGTCATCGATACACTGGAGTTTGTCCAGCGTATCGAC

CTCGTAGTACGTTTGCCCCGAGATAGTCTGATTGGTCCATGAAACGGCGC

TACACTGCGATGTGTCCGGCATATCGGCGGCTGCCACCCCCGAAAAGGCG

GTGACACCGACACCCAACGAAACGACGAGGAGTGTTATTACCACGAGTAG

CCACCGACTGACCCTCGCACCCATCAGACACTTACTCCGTCCAACCTTCG

GTTGCGTCGATCGCGGTCCACGGTCGCCCGTAATGCGGTTTTATTCATAT

ATTCAGTACGAATTCAACCGCAAGTATAGTCATTGGCTGAGACGGTGTTT

CACGGCATGAGACATCAGTCATTCAGACAACAAGAACCGCAACCACAGCT

GTGTGACGGTGACGCCGACACTCCGACTGCGTGGCGTGATATATTGGAGG

AGACAAAATACGTATCATCAGTACTTCACAAAGCGTCCTCGGCTAGCTGT

TTCTGAAGCCTGAGTTCTGTGGCGGAGCGGTTGGACTGGCGGTTTCGACG

AGAGAATCATGGACGGATCGGCGGAGAGCCGTCGCTGTCGGCGGCGGCTG

CCGCCGACGCGACAGCGTCGCCACTCCCACCGTTGGCTAGCCCGGTCGGG

AGTTACCGGTGGAACCGGTCGTCCGGTGGCCGGTTCGCGGGGACGGCCCG

ACGCGTCGCGAGGGCCGTCCACGCTGCCCGTCGGATCATGTTGATGAACT

CCTTGAATGGCCACTGCCAGAGGCGACGCCCGCCTCGGCGGGGCGTCGCC

ACGTACTCCCAGTGCAGATACCGCCACACGTTCTGTAACAGCAAGCTCAC

CACGACGTACAGCAGCCGTACAACCGGATTTTGTGTCGAGGTCGTCGCAA

TACTTTGCTCGGAGAGTCGGTAGCTCGCCTCGATACCGAAGCGTTTCGCG

TAGTGGTATCGAGCGTCTCGTGGTGAGTTGATGAACGGCGCGTCAGCGGC

GTAGCCGTGACGCGCCACCCCATGTTCGTCGTACCGTCCGTTCTGGTAGG

TACAGTCGATGTAGACGGGGAACTCGACGGTCCAGCTGTGACCGTCGAGT

CTCGCCGTCAGACTGTGCTGAATCACGCGACTCCACCCTTCTGAGAGTTC

TCGCTTGATCGTCCGTCCCCAGCGGACGATCGGCATGACGTAGGCGTGGT

TGTGCGCCTGAAGCAGCGTCAAACACTTGCTGTCGTAGAATTCGCGGTCA

AGATAGACGGCCTTGACACCGAGGTCAAGGCCGTCGAGAATACCGAGGAA

CTCTGCGAGGACACTGCTGGCGGTGTCGCCGTCTTCGAGACGGCGCACCG

CCAGCGTGTAGCGTTTGTTCTTCACGCGTGCGTACAGCGTCGCGTAGGCG

TGGAACGCGGTGGTTCCACGCTTCGCTTGTGAGTGATACAGGCCGTCCGT

GTCGTCTTCGTCACCATAGTAGGGCCGCAGGTGGAGGTCTGCGCAGACCT

CCACCTGCTGGGGAAGGACGTCGAGAACGTCTTTCTGGAGGAGCATGTTG

CCAACCTGTTCGAGCGTTTCTAGATCGAATTTAGTGCGGAGATGGTAGAG

AACGGAGTTTTCGTGGGGTGCATCTTCGCTTCTCTCACAGAGCGTTGAGA

CTGAGGTCCCGTCGGCGCAGGCGCCGACGAGGACCTCGTAGATGTCTTCA

GCATCGAGTTCAGCGTTTTCAGCGAGTGAGAGAGCAACTTCCTCGTCAAG

AGAGTTGACGAGGAAGTTAAGGAGCTGGTCCTCGTGGATTTCATCGTCTG

TTTGCTGGTTGTTGGACACATCTTCAGCAAGCAGACCTTCTAACTAAACG

GCTTTGTGAAGTACTGATCATTCATAGCCTCATGATAGTCGTATGAATCG

CTACAGTCGTGATCCCACCCGACTGTTGAACCACCAGCCACTATTGGCTG

TGGCCCGTGCTCGGTCGGTCGACCGGTCGACATTAGAGACTGAACTTGAC

GTGTCAAGAGCGACCGTCTACCGCCAGACCTCCGCGCTGGTCGACGAGGG

ACTGCTCAAACGGACCAACCGAGGGTACCGGACGACTGGCGCGGGAACGG

CTGTTCTCGACGCTGCCGAACGTTTCGAGCGCTCGCTGGCGGCGGCAGAC

CGACTGAAGCCGCTGCTCGAACACCTCTCGCCGTCCGTCCTCACCGAAAA

CCTCCATCTTTTCGCCGACGCGACGGTGATGGCAGTCGACTCTGAGTCAC

CCTATGCCATTGAGCAACGCCTCGAATCCGTCATCAGCGATGTCGACGAG

CGAATCTACGGGGCTGCAACGAGTTTCGGCTCACCGGTGACGCTCGCTCG

GACCGTTGACTGCGTTGAGGGAGGCGTTGACTTCGAGTGGGCTCTCCCGC

AGGCGGTCCTAGAACGCCTCGAATGCCAACACGGCGAGCTGCATGAAACA

GTACGAGCCCACGACAACGCTTCCGTCTACGTCACCGAGGACGTGGTTGA

CCTCTCGTTGTACGACGACACGCTCGTGCTCACAGGGTTCGACACCGACC

GGGGGACGCTGGCGGCCGTCGCCATGACCGACAGCTCTGCAGCCGTCGAC

TGGGCTTGCGACGTCTTCGAAACCCACCGTGAGCGCGGCGAGCGACTAAG

CTAAGTGGGCCACGGCGGCGCCACCACCGAAACCCACGGTGAATAACCTT

CTCGCTCCGTATTGGTATAATTCTGAATGTGTGATTTTCATTGCTCAATA

GGGCACTGTCTAGTTAGTGACCTCCTCAATCGGTGTTCCCATCGAGCGCT

TGATGCGGTCTCTGAAAATTGTAGTATTGCACAAATTGTATGGAAGATTT

AGTTAATCCTACGGATTAGCGTGGAATATCCAGCCTGCCATCGACGGTGG

ACTGCGTAGGAAGTGTCGGATTCACTCCCGCCGTAAACGGCGGGATGCTC

TCCTCGAAGAAAGATAGCCTATTGGACATTCCATAGTGCCGTGTCGAACG

CAGTCAGATTGCGGATGTGACGGAACGGTCCGGACTGATCGAACGCCTTC

TGGGTGTCTCCTCGATTTCACTAGCACAGCGCGGTAACAATCTCATACAC

TCTGCTGTCTCGATACTGCCGACATAGCAGTCTTCAATCTCGGTAAGCAA

CCCGTTAGCGTCTGATCTTGTCAGTTCGGGAGAGGTCACGGAAAGCCAGC

CGATTTACAGTCTGGACGAGGGGTGTGTCACCATCGAAACCATCTGCAGG

GGAAGAACAGTTTCCACTCTCAGTCACTTCATTCACTGCCGCTAGAGAGC

GTGACCTCTTCCTAGAAGTTGCAAAGACGATGGAGCGGGCATTCGACAAG

GCTCAAGTGGAGGCGTAAGTATGCCACGAGGAATGGTTGAGCGGTTCGAG

AATCCATCAGACGAAGGGGACGATGAGAACACTAAGGCCGAAGAGCATCC

GTTCTAGTTTAACGAAGAATCCAGTTGCTGTCTGTTGATTTGATATCGTC

GCAACTACCTGATTGAGAAACCCGGTGGGAAGTATGACCTACAGCCCACC

GGATTCGGTCATAGTTGACCGGATTCAAAGAGCGTTTCCCTCTGATGAGT

TGCGCGAGCGCGCTCGCGCAACGAATCTCGTCCAACGAGAGCGGAAATTC

GACATCGTTGCGCTGTTCTACACACTCTCGTTTGGCTTCGCTGCTGGCTC

AGACCGCTCTCTCCAAGCATTTCTCGAACGCTACGTCGAGATGGCTGACT

GTGACGAACTCTCCTACGCATCGTTCCACGACTGGTTCGAACCAGGATTC

GTTGCACTCCTTCGAGAGATTCTCGATGACGCAATCGAGAATCTCGATAC

CGGACGAGAAGATTTGAACGGCCGTCTCGAACGCTTTCGAGACGTCCTCA

TTGCTGACGCAACCATCGTTTCGCTGTACCAGGACGCCGCTGATATCTAC

ACAGCAACCGGCGACCATCAAGCCGAACTGAAACTTCACCTCACCGAATC

TCTCTCGACTGGGCTCCCGACACGATTCCGGACAACCGATGGGACGACTC

ATGAACGGAGTCAGCTACCCACCGGCGAGTGGGTAGCTGACGCCCTCATT

CTCCTTGATTTGGGCTTTTACGACTTCTGGCTGTTCGACCGCATCGACAA

GAACGACGGTTGGTTCGTCTCTCGTGTCAAAGACGACGCGAACTTCGAGA

TTGTCGAAGAACTGCGAACGTGGCGAGGCAACAGCATTCCGCTGGAAGGA

GAGTCGCTGCAGGCCGTCCTCGACGACCTGCAGCGACAGGAGATCGACGT

CCGCATCACGCTCTCGTTCGAGCGCAAACGAGGGTCGGGCGCCAGCGCGA

CCCGGACGTTTCGACTGGTCGGACTACGTAACGAGAAGACCGACGAGTAT

CATCTGTATCTGACGAATCTGGCGAGAGAAAGCTACAGCGCGCCCGATAT

CGCGCAGCTCTATCGGGCGCGCTGGGAGGTCGAACTGCTGTTCAAGGAGT

TGAAGTCGCGGTTCGGCTTGGACGAGATCAAGACGACCGACGGCTACATC

ATCGAGGCGCTGATCATCATGGCCGCAATTTCGTTGATGATGAGTCGTGT

AATCGTGGATGAGTTGCGGTCACTTGAGGCAAGACAGCGAGAGGGCGAAG

CCGCCGCAGACGCCGACTCGTCGGCGTCGCGGCTCCCTCGGCGTCGCTGT

TCGCTCGCCGTGGAACGCCACGGTCATCTGATCCAACTGTATCTCATGGT

TGAGCTGGGCTACGAACTGCCGGATTTGGACGAGCTGTTGCTGTGGGCGT

CACGAAATCCAAATCCACACAGAGATCGGTTACGTGAGCAGGTTGAACGA

GGTGAGTTCGGCTTTGATCGCTACTAAACTAGAACGCATGGGCCGAAGAG

GAAACTGCAGCGACGTCGTCAAAAACAGAAGCAGAAACTCGAGGGTCAGG

GAAGGCCACGGGTAACCAGTCGAACCAGCAACAGGGCTCGACCGCGGGTA

CCACCGAATCGTCCGCAAGTGAAGTGAGAGAGACAGACGACGAGCCGTAG

AATATCCGTGAGGACTGAGACTCAGCCACGATATACGTAGAACCGGAGCA

GAGCGAGGATATCGAGATTACATTTACTCGCCTGAAAAAGCAGCTGAAAC

GCGAGGGTGTCACGCTGGAGAAGAACAAACACTTCTATCGTGGGATCTTC

GAGGTGGCGTTTGGCGAACACCAAGAGGAAACGAAAGAGAAGATTCGGGA

CTCGCAAAGCAAGACGCCAATCAGTGAATCACTGATTCACGGAGTGTCGA

ATCTATATCAGGTCACAACTTCACTTTCGACTCGATAACGGTGGCCAATC

GACATTTTGTGATTGATTCAGACGTCGCTCTTATGTAGAGCTGAGGAACG

ACCGTGTAGTTACGGTGAGCGAGCCGAAATCAGGAGGTTATGTCTCAGAC

TCAGCCAGCGTTCGGTGGAATGCTTCGCCAGCTCGTTGATCTTGGATTGT

TCGAGTTGGACACCGAGCCGCTCGATGCCCTCATCGAGCGGCGACTCAAG

CAGTTCTGGGAGTATACCACGTGTCCTCGCTGCGGCCACCCCAGCATTCA

CACGTGGGACTCGTCCGACCGCGTTATCTGCCGAGATTGCAATTTTAAAC

CGGTCTACACCTATGGCACGCCCTTCCACGAGAAGCACCTCACCACCGGA

GAGGTGCTTCTCGCGTACCTCCTCTATGCAGACACGTTGCTCAGTATCTC

ACAAATCGCGGTTGTGCTCGACAGGGCGTACAAGACCGTCTATTACGCCA

TTCGAGAGGTGGAAGCCGCGGTCACGCGCGGCTTCCCCCTCGTCTGGGAG

CAATTCCAACACTCCATCTCGGGCCCAACACAAATCGACGAATCAAGCAC

GGTCTGTTCAGGCTACAAAGGCCAAGACCCGCCGCGGACCAGTCGGTACC

GCGGCGGGTCGTCCCGATCGGGGCGCTCACGATGGAAGGGCCGTCACGGA

GACCAGATAACGCTCGTCGCGGCGTGCCGCGACGTGCTTCGGGTGATTCG

CGGTCACCTTGGAATCAGCTACCAGGGAGACCTCGAACCGGTACTGCAAG

AGGCTGAAGACCTCTCCCAGCGGCTGGGAGAGGTCTGGACTGATGGACTC

CAAGCGTATCGAGAGATGGAGTACGATCACCGAACCGTTATCCACGACGA

ACGGTACGTTTCGGCCGACGGCGTCCACATCAACCAGGTCGAGTGCCTCT

TCTCACTAGTCAAACCGTGGCTGCGGAAGTTTCGCGGCCTGTCCAAGCAG

GGCTTGGAACAGGCCGCTCACACCTTCGGTATCGTTCGCTCATTGAACTT

GGTCGGCGCATCCCTCGAAATCGTCGTCGATTGCCTTGCTACGGGGTCAC

TCCACAGTTCTACATAAGAGCGAGGAATCGAGAGTTTAACTCGAATTTCT

GAACCGGAAGGAGAAAATTGTGGGAACACACACCCCTCGTCAAGAGTGAA

AACGATTGAAAGGGTGAGGGGTGTTATCCACAAGAAACAGAGGTCAAAGA

GACAGAACACGGACGAACCCACGGAGAGAGTCGCTAAGACAGAGATTGAC

GGCGAAATAAGAGGAAAGTAGATGAGTAGTTCTTCACGAGATCCAGACGA

ATACTAATTCGTCTTTTTTTGTTGTGGAGAATGTAGCTAGTGGGCTATTC

GAACCAATAAAATATAAATCTTCTAGACATTACTAAGAATTCTGATAAAT

GTGCGTTCTGGTTGATTCCCCGGAAGCTCGATGCGTGTGTTTCCGTCTCT

CTTCCTGTGATTCGTGTCGTTTGCACGCGATACAGTCGTGCAATTCTCAT

CTTGCTACCCGGATCTTCTCGTGCTGTTTCACTCTGGACGAGGGGTGGGT

GGGTGTCTTTACGTCGTGACTTTTATGTGATTATTCTCTCGATACCCTCA

AGATTGGTGCAGAATACAGGTCAACAGCTGTGTTTTGTATCGATGGCTAC

GACTGAAAACAGGAAGCATCTCCTCGAACTTCTGTTCAACCGAATAACGA

ACCTCGCGACTGTTTTCACTCTGGACGAGGGGTGTATGCCGTGCGTCAGG

CGTCTTTGTGTCTGCTCAATGTTTGACATCTTTGCTCTGATTGAGTTATA

CTCTTGATGGGGGGTGTGCATTTTCGAGCCCAGTGGAAGATTAGTGGTAA

TTCACTCGGGACGAGGGGTGGTCAAATATTGGTTTCTGTCCGCTATACAG

TCTTGGTGAGGGGTGTTTCATCGTCCAAATCACTTGCACGGGAAGGACGC

CTTTCACTCTGGATTACCTCGTCTCTTTTACCGACTGACATTGGACGCTC

TAGGCAGTAGGTCCTAGTTATGGGCTAATTTACACTCTTGTCGAGGTGGG

AGAATAATTAAGTAGCCAGGTTGCGCGGTATCTGGTATCGATGGCTGATG

ACCCGTCGCAACTCTCTGACTTCAACGACCGGTACGAAGACCCGGCTGAG

GATCCGCTGTTCAATATTGATGGAGACGACCCTGACCGGGTCGACATTTT

TACTCGCAAGGAACTCCTGAAGGTTGGTCACGTCCCGGAAAGTGCCCGAA

TCGTCGGCCGTGACGAAGAAATCAAAGACATCGCTGCTGAACTCAGGCCG

ATCGTTCAAAATGACCCGCCTAACAACGTCATGATCTACGGCAAGACAGG

GACGGGCAAGTCGCTGGTTGCTCGCCATGTCACAGAACGTGCCCGTCGAG

CCGCAGAGTCAAATGGGGTTTCGGTCGGGACTGTCTACGTCGACTGTGCA

CAGCACAATACACAGACCCGTGTTGCCCGAACAGTGACACGGGAGCTCAA

CAAGACCGAAAAAACTGACTTCGACGTCCCACGAGCAGGGATTGGCAGTG

GGGAATATTACGACTACCTCTGGGAGATTCTGAATCTCGCCTACGATGCT

GTTGTCATCATTCTCGATGAGGTTGACCGCCTGAACGACGATGATATTCT

GATGCAGCTCTCACGAGCCCGTGAATCTGGGAAAGCCGATTGTCACCTGG

GCGTTATCGCGGTGAGTAACAAAATCGAGTATCGGGACCAGTTGAACGAG

CGCGTCAAGTCAAGCCTTCGTGAAGAAGAGTTCGTCTTCCAGCCCTACGA

CGCGAATCAACTCCGTGGGATAATGAAGCACCGCCGTGACGCGTTCCACG

ACGACGTTCTCTCTGACGACGTCATCCCGCTAACTGCGGCACTCGCCGCA

CAGGAACATGGCGACGCCAGGAAGGCGATCGAGATTCTCCGACACGCGGG

CGAACTCGCTGAGCGAGAAAACGCTGACCAGGTCGTCGCAGAGCACGTTC

GCGATGCTCAAGAATGGGCAGAGGTTGACCGATTCGAGGAGTTACTTCGT

GGATCGACGACTCAGGTCAAGTTCATTCTCTACTCACTTGCATTGTTGAC

CGAGGAGAGCTCGAACGAAGACGAATTCTCGACAAGCCGTATTTACAAGC

GCTACCAGAACACTGCTGAGACGGTCGATGCGAAGGTTCTGAGCGAACAC

CGCGTCTATGAACTGCTGAAGGAGCAGGCGTTCCTCGGCGTGGTGGAATC

GACCCGAACTGGCGGTGGACGGGGAGAAGGGAGCTACCTTGAGCATCGAC

TGGTTCAGGATACTGGTATTGTGCTGAAATCTGTGCTCCGTGATAGCCGA

CTGGAAGATCTCGCGTAATCTCCGTTTTCCCCGACTACACACCCCTGGTC

ACGAATGTAAGTTCTGACTTGTTCAGTCTGGTAACCCACACCCCGTGTGC

AAAGTGTAAATATGCGCTATTCATGCGCGAGAGCGCTTAAGGAACAGTTG

AGTCTTTGATTATTCATCTTGTACACGGGGTGTGTCACCTCTCAACTTGT

CATTCGATTCATCTCTCACACGGGGTCCACGGGTAATCAGAAAATTCACT

TCGCACACGGGGTGTGGTGGCCCTCGATCTCATGGAAGTTGCCCTTCCTA

TTCACCGGAATTGACTGGTCCTTTATACTTAGACTTGATTTTGTCACCCT

CTCGCCACTGCCAGTAGTAGTAGCGTTTGTCGTTGATCTCTTTGATCGTA

ATCGTCGCCTTCGTGGGGACATCATCTGGAAGGTCATCCGACCGTTCTTC

GAGTTCCCCTTCCGGTGGCTCTTCCTCGCGACGAACCTCACGCTCCTTGT

GTTCGGCCAACTCTTCGGCATAGCGGGCAACGTCTCGGAGACTGTTCGGC

GATAAGTTATTCAGGGCGTCGACGACTTCTGTGGAGAGGCTCGTCGGCGG

CGTCGGTGGTTCGTCGGACATCGGCTGCTGCCTCGTGTTAACCAACACAA

GGGCTAATCAAAAATTTTGTTGGTTAATCTGCTGATACCAACTTCCGGTT

TTCCTCTGGTTGTAACGCTACTCGAAACTGGTATATCAACAAGTTTCGCG

CTACGTTACAACGCGCTTCGATGAGTCAACTAACATTCGGACGCAAGCCT

AGACTTCCTCGTGGACAAGTACCACCAGTTCGTCACTCTGGACGAACAAC

TTCGTCGCAGGAATAGCACTCAGCGAACACGCCAGTTGACCCATCGCCAT

CTTCGAACTCAATGAGTGACTGGTATGCTTCAATCACTTCGCTGCAATCA

GGACATCGACCAAGCGCCGTCGAATCACTCGTCATGGGGGATTCTGGAGA

CGCATGTGACACAGTCTGTCTCCAGTAGTGTCCACACGATGCCACAGCAA

AGTTGTTTGGTCGACTCAAGTGCCGCCGAGAATAATTGGTGAACCATCGG

GATGTTTGCACCTCAGACTTCCAAGAGCTGTGAGTCACACGTTGTTGGTT

ATATCATTTAACCCAAACAACTGCAAGTTATCCCGCTTTGCAACTGCTTC

TCGGACAGTTTGGGTCGCCCCGCTCCGAGTAAACAGTGCATACTCTTTGT

CGATATCTCCGCCACTGTCAGGGGTCCATCTGATCTCTTCTGTGTGTTCT

TCGAGTGACGCAAGCGCACTATAGTCGAGCGGGGCGTTCGTGAATTTACA

CTCGCCGACGACCATCGTTCCCTCCGTAGTGAAACCGACGACGTCGACTT

CGTGTTCTTTGTACCACCACCGGCCGATATCTAAGAACGTTTTATCCGGG

TACAATCCAGGAAGCGCATCCTGGCAGAGGACTTCAAACTGTGAACTCAC

GAAGTCAGGGAGCTCAGGCTCGATGACTGCCTCATACGCTTCGTCGCCCA

ATCGCTCGTATCGATCTTCGTTCCCGTAGACAAATCGGAACCAAAACCGA

AATAGCGGGTCGAGAATACGGTACCGGCCACGGCGAGAGCGAGCTTTCTC

TTCTGTGAGTGGGACTTCACGTTCGATGAGCCGAAGGCGCTCTAGCTTCT

GGGTGTAGGTCGAAATCTGTTTTCCGTCGATCCCAACCATCTGTGCAATC

TCGTTTGACGTGGTATTCCCTGCAGCGATGGCCTTGAGAATCGCAAAGTA

TCGATTCGGCTCAGTTAACTCAGTTCGGAGGACGTACTCCGGTTCGTTAT

GGAGATATCCCCGCTGTGAGAGGAGGGAGTCAGAAATCACGGTTCCAAGG

TCGTGCTCAAGGTCAATGCCATCGAGATAGTATGGAACACCACCGAAGAT

GCCCCATGCAAAAATTCGGTCTTCAGGCTTGTAGCTGTCCGGGAAGAACT

CCTGGGCAGCAGCAAATCCGAGTTGTCGGAGATCGATTTTCTCGGTGAAT

CGCCCGTAAAGTGGGCTGTTTCCTAACAGGGTTGCTTCCTCCATCATGCT

AATCGATGATCCAACGAGGATGAGTGTTCCACTCGTGTTCTGGAGGCGTT

GGTCCCACAATCGCTGAATCACCGAAGGGATGCTCTTGTCGGCGTCGATG

AGATATGGAAATTCGTCGAGTACGACAATTCCGCCTTGTTCGACAAGATA

TCCGAGTAGTGACTCCCAATTCTGTTTGATGTGTTCGATTCCAGGGAAGG

AACCTGAAGCGACGTCAACGAATTCGTCGAGTTGCACCTGTGGTGTCGTC

TGCGTCGCTTGGTATACGACGGCATCATCTCGCTGCGTGAGTGAGTGTTG

AACGAGTTGTGTCTTTCCGAGGCGACGCCGTCCAAAGATAACAATCATCT

CTGGGCTGTCGGACGCGTAGGACTCTCGCAGCCGAGATAATTCGATTTCC

CGATCCACAAAGCGTTCCATGAACCACATATATTCTCGCAAGAAGTTAAT

CATGCCGAATAACCAAATACAGAATAGGCTATTCTGAAATAGGCTATCTT

CAATTACCCTATCAGACACACCACTCTTGCCAGTAAAACCAGCACCCATC

AACAGCCGCTATGAAGACGTGATTTGGAACCGATAGTGACCCGGCACTCA

CACAGTAGTAATTCGGTCGAGAATGGCGATTCCGTTCCTCGTCACGGGAG

TTCTCCGGACACATCAACTAAGTCACCCATTCAGATTCATTTTGGTTTAT

CCCAGAGTGATTTACCCAAGGGTAAACTACTTTGCGTCAGAGTGCGAACT

GTGATGTATGAGTACCGATCTGGGAACCGCTGGTGGAGAGAGGTCCCGTG

AACTCATCCATTTCGTCACCCAGCAGACGCGGTTCGCGCTCGTCAATAAC

ATCCTCCAGCACCCCAAGCAGCTGCCTTCAATGTACGAACTTGAGGAGTT

CAACCCCAGCGTCAGCGATGCCACCGTCTACAAGCACATCCAGAAGTTGA

TCGACGCCGGCATCGTCAAAGAAGTCGCACTTGACGACGACCAGCGCCGA

CAAGGCTATCCTTGGAAGTTCTACGGCCTGACTGAGGACGGTCGAACATT

CCTTGAGGAACACAATCTGCTCGCCGCTGAGGAGACGCTCCAGCAAATCT

ACGAGACTATCTCCGACAAGCCCGAGAAGATGGTCAAGTACGAGAACGCA

CCCCGTCCAGAACAGCAGTAGTCAAGCTAATTCTGTGCAGCGACCTCGGT

ATCATCCGCCTCAACGCCAAGGTCGTACTCAACGTGGATGTCGCTGTACC

AGACGACCGGCCGCTTTGATCGGCCATCCTGTTCAAATTCGACAAGTCCG

AGTTCTGCGAGGTCGTTGACCGCGGTCGAGACGTTCTTGATGTCACGATT

AACGAGTCGTGCGAGTTCGCGGAGACTCTTCGGTTCTTCGCGGGCAGTAG

TCCGGATCAGTTCGAGATCTTTTCGCTCAGAACACGTGCAAGTGCAGCCT

CGTCTGGAAGTGAGACGCCAGTGTGGTCATCAGTCAGTTCACCAGCGTCG

GCTTTCGCTGCGGCTTCAAGTGCACGGTCGAAGAACTCGTCGTTCGATTC

GACACGGACGGTGAGTGTGTTTCTAGTCATGGTTTGTGGTGGAGATGATT

CCTATTGGTATTCCAGTGCATTTCTACAGGCAGAGCAGCCCAACCGTTCT

GTGAAATTGAGTTCCGCTGCATCTTGAATCTCCCCGAGAAACTCGCAGAG

AACTTTAGGCCAAGAGTAGGCGACTCCCCCCTGACAACGCCCATTGATGC

GGATACTGTGCCGTCTCTGGCTTTCAACAGTCCCTCGCTAGATAACTCGC

CACTCTGCGGATACTCTGTCAGACACACCACTTTTGCCAGTAAAAACTAG

TATCCCTCAACGACCTCAGATTCATCGCACTTCTATTGGGAGAACGCGTC

GATCGAGACGTTCCAGCGTCCAAAGAGTACTTTGGAACAACTACTCCGGC

AAGTCTATTACTTTCCGTTACATACTGTTACATATGCCCGTTGATTTCGA

AAACTACCAGCCATCGGATCTTCCAGGACCGAATACGAATGGACGGACCA

TTCTTCGCTTCCTCGCAGCGACTCCAGAAACAGGCTATCGAGCGGGTGAG

ATTGTTGAGGCGCTCGACATCCCACGAGGAAGTGTTGGAACGACGCTCAG

CAGACTCCATAACCAGGGGTTTGTGCGTCACAAGGGCGAGTATTGGGCGA

TCAATCCCGACGCCTACGATGCACATACTGCGAGCTTGATTGGCTTGGCT

GCCGTGAGCGAGCAGTTTGAGGGCGACTACTACGACGAGAATCCGGACTG

GGATGCAAATCTCCCTGATCTCGATGAGTCTGAAGATGCAGACTCTGGTA

CAGAGTGAACCACCTCGGGGTCAAGCTCCGAGGCACTCGGGTTGATACCT

GAACTGCGATTGCGCTGCCGCCCTTCGTTACGACGGCTCGAGTCTACTGA

CAAGCCCCACGGGTCCAGTTGGGATCACTAACAACGAACCGGTGTGTACA

ACACGAGAGCTACTGTCTGCTTGGTCCAGTTTCAGTACCTCACAAAGCCG

TTTAGTTAGAAGGTCTGCTTGCTGAAGATGTGTCCAACAACCAGCAAACA

GACGATGAAATCCACGAGGACCAGCTCCTTAACTTCCTCGTCAACTCTCT

TGACGAGGAAGTTGCTCTCTCACTCGCTGAAAACGCTGAACTCGATGCTG

AAGACATCTACGAGGTCCTCGTCGGCGCCTGCGCCGACGGGACCTCGGTC

TCTACACTCTGCAAGAGAAGCGAAGATGCACCTCACGAAAACTCGGTTCT

CTACCATCTCCGCACCAAGTTCGACCTCGAGACGCTCGAACAAATCGGGA

ACACGCTCCTCCAGAAAGACGTTCTCGACGTCCTTCCCCAGCAGGTGGAG

GTCTGCGCAGACCTCCACCTGCGGCCCTACTACGGCGACGAAGACGATAC

AGACGGCCTGTATCACTCACAAGCGAAGCGTGGAACCACCGCGTTTCACG

CGTACGCGACACTGTACGCACGCGTGAAGAACAAACGCTACACGCTGGCG

GTGCGCCGTCTCGAAGACGGCGACACCGCCAGCAGTGTCCTCGCAGAGTT

CCTCGGTATTCTCGACGGCCTTGACCTCGGTGTCAAGGCCGTCTATCTTG

ACCGCGAATTCTACGACAGCAAGTGTTTGACGCTGCTTCAGGCGCACAAC

CACGCCTACGTCATGCCGATCGTCCGCTGGGGACGGACGATCAAGCGAGA

ACTCTCAGAAGGGTGGAGTCGCGTGATTCAGCACAGTCTGACAGCGAAAC

TCGACGGTCACAGCTGGACCGTCGAGTTTCCCGTCTACATCGACTGTACC

TACCAGAACGGACGGTACGACGAACATGGCGTGGCGCGTCACGGCTACGC

CGCTGACGCGCCGTTCATCGACTCACCACGGGACGCTCGATACCACTACG

CGAAACGCTTCGGTATCGAGGCAAGCTATCGACTCTCCGAGCAAAGTATC

GCGACGACCTCGACACAAAATCCGGTCGTACGGCTGTTGTACGTCGTGGT

GAGCCTGCTGTTACAGAACGTCTGGCGGTATTTGCACTGGGAGTACGTGG

CGACGCCCCGCCGTGGGGGGCGTCGCCTCTGGGAGTGGTCGTTCAAGGAG

TTCATCAATATGGTCCGTCGAGCAGCGTGGACGGCCCTCGCGGTGCGTCG

GGCCGTCCCCGCGAACCGACCACCAGACGACCGGTTCCACCGGTAACTCC

CGACCGGGCTAGCCAACAGTGGGAGTGGCGACGCTGTCGCGTCGGCGGCA

GCCGCCGCCGACAGCGACGGCTCTCCGCCGATCCGTCCATGATTCTGTCG

TCGAGACCGCCAATGCAACCGCTCGGCCACAGAATTCAGGCTTCAGAGAC

AGCTAGGCGAGGATGCTTTGTGAGGTACTGAGTTTGAAAAGCGAGCAGAT

TGTGCGTGGTTTGGGTTGTGATCTTCCCACCGAGAGGGCTGACGTTTTAG

CCAGGCAAGTATTTATACGACAGAAGAATCAAGATTAGATTGACCAATGA

GTCTTGATGAAGCGGTTGACGAAGCACTCGCGACGTACAACATGTCGCGC

AGCGAAGAGGCCGAGGAAACAGGCCGAGCAGTCGCCACGCTCCAAGACTA

ACAGAACCGTTTAGTTGAGGTTCCGAATCTTTTCAGCGAACGCTGTTTTC

AATCGACCTGGCGTTTCCTTCAGCTCCGGATAGTCTGCGCGTTCGACAGC

CTCCTCAACGAACTCTGTCCAGAGTTCTTCGTGTTGCTCCGCGTGAACTG

CTTCGGGGTCAAGCCCCGAGGCACTCGGCCTTCTCCGCCTGTAGAACCGC

AGCGAGCGACTGCCTCTCGGCGAGTGGCAAATCGATTGTGAGTTTGGTCC

TTCCCCGAATGTCTTATCACGAATTATGGATAATTCGTAAGTAAGATGGC

GCAGTCACCTCCACGGTCAGGCCGCCCACCGATTCAGCAGCTTCAGACGG

TGGCCAACCTCCTCGACACACCCACACTTGCTCGGCTGTATGCCCATACC

TTACAACACGGACCGGTCACCGTGTCCGAGTTGGTCGACGAACTCGATAT

CCCACAGGGAACTGCTTACGACTATATGCAGAACCTCGAAACAGCTGGGT

TAGTAGAGAAAGTCCGTGAGCAGCGCCCGTACGAATACGATGCCGAGTCC

ATCGCACTTACGCTCTCGACAGACGGTGAAACCCAAACAATCACACCAGC

ACTTATCGCCGCTGTTGCCCGCCGCGACCAGGACGAAGATATCGACATCT

ACATCGAGCGACATGGTCTCGACGGGCTTGCTGTCGCCCTCGAGTACGCT

TCCGAGTACGTCGACGGTACGGTCAATCATCGGATTGCGTCCCGGGAACT

TGACCTTTCACCGCTTGAAGCCGAGATCATTCTCCAGGCGCTGGAACCAG

TTGCCACTGAGTACGCCGATTCCGGTGCATGACGACGGTGTATATCGCCG

ATACCGGTGTCTTCGTTCGGTGTGGTGGGCCCGACAAAGACAAATTTCAA

CGGCTCCGTCGAGCACTCTAACAGGCTGGTGTCTCATTGCGTGTCCCCCA

ACGCGTCTACGAAGAACTCGGTGGCGATCCGGCAGCGGACGCGTATCCCT

CGGGAAATATTCCCTATCCCGATGGATTCGAGGAGGGCTGGATCGTGGTT

GCCGACGAACTTGACTACACCAACCCGCTTGTCTCGACGGTGATGGACGA

AGCCCGCCGGTTTATCGCTAACGAGACTGGCCGTGACGAAGATATCACCG

AGAAAGCGGACACGGCACTCGTGGAGCTTGCTGCTCAGGTACTCGATACA

GAGCAGGCGGATCACGTCGTTCTCTTGACGACCGACAAACCTAACGACGA

ATGAGTCAGCCATCACAGAGCGTATCGACCGCTTCATCGAGACGGGACAC

CACGAACGGATCTGGCGAGATTGGAACCCCGGTGAACGGACCATCCGTGT

ACTCCGGGATGCGATTCGAGACGCTCCAGACGAGGTCGTCTCACTGGGGG

AGTTCCACTCGGCGAAGGAGCTGTTTGAAGCAGTGGAGTCCTACGACCCG

GAAGCAGGCTGGAAGCGAGACGTATGTAATCGCATCTCGAGTCCTCGAAG

TCTCGGGAACCTTCTCGCATCCCAGCGCGACCACCGGAGTCTGACCATTC

GAGAGCACGGAAATACGAACCACTATCGGATTCAGGAGTCTTCTCGTGGC

GTCCAGTCCATCGACGTCGAGGCAATCGAAGACCTGTTCGAGCTTCCCTG

TATGGCGAATATGGCCGAGCGCCTCCACGAGAAGAAGCCTGTCCGAAAGG

ACCTGTACAACTTCGCCCGGATGGTGATGTGGCTTCCCCAGTACCAGGAT

AGTGACCTCGAGACGATTGTCGCAGACCTCAAGGACGTCTTCTCGCGGTG

GCCCTGGTACGACGAACAGGTCACCGATTACCAGATTCGCTACGAGTTCT

CGAACACCATTGGAGGCGACACCCCGCTTCCGATGAACTGTGACAACGAC

GATATGCAGCGGTACTGCATCGGCCAGGAGCAGTGCCCCTACTCGATCTG

GGGAAGTCTCCCATTCCCAGACGAGATGTATGACCAACTAAGTGGAGCGG

AGGGTAACGGAAACGAGTTCTGACGCAGATAGCCAACCATCAGTTAGATT

ACTATTCTCAGCGGACAGCGATGTGACGGAGATTGAAAACCCGTCATGCA

CTTTTCAGGCTTTCAGTCGACCAATTCGGGCAAGAATGGGTATCGTGCTG

GAAGGATAGTCTCGCAACTCATCAGCTGGGGTCGGTCAGTTCTGCTAGGG

CTTGATTCTAATTTGACTCCAACCCTCCCGAATACGGGGCTTTCAACACC

TCGTCAAGCACAATCTCGCTGAATCACCATCTTTCGGAACCCGTCGAGGA

CATTAGTAACGCCTGAAAACTGCCCGACGGACCTCTTGCTAAATCTAAGG

GCGTTTCAACTCTCTCTGCACCTCCAAAATCATCCAAAGGAAGGCTAGGG

TACACGGGTGCAAACGCCGCTGGCTCTCTCCGGCCGAAGGGCCATCAAAC

GACGAGCGTCTCTCGTAAACATATCAGAATCCACTCATTAGATGAGGGTG

CGATCCGCGGTGGAGTTTGTCACCCCCGAGAGGGGTGAGGGGGCTGTCGT

AGGCACCTACTCAACAACCATGTCTTCCGACTACGAGCAACAGCGATTAC

GACTTCATAGCGAGCATACTGAACAACCGAGCGACGAACCGTGGACAGAT

CTCGAACTGACGCTCCCGCACGGCAGAAGTCCGACTGGCATCGTCTCAGT

TGTCGAGCATGTACTCGTCGAACTCACTCACGGAGATGTCGGAGCCGAGT

TCGTCTCAACGAGTGTTGCGGGCGTAAAGCGGACACAGTTCATTGCTGTC

GACGACGTCGGGCAACGGTTCCAGAAGCGGCATTTCGACGAGCGTCTCGG

CTGGCACGAAACGACAGTCAGCCGTGAAGCGGTTCGTGAGGAGTTGGTCA

ACCGACTCACACAGCGGTCGTCACTGGGCAGTGATGTGGGTCAGAATGGC

GTCGTTGGTGGCCCCGACAAGTTCCAGGTGGTGCCCGTTCGGGAGCTTTG

AGTTCAGGACTGACGCCTTAACCAACAATAGCACAAGAGAATAACGCACG

TTGGTTAAGATTGTGGTAAGTTGGTGAATTCCACCCCGCACAAAGACAGG

AAACTATTCCTCGAAGTAAGAACTATATCCTGTGCCGCGCAAGAACGAGT

ATGTCCAACAGCGAGTCTCCAGATGGCCCGCCCTCTTTCGAGGATGCGTT

TCGTGGCGACGACGTTGAGCAACGCATCTACGGGACGGTTCTCCAGACTC

GCGAGCCGACGACAGCAAGCGCTATCGCCAACGCCGCCGACTGTGACCCG

AAAACGGCTCGGAAATATCTGGGCTGGTTCAGTGAACTCGGTATCGTCAC

GCGCCACAATAGCCATCCGGCCACGTACGAGCGCAACGACGCCTACTTCG

AGTGGCGGCGAATCAACCAACTCGCGGCCGACCACTCCGTCGAAGACCTC

CAGCAGCGCGTTCGGGAACTGAGCTCGCGGATCGAGGGGTACGAGGACAC

GTATGGTGCTCCGACGCCAGCGGCCGTCGACGCCGTCGCCGCTGCCCAAT

CGAGCGACGACCGGACGATCGATGACGTGTACGGTGACCTCGGCGACTGG

GCGACCGCTCGGAACGAGCGAACACGCTACGAACGTGCCCGTCAGCAACG

CGCCCGTAGCGACTGCCAAGAGAAATGACGGACGTCGAGTGTTACAACAC

GGAAGAAACGACTATTGGCTAGCGCGAGAGAGTCGCAATTGTGACTGACG

ACGTCGCGTATCCTTCGGTCGAGCTCATTCTCGATCTCCACGAGCAGATC

GTTGACGAGGGCGACGCCACAGAACCCGGAATTCGATCGGAGGATGCAAT

CGAATCTGCAGTGCAGTACGTCTCTGAAGGATACTTTGGGGAGGTTCCAC

AGACACTGCATGAAAAAGCGGTACATCTGATGCGGCTTCTTGTTGCGGAT

CATCCGTTCGTCGACGGGAACAAGCGAACGGCGCTCCGAACGGTAGTCGT

CTTCTACATGCTGAACGGGTACACGTTCGACTACGGTGACGAAATTCGCG

CCTTACTGCACCGCTTTGCGACCGATGAATCCGCCGTCGACACTGAGACC

GCAGTGATCTACTTCCGAGCGTGCGCTCGTCGCAACTGATAAAGGGACAC

ACAGAGTATGTTCGACACAGAGATGGCGTCCAGCACCGATTCCTCGGCGA

CGGTCGATGAAGAAGTTCGCCGATTATACGAACGGTATCAGGCGGCCGAT

AGCGACGAGGAACGTCATGCGATCGCCCTTGAGATGGGGAAACTTGACGG

ACGCCGCCACGCAGAGATATACGCCGCACTCGAAGACGAGTAAGCCAACA

GAATTCTTCTGACTCCCCGCCGACTGCCCCCACCCCCGGCGACGACGCTT

CGTGGCCGACCGACCATCGCGACGTGCTTCGGCTCGTCCTCGACGAAATT

GAAGACCGGATAGCGGCCCTGTGGAGTGAGTAAGGGATGTCCCAAAGGTG

AGCCTGTATCGCGAACGGTTTTATCACTCCGCCGAAGGTTAACCAACACA

GAACCATGGCCCTCCTCAGTGTTGGTTAACAACGTGGCAGCGAGTAGTTT

GTCCGCACCTGAGAGGCCGAGGCGCGTCAATACGCGTCAACGTACCTCCA

TCTCGAGAGGATTGTTGGCTCGGTTGATTTCGATGTGAGAACGAACGAAA

CAGTCGTCCGAAGTTGTTTCTTGGAAACTCCATAGACTGCATCATACCTC

ATGAGTGGACAGGTATGACTGTGTGAGAATATTTGGAAAGATGCTGGGTT

CAAATCATGAACTCGGCGGCGATACAACGAGTATCGTAACCGAAGTAAAC

AAACCGATATCTGCCTTCTGACAGGGAATTACAGACATAGTAATCTGGAT

TCGTGTCGTTTTGCCGTGAGAGCCCTCCTTGAGTAAACTCAAATTCCCAA

GTAAATCCGGCACCTGGTTTATGCTCTACCGCTTCGTCCGACCACGCTGG

TCTGGCATGGACCCAGAATATCGATTGCCACCTTTGGTCTGTCTTTTTTT

GGATATCGTACTTAAAACGGTTTCCGGAATACTTCGTTTTCTCGGTGCTA

TTCGTCATCTGGACTTGAAGTTCCTCACCGATCGAACTAGGAGAGCGAGG

AATCGAAAGATCGAACGCCGAATTCTCTGGGCCCTCACACGTAATTTGAA

GTCGGTTTAAATCGTCACACATTGATTTGGACGTATTATTTGGGTCTGAA

GATGATTCATCGTCAGTTCCGTTTAACGTCTGCTTCCCAAGACAGCCGGG

GAATGCTAGTGAAGTTGCTCCGAGTGACAGTAGTAACTTTCGTCGGTTCA

TACACGATATCTGTCTCATATAATAAAAGGCACTCCGGTGGGTTAAGTAT

CGATTTTAGTTACTGGATATTAGACAGTCACATTAAACTGTGATTCAATA

GCAGACATTAATGTACCAATGGCGTCATCGCTACCATCAGTTCGGTGTCC

GTAATTGATTCCAGCGATAAGTGGTCGTCCATAATCGTCTTCTCTGTAGT

GGGGTCCACCAGAGTCCCCACCGCGACTAATTGCATCGGTGTCGAACGTA

GAACCGTATGCATACGTCACATGACCAGAGCAGATGCCTGTATTAGCACC

CTGCTTTTTCACCTCCCAGCTCGTATCGCCTTCACGGTCTTTGATCTTGG

TACCCGAGATGATCCCGAATACGTTTGGTCCATACGGCGCAGAATCCGTA

TTCTCGGCGAGACTGTAATCAACGTTCGCACCGTTCCGCATCTGTATCAC

ACCAGCATCAACCGGCAATCCCGGATCAACACGAGTGTCGTCCGGGGTGC

CAAAGCGGTCTTCTTCAACTTCGTCGTTCTGGTAACACTTATCAACCCCT

TCGTCTAGGCAGTGACCTGCACCAACGATTACCAACTCGTCATTCAAGTC

GTCGTGTGCAGGCGTTCCAGTCGTACAGTATCCCGACTCAGGTATATAAT

AACATCCACCGGGGATATCAGGGAAGTATTCCGTGTCGTAGTACTGGGTG

TCTGGATCAGTATCGCAGGGGAAGTCCGGCTTAACAGTTACCTTCTCTGC

CTTCACCGGGATATCTTCAACTGCACGTTCTTCCTCGGTTCCTCTACCAG

CGACACCGGTAGCCGAGGCTGGGAGATGATCTTCAAACTGAGTGAACGCC

ATGTCGGGGCGAATCTCGTCTCCACTTGATGTTTCCTGAATAAGGTATTG

AACGGAGACCACCTTCCGTCGACGATGGCCGGAGGTATCAGTCGTTACTC

CCACTCGTACCGGAACTGGGAGTGATGAAAGTTGTTCTTCTACTTGTTTG

CGTACATCATGAGCACTCTCAACGATAGCCCATTTCTCTCGGCTGATCTT

GTAGTATTTGGGCTCTCGATTTGGTTTTACTGCTTGGTTTACCACTGCGT

CGTGGTTCGTATGGACTAGTCTACCAAGGCGAGGGACTTCGTCGTCGAGG

TTCACATCTAACTCTGCAAACGGCTCTGTTGAATCACTAGACAGCGTCGG

GATGGGTCGCTAAATCAAAGGAATTGAAGCGGGAGACTGCGAATGTACAT

GACGCAAATCTCTCGCTTCACTAGCGAGATTGTCCCGATTTCTCAAAGAG

TTACTGGTGATGGAGACGAATCCGCCGCCCCACAAGGTGGCGGCGGATTC

GCTGACTACGCGCTCGTTTCCCTCCATTGTCTACGGATTTACCTCAACAC

GTCCTACCGGATGACGATTGACCTACTGAAGGAGATGCCACAAATAACCG

GGGAGATCGGCCTCGACACGGCCGATCTCCCCTCGCCATCCACGTTGTGT

AAAGCGTTCGACCGGATCAGCATGAGCGTCTGCCGAGTGCTGCTGCGCCA

GTCGGCGCAGCTGCACGACCCCTCGAAACACGGTGCAATCGACGCCACGT

TCTACGAACGATCAGCTGCGAGCCGTCACTACTGCCAGCGAACAAGCTAC

CGCGTCCAGAAGCTCAAAGTGACGAAGCTCGTCGATACAGACTCTCAAGC

CGTTCTTGACGTACACTGTTCGACGAACCGGAAAGGAAGTGACGCCGACC

TTGCCGAGCAGATCGCCCGCCGGAACGCGGGCGATCTGCGGTCTCTTGCC

GCCGACAAGGGATACGACAAGAAATCGCTCCGCGAATCCCTTCGTAACCT

CGGGATTCGCCCACTCATCAAACATCGCATCTTCGCTCCCTATGATCACG

CGCACAACGCCAGAATCGACGAACAGCGCTACAATCAGCGCTCTATGTCT

GAGACCGTGAACTCGGCGGTCAAGCGCTCGCTCGGCTTCGCCGTGCGAGC

GCGTACTTGGTTCCGTGAGTTCCGGGAAATAGCTCTGATGTGTGTCGTTT

ACAACATTAAGCGTGCCGTGAAACAGTGAAAAACCTACGCCGTATGGCGA

TTCAACACAGCCCTGCAAACGCATCCTGGGACATGAACGAAATCGCTGCG

CCTCCTACGCCCAAACTGCTGAGAGTATTCAGGAACCTACGCCGACCCAT

TCGAGTCAACTTTCTTCTTTTCTCAGACATCCTGTCACCAGTTAATCTTG

TTTCATTCAGGATATATAACATTTACTTGCATTTTCAGACATCGCTCTTC

TGTAGACGTTCGTTGATAGCGTCCAGCTGTCACTCTGGGATCGAAGAGGG

GAGCGACACTGCGTTAGCGGTGTCCAACACGAATGATATAGAACAGTTTG

TCCGCGCCTGAGAGGCCGAGGGGCGTCAATACGCGCCGACGTGATTCCCA

TGCCCGGTACAGACGCACTCGACGATGCATCAGCCGACCACGAACGGTTT

GAAGCGGAACTCGTCGCACAGGATCAAGATGCTGCCCGCGTGGTGGTAGC

GGATATCGACGAGAGGGGAGCCCATTCTCTGGTCAATGACCTCGTCGACG

ACGACGTCGTCACTCCCATCCCCGACGAACGCGTCCTCGTCCACGAGCCA

AGCGACGAGGCATTCGATTCGATTCTCCAACTCGCGGTCTTCCATCGAGG

CTGGACCGCCGCTCGCGACACCGACGAGGGAGAAGAGTGATGCAACCATC

CGTTCTAGTTTAACGAAGAATCCAGTTGCTGTCTGTTGATTTGATATCGT

CGCAACTACCTGATTGAGAAACCCGGTGGGAAGTATGACCTACAGCCCAC

CGGATTCGGTCATAGTTGACCGGATTCAAAGAGCGTTTCCCTCTGATGAG

TTGCGCGAGCGCGCTCGCGCAACGAATCTCGTCCAACGAGAGCGGAAATT

CGACATCGTTGCGCTGTTCTACACACTCTCGTTTGGCTTCGCTGCTGGCT

CAGACCGCTCTCTCCAAGCATTTCTCGAACGCTACGTCGAGATGGCTGAC

TGTGACGAACTCTCCTACGCATCGTTCCACGACTGGTTCGAACCAGGATT

CGTTGCACTCCTTCGAGAGATTCTCGATGACGCAATCGAGAATCTCGATA

CCGGACGAGAAGATTTGAACGGCCGTCTCGAACGCTTTCGAGACGTCCTC

ATTGCTGACGCAACCATCGTTTCGCTGTACCAGGACGCCGCTGATATCTA

CACAGCAACCGGCGACCATCAAGCCGAACTGAAACTTCACCTCACCGAAT

CTCTCTCGACTGGGCTCCCGACACGATTCCGGACAACCGATGGGACGACT

CATGAACGGAGTCAGCTACCCACCGGCGAGTGGGTAGCTGACGCCCTCAT

CTTGCTCGATTTAGGCTTCTACGACTTCTGGTTGTTCGACCGAATCGACC

AGAACGGCGGGTGGTTCGTCTCCCGGGTGAAGGACAACGCGAACTTCGAG

ATCGTCGAAGAACTGCGAACGTGGCGAGGCAACAGCATTCCGCTGGAAGG

AGAGTCGCTGCAGGCCGTCCTTGAGGACCTGCAGCGACAGGAAATCGACG

TACGCATCACGCTTTCATTCGAGCGCAAACGAGGGTCGGGCGCCAGCGCG

ACCCGGTCGTTCCGATTGGTCGGCCTGCGTAACAAGGAGAGCGAAGAGTA

CCATCTGTATCTGACGAATCTGGCGAGAGAAAGCTACAGCGCGCCCGATA

TCGCGCAGCTCTATCGGGCGCGCTGGGAGGTCGAACTGCTGTTCAAGGAG

TTGAAGTCGCGGTTCGGCTTGGACGAGATCAAGACGACCGACGGCTACAT

CATCGAGGCGCTGATCATCATGGCCGCAATTTCGTTGATGATGAGTCGTG

TAATCGTGGATGAGTTACGGTCGCTCGAGGCAAGACAGCGAGAGGGCGAA

GCCGCCGCAGACGCCGACTCGTCGGCGTCGCGGCTCCCTCGGCGTCGCTG

TTCGCTCGCCGTGGAACGCCACGGTCATCTAATCCAGTTGTATCTCATGA

TCGAGTTGGGCTACGAACTGCCGGATTTGGACGAGCTGTTGCTGTGGGCG

TCGCGTAATCCAAATCCACACAGGCAACGGTTACGTGAGCAGGTTGAACG

AGGTGAGTTCGGCTTTGATCGCTACTAAACTAGAACGCATGCCGGCGAGA

GTTCGCTGATATCTACAGCCAAACCATCACGTACGGTTTACTCACCGCTC

GAACGCGGGTATCAGGCGAGTTCAGCCGCACTGATGCTGCCGACGCTATC

CCGTCCACTATCGGTATTCTGAAAGATATCTTTGACTTCATCTCCACGTC

CGACCCGCCAACAGAAATCGAATGGATTGTTGATGACGTCGCAGACGTAC

TCTCCGGTACGAACGTAGACGATATTCTGAGCGAGTTCTACGCGGATAGA

GCCGAATCCACACCGCTTGTTCACTTCTACGAGACGTTCCTCGAAGAGTA

CGACCCGGAAACTCGCCAGCAGAAAGGCGTGTACTACACGCCAGAGCCGG

TGGTAGAGTGGATTACGAAGTCAGTGAACTCTGTGCTGAAAACCGACCTT

GGGCGCGGAGACGGTCTTGCGGACGAAGATGTTACTATTCTTGACCCTGC

CGCAGGTACGATGACGTTCACAGCTGCGGCGACGCGACTGGCGGTCGAGG

AGTACGCGGAGAAATACGGTCGTGGTGGGGTGTCAGCACTGCTGTCCGAC

CACGTCTTGAAGGACTTCTATTCGTTCGAGCTACTCGTCGCGGCGTACAC

TATCGGCCATCTGAAAATGGCGATTCTGTTCGAAGAACAGGGCTATGAAC

TCGACGACGATGACCGCGTGAAGTTATTCCTGACTAATACGTTAGAGCCA

GAGGAAGCGGAACAGACGCACCTGCCGTTCGCGGCGTCGCTCGCCGAGGA

AGCGGAGATGGCCCACGAAGTGAAGGAGGACACCCCAATTTTGGCTATTT

TAGGGAATCCGCCATACTCAGGGCACTCAGAAAATCAGGGGGAGTGGATT

GAATCCCTCGTCGAGGACTACAAAGAGGGATATCCGGAGTTACAGAAGCG

TGGGCAGGCGAAGTGGCTGCACGACGACTATGTGAAGTTCATCCGCTGGG

CACAGTGGAAACTCGCTGACGCCGCTGAAGGTACGCTGGGGTACATCACG

AATCATGCGTACATCGACAATCCGACGTTCATGGGGATGCGTGAGCAGTT

GCTGTTGGAGTTCGACGAAATTTACGTGCTTGACCTACACGGGAACTCGA

ACAGGCTGGAGGAACCCCCGGAAGGCGGTACTGACGAGAACGTGTTCGAC

ATCCAACAGGGTGTCGCCATCAGTATCTTTGTAAAAACCGACAACGTGGA

TGACGGCGAATACGCTGATATCTACCACAGCGACCTGTGGGGAACGAGAG

AGGAGAAGTACGAAGCACTGAGCGGCAACGACATGAACGATATCGAGTGG

GAAGAGGTGACTCCGCGTGAGCCGCACTACCTCTTCGTACCTCGGGACGA

GGAACTCGCAAGTTCCTATCACGACTGGGTGAAAGTGCCACAAATTTTCT

CAGAATACGGCGACCCCGCACCCGGTATCGTAACCACGCACAACGAATTC

GCTATCTCACTCACCCCGGAACGCCAGAAACAGAAAGTCAGACAGCTACT

TGCTACAGACACAGAAAGCGAAGCGCGCGAGTACTTCAATCTGTGTAGCC

AAGACCAGTGGCTCTACAGCGACGCGAAAGAACATCTGGAAACCACAAAC

TGGGAGGATAAAATCGTTCAAATCCAGACGTCTCCGTTCGACAAGGAATA

CACCGTCTACGACGAACACGTCGCAGTACATCGCCGTGCTGGGCGATTGT

CGAAACATATGCTAGCGAGCGACAACATCAGCCTCTCAGTGCCGCGGCGA

ACGGAACGAACGCCGTTCGACCACGCGTTCGTGACGGATGGACTGATGAC

GCACCATGGGATGTCTACGAAGGAGGTGAATTACCAGTTCCCGCTGTATC

TGTACCCGAACGCGCCGGATGAAACGTCCTCAAAACTCCAAAACACATCT

CGACAGACGAACGTGAATCCGCGTATCGTCTCCCAGCTTTCTGAGGTCTA

CAGCGACGAGGTAACGCCTGAGGAAGTGTTCAACTACACGTATGCTGTGT

TGTACACGCGGACGTACCGTCTGAAGTACTCGGAGTTCATGGAGACGGAC

TTCCCGAAAATTCCGTTCCCTGAGGACGAAGCACTCTTCCGGAAGTACGA

GTCCCTGGGCGCGAATTTGGTGCAGTTACACTTACTCGACCACCCTGATT

TGGAGTCGCCGGGCGTGCAGCTTCACACTGAGGAAGATGGTGATGGTGAG

AATGAGGTTTCGGAGAATACTGGCGAGTACTACCGGCACTACGATGAGGA

AGAAGAGCGGCTGTATATCAATTCTGACCAGTACTTCGCCCCAGTTCCGG

CGGATGTGTGGGAGTACGAGATTGGTGATAGGCCAGTCGTGAAGAAGTGG

ATTCAGAACCGTATCGGTGAGACTCTTTCGAGTAGTGATATTCGGAAGTT

CTGTCGTATTGTTCGAGCGGTGATGGAGACCGTCGATATTCAGGATACCT

TAGATATGTCCTGGGAAGGGGTCGAATCGGACTATCTGACGTTCGAGTTG

GAAGGCCAGCAGACGCTCGACGTATAGTGTCGTTAGGCGTTGCTGACATT

ATATCGAAGAGTTCGGATATTGTATCGTACTCCTGAGTACGCCCGGGCCG

GCGGGAAGCCGCCGACCCGGGTGGAGAACACCGACGACGGTGTAACACAT

GAGCCTACGAGAGGCGCGTGTCCGTCTTCAGGCCGCTCCGAGCGAGAGGA

CCGGCCCGTCTCGTCGTACCGTCCGGAGCGGCTTCAGTTTACCGGGACCT

ACCCGACTTCTGATACTGAATCACCCGACTACGTAGGGTATTTGCGCCAA

ACAAACTCAGTATGCTTACATAGGAAACTAAGCCGCTGGCTCACCTACGA

CCGTCCATGCCGCACGACTCCGACGACGGCACGAACGGGGAGGACGCGGA

CGACTCCCCGCGGACGTACAGAGAATACCGCCGAGCGAAGGGCCGGACAT

CCGGGAAGGTCCACGACGACCACGACGCGCCACGCTCCCCGGCGGAGGAC

CGGGACGCCGACGGCCCCGGCTCCGGCTTCGACGGCATCCTCGGAGCGGG

AGAGGACGTGGAGAAGTCGGCCCAGGAGACGCTGGAGGACGCCCTGGACG

ACTGGGACCCGACCGGGACGGACGTAGCGAAGGGAGACGAGGAGCGGGTC

AGCCTCTCGCTCTCCCGGGCGGACATCGAGGAGGCGTCCCTCACGACCGA

GGAGAAGCGCCTCCGGCTCCTGGAGGACATCCGGGACCGGCTGGCGGCCA

TCGACGGCCCGACGCCGAGCGCGAAGCAGGTCCTCCGGGAGGCCGCGGCG

GACGCGGACCTGAGCAAGAGCGCGAACGCCGTGGACCTCCTGGAGTCCAT

CGAGGACGAACTGGAGCGGCAGGAGGCCGTCGCGGAGGCGGCCAAGGACG

CCGTAGAGCAGTACGTCGAGGACGGCGGGGACCTCTCGGACCCGCTCTCG

GAGTTCGCCACGTTCCTCCGGGGAGCCATGCCGGACAGCCGGGACCTGGA

CGACGACGGGAACCAGGCCCGCCGCCGGGCGCTCGCGTCCCTCGCCGCGC

TGGAGAACGAGGACGCCGGAGAGGACGGCACGCCTGCCTGAGGGCCGTCC

CGTGACCGACGAGACGCTTCCCCCGGAGGACGGAGCCGGCGACCACGAGG

CCGACGCAGAGCCGACGCTCGGCGGGGAGCTGGTCCGGGAGCCGGTCGGT

CCCGCGGACCTCTCGCTCCCGGAGCGGTACGGCCCCGGGGACCTACGCCT

GTCAACAGCAACAGAGACGGACGGAGACAGTCGTAGCGCCGACGAGAGCG

GCGCTGGTCCGTCCGCGGAGGGTTGACTACGCTTGACATGACGGAAGACT

ACCGCGCTGTCGAGGTCCCGGACGCTAAGGACCCGGCGGAATACTCGTAC

CGGGAGAGACGCGCCGAGTTGCTCTCCCTCATCGAGGAGGCGGGAAGCCC

TCGGCTCCTGAACTACGCCGCCTACGGACGACGCTACGACGTGAGCCGCG

AACAGGTCCGGAAAGACGTACAGCGTCTCGGGTCATACCTCAACGAGGCC

GCGGACGATGACGCGGCCACGCTGGAGGGAGAGGCGTTCCTGTGGCGGTG

CGCCCGGGAACTGCTGGAGGACGAGGAGTACCGGAAGGCGGCGCAGACCT

TCCTCGACCTCGAAGAGTGGCGTCGGCAGAGCGACTTAGAGGACCTCTTG

GAGCGTATCGAAGCCTTAGAACAGGAAGAACGGGAGAGCGAAAGCCCGTT

TCGGGTGAAGTAGATGGCCGGACCGACTCGTAGAGACGTGGAGCGGGCCA

TCCGGCAGTACCGGGAGCAGGACCGTCCGGGGGACGCGGACGGGCCGCCG

GGAGACGCGAAACTCCTCGTGGGCGTCTCTCTGGACGCCTTCGACCTCCC

CGCGGAGGCCCGGGAGTACGTCGAGGAGGCCGCCGCGCTGGACCACTCGG

CTCCGGTCGTGGTAGCGGACGACGAGAGCGCCGAGGACGCCGCTCACACG

CTCGGCCTCCCCGACGCCGGGAGAGAGGCCGGCTTCGAGGCGGAGGCGTT

CCTGGCCGACGCACAGGACGGAGACGACGACTCGGCGGAGGGGGACTGAT

GGACAACGGCCCCGTCCGGTGTGGCTCCTGCGGGGAGACGTGGCCGAAGA

ACCTCGCCCCTGTGCTGAAGGGAGAACGCGACTGTCCCGCCTGCTCGCCG

GACACGTTCCGCTAATTAGTCTGAGTTAGCCCTATTCAGGAGGTAGAAGC

CCCGTACTCCGCAGATTTAAGGGATTAGCGTAGTAACTACTCTGTGATTA

GCGAAAGGCGGGACCGGAGAAGGACCCGGACCTCGACACTCCATCCTTCT

CCCGCCGGGACCTAATCGCTTACGGGACGCTCTACGGACCAGAGCGCCCG

TAAGAGACAGACGACACCAGAGTCCGAGGGCCAATGGCATCGACCATGCT

GGGACCATGAGCCATGAGCCAGAACACGGACTCCAACGACCGCAACGGAC

GTACGAACAGACCCATCTCCGGGCGGGACCTCTCCGCGGTCCGGGACCAC

CGGGACGACCTGGAGGACCTGGCGCGCAGTGACCTCCCCGTCTCCTGGGT

GGCACGGGCGCTCCTCGACGCCGAAGCCGCCGCTCGTGGAGAGTGACGGT

GCCGGTGAAACCGAGGACGACGGGGGTCCCAGCGCCCGCCATCACCGCTG

AGGTAGAGGACGACGGCACGCTCCGGGCGGGAGGTGGTCGTCGTGTCGGG

TGACGGGACGCTCGGAAATGAAGGTTCGGACCGCGGGGGCTACGCCGCTC

CGGAGGGGTCGCCAAAAAGTCCCGGGGAGAAGGGGGCCGCTCCCTTCGTC

CCGCTCCGGGACGCGCCGGTCCCGTGGGCGGACGAACCGAACGCGATGGA

GAAGTCGCTTCTCCTGAAAACCGCCGTACTACACCCGGACGTGACGCCGG

AGGGCATCCCGTTCCACGCCCTCCTGACCGAAGTCTACGGGGACGGATGG

GCCTCCGGCGGCCCCGAGTACGGACGGGCGTACCGCTTCATCCAGCGGTA

CGGCGATTTCTTCGACACCCGGAAGCCGTCCGGCGATTTGCTGTGGGTAC

GCCCCACCGGGAAAGCCGTCGCCTTGATACGTAGCATTCGTAATTCCGGA

ACGCCCGGGAGCGCCCCTGACGCGACGGACCGCTGGGACCGGCTCCCGCG

GGACCGGGCCGCGTCGGTTCTCCGGTCGGTCCGGAGGGTGGAGACGGACG

GCCAGCGGTCCCTCCTGCTTCACCTCCTGGCGGACCACGCGGAGACGATG

CTCACGGCGGAGGGGGAGGCGAAGACGCTTCACCTCCCGGACCGGGACGG

CCTCCCGGCGGGGGACCGCTTCACGGCTCCAGAGAAGGCGGCGGGAACGC

GGGAGCGGTTCGAGACGGCGGCGGACGCGCTCACAACTCGGTACGACGTG

GCCTCGTGGGTGACGCTCACACTCCCGCGGGAGTGCGTCCCGTCCGTCTA

CGGCTCCGTGGAGACGCTACGGGAGGCGCTGGACGACCTCCACTCCCGCT

TCCGGTACTCCCGGGCGGACCGGCCACGACCCGGGCACGTCCCGGACTAC

CTGGCGGTCCTCGAACCACAGAGGGACCTCGTGGCCCACCTCCACGTCGT

CTACGGCGGCGAGGAGCGGGTGATGGCCCGGGAGGACCTCCGGGCAGACT

GGGCGGACCTCCTGGACGCGCCGCCGGGGAAGCCGCCGCAGGTAGACGTG

CGGACGCTCTCGCTCTCCCCGACGGAGTGGTCCGTGGCGGCGGTAGACGG

GGAGGACGTGGACGCCTACCCTGGCGTCCGGGAGTACCACCGGGAGGGCT

TCCGCGCTCTTGCCCGGCTGGCAGAGATGGAGCCGGACGCGCTCCGTGGG

CTGGCGGATGACCTCGCCAACGGCTTCCGGCAGGATGAGGGCCGGCCTCT

CGCGGGGCTGGCGCTCCCGTTCGCTACGGAGGCGCGGCTCACTACGACTG

CCCGGGCGCTCCCGACCACCTAACCCGGGGGGGTGACGACGAGCGGACCG

GCCTCCAGCAGGGTCAGGCGTCCGGCTCCGCCTCCGCCTTCACCTTGTCC

GCGAACTTCTCCCGGACCTTCTCTATCTTCGGGACGACGTTGAACTGACA

GTAGGCGTCGTTAGTATTTCCGTCAGCCGTGCTTACCTTCTCGAAGTAGT

CCTGGTGGTGTTCCTCCGCCTCGTAGAAGGTCTCGAGCGGAACTAACTCC

GTGACCACCTCGTCGTCGTACTCCTCGTCGAGCGCATCGATGTAGGCCGA

CGCCTGTCGCTTTTGTTCGTCGTCGTGGAACAGGACGATAGAGCGGTACT

GCGTCCCCACGTCCGGCCCCTGTCGGTTGAGTTGGGTCGGGTCGTGGACC

GCGAAGAACACGTCGAGCAGTTCGTCGTAGCCGACGACCGCGGGGTCGTA

GTCGACCTGCACGACCTCGGCGTGGCCGGTGCTCCCCGAACAGACCTGCT

CGTAGCTCGGGTTCTCCGTCTCGCCGCCGGCGTAGCCAGAGGTGACCTCG

CTGATGCCGTCGAGTTCCTTGAACGCGGCTTCGACGCACCAGAAACAGCC

GCCGCCGAACGTCGCAGTCTGTGTGCTTCCCATAGCCTCGGTTACGTCGC

CGGGGAATTAAGCCCCGCGGCGGGGGCAGTCGCGGCCGGGGCCCGCGGTC

GTCATCCCGATGTACCTCCCCACGAAACTGTACGCGAAGCCAACATTTAT

CCGCCCTGTCTGTCAGCAATTACCATGGAAGACGTATTCGTCGCGAGACT

CATGTCCACGACCCTCCACACGGTCACGCCAGACACGCTCGTCGAAGACG

CGGCGCAGTTGATTCTCGACAACAACATCAGTTCGGTCATCGTCGTCGAC

GAGGACAACCGGCTCGAAGGCATCCTGACGACGACGGACTTCGTCGACAT

CGTCGCCAAGAGCCAGCCGAAAGCGCAGACGACCGTCGAGCGGTACATGA

CCACGGACGTGATTACCGCGGGCGCGCAGGACTCCATCCTCTCCGTCGCG

GAGTCGATGACCGAACACGGCTTTCACCACATGCCCGTCGTCGACGAGGA

GGAGGGCGTCATCGGGATGATTGCCACGTCGGACCTCGCCGCGTACCTCT

CGCAGACCGGGCAGTTGGCGCGGTCGTAGCGTCGTCGCTTCCGGCTGACT

TCCGCGCTACTCCACCTGTTCGGAGAGGCTCTCGACTATGCTCGCCAGCC

GCGGGTCCGTCGCGTGGCCGTGGTGGACCGCGAGGGGCGAGACGCTCACT

TCGCCGTCGACCATCGCGCGGCGGTCGCTCCCGACCGGATAGCGGTCGCG

GTGTTCGTCGTCGAGCGGGAACGGGTTCTCGAAGCCGACGGAGTCCGGCC

ACGAAATGTCCTGTAACCGGACGTACACCTCGTCGTCGTCGAGGTCGTGT

TCGCGGTCGCCGTCCGGAAGCGCGCCGGCGTCCGGGTCGTGGTCGACCTG

CTGGTCGTAGTCCGCGAAGGGGCGGGTCACGCGCATTCGGGGGCTCGGCA

CGTCCGCCGGGGCGTTGACGTTCAGGAGGTCGACTTCGTCGAACACGTCG

GTCCCGAAGACGCGCTCGGTCAGGGCACGGGCGACCCGTGCGGGACGGGA

GAAGTCGTACTCCTCGGGCGGGTGACAGAAGAAGTCCCTGGCGTGGTAGC

TGGAGACGGCGACGGCGGGCGTCCCGAGGAACGCGGCCTCGACGCCCGCG

CCGACGGTTCCCGACCGGCCGACGACGTAGTTGCCCGCGTTCGGGCCGTC

GTTGATACCGGAGACGACGATATCGAAGTCGGCGTCGAGGCCGCGAAGGG

CGTAGGCGACGCAGTCCGCGGGGGTTCCTTCGAGAGCGTACCCCCACGGG

TGGTCGCGGCGGACCGCGTGTTCGTTGCGGGCGCGGCCGACGCCGCTTTG

GTTCTCGGCGGGCGCGACGACGGTCACGTCGCCGACGGCGGTGAGTTCCT

CGCGCATGGCGGCGATGCCGGGCGCGTCGATGCCGTCGTCGTTGGTGAGG

AGGATTCGAAGCGAAGACGAGGTGTCGGTCATCGAGTCGTGCATTTCCGC

CGGTCGCCGTAGCTATTCCGGATTCTGAGCCGAGGCCGCGTCCTCCCGCG

CGTCGCCTGCATCGCCGTGCCGCTGCATCGCCGCGTCGCCGTCGACCGCC

GAGCCTCCTCCACGCCGACGGGCACTCGACCGAACTCCTGTCAGCGTGAC

ATCCATAAACTCGAGACACTCAGGAATAGCCGAATATAATCCAAACGACG

ACTTTATGTAATTAAGTTCGTACTAACGAACGAATGCGCACCGAACCGAT

GCGGCCGGCACGCCGGGCACCCGCCGACATCGCGGTTAACGTTCCGTTAA

CTCGTCTGTTTTCGGTAGTTTCGTCTCTGTCACGGTCGGCGGTCCCGTCC

CCGTTGGCTGTCACGACCGCCGAACGCCCCGCCGCCCTAAGCGAGGTGGG

CGTTGCGTGAGCTCCGTCGGTGGAACCGACGACGTGCCGTGGGACGACGT

GGGCTTCGTCATCAGCTCTCGCTACCGCGTCTCCGTTCTCGATAGACTCT

CGGAGGGGCCGGCGACGCCGACGCAAATCGCCACCGACTCCGAGAGCGCG

GTCGCCCACGTCTCGCGGGCGTTACAGGAACTCAGAGACCGCTCGCTCGT

GGAACTGCTCGTCCCGGAACAGCGCCGAAAAGGGCGGGTGTACGGCATCA

CCGACGACGCCGAGAACATCTGGCACGTCATCCAAACACAGCAGATGGCG

TGAGCGCGGTCGCCCGCGTCTCGCGGGCGCTGCGGTGCGTCGTGTGACCC

CGACGACTCACTTCGTGGCGATTCGCTCCGACCCCTTGTACAGGAGCTGG

TAGGTCCGCTTTGCGAGGTTCATCCCGACGCCGGTCGACTCGACGACGTA

GTACGGGACGAGTTCGCCGCCGAACTTGGATTTGTAGTTGCAGAGCCGCT

CCGTGTTCGCGCCGACGAGGTCGTACTTCGTGACCGACGCGAGCGCCTCG

TCTTCGACGATGTCCTCGATGATGGTCCAGTGCAGCAGGCTGTTGATGCT

GACGCCGTCGTACTCTCCCCGCGCACCGCCGAGCCAGAAGTAGCCCACCT

CGTCGGAGAAGGTGACAATCATCCCGCTGAGATACTCGCCGCCTGGGTCG

CGGGCGACGTACACCCGACACCGGTCGCCGAGGTTCTCGACTAGCGACTC

GACGAACTCCCACGGCATCCCGAACGGTTCGTCCTGCTCGTCGTACCGCG

AGACCACGTCGTCGTAGACGACGCGAGCGCCGTCGAGTCCCTCGTCCGCG

ATGGTGATGTCGAGTTCGCGCGCCTGCCGAATCTCGCGGCGGAGGCTGCT

GCTGAACTGGGCCTGTATCTCGTCGAGCGAGCGCCCGCCGAGGTCGAGCC

GGTAGGTAAACGAGGGTTTGACCGCGAAGCCGGTCCACTCGAACGGGCGG

GGGTCGAGGTACGCCGGCGAACAGAGCAGGCGGACGAACGTCCGGTTCGA

GCGGATGCCGAGCTCCTCGACCACGAGGTCGGCGAACTCGCGGTTCACCG

ACTCGTAGGCGCTCTGTTTCGGGCTGTTCGGCATCATCACCGGGCCGATG

TACGGAATCGCCATCGACGGCGGCGGCGAGACGACGACCCGTCCGAGGGG

GTTTCGGCGGACGAACGTCGGGAACAGCGCGACCGGCTCTTGGCCCTTGA

AGACGCCGAACAGGTGCATTTCGCCGTCGAAGTGCTCGTCGATGGCGGCG

AGCACCGCGGGGGTGTGGAAGACTTCGGTGCCCGACGCGGGGAGCGCGGC

TCCCCACTCGTCGAGGCTCAGGGGTTCGATACGGAGTTGTGACATAGGTC

GTGGTCGTGTGGGTGGGTGGTCGTCGGTCGGTGCGTGGGTTGACGCGTCG

GTCGGTGCGTCGGTCGGCTCGTCGAGCGGGTCGTCATCGGTGGAGGAACC

CCGAGACGAGCCGCGACGGGAGGCTCGTCCCGTTTCCCCGGGACAGCATC

TCGATGCCGGGGCCGAGGCGGCTCTTGTACAGGTGCGCGATGGTTCTGAC

CGGGAGCGCGCCGTGTTCTATCTGGTAGAACGTGACGAGTTCGGGGTTGT

ACTTGCTCTTGTAGCGGTTGAGTCGGCGGTTGTTCGCGCCGACGAGGTCG

TAGCGGCGCATCCCGTCGTCCATGCCGTCGCGCATGATGCGCCAGTCCAG

CAGGTCGTTGACGTCGATGCCGAAATCGAACTGGGGGCGGACGCCGCCGA

GCCAGCGGTAGAGCGTGTCCCCGTTCTGGAGGACGACGATTCCGCTCACG

AACGCCCCGTCGTACCGGAAGACGTAGGGCCGAACTTGGCCTTTCGGGAG

CGAGTCGTACAGGTCCATGACGAACTGCGGCGGCACGTCGAACGACAAAT

CCTGTTCCTCGTAGCGGTTCTTGACCAGCGTGAGAATCGAACAGATGTCG

TCGCGGTCGCCCTCCTCGATACCCGAGACGAGCTTTCGACCGTCGCGGAT

GTTCCGGCGGGCGCTCCGGCTGAACGACATGAGGAGGTCCTCTTCGCCGC

GGGTCAGGTCGACGTTGTAGGTGTACTTCGGCGACACGTCGCAGCCGCCC

CACAGGAACGTCCGCATGTCGGTGTAGTAGTCACCGACGCGGACGTGCGT

GTAGCTCGGGCCGATGTGTTCGCCCAGCCACTCGGTACAGCCGTCGATGA

ACTGGTTGCGGCGGCGCTCGAACTTCCGGCTCTTGATGTTGCCGGCGTCG

AGCATCACGGGCCCGAGGTAGGGGACGCCGATGTGCGGCGGCGGCGAGAA

CGCCGTCTTGACCAGCCCCTTTCGAATCTCGAAGACGGGGAACAGGCCGA

CCGGCTCCTCGGTCTTGTAGCCGACGAGCAGGTGGAGCTTCGAGTTCGAG

TAGTTCGCTTGGAGCCGAAGCGCCTCGTACTGGTGAAAGAGGCTCGCCTG

ACTCGACCGACTGACGTACGTGTCCCACTCGTCGCGGTCGCGCTCGTCGA

ACTCGACGATTTCGATGGTCATAGACTGCGGGCCGACCGGCGGTCTTCGC

ACTGCGTCCGTTGTCGTCGACAGCAGCGATTCCGGGCGACGCGTTCGAGA

TGTCCAGTCCGACCGAACCGGCCGAAATAAGCCGATTCACCGGCCGATTC

GGCGGCTCCGTAAATCGACCCGCGATTCGGTCGGGTCGCGTCGGCGTGTA

TCATTCGAAAAGACATTCTCGAACTACTGTGACGGGAGGACGGAGATTTG

TTATGAAGCGACTAGCCGGCGTTCGCGCCGGCCTACACGCCGTCAGGGGG

TCTCTGCCGCCGCCCGTAGGCGCTCGATACCGTCGGCGTCGATGCCGTAG

CCGTGCGTCGGTTCCCCGGAGATTCTGGTGGCGACGTACTCGGCGATGGC

GTAGGCCATCCACGCCTGACACCACCGCATGAGCGTCATGCGCTTGGTGT

AAAAGCGGTGTTTGCGGAAGTAGAACCGGCCCTCGGACGGGACGTACATG

TTCGCGAGCGCCCAGTCGATGGCGCGCTCGGCGCGGTCGTACTGGCCGGC

GTAGGCGAACACGAGGATGCCCTGCGCCGTCGCGTGAATGTCCCGCGGGT

AGGGATGCTCCTCGTCGAAGTTGGGCGCGCCGTCCTCGTCGAACAGTTCG

CGGGCGTAGAAGTCGAGGCCGCGAGCGAGCGTCTCGTCGTACCGTCCGCT

GCCGACGACCTGTTCGTAGCGCTGGAAGCACTCGACAATGAAGCCGTTGT

GGTGGTTATCCATCGAGAGGTGCGAGGCGCTCGCGGGCTCGCGGTAGTAC

CACCCGCCGCGGTCGGTCTGCTTCGACGCGACGAAGTCGTACACCTTCTC

GGCGCGTTCGAGCAGTTCCTCGTCGCCGAAGTGGTCGTACAGCTCGACGA

ACAACCGCGCCCCGATGGCCAGCGCGTTGAGCGTGTAGCTGTCCTTCGGG

TGCGAGAGGTAGTAGTCCATGACCGCGCCGTCGTCTTTCGGCCGGTAGTT

GAGGTCGTCGAGCAGCACGTCCACGGCGGTTTTGGCGATGTCCGGGTAGT

CGGGGTCGAACTCCCGGCCCGCGAGGAGCGCCTTGACGCCGTAGGCCGTC

GAGACGATGTTCGGTGAGTGCGGAATCCCCGACCGCGGGCCGTTGCGACC

GGAGATGTGCTGGATGACGTGGCGGTGACTGCCGGCGAAGCCGCTGTACC

CCCTGGCCTGGTTCTCGACGAGCCACTCCGTCAGCGATGCCGCGTCGTCG

CGGTACGTCTCCGCCGGGCGGTCGCCGTCGAACCGCTCGGCCAACTGGGC

GCGGGTCCGGTTCGCCATGGCGAACAGCGTGATGCCTTTGAAGTTCCGGC

GTTGCTCCACGAGAAACAGCGGGCGGACGTTGACGGGCGAGCGCTTGATG

AACTCCTGGACCGCGAGGTTGAACCAGCGGTTGTCGATGGGGACCGAAAG

CAGGAGCCTGCTGCTCATGCCGTCGCCGTAGTCCCAGCCCTTGTAGTCGC

GGTCGCGGGCGTAGTCGAGCACGTCGTCGAGCACCTCGACGTACCGCTGG

AGTCGCTCCGTGTCGGCCGCGTCGTCGCCGTCGCGGTGATACTGCGTTGG

TTGTTTCATGGTGCAGAATAGCCTCTCTGAAGGGTGTTGGCTGTCCGAGA

GGAAGCCCGCTCGTGGCTTTGTTATGGGGCCGATACCGACCGACGATGCC

GGATTCTACCGGGAGAGAGAGAGTAGTTCGAACCGCCGGCGGTCGCGTTG

TCGGTGGTCGCGTTGTCGGTCGCCCTCCCGTCCGAATCGTCGCCGGCATC

GTCGGAGTCGCTGGAATCGCGCTCGGCGAGGAACTCGCGGTAGTAGTCCG

CGACGGGGCCGACCCACGCGCCGCGGTCGAGCGCCCGTTCGACGATGCGG

CGGTAGAGCCGGCGGTAGCCGGGGAACTCGTCCTCGTTGAAGTACCGCGG

GTGCCACAGCACCGTCATCACGGCGTCGTTCGCCTCGGCTTCGTCCAACA

GGCGCTCGCATTCGGCCCACGCCTCGTCGAACGCCGACCCGGGGTCGGGA

AGCGACACCTCCATCAGCGTCAGCGGGAAGACGACGAAGTCGTCGTCGAA

GGGTCTGAACGGCCGGTAACCGTGTTGGAAGCCGTGTTCCGAACTCGACC

CGGGGCTGGCGTCGTACTTCAGGCCGAGTTCGCGGTGGTAGCGCCACGTG

TTCGGCCCGAGTTTGAGGTGGTGCTGGCGGCCGCCGACGAGTTCGTGGCC

GAGTATCGCTTCGAGTTCGCGCGTTTCGGTTCGGAGGCGGTCGAAGTCGT

CGCACGACCGGAACGAGCCGTGGATGCCGACCTCCCAGCCCCCCCCGTCG

AGGTCGCGGACGGCGTCGGCTATCGACGCCGAATCGAGGTCGTACCGGCC

GAGGTGTTCGACCCAGTTTTTCGGTTCGAGCCAGTCCGACAGCGACCCCG

TTTCGAGGAGGCTCGGCTCATTGAGGAAGTAGAACGACGAGCGGACGCCG

AGGTCGCGTTCGAGCGCCATGATATCCTCGAACTGCCAGTAGGGGTTCTC

CCGCCGGAGGAGCGTCCGGAGGTGGTAGCCGGGGTTCGACTTCGCCGCGT

AGTACAGCCCCTGATACGTCTTGTACGGGCGGTCCACGTCGTGAGTCAGA

CAGACCGCGAAGCCGGGACCGGAGCCGGGTTCGCCGGGCCCGCTCGGTTC

CGAGGCGGTGTAGCCGGGGCCATCTCGCGCGTCCGCATCTCCCGTCTTCT

CACGCATCCCCGTACTCCGAGAGCGCGGCGACGATTCGCTCGGCGGCGCT

CCCGTCGCCGTACAGCGAGGGCTTTTCGGGAAGTGGGTCGTCGTCGGCCA

GCGCCGCCCGAATCGCGGCCGCGTCCGCGCCGACGAGGACGTTCCAGCCG

GCGTCGACGGTCTCTGTCCACTCGGTCTCGTCGCGGAGCGTGAGACACCG

GGTGTCGAGGTAGAACGCCTCCTTCTGGACGCCGCCGGAGTCGGTGGCGA

CGCGCTCCGCGCCGTCGAGCAGGCGGACGAAATCGAGGTAGCCGACCGGC

TCCACGAGGACGACGTTCTCGGCGTCTGCGAGCCGCGACCAGAGGCCGCT

GGCTTCGAGCGCCCCTTCGGTCCGCGGGTGGACCGGGAAGACGACCGGGA

GTTCCGACGACGCCAGCCCCTCCACGATGCCTCCGAGGCGGTCGCGGTCG

TCGGTGTTGGCGGCGCGGTGGACCGTCGCGAGGACGTACTCGCCGTCTCG

GAGGTCGAGGTCGTCGAGTATCGACGACCGCTCGCGGGCGGCCTCGCGGA

CCCGGAGGACGGCGTCGTACTGCACGTCGCCGGTCACGGAGACGCCGTTT

CGGATGCCCTCGGCCGCCAGCGTCTCGGCGGCCGACTCGGAGGGCGCGAA

CAGCAGGTCCGAGCAGTGGTCGGTGAGCACGCGGTTGACCTCCTCGGGCA

TCGCCCAGTTGTGGCTCCGCAGGCCGGCCTCGACGTGCGCCAGAGCGGGG

TCGCGCTTCGCGGCCACGAGCGCCGCCGCGAGCGTCGAGTTGGTGTCGCC

GTAGACGAGCACCACGTCGGGGGCTTCGTCGGCGACGACGCCGTCCAGCC

GGGTCATCATCTCGGCGGTCTGGGCGGCGTGGCCGCCCGAGCCGACCCCG

AGGTTGTAGTCCGGCTCGGGGATGGCGAGTTCGTCGAAGAACACGCCCGA

CATCGACTCGTCGTAGTGCTGGCCGGTGTGGACGAGCACCTCGTCGTGGT

CGCGTCTGAGCGCGTCGGAGACGGGGAACGCCTTGATGAACTGCGGGCGC

GCGCCGACGACCGAGAGCACCTTGAGACGGCTCATCGGCCCGGCCCTCCG

TGCGACGAGTCGGCCGCAATCTCGGGGCCGGCCTCGGTCTCGGACGACTC

GCCCCGCGGTCCGCCGCCGATTTCGTACACGCGGTGGCCGATGTCACCGA

GGGTGCCGCGGCCGTCGACGACGACGAGGTCGTCGAAGGCGGCCCAGTCG

ATGCCGTCGAACTCCTCGTGCGGCGTGACGACCACGGCCGCGTCGATGGC

GCGGTTCGGGAGGTCGGAGAGCGAGACGGGGGTCGCGCCGAAGGACGCGA

CGACTTCGTCCGAGAGCATCGGGTCGACCGCGAGGACGGTCGCGCCGAGG

TCGTTCAGTCGGTCGATGACGCCCGCGGCGGGGGTCGCGCGGGTCTCGGC

GACGCCCGGGCGGTAGGTGACGCCGAGGATGGCGACGGTCGCGCCCCGTA

TCTCTCGGCCCGCCGCGGCCAGTTCGTCGCGGAGCTTGTCGGCGGTGAAC

GCCGGCATGCTGTCGTTGACCTCGCGGGCGGTCAGGAGCAGCGGCGTCTC

GGCCGCCACTCGGCTCGTGATGAAGTACGGGTACCACGGGATGCAGTGGC

CGCCGACGCCCGGGCCGGGCGCGTGGATGTCGCAGTACGGCTGGGTATTG

GCCGCGTCGATGGCCTCGGTCACGTCGATGCCGAGGTCGTCGCGGAGCCG

CGCGAGTTCGTTCGCGAGCGCGATGTTCACGTCGCGGTAGACGCCCTCGA

ACAGCTTCACGGCCTCCGCGGTCGTCGCGTCGCGCACGGGAATCACCTCG

TTCGCGGTTATCTCGCCGTAGACGAGCGCCGCCACGCGGGTGCTCTCGGC

GTCGACCCCGCCGACGATTTTCGGGTGCGACTCGGTGATGTCGCGGAGCG

CCCGGCCAGACGAGGTCCGCTCGGGGCAGAACGCCACGCCGAACTCGCCG

GCCGAGAGGCCGCTTCGGTCCGCGAGGTGCGGAATTACGAGGTCTTTGCT

CGTCCCCGGCGGGACGGTACACTCGACGACGACGAGGTCGCCGGGCGCGA

GCCCCCGCGCGATGCTGTCGAGGACGGCTTCGAACGCCGCGAGGTCGGGT

TCGCGGTCGTCGGTCAGCGGCGTCGGGACGATGATGACGTGCACCGCGGC

TCCCTCGGCCGCGGCGACGCCGTCGGTGGTCGCCCGGAGCGCGCCGCGTT

CGGCCTGCTCGGCGACTAACTCGGGCAGTCCCGGCTCCTTCGCCACGTGG

CACTCGCCGTCGTTGACGCCGCCAACCACGTCCTCGCTGATGTCGACGCC

GGTGACGTTGCCCGTCGCGCGGGCGTAGACGGCCGCCAGCGGGAGGCCCA

TCTTCCCCAGGCCGTACACCGCGACGGGGACCTCGCCGGCGCGCAGGGCC

CGCGTCTGTTCGTCGGGCGAGCGGTCGGAGCCGTACAGTCCGGGGTGCTC

GCGGCTCATCGTCGACCCTCCGAGGGTTCCGTAGTCTCAGCGTTTTCCGT

GGCTTCCGCGGTCTCTGCATTTTCCGCAGTCTCCGCGGTCTCGACGCTCC

GAACCGTCGATTCGGCGTCGGCCTCGATTCGCCGGGCGAGGTCGATAGCG

CGCAGGCCGTCGGCCGGCGAGACGAGGACGGGGCCGCCGTCGGTCGCGGC

GTCGACGAACGCCTGTAACTCGCGTTTCAGCGGCTCGCCGGTGGAAATCA

TCGGCTGTTCCATCACCACTTCGTGGCGGTAGCGGACGCCCCCGTTGTCC

CGGAGGTAATCCGGGCGCGACTGGCGGAAAATCTGGACCGACTGGTTCAG

GTAGTCCACCCGGATGAGCCGGTCGCGCGCGGTGATGTCGAGGGTCCGCG

TCTTCTGCTGGGTGAGCCGACTCGCCGTGAGCGAGGCGATGACGCCGTTG

TCGAACGTCACCTGCGCCGTCGCGTACTGCTCGTCGGGCGTGGCGGTCGC

GGCGACGCGGTCGACCTCGGCATCGACCAGCGAGAGCACCACGTCGATGT

CGTGAATCATGAGGTCCATGACGACGCCGTCGCCCATCTCGCGGTTCAGC

GGCGGGCCGAGCCGGTTCGCGGTGATGGCGATGGGTTCGACGCCGTCGAG

GAGGTCGGGGAGGGCGCGGACGACCGGATTGAACCGCTCGATGTGGCCGA

CCTGGAGCACGACGCCGCGGTCCTCCGCGAGCGCGGCCAACTCCGCGCCG

ACGCCGAGGTCGTCGACGAAGGGTTTCTCGACGAGCGCGTGGACGCCGGC

GTCGATGCACTGCCGGGCGACCGACGCGTGATAGGGCGTCGGCACTGCGA

CCGTTACCACGTCCACGCGGTCGAGCAACTCCTCGATGCCGTACGCGTCG

GTGCCGAAGTCGGCGGCGACCTCGGCGGCGCGGTCCGCGTCCGCGTCGGC

GACGCCGACCAGTTCGACGCCGCGAAGCTCCGCGTAGACCCGCGCGTGGT

TGGTTCCCATCGACCCGACGCCGACGACGCCCGCGCGGACCGGGCGCTCT

CGGCCGCCGTCGGCGCGGAGTCCGGCGGGGTCTCGGCTCACCTCGCCACC

TCCGCGTAGTTGGCGACCGCAGTCCCGACGCGCTCCACGTCGGCCTCGCT

GAGTCCGGGGTGGACCGGCAACGAGAGAACCCGTGTCGCGGCTTCTTCCG

CGACCGGAGCCGACACCGCGAGGTGTTCGTAGGCGGGCTGCTTGTGGACG

GGCTTCGGGTAGTAGACGCCCGTGGCGATGCCCTTCGCGTCGAGGTACGA

CTTGAGGGCGTCGCGCTCGATACCGTCGCAGGCGACGGTGTACTGGTGGA

AGACGTGAGTCCGGCCCGGCGGCGTGTGAGGCGTCGTGACCGGCGAGTCT

TCGAGGTACTCCGTGAGCGCGGCGGCGTTGGCCTGTCTCGCGCGAGTGAA

CTCGGGGAGACGTTCGAGTTGGACCCGGCCGATGGCCGCGGCGATGCTCG

TCATGCGGAAGTTGTGGCCGACCTCGGCGTGTTCGTAGCCGGAGACGCGG

CCGTGGTCCACGAACCGGGCGGCGCGCTCGGCGACGTCGGCGCGGTTTGT

CGTAATCATGCCGCCCTCGCTCGTGGTCATGTTCTTCGTCGGGTAGAACG

AGAAGCACGCGGCGTCGCCGAAGGAGCCGACGCGTCGTCCCTCGACTGCC

GCGCCGTGAGCCTGCGCGGCGTCCTCGACGAGCAGGAAGTCGTACTCCGC

TGCGAGCTCCGCGAGCCGCGTCATGTCCGCCGGGAGGCCGTAGAGGTGGA

CTGCGATGACTGCGTCCACCCGCTCGCCGGCCCGGAGCCGCGCTTCGAGC

GCGTCGGGGTCGATGTTGTACGTTCGCGGGTCGATGTCGACGAAACCCAC

TTCAGCGCCCGCGAAGGCGACGGCGTTGGCCGTGGCGATGAACGTGAAGG

GCGTCGTCAGGACGCGGTCGCCGGGGCCGACGCCGAGCGCGTGGAGCGCG

GCGTGGAGCGCCGTCGTCCCGTTGGAGGTGGCGACGCCGTGGTCGGCTTC

GCAGTAGTCGGCGAACTCCCGTTCGAAGCCCCTGACCTCGGGGCCGTCGG

CTATCATGCCGCTTTCGAGGACGGCCGCGATTCGGTCCCGTTCCGCCGCG

CCGAGCTGGGGCGCGGCGATTGAGATGTGGTCTGCACTCATCGTCGTACC

CTGTTTTCTCCCCGCAGTTGTTCCGGCAGGTCGCGGTGGACGGCGGGGAC

GCCCACGGCGAGGGTGCGCGGCGGCACGTCGTTCACGACGAGCGCGCCGG

CGGCGACGAACGCGCCCTCGCCGACGGTCACGCCGGGGAGGAGCGTCGCG

TTCGCGCCGATTGAGGCGTGGTTCTCGATTCTCGGGCCGACCAGCTCCGC

GTCGGTGCGGACCGGATAGGGGTCGTTCGTCATCACCACGCCCGGACCGA

CGAACACGTCGTCGCCGATTCGGGTGTTGGTGGGGATGTAGACGTTCGTC

TGGATGCTCACGCGCGACCCGATGGTCGTGGTGCCGTCGACGACCGTCTC

GGTCCCGAGGAGGACCCCGTCACCGATGGTCGTGTGCTCGCGGACGAGGA

CGTTGTGGCCCGTGACGAAGTCGTCGCCGATGATCACGTCGTCGTAGACG

ATGGTGCCGGCGCGGATTCGCGCCCGGTCACCGATGACCGGATCGTCGCC

GTCGCCGTAGCCGATGGTGACGCCGTCGTCGACGTGGGATTCGACGCCCA

CCTCGGCGCTCATCGGCGTCCACCTCGCGTCGGTCGCGCTCCGTCGGCCG

TCTGTGTCGACTCGGCGGTCGGCCGAATCGACTGGAAAATCTGGAATCTC

ATATCATGTCGGCGGGGAGTCTGCCTGCCTTAGCGAAATTCAGTCTCGGT

GAGGGCTTTGTTATGGAGTGATTGCCGCCCGGTTTCCGGTCGCAGTCACT

TCGTAACGCCGCGGCGGATTCCGGTCACAGAGCGCTTGAGAAGCCCCACG

CCGGGCGCGCCGTCGCCGAGCCAGAGCCAGACGGCGCTCCCGAGCAACGC

GAGTTCGAACGCGACCAGAATCCACGCCTCGGGCCGGCGGAGGAACACGA

CGAACCGGCGCAGGTAGAACCACGTCTGGGCGAAGAAGCCGCCCATCGAC

GACTCGCTCCCGTGGGACAGCGGCCAGAGGAACGCCCGAATCACGTCGTA

GTTCCCGAAGAAAAAGCCGTTGAACAGCAGGTCCGCGGGGATGTGCGAGA

GATAGCCGAGTGCGAACGCGACGCCGACCGTCGCCGCCCCGTAGCGCCGC

GCGACGGCGACGACGACTATCGAGACCGGCACCGAGACGAGAATCGAGTG

CGCCATCGAGATACCGCTCGGGAGGACGCCGAACGACCACGCCAGCGGCT

TGTCGACGAGGTCGGGGAACTGCGTTCCGACGAGCACCGCCAGCGCGGCC

CAGCCGTCGAGTCGCCGGCCGGTCACCCGCGCCCACAGCGAGACGAGGAT

GTAGCCGACGGCGGCGTGTTCCCACGGGAACATCAGCGGTCGCTCCGGGT

CGTCGCGTTGGCGTCGGCGGTTCGTCGTCGCGGGGTGAGCGTCGGAATAG

GCGTCTGAAACATTTGGCTTGTGGGGCGCGTCGCGGTCGGTTTGGAGGGC

GGGGATGCCGTCCGCGTCGGGGGTGCGGACTGCCTGTCTGCGTCGACGCG

AACGGGCGAAACGGCGCGCGAGCGCCCGGTCAGGGAACCCGAGTGCTCGT

CCGGATTTTGTTATGGCGACCGTAACGGCCCCGGGTCGGTCGTCGCGGCG

GCGACCCGGCGTCTCTCCGACCGACCGCGGCGGGTAGACGCCGTATAACT

ACTCCCGCGGGGCGCGTGAAACTGCCAGAGAGTATGCGATACCTGTTCTT

CACCAACACGCCCGCGCACGTTCACCTCTACAAGTACGGCGTCAGAGAGC

TTCAGGCGATGGGTCACGACGTGCTCGTCCTCGGCCGCGACTACGGCTGT

ACCAAGGACTTGCTCGACTACTACGACCTCCCGCACGAGATTTACGGCTC

CTGCGGGACGACGAAGTACTCGCTTTTCAAGTCGCTCCCGTTCCACTACG

TCAACATCTTCCGGGCCGTGCGGCGGTTCAAGCCCGACCTCATCTTCGGA

ATGGGCGGCTACGCGGCCCACGCCGGGGCCGTCTCGCGGACGCCCGTGAT

TCTCCTCCTCGACTCCGAGCCGACCTCGCTGGACCACGTCGTCTCCCGAC

CGTTCGCCAAGGCGATTCTGACGCCACACACTTTCGGGAAGGACCTCGCG

GACAACCACTACCAGTTCCGCGGGTTCAAAGAGACGGCGTATCTCCACCC

CGACGTGTACGAGCCCTCGGTCGATATCCGGGCCGAACTCGGCCTCGACC

CCGACGAGACGTTCGTCGTCGTCCGGTTCAACGCGTTCGGCTCGCACCAC

GACGTTGGCCACGGCGGCTTCACGCCCGAGCGCCGCCGCGAACTCATCGA

GCTGCTGGGCGAGCGCGCGACCGTCTTCGTCCTCGACGAGAGCGAGGAGC

GCCGCGACGGCGACCTCGACACCCGCCCGTTCGACTTCCACCCCGCGCTC

GTCCACGACGTGCTCGCCGAGGCGAGCCTGCTCGTCGCCGACACCCAGAC

CATGGTGACCGAGGCGGCGCTCCTCGGGACGCCCGCCATCCGGTCGAACT

CCTTCGTCGGCGACGACGACATGGGCAACTTCGTCGAACTCGAACGGCAG

GGGCTCATCTCCAACCTCAAGTCGTTCGACGACGTGATGGCCCGCGCCGA

GGAACTCCTCACCGACGACGGCGCGAAGGCCCGCTGGGCCGACAAGCGCG

ACGCGTTCCTCGCGGACAAAGTCAACCTCACCGACGTCATCGTCCAGGTG

GCGCTGAACTACGGCTCCGTCGAGAGGGTCGACTCGCTGACGCGACACAC

CGTGCCGGCGTAGTCGATGCCGCTCGGCACTCCCCTCTCCCCCGAAGACG

GTCAATCGACCCATAACTAACCGTCGAACCCTCCAATTTTCGCGTATCGA

CCGGCGCGCGCTCGCGCCCGCCTCGACGCGTCCCGTGACTCGGAGTCGGA

TTCGCGATTCACCGACCACGACCCGCAGAACCAACCCATGAAGCCCAAGC

AAACACTCCTCATCGTCGGCCCGAAAGGCACCGGCGGCATCGACCGATAC

ATCACCGAACAGCGCCGCTACCTCGAAGACCGGATGGACGTGCGCGTCTA

CACGCGCTACACCGCGCCGAAGGGCGAGGGACTCGAACTCGCCGCGCGAA

TGCTCCTCTCGTCGCTCATTGCGGTCGCTCTGTTTCCCTTCCAGCGACGC

CCGGACATCGTCCACGTCCACTCGTCGCACACCTACTCGTTCTACCGCGC

GGCGTTCTTCGCCCTGTTTTCGAAGTACGTCTGGGGCGTCCCCGTCGTCT

TCCACATCCACGGCTCGTCGTTCGACGACTTCCTCGCCACCGACTCGCGG

GTCGTCCGCCGACTCCAGTCGCTCGTCTTCGACGCCGTCGACGACGTGGT

CGTCCTCTCGGAGTACTGGCGCGGCCTCGTCTCCCGACTCGCCGGCGAGG

AGAAGGTCCACGTCATGGCCAACGCCGTCGAACCCGCGGAGTACGACCCG

ACGTACCCGACCGACCCCGTCCACCTCGTCTTCGTCTCGAACCTCATCGA

CCGCAAGGGCATCCGCGAGTTCCTCGACGCCGTCGAGAGACTCGAATCGA

CGCCGGGGCTCGACTTCGAAGTGAGCATCGGCGGCCGCGGCCCCCACGCC

GACCGCGCCGAGGCGGTCGCCGCCGCCCACGACAACGTCTCGTACCTCGG

CTTCCTCTCCGAGGAAGACAAGCGCGACCTCCTCAGCCGCGGCACCGTGT

TCGTCCTCCCGACCTACGCCGAGGGGCTTCCCATCGCCATGCTCGAAGGC

ATGGCCGGCGGAAACGCCATCGTGACCACCCCCGTCGGCAGTATCCCCGA

GGTCATCACCGACGACCGCGGGATTCTCGTCGCACCCGGCGACGCCGAGG

AACTGGCGCGGGCGCTCGCCCGACTCGTCTCCGACCCCGCGGAAGCCGTC

CGCATGGGTCGGACGAACCGCTCGGCCATCGAAGAGCGCTACTCGTGGCA

GTCGAACGCGGACGAGCTGGTGGCGATGTACGCCGAGGCGGCGTAGCGCC

CGAGTCTGTTTCTTTCGGGTGTTTTCGTCCCGACGAGTTTTCGATTTAAT

CCCACACAGGTTCGCCTGAAACGCGGGTCGTCGTTGTAGCGGCGGTCGTA

GGTGCCGCTTCAACCCCACAAGGGTTCGTCTGAAACAGGCGGCGCAGACT

TCGAGGTTGCCGTCGTTGTCGCCGCTTCAAACCCACAAGGGTTCGTCTGA

AACCCGCACGGGGACGGACATATTCGGGTCGCCGGACGGCTTCAAACCCA

CAAGGGTTCGTCTGAAACCATCCTTCAAAGTCCTCTATTGCTTGTTCCCC

ACAGCTTCAAACCCACAAGGGTTCGTCTGAAACCTGTGAAGTTACCCGTC

TCGGGGTGGCGAACGTTGTCGCTTCAAACCCACAAGGGTTCGTCTGAAAC

GATCCGAGAGGCTTCCTCCCTCGGTCCGACTAGCAGCTTCAAACCCACAA

GGGTTCGTCTGAAACCGCTTCGATAGAATCGACAACTCCCACGGAGGTGC

TGCTTCAAACCCACAAGGGTTCGTCTGAAACCGCACTCGGGGCAGGCGTA

ATTACTCGGACGCCAGCGGCTTCAAACCCACAAGGGTTCGTCTGAAACCA

CAGACGTGTGATTCGATACGCGACACCACCGAGCTTCAAACCCACAAGGG

TTCGTCTGAAACACAGTTGTTCCGTCTCGGCTTCCATCGACTCCGCTCGC

TTCAAACCCACAAGGGTTCGTCTGAAACCAATATATTGCCTATGCCCGGC

AGAACGTCCACCCCGAGCTTCAAACCCACAAGGGTTCGTCTGAAACCCGT

CCGAATTCACGGGGGTAGACGGCTGTACATAGCGAACTCACATCAACGTT

TCCGTCGACCCTCAATTCCCCTGTACCCCCCGGGGGGTCGACGGAAATGA

TGTATCTGTGTTTCGAGAACTGGATCGGACGAACGCGCTCGTCCGACTCG

CTCACGGTTGACGCCGTTCGCTTGTCAAGGTGGTGCCTTAGTTCAGGTAA

ACCGACTCCCCGGTTCGTCCGCATCCCCGAACGTCGTGTGGTCGAACGCA

TGAGGGTTGTCGAATTCGAAGACGACAATCGAATCCGTCGGTTCGAGTTC

GGATTCGATTTCATTTTTCATCGCAGTAACTTGGCCTGCTGTGAGCTCAC

CGAAGAAGACCGAATACTGGAGATGTTCCAAACGTCGGCGGAGCAACTTA

CGGTAGATGCGAGTCCTCTTGGCGGGCACGTCGTAGACGACGATAACTTG

CATTTCACCACCACTTCTCCGTCGCGTGATACGGCTCGCCGGTCAGGAGA

TGTTTCTTCAGACTGTACACGTCCGTCTGAATGAGCGTCTTGAAGCTCAC

TTTTCGCTTCAGACGTGGATGTTGCACCGTCTGGTCAAGCGACCGCTCGA

ACTCTTCGAGTACTGTCAACCGTCCCTGTTCAGTAAGTAGACAGCCGGCA

AGCTCTGTTTCGAAGTCGTCGGTCGTAATCTGCTTTCGGTTCACGAGTCG

GAATACGAGCCGGTCCGCGAGAATCGGTTTAAAGATGTCCGCAATGTCGA

GTGAGAGTGTAAATCGTCGCTCGCCCGGCTCGTGAACGAACCCGACCGTG

GGGTCGAGTGCCGTTTTTCGAATCGCAGACACACAGCTCGTGTACACCAT

CCCGTTCAGAAACGAGATGAGTGCATTCGTTTCGTTACTCGGTGGGTTGT

ACTCTCGTTTCGCCAACCTAAACGGCGCTTCTAGAATCGAATCGAAGCAG

TCGTAATATCGTTTTCTCGCGTCTCCTTCGACCGCTCGAAGCTCGTCGAT

ACGCTGTGTGTCTGAAACTGCAGTTTTCAACTCTCGGAGCGTCTCAACCA

CGGAGTCGAAGTCTCCACGCCGACCGGAGTAATACTGGAGGTTTGCTCGC

ATGTTGTGGATACTCGCTTCGATGATTCGGTGACCGATACGGAGGCGTCG

CTCCGTATTATCGTATGCGCGAACTTGTTCAACGACAGTGTTCCCCGAGA

GCTGACTTCGCTTTGGAAGGTACGACCCTCGATAGTAGTCTTTCCAGCCG

AAGATGTGAACGGGTACTCCATGCTTGTTCAGGAGTCCGAGTGCGCGCGT

ATTGAACGAAATCTGTCCGTGGAGATAGAGCGCATCGATCGACTCTACTG

GGAGGTACTTCGTCTCCCCTTCGAGCGTATCGATACGTAGCGTTCCTTCC

TTCCGGGACAGTTCGCCGTCAGCGAAGATATGGTGATTTGCTTTTGTCAT

GGGTCACATCCAACAGAGGTCTTGGTACAGACAGGTGCCACAGTACGGTT

TTTTCTCCAGTTGTGGTGGGGAGTCCCTGCCCACCACGTCTAGTACTCCG

CGAACTGTCGATTCGACCTCTGCAGTCGTCGTCTCATCAAGTACGACTGA

TTCTCGTTTTCGCTCGGTTGGATACGCTAAGACGCCGTCTTTGTCGATAT

CGTGTATCTCTCGAAGGTACCAAAGGTAGAAGAGCAACTGCATCCTCGCG

GGCTTTTCCAAGGCTGAAGAGACCTTCACCTCCATCACGTCTCCCGAATC

CAAGATATCTAACTGAATCCGATTATCGATCATAAACGACCGCCGGGACG

TCCCATAGCTCGTCTCGTCGACGTGTGTCCCCCGTTGAATGTTCGTTGTC

TCGCGGTCGATATCGATGCCGTTTGCCATAAACCAGAGCTCTCGTTTGCA

GACGTGGTAGTACTGCACCATGAGGCCCGTAATCGGTACGTTTGGGGACC

TGCTCGGATCACGTTCGTCTTGGACGTATTCTTCGACGACGTCAGTTGAA

CTCATCTCGCACCCTGCTTAGCCAGTGAGTAAATCATTGACATGACTGAT

GTTATCACGTCAATTAATATTCGTTTCTCTGCTGTGGACCGGCCGAGGAA

GGCCATCGTTCTGCAGGGGCACTTCCCCACCCACGACGAGGAGGGCTTCG

TGACTCGATGTGGTGAGGAATTCTCCCCCACCAACAGTAATCGCTCAGAA

CGTTGTGAACCGCCCTGCGACTGTGTCTTCTTTTCCTCGGAGACCCCCCG

TCTGCAGATCGTACTCGAGGCTTTCGGTGCCAGTATAGCGGAACACTTGG

ACCCCGTCTTCGTTCCGTTCTTTCCCATCGACACGGCTCACGTCCGGGAG

TTGTTCGATGGAATTCAGCGGTAGACTCACCCGGATTCCCGCCGCCTGTT

CTAACTTCTTGTACCCTGTCGACCTGTCCGTTGGATCGTCCGACGTGAAC

AACTCGGTCAGCTCATTCAGCCGCTTTCGTTCGGCTTGCGTCGTTGCAAC

TAGTACGTCGACCGTTTGGTACCCTCCAATGAGCGATTCGCGTGCGAGCT

TTCCAGCCTTCGCGTCATCGATGAGCGTTCGAATCTTCGTCGATGAGAGC

GATTTTTCACGAAGTGCGTCGAAGTACTCGTTCACAGCGTGATGTGATAT

CTCTGCGTCTGGTACTTCCTCATCCTCGAACTGTGCCAGAATCGACGAAA

TGAGTTGGAGGTGAGTTTGGATTCCTGCATCACTCGAACCACGCTCGTAC

ACCCAGTGGGCGGGCGGTGATTTTGTTGGGTCTCCGGGGTTCTCTTCGTC

CGGGTCGGCAAGCGTCCAGACGACTACCTGCCCCCCGTTCCGCCCCCACT

CGTACGACCGATTGCACCGCCCTCCTGCTTGGACGATGCTGTCCAATGGC

GCGATGTCCCTGAATACCCGCTTGAAGCTGAGGTCGACGCCCGCTTCGAT

GGCTTGCGTCGATATCAGTACGAACGGAATCTCACTTCCAGAGAGCGTAT

CGGCGATCTCGATGAGGAGCCGCCGGTCGAACGGCCGGTATCTCGAATTG

AGCGTTGCAACGACGATATCGGTGCCAGCCGGAACTGATAGTTCCCCATC

AACCCCCCGAAGCCCCGACCTGCGAAGTATCGCGTCTACGATTGCTGAGA

CGTTGTTTTCTTGCTTGGTCGCATCGACGTCGTTTGCCCCCAGTACGGAG

TCGATCGCTTCTCCGAGATGTGTCACTCCATCATGGCCGCACAGCTCCTG

TGTAAGTGTCGCCGAGCTGTTGATTGTGTTGCAGATAGCAAGTGTCGAAC

CGCCTTCGCCAGCCGCGGTTGCCGCTACGACTCGCGCCGCCGCCGTCTCG

TGGCTCAGATACCGCTCGTCCGCCGAAAGGTGGAACGAGAGTGCAGACTC

GTCTATCCGGTAGCGAACTCGCTCTGCGTTTTCGAAGTACGTCTCTTTTC

GCGCCGGTTCCAGTCGGACGGGATAGTCTGGCCCGACTTCGCATCGGAGA

CACCCTGTCTTGTCGTGTTCTCTCCCCACGTCGAGGAGCGACGTGGTTTC

GACGTCTCGGAGGAGCGTTGGTTGGGTGGCGGTCATTGCGATCACACGCG

TCTGATACTCGGTGGTCAGCAGTTCTATGAGCCTCGTAATTCCGTCCCAC

CACTCCTTGGGGAGTGCCTGCGGCTCGTCGAGAATCACTAGTCCGTCGTC

AAGTGCCGAGAGCTTCAACCCCTGCCGATTCGACGGACCCACAAGACTTT

CGAAGAGCTGTACGAACGTCGTCAGAACCGTCCCGTCACGCCAGGCTTCT

CCGAGGAAACTGGCGTGTTCTTCTTGGTCTGTTGATGCGACGTCCTCGGA

CTCTCGTTCGTTGCGATAGACGACTGTCTCGCTCAGATAGTGGTGGACGG

TCAATGCGGACTTCGTCGGGTCGGCACCCCAGAGCTCGGGTCTCTCGAAA

ATCGAGCGCGTCTGTTCGATAATACTCGTATACGGCAGGGCGTACACGAT

TGGACGAGTCGGCCCGCCGTCTGATTCGAGTATCCCGCGCGCTTCGAATG

CGGCTGACAGTCCAGTAAACGTCTTCCCGAGACCCGTCGGGAGCGTGAGC

GTCGCAATTGGTGGTGTCTGGGCATCCCCTCCAAGCCACTCGTGGACCCC

GTCGATTGCTTGTCGCCGAGCACGCTCTCGCTCGTCATTCAATGCCCGTT

CGAGCTCGTCGTCCGGCGGCTCCGCCCGCAGGCCACTAATGTACTCTTCG

AGCGTCTCTCGGTCCAACGTCTCCACATCGAAGACGTGTGATTCGGGAAC

TGCCATCGCGTGGCTCTTGTCGGCGAGCGTAATCGCCGCCCAGTAGTGGA

GGGTCCGGTCGTACAGCTTTCGAGGCAGTTTGTCCGAAGTCGCCTTGGGC

CCCGTCAGCTCTCGGCGCGCGCTCACCTCGTGTAGCTCCCCGACCACCGT

TCCCGACTGCACCCACGTTCTGAACTCGTCCCACGTGCTGTCCGTCTCCC

CACTTCGCTGGAGTAACTCGGTCGCTTTCTGTGGCCACCGCTCGCTAATC

GCCTCGAGTTGAGCGGTGAGGACGGAGTTCGACGTCTCGAATGCGTCCGC

GAGTGATTCGGCAGTGTACTGTGCCGCATCCGGGAGTGCTTGGTGGTGTC

GAGCGACCGCTAACGTCGCCGCCAGCTTATCTCGCGCTGGTGCGTCACGT

TGGTCGAGTACGAACCACGTCGCGAGCGCGCCCAATCGTGCGTGATTCTT

CTCTTTTTCGGGCCCCTCGTACTCGTCTCTCACATACGCCTGAAACTGCG

GTGTCGCCTTCCCGAAGTCGTGGAGTGACGCCGCGACACAGAGAAATCGC

CGCTGTGTTTCCGGTCCCCGAAAGAGGCGCGTTGCGTACTCGCTTACGAC

CGTGTTGTGCGCCGTGAGCCGGAGTGACCCGTCGTCTGTGAGCTGTTCGT

CAGGGTACGTCCGCTCGCCGTCGTTCGCTTCGGGATGCGATATGAGTGGG

TACGTCATGCTGAAAATACGGAGGGTGTTCGGTTAGCGGAAGACGACGGT

CCGGTCGCCAACCTGAACCGGCGCAACGTCGGTTTGTTCGCCGATACGGA

CCGGATTCGAGGCTTGCTGTGCGTAGACGTAGTCATCGAATCGGGTCGTC

CGGCGACCTCCCGATTCGCGCACCATGACAGCCGGCGACCGCTCGACACC

GTAGGTTACCTTTCCCTGAGGGACCGCCTCAGAGAGAGATATCGGTACGA

CCGAGTCGATATCGAGCGTCTCGTCGATGTCTTGCACCTCGGGCTCGCGG

TCGACTTCGACGTCCTCGATAACCGCTAAGTACTCCGACTTACCCAAACT

CGGCGGGTAAATCGACGTGCCATTCACGAGGTGCTCTCGGAGGGAGTCGT

AGAACTCCTCGTTTTCGAGAGCGACGTCAATCCGATACGCTGGGTCCGCG

AGAACCTCGTACGAGTGGAGCTGTCGATCCCCCGTCGTATTCTGGTAGGT

GAGCTTGTACGTCCGCCAACTCCCCGCAGTTTCGGTGACGTCCTGTTTGG

GGTCGGTCCCCAGTCCAGTCGTCGGGATGTTCACCGTTCGGAGGTCACTC

AGCGGCGTGATCGCAATCGCCGAATTGTCGGCCCCGAAGACGTCGTAGTA

GGAGTCGCGGTCTGCACCAACTATCGCGGCAAGCATACCTGCGACGGTGG

TCCGCGGTGGGATTCGGTACGTCTGCTTGGTGACGCTTCGGCCAACGCGC

TTGAAATGACCCCACGTAGACCGGACGGTAAACGAGAGGCAGTCGATCGG

CACGCCGTTGGCGTCGATATGCGGTGACATTGTCGCCTCGTTAGGCGAGG

TCGCGTTCGTTGAGCACGTCTATCTCGTGGACGTCGTGTCCCGCCTCGGA

GAGATGCGACCAGAATTCGTCGGCACGGATCGTTTCGTCACCGATGTCGA

GTTCCAGACGGCCATCTCCAACCACGTGGAGCGTGTCGATCCGATCTGCC

ACGCGCTCTAGGGTCTCGACGAGTTCGGTAACGTCGACGACGACGTCCTT

CACCGACCGCAACGATTCGCTACTGTCGTCACCGAGTTCGAGGAGGTTCT

GGAGGCCTCCGACGTGGTAGTTCCCCTCCTCGTACTCGACGCGGACGTAG

AGCCGCGGCTCTTGGCCGAGCTTCGAGCGCGAGGTCGTCTGATTCTTGAG

CGCGCGCCAACAGAGCGTGTCGAGTCGTTCGACGTCTGCCTGCGACAGGT

TCGTCGACTCCGCACCGTTGTTGTCGACGAGCCCCCAAAAAGGGAAAATC

CCGTATTTGATGCGCTTGTCGTCGAGGTCGAACCCGCCCTGTCGATTCCC

TTCCCCAGTCGAGATGACGCTCGTTAGCGAGTCGTATTCCTCGTTCTCTT

CGACCTCGTTCAGCGACTTCGAAGGCAGGAACTGAACCGGTCCCTGGTAG

TTGTTCGGGAGCGAACTGTTGAGCGCATCACGGAACGCTTCGTCTTCGTC

TTTATCGCTCGCCTCGAAGCTGAGCGTCGACCCGAAGTACCGAACGTCAG

TCGCCGCATCGAGGAACGCTTCGGCAACGTCGCCGAGGTCTTCGAGGTCG

TCGGCGTCGTCGACGCCGCCCAAGACGTCTTTGATGAGTTTCGTCCGCGT

GTGTGACCGTCCTTCGGTCTTTTTGACGTAGATATCGAAGCCGTCGTCTT

GGAGCTGGTCTCGGAGATAGCGCTTGAGCCGAACGTCGGTGATGATCCCC

TGTCCGGTGTCAGGGTCCCGGCGCGGCCGGTTGTCCCCAATCGGGTTCCC

GTTCGGGTTGCAGTCTTGTGCGTCGTAAACGAACAGAAGTTCGGATCGGT

TGAGTGTCGTCATTGGTTTGGTTATGTGTTGGATTAGTTCGTGGTGCTCT

CAGCGGGTTCTTCGGTGTCTTGGTCGTCTTCATCCTCTTTTTCGTGGAGG

TCGAAGGCAACCGGCATAGACCGACGCCCATGCGCGTGGCCTAACACGTA

GCAGAACTGGAGCTCACGCTTCTCAATCGGCCACGCACTCGGCATGTCCT

CAGTCGTCTCGAGCAGTCGATCGACCGTCTCCGGGAACAGCATGTCCCGA

TCAGAGCGGTACTCCGAGTCGAGTGCGTAGACCTTCGCTTTCTCGAGTGC

CGAGGTGAGTGCGTTCTCGAGGCTGTTCTTCGTGAGTTGGTCGCCCTTTG

TCTGGGCGTCAAGGGGTCGACCAACGTTTCGTTCGGACTCTTGGTGCCAG

CTCACCTGCCCGACGAGAACGCCGGCGAGTGCGGCCGCTCTCCGTGCAGG

GGCTTCGAACAGGGGCCGGTCGAGGAACGATTCGAGTCTGTGTTCTCGGA

TCGCCGGGAGACTCGTGGTATCGAAGTCAGTTTCAGTTTCAGTTTCGGTT

GTCATGGTTGGTGGTTCGATTGGAACATCCAGTCCGTTCAGTAAGCCCGC

TCTCGAGAGGGTTTCGAGGTGGACCAACTGCTGAGCAACAATCTGGTGCG

GTGGGAGGTCACCACCCTCTGATTCGTCGTGGTATCGCCGAAGGTACTCG

TCGAACAACATCGACGCGTCGAGCGGGACACCGGCGATCAAGCGGTGGTC

GACGACCCGTCGGAAGTCGTCATCTTCGTCGTCACCGCGATACGCGAAGG

CCGAGTCGGTGAACTGGTGGCCCACGATTCGATAGAAGCCGAACTTCCGG

GATTCTTCGAAGTCTTCGGTCGCGAGTTCGAGCAACGACCAGTTGTCGTA

CGGTGCGAACCCACCCCGTTCGGGGTTGAGGGTCGGCCCGTGAACCGTCT

GGGCGAGCGCGTCCGCGAGCTCAGAAACCCAGTACACGGGTGCGGCGGGT

TCCTCTGCGATTACGTTCTTGTCGTCGCCAATGGGGAGCGTAATCGTGTA

GAACCGAAGCTCCGTCTTTGCGAGCTCCTGTAGCGTTTCATCGTCTGATT

CCCGGAGTTCGTACGTAACGCGGGCCAGTGGTGACCCACCGGAGTCGTCG

TAGTCGCTCTCGCGGTCGAGCGATTGAATCGCTCCGTAGAGCGACTGCGC

TTTGAGCGGCGTCAGTTCCCCAGCGAAGTACGGGAGCGCGTACGTCTCGA

CGCCGCCGCGGCGTAACACACACGTCTCGACGAGGTCCTGTCCCTTGCTG

AACAGCATGGCGGTGTCGGCACCCACCGGGTAGTTCCGCCAGGACTGATC

CTGTCGAAGCCCCGGCTGGGCGTCCGGATGTTTCACCGAGAACACGCCGA

TCGGGTTGTCCGGTGTCCCGACGACGCGTTCGACGCGGTCGGTGACGAGA

CCGACGCTCTCTCCTTCGGAGATGCCGTCTCCGGATTCGACGTTCTTGTC

GGCGGCGTTGGCGGTCGCGTACCGCTTCATCGCCTCCTCTAACACGTCCA

GCTCTGCGGGCCAAAACCAGCGTGGGCCGCTCTCCTCACCGTGCGACAGA

CGGCCGGCATCGAGCCGGAGGCGGACTGTGATGACGGTTGGAAGCGACTC

GTCTGGTGGGAGGAGCGCTTTGATAGCCTCTTCGAGCGCCTCAAGCGTGT

CTGACCCTTTCTCGAAGACCGCCGCGAGCTCCTCGACAATCCACCCGTCT

GGGTGCCCGTCTTCGCCCGTGACCGAACGGACACTATCTTGAGTCGTCCA

CGAACGAACGCGTCCGAGAATCGTGCTGGCCACGCCCTCGGCGTCGTTAC

CGTTTTTCGACCCGATTTGCGTGAGGCTGTATTTAGCTCCTCGTCCGGAG

GATTTGTGAGCATACCGGAGCTTGGAGACGTCTTCTACACGGAGTGTGTC

GACCTTCGGGTCGCTCACATTCGGGGTCTCCCCCGTCAGGTCGATGTCAA

TCGTGACGAGTCGACCGTCATCGACGAACGGTTCGAGTTTGCTGTCTGTC

CCGTAGAGTTCGCCACCACTCTCGGCGACGGCGAGCACGCCGTAGAGTGC

CATCACGTCTTCGAGGCTTGCCGGCGGGCGGCCGTGCCAGAATGCATTGA

GTGCATTTTCGAAGTCGTCGATATCTGGACCTGTCATCAGTCACTCGCCC

GTGGAAGCGTTTTGTCGGTGAGGTTCAGGAAGCCAAACCCGTGTTCGCGT

CGCTGTCCAATCCCCGAATCGAGCGCGAGATTCAGGTGGTATCGGTGTGT

CTCGTCTCGAACGCGGTAGCCGAAGCGCCACTTCGAAAGCAGAACGGTGC

GGTCAACCGCGGTCGCGGGCTGGAAGCGAACCGGATAGACGACGTCGTCT

TTGATCGGCTCGATGCGGTCGAAGAGCGGTTCGTCGACCTCCCCCGGGCC

GTCGTAGTAGTCGTCTCCATACAGCTCGTGTGTCTGTTGGAGCGACCGCC

GGATGGCGTCCTGTAACGGTTCCATGCCGTGCTTCGGACGCCAGAAGAGT

TCCGTCTCTGATTCGCCGGCGTCGATTTTCGACGTGTCTAAGTTGTACTC

CTCGGCCAATTGACGGCCAAGTGCACAGTGGACACCGGTTCCCGTCTCGA

GGACACCGGTCGATCCGGCCTCACCCACGTCCGGGGCGTGACCGGTGATG

TCGTCGATAGTGAACCGCATCTGCCCAATTTCGAACGCACGCTCGCGGCC

GAAATGAGCGATGAGCGTGTCTAACAGCCCACGCCGTGGCGACGCGATGC

GGAAGTATCGCCGGTCGCCCTCTTCTATCTGTCCCCAAGGGAAGATATTA

GAGTACGAAAACCCGACGCCGTGGTTCGAATCGTGGGTCTCCGAGAACGT

GTCGTCGTTCTCGAGGCCGCGCCAGATGCGTCCGCGTGCCTTATGGAAAT

AAGACCGGTCGTAGGCCGCATCAGCAACGGCATCGAGCGCTAATTCTATA

CGCACGCCTCATATCACCCCTCTCAAAGTTGTGTGATCGTGTGCCACGAA

AAACACTTCTCATTGTCAGTACTTGTAGCTAATCCAAAATATCACAATGT

CTATCATGAATAGAACGATGGCGCGAAACTTCTCGCGTACGACTAACTTC

TGGAGCGCGCGCGAGCGGTTGAGAACCGTCTGTGCTCACCCAGTTTGTCG

CTTCGGCTGATGGGTGTCATGAGGTTTCCCTTGTCAATACTTGCTTCAAT

CCGGGGCGGTTCTCCTGAAAATCACTTGGCAAGGGGCCTCATGTCCGTGC

CTCCATCTTGGTTGCCGACGTTTTTATTCTATTGTCAGTGAATTTGACAC

ACCCTCCGAGACAGTGCCGTCTAGACTTCAATCCCACAAGGGTTCGTCTG

AAACCATCGCCTTGAGCGCGATGATGTCGATCCCGCGAGCGACGCTTCAA

TCCCACAAGGGTTCGTCTGAAACACGAGCTTCGAGCGCGAAGTAATCGTC

GTCGAGCCGGCTTCAATCCCACAAGGGTTCGTCTGAAACGTGATACCGCT

CTCTGCGCCGTCGTCTGCGGCTTCGAGCTTCAATCCCACAAGGGTTCGTC

TGAAACAACGTCGAAGTGTCCGTTTCCGACCTCGTAGAAAAGCTTCAATC

CCACAAGGGTTCGTCTGAAACGGAGTTCGAGGACTTCATCGACGGCTTCG

CAGAGTGGCTTCAATCCCACAAGGGTTCGTCTGAAACCCTACCGGACGTG

ACGATTGACGCCGATTTCGCGGTGCTTCAATCCCACAAGGGTTCGTCTGA

AACCGATGTCGGGATGTCGAGCAGTCGGACGCGGAGAGTGCTTCAATCCC

ACAAGGGTTCGTCTGAAACGGGAGCGTTGCCGCCGAGACAGTTACATCGT

CGGTGCTTCAATCCCACAAGGGTTCGTCTGAAACCCGAAAAGCGGTCTTC

GAGCTCCTGCCGAGCCCGCCGCTTCAATCCCACAAGGGTTCGTCTGAAAC

AAACGAGCGAACCGAAAACTAACACGTGCGACGAGTGCTTCAATCCCACA

AGGGTTCGTCTGAAACAAGTAGGGGGTTTTAAGGGATATCGCATACACTC

CCGCTTCAATCCCACAAGGGTTCGTCTGAAACCTCATGCAGTAGGCGCAC

GCAGCGCAGACGTGTCCTGCTTCAATCCCACAAGGGTTCGTCTGAAACCC

GTGATGTTCTGCGCGGTTTCGTTCGGCCCGGTCGCGCTTCAATCCCACAA

GGGTTCGTCTGAAACCGGTTCCCGAATTGGACCCCGGCTTCGTCGAGGAC

GCTTCAATCCCACAAGGGTTCGTCTGAAACGGTTGCGTGTAACCGTCTTC

TTTGATGGACTTGTGCAGCTTCAATCCCACAAGGGTTCGTCTGAAACCAA

AGTGTTCCGGGAGGTCGCCGGTCGAGATGCCTGCGCTTCAATCCCACAAG

GGTTCGTCTGAAACCCGTCCGAATTCACGGGGGTAGACGGCTGTACATAG

CGAACTCACATCAACGTTTCCGTCGACCCTCAATTCCCCCGTACCCCCCG

AGGGTCGACGGAAACGAATCTCCTCTCCTCACGACCAGCGGTGCGAAAAA

TCAACCGAGAGTCCCACCGGCCTCAGTCGGCCGGTTGTGGTGCGTCTTCG

GCTTCCGCCACACTCGCGGTTCCGCGTTCGCCTTCGCGGTCGATGTATTT

GCGGACCCAGAGCTCGCCGGTGATGATGCCGGAGATGGCGCTCATGTGTT

CGGCTTCGCCCGTGCGCTCCTCGCGCTGGAGGCGGCGAATCTCGTCGGCG

TCGAAGTACGGGCGTTCGCACGCGGAGTCGAGCAGGTCGTCGATGCGCTC

GCGGAGCGCGCGGTTCGTCCGGTACCAGTCGCCGATAGTGGAGCGGCCGC

CGTAGGTGTGGACGTCGCCGACGAGGCGCTCGCGGATGCGGTCGACGGAG

GTGCCGACGACGAAGCCGGCGGCGTGTTGCCACATCGGGCGCGCCGGCGG

CACTTTCGTCCGTTCGTAGGGGATGGCCGCGAGTCGGCTGTCGAGGCGGC

GGACCAGTTCGACCTTCGGGTAGGCCGTTCCGGCGGGAATCTTCCCGTTG

GTGAGTGGAATCGAGCCGACGCGGTGCGACAGCGGCATCCGCGTCACGGC

CCGGAGAAACCCGGTGTCGCTGAACGGGACCCGGGTGCCGGCCTGGCTCT

CGGCGATGGGGTTGCTGGCGAAGTCGCACCGCGGGAAGTAGTTGCGGTAG

TAGCAGTCCATCGCGGTGCTGAAGAGGTCCGGCTGGTCGCTCTTGCGAAC

CTCCTCGCGGTAGGTCGTCATCGGGTCGAACTCCGTCCGTAGGAGCCGGC

GGGCGTCGTCCACGTCGATGCGGTGTTTCGCGCGGTAGAGCGCCGCCTCG

GGTGACTTGCTCCCCTCGACGGCGGCCCGGCCGATGCCGTCACCCATCAT

CCCGCCCTGTCCGCAGCCCTCGATGAGCACGTCGGGCATGTCCTCGATGT

TGAACACGTTCGCGAGGTTGACGAACGTGGAGAGACCGACCATCCCGCTG

GTCAGTTGGACCGATTTGTCGAGCGAGTCGACGAGGGCGTCGGACTCGAC

CGGCACGCGCCGGTTTTCGAGGCCGAGCACCGCAGAAACCTTCGCGGCGA

GTTCGAGATTGCTCCCGTTCGCGGGGTTCGAGTCGTAGGTGTAGGTCGTG

AGGTCGTGGTAGCGGCTGAGCTCCCCGGCCATCGAGCGACTGTCGAGGCC

GCCGGAGAGCCACAGGCCGATATCGCCGTCTATCGTGTCGGCCATGTCGG

CGATGACCTCGCGGTAGGCCTCGGTGAGGTCGTCGAGGTAGGAGTCGTCG

GGGCTCTGTTCGAACGTGTGGTGCCAGTAGGAGTCGAGCTCGGGCCCCGC

GTCGGCCCGGTAGTCGAGGACGGTCCCCGAGGGGAGGAACTTCACGCCCT

GCACGAGCGTCTTGTCGCCCCAGACGTGCCCAATCATGAGGAGGTCGCTG

ATGGCGCGTTCGTCGACCTGCGGGTCGTCGAGGAGGGTCGTCAGCGCGCC

GACTTCGGTCCCGAAGGCGAACGGCTCGCCCGCGACGTAGAAACACTGTC

GCGTCCCGAGTTTGTCGCTGGCGAGCACGACGCGCTCCGCGTCGCCGTCG

ACGGCGACGACGAGGAACGAGCCGTCGAGTTCGGGCAGCAGGGCGTGTGG

GTCCTCGAACAGTCGGGCGAACAGCCCGTCTACGTCGAGTTCGAGTTCGT

CGAGGTTCGTCACCGCGCCGTATATCGCTCCCACGCGCGGGCCGTCTTCC

CAGACGGTACAGCCCTCGGGGTCTCTCGCGCCGTGGTGGAGCACCGAGAC

GCCGAAGCGGCCGGTCGACCGCGCCGCCGACTCGTACCAGTCTTCGTGGT

GGAGTTCGGCCGCGCAGGCGTCGAGCGTCGCCGCGTCGAGCGCGCCGCCG

ACGAAGCCAGTCATCGACGGCCCTCGCGTTCGGTTCGCTCGGCGGTCTGC

GGCGTGACTGCTGTGAGTGACATCGTTGCCCGATATCCGCGTGCGTCGGG

TTTTGTTATCACGCACCTATACGGCCGCGTGGCGGTGTCGAACGCCGGCG

AACCGAACGGTAGCCCGGCTTCATCGGGCCTCTGTGCCGGCCGGACCCTC

GGCGTTCGCTCGTTCGACGCGCGACGCCCGAAGAAAAACACCGGGAAGAA

CGCCTATAACGCCGCTTGAGTCGATTACACGCTCAATAACAAACTACGCG

ACTCGCGTTCTCTCACGGGATGACGCAACGTGATTCGCGGCTCACGAGAA

CGCTCCTCCTCGTCGGATTTCTCGCGATAGCCGTCGGTATCCTCGTCGCT

CGCGCGTCGCCGGCGCGCGCGTACGAAGTTTCAATCTACCAGTCGACGCC

GGCGGCGTTCTGGGTCGGCGTGGCGGTGGCGGTGTTGACCGCGCTGGTCG

TCGTCGTTCGCGCCCCGAACAGTCGGTTCGCTGCCGGTGGGGTGGCGCTG

GCGGCGCTCGCGATAGCCGCGGTCTCCGCCCTCCCGCTCGTCCGAGCGTA

CCACTTCTACGGGTTCAACGACTCGCTGACGCACCTCGGCTGGGCGAAGT

CGCTCGTCTCCGAGACGCTGACGCCGCTGGATATCGTCTACCCGAGCGGC

CACGTCTCGGCGGGGATGGTCAGCGCCGCCCTCGGCGTCGAACTCACTAC

CGCGATGATGCTGGTGGTGTTTTTCGTCTCGGCCGTCTACCTCGTGTTCG

TCCCGCTTCTGGTCTCGCTCGTCGTCCCCGACCGCCGGGCGGTCCTCGTC

GCCGTGTTCTCGGCGCTGTTGTTCCTGCCGGTGAACCTCAACGGCTTCAA

GCTCGTGTTCTTCCCGTACTCGATGGCGTCGTTCCTCGGCTTGGTGCTTC

TGTACCTCTTCTTCAAACACCAGTTCTCCGGGACCGACGCCGTCGAGAGC

CGGTGGCGCGGCCACGTCACGCCCGTCTCCGTCCTGTTCGCGGTCGGGAC

GGTCGCGCTGGTGTCGTACCATCTGCAAGTCGCCGTCAACTATCTCGTGT

TGTTCGCCGTCGCCGCGGGCATCCAGCTCGTCCAGCGCTGGCGGACCCCG

GACGCCGTCGCTGAACAGCGTCCGGTGTACGGCCTGACGCTCGTCTTCGC

CGCGGCGTTCGTCTACTGGCTGACGCAGTACACGGTCGGCTTTTCGACCG

GCGGCCTGTTCGTCGAAGGGGTGCTCGGCATCCTCTCGGGAAGTTCCGAG

GCCGGGACGGCCATCTCCCAGCGCACCGGCTCCGTCACCGCCGTCGGCGG

CGGTATCTTCGACGTGTTCGTGAAGCTGTTCCTCGTGAACGCGGTGTACG

CCCTGATGACTCTCGGTGTCTTCCTCGGCGTCCTGTTCGGGCGGTTCTCG

CGGCTGTCGGCTCAGGGCCGGTCGGTCCTGCGGACGCTGACGTTCAGCCT

CGTCGTGTTGGTGCCGTACTTCTTCGCGCACCTCCTCGGCGTCATCTCGG

AGACGAACTTCTTCTTCCGACACGTCGGCTTCGCGATGATTCTCGCGACC

GTCGTCGGGAGCGTCGGGCTGTTCTACGCCTTCGAGTGGCTCTCGGGGAC

GGAGTACTGGTCGAAGTACGGGGCGGCGGTCAGGACGGGCGCGCCCGTCG

CGTCGGCGCTCGTCATCGGGCTCACGCTCATGGTGATGTTCCCGTCGCCG

TACATCTACCTGCCGTCGAACCACGTCCCCGCCCAGCAGATGGGGGCGTA

CGACACCGCCTTCGGGATGTCGGACGAGGACGTTCGGTACGTGGGCATCC

GCGGGAACCCCGACCGCTTCGTCGATGCGCTCCCCGACAGCCCCACGCCG

GCGCTCGGCTTCACGACGCCGGACGAGGCGCTCATCGACCTGCCGAGTTA

CTACGACGGCCCGCGGTATCTGATGGTCTCGAAGACCGACCACGACCGCG

AGGTAATCGCCTACCGCGAGCTCCGCTACTCCGAGGCGAACCTCACGGCG

GTCCGGGACCAACCGAACGTGAACCGCGTCATGTCGAACGGCGCGGCCGA

CCTCTACTACGTGTCCGGGTAGGGGCGTCGGGGCACGGGGTTCGATTTTC

ACGCCGACATTAGAACCGCTACCGCCGGTGCCGCCGCTGTTCCTTTCCGA

CCGTCGGCCTGCAGGCCGACGGTCGCTGACCCGCCGCACGGGTTCGGACA

CCACGGCGCTCGGCAGGTCGCTCCGCCTCGTCAAACAGCGCCTAATTCCC

GCGGCCGCGGGAGCCCCGGCCCCCGCGGAGTCCAGTAGCGAGTCAATAAT

TAATCGCCTCGTGAGCCGATGTGTAGCCGAGTGTTACCGTTCGTGAATAC

GCCGGGTCGTCGTCGAAGCGGCGGCCCGAGAGGTGCCTTCGATGAGAGAT

AGGATACGCTCGGTCGTCCGGACGATTCGCCGCCGGTTCGTCCCCGGCGA

CGACGACGGCGACCTCGCAGAGCGGACGGTGAAAAGCGGGATGTGGGTGT

CGGCGATGAACGTCCTCGACCGGGTGCTGAAGGTCGTGATGTTCATCGTC

CTCGCGCGACTGCTCGGCCCCGAGGCCATCGGGCTGATGGGCATCGCGCT

GCTCACCATCTCGGCGCTTCTGAGCCTGACGAACCTCGGCATCGACGCCG

CGCTCATCCAGCGTGTCGATGACGACGTGGACGACTACCTGAACACGACG

TTCACGCTCGAACTCCTCCGCGGCCTGCTGATGAGTTCGATTCTCTACCT

CGCCGCGCCGTCGCTCGCCTCGCTGTTCGGCGAACCCGCGGCGCGGGACC

TCATCCGCGCCATCGCGCTGGTCCCGATTTTCCTCGCGCTGCGGAACCCC

GCGATGGTCTACTTCAAGAAGGACCTCGCGTTTCACAAGGAGTTCGCCTA

CCGGGTCAGCGGCACGACCGCCTACGTCGTGGTCGCGCTCGGCTACGCCG

CGGTCAGCCCGACGGTCTGGGCGCTCATCTTCGGCTACCTCGCGGACGCG

GGCGTCCGGTCGGTCGCGACCTACTTCCTGCACCCCTACCGCCCGCGTCC

CGCGCTGAACCGCGCGTACGCCGCCGAGCTCATCGGCTACGGGAAGTGGG

TCACGGGGTCGTCCATCGTCGAGTTCCTCTACGGGCAGGGCGACGACGCG

GTCGTCGGCTGGCTCCTGACCGCGACCTCGCTCGGTTACTACCAGCTCGC

CTACCGCATCTCCAACGCGCCAGCGACCGAAATCGCCGTCGTCGTCTCCA

GCGTGATGTTCTCGACGTACTCGAAGCTCCAAGAGGACCAGCGCGCCCTC

CGCGAGGCGTTCTTCAGCACCTTCCGACTCACGGCGTTCGTCGCCCTGCC

GATGTCGGTCGGCATCTACCTCGTCGCGCCCGCCTTCGTCGGCGCGTTCC

TCGGTGCGGACTGGCTGCCGATGGTGCTCGCCATGCAGATTCTCGTCGCC

TACGGGCTCTTTCGGACGCTCTTCGCGACGTTCAACCCCGTCTGGCGCGC

CGTCGGCCGCCCCGACGTACAGACCAAACTCGGCTTCCTCAGGGTCGCCC

TGCTCGCCGTCGCCATCGTTCCCGCGACGAGCGCCTACGGCATCGAGGGG

ACCGCCCTCGCGGTCACGGGCATCCTCGCGTTCCCGATGGTCCCCCTCTA

CGCGCGCGAGATGAAACGCACCCTCGACACGACCTACCGGCGGTTCCTCC

GGGAGCTTTCGTACCCCGTCGCGGCCAGCGGCGCGATGGCGATGGCGGTC

CTCGCGGCGCAGAACCGCGTCGGGTCGCCGCTCGTCGAGTTCGCGCTCCT

CGTCGCCATCGGTGTCGTCGCCTACGTCGTCAGCGCCGCGTCGCTCATGA

CGCTCTTCGACTGGCGGGTCAAACAGAACCTCCGGGAACTCGTCTCCGTG

ATGTCGAAGTGAGGGAGGCGTTCGGCCGATTTGACGCCCCGGCGAGTGGT

CGATTCGACTGACTCTCATTGCTAGCTGAAGCTTCGACGTGAATCAAACC

TATCAGAACACAGTCAGAAAATGTGGGTGCGGTGAAACGAACGCCCTCGC

AAGGTTCGGTGGGAGCGTAATTACCAGTTCATAGATTGATTATCCGACGA

GTAGAGACGTTTCACGGGCCAAAGCGGACTCGACACGGGCTACCGATTCG

CCCCGTATTGTCCCGATTAAGCGGTTGTTTCACGGGCTCAAATCGCCGCG

ATATTAGTATTTAGTTGATGCTTATCTGGGAGAATATAAGGTGTGTCTTT

GATATACAACTGGTAACTAACTGGCCTCCATCCGAAGCAATCCCGCAAGT

AATCGTAGCCAATCAGATTATACCCTGCAATAATGATTATCACCTTAACG

AGGGCTTTCCTGAGATATGGTACGACTAACCGATGGAGACGACGACGAAT

CCGCCGCGGATTCGGACTCCATCCTCGAATCGCAACTCAACAGGCGGAAC

TACCTCAAACTCGGTGCGATGGCCGCATCGACTGCCCTCTCTGCCGGCGT

CGCGTCGGCTGCGTCTGGACCAGAGGAGCGCTTCGGTATCCAATGGGACC

GCGTCGTCAACGCGGTGGACGACCTTGGAATGGACCCCAACGGAAACGAA

CCGATAGACGACCAACTCGATTCCGCGTACGAGAGCGGGACGCTCATCGA

ATTCCCTCCGGGTGAGTATCTCGCCACCGAGACGCAGTACTGGAACGACG

ACGTCTCCCGCTTCGGGATGGTCGGCACCGGAAGCTCCCACAAGGACGTG

CAGTTCGTCTTCCCGTCGGGCAACAACGGCGAGAAGTACCGGTTCCTCGA

AATCACGAGCGGGGACCACCACGTCCTGAAGAACTTCTCCATCCAGCAGA

CCGACGACTCCACCACCACGGCGGACATCTGGATGATAAATGACGACGGC

GCGCTCATCGAGGACGTCGAGTGGCTCGGCCGCACCCCCACCGACAACGA

CGCTCGCCGTCAGCTCCTCGCGTACGACTGTTCCTCCGTCGACGGCGTCA

ACGTCGCCCGCCGCGTCTACATGCGCGAGGGAGCCGAGCTTCCGGGCTAC

CCCGACGGCGTCGCGGGCATCCGCGTGCAGGGCGGCTCTGTCGGCGAAAT

TCGCCTCGTGGACTGCCACATCGAACAGCGCGGGTCGTCGTCGTTCCGCG

CGACCCACACCCGCGGCGTCCTCCGCGTCGAGGGCGGCCTGTTCAAGAAC

AACGACAACACCAACATGCGCATCTCCGCGGGCGACCACCCCGAAAAGAC

CTCGTGGATCAAGGGCGCGACCGTCATCATGGACGCGGACAACCTCAACG

AGCACGCCCACGACGGCGACAGCCTCGACAGCCCCGAGGGGCTCCGCATC

GACTCCACCGGCAACGGGTACGCGGGCGTTCTCATCGAGGACTGCGACTT

CATCTTCAAGTCCTGCCCCACCTCGCGCGGCATCGTCTCGTCTCCGACGT

GGGCCGGCCACGGCGGCTTCACCCTGCGCAACTGCCGCATCCAGAACGAC

ACGTCGGTCCAGACGATTCACGCCGACTCCGTCGACACCGACACCGCGGA

CAAGCCGTGGGGCGTGACCCTCGAGAACGTCTCCATCACGGGGAGTACGC

GGTCCCAGCCCGCCGGCGCGGCGGTCTGCATCGACAACGACCGCAACGGC

TCGACCGTCCAGAACAGCTGTATCCACTTCCCGAACGGCGACGTGGACGG

CGTCCTCGTCAACGACGCCACGAACTGCGAGATTCTGGACTCGAACATCA

ACGTCTCCGGGCAGGCGACGGTGTTCAGCGGGGCCGACGTGGACACGAGC

AACATCACGAGCAGCGACTCCTGTCCGCTCCCGAGCGCCGACGGGAGCAC

CGGCGGCGACTCGTCGACGACGGACGACAGCACGACGGACGACTCCACGG

CCGAGCCCCTCCCGAACGAGATTCGACTCGTCGGCACGGGCACGACGACC

CAGTACGAGTTCACGGTGACGGACTCGCTCCAGGCGTCGGGCGACACCAT

CGAGGAGTGGGACGACATCGAAAGCGGCACCGCGACCGGCTGGATTACGA

CCGACGGCGTCGAGGACGCCTACACGTTCTCCGGCGACATCGACTCGTTT

TCGTTCCTCGAAGGCGAGTCCGAGATTCACGTCAACGGCGAGCAGGTGTC

CGAGAGTACCGTCACGGGCGCGACGACCGACGACTCCACCGATTCGAACA

CGGACGGGTCGACGGATTCGACCGACGACTCGACGACCGACTCGACCGAC

CTCTCGCACGAACTGCGCCTCGTCGGTACGGGCACGCCGACCCAGTACGA

GTTCACCGTCAGCGACGCGCTCGAAGCCTCGGGCGACACTATCGAGACGT

GGGACACCATCGACGGTACCTCCGCCAGCGGGTGGATTACGACCGCGGGC

GTCGAGGACACGTTCAACTTCGCTGGAAGCGTCACCGCCTTCGGCTTCGT

CGAGGGCGAGGCCGAGATTCACGTCGACGGCGAGCAGGTAACCGAGAGTA

CCGTCACGGACGCCGTGACGGACTCGGGCACTGACGGTTCGACCGACTCG

GGCACGGACGGGTCGACGGATTCGACCGACGACACCACCGACAGTCAGAA

CGAACTGCGCCTCGTCGGCACCGGTGCCGAGACGCAGTACGAGGTCACCG

TCAGCGGTACGCTCGAAGCCTCGGGCGACACCATCGAACAGTGGGACGAC

GTGTCCGAAAGCTCCGCGACGGGCTGGGTGACCACCGAGGGCGTCGAGGA

CGTGTACGCCTTCACGGGCACGATTACGTCGCTGTCGTTCCTCGAAGGCG

AGGCCGAGGTCTACGTCAACGGCACGCGGGTCGACCCCGCCGTGTTCTCC

CTGCCGAACACCCTCGTCGTCGAGGGCGACGGCACGGAGACGACGTACGA

CTTCATGGTCTCCGGCGACATCCTGAACGACCCGCTCGTCGGGCCGACGG

AGAGCGACGACAGCCTCACCAACGGCAAGGCGAAGGGGTCGGTCACGGAC

GGTATCGACGCCTTCCGGTTCTCGGGCGACATCACGAAGATGAACCTGGC

CGGCGACGCGGCGCTGACGTTCGAAGACAACGACGGCTGAGGCGACGCAG

GAAAACGACACGACGGCACAGCACGGACACAGAAACTACGCGCGGCGCGC

CGCTCGCGCGGTGACGCCGCAGACGACAGAGGCACCCACGTTCGCATTCC

GACGCGGTTTTTTCGATGCTCTACTCGACGACCGAGCGACCGCCAGCTTC

GCCGGGGCCGACGCGCCGCGGGCGAGCTCGCGTTACGCGTCGTCGCCCGC

GTCGTCCGCGCCGCCGGCGGTCGGTTCTTCGGTCACGGTGACCCAGACGT

ACGCCGAGCGGTACGCCGTCTCGGTGCTCGGCTCCGCCGGCGCGTCGCCC

TCGTACAGGAAGTAGGTGAGCCGGAGGTCCTCGCCGGTCAGCGCCGGGGC

GAAGTTGTGGCGGGTCCGCCACGTCTCGCCGTCTTCGACGGTCTCCGTCC

GCGACCCGAGGACTTGCCGCTCGACGACCGTCACCTCGCCGTTCGCTCGC

TCGACTCGCTGGAGCTCGGCCACGAGCGTGTAGCTCGTCGCCTCGCCCTC

GACGTTCTCGACGGAGACGAACAGGGAGGTCTGCTCGCCGACCGTCAGCG

CTTCGGGGTAGTCGGCGGCGACGAACTCGCCCCCGGCGTCCTGCGTGAGA

AGCGACAGGCTCGAATACGACTCGCCCGTCCCCGGCGCGACGAGCGCGTA

GCCCAGACTCGCGCCGGCCACGATGACCGCGAAAGCGAGCGCGACGTTCA

GAAACGAGTCCACCCGCGAGGGCTGGTCGAACGACCGAACCACGTCGGCG

TACCAGCGGCGAATCGGAACGCGGAACCGCTCTGCTTCGGGGAGTCGCTG

CCGCCGGGCTTCACCGACCGCGAGGCCGACGACGATGACCGCCGCCAGAG

CGAGGAGGACGGGCGCGGTGTCCAACCCGACGCCGGTCCAGAGGAGCGTC

AGCCCGACGACCGGCAGCAGCGCGACGCTCATTCCGAAGGCGAGCGCGAG

GCGCTCCGTGAGGCTGAGGTTGGGCTGCCACTCGAACCGACCGAACGACG

GTCCCTCGGTGGGCGCGTCCGACGACCCGGCGGGGAACAGCGCGAGCAAC

AGCGCGTATCCGGGGAGAAACAGCAGTACGGGGAGCCCGACGAGGGCTTG

GACGGTCGGAACGACGCCGAGCGCCGCGACGAGGCTACTCGCAACGACGA

TGACCAGCGCGAAGGGCACGTCCAGCGAGAGTTCGCCGAGCGCCCGGTTT

AACCGTCCTTTGCTGTGTGATGTGGACCCCATTACTGGATAGATGACGCG

ACTAGGCTGTTATTATTGAGCGCCTCACGCCCCCGATTTCGGCGGTACGA

GGTGACTAACCCCCGATTACCTCGCGGTCTGCGACACCGTCGGTCTCGCT

CTCGCTCTCGCTGTCTCCCCGTCGGTCGCTCTCGTCGTCGCCGCCGACGC

GAGACGACTGCGAGTCGCTCGGCTCGTCCCGCTCGAACGACGCGGACGGT

GCCGCCGCGATTCGAAGCGGTTCGTTCGCGCGGCTGTCGAGGAGCATCGC

GGCGCCCAGGGACGCAACCCCCCCGATGACCCCGGCGAGGGTCGCTCGCC

CCGCACCGTCGCGGTCCTTCGACGCGACGCCGCCGACCAGTCGGAGCACG

CCGGCGAGGACGCCCACGACGCCGAGCGCGTAGAGGACCGCCACCGGGTG

AACGCCGCCGTCGAGGTACCGAACCCGGAGCCGCCAGAGGAAGTCTCTGA

GCAGGAGGCACGACAGTCCGGGGACGAACGTCGAGTACCTGATGCCGCTT

TCCTCGTCGCCGTAGCGGGCGCGCATCGGCACGGTCGCGACGCGTTTTCC

CGCGACGTTCAGGTTCACGAGCAGGTCGTTCAGGAAGCCGTACTGGTCGT

ACAGCCGGTCGAACTCCAACTCCGAGAGCGCGTCCGCCGAGACGGCCGTG

TAGCCGTTCTGCGGGTCGCGGATGTGCCAGTAGCCGCTGGCGATGCGGGT

CAGCCCGGACAACAGGAGGTTGCCGAACAGCCGCCACGGGGGCATCCCCC

GGCGGCTGTCCCGAGCCGCGAGCCGGTCACCGACGGCGTAGTCGGCGTCC

CCGTCGACGACGGGGTCGATGATGGCGGGCATGATGTCCGGGTCCATCTG

GCCGTCGCCGTCCATCACGACGATAACGTCCATGCCGTCGGCGAGCGCGC

GCTCGTAGCCGGTCTTGACGGCCGCGCCGCGCCCGCGGTTGACCGCGTGT

CGAATCGGGACGACGCGCCGGTCGAGAGGCGCGCCGCCGTCGGCCGCGGC

GACGACGGGCGCGGCCTCCCGTTCGTTCGCGCGCGCGGCGTGCCGCCGAA

TCACCTCCCACGTCCCGTCGGTCGAGCGGTCGTCGACGACGTAGGCGCGG

TCGACGAACGCGGGGAGCGTGTCGATGACGCCGCCGACGAACCGCGCCTC

GTCGTACGCGGGGACGACGACGCCCACGGCGTTACCGCGATACATCGTCC

CTCGCGTGGGTCCGCTGTGGTGAACGCCGCGCGGCCGCCGGCCCACCGCG

TCGGGGGCGTCGTCCCCGCGGCGACGAATCGAGCTGGAGAGTGCGTAGGA

ACATCGATACTTCGACCCGCAGATTGGGCGGTTCGGGTCTTTGTTAGTCG

TCAGCAACACGTACGTAAACGCGAATTTCAGGCGTTTCAGACGGTTTGTG

CGGGTGTCTAACGTTCTTCGAAACGTGACTGACTACCTTCGAATCAAGTT

ATTCGAGACTGTCATGAAAATCGGTATGTCGCCCGCAGGGCCGGCGTGCC

GGGCGGGGTTCCGTTCCGAGCGAGTCGCCGCGTCAGACGGCGTCTCGGAC

CGACTCCCAGTAGCTCGACGCCGTGATCACGTCCAGCCCGAGTTCGTCCA

CGTAGTCCATCGTCTCGGCGAACGCCGCCGGGCTGACGGAGCGCCCCTTC

CCGGTCACGTCGTGGTACATGAGCACCGCGAGTTGGTCGTACTGGGCGGC

GAAATCGAGCGCCTGCTTCGCCTTCTTCACGTCGTCGCCGTCGGTCCGGC

CGACGATTTGCGGGTCGGATATCGCGCCGGTCGGCCCGCCGACCGTCACG

AAACCGAGGTAGTGGTAGTCGCTCGCGGCGTCGAGCGCCGACTCGTCGTA

CCGGCCGTACGGCCAGATGATGAAGTCGGCCCCGCGCTCGAACCCGTTTT

CGACGAGCCACGCCTTCGACTCCCTGATGCTCGCGCGCTGGTCGACCGGC

GAGAGGTCGCGGAACGAGTCCGACCGCTGGGGGTGGCTCACCATGTCCCA

ACCGGCGTCGACCATCTCGTCCATCCCGTCCCGGGGGATGCGGTGGCTCG

ACCCCACGGTCCACGGGATGACGCCGACGACGCCGGGAAAGCCGTGGGAC

TCCAGTATCGGGTACGCCGTGTCGTACTGGCTCCGGGTGTTGTCGTCGAA

CGTAATCATCACCTTACCCGCGTCGGCCCGGGGTTTGGCCCGGAGCGAGT

CGACGTAGAACCGCGCCGCCGTGCCGCCGCCGGTGTACAGTTGGATGCTT

ATCTCGGTGACGTTCGTCAGGTCCGGCGACCCGGTGACGCGCCGCGGCCC

GAGGTCGAGGCGCTGCCAGCCCGCCCGCCAGATGTGCCGGCCGAGAAGCA

GCGTGTTGTCGTAGTCGGGCGCGGCGAGGCGAACCGTGATGCCCTCGGTG

TCGGGCGTTTCGAGGTTGACCGCCAGCGAGAGGTCCGCCCCGGAGAGGTC

GATACCGCCGTCGAACGTCCGGTAAATCCACACCCGTTCGTCGGCCGGCG

TCGCTTCGAGCCGGAGCGACTGAGACCCGCAGTAGACGACGTCCTCCTCG

ACGGCGAACCGCCCCGCGAACGTCTCCCACCGGCGCTCGTCCTCGAAGTC

GTCGACGAGCCATCCGGCGTCTCTGAACCGCTCGCGGCTGTTGAACGCGA

CCGCAGGCGGGTCTCCCGGCGCGTCGACGCTCGGGTCGTCGTAGCTGAAG

CGTGGGGTCGGAACTGGCGGCTCGCTGGTCGCGGTCGCCTCGGGCGTTCC

GGCCGGTTCTCCACCCCTCGCGACACAGCCGCTGAGCACGGCGGCGCCCA

GTCCGAGATATGAACGCCGTGACAGATTCATACGGATAACTGTCGCCACG

AGTGGATAGTAATGCGACTCGTAATCTCGGTCGCACCGGACTGAACGCGT

CGAACACTGCGGCGACGCCGGTTCTGTCCCGGGTTTTCGTACGTCTCCTT

CACCGGATTCGCCGCCACCGACGAGCCTTGATTCCGCGCTCCTCGCGCCG

TCAGCCGCGGTTACACACCGTTTACCAATCACCTTCCCCGTCCAACTGAC

GGGAAACATGTGCAACCGATGTTGGTCCCTCTCTCTCCCGCTTTCATCTC

GCCCTTCGAGACGACTCGACGCCGCTGCACGGACGCCGGATAGTCGCCCG

GAACGCCGCGAGCGCACGGAGGTTCTCCGCCGGTGAAGCGCAGAACCTAT

CTGCGGACGCTCGGCGCGGTCGGCGTCGGGGCCGCGCTCGCCGGCTGTAA

CACGCCGGACGAGGAGACGCCGACCGAGACGCCGAGGCCGACCGAGGAGA

CGCCCACGCCGGAGCCGGAACTCACCGTCTTCGACGCGGTCGACGACCTC

GGGATGGACCCGACGGGGCAAGAGCCGGTCGACGACATCCTCGACGAGAC

GTACGGCGACGACACGGCCATCGAGTTCCCGCCGGGCGACTATCTCATCA

CCCGCGAGCACGACTGGGACCGCGGCGTCTCGAACTTCCGGCTCGTCGGC

CTCGGCGACTCGCACAAGGACGTGCAGTTCGTCTTCCCGCCGGCGGACCC

CGGCGAGCGGTTCCGGATGCTCCGCATCACCAGCGGGACGGACCACGTCC

TGAAGAACTTCTCCATCCAGCAGACCGACGACGACACCACGAGTGCCGAC

ATCTGGCTCGCCAACGACGACGGCGCGCTCATCGAGGACGTCGAGTGGCT

CGGCCGCACTCCCACCGACAGCCACGCGCGCGACCAACTACTGCTGTTCG

ACTGCACCTCGGTCGAGGGCGTCAACGTCATCCGGCGCGTCTACATGCGC

GAGGGCGCGGCGCTCCCCGGCTATCCGAACGGCGTCGCCGGGATTCGCAT

CACCGAGCGGTCGGTCGGTGAGGTCAGGATGATCGACTGCCACATCGAAC

AGCGCGGGTCGTCGTCGTTCCGCGCGACTCACACCCGCGGCGTCCTCCGC

GTCGAGGGCGGCCTGTTCAAGAACAACGACAACACCAACATGCGCATCTC

CGCGGGCGACCACCCCGAAAAGACCTCGTGGATTAAGGGCGCGACCGTCA

TCGTCGACGCCGACAACCTCAACGAACACGCTCGCGAGGGGGACCGACTC

GACAGCCCCGAGGGGCTCCGCATCGACTCCACCGGCCACGGCTACACGGG

CGTTCTCATCGAGGACTGCGACTTCGTCTACCGGTCGAGTCCGTCCTCGC

CGGGCATCATCACCGTTCCGACCTACGGGAGCCACGGCGGTTTCACGATG

CGTAACTGCCGCATCATCAACGACACCGGCGTCCAGACCATCTACGCGGG

GCCGGTCGACACCGATATCGCGCGGGAGCCGTGGGGCGTGAACCTCGAAA

ACGTCACCATCTCGGGGGCGTGCGAGAGTCAGCCCTACGGCTCTGCGGTC

GTCGTCGACGAGAACCGAAACGGCTCCCGCATCGTCGATAGCTGTATCTA

CCTGCCGAACGGCCGGGTCGGCGGCGTGCTCGTCAACCGCGCGTCCGGTT

GCGCTATCGAGCACTCCAGCATCAACGTCAGCGGCCCGCCGACGCGGACC

CGCGGCGTCGAACTCGCGCTCGACGACGTGACCTACACCGCGACCTGCGC

GTTCCGCGACGAGTGAGCGTCGGGCGCTGAGAAGGCACTGTCTGCGTCCT

CAGTTGTCGGCAGCCGGCCGTGGCTGGATACTGGCTATCGCATGTGGCCC

GGCCCGTCCCACACTGGTCAGCACAGTAGCAAACCTTACCCAGTAGTATG

TTCTACCGGCGAACATGGCCACGTCGGTGAAGATGGACGACGAGACGAAA

TCCCGTCTCGAACGGTTACAGGCCGAGATTCGGCTGAAAACCGGGACGCG

AGTGACGCAGCAGGAGGTCTTGGCGCGGCTCGTCGAGAACGCCGTCGAGT

CCAAGGCGGACCTCATCGACTCGTTCCGCGAGGAGCGCGTCCCGCTTTCG

GCATCCGAGCGCGAGCGGTTCCACGACGGGATGGTTTCGTCCGGCGTCAC

GACGACGGAAGAAGACATCGACGACGTGCTGTACGGATGAGCGTCTTCGT

CGATACCGGCGTGTTCTTCGCGCACCACGATACGGATGCCGACCGACACG

ACCAAGCCGTCAGCGCGTTCGACGACCTGTTCGACGGGGAGTTCGGACAG

CCGTACACGAACGACTACGTTCTCGACGAGACGGTGACGCTCACGCGCGC

CCGAACGGATTCGTTCGAGGCGGCGGACACCGTCGCCGGTCGCATTCTCG

GTGAGGAGCCGTTCCCGAACGTGTTCCGGATGATTCACGTCGAACCGGAC

GACGTCCGGGCGTCGCTGGAGACGCTCCGTCGATACGAGGATCACGACCT

CAGCTTCACCGATGCGACCATCGTCGCGCTGTGTGAGTCGCGTGGCATCG

ACGCCGTGTTGAGTTTCGACACGGATTTCGACGGACTCGTCGACCGCATC

GAACCGGGTCACCGGTCGGTTTAACAGCGCCACGGCGAGCGCCGACAGAG

CAACTCCGGCTCGGTTCCGCTGCAAACCGACAACTGTATCCAAACTAACA

TTTATCCGGACGCGCGACGCAGTTCGACCTATGGTTCGGCTCTGTCATCG

GTACGGTCCCGGACGGCTCCCGTACGCCGGACGAAACGACATCGGCGGCG

GGATAGCGATGGCTGCCACCGGGCTGTTGGCAATCACCCTCCGGTTCGGT

GCTGCGGGGCTGCTGACCCTCGTCGGCGCGATGAACGGGACGCTCGGGTT

CGTCTTCGGCCTCGGACTGCTGGCCGTCCCGGTAGCGATACCGACCTCGT

TTATCGTGGGCACGCTACTCTGGCGACGGTTGCGACCCGACGACGACCGC

CAACGCTACGGTGCGCTTTTCGGCGGGCTGACTGCACTCGGAAGTCTTGT

CACGGGCGGGTTCGGGCCTGCGTTCCTCGTCGGCGTCTCGAACGTCACCC

GCGGTGAGATGGTTCTACGGGAGGCCGTCGTCTTCACCGCGCTCCTGCTA

CCAGTGAGCGTGGTTTTCGCCGTTATCGCTGCCGGTTGGTTGGTCGTTCC

GCTCGGCGCGTTCGGCGGATGGTATCACGAGCGTGCGAAGGCGCGCTCCT

GACGAGGTCCGACCGCTAACCGCGCTCCGCGGCGTTCCACAGCGCCACGG

CGAGCGGCGACGCCACGACGCCCGGCCGACTCAGCGACCTCTGAAGTTGA

GTCCCGGCGTCAGCGTGATACCGGTCCTCGGCTCTTCCGATTCGTCGCCC

CCGCGGTCGTGGTCGGCGGTCGATTCCTCGTCGGCTTCGTCGCCGCCCCG

GTTGAACAGCGCGTGGTGCACGTAGCGGGCGAAGGGAACCGGGTCGTCGA

GGCGCGCGAGGTCGAATCTCGGTTCGCGGTAGATTGAGGCGGCGACCTCC

CAGACGGTCTTGACGAACGACGGGCGCTCGACGAGCGGCGAGTCGTCGCG

GCGGACGCTCATCACGTAGCCGAGTTCGCCCCAGAGGTAGTGGGTGCCGA

CGCCGAGTTCGTAGCCCGGTTCGATGCGGTGTTTCTGCCCGGTCGCCTGC

AACCAGTAGTAGTACGGGAAGTCGGCCCCGGCCCGGACGGTCGAGGGGAG

CGACTGCCACATCCGGGGGTTGATTTCGATGAGTTTGAACTCGCCCGTCT

CGGCGTCCTTGAGGTACTCGATGCACGCGAGGCCGTGCCACTCGATGTGT

CCGAGGAGCTTCCGCGCGACGGCTTCGAGTTCGGGGTCGTACACCGAGGT

CCGGTAGACGCCGCCGCCGCCGGTGTAGGAGTCCCCGCGAATCTGGAGGT

GCTGGAACGTCGCCAGCGGTTCGCCGTGGTCGTACAGCGCCGCGAACATG

TACTTGTCCTCGGTCGGGACGTAGTCCTGTACGATGGGGTCGTGTTTCAT

CTCGGCGTATATCTCGTCGGCGTCGGGTCGGTCGCCGGGCCGGACGTGGC

GGACGCTCTTGACGATGTCGTACTCGTGGGGGTCGTAGCCCTCGACGTAC

TCGCCGGCGACGACGTTGTACCGCGGCTTGATAATCGAGGGCTCGTCGAA

CGACTCGATTTCGCTGAACAGGCGGGTCCGCGGCATGGGGACGCCGGCCT

CGACGGCGGCCTCGTAGAGGAGCTTTCGGTCGTGGACCCGGCGGAGCGTC

TCGAAGTCGGGGACGACGAGGTCCACGTACTGCTCGAACTCGTCGCGGTA

CTTCGAGAAGACGTAGCCGTCTTCCGGCCGCGTCGGGATGACGGTCTTCA

CGTCCGGTCGGGCGGCGATACCGACGAGGGCGTCTTTGTAGGCCACGAGG

TCGTCTTTCGGCGTGGGGATGCGGACGAGTTCGTCGCAGAAGCGAGACGA

GGCCGCCGGCGTGTCGTCGTGTTCGGAGGCCATGACCGTGTGGACGCCCC

GCTCGCCGAGCGACCGGACGCAGGTGTAGTTGCCCGATGGAACGAGTACC

GATTCTCGGGCGTGCGCGCGTTTGGACATGCCTGAACCGTCGCGCGGTGA

CGGAATTAGTATGGGTCGGCTAACGGATTCGAGGTGGGAGACGGGAGCCG

ATTCGCCCCGGTCGACTCGGTGGTCGGAACGCGGGCGGCCGTCCGCGACG

GGGTCGGACCGCGCCGCTGCGCGAGCGCCGCACCTGCGACCGAACTGTGG

ATTCGGACGCGACGAACCGGATTCGATTCGTGAAAGTCGAGCAATCCGAC

GGTTTCGGGTCGGTTATCCACAGCATACCGGGGGACTGGCGAAACTGTCG

CCCCGGGACGAACCGACCGAGTTGGCGAGGCAGTCGCTCTCGACCGAAAG

ACAAAGAACAGTTAGCAGTTCGGCGCGGTGCCGCGCGAGGCGACGACTCA

GATGGCGCGGGCTGACCGCCGCCTCAGAGCACGTCGTCGTCGAGGCCGCC

GGGGTCGCCGGCGGTGTCGAAGAGGTTGTTCTGGGCGCTCTGGACGAAGT

CGGACTGGACGCCGTCCTCGTCGTCGACGGGGGCACCGTAGGCGTCCATT

CCCATCGAGAGGAGTTCCTCGACGGCCTGTTCTTCGCTGATGAACTCTTC

GTCGACGAGCTGTTCGAACTCGACGAGCAGTTCGTCCGGGAGATTGACTT

CTACGACGGGCATAGAGACGCGTTCGTGCTCGACATACTTGAGCGACCGG

GTGTGAGTAATCGCGGTCTGGGTCTGGTCGTGAGCTGTCGTCGGGAGCGA

CACTCCGCGTCGCGCGTCAGTCACAACCGCGTGTCTTATCAGTTCTCCGT

CGGACCCTCAACCCGACGAACGTCGGGGCGTCGATTGCCGACGCCCCGGA

CCGAGGGACCGACGTGACACGCGACACAGACCCGGCGACTGACGACGCCA

CCCCGGCCGAGAGCGACGCGCTCGGCTGGCTTCCGGAGCGCGTCGAACTG

CCGAGCGACGAGTGGGTCATCTACCCGCCCGGCGAGCCGCGGCCGGCGAA

CTTCGCGCTCCGCTTCGAGAAGGCCGTCCCCGCGGTGCTCGTCGGTATCG

ACTCCGGCGACTCGCCGGGCGTGCACGTCCTCCCGCATCCGGACGGCCTC

TCCGTGTCGGCCCGGTTCGGCGGCGTCGTCGACGACTCGGTCCGCGAGTT

CGAGCCGCCGTCGTCCGACGCCGAGCGCGAGGCGGTCGCCGCGTGGACGC

AGACGTGGGTCGAGCGCGTGGACGACCGGGCCGAGCGGGCGCGCATCCGG

ACGGCCTGCGCGCCGGTCCCGAACGCCGGCGACCGCGTCTCGAGGGCGCT

CTTCGACCACTTCGGGAGCGCGGACGCCGTCGCGGCCGCGGTGCTCGAAC

GGAACACCGCCGCGCTCACGCGGGCGTCGGGCGTCGCTGAGCAGACCGCG

ACCGACTTGGTCGTCCACTACGGCACCGACGCGGAGTGGGAGCACTACCG

CCGCCACCGCGACGCCGCCCCGTGACTCAAATCGCGATTTGACTCTCGCG

TAGGGTTATTCCCGTCCGCGACGACTACTGGGTATGTCCCTGACACGACG

CACCGTCCTCGGTTCGACCGCCCTCCTCTCGTTCGGCCTCCTCGCCGGCT

GTGTCGGCAACGGAGGGTCGGACGACGGCGCGACGACTGACGACGAACAG

ACGGACACGCCGACTGACGACCCCACGACCGACGAGCCGACGGACACGAC

AGACGACCTTCCGGACGGAAACGGCACCGACGACGGAACCGGCGGCACTC

GTCCGTCCGGCGGTCCCGGCATCTCACTGGTCAGCGTCGACGACGCGCCC

GACGTGCCGGTCGAGCACGAAGTCGAGATAACCGAGGACCTCGCGACCGA

CGAGCACCCGCCGCAGGTCCGAATCACGCTGACGAACACCGGCGACGAGA

CGGTCGAGGTCGGCGAGGGCAGAACGGTCTTCTTCCAGTACGTCTCGGAC

GCGACGAACGACCTCATCTTCCTCCCCGCGGACGGAGAGTACCCCGCCGA

GGCGGGCTGTTGGCGGCTCGAAGACCACATCGCCGTGACCGAGGAGTACC

GGATTCTCTCGCTCGAACCCGGCGAGTCGCGGTCGGAACTGGTGGACCTC

TACGGCGTCGCCCAGTCCGAGGGCGACGAAGCGAGCTGTCTCCCGGTCGG

CGAGTTTCGCTTCGAGGCCGACTTCGCTACTGGCGGGCTTTCTGGGTCCG

ACGGCGAGAGACAGGAGGCGACGTGGGGCTTTTCGCTCACGCTGGAGTGA

GACACACGGGTTCCGAATACGACCGCTGACCCGTCGCGGGCTTTATTCGG

CTCCCCGTGTGGCGGCCGAGTCCGAGACGACAGACCACTCCGAGGGGTCG

ATATCGTTCCCCTCGACGGCGCTGTGCTCTGGATACGCCGTCATCACGAG

ATACGTCGCCGTCCCGGAGAGATACTCGGTCAGCCGACCGAGGTTCTCGT

CGGCGATGCCGCCGAGGCGGTCCACGAGCATGACCGGCACGCGCTCGGCC

ACGTCGAACGCCTGGTACCCCGCGAGCGCCGTCGCCAGCCCGAGGAGTTC

GACCTCGCCCTCGCTGAGCGCGCCCAGCGACGCCTCGCGGCCGTCGCGGG

CGACGACGAGGTCGAACTCGCTCGTCAACCGCGCGCTCTCGAACCCCGTC

TCGAACAGCGGGACGATTTCGCGAATCGCGTCGTCGAACGCGTCTCGTGT

CTCTTTCTTTATCTCTTGTTTTCGCCGCCGGAGACGGGTTATCTCTTCGG

TCAGCGACTGTCGCTGTGAACGCAGTGATTCGAGCTTTTCGGCCTCCGAT

TCAGCCGATTCGAGTTCCGACTTCGCGTCGTCGAGACGCGCGTTCGCGTA

CTTCAGTTCGCTTTCGATATCGGTTATCTGTTCGGAATCTGATTCGATTT

GCTCGCGGAGGTCGTCGATACGCTCGGCGAGTTCGGCGTCGCGTTCGCGC

GCGTCGTCGAGGCGGCGTTCGAGGTCCGACCGGCGCTCGCGGAGCTCTCG

GAGTTCGGTCTGAAGTTCCCGTTCGCGTCGGCGTCGCTTCTCGCGCGACT

CGACGCGCTCGGTGAGTTCCTCGACGCGCTCTCGGCTGGCTTCGGCCGCC

GTGGTTCGGCGTTCGATACGCTCGGCGAGGTCGTCGAGCCGCTCGGTCAC

CGACGAGGCGTCCGTCTCGGCACCGCAGACCCAACACGCGAGTTCGTCGT

CGACGACGCCGTGGGTCACGTCGGTCAGCAGTTCGGTCCGGTCTTCGGCC

ACGACGCGGCGGTTCGCGTCGTAGACGCCCCGAAGAAGCTCGATATCGCG

TTCCTGCCGGTTGAGTTCCCGCTTCGCCGCGTCGAGTTCGCGCGCCAGTT

CCTCGTCGTCTTCCACGTCGAGTCCGTCGAGTTCCTCGTTGCGCGCGTCG

AGTTGTTCTCGGACCCGCTCGGCGGTCGATTCGAGTCGGTCGATGCGGCT

CTGGACGCGGGAGCGCTCCGCGCGGGCGTCGCTGAGCGCCTCGCGCGTCT

CGTCTCCGCCGGCGGCGTCGGCCGCCGCGCGCTTCGCCTCTAACTCCTCG

ACCTCGGCTTCGAGTTCCGACACCCGCCCGGCCAGCGTCGGCAGGCGGTC

GGCCGCGTCTTCGGCCTCCGAGAGCGCCTCGTCCACGCGGTCGCGCTCGC

GGCGCGCCTCGGCGATGTTCGCGTCGATATCCTCGAAATCGAGCGGTCGC

GTGAGGAGGGGTTCGAGGTCGTCTCCCTCGCGGACGGCCCGCCGAATCTC

GTTTTCCTCGCCGAGGAACGCGAACAGGTCGGCGCAGACGCGGTCGGCCT

CGGCGGACAGATACGGCTCGCCGGTCGTCCGGACGTTCCTTCCGTCTCGG

GTCAACTCGACCGCGTACTCCCCGTCGTCGGTCGAAAGCGAGACGCGCCC

CTCGGTCGCGCCTTCGGTCAGCGGCGACGCCGTCCCGAGGACGGTCGCTA

TCGCGGCGAGAAAGCTCGATTTCCCCTGCCAGTTGCTCGCGCGGACGGCG

TTCAACCCCGGTTCGAGCGTCGCGCTCCCGCGTCGAATCCCGGCGACGTG

CTCCACGTCGAGGTGCCAGCTCATGCCATTCCGGCGGCCGCGACGAGTTC

GAGCGCCGTCCCGGTGTACTCGCTGAGCGCGAGGCCGGCGCTGGAGAGCG

CGCCGATGGGGAGCACCGCCCGGAGCCGCCCGCCCGAGTCGGCCCCGCCG

TCGGGCGCGACGCCGGTCTCGCGGACGGCCTCCTCGGCCATCTCGTCGGC

ACCGGCGTGGTGCTCCTGACAGACGAACCCGCGGTCGAGCGCGTCTTCGA

GCGGGACCCGCGTCGGGCACTCCGCGCAGGACACCTTCACCTGCACGTCG

ACGCCGGCGTCCTCGGCCCCGGCGAGTCGCCCTTTCTTCCCCAGCGACGC

GAGCGCCGACTCGGCCTTCTCTGCGACCGTCTCGCGCGCGATGTCGACGC

TGTTTCGCTCCCAGTCCGAGGACGCCTCGACGGTCGGTTTCTCGCCGTCG

AGGCAGTCGTTGAGGTGGCGTCGCATCGTGCTCCACGACGCCATCGCGCC

CGTCACCTCGTCGGCGTCGATGTCGTCGGTGGCGAGGTCCGCGGCCACGT

CGCCGTAGTCGAGTTCGTCGTCACCGACGAGGGCCTCGTACTCCGCGTCG

AGGCGCACGTCGGTCACGTCGCGACCGTTCCGCTCGTAGACGCGCGCGAG

CAGTCGCTTGTTGAACCACGCCGTGAGCGACTTGTACCCCTCGCTTTCGC

CGCCGTCGGCCCCGGTCCACCGCGCCAGCAGATAGTCGTCGACGGAGTCG

TACCTGCCGGTCGGCGCGGCGAGTCCGTACCGTTCGATTGCCACGTCGAC

CTTGCACATTGTCTCGTTGCACGGAACTGGTGGCGCGACGGTAATAAGAC

TACGCTTGGCGGGTGACCTCGTCTCAGTCGCGGTCGCCGCCGTCGGCTCG

TCGCGTCTCCACCCCCCGCTCGTTTTCTTGTTGCTCGGCTTCCTGCTGTT

CTGCTTCCTGCTGTTCTGCTTCTCGTCGCTCTGCCTCCCGTCGCTCGGCT

TGCCGTCGCTCGTTGCGCTGTTTGGTCCGGAACTGTCGGAAGCGCTCTTC

CTGTTCGCGCTCGTCGAGGTATCGGCGGATATACCGTTTTTCCACCTCCA

ACTCCGGGATGAGCCGCTGGGTGAGGTAGTTGACGCGGAGCCGGAGCCGT

CGAATCTCCGCGAGGAGCCGCAGGAGGACCGCCCGCATCTCCGCCAGCCT

GACGACGTCCTCCACGACCGACTCGTAGGACCCGACGAGCTCGTCGTCGA

GCGCGCTCGTGCCGAGGACGCCGTACCCCCGCCCGTCGAGCGACCGCCGC

GCCGACGTGCTCAGAAAGAACGGGACGGTCAGCCCGACGAGCTTCGTCTC

GGAGACGATGAGCTCCGTGTGAGAGCTCCGCGCCTCCGAGAGCGCGCGAA

GCGCGATTTCGCCCTCCCGCTCTGCGCCCCAGACGTGGAGCTCAGCCGCG

TCGCGGAACGCCTCGTCGGTCTCGCGGCGCAGTTCCGTCCACCGGTCTAA

CAGGTCGAGAAGGACGAAAACGAGACTGTCGCGTCGTCGTTCGAGGATGC

GTTCTCCCTTCCGCGCGAGCGATAACTCGTCGGTCACCGAGAGTAACTCG

CGGCGCGTCGGGGCCGTCCGCCGGTGTCGCCGATTCGGCATTGTATACGC

TACGGTCGCCCCGACGGTTGATATACTTGTGTCACCGACACGTCCCCAGC

CGATTTGACAAGGACTAAGCCGTTGTCAATATCGACTCGAACACGAGCTA

TGTCTGGACACACGGCCGCCGAGTCGACGCGCCCGGCGGTCACGGCGACA

GCCGTGAGGAAGCAGTACGGCGACACCGTCGCGGTTCGGGACGTGTCGCT

CGACGTTCACGAGGGCGAGGTGTTCGGTCTCATCGGCCCGAACGGGGCCG

GGAAATCGACGCTCCTCGGCCTGCTGACTGGGACGCGAATCCCGACCGAG

GGTTCGGTTCGGGTGTTCGGCGAGTCGCCGACCGCGGTCGACCGCTCGCG

GCTGAGCGTGCTCCCGCAGGAGTTCTCACCCCACCGGCGACTCACGGGCC

GCGAACTCGTCTCCTACTACGCCGGGCTCTACCCCGACCCGCGACCGGTC

GACGAGGTGCTCGACCGAGTCGGCCTCGACGACGAGGACGCGGCGACGGC

CTACACCGACCTCTCGGGGGGTCAACAGCGCCGCGTCTGCGTCGGCGCGG

CGCTCGTCAACGACCCCGAACTCCTGTTTCTGGACGAGCCGACCACCGGT

ATCGACCCCGCTGGTCGCCGCGACGTGTGGGACCTCGTCGACTCGCTGGC

CGACGACGGGACCGCCGTCGTGCTCACGACGCACTACATGGCCGAAGCCG

AGCGCCTCGCCGACCGCGTCGGGCTTCTCACCGACGGCGAACTCGTCCGC

GTCGGTCCGCCCCGGGACCTCGTCGAGCGCTTCGGCGGCGAGACCCGACT

CGCGGTCCGGACCGAGTGCCCGCGCGCGCTCGACCTCGACGCGACGCCCC

TCGACGGCCGCCGGTACGGCGACTACGAGTACCGCTTTTTCGACGCCGGT

CCCGATGACCTGACCGCGATGCTCGACCATCTCGACGCGGCCGATATCGC

CTACGAGGAAGTGAGTCTCGTCCACCCGACGCTGGAGGACGTGTATCTCA

ACCTCGCCAGCGAGCGCGGCCACGACTGCATCGCGAGCGCCGGTCCGGCG

GGCGTCGCTGGCGACGCGGGGGTGACGCGATGAGCGCGACGGAGGCGGGC

GGAACGGACGAGACGTCCCCGGCGGCCGAGACGAAACAGACGGGCGACGG

CGGTCGGTCGCGCGCTCTCGCCGGGGCGACCCGCCGGGTCCGCGCGGAGG

CCGTCGCCGAGACGCGCGCGTTTCTCCGGCGGCGGACGGCCGTCTTCTTC

ACGTTCTTCTTTCCCGTCTTCATCGTCCTCATCTTCGGCGTCCTCGTCAA

GACCGGCGCGACCGGCGGTCTGTTCTCCCGCTCGGACACCTACTATCTGC

CCGCGTATCTCGGCGTCGTCGTCGTGTTGACGCCACTGTCGCGGGTCGGA

AGCACCGTCGCGCGAAACCGGGCGTCCCGCCGCTTCGAGAAGCTGGCGAC

GACGCCGCTTTCGCGTCTGGAGTGGCTCTCCGCGCACGCGGCCGTGAACG

TCGCGCTCATCGGCCTCGCGAGCGTCCTGCTCGTCGGCGTCCTCGCCGCC

GTCGGCGACCTCGGCGCGGCCCCCTCGCTCGGCGGCGACGCGCTGGTCGT

CGCCTTTCTCGTCGTCGGCATCGCCCTGTTCTGCGGGCTCGGCTCCCTCG

TCGGCTCGCTCGCGGACTCCGAAGACGGCGTCATCGCCGCCAGCAACGCC

GTCGGCATCCCGATGGTCCTCCTCGCGGACACGTTCGTCCCCGCGTCCAG

ACTCCCAGCGGCGCTCCGCCCGGTTATCGACGCGCTCCCGCTCACCTACT

TCGCGCGCGGCGTTCGCGCCGCGGTCGCCGGCGGCGAACTCCTGACCGCC

GTCTTCGACCTCGCTGTCCTCTGTGGCTTCACCGTCGCGCTGTTCGCGCT

CTCGGCGCGACTGCTTCCGTGGCGTGAGTGAGGCCGCTCACTCGCCGCCA

CTGTTCGGGACGTGGTAGTCGTCGCGGCGGCGCTTGAACCACCGCGTCAC

CCACGAGATGATGCCGAGGTCGCGCTTCGCCATGATGACCGTGCTCGCGG

CGTCGCGGCCGACCGTCTCCGGAATCGCGCCGAAGACGAGCTGTTGGAGC

AGCCCCTCTCTGGTCGCCCCGATGACCGTCAGGTCGAACGCCTCGGTCGC

GGCGACGATGGTCGCCGCCACGTCGTCGCCCTCGCGGACCGAGGTCGTCA

CCAACTCGTCGTCACCGAGCGCCGCGGCCGTCGAATTCACCCGGCGCTGG

GCGTCCTCGCGGTCGCGCTCGCCGGCGTCGGCCGCGACGACGTTCATGAC

GTGCACCGTGGCGTCGTTGGTCCGCGCGATGGCGCGGCACACCTCGGCGG

CGAGTTCGGCGTGCGGGCCACCCGCGGTCGACAGGAGAATCGAATCCACC

TCGCCGTCCGTCGGGCCGATGCGCTCGACGAGCACGTCGCAGGGGGCCTT

GGTCACGACCTCGTCGACGTTGCTCCCGAGGATGTAGTCGCGGCGGCGAC

CCCGGCCGCGCCAGCCCATCAACACCGCGTCGCTGTCGTGTTCCTCGATT

GTGTTGAGGATGGCCGTTCCGGCGTCGTGGCCCAGTCTGACGGTCCCGCC

GACGGGTACGCCGGCGTCGGTCGCCAGCTCCATCACGGCGGCGATGGTCT

CGCGCTTCTCGTCGACGAACCGCCGCCCCTCCGAAATCGGCGTCTGCGAC

GGCACTTCGACGGCGCTCATCACGAGAATCTCGGCGTCGCGGTCGCGCGC

CACGTCGATGGCGGTCCGCATCAACTGCGCCGCGTTCTCCGGGTTGGCAA

TCGGGACGAGCACCTGCGACTCGCGCCGCTCGGGGGCGCGCTCGGTCACG

ACGGTCGGGGTCTCTGCTTCGAGCTTTTCGGCCACCTTGCCCTCGGAGTA

GCCGTAGTACACGACGAGTCCGAGCGCCATCCAGACGGCCGTGGTCACGA

GCGCGACGAAGCCCTCGGAGCCCGCGCCGAGTCCCGGCGTCAGCCCGAGT

TCGAGGAGCAAAAACGGCGTCAGGATGAACTGGAGAACGATGCCGACGAG

CGGGGGCCACGGCATGTACGGTATCTCGTACGTCCGCGGGAGGTCGGGGT

GGGTCGCCCGCATCTTGACGACGGTCCAGTTCACCTGGATGAACAGGAAG

ATGAACATGATGTCGGCGGCGGCCGCGACGCTCTCGATGGGGAGCAACAC

GGCCATGAGCCCGATGAGGACCGCCGAGAGACCGATTGCCCAGTGGGGCG

TCCGCTTGTCGTCGTGGATGCGCCCGAAGAAGCCCGGAAGGACGCGGTCG

CGGCCCATGGCGAACGACACCCGCGACGAGGAGTAGATGGTCGCGTTGAG

CGCGCTCATCGTCGCCGCGAGCCCGGCGAACAGGAGGAGCGGGACGCCGT

ACGGGACGAACTGTCCGGCGGCCTCGATGATGCCGAGTTCACCGAGGTTC

CCGAGGACCTGCCACGTCGGCGCGGCGGTCGTGAGCCCCGCGCGCCCCGC

GAGTTCGGCGGTCACGTCGATGCCGCCGATGGCCGCGAAGGCGACGAGGA

CGTAGATGGGGACCACGATTGCCATCGAGTAGAACACCGCCTTCGGGACG

TTGGTGCCGGGGTCGACGACCTCCTCGCCCGACTGGACGATTATCTCGTA

GCCCTCGAAGGCGATGTACGTGAAGCCCATCGCGCCGATGACGCCGACGA

GGCCGTTGGGCGCGAACGACGGACTGTCGAAGAACGTCGCCGTCCAGTTC

GGCTCGCGCACCGTCGCGAGGATGCCGAAGGCCACGAAGACACCGAGGAT

GAGTATCTTGATGGTCGTGACGACGACGCCCGCCTTCCCCGTCTCCTCGG

CCCCGCGGTAGTTGATGTAGGCGACCGCGAGCACCATCACGACCGCGAGC

AGTTTCTCGACGAGCAGGCGGTCGACGAGTCCGAACAGGACGAACCCGTC

CGGCAGGCCGGCGAACACCACGAAGAACTCGGTGAGGAAGACGCCGAACG

TCACGGCGTAGAGCGCGCACGCGACGGCGTGGGCGAACCAGCTCATCCAC

CCGGCGTAGAAGCCGTTGGGGTCGGCCAGCGCCTCTTTCACCCAGAGGTA

GCCGCCGCCGGCCTCGGGGAACGCCGCCCCGAGTTCGGCGTAGGAGACGG

CGGTGAACATCGCCACCAGCCCGTTGAGTACGAACGCCACCGTGAGCGCC

GGGCCGGCCAGCCCCGCCGCGAATCCGGTCAGCGCGAAGACGCCCGCGCC

AATCATCGCGCCGACGCCGATGAACGTGATGTCGGCGAGACTCATGTCGC

GCGAGAGCTCCGTTTCCACCGACTCCTCAGGGGGAGCGTCGCCGCTCCCC

GCGGTCGATTGCGTGTCGGCCATCAGGTGAACCCTCGAAGGCCGGATAGA

AAAAGCCCGATGGGCGACGGGCCGCGTTCGAGCCGCCGTCGCCGGGTTCT

CACCCGCAGAATGCGGGTATTTTTCGACCGGGTGCGTTCCGGCGCGCTAC

CCGCCGAACATCGTCCGCATCATCGGGTGCATCTCCATGAGCTGTTCCTC

CGCGATTTCCTCGTACAGCTTGTACGTGATGGAGACCGTGAGGAGGAGCG

AGGTCCCCGAGACGCCGCCGATGGTGCCGAGCATGTTCGCCATGACGGCG

AGCAGGCCGACGAGGACGCCGCCGACGACCGTCACCTGCGGGATGTACCG

CTCCATGACCCGCTCTATCACCTGCGGGTTGCGGCGGAAGCCGGGAATCT

GCATCCCGGAGTTCTGAATCTGCCGCGCGGTCGCCTCCGGGCCCATGTCG

GCGGTCTCGACCCAGAAGATGGAGAAGATAGCCCCGCCGACGAGCATCAG

CGTCAGGTCGATGGCGACGCGGACGAGAATCTGCCACGGCTCCTGGGACG

CCGACCCGAGCCACCACATCCACTGGCTGGGCGCGTGAATCGGGTTCAGG

TAGTAGAACAGGCCGCCGGTGACCTGCCCGCTCGTGTACTGACCGAGCCA

CGCGGGCATGCCGGCCCACTGGCTGTTGAGGATGCGCCCGACGAACTGGA

CGTTCGCTTGCAGAGCGCGGACGAAAATCATCGGCAGGACGCTCGCGTAG

ATGAGCTTCACCGGGAAGCGACCGCGAGCGCCCTTGACGCGGGCGTGCGA

GAGCGGAATTTCGACGCGGACGCTCTCGGCGTAGACGACGATACCGAAGA

TGAGCAGCGTCGTGACGAGCGGGACGACGCCCCCCACGCCGAACAGGAGC

GCCTGTACCCCGCTCGCCGTCGCGAGCGACGGGACCGTCTCGGTTCCGGC

GAGGACGTGGGCCCACTCGGAGACGATGCCGGTCGTCGACGCGCCGATGG

CGGGGTCGGCAAAGAGGCCGCCGACGAGACGCTGGCTGACGCCGGCGATG

ATGAACAGGCCGACGCCGGAGCCGACGCCCCACTTGCTGACGACCTCGTC

CATGAACAACACGAGCGCGCCGCCGGCGAATATCTGCGCGAAGATGAGCC

ACTTCACGCCGACCGCGCCGACGCCGAGAGCGCTCGCGACCGCGGGGTCG

GCCGGGAGGTAGCCCCCGGCGAAGACGATGGGGAGGCCTTGGAGGGCGAT

CATCCCGAAGACGAGCAGTTTCTGGAGGCCCTGATAGAGGATTTGGTCGC

CCGGGTCGTCCGTGTCGAGGCCGAGCAAGTCCCCGCCTTGCAGCAGTTGG

AGGACGATGCTCGCGGTGACGATGGGACCGATACCCAACTGGAGGACGGT

TCCCTGTCCCCCGCCGAGGATGGAGCGGAACTGTCCGAAGATGTCCTGGC

TCCCGGTCCCGACGCCGAACAGCGTGACGTTCGTCAGGAAGAAGTACAGG

ACGAGAATCCCGGCGGTCCAGCCGAGCTTTCGGCGGAACGGGACGTGACT

CTCCGGGCGGGCCACGGCGGGCATCCGACTCAGCACTGGCGCTGCGGTGT

CTTTCCAACTCATGGTCGATTGCGAGTGGCTGTTGGCGATTGCATGGTCT

GTCGCGCCGACCGACGTCGTCACTACGCGAGCGGCGTCAGAACGGGGGGC

CGCACCGGACGCCGTCGCTGTGCTCGCGAATCGGCGGTTCAGTCAGTCGT

GGCCAGCCGTCGTGTATAACTGTATCGATGCGGTGACTATCCGTCCGCCG

CCGAGGAGGTCGGCGGAGACGCCGCGGGACCGGCCGCCGAAATCGCCTCG

TCGAACGACTCGCACGGTTCGGCGTCGACGACGCGGAGCGGACGCCGCCG

CCCCGCACGGTCGAACGCGGCCACGCAGTCGTCGTCCCACGGCGGGACCG

CGACGAGGACGACCTCGCGGAGCGTGTCGCGCTTCGTCGCCGCCAGCGGT

CCCCACGGGTGCGAGACGAACTGCCCCGGCGTCCGCCCCGGCGCGGTCGA

GAGGTCGACGCCGAAGACGCCCCGGACCGCCGCGCCGGCGCTCGGGTGGA

CGAAATGGCTGAACACCGGTCGGGTCGCGTCGAGCGACTCCGCCCCGTCT

AACTCGCCCGCGGCGGTCGTGCCGAGCGACAGCGATTCGCGGCTCGGTTC

GGCCCGCGCGGCCCGGCCCAACAGCGCGTCGAGCAGCCCGCGCGTGACGT

ACACCGCCTGCCGCGTCGGGTCGCGTTCCCGGCCGACACGTCGTTGGGTG

CGCCGCGGCCCGACGAGGCGGGTGGCGACGGCGACCCACCAGAGGCCGAC

GCCCAGCACCTCGGCGTCGCTCGCGGCTCGGAGCCCCCACTGGAGGTGCG

GAACTCCGAGTCGAAGCGTCGTGACCTGCACGGCGAGTCCGGCGAGGACG

AAGGCGGCCCCGACTACGGTGACGAGACGAGTGACACGGGCGACGGAAGC

GCGGTTCATCGGTGTGCTGTGGTCGGGCAGCCGCGGTGTCGTTTTCGGTT

TGGCAATCACTAAGTGGTTGTCGGCTGTTTCGAAATATTAGTCCTTGAAA

AAGACTATAAGCGTCCACGCCAAACCACCGATTGCCGTGTTTCACGGCCA

CGAAGCGGCTAACTGCTATCCGTCGTCTTCGATCTGACAGCCCCTTCGGT

GGTTTAGCCTGCCCGGTGTCGCCACCGTTGACCGCTTCGAAACGCGATAC

CCCATCCGGCGCACTCCCCCTTGTTTCGGCATCGCGTCGGCGGGTGCGCG

CTCGACACCCATGACGAGTACACAGCCACGATTGCCGGTCGAACGCTCGC

TCGCTCGAACGACGCTCGGCGTCGACCTCGCGTCGGCGGCGGCGCTCGCC

GTCGCGGCCGTCGTGTTCCGCGGCCCCCCGGCGGAGGTCGGCGACGACGT

CGTCGTCAGCGTCCTCCTGTTGGGCGTCGCCTGCACCGGCCTGTTACAGC

TCGCGGCGCGTGGCGGCCTCTCCTACGCCACGAAGTCCGCGGGTACCCTC

GTCGGCCTGTGGCTCGTCGGCGTGTCGTTCGACCTCGGTGCGGGTCACCT

CCTGATGTGGGTCGGCGTCGTCGCGGGCGGCTGTGTCGTCGCGACGCACG

CCGCGCTCGTGCGCGCCGCCCTCTGACCCGCCTCGCGGCCGCTCGCCGGT

TCGGCGTTCGAAACGAAACTGACAAACGCCCCATCGAGCAAGAGTGACTC

GGATGCCCCAAAACCCGAGTGACGAGTCTACCCTGCGCGAGCGGATTCGT

AGGGCGATACGGTCGGCGGAGGTCGAACCGCGGTACGAAGATATCGCGAT

GAACGTGCTGGCGGTCGCCATCGGCGTCGTCGCCGGGCTCGGGGCCGTCG

TCTTCCGCGTCGGTATCTGGGTCGTCCAAGAACTGCTCTACGGCACCTCG

CTCAACCCGGGCAACGTCTCGTTCGCGCTCGTGCCCGTGACCAACGCCTT

CGACCTCCTGTCCCCGCTCGGGCCGCTCCGCTACGCGGCCATCCCCGCGC

TGGGCGGCCTCCTCGTCGGCGTCGTCGTCCTGCTCACGACCGACGCGGTC

AAGGGCCACGGCGTGCCGAGCGTCCTCGACGCGATGCTGAACCGCGGCGG

CCGCATCAGCCCGAAAATCGCCCTCTACAAGACGCTCGCATCGAGCGTTG

CCATCGGGAGCGGGGCGTCGCTCGGCCGCGAGGGGCCCATCATCCAAATC

GGGAGCGCCGCCGGGTCCTTTTTCGGCCGGTTCGTTCGCTCGCGGCACAC

GCGGACGCTCGTCGCGGCCGGGGCGGCCGCGGGCATCGCGGGCACGTTCA

ACACGCCCATCGGGGGCATCATGTTCGCGCTCGAAATCCTCCTCGCGGAG

TACTACCTCGGCCACGTCATCACGGTGGTCCTCGCCGCGCTGACGGCGAC

GGCGGTCGCCCGCCCGATTCTGGAGTTCTCGCCGACGCCCGGCATCCGCG

AGTTCCTCGTGCCGGCGTCCTACCAGTTGGTGACGCCGGCGGTCGAACTC

CCCATCTACCTGCTTTTGGGGGCCGTCGTCGGCCTCGTCGGCGCGGGCCT

CGTGAAACTGCTCTACGGCGTCGAGCACTTCTTCGAGCGCCTCGACGCGC

CGGACTATCTCAAGCCCGCGCTGGGCGGACTCCTGCTCGGCGTGAGCGTC

CTTGTCGGGACCGTCGTCCTCGGCGTCGCGCCGGGGGAGGCCGCCGGACT

CCTCCTCGGGGTCGGCTACGGCGTCGTCCACCGGAGCATCGACGGCGGCT

TCCTCTTCGGCGCGCTCGTCGCGCTGGCGGTGCTCAAGGCGGTCGGCTTC

TCGATGTCGGTCGGGAGCGGGAGTTCCGGCGGTGTGTTCTCGCCGTCGCT

GTTCGTGGGTGCCACCGTCGGCGGCGCGTTCGGCCTCGCGGTCCACGCGC

TCGTCCCCGGGACGGCCCCCTCGGGCGCGTACGCGCTGGTCGGGATGGGC

GGCATCTTCGCCGCGACTGCCGGCGCGCCGCTCACCGCCATCATCATCAT

CTTCGAACTCACCGGCCAGTACACCATCATCCTCCCGCTTTTGATGGTCA

GCGTCGTCGGGAGCGAAATCGCGAGCTACCTCCTCAACGGCAGTACCATC

TACACCCAGAAGCTCCGAGACCTCGGCTACACGGTCCAAGAGCGCCGCAT

CGGAAGTATCGAGGACGTGCACGTCGCCGACGTGATGACGACCGCCGTCG

ACACCGTCAAAGTCGGAACGCCGCTTTCGGAGGCCGTCAATCGGCTCCGC

GACACCGACCACGGCGGGCTTCCCATCGTCGAAGCGGACGAGTCGCTCGC

CGGTATCGTCGTCCAGAGCGACGCTCAGGGGTATCTGACCGAGAACATCG

AGGCCCTCGACGGCGACGACGACCCGGCGGCGGACCCGCCGGTCGAGGAA

GTCGGCACGCGGACCGTCGTCACGACGACGCCGAACAGCAACCTCCTCCA

CGTCGTGGACGCGATGGAAGCCGCCGACGTGGGTCGCGTCCCGGTCGTCG

ACGGGGCGACGGTCGTCGGCATCGTCACGCGGAGCGACGTGCTCGACGCC

TACGACGACATCCCCATCGAGTCGGCGGTCGAGCCGATGCGCTGTCGCCG

AGGCGACGAGGGGGCGCAGTCGGAGTCGCGGGCGGCGCCCCCCACCGCGG

CGGGAGACCGCTGAGCCGACCGCCGAGCCGACCACCGAGCCGACCGACGA

GTCGGCGGAAACTCAGTTCGCGCCGACCTCGGGACCGATGAGGACCCAGT

GGAGTCGGTCCTCGCCGCCGGGGTGGCGCTCCCACCGGACGTGGTAGACG

GCGGGCCCGTGCGGGCACGACGCACAGCCCTCGGCGCACGGCGTCCGCTT

GTGGATTTCGAGGTAGCCCGCGTGCGGCACGACTCGCGTGAGCACCTCGT

CCGGCCCGCGGAACCGGTCGATTGCCGCCTCGCGGTCGAGCCCGTCACCG

TCGCGGTCGCCGAACTCGCCGTCGCCGTTTCCATCGTCGCCGTCGGGGTA

CTCACTCACGTGGCGGCGTCCGCTCAACGCTCCGCGGTCGCGCCCGTTTC

CGACGTACTCGACGAGCGCTTCGAGTTCGGTCTCGTCGAGTTCGGTCGTC

GTCGCGGGCGAGTCGCTCGGCTCTCGCCGCTCGGGTTCGTCGCGGTCCAT

GCCGAACTCTCGTCGCGGCGTCGGTTTATACGAACCGGTTTGAAAACCAC

TTAGGGATTGTCAAGGACCCCGCGACGCACGCTTCGAGGGGTGGTTTCGC

GTCGCTCAGGACGAGGTCGTGGACTCCGAGTCCGGGCGATCGGTCCCGGA

GGGGTCGATTCCGAGGAGCTTCTGACAGAGCTGTCTGGCGGCGAACGAAC

AGAGGAAGTACCAGACGATCCACGCGGGCATGGGGCCGAAGACGGCGTCC

TGCCAGCCCACCCGGCCGACGAGCGGGAGGATAACGTGCGCGGAGCCGGC

GGCGACGTGGCCGCCGCGGACCTTCCAGCGCATCCAGAGGAACGCCGGGA

TGGTGACGAGCATTATCCAGACCATCGGCCTGAACTGCGCTTTCATCACG

CTCCACTGGTCGCCCATCGCCTCCATCTGCTCCGCCTCGATGCGTTCGAG

CGCGTCGTCGTCGCCGCGCTCTTTCGCCGCCTCCCGGCGCGACTTCAGGT

CGTCCATCCGGGCTTGGTGGCGTTCGAGGAGTTCCGTGTCCTTCAGTCGG

TCCTGCATCACGGTCGAGTAGAGGCCGGTCGCCGCGGCGAGGACGATGAC

CACGGCGTAGAACGGTATCGAGTGCGCCAGCGCCCCGAGTACGACGTTGT

CGATGCCGGCGACCGCGTTTCGAACGCCGGCGTTCCAGTAGCCGGCGAAC

AGGACGACGACGCCGAGCGCGCCGAGTCTGTCCCACCGAGACCACGACGA

GTCGGCGTCGTCGCTCCCGCCCTCGACGCCGGGGTCGCGCGATTGCAGTT

TCGAGGCGATTCGGTCCGGGTCGGACAGGACGAAGCCGCCGGCGGCAGCG

TCGGTCAGGACGCCGCGCTCGATGAGGCGGCCCCACTGACCGCTCGACAG

CGCGTCGTTCACGTCGCACCAGCGGAGCTCTTCGGTCCCCCGTTTCGACC

GTTCGACGACGACCGAGACCGCCTCCCGCATCGCCGGATTGTCCAGTAAC

GTCTCTAAAGAGTCTGCGGGCATTCACTCGCCCCTATTCGAGCGGTCGTT

ATCAACGCTTCCCTCCGCCGGAACACAGACCTGCTGACGCTCGACGAGTC

GGTGCCGGTCCAGCGGGCCGCCGCGGTCGCTCAACTAGTTTGTTTTCGGA

TCTGATACTCATCTCGGTCTGAACTCGTCGACGAATATCGGTGACCACGC

TCGACACGCCGACCTCCCATGGTCGGTTGGATATATACTGTACGGGAGTG

AAACCATCTCGTATGTCGAACGCTAGCGCACCGAATTCCGAACTCACGGG

CCCGACCAGACTCACGCTCAAGGTCTGGCACCCGGACTGCTGGACGCTGG

AAGTGACGGAGAATGTCGACGCCGGGGTCACCGCCCACACCGTGTACAAC

ACGCCCGAAGACGCGGTCAAGGGACACTTCACGGTCTACGCCGACCGGGT

CGAGGACATCGACGACTTCGTCCGCGCGACCGAGCGGTCGCGGTTGACTG

ACTCCGTCTCCGAGTTGAGCCCCCGCCACGAGTTCGACAACGGCGCGTCG

AACGTCGGCAACACGACGCGGGAGTTGATGGTCGAGTACGACCCCGAAAA

CAGCATGACCGACTCGCTTCTATCACACGGCTTCGTCCACGACGCGCCCG

TCCGCGTCGACGACGGCTGGGAGTACTGGCCGGTCATCGACACCGCCGAC

CGGGCCGACCTCCGCGAACGCCTCGACTCGCTGGAGGCGGCGAATCAGGC

CGAAATCATCGTCACCAAGGTCACTTCTGTCGCCGGCGCGAAAAACCACG

TCTCGCACCAACTGGACAAGCTCTCGAACCGCCAGCGCGAGGTGTTCGAA

CTGGCCTGTCGACGCGACTACTACACGTGGCCGCGGGCGACGACGACGCG

GGAATTGGCCGACGAACTCGACATCTCGAAAACCACGCTGCTCGAACATC

TCCGGAAGGCCGAGGCGAAACTCCTCAACCAGTACGCGGAGTCAACTCCG

TAACGCCGCCGCCGCTCGTTATTCAATTCGATAACTTTCTCTCCGGTTTA

CTCCGGACACGGTGGTGTCGGTTCGACGGGTCGCGTCTGCGGTACCGACA

GCGTCTTTCCGTTCAGTCGGCCCCCTGTGGCGCGCGCCACGTACAGTCAG

TACAGCGTTCGATTCCGTGTCTCGTCTGCGTCTCCGAACCGCACTCCGGG

CACCGTTGGGCCGACAGTCGTCCCGTGACTGAGTTCGTCATGGCTGAGTC

CTCGGTGGAGGGCGAGTTATACTAGTGTCTGCCCATGGGAGGGTAGCCCC

CGTGAGAGGCCGGGCGAGTGTACACGCCGCAACTCCGGCTTCGGAGTTCG

CCCGCCTGCTGGAGTCGGCTACAGGCGTGTTCTATCTCGATGGGATGTCC

GTCCACTCCGGAGAGTATCTCGTCTATCGGCGTCGCCCCTCGGCAGTCGA

GAAGCGCGAGTACCGCCCGTTCGAGTTCGTCCGTCCGCTCGTCCGTCTGT

TCGTCTCTCACGCGCGCCGACGCGATTCCGACTCGCGTTTCGTCCCACGT

TCGTTTCCCGGTGTCCGGTCGCCTTCGACTCGCTCGTCGTTGGTCGTGAC

CATATCGGTGGTGTCCGCTTCGCGGATATATACACATCCCCGACTATGAT

ACGGTCCGGCCGGCTGTCCGGGTCGAAACCTTCTCACCAGTTGACACTCG

AAGTCGAACTATCAGACGTGGTTTACGGGTCTAAAATGCCCGTATTTGCG

GTATCTACCGATAACTATCGAGTCGTCTCACCAGCGTTCCTATACATCTT

TTGTTGACAACCCCGACCATAGTAGCACATCCGTATAACTACCCTCTGTT

CAAGGGTCTGGGTAGGATTATCATGTCAGACTCAGATCCGAGAACGGCGA

GGGAAGAGTGGGGAAGCCGGTTCGGCTTCCTCATGGCGATGCTCGGCGCG

ATGGTGGGCGCGGGCAACATCTGGCGCATGCCGTTTACGACGGGCGAAAA

CGGCGGTGGTGCCTTCCTCGTGGCCTATATTCTCCTGTTGTACCTCATCG

CGGTTCCCGGGCTGATGGCCGAGACGATGATCGGCCGCTACACGAACTAC

GGGGTCATCGGAGCGTTCAGGAAGGTTCTCGGGAGCAAGCGGGCGCAGGG

TCTCGGCCTCGTAGTCCTCATCGTCAACGTGGCGCTGATGTCGTACTACG

CGCCGATCATCGGCTGGGCGCTCTACTACGCCGGTCACTCGATTCTCATG

ACGTTCACCCAGCCGGGGTTCCAGCCGCAGGCGTTCTGGGAGGGTTTCAT

CAACAACCCGGCGCTCGTCGTCGGGATGCACACGGTGACGATGGCCGGTC

TGGCCGGCGTTCTCGTGTTCGGCATCCGCCGGGGCATCGAGCGCGTCGTG

AAGTGGATGATTCCGCTGCTCGTCGTCGCGCTCGTGGCGGTCTCGATTCG

CGGCATCACGCTCCCCGGCGGTATGGAGGGCGTCGCGTTCGTCTTCACGC

CCGATTGGGGGTATCTCACCCGCGGCAGTACGTGGGTCGCGGCGCTCAGT

CAGGCGCTGTTCTCGACGGGCCTCGGGTGGGGAATCGCGCTGACCTACGG

GAGCTATCTCAGCCGGTACGACGACGTGCCGCTCGGCGGCGGTCTGTTCA

CCGCCATCGGAAACACGAGTATCGGCCTGCTCGCGGTGTTCGCCACGTTC

CCCGTCGTCTTCGCGTTCGGACTCGAACCGAGCGCCGGGTCCAACCTGCT

TTTCATCTCGATGGCGCAGGTGTTCCCCGAACTCCCCGGCGGGAGTCTGT

GGGCCATCGTGTTCTTCGTCTCCTTCTTCTTCGCGACGTTCACTTCCGGC

CTCGGCATCACCGAGGTCGCCGTCACCACCGTCTCGGAGGAGACGCGGCT

CTCGCGGACTAGTGCTGTGCTCGCGTCCTGCGGCGCGATTTGGCTGTTCG

GCCTCCCGAGCGCCTACTCGTCGGCGTTCCTCGGGCGGATGGACTTCATG

TTCGGGAGCTTCGGCCTCCCGCTGGCGACGCTTTCTATCATCGCGCTCGT

CGCGTGGAAGTTCGGCCCCGAGCGCGCCCGCGTCCTCGACCTCAACCGCA

ACGCCGGCGTCTACATCGGCTCGTGGTGGAATCCGGTCGTGAAGTACGTC

ATCCCCGTCGTGATGGTGTTCATCCTCGGCTACGGCGTCTACTCCAGTAT

CGGGACCGTGAACCAGCGGTTGATGCTGCTCGGCGTCGCGTTCATGGCCG

CGCTCGTCGTTGTGAGCACGCTCGTAATGAGCGTCATCGGCGACGAGCCA

CCGTCCGGTTCCGCGGCCGTCCCGGGAGGTGACGACTGAGATGGCCCTTT

CGACGCTCACGTGGGTTTCGATGCTCGTCTCGCTCCTCCTGTTGCCCGGC

GTCGCGGCCGCGGTTCTCGTCAGGTCGCTCCGCACCGAGGAGCGCAAACT

CGCGCTCTTACGGGAACAGGACGACGTCGATAGCTACTCGCCGCGGGCGC

TTTCGGACCTCCGCGAGTGGATTCGCGCGAATCCCGACGACCCCTACTCC

CCGATTGCCCGCCGCCGTTACAACGAGTGTGTGCGGTCGCTCCGAGCGAT

TGACGAGCCGCATTACGACTGGTCCGACGAGCAAATCGCCCGGCTCGAAC

TGGTGGACGAATGACCTCTGACTCCGCTACCGACGAGCCCGCGGACGAAC

GCGAGTTCGAGTTCGACGAACTCACCACGGACGCGTCGTTTACGACCATC

GACACGCACACAGAGGGCGAGCCGACCCGCATCGTCGTCGACGGTATCGA

CCGCTCGGCGCTCGTGGGTGACTCCGTTCGGGAGCGCCGCGATTCGTTCG

CCGACACCCACGACTGGGTGCGCGACCTGCTGATGCGGGAGCCCCGCGGC

CACGACGACATGTTCGGGGCCGTCGTCGTTGATTCCGACCATCCCGACGC

GGACGTGGGCGTCTTCTTTATGGACAGCCGAGGTTACCTCGACATGTGCG

GCCACGGGACTATCGGCGTCGTCTCCGCCCTCCTCGAACTGGATCGACTC

GACCGGCGCAAGACCATCCGCGTCGAGACGCCGGCCGGACTCGTCGAGGC

GACGCCCGAGTACGTGCCGGACGGGGGCGTCGAGTGCGTCACGATACAGA

ACGTTCGGTCCTTCGTCTACGACCAAGCGACCGTCCCGGTCTCGTTTCTC

GACTCGTCGCTCCGCGTCGACGTCGTCTACGCGGGCAACTTCTTCGCGCT

GGTCGACAGCGACCAGCTCGGCCTGCCCGTCGAACCGCAGCACGCGGCGA

CGTTCGTCGATTGGGGCCTCGAAATCCGCGACGCCGTCAACGAGCGCCTC

GATATCGTCCACCCGCTCACCGGCGAGGAAGGGTCGATCTCGATCACCGA

GATATACGGCACACCCGACGAGGTGGACCGAAGTATCGTCGTCTTCGGCG

AGGGTCAGGTCGACCGCTCGCCCTGTGGGACTGGGACATGCGCGAAAATG

GCGCTCCTCCACGATGCCGGCGCGCTCGATGTTGGCGAGTCGTATCTCCA

CGAGAGCATCGTCGGTACGCGCTTCGAAGGGAGGCTCGTCGCGGCCGAAG

AACGTGACGGCGTAACGCTGACGACGCCGCTCATCACCGGTTCTGCCCGC

ATCACCGGGAAACACACGTTCGTCAAAGACGCGAAGGACTCGCTTTCCGG

GTTCAGCATCTCGGCCGAGTAGCGGGTTCGGCCGGACCGCTCTTCTTCGG

GATTAGTCCAGCCCGCTCGATAGCTCCACGTCGTCGTTCTCCAACTCGCC

GCCGGCCGGCGACCCGCGGACGTCGCCCTCGCGCCAGGTTCCTTGGCGGA

ACCAGAGCCAAGCGATGGTCGCGCCCACGATATTCGAGACGAAAAAGGCG

ATCCAGATGCCCCGAACGTCGGTGGTCCCGAGGAACCAGAAGTCCGTCGG

GAGGAGTCCCTGCGACGCGACGAAGGCGACCGGAAGCCGGATGCCGGCGA

GGGTGACGACCGATATTACCGCGGCGATGAGGGTCTTGCCCGCGCCCCGG

AAGCCGCCGGAAAAGGCCCGCATGATGCCGATGAAGCCGAACGACAGCGA

GACGTACCGCAGGAACTCCGCGCCCACTTCGAGCACCTCGGCGTCGGTCG

TGAACACCGAGACGATGGGTTCGGGAACGAGGAAGATGGCGACGCCGAGC

AGGCCGAGGACGAAGAACAGCCCCTTGGCGGCGACGTAGTTCGCCTGCTC

CGCGCGATCGTACTTCCCCGCGCCGATGTTCTGGCCGGACATCGTCTCGA

CGCCGCGGGCGACGGCGATAGCGGGGAGGAAGATGACCGAGAACACGCGG

GTCCCGATGCCGAACGCCGCGACGACGGAGGTCGAAAACAGCCCGACGAC

GATGAGAAGCGCGTTGATAGAGAGCGCGCGACCCGCCCCCTCGACACTGG

CGGGGATGCCGATGTGGAGAATCTTCCGCAGATATCGGAAGTCGGGTCGC

ATGTCAGTGAGGTGGATTTGGATGCCTCTGGACCCCGACACCATGATGGC

GATGCCGACGACCATCGCGAGGACTCGCGAGATGACGGTGGCGATGGCCG

CGCCCTGAATCCCGAGTCGGGGGAACTGTATCGGCCCGAGCGCCCATCCG

TTGATGAAAAACGGGTCGAGGACGACGTTGAGGACGACGGTCCCGAACAT

CACGAGCATCGGCGTGATGGTGTCGCCCGCCCCGCGCATCAGCGAGATGA

ACACGAAGAAGCCGAACATGAACGGCAGGCCGAGCGCGATGACCTGCATG

TAGGCCGTCGCGCCCGGCAACACGTCCGGCGAGGCCCCGAGGAAATCGAG

GAACGGTCGGACGAGCGGGTAGCCGAACGCGCCGAGCATCGCGGAGCCGA

GGAACGCGAACGTGACGGTCTGTGAGGCCGCGTACTCGGCTTTGTCCGTC

TCCTCGGCCCCGGTGTGCTGGGCGACGAGGACGCTCCCGGCGACGGACAG

CCCCATCCCGAGCGAGATGAGGAGAAACACCATCGGGAAGGCGAACGAGA

TGGCGGCCAGCGCCTCCGTCGAGTACTGGCCGAGCCAGAACGTGTCCGCG

AGGTTGTACGCCGTCTGGAGGAGGTTCGTGACGACGATGGGAAGCGACAG

GTAGAGAAGCGGCTTGACGATGCCGCCCTCGGTGAGGTTGAGCTCGTCTT

GCCCCTTGAACAGGCTCATCCGTCCGCCCCCTCACCCGGCGTCCAACCGA

GGTGGAGTTCCAGTGCGTCCTCGACGATTCGTCGGGTCTCGGCGGGGTAC

TCGTCGAGCGCGACGACGCGGGCGTGCGCGCCGTTGCTCATGGTCGTGAT

GAGCCGCGCAATCTTCTCGGGGTCGGTCTCGTCGAACTCTCCGCCGGCGA

CGCCGTCGCGGACGATGTCTGCGACGACCTCCCGCATCACGCCGTCGAAG

TCGAGGAAGCGCTCGCGGAACAGTTCGTGGTAGGGCGCTTGGGCCTTCAG

TTCCATGACCGCCACGGGAAAGCCGTCCGCTTCGGGGTTCTTCGGGCTGA

ACACCGCGTCGAGAACCGTATTCAGTCGCGTCCGCGGGTCGGCCGACTCA

CACGACAGCTCCGACTCGAACCGCCCGATTAAGTCGTCGAGAAACGCGTT

CAAAAGCTCCTCTTTCGTGTCGAAATGGTAGTGAATCGCGGCGCTCGTCA

CCGACGCCTCGTCCGCGATTCGCTGTACCGTGAGGCCGGCGTACCCGTGT

TCGCAGAGCGCCCGATTGGTCGCTTCGAGTATCTCGGCTCTGGTCTCTGC

GTCCATCTTCTGTCGGGCTTACTAACCGGTCAGTTAAGAGGCTTTGGACC

GCGAATCTCCCCGTCAGAGAATCCCACAGCCGTCCGACTGCCGGTGTCTC

TAAGTAGCCGAGTCGGCTACCGACGTAGTAGTGACCTTCTCGTTGTCACC

GTACGTGGGTGCGCTCTTCCTCGCGGCCGTGATATCCGCCGGCGTCGCGA

GCTACGGGTGGCGATACCGAGACGAACCGAGCGGTCGGTGGTTCATCGCC

CTCACGGTCAGCGCCGGCTGTTGGTCGCTTTTCGAGGCGTTTCTCCTCCT

CGCTGACTCGCCGTTCACCCGGCTTCTGTGGTTCGCGCTGGAGCCGATTC

CCGCGGAGTCGTCGGTGTTTTTCACCCTGCTTTTCGCCCTCGAAATCACG

GGCTACGAGCGGTGGGTCTCGCGGCGGACCGCCGCGCTGCTCGCCGTGTG

GCCCGCGTTTGCCATCGCGGGTGTGGCGACGAACGCCCTCGGCGTCCACG

CGCTTCTGTGGGAGTCGCTGTCGTTTGCGACCGTCGGCGGAATCACCGCG

ACCGAGGTCGTTCCCGGGCCGCTGTTCTGGGTCGACGTGGTTTACAGCTA

CGTCGCCATCGGAGCCGCGCTCGTCGTCCTCGGCGTCCACTGTCTCCGGA

GCCGACAGATATACCGGAAGCAGGCGCTGGTCCTGTTCGTCGCGATTCTC

GTTCCCGCCGTCGTCGGCGCGGTCTATCTCCTGATACCGTTTTCGCCCGC

GAACCCCATCGCTATCTCGTTCGCCGCCACGTCGTCGCTGCTCGCCGTCG

GGCTCTCGCGGTACCGCATTCTCGACATCAGCCCCATCGCGCGCGACCTC

GTCATCGAGCGGATGGCCGACCCCGTCCTCGTCGTCGACCGGAAGGGCAG

ACTGGCCGACAGCAACCCGGCGGCGAGGGCGCTGTTCGACGTTTCACACC

CGCCGCTCGGCTCGCCCGTCGCCGACGCGTTCGACCCGGAGACCGTCGAG

GCGCTCGAATCCGGCGGGGAACTGCGGGTCGTCGACGACAACGGCGAGGC

GCGGCACTTCGAGGTCGACCGGTCGGCCGTGACCGACGGTCGAGACGCGG

TTCGCGGCCACCTGCACATCCTCCACGACATCACCGAGCGCCGCGTCCGC

GAGCGGTGGTTCCGCGCGCTCACGGAGAACGCCTCCGACCTCATCTTCGT

CCTCGACGCCGACGGCTCGGTCACCTATCTGAGCGACTCGGCCGCCCAGA

CGCTCGGGGCCGACGAGGACCCGAGCGAGGTGCGGTCGCTGCGGCGGTTC

CTCCACCCCGACGACGAGGACGCCGCCATCGAGACGTTTCGGGAGGCGTT

GGAGCGCCCCGACGAGGACGCCACTGCCGAACTCCGGTTCCGCAGTTCAA

CCCGCGGGTGGCGCGTCTTCGGCGTCCGGTGTCGGAACCTCCTCGACGAC

CCCGTCGTCGGCGGCGTCGTCGTCAACGCCCAGGACGTGACCGACCGACG

GAAGCACGAACAACAGCTCGAAACGTTCGCCAACATCGTCTCCCACGACC

TGCGGAACCCGCTGAACGTGGCGGAGGGCTACCTCGAACTGGTGAAGGAG

GCCGACCGCCCCGACCCGGACCACGTCGAGCGAATCGAGGCGGCTCACGA

GCGCATGGGCGAAATCATCGCCGACGTGCTCGCGCTCGCCCGCGGCGGAA

CGGTCGACGAACACGAGACGGTCTCGCTCGACGCCGTCGCGGCCGAAGCG

TGGACGAACGTCGAAACCGGGGCCGCGACGCTCGATATCGTCGAGAGCGC

GTCGTTCGACGCGGACCGAACCCGACTGCTCCAGCTGTTCGAGAACCTGT

TTCGAAACGCCGTCGAGCACGGCTCGATGGGCAGTCGGGATTCTCCCGAC

GACGCCGTGGAACATGGTTCCACGGGCAACCAGACTGCGTCTGGTGACGC

GGTCGAACACGTCGGCGACGGCGTGACCGTCACGGTCGGCGCGACCCGCG

ACGGCTTTTTCGTCGAGGACGACGGTCCCGGCGTGCCGCCGGCGGACCGC

CCGAAGCTCTTCGAATCCGGCTTCACGACGGCCGACGACGGCACCGGCTT

CGGCCTCGCCATCGTCCGCACCATCGCGGACGCTCACGGCTGGTCGGTCG

GCTACGAGGACGTTGCGGACGCCGGCGCGCGGTTCGTGATTCGCGGCGTC

GAGCGCGCCGGGGACTCGGCGCAGGAGTCGGGGCCGGCGTCGTCGGACGA

CGCCTGACGACGGGCCACTGGCCGACTAGGCCGCTACTGGACCGCCGCCG

AGCTACCAAAAGCGGTTCTCGTCGAGCCGCCGCAGCGCCTCCTCGATGGT

CGCCTCGTTTCGGGAGAACGTAAACCGGACCCAGTCGGCGGAGCCCTCGG

TGTAGAAACTGCTCCCGGGGACGACCGCGACGCCGGCCTCGCGGACGAGT

CTGTGGGCGAACTCGGTGTCGTCGGCGTCGCCCGGATAGCGCGTCAGCAT

GTAGTACGCGCCGTCCGGTTTCACGGGGTCGAGGCCGACCTCCCGGAGGC

CGTCGTACAGCAGTTCGCCGCGGCGCTCGTAGGCGTCCGAGAGGTCGTCG

TAGTAGTCCGCCGGGAGCGACAGCGCCTCGACGCCCGCCCGCTGGAACGG

CGTCGGCGCGCAGATGCTCGTGTAGTCGTGGACCTTCCGCAACTCGGCCG

AAAGCGGTTCGGGCGCGAGCGCGAACCCGACGCGCCAACCCGTCACGGAG

TACGTCTTCGACAGCCCCGTGCAGACGACGGTCCGCCCGGCGAGGCCGTC

GACTTCGACGGGGCTGACGTAGTCGTCGGCGTAGACGATGTGCTCGTAAA

TCTCGTCGGTGACGACCATAAGGTCTTCCTCGGCGGCGACCTCCGCGACG

AGTTCGAGTTCGGCGGGCGAAAAGACGTTGCCCGTCGGGTTGTGCGGGTG

GTTGAGAATCAGCATGGACGCCTCTTGGGCGGCGTCCCACAGCGCGTCGG

CGTCGAGTTCGAGGCCGTCGGTGATGTCGAGCGGTATCGGCTCCCCTCCG

GCGAACTGGACGGCGGGAATGTAGCTCTCGTAGGCCGGCTCGAAGTAGAT

GACGCCGTCGCCGGGGTCGCAGAGCGCGAGGAGCGTCGAGACGACGGCCT

CGCTCGTGCCCGTGGTGACGGTGACCTCCGTTTCGGGGTCGTAGCGGACG

CCCTTCCAGTCGGCGTAGCGTTCCGAGACCGCCTCGCGGAGTTCCGGCAG

CCCCCACGTGATGGTGTACTGGCTCGACGTGTCGATTGCGGCTTTGGCGG

CTTCCTTCACGCTCACGGGGGTCTCGTCCTCGTCGGGAATCCCCTGTGAG

AGGTTGATAGCGTCGTGTTCGTGCGCCTCGCGGGTCATCTCCCGGATGAC

GGACTCGCGGGTTCCCGCGACGCGCTCGCTCCCGATGCGTCGAGAAAAGG

GAGACGGTGTTAACATACGTGGGCTGGGACTGGCTGACACAAGAAGCTTG

GCGGACGCGCTCGACGCGCCGGGTCGCCCACCCCGACGAACGTTTTAATG

CGATGAGGCCGACGTACCACCTGTATGTCGAACCGTCGTGGGCCGGACTT

CGAAACGAGGATGGCCGAAGACGAGGAGATTCCGCCGTCGCCGCAGTTCG

TCGAACAGGCCACCGTCACCGACGATTCGGTCTCCGACGAGTTCGAGGAG

AACTGGCCCGACTGCTGGGCCCGAGCGGCGGCGACGCTCGACTGGTTCGA

CCCCTACGAGTCGGTCCTTCCGGACGACGAACCCCCGTTCGAGTGGTTCG

CCGGCGGGACGCTGAACGCCTGCTACAACTGCGTGGACCGCCACGTCGAA

GCCGGGGCGGAAAACCGCGTCGCCATCAAGTGGGAGAGCCACCTCGGCGA

GACGCGGACGTACACGTATCAGGACCTCTACCGCGAGGTCAACGAGTTCG

CGGCCGCGCTCCGCGCGCAGGGCGTCGAGGCCGACGACGTGGTCACGCTC

TACATGCCGATGATTCCGGAACTCCCCATCGCCATGCTCGCGTGCGCCCG

CATCGGCGCGCCCCACAACGTCGTCTTCGCGGGGCTGTCGGCGGACGCAC

TCGCGACGCGACTGGAGAGCGCGGACTCGGTGTTCCTCGTCACTTGCGAC

GGCTACTACCGGAGAGGGAGCGCCATCTTCCTGAAGAGCAAGGCCGACGA

CGCCCGTCTCAAGGCCGCCCACGACCTCGAAGAGATGGTGGTCGTCGAGC

ACCTCGGAGAGACCGAACACCTCGGCGCGGGACAGCACACCTACGAGGAA

CTGGTGGCGGCCCACGCCGGCGCGGAGGTCGCCCCGGTCGAGCGGGCGGC

CGACGACGTGCTGTTTCTCATCCACACGTCGGGAACCACGGGCGAACCGA

AGCGGGTCGAACACGTCACCGGCGGCTACCTCGCCCACGTCGCGTGGACG

TCGCAGACGGTATTGGACCTCGACCCCGAGGACACGTATCTGTGTACGGC

GAACATCGGCTGGATTAGCGGCCACTCGTACGTCGTCTACGGGCCGCTGG

CGCTCGGGGCGACGGCGGTCATGTACGAGGGGACGCCGAACTACCCGGAG

CGCGACCGCATCTGGGACCTCATCGAGGAGAACGCCGTCGACGTGTTCTA

CACGGCTCCGACGGCGATTCGCTCGTTCATGAAATGGGACACGTCGCTCC

CGGCGTCGCGGGACCTGTCGAGCCTCCGCCTGCTCGGAACCGTCGGCGAA

CCAATCGACCCGCGGGCGTGGCACTGGTACTACGAACACATCGGCAACGG

GAACTGTCCCATCGTCGATACCTGGTGGCAGACCGAGACCGGCGGGATGA

TGATTACGACTCTGCCGGGCGTCGAGAAGATGAAACCCGGCTCAGCCGGA

CCGCCGCTCCCGGGCGTCGGCGCGCGGGTCGTCGACGGCCGCGGCGAGGA

GGTCCCCGTGAACGAGGCCGGCTATCTCGTTCTCACGCGCCCGTGGCCCG

GGATGCCGCTCGCCCTGCGACACGGCCACCGCTGGGGCGAGGAGGCGGCG

AAACTCGACGTGGACGGCTGGCACTACTTCACCGGCGACGGCGCGAAAGT

GGACGACGACGGCTACATCACCGTGCTCGGCCGCGTCGACGACGTCATCA

ACGTCTCGGGCCACCGCCTCGGGACGATGGAAATCGAGTCGGCCATCGTC

GGCGTCGAGGGCGTCGTCGAAGCCGCCGTCGTCAGCGTCGACGACGCCGA

GGCGGGTGGCATCCACGCCTACGTCACGCTGGAGACGGGCGTCTCCGGTG

ACGACGACCTCCGCGAGCGAATCGCCGCACACGTCCGCGAGCGCATCGGG

CCGATTGCCACGCCGGACGCCGTCGTCTTCACGCCCGACCTCCCGAAGAC

GCGCTCCGGGAAGATTATGCGCCGGTTCCTCGAAAACATCACCAACGGAG

AGGAGTTCGGCGACACGTCCGCGCTCCAGAACCCGGAAATCGTCGGTGAA

CTCGAATCCATCGTCCGTGACGGCTGACGACGTTTCGGACAGTTCTCTCT

AAGCGAAACGCAGTTACTATGGTTCGGGAGCGACTTCCACGCCGATTGTC

GCTTTCACCATGTCGGTTCTGTGCTCTCATCGGAAGTTTTAGTTCGCTTA

TCGACAGTGTTGCGAAACGGATGCGGGACGAAAACTCCGAACCGACGCCG

CTGTCGGCGGGCGGGTACGAGCAACTGCGCCGGGCGACCGAGACGTACCG

CGAGGACCTCGTGCTTCGACTCGGCGCGGAAGTCGGGCTCACACCGGCCG

AGATGAGTCAGTTCGAACCCGCACACGTGACCCAGCGGCGACACCGGGGA

ACGGAGCACTACTTCGTCGTCGTTCCGACCGACGGTCGGGAGGCGTACCT

GCCGAACGAGGTCGAACACGACGTTCGGAAGTACGCCACGACGACAGACA

TCAGCGAGACTGAACGGGTGCTTCCGGTCACGCCGCGGCGTCTGCAGATG

CTCGTGGCGTCGGTCGGAACGCGGGCCGCCGAACGAACCGGCCGCGATGG

GTTGGCGGACGTTTCGACTCGCGACCTGCGTCGGCACTTCGCCCGAACGA

TGCTCGGTGACGGCGTCCCGCCGAGCGTCGTCATGCGAATCGGCGGGTGG

GACCGCATCGAGAGCCTCGCGCCGCTCCTCGGTGAACCGAGCGAAGACGA

GGTGCTCGATGCGCTTTCGCCGGCCCCTGCCGACAGCGACGGGCGCGCCG

TCAGCGGCGACCCCGACCGAACGCGTCAGGTCATCGAAGCCGCGCGAAGC

GTCGGGTCGGCGCTCTCGGCGGTGAACACCCGAAGCGAGGTCGAACAGGC

CGTCTGTGACCGGCTCACCGATTCAGACCGCTACCGTTTCGCTTGGATTG

AATCGGGCGAAACCGACGCGCGAACGTCGCGCACCGCCGCCGGTCTCGAC

GAGACGTCGCTCGAAGCGGTCCGGGGGTCGCTCGCCGGTCGCGGCACCGA

CTTCACTCGACGCGCGTGCGACACCCGCGACGTACAGACGGTGCGTCCCG

ACTCGCTCCCGAACTCGGAACCCGTGCCGATTGCGGTCGTTCCGCTGGCG

CACGGCGACACCGCCTATGGAGCGCTCTACGTCGCGCTCGACGGCCAAGC

GGTTCCCGACGTCGAGGCGGACGTACTGGTGACGCTGGGTCGCCACGTCG

GACAGGCAATCGCCGCCGCCGAGCGTCGGAAGCTACTGCTGGCAGACACG

GTCGTCCAACTGGAGTTCGAATCCTCGGACGAGCAGGACGTGCTCGTTCG

ACTCTCCGCGGCCCTCGGCTGTTCGATTCGGCTCCGCGGCGTCGTCCCCA

TCGAAGAGCGGTCGTTGCTGTGTTTCCTCACCGTCCGGGAGGCGACGACG

GACGCGGTGTTCGACGAGTTGCAAACGACGGACGCCGTCGAGCACATCCG

CCTCATTCGCGACCGAGGGACGGAGTCTCTCCTCGAAGTCGCGCTCTCCG

CGGGGTCGACGATTACGACGCTCACCTCCTACGACGGCACCGTCTCGCGG

TTCGTCGCGGAGGACGGCGTCGCCCGTTTCGTGGGCGAGTTCTCGAACGA

AACGCCGCTCCGAGACGTGCTGTCCGACCTGACCGACGCCTATCCCGCCA

CCGAACTCGTCGCGAAACAGGAGGTCGAACGGCCCACGCGAAACACCGCC

GACTTCCGGGCATCGCTCGTGGCGCGACTCACCGACAAGCAAGAGTCCGT

CCTGCGGGCGGCCTACCTCGCCGGCTATTTCGAGTGGCCCCGCGGCAGTA

CCGCCGAAGACCTCGCTGATTCGATAGATATCTCGTCACCGACGCTCCAC

CAGCACCTCCGAACCGCACAGCAGAAACTCATGACCGCGTTCTTCGACGA

CGACACCGACGAACGCGCTTCGACCCTAGACCCTTAGGCGTGCGACGAAT

TAGCACACGAACGTCCGTTATTGATTGATAGATTATCAAAATTTATGGAC

AATAGTATGAGCCGTTCATCTGTGGCTCTGAAGCCTATACACGTGCCCAC

AGACTATTATGTATATTCGTGGTGTTAACTTTCGATAATTAGTTATCCTA

CTTATGCCCTTTGTCGAGACTCGTTAGCACGTACTATGGCACAGGGTGAC

GCTGAACTGGAGGCACGGCTGGCAGAACAAGACGAGTTCGAACCCACCGA

GGAGTTCGTCGCGCAGGCGAACGTCTCGGACGCCGGCATCTACGACGAGT

TCGAGGAGAACTGGCCCGACTGCTGGGAGCGCGCGGCCGACCTGCTCGAC

TGGGACGCCGACTACGACGAGGTACTGGACGATTCGAATCCGCCGTTCTA

CGAGTGGTTCACCGGCGGCGAACTGAACGCCTGTTATAACTGCGTGGACC

GCCACGTCGAAAACGGGGACAAGAACCGCGTCGCCATCAAGTGGGAAGGC

GAACACGGCGAGACGCGGACGTACACGTATCAGGACCTCTACCGCGAGGT

GAACGAGTTCGCGGCGGCCCTGCGTGACCTCGGCGTCGAGGAGGACGACG

TAGTCACGCTCTACATGCCGATGGTTCCGGAACTCCCCATCGCCATGCTC

GCGTGCGCTCGCATCGGCGCGCCGCACAACGTCGTCTTCGCCGGCTTTTC

CGCCGAGGCGCTGGCGACGCGGATGAACGCCGCGGACTCCCGATTTCTCG

TCACCTGCGACGGCTACTACCGCCGCGGCGACCCGCTCGACCACCTCGAC

AAAACCAACGAGGGACTCGACAGCGTCGACCACGGCGTCGATGCCACGGT

CGTCGTCGACCGACTGGGTGACGACGGCTTCGACCACGACCTGAAGGGCA

ACCAGCACGACTGGGACGATCTCCTCGAAGCCCAGAGCGGCGAGCGCGTC

GCGCCGGTCGAACGCGACGCCGAGGATATGCTGTTCCTCATGTACACCTC

GGGGACGACGGGCCAGCCGAAGGGGGTCAAACACACCACCGGTGGCTATC

TCTCCTACGCGACGTGGACCTCCCACGCGGTCCTCGACATCGAGACGGAA

GACACCTACTGGTGTTCGGCCGACATCGGCTGGATTACGGGTCACTCCTA

CATCGTCTACGGGCCGCTCGCACTCGGCTCGACGACGGTGATGTACGAGG

GCACGCCCGACTTCCCGAACCGCGACCGGATGTGGGAACTCGTCGAGAAG

TACGCCGTCGATATCTTCTACACCGCGCCGACGGCGATTCGCGCGTTCAT

GAAGTGGGGCAGCAAATACCCCGAATCGCGCGACCTGTCGAGTCTCCGCC

TGCTCGGGACCGTCGGGGAACCCATCAACCCGCGCGCGTGGAAGTGGTAC

TACGAGCACATCGGCAACGAGAACTGCCCGGTGGTAGACACGTGGTGGCA

GACCGAGACCGGCGGGATGATGATAACGACGCTCCCGGGCGTCAAGAACA

TGAAACCCGGCTCAGCCGGACCGCCGCTGCCGGGCATCGACGCCCAAATC

GTCGACACCGACGGCGACGAAGTCGAACCCGGTCGGGCGGGCTACCTCAC

GGTGAACAAGCCGTGGCCGGGGATGCTTCGGACGCTCTACGACAACGACG

AGCGCTACATCTCCGAATACTGGGCGGAGTACTCCGACACCGACAGCGAC

GACCCCGACGACTGGGTCTACTTCCCCGAGGACGGCGCGAAGGTGGACGA

AGACGGCTACATCACCGTGCTCGGCCGCGTCGACGACGTCATCAACGTCT

CGGGCCACCGCCTCGGGACGATGGAAATCGAGTCGGCCATCGTCGGCGTC

GAGGGCGTCGCGGAGGCCGCGGTCGTCGGCGGCAACCACGAGGTGAAAGG

CGAGGCCGTCTACGCCTACGTCATCACCGAAGACGGGTACGAGGAGGGCG

AGGACCTCCGCGCGCGCATCGTCGAGGGCGTCGAGGACGCCATCGGCCCC

ATCGCCCGCCCCGAACAGGTCGTGTTCACGCCGGAACTGCCGAAGACGCG

CTCGGGCAAAATCATGCGCCGTCTCCTCGAAGACGTGGCGAACGGCGCGG

AACTGGGTAACACCTCGACGCTCCGGAACCCCGAAATCGTCGGGGAGATA

GAAGCGAAAGTCCGCGCACAGAGCGACTGAGCGGCCGCCTTCGGCCGCCC

GTATCGAGCGTAACTACGAGACGACCAACCGATCTATGTCACGCGAATCC

ACCGAGTCAGAGATTGACTCACACGACGCGAGCACCGACCGGAGTACGGC

TCCGAGACACGACGAGATAGACTACCTGTCGCGGGAGGTGAACCTGCTGA

AGCCGAGCACCCCGTTCATGCGAGACCACCTGAAAGTAATCTGGACCGGC

TTTATCGCGTGGGCGCTGCTCACGTTCGGACCGCCGGTGCTTACCTACTT

CGCACCGGCGACGATGACCGTACAGCTCCCGGTCATCGGCTTCCCGCTGC

ACTACTTCCTCGTCGCCGTGTTGTCACCGACGAGTTCGCTCGTGCTCGCG

TTCATCTACTCGCGCAAGCGCGACCAGCTCGACGAGCGGTACGGCATCGA

CCACACGGCCTCGCAGACGCGCTCGAAGAGTGAGAGCGAGGCCGCCGTCG

CCGACGGGGGGCGCGTCGAATGACCGCGAGCGTGTTCCTCCAGTCGGGAG

ACCTGCTCCCCGAGGCGCTCAACATCTCGTTCAAACTCGTGCCGGCCATC

ATGGTCGTCGGGATGTTGGCTCTGTTTCTCGCCATCGGCTACGTGTTCAA

GGTCGCCGACACCGAGGGGATGTGGGTCGCCGGCCGGTCGATTGGCAACA

TCGAAAACGGGATGGCCATCGGCGCGAACTGGATGTCTGCGGCGTCGTAC

CTCGGGTTAGCGGGGCTCGTCGCCCTCTCGGGCTTCTACGGGCTCGCGTT

CATCATCGGCTGGACGGCCGGCTACTTCGTCCTGCTCATCTTCCTGGCCG

CGCAGATGCGCCGGTTCGGGAAGTACACCGCACCCGACTTCGTCGGCGAC

CGATTCAACTCCGACACGGCGCGGGCAATCGGCGCGCTGACGACCATCCT

CATCGGGTTCGTCTACTCGGTCGGGCAGGCCCGCGGGATGGGCCTCGTCG

GGTTGTACGTCTTCGGGACCGACTACGTCACGATGGTCATCGTGATGATG

GCGATTACGGTGGGCTATCTGACCATCTCCGGCATGATGGGCGCGACGAA

GAACATGGCCGTCCAGTACGTCATCCTCATCATCGCGTTCCTGACGGCCG

TCTACGTCGTCGGCTTCACCGGCGGCTACTCGACGGTCCTCCCGCACATC

GAATACGGCCAACTCATCGGCGAACTCTCCACCGAGTTCTCCGAGCCGTT

CGCCAACGCCGGGTTCTACCTCTGGATTGCCACGGCGTTCTCGCTCATCT

TCGGCACCTGCGGGCTCCCGCACGTGTTGGTGCGGTTCTACACCGTCGAA

AACGAGAAGACGGCGCGGCAGTCGACCGTCTGGGGGCTGTTTTTCATCCT

GCTCCTGTACTGGAGCGCCCCGGCGCTGGCGGCGTTCGGCGTTGACCTCT

ACGACGCCTCCCAGTACGGCCCGACGTTCGCGGCCAACGGCGGCATGAGC

GGCGGTGAGGGCGACCTCATCGTGGTGCTCGCGGCGCAGCTTTCGAACCT

GCCGACGTGGTTCGTCGGCCTCGTCGCCGCCGGTGGCATCGCCGCCGCCA

TCGCCACGACGGCGGGGCTGTTCATCACCGCCTCGTCCGCCGTCTCTCAC

GACATCTACACGAACATCATCAACCCCGACGCGACCCAGCGTCAGCAGGT

GTTCGTCGGTCGCGCGACCATCGTCGCGCTCGGCATCATCGTGACGGTAA

CCGCGTTCGACCCGCCGGCGCTCGTCGGCGAACTGGTCGCGCTCGCGTTC

TCCCTTGCGGCCATCGTGCTGTTTCCGATGTTCTTCCTCGGTCTCTGGTG

GGAGAACACCAACCGTCAGGGCGCGCTCGCGGGCATGAGCGTCGGCCTCA

CGCTCTGGGTCGCCGCCGTGGTCAACGACCTCATCTTCCACTTCAGCGAC

CTCTTCGCCGAGTTCGTGCCGGCCATCGGCGCGGCGCTGGTCGGCACGCC

GCTCGTGTTCGTCGTCACCATCGCCGTCTCGATGGCGACTGACGAACCGC

CCGAGCGAATCAAGAAGATGGTCCGGCAGTGTCACAGCCCCGAACCGATG

GGCCAGCAACAGTCCGCAGAAGACGTGGTCATGAGCGCCGACGGGGGCCA

GTCCCCCGCGGACGATTAACCAATGTACGAACACATCCTCGTTCCGACTG

ACGGAAGCGACGCGGCGGAGTACGCCGTCGAACAGGCGGTCGACCTCGCC

TCGAAGTACGGCGCGACGGTCCACGCGCTGTACGTCGTCGACGTCGACGC

CACAAGCTACTCGCTGGGAACGGAGCAGGTCGACCGCATCCGGCAGGGCC

ACCTCGACGACATGCCCGAAGTGAAGGACGCGGCCGACGCGGCCACGGGC

TACGTCGCCGCCGCCGCCGCCGAACACGGACTGGCCGTCCGCGAACACGT

CGTCCCGGGCGAACCGGCGCGAGCCATCAGGAAGTTCGTCGAGGACAACG

ACATCGACCTCGTCGTGATGGGCTCGCACGGTCGCTCCGGTCTCTCCCGG

GTCGTCCTCGGGAGCGTGACCGAACGCGTCCTCCGGCGGACGCGGCTCCC

GGTGCTTGTCGTCGACGTCCACGAAAAGAAAGCGGAAACGGAAGCGGAAG

CGGACGCGTAACCGATGTACGACGCGACCCACGCGAGAACGGCCACCACC

CGCGCGACGACGACTACGTCGCCCGCGACTCCCGGCGAGGTGCCCTGTCG

ATGAGCCTCGCGGAGACGATTCGCACCCACGTGCGCGAACACCGCTCGGG

GATGCTAACCGACCTGCTGTTCGCCCTGGCGTGGGTGACGGTCGTTTCGG

TCCTGTTCGACGTTCTCGACGGACCGCAGTGGGCGTTCTACCTCTGTATG

GGTGCGGGCGTCGTCGCGTACTTCGGCTTCTTCGCGTCGGTCGAAGCCGC

GAGCGAGAAGCGCTGACGCCGGATTCTCTCTCGGACTATCTTCTCGTCCT

GACTCGGAACGGTTTCGGGTTCGCGCTCGCCGACGCGCTCACTCGTTCGT

CGCCGTCGGTGACGGCGGGTCAGATATCGTCAGCGCCGCGCCGACCGGTA

CCCACGCGACGAGCGACATCACCGTTACCAACGCCAACACGACTGTACTC

ACCCGCTGTCGCGTTATCCCGACCCCCCTCGTCATCGAGTGTTCGCCTCT

CTGTCCGTCCTGCCGTGGCCCCTGATTCCCTCAAATATCATACTACTGCC

AAATGCGGATTCACGTCCGATAATTAGGCGATACAGACTGTCTGGAATAT

CATATAATAATATCGAATAAACACAACTCTTCAAATATCAGTACTTCACA

AAGCGTCCTCGGCTAGCTGTTTCTGAAGCCTGAGTTCTGTGACCGAGCGG

TTGCACTGGCGGTCTCGACGAGAGAATCTTGGGCGAACGGACGGAGAGCT

GTCGCTGTCGGCGGCGGCTGCCGCCGACGCGACAGCGTCGCCACTCCCAC

CGTTGGCTAGCCCGGTCGGGAGTTACCGGTGGAACCGGTCGTCCGGTGGC

CGGTTCGCGGGGACGGCCCGACGCGTCGCGAGGGCCGTCCACGCTGCCCG

TCGGATCATGTTGATGAACTCCTTGAATGGCCACTGCCAGAGGCGACGCC

CGCCTCGGCGGGGCGTCGCCACGTACTCCCAGTGCAGATACCGCCACACG

TTCTGTAACAGCAAGCTCACCACGACGTACAGCAGCCGTACGACCGGATT

TTGTGTCGAGGTCGTCGCGATACTTTGCTCGGAGAGTCGATAGCTTGCCT

CGATACCGAAGCGTTTCGCGTAGTGGTATCGAGCGTCCCGTGGTGAGTCG

ATGAACGGCGCGTCAGCGGCGTAGCCGTGACGCGCCACCCCATGTTCGTC

GTACCGTCCGTTTTGGTAGGTACAGTCGATGTAGACGGGAAACTCGACGG

TCCAGCTGTGACCGTCGAGTTTCGCTGTCAGACTGTGCTGAATCACGCGA

CTCCATCCTTCTGAGAGTTCTCGCTTGATCGTCCGTCCCCAGCGGACGAT

CGGCATGACGTACGCGTGGTTGTGCGCCTGAAGCAGCGTCAAACACTTGC

TGTCGTAGAATTCGCGGTCAAGATAGACGGCCTTGACACCGAGGTCAAGG

CCGTCGAGAATACCGAGGAACTCTGCGAGGACACTGCTGGCGGTGTCGCC

GTCTTCGAGACGGCGCACCGCCAGCGTGTAGCGTTTGTTCTTCACGCGTG

CGTACAGTGTCGCGTACGCGTGGAACGCGGTGGTTCCACGCTTCGCTTGT

GAGTGATACAGGCCGTCTGTATCGTCTTCGTCGCCGTAGTAGGGCCGCAG
[truncated: 3,216,176 more chars]
