# Supplementary material for: Mutations Affecting HVO_1357 or HVO_2248 Cause Hypermotility in Haloferax volcanii, Suggesting Roles in Motility Regulation
Source: Genes (Basel). 2020 Dec 31;12(1):58. doi: 10.3390/genes12010058 (PMC7824242; doi:10.3390/genes12010058)
Supplement: Supplementary file 1 [file genes-12-00058-s001.zip › genes-12-00058-s001/genes-1028798-supplementary/Collins et al. 2020 Supplementary Figures 2/File S2 Hfx. volcanii H295mod1 pHV3 reference sequence H295mod1 of plasmid pHV3.docx]

**Supplemental File 2: *Hfx.* *volcanii* H295mod1 pHV3 reference sequence H295mod1 of plasmid pHV3**

>Hfx_volcanii_H295mod1_pHV3 reference sequence H295mod1 of plasmid pHV3 (437910 bp)

CTGTGTCTTCCTCGTGACCGCCTCGGCCGTTACAAACGCGAGGGTGGTGT

CTGACGGTCTTACTTGCGCGATACTACTCGTTGATCGTCGAATCCAAAAC

CGGGTCCGAAGTGCCGTTCAAACGGCCGTATTTGTCGCTGTTACAAACGC

GAGTCCACCCCTTCACATTTATTACGGGTGAGTATGAATACCGAACTCAC

ATGACATCGGACGAGAGCGAGGGTGGGACCCGCGACCCGCTGTTCCGCTA

TGACCAACCCATTTTCGCGAACGAGGATATCCTCAAAATCTCTCATCTCC

CCGGTCCCGACAAGATTGTCGGCCGCGACGAGCATATGTCGAAAGTCGCA

CAGGCGCTCAACCCCGCCATCTTCGGACGCGAACCGACCCATCTGTTCAT

CTTCGGCAAGACCGGGTCGGGAAAGACGCTCACCGCTCGCCTCGTGAGCG

AGCGACTCCAACACGAGGCGGTCCGCGAGGACGTCGACGTCCGCATCGCC

GTCATCGACTGCGGCGAACAGCACACCGAGGCCTCGGTCATCAAGACGCT

CGCCTCGCAGGTCAACGACCCGTCGAAAAGCGGGATGACGATTCCCGAGC

GCGGGCTGTCGACCGGCGACTACTACAACCGCCTGTGGCAGGTGCTCGAC

ACCTGTTCCGACGTGACCATCGTCATCCTCGACGAAATCGATATGCTCCG

CGACGACGAGGTGCTCCGAAAGCTCTCGCGCGCGGGCGAGAATCAAAAGA

TAGTGGACTCGCGCATCGGCATTATCGGCATCTCGAACAAAATCGACTAC

CCCGAGGAACTCACGGAGCGCGTCAAATCGAGCTTCGCCCACGACGAACT

CGTCTTCCCGTCGTACGACGCGAACCAACTCCGCGAAATCCTCGAAAACC

GGAAGGACGCGTTCAAGCCCGGCGTCCTCACCGACGACGTGATTCCCCTC

GCGAGCGCGCTCGCCGCCCAGGAACACGGCGACGCCCGGAAGGCCATCGA

CATCCTGCGCAACGCCGGCCGAATCGCGCGAAACGAGAGCGCCGAGACCG

TCACCGACGACCACGTGCGGGCGGCCAAGGAGAAAAGCGAGGCCGACCGC

TTCAGCGAACTCCTCGAAGGGACCGCCACGCAGTCGAAGGCCGTCCTCTA

CTCGCTGGTCCTCCAGACCGGCGGCCGGAAGACCGCCGAGATTACGACGA

ACAAGATTTACCGACAGTATCTCACCGTCGCCGACGACCTCGACATGGAC

CAACTCTCCGAGCGCCGCGTGCAGGAACTCCTGCAGGAACTCGACTTCCT

CAACGTCATCCAGTCGGAGGTCCGCGGGCGCGGCCGCGGACAGGGCGTCC

ACGGGACCCACCGCCTGCTCGAGGACCCGGACATCGTCAAGCGAGTGCTC

CTCCGGGACAGCCGTATCTCCACGCTCGAATAGCGACGCACGGCACGCGA

CGCCTGCGTCCGCACGGGTCTCCGATGCTGTCGGCCGCGTCGGAAAAAAG

CTACCGCACGAACCCTTAACACACGTCCGTCTCTGTCGATAGACGACCGA

AACCATGGACGAAGAGTTACGCTCGTTGCTCACGAGTCTCGTCGAAATCG

AGACCGAGAATCCGCCGGGGGACGAGGCGGCGGCGGCGCAGTTCGTGTCC

GACTGGCTGGACTCCCGAGGCGTCTCGGCGACGCTCGTCGAGGAACCGTT

CCCCGACCGACCGCAGGTCGCGGCCCGCGTCGGCGACGGCGAACCCTCGG

TCGTCCTCAACGGCCACCTCGACGTGGTCCCGGCGGGCGACCGAGACCAG

TGGACCCATCCACCGTACGACCCGACCGTCAGAGACGGCAGACTGTACGG

TCGCGGGAGCGCGGACATGAAGTGCGGCGTCGCCCTCGCGATGCTCGCGA

CGGTCGAGTTCGCCGACGCGTTCGAGTCCGGCGCGCTCGACGGCTCGCTC

GTGTTCCACGCTGCCGTCGGCGAGGAGACGGCCGAACCGGGGACGAAGAC

GCTCCTCGAACGCGGCTACGACGGCACCTACGGCGTCGTGCTCGAACCGA

CGGCGTTGCGGACCGCGACGAGCGCGAAGGGGCTCGGTTGGTACGAGATT

TCCGTCGGCGGCGACCCGTCGCACGCGAGTCGGCCCGACGAGGGGGACAA

CGCGATTGGGAACGCTCGTCCCGTTCTCGACGCGCTCGAAGCGTACGACG

AGGAGATTCGGGCACGCTCCGACTCGCTCGTCGGTCGCCCGACCGCGACG

GTCACGCGGTTCGAGGCGGGGACGAAGGAGAACGTCGTTCCGGAGAGCGC

GACCATCACGGTCGACAGGCGGTTCGTCCCCGCTGAGACAGTCGAGGAAG

TCGACGCCGAAATCGACGCGCTGCTCTCCGACGTGACGGCCGAACACGAC

CTGAACGTCGAGTGGACGCGAACGCGAGTCTACGAATCGGCGGCGGTTCC

GACCGACAGCGACATCGCGGCGCGCTTTCGGGACGCGGCCGCCGAGCGGG

CGGATATCGACCCCGAGCCGTGGGGGATTCGGGCGGCGACCGACGTTCGG

AACCTCGTCAACGACGCCGGGATGGACGCGATTACGTGGGGGCCGGGGTC

GATGGCGCAGGCCCACGCCTACGACGAGTACATCGAACTGTCGGAGGTCG

AAGCCGGGTACGACATCCTCCGCACGGCGCTCCGCGGGCTGTTCGAGGCC

GGCGCAGACTGACACCGACTGTCGGCTGACGGGCTGAAAAAACGTGACGC

CGCGTTCGGCTATTCGACGAGTTCTTCGAACCGCTCGACCGCGGCGCTCG

CGACCGCCATGTCTTCGGACACCTCGCCGCCGCTGACGCCGACGGTCCCG

ACGAGTTCGCCGTCGACTTCGAGCGGATAGCCGCCGCCGAAGACGACGAG

TCGATTCTCGTCCGTCGTCTGGAGGCCCCACAGCGACTCGCCGGGCTGGC

TCCCCTCGGCGAGTTCGTGCGTGGGCATCTGCAGCGCGGCCGCCGTGTAC

GCCTTGTTTCGAGAGATGTTGACGCTCGCCAGCCACGCGTCGTCCATTCG

GTGCTGTGCGATGAGGTTACCCTCCGGGTTCGCGACCGTAATCACCATTC

GCAGCCCCATCGACTCGGCTTCCGCTTCCGCCGCTTCGATGAGTGTCTTC

GCCGTCTCGAGATTGATTGCATCGGACATATCGCTTCGTCGAAACCGGTC

TCGACGGCCCTACTTATACGTGCGCCGCGGGTCTCGAACGAGCGGCCGAT

TTCGCTCCTCTCTCCCGATTTCGGGCGTCTGAGTGACCCTCGAACGACGC

TGACGGGAGAACAACAACTATTGGTTCTCTTATACTTATTGTCAACCCTC

GGAATCGGGTCGAAGACGCATCGAGAAGACGTTCGGTTCGAGGCGAACCG

ACCCGATAGCCGGTCAGTCGTACAGCGGGTACTCGGCGGTCAGTTCGTCG

ACCTTCTGGTCGACGAACCCCTCGACCGACTCGTCGTGCGGCGCGTCGAC

GATTTCGTAGATGAGGTCCGCGACGGTCCGGCAGGCCTCCTCGTCGAAGC

CGCGGGTCGTCAACGCGGGCGTGCCGGCGCGAATCCCGCTCGGGTTGAAC

GCCGAACGACTCTCGCCGGGAACCGTGTTGGCGTTGAGGACGATACCGAC

CTCTTCGAGCGCCGCTTCCACGTCCTTGCCCGTCGTGTCCGGGTGCGACG

GCCGGAGGTCGACAAGAACTAAGTGCGTGTCAGTCCCCTCGGAGACGAGT

TCGAGGCCGCGTTCCTGTAACCGCTCGCCGAGGGCGGCGGCGTTATCGAC

GACCTGCGAGGCGTACTCCTCGAACTCGGGGGTTAGCGCCTCGCCGAAGC

CGACGGCCTTGCCGGCGACGTTGTGCAGTATCGGTCCGCCTTGGAGGCCG

GGGAAGACCGCGGAATCGACGTCGTCGGCGTGTTCCTCGTCGCACATGAT

GATGCCGCCGCGACCCGACCGAATCGTCTTGTGGGTCGAGCCGGTGACGA

AGTCCGCGGTGCCGACGGGCGACGAGTGTTCGCCCGCGGCGACGAGGCCC

GTGATGTGGGCGATGTCGGCGAGGTGGTACGCGTCGACCGAGTCGGCCGC

GGCCTGTACGCGCTCCCACTCGACCTCTCGCGGGTACGCGGAGTAGCCCG

AGATGATGATGTCCGGGTCGAACGCCTCGGCTTTCGCGGCGAGTTGGTCG

TAGTCGACGTAGCCCGTCTCGGCGTTGACCTCGTACTGTTCGACCTCGTA

CGTCTTCCCCGCGACGTTCACCGGGTGGCCGTGGCTCAGGTGGCCGCCGT

GGGTCAGGTCCAAGGAGAGAATCTTGTCGCCGGGGTCGAGCACGGCGAGG

AACACGCCCATGTTCGCCTGCGAGCCCGAGTGCGGTTGGACGTTGACGTG

CTCCGCGCCCCAGAGCTCCTTGGCGCGGTCGATGGCGAGCTGTTCGACCT

CGTCGGCGTACTCACAGCCGCCGTAGTACCGGCTCCCCGGGTAGCCCTCG

GCGTAGTTGTTCGTCAGTTCCGAGCTTTGGGCCTCCATGACCGCCTCGCT

GACGTGGTTCTCGCTCGCTATCATGGCGAGCGTATCGTTCTGTCGTTCGC

GTTCGCCCGCCAGCGCGTCGGCGACGGCCGGATCGGTCCCTCGAACGGTG

TCGTAAGGCATCGTGGGTGCATCTCGACTCTGCGGTATAACGCTTCGCCG

TCCCCCGAGACGAGGGCGGTACGCCGAACCAGAACCACAACGTGACAGGA

CGCGCACGTTCTGTGTTCGGGTCGATGAGCGCGGTGAGTGCCGCTTGGAT

ATCTTCGAAAGATATCCTGAATTGGGTTCGCTCACGGCCGAAATCGACGT

ATTACGTGTTCAGTTCTCACCGAGACGAGTACCCACCGGCGATAGCTCTA

TATTGGAACTTCGTCACGTCTCTCACATACCGTGCCAAGAACTATCACAG

ACCACGGAACGTCCCGAGGGGACACGCGCACGCCCCGGCGAGCCGTTCGG

CCGACCGCCGAGGTGAGCCGCCGATGAGTGAGGTCGAGTCCCTCCCGGTG

CAGTACAGCGACATCGAGCGCGCGAGCGAGCGACTCGAAAACGACCGCGT

CGTCAAGCGGACGCCGATAGAGCGGAGCACCTCGCTCGGTGACCTCGTCG

GCGCGGACGTCTACCTGAAGATGGAACACCTCCAGTGGACGGGGTCGTTC

AAGACGCGCGGTGCGTACAACAAGATTCGACAGGATACCGACCGCGGCAT

CGACGAGTTCGTGGCCGCGAGCGCCGGTAATCACGCGCAGGGCGTCGCGC

TCGCGGCGACCGAGTGCGGGGCCGACTCCACGGTCGTGATGCCGGAGAAC

GCCCCGCAGACGAAAATCGACGCGACGCGCGACTACGGCGCGGAAGTCGA

ACTCGTCGGCGAGGACTTCCAGGAGACGATGGCGTACGCCCAGTCGCTCG

CCGAGGAGTCGGACGCCGAGTTCGTCCACGCGTACGACGACTGCGACATC

GTGGCCGGACAGGGAACGCTCGGCGTCGAGATGTACCGCGACCTCCCCGA

GATGGACACCGTGGTCGTCCCCATCGGCGGCGGCGGGCTCATCTCGGGTA

TCGCCACCGCCATCAAGCACCACGACCCCGAGATTCGCGTCGTCGGCGTG

CAGGCTGAGGGCGCGGAGACGGTCCACGAGAGCCTCGACAAAGGCGTTCC

CGTGACGCTCGACGACACCAACACCATCGCGGACGGCATCGCCACCGGCG

GCATCTCCGAGTTGACGCTCCGAACCATCGAGGAACACGTCGACGAGGTC

GTCACGGTCTCCGACGGGGAAATCGCGGAGGCCATCCTCCTCTTGCTCGA

ACGCGCCAAGCAGGTCGTCGAGGGTGCCGGTGCCGCCTCCGTCGCCGCCG

TGCTGAGCGACGACCTCGACGTCGAGGGCGAGACCGTCATGCCGCTTCTC

TGCGGGGGCAATCTGGACATGACGATGCTCCAGACGGTGCTCGTCCACGC

GCTCACCAGCCGTCGCCAGACGCTCCGTCTCCGGGTTCGAATCCGGGACG

AACCCGGCGAGATGGCGCGACTCTCCCGGCTCATCGCCGACCGCGACGCG

AACATTCACGACGTTCGCCACGTCCGCGCCGTCGACGCCCTCGACGTCGG

CGAGGCCCACCTCCTGTTCCGCATCGAGACCAAGGGAGCCGAACACGCGG

CGGCCGTCGTGGACACGATAGAGGACGCGGGCTACGCGGTCGACGACGTT

TCGGAGCCGAGCTAGCGCCCGGTTCCTCGGTCTCGACCGTTCTCGCAGCC

GAATCCCGAATGCCTCTCGATTTGATACGACGTGTGAGATGCGGCGGGAC

GCGGCTTGAACGACGAACGCACTGATGTCCTCTCCGCGAAGCGACTCACC

GATACCGACGAAGCGTCCGCTACGTCTCTCCGGCCGATTGCCGGATGCGC

GCCACGACGTCTTCCCGACTCACGTCGGGGTCGTCGTCGGTCGTGAGTCG

GCCCATCACGAATTCGAGCAGGTCGAGGTCTTGGAGCAGGTCGAGCGCGT

CCATGCGTTCGAGGTCGAGCGCCTTGCCGAACTCGTAGACGGTCTTGGCG

TCGTTGGCGGCGTCGGCGAGTTGGTCGATTGTGACGGACTCGGGGAGGCC

GATGCCGTCGGCGAGCGCCTCGGGCGACCCGTCGCCGTCCGCCGCGGGTG

CCGGTGGTCGCTCGACGCCCGTCGGGTCGCCGGCGAGCGACGTTTCGTAC

GACGACGGTTCGTGGACGCCTTGGTCGATCATGTACCTGCGGACCGTCTC

GTCCGTCACGTCCATCTCGAAGGCGTCGGCCATCCGCTCGAACGTCTCGT

ACCGCGCGTACACGGCTTCGAGGTACGTCGTGTCTTCGAACGCCGGCACG

TTCGGGTTCCGAACGGCTTCGTGCGGTTCGGAATCGGCGTCGTCGTCGGC

CGTCTCGGTTGAACTCGTCGTCGGCGCATCGGTCGCGACCGTGACGACGA

CCGCCGGGTCGCCGTCGAGCGAGACGTCCGCGGCGTCGACGGCGACGTGC

TCGCGGTCGCTCGGCAGCACGTCGTCGACGCCGAGCGTGAGCCCAATCGA

CCCGTCGGAGTCGAGCGACGCCCCTCGGATTCGAACGGGGATGTCGCCGT

CGGCGTCCGACGAGAGCGCGAACTCGACATCCGCGGACGCGCCCGAGTCC

GTGACCGTCACGGACGACTCGCGGACGCTCGCGGCGTCCGTCCGCTCGCA

CTCTTCGAGGAATTCGCCCAGCCGCCGAAGCGATGTACCCACGCTCATTG

TCCGTTTCGACGGACGCGCCGAGACTCCCTGAAAAGCGGTGTGGAATACT

GAGTCAGTTTATATAGCCCCACAGAATATGCGGCGAAAAGTATAAACAAA

AATCTAATGTTCACACTACTATATCGTGCCCACGAGAAACGCTTCTGTCG

CGAGCGTCGACGCGAGTTCGCGACCTTCAACCGAACCGGCAGTCCGGCTT

CCCCGGTGTCGAATCTCTGTATCCACGAGGTGAAGACACGATGAGTCTGA

TAACCGTTGCCGACATCGCGCACGACGACCTCGCGCTGACGCCGACGATT

CAGTCGGAGGCCGCGAGCGAGATAGACGTCATCTCTCAATCCGCGACCGA

CCCCGAGACCGGCCTGTTTTTCTTCGTCGTGAAGGGCGCGGACGTCTCGG

CGTTCGAAGCGGCGCTGGAGGTCGACCACACGGTCAGCGACTGGCAGCTC

GTCTCCGAGCGGGAGGAGTCGGCGGTGTATCGCATCGGACACACGCCCGA

CACGATTCTCCTCTCGCCTATCATCACGGAACTCGGCGGGCTCATGCTCG

ACGCGTGCAGCAACGACACGGCGGGCTGGCGGGTCCGGCTCCAGCTTTCG

GACCGCGAGGCGCTCTCAGAGGTGTGGGTGTACTGCGAGGCGAACGACAT

CTCCTTCGAGCTGAACCGGATGTTCCGACAGGACGGCTGGATGAACGGCG

AGCCGAGCAAGCTGACCGACGCCCAGCGCGACGCGCTCGTCGCCGCCTAC

GAACACGGCTACTTCGAGGAGCCGCGAGAGGCGGCGCTGGACGACCTCTC

GGAAGTACTCGACATCTCGCCGACGGCCGTCAGCGGACGCATCAGGCGGG

GGACCGCGGAGCTGGTCGAGTCGATTCTCCTCGACGAGTGACGGAATAAC

CCCCGAAGAAAGGCGGGAGTCAGACCGCGAGGACGTAGTCGTCCTGCGGT

TCGTAGATTTCGCCGCTCATCCGCAGGTCGCGGACGGCGTCGAGCGTTTC

GCCGATGGCGACGCCGTTGTCCGCGAGCGCCGCGGCGAGCCGCCCGAGTT

CGACGCCGTCGGGGTGGTCCTCACCGAGTTCGGTGAGCGCCGTTTTCACG

CCGTCGCTCTCGACGTCGACGTCGAACGCGACGCGCTCCCACTCGACGTC

GTGGCCGGTGACGTTCGCGTGTCGCCGGTACAGGTCGACGGCGTCGTTCG

GCTCCGTGACGGAGACGTGGAGGTCGCACGCCGGGCAGGTGACGACGCCG

GCGTGTGGTTTTTCGGCTAGTTTGGCACTCTCGTCGTCAGGCATTGTACG

CGTCCTGCGCGAGGCGGTGCAGTTTGTGGACGGTGTGTTCGTTCGGACCG

AGCTGTGCCTGCCCGAGCGCGCCGGACGTGAGCCCCTCGTACTGGCGTTC

GACGAGCTCGAAGTCCTCTTCTTGGAGCTGCCGGCTCGTCTGGACGAACT

TCTCTTCTTCTTCGGACAGCTCGGAGTCGCGGAAGTAGTAGTCCGCGATG

AGCTGGAAGCGCCCCTCGTCGATGGGGTCGATGATGTACGTGCCGTAGCC

GTCGGCGGTGCCGTACATGTTGACCGTGAAGTTCGGCCAGAAGTAGTAGA

ACTTGGCCTCGTGTTCGGGGTGGATGCGCATCTCGTCGTCGACGTCCTCT

TTGTGCTTGTAGTGAAGCACCCAGTGGTAGTCGTTGACTTCGAGTTCGGA

CTCCATGAGTTCGAGTCCCTGCACCCAGTCTTGGTGGTTCGCCTGACAGT

GGTCGCACTCCGAGTAGTTCCCGCTGAACGTCTTCCAGTTGCACTCGACT

TCGGAGACGTAGCGCCGGGCGTGTTCGTACTCACCGAGCGGCAGGTCTTC

GAGTTCCGTCTTCATCTCCCCGGCCTGTTCGGCAAGCGAGAGGCTCGGTT

CGTCCTCGAAGTTGACGAACACGAGCGGCCCGATGGCGTCGGTGTTGACC

TCCATGAGGCCGTTTTCTTCGGGGTCGAGTTCGCTGACGGCGTCGTCGTC

GAGGTCGGGATTCAGACTCGCCTCCTCGAAGCTCTTGGGGGTGCTCTGGA

GCGACCCGTCGAGTTCGTACGCCCAGAGGTGGTACGGGCAGGTGATGCGG

CTGAGGCTGCCGGGGTCGGTCATCGGCGTGTCGTCGAGCATCTTCGACCC

GCGGTGGGCACAGACGTTGTAGAACGCCCGCACGTCGCCCTCGCCGTCGC

GGGTGACGATTATCTGCTTGTCACCGATGGTTCGCGTGAAGTAGTCGCCC

GCGTCCGGGATGTGGGTCTCGTGGCCCGCGTAGACCCAGTACCGACCGAA

CACTTTCTCCTTTTCCATCTCGTGGACCTCCGGGTCGGTGAAGTACCTCG

CCGGCAGCGCGTTCGTTTCCGCGGTGATGTCGGCACTTACCGCACCGATC

TCGTCACGATTGTTATTCCACCGTGTCATGGGAGGAAGAAACACACATCT

GTGGAAAAGGATTCGTCGGAAATAGTGGACCCGTATAAGTGGCGGTGGAT

ATCCGCGAGCGGCCGCCGTTCGGCGGCGAGCGCCGCTCTCGGCGGGCCGA

GCGTCGTTCCTCTGAAAAAGGGGCTCCGTGATACAGGTCAAGAGTCAAGG

GGGGACGTTCCGAACCGCCGGGCACAATGAGTGTCCGCGACGACCGATAC

GATGTCGCCGTCGTCGGGGTCGGCGGGATGGGGAGCGCGACCGCGTACCA

CCTCGCCGACCGCGGCCTCGACGTCGTCGGTCTCGAACGCTACGACATCC

CTCACTCGCAGGGGTCGTCCCACGGCATCACCCGCATCATCCGCCGCGCG

TACTACGAACACCCGTCGTACGTCCCGCTCATCGAGCGGGCGTACGACCT

CTGGGACGACCTCGCCGACGAGACCGGCCGCGATATCATCCACCGAACCG

GCTCCATCGACGCCGGCCCCGTCGACAACGACGTGTTCGCGGGTTCGAAA

CGCTCCTGTGAGGAACACGACATCCCGCACGAGGTGCTGACCGGCGCGGA

GGTCAACGAACGATTCCCCGGCTACGAACTCCCCGAGGACTACCGCGCGG

TCTATCAGGAAGACGGCGGGTTCGTCGTCCCCGAGCAGTCGATTATCGGC

TATACCGAAGCGGCACAGGCCCGGGGTGCCGAAATCCGCGCCCGCGAACG

GGTCGAAGACTGGGCGGAGACGAACGACGGCGGCGTGCGCGTCCGCACCG

ACCGCGGCGTGTACGTCGCGGACGCGCTCGTGCTCGCCGCGGGCGCGTGG

AACTACAAACTCGCTGACGCGCTCGACGGCCTCGCGATCCCCGAGCGACA

GGCGCTCGCGTGGTTCCAACCCGAGTCGCCGGCGACGTTCGCGCCCGAGG

AGTTCCCCGTCTGGAACCTCAGCGTCCCCGAGGGTCGGTTCTACGGCTTC

CCGGTCCACGACGTGCCGGGGTTCAAACTCGGCAAGTACCACCACCGCGA

CGAGGAGGTCGACCCCGACGACTTCGACCGCGAGCCGAACCGCGCCGACG

AGGCGATACTCCGCGATGTCACGGAGAAGTACTTCCCGGAGGCCGCGGGG

CCGACGATGTCGCTCGCGACGTGTATGTTCACGAACTCGCCGGACGAGCA

CTTCATCCTCGACACGCTGCCGGACCACCCGCAGGTCGCCGTCGGCGCGG

GCTTTTCGGGACACGGCTTCAAGTTCGCCAGCGTCATCGGCGAAATCCTC

GCTGACCGCGCCGGCGACGGCGAGAGCGACCTGCCGCTCGATATGTTCTC

GCTCGACCGGTTCGAGTGACGACGGGCGGTCGAGCTTGCCGCTCGTCAAC

CGATTTGACGATATAACTTCCGTAAGTTTTCTTCATTTGCGTATAAATGC

CTCTATTATCGAAGCCGACTTGTTATGATGTGTAACACCAATGGGTTGGT

TGAATCGTGTGGGGTGTGGACTCGTGTCACGGTCGGTGCGGACGCGTCGA

ATCCGACGCGGGTCGTCTCTCGACGGTGACAGTGGGTGGCCCCAAAGCGG

GGACAACGATATGACCGAACACGAATTCATCTGGCGGTCGTCAATGGAGG

CTGTATCGAATGGCGAATAGCAACGACTCGACCGGTCGGTCGACGAGCGA

GTTACAGGAAGACCTGTTTTATCCCGATATGGAGCGCGAACCCGGTGACA

GAAACATCCAGGGGTTGGGGTTCGACATCCATCCCGTCGTCTTCCCGGCC

GCGCTCTTGATAATCGGCGTCTTCGTCGCTGCGACGCTCATTCTCGGTGA

GCAGGCGGCGAGCGTCTATTCGGGTATCAGGGGCTTTTTCGAGGGGAACT

TCGGGTGGTTCTTCCTCCTGTCGGTGAACCTCTTTATCGTGGTCTTGGCG

TACTTCGCGCTGAGTAAGTACGGAAAGATAACCCTCGGCGGCGTCGAGGC

GGAAAAGGAGTTCAGCGACTTCTCGTGGATGGCGATGCTGTTCAGCGCGG

GCATGGGTATCGGGCTCATGTTTTACAGCGTCTCGGAGCCGCTGTACTAC

TTCAGTAACGTCCCCGGCTTCTTCGACGCCGAGGCCGGGTCCGGCGGTGC

GGGTATCGCCGCGATGACGCAGACGTTCTTCCATTGGCGGTTCCACCCGT

GGGCCATCTACGGCCTCGTCGGCTTGGGACTCGCGTTCTTTTCGTTCAAT

CGGGGGCTGCCGCTCACGTTCCGGTCGATTTTCTGGCCGCTCCTCGGCGA

GCGAATCTACGGCTGGCCGGGCCACCTCATCGACCTCGTGGCGGTGTTCG

CGACGCTGTTCGGCCTCGCCACGTCGCTCGGCCTCGGGGTCGCACAGATA

AACACCGGGCTGTCGTACATCCTCGGCGGGAGCATGCTCGGCGTCCTGAG

CGTTCCGAACAACACCGGGATGCAAATCGCGCTCATCGCCGTGATTACAC

TCATCGCTACCGCCTCTGTCGCGGCCGGCCTCGAAGGCGGTATCCGGCGT

CTGAGCACGGTGAACGTCTTTCTGATGTTCGTGCTGTTGACGTTCGTGCT

CGCCGCCGGCGCGACGATGAGCATCTTCGGCGCGTGGGTGCAGGGCCTCG

GGAACTACTTCAGCGACTTCCTCTCGCTCGCGTTCTTCACCGGGACGCTC

GGCGACGGCGCGTCCACCGTCAACGCTTGGACGGTGTTCTACTGGGCGTG

GTGGATTGCTTGGTCGCCGTTCGTCGGGATGTTCGTCGCGCGCATCTCGA

AGGGGCGGACGGTCCGCGAGTTCGTCTTCGGCGTGTTCGTCCTGCCGTCG

CTGTTCTCGACGGTCTGGCTCTCGGCGTTCGGCGGGAGCGCGCTGTTCAA

CTCGCTTCAGGGTAACGGCGCGGTCCTCGCGACCTACAACGAGGCCGGCC

AGACGGTCGCCATGTTCGCGCTCCTCGAACAGTTCCCGTTCAGCGCCGTC

TCGGGGCTGCTCGCCGTGACGCTCATCGTCACGTTCTTCGTGACCTCCTC

GGACTCGGGGTCGCTCGTCGTCGACCATCTCACGTCGGGCGGCAAGCACA

ACGTGCCGCGGACCCAACGCATCTTCTGGGCGGTCATCGAGGGCCTCGTC

GCGTCGCTTCTCCTCTACGGCGGGGGTCTCGGCGCGCTCCAGACGGCGGC

CATCGCGACGGGGTTCCCGTTCGCCGTCGTCCTCGTCATCATGTGCTACA

CGGTCTATCTCGGCCTCGACAAGGAGTACGAACTCCTGCAATCCGAGGAG

TTCGCCGAGCGAATCGAGGACTTGGCGGACGAGGACATCGACATCGACGT

CGGTCGCTCTCAGACGTCAGTCGTGACCAACGTCCGGCAGAACCCCGGCA

CGACGGACGACTGACCGCGGAGCCACCGAACCGATGACTCGACGCTATCG

ATGAACGCGCTCGCTGTCGTTTCGGCGGCGTTCGCGGTCTTCCTTTTCGT

CGTTGCACTGTTCGCGATGACGGCCGGTGAGCTACGGGGCGCAGGTCTCG

CGTTCCTGAGCGCCAGTCTCGTCATCTACCTCAGAGAGAAGCACCTCGTC

GGCGAGTGACTTTGTCGGCGGGTGACTCCGGTCAGTGCGCTCCTCGTCGG

CGCACGGCTTTCGTCGCCGCGCGTGTACTCGTCGTTCTCTGACGCTGATT

CTCGTCGGACCACGCGACGCACCCGTGGTGAGGCCCCTCCCTCGTCACCC

CCCGGTATCTATACACCTCAGATAGTGGACCCGGAGAGATATCCGAAGGC

CGCAGTCACTACTGTTTGGTACATGAACACATGGTCTACGTTTCCCGAAC

GCGCCGGAACGGTCGTCGTCGGGGCCGGCTGTGTCGGTTGTAGCGCGGCG

TATCACCTCGCCGAGTCGGGTCGCGAAGACGTCGTCGTCCTCGACCGGGG

TCCGCTGTTCGAGACCGGGGGGTCGACCTCTCACGCCCCCGGACTCGTGT

TCCAGACGGCCGGGAACAAGCTGATGACGGAGATGGCGTCGTACACGCGC

GACCTCTACGACGGGTTCGGTGACTTCCGGACGTGCGGCGGCATCGAAGT

CGCCTACACCGAAGACCGCTGGGACTTCCTCAAACGCAAGCGCGAGTGGG

GTCAGTCCTACGGCATCGACGAGGGCCAACTCCTCACGCCCGAGGAGGTC

GAATCGCGCGTCCCGCAGATAAACGCCGACGTCATCTACGGCGGCTACTA

CGTCCCCTCGGACGGGAAGGCCGACGCCGTCAGCGCCGCCGAGTCGATGG

CGGAGGAGGCGCGCGAGGCGGGCGTCGAGTTCTACGGCCACACCGAGGTC

ACGGACATCGACACTACCGACGGCAGCGTCGACGCGGTCGAAACCGAACA

CGGCCGCATCGAGGCCGACGAGGTGCTCGTCGCCACGAACATCTGGGGGC

CGCTGTTCGGCGAGATGGTCGGCACGGACATCCCGCTCGTCCCCTGCGCG

CACCAGTATCTCGTCTCCGACGAACTGGACGAGCTCGAAGGCGCAGAGCG

CGAAATCGAACAGCCGCTCCTCCGGCATCAGGACCACTCGCTGTACTACC

GCCAGCACGGCGAGCGCTACGGCATCGGCTCGTACAACCACGAGTCGCTG

CTGGTCGACCCGGCGGATATCTACGGGCCCGACAAGCTCGACGACCTCGG

ACTCGAATACCCGTCGCTCCGGGAGTTCACGGAGGAGCATTTCTACGAGA

ACACCCACCCGGACCACGACCGGTCGGCGTTCGACGCCGCGAGCGAACTC

GTTCCCGGCTTCGAGGACTGCGAGTGGACCTCCGCCATCAACGGCATGTT

CTCGTTCACGCCCGACGGCATGCCCATCCTCGGGCCGGACGAGGACACCG

AGGGTCTCTGGTGGGCGCTCGCCGTCTGGGTCACGCAGTCCGGCGGCGCG

GGCGACATCGTCGCCAGCTGGATGGAAGACGGGACGCCGCGGCTCGACGG

CTCCCGCGTCAACGCCTCCGGCGCGCACGTCTCCCGGTTCCAGTCGCACA

CGGGCAGCCGAGAGTACACCTACGGGCGCGGCGCGCAGCAGTACCGCGAG

GTGTACCAACTCGTCCACCCGCGCGAACAGCCGGACGGACAGCGCGCGCT

CCGTCGGAGCCCGTTTTACGACCGGCAGGCCGACCTCGGCGCGGAGTTCT

ACGACAGCGACGGCTGGGAGGTCCCGCAGTGGTACGAGTCCAACGAGACG

CTCCTCGAAACGTACGACGTGCCGGACCGCCCCGACTGGCTCTCGCAGAA

CTGGTCGAAGGTCCAGGGCGCGGAACACCAGCATCTCCGCGAGAAGGTCG

GCATGGTCGACATGACGACGTTCACGCCCATCGAAGTGTCGGGCCCGGGC

GCGCTTGAGTTCCTGCAAGGGCTCCTGACGAACGACATGGATGTCACGCC

GGGCCGGATGCGCTACTCCGCGATGCTCAACGAGGACGGCGGCGTACTCG

CCGATCTGACCGTCGCCCGCCTCGGCGACGAGCGGTTCGTGCTGTTCACC

GGGGGCGGAAGCTCGGCGACGCTCCACTCGCGGTGGGTCACAGAGCACGC

GCCCGACGACGGCTCCGTCGACGTGACCGCACACGTCTCCAGTCGAACCG

GTATCGGCGTCTTCGGCCCCGACTCCCGGAAGGTGCTTTCGGACCTCGTC

GAGGCCGACCTCTCGAACGACGAGTTCCCGTTCTACTCCGCACAGGAGAC

CTACCTCGGTAGCATCCCCGTGACGATGCTCCGACTCTCGTACGCCGGCG

AACTCGGCTGGGAAATCTACGCCCCGACCGAGTACGGAAGCCAGCTCTGG

GACGCGATTCGCGAGGCCGGCGAGGAGTACGACATCGCGCCCGTCGGCTG

GGCCGCGCTCGACTCGACGAGCATGGAGAAGGGATTCCGCCTGTGGGGGA

CGGACCTCACGCCCGAACACAACCCGTACGAGGCCGGCATCGGCTTCGCG

GCCGACCTCGACACCGACTTCGTCGGGAAGGCGGCGCTCGTGGCGGACAA

AAACGACGACAGCCCGAGCGACCGCATCGTCCCGATAACGCTCGACGAGG

AGGAAGCCGTCGTCGACGCGGGCCACCCGGTGTTCGTCGACGACGAGGTC

GTCGGCTACTGCTGTCGGGCCGACTACGGCTACACCATCGACGCCGGCAT

CGCGTACGCCTACCTCCCCGAGGAGTACGCCAGCTCGGGGCAGGACGTCG

AAATCCGGTACGAGGGTGACGCTCACCCCGCGACGGTCCGGTCCGGGCCG

CTTTTCGATCCCGACCGCGACAAGATGATTCGGTAAACGCCGGATTTCGT

CGCGTCACCGCCTCCGCCACCGTCTCCGCCACCGTCTGCGCCTCTGCCTC

CCCCTCAATTTATCGGCGCTGTCGAACCGCAGTCACCGATTGTCCGCCGA

GGCCGTGCTGAACTCGGCAGATTTGTTGGGGATCGGGACTGCCTGAAAAA

TACTTATCCTCCCGCTGTCACTCGTCTTCGACGGTCTTCAGGTCGAGCCG

CGGGGCAAACGACTCGTACGTCTCGTCGCTCGAAACGATAGTGTCGCCAT

CGGATTCGACGAGATGGAGCGCATCGAAGGGTGTGAACCCGTGGTCTTCG

ACGTAGGTCGCGGCCGTGAGAACCGTATCTACGTCCCCACGCACTTCCAA

CAGCGCGGCGACGTTCGTGATGACCCGCTCCGTGTCCCGCTCTTCTCGGT

ACGCGACCATGAGGAGTTCGATGAGCGTGAACTGCGACGTCCACAGTTCG

TCTCGATGGTTTCGGTAGACCGCCTCTGCGGCGTCGCCCAGCCAGTCTTC

GTCCTTGATGAGCGCCAGTAGGAAGTCCGTCTCGGCTACATTCAGCGGCC

GGCCTCGTCGAGCGCCGCGTTCCGCGCTCCGCGACGGAGTTCTTCGGCCG

TCTTCTCGACGTCCGCGAACTCGGTGCGGAGCGCACCGAGCGGGTCGTCT

TCGATTGGAATCAGCTTGATACCGTCGTGCAGTTGGACGATGTGGTAGTG

TTCGCCGTACCGCTCTCTGATCTCTTTAGGGAGCGTGAGGCGTCCGCGAC

CGTCCAGCGTCGCTTCGGACATACGGTCTACTACGATGGGTAGAAAGAAG

AATTTTCCCCATAACCCGATTTGCCACCCACTCGGATAGAACCCTCTCGT

TCCAGAACGCTCCCGTTGTCCGTACCACTTGACTTCTGTCCGGCGCTCCG

AGTTTCGACGGAGGTGGATTATCCGAATTATTCGGAATCTTCGTCGTCGA

CGCCGCGCTGTTCGAGGACGCGCTCGACGATTTCTTCGGGGTGTTCGGTC

GCCACGGCGAGCGCGGCGTTGTGGAGTTCGCTCTTCGTGACGCCCTTGAG

GCCGTGGTCGCGGGTCAGTTCGCGCTCCACGTCGTAGGTGATGATGTCGT

CGAACTCGTCGGCGACCGCTTCGAGCACGTACAGCGCCTTGTGGACGGTC

TCGTCGAACTCGAAGGCGGGGCGCTCGCGGGGGTCGACGGGTTCGGCGGA

CGCGGATTCCGTCGCTGACTCGTCGTCCGATTCGACCGCGTCGTCGGTGC

CGGCGTCTTCGTCCGCCGGTTCGGGTTCCGGCTCTGGCCCGGAGTCGGTC

TCGCCGCTTCCCATCGCGGCGTCGAGGTCGCCGAAGGGGTCTTCGCTCAT

CGGGCCACCTCGCCTTCTTCGACGATTTGCGCGAGTTCGTCGTAGCACGC

CAACTGGTCGCAGGTGCTGTCGTAGTCCCGCAGCGGCATGTGCTCCCGAA

TCGACGCGTCGATGGCCGACCGATGCCGAATCCCCGGCTTCGGCAGGTCG

CCGTCCCACTCGCCGTCGTCGATGGCCTCGAACACGTCCGGGTCGGTGTA

CGCGAAGTTCGGGAGCTTCGAGGCGATGTTCGGCTTCGAGACGAGCGCCT

CCACGAGCCGTCTCGTCGGGCGGTCGTGGTCGATTCGGTCCCGCAGGTCG

GTCGGCACGACCGCGAGGATTTCGAGGTCGAAGTGTTCGCGCGCGGGCGT

GATAAGTCGCTTGACGGTCTTTTCGAGTCCCGAGAGCGCCCCCGATTCGG

GGCGCATCGGCAACACGAGGTTCTGCGTGGCGTACAGCGCGTTGTCGTTG

AGTTTCCCGCGCGCCGCGGGGCAGTCCACGACGACGTAGTCGTAGTCGTC

CCCGAGAAGCGGGTCGATGACGCGCTGTTTGAGCCGCGCCGACCCCATCA

TCACGCCGCCGAGGTCCGACTCGACCTGTTCGATGCGGTCGTTCGAGGGG

AGGAGGTCGATTTCGAACCCGGTCTCCAAGATGAGCTCTCGCGGCTCCAA

CTCGGCGTCGTCGAGGAGCACGTCTCCGAGATGTGCCTCCGAGTAGAACT

CTTCTCCGAACCCGAGACCCGTCGTGGTGTGTCCGTTCGGGTCGAGGTCG

ACGAGCAGCACGTCGCTTCCCCGGTGTGCGAGTTCGCGGGCGAGGTTGAT

GGAGGTCGTCGACTTGCCGATACCGCCTTTGAGGAGACAGACGCTGACTG

CACGTGACATGATGGGGATATCCGTACTATCGAGATTCTCGGCGTAGTTG

AAATAATTATGGTTACTCCAGCTCTTTGAACAACTCACACACTCCAAATA

ATCTGAATAATCTAAACTCAGCGAGTTGATCGACTCGGTTCGCTCTCGCC

TCGACTGGCGGCCCGAATTGTCGCCGGCAGTAGCTATTTGGGCCCGTTGT

GTGCAGGTAAATTCGTGACAGATTGGTTGCCCGACCGAGGGCTGCGCGCC

CCCGAACTCTCGTCGCAGACGGTGACCCTCGCGGTCGCCGCCGCCTTGCT

CCTCGCGGTCGTCGGGCTCCACCCCGTCTCCGTCCTTCCTGTCCGGTCGC

AGTACGCGCTTTCGGTTTTCGTCGTCGCGCTCCTGCTCTGGTTGACGAAG

CCGGTTCCGTACGCGATTTCGAGTCTGGCCTGCGTCTCCCTGTTGTACGC

CCTCGGCGTCGTTGACACCTTCGAGGCCGCGGTGAGCGGCTTCACCTCCT

CGCTCGTGTACTTCCTCCTCGTGTTGTTACTCCTCGGCAACGCGGTCCGG

TCGGTGGGGTTGGACACCCAGTTAGCGAACCGACTGCTGTCCCCGGACAG

CACCCCCCGCGGGGCGTTTCGTTCCATCGCGCGGAACGTCCTCGTGCTGG

CGCTGTTCATGCCGTCGGCGATGGCCCGCGCGGTCGCGTTCATCCCCATC

GTCGAGGAGGTGACAGAGGAGCTCGACCTCCCGTCGGGAAGCGGGTTCGA

GCGCGCGACGTTCCTCCTCTTGGGTCACATCAACCCCATCGCGTCGATGG

CGCTCATGACCGGCGGCGGGATGGCGCTCGTCACCTCGGGCATCCTCGCG

ACGAGCGTCCGTCCGCTCTCGTGGGTCGAGTGGGCTGTCTTGATGGTCCC

ACCCACGGTGGCGCTCTACGTACTGGCCGCCGCGGCTGCCGGTCTCCTCA

ACACCGTCGACGCCGACTCGACTGTCGACCCCGGCACGTCGGGGATTGCG

GATGCGTCCGCCGACGGCGAGTTGACCCGCGAACAGGGACTCGTCGCGGC

GGTGATGGCCGGGACCGTCCTCGCGTGGGTCGTTGGGTCGTTCGCCGGAC

TTCCCGAGATTCTCCCGGCGGTCGCCGCGGTCTCCGTCCTCGCGCTTCCG

GGAGTCGACGTTATCGACGCCGACGACGTCAAGGGACTCAGTTGGGGCGT

CCTCTTTCTCATCGGCGCGACGCTGTCGATTCTGGACGCCCTGAAAGCGA

CCCAAACTATCGCGCTCGTCGTCGACCTCCTCGCGCGGTTCGTCCCGTTC

GAGGCGTTCAGTCACTGGCAACTCGTCGGACTGCTCCTCGGGTTCGCGGT

CACCGTTCGCGTGCTGTTTTCGACCGGGTCGGCCGCTATCGTGGTGATTC

TCCCCATCGTGCTGCGATTCGCGGAGACGTTCGGCGTGACGCGGCTCTAC

CTCGGACTCGCGGTCCTCCTCGTGGTGGGGTCGACGACGATTCTCCCGTT

CAACACGACTGCCGTCCTCGTCTCGCTGGACCGCGGACCGCTGACGAATC

GGGACGTGACGCTGTTCGGGTTCGTGACGATGGGCCTGTCGCTCGTCGTC

GTGACCCTCTCGTGGCTCTTTTATTGGCCGCTCGTCGCCTGAGTCGGCGA

CCGGCGCACCGACCCGTCCGCGCGAATCCAGGTTTCGGTGACGCCCCCCG

TTGCGACAACCACCGGGCTATTTGTAGGTGAGCGGTATCTGCCCGGTACG

GGTGAGTTCGCATCTTACGTGTTGAGTTTACGACGGCCCCGACCGGCGTC

ATGTCGAAGATAGACCGCCGTCTGCGAGACGCCCGGAGCATCGAGTTGGA

CAACGCGTTTTACGTCGAGGACGGGACGTGGATCGAGTCGCTCACCATCG

CCTCGAACGCCCCCTTCGACGTGGAGTCCCTCCTCGCCGACATCTCGGGC

GTGACCGTCTTCTACGACGAGGAGATTCCGACCGCCTCGACCGACGTGCA

GATACGTCGAGTGACGATTCTCGCCAACGAGTCGTACCCGTTCATCCTCG

GACTGGTTCTCAGACAGGAGACGATTCCGAACCGCATCGTCCTCCAAGAC

GGCGTCTTCGAGGTCGTCGCCACCGCCCAGGACTGGGACCACTTCAGGGA

ACTCGCCGACGAGATTCAGGAGACGCTCGGGGAGTTCGAACTCCGCTCCG

TGACGCAGGACGAAGCCCCGGGTGAACCGCTCGACAGCGGCCGATTAAAG

GAGGTGTTAATCTCTAAGCTGACCGACGACCAACTCGCGGTTCTGGAGAC

GGCATTCAACCACGGCTACTTCCACATTCCGCGGGAGACCTCTGAGACCG

AACTCGCCGAGGAACTGGGTATCGCTCAGTCCACGCTGAGCGAACGACTC

CGGACCGCGGAGCGAAATCTGCTCGAACTCATCTACGGTCCGCGACAGGA

GTAGCTCAGCCCTTGTTGGAGAATATTGCCGCATCTCGGCGACCGAGTAT

ATAAAGAACGATAGATATCGGGGCGTATCGGGAACAGCACCATGGAAGAT

ACTCCATAACAGGCTGTCGCAATGTCGGAGAAAAATCGTGATAGTTCGGG

TGTCGAACGTGGAACTGGCCTTACGCGACGCGACTACGTGGCGGCGACTG

CGACCGCCCTCGGCGTGACCGGACTCGCCGGGTGTACCGGTGGCGGCGGG

AGCGGGAACGGCGGGACTGACGGCGGGTCGAGCCTCACTCCCGACGTTCC

TTCTGGAACCCCGGAGACGGTCGAGACGAAGTACTGGCGCGAGTGGGAGA

CCATCGACGCCGACGAACCGCCGCTCGACTACAGCGCGACCGCGGGTGCG

GTGTTGGACCCCGTCCCGCTCGAATTTTCGAGCGAGGACGACCCGTGGAT

GCGGGAACACGCGCTCATGGTCCAGCGTGGGCTCGACGCCCTCGGCGTCA

AGACGCAACTCAACGATCGACCGCTGAACCAACTGTACGCCCAGAGTTGG

GACACGAAGGGGCTCGAAGCGATCATCTCGATGAGTACCCACGGTCCGGA

CCCGCAACGCGGGCTGGACCCGAACCCGCTTTTGATGCGGCGGAGCGAGA

CGAGCCTCTCGAACTACGACAACTACTACCACCCCGACCTGCAGGAGGTG

CTGACGGAACAGAGTCAGACGACCGACCGACCCGAGCGCGAGGAGCTCGT

CGCCGAGGCGCAGCGCATCTTCAGCGAGGACGTGGGCGCGCTCATTACGC

TGTTCCCCGATATCATCACCGCGGTCAACACGGAGCGCTGGAGCGGCTAC

GTCCAGACGCCGGGCAACGGGCCGACGAAGGACTCGTTCGTCTGGTCGGA

GGTGAACCTGCAACCCCAGACCGACGACCGGACCTACATCAAGGGCGTGA

CCACCTCGATGAACTCGCTGAACCTCCCGTGGGCCGCCGGCGGCGCGGAG

GCGAAGCGGCTGACGTTCATCTACGACGGCCTCTTCGACGCCACGCCGGA

CCTCGAAGTCGTCCCCGCGCTGGCGACCGGCGGCGGGTTCGTCGACGACA

CCACCGTCGAACTCACTCTTCGCGAGGGCGTCGAGTGGCACGACGGCGAG

CCGTTCACCGCGGAGGACGTGAAGTTCACCGTCGAACTGTACAAGGAACA

CTCCTCGACCAGTCAGACGCCGTTCTACGAGCCGGTCGAGTCGGTCGAGG

TGCTCGGCGACCACGAGGTTCGGTTCAATCTGAAGGGCCCCGACGCGGCG

TTCATGACCCAGCGCGTCGTTCGCAGCGTCATCATCCCCAAGCACAAGTG

GGAGGACGTGGAGAACCCGTCTCAGCACAACCCCGAGTCGCCCGTCGGCA

CGGGGCCGTTCACGTACGAGAACTGGGAGCTGGGTACTCGCTTCGAGGTG

TCGAAAAACGAGGGTCACTGGATGTTCGACGACGACTGGCGGGCAGACGT

GCTCGGCGACCAGGCCACGACCGGCGAGGGCGTCGACCGCGTCATCTGGA

TAAACGTCGGCAACATCGACTCCATGATCGGCTCGCTGCAGGGCGGCGAA

ATCGACGCCATCGGCACGACGCTCTCGAACTCGCAGGCCGACCGCGCCGC

GAGCACCGCCGGCGTCGAGAAGATGACCTCGGGCAACTTCGCGCCGCTCG

ACACGAAGCTGATGTTCTCCTGTCCGCTCGTTCGCGACAAGGAGTTCCGC

GTCGCTCTCGCCAAGTCGATAGATACCGAGGGCTTCGCTGCCGACGTGCT

GCAGGACCGCGCGACCGTCCCGAGCGGGGAGAACCCGATTTCGGACCTCA

CGCCGTGGCACACGTCGGATACCACCCACTACGAGTTCGACATCGACGGG

GCGAAATCGCTCCTCGAACAGGCCGGCTACACGCGGGACGACGACGGCAA

CCTCCGGTTCCCCAACGGGGACGCGTGGGCCGCCTTCGTCGAGCGTATTC

AGCCCGGGAACATGCACAAGCGCCGCGACGAGCTCGGACAGGCGGACTTC

TCGTGAGGTGACACGCATGTCGAACACTACTCACTCGTGGCGTTGGGGGG

TCCGACCGTGAGCGAGTTCCAGCGGTTTCTCGCAAAGCGGCTCGCCATCT

CCATCGTGCTCACCCTCGTGGCCGTCTCGGTCATCTTCGTCGTCTTGCGG

TTGCTCCCGGGGAGTCCGTTCGAATCCCTCGTCACCGCGGGCAACTTGAG

CCAGCAGCAAATCACCGAGATTCGAGCGATGTACGGCCTCGACCAGCCGA

TGTGGCGGCAGTACCTCAGCTACCTCCAGAGCATCCTGCTGTTTCAGTTC

GGCTACTCCATCCTGCGGAGCCAGCCCGTCTGGGGGGTACTGGCCCCGCG

GCTCGTCAACACGCTCATCCTGCTCGTCCCCGCGTTAGTGACGACGGCGG

TCCTGAGTTCCCTGCTCGGGATGTACGTCGGCTGGAACCGCGGGAGCAAA

CTGGAGAAGCTCAGCATCATCGCCACGACGTTCCTCCGGTCGACGCCGGT

GTTCATCACGGGCATCCTGTTCGTCATCATCTTCGCGTACAACCTCGAAC

TGGTGCCCGCATTCGGGATGCGCTCTATCAGCGCCTCGCCGGAGGGCTAC

GTCGAGACGTTCGCCTCGTTCGACTTCCTGCACCACTACATCCTGCCGTT

TACGGTGTCGGTGCTGTACTACAGCGGCGATTTCCTGCTGTTGGCGCGCA

ACGGCGTCGTCGAAAAGCGGGGGTCGGAGTTCCTCAAGCTCCACCGCGCG

AAGGGGCTCTCGGAGATGCAGCAGCTCGCGCGGGCGGGTCGGAACTCCAT

GCTGCCGATTCTGACCTACTTCGCGCTGCGTCTCGGGATGATTTTCCAGG

GACTCATCCTGCTCGAAGTCGTCTTCGGCTGGCCCGGCATCGGCCGCGAA

CTCGTGCTCGCCATCCAACAGCAGGACTACCCGCTGGTGCAGGCCGCCGT

CTTCATCATGGCGCTCGCGGTCATCGTGGCGAACCTCGTCGCCGACGTGC

TGTACGCCTACTTCGACCCGACCGTCTCGACCAGCGGAGGTGGTGCCGCG

TGAGCACCGAGACGAAGCCCGAAGCCGAACTGAAAAAGCGCGTCGAGGGG

GTCGCGGAGACGCTCCGCGACCAGTTCACGTTCCTGCGACGTGACCGCCT

CGCGTTCGCCGGCGTCCTCATCGTCGCGGCGTTCGTCTTCCTCGGCCTCC

TCGGCCCCGCGCTCGCCCCGCACGACCCCATCGAGCACACCGTCCGCGAC

GACGCCGGCTCGATGCTGCGGCTGTCCGAACCGACCGGCGCGGCCCCGTT

CGGGACGACCGCCTACGGGAAAGACGTCCTGAGCCAGTTCCTCGCCGGCG

CGCAACCGACGTTCATCGTCGGCCTGTTCGGCGGCATCGGGACCGGTGCG

CTCGGCTTTCTCGTGGGGCTCGTCAGCGGGTACTACGGCGGCTGGGTCGA

CGAGGTTCTCATGCGTCTCACGGACCTCACGTTCTCGCTGCCGTTCACGC

CGATGGCGCTGTTACTCCTGACGTTCGTGACGCCGAGCGTCTGGCTCATG

ACGGGTATCATCGTCGCGTTCCTCTGGAAGATGCCCGCCCGCGTCGTCCG

CTCCGAGGTCCTCTCCGTCAGAGAGCGCACCTTCGTGAAGTCGGCGCGCG

CCAGCGGCGCGAGCGACTTGCGGACGATGCTCTACCACGTCGCGCCGAAC

GTGCTCCCCATCGGCTTTCTCTACACGGCCTACGGCATCGCGTGGGCCAT

CGCCGCGCAGGCCAGCCTGGCGTTCCTCGGATTCGGCGACCCGACCGTCA

CGAGTTGGGGCCGGATGCTCCGACAGGTGTTCGAATCGGGCAACATGCGC

GTCGCTTGGTGGTGGGTGCTCCCGCCCGCACTCGGCATCGCGGCCATCAC

CACCTCGGTGTTCCTCATCGGCCGCGCCTACGAAGAAGTCATCAACCCGG

AGATTCAGACCGAACAATGAGCCTCCTCGATATCACAGACCTCACGGTCA

CGTACTCGACCGACAGCGGAACCGTCCACGCCGTCAACGACGTGTCGTTC

AGCATCGACGAGGGCGTCAACTACGGCCTCGCCGGCGAATCCGGCTCGGG

GAAATCGACGCTCGCCGAGGCGGTTCTCGGTCTCCTCCCCAGCAACGGCT

CCGTCGAATCCGGCAGCATCGAATTCCAGGGCCGGGACCTGACCGGCCTC

AGCGAGCGCGAGCGTCGCGACGTGCTCTGGGAGGACATCGCCTACATCCC

CCAGAGCGCGATGGACTCGCTCGACCCGGTGATGTCCACCGGCGCGCAGA

TTCGGCAGGCGATTCAGACCCACCGGAACGTCACCGACGACAAGGCGCGT

TCGCGGGTCCGAGAGCTGTTCGAAATCGTCGGACTCGACCCCGACCGAAT

CGACGACTACCCCCACGAGTTCTCCGGGGGGATGCGCCAGCGCGTGACCA

TCGCCATGGCGCTCGCGCTCGAACCGGACCTCATCATCGCCGACGAGCCG

ACCACGGGGCTCGACGTCATCGTTCAGGACAAGATTATCGACAAGATTCT

GGAGATACAGGACCGGATGGACAGCTCGTTGCTGCTCATCACCCACGAAA

TCGGCGTCATCGCCGAGACGTGCGACGAGCTTTCGATACTCTACGGCGGG

AAGGTCATGGAACAGGGAAGCGTCGACAACGTCCTCGTGAACCCCACGAA

CCCGTACACGATGGGGTTGAAGAACTCCTTCCCGGAGATAGAAGAGGGCG

GCCAGGACCCCGTCTCGATTCCGGGGTCGCCGCCGAACCTGAGCGAGGCA

CCCGGCGCGTGCGTCTTCAAGGACCGGTGTCCCTTCGCGACCGAGGAGTG

CGAGGCGTCCCATCCGGACCTCGTCGACCTGCCGAACCGTAACCACCGCT

CGGCGTGCCACCACGTCACCAAAGCCGCCCAGATGCGGTGCGATGCCACC

GACCCGGAGACGTGGGGCATCCCCGACAGTCACGACGAATCGGACCGCGG

CGAGGTGCTCCTCGAAACCGACGGTCTCGAGAAGTACTACGAGCAGAGCC

AGCCGCTCTTGGAGCAACTCAGGGGGAACGACCCGAACTACGTCCGCGCC

GTCGACGGCGTTTCCCTGCGCGTCCGGCGGTCCGAAATCCTCGGCATCGC

CGGCGAGTCCGGCTGTGGGAAGTCGACGCTCGGCGAGACCATCGCGCTCC

TGAAACAGCCGACCGGCGGCGAGTTCGTCTTCGACGGCGAGCCTTACGAG

CACTACGTGGACGGGAACATGCGCGAGTTCCGCCGGAAGGTTCAGATAAT

ATTCCAGGACCCCTTCGACTCGCTCAACCCGAGACAGACGGTTCGGCAGT

TGGTCGGCGAACCGCTCACTATCCACGACTACCGGACCGACGAGCGCGAA

CGGGCCATCATCGAGACGTTGGAGAAAGTCGGGCTCACGCCCGCGCGGAA

GTTCCTCGACCAGTACCCGCACCAGCTATCGGGGGGCCAACGTCAGCGCG

TCGCGGTGGCCAAGGCGCTCGTTCTCGACCCCGACTTCCTCATCTGCGAC

GAGCCGGCGTCGATGCTCGACGTGTCGCTGAAGGTGAACCTGCTGAACCT

CCTGCGCGAACTCGCGGACACGGAGGACATCGGTATCGTCTACATCTCTC

ACGACCTCGCCAGTCTGGTACAGGTGTCGGACCGACTCGCCATTATGTAC

CTCGGGCGGGTCATCGAGGAGGGCGACGTCGAATCCATCGCGGCCGAGCC

GAAACACCCGTACACCACTTCGCTCCTCGCGGCCGCCCCCGAGAAGGACC

CGACGGTCGACCGCGACCGCGTTCTGCTCGACGGCGAGCCACCGAATCCG

GTCGACCTCCCGTCGGGCTGCGTCTTCGCTCCGCGGTGCCCGAAGGCGGA

AGACGAGTGCCGGGAGAGCGAACCCGGACTCGATACCGTCCGAAGCGGCG

AGTACCGCGCCGCATGCTACTTCCCGGACGGGGAGACCCCCGCCGCAGAC

GACCTCGCCGACGACCGCTCGGACTCTCAATACGGGGACTCTGAGTTCTC

GCCGCCGGCCAGCGGCGACTCGGCCAGCGACTGACGCCGCCCTTCGACTC

TCCTCGCTTCGTCTCCTTAGCGACCTCCCAGTAGCTCCGTGTCCCGATTC

GAGGTTCGGCTGACGCATTAGAATTGCATAAACCAAACAATATTATAAGG

AAGAATATTAGAATAATTTTCAAATTATTGAATCTATTTACTTATATTTG

TTTCTTCTGTGTGTTTACTTCTGTGAATATATCTCAGATGTCCAAATAAA

ACCGGAAAATTCTGTTAATATCTATCCTATATAGTCAAAATGGGTATAAA

AAGCTGACTTCAGCTATATTGCGGGGTTCCGTGGTTGATATATCTATTTA

CGCCGGATGTAATATTGTGTTTAAATTGATTTACTATTATCCGGTCTGGG

TTGCGGTCGACCGCTCTCCCTACGCCAACTCCGAGATATCGAACACCGGC

TTGCACGTCCCGCCTTCGATGAACGTCTCGAAGGCTTCGTCGGCTTCGAA

CAGCGAAAACCGGGTGTCGACGAACGTCTCGCAGTCCACGTCGCCGTCGC

GAATCAGTCGGAAGGAGCGCTCGAAGTCCTCGTACATCGAGGCGTACGAA

CACTGGAGGTCGATTTCGGCCCGAACGAGCGGGGAGTAGGGCATCGACGT

CTCGCCGGTCTGGCCGACGAGGACGATTTGGCCGCCCTTGCGGACCTCGT

CGACGGCCGTCGTGAGCCCCGAGGGGTGGCCGGTCGTGTCGAACACCACG

TCGTAGCCGACGCCGCCGGTGTGGCGCTCGCGGCGCGCCTCGGTGTCGTC

GTCGGCGACGTTGACCGTCTCGAAGCCGAGTTCCTCCGCGAGCGGGAGGC

GATAGTCGGCGTCCTGTCCCACGCCGGAGACGACGACGGTGCCGCCCTGC

GCGCCCGCGACTTGGGCCGTGAGCAGACCGATTGGCCCGGGGCCTTCGAC

CAACACGCGGTCGCCCGCGCCGACCCGGGAGTTCTCGATGACGGCGCGCG

CGGCGATGCTCGTCGGCTCCGCGACCGCGGCGTGCTGCTGTTTCACGTCG

TCGGGAATCGGGTGGAGGGCGCGCTCCGGGACGGCGATGAACCGCTCGTA

CGCGCCGTCGTGGTCGACGCCGGTGATGACCGCGTCCTGGCAGACGTTCT

CCTCGCCGATGCGACACTGATAACACTCGCCGCAGCCGCGAATCGGTCGT

TCGACCACGCGGTCGCCGACGCCGAACTCCGTCACGCCGTCGCCGACTTC

GACCACGCGACCGGCGTACTCGTGGCCGATGACCGTCGGGAGGTCCATCC

GCTCGAAGGCGGATTCGAACTCGTAGATTCCCGCGTCGCTCCCGCAGAGG

CCGGCGTAGTCGACCTCTACGAGCACCTCTCCGGCACCCGGTTTCGGTTT

CTGTCGCTCGACGAACTCCATAGCGCCGGAGCTACGCTCTACTTTGGCAA

GTCCTCGCATGAGAGCACATGTGGCACGGGTCGGTAAAAACGTATTGCAT

GCGGCACTCCCGTCGCGCGTTCCGCAACTATCATGTCGGCACCGTGCGGA

CTGAGACCATGGTCGCATACGAGCACACGCCAGTCTCCGTCAGTGACAAG

CGCGTCGTCGTCGTCGGCGGGACGAGCGGTATCGGTCAGGCAATCGCCCT

CGGATTCGCCAGCGAGGGCGCGGACGTCCTCGCAACCAGCCGACGCGAGT

CGGCGGTCGACGAGACGGCCGACGCCATCGAGGAACTGGGAGCGAACGCG

GTTCGGGAGACCTGCGACGTGACGGACCCGGCCTCGCTCGAACGCGTCCG

CGAGCGCGCCGTCGAGGAGTTCGGCGGCGTCGACGTGGTCGTCGCCTCGC

AGGGCGCGATATCCCGCGAGACGGTCCGCGACATCGACGACGACGCGTGG

GACCACGTCACCGACATCGCGCTCGGGGGCGTCCGCCGCGTGACGCAGGA

GCTGGCCCCGGCCGTCGCCGACGGCGGGTCGATTATCAACATCTCCTCGT

TGGCCGCGCGGCTCTCGATGGCGAACCTCCCCGCCTACTCGGCGGCGAAG

GGCGGCGTCGAGGCGTTCACCCGCGCGTCCGCGAAGGAACTTGCCCCCGA

GATACGCGTCAACGCCATCGCACCGGGCTTCGTCATCACGCCGCAGAACG

CCGACACCTACGCCGAAGGGACCGAAAAGCGCGAGCGAATCGACGAGCGA

ACGCCGCTCGGCAGAGTCGCGGAACGGGAGGAAATCGTCGGCGCGGCGGT

CTTCCTCGCGAGCGACGCGTCTTCGTTCGTGACTGGTGAGGTCGTCACCG

TCGACGGCGGGTTCGCGGACAGCGCGTTCTAACCCCGTCTTTCGTCTCCC

GTCTCCCGTCTCTCGTCTCCCGTTTCGCCATCGACGCGCTCTCGTGAGCG

CGCGCTAGCCCCTCCCGACGGGAGTTTCGATGTCGCTTCCGACAGGTTTG

CCGCCGGCAGACGCCGGAGACTGCTGGACAATCGTTCTGTAATAGGGAAC

GAAGGGCGAGGCCGAGTGACTCGTCCCCCGCGTTCGCTCTTGCTCCGGAC

TCTCGGGAACCCCGCCCCCCTTCTCGGCGTCGGCCACCCGAATTACAAGC

GTATGTGTGTAATCTAAAACCGGCGCACCGCACGTCGCGTGGGCTGTTCC

CGGACTCTCTCGTTCGTGCGTGCTGTGATTGCATTCTGTGATAGAAAACG

GCAGTGTCTGGCGAACTCTCGGCCCACTCGTTCCTGATTATCGGCCGAGA

CGAGCCGTAATAATCGACTTGTCTCTCTCGGTGGTCGTTTTCGACGACAC

CGGTGTGGGTCGTCAGTCCGCGCTCGTGGGCTCCGACACGGCCGTCTCGT

CGCCGCGTTGCCGATTCGACCCGCCGACTCGGCGTGTTCTGTTACTGGGA

ATTCGTGTGTCTCTCTACGTCACACGCGTCGGGCTCGTGTCGCCTCTTCT

GCCTGTCATCTCGCGCTCGTCGGCGCTCGTCGTCAACGGACGAGCGAACT

ACACCCGGCCGGGAGTTCGAGACCGAACCCGCCGACGTCTCGGGGTTTTC

TGCATTGCCCCGCGCCGCCCATACCGGGACGTACTTTTATTACAATCGCT

GGTAATGAGGATACTGAATGCATTACCACCAGCTAGCGGTGTCGGGAGAG

CGTCGTCTCACGGCAAGCCGCGACTCCACGACGTACGACCTCACGAGCGC

CGACGCGGACCTTCGGACGTTCGGTGACTTGGCTCGGGTGGCGAGTATCG

CCCGGACCTCGGTCGACCGACTCGCCGCCGAACTGACCGAGGACGCCGAC

GTGGTCGACGACGCGTTCGTCGACCGGCACGCGACGGTTCCCGTCGACGC

CGAGGAAATCTGGGCCGCGGGCGTCACCTACCAGATAAGCGAGCAAGCGC

GCGAGGAGGAGAGTTCCATGCCCGATATGTACTTCGACGTGTACGACGCC

GACCGGCCGGAGGTTTTCTTCAAGGCCACGCCGTCTCGGACCGTCGAACC

GGGCGACGCCATCGGCGTCAGAGGCGACTCCGAATGGGACGTGCCGGAGC

CCGAACTGGGTATCGTGCTTCGCCGGGGGGAAATCGTCGGCTACACCGTC

GGCAACGACGTGAGTAGTCGGTCGATAGAGGGCGAAAACCCGCTGTATCT

CCCACAGGCGAAAGTGTACGACCGCTGTTGTTCCATCGGCCCGTGTGTGG

TGACGCCCGAAGACGTGGAGGACCCCCACGAGTTGGAGATGAGCATGACT

ATCGAGCGCGACGGCGAAGTCATCTACGACGACGCCACGAACACCAGCGA

GATGGTCCGCAGCTGCGACGAACTCGTGTCGTACTTCACGCGACACAACA

CGGTGCCCGAACTGGCGGTCATCCTGACGGGCACGTCGCTCGTCCCCGAA

CAGCCGTTCGACCTTCAAGAGGGTGACCACGTCGACATCACTATCGAGGG

TATCGGAACCCTCTCGAACTCCGTCACCACCGTATGAGCCCCGCCCCCAC

CGACATCGTCGAGGAGTTCACGCGCCGCGACTGGCAGGGAGACGACGTGA

CGGGCACCGTGCGGGTCGCCATGATCGGCCTCGGCTGGTGGACCCGCGAC

GAGGCGATTCCCGCGGTCGAGGCGTCCGAGTTCTGCGAGACGACGGTCGT

CGTCAGCAGTTCGAAGGAGAAAGCCGAGGGCGCGACGGCGTTGACCGAGT

CGATAACCCACGGCCTCACCTACGACGAGTTCCACGAGGGGGTCGCCGCC

GACGCCTACGACGCGGTGTACGTCGTCACGCCGAACGGTCTGCATCTCCC

GTACGTCGAGACCGCCGCCGAGTTGGGGAAGGCGGTCCTCTGCGAGAAAC

CGCTGGAAGCGTCGGTCGAGCGGGCCGAAAAGCTCGTCGCCGCCTGCGAC

CGCGCCGACGTGCCCCTGATGGTCGCCTATCGGATGCAGACCGAGCCGGC

CGTCCGGCGCGCCCGCGAACTCGTCGAGGCCGGCGTCATCGGCGAGCCGG

TGTTCGTCCACGGCCACATGTCCCAGCGCCTGCTCGACGAGGTCGTCCCC

GACCCCGACCAGTGGCGGCTCGACCCCGAACTCTCCGGCGGCGCGACCGT

CATGGACATCGGGCTCTACCCGCTGAACACCGCCCGGTTCGTCCTCGACG

CCGACCCCGTCCGCGTCAGGGCGACCGCCCGCGTCGACGACGAGGCGTTC

GAGGCCGTCGGCGACGAGCACGTCAGTTTCGGCGTCGACTTCGACGACGG

CACGCTCGCGGTCTGCACCGCCAGCCAGTCGGCTTACCAGTTGAGCCACC

TCCGGGTGACCGGCACCGAGGGCGAACTCGAAATCGAGCCCGCGTTCTAC

AACCGCCAAAAGCGGGGATTCCGACTGTCGTGGGGGGACCAGTCCGCCGA

CTACGACTTCGAGCAGGTAAACCAGATGACGGAGGAGTTCGACTACTTCG

CGTCCCGGCTCCTGTCGGATTCCGACCCCGCGCCCGACGGCGACCACGCG

CTCGTGGACATGCGCGCGATGGACGCGATTTACGCCGCGGCGGAGCGCGG

GACCGATGTCGCCGTCGACGCCGCCGACTCCGATTCCGCCGACTCCGATT

CCGCCGACGCTGCCGCCGCCAACCACGACGCCGACCCCGATTCCGACGGG

ACGTAGGACTCGATTCGGCGCGTCGTCGCCCGCAGTTTTATTATCGGGTT

TCCCGCAGTTCCCCGCATGATGTTCGGCATCCTCGGCACGGCAGGCATCG

GCGTCAAATCGGTCATCCCCGCGGTGCAGGCGAGCGAGCACGAGGCGGCC

GCCATCGCGTCCCGCGACGAGGCGCGAGCGAGCGCCGTCGCCGACGAACT

CGGCATCCCCACCGCCTACGGCAGCTACGAGGCGCTGTTGGCCGACGACT

CCCTCGACGCGGTTTACATCCCGCTCCCCAACGGCCTCCACGCCGACTGG

GTTCGCGCGGCCGCCGACCGCGGGCTGCACGTCCTCTGCGAGAAGCCGCT

GACCGCCAGCGCCGACGAGACGGCCGCCGTCTTCGACTACTGCGAGGACG

CGGGCGTCACGTTGATGGAGGCGTTCATGTACCGCTTCCACCCGCTGACC

GAGCGGGCCGCGGAACTCGTCGCGTCTGAGCTCGGCGCGGTCGTCTCTGT

CACGTCGAACTTCTCGTTCCGCCTGCCCGACGGGGCCGACGACATCCGCA

TCGACCCCGACCTCGCCGGCGGGAGCGTCATGGACGTGGGCTGCTACGCC

GTCAGCGCGGCGCGACTCTTCCTCGGGACGCCCGACCGGGTGTACGCGAC

CACGACCGACACGCGCGACTGCGGCGTCGACACCCGGATGTCGGGCGTCT

TGGAGTACGACTCCGGCGCGACCGCCCGCGTCGAATCGTCGTTCGACACG

CCCGAGACGCAGTACTACCGCGTCCAGACCACCGACGGCCGGCTGGAGGC

GAACCCCGCGTTCAACGTCGACCCGACGGCCGCGGCCGAACTCACTTACG

CGACCGACGGCCGCGTCGTCACGGAGACGTTCGACCCGACAGACAGCTAC

CGCCGCGAGGTCGAGGCGTTCGCCCGCGCCGTCGAGACCGGCGAGACGCC

GCGCGTCGACCGCGAGGAATCCGTGTCGGTCATGCGGACCATCGACGCGA

TTTACGAGAGCGCCGAGACCGGGGCCGCAGTCGAGCTGGACTGACCGCCC

GCCGCTGTCCGTTTCGACTCACCCCAACCGAACGTCGGAGCGGAACTCGG

GCCGCCCGGTCGCCGCCACGTCGAGGCGGAACAGCGCGCCCGCAGCGTCG

CCGGTGCTCTCGTCGCCCTCGCCAGCGCTCCCGTCCCCGTCGCCGCCGGC

GGTCGTCACGTAGAGCGAATCGAGGTCCGGCCCGCCGAAGGCGACGCTCG

TCACCTTCTCCGTCGGCACGTCGAACCGGCCGAGTTCGGTGCCGTCGGCG

TCGTACTCGACCACACAGCCGCCCTCCCAGCGGGCCGACCAGATGTGCCC

CGCCGAATCGACCGTCATCCCGTCCGGTAGCCCCGGCGTCTCCGGCGATT

CGACGAACCGCTCGCGGGCCGAGACGGCTCCCGTCTCTTCGTCGTAGGCG

TACCGATAGACGGTGCGCGCCTCGGTTTCCGTGAAGTAGAATCGCTCGCG

GTCGCGGGTGAATCCCATCCCGTTGGGGATTCCGACGCCGGTTTCGACCG

TCGTCACCGTTCCGTCGGTGTCGAGGCGGAACAGCCGCCCGCCCGCCGTA

TCTGACGGCATCGTCCCGCAGAACACCCGCCCGGCGGGGTCGGCGATGAC

GTCGTTGAACCGGGTCGGTGAGTCGACGATTCGCGCGCTTTCCCGGCGGT

CGCCGTCGACGACGCGCCCGACGCGACCGCGGTCCATGAACGCTAGGAGC

GACCCGTCGCGCTGTATCGTCACGCCGGCGATGACCGACGTTTCGACCGG

ACAGTCGTGCGCTCCGGTCTCGGGGTCGTAGCGGTGGAGTCGCCCGGATT

CGATGTCTACCCAGTACAGCCGCTTCTCGTCGGGGTGCCAGACCGGTCCC

TCGCCGAGTCGGCACGACGTGTCGACCACTCTCGTGACGGTCATGAGCAA

CCGTTCGGAACCGACCGCAAAATACGCTTCCCTCGGGTTCGGGGCGCGGC

ACGCCGACACTGCGCCGCCGTGCTGCTACGCCACCGCGCCCCGTGTCGCC

GTGCCGTCGCACCCCCGAACCCCCGTGCCCGCCGCTCAGAAGTGCGAGAG

CTTGTCGCGTCGGTAGAGGTCGAGCGACAGCACGGTGTTCGGTGACCGCT

GGCGGACCGCGTAGGCGATGGCGTCGGCCACCTCTATCGCCTCCGTCACT

TCGCCCTCCTCGAACGACGCTTCGAGCGTCTCGCCGCTCTCGGAGCCGAA

CTCGGTCCGAACCTCGGTCGGATTGACGCAGGTCACGCCGACGTTCTCCT

CGCCGATGGACCCCTGCAGGCTCGACGCGAACCCGCGGACCCACCACTTC

GTCGCGGCGTACACCGGGTTGTGCGGCCGCGGGTGGTTCCCCGACATGCT

CCCGAGGAAGACGAGGTTGCCCTCGGACGCTTCGAGGTGCGGAAGCGCCT

CTCTGGCCGTGTAGAACATCCCGTCGACGTTGACGCCGGTCATCAGGCGG

TACTCCTCGTCGGTCAGCTCCGCGACCGGCTTGTCGATGCCGAGGCCGGC

GTTACAGACGACGATGTCGAGCGAGCCGAACCGCTCGACGGTCGCGTCGA

CGAGCGCCCGGACCGCGTCGGAGTCGGTCACGTCCGTCGGCACGACGCTC

ACGGCGGCGTCCGTGGCGTCACGAATCTCGTCAGCGAGCGATTCCAGTCG

TTCGACTCTGCGGGCGGCGAGACACACGTCCGCGCCGTCCTCGGCGAGCA

CGCGCGCGGTCGCCGCGCCGATTCCCGAACTCGCACCCGTGACGAGCGCC

GTCCGGCCGTCGAGCGGGTCTTCCAAGGCGGTTGGTCGGTCGACCATACG

CCGTGATTGTGACCCTGCCGCATACGTGTTTCCCCACGGGAATCTGGCGA

CGGACGCTTGGAACACGTTTATTAGACTTCCGTCGATTGTCATCGTATGG

CACGTATTGCGGTCACCGGAGCGGCTGGTAACGTCGGGAGAGTCACGGTC

GAAGCGCTGGCGTCGGACCACGACGTGACGCCCATCACGCATCGCGAGCG

CGAGGGACTCGACAGCGTCATCCTCGACGTGCGCGACGAAGACGCGCTGA

CCGAGGCGTTCGAGGGCCACGACATCGTCGTCCACCTCGCGGCCAACCCG

AACCCCGACGCGGCGTGGGACAGCGTCTACGAGGTCAACATCGGCGGCAC

GTACAACGTCTACGAGGCGGCGCTGGCGGCCGATATCGACCGACTCGTTT

TCGCCAGCACGAACCACGTCCACCAGATGTACAACATCGCCGACGCGACC

CGACCGGAGACGCTGGCGGCCGACGCCGAGGCGGTCGGCGTGTCGGACCC

GCCGCGCCCCGACTCGTACTACGGCGTGAGCAAGGTGTTCGGTGAGGCGC

TTGGCAACTACTACGCGGACCGACACGGACTGGAAGTGCTCAATCTCCGC

ATCGGATGGCTCCTGACGGCCGACGAGGTCCGCGAGAAGATGGACGAAGA

GGAGTCGGTCGCGCGCTACGTCCGCGCGATGTGGCTCAGCCCCGGCGACT

GCGAACAGGGGATGCGCCGGGCCGTCGAGGCGTCGCTTCCCGATTCGCCG

CTCGCGGTCAACCTCATCTCGGCGAACGACGACCGATATCTCTCGCTCAC

GGAGACGATGCGCGCCATCGGCTACCGCCCGCGAGACAACTCGGCGACCG

TCGTGGAGTGAGCGAGCGGGGCGGTGGCTGAGCGACCGTGGAGTGACGTT

CGTCGATGCCGACGCACCGCTACTGCGAGTCGGTGTCGCGGGCGACGACG

CACGACCCCGAACGGCGTCTCGTTCGCCTCTCGAAGGAGCGTTCAGGTTC

TCGCGCGGTAGTTTTCGGGGCGAATCCATCGCGAAGCGCGTTGCTGGGAC

CCACCGTTTTTATATCATCATCTGCTATCCACAACCTATATGTGTGGGTA

GATAACATGGGTACTACATGGTCGAGCATGACAGTAGCGATGAAAGTGTA

AACCGTCGAAAATATTTGAAGGCCCTGACCGTGGGTGCGGCCGCTGGCAT

CGCGGGGTGTACCGGCGGTGGCGGAACCGAGACGGAGAGCACCGAGAGCG

GAAACGGGAACGGTTCCGGCGGTTCCACCGACGACACCGAGACGAGCGGG

AGCAGTTCCGGTGAGTCGTGGGACTCGCAACTCGAAGTGCTCCACGGGTG

GGCCGGCGGCGACGGCGAGGCGGCGGTCACCGCGCTCATCGAGGCCTTCG

AGGAGGAACATCCCGAGATGGATACGAACTTCCAGGCGGTCGGTGCGAGC

GCGAACGTGAACCTCAACGCGACGATTCTTCGACGGCTCGCGAACAACAA

CCCGATGAGCTCGTTCGCCAACTGGCCGGGGAAGAACCTCGAACGGTACT

CGGGCGCACTGATGGACCTCGAAGCCGACGTGTGGGACGCCGAGGGATTC

AAAGACACCATGCAGTCCCGCGCGGTGGAACTGTGTAAGTTCAACGACAA

GATGCCGGCGGTCCCAATCGGGTCCCACCGGATGAACAACCTGTTCTACA

ACACCGCCGTCTTCGAGGAGGCCGGCATCGACGCGTCGAGCCTCGATAGC

GTCGACGCGCTCCTCGACGCGCTCGAAACCATCGACCAGAACACGGACGT

GACGCCGATGGCGCAGGCGATGGTCGCGCCGTGGACGAACCTCCAGCTTT

GGGCGCAGATTCTGACGAGCCAAAGCGGCGTCGAGGCGTACACGAACTTC

ATCGAGGGCAACCCCGACAGGGCGGCCGTCGTGGAAGCGCTCGAGGCGCT

GAAGACTATCAACGAGAACTACATCACCGCCGACGCCTCGTCTATTAGCT

TCACGACGGCGGGCCAGAAGGTCATCTCGGGGAAGGCGGCCACCATCCAC

CAGGGGAACTGGGTCTACGGCATGTTCCGCGCCGACGACAGCTTCAACTA

CAAAGAGCAGTGGGATTGGATACCGTTCCCGGGCACGGAAGGTATCTACT

TCTACCACGTCGACTCCATCGTCGCGCCGAGCAACAACCCGAGCCGCGAG

GAGACCATCGCGTGGCAGAAGTTCGTCGGCTCGAAGAAGGCCCAGATCGC

CTTCAACAACCCCAAGGGGTCGGTTCCGCTCCGCACCGACATCGACCCGA

GCGAACTGACCGACTTCCTCGCGATGACGTACGAGGACCTCACCGATTCG

GAGGCCTACCCGCCGACCATCGCCCACGGGCTCGCAGTCACGCCCAAGAC

GATGGGCGCGTGTAAGACCGCCTTCGGCGACAACTTCATGGGGCCGTTCA

ACGTCGAAGCCACCGCCGACGCGCTCGTCGCAGCGGTCTCGGAGTGAGAC

CTCGGCTGAAACTTCACCCTCATTATACACAAATAAAATATAGGTATATC

CATGGCTACACACGATAACACGGAGCACGTTTCTGACGCGACCGACGAGG

CGGTCGGGTGGGAGTCGAAGCTCCGGTATTTCCTCAACAGCGACTTCGTC

CGCTCCGCGCCGTACTGGGGAATCCCGTTCGTCCTCATGAGCATCGCCGT

CTACGGTGGTACCGGCTACAACTTCGCCATCTCGTTTACGGACTACGAGG

GCCTCGGGACTCCCGATTACTCCACGCTCGACTTGGAGATGTACGCACAG

GCGCTGTCGAGCGACGCGTTCATCGCCGCCGCGCAGAACAATCTGGTCCT

CCTCGTAGGCTTTACGACTATCTGTCTGGTGCTCGGCCTGTTCCTCGCAA

TCCTGTTGGACCACGGCATCCGGTTTTCCGAGAAGTTCCAGACGGTCTAT

CTCCTCCCGATGAGCCTCTCGTTCGTCGTCACCGCGCAACTGTGGCTCTG

GATGTTCAACGTCGAAAGCGGCATCCTCAACCTCGTCGTGACGACCCTCG

GGTTCAATCCCGTAGACTGGCTGGGGAACCCATCAATCGCGCTCGGCGCG

GTGATATTGGCCCTCATCTGGCAGTTCAGCGGATACACGATGGTCGTCTA

CCTCGCGGGGCTCCAGTCGATTCCCGACGACCAGTTCGAGGCGGCCCGCG

TCGACGGCGCGAGCATCACTCGGACCTACCTCCGCATCATCGTCCCGCAA

CTGAAGGAGGCGTCCGTCAGCGCGGCCGTCGTGCTGATGGTGTTCGCGCT

GAAGGCCTTCACCTTCCTGTACGCCCTCGTCGGTCGCTACCGCCCGCCGA

ACGGGACGGACATCCTGGCGACGCTCATGGTTCGCCGCGCGTTCAAGTTC

GGTGAGTGGGCCTACTCGGCCGCCATCGCGACCATGCTTCTCATCATGGC

GCTCGGCGTCATCGGACCGTACCTCTACTACCAGTACAAACAGGGGGGTC

TCTGACCATGTCACAGTCGTCATCCACAGGCAACTTCGACGTCGCATCGC

TCGTCGAGGACGTGAACCTCCGGCGCGTCGCCCAGTACGCTCTCGTCGTG

TTCTTCCTCGGGTTCTTCCTCGTCCCGCTGGAGACGGGAATCATGACCGC

CATCAAGACGAACGAGTCGGTCGCTCGCTCGCTTCCCTTCGCGCCGCCGG

TCGGTGAGGGTTTCACCCTCGGGAACATCCAGTTCGCCCTCGAACAGCTC

TCGGGGTCGTTCTTCAACTCGCTCATCATGTCGATTCCGGCGACCATCGG

GAGCGTCCTGTTCGGGAGCATGGCAGCCTACGGCCTCACGATGGTCAACT

GGCGGGCGCAGATGGGTATGCTGATGCTGTTCGTCGTCGGCGTCTTCGTC

CCCTATCAGGCCGTGTTGGTCCCGCTCGCACGCTTCTGGAACAACATCTT

CCCGCTCGCGCGGATGATAGAGCCGATGGTGGCGTCGATACCCTTCTTCC

AGGGGTACCACGCGGAACTCGTCCCCCTCGTCATCACCCATATCGCCTAC

GGGATTCCCATCTGTACGATACTGTTCCGGTCGTACTACCAGAGCCTCCC

GAACTCGCTCGTGGAGGCCGGTAAAATCGACGGCGCGAGCATCACGAAGA

TTTACCGGCGCATCATCCTGCCCATCTCGAAGCCGATGTTCGGCGTCGTG

TTCATCTACCAGTTCACGCAGATTTACAACGAGTTCCTCTTCGCGTTCAC

GCTCGTCACCGGGTCCGACGCGCCCGCAGCACCGGTCACGCTGGTGCTGC

CCGCAATCGGGGCGTCGACTTCCGGCATCAACTTCGGTATCAGGATGTCC

GCGGCGTTCCTCGCGGCGGTTCCGACGCTCATCCTGTACGTCGCGTTCGC

CGAACAGTTCGCGAAGGGACTTCGCACGGAGGCCTGACCCATGGGACAGA

TTCAACTCACCGACCTGACGAAGCGCTTCGGCGACACGGTCGCCGTCGAC

GACCTCTCGCTCGACATCGACGACGAGGAGTTCCTCGTGCTCGTCGGTCC

CTCGGGGTGCGGCAAATCGACGACGCTCCGGATGCTCGCCGGCTTGGAGA

CCCCGACCAGCGGGGACATCTACATCGGCGGGGACCACATGAACTACCGC

GTCCCGCAGAACCGCGACATCGCGATGGTGTTTCAGGACTACGCGCTGTA

CCCCCACATGACCGTCCGGCAGAACATCCGGTTCGGGCTCGAAGAAGAGG

AGGGATACACGTCTGCGGAGCGCGACGAGCGCGTCGTCGAAGTCGCGGAG

ACGCTCGGCATTGCCGACCTGCTTGACCGCAAGCCCGACGAACTCTCGGG

CGGTCAGCAACAGCGGGTCGCGCTCGGGCGCGCCATCGTACGCGACCCCG

AGGTGTTCCTGATGGACGAGCCGCTGTCCAACCTGGACGCCAAGCTCCGG

GCGGAGATGCGCACCGAGCTCCAGAACCTGCAGGACCAACTCGCCGTCAC

GACCGTCTACGTCACCCACAACCAGACGGAGGCGATGACGATGGCCGACC

GTATCGCCGTCATGGACGACGGCGAACTCCAGCAGGTTGCCTCTCCCTTC

GAGTGCTACCACGAGCCGAACAACCTGTTCGTCGCGGAGTTCATCGGCGA

GCCGATGATAAACCTCGTCCGCGGAACGCGCTCGGAGTCGACGTTCGTCG

GTGAACACTTCTCGTACCCGCTCGACGAGGACGTGATGGAGTCCGTCGAC

GACCGCGACGACTTCGTCTTGGGGGTGCGCCCCGAAGACATCGAAGTCGC

CGACGCGGCCCCCGACGACGCCGCGCTCGACGATCACGATCTCCAGATGG

ACGTGACCGTCGTGGAGCCCCACGGCGACCAGAACGTCCTCCACCTCTCG

CACCCGGACCAGCCCTCCGCCGACGACGCCCTGCAAGCGGTCACGGAGGG

GATGCATCTCGTCACGCGGGGCGACCGCGTCACGGTGACGATTCCGCCGG

ACAAGATTCACCTGTTCGACGCGGAGACCGGAACCGCGGTGCACAACCGA

CGTCACGACCAGGAAGCCGACTTCACACAACTCGAACAGTAGCCATGGCA

CGATTGACACTCGACGACGTAACGAAGGTGTACACCGACGAGGGCGGCGG

CGATATCGTCGCCGTCGAAGAGATATCGCTCGATATCGACGACGGGGAGT

TCCTCGTGCTCGTCGGCCCCTCGGGCTGCGGGAAATCGACGACGCTCCGG

ATGATGGCGGGACTCGAAACCGTCACCGAGGGCGAACTCCGACTCGAAGA

CCGCGTCCTCAACGGCGTGTCCGCGCAGGACCGGGACATCGCGATGGTGT

TCCAGTCGTACGCGCTGTACCCGCACAAGAGCGTCCGCGGGAACATGTCG

TTCGGCCTCGAAGAGTCGACCGGCCTGCCGGACGACGAGATACGCCAGCG

GGTCGAGGAGACGACCGACATGCTCGGCATCAGCGACCTGCTCGACCGCA

AGCCCGGACAGCTTTCGGGCGGCCAGCAACAACGGGTCGCCCTCGGACGC

GCGATCGTGCGCGACCCCGAAGTGTTCCTGATGGACGAGCCGTTGTCCAA

TCTGGACGCCAAACTGCGGGCGGAGATGCGGACCGAACTCCAGCGCCTGC

AAGGCGAACTCGGCGTGACGACCGTCTACGTCACCCACGACCAGACCGAG

GCGATGACGATGGGCGACCGCGTCGCCGTCCTCGACGACGGCGAACTCCA

GCAGGTCGGCACGCCGCTGGACTGTTATCACCGGCCGAACAACCTGTTCG

TCGCGGGCTTCATCGGCGAGCCGTCGATGAATCTCTTCGACGGGTCGCTT

TCGGGCGACACCTTCCGCGGCGACGGCTTCGACTACCCGCTTTCGGGCGC

GACCAGAGACCAACTCGGCGGCGCGAGCGGCCTCACGCTCGGCATCCGAC

CCGAGGACGTTACCGTTGGCGAGCGGCGCTCCGGCCAGCGCACCTTCGAC

GCCGAAGTCGTCGTCGTCGAGCCGCAGGGGAACGAGAACGCCGTCCACCT

TCGATTCGTCGACGGGGACGAGGGGACCCAGTTCACCGCGACAACCACCG

GACAGTCGAGAGTCGAAGCGGGCGACCGCACGACCGTCTCGTTCCCCGAA

GACGCGATTCACCTGTTCGACGGGGAGACCGGCGACGCGCTGAAGAACCG

AGAGCTCCCGTCGAACCGCGCCATCGACGCGTTCGTCTCGAACTGACCGG

TCTCCGCGGTTTCCTTTTCGTCCGCGGTTCGCCCGCCGCTGTCAGCCGCC

AGGTTATCTGCCGTCAGCGTCGCGACTGCAACCGCGAGTCGCGTCGCTCC

GGTCTCGAACTCAAGCGAGTGAAAACGCGGCCGCGGCGGACGCGCCGCGG

GGCGAGAGATGCTCACGGTTCTCGCCCGCGGTGGTGCGTCCGGGTTATGG

GTGTGGTATGTCAGGCTGTCGTGGCCGCTGTGGGACAGTCTCGCTCTCGG

CGTCGGGTTACTCCTCGTCGAAGAGCGTCTCCCCTTCGACCATGTGTGCT

TCGACGGCGTCGAGGTCGAGCGTCAGCCCGAGGCCGGGCTTTTCGGGAAT

CTCCATGTGACCGTTCTGAATCAGGTCGTCCTCTTCGACGAGGTCCTCCC

ACCAGCCGAGCTGGTAGGAGTGGTATTCGAGGGCCAGCGAGTTCGGGATG

GCCGCGGCGACCTGCGCGGAGGCCATCGTCCCGATGGGCGACGAGACGTT

GTGCATCGCCACGGGGATGTAGTACATGTCCGCGAGGTCGGCAATCTTCC

GCGTCTCGCGCATGCCGCCGACGCGGGGCAGGTCGGGCGCGATGATATCC

ACCGCCTGCGGTTCGAGCAGCGTCCGCTGGCCGAACTTCCGGTAGACGTT

CTCACCGACCGCGATGGGCGTCGTCGTGGACTGCGTCACGAGCTTCTGCA

CGTCGTGGTTCTCCGGCGGCACGGGGTCTTCGAGCCACCACACGTCGTAG

TCTTCCAGCTCGGACGCGAGGCGCTTGGCGCTCCCGCCGGTAAAGGACCA

GTGGCAGTCGAACGCCACGTCCGCGCGGTCGCCGACGGCCTCGGTGACGG

CCTCGACGATTTCGACCTTGTGGTCGATTTCGGGGTTTCGGAGGTGGCGG

TTCGCGCGGTCCTTCTCGTGGCCCGAGGGCACGTCGAGGTCGAACTTGAT

GGCGTCGTAGCCGAGTTCCTCGACCACGCGGACGCCCTCCTCGGCGCAGG

CCTGCGGGTTGGCCTCGTCTTCGGTGTGGAGGTCGCAGTAGACGCGCACC

TCGTCGCGGTACTTCCCGCCGACGAGTTGATAGGCGGGCACGTCGAGGAG

CTTTCCGGCGACGTCGTGGAGCGCGATTTCGATGCCCGAGATGGCGGAGA

TGACCTTGCCCGAGACGGAGCCCTCGCCGGACATCTTCTGGACGAGATGC

TCGTACAGGCGGTCGATGTCGAGGGGGTTCTCGCCGACGAGGAACGGCTT

CATCCGCTCGATGATGGCGGTGTCGCCGCCGCCCCAGTAGGCCTCGCCGG

TGCCGACGACGCCCGCGTCGGTGTAGACGCGGACGAGAATCCACGGGTAG

TTCCCGTCGACCATCGTCGTCTGTACGTCCGTGATTTCGGCGTCGCGGAC

GCCACCTCGCGGATTCGTGATGTCTATCGTCTCCGCGGACAGGTCGCGCA

TCGTGTACTCCGCGTTCGGGTCGCTAAGCTTCGCTTGCTCAACCATTGCG

CACGTATATCTCGAAAGCCACTACAAAAGTGTGTGGGTCGGTAGTCCGTC

ACTCACCCCGACGCGCGCTCCCGAACTCGCCCCGTCGTCGACAGCCAACA

AATAGTGACAGCTTCGCAAGAGTTGTATGTCGGAAGACAGAACGTGTACC

CGATGACGGACCCTAGTAAAAACTACGTCAACGGTGAGTGGGTGACCTCG

GAGACCGGCGAGACGACCGAGGTGACGAACCCCGCGAACCCGAGCGAAGT

GGTCGCGGCGTATCAGCACTCGAACGAGAACGACGCCGCGGCGGCCGTCG

ACGCCGCCGTCGCGGCGGAAGACGAGTGGCGAAACACGCCCGGCCCCGAG

CGCGGGCGCATCCTCCGCGAGGCGGGGACGCTCCTCGCCCAGCGGAAGGA

CGAACTGACGGAGATTCTCACCGCCGAAGAGGGGAAGGCGCGGCCCGAAG

CGGCGGGCGAGGTACAGCGCGCCATCGACATCTTCCACTACTTCTCGTCG

AAGGCCGCCGACCTCGGCGGCACGAAGAAGGGTGCGAGCGGGCCGAACAC

GAACCTCTACACGCGACAGGAGCCGGTGGGTGTCGCGGCGCTCATCACGC

CGTGGAACTACCCGATTGCGATTCCGGCGTGGAAGCTCGCCCCGGCGCTC

GCCGCCGGCAACACCGTCGTCCTCAAGCCCGCCTCCATCGCGCCCGGCGT

CGTCATCGAAATCGCCCGCGCGCTCGACGAGGCCGGCCTCCCGGACGGCG

TCCTCAACGTCGTCACCGGACCCGGTAGCTCGGTCGGCAGCGAGTTCATC

GGCAACGAGGGCACGGACCTCGTCTCCTTCACCGGCAGCAGTCAAGTCGG

TGAGATGGTGTACGAGCAGGCCACAGACGCCGGCAAGCGCGTCCAGACCG

AACTCGGCGGGAAGAACCCGACGCTCGTCGCGGACTCCGCGAACCCCGCC

GAAGCCGCCGACATCGTCGCCAACGGCGGGTTCGGAACCACCGGCCAGTC

GTGTACGGCCTGTTCCCGCGCCATCGTCCACGAGGACGTGTACGACGACT

TCGTCGCCGAGCTCGTCGACCGCGCGGAGTCCCTCGACGTCGGCCCCGGT

ACCGACCACGAGATGGGCCCGCAGGTCAGCGAGTCCGAACTCTCCTCGAC

GCTCGAATACATCGACATCGCCGAGGCCGAGGGCGCGACGCTCGTCGCCG

GCGGCGGCGTCCCCGAGGGCGAGGCAGTCGAGACGGGTCATTTCGTCGAG

CCGACGGTGTTCACCGACGTGGACCCCGACATGCGCATCGCACAGGAGGA

GGTCTTCGGCCCGGTCGTCGCCGTCATCGAAGTGAGTGACTTCGACGAGG

GGCTGGCGGTCGCGAACGACGTCGACTACGGCCTGTCTGCCAGTATCGTC

ACCGACGACCACACCGAGGCGAACCGCTTCGTCGACGAGGTCGAAGCCGG

CGTGGTGAAGGTCAACGACAAGACGACCGGCCTCGAACTCCACGTCCCCT

TCGGCGGGTTCAAGCGCTCCTCGTCCGAGACGTGGCGCGAGCAGGGCGAC

GCCGGTCTCGACTTCTACACCATCGAAAAGACCGTCTACGACAGCTACTG

AGCGTCCCGCCCGGTCGCCGACGGCGACCGTCTGCCATCACCTCGTTATT

TTGCTCCCTCTTCTCTTCTCCTCTCCTCTCCTCTCCTCCTTCGTGTCCCA

CCCCCGCCGATGCTGTTGTTCCGGCTCGACACCTTTCCATTCTCTATATC

AGAATAGGATGGACGGAACTATTGTCGTGAAAATTACAAGCCTACTCGTG

TAATTGTCTATCGTCGTCGTCCGTCGGGAACTACGTCAATACGGTCCTGC

GTCGATGATTCGTGCGGAGCTGCTACTGTTCCGAGATAGCGAACGAGGGG

CGTCAGTAGTTGATGTTGAGTTCGACGACGTTCGCCGCGCTCAGGACGCG

TTCGGGAAGCGCCCCGTGGAGGTCTTCGTCGCTGACGCGGCTGGCCGGAG

CGGAGACGCTGATGGCCCCGAGCACCTCGCCGGACGAGGATTTGATGGGT

GCGGCGAGACAGCGCAGCCCTTCGAGACGCTCGCCGTCGTCGATGGCGTA

GCCTCGCTCGCGAACTCCTTCGAGCACCTGAAACAGCTCTTGCCGCGTGC

CGATGCTCTTCGGCGTCTTCTGTTCGAGTCCGTGACGCTCGATTATCTCG

TCCACCCGCGACTCCGGGAGGTTCGCGAGAATCGCCTTCCCGAGCGCCGT

CGTGTGTAGGTTCGTCCGAAGGCCCGCGTAGGTGTCGAGGTCCACGGCGT

CGTCGCCTTTCGCTCGCATCAGGTAGACGCCCATCCCCTGTTCTTCGACG

AGGAGGTTGGCGAGTTCGCCCGTGTCGGCGGCGAGCGATTTGACCTCTGG

CTCGGCGACCTGATACACTTCCATCGACTTCCGACTGTGGCCGCCGACTT

CGAGAAACTTCAACCCGACGCGGTAGTCGTCGCCGTCCTTGAGGACGTAC

CCGTGTTTCTGGAGCGTCGTCAGGTGGTTGTGGACCGCGCTCTTTCCCAT

GTCGAGGTCGCCGGCCAGTTCCGTGACCCCGCAGGGTCCTTTCTGCATCA

ACTCCTCGACGAGCGCCAGCGTCTTTTCCGTGGTTCGTACCGGATGTTTG

GCGTCCATGATTATTCGTTATCAAGTATCTGAATAAGTGTTCTGGTCGGG

AGAACGAGGCGCTGTGAACGCGGACTGTCGTTGAGGCGGGACCGCGGATG

CTCTCGCAGCAGATACCTGTTTCACCAGCGGCCCCTTCGTCTCGGTGGAC

TGGCCGGCCGCCTCCCGTTGTCGGCAGTCTCTCGAACAGGGGTTTCACCA

CGACCACCACTGTCCGTCCCTCGACTATCTCTCTTTCCTCAGTGGTTTTT

CTTTTGAATCACAATATTGGAAGCGGTGAACTTCCGTTCCCATCTCATCA

CCGCTCGTCTCTCGCCTTTACTTTTCTTCCACCTCTCCGTCGAAACAACA

TCCAGTGTTGTTCGGATGTTCTTGAAACTCCGGATTAACGCCCTTTGTGT

CCTGATCGCTGTCTTCCCTGTCGTGTCTGTGACTGCTGGTTTCGTCTCGT

AGCGTTGGCAGCGACTGGCAAGTGTAGACGCTTCCTCCTGCTGTTGTCGT

GCAATTAATACGCACTACCATGTTGGATGTCTTTGACCCATCGTTCAGTT

TCCGGCCCTCGCTCGTCGTGACTCGTAGTCGAGGACGAACGGTTGGCTCC

ACGGTGGTGACTCGGCATCGGGGAGCGCCGTCGGCGGAGCCGACGTTTCG

AGGCCCGCTCAGCGGGTCAGTCGTCCGCCGTGCCCGTCTGACTCGCGCCG

TCCGGTCGCTCGTCCGCGACTTCGTAGAGCGCGTTCGTCACCGTTCCGTG

TTCGGAAGCGTGCGGACGACCGGGGGAGTCGTCGTAGCCGTAGTCGAAGA

TGCGGTTCCGGTTGAGACTGAGCTTCGTGAACTCCGGTCGGAGCAGGTCG

AACAGGTCGAATCGCGATTGGAGTTCGGGGAACTGCGCTTGATAGTCGAG

AACCGCGCGTCGCGCGTGCGTCCAGAACTCCCGTTCGGGGTACGACTCGT

GTTCTTCGAGCACGTCGGCGACGTACCGGAGGACGCAGACGAACAGCCCG

GAGAAGATAAACTGACAGAGCCCTTCGGGCGGCTCTTTCCGAAGCACGTC

GTGCACGTCGTCGGGGAGCGATTCCAACTCCGGGAGCGACTGGTCGCTCA

CGTTCACGTCGTCCGCGAAGTCCTTGACCGCGAGTCGCGTGGGGACGCCG

TCTTCGACGACGAGGATGGTGTTTTGGCCGTGCGGGGAGAACACCGTTCC

GTAGCGATAGAGGAAGTGCAGGAGCGGCGGCAAGACGGTGTCGAAGAACT

CGCCCAGCCACTCGTCGAGGCTGAGCCCCGACTGTTCGACCAGCCGCGAG

ACGAAGGGTTCGCCCGCACCGTCGACGTGCATCAGCGACGAGAGCGTAAT

CGCCCGTTCGTCGTCTTCGAGGAACGTGTAGATGGACTCCCGCCAGACCG

CGCCCAGCAGTTCGTGGTACTGGTACGGCGACCCGTCGATGTCGGTGAAG

TCCGCGTGGTCGTAGTTGAGCCCCGCAATCTCGCCGGGGAGAATCAACCC

CTGCTCTTGGAGGAAGTCGTCTCGCTCGTAGATGCCTTTGATGTACTCCG

TGACCGTCGGCGCGAGTTCGGTCCGCTCGCCGGGAAGCCCCCGCCAGACG

AGCGTGTTGAGAATCCGCATCGGAACTTTCACGTGGTGTTTCTCGGCGTC

GTCGACGTTGACGAAGGTCCGGACCGACTGTTGCGGGAGGTACTCGTCCG

GCCCGTCGCCGAGCGGGACGATATCGCCGGCCGCGATGTGGTCGGGAAAC

AGCGGGACGACGGTGTGTTCCCACTGCCAGTCGTGCACCGGCATGAAGAA

GTAGGCGTCCGGGTCGTACCCCTTCGCGTCGAGGCGGCTTCGGAAGCGCT

CGTAGTGATCACCGAGTTCGCCGCTGACGAGCGAGTCGTGGTCGACCCCT

TCGGCTCCGACGAACGTCGCGTGCTCCTTGCGGACCGCCGCCCACGAGAG

CGTCACCGGCGCTTTGCGCTCGGGCGCGTACCGCTTGTAGTCGTCGTACC

CCCAGCCGAGTCGCCCCTTGTTGTACGTAATCCACGGGTGGCCCTCCATC

TCGCCTTCGAGGCGGGCGTAGTCGAGGTCCGTCGGGTCGAAGCCCCCCCG

GTCCCGCTTTCGCGACTCGATGTGGGCGTCCGCGAGCAGCGTCCGCTTGT

ACTCGCGGACGAGGTTCCCCTCGGTCAGGCCGTCGAGGTCGGTCGTCTCT

CCGAGGTCGCGAAGCAGTCGAAGGGGGTCACGGAGCGACTCCCACGTCCC

GTCGGCCCGCCGCTCGACCGTGTCGCCGTAGACGTGGAGGCTGTCCATCA

GCCGGTCGGCGGCCTCGAACCGGTAGCTCACCTCGCCGAGGTCGAACCGG

TAGCCGTGGCGGTCGTCGCCGGCGTCGACCTTCCGCGGCTCGATTATCTC

CTCGTAGGCGAACTCTTCGAGCATCTTCGTCAGGAGGCGGCGTTCGACCG

TCTCCCAGACGTCTGCGTCCAGTGCGTCCCGTAGCGTGGTGTCGTTCGGA

TTCATCGGTATCGTTAGTCGTCTTGCGTCGGCGTGGGTGGTGTCTCGCTT

CCCTCGGCGCGGGAAGCGTTCGGTGCGTGGTCGAGGAACTGCGCCACCGA

GAAGTCCTGAAAGACCGTGTCGACGTCGTCGGGATACACCTCGCGGCCGA

CGAGCCGGTTGACGAACTTCGCGTTCCGGTAGCAACCGAGGCCGAGGTCC

GGGACCCCGACGCCGTGGGTGTGGACCTCGGCGTTCTGGAGGAACACGTC

ACCCGGTGCGTCGACCGCGAGCCGGTGGTCTTCGGTCACGCCGAACCGCC

CCTGTTCGTCCCAGTCGATGGCGTCTTCCAGCGGTTCGAGGAACGCTGGA

ATCGGTCGCTCGTAGCCCGTTCCGAGAATCACGACCTCGCTCTCGTGGAC

GAACGACTCCTCGGCCTGCCACTGGTGACAGTCGAGGGCGTACGCGTCGT

TGACGGCCTCGATGTCGCGGACTTCGGTCATCGCAAACAGCCCCACGTCG

GGGTCGCGGCCGCCGACGGAGCGCCGGTAGAGCAGGTCGTAAATCTCGGC

GCTCGTCTCCGGGTCGATGCCCTTGTACAGCAGGTCCTGATTCGGGATGA

GGTCGTCTTTCACCGCCTGCGGCAGGTCGTAGACGTACCGCTCGTACTCG

GGGGTGAAGTGCTGGAGGCCGAGCTTCGAGTACTCCATGGGGAAAAACCC

GTCAGAGCGAGTCAGCCAATCGAGCCGGTAGCCGTGGTCGGGCTGGCGCT

CCAGGAGGTCCTGAAACACCTCGGCGGCACTCTGCCCGGACCCGACGACG

GTGACCGTGTCCGCCTCCCCGACGCGCTCGCGGGAACCACGATACCGCGC

CGTGTGGAACACGTCTTCGGTCGGGTGTCCACGGAGGTGTTTGGGGACGT

GCGGCCGGGAGCCGATGCCGAGCGCGAGGTTCTCGGCGCGGTACTCGAAG

CGGTCGCCGGTGTCCGGATTCCGCGCGGTGACCGCGTACCACTCCTCGCG

GTCGTCCCAGCGAACCTCGGTCACTTCGCGTTCGAACCGGCAGGCGTCGA

GTCGGTCGGCCACCCACCGGAGGTAGTCGTTGTACTCCCGCCGGGGAATC

TGGAACGTCTCGTAGAAGTAGAACTCGTAGACGCGGCCGGTCTCCCGCAG

GTAGTTGAGGTAGCTGTGGGGGCTCGTCGGGTCGGCCAGCGTCACGAGGT

CCGCGAGGAACGGCACTTCCAGCGTCGTCCCCTCGATGAGCATCCCCTCG

TGCCACGCGAACTCGGCGTCCCGTTCGAGGAACGCCGCGTCCACGTCCTC

GGGCGCGCCGTCAAGCAGGGCCGCCAGCCCGAGGTTGAACGGGCCGAGAC

CGATTCCGACCACGTCGCGGACGCGGGGCTCGCTCATCGGCCCACCTCCG

TCTCCGCATTCTCGGCCACCGTGTCGAAGAAGCGGTCGCGCTCGCAGTGC

ATCAACAGCGCCTCCTTGTCGGGGAGGTCGATGCGCCCCTGCGGCTCGTA

GCCGCATCGCTCGAAGACGTGGATGACCCGCTCGTTTCGCACGTCCGGTT

CGCTGACGACGCGAGTCGCGCCGGTCGCGCGGAACTGAAACGCCGTCATC

GCCCGCAAGAGCGGTATCGAGTGGCCGTGTCCGAGATACTCCGGCGGCCC

GATGAGAAGGTGGACGCCGCGGTCTTCGGGGGCGGCGTCGTAGCACGTGG

CGACCACGTCGTCGGCCGCGCGGTACGCCTCCCAGTAGCTCATGGGGGTG

TGGTCGAGCAGGCCGACGTACGGCGTGAGATGGTCGTCGGCCAACTTCTC

GTCGAGTTTCGCCCGAAACGCCGGCTTCGGAAGGTCGAGTTGCCAGTACG

GCTTCACGTGGTCCGAGCCGAGCCACGCGTGAAGGCGGTCGACGTCCAGC

GCCGGGTCGACTTCCGCGAAGGCGATGGTCCGGTCGATGGTCTCGTCGCG

GACGCGATACGCGTATTCGCGGCCGGCGTCGGCAGTCGCCGCGTTCGCGG

TCGCGGTCGCCGGTGCGTCGGTCATGGCGCTGGCACCTCCAGTTCCGTGA

CGAGCGGGTTGTCGATGTCGGCGTACACCGACTGGTTCGCCAGCGACCCC

TCCAGCTCGTCCATGTCGTGGAACCGCGTGAGCAGGTTCGCCTTGCACGG

AATCGTCGGGTTGGTGAGGAGGTCGTCGAGCAGCGACGACGACGGCCGGT

CGTACTCACGGAGCGATTCGAGTTCGTCGCGGAGGACGCCAAGGAGGCGT

CGCTCGTCCACCAGTCCGGCCGTGCCGAAGGCGTTGACGAGGCCGAAGGC

GTTGTTGATGACGACGTAGTACCGGATGCGCTCGTCGGCGACGGCGTCCG

GACAGACCGTGTCGGCGCGCTCGCCGATGCCGGGCAGGAGCGCGTCCACC

TCGTCGTACATCGACTCGGGGAGGTAGTAGCCCTGATTGTCGCGGTAGCG

GAACGTCTCGGGATAGCCCCCGTCGAGGGTCAGCACGCTGTTTTGCTGGT

GGGCCTCGACGCCCAGCCCGCGTTCGAGATACAGCCACAGCACCGGTCGA

ACCGACAGTTCGAGATACTGTCTGAACCAGTCTTCTGCGACCGCCGGAGT

GGCCCGTCCCTCGTGGTCGGCGATGCCCTCCACGAGCGTCGCCAGCCGCG

ACGACCCGTCGTCGAGGTGGTCCTGACAGAGGCCGACCACGGGCGTCGCG

TTCGCCGCCGCGTTGCCGCGGAAGGGATTCTCGCGCAGGACCGTCTCGAA

GCCGGACTCGCCGTCGCCGAGGTCGACCGTCAGATACGCCGGGTCGCGGA

CGACGTCGAACGCCGGGAACCGGGATTCGAGGTCGCCGCCGAGTTCGGTG

TCCAGCAGTTCGGTGATGGCGACGCCGCGCTCCAGTTCCGGGAGCTTGTT

CGTCCGCTCGGAGTTGGTGACTTTGACGTTCAGCGACCCCTTGACCATGA

ACGGCGCGTCCGGGGCGTACAGCGTCCGCACCGACGAGGTAGGGTAGAAC

TCCCGGCCGACTTGGCCGACGTATTCGAGGCCGTCGCCGAGGTGCGCTTG

CACCTCGGGTTGCGAGCGGAGGTAGTCCGCCTGCCACGGGTGGACGGGAA

GCAGCACCTCGTCGCCCTCGACGTGTTCGGTGACGAACGACTCGGGGACC

GAGTCGTCCGCGCGGAGGGCCGACTTGACCCACTCGGCCGCCGACTCCTC

GCGAGTGGAGTTCTGCGAGACGAACTCGGGGTCGGCGCTGAAGTATGCCA

GCGGGAACGACCCGCGGAGTTCGGGCGCGTAGGCCCGCGACTCGTGAGCC

GGGATACCCTGTCTGCTCTTGGGCGTCGGGTGGAGCGGGTGGCCGAAGAC

GAGCGACTGTTCGGCGTCGCGGAAGGTGGTGTCGAAGCCGTACAGTCGCT

CCTCGTCGCCGCGCCGGGCCTCGACGAACCGCTCGATGTTCTGGCAGCTC

TTGACGACCCGCAGGAGCAGTTCGTCGGCCGCGCCGGTTTCGTCGCGGTC

GAGTCTGAGTTCGCTCGTGACGAGCGTCACGAGCACGGTGTAGTCGAGGG

GGAACGTCTCGCCATCGGGAAGCCGGTAGACGCCGGGCAGGTCGAACTGG

TGGCGACCCGTCGGCGACCGGTACGAAAGCGGGACGTAGAGGTCCACGCC

CTGCTGAGAGAGCGGCGCGTGGACGACTACCTCGGCGTCCGCCTCGACCG

GCACGTCCCCGGCTGGGACGACGGCGTAGTCCCCGGTCTCCCGGAGGTAG

CAGTTCAGGAAGCTGTGTATCGTCGCTGACTCGGCTGTGTCGTTCGGATT

CATGCGTGTCGGTCGTCGGTCGTCGGTTCGAGAGCGGTTCCGCGGTCCGA

AATCTCGCGCAGGAGGTCTCGGAGGTCCTCGCGCGTCGTTCGGGGGTTCA

GGAGCGTCAGCTTCAGACACGTCTCGCCGTCGACGGTGGTGCGGGCCACC

ACGGCCTCGCCGTCGTCGAGGAGGGAGTCGCGGATGGCCTCGTTCAGGTC

GTCGGCGCTCGGGCCGTTGACCGGGCCGTCGGCCGGGCCGCCGCCGTCTC

GTCCGTGGTCGGGGACGTACCGGAACACGACGACGTTCAGGTCGGAGTCG

TGGACGAGCCGCAGGTCGGGGTCGGCCTCGACGAGCCTCGCGGCGTCGGC

GGCGAGGTCGATGGTGTACTCGACCATCGACGCCAGCCCCTCGCGGCCGA

GCGCCTGCATCGTCACGAACGGTTTGAGCGCGTCGAATCGCCGGGTGGTC

TGGACCGACTTCGAGACGAGGTTCGGCACGCCCGCGTCGTCGTCGCGCAC

GGGGTTCAGATAGGCCGCGTTGCGGTCGATGAGGTCGTACGCCGACGCGT

CGCGGACGAGCACCGCGCCGCAGCTAATCGGTTGGTAGAACAGCTTGTGG

AAGTCGACCGCGACGGAGTCCGCGGCCTCGATGCCGGCGAGCTTGTCGGC

GTGGTCGTCGCTCAGGGCGAGCGCGCCGCCCCACGCGGCGTCGACGTGGT

ACCAGCAGTCGAGTTCGGCCGCGCGCTCGGCCAGCGGGCCTAACGGGTCG

ATGCTTCCGAAGTCCGTCTTTCCGGCGGTGGCGACGATTGCGAACGGGCG

TTTCCCGCGCTCGCGGAGGTCCGCCACCGCCTCGTCGAACGCCGCCATCG

ACAGGCGGCGGTCGTCGTCGGTCGGGACGGTGACGACGGCGTTCTCGCCG

AGGCCGAGGTGCGAAGCGGCCTGCGTGGCGGTGAAGTGGGCGTCGGCCGA

ACAGAGGATGCGCAGGTCCCGCGCCTCCGGCGGTAGCCCCTCGTGCTGGA

CGTCGACGCCGTACTCCTCCAAAACGACCTTGTTGCGGGCGAGGAGCAGG

CCGACGAAGTTCGACTGCGTGCCGCCGCTCGTGAACACGCCGTCGCCGTC

CTCGTAGCCGAACAGGTCGCACAGCTCCCCGACGAACCGCCGTTCGAGCT

GCGTCGCGGCGGGGCTCTGGTCCCACGAGTCCATCGACTGGTTCGTCGCC

GTCAGGAGCGCTTCGGCCGCGAGCGCCGGAATCGTCGGCGGACACTGGAG

GTGCGCGATGCAGGTCGGGTCGGACACGCCGACCGAGTTTCGCAGAATCG

GCTCGGTGCGGTCGAGCGCCGCGGCCACGCCGTCGCCCTCGTGGGGCAGC

ATCTCGAACCGGGCGAGCGCGTCGTCGACTTCTTCGGGCGTCGCCCCCGA

GTACGGCGTCGCCTCCGCGGCGAACTCGTCGAGGACGAGGTCGCACGCCC

GCCGCATCGCGTCGCGGTAGCGCGCCCGGTCGTCGTCGTCGCCGCTCAGG

AACCACTTCGCGGCGTCCGGTCGCGGGTCGCGGCGCTCGCCGTCGACATC

GCCCACGCCGTTCATGCGGCGACCTCCCGGCGGCCGTCGGCGTCGGCGTC

CTCGATTGCGGCCTCGACGGCGTCCGCGAAGATGCTCCCGACGGCGTCCA

CGTCGCTCGGACTGACGACGAGCGGCGGGAGGAACCGGACGACGCTCCCG

TGGCGGCCGCCGAGTTCGAGGATGAGGCCGCGGTCGAAACACGCCTCGCG

GACGGCGTCAGCGAGGTCCGGATTCGCCGGGTAGCGCCCGCAGGCGTCGG

ATTCGCCCGCGGGGTCGACGAGTTCGACGCCGAGCATCAGCCCGCGACCG

CGCACGTCTCCGACGGCGTCGTGGGCGCGCTCGGTCGCGTCGAGGTGGCC

GCGGAGCCGCTCGCCCATGCGCGCGGCGTGGTCGTCGAGGTCGTGTTCGA

GGACGTGTTCGATGGTGGCGATACCGGCGGCCATCGCCAGCTGGTTTCCC

CGGAAGGTGCCGGCGTGCGCACCCGGCTCCCAGACGTCGAGCGACTCGTC

GTAGACGACGACCGAAAGCGGGAGCGACCCGCCGATGGCCTTCGAGAGCG

TGACCGCGTCCGGGACGATGTCGGCGTGCTCGAAGGCGTACAGTTCGCCG

GTCCGGCCGAGACCGGTCTGAATCTCGTCGACGACCAGCGGGATGTCGCG

TTCGCGGGTCATCCGCCGCACCTCGCGGGTCCAGTCGTCGGGCGCGGGAA

TCGCGCCGCCCTCGCCCTGCACGAGTTCGACCACCATCCCCGCGGGCTCG

GTGATGCCGCTTTCGGGGTCGTCGAGGGTGCGCTCGACGTACCGGGCGGC

GAGTTCGTGGCACTCGTCGCCGCCGACGCCGAACGGACACCGATACTCGT

ACGGATACGGAAGGTGGTGTACGTCGGGCATCAGGCCGGGGACGTTCTCC

TTCGGGTCGGTGTCGCCCATCAGGCTCAGCGCGCCGTTGGTCATGCCGTG

GTAGCCGCCCTGAAACCCGAGAACGCTCCGGTTGCCCGTCGCGGTTTTGA

CGAGTTTGAGCGCGGCCTCGACGGCGTCGGTCCCGGCGGGGCTACAAAAC

TGCACCCGCGCGCTGTCAGCGAACTCGTCGGGGAGGCTGTCGAACAGCGT

GTCGACGAACCGCTCCTTGACGGGCGTCGTGATGTCGAGGGTGTGGAGCG

GGCGGTCCTCCGCGAGGACGCGTTCCATCGCCTCGACCACTCTCGGGTGG

TTGTGTCCGAGCGCGAGCGTGCCGGCCCCCGCGAGGCAGTCGTAGTACTC

CTCGCCGTCCATGTCGGTGACGGTGACGCCCGAGGCCTCCCGTATCGCGT

GCGGGAGGTGTCGGGGATAGGTCCGCGCGCTCGACTCGCGGGCGGCCTGC

TGTTCGAGCAACGCCTCGTTGCCGGCTGCCGGGTCGGTCATCGCGCTTCC

CTCCGTTGATTCGTCGTCAGTCTATCGCTGTCGAGCATGCATGTTTTAGG

CCGGCCTAAAAGCACAAAAGGTTTACTGCAATCTGATTTTCAGTGAAAAT

TTCGGGACAGCGCAACCGCTCGTCGGCCGCGACGACCGACTCGCGGCGAC

CGGTCCTACGGTCCAGTTGGGTGCTCTTCCGACGGACGGAGTCGGTGGTC

GGTCGCGTTCCGGGGTCCGCCCCGCCCCACCCCGCCGCGGACTCACCTCG

CTTTCGCTCGGCGGAACACCTCCATGAGCGGGGGTTCTCCCTCGACGAAC

CACGCTGCGGTGACCGCCGGGGACACGCCCATATCTTGGCTCACTGCCAT

ATTTTTAGGATAGCCTAAAATATGATAAGCGTTCTGGCGGGACGCGCCGC

TTGACGGGTGGGCGAAGAGAGAAAGCGGGGCCGCGAACCCGGGGCGAGTT

ACAGGTTCCCGTTGGCGATGTCCGCGACGCGCTGGGCGTCGAACAGGCGC

TCGTCGGCGTCGTACAGGGTCCGCGCGAGGCGGTCCGTCACGACGAGGTT

CGTGATGGGGCCCTGATAGAGCGCGCCGGCGCGGTAGACGTCGCCGTTCT

GGACGGCGGTGAGTTCGCCCGCCACCTCGTGGTTTTCCATGAACGAGACC

ACGGTGTTCTGGAACTCCTCGCGTGTCTTGGCCTCCTGTCCGCGCACGAG

GAGCACTTCGGGGTCGACCTCAAGCAGCGTCTCGAAGTCGACCGCGCCGC

GGTTGCTGTAGAAGTCCCGCACCTCGGTCGTCGCGAGCGCGTCGTTGACT

TTCAGGTCGTTGAGATGCTTGAAGCTCGTCCCCTCGTCGATGACGTAGGG

GTAGAACTCCTCTGGCTCGTCGCCGCCGGCCCAGACGACCGCGGCCGAGG

GCCGCTCGCCCTGCGCGGGGACGACCGGCGCGAGGTTCGACTGGAACTCC

TCGTGGACCTGTTCGAACGCCTCGAAGCGGTCCTCGCGCTGGAACACCTG

CGCGAACTTCTCGAACGCCTCGTAGAGAGTGTAGTAGCGGTAGTCCTCGT

GCCACGCGTAGCCGCGCGAGAAGATGCTGTTGCCGAAGAACGGCCCGACC

TGCGTGCTGATTTCCTCGATGTCGGCCTCGCTCCACTGGCCGCGGTTCAG

GAGGAAGTTCGGGTCGGCGATGTGCACGTCCGCGTTCAGCTCGTAGAACT

GCTCTTTGCCGACGCCGCTGTCGCCCCACAGCTGGCGGATGGACGACTTG

TCGACGCTCACGTCCGGAATCTCGTCGTAGTACTGCGTGTGGTACCGGCC

GGGGAGCCAAACGCCCATCGGCGTGTCGAGCCCGAGCGCGACGCCCATGT

CGGCCCAACTGCCGTTGTTCGCAATCCACGCCTCCGGGACCGAATCGAAC

GTGACCTCGCCGACCGGCTCTATCGACACCGAGTAGGACTCCGCCGCCTC

GGTCGTCGTCTCTGTCTCGGTCTCCGTCGCCGCCTCGGTCGTCGCGGCCG

TGGTCGTCTCGGGCTCGGACGCCGTCGTCGTCGTCTCCGACCCGCTCCCG

CCGGTACAGCCGGCGAGCAGGCCGCCTGCGATGACCGCGCCGCCGTACTG

GAGGTACTCGCGCCGCGTCGATTCCCGTCCGCTTTGTCCGTCGTTTGCCA

TGTCTTTTAGGCCTCCCTAAATGGTTAAAACGTCTTCGGAATTTAGGCGA

GCCTAACTACCTTCGCGCGGGGCGGGGGAAGGAGGCGGCTCAGTCCGCCG

CGTCGACGGGCGCTCTGACGGCGAGCACCGAGAGGTCCGAGTACGGCGTC

GAGTCGGGGCCGCTCCCGCCGGCCGAATCGCGCAGGCCGCCGAGCGTGGT

CCGGGTGATTCGCTCGTCGTCGTGGGTCAGTCGTTCGAGCACGAGCGCGT

CCAGCGACGCGTCTGCGTCCGAATCGAGGAGGAACTCGGCGATGTCGCCC

GGCATCCAGTCGAACGGTCGCGGCAGGACGAGCAGGTGGCGCTCGCCGGC

CGCCTCGGCCAATCGGTCGAGGTCGGCGTCGAGCGACCCGCTTTTGTGGA

GCGTGACGAACCGCGTGTCCTCCATCGGCGTCCGGGCGCGACTCGCGGCT

ATCTGGAGCGACGAGATGCCCGGAATCACCCGCACCGGTCGCTCGACGGT

CCGCTGGACCTTTCCGAGGAACTGGTAGCCCGAGTGGTTCGGGTCACCCA

TGAGCACCGCCGTCCCCCGGTCGCCGGCCGCGACGCGCCGCCCGAACTCG

GCCAGCGCGTCGGCCTCGTCGGCGTAGCCGCAGGTGAGCAGGTCGGCGTC

GGTCTCGTCGGCGACGAAGTCGACGACCGTCTCGAAGCCGACGACCACGT

CGGCCTCTCGAATCGCCTGCCGCCCGCGGGGCGTCAGATACTCAAGATTC

CCGGGGCCGACGCCGACCGCGTAGACCGGCGCGTCGGCGTCCTCGCCGCC

CCCGTCGGTTTCGTCGATGCTCGGTTCGGGCGCGGCCGCGGCGAACGCCG

CCGGGTCAGGTCCCGAATCGAGGTCGTACGGGTCGCTCTCGTCCTCACTC

ATGATTTGCGAGGTCCACGTCGCCGTTGCGAACGTCGCTGGCGACGTGAA

TCAGTTCGTTCGTGAGCGCGGCGGCGAGACCGCTTCCGCCGCGTTTCCCG

ACGTTCGTCACCGCGGGCACGCCGTACTCGTCGGCTACGTCGCGGACGCG

CTTCCTGCTTTCTTCGGCCTTCACGAAGCCGACGGGCGTCGCCACGACGG

CCGCCGGTCGGGTTCCGTTCTCGATGCAGTCGCAGAGCGCCAACGCGGCG

GTCGGCGCGTTGCCGACGACCGCGATAGCGCCGTCGTAGACGCCCTCCTT

GTCGAGTTCGAGCACCGCGGCCGCGGTCCGGGTCATGCCCGTTTCGGCGG

CGAGTTCGGCCCCGTTGCCGATTGCCTTCCGCACCTGGCAGTCGTGGCCG

CGGCCGGTGATGCCGGCTTTCGCCATCGTGATGTCGGTCACGACGGGGCG

CTCGTCGAGGACGGCCCTCGCGCCCGCGACGACCGGGTCGTTCTGGCACC

GGATGAGGTGCTGGAACTCGATGTCGCCGGTCGAGTGGACTGCCTTCTGT

CGGAGTCGGTCGTTAAGCGTCTCGTCGGGGACGAACGTCCGGACGATGTC

CATGCTCGTCTCCGCGATGTCCATCGCTTCCTGCGTCGTCGCGCCGAGGT

CGGCGTAGGCGTCTCGTTCAGTCATTGGATTGGGTCTGCGTCATCGCGTT

CGTCGTGTTCGCTTCGAGGTCGCCTTCGACGGAGAGGTTGATGTCCTGCA

ACCGCTCTCTCACGTCGTCGTCGGCGTCCCACAGGTCGCGGTCGATGGCT

TCGAGGAACGTCGCGGTGATGCTTTCGAGCGCCCACGGGTTCACGTCGCG

CAGCCACTCCTGTCGGTCCTCGTCGAAGGCGTAGGCGTCCGCGAGGTGCT

CCCAGAGCGTGTCGCTGACGACGCCCGTGGTCGCGTCCCAGCCGAGCGCG

ACGTCGACCGTGGTCGAGAGGTCGCCCGCGCCCTTGTAGCCGTGTTCCTC

CATGCTGTCGAGCCAGTCGGGGTTGAGGACGCGCGCCCGCATCGCCTTGC

GGACCTTCTCCTCGTTCGTGTAGACGCTGACGTTGTCGGGGTCGCTGGAG

TCGCCGACGTACGAGGCCGGGTCGCTCCCGGAAATCTCACCGACCGCGGA

GACGAACCCGCCGTGGAAGGCGTACCAGTCGGAGGAGTCGAACTCGTCCT

GTTCGGCGGTGTCCTCGATTTTCACGGTCGCCTCGACCCCGCTCAAGCGG

CGCTCGAAGGCGTCGTGAGCTTCGGTCACCTTGCCGCGACTCCCGAGGGC

GTAGCCGCCCCACTGGACGAACACGTCCGCGAGGTCGCTCCGGTCGTCCC

ACTCGCCCTCGTCGACGGCCTTGTTCGTCCCCGCGCCGTAGCCGCCGGGC

GTCGTCGTGAACACGCGGTGTTTGGCCAGTGTCTCGGCCTCGTCGGGGTC

GACGCCCTCCGATTCGAACTCGGCGGCGTCCTCCTCGACGTGCTTTTTCA

CGTAGTTCATCTCGTGGGGCTCGTCGAGGTCGACCACCGCGTCGACGGCG

TCGTTGACGACGCTCGCGGCCTGCGGGAACGCGTCGCGGAACAGCCCCGA

GACGCGGGTCGTCACGTCGATTCGCGGCCGGTCGAGGTCGTCCAGCGGAA

TCGGCTCCACGTCGTCGACGCGGCCCGCGTCGGTCCACACCGGTTCGACG

CCCATGAACGCGAGCACCTGCGCGATGGTCTCGCCGCGGGTCCGGACCGT

CGGCGTCCCCCACGCGACGACCCCGAACTCCTCGGGATACTCGCCGTGGT

CGTCGTAGTGGCGGTCGAGGACGCCCTCGGCGACGCGCTCGCCGACGCTC

CACGCCGGCTTGGCCGGGACTTTCCGCGGGTCAAGCGTGTAGAAGTTCCG

CCCGGTCGGCAGCAGGTCCACCCCGCCGCGGGTCGGCGCGCCGGAGCCGC

CGGGGCGGACGTACTCGCCGTCGAGGGCGTCGGCGGTCTGCGGAATCTCG

TTTTCCGCGGCCGCGACTCGCGGGGCTGCCTCGTCGCAGATGAACGCCAG

CACCTCGCGGAGGTCGTCGTGCGCGCCGCGCTTGGCCCGCGCGTCCCCGA

GTTGGTCGATATCGACCACGAGCAGGTTCATGTTCACCTCGTCGTCGGGG

CCGGCGTCGACCTCCGACTCGGGCACGTCGAAGTCGCGTTCCGCGAGTGC

GGAGACCAGTTCGACGCACTGGTCGTAGACGCGGTCTGCGGCCTCCGAGA

GATACATCCCGAGGTCGTCGTCGTACGTCCCCGGCTCGTCCCGCATCCGG

TCGTAGTCGACGCCCATGACGCCCGCGACGCTCTCGCGGAGACTCGGCGT

GTCCGCGTTCGGGAGGCGGGTGAGCGCGACGAGGTACTCCACGAGTCGGT

CGTTTGCGGGCGGTTCGCCCATCGTGTGGAGGCCCATCCGAATCTGCGTC

GTCTTCACGTCGGTCAGGTACTCGTGGACGCGCTCGACGAGTTCGTCGAA

CTCGACTTCGGCCTCGTCGGAGCCGACATCGCCGTCCGCGCCGCGGATGG

CCTCGGCGTCGTCGAACCCGAGTTCGGCCGCGAGGTCGAGGTCGTCGACC

GCGTCGACGAGGAGTTCGCGGAGTTGGTCGCCGCGCTCGGGGCGGGCCTC

GTCCATGCCGGCCTCGCGGTACTCGCGGGCGAGTTCCTCGAGGTCCGCGA

GGTCGTCGTAGGTTCCCGCGGTGCGCATGACCGGCGTGAGGTAGTCCACG

ATGGCCGCGTACGACCGGCGCTTGGCCTGCGTCCCCTCGCCGGGGTTGTT

GATGATGTACGGGTAGACGTTCGGGAGGTCCGAAACCAGTTGGTCCGGCG

CGCTCTCGCCGTTCAGCCCGACGGTCTTGCCGGGGAGCCATTCGAGGCTG

CCGTGGGTGCCGAGGTGGACCACGGCGTCGGCCTCGAACGACTCGCGGAG

CCACGCGTAGAACGCCACGTAGTCGTGCGGCGGCTGGAGGTCCGAATCGT

GGTACACCTTCGAGGGGTCCATCCCGAAGCCGCGCGGGGGCTGGACGGTC

ACGAGGACGTTCTCGAACTCGACGCCCGGAATGGCAAAGGGTCGCTCCGG

CGGGTCGCCCCACTCCTCGACGACGTTGTCGCGGAAGCGGTCGTCGGCGT

CAGCGAACCAGTCGGCGTACTGGTCGGGAGCCACCACGTCGACGCTCAGG

TCCCGCACGTCCTCGGGGGCGACCCAGCGGTCGTCCAACGTCAACTGCGA

GGTGAGGTCGTCGATGAGCGCCTGCCCGTCCGCGGGGAGGTCACCGACGG

CGTAGCCCCGCTCCCGGAGTTCGGACAGGAGATTGACCGTGCTCTCGGGG

CTGTCCATCCCGAAGGCCGTCCCGATGCCGTCGTCGCTCGGCGGGTAGTT

GTGGAGGACGACCGCGACGTTCTTCTCGTCGTTCGGGAGGTGGCGGAGCC

GCGCCCAGTTGACCGCGAGGCGGGCGACGTGGTCGACGCGGTCCTCGATG

GGGAAGTGCTGTTTCGGCGCGCTCCCGACGCCCGCCTCGTCTTCCATCCG

CTCTTTCCCGCTTATCGGGTGGGTGATGACGTTGCCGTCGAACTCGGGGA

GCGCGACCGACAGCGCGAGTTCGAAGCCCATCACGCCCGTGTCGCTCGAC

TCGTACCGGCTTCGAGAGCGCATGGTCGTGATGGCCTGCAACACGGGGAC

GCCGAGTTCGGTGAGGAACACGTCTTCGGCGGCCGACCCCTCGTCGTCGG

CGTCGCGGCCCCGCTCGCTCATCGACAGCGAGAACATGAAGGAGCTGACG

ACGGCGTCGACGACGGGGCCGTCGTCGTCCGAAAACCAGTTCCGGGCGAC

CCACTCGGCGTTCTCCTGTCCCTCCTCGTCGGTCGCGGGGTTGCAGAACG

CGGGTAAGACGTTCGCGCCGAGCGATTCGAGGCGCTCCACGAGCGCGTCC

ACGTACCGGGTGTTCGCGTGCGTCCAGTGGGACTCGTAGAACCAGACGCC

GATGGTCGGCTTGTCCGGGTCGTGCGTGTCGAGCAGTTCGTCGTACTCGA

CGCCGGGGTAGTCGGGGTGGTAGACGCCCTCTGTCGGGAGTTCGACAGGG

TCGTCGACCTCGGTCTCGACGCCGGCGTACTCGGCGGCGAGGAACCGACA

GAGGTTCTCGACGTTCACCGCGCCGCCGCGGTCGAGGTACTCGCAGACGC

GCTCGCGGTCCGCGTCGGCGACGGTCGTGTCCCGGCGAGCGAAGGCGTCG

CCCGTGGCCTTGACGACGAGCGGCACGCCCGCCGCTTCGAGTCGTTCGAC

CGCGTGGTCGTAGCCGGGCATGCTGTCTTCGGCCCCGTGGAGCCAGAAGA

CGGCCGCCGTCGTCGACTCCAACTCGTCGACGAAGGCGTCCGCGTCGCTC

ACGTCGTCCAAGTCGCTCGCGGAGCGGACGACGAGGTCGATTCCGTCGAG

TCGGCGCGCGGCCCGCTGGACCGCGCCCAACTCGTTTTCGGTCGCTGTGT

ACAGTCCGATTGTCGGTGTCATTGATAAAGTGTTATTGTCTTACTGCAAC

TACGTTTTCGTATTCGATGACGACTCAGACGCTTCCGTTCTCGGCTATCG

TCGGGCAAGACGAACTGAAACAGGCGTTGTTGGCGGTCGGAGCCAACGAC

GACCTCGACGGACTCCTCGTTCGCGGCGAGAAGGGCACCGCGAAGTCGAC

CGCGGTCCGCGCGCTTTCGGACCTCCTGCCCGAACAGGCGGTCGTCGCGG

ACTGTCCGTACGGCTGCCCGCCGGACCCGGACGACCCGGCCCGCCAGTGC

GACTCGTGTCGGGCCCGCGACGACCCCGCGGTCGAACGCCGGTCGGTCCC

GCTCGTGACGCTCCCGCTCGGCGCGACCCGCGACCGGGTCGCGGGCACGC

TCTCCGTCGCCGACGCGCTGGACGGCGAGGCGTCGTTCGACCCCGGGCTC

TTGGCCCGCGCGAACCGCGGCATCCTCTACGTGGACGAGGTGAATCTCCT

CGACGACCACCTCGTCGACCTGCTCTTGGACGCGGCCGCGAGCGGCGTCA

ACCGCGTCGAGCGCGACGGGATGAGCGTCGAACACCCCGCCGAGTTCACG

CTCGTCGGGACGATGAACCCCGAGGAGGGCGACCTCCGCCCTCAGCTCCG

GGACCGCTTCGCCCTCCAGGCGACCGTCGTCGGCAGCCGCGACGTCGACG

ACCGCGTGGAGATTATCGACCGGGCGCTGGCGGGCGGCGACGACCCGGAG

ACGGTCGCCGACGCCCACCGCGAGGGGACCGCGGCGCTCGAAACCGCGCT

CCGCGAGGCGACGGCCCGCCTCGACTCGGTCGCCCTCCCGACCGAGTTCA

AACGCGACATCGCCGAACTCTGCGTCGACGCCGGCGTCGAGGGCCACCGC

GCCGACATCGCCATCGCCCGCGCGGCCCGAACGCTCGCGGCGCTCGACGG

CCGGACGAAGGTGCTCGAAAGCGACGTGCGCGAAGCGGCCGCCCTCGCGC

TCCCGCATCGACTTCAGAGCCGGCCGTTCGACGACGACCCGGACGTCGAC

GACGTGCTCGACGACCACTTCGACGAGGACGAAGACCGGGACGACGACGG

GGGCGGCGACGCTAACGACGGGGACGGTGAGGGTGACGATGCGGGCGATG

CCGACGACGGCGGGAGTGACGACGGCGACGCCAGCGACGGCGAGGAACGC

GCCGAGGGAGGCGACAGCGACGCCGACGCCCCCGGCGCGGCTCCCGACTC

TGATGCCGGTGGTGACGACCGAGGCGGCGAAGTCGGCGGAGCCGACGAGC

GCGGACCCGAAGACGGTGATTCGGAAGGCGGAAATTCCGAAGACGGCGAT

ACCGACGGCTCCGGCGACGACCCCGACGAGGCCACGCCGCTTCTCCCCGG

TCAGAACCGGGCCGCCGTCGGCGACGCCGAGCGCCCGCCGGTCGAGGACG

CCGCCCTCGACGCGGAGACGCCGGGTGACGGCAGCCGCGCCGCGGTTCGG

GAGTCGCGTCAGGGTCGAGGCTCGCGGGTACGAACCGAACCGGCGACCGA

GACCGACGACATCGACGTGCCGGCGTCGGTCCGGGCCGCCGCCTCGGCCG

GCCGGTCCCGCGTCACCCGGTCGGACCTCAGAACCGCGGTCTCCCGCGGG

TCGGCCGCGACGCTCGTCGTGTTCGTCGTGGACGCGAGCGCGTCGATGCG

CGCCGCTATGCGGCAGGCGAAGGGAACCGTGCTGTCGCTCCTCGAAGACG

CCTACGAGCAACGCGACGAGGTCGCGTTCGTCGCCGTCGCGGGCGACGAG

GCCGAGGTGTTGCTCCCGCCGACCGACAGCGTGACGCTGGCGGCGCGGCA

CCTGAAGGAGCTTCCGACGGGCGACCGGACGCCGCTTCCCTCCGGGTTGG

ACGCCGCTCGCCGGGTCATCGACCGCTCGGACGCCGACGCCGCGCTCGTC

GTCGTCGTCACCGACGGCCGGGTTACCGTCGCGGACGGCAGCCCCACCGG

ACAGACCCGGTCGGCGGCGCGGAGCCTCGCCACCGCCGACGCGTCGGTCG

TCGTCGTCGACGCCGGCGATGACGGCATCGGCGTGACCGACATCCTCGTC

TCCGAAACGGACGCGCGGCGCATCCCGCTTTCCGACCTGTCGGCCGAGCG

GGTCGCTGAGGCCGAGCGCTTCGCGGACGGGCAGTAGCGGGGTATCGCTC

GTCTCGTGTCGCATGGTTTCTTCTATCGAATCGGTCGCGTGCGGAGTCGG

TAGGCTCCGCGCGTGACGCCCTCGTCGTGGTACTCGACCGGCTCTTCGAC

CACCGCGAGGACGCCCCCACCCGCGGCGACCGCGGTCGGGATGCCGGGGA

GGTCGTCGGTCCGGACGTGCCCCGCCGTCGCACCGAACGTGTGGACGCCG

TGGCGGTCGGCCTCTCGCTCCCGGAAGTGCTGGGCGGACGGTACGAAGAC

CCGCGAGCCGGCCGCGACGGCGGCGCGCGCGAACCCGCCGGTGTCGCGGG

TCCACAGGCGCTCGCCCCCATCGACGGAGACGCCGACCGCGGTGTGTTCG

TCGGGATGTCGCGCGTCCGGGTCGCGGGTCGACTCCGCGAACGTGTTCCC

CGTGACGAAGGCGGCGGTTTCGCCGGCGACGCAGACGTGGGTCGGATAGG

CGTAGACGGTCTCGTCGCCGACGCGCCGCTCGGAGGCGAGGTCGATTCGC

CACCGCTCGTTCCCCTCGAAATCGAGGAGGTAGCCGCGTTTGTCGCCGTG

GCTGGCGACGGCCAGCGCGTCGCCCGCGAACGCCACGTCCCCGACCCGCC

GGTCGCCGTCGGTGCCGGGGTCCCACGACGCGACTCGCCCTCCGGTGTGG

GCGTCGAGGAGCACGAGACCGTTCTGTTCGGGGTTCGGACAGCGGTTGTA

CGCGACGGCGACGCGGTCCCCCTCGGCGGCGACCGCGATGGGCGAGGCGT

GGACGCGTCTGCGCCACGCGACCCGGCCGTCGCGGCCGAACCCGAGGACG

ACGCTCCGCCAGACTCGCTCGTCGCCGTCGCGTTCGTACCGTCGGGCGGC

GACGACGACTCTGTCGCCCGTCACCGCGAGGTCGACGACGTAGGGTCGGA

AGAACAGCGAGTCTCTCGCCGCGGTGCCCACGTCGTCGGTCGTCGCGTAC

GTCCAGCGCGCGTCGCCCGTCTCGGCGTCGAGGACGCGAACGAGTCCGTC

GCCGCCGCGCTCGCCGACGACGAGGAAGTCGTCGGCGGCGGCCATCGACA

CGACGGGGTTGTCGCCGCGCGCCTCCCAGCGGTCGTCGGAGTCGGATGCG

GAGTCGGCAGCGGCTTCCAGCGCGACGACGCGGCCGGCCGCGGTTCCGAC

GAAGACGGCGTCGTCGGTCGCGGCGACCGCCGAGCGCGTCCAGTTGTGGC

GACTGCTCGCGGGGTCGACTTCACCGAGGTCGCGGCGGGCCGAGAGTGGA

GCCGATGGCATCGGTTCAGTCCTCGACGGGGAAGGCGTCGTGAAGCGTGT

GGTGGGCCTCTCCGAGGTCGTCGAGCAGCGCGTCCGTGCCCTCGCGGAGA

CGGCGAAGCGACCGTTCGGCGTCGCGGACGGCGACGAGTCGGCCGCGGAG

GTAGCCGGCCTGCGGGTCGTCGTCGTCGATTCGCGTCGCCTCCGATTCGA

GGTCGACGAGCGCGGCGTCGAGATGTCGCTGGGCCTCGTCGAGCGTGGTC

GCGTTCGCGAGCGCCTGAATCGGGCCGGGGTCGGCGTCGCCGAGGTAGTC

TTCGAGTTCGGCTTCGGCGTGTCGCTTCGCGTGTTCGTCGGTCGTTCCGG

TGATGTTCAACAGCGCCAGTCGGAGCGCTTCGAGTTCTGCGCAGGCCATG

ATTAGAGTGATTGGTGGACGAGGTTCGAGACGATGCGGTCCCGTTCGAGG

TGGGACGAGACGATGCGGTCGGTGTCGTCGGGCGTCACGCCGCCGTACCA

GACGCTGTCGGGGTAGACGGCGACGATCGGGCCGTCGCCGCACTGGCCGA

GACACGAACTCCGGGAGACGTGTACGTCGCAGTCGTCGGCGTCGCGCACG

CCCTGTCTGAGTTGTTCGAGGACGGTCGCCGCGCCGCTCGCGGCGCAGGT

CTGGTTCGTGCAGACCGCGACGTGCTTCTCGGGGGCGTCGTGGACGTGCG

GGTCGTCGTCAACGTCGCTCCGGTCGGCGTGTTCCGCCTGGTGGACGAGC

GAGCGAAGCATCGCGCGAGCGCCGCCCTCGTCGTCCTCGTAGCCGGCGAG

TTCGACCTTGTACTTGCAGGTGTCGCACGACATCTCGACGCTCCCCGACC

GCGCCTCTTGATAGCGGTCGGCCAGCGTCTCGACCACCCGGGCGTCGGTG

CCGAGCGGTCCCGACGCGCCGGCGTCGACGTAGGGGTACTCCTCGTCGAA

CGTCTCGGCGGTGTCCACGATTCGTTCCGTCAGCACGCCGTCACCGAGCA

TGTACGGGAGGACGACGACGGCGTCCGGGCGGTCCTTGGCGACGGTGTGA

AGCGTCTCCTCAAGCCGGGGCGTCGTGACGCCGATGAACGCCGATTCGAC

GCGGGTGAACTCGCGGCCCTCGTACAGCAGGCGCGCCAGTTTGTGCACGT

CGCCGTTCGAGTCGGGGTCGCTCGACCCGCGGGCGCAGAGCACGACCGCC

ACGTCGTCGTCCTCGCGGTCGACGCCGAGGTCGGATTCGACCGCCCGCGC

CCGCTCGTCCAACAAATCCACGAGCGAGGGATGGATGCCGAGATGTGAGC

CGAACCGGAACTCGGTGTCGTCGTGGGTCGCGCGGGCCCGCTGGACCGCC

AGCGGCACGTCGTTTTTGACGTGGCTGGCGGCGAAAAGCGAGAGGGGAAC

GACGGTCATCGTCCGGCAGGTCGGAGCCATCGTCTCGATTGCGTCGTCTA

TCGACGGCTCGGCGAGTTCGATGTACGCCGCGTCCACCGGGACCGACAGC

CGCGATTCCAGTTTCGCGGCGAGGGTCCGCACCTGCTCGTTCGACTCCTC

GCGGCGCGAGCCGTGGCCGACGAGGAGCACCGCGTCGTCGTCGAGCGCCG

GGTCGGGCCGTTCGAGGCTCATGCGTTCTCCGCCTCTTCGAGGCTACGGA

CGAGCTTTCGACGCCGCGTCGGCGCGAACAGGTTCGTCTCGTCGCTCCCC

TCGTAGACCCACTCACCGAAGCCGGCGGCCGCGTCGTCGTCGATACCGAA

CCAGTAGCCGCCAGAGGAGGTCTCGCCAATCTCGCCGGTCGGCGCGTCAA

CGGGGCAGGCGTCGAGCAGTCGGTCAACGGTTTCGAGGAGCGCCCGGTCG

TCGTGGACGAACGAGATGCCGACCGTCCCCGACGAGGATTTGAAACAGAC

CGTCCCGCAGGATTCGAGCAGGCCGCGGAGGAGCTGTTTTCGCCGGGCGT

CGAACACGTCGAAGCGGTAGCCGCCGGCGTCGCCGCCGACGGGGAGGCCG

AACGCGGCGCTCGCGCTTCGCACCACGTCGCCGTCGACCGTCACGCGGTA

CTCGTCTTTGACGCGGGTGACGGCGGTGTTGTGGGCGTACTCTCGCGCCG

TCTCCCGGCGGTCGATGTCGCCGCCGCCGGCGATGACCGCGAGGCGGTTC

GCGCAGGTCTCGTCGCCCGTCGAGACGGTGAACTGCGCTCGTTCGATTTG

GCCGCCGCCGGCGACGTGGCCCCAGAGGTAGGCCGTCTCGGGCGCGCTTT

CGAGGAGGTCGTCCGCGGGGGCGACGCCGGCGTTCGCGTCGCTCACGCGT

CCACCTCCCGGACCGAAATCGCGCCCGGCGGGCAGGCTCGGGTCGCGAGC

GTCGCGTCGTCGTCCGACTCCGCGTCGCCTTCGAGGACGGCGACGACGCG

CCCGCCCGTCTGCTCGACGCGCACGACGCCGGCCGCGTCGGGGTCGATTG

TGGCGAGTCCGTCGTCGTCCTCGACGAACCGCCCGTCGCGGGCGAGACAG

GCGAAGATGCCGTCGCAGGCGTCTCGGTCGATTTCGATTTCGAACATCTC

AGTACTCGTACTTCTGCTCGTAGCCCCGCGGCGTCACCATCCGGTCGTCC

CAGACGTACGTCTCCTCGTTGCCGACGAGCAGCGTCGTCGTCATGTCCAC

GAGGTCGGTCTCACCGAGGTCGGGCAGGTCACCGAGGTCGACGATTTCGA

CTTCCTCGTCGTCGCGGCCCGCGCCGTGGACGATGCCGACCGGCGTGTCT

GGCGCGCGGTGTTCGAGGAGAATCTCGCAGCACTTCTCGTAGTTCGACCG

GCGCTTGCGGCTCCACGGGTTGTAGATGGCGATGGTGAAGCCCTCCTTGG

CGGCCGCGTGGAGGCGCGACTCGATGGTCGGCATCGAGGTCAAGTGGTCC

GAGAGGCTGATGGTCACGGTGTCGTTGACGAGCGGCGCGCCCAGTCGGGC

CGCGCAGGACTGCGCCGCCGGGACGCCGGCGACCACGTCGAAGTCGACCA

TGCTCGCGGTCGCGCCCTTCGATTCGAGGATTTCGAGCGCGAGGCCGGCC

AGCGCGTAGACGTTGGGGTCGCCGCTGCCGACGATGGCCACGTCGTTGCC

GGCGAGCGCCCGGTCGACCGCCTCCTCGGTCCGGGAGACCTCGCCGCACA

TCGGCGTCGAGTAGATGTCCTCTGCGCCCTCGACGACGTCGTCGGGGAGG

AGTTCGATGTAGGTCGTGTAGCCGACGATGTGGTCGGCCTCGCGGAGCGT

GTCTTTCGCCAGCGTCGTCATCTCGCTCGCGCGGCCGGGACCGAGGCCGA

CGGCGACCAACTGCCCCGGCTCGCCGTCGAAGTCCTCGATGGTCGCTTCG

ACCTTCTCTGCGGTCTGTTCCTTCTTCGACCCGCCCGAACTGCCGCCGCA

CTTCGACGCCGAGTCGTCGTCCGTCGTCGAGGACGACGCGCCGCAGTTCG

AGGTCGTGTCCGTCTCGGTCTCCGTCTTCGTCCCGCCGCAGTTCGATTCT

GAGTTGGTCGTGTCGGTCGATTCAGTCGATTCGGTCGTCGCACCGCCGCA

GTTCGATTCGGTTGTCGTGTCTGAGTCGCTCATGGGTTAGAAGTCGTCGA

CGTCGCGCCCGCCGCGGGGCGTGACGAGGTACGTTTGGTAGTCGTTGCTC

CAGGGTTCGGTCTCGTGGGTGCCGATGAGAATCGAGGTTCCCATCCCGCC

CACGTCGTCGTCGTGGGCCTTCGCGTCGCCGAGGGTCGTCACCGAGAACG

TCTCGTCGTCCAGATTCCGGCCGGCCTCGCCGCGGCCGGCGTCGTTGAAG

ATGCCGACCGGCACGTCGTCGGCCCGCTCCTCGCGGACCACGTCGATGGC

GCGTTCGTAGTTGCGCCAGCAGTTGTACAGGACGATGACGAAGCCGCTGA

TGGCGGCCGCGCGCAGTTTCTCCTCGATTTCGTCCCAGCCGCGCCACTTG

TCGGACAGCGAGACCGTACAGAAGTCGTTCGACAGCGGCGCGCCCATCAT

GGCCGCGCCGCCGAGCGCCGCGGTGACGCCGGGGACGATTTCGATTGGCA

CGTCGGTCGCCTCGTCGCCCTTCGCCATCGTGAACACGAGGTCGGACTTC

CCGTAGACGTTCGGGTCGCCGCCGGAGACGTGGACCACGTCCTCGCCGTC

GCGGACGCGCTCGAACGCCTCGCGGGTCAGTTCGACCTGCTTTCCCATCG

ACGAGCGGACGACCTCCGGCCCGTCGTCGCTCCCCTCGGGCGGGAGGGTT

CCGTCGTCGCGGAGGAACTCCTGATAGAGGTTCGAGGCGACCACGCAGTC

CGAGGTGGAAATGAGTTCCTTCGCCCGCTTCGTCATGTGGTCCGGGAGAC

CGGGCCCGATGCCGACGACGTAGAGGGTCCCGTAGTCGTCGGGGACCTCG

TCGCGGTCGTCGGCCGCCGGTTCGGTCGCTGACTCGGTCGCCGGTTCGGT

CGGCTCGCGACCGTCGTTCATCGTCCCACCGCCACGGTCACGGCGTCGTC

GTAGCGCCGCTTTTCGACGACCAGTTCGTGGTCGCGGCCGCCGGCGATGG

CGCTCGCCTCGGCGATGCCGGGCCAGCCGATGAGTTCCTTCGACCGCGAG

GGGGTCGGCCCTTCGAACTCGGTCAGCGTCTCCTTTTCGAAGTAGACGAC

GCCCGCGCCGATTTCCTCGGCCGCGTCGAGCATCCCCGGTTCGTCCGCCT

TTCGCGTGCCGGTCGCGACGAAGTCGACGTCGTCCCAGTCGCAGTCGGCG

TCGTCGAGCGCTTGCTCCCACGCCGTCAGGAACTGCGCTTTCTTCGCGCC

GGAGACGCTCCCGGTCCCGAGGACGACGCCGTCGCCGCCGTTGCGCTTCA

GGACTGTCACGTCGTCGCCGACGAGGACCGCCTTCGGGCCGTCGAGTCGG

GCGACGGGACCGAGTTCGTCGTTGAGGACGGCGAGGTTCGTCGCCACGGT

CGAATCGCCGTTGACGACGTGGGAGTCGAGCGCCTTCGCCTTGCTCTCGA

CGCCCTGTTTGCCCGCGGCCTCCGAGGCGGTCGTCATCGCCGGGACGGCC

CCGAGTTTCGAGAGGTCGTGGGCGACTTGGTTCGCGCCGTGGTGGCCGCC

GGTGAGCGGAATCGCCCACGTCAGTTCCTCGTCGACGACGACGACCGCCG

GGTCCTCCCACTTGTCGTCGAGGAGGCCCGCGGTCTTCCGCATGGCGATG

CCGCTGGCCATGAGGCCGACGAAGCAGTCGTACTCGCCCCAGTGGTCGGC

GAACACGTCGCCGTGGTAGGTGAGGACGTCTATCCGGTCGTAGTCGTCCG

CGAGGTCGCGCTTGATTTCTTCCGCCGTGTCGAGCTTTCGTTCGAAGCTG

ACGATGGCGATTTCGTCGGCAACCTCGCCGTCGCTGTCTGGCGTTTGACA

GTGGCCGCTTCCGGAGTCGGTGTCCGAGTTGGTGTCTGTGTCACTCATTG

TCTGGCTCCTGTGTTTCGGTGTCGGGTTCGTCGTTCGGTGCGTCGCCGCC

GCGGGCCCAGTCGCCGTAGAGGAACGACCGCTCGTACGCCGAGCCGTTCA

CCGCCTCGCCGATGATGACGAGCGCCGAGGCGCGGTAGCCCGCGTCTTCG

ACCCGCTCGCCGATGGTCTCGATGGTGCCCTCGATGACGTCTTCGTCCGG

CCACGAGGCGTGGTAGACGACCGCAACGGGCGTGTCGGGGTCGTGGCCCT

CGTCCAGCAGGCGGTCCATCGTCTCCGAGACGGCGTGGGTGCCGAGGTAG

ATACAGACCGTGGTGTCACCGAAGCCGACGAAGTCGCTGATGTGGTCCTC

CTCGGCCGAGAGCGTCCGGCCCCGCGGCCGGGTGAAGACGACGTGATTCG

AGACCTCGTTGAGCGTCAACTGGGTGCGGAGCGTCGCGCTCGCGGCGAAC

GCCGAGGTGACGCCGGGAACGAGGTAGCTCGGCACGCCAGCCTCGGCCAG

CGCGTCCATCTGCTCCAAGGCGGCCCCGTAAATCGCCGGGTCGCCGCTGT

GGAGTCTGACGACGGTCCGACCCGACTCGTAGGCGTCGGCCATCAGCGGA

ATCAGCTCTTCGAGGTCCTTGCCGACGCTGTTGACCCGCTCGGCGTCGGC

GCAGAACTCGTCCAACAGTTCGCTGTTGACGAGCGACCCCGCGTGCACCA

CGAGGTCGGCGTCCTCGACGAGTTTCCGCCCGGCGACGGTGAGCAAGTCC

GGGTCGCCCGGTCCCGCGCCGACGAAGGGGATTCCCTCCTCGTCGGTGAA

GCCGGTGTCCGCCTCGCCGTCCGCGCTCACGCCGACCCCCCCGATTCGAC

GCCGTCGAACTCGGCGGTCGCCACGTCGCGCTCGACGCCCTCGCGCTCGG

CGTAGGCCAGCGTGTAGTAGTCGCGCTCCGCGAGTTCGTCGGGGTCGTCG

GTGACGACCGTCTCGCCCTGCTCCATGAACAGGCGGCGGCCGAAGCGAAC

GTCGTAGCCCGCTTCGGTCAGCTTCTCGTGGGTCGCCGGCGCGTCGGTGA

CCTTGAACAGCACCATGCGGTCCGGGCCGGTCGGCGCGGCACCGTTGGCG

GCCTCGCGGAGCGCCAGCCCGGTTCCGGATTCGATTTCGACGCCGAGCGC

GGTCGTGAAGGCCGTCATCGCGCTCACGCCGGGGACGACTTCGAGGTCGA

CCTCGGGGTGGAACGCGTCGAGGGTGCGGCGGAGGTGGCCGAACGTCGAG

TAGACGTTGGGGTCGCCGAGGGTCACGAACGCCACGTCCTCGGAGTCGGC

GCGGGCGGCCACCTCGGCGGCGGCCTCCTTCCACGCGCGGCGGAGTTCGT

CGGGGTCGCGCGTCATCGGGAAGTCGAGGTCGCCGATGCGCTCCTCGGGG

ACGTGGTTCAGCGCGACCGTCCGCGAGAGGCGGCCGGGCGAGTAAACCAC

CGCCGCCGATTCGAGCAGGCGTTTCCCCTTGACCGTCACGAGGTCGGCCT

CGCCCGGGCCGAGACCGATGCCGTAGACGGTCATAGCCCCTCCGCGTCGC

GTCGGCCGACGACGACGTACACCGGGTTGTTCGACCGGAAACTCGTCGCG

CCGGCGAGTTCGTAGCCGTGGCTCACCTGCAGCTGGACGACCGCCGAGAG

CAGGCCGCGCTCGCGGAACGCCTCGATGGCGTCGCCGGCGGGTTCGACCC

GCGAGACGTTCATGACGATTCGGTCGACGCCGGTCTCGACGGCGTGGTCC

AACACGGCTTCGAAGTTCCGACTGCCGCCGACGAACATCGCGTCGGCGTC

GGTCGGGAGGCCGTCGGGAGCCTCCGCCTCGCGGAGCGTCACGTCGGCGT

CGTAGTCGTTCGCCGCGAGGTTCTTCCGCGTCACGTCCAGTCGGTCCGGC

TTGCGCTCGACGGCGGTCACGCGCTCGACCCGCCGGGCCGCCTCGATGGT

GACCGAGCCGGTACAGGAGCCGACGTCGACGAAGTGGTCCGTCGGCCGCA

AGCCGAGTTTGTCGAGCGTGACCGCTCTCACCTCCGGTTTCGTGGGTCCC

GCCTTGGCGTCGTGCGGGAGCGTTACCTGTGACATTCGACTCGACAATCC

CGGCGCGGTTTGAAAACAATTGTGCTTGTTGTACCACAAACAAGGTTTCA

TAAAATCCGCCCCGGAGTTATTAAATCGAGGTTGCTGAAACACAATCTCG

CTTGTTCTCGAAAGTTTTACAACTGTGCTTGGTTTTCATCAATCCGAGTT

TACCATGGCTACGGCGAACACCGTCAACGGTCGCATCGAACGCGCACGAA

CGGAACTGACCCCCGCGCAGATGGCTGCCGGTTCCCTCTTTATCGTCGCG

CTGGGCTTTACGCTCCTGTTCGTGCAGGACCCGCTCGTCCACGACTCGCT

GCACAACTTCCGCCACGCGGCCGGCATCGCCTGCCACTGACGATGTTCGG

GGAGTACCTGACGCGGGGAGTGAAGGCGGGGGTCGTCGCCGGACTGGTGT

TCGGACTGTTCATGGCCGTCGTCGCCAACCCGCTCGTCGGCTACGCGGAC

GACCTGAACCACGCGACAGTCGAGGAGAGCGGACATACTCACGAGGCCGA

AAGCGCCCACAGTCACGAGGCCGAGGGGGCTCACAGCCACGAGTCCGGTG

ACTCCCACGCCGGCGAGAGCGGCGGCCATCACGACACCGCGGTGTCGATG

GCGGTCACGAAAGCCGTGAGCGTCGCGGCCGGCGGCCTGTGGGGCGTCGT

GCTCGGCGGCGCGTTCTTCGGGGTCGCCTTTTTCTTCCTCGAACCGGCGA

TTCCCGGACCCGACGCCGCGAAGAGCTATCTGATGGGATTCGCGGGGTTC

GTCACCGTTTCGGGCGCGCCGTGGTTGGTGCTCCCGCCGGTCGCGCCGGG

GGCGGAACAGTCGCTCTCGACGGCGACGCGGCTCCCGCTCTACGCCGGGA

TGATGCTCGCCGGGGCGCTCGCCTGCCTCTCGGCCGGCTACGTCTACACC

CGCCTGCGCGAGTCGCGGGGCCTCGGTGCGGCTCTCGTCGGCGCGGCCGT

CCCGTTCGGCCTGCTCGCGGTGCTGGCCGTCGTCGCCCCCGAAAACGCGG

TTCGCGGGGCGCTGTCGCCGGCGCTCGAAACCGGACTCACCGGCCTGTTC

GCGTTCGGACAGGTCCTCCTGTGGCTGGTCCTCGCGGCGGCGCACGCGCG

GTTCCGGCCGTCGAGTGAGTCCGAGTCCGAACCGGGGATTCCGACCGCCG

GCCGGGACTCGGCTCCCGCGGCCGACTGATGCGCGACCGAACCGACGAGG

TCCGCGAGCACGGCTTTACGGACCACGTGTTGGTCTGTACGAACGGCCGC

GAGTCCGAGTACGCCGACTGCGCCGAGGCCGACGGCCCGGCGGTGTACGA

CGCGGTGACCTCGTGGTTGCGCGACCGTGGCGTCTTCTGGTCGCGGGTCC

ACGTTGCAGAAACGAGCTGTCTCGGCCTCTGTAGTGCGGACGGGGCCGCG

GTCGCCATCCACCCCCGAAACCGGTGGTACTCGGACGTTCGCCCGGACGA

CGTGCCGGCGCTGTTGGCCGAGGTGTTCGGTCCCGACGCGTCGAACCTCG

GCGTTCGACCCCGCGACTGACCGTCTCGGGGACGACCGTCGGTGTCTGCG

CCGCGCCGTTCTCACCGCCTCTTGTCGTTCCCTCCCCGACGCGGGCGGGG

TTGAACGCAGAACAATAATATCGGTGCACACCTGATTACGGTCCATGGAC

GAGAAGGACCTTCGAATCCTCAAGGCCATCGGGACGCTCGAATCGGGGAG

TCCCGACCGGATAACCGAGGAGACGGGCATCCCGAAGTCGACCGTTCACT

ACCGCCTCGAACGGCTCCAAGAGCAGGGCATCATCAAAAACGACATCTTC

GATATCGACTTCGAGAAGGCCGGGCTAAACCTCACGCTCATCACCGAGGT

CTGGGCCGAGTACGGCTCGGAGTACCACAAGGAGGTCGGAGACAAACTCG

GGGAGGTGCCCGGCGTCAACGAGGTCTACTTCACGCTCGGCGACACCGAC

TTCGTCCTGATTTCCCATCTCGCCTCGCGCTCGATGGTCGAGGACCTCAT

CTCCGAGTTCGAGGAGATAGACGAGATTTCGCGGACGAGTTCGACGTTCG

TCATCACGACCATCAAGGACGAGCCGAACCCGCTGAACGACTACGAACTC

GAAGAACTGAAAGCGGCGCTTCTCCCCGACGTGTCGTGACGCGGCCCGTG

CCGTATCCGCACGAACCCCGCCGCACCGCGTTTCGACCGCCGGAAGCGAA

AGCGAATCAGTCTTCGAGCTGCTGGGCGCTCAGGTGCGCCTTCGCTTCCG

TCCGGGTCGGCAGCCCGCCGGCGACGGCCAGCAGGACGACGCCGAGCAGG

CACCAGCCGCCGACCATCGCCCACTCGTAGGGCCACGCCAGCGCCGAGGG

CGAGCCGGGGAGGTAGAGACCGACGAAGAACACGGTCAACAGCAGCCCGA

GCGCGCCGACCGCGTAGCCCGCCGGCAGCTTGAACGGCCGGTCGAAGTCG

GGGTCGCGGTACCGGAGCACGAGGAACGAAACGACGACGAGAAGCCACGC

CGTCACCATGCCGAGCCCGCCGGCGTTGACGACCCACACGAGCATCTGCT

CGCCGAACAGGGGCGCGAGCGCCGCGAGGCCGCCGACGAGCACGATTGCG

GTCGACGGCGTGTCGTGGTCGTCGTGAATCGTCGCGAGCTTCTCGGGGAG

CATGTGCGAGTCGGCCATCGCGTAGATGGCGCGGCTGGCTCCGAGGACGA

ACCCGTTCCAACTCGTGAGGATGCCCGCGAGGCCGGCCAGCGCCATGATG

CGGCCGATGGTCACGCTGTCGAACGCGATTTCCATCGCCGTCGCGGCGGG

GAGCGAACTCTCGACGAGCGTCGTGCCCGTGGCGACCTGCCCGGCCGCCC

AGATGACGGCGATGTAGAACGCGGCGGCGCAGGCGACCGAGAGCCCGATG

AGCCCGCCGAGGAGCTTCGGTGACACGTCGGCCTCGCCCGCGGCCTGCGG

AATCACGTCGAAGCCGACGAACATGAACGGCGTCATGATGGCGATCGTGA

ACACGCCGCCGATGCCGGCTCCCGCGAGCGGCGGCGACGACGGCGACGTG

CCGTTGAACAGCGCGCCGAGAAGGAGCGTCAGCCCCGACAGCGTGATGAC

GAGCGCGAGAATCGCCTGGAACTGCGCCGCCGGCCGGACACCCCGATAAT

TGAGGTACGTGATAACCACGGAGCCGATGACGCCCGTCGCGACCCACGTA

CCGTAGACCGGCTGGCCGGCGACCGTCCAGAGTTCGAACACGTTGAAGCC

GGGGACGATGTACGCGAGGGCGCTCGGCAGCGCGACCGCTTCGAACGCGA

CGACGCTGATGTAGCCGAAGATTATCGCCCACGTACAGGCGAACGAGCCG

GCCGGCCCCAACGCCCGCATGCTGTAAACGTGCTCGCCGCCGACGAACGG

CATCGCGGAGGCGAGTTCGCCGTAGATGAGCGAGACGACGGTCACCATGA

ACCCGCCGAGGACGAACGCCGAGATAGCGCCGGTGACGCCGCTTTGGTCG

ATGAAGTACCCCGTCTGCACGATCCAACCCCATCCGATCATCGCTCCGAG

TGCGAGCACGAACACGTCGCGCTTCGAGAGGACGCGTGAGAGTTCTCCTG

TCATGGTTATGAGTGACATCACCACATTCTAACTTACCCACTATATATCT

TACTGTGTTCAATTTTTAGTCTCTATTAGAAACACAGTTCAATAGTAATC

CGTCATCTGGGACGACAAATCGAGCGACGATTCACCGGAAAATCGCCAAA

CGGCCGTCGGTTCGAATAGTAGTCAACTCGTCGAGCAGAAAGAAACGGCG

TCCGCGCTCGATTCGCGTTCCGGGCGTCGTCGCGGGCGTCGCGCCCGGTG

TCGCGTTGGGTGTCGCTCCGGGTGCTGGGTCGGGCGTCGTCGCGCTCACT

CGTCGGCGGGCGCGTCCTCGACCCAGACCGTCTTCGTGTTGACGAACTCC

CGAATCCCGTGCCGGGAGAGTTCGGTCCCGTAGCCCGAGTCCTTCGTCCC

GCTGAACGGGACCCGCGGGTCAGACGCGACGACGTGATTGACCGCGACGT

TACCCGCGGTGAGCTTCGTCGTGAGCGCCTCGCCGCGTTCGGCGTCGGCC

GTCCAGACGCTCGCGCCGAGGCCGTAGGGCGTGTCGTTCGCCACGCGGAC

CGCCTCCGCCTCGTCTTCGACTCTGATGACGGTGGCGACGGGGCCGAACA

GTTCCTCGGTCCTCGCGGGCGCGTCCTCGGGCACGTCGGTCAGCACCGTC

GGCGGGTAGTAGTGGCCGGGTCGGTCCAGCGGTTCGCCGCCGAGGCGGAG

CGTCGCGCCGGCCTCGACGGACGCCGTGACCTGCTCGTGGAGCGTCTCCA

TGAGGTCCGCCCGCGCCTGCGGCCCCACGTCGGTCTCGGGGTCGGTGGGG

TCGCCGACGGTCAGTGCGCGCATCTCCTCGGTGAAGCGCTCGACGAACTC

GTCGTAGACGGCCTCGTGGACGAGAAAGCGCTTCGCGGCGATACACGACT

GGCCGTTGTTCTGCGTTCGCGCCCGTGCGGCCCGCTTCGCGGTCGCTTCG

AGCGGCGCGTCGTCGAGGACGACGAAGGGGTCGCTCCCGCCGAGTTCGAG

CACCGTCGGCTTGAGTTCGCGGCCGCTCGTCTCCGCGACGGCCCGCCCGG

CGCGGGTGCTCCCCGTCAGGCTGACGCCCCGAACTCGGTCGTCTTCGATG

ACGCCGGCGACGCGCTCGGCGGGAATCCGGAGCGAGGTGAACACGTCGTC

GGGGTAGCCCGCCCGCTCGAAGACGGAGGCGATTGCGTCGGCGCAGCCGA

AGACGTTCGGCGCGTGCTTGAGCACGGCGACGTTTCCGGCGGTGATGTGC

GGGGCCGCGAACCGGAACACCTGCCAGAACGGGAAGTTCCACGGCATCAC

CGCGAGCACCGGGCCGATTGGCTCGTAGGAGACGTACGTCTTCGACCCCG

GCACGGTGCCGATGTGCTCGTCCTGCAGGAACTCCGACGCGTGTTCGGCG

TAGTAGTCGCAGACCCACGCGCACTTTTCGACCTCGGAGACCGCCTGCTC

GACGGGTTTGCCCATCTCCTCGGTCATCAGCGTCGCGTACTCCTCGCTGT

GTTCGCGCAGCACGTCGGCCGCGGCCGCGAGCAGTTTCTCGCGCCGCGAA

ATCGGCGTCTCGCGCCACGATTCGAACCGGTCCCACGCGGCGTCCAATCG

CGCGTCGACCGTCGCTTCGTCGTGTTCCTCGTGCGTCTCGATGACCTCCT

CGGTCGCGGGGTTGATGGATTTCATGGTCGGCTCTCGGGAGCGCGGCGGT

CGCTCGTCGCTCCCGCTTGGACCCACTGTCGTCCCATATGTTGATAAATG

TTCCAGACACAGCAGAATACTCGGACTGTGTTCAAAACACGTCGGTGACG

AACGCAGTCGAGTACGGAGCTCCCAGGCGCGTTCGAGTCTGGAACGACCG

CCCTCGTCCCGACACACCCGCAGTATTCTGTATGATATTCCACACAGAAT

AATTTATTAGGATTCAGTTTGGAACTTGTGTTCATGAGAGAACGGACGAC

GAACTCGTCGGTCGAGGCGGCGTACGACGACTACCTCATGCCGATTTGGA

AATCGCTGAACGTCCCGGTCGAGCGGGCGTCCGGCTGTACCCTCGAAGAC

TTCGACGGCAACGAGTATCTCGACGTGTTCTCGGGCATCTCGGTGACGAA

CGTCGGACACAACAACGAGGCGGTCGTCGAGGCCGCCACGGAGCAGTTGG

AGGAGTTCGTCCACGGCTGTTCGTACGTCCATCCGAACCGGCCGGTGGCC

GACCTCGCCGAGACCATCGCCGAGGTGACGCCGGGCGACCTCCAGAAGAC

CTTCTTCTGTAACTCCGGCACCGAAGCCGTCGAGGGAGCTATCAAACTCG

CCCGGAAGTACACCGGCTCGAAAGAGGTCATCTCGCTGGAGATGGGCTTC

CACGGCCGCACCCTCGGCAGCCTCGCGCTCACCGGCAACAGGGCCTACAA

GCAGGACATGGCCCCGACCATCAACGACGTGGCTCACACCGCGCCGCCGT

ACGCCTACCGCTGTCCCCGCTGTGACGGCGACACCTGCGGCGCGGACTGC

GCGGACGCGCTCGAACGGGTCATCGGCTCGCACACCAGCGGCGACCTCGC

GGCGGTCGTCGTCGAACCCGTCATGGGCGAGGCCGGCATCGTCGTCCCCT

CGGAGGCGTGGCTCAAGCGCGTCCGGGACATCGCCCACGACCACGGCGCG

CTCCTCATCGCCGACGAGGTCCAGACCGGCTACGGCCGCACCGGCGAACT

GTTCGCCAGCAAGCAGTTCGGCGTCGAACCGGACATCCTGACGCAGGCGA

AGGGCATCGCCAACGGCCTGCCCCTCGGCGCGTTCACGGCACCCGCGGAC

ATCGCGGACGCGTTCGGCTCCGGCGACCACCTCTCGACGTTCGGCGGCAA

CCCCGTCGCCTGCGCGGCAGCGCTGGCGACCATCGAGGAACTCCAGAACG

GTATCGTCGACAACGCCTGCGAGAACGGCGAGTGGCTCTCGGCCGAACTC

GCCACGCTCGAATCCGACTACGACGTGGTCGGCGAGGCCCGCGGCCTCGG

GCTGATGCAGGGCATCGAACTCGTGGACCCCGAGACGACCGGCCCGCAGC

ACGTCGCGCCCGCCCCGGACAAGGAACTCGCGGCCGCGGTCGGCGAGCAC

CTCCGCGAGGAGTCCAACGTCGTCATGGGCGTCGGCGGCTACTACAAGAA

CGTCATGCGCTTCCAGCCGCCGCTGACGATTTCGCAGGCGCAGCTCGGCG

AGGCCGTCGACGCGCTCCGAACCGCGCTGAACGACCTGGCCTGAGCCGCC

TGCGTCGACCGCGACACCCGCTCACGGCTTCATTTCGATTGCTCTCGGTC

TCGCGGCTTACGGTGGTGATAGTTGTGGATTGGTGCCGGGGATAGCCCCC

GCTGAAACGGCCGCCTCCGCCGGAAGCGCGTCTCGACCGTCGGTCTGGGT

CAGGGTGTGTCTTACTCGTCGACTTTCACCATGACCTTGATTGCCTCGCG

GTCGTCCATCGCCTGATAGCCTTCCGGGACGCCGTCGAGGTCGACGGTCT

TCGTCAGGATGGGCGACGGGTCGAGGGTGCCCTGCAGCACGTCGTCCATG

AGTTCCTCGATGTAGTTCCGGACGGGTGCGCCGCCGGCGGAGAACGAGAG

GTTCTTGTAGAATATCGGCTGGAGGAAGTCGGCGGACTCGACGTGCGGGA

CGCCCACGGCGCCGATGGAGCCGCCGGGACGGGCGACCTGCGCGGCGGTT

TCGAGCGCCGACGCCGCGCCGACGCATTCGAGGACGTGGTTCGCGCCGCC

GTGGGTGAGTTCGAGGACTTCCTCGACGGCCTCTTGGCCGCGGGACGAGA

CGGTGTCGGTCGCGCCGAGTTCGCGGGCGACTTCCAAGCGGTCCTCGTGG

TGGCCGACCGCGATGATTCGCTCTGCGCCGATGCGCTTCGCCGCGAGGAC

GCCGCACAGTCCGACCGCGCCGTCGCCGATGACGACCGCGGTGTCGCCGG

CTTCGAGGTCCGCCAAGACGGCGGCGTGGTGGCCGGTCGACATCACGTCC

GTGAGCGGGAGGAGCGATTTGAGCGTCTCTTCGTCGTCGGCGTACCGCTC

GGGGACCCGAACGACCGTGCCGTCGGCGTGGAGGACGCGGACCTTCTCGG

CCTGTGCGCCGCCGGTCTCGTCGCCGTGCGGACCGAAGAACCCGCCGTCT

ACACACGCGGTGTGGATGCCTTTGCGACAGAACTCGCACTCCCCGCAGCT

GTACGCGAACGGGACGAGCACGCGGTCGCCCGGTTGGACGCTCGTGACGT

CCTCACCGACTTCCTCGACGATACCCATCGGCTCGTGGCCGACCCCGGAG

CCCTCCTCGTGTTCCTCGTGACCGCGGTAGAACCAGAGGTCGGACCCGCA

GATGGCGGTGTGAGTGACGCGGATGACCGCGTCGGTCGGTTGTTGAAGCT

CCGGTTCTGGTTTTTCCTCGATGCGAACGTCGCCCGGACCGCGATAGACT

GCTGATTTCACGTACGCTGTGAGGTACGGACCTTGCGGGCATAGATGCGA

TGCTATATGAATAGTCCATTTAACTGCGGCCGGGCGGCCGTCCCGCTCGT

CCACGGGGAAGCGAGCCGACCGCGTGGGCACGTGGCCCAGCGGCGGTTCA

CTGCGTGGGCAGGCTCACTGCGTGGGCGGTTGCATCCGCGCCTGCATGCC

CAGCGAGGAGGTAATCAGTTCCCGTTCGCCGCGTCGGAGGAGACTCGACA

GCGACGGTTGCGAGATGCCGAGTTTGGCGGCCAGCTCCTCGGCCGTAGTC

TGTCGCGGACTCTCGTAGTAGCCGCGCGAGACGGCGAGCGTCAGCGCCTC

CCGCTGGCGGTCGGTGAGGCTGTCCGTCGGTTCGTCGGACGCGGACCCCT

CCGACGCGATGGACACGAGGTCCAGCGAGATGCCGTGGGCCTCACAGCTC

TCTCGGAACTCGTTGAACCCCTCGTACTCCTTGAACACTTTCGTCTCGTG

CCAGCCGTCGGCGGTGACGACCGTCGGTTCGACCTGTGCGCCCCCGAAGT

CCGGCTCGAACGCCTTCGAGACCGACTCCGCCAGTTCGACGGTGAGCTTG

TAGACGCCCTTGTCGGCGGTCTCTCCGATTGCGTCGGCCGCGACGACCTC

GTCGCGCGTCAGGAGGTCAGCCTTAGTGACGCCGTCTTCCCGCTCGAACT

GCACGGTGAATATCTGCACGTCCGGTTGCAGACAGAGTGCGTGAACGCAC

TCGACCTCGTCCGGCCGTAGCGCGGTCGGAATGCTGACCAGCGGAAGCGA

AGTCGACCGAAGAACGAACTCCGTGGTGATAGCCATTGTGTGGTATCACG

CCGTCCGGCGAGTTAACGTTCTGGGGTCTCCGCTGCGTGGGGGTGACCCG

ACCCTTCGATTCAGGGGCGGTCCAGCTCTTTGTCCCACGCCTTCCACGTC

TTCAGAAGCGGGATGATGGTGTAGTCGTCGACGATTTCCTCGCCGTCGAA

GAGGTCCCAGACGTACTCTTTCAAGGTGTTCAGGTCGGTGTGCCACGCGC

TAAATACGAGGTCGTACTCGCCGAGACAGGAACTCGTGTTGTAGACGAGT

CCCTCGTCGATGAGCATCGTGATGAACTCGGTCCGCACCTCGGAGGAGAC

GTGCTGGTCGAACGAGACGAGTATCTGCGCGTCGGCGAGGTCGGCCTCCT

GGAGGACGATGACCGCGAGGATTTCGAGCACGTCGCTCTCTTGAAGCTTC

TCGCGGCGGCGACGGACCGCGGTCCGGGAGAGTCCGACGCGCTCGGCGAG

TTCGGTGTCTGAGATGCGGCCGTTCTCGTTGAGCGCGCGGAATATCTCGT

AGTCGTGCTCGTCGAGGTAGTTTTCGAGGACCGCCTCCGGCGTGTTCGGG

TCGGGCGGCGCGTCGGCGGACGTGAGGTCGGGGTTTTCCATGGTCGGCTG

TATTGGTGGGGTTCGATTTGAAACTGTGGGTGGCGTAAACGAACCAAAAC

GATACAATAGTTGTGTCGAATTGGCTAGTTCACGGCTTGCGGTGGGTTGG

TGGTGGGTAGCGAGCGATTCGGTCGTGGGACGCCGCGCGAAACGCGGCGT

CGAGGGCGATTCAGCGGCGAGGCCACCGGCGCGACCGAAGCGTCACCACC

GGGTCACCACCCCGTCTCGTCCCGCGGTAGCGGGTCGTACGGAACCTCGT

ACCCGAACGCTTCGGGGCCGATGACGTCGAGCGCTTCGGGCGTCGTGAGC

AGTTCGGGCGCGGGCACGCCGAGGACGCGGACGCGCTGGCCGTACCGCAG

GGCGTCGGTCGTGACCGGCGCGCCGGTGTCGGTGTCGACGAGGCAGATGA

GGTCGGGCACGGTCGTCCGGAGGTCGCCGTCGTCGTCCCGCGCGACGAGG

AACTCGTTTTGGAACTCGATTTCGAGGGTCGCGTCCTCGTCCAACCCGGC

GAGCGTGACGCTCCCGAGGGCGAAGCCGTCGCGGTTGCGGCGGTGCACGT

CCACGATTTTCCCGGCGAACAGCTCCTCGCCGCCGAGCAGTTCGCGGCCG

GCGTCGACCGGGTCGGTGCCGGCGTCGCGGGCGCGTTCGACCGCCCGCCC

GAGTTCGGTGGCGAGCGAGACGGTGTGGGGAACCGCGTACTCCGAGACGA

ACGCGCCGGTCATGAGCGGGAAGGCGTAGCCCGAGCGGCCGCCCATCTGG

ACGGTGATGGCCCGCGCGAGGTCTTCGAGGCGCTTGGCCGAGTCGATGTC

CCGGTAGACGACCTGATTGCCGCGCTCGTCGGTCGTCGCCGCGTAGTTCA

CGGGCGTGCCGTAGATGAAGAACGTGTCCATCTGGAGTTCGGGGAACGCC

CGGCCCATGCCGTCGGCGTCGACCACGGGGAGGTCGGTCATCGCCGCCAC

GCACAGCGGGGCCATGCTGTTCGCGCCGCCGATTTCGCCGGGGATGAGCG

CGTCGACCGTCTCGCCGGAAAGCTCCTCGATGGCCCGAAGCGACTGCACT

TCCTCGTCGCCGCCGGCGAACTTCTCGACGCTGATGGTGGGCGCGCCCAT

CCCGCCGACGCTCGCCACGGTGGCGTCGGGCGGCAGGTCCCGCGGGTCGA

CCAGTTCGACCGAGTCGGGGTAGGCGTCGTCTTCGAGGAGCGTCTGGAGT

CGCAGGCGGCCGAGGCGGGGGTTGCCGCCGCCGCCGGTGCCGAGGATGCC

CGCGCCGGTGCCGAGCGCTTCGAGGTCGTCGAGCGTTATCTCCGTCAGGT

GTGGGATGTCGAAACTCATGGGTGGTCCGCGAGTGGTTCGTAGGTCGCGT

CGAGGCCGAACGACGCCGGCCCCCACACGTCGAGCGCGGCGGGCGTCCGC

ATTATCTCGGGCGTCCGGATGCCGAGCACGCGGACTCGCGCCCCGTACCG

GAGGCTCTCGGTCGGAATCGGACCGCCCGTCTCGCGGTCGAGGACGGTGA

TGAGGTCGGGGACGGTCGCCACGTAGCTCCCGTCGACCGCCGCGCCGAGG

TTCTCGTTTTGGAACTCGATGCGCATCGTCGAGCCCTCGTCGCCGTCGAG

GCCGTCGATATCGACGTGGCCGAAGACGAACCCGCGCTCGGTGCGGCGCT

GGACGTCCACGATTTTCCCCTCGAACAGCGACCGCGCCTCGCCGTAAATC

GACTCGCGGGTCACCTCGCGGATGGCGCTCATCGCGTCGTCCTCGGCGAG

TCGGAGCGCGCGGCCGAGGTTCTTCGCGAGCGACATCGTGCCGGGGATGG

CGGTCTCGTTGACCTGCGTCCCGGTCATCGGGTAGTCCGAGACGTAGGCG

ACGCCGCCCATCCTGACGGTGACGCCGCGGGCCAGCCACTCCAACTGGTC

GTTGTCCTCAGTCTCGATGAGGCAGGTGCTCCCCTGCTCGTCGCTGACGG

CCGCGGGCGTCCCGCTGACGCCGTAGATGTTGAACGTCTCGTGTTGGAGT

TCGGGGAAGGCGCGTCCCATGCCGTCGGCGTCCACCACGGGGAGCCCGCG

GCGGGCCGCGACGGCGAAGGGGAACGTCGAGTTGATGCCGCCGCACTCGA

TCGGCATCGTCGCGTCGGCGGTCTTCCCGAGTTCGCGCTCGATGCGCTCC

ATCGAGGCGACGGCCTCCCGCCCGCTCGGGAGCTTTTCGACCGAGACGGT

CGGCGCGCCCATCTGGGCGGTCGGGATGACGAAGTCGTCCGCGTCCAGTT

CGTCCGGGGGGAGGAGTTCGACGGGGCCGAACTCCTCGATGGCCTGCTTC

GCGACGAGTTTGCCGACGTGGGGGTCGCCCCCGCCGCCGGTGCCGAGGAC

AGTCGCGCCGAGCGCGATGTCTTCTATCTCCTCGACGCCGATTTCGACCG

TCATCAGTGGTCGAGCGCCCCGGCGGCCTTCACCTTGATTCGCACCGCGT

TGCCGGGCAGGTACGAAAGCGGGACCTCCTCGACGTCGACGATGTCCACG

GTGTCGGCGTCCGCGCCGGCGGCGAGCGCGTTGTCGGTGGCCTCGTCTTT

CGCGTGCTGGATGGCCTCCTCGCGGTCCATCTCGTCGAGGCTGTAGATGC

GGTCGACCTCGCCGGAGACCTGCGCGATGGCGACGCCGACGGCGTTCGCG

ACCTCGTAGTGGTCGGGCTTGTGGACCTCGCTCGCGCCCGCGATGTCGTC

GGGGACGAGGATGCTCCCGCCGCCGACGATGACCACGGGGACCGGCTCGG

CGCTCGTCTTCATGCGGTCGACCTCGCGCTCGACGCGCTCTCTGACGTAC

TCGCGGGCGGCCTCGACGATTTCACCGTCGACGTCCGGCGTCTCGGAGCC

GATGTCGATGGTGCCGGCGGCGACTTCGAGGTCGGTCGCGGTCAGCGTCT

CCCCGCCGAAACACTTCGCCTCCTCGGTCAGTTTGTAGCCGACGCTCTGG

GGGCCGACGGTGACGCCGCCGTCGGTCGAGACGATGGAGCCGCCGCCGAT

GCCGATGGCGATGATGTCGGGCATGCGGAAGTTGGTCTTCACCTCGCCGA

TTTCGACGGCGACGCTGCTCTCGCGGGGGAAGCCCTCGGTGACCGCGCCC

ACGTCGGTGGTCGTGCCGCCCACGTCGACGATGATGCCGTTTTCGACCGC

GGAGAGGTACGCCGCGCCCCGGACCGAGTTCGAGGGGCCGCTGGCGACCG

TGAAGATGGGGTAGCGGATGGCGTAGTCGACGCTCATCAGCGTCCCGTCG

TTCTGGCCGAAGTAGAGGTTCGCGTCGATGCCGCGTTCGTTCATCGCGTC

GACGAAGGCGTTCGCGGCCTCCGAGGCGACGCTCGTGAGCGCGGCGTTGA

GCGCCGTCGCGTTCTCGCGTTCGAGCAGGCCCACGCTGCCGATTTCGTTC

GAGACCGAGATGGGCACGTCGTCACCGACCTCCTCGCGGATGAGTTCCGC

GACCCGCGTCTCGTGGTCGTCGCGGACGGGCGAGAAGACGCTCGTCACGG

CGAAGGCGTCCACGTCGGAGAACTCGCGAATCTGCGCTCTGACCTGCTCC

TCGTCGAGGTCGTTGAGGAGTCGGCCGTCGAACTCGTGGCCGCCGTCGAG

GATGGCGACGTTGTTGCCGATGGCCGCGGCGAGGTCGTCGGGCCATTCGA

GGAGCGGGCGGATGCTCTGGGTCGCGGGCGCGCCGATGCGGATGACGCCG

ACCTCGTTGAGGCCGCGCCGCTCCGTGATGGCGTTCGTCGCGTGGGTCGT

GCCGAGCATCACGTAGTCGAGTTCGTCCTCGGAGACGCCGCTGTCGTCGA

GGACCACGTCGAGGGCGCTGAGGATGCCCGAGGTGATGTCTTCTGTCGTG

GGGGTCTTCGTCTTCGCGAGGAGGTTGTCGTCGCCGTCCATGACGACGGC

GTCCGTGTTGGTGCCGCCGACGTCGATTCCGATTCGGTAGTCAGTCATGT

CTGGGTTGCTGGAGAGTTTCGATTGGTTCGTAGTCGATGCTGTAATCGAA

GTACGCCGGGCCGACGAGGTCGAGTCCCTCCTCGGTCCGCCACTTCGGTG

AACACGGCATGCCGATGACCCGCACCCGGTGGCCGTAGGCGAGTCGCTCG

GTGGTGACGGGGTCGCCCGTCTCGGCGTCCACGACCGTAATCAGGTCGGG

GGTGCTCGCCACGACGCCGCGCTCTGAGTCGCGGGCGACGAGGTTCTCGT

TTTGGAAGTCGAGGGCGAACGTCCGGCCCTCGCAGTCGTCGATGCCGTCG

AGGGTCGCCTCACCGACGGCGAAGCCGCCTTCGGTCCGGCGCTCCACGTC

GGTTATCTTCCCCGCGAACAGTTCGTAGCCGTCGGTGAGGTCGAGGACCG

CCTCGACGGGGTCCTGCGAGCGGTGCTTGGCGGTGCGAATCGCCTCGCCG

ATGTCGTGGGCGAGGGACATCGAGTCGAGAATCGCGTGCTCGCGGACTTC

CGCGCCGGAGAGCGCGTAGGTGCCTATCATGCACGCGCCGCCCATCTCGA

TGCTCGTCACGCGGGCGAACGCCTCGGCGTACTCGTTGCTGACGGTGTTC

ACGAGAAGCGAGTTGCCCTTCTCGTCGGCGATGCTCATCGGGGTCGCGCT

GACGCCGCCCATCGTGAGCGTCACCATCTGCACCTCGGGGAACGCCCGGC

CCATGCCGTCGGCGTCCACGAGGGGAAGCCCGAGCGCGGCGGCGACGGCG

ATGGGGACGGTGCTGTTCAGCCCGCCCGCCTCGATGCTCATCGTCGCGTA

GGCCTCCTGTCCGAGGTGCGTTTCGAGCGCCTCGAAGGCGGCCAACGCCT

CGGTTCCCTTCGGCATCTTCTCGACCATCACGGTCGGCGCGCCGAGCATC

GCGCTCGGGATGACGAGCGCGTCGTCGGGCACGTCTCGCGGGTCGATGAG

GTCGACGGGACCGTGCTCTTCGATTGCCTGCTGTGCCATCAGCTTCCCGA

TGTAGGGGTCGCCCCCGCCGCCCGTCCCGAGGACGGTCGCGCCGGTGGCG

AAGTCGTCGATGTTGTCTGTGGTTATCTTCATGGCTCTTGGTTCGATGCG

ACCCCCTCGTCGGCGACCGCCGCGACGGCCACCTCGGGCACGTCGTCGAA

CGCGTCGGCGTACGCCTCGTCGTAGATGTGACACCGGACGCTGGGGCCGG

CGTCGACCGCGACGAACGCCGGTTCGTCGGCCGAACACGCGTCGGTCGCG

TAGGGACACCGCGTGTGGAACCGACAGCCCGTGGGCACGTCCCGCGGACT

CGGAATTTCGCCCCGGAGCGGTTCGTGCCGCGGCTTGTACGCCTCTTCCT

CGTCGGTCGTGACGGGAATCGCGGACAGAAGCGCCCGGGAGTAGGGGTGT

CGCGGCCGCTGGAAGACCTCCTGCGTGGGGCCGCGCTCCTGAATCTCACC

GAGATACATCACGGCCGTCTCGTCGGCGAAGTTGCGGACCAGAGAGAGGT

CGTGGGTGATGAACAGGTAGGTGAGCCCGAACTCCGCCTGTAAGTCCTCG

AGGAGCGCGATAATCTTCGCCTGCACGCTCACGTCGAGGGCGCTCGTCGG

CTCGTCGAGCAAGAGCAGTTCCGGCTCGACCGCGAGCGCGCGGGCGATGT

TCACCCGCTGTTTCTGCCCGCCGGAGAGTTCGTGGGGGTACTTGTTCCAG

TACTCGTCGTTCAACTCGACGCGTTCGAGCAGGTCGACCACGCGCTCCCG

GAGGTCGGACGTCGCCACGCCGCGGGCCTTCAGCGGGACCGCGAGCGACT

TGCCGATAGTCCGCCGTGGGTTCAGACTCGACGTGGGGTCCTGAAACACC

ATCTGGACGCGCGACCGGAGGCGAGCCAGTTGCTCGTCGGCCGCGTTCGT

GATGTCCTCGCCGTCGAACCTGACGGTCCCGCCGGTCGCGCCGGTCAGGC

CGATAATCGTCCGAGCGACGGTGCTTTTGCCGGAGCCGGACTCGCCGATG

AGCGCGAGGGTCTCCCCGCGCTCGATGTCGAACGAGACGCCGTCGACCGC

TTTCACGTAATCGGACGTGCGCTTGATGATGCCGCTTTTGACGGGGAAGT

ACTTCTCGAGATCGTCGACGTCGAGCAGGGGTTCGGACACGTCAGTCACC

CCCCGTCTGCTTGCCCGGTCGCCGGGTCATGTGGCTCCGGGTCTCTTCGA

CGGTCGGCCGCTCGGACGCCGGGACGACCTCGTCGTAGAGGACGCACTCG

ACGCCGGTCTGCGGGCCGCGCTCGTACATCTCCGGGCGGGCCTCCTCGCA

CTCGGCGGTGGCGTAGGGACACCGCGGCGCGAACCGACAGCCCGGCGGCG

GGTCGAGATACTCGGGAATCGAGCCGTCGATGCCGGTCGCCATCTCGTCG

CCCGTCAGGCGCGGAATCGACGCGATGAGCCCCTGCGTGTAGGGGTGTTT

CGGGTTCTCGAACAGCTCGTCTGTCGGCGCGGTCTCGACGATGCGGCCGC

CGTACATGACGTACACGCGGTCGCTCACCTGTCGAGCGACGCCGAGGTTG

TGGGTGATCATCAGGACGCTCAGGTCGTGGTTCTCGATGAGATCCTTCAG

CAGGTCGAGAATCTGGTCGTGAATCGTCACGTCCAGCGCGGTACCGATTT

CGTCGGCGATGAGCAGGTCCGGCTCGTTCAGCAGGGCCTGCGCGATGAGC

GCGCGCTGACGCATCCCGCCGGACAACTGCGACGGGTACGAGTCCATGAT

GTTCTCGGGGTCCGGCATCTGAACCTCGCGGAGCATCTCCAAGACGCGCT

CGCGCGCCTCGTCGCGCTCGCCGCTGAAGCGGCGGCGGAAGAACTCCACG

ACGCCCGTGTCGCTGTCGCCGCCGAACTGCGCGGTGTCGGTGAGCTGTTC

CCCGATGGTGAACGTCGGGTTCAGGCTCGACATCGGGTCTTGGAAGACGA

GGCTCATCCGCTGGCCCTTGACGCGCTCGAACTCGCGGTCCGAAAGCGAC

AGCAGCTTCGTCCCGTCGAACGTGATGTCGCCGCTGACGCGGGCCGGCGG

CTGATTCAGGAGGCGGAGGATGGACTTCATCGTCACCGACTTGCCGCAGC

CGGTCTCGCCGGCGATGGTGACGACCTCGCCTTTGCCGATAGAGAGGTTG

ACGCCGTCGATGACCTCCGCGAGGCCGTCGAACGACTCGAAGCCGACTTC

GAGGTTCTCGACGGTCAAAAGCGGGTTCTCGGCGACCGGCTCGTCGAGTC

TCATCGGTTCACCTCCACGTCGAACAGGTCACGGAGGCCGTCTCCGAGCA

TGTTGAACCCCATCACGAGGACGAAGATGGCGAGGCCGGAGAAGACGCTC

ACCCACCACGAGTCGGGCAGGTACGACGTTCCTTGGCTGACCATCGTGCC

GAGGCCGGGCTGGGGCGGCTGGACGCCGACGCCGATGAACGACAGGCCCG

CGCCGATGAGGATGACGAAGCCCGCGTCGAGGGTCACTTTGACCAGAATC

GGCGACAGCGTGTTCGGGAGAATCTCTCGAAAGAGGATGTGCGGCGTGCT

CGCGCCGGCGAGTTTCGCGGCCTGCACGTACTCCTCGCTGCGCTCGCTGG

CGACGATGCTCTGGACGAGGCGGGCGTGCCACGTCCACCACAGCGCCGCG

ATGGCTATCATCGCGTTCGTCAGCGTCGGCTCCAAGGCCGATGTGATTGC

CAACGCCATCACGAGCGGCGGTAAGGCGAGCGCCGTGTCGGTCGCCCGCA

TGATAATCGTCTCGGTCCAGCCGCCGTAGTAGCCGGCGACGAGGCCGAGC

AGCACGCCCACGGGGACGCCGAAGCCGAGGACGACGGCCACGAGCATGAG

CGACAGCCGGTAGCCGAAGAGGATGCGCGAGAGCACGTCGCGGCCGACGT

GGTCGGTCCCCAGCGGGTGGTCGACGCTCGGCGGCTGAAGCGTGTTCGAG

AAGTCGGTGAACTTCCCGGCGTGTTCGGGGTACGGCGCGACGACGGGCGC

GAAGACGGCGAGCAGGACGATGGCGATGATGATGCCGAGACCGACGACGC

TCATCGGCCTGCTGGTAAAGCGCTTCCACGACCGTTGCCAGAGTTCGCGG

CGCTCGGCCGAGACGACCCGGTCGAGGACGCTCGTCGACTCGGCGCTCAT

GTCGCCTCCTCCATCGCCAGTCTGATGCGCGGGTCGACGCGACCCAACAG

CACGTCGACGAGGAAGTTGATGCTCACGAACGCGACGCCGATGGTCATCG

TGACGCCGACGACGGCGTTGAAGTCGTTGTTCAGCACCGCGGTGACGCCG

TAGGACGCGAGGCCGGGCCACGAGTAGACGAGTTCGATGAGGAACGCGTT

GCCCAGAAGCGAGGCGTACAACAGCCCCAGAATCGTGAGCGTCGGGATGA

ACGCGTTCTTGAGCGTGTACTTGTACGTGACGAGCCACGAGGGGAGGCCG

AAGCCGCGTTCGGCCTCGACGTAGTCTTGGTCTTTCACGTCAATCATGCT

CGACCGGGTGATGCGCATCACCTGTCCGATGCCGGCGAGCGAGAGCGCGA

GCGCCGGGAGGATGATGTGCGCCCACGCGTCGACGTGGGCCGCGAGGTTC

CCCGACAGCAGGGTGTCCACGAGCAGGAAGCCCGTGAACCGCGAGACGCC

CGAGCCGAACTCCGAGCCGAGCCGCCCCGTGATGGGGAGCCATTCGAGCA

GGTAGCCGAATATCAGCTGGAACATGATTGCGACGAAGAAGCCGGGCACG

CTCACGCTGAAGAACGCGACGAGCCGCGTCGCGTTGTCGGCGAGCCCGTC

TTTGTTCTGCGCGGCGATGACGCCCAGCGGGATGCCGATGACGACGGTGA

GGAACATCGACACCGTGATGAGTTCGAGCGTCGCCGGCAGGTAGTAGACG

ATGTCGGTGCTGACCGCGCGCTTCGTTTCGAGGCTCGTCCCGAGGTCTCC

GACGAGCAGGCCCCGCATGTAGTCGAGGTACTGCAACGGAATCGGCTTGT

TCAGGCCCATCTCGGCGGCGAGCGCCTGCACCTGCTCTTCGGACGCGAGC

GCGCCGAGGGCCATTCGCGCGGGGTTCCCGGGGAGCACTCGTGACGTGGT

GAAGATGATTATCGACAGGCCGATGAGACTCACGAGGATGCCCGCGAGCC

GGCGCACGAGATACTGCCAGTACTTCATTCTTGGTAGATGTCGGGGAACC

AGTAGTCGAAGCTCATCGACGGCCGGAAGGTGTAGCCCGCCACGTCGGCG

GCGAAGGCGTGTTTCTTCGACTGGACGAAGATGAACAGGTCGGGGTACTG

GTTCGCGATGCGTTCTTGCACCTCGGCGTACAGTTCGGCGCGGGCGTCGG

CGTCGACCGTCGAGCGCGCCTCGTCGATGAGGGAATCGACGTTCTCGTCT

TCGAGGTGTTCCATGCTCATCCACGTCGAGGCGGCCTCGGAGTGGTACTG

GTTGTAGAACACCGTGTCGGGCGAGGGGTACACCGGCCCGTAGAACACCT

GATTGATGTGGGGCGTGTCCTCGACGCTCGTGGCGAGTTCCGTCATCGTC

CCCCACGTCTGGGGGTTCAACTCGACGTCGATGCCGATTTCGTCCATGTT

CTGCTGGAACAGCAGCCCCATCTTCTCTTCGAGGCCGTAGTCCTTCACGT

AGGTGTTCTGGACGGTGATGTCGCCCTCCTCGTAGCCGGCGTCGGCGAGA

ATCTGCCGCGCCCGCTCGGGGTCGTAGGTCGGCTGGACGATGTCGTCGTT

GTGGACGCCGAACGACGACGGGAGCGGGCCCTGCGCGGGGAGCGACCCCG

GCGCGATTTCGTTGCGCGCCGTCTCGTAGTCGAAGCCGTAAGCCATCGCC

TCGCGGACCGCGGGGTCGTCGGTCGGGGCCTTCTGGGTGTTGATTTTGAA

GTAGAACGTCGTCACCGTCGGCACCGACTCGACGCGGATGTCGTCTTCGG

CAGCGAGCGCCTCGTAGGTCTCCTCGCTTTGGTACTGGCTCGACATGTCG

AGTTCGCCCGTCTTCATCAGCGAGCGGACGGTCGGGTCGTTGGTGATGAT

TTGGACGGTGACGTTGTCGTACGCGCCGTCTTTGAACGAGCCCCAGTAGT

CCGCGTAGCGGCTGAACGAGATCTGGGCCTGCCGCTCGAAGCCCGCGAGC

TCGTACGGGCCGGAGCCGGCGTCGTTGTCGTTGAGGAACGACTGGGCGTA

GTCGCCGCGGTCGCCGAAGTCGCCGTCCTCGGCGTTGTCGAGGACCTGCT

GTTTGTCGACGACGAACAGTAACACGAGCGTCGCGAGGAACGGCGAGTGG

ACGCGGTCGAGCGTGAACGACACGGTCCGCTCGTCCTCGACGGTCACGTT

CTCCTTCGAGAGCACGTTACCGAGCAGCGACGAGTAGCCTTGGTTGATAT

CGAGGAAGCGCTCGGCCGAGAACTGCACGTCCTCCGCCGTAACGGGGTTG

CCGCTGTGGAACGTCGCGCCCTCGCGGAGCGTGAACGTGTAGGTCTGGTT

GTCGTCCGAGACGGTCCAGTCCTCCGCGAGGTGCCCCTGCAAATTCCCCT

CCGTATCGGGGAACACGAGCGGGTCGTAGAGGTTGACCAACGCGAGCACC

TGCGTGTAGTCGGTCCCCTTCGCGGGGTCGACGGTCCCGAAGCGCTGGGT

CGCGCTCATGCGGAGCGTCGAACTGCCCGAGTCGCCGCCGGACGACGACT

CCGTCTCTGTCTCCTCGTCGTCGCCGCCCGAGGCCGTGGTCTGTTCTTGG

CCGTCGTAGCTCTCGCCGCCGCCGGAACAGCCCGCGAGACCGGCGATTCC

GGCCGTCCCGAGCGCCTTGAGGAAGTTCCGCCGACTCGCCGCGTCTCCGA

ACCGAGCGCGGACCGACTCCGACGCCGAACCCACCTGCGACCGCATCTGT

GTGATGTCTTTTCTCGACATACGTTACTCATCCTCACAGAGGTGCAATGG

AACATAAAGATACCGTATAGTAACCTAAAACAATATTCTCGGTTCAAAAC

GAAATGCCCGATGAACAATATGGAACAATGTGGCTGTCTGTCGTCTGCCA

CACGTCTGGTCGTGTCTCCGTAATCGACGGACTGCACGACCGTCCGAAGC

ACGTCGCCGGCTGTCGTGGACGATTGGAAATATTAGTTTTCCGCCCGCTG

GCGCTCGCTCACCGCTCGCGCCCGCGCTCTCCCACGCTACTGCGACGAGA

GCCCGTTCCCGACTGTACAGTTCTCGGTCGTTCGAGACGGATTATTATAA

GAAAGCAATGATATTTTGAAATATGACAAATTTATCTGGGGCCTCGTAGG

GGGCCTATCATAAATTCACAAAGTGGATTCTAAAACGCCTATTTATGGCT

ATAACATCTGGTTTATCTCCTTATTACTGGATATTTATTAGTCATTGTGA

CTCGACAACTGATTGCCTATCTATACTGAATCCTATATTTTAAGAAAAAT

ATATGCTTACTTACTTTTTATGACCTCACACAAGACGCTGTTCCGAACCG

TCTCTCCGGCTTGGTTGCACCCGGCTTGTTCGCCCCGTTCCATCTCGCAG

AAGTGATTCTGAAATCGGAAATCTAGTTGACAATTTGGCACGTGGTGTGA

CTCGGACGACGCTTAAGTCCCCGCGGGGAGAACGGCCACTCGATGGCATG

GGAGTTCGAGCGGGTCGCGGGTCCGTTTCGGGCCACCGAGGGCCCGATTT

GGACCGGCGACGCGGTTCGATTCACGGAGATAAAGGAGAATCAGGTCCTC

GAATACGACCCGGGGACGGGAGAGACGTGCGTCTACATCGACTCGACCGC

CGGCGCGGTCGGCCTCCACGAGGGACGCGACGGCGAACTGTACGCCTGCG

AGGCCGAGGGCCACCGCATCGCCGCGTTGACACCGGGTGAATCCGCGTCG

GTCGTCGTCGACGAGTTCGAGGGGACGCCGCTCAACGGGCCGAACGACCT

CGAAATCGACCACGACGGGGATATCTGGTTCACCGACCCCGACGACAAAG

ACCGGGGGCTGCTCGGTCACACCTCCGTCTTCCGCGCCGAGCGGGTGGAT

GCGGACGCGAGCGACGGTTGGCGACTCGTCCGCCTCACAGACGAGATGGA

CCGACCGAACGGCATCCTCCTCTCGCCGGACGAGTCGCGACTGTACGTCG

CAGAATGCACCTACGGCCCGGACCAAGAGACCGACCTGCGGGCCTATCCG

ATTCGCGACGACGGGTCGCTCGGCCCCCGCGAGATACTGCACGACTTCGG

TGACCATCGCGGTATCGACGGCATGACGCTCACGGAGTCGGGCGACATCG

TCGCCTGCGCCGGCTGGGAGAAAGGCGGCCCCGGACCGTCGATATATGTG

TTCTCCCCGGAGGGCGAGGTGCTCGAACGTCACCCGTTCCCCGAGAACCG

CCCGACGAACTGCACGCTCGGCGGGCCGGACTGCTCGACGCTCTACGTCG

CGGACATGACGGGCAGCCTCTACGCGGCCGAGACCGACCGCTCGGCGTAC

GTTCGCTTCTGACCGAAACGGGACCGCGAGGACAGCTACGCTGGAGTGGC

TGTCGTCGGAATCGACCCCGACCGCGGCGACTCCGACGCGGGCGTCTCAG

CGGAGCGGTTCGAACGCGTCGAGCCCCGCTTGGGACGCTTCCATGTCTTC

GGACACCGCGCCGCCGCTCGCGCCGACCGCGCCGACGACGACGCCGTCGT

GTTCGACCGGGAACCCGCCGCCGAAGACGACGACGCGACCGTTATCGGTG

GTGTGGAGGCCGTACACGTCGCCGCCGGGTTCGGCTCCTTCCTTCAGTTC

GTGGGTGGGCTTCTTCACCGCGGCCGCGGTGTAGGCCTTGTTCTGTGCGA

TGTTACTCGCGACGAGCTTCGCACCGTCCATCCGGCGGAAGCCGAGGAGG

TTCCCCTCGCTGTTCGTCACCGCGAGGTTCAGCGGAACGCCGAGTTCCTC

GGCTTTCTCCTCCATCGCGTCGAGAATCGTCTTGGCTTCGGCTAGCGTCA

GCTCTTGTACCATCGACTCGACCGTCGGCGGCTCGGAGCAAAAATCCACC

GCTCACGGGGAGAACCGCCGCCGGAACGGCCACAGACGGCGATGCAGCTA

TTCGAACCGAATCGAACGCTGCGGACTCGACTGCGCGTGTTCGCGTTCGC

GTTCCCCTGTCGTCGTCGCGTCGGCTTGAACGTACGGGTTACCGCGGGAG

GGCGTTCCCCGACCGCTCGAACGACGCCACGTCGCCGGCGTCGGCGGCGT

CGATGAGGTCGTTGTTGACGAACAGGCGCTCGTCGGCCGTGTCCGACAGC

GAGAGGAACGTTCCCGTTCCCTCGGTCGCCCGCGAGTTCCGAACCGTGAT

GTCCGTCGTGTCGGAGACCGAAATCGCCGCCGCGTCGTCGTCGCGGTGGG

CCTGTCGGCCGACGAAGCCGTCGACCAGCAGGCCGTCCACGTCCTCGCAG

GCCAGTCCGCTCCGGTAGTAGTCGGGCAGGTCGCCGTCGTCGTCCCACTC

GACGGTCACGTCGCGCATCGTCACGTCGCGGGCGTTCTCGACGTGGACGC

CGGCGATGTCGTTGGCGAAGATGGGCGTGACGACCGAACTCGGCTGGAGG

TCGAAGTTACCGCCCACCTCGTCGGCGTGCTCGCTTCCGGCGAGGTCGAT

GCGGACGTTGTCGAACTCGATGGTCTCGATATCGGCGTCCGCGGAGCCGT

AGACGAGCGCGCCGTTCTCGCTGCGCGCGACGATGTTCGAGAAGCGCACG

TTCCGAATCCCGCCGAGGTCGGTGTCGCCGTCCCGCGGGACCGACGTGAC

GTAGATGGGCTCGCCTTTGCCCCACCACGGGCCGGGGAGCATGTTGGAGT

TGACGACGATGTCGGTGAAAAGCACGTTCTCGACGACGCCCGAGTCGCGG

TGCTGGATGCCGAGGCCGCGGTTCGATTCCTGCACGACGATGTTCTGGAA

CAGGCAGTCGCGAATCGTGTCGTCGGTGCCCGAGCCGAACTTGATGGCGC

AGGCGCTCGACCGGAGCGTGCAGTTCGTCACGACGATGCCCTCGCAGGTC

GTCGGCTCGTCGGGGCGCGCGCCGAAGGTGATGCTGTCGTCGCACGACTG

GATGGTGCAGTCGGAGATGTGGACGTTCTTCGTGTCGCCGAGCGCGATGC

CGTCGCAGTTCGGGATGAGCAGGTGGTTGAGGATGTCGACGCCGCGGATA

TCGACCTCCTCGGTTCCATGGAACGACAGCGTCCACGCGGGCATGTCGCG

GATGGTCACGTCGCGGACGCGGACGTTCGTGCAGTTGTTGAACCGGAACA

TCGGCCCGGGCCGGAAGTCGGGTTTGGCGACGGGCCAACCGTCCGTCCCA

CTCGTCCGCGAGAGATAGGCGTCGCCCTGCCGCGCCTCGTGGGGACCGTT

CGAGATGAGCGGGTGGGACGCGCTCTCGTTGGAGTGGCCCTGAATGGGCG

TGTCCATCCGCATGAACTCGGTTCCGAGGCCGTCGAACTCGCCCTCGCCG

GCGATGGTGACGTTCTCCACGTCCTCGGCGAGGAAGAAGATGCGCTCGTT

GTCGGGGCCGATGAACTTGTCCGTGAACTCCGACTCGTCTTTCGCGGGGC

TGACGACCGCGCCGGCTTCCAGATAGAGCGTCACGTCGCTTCGGAGATGG

ACGGTTCCGACGGTGTACGGCCCGCTGGGGACGGTGACGGTCCCGCCGCC

GGACTCCGAGCAGTCGTCGATGGCCGTCTGGAACGCCTCGGTCTGGAGGT

CGGCCGTCTCGTCGGCGTAGGCTGTCACGTCGAAGGCACTCGCTGCGGGT

GGCATGTACGCGTACCCTCGCGGTGACCTATCTTTATACTACTGGTGGTC

GGAGTTCGGCCGTGAGTGATGCTCGCCCAAACGTCCTGTTCGTGATGACT

GACCAGCAGCGGTCGGACACGATTCGGGCGCTCGGAAACAGTCGAATCCA

CACCCCGAACATCGACCGCCTCGTCGACCGCGGCGTCTCGTTCACCAACG

CCTACTCGCCGGACCCGATCTGCATCCCGGCGCGGCACACGATTCGAACC

GGCTGTGAGGCCGTCTCGACGGGCTACCTCGGGAACGAAAAGCGCGACGC

GCGACACCTCGAAGACCGGTGCGGCCCCTTCCTCGCCCGGAGCATGCGCG

AGCGGGGCTACCGGACGTTCCTCGTCGGAAAGTACCACGCCCACCCCCGC

GACATCGACCTCGGCTACGACCACCACCTGAGTTTCGAGGCGTTCCGCGA

GGAGACCGGCCACGAGGTCAAGACGACCGCCCGGCAGGGCGCGATGGTCC

ATCTCCCCCAGCAGAACCGACTCCCGCCGGACGACAATTACGAGGCGTGG

ATGACCGACCGCGCGGTGGATCTCGTCGGCCGCGACACCGACGAGCCGTT

TTTCGGCCTCGTCTCGTACGAGCCGCCGCACCCGCCGTTCGCGCCGTCGC

CGCCCTTCGACCGGATGTACGACCCCGAACGGCTCGAAAACCCGGTGAAA

GGCGACCTCGAAACCGACCACATGGACGAGAAGATCCCCGCCCAGAACCA

CCACTTCTGGACGACCCGCGAGGGCGGCATCGACGACACCACCGTCCGGG

TCATCAAGGAGTACTACTACGGGATGATTTCCGAGGTCGACCGCCGCATC

GGTCGCCTGCTCGACGCGGTCGAGGCCCGCGACGACGCCGACAACACCCT

CATCTGCTTCTTCTCGGACCACGGCGAACTCCTCGGTGACCATCGCGGCT

GGGAGAAGTCGTCGTTCTTCGAGTCGTCGTGTCGCGTCCCGTTTCTCGTG

AGTTGGCCCGCGGAGCTTCCGGCCGGCGAGCGCCGCGACGACCTCGTGTC

GCTCACCGACCTGTTCGGCATCGCGACGACCGCCGCGGACGACCAGGAAC

TCCGCGATGGCGTCGACGTGCTCGGGACGATTCGGGGCGACGCGGAGTCC

CGCGAGCGACTGTTCGGCTACCACCGGACGCCGCGGCTCCCGCCGAACTT

CACCGGGATGGTCCGCGAGGGCCGCTGGAAGTACGTGTTCATGCGCAACG

GCGGCCGCGAACAACTGTTCGACCTCGCGGCCGACCCGAACGAACTCGAC

GAGTGCGCCGCCGACAACCCCGAGGTCGTCGCCGAACTCCGCGACGCGCT

GGCGGCGAAGCTGGCCGACGCGGGCGCGGACGAGTTCGTCGAGAACGGGA

CGCTCCGGCGCGCGCCGTACCAGGAAATCGACATGGGCCGACTCGTCCGC

GAGCCGTACCCCGAGGAGCCGGCGGACGCGCTGGACTGACGCGTCGTCGT

CGCCGGGTTCTCTCCCCCGCTCTCCCCGCCGTCCGGTCCCCGAAATCGCC

GGTCCCGCCGTACTTTTATGACCGTTCCGCGTAACCGACTACCACGAATG

GAGATCAGCGACATCCGTACCATCCGGTTCAGCTGTGCGTCCCACATCGA

CCCCGACGAGAAGGGTCACGGCCACCCCGGCGAGCCGAGGGAGTCGACGC

GGACCATCACCCACGTCGTCGTCGACGGCGGCCCCGACGGCTACTGCCGC

GGCGGCCACGAGGCGGCCAACGAGTTCGCCAAGAAGCACCTCGTCGGCGA

GAACCCGCTGTACCGCGAGAAGCTCTGGCAACTGCTCTGTCGGGCCGAGC

GACTGAACAAGGGAACGCTCACCGACAAGCGCGTCGCGCCCATCGACTGC

GCGCTCTGGGACATCGCCGGCAAGGACGCCGGCCTCCCGGTCTACCAACT

CATCGGCGCGGCCCGTGACTCGGTCCCGGCGTACGGCAGTACGATGGTCG

GTGACGACGACCCCGACGGACTCGGCTCGCCCGAGGCGTACGCAGACTTC

GCCGAGGAACTCGTCGCGGAGGGGTATCAGGCCGTCAAACTCCACACGTG

GATGCCGCCGTTCGGTGACGACCCCGAACAGGACATCGCCGCCTGCCGGG

CGGTCCGCGAGCGCGTCGGCGACGGCATCGACCTCATGCTCGACGCCCAC

CACTTTTACAGCCGGAGCGAGGCCAAGAAAATCGGGAAGGCGCTCGAAGA

CCTCGACTTCCGCTGGATAGAAGAGCCGATGGACGAACACTCGATGTCCT

CCTACGAGTGGCTCTCGAACGAACTCGATATCGCCGTCGTCGGCCCCGAG

ACCGCCGAGGGTCGGATGCACACCCGCGCCGAGTGGATTAAGCGGGACAT

CGCAGATATCATCCGCGTCGGCGTCTACGACGTTGGCGGCCTCACGCCGG

CGTTGAAGGCGGTCGGCCTCTGCGAGTCGTTCAACATCGAGTGCGAGATT

CACGGCCGCGACGCGCCGAACCTCCAACTACTCGGGACGATGGCGTTCCC

CGGCCGGTACTACGAGCGCGGCCTCCTGCACCCGAAGCACGACTACGAGA

CGTACTTCCCCGGTCTCGAACACCCGCCGGACGAAATCGACGACAACGGC

GTGGTCGAACTGCCGAAGACGCCCGGCCTCGGCTACGCGTTCGACTGGGA

CTACATCGACGCCAACCGCGTCGCGTAACTACCTCGGCGGGCGGGACGGA

ACGACCCCGAGTGCGACGAGAGAGAACGACGACGCGACGGCGGTCGCTCG

CGAAACGCTATCCCCGTTTTTCCGAGTGGTGATTCAGTTCGCCGTTCCGG

TCGGTGGCCGAGTCTCGGAGGTCAATTGTACGCGCCCCAGAGTCGGAGCA

ACAGGTAGCCGCCGACGGCGACGCCGACGAGCGAGATGCCCCAGACGCCG

AACATTCCTGCAATCGTCCCGTCGAGGACGAAACGCGCGAGGAGAATCAT

GCAGACGCCGGCGAGCGCGGTCATAATGAAGTTCCGGTTCGACGCCGCGG

CCTTCCGGAGCGAAGGCACGCGCGATGCGTTGACTGACATACGTACCCAC

ACGGTCGCTCTCCGCACACAAATAGATACTGCTCGCTCGGAGGTCGTTCG

ACCGGCTCGGACGGCTCTGCGGGGCGAAAACGTGGTGCTGGAAAGCGGCT

GACGGTCAGCGGTCAGCGGTCGGTGGTCGGCGGTCGATAGTTGGCCGCGC

ACGGCGTCCGCGAGCCCGGTTCAGTCGTCGACGATGCTCGGACTGTCCCA

GTACTCGTGGTCTTCGTCGGCGCGGAAACACCGCGCGGCGTGGCCGGTCT

CGTCTTCGAACGCGCCCGGGTCGTTCGTCTCACAGACCTCGCGCGCCTTC

GGACAGCGCGGGTGGAAGTGACAGCCGCTGGGCCGGTTCGTCGGGTCGGG

GATGTCGATTTTCCGAATCGGAAGGTCGTCGTCCTCGTCTCCTTCGTAGA

GGTTCGGGGTCGCCCACTTCAGCGCCTGCGTGTAGGGGTGCTGAGGGTCA

CCGATGACCTGCTCTATCGGCCCGACTTCGACGATGCGGCCGAGGTACAT

GACGGCGATGCGGCCGCCGGACTTCTCGGCGATGTACCGGGCGTTCGAGA

GGTCGTGGCTGATGAAGACGTAGGAGGTGTTGAACAGGTCTTGCAGTTCG

AGCATCAGGTCCATCATCTCCACCCGGAGGCTCACGTCGAGGGCGCTGAT

GGCCTCGTCGGCGAGGATGACGTCCGGGTTCAAAAGCAGCGACCGAACCA

ACACGACGCGCTGTTTCTCGCCGCCCGAGAGCTGGTGGGGGAACCGCTCG

ACGAAGTCCTCGGCGGGCGTGAGCCCGACGCGTTCGAAGAGCGCGTAGAT

GCGCTTTCGGCGCTCCTCCTCGCCGAGACTCGGGTTCCACTTTTTCAGGG

GGAGCATCAGCGAGGTGAGGATGCGCTGGTTCGGATTCAGCGCGCTGCCG

GGGTCCTGGTGGATAATCTGGAGCGACCGCCGAATCTCCGAGAACGGGAT

GTCCGGGTCGCGGCTGTTTTTCGCCTCCCAGATATCCTGTCCGCGGTAGG

TAATCGAGCCGTGGGTCGGCTCCTGCGCGCCGATGATGGCCTTTCCGAGC

GTCGACTTACCGCAGCCGGACTCGCCGATGAGCGTGACCACGTCGTTTTC

CCCGATGTCGAGCGAGATGCCGTCGACGGCGCGGACCGTCTGTCGCTCGC

CGAACAGGCCGAGGAAGCCGCTGCTGTCCGTGAAGTGGACCGATACGTCG

TCCACCGAGAGGATGGAGTTATCGACGCTCATTCGGCCTCACCGCCCTCT

TCGGCCGCTTCCATCGCGGGAATCGGAATCTCGTCGGACACGTCGTCGTG

GTAGAAACACGCGGTTCGGTGGCCGCCGCCGCGGTCCGCCATCGGTGGCT

CCGCCTCCGCGCAAGACTCGTCGGCCATCGGGCACCGCGGCCGGTACGAA

CAGCCGGGGATGTTGGTCACCGGGTCGGGCTTCGACCCCGAGATGGACTG

CATCTCCTCGATGGGGGCGTTCAGGTCCGGCGTCGCGTTGAGGAGCGACC

GCGTGTAGGGGTGCGCGGCGTCGTACAGTATCTCCTCGGTCGAGCCGATT

TCGACGATGTCGAACGCGTACATCACGACGAGCCGGTCCGCGAGCTTGGT

GAGAAGCGGCAGGTCGTGGGTGATGAAGATAATCGTCAGGTCGTACTCCG

CTTTGAGCTCCTTGAGCAGCGAGACGATAGAGCGCTGCATCAGGAGGTCG

AGCGCGGCGGTCGGCTCGTCCATCACGAGCACCTCCGGTTCGAGGATGAG

CGCGAGCGCGACGAGCGCGCGCTGTTTCATCCCGCCCGAGAGCTCGTAGG

GGTACGAGTCGAGGACGCGGTCGGGGTCTAAGTAGAGGTCGCCCAGAATC

TTGCGGGCGCGGTCGAGCCCCGCCTCCACGTCGCGGTTGTGGTCTTCGAG

GGTCTCCCTGAAGTGGCCGCGGACCTTCTTGACGGGGTTGAACGAGCTCA

TCGCACCTTGGAACACCATGGCGACTTCGCCCCAGCGGAACTTCCGCAGC

TCTTCCTCGTCGAGGTCGAGCACGGAAATCGGCTCGCCGCCGTCGGGCGG

GTAGTAGGTGACCTCGCCGGAGGTCTCGCCCGGACTGACGACCGCGTTCA

TCAACGCGGACGACGTCATCGACTTGCCGCTGCCGCTTTCGCCGACCATC

CCGAGCGTCTCGTTGCGGTAGATGTCGAAGTCGACGTTGCGGACGACTCT

CGATTCGCCTCGGTTGGTCCCGAAGGTGACCTCCAGGTCGCGGACTTCGA

ACACGACGTCTTGGTCGGGGTCCGAGTCGTTCTGATTCGACGTTCGCATT

GCTTGGTTGGTAGTCATCGTCTGGTTATAGGTGTTGGCACGGGGTCAGTC

CCCGCCGGTCGCCTGCGATTGGTTGTTCTTCGAGGTGCCTTTCTTCGAGG

TGTTCGCGTGGCGCGCCCGGAGGCGGACGTTGAACAGGCTGTCGAGCCCC

TGCGACAGGAGGACGAGCCCGAACGACAGCAGGAAGATACAGACCATGGG

CGCGACCATCCACGGGAGCATGTCCGGGCGGGTGAGCGCGTTGCCCTCGT

ACGCGAGGTTCATCATGACGCCCCAGTTGAACGTGGTGAACGGCAGGATG

CCGAGGAAGTACAACCCCACGGACTCGAAGATGATGCGCCGCGAGGTGAG

CGCCCCGTTTATCATCACGTACGGCATGAGCTGCGAGAGGATGTCCCGCC

GGAGAATCGACGACAGCGGGATGCCCATGATTTCGGACGCCTCGACGTAG

GACTCCTCGCGGATGCTCAGCACCTGCGAGCGGACGGTCCGCGCCAGCCC

CGGCCAGTTGTCGATGCCGAGGAACACTCCGACGATGTAGGGGTTTTCTG

GCTGGAAGATGGCCGTGATGACGATGATGAGCGGCAGACCCGGAATCGTG

ATGACCACGTCGGTGACGGTCATCAGGAACGAGTCGATGCGCGTGCCGTG

TTCGAAGCCGGCGACGACGCCCACGAGAGCGGCGAAGCCGACGCTCACGA

CCGCGCCCGCGAATATCATCTTGAACATGCCCGGCGTCGCGTGGATGAGC

TGGGCGTGAATCGGCTGGCCGAACACGTCGGTGCCGAGCGGGTACGCCAG

CGATTCGAACGGCGCGGCGAGGACGGGCGCGTCACCGCTCGTCGGTCGCT

CGACGAGCCAGACGCCGACGGTGCCCATGAGGACGAAAAAGGCGAGAATC

GCGCCGCCGACGAGCGCGCGCCAGTCGTCGGCCGCGATGCGCGCGGGTGC

GATAACGTTCATTTCGAGGAATCGCTTTGCGCGGTCGGCACCCGATTCGG

GGTCCGGGTCGATGTCCTCCTCGTAGAGGTCTTTCAGGTCGAACGTGTCG

GAATGTGCTTCACTCATGGTTACTCCTCACCCACCGAGATGCGCGGGTCG

ATGAGCGTGTACGTGAGGTCGGCGATGAAGATGGCGGTCAGCGAAATCGC

CGTGAACACGAGGAGCGTGCCCATCAACAGCGGGTAGTCTTGCGTCTTGA

CCGCCTCGAACATCAGGAGACCCATGCCCGGGTACGCGAAGACCTCCTCG

ACGATGACGGCGCTGCTGAAGACGCCGGCGATGCCGATCATAAACTGCGT

GTACATCGGGAGGATGGAGTTGTGCCCGAGGTACTGCGTGGCGATGCGGG

TCTCGCGGAGCCCGCGAAGCTGCGCCACGCGGATGTAGTCCTCGCCGAGG

ACGCGAACCGCGTTCCCGCGCATGGCGAGCGCGCCGGAGGTCGCGAGCGC

GCCCATCGAAATCGCCGGGAGCGCCGCGTGTTGCACGAGACTGAGCATGA

ACTCCGGATTGTACCCCGCGGCGATGCCCGAGCCGATTCGGCCGCCGGTC

GGGAAGTAGTTGTTCTGGATGGCGAGGAAGTAGATGAGCACGACGCCGAC

GATGTAGTACGGAATCGAGTTGAGTCCGATGATGAGCGATGACACCGTGG

AGTCGAACCGCGACTTCTCCCGGTAGGCCGTCATCACGCCGAGGAAGATG

CTCAGCGAGTAGCCCAGCGCGAGCGCGTAGACGCTGATGAACATCGACCA

CGGGATGCGCTGCATGATGAGCGTCGTGACCTCCTCGTTTTTGAAGAACG

AGGTGCCGAGATCCTGCCGGAGAATCACGTCAGACGCGTAGTTGAAATAC

TGTACGTGCATCGGCTGGTCCGGGTTCACGTTCGTGTAGAGCTGTATCAT

CCTGTTCAGTCGGTCGATATCGACGCTCCCGCCGCTCTGCGAGAGGATCA

CGCTCCGCATCGCTTCCGTCGGGCCACCGGGCATGGCCTGGTACAGCAGA

AACGAGACCGTCATCGTGACGAAAAACGTCAGTATCGCTTGCCCGAGACG

CTTCAACAAATACTTTGGTGTCATTATTAATAGTTTGGCTTCTAGCGTGA

GAGAGCTTAATTGTGGTGGTTAAGCCCGTCGAGGCTGCCGTCTCACCTCA

CTGCGAGTTGGAGCTAATCCATCCGTTTTTGAAGGAGAACTCGCCCGGTC

GGTTCGTGTTGAGTTCGTAGTCGTCGCCCGTCGGCATGGAGAAGTCCTGC

GTGTCGCCCCAGTAGCCGGAGTCTTCCTCGACGAAGACGAAGTCGGGAAG

CGCGTAGTTGAACCACTGGACGAGGTCGCGGGTCCGCTCTCTGACCTGCT

CTGCGGACTCGGAGTTCGGCAGGTCGTTCATCAGCTGTGCGGGCTGAATC

TCTTTGCCGCTGCCGGAGATTTCGGTCGCGCCGACCTCGCTCGGGATGGT

CGTCGTAAAGGGCTTGCCCGTCGGGCCGGTCTCGTTCCCACTGGAGGGGT

CACCGACCTCGATGCCGTAGAAGTTGTTCGAGAAGTACGCCGTCGGGTGC

CACAGCGCCTTCGCGACGTGCCATATCCAGAAGATGTCCGCCTCGTACGT

TTGGAGGCGCTGGTAGTAGTCGGTCGAGCTGACCGTGCTGATTTCGGTCT

GGATGCCGAACGCGTTGAGGTGGTCGTTGAAGACCTTCGTCGCCTGCACC

TGGGTGTTGTTGTTCTGCGTGAGGACGGTGAACGTGAAGGACTCGCCATC

AGGGTCGACCCACGTCCCGTTCTCCTTGGTGTAGCCGGCGTCTTCGAGCA

GCTGCGTCGCGGTTTCGAGATCCTTGCCGACCGGGTAGTCGATGAGCTGG

TCGACGAAGTCCGAGCCGAGGTACTCCTCGTGAATCGTCGAGCGCAGCGC

CGTCTGGTTCTCGGCGGGCGCGCCGACCGTTCCGGCCTGCCGCATCGCGG

CGACCATCGACTCGAAGTCCACCGCGTTGACGATGGCGCGTCGGACGGGA

AGCCTCGCGATGTGCTCGTTCTTGAAGTTGAACGTGAACTTCTGGGTGCG

GAACCAGTTGAGCCGGTAGACGTTCTCGATGTTGTCCGGGTACCGGGACT

GCTGGGACTCCGACATGAGTACGTCGGGGTTCATGTCGAGTTGGTCGTTC

ACCGACAGCGACTCCACGTCGGAGGTCGTCTCGGGGATGATGCGGACGTT

CGGGACGTCCGTCCGGCTCGCGTACGGGTGGTCTTCGAACTTCTCGAGGA

CGGTCTCCGAGGAGTTGAAGCCGGTGACGCGGTACAGCGCGTTGCCGAGA

CCCTCGTCGATGAGCTGTTGGGTCGTGATGCTCATGTCGAGGAGGTCGCT

CGTCACGCTCTCGCGCTCGCTCTCATTCGATGCCTCCTCGTAGCGGGTGA

GGTATTCGCGGTAAATCGACCGCGGGGTGTTGACGAACGTCTCGGCGACG

TTCGCCTTCATCAGGTTGGGCGAGACGGCCGTCTTGAACGTCCGCTCGAT

GGTGTAGTCGTCGACCAGCGTGTTGCTCTCGATGGGTGACTGCTCGGGCG

ATTGCAGGCGGTCGATTTCGAGACCGATGTAGTAGTCTTCGGCCGTCAGG

TCGTCGCCGTTCCACCACTTGAACCCCTCGGGGAACGTCAGCGTGAGCGT

CCGCCCGTCGACGGTCATGTCTTCGAGCAGGTCGGGTCGGACCGTCCCGT

CGGCGTAGCCGCGCGCGACTTCCTCGAACAGTTGGTCCTGCAGGGTGTGC

GCGTAGTTGCCGAGGCTGTACCGGTTGAACTGCACGTCCGTCGCGGGTCG

GCCCGCGGGGTAATCGAGGGTGTCGTCGGCGATGCTCGCGCCGCCCTCGG

TGGTCGTGCCGCCGTCCGTGCCGTCCCCGGTACTGCCGTCTTCGGTCGTC

TGCCCGGAACTATCGAAGCAGCCGGCCAGTCCAGCCGTGCCAGCCACCGC

CCCGTACTTGATAAGGTCTCGTCTACTCCAGTTGGTTGAACGCTTTCCAG

CCATGCGGTATCATCTATCATGATACTCCCCGTTAATATAGATACTGGAT

TTGTCCATCCCAACCATGATAGAGAAAATAACAGTTCTCTGCCCGTTCGT

GCTTGTTTTCGCCCCGTCTCACGCGCTCCTCGCCGTCCACAGCAACAGTC

CGAGCATCAGGACCGCCAACCCGCCGAACGTCGTCAGCTGGAGGTAGACG

AGCGCGTGGGTCGTCGTCCCCGGTTCGATAGACGGGAGCGAGATGAGTAG

CAGGACGATAGAGGCCAGCGAGATGACGAGGACGAACTTCATCCGGGTCT

CGTAGTCGCGGAGCACGTCGGCGAGACTCGTTTCGGGCATGCCTACGTAG

AGTACCGCGGTGTATTTCAGTATACTGCCCCGGCCAGCCTCACCCTCACC

CTCGCCGTCCCTTTCCCCGGTAGACTTAGATAAGACGAGACGAAATGCAG

GTGCATGGACAGAGGATTAGTACTCATCGAGAACACTGACTTACACGCGG

ACCTCCTCCGGGAGGCCAGAGAACACGCGCTCGGCGCGGACGCCGACCTC

GTCCTCCTCGTCACGCTCACCGAAGACGAGTTCGAGGAGACCCAGGAGGT

CCTCGACACCATCGGCGACGTCGAGCACACGTCGTACACCGAACAGGACG

CGTTCAAGGGCGCGATGAACGACGCCGAGGAGGTCGCCCGGAAGGTCTTC

GCGACCGACGACGAGGTGTCCTACGAAATCGTCCCGCGAATCGCCGCCGA

GAAAGAGCGCGCCGAGACGGTCATCGAAGTCGCCGACGAGGAGGGCGCAG

ACCACGTGTTCATCCTCGGCCGGAAGCGGTCGCCGACCGGAAAGGCGCTG

TTCGGCGACCTCGCGCAGTTCGTGATTCTCAACTTCGACGGCTACGTGAC

GCTTCACACCGAGTGAGCGTCGTCGCGGCGGACTGAGCCGACTCTCGTTT

TTTCTGCCCCCGCGACCGCGTAGCCGTCTGTACTCAGTCGTTCGAGACTA

CTCCGGCGGGCTGTCCCACTCCTCGCCGTCGTCCTGCGGGTGGTAGCCGA

TCTGCGAGCGGGCGTGTTCGAGGTCGTACCACCGGCGCTGGTTGTCGCTC

ACGCCGCTGAAGATACCGAATTCGACGTTGTCGTCTTGCAGACAGCAGTC

GATTTCGTGGGCGAAGTCGCGGCGGGACTGCCACATCGACTTCATGCGCC

CGACGGTTTCCTCGTACTCGGCGCTCCCGCGTTCGAACTCGCCGTTCTCG

ACGGCGGCCTCGGCGTCGCCGTAGGGGTGGTCGTACTCCGGCATCCGGAC

GCTGCACACCCGGAGGGCGTAGAACTGCTTGGGGTATTCGTAGTTCTCGA

CGTAGTACCGACCCATGTCCTCGCCGTACGCCTTCGACGCGCCGTAAAAC

GAGTCGGGGCGGACGGGGTCCTCGTGGCCGATGACGAGTCCGTGACCCGG

CTCATATATCTCGGGGGAGAACTCGTCCTCGTAGCCGCCCATGACGTGAT

TGGTCGAGAGGAAGACGAACGACTCGATTTCGTTCTCGCGGGCGACTTCG

AGCGCGTTGTACATCCCGATGATGTTGGGCTCGAAGATGTCCTCCCACGT

CCCGTCGGTGTAGGGGTACGCCGCCATGTGGACCATCGCGTCCTGCCCCG

CCGCGGCGCCGTCGAGCGCGTCGTAGTCGGACACGTCGCCGACGACGGTG

TCGTAGCCACCGTAGGGGTGGTCGTCGGGACGGTCGGAGCGGTTGAAGTA

CGTGAAGTCGTAGGCGTCGTCGTCGTGCAGGTGGTCTATGACTGCGGTGC

CGCACCGGCCATAGACGCCGGTCATCAAGACGTCCATACGAATCCACTCT

GACCAAATCGTAATAAATTGATGGGTGACCGTGACGTCCGACTTCGCTTC

GGGTATCGCTTCGGCCCGCCCGTTCGGGTCACGAAGCCTGAATGCCGCGC

ACCAGATGGACGACTCGCGGGCCCTCTCGCGCGTGAACGCCGGGTTCAGG

CGTTGACGATGGGTTTATCACGGTTGACTCAGATTGACTGTACCATGCCA

CTCGCAGACGACCGTGTACGCAGTCGTCTCCGTGGCGTCGCCGTCGGCTT

GCTCACGCCGTTCGACGACGACCTGGAGATTCAACACGACAAACTCGAAG

CGAACGCGAAGTCGCTTTCCGGCGACGGTATCTCGTCGTTCCTCGCGGCC

GCGAACATCAGCGAATACCACTCCCTGTCCCAACAGGAGCGCGTCGACGT

GACGGAGACGGCGGTCGACGCGCTCCCCGACAGCGCCTGCGTCCTCGCCG

GCGTCGGCGGGTCGACGAAGGCCGCGACGGACCTGATGGAAGCCTACGAC

CGCGTCGGCGTGGACGCCATGATGGTCATGCCGCCGGACCACACCTACGT

CCACGAGCGGGGACTCATCCGCTACTACGAGAAACTCGACGCCGCGACCG

ACACGCCGCTCGTTCCCTACGTCCGCGGCTTCGACCCCTCGGTCTCCTTC

CTCCGCGACCTCTCGCGCATCGAGGGCGTCGTCGGCGTCAAGTACGCCAT

CGAGGACCCGGTCAAACTCGGTTCCGCCGTCGCCGTCGCGGACGACGACG

TGGTCTGGGTGGACGGCCTCGCGGAACCCTACGCCGTCTCGTTTTGGAAC

GAGGGCATCGAGGGGTTCTCGGCGGGCGTCAGCAATTTCCGCCCGGAAAT

CGGCCTCGCGCTGTTCGAGGCGCTCACCGAGGAGAACTGGGAACGCGCCC

GCGAGATTCGCGACCTGACGCTCCCGTTCCAAGCTCTCCGCGGCGAGACG

GGAACCGACAACGACCTTCCCGGCGCGATAAGCGTCCCGATAGTGAAGAA

GGGCCTCGAACTGGCCGGCCTGCACGGCGGGGAAGTCCGCGAGCCGATTC

GGTCGCTGTCGGCCGACGACGAGCGCCGCGCCGAGGAACTGTACGAGGAA

CTCGACGACGGCATCGCCCGCGTCATCGACTGACTCACCGACATCGACCC

GAGCGCGCACCGACGACTCCGACCGCACCGATATCGACTGACCACGCTCA

CCGCACCTGCCTGACGCTACCCGGCGACAGCCGTCGGAAGCACCCTACCC

CATTTTTCGCCGTCTCGACGTTCCCTCGCTCCCTGTTGAGCCGTTTTCAC

GTCTCACCGGCCCGCTCGTCTCCCACCGGACTCCCGGCCCGCACTAATCA

TAACACACCTTCAGCTCCGGCAGTTGAGCGGCCACATTCCTAGACTGACC

GCATGAGGGCAGCGTCATACTCCATCTTGTTCGCCGGTGGGGAACGAACA

TCAGGCGAATCGTGCCCAATTATTCACTCTGGATGGGATTTGCGTTCCCC

GGTGGCGAATATTCGGATTTAGGCCTCTCTGTGGGCTCTGGATTGGGTTT

AGGGGGGTTGGCGTTTTGGTCGGCTGTGCGTCACTCACGACGAACGCGCG

AATTCGACGCGATATTCGCCCATATATTACAAGCATATGACTGTAATTTT

CCCGCCTCGGGAACCGATGATGCCACTTCGGCGTCTGTCTCCGTCCAGCA

CCGGCGCGCCCGTCGTCGCGCGTCGGTCGCCGGAGGTAGGGATAAGTAGG

TGTGGCGTGTGCGTGACTCACACGGTATGCAGCACGACGATATCCAGCAC

GACGACGTTCAGTTCCACAACGTCAGGGCGCTCCAGCGGGTCAGACCGCA

CGAGGGACTTCGACTGCTCCGCGTTCCGGAGGCCGTCAGGAGCGGCCTGA

ACACCGGCGCGCAGACGCGGATGTCTCACCCGGCGGGAACCGAGATTCGA

CTCGTCCCCGATGAGACGGTTCGAATCACGCTCTCCTCCGAGGAGCGAGA

CAGCCTCGTTCGACCGTTCTGGGGAGACTTTCAGGCGACGGGCGAGGAGT

TCACGCTCGGGCCGGAGCCGAAGACGGTCGAACTGTCTCCCCCGGAGTCG

CTGTCCGACCTCGACCCGGCGGCGACCGGCGAGATGCGTTACGCGCCCGA

GGTGTGCCGACTCGTCTTCCCCGGCGACCACCGCGGCGGCCACATCTACT

ACCACGGCGTCGAGGGCGCGCGGCGGCCGCCGACCGCCGACGAGGTTCCG

AGCCTCCGATACCTCGCGTACGGCACGTCGATTACCGAGGGCGAGGACCC

GTCGGCGGAGATGCTCACGTACGTTAACCAGACGGCCCGGAGACTCGGTG

CGGACCCGATAAATCTCGGCTCCTGCGGGACAGCCTACTGCGACCCCGCG

ATGGCCGAGCACATCGCCGCCCGCGACGACTGGGACGTGGCGACGCTCTC

GCTGTCGGTGAACATGGTCGGCCGATTCTCGCCCGACGAGTTCCGCGAGC

GCGCCGCGGACATGGTCGAAACCGTCGCCGCGGCGAACCCGACCAAACCC

GTCGCCTGCATCACGCTGTTCCCGCACGTGCGCGAGTACAAGCGCGACCA

CGAGGAAGCGACGCTCTCCGAGACGTTCCGCCAGCACCTTCGTGACGTGG

TTGACGAGTGCGGCTACGACAACGTCCACCTCGTGGAGGGCCCCGACCTC

CTGCCGACCGCCAGCGGGATGACGACCGACCTCGTCCATCCCGGCGACGA

CGCGATGGTTCGCATCGGTGAGCGCCTCGCGCGCGAGCTCGAATCTCTCA

TGGACGATTGACCGGGATATGCGCGACTCAGACGCTCTCGTAGTCGAATT

TTCCGAACGAAATCTCCGCGAGCGTAACACCGAAGCACCCCCCTGTTCGA

TGGACGTGTATGACCGTCGCCGCAAATCTGGGCTACATGCTCGGGGTGCT

CTGCCTCGGGGCGCTAGCGAAGCGTCTCGGCCTCCTCGACAGCGGCCGCC

GCGACAAACTCACGTTCTTCGCGTTCGCCTTCGCGCTCCCCGCGCTCGTG

TTCACCTCGACGTACGACCAGCCGATTCGCGAAGTTATCGAGCCGACGCT

CGTGTTGGGCTTTTGGCTCGTCCTCTTTACGATGCTCGCCGTCGGCTGGG

TCGTCCACCGGCGGGTGTCGCCCGACTCGGTTCGCAGTGTCGCCATCGTC

CAGTCGTACTACTCGAACCTCGGCTTCCTCGGGCTCCCGCTCGTCGATTC

GACGTTCGGTTCGCTGGCGTCTGCGAAGGCGGCCGTCATTCTCGGCATCG

GCGCGCTCACCCACGTCCCGCTCACTATCACGGTCCTCGTGCTCGTCAAC

GGCGCTGACGTGTCGTTCAAAGAGGAGTTCATCGGCGTGCTCAAGACGCC

GGTCATTCCGGCGCTCGTGTTGGGGCTCGCCTTCTCCGGGCTCGGCCTCG

GCGTCCCCGGTGTCCTTCTGACGGGGCTCGACGCGCTCTCCTCGCTCGCG

CTCCCCGTGGCGCTGCTCTCCATCGGCGCGTCGCTCACGATTTCCACCGA

GTCGTTCGACTTTCGCACCGTCGGGGCCGTCGTCGGCAGCAAAGTCGTCC

TGATGCCGCTTCTCGCCTTCGCCGTCTTCTCGACGTTCGCGTCGAGTTGG

TCGACGGTCCAAGCCGGCGTGACGATGCTCGCGACGCCGACCGCCGTCTC

GTCGTTCATCTACGCGAACGAACTCGGCGGCGACGCCGACCTCGCGTCGA

TGAACGTGTTCGCGACCACCGCCGTCTCGGTCGCCACCCTGTTCGTCGTC

CTCCAGTTCCTCCTTTGAGGCGGCGGCGGCTCGTCGATACCAGTCCGAAC

CTCGCGAATTCGCTTCGCTCACACCTTGCGACGGCCACGAGCGCGCGGCG

GCCATCCGCGCTCGCCATGAGAAGCTTACTTGGTGATGGGTGTGCATGGC

TAGGTCGAATGTCGAGAACCTACGGTAACTTCATCGACGGCGAATGGACA

GATTCCCAGTCGGGAGAGACGTTCGAAGTGACGAACCCCGCGGCGGCCGG

CGAAGTCGTCAGCGTCCCCCCGGCGTCCTCCGAAGCGGACGTCGAGGCGG

CGCTCGACGCGGCCGTCGCCGCGCAGGACGAGTGGGCGTCGACGCCCGGT

CCGTCCCGTGGCGCGGTGCTCCGCGAGGCCGGCAACAACCTCGAAGCCCG

GAAGGAGGAGGCGACCGAAGTGCTCGTCCGCGAGGAGGGCAAGACGCGCT

CCGAGGCGGGCGGCGAAGTACAGCGCGCCATCGACATCTTCCACTACTAC

GGCGCGAAGGCGCGGGACTTCGGCGGCACCGTCAAGTCTGCGAGCGCCCC

CGGCAAGGACCTCTACACGAAGCACGAACCCCTCGGCACGGTCGCGCTCA

TCACGCCGTGGAACTACCCCATCGCCATCCCGGTCTGGAAGCTCGCCCCG

GCGCTCGCGGCCGGTAACACCGCGGTCCTCAAGCCGGCGTCCGAAGCGCC

GGGCGTCGTCTCCATCGTCCTCGAATGTCTCGACGATGCGGGACTCCCGG

CCGGCGTGGCGAACACCATCACCGGCTCCGGGAGCGAGGTCGGCGGCCCG

CTCATCGAGAGCGAGCGCATCGACGGCGTCTCCTTTACCGGCAGCACCGC

CGTGGGGACGATGGTCGCCGAGACGGCCGCGGTCGACCTCAAGCGCGTCC

AGTGTGAGATGGGCGGCAAGAACCCGACCGTCGTGATGCCCAGCGCGGAC

ATCGAGGCGGCCGCCGAGACCGTCGGCGTCGGCGCGTTCGGCACGACCGG

CCAGTCGTGTACGGCCACCTCGCGCGCCATCGTCCACGAGGACGTGTACG

ACGAGTTCGTCGCGGCCGTCTCCGACTACGCCGAGTCGCTCGTCGTCGGC

GACGGACTCGACGACCCCGACATGGGCCCGCACGTCTCCGAGAACGAACT

GTCCTCGACGCTCGAATACATCGACATCGGCTCCTCGGAGGGCGCGACGC

TCGAAGCCGGTGGCAGCCGACTCTCCGGCGACGACTACGACGACGGCTAC

TTCGTCGAGCCGACCGTGTTCTCCGGCGTCGAAAACGACGCTCGTATCGC

TCAAGAGGAGATTTTCGGTCCCGTCCTCGCGGTCGTCAAGACCGACTCCT

TCGAGGACGCGCTCGACCTCGCCAACGACGTTGACTACGGGCTGTCGGCC

AGCATCGTCACGCAGGACCTCAGCGAGGGCAAGCGCTTCGTCAACGAGGT

CGAAGCGGGCGTCGCGAAAATCAACGAGAAGACGACCGGTCTCGAACTCC

ACGTCCCCTTCGGCGGCTACAAGGACTCGTCCACTAACACGTACCGCGAG

CAGGGCGACGCCGGCCTCGACTTCTTCTCGTCGGTGAAGACCATCTACGA

GAACTACTGAGCCGCGGGCCTCCGCGCGCTCAGGAGAACTTCGTGTTCAG

TTCGATGACGTTCGCCGCGCTTCGGACGTAGTCCGCGAGTTCGCCGTGCA

GGCGGTCCTCGGTGAACCGGCTCGACGGGCCGGTGATGCTAATCGCGCCG

AGGATTGTCTCGTCGTTCTTGACCGGCGCAGAGACGCAGCGCAGACCGTT

GATGTTCTCCTCGTCGTCGATGCCGTACCCTTGCTCGCGGATGGTTTCGA

GCTCCTCGAACAGCTCCTCCGGACTGGTGATGGTGTTCGCCGTCTTGGGC

TCGTACTCCATCGCGGCCGCGATTTCCTCGATGCGCTCGGGCGGCAGGTA

CGCCAGAATCGTCTTGCCCAGCGACGTGGAGTACATCGGCTGTTGGGTCC

CCACCCGCGACAGCGTCTCGACGGCGTGTTCCCCCTGCGCCTTGTGGAGG

TACGAGACCTTTCCGTACTCCTCGACGCCGAACTGGACGATTTCGCCCGT

CTCCTCGGCCAGCGAGTCGACCTCTTTTTGAACGATGTCGAAGTTGCCTA

TCTGGTCGCGGACGTGCGAAGCCACGTCGAGAAACCGCAGGCCCAGCCGG

TAGCCGTCGCCCTCTCGGACGATGACGCGCCGCTCTTCGAGCGTTCGGAG

GTGGCTGTGGACCGTGCTCTTCGAGTGGCCGAGCTCGTCGGCAATTCGCG

TCACGCCGATGCCCGCCTCCTCCTGGAGGTGGTCGATGATATTGAACGCT

ATCTGGACCGAGCGGATTGTTCGGCCGGGGCGTTTGTTCGGTGGGGAGCT

CATGGTGTGGTAGTCGAACCAGAGCCGTATATATTTGTTCCCGCCTGGGG

AACGTATCGGCTGCGACGAACGAGTACCCCCGGCTCCGGACTGGCTGACC

GACTGGCCCGCGCGGCCGCCTTCCCGCGGTGGCGCGTCGCCATGGGATAC

ATTTTTCATCCCTGCACGTCTTCGTGTGGGTACTGTACCCATGCCAGCAG

ACGAGCTTACACCAGTCCAGCGCGCCGCGTCGTATCTCCGGTCGTTGGAC

ATCGACGGTCAATCGTGGATTACCGGCGTCGCCATCAACGGCCTGCTCGC

CGAGGGCGGAGACGAGTCGGTCGAGCGCGCGAAGCACTTCGTCGACACCG

CGGTGGCGACCCAGAACGACGCCGGGCAGTTGAGCTACGGCCCGAGCTAC

CCAATTGAGGTTTTCAGCCACGGCCGCGAGTACGAGGCGAGCTGGGAGCT

GACCGTCAAGAAGTGCATGAACACGAACAACAGCACCGCCATCGGTCACA

GCGTCCTCGACTTCTACGACCGCACGGGCGAGGACCGCTACCTCGACGCG

GCCGAACGCGCCTACGAATCGCTTCTCTCCTTCGAGCGGACCGAAGACGG

CGGCATCCCTCACCACGACCCCGAACTCGCGGGCATCAAGTCGCTGTGGA

TCGACTCCGTCTACATGATGTGCCCGTTCCTCGCGCGCTACGGCGCGGCC

GCGGGCGACGCCGACGCCTTCGACGAGGCGGTCGAGCAGATTCTGATTCA

CGCCGCGCACCTCCGCGACGGCCACACGGGGCTGTTCCGCCACATCTGGG

TCGAACAGCCGAACCACTACCCGCAGGGCGCGTTCTGGGCGCGCGGCAAC

GGGTGGGCCGCCGCCGCCTCGGCGGACGTGCTCGAACGGCTCCCCGAGGA

CCACCCGAAGCGCGACGACCTCCTCGACCTGTTCCGGTCGCACTGCGAGG

CGCTCCTTCCGCTCCAAGACGGTAGCGGTTTCTGGCACAACCTCGTCGAC

GACCCGCACACGCCGCTCGAATCGTCGGGGACGCTCATGTTCGCCTACGC

GTTCGCCCGCGGCATCGACCTCGGCATCCTCGATGCGGAGACCTACCGCC

GACCCGCCGAAGACGGGCTCGCAGCCGTCTCTCGCGTCGTCGGCGAGGAG

GGCGCGGTCAACCGCGTCGCCGGCCCGCCGGGCGGCCCCGAGGCTCCGCT

GACGGACACGCCTTACGGACAGGGCTGGTTCCTGATGGCCGCGGCCGCGC

TCGACTGAGACCTTCGCCCCCAGCGTTCGTTTCGACTCTTTCGGCCTGGC

ACCACAGTTATGCGCTCCGGCGCACATTGACGAACGAACTCCCGCGACAG

GAGACACTATGACTTACCACGCAGGCATCATCGGCACGGGCGGCATCGCA

GGGATGGGCATCCTCGGCATGCACGACGAGGAGGCCATCGGCAGAGAGAA

AATCGACGCGAGCCACGCGGGCGGGTACGCCGCGACCGACGACATCGAAC

TCGTCGCCGTCGCAGACGTGGACGAGTCGAAACTGGACACCTTCGGGGAG

GCGTGGGACATCCCCGCCGACCGCCGGTACGTCGGCCACGAGGCGATGCT

CGAAGCTGAGGACCTCGACGCGGTGTCGGTCTGCACGCCGTCGTTCCTCC

ACCGCGACCACGTCGTCTACGCCGCGCGCTCCGCGGCCGACCCCTCGGTC

GTCTGGTGCGAGAAACCCATCGCGTCGGCCGTCAGCGACGCCGAGGAAAT

GGTCGCCGTCTGCGACGACACCGACACGGAACTCGTCGTCAACCACTCGT

TCCGGTTCACGGACAAGCTCCGTCGGCTCCGCGACCTCGTCCGCGATGAG

GGTATCCTCGGCGACGTGAAGTCCGTGAGCGCGCAGTACCGCATGGAACT

CATGCGGAACTCCACGCACGTCCTCGACACCCTCGTCTACCTCCTCGACG

CGCGGGCCTCGCGGGTGAGCGGCCACATCACCGGCGAGAACGAGGCGCTC

GACGCGCTGGACGCCACGGAGTCGGTCGTCGACGCCGGCGGCGGCGGGCA

CGTCGTCATGGACGACGGCACGTTCGTCACCGTGGACTGCACGATTCCGC

GCGACGTCTCCTCGATGACGCTGAGTTTCATCGGCACCGAGGGCAAACTC

TACATGAACAACGACGACGGCGAGTGGCGCTACTGGTCGCTCGAAGACGG

CGAACACGTCGAGCGGTCGCTCCCCGGCATCGAGGGCGCGTGGACGTGGG

AATCGGACTACAAGGGGTCGTTCGCCAACGCGGCGGCCCACGTACAGGAA

CTCCTCGGCGGCGACGCCGAAAACTACTCGCCCGGCGTCGCGGCGACCCG

CTCGCTCGAAATCATCGTCGGGTTCTACCTCTCGCACTACACCGATTCGA

CGGTCGATATCCCGCTTCCGGGGCCGCTCCGGGAGGTCCCTATCACGTCG

TGGTGAGCCGCAGTCGGTCGGTCAGTCGCTCACGCCGGACAATCATCCGG

CATGGCCGGAAGAGCTTCTGGCGCCGGGGATCGAGCTGAATCGGACGTAT

ATCGAGGATAGAGGCCCAAATAGTGCAGTATCGGGTTTTCTATTGCTCTC

TCCCGCAGTGGTCTTCTCGTCTGGCCGGTCGAATCTGTGCTCCAGGATAT

ATCCGTGACACTCCGCATAGAAATAATTCCAAATTACAATTCTATGGTAG

TAATGGTTAGGGCGTGCAACCCGTTTGGACGGCAATCGCGTACATCGAGC

CGTCGTACCACTCGGTTAGCGGGTGACGCTCCACGGTCGATTCGAGCCTC

ACCGGGTTTCCGCCAGTGGCGGATAAGTATCCGGAAGGACCGGAGCGAAC

GGTTATTCCGGAAGACGGCCATTTGTGCGCCGCTACTCCGGGGTAAATTC

GATTTTGGGGTAATTCGTCCAGTACTATTCTCGGTGAGAAATTCGTTGCG

GATGGCGGGCGCTCCGCCATGGCCGGACAACTGTCTCCTTCGAGCGTCAG

ATCATCCGGTAGACTTCGTCCATCCAACTGTCGTCGGTCTCGACGGTGAT

GCCGCTCATGACCTCTGCCCAGTCGGCTTGCGCCTCGCTGTTGTCCATTA

CCTCTTGGATGGCCTCCGGGTCGTCGACTTCCATGAAGCCGAAAACGTGG

CCGTCTTTCTCGAAGACGCTGTACGTTTGCAGGCCGGCGTCGGAGTCCAA

GTAGGCGGATTCGAGCGCCTCGGGGACGTTCTCGTGTTTCGTTCGGTACT

CTTCTCGCTTGCCGTCTTTAATCTCCAGGTGGAACGCGATTCGCGCCATA

CGGCCTCCGATTCGGCGGCCCCCGTCTTAGCTTTTCGGCGGGTAAGATTT

AGTACGGGAACGCCCGTCGTCGATTGTAATGATAGATACCCACACCCACG

CGTGGGGTGCCGCGAGCCCGGAACACCCGTGGACCAACGGCCCCATCTTG

GACCTCGTGGACAGCTTCGACGTACACACCGTCTACACCGCCGAGCGCCT

GCTCGCCGACATGGACCGAAACGGCGTGGACGAGGCGGTCGTCGTGGGCT

ACCCTATCTGCGACTGGACGGACAACTGGTACACCCGACGGGTCGCCGCC

GAGTACGACCGACTCCACGGCATCGTGATGCTCGACCCCTTCGCCGACGA

CGCGGTCGAGCGTCTCGACCGCTGTATGGAGACCGACGGCGTCCTCGGCT

TCCGCCTCGGCGCGGCCTGCCCGTACGATCGCATGTGGGAGACGTTCGAC

CCGAGCGTGACGTGGCTCCGAGAGAGCGTCGAGGAGACCGCGTTCTGGGA

GGCCGCGGTCGACCACGACGCGACCGTCCAGATTCTCTGCGACCACGGCC

AACTCGACCAGGCGCTCGAACTCGTCGAGGCGTATCCCGAACTCACCTAC

CTGTTCGACCACTTCGCCCACGCCGGCCCCGAAACCCCCACCGACGAGGG

GACGTTCGGCCGGTTCGCCGACCTCGCGGAACACGACTCGGTCGCGGTGA

AGGTCTCGGAAATTGTCCACATGTCCGACTCGGCGTTTCCCTACGCCGAC

ATGCACGACCACGTCCGGTGGCTCCTCGACACCTTCGGTCGCGAGCGGGT

CGTCTGGGGCTCCGACTACCCCAACGTCAGCGACGTCGCCAGCTACGCCG

AGGCGTGTAACTGGCTCAAGCAGGTCGACTCGTTATCGAAGGCCGACCGG

TCGTGGCTGACCGAGAAGTCGTTCCGGCGGCACGTCGACCTCGGCTGAGC

GTAACCGGTCGTCACCGCGTGCCTCGCTCGCACTGTCGTCGATCCGGTGG

CTGAACTCTCCATCTCCGAATGACACCGGTATATTTATTATAATGAACTA

TAGTGGTTTGTAATAGCACATCATGACTGATAGTGGTTCACAGAACCGGC

TCAGTAGGCGGCGGTACGTGACCGGACTCGGAGCAGGTCTCACAGCGGCA

CTAGCGGGTTGTATGGGTGGCGGGAACGGCGGTGGCAGTGGTTCCGATGG

CGGTTCCGACGGGGGAGGCTCCGGCGGCGGTGGCGGCGACTCCGGGTCGC

AGTCACTCGACTTCTGGCTGTTCGGCGGCATCCCCGCCGAACGAGAGTAC

ATCAGTAACCACTACAGCAGTTACAGCGACCACGACGTCACCTACCAGCA

CCAAGAGTGGGGGCAGAAGTACCAGATTATCGCGTCCGCGGCGGCGAACA

ACAACCTCCCGGACGTGATGGCCGGCCAGACTCAGCAGATTCCGGATTAC

GTCGGCGCGAGCGCCATCCAACCGCTCGACCAAGAGCAGTTCGCCGACCA

GTTGGAGGAGATAAACAGCCGGTTCATCCAGGCGAACGTCGACACGCAGA

TTTACGACAGCCTCGGCGACACCGAAGGAGAGCGGCAGTGGGGGCTTCCC

GGCGGCTACGCCGACCTCGGTCCGTTCGTGGACATCCGAACCGACTACCT

CGAACAGACGAGTTTCGACAGCCCGCCGCGCAACTGGGAGGAGCTCATCC

AACTCGGCGAGGAGATGCAGGAGCTCGACGAGGTGTCGGCGGCGATTACC

GCGCCGGGGACCGACTTCGGCCTGACGACCGGCTACTTCATCGGCTTCGT

CTACGCCAACGGCGGTCGGTACTTCGACCCCGAGACGCTGGAGGCGACGG

TCGACCAACCCGGCTTCGTCGACGCCGTCAGGCTCTATCAGGACATCGCC

GAGGCCGGTCTGTTCCCCGACTCGATGGCCGAGAACGACCACATCGGTGC

GGGACGGCTCCTCCGCGAGGGCGAGTCCGGCATCTTCATCACGTACTCCC

ACGCGAACGCGGTCTACCAGACGACCGGCGCGCCGCAGGCGTGGCTCGAC

GGCGAGGGCCACACCGTCACCCGCGCGCCGCTTCCGGACAGCCCGTCGGG

GAGCTTCGAACCCCGCGACCTGCTCCTGCAGAACGCGCAGGGCTTCATGC

TCGGCAACGGCACCGACTCGGAGGCCGAACTGCAGGCCGCCTTCGACTTC

ACCGAGTGGTGGAACTCCGAAGAACAGCTCGCGCCGTGGACCTACGACGC

CGAGAACGACGTGGGCATCCGCGGGCGCGTCCCGACGCTCGAAAGCGCCT

TCGAGGACCCGAGCGACCTCTTCCGGAGCCAGTTCGGCGACCTCGTGACG

CTGTACGAGAACGACGACCTGTTCACGCGCACCTCGCGGTTCCCGTCGTT

CTCGGGCATCGCGTCGGTCCAGAGCATCATCAACACCGAGGTCATCCAGC

CGGTCGTCCTCGGCAACGCGACGGCAGAGGAGGCCTGTAGCGCCGCCAAC

GAATCGGTTCAGGAAGTCATCGACGAGGAGCTGGCCTGACGATGCGACTC

GAACCGTCGCTCGAACGACGGCGGCCCAACGGCGGTGAGTTACGACCATG

AGTTACTCGAAGCAGGCGAGGGGCTCGTTCGAGAGCGTCTCCAAGCGGAT

TCCGGAGGGCGTCAAGGTGTACCTCCCGGTCCTGCCGGTGACGGCGCTCG

TCGGGCTGTTCATCCTCTACCCCATCGCGCAGGCGATCTATTCGAGCTTC

TTCAACAAGGACCTGCTCGCGCCCAGCGAGGCCGAGTTTATCGGACTGGC

GAACTACGCCGAAATCTGGTCGAATCCGACTATCCATCAGGTGCTGTGGA

ACACGCTCATCTGGGTCGTCGTCGGCAGTTCCGCCGCTATCGTCCTCGGC

TTCCTGATGGGTTGGCTCCTCCACGAGAAACTGCCGTACACGAGCGTCGC

GAGCGCAATCGTGCTCATCCCGTGGGTCCTCCCGCGGGTCGTCGGCGCGT

CCATCTGGCAGTTCATGTTCGGCGGGTCGCAGGGCATCATCAACGAACTG

CTCGTCCAGCTCGGCGTCATCGACGAGTACATCGTCATGCTCGGCTCGAC

GGAGCTCTCGCTGTGGCCGCCCATCATCGGGATGATTTGGCGGCTCGCAC

CGCTTTTCGCGCTGTTGACGCTCACGTCCCTGCAGGGCATCGACGAGCAT

CTCTACGAGGCCGCGCGCATGGACGGCGCGACGCCGTGGGAGCAGTTCCG

GTTCATCACCATCCCGATGATGCGGTACAACCTCGCCATCGGGTTCCTCC

TGATGCTCATCTACAACATCAGGAACTTCTCGATGATTTGGGTGATGACG

AAGGGCGGCCCCGGCGTCTCCAGTAGCACGCTCCCGGTCATGATCTACCG

GACGGCGTTCGTCGACTTCGACGTGGGGCTGGCGTCGGCGCTGTCGCTCA

TCCTGTTCGTCGTCCTGCTCGTGTTCTCGTACTACTACATTCAGGTGTAC

GACCAAGTGCGGGGTGACGTCTGATGGCGGCGAAAGACCCCGACACGGCC

GCCGAGTTCCGGCGGAACGAGTCGTGGCTGTTCGACACCACGGCGGGCGC

GTACGTCCGCAAGACGGCGGTTTCGCTCCTGACGCTCGTCGTCTTCGCGT

TCGTCCTCTTCCCGCTGTACTGGATGGTCGTGACGGCGTTCAAGACCACC

GCGGAGATTCAGCAGATTCCGCCGACGGTGTTCCCGAACGATTTCTCGTT

GGAGGGCTTCCGGCTCGTCGTCGAGTCGTCGATTCAGGCGGGCGGCTACA

CCTCGCTTTTCGCTAACCTGTTCGGCTGGCAGGCGTCGTCGGCCATCGAC

ATCGACATCCTGTCGCTCGTGCTGAACAGCCTGAAAGTCGCGATCGGCGC

GGGCGCAATCGCGCTCGTCTTCGGGACGGCCGCGTCGTACGTGCTCTCGC

GCCGCGACTTCGCCGGGAAGAACGCGCTCATGAGCATCTTCCTCGCGTCG

CTGATGTTCCCCGGGACCGCCATCATGGTCCCCGAGTGGGAACTCGTCTC

GGCGCTCGGCCTGTTCAACACGCACCTCGTGTTGATCTTCATCTACGGCG

CGATGACCGCGCCGTTCGTCGTCTGGCTGATGAAGGGTTTCTTCGACGAC

TTCCCGGACTCGATTATCGAGGCCTCCCGCATCGACCAGTGTAGCTCGAT

AGAGACGTTCTGGTACGTCATCGTCCCGATGGCGCGGAACTCGCTCATCG

CGTCGTTCATCTTCGCGTTCCTCCTCGCGTGGAACGAGTTGGTGTTCGCG

CTGACGCTCCTCGACAACTCGCGGTACACGGTTCCGCCGGGACTCCTGAC

GTTCGTACAGGGGTTCAACACCCAGTGGAACGTCGTCGCCGCGGCCTCGA

TTATCATCTCGCTTCCCGTCCTGCTCGGACTGGCCTACATCCAGCGCTAC

TTCGTGCAGGGGCTCACCGGCGGCGCGATTAAGGGATAATACCCTTCGCC

CGCCGTCTCTCGGTGCCGCACGTCGCGGTCAATTTTCGTCGTCAGTCGCT

AGGTTTAAGGGGTGTTCTACGGTAATGTTTTACAATGACACACCTAACAG

TAGACGGTGTCACGAAGATTTTCGGCGACCCCGACAAGGACGGTGTCGTC

GCAGTCGACGACCTCGACATCGACGTCGAAGACGGGGAGTTTCTCGTTTT

GCTCGGCCCGAGCGGGTGCGGGAAGACGACGACGCTCCGGATGATCGGCG

GACTGGAGACGCCGACCTCCGGTGAAATTCGCTTCGACGACGTCGTCGTC

AACGACATCAAAGCCCGAAACCGCGACGTGGCGATGGTGTTCCAGGACTT

CGCGCTCTACCCGCATATGACCGTCCGGGAGAACCTCGGGTTCGGCCTCA

AGCGGGAGGACAACGGCATGTCGACGGCGGACGTCAACGAGCGGGTCGAG

GAGGTGGCGACGATGCTCGAAATCGAACAGCTCCTGAACAACAAGCCCGC

ACAGCTCTCGGGCGGACAGAAACAGCGCGTCGCGCTCGGCCGGGCTATCA

TCCGCGAGCCGCGGCTGTTCCTCTTCGACGAGCCGCTTGCGAACCTCGAC

GCGAAGCTCCGCAAGACGATGCGGACCGAAATCGACGAACTGCAGTCCGA

GGTCGGCATCACCTCCGCGTACGTCACCCACAACCAGGAGGAGGCGATGA

CAATCGCCGACAAAATCGCCGTCCTCAACGACGGCGCGCTCCAGCAGTTG

GGTCGCCCGGAGGAGGTGTTCAACGAACCGGCGAACCTCTTCGTCGCGCA

GTTCGTCGGCAGTCCGGACATGAACATCTACGACGGCGTCGTCGAGGAGG

CCGCCGACGGCGTTCGCGTCGAACTCGACGGCGTGAGCTTCGAACTGCCG

AGCGACGCCCTCGACGCGGAGGTCCCGCCCGGCGGCGTCAACGTCGGCTT

CCGGCCGCAGGACTTCTACCAGACGGGCAAGCGGCGCGCCGACGGACTGA

CCTTCGACGTCGATATCCGCGTCATCGAGCCGGTCGGAACGAAAGCCATC

GTTCACGGCGACGGCCCCATGGGCGACGTCACCGCCGAAATCGGCGAGTT

CCACGGGCTATCGGCCGGCGACACGCTGTCGCTTTCTATCGCGCCCGAAC

ACGTCTACCTGTTCGAGGCCGAGACGGAGGCACTGCGCAAGGGCCGGCGT

ATCGAGAACCGGGCGTCCGTCCAATCGCAGTCCCAGTCGTCGGAGTCGGA

CGGCGTCGAAATATAACTCGGCGCGACTGCCCCCATCGAACGTCAGCGGG

CGTGGTGTCTCGCGGCACCCAGAGCCGCGGCTTCAGCCGCGCTACCGCCG

AAATTCTCCGCCCGCACCCAAACATATTAATCGGTGTGTTCACATGATGT

AAAACGAGACGAACCCACACGGTTATTCGCAACTATGAAAGCCATCATTC

AGACCGGGCCGCGCTCGGTAGAGACACAGGAGCGGGAGGTTCCCACTCCC

GGCGCAGACGAACTGCTCATCAAGGTCCACACCGCGGGACTCTGCGGGAG

CGACGCTCACGCCTACAAGTACGACGGCGGCTACGAGTGGATTCCCATCC

CGCGAATCATGGGCCACGAGTACTCCGGGACGGTCACGGAAGTCGGCGCG

AACGTCGAGACGTTCGAGGTCGGCGACAAGGTCGTCGAGGAGCCGATTCA

CGACTGCGGTCACTGCTTCCAGTGTCGGAACGGTCAGCCGAACGTCTGTC

AGAACTTCTCCATTACGGGGATGCACCGCGACGGCGCGTACACGGAGTAC

GTCACCGTCACCCCCGAGCACGTCCACGCGGTTCCCGAGGGCGTTCCCCT

CCGGCACGCCGCCATCACGGAGCCGACGAGCATCGCCACCCGCGCGGTCC

TCGACCAGTCGGTGACGACGCCCGGCGACAACGTCCTCGTCGAGGGGCCG

GGCCCAATCGGCGTCCTCGTCGCCGCCGTCACCGACTCCCTCGGCGCGAA

CGTCGTCGTCTCCGGACTCAGTCAGGACGCGGCGTATCGCCTCCCGCTGC

TCGAAGACCTCGGCATCGACACGGTGAACCTCGAAGAAGAAGGCGGCCTC

GATGGGCGGGTCGAGTCGCAGACCGACGGCATCGGCTTCGACGTGGTCTT

CGACGCGACGGGTCACCACAGCGGCGTGGTCTCCGGGAACGACGTGGTCC

GCAAGGGCGGCCAAATCGTCGTCGTCGGCATCCCGAACTCGGCTAGCGAA

CTCAGCCTCACCTCGACCGTCCGCGGCGAGGTCGACATCAACACCTCCTA

CGGCTCGACGTGGACGAACTTCGAGCAGGCGCTTCGCCTGATGGAGCGCG

GCGAAATCGCGGTCGAGAAGATTCTCGACACCTCTTACAGCGTCGACGAC

CCCGCCGCGGCGTTCGAAGCGTTCCTCGGTTCGGAGACCTGCAAGCCGGT

CTTCCAGTTCGACGGCTGAACAGCCCCGCATTCCGGCCGCGAACCGCTTG

ACGACAGAAACTATTTTACCCGTGCCGATTAGATGTGAGGTCGTACCAAA

CAAATCATGGAGATTACAGACATCTCAGCGACGAAAATCAGCAACGAGTC

GTGGGGCGAGTTCATCGAGTTCCCGCTCGTCACCATCATGTCGAAGTACG

ACGAGTACCGCAACGTCGACGGTGAGAACCCGCAGGCCCGCCGCAAGTGG

ATGGGCCCCGTGGGCGACGTGGTGGTCGAGGTCGAGACGGACGCGGGCAT

CACCGGCGTCGGCGTCGGTAACTGGGGTACCGGAGCCATCGCCACCGTCG

TCGAGGAGACGCTGTCGAAAATCGTGGTCGGGGAAGACCCGATGCAGCGC

GAACTCCTCTGGGAGCAGATGTACCGCGCCACGCTCCCGTTCGGCCGGAA

GGGCGTCGCGGTCATGGCCATCAGCGCGGTCGACCAGGCGCTGTGGGACA

TCGCCGGGAAGGACGCCGGCAAGCCGGTGTACGAACTGCTCGGCGGCCCC

ACCAAAGACGAGATTCCCGCCTACGCGTCGAACCTCCACCCCGTCGACAT

GGAGAAGCTCGAACGCGAGGCGGTCCAGTACGTCGAGGAGGGCTTCGACG

CGATGAAACTCCGCTTCCTCCACGGCCCCGAGGACGGCCGCGCGGGCATG

AAGAAAAACGAGGAAATCGTCGCGACCGTCCGCGACGCCGTCGGCGACGA

CATCGAAATCGCGGGCGACGCCTACATGGGCTGGTCGGTCAAGTACGCAA

AGGAGATGACCCAGCGCCTCGGCAAGTACGACATGGCGTGGGTCGAAGAG

CCTGTCATCGCCGACGACATCTCCGGCTACGCCGAGGTCCGCGAGGCCGC

GCCGATGCCGATTTCGGGCGGCGAACACGAGTTCACCCGCTGGGGCCACG

AGGACCTCCTCGAAGAAGGTGCGGTCGACATCCTCCAGCCCGACGTGGGT

CGCGTCGGCGGTATCACGGAACTCCAGAAGGTCGCCGACATGGCCGAGGT

CCACGACGTGCCCGTCATCCCGCACGCCGGCACGAACCCGACGCTCCACG

CCATCGCCGGTCACACGAACATGCCGATGGCGGAGTACTTCCCGACTCCC

GAGTGGTTCGAGGAGCGCCAGGAGAAACGCGAGTCGACCTACGCCGACGC

CATCTTCCAGAACCCGCCGTCGCCGGAGAACGGGTCGATCCCGCTGCCCG

ACGAGCCCGGCGTCAGCACGCAACTGAACCGCGAGGCGCTCGAACACTTC

GCCATCGAGTAACGATGTCGGACATCAGCTTCGAGTACAACGTCCCCGTC

TTCGCCGGCGCGCCCGACGAGGGGACCGAGCCGACGCACCGCGACACCCC

GCAGTACGAGGCGCTCGACTGGGTGACGACCAAGATGGGCGTCCAGAAGG

CGGAGGAACTCGGCTTCGACGCGGTGTGGGCACCAGACCACCTGCTGTTG

GGCCGCGACCACGCCGAGTACGAATGTTGGACGCTGCTGTCGGCCATCGC

CGGCTTCACCGAGGACATCAACCTCGGCTCGCTCGTCCTCTGTAACGACT

ACCGCAACCCGGCGCTCGTCGCCAAGATGGCGGCCACGCTCGACGTGATT

TCCGACGGGCGGCTGGAACTCGGCCTCGGCGCGGGCTGGCACGAACCGGA

GTACGACGCCTACGGTTGGGAGTACCGCGACGGCTTCGAGCGCCTGATGC

GGCTCGACGAGTCCATTCGTCTCATGAAGCGCATGTGGGCCGCCGGCACC

GACGGCGCGAGCTTCGACGGGAAGCACTACCAAATCGAGGACGCCTACTG

CGTCCCCGGTCCCGTGCAGGACCCCCATCCGCCCATCCTCGTCGGCGGGC

AGGGTGAGGAGGTCACGCTCAAACTCGTCGCCAAGCACGCCGACGTGTGG

AACACCGACGTGTTCAACGGCGACGTGGAGACGCTCGAACACAAAATCGG

CGTCATCGAAGACCACTGCGACACCGTCGGCCGCGACCCCGACGACATCG

AGTACTCGTGGGACGGCCACGTTATCTGCACCCGCGACGAGGAGAAGTTC

GACCGCCTGCTCGACCTGATGACGCCCATCCAGTTCGAAGCGGAGTATCA

GGACCAAGCGCCCATCGTGACCGAGGAAGACGCTCGGGAGTACTTCATCA

TGGGAACGCCCGAGGAGTGCGCGGAGGCCATCGAGCGCCGCATCGACGCC

GGCGTGACGAAGTTCCAAGGCTGGTTCATCGACTTCCCCGACACCGGCGG

CATGGAACTGTTCGCGGACGAAGTCATCCCGCAGTTCAGCTGAGACGCCC

CGCGGGTCGCGCTCGCGTCTCCCTCGGGCACTCGTTTTCTTTTTGCCACC

CTCGCATCATACGTCTACCTGACTACTCGCAATATCTGAATCATCTAAAT

TATTTAGACAATTCAGATATTCCTCACATCGGCTCGTCGCTCGTAACGGC

TACGCTATTTCCGGCCATAGCGGAAAGACATTTCAGGGATGAGGATTCAT

AACCAACTATGGCGAAGGACGTTCCGGTCAACGCGGTCAAAATCTCGCTC

GAACTCGTCGACCTCCTGCGGGAACTCGACGGGGCGGGCGTCTCGGAGGT

AGCCAAGCGACTGGACAAGCCGACCAGCACGATTCACGACCACCTGCGGA

CGCTGGAGCAACAGGAGTACCTCGTCAAGGAGGGCGACACCTACTACGTC

AGCACGCGATTCCTCCAATTGGGCGCGCACGCGCGCTCTCGACAGAAGGT

GTTCAACATCGCCCGCCCCGAGATAAACGAACTCGCGCAGGTGACCGGCG

AGCACGCGAATCTGATGATCGAGGAACACGGTATCGGCGTCTTTCTGTAC

CGCTCGCGGGGTCCCGACGCCGTTCGTCTAGACACCCACGCCGGGATGCG

CGTCCCGTTGCAGACGACGGCGCTCGGGAAGTCTATCATGGCGAACCGGC

CGCGGGAGGAAGTCGAGGGCATCTTGGACCGCCACGGACTGGAGGCGGTG

ACCGACCGCTCTATCACCGACCGCGAGGAACTGTTCGACGTGTTGGAGCA

GGTCCGCGAGCGCGGTTACTCCTACGACGACGAAGAACGGGTCAAGGGCA

TGCGCTGTGTGGCCGCGCCGATACTCAACGAGGACGACTACGCCATCGCC

GCCGTGAGCGTCTCGGGGCCGAAAAGCCGGATGCGCGAGGAGCGCTTTAC

CGAGGAGTTGCCGAACCAGATTCTCAAGAGCGCGAACGTCATCGAGGTGA

ACCTCACGTACTCGTAGTCGGCGGGTCGGCGGCCTCGGGCCGCCCGCCTC

GCTCCCGTCGTCGCAATGCTGCGTAGAGCGCAACAGTTACGTACCACGGT

GTGACAGAGTGTAACGATGCAACTCGTTCGATACACCGCGGGCGGTGCTC

CCGAGTGGGGCGTCCGCCGCGACGACGATGTCGTCCCGCTTTCCGGCCTC

CGCGAAGACGTGACCGTCCAGAACCTCAACAGCCCCGGCTTCCTCACCGT

CGTCGAGGACGCCGCGGACGCCGCCGAGGACCAGTCGGTCCCCGTCGAGG

ACGTAAAGCTCCTCGCGCCCGTGCCGCGCCCCGGTAAAATCGTCTGCGTC

GGCCTCAACTACCACGACCACGCCGAAGAACAGGACGAGGAAGTTCCCGA

ACGCCCGCTCCTGTTCGGGAAGGCCGGCACGTCCGTCACGAACCCCGGCG

ACCCCATCGTCCACCCGGCGGCCATCGACGAGGTAGACTACGAGGTCGAA

CTCGGCGTCGTCATCGGGCAGACGGCCAAGAACGTCGACGCCGAGGACGC

TTTCGACTACGTCGCGGGCTACACCGCCATCAACGACGTGAGCGGCCGCG

ACGCGCAGTTCGACGACGGGCAGTTCTTCCGCGGCAAGAGCTACGACACG

TTCGCCCCGATGGGGCCGACGTTCGTCCCCGAGGGCGACGTCGACCCGCA

CGACCTCGACGTCGCCTGCCGCGTCAACGGCGAGACGAAACAGGAGTCCA

ACACCTCCGAGTTCATCTTCGGCGTCGACGAGGTCGTCGAGTACATCAGC

GGCATCACGACGCTCCGCCCGGGCGACGTCATCTCCACCGGCACGCCCGG

CGGCGTCGGCATCTTCCGCGACCCGCCGGAACTGCTCGAACCGGGCGACA

GCGTCGACGTGGAAATCGAGGGCATCGGCACGCTCACCAACCCGGTCGTC

TCCGAGCGCGACGAGTAACCGCCCGCCGTACAGCACAATTATTTATAGGA

TATCCCGGTACTACCGACTATGAGCACGCACACAGACACCCGACTGAGAG

ACAGGCATATCGTCGTGACCGGCGGCGCGCGCGGCATCGGCCGCGGCATC

GCGGTTCGCTGCGCCCGCGCCGGGGCCGACGTATCGATTTTCGACACGAA

GCCCGAGGTGGCCGCCGAGACGGCCGACCTCGTCCGCGAGGAGGGCGGCG

AGGCCGAGGTGTACGAGGTCGACGTGACGGACGCCGAGGGCGTCGAGACC

GCCGTCGACGCCGCTATCGACTCGCTCGGTGACATCCACGGCCTCGTGAA

CAACGCGGGCGTCCAGCAGGCCGTCCCGCTCCTCGAAACGACCGAAGACG

ACTGGGACCTCCACTTCGAGGTGAACGCCAAGGGGACGTTCCTCGTCTCC

AAGGCCGTCGCCAGCCGGATGGTCGACGGCGACATCGAGGGCAGCATCGT

CAACATCTCGTCGGTCGGCGCGGAGCGGCCGTTCTCCGGACAGGGCGCGT

ACGGCGCGTCCAAGGCGGCCGTCCTCGCCTTCACGACCGTGCTGGCGAAG

GAGCTGAGCGACCACGGCATCACCGTCAACGCGCTCAAACCGGGCACCGT

CGAGACGCCGATGGTCGAGGAATGGCTCGACGAGCACGCCGAGCAGTCGG

GCAAGTCGCCGGACGAAATCCTCGCGGAGACGCTCGACGTGCACATCCTC

GACCGAATCGGCCAACCCGAAGAAGTGGGCCACGTCGCGGTCCTGCTCCT

CTCCGAGGAGGGCGACTGGATAACCGGCGAGTCCATCGCCGTCGACGGCG

GCTACCTCAAACACTAGTGTTCGTCGCGACGACTTCGAAAGCCGCCCGAA

GCGTCCGAAATCGATGCTTTCGACTCCCCGAGCCGGAGCCGGAACCCGAG

CGTTCGGCCACAGCACTCACACGCAGTCCGGTATTATCGGATATTGACTG

TGTAGAACCTGGCGGCCGGGCGAACTGAACCGAGGCGTGCCCGTCGCGGC

GCGCGCGACCGCCGTGAGGATGCGAAATCAAGCGACTCGGAGATGTCCGC

ACCGACTCGCGCCGCCGCAGTTGGTGGCCTGTCGTCGCCGTCCGTGACTC

GTCCAATTCACCTCTGGCACTCAGCGACGAGCTCGTGGCTTTGAGCACCG

GATTTCGCCTCCGTGGCAATAATAGTCATAAACCTAATTCACATATCGGT

GTGTCGTGTAACAGCACGTGGGTGGAGCAATCCGACCCATGTGTAATCAG

CATCCCGTGTATCCAGCACGATAGCCATCAGTACGTCAGTCATACTGTAC

GCACGGTAGCCCCCACCGAGTACGCCTGCCCAACTGATACTCGTGAACAC

GTGGCGCGGCTACCTACCGAGCACAACGTCCCATTGATGCTCTATTTTTC

GGTCGTCTCCCGTCGGCTACGTAGCGTCTGCCTTCCCGACTCCGGCCCCG

CGTTATGCCTAGTCTCCGCGTGAGTCGTCGAACCCCATCTATTCCATCCG

ACATTGCCGGATATTACTGGTTCGATTCCGACGAACGCGCCTCTCCGAGT

GTGACGAATCGCTCTCCAAACCTGAAGATATTTCACGCCACCGTGTGAAA

AGCGAGTAATGGGTACCGACCAAAGCACGAACCGTCGCCTAAGCAAGGTA

GAGCGGGTCATCGAGACGTACGACTTGGACGGATTCGGCGAGCAGCTCGC

CGACCGCTGGACGGCTCCCGAGGACAGCGACAGCCTCCGGGACCTCGCGG

ATCTGATGAACGAGAAGGTGCTCGACGCCGCGCTCCGCAAGGCGGGCGAG

GACGTCCTCGAAGGCGAGGTCGAGAACCTGTACACGCTTCTCACCGGCGA

CGAGACCACGGAGGGGATGCGCGTTCAGGCGAAGAACACGCTCCAGTCCC

GCGGCGTCGACGTCGACCAACTGCTCTCGGATTTCGTCTCTCACCAGGCG

GTCTACACCTACCTGACGGATATTCGCGGCGTCTCGAAGGACTCCTCGTC

CGGAAACAGAATCGACAACGTCATCGAGTCCATCCAGCGACTCCGCGGCC

GACTGGTCGCGGTCATCGAGCAGAGTCTGAGTTCGCTCCGCAACACGAGC

AAACTCCGACTCGGTGACTTCGATGTCCTCGTGGACACGCAAGTGTACTG

CCGCGACTGCGGGACGCAGTACGAGGTGGTCGAACTGCTCCAGCGCGGCG

GGTGCGACTGCGACGAGAGCGAATCGAACTACTGATTACATACTTAGCGC

TGTCACATTATCCTCGACTCAACGGGTGATTTTATATTGGTGTACCTGTT

GGGTCAAGGTATGAGCTCAGAACAAGTGTCAAAATCCAAAGTCCACCTTT

CGGTCGAGAATATCGGTGGCATCGACGACCTGTCAACGTCGTTCGAGCCG

GGTGTGACCGTCTTGGCGGGTCGAAACGCCACGAACCGAACGTCGCTCTT

GCGGTCCATCATGGCCGCCCACGGGAGCGACGATGTGGCGCTCAAAGGCG

ACGCCGACGAGGGCTCCGTCGAACTCACTATCGGCGACGAGGCGTACACT

CGAACGTTCGAGCGGCAGGGCGACACGGTCATCTCCGGCGGGAACCCCGT

CCTCGACGACCCGGACCTCGCGGACCTCTTTGCGTTCCTGCTCGAGACGA

ACGACGCTCGACAGGCGGTCGCCCGCGGCGACGACCTCCGCGACCTCATC

ATGCGTCCGGTGGACGTCGAGTCGATAAACGAGCAGATCGAGTCGCTCCA

GCAGGAGAAACGCGACATCGCCGACGAACTCTCGACGCTCAGCAACCTCT

CGGACCGCCTGCCGCAGCTCGAACGCCGGCGGACGAAGCTCGAAGACCAA

ATCGAGGAGAAGGAAGACGAACTCGCGGAGAAAGAACAGGAAGTCGAAGA

GTACGACGCCAGCGTGAGCGAGCGGCAGGAAGTCGAGAGCGAACTCGAAT

CCAAGCTCGGTGACCTCCGGAGCAAGCGCCGCGACATCAACGACGTGGAC

CGCCGCATCGAGACGGAAAAACAGAGCCTCGACGCGCTCGAATCCGAGGA

AGCGGAGCTTGAGGACGACGCCGAGGACCTCCCGGAGGTCGCCGGCGGCA

AAATCGACGAAATCGAGTCCGAAATCAGCCGGCTCCGGACGCGTCGGCAG

GAGATTCAGTCGACCGTCAACGAGCTTCAGTCCGTGATTCAGTTCAACGA

GGAGATGCTCTCGGGCACGTCCTCGGACGTCGTGGACGCGCTCCGCGACG

CCGACGACGCCGGCGGGAGCGTCACCGACCAACTGCTCGGCGACGACCAG

GTCGTCTGCTGGACGTGCGGTTCCGAGGTCGACAAGTCCGAAATCGAGGG

GACGCTCGAACGCCTCCGGACGCTCCGCGAGGAGAAGCTCGAAGACAACC

GCGACCACCGCGAACAGCTCTCCGAACTCGAAGACGAGAAGTCCACCTAC

GAGCGCCGCCAGCGCGAGCGCGACCGCATCGAGCGCCGCCTCTCCGAAAT

CGAAAGCGAGCGCGAGACCCGCCAAGAGCGCATCGAGGAGCTCAAATCCG

ACCGCTCGGACCTCGAAGCCGAAATCGACTCGCTCGAACAGACCGTCGAC

GAACTCGAAAGCGAGGAACACGGCGACCTGCTGGACCTCCACCGCGAGGC

GAACCAGCTCGAATACGACCTCGGCCGCCTCCGCAGCGACCTCGAAGACG

TCGAAGACGAAATCGCAGACATCGAGCGCAAACTCGACGAGCGCGACGAA

CTCGAAGCGGCCCGCGAGGACATCGAAGACGAACTCGAAGAGCTTCGGAC

GCGCATCAAGCGCCTCGAATCCGAGGCCGTCGAGGAGTTCAACACCCACA

TGGACGACGTGCTCGACGTGCTCGAATACGACAACCTCGAACGCATCTGG

ATCGAGCGCACCGAAAAGCGGGTTCGGGAGGGCCGAAGGAAGGTCACGAA

ATCGGTGTTCGACCTCCACGTCATCCGGACGAACGAAGACGGCGTGAGCT

ACGAGGACTCCGTCGCCCACCTCTCGGAGTCCGAGCGCGAGGTGACCGGC

CTCGTGTTCGCGCTCGCGGGCTACCTCATCCACGACGTCTACGAGACGGT

GCCGTTCATGGTCCTCGACTCGCTCGAAGCCATCGACTCGAACCGCATCT

CGCGGCTCGTGGACTACTTCGCGGACTACGCCGACTACCTCACCGTCGCT

CTCCTCGAAGAGGACGCGCAGGCGGTCGACAGTTCCTATCCGCGCTACTC

GCCGGCGTAACGACGCCGACAGCGCTTCGTTTTTCTCGTTTTTCATCCCC

GCGTCGAGTCGCGTCGCGAACGGCGCATCTCGTTTCGAGTTACACTATTA

GGTGCGTCACGAACTGGGCATTGACGGCGACAGGGCGACGCGCTCAGGAG

AGCGCGATGCTCAGTTCCAACTCCTCTGCGATGCCGAGGAGGACTTCGGT

GAGACGGTCGAACTGCTCGCCTTTCATCCGGTTGGAGGGGCCGGCGACGC

TCAGCGCGCCGAACACGTCGCCGTCCGGCCCCATGACGGGCGCACCGACC

GCGCGAACCCCGCTGATCTGCTCTTCGACGTTCAACGCGTAGCCGCGCTC

TCTGATTTCGGCGAGTTCGTCGAACAGCGCCTCCTCGTCGTGAATCGTCT

GTTCGGTCTCCTTCGGGAGGCCGATTCGGTCGACGATTTCTCGGACCCGT

TCTCGCGGCATGTTGGCGAGGATGGCTTTCCCGCTCGCCGTCGAGTGGAG

ATAGACGCGCTTTCCCACCGTCGACTGCGTCCAGACGCCGTGTTTTCCGG

GTGCGGTGTGGACGTACACGCCCCGCCCGTGTTCCTCGACGGTGAAAATC

GAGCGCCGTTCCGACTCCTCGGAAAGGACGTTGGTGTAGTGTTCGGCCTT

CCCGAATTTGCGTTTCCGCGTTCGGACGAAGTTCCCGAGACCGAGGAATC

GAAGCCCGAGATGGTAGGTATCGCCCTCTTTAACCACGTATTCCTGCGCT

TCGAGAGTCGTCACGTGAGTGTGTGCGGTGCTCGGCGCGATGTCGAGTTC

GCGGGCGATTTCGGTCACGCCCGCCCCGTCGCGCTCCCTGAGTAGTTCGA

CGATTCTGAGGGTTGTGACCGTCGTTTTGAGCGAACGGTCGTTCGCGGTT

TCGGTCATTATCTGTGGGTCGTCACCATTGCACATAGTATTTTCGCCGCT

CCCGAATACCAGTTGTTCGATAACGAATGCGGGCCACGCTGACGCCGAAA

CGAGTTGTTTGTGAACTATGTGCATGCTTTGCGGCTGGATTGGGGTGCGA

CGCCCCGCCTCGAAGACGGCGAGGTCAACGACCGAAAACCGGCAGAGAGC

GGTCCGGAAGCGTCCGAAACGCGGGAGCGACAACGCTCGTCATATTTCGG

CGTCTCCCGGTGTTTGGATTCGTTCCTCGTGAATCGTGCGTTCAGTGGGT

GATTCGAAGCCCGAACGTTCACCGATCCAGAAGTGTTATTATCGTTTCTC

CAGTTAAATCGCACGAGACGCAATGAACCACAACGAGACGTTCGAGACGG

TCGAGTCGCTGGAGGCGGAACTCGTCTCACTCACCGAGTCGCTCTGGGAG

CAGCCGGAGCTCGGCCTTCACGAGACCGAATCCGCCGAGTTGCTCTCGTC

GGTTCTCCGCGACGGGGCGTTCGACGACCTCGACGCCGTCGAACTGCTGA

ACACCGGGTCGGAGTTCATGCGCGAGCACGTCTCCGACGACGCCCGCATC

CACTACACCATCCCCGACGGCGGCGGCGCGCCGAACGTCGTCCCCGCGGA

GGCGACGGCGTGGTTCTACGTCCGCGCGCCGACCCGAGACGAGGTCGACC

GCATCACCGACTGGCTCGACGACGTGGCCGAGGGCGCGGCGCTCATGACC

CAGACGACGGTCTCCCGGCGGTTCCACACGGGCTGTTACGACTACGTGGC

GAACCACGCGCTGTCCGATGCACTGCTGGAGAACATGCGACTCGCCGGCC

CCATCCCGTACACCGACGAGGACCGCGAGTTCGCCGCGGAGCTTCAGGAG

ACGCTCTCGGCGGAGACAATCGAGGCGCGGACGGCGCAGCTCCCCGAGGA

GCGGCGCGAGGCGGCGCGCTCGTCGGTGCTGTATCCGGACGCCCAGCCCT

CCTACGACGTGGGGACCGTCCTCAACGGCTCGACGGACGTCGGCGACGTG

AGTTGGATTACGCCGACCGCGCAGTTCTGGGCGGCGTCGTGGCCGGTGGG

GACGCCCTCGCACACGTGGCAGGCCGTCGCCACAAACGGGAGCTTCGGGT

CGAAGGCCGCGGTGTACGCCGCGAAGGTCCTCGCGGCGACGACGCTCGAC

CTGTTTTCGGACGAGGCGCTGGTGGCGGCGGCCCGCGAGGAGTTCGAAGC

GTCCGTCCCCGGCGACTACGAGTGTGCGTTGCCCGAGGGGACGGAGCCGC

CGTTCCACCTCACGTTGTAGGTGGCGAGCGTCGGGAAAATCAGCTCTCGT

TTCGGTTTGCGGTTTCGCTCGATTTCGTCCGTCTATCCGTCTCTCCGCCT

CTCCGTCGCCGAGGCCCGCGTTACTCTAGTTCCGACCGGAGATGCGTCAG

GATGCGGTCGGTCGTCGACTCGATGACTTCTTCGAGGAACTCCGGGTCGG

ACTGCGTCGGGTCGCCGAGGCCGCCCTCGACGGTCAGGTCGTCGAAGTGG

GCGTACTGGCGGACCTCGTATCGGTTCTTCTTCGTCTGCGGGGTCTTGCG

GTCGCCGCGGACGAGGTCGGGATGGCAGTGCTCGATGACGCTCGTCTCGT

ACTCGCCGGCGTGGCCCCACTCGGTGCCGAACCGCGCCTCTAACTGGTCG

CGGGCGAAGTCGGTCCAATGTGCGTAGTACGAGTCGAGTCCGTGGTCGCG

CTGGAGTCGGTCGAGGGCGAGTTTGTGCGGTTCGGTGTTGCCTCCGTGGA

AGTTGGCGACGAGAAACCGGGTCCCGCCGTGGCGCTGGACTGACCGGCCC

ACGTCGATGATTATCTCCTGGTACGTGTCGGCTCCGAGCGAAATCGTCCC

CGCGTAGTTGATGTGGTGCTCCGAGTAGCCGTACGGGAGCACCGGCAGCA

CGAGGAGATTCAAGTCGTGTTCCGGGGCCGCCGCGGCGAGCTCCGCGGTC

AGGTTCTCCGCTCGGATGGAGTCGACCGAGGTCGGGAGGTGCGTCGAGTG

CTGTTCGGTTGCCCCGCAGGGGAGCACGACGAAGTCCGCCTCGTCGAACG

CGGTTTCGGCTTCCTGCCACGTCATCTCCGCTAGGAGCGACTGTGACTCC

GTTGAGTCCATAGAGTGTCTCACGTAGAGATATACTGAAAACGACGGGTG

CGTCGATACCCGTCTTGCTCGCCGGCTGTACGTCCGACTCCGGGTCCGAA

ACGACGAGCGAGGGCGACGGCGGTGGCGGCGAAACGACCGCGGGCGGCGC

GACCGAGACGGCCGCCGGCGGTTCCGACGCGACGATAAACATCGGCCAGG

CGCTGAACCCCGTCCGATTCGACCCGATAACGCAGCTTTCGAACCCCGAC

GCGCTCGTCGCGAACCGGGTGTTCAGTCAGCTGTACACCTACGGCGAGGG

GACGGACGTGGTCCCGAACCTGGCGACAAGCATGCCGACCATCGAGCGGG

ACAACACCCGATACATCGTCGAAATCGTCGACAACGCGATGTTCCACAAC

GGCGACCCGGTCACCGCCGAGGACGTGGCCTACTCGTTCCGCACGCCCAT

CGAGGAGGAGACGCCCCTCCTGGGCATCTTCAGCGTCATCGACACCGTCG

AGGCAATCGGCGAGACGACCGTCCAGTTCGACCTCTCGAACCCCTACGCG

ATGTTCCAGCACGTCCTCACCCATCAGGTCGTGCCGGAGGCCGTCCGCGA

GGAGGACAAAGAGGCGTTCAGTACCCAACGGCCCATCGGCTCGGGACCGT

TCAAGTTCGTCGAGTGGGAAGAGAGCAACTACGTCACGCTCGAACGCTGG

GACGACTACTGGGGCGACGAACTACCCGACGTCGCGGGCGTCGAGTTCAC

CCCTATCACGGAGTCGACGACGCGCGTCACCAACCTCCGGACGGGCGACG

TGGACGTTGTCGAGACGATTCCGCCCCGACTTTGGGAGACCGTCGAGAGC

ACCGAAGACACGAGCATCTCCGAAATCGAGAGCGTCGGGTACTTCTACGT

CGCGTTCAACTGCAACGAGGGCCCGACCGCCGACAAGCGCGTCCGCGAGG

CCATCGACTACAGCTTCTCGATGGACGACGCGGTCGACCGCTACATCGAC

CCGGCCGGACTCCGCCAGTACGGCCCGCTTCCGGGGCAGATGGTCGAAGA

GTGGGACATGCCGCTCGAAGAGTGGATGGAGATTCCGAACGACAAAGACG

TCGAGCAGGCGACGGCGCTGTTCGAGGAGGCGGGCGTCCCCGCCGACTGG

GACGCGAAGATTATCGTCCCGCCGGACGACAACCGCGAGAACATCGGCCT

CTCCATCGCCAACGGCATCAAGGAGGCCGGCTACGAGGCCCACGTCGAAC

GGCTCGACTGGGGCGCGATGCTCAGCCGCGCGTACACCGGCAACGCCTCG

GACTACAACATCTACGTCCTCGGGTGGGTCCGCTACCCCGAACCCGACGA

CTTCATCTACAACCTGTTCCACGAGGACTCGGAGGGCGTGAACCAGGGCG

TCTACTACAAGAACGACGAGGTGATGGAGCAGATTCAGGGCGCGCGGGAG

TCGACCGACCGCGACGAGCGCCAACAGCTCTACCAGTCGGCCATCTCGAC

GCTCCTGGAGGAGAAGGTCCACCTCCCGGCGTTCAACTACAAGAACTCCT

ACGGCGTGAAGTCCACCGTCAGCGACTTTCAGGTCCACCCGGTCTCGCCG

CAGAACCCGCGGCTCCTCACCGGCTACAACAACGTCTCCGTCGACAGATG

ACGGTACCCTGTCGGTAACAGTTACCAAACGTTATATGCTCACTCCCCAT

CCACTCCACTCATGACGACAACCGGTTACATCGGACGACGATTACTCTAA

ATCGTTCCGGTCTTGCTGTTCGTCGTTACGGTCACGTTCGTCCTCATCAA

CTCGATACCCGGCGACCCGGTTCGCATCTTGGTCGGGCCGAGCGCCGACG

CGGCGGCCATCGAGGCCGCCCGCGCGAGATACGGCCTCGACCAACCGCTG

TACATCAGGTACGTCAGGTACGTCGCGGCCGTCGCCACGGGCGACTTCGG

GACGAGCATCCACTACGGTGGAGTCGCGGTGACGGTGAAAATCTTCGAGC

GGCTTCCGGTAACGCCGCTGCTCCTCGCGTCGAGTTTCACCCTCGCAATC

GGCTCCGCGATTCCGCTGGGCATCTTCGCCGCCAAGAACCGTAACAACGG

CTACGACCACCTCGCCCGTATCACGTCGCTTATCGGCGTCAGCACGCCGA

GCTTCTGGGTCGGCCTGCTTCTCATCATCGTGTTCGCGTACTACGGCGAC

CTCCTGCCGCCGAGCGGACTCGTCATGCCGTGGGCCGACCCCGCGTCGGT

CGAGGGGGCGGCGTCGAGACTGGACGTGCTCGCGACCGCCGCGTCGCATC

TGATTCTCCCCTCTATCACGCTGGGGACGCTTCAGATGGCGGCCATCACG

CGCATCGAGCGGTCGTCGATGCTCGAAGTGCTCAACAAGGACTACGTCCA

GCTCGCTCGCGCGTACGGCGTCTCCGAGCGGACGATTCTCCGCAAACAGG

CGTTCAGGAACGCGCAACTGCCGGTGCTCACGGTCATCGGCATCCAGATG

ACGACCGCCCTCGGCGGCGCGGTGCTGACCGAGACGGTCTTCAACATCAA

CGGGATGGGGAACCTCATCATCACCGCGGTCCGGGCGCAGGACTACCAGC

TCATCATGGGCACGACGATATTCTTCGCCATCGTGTTCGTCCTCGGCGTG

CTCGTCGTGGACGTGCTGTACGCGGTCATCGACCCGCGCATCTCCTACGG

GGGTGGTGCCGGTGAGTGATTCGACGGCCGCCGCCGGCGACTCGGCCCGC

ACGGGCGGCGTCGCGGACGCCGACGAGCGCTCCGCCGTCTCGCGCACCTC

GCAGGTGCTTCGACAGCTCAAGCGCAACTCGGCGTCGCGCGTCGGTCTCT

ACGTTATAGGGCTCGTGACGCTCGTCGCGCTGGTGACGACTATCGACGCC

GACCTGTTCGACTACCAGTTCGCGGCGACGTTCTGGTACCACCCGGAGAA

CGACCCGTCGGGGAGCACCATCTTGCTCCCGCCGGTCGGCATCGAAAACA

GCTTCGGGACGGGGACGTGGGCGCATCCGCTCGGGACGGACCACCGCGGC

CGCGACGTACTCGTCAGGCTGGTGTACGGCACCCGCATCGCCGTTCAGGT

GGGTCTCATCGCGACGGGCATCGGGATGACCATCGGGACGGTCGTCGGCG

CGGTCTCCGGCTACTACGGCGGCTGGGTCGACGACGTGCTCCAGCGGTTC

GTCGAATCGCTGTACGCGATTCCGTTCCTCATCCTCGTCATCGCAATCAT

GTCCGCGTTCGGCCGCGAGCTCGGTATCGCCATCATCGGCGTCTCCATCA

CGACGATTCCGGTGTTCACGCGCCTGATTCGCTCGCAGGTGCTGAGCGTC

CGGGAGATGGAGTACGTCGAGGCCGCGAAGGCCGCCGGCGTGCGCGACCG

GCACATCATCTTCCGCCACATCATCCCCAACAGCTTCGCGCCGGTGCTCG

TCCAGTCGACGCTTCAGGTCGGCGTGAACATCCTCATCGTCGCGTCGCTG

TCGTTCCTCGGCTTCGGGGTCCAGCCCCCGACGCCGTCGTGGGGGCAGAT

GCTCGCCCACTCGCGGCAGTACATGATCGTCAACTCGTGGTCCAGTCTCT

GGCCCGGCCTCGAGGTTCTCGCGACGGTCGTCGGATTCAATCTGCTGGGC

GACGGCCTGCGCGACGCAATCGACCCGCGGTCGAACGACTGACGATGAAC

GGACGACACCTCTCACCGACCGTTCGCCCCGGGGCCGGGAGTCGCCCGGC

CGCCGTCAGTCGCTCGCCCGCCGAACTATCGCTCCCTCCCCGGGGCGGCG

TCGGCATTCGCTCTTGCGGTTTTGCTGAGGCACCCATCTCACGCACCGAC

GCCCCCCACGATTCCACGACCAATGTCTGATACGCTACTCACCGTCCGCG

ACCTGCACACCCACTTCCGCACCGACGCGGGGACCGTTCGGGCCGTCGAC

GGTATCTCGTTCGAGGTACAGCGCGGCGAAGTGCTCGGCATCGTCGGCGA

AAGCGGCGCGGGAAAGAGCGTCGCGGCGCGGAGCATCATGCGCCTCATCG

ACTCGCCGGGCGTCATCGTCAACGGCGAGGTCCGCTTCGAAGACGACCTG

CTCGTCGGCGTCGAGCGCGGCGACGACCCCGACGGCGAGCCCCGACAGGA

CCCCGAGATGCTGTCGAGCCGCGAGATACGCGAGCGCATCCGGGGCCGCG

AAATCGCGATGATATTTCAGGACCCCACGGAGAGCCTCAACCCGGTGTTC

ACCGTCAGCGAGCAGTTGAGCGAGATTATCGGGCTCAATCGCGACGTGTC

GGCGGCCGAAGCCGAAGCCATCGCCGTCGAGCGCCTCCGCGAGGTCGGCA

TCCCCGAGGCCGAACGCCGGATGGACGACTACCCCCACGAGTTCTCCGGG

GGGATGCGCCAGCGCGTCCTCATCGCCATGGCGTTCGCGTGCGAACCGAG

CCTCGTCATCGCCGACGAGCCGACCACCGCGCTCGACGTGACCGTCGAGG

CGCAGATTCTCGACCTCGTGAAGGACCTCGCGGCGCGCTACGACACGTCG

TTCGTCTGGGTCACCCACGACATGGGCGTCGTCGCCGAGATATGCGACCG

CGTGGCCGTGATGTACCTCGGCGAACTGGTCGAACTCGCCGACAAGGACG

ACCTGTTCGAGTCCCCGAAACACCCCTACACCCGGGCGCTCCTCTCGTCG

ATTCCGGTGCCCGACCCGCGGGTGTCCCGCGAGACCGTTCCGCTGTCCGG

CGACGTGCCGTCGCCGATTCACCCGCCGAGCGGCTGCCGGTTCCACACCC

GATGTCCGAGCCTCGTCGCCCCGGCGGAACTCGACCTCAGTGACGACGAG

TGGGACCGCGTGCGCCGGTTCGTCAGAGCCGTCCAGCGCGAGTCCCCGGA

CGCGGCCGCTGACGACGCGCGGGCGACCTACTTCCCCGACGACCTCCCCG

CCGGAGAAGCCGTCGAGCGCGCGCTCTCGCTCGCAGCCGAGGGTGCGTGG

CGCGACGCCGCGTCGCTCCTGACCGAGACGTTCGTCGAGCCGAGCGTCTG

CACCTCCGAGGTGCCGGCGCTGGAGTCCCCCGACGAACTCGACCGTATCG

TCAGCTGCCACCACTACTCCTGAAAAAGCGTCGCCGTCCTTATTTCGCCC

TCACGCCGACCGGAATCGGTCTGCGACTTCGGCCACGGTTTCGAAGACGA

CGCCCGGCGTGTCGGCGACGTGTTCGACGAACGATTCGAGCCGGACGAGC

CGGTGTGACTGCCCGATGACCTGCGGGTGCATCGTGAGGACGAAGACGCC

GTCATCGACGTTGTCGACCATCCAGTCGAACTGCTCGCGCCACTGGCGGA

ACACCGATTTCTCGTTCGCGTAGCCCCGCTTGCGGTTGAAGGCGAACGCC

GGGAAGTCGTCGCGTTGCCACGAGACGGGTACCTCGACGATGTCGGTCTC

GGTGCCGCGCTCGAAGGGGGCGTCGGCGGGCGCGGCCCACCCCTCCCGAA

CGCGGTACGGCTCGAAGTCGGTCGCCATCTGGCTGGAGTCCCACTCGAAG

CCGAGTTCGTCGAGGATGCCGAGCGTGTGGGTCGAGAAGTCCCACGATGG

CGAGCGGTAGCCGGTCGGCCGCCGCTCCGTGAGGTCCACGATGCTCTCGA

CGCCGAACTCGTCCAAGCGGTCGAGGACGCGGGGTGCGCCCACGTCGACG

CCGAACAGTCCTCTCGACAGTTTCGTCGGGCTGTCCGGCGGTTTCCCTGC

CGCACCCGAACGCTGGCGGCCTCTCGTCTCTCGTCCCGCGAGCTACCGAA

ACGCCGGCGCGACCTCTTCGGCGAACAACTCGGCGCTCGTCGTCTCGGGG

AAGTCGACGAACTCCACGACGACCTCGTCGAAGCCCATCTCGACGAACGT

CGCCAGTCGCTCGCGCACCTGTTCGGGCGTGCCGACGAGCGGGAACTCGC

CCTCGGCGACGTTCTCGGGCTTGAACCGCGGGAAGTGCTCGTCGAGGAGG

GCGTCGAGTTCCGCCTCGTCCTCGCGTAGCAGACACCGCGCGAACCACGA

GCGCTCTATCTCCTCGAACGGTCGGCCGTAGCCCTCGCAGTGGCGTTCCA

GCACGTCCAGTTTGTGTTGCATCACCTCCGGCGGTCCCCAGAAGTTCCAC

GAGTCGGCGTACTGAGCGGTGATTCGGAGCGTGAACGACTCGCCGCCGCC

GCCGACCATGATAGGCGGGTGCGGGTCTCGGACGGGGTGGGGCCGGCACA

TCGCCTCGTCGAGGGTGTAGTAGTCGCCGTCGAAGCTGACCGTCTCCTCG

GTCCACAGGCGCTTGAGCAACCGAATCGACTCCTCCAGCATCCGAAGGCG

AGTCGGCGCGTCCGGCCAGTCGTAGCCGTAGGCCCGCGCCTCGCTTTCCT

TCCAGCCGGCGCCCATGCCGAGTTTCAGTCGGCCGTCGCTCACGGCGTCG

AGCGTCGCGGCCTGCTTGGCGAGGAGCGCGGGGTTTCGGAAGTGGTTGTT

CACCGTCTTCGGGTAGAGGTACACGTCCTCCGTCTCGCGGGCGATGGCGG

CCAGCGTGACGAAACACTCCGTCGTGGGGCCGCTGCCGGTCATGACGTGG

TCGGGGACGGCGACGCCGTCGAAGCCGAGGCCCTCCATCGCCACGGCGAA

CTCCACCTGCGCGTCCCACGAAATCTCGTCGCAGAACGCCAACTGGGAGT

GAACGGAGTCGCCGGCGCTGGTCGGAACGTTGACTGCGAACTGCATACGA

ACGGCTCACGCGCGACCACGAAAACAGTTACCACTTCTCTCGCTTTCTCA

CTCTCACTACCTCACTCAGTTTCTCATTTCCCCACTGGTCTCCTCACTCG

CCGCCACCCCAACACGTACTACAATTCGAGCCGTTCGGTATTACATGACG

GACCACCAGCACGCGGCAATCGTGACCGGCGCGTCCAGCGGTATCGGACG

GGCGGTCGTCGAGCGACTCCAAGCGGACTATGACTACGTCGCCTGCTTCG

ACATCGAGACCGGCGAGACCGACTGGGACGACGAAGGCGTCCACGAGTTC

GCGGTCGACGTACGCGACCCCGACGCGGTCGCGGACGCGGTGGCGGCGGT

CGAGTCGGTCGCTGACGTGGCGGCGCTCGTGAACAACGCGGGCATCTCGC

GGGCCGTCTCCCTCGCGGACCTCGACCCAGACGAGTGGGACCGCGTCCTC

GACGTGAACCTCAAAGGGCAGTACGTCGTCGCCCGCGCCGTCGCCCCGGG

TATGGTCGAGCGCGGCGAGGGTGCCATCGTCAACGTGTCGAGCGTCGCCG

ACCTCCCGCTCGAACGCATCGGAACGCCCGAGGAAGTCGCTGACGTGGTC

GCGTTCCTCTGCGACGGCGCGAGCTACGTGACGGGGACGGTGCTGACGGT

CGACGGCGGGTCGATGCTCCGGTGACCGAGGCCGCCGGTCGCCGGACGCC

GAGCGAACAGCCAGTACCCGCGAACTGACCGAGACATACTAAATCCAAAG

ATAATATTGTAATTTGATAGTTCGACGCCTAATCTCTCGTATTCTCCGAC

CAGCACTGTCTCGGCGCTGCTCACTCGGCCGTCTCCGGACCAGCCAGCGC

GTCTATGACGAAAACGGCTAGTACAGCGCCTTCAACCGACTTGTAGCGCC

GTCACAACTCGATGACGAACTCGGCACCGAGCTGCGTCACCGAATTGGCG

CGCGTCGGCGCGCGTCGGCGTGTCTCTGTGCGTGCCGGTGCGTTCCGGTA

CGTGCCGGTGCGACTGACCGCTTGAAGTTTGACCGTTGTCTCCTGAACCG

CCTCCGTTCGCGGCCGCGTGGTCGGTGTCACTACCGGTCGAGTCGTCGTT

CTCGATTTCGTATTTTCATCTGATATTTTAGTGAACAGAGAAAATATCGA

CAAAACAATATTTTTAGCATCCGCGTTCGTCCCGCACCGTCGAGTCTTTC

AAAACATATTACGTCACCGCCGGTGTACCGGACGCTCGGGCACAACCATG

GACTATGTCAACGAAGGCGACACGGTCGTCGTCAGGCTGGACCCCGGCGA

ACAGGTCCTCGACTCGCTGGCCGCGGTGCGCGACGAACTCGACATCGAAC

ACGGCTTTCTCACGGGTATCGGCGCGGTCGACGCCGTCACGCTCGGCCAC

TACGACGTGGACGACCAGGAGTACCTCGAAGAGGAGTTCACGGGCCAGTT

CGAGGTGACGAGCTTCCTCGGCAACATCGGCCCCGACAAGATTCACACGC

ACATTCAGGTCGGCACTCGCGACTTCGAGACGCTCGGCGGCCACTGTTCC

GGCGCGCGCGTCTCCGGGACGTTCGAGGTGGTCATCCACCTCGGCGAGAC

GCCGCTGACCCACCACCTCGACGAGCGGACCGGCCTCGACGTGTTCGACA

TCTGAGCCGGCCCGGACCGAGCCGAGCCGGACTGAATCGAACTGAATCGG

ACCGAGCGCGTCGCGGTGCGGACGCTCGGTCGCTTTTTCCCACGCCTGCC

GTGAGTCGAGAGCGTCGCGTCCGTCGGCTGGTGTGAGGCAGTTCGGAGAA

AGAAAAGACGGCGCTGTCGGGGCGTCGGCCGGTGGTGGAGGGCCGAAGCG

AGGGGGGCCGGAGGGCGAGGGCGTGTGTCGGCGGACTGGCCGACGCCCCG

AGCGCACCGAACGAGGGTTCCGGGGGTTGTCCGCCGACGCGGGTTCCGCC

GCGGAGACGCGACCGGGGACCGCTGGTCGCGCCGCCGTCGGCGGGTCGCA

ACCGCCGGTGCGTTGGAGGGCGCGGCCGGCGGCAACTATTCGTAGTCGAC

ATCCCACATTGGCGGTTTCTCGGTTACTACCGTTACTTACGTGAGTAGCG

TGAATAACGTGTATTCTCCGGAAGTTCGGGGTTACGGGCGTTCTCTCGTT

CGCCTCCGGACGAATCACCCAATCGAATGACTTTCGACGCGGGGGGTCGT

GGCCGAGAGTGACAGATGAAACGACACCTCCGCGTTCACATCGTCCCCCT

GTGGCGCGAACACGACCGCATCGTCGGCCCCATCGAGGACGACCGACCGG

ACAAGGTCTACCTCCTCGAACACGAGGACTCCGCGGTCGAGCGCCCGACC

TACCACGAGGCGGTCGTCGACCGCATCGCCGACGTAGTCGGTTCTCCGCC

CGACGTCGAATACTTGGACCTCTTCGATATGTACGAGGTGATGGGCGCGG

TTACGACCATCGCCGACTGGCACCCCGACGACTTCGTCCGCGTCAACGTC

ACCGCCGGCACCAAGCGCGCGGCCGTCGGCGCGACGATGGCCTGCATGGA

CGAACACACCGACGCCGAACCGTACGTCGTCGACCCCGAGGTCCGGCCGC

ACGGCCTCGACGCGCCGGTCACGGAGGGCTTCGCGCAGGCGTCGCTCCTG

ACGACCTACCAAATCGACTCGCCGTCTCCCGACCAGGTCGCCGCGCTCGC

CATCATCGAGGCCCACGACACCGACGCCAAGCACGCGAAAAAGAAGACGC

TCATCACCGAGGCGGCCCGCTACGGCCTCGAGTTCATGCGCGGCCGCGTC

GACGGCGAGGCCGACGACTACGACCCCGTCACCGGCGACTACAACGTCCT

CGACAACCGCGTCACCGCGACGCTCGAACATCAGGGCTACGTCACGGTCA

CCCAGCGCGGTACTCGCCGCTATCTCGAACTCACGGAGGAGGGGCGACAG

ACGCTCCGCGCGTTCCGCCACCGGGCCGAATCGGTCGTCTCGGACCTCGA

AGCGCGGACCGACGACCCGTCCGAGAGCGTCGATTTCGCCCTCGACAACC

CGGTCGACGCGCTCGTGGAGAATCGGTAGGGCGAGTTGCCCTCGCGGTTA

CTGCCTCCGCCGCTCCCCGGCCGCCCCGCCGCTTCGAGGGGAACTGTTAG

TACGGTCGCGACACGAGCCAGATATGTATGCCCTCCAACATGCACACGCG

CAGAGCCGTTCTCGCGGCCGGCGGGGCGACCGCTCTGGCGTCGCTCTCTG

GCTGTGTCGGCCGCGCGCTCGGTGCCCTCGACCTTGAGCGAGACTATCGG

CGCGACGCGCCCGTCGGCGACGTGACGGGCGCGTGGCCGACCGCCCAGCA

CGACTTCGCCAACACCGGTTACACGACCGACTCCGGCCCCTCGGCTGACG

CGAGCGTCGAACGAATCGCGTCGGGCGACGCCGCGCTCGCCACGTCAGTC

GCCCTCGCCGACGGGCGCGGCGTGCTCGGCCACAGCGACGGCGACGGCGA

GGACGGCGTCTATCGCGCGCTCGACCTCGATTCCGAAACCGCCCCCGCCG

AGTCCGACGACGCGTGGACTATCGATTACGCGCACGGCAAGTCCACGCCC

ACGCTCGCCGGCGACGCGATGTTCGTCTCGACCGCCGAGTTCGTCGCGGC

TTACGACGCCCGGACCGGCGAGCGGTGCTGGCGGACGACCGAGGGCGGCT

ACGGGACCCCCGAAAACGCGCCGGTGCTCGCCGCGGAGACGCTCGTCGAC

GGCGGGAGCGCGCCCGTCTTCGGGCGAGACCCCGAGACTGGGGCGGAGCA

ATGGTGCTACGACGCCGGCGAGGCGTATCCCGGACTCGTCGCCCGCGACG

GCGTCGTCTACACGCCAATCGGCGCTGACCACGAACAGACCGGCGTCGCC

GCGCTCGATGCCGCCACCGGCGAGGAGCGATGGCGGCGCGAGGACCTCCC

GCAGAGCGGCGTCCCGCTCGCGGTGGGCGGCTCTCACCTGTACTACAACG

CCCACCGCGGGAACGTGTTCGCTCTCGCCCTCGAAGACGGGTCGACGCAG

TGGCAGGCGTCGATTCCGCTCCCGGAGAACGGCAGTCCGCGGACCGCCGT

CGCGGGCGACACGGTCCACGCCCAGACCTCGCGGGGGAGCCTCGCCGCGT

TCGACGCCGCCGACGGCGCGACGAAGTGGACGCTGTCGCTCGATGCGGAT

ACGTTCGCCCGACCGCCGGTCGTCGCCGGCGACACCCGCTTCGTGGGGAG

CGACGACCGTCTCTACGCGGTTTCGGCGGCGTCCGGTGAGAACCTGTGGT

CGACGGCGCTCGACGCCCGACCGTCGGGCGGCCTGTCGGTCCGCGGGTCT

GAACTCTACTTCGCCGGGATGGGTCGGAACCCCGGCGTGTTCCGCGTGGC

GGACTGACGCCCACTCGTCGCCGCCGATTGCGTTTCGCGCCGACCGACGA

TGCCACCTCCCGTCAGTCGAACGTGTACGTCCGGTATCGTCGAATCGCGT

AGCGGTACGAGCCGTAGGAGACGCCGACGGTCACCAGAAGGTACAGCCCG

ACTCCGGCGGCGAGAAGCGGCGTCGGAGCGAGGTGCCCGGTGACGCCGAA

CCACGTCGCGACGAGGCCGATGACGGTTCCGGGGCCGACGACGAACATGA

ACGCCATCAGCACGAGCGTCGAGGGGACGACCGTGGTCGCACCCCAGAAC

TCGCGTTCCTCGTATATCGGGTAGGCCGCGCCGATGCCGACCGAGAAGGT

CGCGGCGGCGAGGCACATGCACACGCCGACGAGCGCGAAGCCGACCGCGG

ACAGCGGCGACGTACCGAGGGCGACGGTCACCAGCGGGAGCGCCGCCGCG

ACCGGAGCGCCGAGAAGCACACCGGCGAGGACGCGTCCGCGGACCAGCGT

TCGCGGCGCGGTGGCGGTCAACAGCAACATCGGGAACTGCGGGCGGTCGT

CGCCGACGGGGTTGAGGCCGAACGTCGCGCCCGCGAGATAGGTTCCGAGG

CCGGCCCCGCTCGCGGCGAGTATCGGTCCGAGGCCGTCGGCCGAGGACTG

GACGATGGTCGTCCCGAGCGGGCCGAGGAAGAACACCAGCATGACGAGGT

GCGTGAACTGCTGGGGGTGGCGGACGCCGCGGACGAGCAGGCCCCACGCG

ACGTTGCCGCCGTTCGTCCCAGCGAGCGGCTTCGGCGTCGAGAAGCCGCC

GGCAGACTCGTCAGCCTTCGCGCTCGCCGCCGAGCCTCGGTCGCCCCCGG

GCGCGCGGTCGGTGAACCAGAGCGCGCTCGCCTGCCGGGTGGCGACGACG

AGGCCGAGAGGCGTGAGCGCGGCGATGGCTGCGAAGGTGGCGACGGCGGC

CGCCGAGAACGGTCGGGCGAGCGGGGTGCCGACGAATGCGAGCGCGAGGT

AGTCGGTGAGCGGACCGAACGTCAGCGTCGCCGCCAGCGTGTCAACCGGA

AGCGACCCATCGCCCAGCAACCGGCCGAACGCCTGCGAGACGAGGACGAA

CGCGGCCATCGCGACGAAGCCGACGACCTTGAGAACGCGCCGGACGCCGG

GCAGACGCTTGAAGCCGCGGAGCACGGCAACGCCGGCGGCGTAGCCCCAG

ACGGTCGTACAGCACGCGAGCGGGACGACGACGACAGTCGTCGTGAGCAG

CAGCGACGGCGCGCCGAGCCCGGCCGTGAACGTCGCGGCGACGACCGCAA

TCGGGAGTCCGAACCAGAGGGCGATTCGGGCGATTTCGGCCGTGATGAGT

CCGATGACGACGGCCCGCGGGTGGACCGTGGTCAGCACGAGCGACTCGTG

TTCGACGCGGCCGATTCGTTCGAGCGTTCGGAACGTCGCGAGGAGCGTGA

GCACCACGGGCACCGCGGTGGCGACGAGGCCGAAGAGGGGAATCTCGGTC

ACGGACTGCGCGCTCCGACCGGCGACGTAGACGGCCGGGAGCGAAAACAG

GAGGCTGCCCCCGAAGAACAACAGGAGCAACAAGAGGCCGACGACTCGGC

GCGTCTCGGAGGTGTACCCCCGGATACTCCGAACGAACTCGGCGCGCCCG

ATTCGGAGGCCGCGGCGAACGTCTCGTCGGAGGCTCATTCGTCGGTGGCA

CCCGACAGCTGCGGTTCGTCGCTCGTGACCGCGAGGAACGCGTCTTCGAG

CGAGCCTTCGCCGCCCGTCTGGGCGCGCGATTTCACCTCGTCGGGCGTCC

CCTCGGCGACGACGCGGCCGTCGAACAGGACGCCGACCTCGTCGGCGACG

GCCTCGACCACGGGCAGGATGTGCGTCGAGAGGAAGACGGTCGCCCCCGA

GTCGGCCACGTCGCCGATGGAGGTCCGAATCTGCCGCGCTGCGCGGGGGT

CGAGTCCCGAGGTCGGCTCGTCGAGAAACAGCACGTCCGGGTCGTGGAGC

ACGCTCTGGATGAACGCGGTCTTCTGTTTCATCCCCTTGGAGTACGTGCC

GATGCGCTTGTTCGCGTCGCCGACGAGGTCGAACTGGTCGAGGAGGTCGT

CGATGCGCGCCTCGGCGGTCTCCGACGAGATGTCGCGGAGGTCAGCGACG

TAGTCGAGCTGCTCGCGGGCGCTGAACTCGTCGTACAGCGGCGGCGTCTC

CGGGAGGTAGCCGACGTGCGGCGCGAGTTTCCCGCGGTCGCTCGCGTCGA

GGCCGCAGATGCGGACCGACCCCGAGGTGGGCTGTGACAGCCCCGTCAGG

ATGCGCATCGTCGTCGTCTTCCCCGCGCCGTTCGGCCCGAGAAAGCCGTA

CACCGTTCCGCTCGGAATCGACAGCGAGACGCCGTCGAGCGCGACCTCGG

AGCCGTAGGCCTTCCTGAGGTCGGTCGCTTCGACCGCGGCGTCCGCGTTC

GTGGAGGGCATGGGACGAGCCTCGATTCACTCTCCGGACGCGTAATGGTT

GCGGCTTGACACACGGGCAACAGAAACGCGTCGATTCGCCCGCCGTCTCA

GGGAGTCGTCGTCTCGGGGTTCGTCGGGCGAATCGACCGGTAGAACGCGG

TCGAGTACGCGGCGAAGAAGCCGCCGATGAGGCCGCTGACGAGCACGAGT

CCGACCGCGACGGCGACCATGCCGACGACGCCGATTGCCGGGAGGCTCGA

AAACGCCGACGCGGCGGGCGCACCGCTTCCCGGGCCGGCCACCGCGGTCC

CCTGATGCCGGAGCGACGGGGTGAGGAGGAGCGAGGCCACGCCGCCGAAC

ACGCCGGCGACGAGGCCGCCGAGGCCGACGATGACCGAGTAGCCGAAGAC

CGATACGAGGTTGTTTCTGACGCAGGCGACGCTCCGCTTGACGCCGTCGA

CCGCGCCGAGGTCGTCGATGACGATGGCGTGGCCGAAGAACTGCACGACG

AACGCCACGGCGAGGTAGGTGAGCACGACGGCGAGGACGACGATGGCCAG

AATCGCGAGCCCGACCGTGCCCAACTCGATTCCGCTCCCGAGGACGGCGA

GGCCGACGAACACCGCGGCGAAGCCGCCGACGAACGACAACACGAGGTTG

ACGACGAGCAGGCTGAAGTAGACGACGAGCAGCGAGACGTAGTGCGACTT

CCCCTCGCTGACGAGCGTGCCGAGCGAGGTGCGACCGTCGATAGCTTCGT

TGGCCATGCCGAGGACCCCGCCGAGGAAAAACGGGATGACGAATACCGTG

ACCCCGCTGAAGACCAGCGAGACGATGCCGCCGACGAGCGGGCCGACCGC

CTGTGCGAACAGCGACGGGAGTTGGACGAGGCTGAACGCCATGGCGACGA

CGAACAGTATCGGGTTCCGTCTGAGAGAGCCGGCGGCTGTGCCGATGGAT

TGGAGGGCTGCCATACCGCCCACGTCGCGGTACCGCACAAAGAATGTTGG

GTTGACAGGTCGTCCCTCCCGTCCCCGGTCTCCGACCCACAACTCGGTCG

TTGACGAACGGGAGGCGGCCCGTGTTGCCGAGACCGCGATTACGCGTGTA

CCCGTGTAATCGGCGCTTTCACGGGAGACCGGCTCGGTCACGAAGGAACG

CGTCGACCGCGGCCGCGACCCGCTCGGGGTCGTCGCCGGGGCCGCCGTGG

CCGAGCGCGTCGAACTCGACGAACCGACTGGTCGGGAGGGCGTCGTGGAC

GTTTCGGGCGCTCTCGCGGAGGAAGTCGGGACCGTCGGTGCCGGCCAAGA

CCACGGCCGGCGCGTCCACGTCCAAGCGGGCCGGAAGCCGGTACTGCTCG

ACCGCGCGGTTCATCCGGACGACCTCCTCGGCGAGGGCGACGCAGTCGGG

CCAGACGGGCCATTCGTCGAGCCACGCGTCGAGGTCGTCGATGCCGTCGG

GGTGGAGCACCTGCTCGACGTAGCGTTTGACCGCCTCGCGCCGCTGTCCC

GCCTCCACGAGCGCGGCCATGCGCTCCGAGAGGTTCGCCTCGGTTCGGTT

CGCTTCCGGGAGTACCGCCGGTTCGTAGGCGGCGACGGCGGCCACCCGCG

CGTCGGTCGCCGCTTCGAGGGCGGTGAGCGCGCCGTAGGAGTGACCGAAC

AGCACCGGGTCGCGCCCGGTCGCCGCGCGAATCCCGTCGACGAGTTCGCG

GACGCACTCGACCTCGCGCTCCAGCACCGCCTCGGCGGGCGTCGTCTCGG

CGTCGTCCAGACAGGTGCCGAACCCGGGTCGCTCCGGGACGACGACCTCG

TAGTCCCCGAGGTGCGGGACGACCGGGTCCCAGAACGCCGGCGGTGCCAT

CCCGCCGTGACAGCACAGAAGCGGCGTGTCGTCTCCGAATCGTTCGTCGG

ACGGTTCGGCTCCGGGTTCGAATCGAATCGGTTGCATCGCACCCTCGCTT

GTCGCCGGCGTCGGCTAAGTCGCACTCTCAGCCGTGCGGCCGTTTACATG

GGGGCCGAAGACGGAGACCGCCTCGGCCGCTTCGACACGCCCATGACCGA

CCGCCGAGAGAGCGGACGTATGGGGCTCATCGCCGAGTTCGACATCGACT

GCGACGCGCTCCCGCTGACCGGGGTCGCGGCGGCGGTTCCGGAGGCGACG

CTCGCGCTGGAACTCCAGTACAACCACGGCGAGCGGCCGATGTTCATCGT

GACGGCCACCGGTGGAGAGACGCGGGCGCTCGAAGGCGCCATGGACGACG

CCTTCGACGTGGCCGACTGGACGCGAATCGGTGAGTCGGGCGAGACGCGC

CGGTATCAGGCTGCCCCGGCGCGGAGCTTCGAAGAACAGCTCGGAGACAG

CCTCGACGACCTCGCCGGACTGGAGGCCCTCGCCACCGCCGACGCCATCA

TCGAGCGCATCGAGGTCCGGCCGGCGGGCTGGCGGCAGACCGGCTGGTTC

GCAGACCGCGACGAATTCGGGCGATTCGCGTCGTTCTGGCGGCGGAGCGC

CGGTTTCGAACTCCGACGACTCGCCCGCGACGGCGACCCCGAACCGCCGG

GCGAGGGACTCACCGACCGACAGAACGAGGCGCTCCGGACGGCGTACGAA

CTGGGCTACTTCGACATCCCCCGCGGGGCCACCCTGCGGGAGGTCGCCGA

CGAACTCGGCATCACCGCGTCCTCGGCCTCCGAGCGACTCCGGCGGGCGC

AGACCCGGCTCATCGCCGAGACGGTGGCGACGACGTGGCCGCCGCTTCCG

AACTGACGAACCGCGGAACGGACCGCCCGGCTACCCGCCCGCCCACCCAA

CCGGCCGAGTCGCGTCCGGCGACGAACGCCCTAACTCAGCCGAACGACGG

CGCTCCGGCCGGCGAAGTAGACGCCGCCCCCGGCGACGCTCGGTCCGATG

TCGAAGCGTTGGCGCTCGTCTCCGGCCAATTCCTGTCGCGTATTCCCGGT

GGCGGCGTCGAGGACGGTCATCGCGCCGCCGCTGTCATCCCGCGTCCGGA

CGAACACCGTTCCCGCGCCGACGGCGATACCGCTCGCGTAGCCCCCGGCG

TAGTTCCGGTTGTCCCAACAGCGGGTCCCGTCAGCCAGCGCGAGCGTGAC

CAGTCGGTCGTCGGCCGCGACGTACACCCGACTGCCGTCGGTCGCGATGG

TCTCGGGCTGCCTCGGTCGGCGTTCGCCCCCTGCCCGCCGCGGTTCGAGC

CGGTAGCGCCAGCGCTCCTCGCCGGTCTCGGCGTCGAATCCGAGCACCGT

TCCCGCCCCGGTCCCGACGAGGACGGTCCCCTCGACGACCGAGACGTTAC

AGTCGATTCGGGCGCTCGTCTTCGCGCGCCAGCGCTCCGTCCCGTCGGCC

GTATCCAGCGCCCGGACGTAGGTGTCGTCGCCGGCGGCGTAGACCGTGTC

GCCGTCGACCGCGGGCGTCGAGTCGGAGGGGAACCCGGTCGCGGCCCGCC

AGCGAGTCTCGCCGGTCGAGAGTGCCAGCGCGTGCGTCGCCACGCCCGAC

TGGACGTAGACGGTGTCGCCCGCGACGATAGGGCTCGCGCCGAAGATGCC

GCCGTCGAGGTCTTCTCGCTTCCAGCGCCGGTCGCCCGTCGCCGCGTCGA

ACGCGTGGGTCGCGGCGTTGGTCACGACGACGAGCGTCCCGCCGCCGACT

GCGGGCGCGGGTTCGGGGTCGGTGAGCGGTCTGGTCCACATCCGCTCGCC

GGTCTCGGCGTCGACCGCGGAGAGTTCGTCGTCCATGTCGTGGACGTAGA

CGGTGCCGTCGGCGACGACCGGGACGTGGTGGCCGACGTAGGTCTCGACG

GGCACCGTCCAGTCTATCGCGAGGTCGCCCTCGGGGACGGCCTCGGCCGC

GACGGCTCCGGTGTTCGTCGCCGGACCCAGCACGCCGCGCCAGTCCGTCG

TCGCCGGTTCGACGGGGTCGTACTCGGGGCACTCTCCGTTCGTCGGGTTG

TGCTCCGACCGCGTTCCGAGACAGCCCGTTGCGCCGATGGCGGCGGTCGC

CCCGAGACTCGCCAAGAGCCGTCGTCGGGTCGGTCGCTTGCCCGCGTCGG

TCATGCGACCTTCACCACCCCGCGGTGCGCGTGGGTCAGATACATCGCGT

CCCCGGTGACGACCGGCGACGCCACCGGTCGGCCGTAGTACGGCTCGGCG

AAGTAGTTCCCGCTCCGGAGCCACTCGTCGGCGTCGCCGCCCCACTGCCG

CGTCCCGTCGGCGGCGGAGACCGCTCCGGTTCGGATGTAGACCCGCCCGT

CGGCGACGGGCGGACGAGCGAGATACGGGAGCGCCGTCGGGTCGCGGCCG

TGGCTCGGCGGCGCTTCGTTCACCACGCGCCACCGTTCGACGCCCTCGCT

CCGCGAGAGCGCGTAGAGGTTCTTCGCGCCGACGTAGACTCGTTCGTCGT

CGACCGTCGGCGGGCCTGCCGCCGCCGAGCCGGTCACGAACTGCCACCGC

TGCTCGCCGGTGTCGGGGTCCAGCGCGTAGACGGTTCCGCCGACGGTCGC

GTACAGCGCGTCGTCGGTGACGGCGAGGCCGTTGGGTGACCGGCCGTACC

GCCCGTCGAGCACCGTCCGCCAGCGCCGCCCGCCGCCGTGGTCGTAGCCG

AGAACGACGACCGCCTCGCGCTCCCGGTCGAACGGCGTGAAGGCGGCGTA

GACGCCGCGGTCGTCGGCGACGGGCATCGTGGTTTCGACCGGTTCGTCCG

GACCGAGGAGGCGCTCTGCCGGCCCCGGTCGGTCGGCGGTCCACAGCCGG

CTCCCGTCGCTTCCGACGGCGTGCAGGCGACCGCTCCCGCTTTCGACGTA

CAGCCGGTCCCCGTACGCCGTCGGCGTACCGAGTCGGCCGCCGAGGTCCG

CGGTCCATTCGTTCCCCCCGTCGCTCCCGACGGCGTGGAGCGCGCCGTCG

TACCCGCCGATGACGACGGTTCCTGAGTCGTCGTCGACGACCGCGGGGCC

GCCGCCGAGGCCGTCCGCGAAGCCTTCGACCCACCGGACCGACCCGGACG

CGGCGTCGATTGCGGCCAGCGGGCTCACGAAGTCGTCCGGCCTGTAATAG

TGGGTGGCGACCCCAGTGACGTAGACCGTGCCGTCAGCGACGACCGGCGG

GGACAGCGCGCCGTAGAGCGGTGGGTCGGCGGAACTCCGCGAGACGCTCC

ACGAGACGGTTCCTTCGGTCGGGCCGTCGGGGGCGCGACCCGTCGCCTGC

GGGTCGTAGCCGGCGGTCGGCCACGCCGTCGTCGGCGCGTCGAACGTCGC

GTCCGGGGCCGGCGACGAACCGCCGCAGCCAGCGACCCCGCTCACGAGCG

CGGCTCCGCCCGCGGCCAGTACCTCTCGTCTGGAGAGCATACGCCCGCCG

TCTCGGCGCGGAATAAAAACCTTCGTGGCTCCCCGCTCGGAGACCCGCCG

ACCGCGGCCGGTCGCGCCGCCGTCGGAACCAACAGGAGTATTATCAATCG

GCACACACGTCCCCGCGATGCCCTCCTATTCGAGACGCGACGCGCTGAAG

ACGATTCCGGCGCTCGCCGCCGGTCTCGCCGGCTGTGCGAGCCTCACGGG

CCGCGACGACTCGCTTCCGATGCCGACCGCGTGGACGGCGGACGTGCGGA

CTCCGACCCGCGCCGTTCGGCCGCCGTCCGGCCCCCTGCTCGTCGGGACT

GAGAGCCAGTTTCGTGACGACCCGATGGTGTCCGCGCTCGACCCCGCGAC

CGGCGAGGAGCGCTGGGCGGTGACCGGTGGCAAAGGCCGCCGGTCGCCCC

TCGGCTTCGACGACCGCCACGCCTACCTGTTTTCGAGGGCGGAGCGGGCC

ACGGCGGTCGACTACGAGGCGGGCGAGCGAGCGTGGTCCACCGAACTGAC

GGGCGTCGATAGAGCCGACCCGGGCGTCGTTCAGTACCCGCCGGCCGTCG

CCGGCGACACCGTCTTCGTCGGGAGCGCGACCGAGGAACTGCTGGCGCTC

GACGCGGCGACCGGCGAGGTTCGGTGGCGCGCGCCGCTCCAAAACACCGT

GTTCTCTCGGCCGCTCGTCGCCGACGGCCGCGTGTACGTCGGCGGCGCGG

ACTACTTCCTGTACGCCTTCGACGCCGCGTCCGGCACCCGACTGTGGCGG

GACGAGCTCGCCGCCCCCGTGACCCGTGGGCCGACCCGCGTCGACGACCG

ACTGGTGACCGTCGTGGGGTCGGACATTCTCGTCCGGGGGCACAGCGGGA

CCGTCCCGTTCGACCCGACCGCGCTGTACGTCCACGCGACCGACGGGACG

CTCGTCGACGAACAGCTGTTCGAGCGAACTCCCGATGGCGGCAGAGTGAA

CTGGGCTGTCGCCGTGGGCGGTGGCGTCTACGTCGGACAGGAATGGCAAC

TCGCTCGCCTCGCCCCGGAGGTGCTCGATGCCGAGTGAGCCGCCGGAACG

GTCGGGTTCGGCCGGGAATCGGAACCGACACGTCTCCCGCCGCGCGGTGC

TCGGCGGGCTCGCGGCCGTCGGTTCGCTCGCGGTCGCCGGCTGCGGTTCG

ATTCCCGGACTCGGCGGGAGACGGCCCGTCTGGCGACGCGACATCGACGG

TGCGTACATGGCCGGTCCGCCCGCCACCACCGACGAACTGGTTCTCGTCG

GGATGCAGGACAAGGCGCTGTACGGCCTGCGGCGCGAAGACGGGTCGACC

GCCGTCCGGTTCGAAACGGGCGGCCCGATAGAGACGCGACCCGTCACCCC

TTCGTCCGGCGGTCCGTACCACGTCCACAGCACCGACGGCGACCTCTACG

CCGTCGATGCGGCGGGCGACGAACTGTGGCGCGACGAGGGCACGGCGCGC

CGGGCGCGACTCGTCCGCACCGACTCGCTCGTCGCTGAACTCGACTTCGC

GCCCCCCGAGAACACGCTCACGGGCTACGACCCCCAGACCGGTGACCGAC

GCTTCGACCGGGCTGTTTCCTCGCACTCCCTCAACGGCGTCACCGACGAC

TCGCTCGTGGTTCCCGTGCCGGTGGGCGGGAGCGACTCGCGCGTCGTCTC

GCTTTCGCCGGCGGACGGGAGCGTCCGGTGGCGAACTGAGCCCCGACGGT

GGTATTCCAACGTGGTCGCGGACTCTCGACTCGTCGTCGCCGCGAGAGAC

GGGACGCTGGCAGCCTACGAGCCGTCAGACGGGAGCGCTCGGTGGCGCGT

CCCCATCGGCGACGTGGGACGGACGATGGTGCTCGGGTCGCAGGCGTACC

TCGCCCGCGACCGGGAAGACGGGCGGCAGGAACTGCTCGCGTTCGACCGC

GAGACCGGCGAGAAACGGTGGGCGAACCCCACCGGCTACCAGATTCGAGC

GGTCGAGGCCACGGACGACGCGGTGTTCGTCGGGACCCGCGTCGACGACC

CCGACGGCGGGGTTCTCGGCCAAATCGACTGCTTCGGCCTCGACGGGACG

CGGCGGTGGCAGACCGTGACCGGACTCCCGTCCGTCGACTCACTCGGCGT

CACTGACTCTCGTGTCGTCGCCGTCTGGGACCGCGGCTTCGAGGTGCTGG

CCCGAGACACCGGCGCGTCGCGGTGGTCGTACGAACCGGAATCGGCCAGT

CGGCTCTCGTTCCGCGTCGAGTCGGCGTCGGTGTTTGTCTCGTACGTGGA

CGACGGCGAAGCCGCGCGGTTCCCGCTGGACTGACTCCGCGTCCCCGAGT

CTCCGATTCGACGACGCGCTCCCCACCAACTTATTGCTGTGCCGAGGGGA

TACCCCGTCAACGAGGGACACGAACCGATGGACGACACACGCCGCGCGTC

TCCGCTGGAGCGCCTCGTCGAAGCGGCCGAAACCCGAACTGACGATGAGG

TGAGCGACTCTCTCGGGGAACTCCTGACGGCGTCGCCGGAAGACCGAAAG

CGGGCGCTCCGCGAACTTCGAGACCTCGCGAACGACAGGCCGACCGCGTT

CGAGTCGTTTATGCCTGCGGTCACGCCGTTTCTCACCGACGACGAGCGCG

CCGTTCGACTGCTGACCGCGAAGGCGCTCGTCGCGGTCGCGGAGGCCGAC

TCCGACGCGGTCGCGCCCGCGGTTTCGGCGCTCGCCGAGCGCCTCGCCGA

CGAAGACGAGTTCTACTACGTCCGCGCGCGGTCGGCCGAGGCGCTGGGCT

ACGTCGCGCTCGACCACCCCGACGCGGTCGCCTCGCCGGCGGTGCTCGCG

GACCTCCGCGTCGGCCTCTCGTTCGACGAACCCGAGGTGAAACAGAAGCT

GGCGAAGGCGCTCGAATGCGTCGCGCTCGGCGACCCCGGGCGGCTTCGCC

ACCGCGTCTCGGCGCTCGCCGAACATCTCGACGACGGGGACGAACTCGTC

CGGTACCATCTCTGTACGGCGATTGCGGGCGTCGGCTGCGATTCGCCCGA

CTCGCTCGCGGCGGTTCGCGGGGCGCTCTCTGCTCGGCTCGTCGACGAGA

ACGCCTTCGTCCGGGGCCGCGCCGCCGAGGCGCTCGGGCTGTTGGCCGGT

GAGCGCGGCGACCGCCGTGACCACGGTGAATCGGTGGCGGTTCCGGACTC

GGCGCTCGGTGCGGAGTCCGACGAGGCGGCCGCGTTCGTCGCCGAACGAG

TCGGGTTTCTTCGGGCGTCGATGGAGGGCGATACCGCGGCTGCGGCGTCG

ACGGTCGAGGGCGTCGGCACGCTCGCCGGCGTTCGACGCACGACCGCGGA

CGCAGTCACCGAGATAACGTCGCCCGACGCAGAGGGTGTCTGTCCCCACT

GCGGTATCGACCTCGCCGAGGGCGCGCCGCCGATGTGTCCGAGCTGCGGC

GCTCCGTACTGAACTCCGCGTCCGGCGTGACCGACGAGCGGTCGTCCGTC

ACGGCCGTGCCGACCGAAACTCGCTCCCGCGCCTCGGACGACAGCCGCCG

CCGTTCACCTGTCGGCCGCAGCGAACGTGACGCCCGTAATCTCGAACCGC

GCGCCACCACCGTCTGACTCGGTGACGCTGACCGTCCAGCCGTGGGCCTC

CGCGACCTGCTTCACGATAGCGAGCCCGAACCCGGTTCCCGTCCGTGACG

TCGTCTGTCCGGCTTGGAAGACGCTGTCGCGTCGGTCGGCCGCGATGCCG

ACCCCGTCGTCCTCGACGTAGAACCCGTCGGGGAGTTCGCCGACCGTGAC

CACCACGCCGTCGCCACCGTGTTCGATGCTGTTTCTGACGAGGTTCTCGA

TGAGCTGTTGGAGCCGACTCCGGTCGGCGACGACCGTCGCCGTCAGGTCG

GTTTGGAGTTCGGCGTCACCCGTTTCGATGGTCTCCCAGCAGTCGGTGAC

GAGCGTCCCCAGCGACACCGGTTCTCGTTCACCGACGTTGTCGCCTTTCC

GGGCGAGCGTGAGCAGGTCGTCGATGAGGTCGTCGATACGGGCCAGCGCT

CGAGCGGCAGTCGCGAGCTGGTCGCTCTCGTGGTCTTCACGAGCCAGTTC

CACGTTCCCCGCGGCGACGTTCAGCGGGTTTCTGAGGTCGTGGCTGACGA

CGCTCGCAAACTCGCTCAGTCGCTCGTTTTGGCGCTGAAGCTGCTGTTCT

CGCTCGCGTTGCTCGGTCGTGTCGCGATACATCCAGAGATGGCCGGTCCC

GTCGGGGAGTTCGATCGGTTCGTAGCTTCGGTTGAGCGTCCGACCGTTCG

TCAGGGCGACTGACTCGTTGTGGACCGACTCTGTCCCGGCGATTAGTTCG

TCGACGCGCTCGACGAAGCCCTCCGAATCGACCACGAGTTCGCTGGTCTT

CCGTGCCAACTCTCGGCAGTCAGCGCCGATGAGTTCCGTCGACGGTTCCG

AGATGCCGAACAGTTCGAGCAGTCGGTCGTTAATCGCGAGGACGGTCCGG

TCGCTGTTCTCGGCGAGGACGCCGACCGGGAGCGACCGGATGAGCGTCGA

GAGCAGCGCGTTGGTTCGTTCGAGTTCGTTCTCGCGGCGGACGCGTTCGG

TCACGTCTTGGAACAGCGAGATGATGCCGACGACCTCGCCGTCGTCGTCG

GTGATGACTCGATTGTGCCACTCACAGCGGATGTGTTCGCCGTCTTTCCG

GACGTTCTCGTCGATGCTGTGGTAGCCGCCCTCCGCGACCGCGAGCTGGT

CCGTGACGGCGTCGACGTTGTCGTAGCTGTCGTCCGCGACGATGACCTCC

CAACTGTGGCCGCGGAGTTCTTCCTCAGTGTAGCCCAGAATCTCCTCGCC

GCGCTCGTTGAGCCCGATGATCTGGAAGTCGCTGTCGTATTCGAGGACGC

CGAGCGGTGACTGTTCGATGAACAACGAGAGGCGCTGTTCGCTCGCTTCG

AGCGCGCGTTGCGAGCGGTGCCGCTCGACTGCGTTTTCGATACGGTTCGC

GAGCACGGTGTACTGGCTGGTTCCCTGTTCTTTCTGGAGATAGTCGGTGA

CGCCCGCGGAGATGGCATCGCTGGCGACCTCCTCGCTTCCTTCGCCGGTA

AAGAGAATAAACGGGAGGTCGGGTGCGTCCTCGCGGAGGGCTTCGAGAAA

GTCTAACCCGTCGCGCTCGGGCATGTCGTAGTCCGATACGACACAATCGA

ACGTCGAATCCCCCGCGCGATTGAGTCCCTCGGCGACGGACGTGGCCGTC

TCGACGCGAAACCGGTCGCATTCCCGTTCGAGGAACGCCGCCGTCAGTTC

GGTGAAATCCTCGTCGTCGTCGACGTACAAGACGACCACTTCGTCCACTA

GCTCGCTCATGTCTATCCCGCTTAGACGTGTGCTGAGAAGGTACTTAACG

ATAGTCGCAGTTTGAGGACCGAGCAGGTCGTTGAGTCACGTTGGGCTCAC

ATTCACCGACCTGCTCGACGAGTGCTTTCTGGTGAATTTCGAAGCGACGT

GAACGCGTGAACCGGGTCGCGTTTGCGGACGGCTGTTCTCTGCGAAAACG

AATACGCTAAGCCAGTCGCCCCCGGTGGTTGCTTTGACGTTCCTGAATAT

CTGACTGGCCTAACGTTCGGAACTGTTTTGTATGTTTAGGCTGGCCTAAA

TAATATGGTGGACGATTCCGAAAACAGCAAGACACTGACACGGCGTGGCT

GTCTGAAGTACGGCGGAACAATCGCGGGAAGCGGGCTCCTTGCAGGCTGT

TCTGGTACTTCCGAGCCAAGCAGTTCGTCTCCGAAGTCGCGTACGTCATC

GACGCAGACGGCTGTTTCGGAGACGGTCTCGGAGACGGATACGGCGACCG

GGTCAGATGAGCCGTACACTGTCACAATCGAACCGATGGGTGAGGTTGCG

TTCGACGGCCCGCCCGAGCGGTGGACCGCATTACTACCCACTTTTGCGGA

TATGGCGTTCGCACTCGGTGGTGGACAGACGCTTGGTATTCAGAACCACG

ACCGTTTTGCGAGCGAGGCCTTCGAAGAGCTTCCCGGGATAGACTTCGAC

GCAGATGACACCGTGGAACTGGTTGCCGACGGTGTGAGTAAGGAACTGTT

CTATGAGATGAACGCGGACGTTCATTTCATAGACCCCCATATACTGCGAC

TCTGGTACGGCTGGGATCAGTCTGACGTCGATGAGATTGCGGACAACGTC

GGCCCGTTCTTTGGCAACTTTATCCGCCGCCACAGCGACGACTGGCACGA

TTACCGTTACTACACCTTGTACGAGGCCTTCGGACTGATGGCTGAGGTCT

TTCAGGCACAGGACCGATACCAGGCATTCGTCGAACTCCACGAGGAGATG

CTTGCGTTGGTCGATGAGCACTTGCCGCCAGCCGACCAGCGTCCAACCGC

CTTACTGGTATATCCGGCCGACGGAGCGGGCTTCCAGTTCTACCCGTTCC

GGTTTGATGACGGGGGAGTCAGCACTAAGCAGTGGCGTGATCTCGGGCTG

ACCGATGCGCTTGCGGCGACGGATGTCGGTCATTACAGTTTCTCTGACAG

GGGGACGCTCGATATCGAAGCCCTGATGGAAATCGACCCCGAGGTGCTGC

TGGTGCGAAACTATGGCGGGGCGTCCGAGTCAACGTTCCAGAAGGAAGTC

GTCGAACCACTCCAAGACCAAGCGGGGTCTCATAGCCTGCAGGCGGTTGA

GGACGACGCGGTTTACAGTGCCGGTTACCTTGACCAAGGGCCAATCATCA

ACTTCTACCATACCGACCGGGCGGCGAAAGATATCTACACGGACTCGTTC

GAGAACGTGACGCTATTCGACCGTGAACGCGTCGCAGAAATCGTCCACGG

AAACATCTGAGACGGGTTAGAATAATACCTACCGCGGGACCGTTTCTGCT

TCGGGCAGTTACGCCGAGTATCGCTAGTTCGCCCTCAGCGTGCTCAGTTC

GAAGTCACCCGGGTACGCGACGCGAGGGTACAAAACGATACTGAATTTTT

GACATTTCCGGGCGTGTAACGATACTCACCGTCGGTTCGCAGGAGGGCGA

CCGTCACCACGCTCGCTAACTCAGTCGCACTCGTTCTCTGAACGTTGGCC

TCCAGTGAATCGGTTCCACTCGGCTCGACCGAGGGCACTCAGAGGTCATC

GACCCGACGCAGAAACGAACAGAGTGCGTCGGTGGCCGTTTCGAACAACT

GTTCGCCCTGCTCGGCGGACGCAGACGTCGCATCCCCGACTGCACCGTTC

TTGCTGAACTCTTCTGTGAACCGATTGACGACGCCACCATCGACCGTGTC

GTCCCAGGTTGCGGCGTCGCCGTCGGTTGGGCTGCCGACCTCGTCGGGAC

AGATGTGAAGGTGCATGGATGTCTCGAGTTCACCGGCGTGGTTGACGTGG

TCGTCAACGGCGCTCATCCACTCCCAGAGGAACACTTCGAGGTCAACCGT

CGAGTCGTCGGCAACGGTACGCGTTAGGTTTCGGAGCGTCTCACCGTTTC

CACCGTGGCCATTGACGAACACGACCGTCTCCACGCTCGAGTTCGACAAC

GAAGTGAGAATCTCATAGCTGTACCGCCGAAGCGTTTCCGCGGAGACCGA

TAGCGTCCCCGGGAAGTGACTATGATACGGTGCGATGCCGACTGGAATCG

TCGGCAGGACGACCGAGTTGAGATCACTGCGCCGGCCAGCCTCGCTCGCC

AGTTCCTGTGCGATAATCGTATCCGTACCGACGGGTGCGTGTGGCCCGTG

TTGTTCCGTGCTTCCAACGGGGAGCAGCGCTACCGTCGGTGCCGCGTCTT

TGACGTCCGTCCAGGAACTCGTCTCAAGGTGCATACCGTTCGGTGCTGAG

CACGCTACATAAGCGATACCAATCCGGTCACTCGGCGGCATACCGTGTCG

GCCGCGGGCCAATACTGTACGTTGTGCTGATTCGGGGCGCGAATCTTGCA

TGCGATTCCCAACTGAAATCGTTCGATTGCTCCCGTGTGACGAGCGCTCA

ATTGACAGTTCCACGGATCTGCGCGACACCATGGCGAGCCTGGATTATTT

CGGCAGTCGCTCGACCAGCCCATCGATTTCGGCGTGCGTGTTGTACACGT

GAGGACTGACACGAATATTCCCTTCGCGCAAGGCTGTATGGATGCCAGCA

TCCGCAAGCGCTCGATGGGTTTCACGGGCCACGCTTTCCGACGAATGGCT

GAGTACGACGATGTTGGCGTGCCGTTGAGGGTCTTCCGGCGTTACAACCT

CGAACGAACTCGATTCAGCCCCCTCGATGAGCTGACCGACGAGTCGCTCG

CCATGAGCGCTGATCTGCTCGATGCCGATTCCGGTCAAATACTCGACCGC

CGCAGTCCAGGGCATGAAGTTGTTGAAGTTCGCCGTTCCAAAGACGTCGT

ATCCGGACGCACCGAGTTCCGTATCAAGCGACAGATTCTGCATTTCGTCC

AGCGTCGTTCCGGCTTGCATCGATAACCAATAAGCGTTCACTGGACCAAT

CGCATCGAGGACCTCCGGTGTGAGCCACGTAAAGCCCGTGCCGTACGGTC

CGCAGAGCCACTTGTAGCCACAACTGATGAGCACATCGACCGGTGTTCGC

GATACGTCAATCGGCCGCGCCCCAACGCCCTGCGAGCCGTTTAGCACGAA

GAGCACGTCGTGTTCGTGACAGACGGTTCCGATCTCGTCGATATCGAGCC

GCCGCCCAGTGAACGAGTCCACCCACGTCGTACAGAAGGCGCGCGTCCGC

GGGGTCGCTGTCTCGGCGAGCTGCTGTGTCAAGGGTGTCGAACACTCGGC

ATCGACGAACCGCAGTTGGACCCCCTGGGATTCGAGCAGATGCCACGGGA

GAATTGTCGCCGGGAAATCACCCTCGACGAGGATTACTTCATCACCTTCG

TTCCACTCAAGCCCGTGAATCAGCAGGTGCAGCCCGTACGAGAAGCTGTT

TCCGAGGATAATTTCGTCGGGAGAGGCGTTGATGAGTGTCCCGAGTGCCC

GTTTGACCCCGGCTGGTACCTCGCGAAAGGCCTGTTCTGGGAGCCGATGG

GGCGCTCGTTTCAATGTAACCGCATCGTCAACCGCCTCGACGGCTCGCTT

CGGTAAGGGCCCTTGATGGGCGCAGTTCAGCCAAGTATGTCCATCGAACG

GCCCGAAATCCGCATTCCAATCCGCCATGAGTCTCGTAGCCCTAGAGCCG

ACTTAGTAATTCACCCGAAGCATTCTGTGTCGAGCTTATCTCCTAAGCCG

ACCGGGTATCTGTATCCACTATCGAGAACGTCCCTCCGCACCGTGCTTCG

TGAGGTACTTGGAGAGGTTATCTCTGCGCCGGCGATGGGTGTCGTCGCAC

ACGCGTCGGCGGTGATCGATGTCGACACGAAGTCAACGCGGTTCATCAAA

CAGAGCGAGCCGAGACGTTCTGACCTCAGGACTTTTGGGGACCTCGTCCG

TCGTCTTCCACTGACCATGACCCTCGTCGTCGAGTGTCGTCTCGTCTCGG

AGCGGTTGCCGCTGACCGACGTCGCCGCCGCCGTACCGGACGTGACCCTC

CGGGTAGAGAACACGCTGACGTCCGCGCGGAGCCGACCGGTGCTTATCTT

CTGGGCTGATGGCGGCCGGTTGGACTCGGTCGACGCGGCGTTACGGGACG

CGTCGGTGGCGACTCACAGCGTGCTCGGGTCGACCGTGGACCGCCGTCTC

TATCGCGTCGAACTCTCGGAGCGGCCGCCGGCCATCTACACGGAGTTTAT

CCGGCTGGACACCGCCCCCATTTCAGCGACGATAACCCCGAGCGGATGGG

ACGTGCGGACGCGGCTCTCCGACCGAGCGACGCTCGCCGAGTTCAACAAG

GACTGCGAAGAGAACGGTATCACGTTCCGACTCGACCGCGTCGTCGACGT

CGCCCCCTCGGACAGCAACGAGTACGGACTCACCGCGAAACAGCGCGAAA

CGCTGCTCGCCGCCCACGAGGCGGGTTACTTCTCCGTTCCGCGAACGACT

TCGCTGGCGGAGTTGGGGGCCGAACTCGGCGTCACCGCCCCGTCCGTCTC

CGAGCGACTCCGGCGGGCACAAGACCGACTCGTCCGGCACACTGTCGCCT

CAGATAAAAAGACGTCTTAAACACCACAACAGGTTAGAGTCAGTACTTAT

CGCCGCACGCCCCCGGGATACAGACATGGCATCGAAGCTACAATCACGGG

TGCGAAACCTCCCCGTGGACGGCGTCGGACGCCACCCGAATCGGCGGGTA

GGCCGCCGCATGACGACGGTTGACGGGGTGACCTGCGCGTGACCGATACG

GTCGGCACCGAAGCGGACGCGGCCGAACGCGACGCCTACTTCGTCGGCGG

CGGCATCGCGTCTCTCGCCGGGGCCGCGTTCCTCCTTCGAGACACCGAGA

TACCCGGCGAGAACGTTCACGTGCTGGAAAAGCGGCAGGTGTTCGGCGGC

GCGCTCGACGGGCGCGGAACCCCCGAGGAGGGCTACGTCCTACCCGGCGG

CCGCATGTTCAACTTTCCGACCTACGAGTGTACGTGGAACCTCTTCCGGT

CGATTCCGTCGCTCGAAGACCCCGACACGACCATCAAGGGGGAGATGGAC

GAGTTCAACGAGAAACACGAGACGTACGCCGAGGCCCGACTCGTCGGCGC

GGACCAAGAGATACTCGACGTCTCCTCCTACGGGTTCGAGACGCAACACC

GTCTATCGCTGCTCCGCCTCCTTCTCACTCCCGAGGAGAGGCTCGGTGAG

ACACGCATCGAGGAGTGGTTCGACGAGACCTTCTTCGACACCACGTTTTG

GTATCTGTGGGCGACGACGTTCGCCTTCCAGCCGTGGCACAGCGCGGCGG

AGATGCGCCGGTACATGCAGCGGTTCACACGGGAATTTCCCCGACTCCAC

ACGCTGAGCGGCGTGTCGCGGACGAAGTACAACCAGTACGACTCCGTAGT

ACGACCGCTCCGCCGTTGGCTGGAGGCACGCGGCGTCGATTTCTTGGGTG

GCCACACCGTCACCGACCTCGATATCGTCCCGGCCCGCGCGGGCAAGACG

GTCGAAACCATCCGTTACGAGGACCAAGACGGCTCCGCCGGCACTATCGC

CGTCGAGCCGACCGACGTCGTCTTCGTGACGAATGGGTCGATGACGGACG

CGCTGAGCCTCGGGTCGATGACCGAGGTCCCCGACCTGCACGACAGCGGC

ACCTCGTTCGAACTCTGGAAGGCACTCGCGGACGACTTCCCCGAGTTCGG

GAGGCCTTCGGTGTTCGCCGACCACGTGCCCGAGTCGATGTGGGAGTCGT

TCACGCTCACACTCCGCGAGCCGGACTTCCTCGAACACGTCGTGGAGGTC

ACCCGCGAAGAGCCGGGGAACGCGCTCGTGACGTTCACGGAGTCGAACTG

GCTGCTCTCCATCGTCACCGCCGTCCAGCCCCACTTCGCGAACCAGCCGG

ATGACGTGAAGGTCATCTGGGGGTACGGCCTCTTCCCGGAAGAACGCGGG

AATCACGTGAACAAGAAGATGGAGGACTGTACGGGCGAGGAGATTCTGGC

GGAGCTCTATCACCACCTCGGCTACACCGACCGTCTCGAAGAGCTCCGCG

AGACGGCCACCTGTATCCCCTGTATGATGCCGTTCATCACGAGTCAGTTC

ATGCCGCGGACGCCCGGTGACCGCCCCGAGGTCGTCCCTGCGGGATCGAA

CAATCTCGCGTTCCTCGGCCAGTTCGCCGAGGTTCCCGACGACGTGGTGT

TCACCGTCGAGTACTCGGTTCGCTCGGCACTGATGGCGGTTCACGAACTG

TTCGACGCCGAGGGCGATGTGCCGCCGGTGAGCACCCATCAGTACGAGCC

CGACGTGCTTCTCGACACGGTACGGGCCGCTTTCAGATAGCGACCTCGAA

GACGACGCTGGCTCGGATTCGGCGGCCGTCTCCAACCGTTGGCGTTCGAA

CTCCTCGACGGCGATATCTGTGTTCGCAACGAGCCGAATCGACGACGGTG

AAGCCAGTTGCCCGCGGTCCGTGAGGTGGATGCACCGTTCCTGATTACTG

ATGACTTCAGGGCTCTTCCTGAACTTCGAGAGCTAGTGAGTGCGGCTGTT

GCATTATCACCGATCAACAACCTCGGAGGCTGATTTTGATGCGCGACTTG

CGTTCAGCAACATCGAGACTGAATCGGACCTGCGGTAGACCCGCTTTCTG

CCCGTTGAGGGACCTGGGGTCAAATTCAGTTAGAAGTCGCCGTTGATGAT

ATTCGCGACCTGTTCGCGGTCGAACAGTTGTTCCTCCTTGGAAAAGTCGG

GATACGGACCACCGTCGTAATCAGGCCACTCACCGAACTGCTCCGGATAC

AGCTGTTTGGAGACCATCTCTAACTGGAAGAGGTTCATTATTGGGCCGCC

GTACCGGGCGCTAAGCGGATACACCTGGTCGTTTTTCACTGCCGAAATTT

CTCCCGCAACCGGGTCGTTGTGGAACTCGTTTTTTACTGCTCGCCAATCG

TCGCTCCTGGCAAACGCGTTTTCGACGAGGATGACTTCCGGATCGGTCTC

GACGAGGGTCTCCAGGTCAATCGTGCTGCTCTCTTCCACATCCGAGAGTG

CGTCGGGGACGCCGAACGGACGCGTGTGCGACCGAATGAATCCCGGGCCG

TTCATGTGGAAGGTCCAGATGGTATCCAGCTCCGGAGTCGTCATGATTCG

CGCCGTTTGCGGCCGGTTATCGCTTGCTGGGAGACTCGACGAGATTGTCG

AGTCGAGAGTGGTTTTGACGTCGGAGAGCGCTTCGTACCGCTCCTGTTCT

CGGAAGACTTGCGCTACTTTGCCGAAAATATCCCAGAGGGAATAATACTG

GTACTGGTCTGCCCAGTCTGCCGGCGGTTCGCTGTGGTTCCGACTGAGGG

AGTTCCCGAACCAAGGGGCGATTTCCGTTTGGACTTCCTCGACATCCGCG

GTGTCCCACGCATCCATTGTCGAGACGTATGCCGGGTCGGCTAGGTGGAG

GTCACTGTCGAGTTCGTACAGGGTTTCCTTGTCCGGATTCCACGAGTTGT

AGAGATTCGACCAATCGACCGAGACTCCATTGAGCCGTTCGAGAAGCTTG

TCGTAATTGCCCTCGAAGTTGGACGGGCTGTACATCGCATTGAGGGCATC

ACCGTGACCGAGAGCGACGACCATGTCTGCGTGGTGAACGAGAACGGTGA

ATACGCTCTTGGGAACCTCGTCGAGTTCGATAGTACCGGCTGGTGCCATC

GTCACCGAATAACTCTCCACCTCCGGTTCTGTGGTCTTCGTCGCGGTTGA

CGAGTCGTTCGCGGTTGTCTCAGTCAACGGTGACGCAGAGTTCGACTGTC

CGATACAGCCAGCGAGCAGTCCCCCACCGATGACTGCCCCACCGTACTTG

ATTGCGTCTCGTCGCGTCGGTGTCTCGTGATCGCTGGAACCGTCTGGCAT

ATGGTTTAGGCTCACCTAAATACGCAAAAGCGTTCCGAATTTTAGGCAGG

CCAAAAATATCGGCGAGGGGCACCGCGGCGTGGGCACATCGGCGGCCCAC

TCTCGGTGAGACCGGGTCCGCAACGCGGACACACTCTGCCGTCAGGAGCG

TTCATCTCCTCGACCGCGTCGTCGGTCCCATCCCGAACAGACCTAATTGT

ACCAACCCCCGCTGCAACCGACCCGGACGATTCGTTCCCCAAATAGTGCT

GACAGAGCTGTACGCGGTCGGTGAGGAGCGCCGACGTTTCTTCGCCTTTG

GCGTCCATTTCACCGAGTGTTGGTGCCCCTCCATCGTGAGCGGGCAGACC

ACAATTACCGTTCTTCCGCAGCTGAGGATGATAATGTTTAATATTTTGTC

CGCCCTGTGTTGTTATATGAGCGTGGTCAGCGTCTCGATGCCGGAGGAAT

TGCTTAATCGAATCGACCAGTTCGCGGACGACCACGGCTATACTGGTCGC

AGTGAAGTAATTCGCGAGGCGAGCCGAAACCTTCTCGGTGAGTTCGAGGA

CAAGAAACTCGAAGACCGAGAGTTAATGGGCGTCGTTACAGTCGTTTTCG

ATTACGAAACGACAAGTGTCGAGGAGAAAATGATGCACCTCCGCCACGAG

CACGAGGACATCGTCGCATCGAACTTCCATAGCCACGTCGGCGGCCATCA

TTGCATGGAACTGTTCGTGTTGGAAGGGTCGCTCGAAGAAATCTCGACGT

TCGTCGGAAAGATTCGGGCGACGAAGGACACGCTCACAATCGACTACTCA

GTACTCCCCGTCGACGACTTCGGCCCGCTGGCCGACATGAACTAATTCTG

CTGCCCTTCTCTGCGTTCACGCCGCCCCTAGCAACATCTACGAACTTCAA

TCTTCGTTAGTATCAGCTCTAGATGGGAAACTCACAAGAACCGGTTGATA

TGTCGAGAGAGTTGCCAAAACCAATGGCTCAAATCTCGCGTCGAAGACTC

AGTACCTCACAAAGCATCCTCGGCTAGCTGTTTCTGAAGCCTGAGTTCTG

TGGCGGAGCGGTTGGACTGGCGGTTTCGACGAGAGAATCATGGACGGATC

GGCGGAGAGCCGTCGCTGTCGGCGGCGGCTGCCGCCGACGCGACAGCGTC

GCCACTCCCACCGTTGGCTAGCCCGGTCGGGAGTTACCGGTGGAACCGGT

CGTCTGGTGGTCGGTTCGCGGGGACGGCCCGACGCACCGCGAGGGCCGTC

CACGCTGCTCGACGGACCATATTGATGAACTCCTTGAACGACCACTCCCA

GAGGCGACGCCCCCCACGGCGGGGCGTCGCCACGCACTCCCAGTGCAAAT

ACCGCCAGACGTTCTGTAACAGCAGGCTCACCACGACGTACAACAGCCGT

ACGACCGGATTTTGTGTCGAGGTCGTCGCGATACTTTGCTCGGAGAGTCG

ATAGCTTGCCTCGATACCGAAGCGTTTCGCGTAGTGGTATCGAGCGTCCC

GTGGTGAGTCGATGAACGGCGCGTCAGCGGCGTAGCCGTGACGCGCCACC

CCATGTTCGTCGTACCGTCCGTTCTGGTAGGTACAGTCGATGTAGACGGG

AAACTCGACGGTCCAGCTGTGACCGTCGAGTTTCGCTGTCAGACTGTGCT

GAATCACGCGACTCCACCCTTCTGAGAGTTCTCGCTTGATCGTCCGTCCC

CAGCGGACGATCGGCATGACGTAGGCGTGGTTGTGCGCCTGAAGCAGCGT

CAAACACTTGCTGTCGTAGAATTCGCGGTCAAGATAGACGGCCTTGACAC

CGAGGTCAAGGCCGTCGAGAATACCGAGGAACTCTGCGAGGACACTGCTG

GCGGTGTCGCCGTCTTCGAGACGGCGCACCGCCAGCGTGTAGCGTTTGTT

CTTCACGCGTGCGTACAGTGTCGCGTACGCGTGAAACGCGGTGGTTCCAC

GCTTCGCTTGTGAGTGATACAGGCCGTCTGTATCGTCTTCGTCGCCGTAG

TAGGGCCGCAGGTGGAGGTCTGCGCAGACCTCCACCTGCTGGGGAAGGAC

GTCGAGAACGTCTTTCTGGAGGAGCATGTTGCCAACCTGTTCGAGCGTTT

CTAGATCGAATTTAGTGCGGAGATGGTAGAGAACGGAATTTTCGTGTGGT

TCATCTTCGCTTCTCTCACAGAGCGTTGAGACCGAGGTCCCGTCGGCGCA

GGCGCCGACGAGGACCTCGTAGATGTCTTCAGCATCGAGTTCAGCGTTTT

CAGCGAGTGAGAGAGCAACTTCCTCGTCAAGAGAGTTGACGAGGAAGTTA

AGGAGCTGGTCCTCGTGGATTTCATCGTCTGCTTGCTGTGGTTGCTTGGA

CACATCTTCAGCAAGCAGACGTCTCAACTAACAGGCTTTGTGATGTACTG

AGAATGGCTCGATCGTCTGGAACCGCTTCGTCCACGTCATCGCCACGAGC

CGGGAGGATCTGCTCCGGTTCGCGGGGGACGGTGACGTCGGCGTTCTGCG

CTTTGATGAGAATCGTTCGAGCCGATTTCTGGACCGTATTCCGAAGAGCA

TTCGTGAAGCGCGTGTGCCAACTGCGCCAGAGTGTCGATTGGTCAGGCAC

TGTCTCCAGCCCAAGCCGCTCGCAAAGTACCGGATGACTTTCGAGGTACT

CGTAGAGTGCTGTTTCGTGCTCCCATCCGTGAAGTTCTTTCAGAACGAAC

ACGCGAAACAGGGTGTCCATCTCGTAGTGTGTCGGATCCTCGTAGCAGTC

GTGGGCCTCGAATTGGAAGTAGGCCAGCGGAAGTTCGGAGACGAACTGCT

CAACAGAGATGTGCTCATGGTGCGTGAACCAAGCTCCTGAGACGATCCGG

ACATCCGACTCTAATCCGGTAAGTGAGGTGTGGTCGTACAACGGTGTCGA

ATCGTACGCTGGCCACTCAACGTACGACAGTCGTGCAATTTGTCGAAAGA

CGGTTCTCCGAGATTCAGTAGTAATGGGCACTACCGGACAATCGTTCTGG

CCCCCTTAAGAACACGTCTACATCTAACGAAAAGGCCCGCTTCTTACAGA

AGCTGACGTTTTATTCGTGATCTTTTGGTTTAGTCGCTTCCGCGCTCCCC

TGTTCAGAAATGTCAACAGCTTCGCTTGCCGAGGCAGCCTGTCTTTTCTC

TTTCACTCGTTGTTCAATCGACGCCTGCATCTCACGCATAAATCCGTCCA

CCGTGTTACCCATGAGGATGACCGGTCGGCCATCGGTGAACAGTTCAGTT

AGAACCTGCTCCGGGTCCGCATTTTGACTGTGGCACTTGGTTGGAACATC

GACGTTTTCGGCGAATGCGCGTGTGTTAGCCCCACCGATGTGAGCTTCAT

CGATTAACTCTCGCTCGAATAGCAACTCGATATATTGAGCAAATGACGCT

GTACGCCCCCGCCGGTCTCTTCGGAGGTACACGAACGGGGTGATACATTC

GTCATCGACGAGCACCCGTCGAACCATCTCAGTACTTTCGACGTCGTTCA

CCTCGGCAGCGTTGAAGATGAGTCCGTCTTCAAGTTCTATCCACGTTGGT

TGGATCGCACTGAGGAACGATTCGATCTCCTCGGCTGGAAGTGACGGTTC

GCCAACAACATCTAACACCGCATTGACTGCGTGGATCGTTTCAGAACCGA

TTAGCCCTTCGTGTTCGTCGGGAATATCAACCTGCTCGATAGTCCCTCCG

CGGCGTTCGATCTCTTTTTGCATATATTCGTGCAACACCGGGTGTTGCTC

GCCGCTCACAACGTGTGTTCCCGCTGGGACCGACCGCGCAAACGAACGTG

CAAGGTCTGTCCTGGTCTTTCCAAGGGTATCTGTGTGGTCCTGACGGACG

TTTGTCAACACGATGATGTCCGGGTTGGTCAGACGCTGATTGAACAGCCG

GGTCGTATACTCTGTAATCCCCTGATTCTCGAAGATAGCGATGTCGTCTG

GCTCGTACGCATCGAGTTCGGGGACGAACTCAGCGATGAGACTGATATTC

TCGTAGAGCGTCGTTCGAGGCCCCCGTCGTTCGATAGGATGGACCTCGCC

GTTGTGGATCAGTAGTGGATGGTTGCCGGTGATCTTCGTGAGCGTGTCGT

ACCCTCGGCGGTTGAAGACGTCGTCGAGCCGTTGGGTGGTTGAGGACTTA

CCGCGAGTCCCCGAGACGACAATCTGGGTATCGATCTGATCGAGGACCTT

ACGATGTCGAATACCTCTCGTTGCTTCACTAACCAGCCCTGTTAAACTCG

ACGAGCCGACTTCGATTGGCTTCGGGCCAGTCTTCCACTCGTACTCTGCC

CCAACCAAGAACATCGGCAGCGCCCCCGCCGCATAGTCGAGGAACTGGTC

CGCCGCGGACTGTGCTGCCATTTCCGTCGGGAAGCGATCGGCACTATGTC

CCAGTACCCCGTTTGCAGTTTGGAGCCGCCAGACCCAGTTGTTTCGTTCC

TCCGGAAGCTCTCCTGACGGTGTGGCCTCTTGTTCGACGATTATGTCGGG

CGCGTCGACATCGGTGTCTGGGAAGGACTCTTCGTTCCCCGATAGTTCTG

CAACAGCGGCGCGAACAATGTCAATTGCATCGCGTGCTGCTCGGGATGTG

TCGTAGTGGCGGCTGCCGGTTGCTAGTACCTCCGCTGGGCCCCTAAGACG

CCACGACCACCCGAACCCACGGCCGTAGATCTCGAAGGCAGTCCGACTCA

TGTTTCACCCCCTTCAGAGAGAACCGACGCAGAGAGAACCTCATCTTCAC

CCGGGGGTGACACAGTATACCAGTACGCGACACCCAGGGCAGCCACCCAG

AGAACCGCTGCAATCCCCAGGACAGGCACCGTCAGCTCTTGTGGAAACCC

ACGAGGTTGGGGAGCACTGAACAGTCGAGCGACGATCAAAGCGGGGACAA

AGACCACGATCTGAAGCGGGACGACGAGGCGGCGCTCGAATGGTGCCGAC

GCATGCGCGTTGTACGCCCCAATACCTGCCAAAATCGCAGTAAAGAACGC

CGATAGCCCTCGCTCGATTGGTACGACAAACGTCAGCGATACTGCGGCGA

AGATCGCGACTGCGACAGTCACTCCAAGAAGCACTCGTCCGTACCGGAGT

GTGAGGTAGTTCGACAGCTGGATGAAGCCAAACGATAGCGCAAGCAAAAG

CACGTACAGGAGTACAAGCCAATAGCTCGCTAGCGCGAAAATCGCGAGGA

GGACCGCACCAATGATTCCGACCCGAACACCGAACCGGCCCCGGAGCCGT

TCAGATAGAACTAGCCCACCAGCGAACAGTCCGGCCACAATCTCTCGAGA

GAGGATGACGGATTCGGGGTCGATGGGCACTGCAACACCTTTGTAGATAG

CGACATCTGCCGTCGAAGAGAAGAGGACTGGGGGCGTCAGCGTACCAAAT

TCACGCGCAAATCCATTCGAAACCAGCACCCAACCGAGCGCAGTGAGGGT

AACGAACAACCCGACACTCGCCAAGAGGTCGTTTCGGCGATACTCCGGCT

TGATTCGATGGTAATTGTACGCTGCGAGTCCAGGGAGAATACTTCCCAGA

AACGCGACCACTCCCACCTCAAGTCCTAATTGCAGAACAAAATAGAGCGT

GACTACTGGAACGGCTGTGCCGATGACTATCGCTGCAATGAGCTCGTCAC

GACCAAATATCAACGTTCGACGACGGAGGATCCATAGGCCAACGTACGCG

GCTGCCGCGCTCAACACAAAAACGGGGAGCATGACGAAGTTCTTCAAAGA

GTATACAGCAAGAACGGGAATGGTGATTGACCCTCCCATACGATACCCGG

TGAACTGAGTGATAGCCCCGACGCTCAAGAGACCGATCACGGCGACTAGT

GTGGCCACCCACATTGTGACTATCTACCAGCTAATGCACCTTTATAGTTT

CAATTCTGTACGGTCGCTTACTGGAGAGTTTGCTGTACACGTCCGTACAG

TTCGGATTTATTGAAGGGCTTCGCCAGGAAATCGTCCGCGCCTGCCTCGA

GCGCTCGAACGATGTCCTCCTCCCGACTCCGAGATGTCAGCATAATAACC

GGCATATCCTGCATCCCGTCGTTCTCCCGGATACGGTCCAGAACCGAAAA

CCCGTCCATCTCTGGCATCATGACGTCGAGAATGACCGCTTTCGGTAATG

AATCCGGGGACTCTTCTAAGTACTCCCATGCCTCCCGACCATCTTTTACA

GTCTCCACATCGAACTCCTCACTAAGACTAACATCCATCATCTCTCTGAT

GTCTTCGTCGTCGTCGGCAGCCAGAATTTCCGCAGGCATACTAGCAGCGT

ATCCGTCCCGTATTCTTATATTGACCGACATACGAAAATCCCCTCGATTA

GTGCGTTTTTTCAGTCGTCAGCGACCTCGATAGCCCACGAGGGTGTCTCG

CAAGAAATAGTTAACCGGTTAGACAGTCGAATCTCTGTAACTGTGTTCGG

TACTACTGACGAACTGGACGAACCAATCCGCGTTCTCTACGTGAACTCCG

ATCCCGCGTTCGCGGAGTTGGTTCAAACCAAACTCCAACAGACGAGTCCC

GAGATCGACTGTGTTTCTGCCGACGGTATCGACGAAGCACTGCACCGGCT

TGCTTCGGATCGGGTCGACTGTATCGTGACGGCGTATTCGCTCGGAGATT

CCGACGGCATCGGGCTCACGGAGTCGATTCGCCAGGAGAACGACGAGATT

CCGATACTCCTCTTTACCGGAAAAGGCAGTGAGTATATCGCGAGTGAAGC

GACTCGTGCCGGCGTCTCGGACTACATCCCGGTCCGGTCGGAACGTGATA

ACTTCACACTACTCGCGGCCCGTATTCGGACACTCACGACGGCTGCCCGC

AAACGCACGGAGGCCGAGCAGACGAAACGGCGCTTCCGACGAACGCTCGA

ACGCGCAACTGACGCGATTTACGCCGTCGACAGCGACTGGCGCATCGAGT

ATATGAACGAGAAAATGGCCGAGCGAGTCGACCGTGACCCGGACTCGGTC

GTCGGAGAAACCATCTGGGAAGAGTTCCCGTCGGTCGTCGGAACGGAACT

CGAAGACACGTATCGAACGGCAATGGAGACTGGTGAACCAGTATCGTTCG

AACAGTATCTGGAAGAGCCGCTCGACTACTGGGTCGAAGTGCGAGCGTTC

CCAGATGACGACGGGCTGACTGTCTTTTCGCGTGAAATTACTACCGAGCG

AGAACGGGAACTGAAGCTAGAGCGCAGTAACGCGGTTCTGGAGAACATCC

ACGACATCGTGTTCGTCCTCAACGAGCAAGGTGATATCGATTCCGCGAAT

ACAGCTGCAAAGCGGTTACTCGCAGGGGATCCGTCGGCCCAGCTCACAGG

ACAACAGCTCGAAACGGTCGTCGGTGACCGCGTTAGCGATTCCGATATGA

CGCGATTCTCCCAGGCAGTCGAATCGACGCTCGACGAGATCGAGAGCGAT

GGTGGGTCGATGGGATTGTACGATGCAGACCTCCAACTCGACGTCGTGGT

CGGGACCAGCGAGCGGACATTCGACGTCCGGGTAACCCCGTTCCAGAGTC

GAGCGAGTAAGCAGGTCGTCGTTGTCGCTCGAGACGTGACCGAAGAAAGC

GAGGTGAAGCGTCAATTGGAACGAGAGCGGGACGCACTCCAGGATCTCCA

AGCCGTTATGGCAGAAAGCGATGTCTCGATCGAGTCACGCCTCGAGAATC

TCCTTGAAGTCGGTTGTCAGACGCTCGGACTCGAAATCGGCATCATCTCG

CGGATTCAGGACAGCGACTACACGGTCGAAGCAGTTCATGCACCAGAGGC

TGATATCGAAGCTGACGATCAGTTCGATCTCGAATCGACGTACTGTGCGG

AAGTCGTCGGCACGGATTCGGTCTGTTCGTTTGCAGACGCCGTTAGCGCC

GGGAAAGAGACGCATCCTGCCTACCGTGACTTCGAGTTAGAATCGTACAT

CGGCGTACCCCTCATCGTCGATGACACGCGTTACGGAACCGTCAATTTCT

CGAGTCCGACGACGCGAGTCGCGCCGTTTGGAACGCTCGAACGAACGTTC

GTCGAGCTGGTTGCACAACTCGTTAGTACCGAACTCTCGCGTCGTCGTGA

CCGTGCCGAACTCGAACGTCAGGGGTTCCTCTTCGACCGCGTTCAGGACG

TCGCAAACATCGGTATCTGGGAACTCGTTCCATCGAGCGGCGAACTCACC

TGGTCTGATGGGGTCCGCCAGATCCACGGCGTCGACGAAGACTACGAGCC

CTCTCTCGACGGCGCAATCGAGTTCTATCACCCCGACGACAGAGAGACGA

TTACCGAAGCGGTCGACCAGACAATCGAAGACGGGGAACCCTACGACCTC

GACCTTCGAATCGTTCGACCCGACGGCGAGGTGCGTGACGTTCGGGCGTG

GGGGAAATACGTTGAGGACACACCACGGGGTGACCCAGTACTTCGAGGCG

TTTTTCAGGACATCACGGAGCGAGAAGCGGAACGCCGGAAGCATCGAGAA

CTCGCGGAGGAATACGAGGCGTTGCTCGAAACGTCCGGGGACGCGATATT

CTTGCTTGACGTCGATACCGCTGGTGAGAACCCCTCGTTCGAGTTTGCCC

GACTCAGCCCCGGATACGAGTTACAGACCGGAATTACGACCGAGGAAGTT

CGCGGCAAGTCGCCACGAGAGGTGTTCGGCGACGAGCAGGGTGCTGAACT

CGAGACCAAGTACACTCGCTGCGTCGATCAGGGTGCCCCTGTCTCGTATC

GCGAGGAACTCGACATCGGTGACGGTGCTCAGTTCTGGGACACCAGCCTC

GCACCAGTACTCGTCGACAACGAGATTGTTCGAGTCGTGGGCATCGCTCG

AAACGTCACCGAGCAGGTCGAACGAGAGCGCGAACTCGAGGCGACGAACG

AGCGACTGGAATCGCTCATCGGGGCCACGCCACTCACCGTCATGGAAATC

GACACCGATGGCAACGTCATCCGTTGGAACGACGAGGCCGAGAATATGTT

CGGCTGGTCGCGAGACGAAGTGCTTGGTGAGTTCAACCCGATTGTTCCGG

ACGAGCAGCAAGAAGAGTTTGCCTCACACAAGCAGCGCGCCTTGAGTGGC

GAGCGGATTCGGGCGAAGGAGATACAGCGGGAGACAAAAGACGGAGACGA

ACTGGACCTGCTCTTGTCGGTCGCGCCGATAACGGGCTCCGACGGGGAAG

TCACGAGCGTCCTTGCCGTCTTGGAGGACATCACCGAACAGAAACGGTTG

GAAGCTCGCCTCCGCTCGCTTCAGGACACCGCACAGCAGCTGAGCGGCGC

TGAATCAAGCGACGAGATCGGAGCGATTGCCGTCGATGCCGCGGCTGAGA

TACTCGACCTCGGTGTTACCGGAATCTGGGAATACAACGAGCGAGAGAAC

GCGCTCGTCCCGATCACGGAAACGCCTGCAGCACGAGATCTGCTTGGCGA

GTCTCCCCGATTCACTCCTGGAGACAGCCTTGCTTGGGACGCGTTTGAGT

CCGGAGAAACTCAGGTATATGATGATGTCCAGGCCGAGGGGCAACCCCAC

AATTCGAACACAGAGATACGGAGCGAAATTCTCGTCCCACTGGGTGAGTC

TGGACTCATGTCGACAGGGTCAGTTTCCACGAAGGTCTTTTCTGATACGG

ACGTCGACCTGTTCCGGATTCTCGGTGCGACTGTCGAGGCAGCACTCGCA

CGAGCGAACAGGGAAGAGGAACTACGCCGACAGAACGAGCGACTCGACCA

GTTCGCGAGCGTCGTTGCCCACGACCTTCGGAACCCGCTCTCTGTCGCAA

TGGGGTTCCTCGAAATCGCGGAGGAGACCGGGAACGCGGAGCACTTTGAA

AAAGTAGAATCCGCACACGACCGAATTGAACGACTCATCGAGGACCTCCT

GACGCTGGCACGTGGGGAGACGAAGATCGAGAATGCAGGAGAGATCGACC

TCGAGAGTATTACGACAGAAGCATGGGGATATGTAGACACTGAGGAGGCA

ACGCTTACAGTTGCAGATGGAGTTCCGGAAGTTACCGGCGACGCAGGGCG

GTTGACTCAGTTCTTCGAGAATCTCTTCCGGAACGCAGTCGAGCACGGGG

GTGCTGATGTCACGGTGACCGTGGGTGGACTGGACGGAGACGATGGATTC

TACGTTGAGGATACCGGCAGCGGGATTCCCCAGGAGCAGCAAGACGACGT

GTTCAAACACGGCGTCACGTCCAGCGAGGGGGGAACCGGGTTCGGCCTCT

CGATTGTGGCCGACATCGCGAAGGCGCACGGTTGGACAGTTTCGGTGACA

GACGGAAGCAATGGCGGGGCACGGTTCGAGTTCAAGCGGTCGAAATAGTA

CCCGTTGTCAGTGAGCAAACCGTGCCTGCGGCCTATGCATTGAGAGAACC

AGTCTCAACTTATCACATTCTTGGCGCACTTATAAGCCTACAAACGAGAC

TGTCTGATTTCCGAATGGGTAGGCCAAATCAGGGTGCGAAAACCGGGTGA

AGTCGATTCAGCGACCGGACACGAGTCTACTGGTCAACAGATGTCGAGAA

AACGAGTGAAAATCGACAGAGCAGCCGCTACCGAACTCTCAAACCGGAAT

GAGAAAAGCGTTACCCGAACTCACGCAGGAAGTCCCCTAACGCTCGCACG

AAGGTCTTCGCCGTGATTACAGGCGGCGACCGAATCGGCTCGTCGAGCGC

GACCTTGATCAGATAGTCCGTGAGCCGCCACAGGTTGTAGATCAACGTCG

ACAGCGCAAAGTTACAGAATCGAAGCCGATAGTCCGTCGACGACGTCTTC

GGCATGAAGCTCTTGATCGACTTGTACTGGTTCTCGATGTCCCAGCGCCG

ACTGTACCCGTTCACGACACTTCGAATCTTCTCAGGTTCCACGCGGTCCT

GATTCGTCACGAACACCGCGTACTTCCCCTCAGCATCGTCGCTCGTACTC

GGCGCGTAGAGGAACTCCGCTTCGTGGTGAACCTCGCCATCGATTCCCAA

TGGTACGTCGTGCTTGACAGCTGCGTCTGCCGTCGGATGCTCCTGAATAT

CCTGAACCGCCGTCAACTCATCTTCATACTTCGGAACTGGTGAGAGGTAC

GTGATTTCCCGATCGTGGACGTCCGCGTACACACGATTCACGTAGAATCC

TCGGTCGAACATCACCGTGTCCAGATCGACGAACCGCTCGGCGCTGTCCA

ACAGCCGACTCACGAGGTCGGCTTTCGAGTACGATGGCGAGTCGTCGGGT

TCCCATGCGGAGTTCTCTTTCACCGGCTCGATACCGAGAACAATCGGTGC

GTGGTCACCGACCAGTGTGATCGTCGCGTATTTGTATCCACGCTTGTACT

CGCCGTCTTTCTTGTACCCGCTCACCATCTTCGGGTAGTCCGGCTTGGCG

ATACCCGCTTCCTTGTCTTCCCACGGCCAGACGTGGAACTGCTCGTGGGT

GATGTCGATGGCTGCAATCGTCTCACGCGACTCGAAGGGGTTCTGACCGC

GAATCGAGTTGATGATGTTGTCCGTCGCGGCGTCGAACGCTGTCATAATG

GCGTCTCGAATCCGGTCGGTTTCCGGCATTGAGTCGTCGTCCTCGAAATC

CTCGAACGTGAGCTGACTATCCGAGTCCTCTGGAGTAGCAATCTTCTTCA

TCGCCCGCAGGAACGTCGAGTCGTCACAGATGAGGTCATCGTCGGTGAGC

CAGCCGTACTCCGATTCCGAGTGAGCACTCCCCTTGTTCGCACAGATCCG

CGCGAACATATCCAGAATTACCTCGTCGGAGTACGTCTTGTGGGCCGCTC

GGTGCGTATCGAACTCGGGAAGGACGTGTTTTCGAGCGAGCGTCAGCGTC

TTCTGGGCTTTCTCCTTCTTGTACTCGCTCTCGCTCTTCGAAGTCGAATT

CTCAGGTTCCGTCGGAATGGTTGGAACAAGGCCTTCCGTGAGTACGCCGT

GATTGAGCGCGGCCTGTCGAACGCCAGCGACAGTTCTGTTCAGAACCTCT

TGTGTGTTCTCACTGAACTGCGCGTGCGTATACGAGAGCTGTTGCTGGGT

CGGAGTATTCGAGAGATCGGTGGTATCGAGGTGGAAACTCTTGACCAAGC

TCGGCTCCCGCCCGAGTCGTTGGGCTAATTCGTTCTGTGAGATACCCCGA

ATCCCTTTGAACAGGAGCGTGCGGAACATCTCATCGGTTCCAAACGTCAC

GCGGTTCTGATCGCGAGCATCCTCGAGGTTGTCGACGGGGATCGATAGCT

GACGAATCACGTCCCAGAGGTGGTCTTCACGCTCACAGAGCGTCTCCGCG

TGGACAATGATTGAGTCAGCAACAGCCGACGGGTCAGTCAGCATTGAACC

ACCTCGGGGGTGAACTGATCTGCTTCTGAGGTGTGCTCGACCAAATACTG

ATGTAATTCTGTGTAGAGACATCCGCTATCCAAGAACAGAGATCAGTATA

GAATAGAGTGGAAATAGTGTAGAGCATTTTGCGATGATTGTATGCTCTGA

GCTTATTGTGGGAGATACATCAAGGGGTATGCGCGCTATACTTGGAGAAT

TAGTTGTGGCCATAATTCGGTATGGGTCAAGAATCAGTATGAATCAATTT

TCGGTTGGATTTATAGTAACGTGCGAGAAGAAGGAGGTACGATGGGCTTG

GACGAACTTGAGGGGTTTCTTCGGAAGAAGGGAGCGGCAGAACTCATTAC

GGAGATTGGGACGGGAACAGCGACATTCAACGCGCTCGTTGACGCGGTTG

CAGTAAGTGGCTCGACAGTTTCCTCGCGACTCTCAGAAGGTGTTGAGCGC

GAAGTGCTCACGGTCAGCCATAAGCCAACCGAACACGGGACAGAGAAGCG

GTATGCGCTCACGATTCTTGGACGCCGTATCTACGATTGGGCAGCGCAAA

CGGAATTCGAACGGAAGGTCCGGAAGCTTCGCCGGGTTCAGCACGAACGT

GAGACGGCGTTCGAGCGGATGGTTGGGAAGCTCAATCGAGATATGGAGAT

TCGCAAGATGGTCACCGATTCGGAACCCGTAGAGGATCAAGAGATTGATC

TGCCTGAGGGGGCGTCTTTGGTGCCGAAGATGGCGTCAGAAGAGAAGCTT

CGTGAAGCCAAACACGAACGTATGGAAGCGAATCTCAAGCCAATCGAAGA

ACTCGAGGACGCCGGTGGAACAGGAGAGGACAGCGACTGAGACAAACGAT

CTATAGCGGCTCCGCTGCCTCTTGTGAAATCCTCAGCCACAAATGGACCT

GTTCCAGCTGCCGTTCAGGGCAGGAAACACACCAGACGCCTCATCCACCG

TCTCATGGCTCATGAGTCACGAGATGATCTATATTTTAAGTAGTTTATCA

AGATACCAACTTCTTCTTATAATACTAAACCTAACTAAACGGACAGAGAG

AATATTCTCAAGGGGCTTTATTTGAATCCTCAATAACCAACAATTCTGGG

CTCTCGACCGCTAAGCCCATCAAGCGAACATTTTGAACGGTCAATCTTGA

GTCCTTGGTTAACTCTGAAATTGAAGGTCGTTCAATGACTCCAGCTCGGA

CACCGAATTAAGTTAGACCCAGTCCCCGCCGAAATTACACCAACAGCAGA

TTTGCTCAATTTCGCCACTAGCCGCCATTTCAACTAGCAAACCAATACAA

GCGACGCAAGTACCTGGCCATGCTGCTATGGCTGGTACCCATGCGTTCTA

GTTTAGTAGCGATCAAAGCCGAACTCACCTCGTTCAACCTGCTCACGTAA

CCGATCTCTGTGTGGATTTGGATTTCGTGACGCCCACAGCAACAGCTCGT

CCAAATCCGGCAGTTCGTAGCCCAACTCGATCATGAGATACAACTGGATT

AGATGACCGTGGCGTTCCACGGCGAGCGAACAGCGACGCCGAGGGAGCCG

CGACGCCGACGAGTCGGCGTCTGCGGCGGCTTCGCCCTCTCGCTGTCTTG

CCTCGAGCGACCGTAACTCATCCACGATTACACGACTCATCATCAACGAA

ATTGCGGCCATGATGATCAGCGCCTCGATGATGTAGCCGTCGGTCGTCTT

GATCTCGTCCAAGCCGAACCGCGACTTCAGCTCCTTGAACAGCAGTTCGA

CCTCCCAGCGCGCCCGATAGAGCTGCGCGATATCGGGCGCGCTGTAGTCC

TCTCTCGCCAGATTCGTCAGATACAGATGGTACTCGTCGGTCTCCTCGTT

GCGCAGGCCGACCAGTCGGAACGTCCGGGTCGCGCTGGCGCCCGACCCTC

GTTTGCGCTCGAATGAAAGCGTGATGCGGACGTCGATTTCCTGTCGCTGC

AGGTCCTCAAGGACGGCCTGCAGCGACTCTCCTTCCAGCGGAATGCTGTT

GCCTCGCCACGTTCGCAGTTCTTCGACGATCTCGAAGTTCGCGTTGTCCT

TCACCCGGGAGACGAACCACCCGCCGTTCTGGTCGATTCGGTCGAACAAC

CAGAAGTCGTAGAAGCCTAAATCAAGCAAGATGAGGGCGTCAGCTACCCA

CTCACCGGTGGGTAGCTGACTCCGTTCGTGAGTTGTTCCGTCGGTTGTGC

GGAATCGCGTTGGAAGGCCCGTCGAGAGTGACTCTGTGAGGTGGAGCTTC

AGTTCGGCTTGATCTTCGCCGGTTGCTGCGTAGACATCAGCGGCGTCTTG

ATACAGCGAAACGATGGTTGCGTCAGCAATGAGGACGTCTCGAAAGCGTT

CGAGACGTCCGTTCAAGTCTGCTCGTCCGGTATCGAGATTCTCGATGGCG

TCATCGAGAATCTCTCGGAGGAGTGCAACGAATCCCGGTTCGAACCAGTC

GTGAAACGCCGCGTAGGAGAGCTCATCGCAGTCAGCCATCTCGACGTAGC

GGTCGAGAAACGCCTGCAAAGAGCGGTCTGAGCCAGCAGCGAAGCCAAAC

GAGAGTGTGTAGAACAGTGCAACGATGTCGAACTTGCGCTCACGCTCGAT

GAGATTCGTTGCGCGAGCGCGCTCGCGCAACTCATCGGAGGGAAACGCTC

TTTGAATCCGGTCAACAACTACCGAGTCCGGTGGTGAGTACACATCATCC

ACCGGGTTCTTCAACCGGGTAGTTGCGACGATATCTAATCAACAAACAGC

GACCGCATTCTCTGCTAAACTAGAACGCATGACTGGATACCCATGGATGA

GGCCTATAACTTTCGGTGAATTGAGAGGAATTAGATGAGGACCAAAGTCA

ATCCAGAGACCTACTGTGACAGTCTTCACCCTTTCAGAGAACAAACACCC

TCACTTTTCCACCTAAGCGAGGTTATCCGGACAACCACACCAATCAGCGA

TGTTCAGAAGCTCGTCACATCGGTTCAATGTCGTTCGTGTGAACGATCAG

TGAACTCGACACTATCGCTCGGTGTATTCACTGCCGACTGAGCTAGAGAG

CCGACAAACTGAGGACACAGAAGAAGTAGCACACAGAGGAAGACAGAGTG

TTCTAAGGTCCCCCTTCGTTGGAATCACTAGTCGAGTCAACGGATACACC

CTTGGCCAAAACTAGTGGAATTCTACATCCCTGAATTCGAGGGTGAACTA

GATTCCGGGCTCTACACCCATGGTCTACAGGGGTGTACCCACGTCGATTT

TCCGCAAGGGTGTACGCAGATCGGAACCAATCGAGGGTGAAAACGAATCA

CTATCGGCCCGATTTATTCGACAGTACTGATTTCTAATTTGCTTGTGTTT

ACCCACAGGAGTCCCGCTACTGCACGAATCTCATCCACATACAACACTGG

CCAATGGAAACATCACCTCCTGTAATGCTCACCAAACACAACGTCAATAC

GCTAATTCGTCAGTACACCCTCTCGTTGGTGGGGTGTATCGACTGTATTC

GATTGCCGTCAACGCCGAAATTGGGTGGGGGTTGACGACTGCTCGTATTG

GGTTTTTCGACGACCACCCATCTTGTGCAATGGCGCTCCTGAGCGTACTC

TGGGGGGCTTCTCGACGTGACACACGGGTTGTGTCTCTCGAATGGAGCGT

GTGCTTTTGCTCGTTGATTTTGAGTTGCCCCAATTGAATCGACGAGTAGA

TTGTATTCCCGGGGGATTTCGAGGGTGTAGTTTGGTACTGTCGGACTCGG

AGTTCCAACGAGGCTGGCTAGAGAGGATGAGCTGGCTTTGAGTTCAGCTG

TCGACGAGACGGCGCGATGGTGCAAGCGCGTACGTGGTGAGTCCTCTCGA

AGACCAAGGAATCGAGGCGGTGACTCTCGACCAAAGAATGAACGCGGCCA

CCGACAGAAATCCCCGTCGCCGTCCGCGAAGCGAGGGACGGCAGACCAGG

TTACGACTTGCGCGGGTGCGGGTCGAATGCGTCCGTCTCGACTGGCGAGG

CGCACAACACGCACCCGTCGTCCAGAATCCGCTCGCGGATGCCCGCGTCG

ACACACACCTCGACGCTGCACACCGGGCAGTCGAAGACGAACTGACCTGG

CACGGCGATCACGACCGAAGCACAGACCCCAATCAGTATCTATCACACCC

CATCATAGGGCGGGAATACATAGGCGAGTTATCAACTACACGTCGAGAAT

CGTATCGAGAAGCTTCGACTGGGCGGCCGAAAGGTGTTCGGTGAACGTCG

AGCGGTTGATATCGAGCGCCGCGGCGACCTCCGTCGCGTTCGCCTCACGG

GGGTGCTCGAAGTAACCCATCTCGTGGGCCGTCTCGAGGACTTCCCGCTG

TCTCGCCGTCAGTTCGTCGCGGTCGACGAACACGAGCGAGCCGGTAGCGG

AGTCCGGCTCTGACTGCGTGAGTCGCCGGAGTGACACGCCGTCGAACGCC

GATTTGAGGTTGACGACGATGTCGCGGAACGACTCGAGGTCCGCGGCGAC

GAACGACAGCACGACCGCGCCGCAGTCCGCTTCGAGGTGGCGGACTGGAC

AGCCGTGACCCTCGATGAGTTCGCAGGCACACCGCGACTCCTCGGTGAAA

GAGTGGGTGACGCGATAAATCGTCTTCTGTTCGTGGTGAAATATCTCGTC

CGCGTCATCAATCGCGGCCGACGAGGGGGCGGTAAACTCGACTGCGGCGG

TATCGCCGGTAGCGCCGACCGACCGCGAGACGGATTCGACTGCGGTCTCC

TTCGACGCCTCCGACACCCGACAGATGGACGGGTCGGCCACGTGGATTTC

ACCGTGCACGCCCTCATTCATAGGCCGGCGTACGGTGGTGGTTCTGAAAT

ATTCTTCGGCGAATCCGTTCGCAGTCACGCGGTTTCGCTTCGGATTCCCT

CCGATTCCTCGACGAGGGCGGTATAAAGTCCCCGCACCCTATGGTTCGGG

TGTTACTGGCCTCGGTGCAGATTTTCGAACGAATCGAAGTATGCTATATA

CGAGCTTGCTCGGGGCCAAACGGGTGAACGGATCGGCGCCCGGTTGGAGT

GTGAGGCGGCGCGTCGGTACGCGGGGTGAGTCACCGTGAGACGCGGGACG

GTCGTCGTGTTGCTGCTCGTCGTCGCGGTGCTCCTGCCGACGTGGTACGT

GGCGCTGCACGGCGAGCCGCCGAGCGAACAGATAGAAATCGACCAGAGCG

TCACCGAGATGCGGCCGCTACAAGAGATCGTCGACACGCCGGAGAAGCTC

GCGCCGAGTCAGGTGGGCGTCATCGTGTGGGTCGCGCTCTTCGCGCTCGT

CGGCGTCCTCGCCGCAGTCCACCGGTTCATGGACACGGCGGTCAGAGCCG

GCCCGAGCGACGAACCGACCGTCGCAGACGGTGGTCGGGTCGGGCTTCCG

TGGTTCTGGACGGCGGACCGCTGGGTCGTCGAGTACGACGACGCCTCCGA

CGCTATCGAGGGAATCGTGGCGATGGGCGGGCTGACCATCCTCGCTATCG

TCTTTGCGGCGCTGTTCACCGGCGAGTATCTGACGCTCGCGCGGACCCAG

TACTTCGGCGTCTACGCCGCGGGGATGTTCACCGCGCTCGCTTTGCTCAC

CGTATCGTACTACGCGTGGTTCCTCCCCCACGTCGAGGTCGCAGAGCGGA

GGGGACACTAATGAGACTCGACAATCAGCACGCATCGGACGACTGCGACG

AGCACTGCGCTGACTGCGGGGACGACAGCAACCACCAGCCGAGTATCTTC

TCCGACGACCGCGCCTCGCTCGCCCGCCGCGACTACGCGAAACTGCTCGC

CAGCGTCGGGGGATTGACCGCCGTCGCGAGCCTCGCGGCACCGTTGGCGG

GGCTGACTCGCGTCTTCGAGCGTTCGTACACCGGCCCCGTGTACTCGGAC

GGCATCTACCTCGTCGACGGCGACGGCGAGCGCATCGGCGAGAACGCCCT

CAGTGAAGGTGAGAAGATGACCGTGTTCCCGGAGCCCCGGCCGGGCATCG

AAAAGGCACCGACGCTGCTCGTGAGACACGCGGAGGACGCCTACGGTGGC

GCAACGAACCTTGAGTACACGGTGTCGGGCTACGCCGCCTACTCGAAAGT

CTGCACCCACGCCGGCTGCATGGTGTCGAACGAGGACGGAGCGACGCTCG

TCTGCCCGTGTCACTTCGGCAAGTTCGACCCGACCGCCGGCGCGAAGGTG

GTCGGTGGGCCGCCGTCGAGGGCGCTCCCGCAACTGCCAATCACGTTGTC

GAGCGAGGGCAATCTCATCGCGACGGGCGACTTCAACGGGCCGGTCGGCC

CGGGGGGCGAGTGATGTCTCGACTCGACCGCCTCGCCGACCGCGCCAACG

ACGCCGGCGACGAGGCGTACGACTGGGTAGACGAGCGGTTCGACCTCGAC

ACCGGCCGCGCGTTCCTCGGGAAGGCGTTCCCCGCCGAGGACTCCTTTCT

CCTCGGCGAGGTGGCGCTGTTCTGCTTCCTGCTTCTCGTCCTCACCGGGA

TATTCCTCGGGATGTTCTTCGAGCCGTCGACGAGCGGCGTCGAATACGAG

GGGAGCGTCGCGAAGTTCCAAGGCCAAGAGGTGCCCGAGGCGTTCGCGAG

CGTCCTGCACATCACCTACGACATCCCCTTCGGCATGTTCATCCGGCGGA

TGCACCACTGGGCGGCTCACCTGTTCGTCGCGTCCATCGGGCTGCACATG

CTCAGGGTGTTTTTCACCGGGGCGTACCGCAATCCGCGCGAGCCGAACTG

GGTCGTCGGCACCGGCCTAGCGGGCCTCTCTATGGGCGCGGCCTACACGG

GGTACGCGCTTCCCTTCGACGAGTTCGCCGCGACGGCAACCGGCATCGGC

TACAACCTCGCCACGTCGATTCCGCTCGTCGGAGGCGTGGTCGGGAAGGC

GTTCTTCGGCGGGGAGTTCCCGTCGAGCGCGACGATACCGCGGCTCTACT

TCCTCCACGTCCTCGTCATTCCCGTGGCAATCGGGGGGCTCCTCGCCGTC

CACATGGCGATTCTCGTCCGCCAAAAGCACACGGAGGCCCCCCGCGATGA

CGACGTGCGAGGACTCGAAAGCCGGGACCGACGCCCGGTCGAGGGGTCCC

CGTCGAGCGACGGCGGCACCGAACAGACCGTCTCGAGAGACGACGACAGC

GTCGTCGTCGGCCTCCCGGCGTTCCCCAATCAGGCGGCCGTGAGCGCGGT

CGTGTTCTTCCTCACGCTGGCCACGCTGGCGATGCTGGCCGGGTTCCTCC

CGGTCCACAACATCGCCGAGTACGGGCCGAACAACCCCGCTGGGACGCCG

GAACTCATCATGCCCGACTGGTTCCTGATGTGGGTCTACGGCCTGCTGAA

ACTGCTCCCGTCGTGGATGGGATTCACCGTTCCGCTCACCGACATCCACG

TCTCGACGGAGTTCGTCGGCGGCGTCCTGCTGCCGACGCTCGTGTTCGGC

GCGGTGGCGGTGTGGCCGTTCGTCGACTACCACGACGAATCGGTCCACTT

CACGGCCGACCCACTCGACCGGCCGTGGCAGACCGCCGTCGGCGTCGCCG

CGATTCAGTTCATCATGATTGCCTCCATCGCGGGCATGAACAACCTCCTC

GGCCGGGCGCTCGGCGTCGGAACCGGCGTCGTCAATCCGATTTTGACCGT

CGCGTTGTTCGTCGTCCCCATCGGCTCCGCAGCCCTCGTCTACCGCATGC

TGGGCGACGACGCGGACGAGAGCCGAACCGGACGCGTTCCGGGGGAAGCC

GGCGATGACTGACGCGAACACCGGCGGGGCTCCGCGGCGACGCATCGAAC

TCTCGCCTCGGACGTACCGACACCTCGACCGCGCGAGTAAGCTCCTCGGC

GTGGCGCTCGTCGCCGTCGGCCTCGACGCGGGTGGTGACACGCTCGTGGG

GATTTCGCTCGGCGTTCTGGGCGCCGGGCTGGCACTAACGACCGTCTTTT

TACAGATAGATTCATGAACCGAACGAACACCTCGACCCCCGACGACGAGT

CGGGAACGGACGAATCGCAAGCCGAACAGACGGACGACCCGCCGGCGGTC

GGTGACCCGCCGGGAGAGCCGGCGACCGAGGGAACGAGCGTCTCCCGTCG

TCGGTTCCTCGAAGGCGTCGGAATCGCCTCGCTTCTCGGCATCGGCCGGT

CCGCTGCCGACGACGACCCGCTGTTCCAGATGGGCGGGCTGAACCCCGTC

GACGACCCCATCGGGAACTACCCCTACCGGGACTGGGAGGACCTCTACCG

CGAGCAATGGGACTGGGACTCGGTCTCTCGGTCGACGCATAGCGTCAACT

GTACCGGCAGCTGCTCGTGGGACGTCTACGTGAAAAACGGGCAGGTGTGG

CGCGAGGAGCAGGCCGGTGACTACCCGCGGTTCGACGAGAGCCTGCCCGA

CCCGAACCCCCGCGGCTGTCAGAAAGGGGCGTGTTACACCGACTACGTCA

ACGCGGACGAGCGAATCAAACACCCGCTGAAGCGCGTTGGCGACCGCGGC

GAGGGGAAGTGGCGGCGCATCTCGTGGGACGAGGCGCTCACCGAAATCGC

CGAGCACGTCGTCGACGAGGTGCAGGCCGGTCGCTACGACGCCATCAGCG

GCTTCACTCCCATCCCGGCGATGAGCCCCGTCTCGTTCGCCAGCGGGTCG

CGGCTCATCAACCTCCTCGGCGGCGTCAGCCACTCGTTTTACGACTGGTA

CTCCGACCTGCCGCCGGGCCAGCCCATCACGTGGGGCACCCAGACCGACA

ACGCCGAGAGCGCCGACTGGTACAACGCCGACTACATCATCGCGTGGGGG

TCGAACATCAACGTCACCCGCATCCCCGACGCGAAGTACTTCCTCGAATC

GGGCTACAACGGGACGAAGCGCGTCGGCGTCTTCACCGACTACTCGCAGA

CGGCCATCCACACCGACGAGTGGCTCAGCCCCGATTCGGGCACCGACACC

GCGCTCGCGCTCGGTATGGCCCGGACCATCGTCGACGAGGGACTGTACGA

CGAGGCCCACCTCAAAGAGCAGACCGACATGCCGCTTTTGGTCCGGCAGG

ACACCGGGAAGTTCCTCCGCGCGAGCGAGGTTCCCTCGGTCTCGACCGAC

GCCGACCGACCCGAGTGGATGTTGCTCATGCTCGACTCTGACGGACGCCT

CCGCGAGGCACCGGGGTCGCTCGGTGAACGCGACGGCCAGAAAGACTACT

CCAAGAGCATCGAACTCGACTTCGACCCGCAACTGGACGGCGAAACCACG

GTCCGAACCGAAGACGGGCGCGTCCGCGTCCGGACCGTGTGGGCCGAACT

GCGGGACGAACTCGATGACTGGGGCCCGGAGACCGTCCACGACGAGACCG

GCGTCGGCGAAGAGACCTACCAGCGCGTCGCCCGCGAGTTCGCCGAGGCC

GACAAGGCGAAGATAATCCAGGGCAAGGGCGTCAACGACTGGTACCACAA

CGACCTCGGGAACCGCGCGCTCCAGTTGCTCGTCACGCTGACGGGCAACC

TCGGCGAGCAGGGGACCGGCCTCGACCACTACGTCGGCCAAGAGAAGATA

TGGACGTTCCACGGCTGGAAGTCGCTGTCGTTCCCGACGGGCAACGTCCG

CGGGGTGCCGACGACGCTGTGGACCTACTACCACGCGGGCATCCTCGACA

ACACCGACGCCGACACGGCCGCGAAGATACGGGAGTCCATCGACGAGGGA

TGGATGCCGGTCTACCCCGAGGAACGCGACGACGGCTCTCGTCCCGACCC

GACGACCATGTTCGTCTGGCGCGGCAACTACTTCAACCAGGCGAAGGGGA

ACGTCGCCGTCGAGGAGGAACTGTGGCCGAAACTCGACCTCGTCGTCGAC

ATCAACTTCCGGATGGACTCGACGGCGCTGTACTCGGACATCGTGTTGCC

GACCGCGAGCCACTACGAGAAACACGACCTCTCGATGACGGACATGCACA

GCTACGTGCATCCCTTCACGCCCGCGGTCGAACCGCTGGGGGAGTCGAAG

ACCGACTGGCAGATATTCCGCGAACTCGCCGCGAAGATTCAGGAGGTCGC

CACGGAGCGCGGCGTCGGGCCGATTTCGGACCGGAAGTTCGACCGCGACA

TCGACCTCCAGTCCGTCTACGACGACTACGTCCGCGACTGGGAGACCGGC

GAAGCCGACGCGCTCGACGAGGACCGCGCCGCCTGCGAGTACATCCTCGA

ACACTCCGAGGAATCGAATCCGTCCGAGAGCGACGACCAGATAACGTTCG

CCGACACGGTCGAACAACCCCAGCGGCTCTTGGCCGCCGGCGACCACTGG

ACCTCCGACATCGAGGACGGGGCGGCGTACACGCCGTGGAAGGACTTCGT

CCAGGACAAAAACCCGTGGCCGACCGTCACCGGCAGACAGCAGTACTACA

TCGACCACGACTGGTTCCTCGAACTCGGCGAGCAGTTGCCGACGCACAAA

GAGGGGCCGACGCGGACGGGCGGCGACTATCCGATGGAGTACAACACGCC

CCACGGGCGGTGGTCTATCCACTCGACTTGGCGAGACAACGAAAAACTGC

TCCGCCTCCAGCGCGGGGAGCCGGTGGTCTACCTCCATCCCGAGGACGCC

GCCGAGCGCGGCATCGAAGACGGCGACACCGTCGAGGTGTTCAACGACCT

CGCGGAGGTCGAACTGCAGGCGAAGCTCTACCCGAGCAGCCAGCGCGGCA

CGGCGCGGATGTACTTCGCCTGGGAGCGGTTCCAGTTCGACGGCGACACG

AACTTCAACTCGCTCGTCCCGATGTACATGAAGCCGACGCAGTTGGTCCA

GTATCCGGAAGACTCCGGCGAACACCTCTACTTCTTCCCCAACTACTGGG

GGCCGACGGGGGTCAACAGCGACGTCCGCGTCGACGTGCGGAAAGGCGGG

GGTGATGCCGAATGAGCACCGACGACGGGCCAGCAGCACAGACCGACGGC

GACGGGGTCGACCTCGCCGACGGTGTCGACCATCAGGTCGCCATGGTGAT

GGACCTGAACAAGTGCATCGGCTGTCAGACCTGCACGGTCGCGTGCAAGT

CGCTCTGGACGGAGGGCGGCGGCCGCGACTACATGTACTGGAACAACGTC

GAGACGAAGCCCGGGAAGGGCTACCCGCGCGACTGGGAGGACTCCGGCGG

CGGTTGGACGGACGCCGAACACACCGAGCGAAGCCCCGGCGACATCCCCG

ACCAAGCGGACTACGGTGACGCGTGGTCGTTCAACCACGAGGAGGTCATG

TACAACGGCGGCGACTGGCCGCTGCGTCCCGACAGCGACCCCGAGTGGGG

GCCGAACTGGGACGAAGACCAAGGCGCGGGCGAGTATCCGAACTCGTACT

ACTTCTACCTCCCGCGCATCTGCAACCACTGCACGCACCCGTCCTGCGTG

GAGGCGTGCCCGCGGAAGGCCATCTACAAGCGCGAGGAGGACGGCATCGT

CCTCATCGACCAGGAGCGCTGCCGCGGCTACCGCTACTGCGTCGAGGGTT

GTCCGTACAAGAAGGTGTACTACAACGCGACGCAGAAGACCTCCGAGAAG

TGCATCTTCTGTTACCCCCGCATCGAGGGCGAGGGCCCCGACGGCGAGAC

GTTCGCCCCCGCCTGCGCGGAGGACTGCCCGCCGCAGTTGCGACTCGTCG

GGTTCCTCGACGACGAACAGGGGCCGATTCACAAGCTCGTCGAGGAGTAC

GAGGTCGCACTCCCGCTGCATCCGGAGTACCAGACGCAGCCGAACGTCTA

CTACATCCCGCCGTTCGCGCCGCCGCAGCACTCCGAAGACGGCGAGACGG

TGGACGTGGACCGCATCCCGCGGAACTACCTCGAAGAGCTGTTCGGCGAC

CGCGTCCACGACGCGCTCGACACCATCGAGAAGGAACGCGACAAGGTCGA

CCGCGGCGGGGAAAGCGAACTGCTGGATATGCTGACCGACACCAACCCGG

CCCGAAAGTACCGGTTGGAGGTGTTCGACTGATGCGAGCAACGCGAGCGA

AGTCGGGCTCGCCGGGACTCGTTCCGGAGGTGTTCGACGATGAGTGAACG

TCGAACGGCCGCAGTCGCCGCAGTCGCCGTCGTGGTGCTGCTCCTGCTCG

TCGCGGTGGCCGGCCCCGCGGCGGTGTCCGCGCGCCCCGCGAACGAGATT

CCCGTCCAATCGGTCGCTGCCGACGACCGACCCCAGCGGCCCACGAGCGA

GGCGTGGAACACCGTTCCGTCGGTGAACGTCCCGCTGACGAGCGCGCCCA

GTGGCGTTCCGAACGCGAGCGACACCTCCGTCGAATCGGTTCGCGTCCAG

TCGGCCCAGACCGACGAGCGGTTGTATCTCCGACTCTCGTGGGCCGACGG

AACCGCCGACCGCAACGCCACCAGTCCGAGGTCGTTCCTCGACGCGGCCG

CGGTGCAGGTACCGGTCAACACCTCGGTCCGCCCCCCGATTTCGATGGGG

AGCACGCGGAACCTTGTGAACGTCTGGTACTGGAGCGCCGACGGGGAGAC

CGAGGAACTGCTCGCCGGCGGCCCGGGGTCCACGACCGAGTTCGAACGGA

CGGCGGTGGAGACGACGGCGAGCCACGACGACGGCCGGTGGACGGTCGTC

ATGTCGCGCCCGCTCGATTCGGATGCCGCCAACCGGACCTCGTTCGCCGT

CGACAACGACGTGGACGTGGCGTTCGCGGTGTGGAACGGCTCCGAGATGG

AGCGCTCCGGCCGGAAGTCGGTCAGCGAGTGGTACCACTTCCCGTTCGGC

CCCGGACCGCAGGGGCCGCCGTACGAGTCGATACTGTGGACCGTCGCCGG

CCTCGCCATCGTCGGCGTCGCGCTCGTGACTATCGAGGCGGTGCGGAAGA

ACTGAGGACACGACGATGGACACGAACACCAACGCGGAGACGCGAACTGA

CGACACGAACCGCGACACCGACCGAGTCGATTCGAGCGGGGTCGACCGAC

CCGCGGCGGCGCAGGCGGCGATCTATACCGCGCTGGCGACGTTGCTCGAC

GAGCCAGACGAGCGGCTCCACGAGCGACTCGCCGCCGGCGAAGTCGACGA

GACCGTCCGGGGGCTCCTCGATGCGACCGGACTCTCGGTCGCCCCGCCGG

AACTGACCGTCGACGACGACTACGAGACGGCCTGCGCCCGGTTCAACGAC

CTGTTCACGGTGGGCTACGCCGAGTACGCGGACCGGACGGACGGCTCGCT

CGACAGCGAGGGGCCGCGTATCTCGCTGTACGAGTCGTCGTACCGGCCGG

ACGCGGCGTGGAACGACGTGAATCTCGACCTCGCACGCGCCTACGACTAC

TTCGGGCTCGAAATCGACCGAGAGGCCCGCGACAACCACGACTACCTCCC

GTACGAACTGGAGTTCGCGAGCTACCTCGCGCGGCTGGAGGCCGCGGCCG

ACCCGGGTGACGGGCGCGCCGACGACGCGGCGCGGGCACGGCTCGATTTC

CACGACCGTCACCTCCACGTCGTCGCCGGGGGCTTGGCTGAACGAGCGGC

CGACACGCCCGGTACGGGTCTGTACGGCGAAGTCGCGAGCTTCCTCGACC

GGTTCGTCGCCGCCGACCAGCGCGCGCTCGCCGCGCGTTTCGAGGGAGGT

GACGGACCGTGACCGGTCGGGGCGTCGAGGACGCCGTCGGCTCGAATCCA

CGGCCGTCCGGAGTCGCCGCGCTCCGGTCTCGAACGCCCGACGGGGTGCT

TCGAACACTAGCAGGCGTCCTCGCAGTCGTCCCGCTCGCCGCGGTGACGG

CGTACCGCGTCGGCCACAACGTTCCCGGCGGTCTCCCGGCGGGCGTCACG

ACGCTTGCGGCCGACTGGTCGGCGCTGGCCGTGGTCGGTCCCGCGTTCGC

GGGGCTGCTCCTCGCGGCGACCGCCGACAGTAAAGTCGAGCGCGTGGGAC

TCGCCTTCGCCGGCGGATTTGGAGTCCTGGCACTCGGGACAGCGGCCGCG

GCGTGGCAGCCGGCTGCAATCGGCGTTTCGGTCGGGGTCGCGGTCGTCGC

GGCGGACCGGTTCGTCGCGCCCGGACGGAAACGAGAGTGGAACGGAGCCC

GACGAGCCGCGCCGGTCGGCTTCGCCGCGGTCGGTGTGGCGACCTCGCTC

GCGGCGGCCGCCGGCGTGTGGCCGGCGACGCTCCGACCGCTCGGCTCCGG

GGTCGCGCTCGCCGCGGTCGGCGTCGTCCCACTCGCTGTCGGCTGGGATA

GAATCAGTGCGCTAGCCGGCATAACCGCGGGACTCGCCACATTCGGAATC

GTCGCAAGCGCCCCGTACGTCGCGGGTGCGGTACTGCTCGTCGGCGGCGG

TGTCGTCGGCGTTCCGACGAGTCTCGTCGCCTTCGCCGCGGCCGGCGGAA

CCGCCGGCGCCGTCTCCGCGCTCCGCGACGGTCGGCCCGCCGTCGCGCTC

GGAGCCGCGTTGTTCTGCGTGGCCGGCGTCCCCGCGACAGTGCTCCGAGC

AACCGGGGTCGTCGTCGCGGCTGCGTTGGTCGCGTACGACGGGGGTGAGC

GAGCGTGAGAGACGAAGAGGAGGCGACCGACGGGCCGCCGGACCCGCAGC

TACACCCCGAACAGAGCCCCGGATTCGGTGTCGACCCTGTGGGCCTCGAA

GACATCGAAGTCGACCGCGACGTGACCATCGGCGAGGCGACGCTCGCCGA

CCTCGGCGCAAGCGACACGGAGCCCGTCGAGGACCACCCGGTCTCTGACC

TGCTCGCGTCGCTCGCCGGTGACGGCGCGGTCGAGCGTCGGCGGGCCGCA

CTCGCGCTCGCCGAACGGGAGGGCGACAGGGTCGTTGTGGAAGCGTTGTC

CCGCGCGGCGACGACAGACGAGGACGCCGAAGTGCGGCAGTTCGCCGTGG

AGGCGCTGGCGAAACACGGCGGCGACCTCGCGGCCGAGACCGCGCGGGCG

CTGACCGACGACCCCGACCCGTGGGTTCGGGCCGAGGCCGTCGTCGCGCT

CGACCGCCTGGACCGGGGAGAACACGAAGCGGTCATCGAAGCCGCACTTG

ACGACGAGCACCACGCCGCCCGGCGAAACGCGCTCGTCTCGCTTTTCAAA

CTCCGCGGCGAGGGCGCGTGTGACGCGCTCGTTGCCGCCGCCGACGACCC

GAGCGAGCGGGTTCGAGAGTGGGCGGCGCACCTGCTCGGCGGGGTCGAAA

CCGACCGGGCGGCCGAGGCGCTGTCCCGACTCGCCGACGACGAACAGAGC

GTCGTGCGAGAAACGGCGGTTCGGGCCCAAGAGGTCGATTCCGGGAGCTT

CCGACGCCAGTTCACGGGCGTCCTCGACGAGACCGACCGCACCCTGCCCG

GCGAAGACGACCTCAACCGAACGCCGAACCTCTGACCATGAGCGAACACG

AACCGACGAACCACGAACCGGCGAACGACGAGCCAGACACCGAGCCCGAG

CTTCGAGACCGCGCCGAATCGGCGCTCCGAGCCGTCCGAGACCCCGATGC

CGACCTCGACGTTTTCGAGGCCGGACTGGTCGAATCGATAACCGTCGACG

GGGCGAGCGTGACCGTCCGCGCGGCCGTGACGGAGTTCGACGACGCGAAC

GCGACGCAGGTCATGCGCGCGATGGCGCAGGCGGTCCGCGACGTACCCGC

GGTCGAGAGTGCGCACGTCGAACCCGTCTCGCCGTCGTCCGGCGGCGGTG

CAACGGGCGTGGACGCGTTCGATACCGTCATCGCCGTCGCCAGTGCCAAG

GGCGGCGTCGGGAAGTCGACCGTCTCGACCGGCTTGGCGTGCGCACTCGC

CGGCGAGCACTCAGCCGGGCTGTTCGACGCGGACATCCACGGCCCGAACG

TCCCGTCATTGCTCGACGTCGAAGGTCCCGTCCACTCGGACGACGAGGGC

CACCCGCTTCCCGTCTCGGTGGCGGGGCCCGACGCCTCGCTCGACGTAAT

GAGCGTCGGATTGATGGAGTCGGGCGCGCCGCTGGCGTGGCGGGGCGCGA

TGGCGCACGACGCGCTGACCGAACTGTTCGCGGACACCGCTTGGAGCGCC

GACGACACGCTCGTCTTGGACCTTCCGCCGGGGACCGGCGACGTGGTTCT

GACGACGCTTCAAGAGATTTCCGTCGACGGCGTCGTCGTTGTGACGACGC

CCTTCGAGTCGAGCCTGGAGGACACGGCGCGGAGCATCGAACTCTTCCGG

GACAACGAGGTTCCGGTTCTCGGGGCGGTCGTCAATATGCGCGAGTTCGC

CTGCCCCTCCTGCGGCGACACTCACCGGCTCTTTCCCGGTGAGGCCGCCA

GCGAACGGCTTGACGCGACGGTGCTCGCCGAGCTACCGTTCTCCCCGCAG

TTTCAGGAGACGCCCGCGCCCGGCGACGCCGCACCGGCGTTCGAGACACT

CGCGGAGTCGGTCTCCGAGGCGGCGGCGACCGCGTGGGATGTCGACGCTC

CCGACGGCGCACTCGACATCCGCGGGGACCCGCCGGAACGACGGAAGGAG

CGGGTCGCAGAACGGTTCACCGCGCTGGCGAGCGGCGAAACGTTCGCACT

CGTCAGCGACCGCGACCCGACGCCAGTCCGGCGGTTCCTCGGCGGGCTGA

CCGACCGCGCCCCCGCGGAAATCGACGGGTTCAGCGTCGAACGCCGAACG

CCGAACGACTGGCTGTTGACTGCGACGAAACCGTAAGCGAGAGCCGATGG

AACGACAGCCACCGGTGACCGGTATCGCGCTCGTCGCCGGCCTCTGTGCC

GCGCTCGACGCGGAGAGGTGGATGACACACGCCCTGACCGGCATCCCCAG

AACGTGGCCCATTTCCGTGTCTCGGAATCTATCCGGCTCCGACGAGTCCT

CAGGCCCTCCGGTCCGACGAGGTACGAACCGGACGCGGGGGCCGTGTGTC

TCCTCGAACTGACGCCAGTGACTGGTCGTGGCTTTTACCCGCGTCGAGAC

GCCACCCCCGCTATGGAACTTCAGATTACCGAGCTGGAGGACCTCGAGGA

GAGAGTCGGGAACGCGCCCACTCGAATCGGTGAACTGCAAGCGGGCACCC

GAGTCCAGTCGGACACCTTCTTCTCGCAGTCGTTCATGCGCGACCACACC

GAGTTCGACTCGTTCGCCGGGTTCTGTGAGCAGAGTCCGTGGGAATTCGA

CGACATCGACGACGCCCGAGACATCTCGCGTGACCGCCTCAACGAGTATA

TCGTCGCCACGACCGATTTCGAGACGTGGGAGGGGATGAAAACGCAGGCC

GCGGAGGAAGAGATTATCGACCAACTCGTCTCCTGACTACCCCACGACCG

GGGCCGCGTTCAACAGGAGGTACAGGCCGTAGCTGATGACGCCCGCGCCC

AGCATCGACCCGACCCACGACCCGATCGTGATGCCGACTTTCCGCGGTGA

GACGCCGCTTCCGCCCGACGAACTGGCGGCGAGTCCGCTTCCGACGATAC

TCGCAATCATCACTTTGTTGAACGAGATTGGAATCCCAAGGACAATCGCG

AGCTGTGCGATCAGAAAGGCGGGAATCAGGGCGGCTATCGACCGCCTCGG

CCCGAGCGACGCGTATTCGTTCGAGACGGCTTGAACGAGTCGTGGCCCTC

GAATCCACGCCCCGAGGAGGATGCCGCCGCCACCCAGCGCGAGCAGGTAA

ATCGACGAGAGCTGGAGGTCAGTCTCGAACACGGCTTCCAGCGGACCGGT

CGCCAGGCCGACTTGCGTGCCGCCGCTCGTGAACACGACGACGAGCCCCA

GCGCGACGAGGAACTGATTGATGCCCGCGGTCTCGTCGCGGTGGAGCAAC

GCCCGTGTCCCGACGAGCGCAACGAGCCCGCCGACCACGCGCACGACGAC

CATCCCGAGGGTGTACGAGCCGCCGACGACCGTCGGAAGGAACTGGTACG

TGGTGGCGAGGTATCGGGCGACTGACCCCTGTGTACCGGCTGCGGTTGGA

ATGACCGTGAGCTGGATGTTGGCCAACGCGTAGCCGACCGCGCCACCGAG

GACGGGAATCCCGACGGTTTCGGGGATGGCGTCGCTTCGAAGGCCGCGCG

CGAGTCCGTACGCGAGACGCCCTCGACGAGCGGAATCGCGAACCAGGACC

CGAGGATGACGACGTACTCGTCGACGGCGATGACCGACGCCATGCTGTCA

ACGCTCCCTCCTCGGTCGCTGGCGGTGAGAGAGAGAGATTGCCACTGGCT

ACAACGACGTATGCCCATGTATGAGAAAACCGTGGTAGGGGACGGATGAA

CCCTCCGAATTCGGGTATATCGACTGGTAATGAGCGAATCGTCTGGATAA

CTAATGCAGTTCATAGCGGAACTGACCACGGCCGTCCGCGACGTGATTAG

CGACCGATTGTATTGATTACTAATAGCAATATTACCGGCGGAGAAAAAGA

TAAGTATAATCTGGAAAATCCAAATGTGTTGAATAATCTGAATAATGTAA

TATGGTTGTGTCTATCATCGGATTTAAGCTTATATTTTGTGCTGTTGAGG

TACTACTGTATCGGGATATGAGATACTGATATATTCTCACTCCACCCGGC

CTATATTAGAAGTGTTAGTTATCTACATGACTGGTGACAAACAGACTCCC

GTAGTAACCCCGCCTCGGCGTTTCTCTCGGTGTCAGTCGTTGACGGACTC

AGCGCGCTGCACCGTCGGTACACGACGAATCATCTCTCACGCCATCGACG

ACGCTGGCCCGCACGGCAAAAGGAATACAATGGTACTACTACCAGCACGG

GGAAAACGGAGGCAACGTCGTCGAGTTCCCCCACGTTCTCGGCCAAGAGT

ACGACCTGAAGGGGTCGTTCAGGTTCAGTAACACGGACCCCATGGCCTCG

ACGGGGGACAGATAAATTCTATTAATGGTGGAATCACACCGAGAAATCGG

TAGCTACCGCAGTCGACACCGTCGCACGTGGGTTTGACTCTCGCTGCCGT

CGTACCGATGAAGACCGGCCGTGCATAACTGACCCACCCGTGTACCCCCT

GCGTGGCTTCGGGCTGCTCCCCGAGTACTTCGTTTTCAGCGATTGTAATT

ACAGGCATATGTCTGTCATGCCTAGCTCTTTTCGACCCGGCAGTTCAGAG

TCACAGAGCCCGAAGTCGTCGGTACGACTCGGAGAGCGCTCGCGCGCTCT

CGCCGGACACGTCAACGACGAGATAGTCGGTGTGCGGGCGGACGTAGTCG

AGGAGTCGGCGGACGCGCTCGGCGTCGACGGCTTCCAGCGAGTCGAAAAC

GAGGAACGGAACGTCGTCGGACACCTCGTGGACGAGGTAGCCGGCGACGG

CGAACACGAGGCCGACGAGTTCGCGCTCCGACTCCGAGAGGTGGTCGATA

ACGTCTTCGTAGGTCTTCCCGTCGTCGGTCCGGCGCTCGATGTGGAGAAC

GAACCGTCCGGTCGAGAGCGCGTCGTCGCCGCCGTCTCCGTCGTTCCGGT

GTTCGACCCAGACACGGGCGAGTCGGTCGTGTTCGAGGGCGTCGAGAACG

TCCGCCATGCGGTCGTTGAACGCCGACACCGTCGCGCGTTCGAGCCGTTC

GATGCGGTCTCGGAGGCCGTCGATTCGGTCGTCGATTTCATCCTTCCGTG

TTTCGAAGTCCGTCCGTCGGTCGAGTTCGGCGTCGAGGGCTTCGAGTTCG

TCGACGACGGCGTCGCGTTCCTCGCGCCGCCGTTCCAACTCGAATTCGAC

GCGGTTGACCTCGCGGTGGGCGTCGAGGAGCGCGGCGCGGTCGCCCTCGC

CCGCCTCGGCGTCCTCGGCGTCGCGTTGCAGGTCGACGATGTCCGATTCG

ACATCGTCGCGTCGGGCTTGAAGGTCTTCGACGCGGGACCGACGCTCGGC

GCGCTCGCGGCGTAACTCCTCGCGTCGGGTTTCGAGGTCCCGTCGTCGCT

CTCGCAGGGCTTCGAGTTCCGACCGGCGTTCTTGGAGCCGTTCGATGCGG

TCTGCGAGTTCGGCACGCTCCTCGCGGCGGTCGTCGCGGAGCGCTCGGAG

TTGGTCGAGCGTCGATTCGATGCTCGCTTCGGGAACCGACGTTCCGCACG

TCCAACAGACGACGCTGTCGCCGTCGTCCGACTCGCCGTCGCTTTCGTCG

TTCTCGCTCTCGCTCTCGCGTTCGACCTCGCTCGCGGCGGCGACGGGCGA

TGTCTCCGACGTGAGGAACTGCTCGTTGAACTGGAGCGTCCGCTGCACGT

CCGTGATGTCGGCATCCAGTCGTCGTTTTCGCGCCCGTGACTCCGCGAGT

TCGGCGGTCAAGTCGTCGAGTTCGGCGGCCGGGTCGGCGGGACGCGAGTC

CAGTTCCGATTCGATGTCGTCGATTTCTGCGTCGAGCGCGTCCAGCGACT

CTCGCTCTGCTTCGAGTTCGAACTCAATGTCGTCGAGCGCCGCGTACAAC

TCCCGTCGCCGGCCCTGTCGCGCCTCTGCATCGGACCCGTTAGCGTCGGG

TTCGGGATTCGCCTCGGCGTCGGTGTCGCCGTCGACCGCCGCGGATTCGA

TTCCGTCGAGGGTCGCCTGCCGGTCCGCTAACTCGGCCTCGAGTCGCTCG

ATAGTCGCGTCGAGTTCGTCGCGGCGGTCGCGGAGCCCCGGCCGCTCGGA

TTCGAGCGCCGCGAGCGCCTCGATTCGGTCGTCCAACTCGGCGCGTTCGG

CGCGGAGGTCAGCGATTTCCTCGTTGACGGCGTCGATGTCGACCGAATGC

ACGACGAGTTTCCGCAGGTCGCGTTCGGCCGTGACCGCCTGTCGGGCCTC

GTTCGAGCCGAGGAGGAAGGCGAACAGGTCGGCGGCCGCCGGGTCGTCGA

GATACGGGTCGCCGGACGCGACGACCCGGTCGCCTTCTCCCGCGAACGTC

CGCACGTACCGCTCGCCCTCGACGGCGAGTTCGACCCAACTGTCGTCGGT

ATCGGCGTCGCAGTCGGGCGAGTGCTCGTCGCTTCCCAGCGCGGTCACGA

GACCCCGAAGGAGCGACGTTCGGTCGGCGGCGTCCCCTGCCGTGAGGACG

GTGACGCCGCGTTCGAACGCGACGGTGGTCTCGTCGAAGCCGCCGATTCC

GGCGACGGTCAGGCGGGCCGGTCTGTTCGTCGTGGCCATACGGTGACCAC

ACCGGGCCCCGTGAAAACAGTTGGTGACGAGTCCGAAATCCGTTCCATAG

ATATCTTTGGAACGTGTTTCTCGCCCCGAGTTCGAATCCGCGTATCGGCG

TCTTGAGACCGCGGTTTGAGCCGTCTCGGGAATCAATCGTCGATTCAGTC

GTCGATTCGTCCGCGACGGCCTTCGTGCCATTCGTCTTTCTGCCTCGTAT

CTCCGTCTGACGGCGGTGGCATACAACTCGTTCCACAGATATCGGTGGAA

CGTGTTTCGAACCGCGGCGGGCTTCGGAACGGCTTTCGAACTCGGGACCC

AGTCGCTTCGGGATGCTCGATATCGGGGAGGACGATGCGCGAAGCGGCGT

TAGTCGCAGTCGCAACCGCCGCGGTCGATGAGGGATTCGACGGTGTAGTG

AGCGTTACAGTCCGGGCAGTGGACGCGAACGTCGACGTACACCTCGGCGG

TCTCGGGATCGACGTCGAGTTCGTCGGCGGCCGCGAGGTCGGACAGCGAC

TTCTCGGTGACGCGCTCGACGCGACCAAGCAGGCGTCGGATGGTTTCGGT

GCGGTGCTCGCGGAGGTCGCCGTCGTCGCGGTCGGTCTCTGAGGGCGGCG

TCACGCCGCGGTACGAGGTGAGATAGGTGCGCATCGCCCGATGCGAGACG

AAGTCGGCCTCCAGTCGCTCGACGTCGACGCCGGTGCGTTCGAGTCGGTG

GACCGCTTTCGTCCGCGCCCCGCTCGACACGTCGTCGTCGGTAAGCACGC

GGTACAGGGTCTCGATTTCGCCGTCGAGGGCGCGCTCGTTTCGCCCGTCG

AGCGCCGCGCGGAGGAGCCGACGGTTGAACGCGTCAGCGAGGTCGCGGAG

GCTCGACCGCGCGCCCGCCTCAGCGGTCCAGCGGCGTTCGAGCTCCGCGC

CGAAGCCGTCGAAGCCGCGCTCGCGGAGCAGTCGGCTGACCTTGCTCTCG

GGGCCGCGTTTCCCGCCCGGGGAGTCGTGTGAATCGCTCACTACCGACTG

CTATTCGGTGCGTGCACATATGCTTGACGCGGGGGGAGACGTGGTTCCGG

GCGAGGGTGCGAAGGTGGAGACAGAGGACGGCGGGTGCAAACGAGAAGCC

GAGACGGAGCCTCAGTAGTTGGCGGCGTCGCCGCGACCGGCCGACGGGCC

TCGGCCGGCCGACGATTCGCGGTCCGAATGTACGGCGGCGAAGTCGTCTC

GGGCGTCCCCGCCGACGCTCGTCGCAGGCGACCCCGCGGGCGTCGATTCG

GCGTCGCCGGCGTTCTCAGCGACCCGGTCGGTGGGAGACTCAGTCGAGTC

CTCGGCGGTTCTGAGGGTGACTGCGACGGTCGTGCCGCCGTCGGGGTCGT

TTTCGACCGCGATGCTCCCGCCGAACTGCTCGACGAGCGCGGCGACGAGC

GAGAGACCGGTTCGGGAGTCGGGCGTCTCCGCGACGGTCTCGCCCGCGTC

GAGGAGGGCGTCCTCGGGGAACGACGCTCCGTCGTCGGAGATGCGGACGG

TCACGGTCTCCGCGCCGCAATCGGCGCGCAGACGGACCTGCGGGCGCTCG

GCGTCCGCCCGCTCGACCGCCGTGACGAGGAGGTTCCGAAAGACCGCGGA

GAGCATGCTGTGGGCGAGGACGTTTACCTCGGGGAGGTCGCCGGCGACGA

CCACGTCGGCGTCGTACCCGGCGCGGACCTTCTGAAGCTCGGTCTCGACG

GTTTCGACAAGCGGCTTCGGTTCGAGGTCGGCGGCGCGGTCCGAGGTGAG

CACGTCGGTGAACTGCGCCGTGCTCTCGGTGAGGTCGATGATGCCGTTGC

TCGCGGCGACGATGTTCTCCAGTCGCTCGTCGGCCCCTGACCCGGCCTCC

TTCCGCACGACCGACGCCCACCCGCGGATGACCAGCGCGTCGTTGCGGAT

GTCGTGTTGGAGGACGCGGTTGAGGACGGCGAGCTGTTCGCGCTGTTGGC

GGAGTTCTGTCTCCCGTCGCTGGCGCGCGACGGTCCACCGATAGAGGCCG

AACACGAGGAGCACCGCGCCGACCGCCTGCGCGCCGTCTTCGAACAGGTA

GCTGAACCAGACGGGCTGTTCGACGAACTCGTCGAGGAAGTCGGTCAGCG

CGAACAGGTACGAGGCCGCGACGCCGCCCGCCAACGGATAGAAGACCGAT

GGGTCGCGGATTCGTCGCACCACGAACAGACACGCGAAGAGCACGGCGAC

GACGACGCCCTCGCCGACGAGGTCGAGCGCCGCCGTCGTCGGGTCCGGCG

GGAGCGCCGCGAGCTGCGCGACGACCGGGATTGCCGACCCGGCGGCGACT

GCCCACGGGAGGTATCGATGCGTGGCTCCGGCGGGTTCCTCGCTTGCCTC

CGCTGATTCGTCGTCCATTCCTCGCGTCGGTGTTTCGGGTTTCATCTCGG

TTCACCACACCCCGAGCGTCTCCCGCGTCCGACCGGCGGCGTCTGTCTCG

CTTGCATACTCTCGTTGACGGCGATGTCCGATGAAGTGGTTGCTATGTGG

ATTGTCTGCCTCACTTTCCGTTCGGTAGTCGTCCCCCGTGTTCGGTAGTC

GTACGGAGTCGAAAATAGTTCGCGGGTCGACGCGTGCCGACGCGTGCCGT

CAGCTCAAGTAAACACCCAGACCGCGGCGACGAACAGCAGCGACCCGAAG

ACGAACGTGACGTCTCGGGTCCGCACCATCATGCCCTCGGTCCGGAGGGC

TTGGCTCTGTTCGTCGCCCAGCGAGCGCGAGAACCCCTTCGTCTCCATCG

CCTCGACGTTGACCCGCGAGCGCTTCGCCACGTTGAAAATCAGCGGGTAG

AACGCCTTGACCGAGAGCGTCAGCAGGTAGGAGAAGTAGCGCCACCTGAA

CAGTCCCTGTTTTTCGGGAACCTTGCTCCGGAGGCGGAAGGAGTTGATGA

GCGCGTGGTACTCCTCGACGAGAAGCGGCATCATCCGGTAGCCGTAGGCG

ATGGCGAACGTGAGCTGACGGGGGACGCCCAGACGCAACATCCCCTTGCT

GAGCTTCTGCGGGCTCATCGCCGAGAAGACGGCGAGGCTGATGACCGAGA

TAATCGTGAGCTTGAGGAAGAACGGGACGAGCGCCCCGACGGCGTCGATT

CCGACTTCCTGACTGGTCGTGGCGGTGTGGATACCCCGACCGACCGCGGC

CGCGACGATAGTTCCGGTCTCGGCGAACACGCTCACATCACCCGTCGTGA

CGCCTTCGACGACCCGACCGGCGTTGTCACCCAGGTGGCCCACCGCTCGC

ATCGCCCCGCCGATGAGCGGGACCAACACCACGAAGAAGCCGACGTTGGT

GACTTGGCCGAACAGGAGCAACGCGAGGAGGTATTTGCTGACCTGCGAGA

GCGCGGCGAGCACGAAGGCCGCCGCGAGCAGGATGGCCAGCGGGAGCGTG

TCGTAGAACAGCCACGGAATAATCATGAACAGCACGGTCCACACGAGCAC

CACGCGCGGGTCGAAGCTGTTCAACAGGGCGTCCTCGTTGTCGTAGGCGG

TCCGCATCAGGTCCACCTTGATGTCGGTGACCGAGATGTCGGTGAGCGCG

TCGACGTACTTCATCGCGCCCCTCCGTCCGACTCGGCTTCGTCGTCGCTA

TCGCCGGCCTCACCGATGCCGCCGCCACCGCCAGCTTCGACCACGTCGTC

ACCGAGCGACCGAGCCAGCGTCTCGCACATCGCGTCGGTGCTGAGGGCGG

GGGATTCCAGTCCGAGTCGCCGGCTCAGTTCGACCACCTGCGGCTGGCGC

AGGTCGGTCTCGGCGAGGAGGTCCGGGTCGGAGAACACCGCCGCCGGCGG

CGCGTCCGCGAGCACCTCGCCCTCGCCCATGACGAGGACGCGGTTGGCCC

ACGCCGCGACCAGTTGCAGGTCGTGCGAGGCGACGACGACGGTTTCGACG

CGGCTCTCGGCCTTCCGGAGCATGCCGGTCACCTCGCGGCGGCTCTGGAG

GTCGAGACTGCCCGTGGGTTCGTCCAGGAGGACGACCGTCGGGTCGGTCG

CGAGCCCGATGCCGAGCGACGCGCGGCGCTGTTGGCCGAGGCTCATCAGG

CGACCGTCACGGTCCGCGAGGTGTTCGAGGTCCAGATACGCGAGAATCTC

GTCGACGCGGTCGTCCACGTTCGGGGTGTCGCGGTTCTCGAGGTAGTAGG

CGATGTCCTTGCGGACGGTGTCCTCGACGAACATCTCCTCGGGGTTCTGG

TGGATGTAGACGGTGTCGTCGGCCAACTGCTCGGGAAGCGTCTCGCTCGT

GTCGCGTCCGAGAACCGTCACCGTCCCCCGGTCGGGGGACTCTACCCCCG

TCAGCAACCGCAACAGCGTCGACTTCCCGGAGCCGTTGGCACCGACGAGG

GCGACCCGGTCGCCCGCGTGCAGTTCGAGGTCCAGCCCATCGAGGACGTG

GTTGTACCCCTCTCGAAGCGTGGGGTAGCCGTGACCGACACCGCGCATCG

TCACCAGCGCGTCCCTATCCTGTTCGCCGTCGGTCGGCGCGCCGCCGGTG

TCCGCGCCGGCTGTCGCCACCGCGCCGCCGTCGACCGCGGCCCGCGAACC

GCGTGCCGTGGCGGGCTGAAACGCCGTTGCCGCCTCGTCGACGGTCACGG

GATACCGACCACTGGGTAGGGTGCCCGCCTCGGACGGCAGGCCGTCGGCG

ATTTGCGTGACCTGCGGCGGGTGGATGTCGTGGGCGAGTAGGTCGTCGAG

TCTGTTGAGGCCGACTTCGACCGGTTCCTTCCAGGCGACGCCGCCGTCGG

AGACGAGCACCATCTCGTCGCAGTAGTCGGCGATGAACTCGGAGTGGTGT

TCGATCACGATGACCGTCTTGTCGCGCTCCTCGTTGAGTCGTCGGAGCTG

TTCGTACGTCTCGCGGGCGTTTCGGGGGTCGAGCTGTGCGGCCGGCTCGT

CGACGAAGATGAACTCGGGGTCCATCGCCAGCACCCCCGCGAGCGCGACG

AGGTGTTGCTGGCCGCCGCTCAGCTCCCAGATGAACCGGTCTTCGAGGTG

GTCGAGACCGAGGGTTTCGAGCGCGCGGGTCGCCCGCTCGGCGTAGTCGT

CGAGCCCGTGGTTGAGGGGCGCGAACTCCACGTCGTCGCGGACCGTCTCC

TGAACGAGTTGGTTCTCGAAGTCCTGAAAGACGTAGCCGACCGTCTTCGA

GAGTTCGGCCACGTCCGACTCGCGGGTGTCGGTGCCGGCGACGCTGACGC

GCCCGTCGAACGTCCCCTCGAAGAAGTGCGGGATGAGGCCGTTGAACGTC

TTACACAGGGTCGTCTTGCCGCTGCCGTTGCCACCGACGACGGCCGTGAA

CTCGCCCGGCTCTATCGTCACGCTCGCGTCGCGCAGTACCGCCTCGTCGC

CGCCGGGGTACTGAAACGTCAGGTCCTCGACGACGATGTTGTTGCTGCTC

ATGAAAACAAAAGATGGGGGTTTAGTCCTGCAAGCCGTCGACCTTGCGCT

GGCGGTAGGCGACGAGCCCGAACGAGATGACCGCCGCGATGGCAATCGGC

ACGAACAGGAACGCTTGGCCGTACGTTTCGACGAACTCCGGCGTGAACGT

GACGAGCCCGCCGCTGGTCTCGCTCAGCGTCTCGAACGCGAACGCGACGG

GTATCAGCGCCACCCACGCGATGAGTCGCTTCAGCGACGTGCGCGTGAAC

ATCGGGCCGTCGCGGCCCTCGACGGGGCTCATGCCGAGCAGCGGTTCGAT

TTTCCCGCGGAGTCGCGGGTAGAGGAACAGCGTCGGAATCGCCCCGAAGA

CGACGCCGGCGATGATAATCTGAAGCAGCGCCCCGATTCCCTCGGTTGCC

CACACAGTCTCGGGGAGCCACGAAATCGCTTCGAGCTCTTCGACGCCGAC

GTAGAACTTCCCGACGTCGATGAACCACGCCGCGGTCTCTTCCATCGCCT

TCGCGAGGAAGCCCACCGCCGCGATTTGCTTGACGTTCTTCGGGTCCGTG

ATGAGCGACATCGCGAAGAACCACGACAGCGTGATGACGATGAGGCCCTC

GACCGCGCCGAGCGCGCTGAAGTCACCGATGAGAATTTCACCGAAGACGA

TGCCGCCGACCGGAATCGCGAGACAGCCCCAAAACGACCGGAAGAGGAGA

ACGACCGACATCGCGACGAACCAGAACGGTCCGATAGAGACGCCTAACCC

GCCTATCGAGAACTCCGGCAGGAGTTCCGTAATCATGCTCTGCAAACCGT

GCAGCGACATGACGAGGATGAACGCTATCATGTCCCTGTGGTCGAAACTG

AACGCACTACTCGAGTTGTTGGACGTTGTCACCATCCGGGTGGTGGTCGT

TAAGCGAATTGGTTAATCGTTGGTATGAGCACAGCGTACGACTGTATATC

CGACTATACTCTGTGGAGTAATCGGTGGCAGTACATAGCAATCGCATCAG

TATATCGCGCTCCGGACGCCGACCGGCCGCGGCGCTCGCGAACCGATTCG

GATGCGACTGCACGAACTGCTCGCGTCAAAAAAAAGAACGGCACGGAACC

GGGAATCGAGCGGTGGCGATGCCGCCGACGCCGACACCGACGCTGACGCC

GATACCGACCCCGACCGTCAGGCGTCGGTCCCGCTCACGCAGCGCTCAAC

GTCAGCGCGGGACTTCATCTCCAAGACGTTGTACGGGGCGTCGATGTCCG

CGGCGACCGACCGGAACGACGGGAGCGGTTCGTGCGCGTCTTCGGTGCCG

TCGTTGTCGTGGAGGTGCGCGACCTGAATGCGGTCACCGAACCGCTCGAC

AAACCGCTCGTAATCGACGCCGGTCGCCTTCGCGTGCCCCACGTCGAGCG

TCAACTTCAGGAACTCGTCGCCGACGCCGACGTCGTCGAGGAACGCGGCG

AGTCGGTCCGGGGTCGCCGTGTGCCGACGCGTGCCCGGTTTGTCGCGCTG

GTTTTCGAGGCAGACCGGGACGCCCACTTCGGCGGCGAACTCGGCGCACT

CGCGGAGCGTTCGAACCGCCTGCTCGCGGGTGTGCTCGCGGACGTGGTCG

GGGTAGCGCGTCCGAACCGACCCCCCGTGGACGATGACGCCGCCGGCGTC

GATTGCCGCGGCGAGCTCGATTGCCTCTTTGGTCGCCGCGACCGCGGCGT

TCCGAAGGCGCTCGTTGACGTTCCCGGGCGCGACGTCGAGGTGCGGGCCG

TGGACCGTGTAGCTCACGTCGAACTCCTCGGCGACCGCCCGCAGGCGCTC

GGCGTCTCGCTCGCCCGGTTGCACGTCCAGATACCCCGCGCGGAGTTCGA

CGTGGTTCAGGTCGAACCGCCGGAGGAACCCGGCGAACGAGGCCACCTCG

TCGGCGTAGCGGATGTCCATCGACGCGCCGAACCGCGGTCGCTGGACGGG

CGTCGCCTCAGACATCGACCCGGCTCGCGGGGACGAGTCGGTCGCCGCGG

AGGACCGGCACGACCGACCGGGAGTCGATAGCCGTCTCGAACTCGTGGAG

GTCGCGCCGCCGAATCTCGTGTTTCTGCTCGTATCGCGGCCCCTTCGCGG

TCACGAGGAGGCGCGCGTACCGCTCGCGGTCGCGGTCGGTGAGGCCGTCG

CCGTGGAGGCACTCGTCGATGAGCGCCGCGCCGAGGGTGTCGTCGGGCGT

CGGCTGGCCGCTCGACCCGCTGGCGACGATAGTGACCGGCCGCCCGCGGG

CTTCGAGCAGGTCCGCGACCGCCGCGGCGTTCGTGTAGCCGCCGACGAAC

ACCTCGACGTCGGACCCGCCGCGCTCGCGGAGCGTCGAGACGGCGCGCCC

GCCGTTCGTGGAGGTCATCGCGGTCGGTCGCCCGTCGAGGTCCAACTGCT

GGACGTAGCTCGGCGAGTTGAAGAAGTCGTAGCCGGGGTTCGGCTCGTAG

TCGTCGGTCTTGCCGCCGCCGATGAGACTGTCGGGGTGGTCCTCTTGGTA

CGCCGGTTCGTCGCCGCGCTCGTCGGTCACGTGGACGTACTCCGCCCCGA

GCGCGAGCAGTTCCGCGACGGTCGCCGAGAAGTGGGTCACGTCCACCACC

ACGTAGTCGCCCGGCGTCGGCTCCGCCGGGATGTTCTCACAGCCCTCTAT

CAACGTCTCTACGAACGGCTCTGTCTGTCGTCCATCTGCGGACATAGCCC

AGGCTCTCGACAACACCCACAAGTCAGTTGCTAGTAGCGATATATATCCG

ACCGTACTATCCGTAGTTCGGACGCCGCGTTCGACGACGCCGGGATCGGG

AGAATACTCGGAAAGCAAACTATTTTCTACTAGATATTATGATAGTTCTC

TCTCGCCGGTCGCGGTCCCGACCGCGGCTTGGAACGACGACGTTCTTCCG

ACGAATCGTCTGTCTCACTCCCTGTCGATTCGGGGGCGTACAGCGCCACA

CTCGGCGTTTCTTCGTTCGAGCGAGCCGAGCAGTCGGGAACGTCCCATCT

CGGTCGTGATTTGGACGCGTTCCCGAACTGTTGGATGCCCCGAAACGCAC

CCGCTCCACCGCGGAGCGTGCGACCGCGTTCACACCGCCACTAAGATATC

ACCGAAACACTCTAAATCGCCCGAATTTTACCTGTTCGTGGCAAACGACG

CCGTCGTCGTGGGTGGGGCTATCGACGCGGGAGTCCCGTGGTCGGCTCCC

AGCCCGCCGGCGGTTTGCCCCGACTTCGGTCGCTTCGGCCACGAGACGGA

CAGACAAACTTATCAGTATAACTACTGTCTTACTGTTTGATTAGTGAGCT

TCGAAAGCGTCCGTGGCCGCCGCGTCGTTGCCGCCGACAGCGTCTTCCAC

CGCCGGCCCAACGTCGATTCGAGATGACTCTCGCCACCGAACGACTCCGC

CGATTCAAGACGCCGCTCCGGTACGCGATGGGTTGCCTCTACGTCCTCGC

CGGCGTGATGCACTTCGTCGTGCCGAACGCGTACGCGCAGGTCGTTCCGC

CCGTCTTCCCGGCGGCGCTCGCGCTCGTCTACGTCTCCGGCGTCGTCGAA

ATCGCCCTCGGCGTCGGCGTCGGCGTCCGCCGCACTCAGCGCGTCGCCGC

GTGGGGGCTCATCGCACTGCTGATTGCGGTCTTCCCCGCGAACGTCTACA

TGGCGACGAGCGACATCGTGCTCGACGGCGTTCCGGCGGCGTTTCGGGAG

CCCTCGGACGCCGCGCTGTGGCTTCGCCTGCCGCTTCAGGGCGTACTCGT

CGCGTGGGCGTGGTGGTACACTCGCCCCGAGTCCGTCGAGGCCGACGAGT

CGCGTTCGGTGCGGTGACTGTCTCCAAGCGAATTGGTGTGAATCACCACG

CTGTCACGCGGTTTTTCGCGTCCAAACGGAACTGATTCAAGCGCGCCGGT

GCGAGCCGTACCCGATGACCGAAGATGACACTCACGACCGCGTCGTCCCC

GGAAGCGACGAGGCCATCGACTCGGCCGACGTTCGGGGCTACGACTTCGA

CGGCGCGTTCGACTTCGACGAGTTCCTCGACGCGTACGCGACGACCGGCT

TTCAGGCGACGCAGCTCGCCGAAGCCATCGACATCGCGGAGGAGATGCAG

GAGGCGGATGCGACCGTCTACCTGACGTTCACCTCGAACATCGTCTCGTC

AGGCCTGCGCGAGGCCGTCGCCTACCTCGTCCGCGAGGGGTTCGTCGACG

TGCTCATCACGACCTCTGGGTCGCTGACCGAGGACGTCATCAAGACCGCC

AAGCCGTTCAAGATGGGCTCGTGGGACGCCGACGAGGGCGAACTCCGCGA

GCGCGGCATCAACCGCCTCGGCAACATCTACGTCCCCTCCGACCGGTACG

TCTGGCTCGAACAGTACCTCTACGACTTCTTCGAGGACTTCTTCGCGGAG

GAGAAAGTGCGAACGCCGACGGCGTTCGCCCGCGAACTGGGCGCGACGCT

CGACGACGAGCACTCGGTGTTGAAGCAGGCGGCCGACAACGACGTGCCGG

TCTACTGCCCGGCGCTCACCGACGCCGAGGTCGGTAACTTCCTCTACTAC

TACCGGCAGGGCTACGACAAGGAGGTCGGCATCGAGATTCTCGACGACTA

CGACTCGCTCATCCAGGACGGCCTCCTCGCGGACGAAACCGGTCTCATCG

CCGTCGGCGGCGGCGTCCCCAAGCACCACGCCATCATGACGAACCTCTTC

CGCGGCGGCGCGGACTACGTCGTCTACATCTCGACGGGGATGGAGGGCGA

CGGCTCGCTGTCGGGTGCGCCCCCGAACGAAGCCGTATCGTGGGGGAAAG

TCAAGGACCACGACACGAACTACACGCAGGTCGAAGCCGAGGCGACGCTG

GTCTTCCCGCTCCTCGTCGCCGGCGCGTTCAAGAACTAGCGGGGCTACGC

GCCCCGCCGAGCGATATTTATCCTCGTGAGCGAGTGGTTGCTCACCCAAC

ATGAACGAAGACGACGCGGTCGGCGGCGAACTACGAACGGTCGGTATCCT

CGGCGGCATGAGCAGTCAATCGACGGTCGAGTACTACCGGCTCATCGACG

AGGGTATCAACGACGTGCATGGCGGCCACCACGCGGCGGAACTCCTGATT

CGGAGCGTCGACTTCGGCACCGTCGAGCGCTACATTCGGACCGAGCGGTG

GGACGACGCGGCTGACTATCTCGCAGCCGCCGCGACGGACCTCGAAGCCG

GCGGCGCGGACTTCGTCGTCATGGCGACGAACACGATGCACAGGGTCGCA

CCGCGCATCGAAGCGGCGCTTTCGATTCCCTTCCTCCATATCGTCGACGC

GGCCGCGGACGCGATTCTCGCCGACGGACTGGAGACCGTCGGCGTCCTTG

GAACTCGGGCGACGATGGACGGCGCGTTTTACCGGGAGCGGTTCGAGGAA

CACGGCATCGATGTCATCGTCCCCGAGGAGTCGCGTCGAGACGAAATCGA

CCGAATCGTCTTCGAGGAACTGACCAAGGGCGAGATTCGCGAGGCGTCCC

GCGACTACTACCTGAAAGCCGTCGACCAGTTAGTCGAACGAGGTGCCGAC

GGAATTGTCCTCGGCTGCACGGAAATCGAACTCCTCGTCGAACAGGCCGA

CCGGCCGGACGTTCCGCTGTTCGACACGACGGCGCTCCACGTCGAGCGGG

CCGTCGAACGAAGCCTCGCGGGACGGGTAGACGGATAGCGGAGTTCGGTC

GTCGGGATGTCAGTTCCCACCCTTCGCGAGCGACTTCGGGTCCGTGGCGT

AACTAAGGAACCACCCACTCAGTGGGGAGTGACTCGCTTGAGCAGATAGT

AATCCGATGGCCGAAAACGCCACGCGGCCGTTCGGCGGCCGCGACGCGGT

CCTGAATCGGCGGTGACCACACCTCCTGTACAATTACACTTTACAAAATT

AAACCACAATTGATTTTTGCGGCGGGCGGAGAATATCGAGGAGACAACGG

TCGCTACCCACTCTCAGGAATCATGAACGAAGCACGCCCTCTACGACGCC

GTACCGTACTATCAGTACTCACAGCGGCGAGTCTCGGTGGTCTCGCCGGC

TGTCTCGGAGGGGCCGACGACAGCGCCCCGGACGCGAGTGAGAACGAATC

CACGACGTTCCGAGTGGGAACACAGTGGAACCCGGACTCGCTCGACCCCC

TCGTCAAAGGGTGGGTGTTCAGGAAACTGAGCGTCATCGAGCCGCTGGTA

ATCACGGACTACGATGCCTCGGTCGCGCCGGGCTTGGCGACCGATTGGAG

CGCCACCGACGAGAGTCGACAGTGGGAGTTCACCCTCCGCGACGACGTGA

CGTTCCACGACGGAACGCCACTTTCGGCCGCGTTGGCAGTCGAATCGCTC

CGCCGGAGCTTCGCGTCTACGTCGCTTGCCGGCCTTCCCGTCGAGTCGGT

TTCGGCCGCGGACGACCGGACCGTTCGCATCCGGACCGAACGTCCGTTCG

CGCCGCTGCCGGCCCACCTCACCCGCGCCGAGACCAGTATCGTCTCGTCC

GAATCGTACGACGACGACGGGGCGGTAACCGAGTTGATCGGGACCGGCCC

GTTCAGGTTCGACTCGTGGGAGCCGGGCGAACGAATCACGGCTGTCGCGT

TCGAGTCGTATCACGGAACGGTTCCGAGTATCGACGAACTCGTCTACGAG

CGCGTCGCGGACGAACAGACCCGTCTCCTCAAACTCGAAAATGGGGAGTT

GGACATGGCTCGTCAGCTCTCCACAGAAACGACGCCGGGTCTAGAAGCGC

ACGACCACTTGACGGCCTACGAGTACGAAGTGCCGAGAACTCGCTATCTC

GTCTTCGACACGACCTCGACGCCGTTCGGGGACCGCGAGGTTCGCCGAGC

GGCCATGTACGCCCTCGACCGGGCTGGCATCGTCGAGAGCGTGCTGAACG

GCGGCGGGCCGGCCGCCGTCGGACCGTACCCGCCGGAGCTGACCGAATGG

GCCAACGAGGACCTCGAGCCGTACGCGTACGACCCCGAGAAGGCCCGGGA

ACTGCTCGAAGCGGCGGGGTGGAAGGCGACCGAAAGCGGCCGCGTCCGCG

ACGGGGAACCGCTGGAAATCGAACTGTGGACCTACGACGCGTGGTCGCTC

CCAATCGTCGCGCAGGTCGTCCAAGAACAGCTCTCGGCGGTCGGCTTCGA

CGTCTCGCTGCGACAACTGGCGTACAGCACCATCCGGGAACGGGCGAATC

GGGACTCGTTCGACGCCGTGCTGTGGTCGAACTCCCTTTTGTGGTACCCG

GACCCGGACCGGCTCGCGGACTTCGTCCACTCGACGGAGGCAACGATGTT

CAGCGGCTACGAGAACGAGCGGGTCGACCGACTGCTCGAAGCAGCCCGGA

CGACCACACACCGCGCCGAGCGGAAGCGCCGGTACGACGAGGTGCAGGCC

ATCGCCCAGCGAGACGTTCCGATTGGCTGGGTAACACACGTCACGAACGT

CGTGGGCACGAGCGCCGACGTCGAGGGCTACCGGCCGCTGCCGACGGAGA

CGTGCTACCACCTCGAAGACGTGACGCACCAGTCGAGGGCATGACGGAGC

GAGTGCCACCCACAGGACTGAGCGGCCACCGCAGCGGCCGCAGCGACCGC

CGCGGTCAGAGCCGTCGGAGGCAGTCGTGAGCCGGCACCTGCTGCGTCGA

GTCGGCGGGGCGGTGCTGGTGCTGTTCGGCGTGTCCGCCCTCACCTACGG

CTTCGTCTTCCTCACGCCGGGGGACCCGGCGGTCGCCGCGCTCTCCCAGC

AACTCGGCCACCAACCGTCGGCGGCGGCGGTCGAACAGTTCCGGGCGACT

CACGGGCTGAACCGCCCGCTTCCGGTCCGGTACGCGGACTGGCTCAGCGA

GGTCGCGCGCGGCGACTTCGGCACCTCGTACCAGTCGGGGCGGCCGGTGC

GGTCGATGCTCGTTCAGCGCCTGCCCAACACGGTCGAACTGGCGGTCGCG

TCGACGGTCGTCGCGGTCGCGGTCGCCGTCCCCGCGGGCGTGACGAGCGC

CGTGCGGGCCGGTGGCTACGTCGACGACCTGACGCGGGTCGCGTCCCTCG

TCGGCGTGTCGATGCCGAACTTCTGGCTCGGCTACTTGCTCATCATCGGC

GGCGCGCTCCAACTGGGTCTGTTTCCGGTTTCGGGAGCGGGCGACATCTC

GCACCTCGTGCTCCCGGCGGTGACGCTCGGCTCGGGGATGGCCGGCGTCA

TCGCGCGGCTGGTTCGAACGTCAATACTCGACACGCTCGGGACGGAGTAC

GTCCGGACGGCCCGGTCGAAAGGGCTGCACGAGCGCGTCGTGGTGTACAA

ACACGCGTTCCGGAACGCGCTCGTCCCCGTCGTGACGGTCATCGGGCTCC

AGTTCGGCTACGTCCTGAACGGGGCGGTCGTCGTCGAAGCCGTCTTCCAG

CGGCCGGGGTTGGGGACGCTCCTCGTCGACGCGGTGTTCGCGCGGGACTA

CCCCGTCGTGCAGGGCGTGACGCTCCTCGTCGGCGTCGCGTTCGTGAGCG

TCAACCTCCTCGTCGACCTGACGTACCGCTATCTCGACCCGCGTATCGAT

ATCGGAGGGACGCGGCCGTGAACGGGACCGACGAATCGGTCGCGTCGGAG

GCGGAACGCGGGTGGCGACGGTGGTACGCCGCGCTCGCGACGAACCGCGC

TGCGAGGGTGGGCGGCCTCATCGTCGTCGCACTGGTCGTCTTCACGGCGT

TCGGCCCGCTCCTGTGGCCTCGTGATCCCGTCGAACAGCACCTCGTACAC

CGGCTCGAACCTCCTTCGCTCGCGCACCCGCTCGGGACCGACCGGCTCGG

CCGCGACGTGCTCGCCCGACTGCTCCACGGTGCGCGCCTCTCGCTCGGCG

TCGCCGTCGTCGTCACGGGGGTCCGTCTCGCGCTCGGGACGGCCGTCGGC

CTCGTCGCCGGCTACGTCGGCGGCTGGGTCGACGCGGCGCTGATGCGACT

CCTCGACACGCTGCTGTCGTTCCCGGGTATCGTCCTCGCCATCGTCGTCG

CGGGTATCCGCGGCCCCAGCCTTCTCAACGCCATGGTCGCGCTCGCGGTC

GTCGGCTGGGCCTCCTACGCCCGACTCGTTCGAAGCACCGTCCTCTCGGT

GCGAGAGCGGGAGTTCGTCGCCGCGTCGCGGCTGCTCGGGTCCTCTCGGC

TGCACGTGATTCGCCGGCACGTCCTTCCGAGCGTGGTCGGTCCGGTCGTC

GTCCTCGCGACGCTCGACGTCGGCGGCGTGATTCTCGGTGCCGCCGGCCT

GTCGTTTCTCGGTCTCGGTGTCCAACCCCCGACCCCGGAGTGGGGGGCGA

TGCTCGCCGGCGGCCGGAACCACCTCCGCGACGCCTGGTGGCTGGTGAAC

GCTCCGGGCGGCATGATACTGCTCACCGCGCTGGGGTTCAACCTCCTCGG

CGACGGACTTCGGGCGGCGCTCTCCGCGACAGAGTCCGACCGGGTAGAAC

GCGAACGCGCGTGAGACGCCCCTCACGGCGCTCGTCGTCGCGAACGATGA

GGACGGAACGTTGATAGAGCTACGAACGTTCCCGTCCGCTCGTCAGGAAC

TCACCTGACCACGATACGACTGCGCGTTCCTCGTGGAACGCATGCCAACC

GATGGCCGGGTGGAAAATGAAGTTATCTGAAAATAGAATTTGAGATGTCT

AATCCAGGTGACGGCTCTCTATTCGAGCAACCTCGACTGGAGTGACCGCG

ATCCGACCGACGGGGGGACTCGTGTTTCCCGCCGCCTATTGAGTGCCCGG

TAGCTCTTTTGGGGTTGGCATAGCCCTGCAATCAGTACGGACAGCTCATA

GTAACGGCCTTTATCACCCAGTCATGTTGACCGGGTGATGGTATCAACCA

GGAGAAGTTTCATCGGAGGTTCTGCCGCGGCGGTGGCGGGTCTCGCCGGC

TGTCTGACGACGAGCAGTTCCGAGGGTGACGCCGACGCGACGGGGTCCGG

TGGCGGCGACGCGGAAACGTACGAGGTCGGCTATCGGGACTTCCAGACGA

ACATCGAGGCCACCGCGTTCCCCGATATGCTGTACTTCTACTGCGTCCAG

TCCGGGTGGATGAACTGGCCGTCGCTGATGCAGTCGTTCGAGGACTCCTA

CGGCGTGGCCGTCCACGACGACGACCGCTCGTCGGGCGAGGCGCTCCAGC

ACATGCGCTCGCACTCGAACAACGTGACCCACTCGGGGTACAACGGCGGC

TACACCTACGGCATCGTCGCCCGGAACGAGGGCTTCACGCAGGCGTACAA

GCCGCAGGGGTGGGACAAGGTGCCTGACTCGCTCAAGACCGACGACGGCC

ACGTCACGGCGACCCGTCGAGTGACGACGGCCGTGACCTATCGGAAGGAC

ATCTACGAGGAACGCGGCCTCGACGAGCCGACCACGTGGGAGGACCTGCT

CCAGCCCGAACTCATGCAGGACCTCGCGCTCCAGACCCCGCAGGCGGCGG

TCGGGCTCGCGGCGGCGCTGTCGATTAACAACGCCCGTGGCGGTAGCATG

AACGACCTCCAGCCCGTCATCGACTACTACGAGCGGATTCAGGAGGGCGG

CGCGGAGTTCACCGACAACTTCCTCGCGCAGTTCTCCCGCGGGGAGTACG

CCACGTTCATCCGGTACGACTACTCCGGCCTCGACCTCAAGTACAACAAC

GACGAGGTCGCGGAAGAGAACGTCGGCGTCGCGCTCTTGGGCGGCGAAAA

CGGGAACAAAGGCGCGCTCAACATGCCGTACGGCTACGCGCTCTTGGAGG

GCGCGCCGAACCCGGCGGCCGGCAAACTGTTCATGGACTACGTCCTCTCG

CTGGAGGGCCAACAGCAGTTCCTCGACGCCTACGTCAGGCCGATTCGCTC

CTCGGAGCTGGAGCTTCCCGAGGAGTTCATCGACCCGGCAGAGTACGACC

GGACGGAGTTCCAAGTCGACTACGGCCAGTTGGTCGACCAGCAGGAGGAC

ATCATCCAAGAGATAACGCGCGGCGCGAACATCTGAGGATGTCCGACCGA

CACGCCGAGAACAGCTCGCTCTCGACAGTCCGGGCGCTCACGGCGCGAGT

CGTCGGCGCGGTCGAGACCGTCGCGAACCCCGTCACCGAACGGGAGCGCG

AGTACCGCCGCATCGTCGCGCTGTGCGCGCCGTTTGCCGCGTTGGTGCTC

GTCGCGGGGCTGTATCCCCTCTCCGAAGTCGTTCGCATCAGCGTCTCGAC

CGCCCAGTATCGGTCGGTCGGGTTCTCGCTCGACGCCTACGCGACGCTGT

TCGGGGACCCCTATTACCGCCGGGTGGCGTTCGACTCTCTGTGGCTGACC

ACGGGAACGACGCTCGTCTCGGTCGGGCTCGCCGTCCCCATCGCGCACGC

TCTCGAAAAGTACGACCTCCCCGGCGAGGACCTCCTCGTCACGCTCGTCT

CGTTTCCCATCAGCCTCCCCGGCATCGTCGCCGCGTTCATGATTCTGGTG

CTGTTCGGAAACAACGGCGTCGTCACCGCGGTCGCGGCCGCCCTCACCGG

GAGCCGCCCCACCGACCTCGCCTTTGCGGTCAGCGTCCCGGGGCTGTTCG

TCGCCTTCGTGTACTCGATGGTGCCGCGCGCGACGCTCATCCTCCGCGGG

ACGTACGCCGAGGTCGATTCGGCAGCCGAGGAGGCCGCCCAAGCCCTCGG

CGCGACGCCGTTCGAGACGTTTCGGTACGTCACGCTTCCGCAGATTCGAC

CGGGTATCACCGGCGCGCTGATTCTCACGTTCCGGACGGGGCTGGCGATT

TTCGGGACGCTCGTCGTCATCCAGACGCTCGTCGTCTGGACGCTCCAACT

CTCGCGGGAACTCGGCGTCGGCTACGACGTGCAGGTCGCCGGTGCGATGG

CGACCGTCTACTTCGCGTTCACGTTCCTGTTCACCGTCCTCGGACTTCGG

TACACCAGCGCGGAGGTCGGCATATGAGGCCGTCCCCGTCCCCGTCCCTC

TCGCGTCGGCTCGGGCGGTCTGTCGTGGCGCTGACCCTGTTCGCGACGGT

CGCGTTCCTCATCGCGCCCATCGTCCTCACCGTCGTCGCCTCGTTCGCCG

CGAGTTGGACCGGCGTTCTCCCCTCGGGCTTCGTCACCTTCGAGAACTGG

CGGGCGGTGCTGGGGCTCGCGGACACGATTGGTTCCCAGCGCAGTATGGG

ATGGAGCCCGCTGTTCAGGCTCCCGCTCGGCGGCGAGACGCTGACGGTGT

TGGTCCCGGCGGAGTTGGCGTTCAGCATCGGGCTGGCGCTCGGCGGCGTC

CTCATCAACCTCGTCGTCGGCGTCCCCATCGCGTACGCGGTCGCTCGCTA

CGAGTTCCCGGGCAGAGAGTGGGTCAACGCCGTCTCGGTGCTCCCGCTCG

TGCCGGGCGTCATCCTGGGCGTCGCGTTCCTCCGGGCGTATCCGGACCTC

TCGGCGACGAGTTTCGGGCTCATCGTCGGCTACTCCCTGTTGAAAGCGCC

GTTCATGGTGATGGCAGTCCAAAGCGAGTTCGAGTCGATGCCGCTCCGGC

AACTGGAGGAATCGGCGCGCTCGCTGGGCGCGTCGTGGCCGCGGACGTTC

CTGACGGTCATCGTGCCGAACGCCCGCTCGGGCATCGTCTCGGGAGCCAT

CATCTGCTGGGCGCTCGCGGCCGCGGAGTTCAACTTCTCGTACATCGTCT

GGGCGAACGGTACCCAACCGCTCGCGCTCTTCTTGCAGCGACACATCTCG

AACAGCTCGTTCACCAAGGCGGCGGCCGCCGTGTCGATGTTCTTTTTCAT

CATCGTCGCGGTCACCCTCCTCTTGCAGCGGGTCGGTCGCCGCGGCTTCG

ACATCAGAGGTAGCTAATGGCAGACGTGGCACTAGACGCGGTTCGGAAGG

ACTACGACGACACGACGGCGCTGGACGGCGTTTCGCTCACCGTCTCGGAC

GGCGAGATTCTGGGCGTCCTCGGCCCCTCGGGGTGCGGGAAGACGACGAC

CCTGCGAACCGTCGCCGGCTTCGAGACGCCGACCGACGGGACGGTCAGGT

TCGACGGCGAGGACGTGACGAACGTCCCCCCGGAGAACCGCAACGTCGGG

TTGGTGTTCCAGGAGTACGCCCTGTTCGACAACATGACGGTCACCGAGAA

CGTCGCGTTCGGGCTGAAGATGCGGGGCGTCGGGAAGCGCGAGCGACGGA

ACCGGGCCGCAGAACTGCTCGACATGCTCGACATCGACGAGATGGGCGAC

CGCGACCCGACGACACTCTCCGGCGGCCAACAACAGCGCGTCGGGCTGGC

GCGCGCGCTCGCCATCGAGCCGAACGTCCTCCTGTTGGACGAGCCGATGA

CGGGACTCGACGCCCAACTGAAGGCGCGCCTGCGGACCGAAGTCGGCGCG

CTCCTCGCCGAGTTGGACGTCACCGGCCTCTACGTCACCCACGACCAAGC

GGAGGCGATGGTGATGTGCGACCGCGTCGCCGTGTTGAACGAGGGTCGCG

TCGAACAGGTCGGCACGCCCCGAGAGATCTACGAATCGCCGGCGACCGAG

TTCGTCTCGGAGTTCGTCGCGCTCGACGCGCCCGACCTCCCGTTCGTCAG

CTGGTGACTCGGCCGTAGCGGCGCGAGTGCGACGGCGTCCGAGGAGAGGT

GCGGCAGTCGGTACACGCGGTCATCGCGTTCGAGGCGGAGCGTCTCGGTT

CCGAACAGGCGGTCGGTGGCGCTTCGGACGCGCTCCAGTGGCACGGCGTT

GACCGCCAGCAACACCGCCACTTTCGCTCGCCGCCTCTCGCTGGGAGTGA

GCATTGTCGATACAGGGACAGTTCACTCGGGACAGTTAGGTGGTCTGACT

CGCGTCGGGCGTCGGAGTCGCTCCCCGAGTCGTCGCGCGCCGCCGGGCCG

ATTCGAGCGCGGGTTTCAGCAGTTATATGATTGGATGCGTAAAACGACGC

ATCCGATGGCCTCTCCACGGTACGATGACGCCTCACCGTCGTCTCTCCCC

GCCGGCATCGGGTTTCTCCCGGTGCTCTCCGCGAACCTTCTCCCCCTCGT

CGGCGTGCTGTGGTTCGGCTGGAACCCCGAAACCCTCCTCGCGGTCTACG

CGCTCGAACTGCTGTTGTTGTTCCCGCTTTCGGGCGTGAAAGCGCTGTTC

GCCGGCCGACCGCCGACCTCGTCCCGCGAGGGCGGCGTGCTCAACCGGTC

CGAAAGCGACCTCGTGGATAAGCGCGGGAGCGTCACTATTCACGACCGAC

TCCCGCCGGTGTATCCCCGCAACGTCCCCTTCGCGACGGCCGTCGTCGGC

GGCGGCGCGTGGGTCGGCGTCTTCCTCCTCGCGCCGCTCTCGGAGGTCGT

CTCGGTGCTCGACGTGCTCGCCCGCCCGGAGGCGGTCGTCAGCGTCGCCG

CGCTCGTCGTCGGGCAGGTCGGCGAGACGGCGGCCGCGTTCCTCCGCGGC

GACCGATACGCGGAGCGCTCGCCGTACGCCCTCGTCGAAATCCCCGCTCG

CCAAGCGATGTTCCTCGCGTGCTTCCTGTTCGTCGTCGTCCTCGGCGGCG

CGACGGTCGCCCTCGCGGCGTTCGTGCTCGTGAAACTCCTCTTCGAGTGG

TCCGGCTTCCGCGCGGACCGGGGCGGCGGCCGGCTCACTAGTTGGCTCGC

CGGCCCCGACAGCGAGGCGACCCGCGAGGCGCTCGACGTTCCCGACGGCC

GACCCGCGGCCGAAATCGACGTGGACCGCCGTGCGGTCGTCGCGGCCGCG

GTCTGGCGCGCGGTCACGACGACGGGTCCGTTCTACGTCACCATGGCCGC

GATGGTCTGGATTGGCGGAACCGCCTTCGTCGCCGAGGAAGCCTCGCTCG

CGGTCTGGGTCGGATTCGGCCTTTTCGGACTCGCCCTCCTCGGGTTCATG

CTCGCCGGCGACGTGGTCGAGGACACGCTCGCGAGCGGGTGGCTGACCTA

CCGGCGGTTCGGCGGCCGACTCGTCGCTCACGACCGCTTGACCGGCGAAC

CGCAGTGGGCGGCGGCGGTCGGTGACTTCCGCGACGCCGAGGTCGTGGCG

GCCCGTCCCACAGACCGCTATCTCGGAACGCGAACCATCGCGGCGACGGT

CGGCCGGGGCGACGACGAGACGGAGCGCGCATTCGGGCCGGTCGCGGACG

CCGAGAGAGTCGTCGAGGCGTTCGACCTGCCGGTCGAGTCCACGACGCTC

CCGCCGCTCGACCGGCGCTTCGTCTGGGCCGCCGTCGCTTCGGTCGTCCT

CACGGCGGTCGGCGTCGTCACCGTGGTGGTCACCCCGCTCGGCCCGTCCA

CGAGTTGGCTGCTCGCGTTGTTCCTGTTCCCGGCGTTCGCCATCGTTCCC

AAGGGGTTCTGGAAGCTCGCGCACTCGTGAGGTGAGACGACTAGAGCGGT

TTGTGACGAACGAAAAGATGAGAAGCAGAAAGCGCGTGAGTGAACGAGTA

GCGTCACGACGAGGTCGCCCGCGACGGCCCGCGGGCGGACTACCCTCGCG

AATGTAACGAGTAGGCGATGAGCACCACGCCGCAGAACTGGAACAGCCGC

GTGACGAGCGTGAGCGGCTGTTGGTACTGGAGGCCGATGTAACCCTGACG

CAGGAGCACCGAGCCGACGAAGGCGACGCCGAACGCGACGGCCGTGAGGA

GGAACAGGCCCGCGGACAGCGCCCGCATCGTCCGGCTGTCGTGACGGCGG

TAGCCGCGATAGGCCTGATAGCCGACGTAGCTCCCGACGAGCGTCGACCC

GAGGGCGAGCGAGACGATAAGCCAGTCGAGGACGGCCGACAGCGTCGCCA

TCTCAGAGGTCCTCCCACATCCGTTTGAAGCGGTCGGCGGTGTCGGATTC

CCCCTCGCCGGGGAACGACTCGTCGTCGGTACGCGTCAGCGACGCCTCGA

ACGACCCGTCGTCTAGCGCCACGGAGAACTCGGAGAGCGTGGCGGTGTAG

ACGCTGTAATGGTTGTTACTGGTTCGAATCTCGGTCTGCTCGTCGAGGAG

TCCGTACGCTTGTAATCGTTCCACCCGGCGGTAGACCGTCGGTTTGGACA

TCTCACACTCGTCGGCAAGCTCTTGTGCGGACATTGGTTTGGTACTCGTC

GCTGCGAGGATGTCTCGGGCGTACTCGTCGCTGAGGAGGTCGAGAATATC

GCCCAGCGCCGGATCCTCACTCACGGTGGTCGAATGACTGCTCGTCGGAT

TAATAGGCCCACGGGTTTCCACCCCTGTTGGTCGCGGCGACCGAGTTGGA

GTCCGAATCGGGGCTGGAGCACGGCGGGGCTGTCGAGAGAGCACGATGGT

ACGGGTTAGTGCGTCGATACTGCGGGGTCGTACGGCGATGCTGTGGAGGG

GCTGCTGCGTCAGCGATGACCGCGATGGGTGCCGGGGTGCTGTCAGAGGG

CACAGTCCGGTACTGCTCGGGAGGGCAGTCCGGCGTCAGTTCGGCGGGGC

CCATCGCAGTTCGACCGACGCGCCGTTAGCCCATATAAAACGGGGGCCAG

TTTCTGACCCAGAAACTCGCCCGTTTTCAGGCGGGACAGCCGACCGAGCG

ACCGACTTCGACGAGGCGGTCCGTCTCGACGCCGGTCCCGCGGACGGTGT

ACCCGTAGCCGTCGCAGTTCCACGACACCGAGGTGGTCGGTCCGTAGTCG

ATAGTCGCCGGCCGGCCGCCGACCGTCGCGTCGCGCTCGTCGGCGTCGAT

GTCGAGGGTGTAGTTGAACTTCGCGACCGTGAGTTCGCGGCCGCCGGCGG

CGTAGCGGAGTCCGACCCCGTCGATGCGGCCGGTGGTCCGCGTGGCGTAG

ACCAGTTCGTACGCGGACGGAACCGTCGGCTCGGGAACCGAAATCGAACT

CCGCGCCACCAAGTCGGCCCTGCTCCGGAACCACGACGTGTTCGGCGTCT

CCTGACGCTCGACGGTCGTGCCGGCGTCGATGTCCGGGCGGAACGTGTCG

GCCGACACCGCCGCGTCGAACGTCACGTTCGTGTACGTCGTCGTCACCGA

CCGCCGAGTCCCGTCGGCCGTCCACGCCGTCTGCTTGCGAAGCGGGTAGA

ACCGCTCGGTGTCGAGCCACAACCGCTGGCGGTAGCCCGCCTCGCTCCGG

TTCGTCTCGGGTACGATGTCGAGGACGTACGCCTCGCGCCCGCCGACGGT

GTCGGTCTCGACGAACTCGACGGTGTAGCTACGGTTCGCGTCGGCTCCGG

GGGCGACACCGGCGTGCCGCGGTACGACGGGGAGCGGTGACACGCCGATG

GACTGCGGACGACCGGCGTCGTCGGTCAGCCCCGCGGCCGCGACGAGCAG

TTGGAGCCGTTCGGCCGTCCGCGACTCGGTCGGCGGCCCCGTCAGTTCGA

TGACGGTCACCGCATCCCTGTCTGTCTCGTGAAGCCAGAGCGTCGACCCG

TTCGAGACCTGCCGGTCGTACTGTCGATTCGCGGCGTTCCGGAAGTTCAC

GCGCTTCCGTTCTGTCTCCGGGACGAGCGTCACGTCGGCGACGGTCCGAG

ACGTAACCGTGTCGTCGGCTCGGACCTCGACCGTCTGTGTTCCGGTGAGC

GCGTCGATGCCGCGGTAGCGCTCGGTCACGTTCGCGTCGACTGCGGGGCT

CGCGTCGCCTATCGCGCCCCCCGTCGTCCAGAGGACCGCGGAGAGCGCGA

CGGCGACGAGCACGAACGCGGCGAGCGTGCGTCGGTTGAGTCGGAGTGGA

GGGCGGGTCATACTGGAAGCACTCTGGCGGAAGTTCGACTGCGTGTCGGA

GTGCCGACGGCTAAATACTTCTGGACGCCGGGCCGAGCGCCCACCGGACC

GATAGCTACACGCGCAAGGCGCGAGACGGGTCTGATATGTCGCCCTCCAC

GACGCCTTCGACGCGAACGCGGGCGTTGGTCTGCGCCCTCCAGTGGGTCT

GGTGCAGACGTTCGTCGCCGAGACGACGCTCGTTGCTCGACTCGCTGACG

GGTGACTCGACGACCGGTCGACGGCGCTTCCGACCGTACACGCCTCGAAC

GCCGGGGAGTTTATGCGCGTGCGGATGGTACTCAACCGATAATGTCAAGC

GAGCGGAGAGAGCTGTTCGAACACATCGAATCCGATCTCATCCTACGAGA

GTTCTTTCAGGAGGTAACTGAATACGCGATATTTCTCCTCGATGCGGACG

GATACGTCGCAACGTGGAACGAGGGCGCGAGACGAATCAAAGCGAACCGA

AAAGAGCGCGAAGAGCGACTCATGGTGCTGAATCGGATGCTTCGGCACAA

CCTGCGGAACAATCTTAATATCGTCAGCGGGTACGCACGAGAGTTGCGCC

GAGACCTCGACGCGGTCGACCTCCCGACCGAGAAGGGCGACTACGACCAG

TTGGCCGAGACGGTGACGAACCTCAGCGAGACGACCAGCGGTATCGATGC

CGAACTTCGAGCGCTCGACGAGATAGTCACCGCGGCGAGTCAGTTTTCGG

TCGAGGCGATGGTCTCGAAGACGGACCGAATCATCGAGAACGCCGACGAC

CTCATGTCGACCGGCGAGAAGGCCCGGCGGTTCGAGAACGTCGTGGAGGA

ATCCACCGACAAGCAGCCGGTCCGCGTCGACGACGTGTTGTCGGATATCA

AGCGGACGTACGAGCGGCGATATCCCGATGCGACGATTCGATTCCGGGCA

GGCGAGCAGACCGTGCTCGCGAGCGCGCACTCGCTTTCCCTCGCGCTCGA

CGAACTCGTGCAGAACGCCATCGTCCACAACGACGAGGATGACCCCGTGG

TCACCGTCGCGTCGTGCCGCATCTCGGGCGAGCGAGTCGAAATCACGGTC

GAAGACAACGGGCCGGGAATCCCGCAGACGGAACGCGAAATCTTGGAACG

CGGGGAGGAAACGCCGCTGTCGCACGGGAGCGGGATGGGACTGTGGACCG

TCTCCTGGTTCGTCACGCAGTTCGACGGCGAGGTTTCTATCGTCGACCGC

GCCGGCGGTGGGTCGACGGTCGAAGTCAGGCTCCCCACGCCGGAGCACTG

ACGCGGCTGTATCGGCCTCGCCGCCCGAGAACGCGGGGCGACGGCCTCAG

ACCGACCCGATGTCCTCTTTTTTCCGCATGAGGTAGAGGAAGTACGGTCC

CCCGACGAGGCCGGTGATGATGCCGACCGGGAGCTGAATCGGACTCAGCG

CGAGTCGCGCGCCCACGTCGGCACCGACGAGGAGCGCCGGCCCGAGGAAC

GCACAGCCGATGAGGAGCCGCTTCGAGTTGCTACCGACGAGGTTCCGGAC

CATATGCGGGACGATGAGGCCGACGAAGCCGACCAGTCCCGCGACGGCTA

TCGCCGCCGCCGTCGAGAGGATGGCGATACCGGCGACGGCGAAGCGGACC

TTCTCGACGGGCATGCCGAGCGACTGCGCAGTCTCCTCGCCGAGGAGCAG

CACGTCGAGTTCCTTGGTGACCGCGAGCGCGAGTCCGAGCGCGACGATGG

TGAACGGGAGCGCGATACGCACCTGCGCCCAGTCGGTTCCGAGCAGCGAC

CCGGACAGCCACGCCTGCGCGGACATCACGACGCCGAGGTCGTTGATGAA

AAAGAACATCGCGCGCTGGACGGAGCCGAAGACCGTCCCGACGACGACGC

CCGCAAGGACGAGTCTGACCGGCGAGGTGCCGTTCTTCCACGCGATGAGG

TAGACGAGGAGGAACGCGATTGCGCCTCCGACGGCGGCGAGCACCGGCAT

GAGCGGGAGCAGTCCCGACAGGAACGTCAGCGTGACGAGCACGACGAGCC

CCGCGCCGTCGCTGATGCCGAGGACGTACGGACTCGCCAGTTCGTTTCGG

GTGACGACCTGGAAGATTGCGCCCGAGACCGCGAGGTTGGCACCGACGAG

GACGCCGACGAGAACCCGCGGCAGGCGAATGTTCCAGACGACGAACTGGC

GCTGGGTGAACCATTCGGGGATGTCGCCGCCGAGCAGGAACGCACGCCAC

ACGTCGGCGTCGAACACGACCGCCGAGTCGAAGACAGCACCCCACGCTTC

GGAGAGGGTGAGGGGGTACGCGCCGAAGCTCACCTGTAAGAGCGTCGCCC

CGAGGAGGACCGCGAGGCTGCCGACGATGACGAGGGCGAGCATCGAGTCG

GCGTACCGGTCGCGCCACTCCGCGCGAACCGCACCGACCGCGTCTGTAAC

CTTGCTCATCGGGCGTCAGAAGTTCCCGTTGACGATGTCGACGAGGCGCT

CGCGGTCGAACAGCTGTTCGTCTTCGGGCACGTCTCCGACCGCACCCGGC

CACTCACCGAACTGGTCGGGATACACCATCTTGGCGACGGCTTCCGTCGA

GAACATATCGACGATTGGGCCCATGTACTGCCCGGCGCTCCGGATGAGGT

TGCCCTCCTGAACGGCCGTGAGCCGCTGTCCGCTCTCGTTGTTCTCGAAC

GGCTCGACGACGGTGTCGACGAACTCCTCGCGGGTCGAGGAGGTGAGCGC

GCCCACCGCGCCGATGTAGTCGGGGTCGACGTTGATGAGCTCCTCGTAGC

CGATGGGTCCCTCGGGGTAGCGTCCCTCGAAGGCGTCTTTCATACCGACC

CGGAAGTACGACTTGGTGTTGTTGCCGAGGCGGTGGAGGGGCGCGACGCG

GAACTGGCCGGAGTCGGGGCTGACGCCGCGCCAGATGGCGGCGACGGTGG

GGCGTTCGTCCTCCGGCGGGAGACCGGCTTCGATAGTGCTCATGAGGTCG

TCGTGGAACGACTGCCACGCCTGGAACTGCTGTCGGCGCTGGAACACCGT

TGCGACCTTCTCGAACGCCTCGTAGAGCGTGTACGGATTCGGCTCGTTGC

TGACGGCGGCCGTCGGGGCGAACCGGAGAGTCGTGCCGAGGAAGGGTCCG

GTCTGGGATTCGACTTCCTCGTAGTCCGAGGCGTCCCAACTGACGTAGCG

GCCGAGCGTGGCGCGGGCGATGAGCCACACGTCGGCGTCGGCGGCGTAGA

AGTTCTCCTTGTCGAAGCCGGATTCCGCACCGCTCGCGAGCTTGGTGATT

CGACTCTCGTCGAATTCGAGCCCGAGGAGGTCGTAGAACTTGAGCGGCGC

GCGTTCGAGGCCGGTGGTCGCACTCGGATGGATGCCGAGCGCCATCCCCA

TATCGAGGAAGGGCCCGGAGGCGACGCCGTACGTCTCCGGCACTGCTTCG

AACGTGTACGGGTCGTTCGGTTCTATCTCGACGGTGTACTGCCCGGCGCT

CTCGGTCGTCTGTTCGGTCGTCGTCGACTCGGTGGCGGCCTCGGTTTCGG

ATTCCGCGGCGGCCGTCGACTCGGAATCCGCGCTCCCGCCCGCGGTACAG

CCAGCGAGAGCCGATGCGAAGAGTCCTGCACTTCCTGCGAGTATCTGTCG

TCTTCGAACCATATTATTTAGGCCTGCCTAAAGACAGATAACCGTTTCGA

AGTTTAGGCAACCCTAATAACGGGGTGTCGCACGACAATGTCGGGACACA

CGCATATTTCGACCGTAGATATAATGATTATAACTGTGGTATCACCCATC

TCTAATCCGGGTTGTCCAGTTAGCGAATATCCGAATTATCAGATGGGGTT

ACTAACAACTCTCGCGTCGGTTTTCGACGCTCGCGTCCGCGGTGACGGGC

GTTCGGTCGGTCGTGTTACGTCGTGCTGTCGCGTCGAATGGCTCGTCCCC

TCCCGGGAGTTAGCCGGTGGACCGGTCGCAAACTGCTGTCGAAGTTGGGC

TAATCCGTTCTCGCGTTTGTCGTCACCGCGCCGCCGTCCAGTCCAGCGCG

AACGGTGCCAGTCTCAACCCTCCCCACGGAGTGTATTCGACCGACCACAC

CTACCCCATCTGAGTAATCCGAATGATTTGAATTTCCCGAACTACCTCGA

TTTGTGAATTATGTGGATTGTCTGGAGTACTTCGGTAGTGTCGATACTGT

CGATACGGCTCCATCGAGTCGATACATAATTCGACAGTAGGCCATTATCG

ACCAACTTTACAGTATTTAGATACCCTATCTAAGTCACATACTTCACATT

ATCTGAATTACCCACAGTCTCGGAGTGTATCACCACGGCCGCGGATTCGG

GCGCGTCGGAGTCAGCTGTCTAAACGTGCGTAACAAGGATATTGTAGATA

ATCCAAATTATCTAGATTCGTCGAGTAGTTCAATTAACTGCAATTCTGTG

GCTCGTTCGTCGTCGTCGCAGTATCCACCCAGATGAGAGGAGATACATAA

TCGGTCTACTTCGGCTTATCATTATTCGTCTCGTTATTCAGATTATTTCG

CTATCGTAGGTTACCTCTATGGCCCACAGTCGAGATACTGTTCCACATCC

AGATACTGTAAATTGTTTAAATTATCAGAATTGCTCGGATGTCCCCCCGG

AACTGCACATCGTTGAGGAATCCGGTGGTGTTCGATTTTCCGGAAAATCC

TACTAGCTCAGAATATCGAAAGAATCCACGAAATCAGGATAATTTAGATA

ACTCGACAAACCCGAATTACGACCCGCCGTTCGATTTAACTCGGTCTCGG

GAGTGAGCACAGGCGACCGTGACGACGCACTTCGACGACTTCGACCACGA

GAGACACATGCGCGAGAGTTTCGAACTCGCCCGAAAGGCGGCCGCTCGCG

GCGACGAACCGTTCGGGTCCGTACTCGTCCGCGACGACGAGGTCATCATG

CGCGACTCGAACCGAATCGTGACGGAGTCCGATATCCGACGCCATCCCGA

GTTACAACTCGCCTATCGGGCGTGTCGGGAGTACGACGCCGACGAGCGAG

CGGCGATGGTGATGTACACCAGCACGGAGCCCTGTCCCATGTGCGCCGGC

GGAATGGCGACCGCCGGGTTCGCGCGCGTCGTGTACGGTGTCGGCGGCGA

CGAAATCGGGGAGTTCACGGGTTCGAATCCCGGCGTTCGGTCGGCCGCAG

TTCTCGACGCCGTCACCGAGGTCGTCGGCCCCGTGCTGAACGACGAGGCC

CGGCGGGTTCACCGGGAGTACGAGTGGTAGGAGCCAGCCAACCACCGCGA

CGAACGCCGACTCAACCGGAGCGCGAACGCCCGGAGCATCGGGCGTTTCG

AACAGTCAGAGATTCAATAGTTCATTCGAACACCCGTATCAAATATCAAT

CACAGCTACATTCACAGGTCGACACCGTATCGACGAGCGTTCGGGCCCAC

TCGCTGAATCTGAATGTCGATACCGGCGGCGTATCTCCTCTAGCTGTATA

TTCACGGCTGACGAGGGCGCGTTCGAATCGAGCGACGTGTTCGGAACCGC

TCCATCTCGTCTCCGAATCTCTCATTCGTGCACCAAACGGCTGTTTGTGC

TCTGTAATGGGTGTACTACTCAGTTCGGCGGTCGATGAGGTACGTTCGAC

GACGGCCGTCGTTCGCAAGCCGAGCCTCCTGTATCTGACTACCTAATTGT

TCTCTTAGTAATATTGAAAATTGATACGAACGGGCAACTCGGTGAAGTCA

CGTCAAGCTACGGACCGACCCGTCGTCGGCCGTTATCGAGACGAGACGTT

GCGCTCGGACTGAGCGACAACGAGTATTTACATAATTCGAACGATGGTCT

ACCTCACGGCGAACCACCGCAAATCATTACAACCATATGCTTGTAATCGA

TTTTCACTTAATCCCATATATCTGTATAATGTGCACACATGACACCATAC

ATATTTACATAATGTAAGTTAGTGATGATAGTGGTGATAAGGCGGTGCGG

TGACCGAACGGTGGAGTGTATCGTCGGTCGCACTCGTCGGGTGAGCTCAG

GAACGAAATCACGGCGAACCGCCCGAGTTCGACTCGAAGTTGGGCGAGTC

TAAATATTTAATATGTTTCGCAAGAGACGTTTCGACTGTCGATATGAGTA

GCGACAGAAACTACGAACGAGAGTTCGTACGGACGTTCTTCACCTCGCCG

ACGGCGGTCAGCGGCGAGAACTCGGCGAAGATGTTGCAGAGCGCGGCCCA

ACTTCGGGGGATGCAGGCCCCCGACACATGGATTCCGGACAACGAGGACG

CCACGGCACCGTCGATGCGGGCCGAGGGCGTCCAGAACCTCGTCGAGGTC

GTCTCCGAACACGGAGCCGACTTCCCCGGCGAGATTCACCCCCGCGTCGT

GTGGCACCGCGACGACCCCTCGACCCGCTACGAGGGCTTCAAGCAGATGC

TCGAAATCACCGACCCCGAAAACGGTGCGGTCGAGCACATCGACGGGTTC

GTCATCCCCGAGGTCGGTGACCTCGACGACTGGAAGAAGGCCGACGAGTT

CATCACCATCATCGAGCACGAACACGGTCTCGACGAGGGAAGCCTCTCGA

TGTCGGTCATCATCGAGAGCGGCGAGGCCGAACTCGCGATGAGCGACCTC

CGCGCGGAGAAGGGCAAGCCGTCCAACAACCTCGAACGGCTGTTCCTCCT

CGTCGACGGGCAGGTCGACTACACGAAGGACATGCGTGCGATGACGCCGA

CCGGCGAGCTTCCGGCGTGGGCCGAACTGCGGCACAACACCTCCCGCGGG

GCCAGCGCCGCCGGCTGTATCGCCGTGGACGGCCCCTACGACGACATCCG

CGACGTGGAGGGCTACCGCGAACGCATGGTCGAGAACCGCGCCATGGGGA

TGACGGGTATCTGGGCGCTCACGCCCGGGCAGGTCGTCACCGCAAACGAG

GCACCGCTGCCGCCGAAGACCGGCAGTTGGCTCCTCGAACTCGACGACGA

CGAAATCGAACTCGACGCCGAGGACGGTCGACAGGTGTACGACGGCGACG

AACTCTCGCTCGAACAGGTCGGCGACGACAGCTACGTGCTCCGCGTCGAC

GGCGAAGAACAGGAACTCGACGCCGAGGAACTCCACGAGGAGCTGCTCGA

CCTCACCACCTACGTCCCGAGCATGGACGACATCGTCGACTCCATGGAGG

AGTTCGAGGCGGCCAAGGAGGCCGGCAAGGGCGCGATTGCGATGACGCAG

GCGACGACGCTCGTCATCGACGGCGTCGAAGTCGACATCGCCAAGGACCG

CATGTGGGACGAGGCGACCTATCAGGCCGCGATGACGCCCGTCGCCCTGT

TCCAAGACGTGTACGAGCACCGCCCGGACCAACACGACGCCCTCGAGGAG

ATGTACGGCGAGGGCATCGTCGAGCGCGCGATGGCCGTCGGGACGGACGA

CTAACGACCAGCAGTCAGCCTGACAACGCCAGCCAACCCGCACCCGCCAT

TTTCGCGCTTGGTACCTGAGACGGCGGTGTTTGCCTACGAGAGCGCCGCT

TCCAGCGACTCGACGGCGTTCAGCAGTTCGTCGGCGAATTCGCGTTCGAG

TCGCGTCATATCCACCTGATACTTCGGGCCGCCGACGCTGAGGCCGCCGA

CGATACGCCCGTCGCCGTCGTGGACCGGCGCGCCGACGGCGATGAGATTC

GGCGCGAACTCCTCGTCGACGACGGCGTACTCCTGAGTCGCGATTTCGTC

CAACGCGTCGAACAGCCGGTCGGCGTTCGTAATCGTACCCTCCGACTCCC

GCGGCAGCCCCCAGTGGTCGACTATCGACGCCACCTCGTCGCGGTCCAGT

TCCGAGAGGATGGCTTTCCCCGCGGCCGTGTTGTGCAGATGGTACTCCGA

CCGGTAGCGGATGCCGTTCGCGTCCCGTCCCTCGTCGGTCGACGAGCCGT

AACACATGAGCAGCCGACCGTGTTCGAAGATAGTGAAGTTCGCCTCTTGG

CCGGTCGCGTCGGCCAGTTCGTCGACGACCCGCTCGACGACCTCGTCGCT

CGGATACCGATGTCGGGCGCGCTCGCCCAACAGCGCCACCCTGAAACTGA

CGCGGTAGACGCCGTCGCGCTTGACCAGATACCGACTCTCCAGGAGCGTG

TTGAGGTGACTACAAATGGAGCTTTTCGAACTGTCGACCATCGTGTCCAG

TTCAGCGAGTGTGAGACCGTCGTGTTCCAGAATGAGGCTGAGAATCTCCA

GCGAGGCCTGGGTCGTCTTGAGCCGTCGCCCGGTCACGTTCTCAATCATA

CACCAATTATTATCATGATGGTTCATAACTGTTGCCATAATGTAAATCAG

CTGAACTGCCCCGCCAGCCCGGTCAATCCGGGCATGTTTCTCTCGTATTC

CCGCGGGAACAAACGAAATAGAACGGTGGGATGCGTCGAACGCTGTTCGG

TTCGCGTGCGTCGTGGCTGTTCGGTCGGCCGTCAAATCGCCCGCGACCGT

TTCACCCAGCCGATTCGCGTGATTGCCTCCGCGCCGAGTTGTCCGAGGAG

GCCCGCGGCTTCGAGGTCGTGTTCCTCGAACGCGTCGACGACCACGTCGC

CGGCGCTGAGCACGCCGTGGCTCCCGATGGGAACGTACAGCCCCGATTGG

AGCGCGCCCCGACTGTGGTCGTCTTCGGTGTGCTCGTGGTTCGCGTGGAT

GACCGGCTCGCCCGTCCGGTACGTCCGCGCGGCCGGCGTGTCCTCGCTGA

CCGAGTAGTTCGGCCGGTCGCCGGCGCGTTCCAGACACTCCTCGGTCGCT

TCGATGGTTTGGAGCATGTTCCGGTTCTCGTCGACCAACCGGACGGTCGT

GTTGACGAGGCCGAGGACGTTCTTCGCCGCGTCGGCCAGTATCTCCGCCA

CCTCCGCCTTCGAACTCGCGTTCCGTAACTCCTGGCTCGTCCGGCGAAGA

ATCTCGATGCGCTGTTCCTGACGCTTCGTGTCCGTCACGTCCCGCACGAC

ACCGTAGAGCGTGACCGTCTGACCGTCCTCTACCAGCGGGTCGCACGAAA

GCCGTATCGACCGCTCGGTCGTTCGCTCGCTCGCGTACCGCACGTCGATA

GCAAAGGGCTCGCCGTGTTCGATAGCGTCGCCGAGGCGAACCAACACGCC

GTCTTGGTCGCCTTCGGCGTACTGCTCGACGACGTCCGAGACGGTCGGCG

TCGCCGCCGCGTCGAACCCGTGTATTCGCTTTGCCTCGTCGCTCCACCAC

AGTTCGTCGCGCGACACGTCGTAGGACCACACGCCGATACCGGTCAGTCG

CTGGAGGCTCGCGAGCAGTTGGTCGACCTCCGTCTTGGACGCCGTCGGGA

TGGCGCGCGCCACGTCAGTTTGATTCCAGTTCGGTGACCTGACCATGAAT

GAGGATACGTACCGAGGACGCGACTATAAATCCCCACGTCGGTCGTGAGG

GCCGTTCGGGGGACGAGAACGTGTCGCAACACCAGAGGATTTAATCCGTG

CGTATTTGTATCATTGATGATGGCAGTTGAAGACGTGCTCGACTCCCCAC

GGGGAGGAGAGACATCGGCGACGACGCTCGACGTGGACCGTCCGGACGCG

ACGGCCTACACCGCACTGGCGACGGAGTTACGGGACCGCGTCGACGGCGA

CGTGAAGTTCGACGAGTACGCGCAGGTGCTGTACGCGACCGACGGCAGCA

TCTATCAGGCGCGACCCGCGGGGGTCGTCCTGCCGAGGGACGCCGACGAC

GTTCGCGCCACCGTGGAGACCGGGGCAAACCACGGCGTCCCCGTTCTGCC

ACGCGGAACGGGCTCCTCGCTGGGCGGCCAAACCGTCGGACGTGGCTGCG

TCGTCATCGATTTCACGAAGTACATGGACGATATCGTCGATATCGACCCG

CAGGCGAGGCGTGCCACCGTCCAGCCGGGGTGCGTTCAGGACCACCTCGA

CGCCGCGCTCGCGGACCACGGCCTCAAGTTCGCGCCGGACCCCGCCTCCT

CGAATCGCTCGACCGTGGGCGGCGGCATCGGCAACAACTCCACGGGCGCG

CACTCGGTCCGCTACGGCATCACCGGCGCCTACACCGAGGAGTTGGAAGT

GATTCTGGCCGACGGCTCGCTGATTCACACCCGCGAGGTCGTCCTCGACA

GCGACGAGTACGACTCGATTCTCGCCCAAGACGACCTGGAGGCGAACATC

TACCGGACGGTCCGGGCGCTCGTCGAGAACCACGAGTCGGAAATAGAGAA

CAAGTATCCGAGCCTCAAGCGCTCGGTGTCGGGGTATAACCTCCAGAAAG

TCATCTACGAGAACGACGACGGCGAGGCGGTCATCAACCTCTCGAAGCTG

TTCGTCGGCGCGGAGGGCACCCTCGGCGTCATCGTCGAGGCGACCGTCTC

GCTCGTCACCAAGCCGGACGAGACGGCGCTCGCGCTGTACTGCTTCGACG

ACCTCGTGGACGCGATGGAGGCCGTCCCGGAGGCGCTCGAATTCCCCGTC

AGCGCCGTCGAACTCATGGACGACGAGGTGTTCCGCCTCGCCCGCGAGTC

GGAGGGCTACGCCGAGTACGCCGAACCCATCCCCGAGGGCGCGAAGGCGG

CGCTGATGCTGGAGTGGGACGACGAACTCGTCGACGACTTCGAGGCCGCG

GTCGCGGACACGAACGCCCACTTCGTCGACTCCGGCGCGGCGTTCGACGT

GCTCGAAGCCTACTCCGAGGAGAGCCAAGGCCGACTGTGGAAGCTCCGCA

AGGCGGCGATTCCGCTGTTGATGAGCCTCGAAGGCGACCCGAAGCCGTAC

CCGTTCATCGAGGACGCGACCGTCCCGCCCGAAGAACTCGCGGAGTACGT

CCAGAAGTTCGAGGACGTCCTTGAGAACCACGGCACGTCGGCCGCGTACT

TCGCCCACGCCGGGTCGGGGACGCTCCACATCCGCCCGATTCTGAACCTC

AAGGAGAACGAGGGCATCGAGAAGATGCACTCCATCACCGACGACGTGAC

CGACCTCGTCGTCGAACACCACGGGGCCTTCTCGGGCGAGCACGGCGACG

GGATGGCCCGGACCGAGTTCAACCCGAAGATGTACGGACCCGAACTCTGG

GGCGCGTTCAAGGAACTGAAGACGGCGTTCGACCCCGACTGGCTGTTCAA

CCCCGGAAACGTCGTCTACCGCGACGGACCCTCGGACCCCGGCCCGGACA

GCGACCGGGGCGTCGGCGCGGACATGCGCGAGAACCTCCGCTACGGTCCC

GACTACCAGTCCGTCGAGCCCCAGACCGACCTCGACTTCGACGACGAGGG

CGGCTTCTCGCACCTCGTCGAACTCTGTAACGGCTGTGGCACTTGCCGGC

AGACCGACTCGAACACCATGTGCCCGACCTACCGCGCCTCGAAAGAGGAG

GTCCAGACCACTCGCGGGCGCGCGAACATGCTCCGGGCCGCCATCTCGGG

CGAACTCCCCGAGGACGAACTGTACTCCGAGCGCTTCCAGACCGAGGTGC

TGGACCTCTGTGTCGGCTGTAAGGGCTGTCAGAGCGACTGCCCGACCGGC

GTCGACATGGCGAAGCTGAAATCCGAGGTGAAACACCAGTACCACCAGAA

AGAGGGCACCAGCCTCCGCTCGCGGGTGTTCGCCAACGTCGACACGCTGT

CGAAACTCGGGAGCAAACTCGCCCCGCTTTCGAACGTCGCCGCGGAGCTT

CCGGGGAGCCGTCTCCTGCTGGAGAAGGTCCTCGGCATCGCCAGCGAGCG

CGACCTGCCGAGTTTCAGCAGCGAGTCGCTGGAGGAGTGGTTCGAGGCCC

GCGGCGGGTCGTCGGTGCCGCCCGCCGAGGCCCGCGGGAAGGTGCTTCTG

TTCCCCGACACCTACACCAACTACAACTACCCCGAACCCGGCAAGGCCGC

GGTCGAGGTCCTCGAAGCCGCGAACGTCCACGTTCGGATTCCGAGCGACC

TCGAATCGAGCGGTCGCGCCGCGTTCTCGATGGGGATGCTCGATACGGCC

CGCGACCGAGCGCGACACAACGTCGGGCGACTTCGCCCCTACGTCGACGA

CGGCTGGTCGGTCGTCTTCGTCGAGCCCTCCGACGCGGTGATGTTCCAAG

ACGAGTATCTCGACCTCCTCGACGGGACGGCCGTCGAACTCGTCTCGGGG

TCGGCCTACGGCGTCATGGAGTACCTCGACGCCGCGCGCGCCGACGACCA

CCTCGACTTCGACGCCTCCGCCGAGACGCTGACGTACCACGGCCACTGCA

ACCAGAAGGCGACGAACAAGGACCACCACGCCGTGGGCGTCCTGCGACGC

TCCGGCTACGCCGTCGACCCGCTCGACTCCAGTTGCTGCGGGATGGCCGG

GTCGTTCGGCTACGAGGAAGAGCACTACGAACTCTCGAAGGGCATCGGCG

AAATCCTGTTCGGACAGGTCGACGACAGCCCCGGCGACGTCGTCACGGCA

CCCGGCGCGTCGTGTCGCTCCCAACTCGGCGACAGACCCGGCGAGTCCCG

CCCGCCCCACCCCATCGAGAAGATGGCCGAAGCCCTCGACTGACCACGCC

ACCGACAGCCGACAGCCGATAGCCTGACGCGCTCCCCCCGAACCAGCCAG

CCTGACGCGGTTCACGAACCAGCACCGAACTCGCCGCCTTGCACCCGCGG

CGACGTTTCGTGCCGTCAGTTCCCACCCGCGGAGCCGACGTTCACGGCCG

GCGATACTTATTACTGACAGCAGCGGCTGCGTAGAGACGACGATGCTAGA

CGTACTGGAGACCGTCGAACTCTCGACCGCGTTTTTCGCCTCGCTCGGCG

CGCCGGGGCTGTTGCTCGTGGCGTTTCTCGAATTCTTCTTGCTACCCGTC

CCGCCGGACTTGGTGCTGGTCCCGCTGTCGGCGACGAACCCCGAGTTCGC

GCTCCCGTACGCAGTCGTCGCGACCGTCGGGTCGGTGTCGGCGGGGCTGG

TCGGCTTCCTCATGGGCAAGAAAGGCGGCCGGCGCGCGCTGGACTCGCGG

TTCGCGGGCGAGCGGATTCACAAGGTCGAGCGATACTTCGAGCGCTCCGG

GTTCGTGACGGTCGCGTTCGGGGCGTTCGCGCCGATTCCCGAGGGGTACG

AACTGCTCTCTATCGGGTCCGGCGTGTTCGACCTCGACCTCCGGACGTAT

CTGGCGGCGTCAGTGCTCGGGCGCGGCAGTCGGTACACCATCGAAGCGCT

GCTCGCGGTCTATCTCGGCGAGGCCGCCCGGTCGCTCACCGAGGTCGACG

TGTACTCGATTATCGGCGTCGCCACCGCGGTCGTCCTCGTCGCGTACGTC

GTCCGCAATCGGTGGTTCTCGGACCGCTCGGCCGAGCCGGTGCAGTAAGT

CGGCGGAACCGCGGTCAGGCGACTTCGTCGCCGCTTCGCCACTGGTCGAT

GACTTCGAGCAGCACCTCGGTTTCGAGGGTTTCGTTGCGCCAGTCACCGA

CCGCGTCGGTCAGCCCCGTCGCGTCGACGACGTTCTCCTCGTTCGCGTAG

TCCGCGACGGGGTTCTCGACGACGAACGGAATCATCATCTTGTTGTCCTC

GTGTTCGAGCATGTGGCAGTGCCACGGGAACTGTCCGGTGTAGCCCTCGA

ACGTCACGAGTATCCGCACGCGTTCGCCGGGGTCGACGCGGACGGTGTCT

TTCGGCCCAAGTTCGTTGGGGTCCGGCGGTTGCGTCCCGTCGGGTCCGCG

GCCGATGACTCTGAACGTGACCAGATGCAGGTGAATCGGGTGTCGCCCGC

CCGACTCGTTTTGCAGTTCCCATATCTCGGTCGCTCCGAGTTGCGGGTAG

ACCGGCGCGTCCTCGTCGCCGAAGACGTGGCCGTTCAGCGTGTGCGTGAT

GAGGCCGTTTCGAACCTCGGTCCCGAGCGTCATCTCGCGGGTCACCCGCG

CGTCGCTCTCGTCGTACGAGGCCGGCGTCGGAAGCGACAGACTCGTCGGG

TCGGCGCTCGCGTCCTCCGGCGGCGTCGACGGGTCCGAGACGCGGAACTC

GACGAGGTCGGTCAGTTCGGGACCCATGTCGGCCCCGTTGGCGAGCGTGA

GCGTCTCGCCCGCGTGGTCGGAGAAGTCGACGACGAGTTCGCCGCGCTCG

AACGGCGTGAGCAGCAGGGAGTCGAGGTCGCCGTTCGGGCCGATGGGGAC

GACCGATTCGAGGAAGCCGTGACCGGGGGCGAACTGGTACATCGTCGGGA

CGCCCGACCCGCTTTCGCTCTCCAGTTGCAGGTCGAACGAGCGGTGATTC

GCGCCGTTCAGGATGCGGAACCGGTACCGCCGCGGTTCGACCTCGACGTA

CGGCCACACGGCCCCGTTGACGACGGCGGTATCGCCGAGGAACGCCGAGA

CGAACTCCTCGGGGTAGTGCAGCGAGCCGTCGTCGTTGAACTCCTTGTCT

TGCAGCAACAGCGGGATGTCGTAGTCGCCCGACGGCAGGCCGAGTTCGCG

CTCCGCGTCGGTCGTAATCGAGTAGAGACCGAGAAGCCCGGCGTAGGCGT

TGAGTCGCGTGATGCCCAGCGTGTGGTCGTGGTACGTCGACGTCGTTCGC

CCCTGTTCCATCGGAAGCTCCTGCCACGCCGAGTCGAACCGCGGTCCCTC

GACCCCGCCCGGCGAGGTCCACATATCGGACTGCCCGTCGTTCGCGGGGT

CGAGTTCAAGGCCGTGGAAGTGCGTGACGGTCCGAACCTCCGGCACGGGA

CCGTCGTAGCCGGGGTGGTTCTCCGCGGTCGTGCCGCCGAGTCGGTCGTC

GACGGGAAAGAGGTGTTCGCTCGGGAGGCCGCTGTTGTCGAAGCGAACGT

GGACCGGGCTCCCGGCGTCCGCCTCGATGGTCGGGCCGGGATACGACCCG

TCGAACCCCCAGACCGTCGTCTCCGGCAGGTCCGGGTGAAGCTGTTGGGT

GAACTCGGTGACCGCTATCTCGTACGCGTCCGCGCCGTCTCGCTGTCCGT

CGGGCTCTCGGACCGACGGAATCGGAAGCGGTTGGACGAACTTCTCCAAC

GTCGGTGACGCCGCCGACACCTCCGTCGTCGTCTGCGGGAGCGTTCCGGC

GATGCCGAGGGCTGCGCCTGTCTGTAAGAACCGCCGCCTAGACCAGTCTG

TCATGATGGACCCTGAACTACGACTTGGGAGTTATTATATTAAATCTTCT

GAATGTCCGAATCAGGCCCGAGAACGGGCGTCGAGAGCGTGGCTGACCGC

GGCGATTCGAGTGCGAGACGGGCGGGCCGTCTCGCTTCGCTAGGTCGCTC

GCCGGCGCGCGAGGAGGACCGAGCCGATTACCAGCGCGGCGAGCGCGACC

GAGACGCCGAACCCGGGGACGGGCGTGTCGGTGGTGGTCGTCGCGGGCGT

GTTATCGTCGGTCGTCGTCTCCGGAGTCCCTCCCGAGTCGCCGTCCGACC

CGTCGGAACCGTCGCCTGCGTCGTCACCGTCGTTCGAGTCGTCGCTGTCG

TCACCAGTGTCGTCATCGTCGCCGTCGTTCGAGTCGTCGCTGTCGCCCGA

GTCGTCATCGCCGTCGTTCGAGTCGTCGCTGTCGTCACCAGTGTCGTCAT

CGTCGCCGTCGTCCGGGGAGTCCGTGGACTCGGGCGTGTCGGAGTTCCCG

TCGTCCGTATCGTCATCATCACTCTCGTCGTTATCGTCGTCATCTCTATC

GTCGTCGTCATCGTCACTGCCGCCGCCACCACCACCACCGCCGCCACCGG

CGTCGTCGTCGGAACCGACCGCGAGCGTCTCGACGCCGTCGACGCTCATC

TCCTTGCCGTCGGCGTCGAACTGGGTGAACCCGTTGAGCCGGTACGAGGC

GTCTTCGTCCACGTCCTCGCCGGCGGTCACGCGATACTGGAGCGTGACGT

TCTCGCGGTCGCCGTAAGTGGCGAACAGTTCGTCGTTGGCGTCGCGCACC

CCGGAGAAGTCCGCGCCGTCGGCGTCGACGATTTCGACCGACGCGAAGCC

GGGGTTGAACTCCTCGACGACGGTGAAGTTCGTCGCCTCGTCGCCGGTGA

TTTCGACCGAGACGACGGCCGATTCGCCGGGGTCGAGCGTCTCGTCGTCG

ACCGACCGGGTGAGGTCCCAGTTGTCGTCGTCGTCGGACCCGCTTTGGAC

GTCGAGCTCGTCCGTTCCACCGGTCGCCGCCTCGGAGCCGTTGACGTCGG

CGAAGCCGTCGAACTGATAGGCCGTTCCGGCGTCCCCGTCGTCGGCCGCT

GTCACCTCGTAGACGAGCGAGACCGTCTCGCGGTCGCCGTAGGTCGCGAA

CAGTTCGTCGTTGGCGTCACGGACGCCGGAGAAGTCCGCGCCGTCGGCGT

CGACGATTGCCACCGAGTCGAACGCGGGGGTGAACGCCTCGACCAGCGTG

AAGTTCGCCGCCCGGTCGCGGTTGACCTCGACGCGGACGGTCGTCGATTC

GCCCGGTTCGAGCGTCGCCTCGTCGACCGACCGGACCACGCTCGCGCCGG

CGTCGGACGAGACGCTAATCTCCTCGTCGCCGGTCGTCGAGGCACGAATA

TCTTCGAGCCCGAAGTCGCCGTAGCCGGTGAGGTCGTGGGTCGTCGCGGT

CGCGTTATCGGCCGCGGTCACCTCGTAGACGAGGGTGACGTTCTCGCGGT

CGCCGTAGGTCGCGAACAGCTCGTCGTTGCCGTCGCGCACCCCGGAGAAG

TCCGCGCCGTCGGCGTCGACGATTTCGACCGACGCGAAGCCCGGACTCAG

CTCTTCGACCACCGTGAAGTTCCCCCGTTCGGAGGCGTTTATCGCCACCG

TGACCGTCGTCGACTCGCCGGGCGCGAGCGACGACTCGTCGACCGTCCGA

GTGAGCGTCCCGCCGTGGGCGTCCGCGGCGACCGGCGCGATAGCCGCCCC

CAGCCCGAGCGACCCGACCACCAGAAGGGCCGCCATCACGACGACGGGGA

GCGCACGTCGCACCTCCGACGACAGCCTCGGGCGTGAACTGTCCGGTCTC

ATGGTGAGCGAGCGAGGTTCGGTCCCGACGGGTCACTCGTTCCGCGAGCG

ACGTGCGAAGCGGCCGTCGGGGGCTTCGATTCGCCCCGGCGGGTCGCGGT

CGTGTGACGGGTCGCGGCCGCCGGCGGTGTCTGTTCGACTCGTCGGTCTG

GAGACGGGCGTCTCGGGCGCGCGCTACCTCTGAACGCCGATAGCTGGCTC

TCGTTGGAACGCATGCAATCAGCGTGTGAATCGAAATCGCCATGCAAATA

GTTAACGATCATCTGACTCGGTCCCGCGCTCGGCGGGCCGCGACGCCACT

CGCGGGACGCCGCTCGTCGGAGTCCGAGCCCCATTCGGGGCGATATTCGG

TGAGTCGCGCCGCGAATCCGCGCCCGTTTTCGGCGTTTCAGCGGACGTAG

TCGGCCAGTTCGTCTCGCGCGCGCCGGGCCGCGTCGTCGGGGTCGTCGGG

GTAGGTGTACAGTTCGAACGTGGCGAAGCCGTCGTACCCGATGTCGTCGA

GCGCGCCGAAGACGGGGTCGAACTCGATGTCACCTTCGCCGGGGACGAGG

TGGTAGTGTTTCCCGCCGCGGCCGCCCGCGATGTCTTCGAGGTGGACGCC

CGTGATGCGGCCGGCGCACTGGCGGATGCTCTCGGCCGGGTCCTCGCCGC

AGACGGCCGCGTGGCCGACGTCGAAGTTGACGCCGAGCGCGTCGCTCCCC

ACGTCGTCGATGAGGGTGAGCACCTCCTCGGTGTCCTCGACGAGTAGTTC

GGGTTCGAACTCGATGCCGAGGTCGACGCCGACGCGTTCGGCGTAGTCGG

TGAGTTCGTCGAGCGAGTCGAGCAGGTGGTCGTAGGCCTCGTCCGGCGGC

GTTCCGGGGAGCGCCGACCCGCTCGCGACGCACGCCGCGGGCGCGCCGAC

CAGCGCCGCGAAGTCGAGCGCGGCCTTCGTGTAATCGATGCGCCACCGGC

GGTCCTCCTCGTCGCCCGAGATGAGCGTCGGGTCGAAGAACGCCGAGGGC

GGCGCGTCGTCGTAGTAGCCGCGGGTCGTGTTCGCGTTGACGTTCGACAC

GGAGAGGCCGGTCTCGTCAAGCGCGGTCAAGACGCGCTCGCGGTCGCGCT

CGTCGAAGTCCGCGAGGAACGCGTGCGGCACGTCCGCCAGTATTTCGACC

CCGTCGTAGCCGTGGTCTGCGATTCGTCTGATTGCCTCCGGCAGGTCGAA

ACGCGTGTACGCGTTCGTGGAGAAGGCTAACTGTACCATACGTCGTGGTA

CAGTCACACATGAATAATTGTTTTGGTACTGTCACTCATTCGGGCCGAAA

AACGTGAAGCGAGCCGCCGAGGGAGCCCGTCGGTTCACGACACGTCGAAC

GCCGACGAGAGGCCGACCGCCGGGACGACGAACGCCGCCGCGACGACGCC

CCACACGGGTGTCAGCAGTCCGGAAAGCGCCCCGTTGAGGACGACGAGAC

CGAGGACGCACGCGCCGACGGCGGGGCCGATAGTGCTCGGTCGAGGGTCG

GCGTAGGCGGTCAGGAGGTCGCGGCCGGTCCACGCCGCGAACCAGCAGAG

CAAGCCACCGGCCACGACGACCGTCGCGGTATCGCTCGGGCCGACGCCGA

CCGCCGCGCCGGCCGCGACCACCGACCCGACGGCGAGTCCGACACCGGCC

ATTCCGACCAGCACCGCCCGCGAGTCGCCCGCACCGGTCTCGCGCTCGGC

CATGTACGTGACGCCGGCGATGTAGAGCGCGACGACGACCGGCACGAAGA

ACACCCACGCCGGGAGCGTCAGGGTCGTCGGGGCGGAGGGCGACCCAGCG

ACGACCGCCATGCCGAGAGCGACGTTCGCCCCCCGACAGCCACCCATGGC

GGCGAAGCCCAGCGGCGTGCCCTTGAGCGCGCCGTCGTAGAGCGCGATGA

ACGAACCGAGGACGGCGGCGACGATTCCGGCGGTCGGTCCCCCGGCGGCC

GCGGCGACGACGACCCCGGCACCGAGCAGCGCGAGGCCGAGATTTCTCGC

TCGCGTGCGCGACACGTCTCCCGACGGAATCGGTCGCTCCGGGCGGAGCC

GGGCGTCCTCGTCGGCGTCGGCGTAGTCGTTGAGCGTCGTGCCGGCCGCG

TAGAGCAGCACCGAGGCGAGCGCGGTGCCGGCGAGCGACGAGAGCGGAAC

CGCTGGGCGGCCGCCGCTGACCAGCACCGCCCCGAGGATAACGTCGGGCG

GCGCGGTGAACAGGTTCGGGACGCGGACGAGTCTGGCGAGGCTCGAAAGC

GAGCGTCGAAGACCGCCGGTCGCGGGTGTCGCGTTCGTTCGGTTCATCGT

CGGCCAGTCGCTGGGACGGGTGCTCGCGTTCGAGCGAGGCGGTCAGGTCC

ACCCATGGGCTTCGAGGTACTCCATCGCGGTTCGGGCCGTCTCCGCCGCA

GTCTCCTGATAGGGGTACAGTTCGACCGTGACGTGGCCGTCGTAGCCGGT

CGCCTCGACCGCGTCGAGGAAGCCGTCGATGTCCATCGCCCCCTCACCGA

GTTGGGTGTGTTCGTGGGTGCGGTCCGCGGGGATGTCTTCGAGGTGATAA

TGTGGCGTGTGGGGTTCGAGCGTCTCCACCAGTTCGACCGGGTCCTCGCC

GACCGAGTAGAAGTGTCCCGCGTCGAAGTTACAGCCGATTCGGGGCGAGT

CGACCCGGTCGACGAGGTCGAGGAAGTCCTCGGGGGTCTCGATGAGCAGG

TGCGGTTCGGGTTCGACCAGCACGTCGACGCCGAGTTTCTCCGCGGTGTC

AGCGACCTCGCGGAGCCCTCGGACGAAGGCGTCGAGCGCGTCCGCCCGCG

TCATGCGCTCCGGGACGGGGCCGCCCGGCGGGACCGAGATGCTGTCCGCG

CCGAGCGCGGCCGCGGTGGCGAGCGCGTTCTGCGTGTGCTCGACCCGCGC

CTGTCGGTCCTCGGCCGCGAGTTCGACGAACGAGGGGTGGTGAAACGCCT

CGGTGTCGCGGTTGAACGTCGCCGCCCGACTCGCCGCGGAGGGCTCGATG

GCGCTGAGCATGAACGCGTTACAGTTGCTGACTTCGATGCCGCAGTCGTC

GAGAAGCGTCCGAACGCGTTCGAAATCGTCGCCGTCGGCCGTCCCCGGGA

ACAGATGCGGGTCGTCGAGCAGGATTTCGACGCCGTCGTAGCCGGCGTCG

GCGATTGCCTCGACCCCCTCGGTGAGAGTTCGCTGGCGGAACGCGTTCAT

CGAGAAGCCGAACTCCATCGGTCACAGCTCGTAGTGGAAGTTCGGTGACT

GGCCGAAGAACTCCCGGGGGTTGTCGCAGACGACCTTCCGGACCTCGTCT

CGGTCCCAGCCCCGGTCGAGCATCTTGTCTCGGGCCTTCGGGACCGCGAG

CGGGTCCGAGGGGTCCCAGTCGGCGGCGCTGTTGAGCAGCATCCTGTCGG

TGCCGTACTCTTCGAGGATGTCGATGGTCGTCTCGGCGTCTATCTTTCCG

GGGTAGAGGGTGAAGCCGAGCCAGCAGTCGGTTTGCAGCGAGATGTCGAC

CGTGTTCTCGGTGTTGTGGTCGATGACGATTCGCTCCTCGGTCACGTCCT

CGTCTTGAATCATCTCGACGAGTCGCTCGGTCCCCTCGGGTTTCTGGGTG

TGCGGGGTGTGGATGATGACCGGCAGTTCGCGCTCCTCCGCCATCCTGAG

TTGGCGTCTGAACGCGTACTCCTCGGCCTCGGTCCCCTGGTCGAGCCCGA

TTTCACCGAGTCCGACGACGTGCTCGCGGTCGAGGTACTCGGGGAGGCCG

TCCATCACGGCCTCGGCCATCTCGGGGTAGTTCGCCTCCTTCGGTTCGAG

GCCGACCGTGACGTAGTGGTCGATGCCCGCCGCCCGCTCCGCGCGGTCGG

TCTCGAACTCGATTATCTGCTCGAAGTAGTCGAAGAACGACGCCGCGTGC

TGCTTGTCCGTCCCGCTCCAGAACGCCGGTTCGACGCAGCATTCCACGCC

CGCGAGTCGGGCGCGCTCGTAGTCGCTCGTCGAGCGAGACACCATGTGCA

TGTGCGGGTCGATAATTGGTATCGACATGTGACCTACCTCTCTGATGGTA

GTTAATCATTGTTTCGGTGGATTCGGTGACGGCCGGGGGGCGGGGGCGCC

ACGGCCGCGCAACGCCCGGCCGACGCGGCCGCGAGCGACGGCGGAGACAG

CTATTTACTCGTCCGCGCCGACGCCGTAGTCGATGCCGGAACACTGTCCG

TTCTGCGGCGAGCCGATAGAATCGACCCGCGTCACGCCCGAGGACGGTCG

CCCCTACGAGGAGTGGCGCTGCGCCGACTGCGACGAGACGTGGCGACACC

CCGAGGAGACGGTTCTCGACCGGTCTCTCGATTTCAGCAAGCACGGCGAC

CAACTCGACAGGCGGGACGCCGACACCGACCTCGACTGGTGAACGGTCGC

CCGCCGGTACCGACGCGGAAACAATCATGATTGACGGACAAGGTTATGTA

TCGGCCACCGCAATCGTGAGTCGATGACAGTCGGAGTGTGGATTATCGGA

GCCAGAGGTAACGTCGCGAGCGTCTCGATGACCGGCGCGCGCGCAATCGC

TCGCGGAGTCGCGGACACGACCGGGATGGTGACGGCCCGCGAGCCGGTGG

CCGCGCTGGACCTCCCCGCCGTCGACGACCTCGTGTTCGGGGGCCACGAC

ATCCGGTCCCAGCGAATCGAGGAGACCGCCGAGGAGATGGCGGGCCACGG

CGGCGTCGTGGCACCAGACACCCTCGACGCGGTCCGCGAGGACCTCCGCG

AAATCGACGAGCGCGTCGAACTCGGGACCGCCCGGCGGTGCGGCGAGGCG

GTCGAAGGGATGTCCTCGGAGACGACGGGAGAGGACGTGTCGGTTGCCGA

CATCGTCGAGGAGATACGGGCCGACTACGCCGCCTTCGCGGACTCGCAGG

GCGTCGACCGGTTGGTCGTCGTGAACGCCGCGTCGACCGAGCCGCCGATT

CCGACGCCCGGCGACTACGACACGCTGGCGGCGTTCGAGACGGCGGTCGA

GCGCGACGACCCGAACCTCCCCGCGAGCGCCCTGTACGCCTACGCGGCGC

TCCTCGACGGCCACCCGTACGTGAACTTCACCCCCAGTACCGGCTCCTCA

CTCGGCGGCCTCCGCGAACTCGCGGAGCGAAACGAGGTGCCGCACATGGG

CCGCGACGGGAAGACCGGCGAGACGCTGATGAAGTCCGCGCTCGGGCCGA

TGTTCGCGGGGCGGAACCTGCGCGTCCTCTCGTGGGAGGGCCACAACATC

CTCGGCAACAGCGACGGACTCGTCCTCGAAGACGACGCGAACAAGGCGGG

GAAGATAGAGAGCAAGGGGAGCCTGCTGGACGACATCCTCGGCTACGAGA

CCCACAACGCCGTCCGCATCGACTACACGCCGTCGCTCGGTGACTGGAAG

ACCGCGTGGGACCACGTCCACTTCGAGGGCTTCCTCGGGACGGAGATGAA

GATGCAGTTCACGTGGGAGGGGGCTGACTCGGCGCTCGCGGCCCCGCTCG

TTCTCGACTTGGTTCGGCTGGCCGCCCTTGCCGACGAGCGCGGAGAGGGC

GGGACGATGTCCCACCTCGCGTCCTTCTTCAAGTCGCCCGAGGGCGTCGA

CCGCCACGAACTCTCCGAGCAGTTCCGCCTCCTCTACGACTACGCGGAGC

GCCACGCGGAGGGAGCGTGACGACTCGCGACCCCTCGGCCGCTGGCGCGT

CCGGTGCCGGGCGCGTCGTCGTCCTCGATATCGTCGGCCTCCAACAAACG

CACCTCGACGAGGGGCTGGCACCGAACGTCGCCGACTTGCTGGCCGGCAA

GCCGTCCGGCCCGCTCGAACCCTCGTTTCCGAGCGTCACGGTGCCCGCGC

AGACGACGCTCTCGACCGGGCAGTCGCCCGCGGCCCACGGCGACGTGTCC

AGCGGCGAGTTCGACCGCGAGCGACAGGTCGCCGAGTTCTGGGAGCGCGA

CCGAGGAGGCCGAACCCGGCTGTGGGAGGTCGCGAGCGACGAGGCGGGCC

TCACCACGGGCGCGCTGTTCTTCCAGCACCTCATCGGCACGAGCGCCGAC

GTGGCCGTCACGCCGTCGCCCATCGAAGACGAGGACAACAATCTCATCGA

GATGGACTGCTGGACGACCCCCGACGGCTTCTACGACGACCTCCGCGAGG

AGTACGGCCACTTCCCGCTGCACAACTACTGGGGGCCGGTCGCCGACGAG

CGGAGCAGCGAGTGGATTCTCGCGGCCGCGCGGGAGTCGATAGCGCGGTC

CGACCCGGACCTCCTGTGGGTGTACGTGCCGTACCTCGACTACGACGCCC

AGCGGCACGGCCCCGACTCGCCGGAACTCCGGGAGGCCGTCGGCGTCGTC

GACGACCTCGTCGGGGAGTTCCTCGACCGGCTCGAAACGACGCCCCGCTG

GGCCGAGACGGCGGTCGGCGTCGTCAACGAGTACGGCTTCAACGCGGTCG

ACACCCCGGTGTTTCCGAACCGGGCGCTCCGCGAGGCGGGCCTGCTCGCC

GTGCGCAACGACGACGCGGGCGGCGAGAAAGTCGATATCGCCGGGTCGTC

GGCGTTCGCCATGGTCGATCACCAAATCGCGCATGTCTACACCGACCGCC

CGACGGCCGCCCGAAAGGTGCTCGAATCGCTCCCCGGCGTCGACGCGGTG

CTCGAAGGGGAGGCCCGGGGCGACGCGGACCACCCGAACGCCGGCGACCT

CGTCGTCGTCGCGGACGCGGACGCGTGGTTCCAGTACTACTGGTGGACCG

ACGACGCCGATGCGCCGTCGTACGCGACGGAGATAGACATCCACGCCAAG

CCCGGCTTCGACCCGTGCGAACTGTTCCTCGGCGACGAGGGACTCGTGAC

GCTCGACCCGACGAAGGTGTCGGGGTCGCACGGCCGCATCGACCCAGACA

CGCGCGGCTTCTACGGCTTCGGCGGGCCGGCGGCACCCGACGGCCCGTCC

GACCGAATCGTCGATGCGCGGCGGTTCGCGCCGACGGTCGCGGACCTCCT

CGGCATCGCAGAGGACGTGTCGCTCGCGTTCGAGCGCCCGTCGCTGTTCG

CCGGGCCGCAAGTGTAGGTTCGACACCGCCGCGACGCGCTCCAGAACTGC

TTTTCCGCTCCGCTCGACGACAGCGAACGTACGATAGATACAAAAGAAAA

GCCGCCGCGGACCGCGGTCGGAACGCGTCTCGCCCGCTCAGTTCCCGCCC

GAACTCGCGATTTCGATGGTGCCGCGCATCCGTAGCTGGTGGGCGCGACA

GACGTACTCCGCCATCGCGTCGGTGACGGTGAACGTGACGGTCTGTGACT

CGCCCTGCGTCGAGAGGTACGACGTGGAGATGTCGTTGACGGTGGAGTCG

TCGCCGTTGACGATTTCGAAGTTGTGGGGCGCGCCGTTGCGGTTCGTCCA

CGTGAGCGTGTACTCGCCGCCGGCTTCGAGGGTGAGCGTCGGATTCGTCG

CGTCCGCGATTCCGGACGGCGCGACGCCGGTCCAGCCCTCGTTGCTCTCG

GCTTCGAGTTCGATGGTCGAGCCCGACGGGATGACATCGCTCCCGTCGTC

GTCGCTGCCGCCGTCGTCGCCGCCGCCGCTCGTCGCCTCGACGTGCGCAG

TGACGCGGTCGTAGACGAGCTCCAGCCCGTTGCCGGAGCCGCCGATGCGC

GTCAGTTCGGTATCGTCGAACCAGACGGTGCCGGTGGCGTCTCCCCAGCC

GCCGAACAGCAGGTTAAGCTGGAACTCGCCGGGCGACGCCCCGGTCGAGA

ACGTCGTTTCGAGCAGTTGCCAGCCCTCGACGGGCTCCGTGTAGTAGTCG

GTGACAGAGCCCTGTCCGAGTGAGTGGACGTTGATGGTGACACCGTAGGA

GCTTCCGATTTCCCCGGTTCCGTCCTCGGAGTTGCTGAAGTCGTCCGAGG

TCCTGACCCACGCCCGGAAGCGGTACTCCGAGTTCGGTTCGAGGTCGTCG

CGGTACTGGGTCCACGACGCATCCGCACCCTCCGTGGAGTCGACGCGGAC

GCTGTACTCGCCCGTCCGCGAAACGTCCGAGGTGTAGGTGAACTCGGCCG

TGCCGCCGTAGGTAGTCCCCTCCCAGTTCGCCGGGGTCTCGGGGTCCGAC

GGGGAGTCGGTTCCCGCTCCCTCCTCGAACGACGCGTTCGGCAGGAGGTT

GTTCCCGTCGGGGTCGGTCAGCGAGACGTTGTCGAACCACGCCGTCCCGG

TGGCCGTGCCGTAGCCGCCGAACAGGCAGTTAATCTGGACCGTCGTGGTG

TCCGCGCCCGTGGTGAACGTGGTACTCACCTCCGTCCAGTCGTTCGTCCC

GGTCAGCGGCTCCGTCTCGAAGTCCGTGCTGTGGACGTTGAACAGCGCGC

CGCGGGCAGAGTCGCTGGCCTGCACGTTCTCGGTCCGTATCCAGCCGCTG

AGGGTGTACTCCGTCTCGGGCTGGACCGAGATGCCGTCGACGAGCCAGAC

CGCGTCCGCGCCCTCGGTGGACGAGATTTGGACGCTCTGACTGCCGTCTT

GGCCCGCGTCCGCGTAGGTGTACTCGGCGGTGCCCGTGAACGTGTTCGTC

GACCAGTCGGCGGGCATCGCCGGCTCGCTGCTGCCGGTGCTCTCTTCGAA

GGAACCGTTCGAGAGCACGTCGGTGCCGTCCGGGTCGGTCAACGAGACGC

CGTCGAACCACGCCGTCCCCGTCGCCTCGCCGTAGCCGCCGAACAGGCAG

TTAATCTGGAGCTCTTCGAGGTCGCCGCTGTCGATGGTGGTGCTCAGTTC

CGTCCAGTCGTTGGTGCCGGCCAGTCCGGACGGGATGGTGTCCCACTGCG

TGCCCGAGTCCGTGTTCGCTATCTGCTCGACGTTGATGGTCGCGCCGAGC

GGTCCCTCACCGATTGCGGTCCCGTCGACGAGTTCGAGGCCGTCGGTCCT

GACGTACGCGGAGAGGGTGTACTCGGTGTTCGGGTCGACCGCGACGGTCG

TGTTCCACGAGGCGTCCGCGCCCTCGGAGGACGACACCTGGACGCTCCGG

TCGCCCTCGTAGCCCGTCTCGGCGTACGAGAACTCGGGCGACCCGGAGTA

GTCCGTGTTCGACCAGCCGACGGGCATCGCGTCGGCGTCGCCGGTGTCGC

CGCCGGCGGGCGTCTCGAACGAGGGGTTTTCGAGGAGGTTCGGCAGGTCG

TCGCCGCCGTCGCCGCCGTCGCTCGTGTCCTCGTTTGCCTCGTACGCCGG

GATGAAGCCGTCGGCGTGGGTCGCGCCGGCGAGCGACGCCGCGTCGACGA

GAACCGTGTCCCGGTAGTTCGACTCGTCGCTAATCATCTGGTAGACCGCC

TCGCCGGACGCGTCGTCGGCCGGCGTCTGCGCGAGCGCGACGAGCGCCCA

CATGGCGACTCGCCCGTCTTCGTCGTCGAGGAGGCCGTTGTCGAGAATCG

CCCCCCGCGTCTCCGCCGTCGACGGGAGGACGCGGAGTGCGGTGAGCCTG

ACGCCCGCCGAGGAGTGGGTCAGCGCGCCGAGCGCGGCCTGAATCGCGGT

CGCGTTTCCGGTGTCGGCGTCGAGCGCGCCGAGGCCGTGCATCGTCCGCA

GCGCGTGAATCGCCGCCGGGTCGAGGCCGGTTTCGTCGAGCGTCTCCGTC

GCGACGAGGTCGACGAGCGCCGAGAGCGCGCCGGTCTCGTTCCGTTCGAC

CAACAGCCGTTGGGCCGTCTGCCGCCAGAACATGTTCGTGTTCGACAGCG

CCGCCACGAGTTCTGAGACGCTCGCGTCCGAGAGGTCGGTCGGCTCGTAG

CCGTCGTCGTCGCCGTAGACGACGCGGAAGAGTCGGGCGGTCGCGTGGTC

GCGCACCTCGGACATGTACGCGTTTCCGGGGCCGTTCTCGAAGCCGTCGG

GTGTCGGGTTGTGCTGGTAGATGTAGTTGTACCAGTCGATGAACCAGAGC

ATGCCGTCCGGGCCGACGGAGGAGTACGACGGTGCGAACCACGCATCTGT

CGCGGCCGCGATGTTGTGGGCGTAATGGTTGGTGTAGCCCGCGCCGTCCT

GCGAGAGGAAGAACGTCCCGAGCAGGTTACCCGTTCCGCCGCCGACGAAG

CCGGTGTTGTTCCAGTACTTCTCGGGATATTCACGCGCCGTGTAGATGGT

GTGACCGGTCGCCGCGGTGTAGCCACCGTGGAGGTCGACCTGCCGGACGC

GGTCCGTGACCGGAAGGAATCGGTTCGTGTCGGACGCCGCGCCGAAGTCG

TTCGGCCCGGTCCCCTCGATGAGGTCGTAGTACTGATGCGGGATGGCGAA

GTAGTTGCTCGGTCGCCCGGAGGTCGCCGCCGAGCCGAAGGCGAGGCCCT

CCTCGGTGAAGCCGAGGCCGGCCTGGTTCCCCGGGAGCGTGCCGACGATT

TCGAAGTCCGTGACTGAGCCGTCTTCCAGCTTGAATCGGAAGACTGAGGA

GCTGAAGTTTCGCTCCTCGCCGGCGACGGTGCCGCTGAAGCCGGAGTAGC

CGACCTGTCCCCAAATCCAGTTGTCGATGCCGTGGGCCAGTTCGTTGGGG

CCGGCGTGGGTGTCGCCGTTGCCGAACCCGGAGAACAGGACCGTCCGCTC

GTCGGCCTCTCCGTCGCCGTCCGTGTCAGCGAGGTGGACCATCTTCCCGC

TCTCGCCGGGGTCGTCGAGGTCCGCAACGACGACGCCGTCGTCGACGACC

ACCATGCTCGTCGGAATCGAGAGGCCGTCCGCGAAGACGGTGAACTCGTC

GGCCTCGCCGTCGCCGTCGGTGTCCTCGCAGACGACGATTTTGTCGCGGT

TCTGCCCGAGTTCGTTCGGGTAGTCGCGGGTGACAGACAGCCAGACGCGA

CCCCGCGCGTCGAACTTCGCGTCGAGGATGTTCCCCCGCGCGTCGTCCGG

CAGGTCGGCCTCGGTGACGAACGGTTCGAGGTCGAACCCCTCCGGCGTAA

TCGTCCGCCGGACCGTCTCGCTGGGGTTCAGCGCCCGCTGCATCCTGTTC

CAACTCGTCCCGCTGCCGACCTCCTCGGGGACCTCCGGCGTCAGGAGGCT

CCCCTCCGGCGGCGGATAGTACGGGATGTCGGCGTCGATGAACTCCAAGT

CGTTGAGGACCGTGGTGTCGTCGGCGATGGTATCTTCGTTCTTCGTGACC

CAGCGAATCGCGTTCTCGATGAGCGCCTTGAAGCCGTCGGTCGCCCACGG

CGCTCTGCCGTGACCCCACGCGGTGTAGAAGACGCGGCCGTCGCCCTGCG

TGCGGGTCCACGACCACGGTTCGCCTTGGTCGCGTCCCTCGTCGTACTCG

GGGATGCTGTCGCCCTCGGGGAACTGCGCGTAGGCGAGTACGTCGATATC

GTCGTTGAGGTTCGTGTGGCGATACGTCTCGTCTTCGACCGTGATGGGGT

CGAGGGTCGACAGGATGGGGTGGCTCGGCTGGGCGAAGTTCGTCGTCATC

TCGCCGTAGTTGTGGGCGGCGAACTCGCCGCCGACGAGGTTCATGTAAGC

GTCCGAGCCGGTGAAACAGGCCGAAGCGCTGTGAATCGGGATGAAGCCGC

CGCCGTTCTCGACGAACTCGACGATGGACTGCTCCTGTTCCGCGGTCAGC

GATTGACCGTAGTTCGCGCCGCGGTTGTCTATCATAACCCACGCGTCGTA

GCGGTGGAGCACGTCGGGTTCGAGGTCTTCGAGTCGGTCGGTGTACTGGA

CCTCGATACCGCGGTTCAGCATGTACCCGGTGAGCTGGACCTGCCGTGCG

GGCGCGTTGTGGCTGCCGCCCTGCGGGCCACCCATCATGAGGACGCTGAT

GGTGTCCTCGGCCGGCGGCGGCGTTATCTCGACGCTTCCCCGCATCTGCT

CGGGGTGTTCTTCGGCGTAGTACATCGCCATCGACGACTCGGCGGTGAAC

GTGACCGTCTCCGAGCCGCCCGACGCCTGCATCGTGGAGAACGACTCCAT

CACCTCGCCGGTCGGGTCCGCGAGCACGAACGTGATGTCCTCGTCCATGG

CGTTCGAGAGCTCGATTTCGTACTCCTCGCCCTCGTGCACGACGAGCGTC

GGATTGGCCGTGTCCGCCATCTCGTCTGGCTCGGTCGCGACCCACGAAAA

CGAGTCCGCCGCGGTCTCGATTTCCGTCCCGTTTCGCCAGCTGTTGATGC

CCGTCAACAGGGAGTTGGTCGTCAGGTCGCCCTCGCGCCAGTCGGATATC

GCGTCCGCGAGACCCGACCCCTCGATGACGGACTCCTCGTTCGTGTACGC

CGCGAGGACGTCGCCCTCCGTCGCCACGAGGCGAATCGTGTCGGTGGCGG

TGAGCTGTTGCGCCTGCGCGACGCCGCTGGCCCCGAGGAAGTTCGCCGCC

CCCGCGCCGCTTACCTTCATGAAGTCGCGTCTGTTGATGTTCAGTCGGTC

GCCGTCGTCGTCCGGGTTCGATTCCCGTGTCGTGTCGTCGTGTGTCATAG

TGTTGCGTCGGTCGCTGTCGTCGGTTACCGTTCGATTCAGGGTTGCGCTG

CTCTCGGGGAGCGCACCCCGGCCGCGTCCCCCGCCAACGGGCCGAGCGGA

CAGACGGCTCTCCCCCCGCGCGCTCGCCCGAACCGCTCGACGAGGTGTGA

CGGGTGTATCGCCGTTACGTCCGCAGGCTCATTGTGGTGGGCTACCGAAT

TTCATGAACTAACATTACTTGAATGTACTGTCCGGCGGCAAGGAGCGACC

GCCCGGTTCACCGCCTCAGTCGCCGGTGAACGTTGGCCTAACCCCAATGT

TAATTATTGTCTGTCGTGTTGCTTCAGTTGCACGTATGAACCGCGCACAC

ACACAGTCTGTCGTACCGGCGGCGACGCCAAGGGGCGTCGCGGGTTCGAA

CCACGGGGGCGGACCGACCGGGCGCGAGCGGCCCGAAGCGGGTGGCCGAT

GAGCGTCGTCATCGTCGGCACGCTCGACACCAAAGGCGAGGAAATCGGGT

TCGCCCGCGACGTGCTCGAAGAACAGGGGGTCGAAGTCCACCTCGTCGAC

GTGGGCGTGCTGGGCGAGCCGGAAATCGAACCGGACACCGACGCCGCGGC

CGTCGCCGAGGCGGGCGGGAGCACCCTCGAAACGCTTCGGGAGGCCGGCG

ACCGCGGCAAGGCCATCGAAATCATGGGCGACGGTGCCGCCGTCGTCGTG

TCCCGACTCCACAGCGAGGGCCGACTCGACGGCATCCTCGGGCTGGGCGG

CGGGGGGAACACGTCGGTCGCGACGGCCGCGATGCGCGCGCTGCCGATGG

GCGTTCCCAAACTGATGCTGTCGACGATGGCGTCGGGCGACACGGAGCCG

TACATCGGCTACCACGACATCGCCATGATGTACTCCGTCGCGGACATCGA

GGGCCTCAATCAGCTCTCGCGGACCGTCATCTCCAACGCGGCGCTCGCGA

TGGTCGGCATGGTGTCGAACGAACCGGACGTGGAGACCGAGGAGAAGCCC

ACCATCGGCATCACGATGTTCGGCGTCACCACGCCGTGCGTCCAGAGGGC

GCGCGACTGGCTCGAAGCGCGCGGCTACGAGACCATCGTCTTCCACGCGA

CCGGGACCGGCGGCCGGGCGATGGAGTCACTCATCGAGGAGGGCGTCATC

GACGGCGTGCTCGACGTCACGACGACCGAGTGGGCCGACGAACTGGTCGG

CGGCGTCCTCGCGGCGGGGCCCGGCCGACTCGACGCCGCGGCCGAACGCG

GCATCCCGCAGGTCGTCTCGACCGGCGCGCTCGACATGGTCAACTTCGGG

CCGAAAGACTCCATCTCAGACGAGTTCGACGGTCGGCAGTTCCACGTCCA

CAATCCGCAAGTGACGCTGATGCGGACGACGCCCGAGGAGAACGCCGAAC

TCGGCCGCATCATCGCGGAGAAACTCAACGCCGCGACCGGGCCGACCACG

CTCGCGCTCCCGCTCGGCGGCGTCTCGATGCTCGACGCGGAGGGCGAGGC

GTTCTACGACCCCGAGGCCGACGACGCGCTGTTCGACGCGCTCCGCGAAC

ACCTCGACGACGACGTCGAACTCATCCAATCGCCGGCGAACATCAACGAC

GACGAGTTCGCGCTCACCCTCGCGGAAGCTATCGACCGCCGGATGCGGGC

GGCGGCGTGAGACGACCGACATCGACGTGAGGCCGCCTCCGACGACCCAA

TAGCTCCACCGAGACGGCCGCGTGTCGCCCCGCGAGCAGCGACGCTCGAA

ACACCGACCGGCGGAATTTCCCGCTCGCAGTCGGTCGTTCGACCTGTTTT

CTACCTGTATAATCACAAAGTTTAACTATATCATGACACCATGTCACTAC

CAGCGTTCCGAAGAAACATGACTGATCAACCAGCGCTAACTGACGAATCC

AGGCGACGCTATCTGAAAGCAATCGGGACGGCCGGACTCACCGCCGGTCT

CGCGGGCTGTTCCGGCGGGGGCGGCGGTAACGGCGGCGGCGGTGACGGCG

GCGGAGATTCTGACGGCGAGAACAACGCCGGGGGCGGCGGGGGTGACGAC

TCCTCCGGCGGCGGCGAAGACTACGAGGCAATCGGGAACTTCCCGCCCGA

GGGGAACTCCGTGAGCATCGGTTTCAACGGGCCGACCTCCGGCGCGTTGG

GTCCCGACGGCCAAGACCAGGAGAAGGGCTTCGACCTCGCGGTGAAGCAC

CTGAACGAGGGCGGCGGCCTCGTCGACTACTGGGACCGACTGAGCGGCGA

CGGCATCATGGGCTACCAGGTCGAGCCGACGAAGGCCGACACCGCCGGGA

GCGCCGACACGGCACAGGACAACATCGAGCGGATGATTCAGCGGGACAAC

ATCCAGTTCTGGACGGGCGGCATGTCCAGTACGGTGACGATGGCGATGCA

GAACGTCGCCCAGCGAGAGAAAGTTCCGTTCATGGGTGGGAACTCAACGT

CCGCCGGCATCTCCGGGGAGAACTGCTCGCGATACTACTTCCACCCGACG

TTCCACGCGGAGATTATCGGGATGGCGATGGGCGAGGCCGCGCCCTCGGT

CCTCGGTGAGGACCGCTCGCTGTTCCACATCTACATGGACTACTCCTACG

GGCAGTCGAACCGCGACGCCGCCCGCAAGTACCTCACCGAGCAGGGGCCG

TGGGAGGACGCCGGCGGTGCCGCCATCGCGGAGGGCGAGACCGACCACAG

CTTCCAGATTCAGGCGCTCGAAGACTCCGGTGCCGACACGCTGTACTTCT

CCAGCTTCGGCAACTTCGCCGCCAGCGGCCTCGCGCAACTGCGAGACGCC

GGCCTGACCGACGACATCGACGTCATCATCCCGCACGTCAGCGCGTTCAC

CCTCGACCCGCTCGGCGCGGACGCCGAGGGCGTCCTCGGCATGGAGCCGT

GGAACCCGAACGCCGACAACGAGGCGAGCCAAGCGTTCGTCGAGTCCTAC

CAGGCGGAGTACGACGAGACGCCGAATCAGAGTTCGCTGCACACCTACGA

GTCGATGATGGTGTACGCCGCCGCCGTCGAGGAGGCCGGCACGTTCCACC

CGCCGACCGTGGTCCGAACGCTCGAAGACTTCGAGTGGAGCCTCGCGTGG

GGCGACTCCGCGTTCCGGACGTGCGACCACCAGGTCGAACGCCCGTGGTA

CATGGTGCAGGGCGTCGGCGACGACCGCGCCGAGGAACTCGGCATCCGGA

CCGAAATCGTCGAGACGACCGACCCGCTCGTCTACGAGTGCAGCGAGTTC

CCGGCGTCCAACTGCGCCATGGGCGACAACGAGTACGGAGACGAGTGACA

GCCTGACAGCGCCTCGATACGAATACAACGTACCAACTCCATTTTGGGCA

GTCGAGGCTCCCTCCGCGGTCAGTTCACCCGCGTTCGACTGCTCGAACGC

CGCTTTTTCTCGGTCGCACGGACCTCGCGTTGACGCCCGGAGACGACCCG

CGCCGCACCCGGAGAGCGGGCCGCTGCCGTGTCAAAAGTCAGCGAAACGC

AAGATTAACATACTGAAGAATTCATGGATAATCGTGCACACCATGAACTG

CTTACTCACAGCTGTACCGAGGTGGCTGCGATGAACGTCGACGGCGTCGT

CGGATTCCTCTTCATCGCGCTGAGCGTCGCGTCGCTGTATCTGCTCGTCG

CGGTCGGACTGTCTATCGTGTTCGGGTCGCTCAAGTACGTGAACATGGCC

CACGGCGTCCTCTACCTGAGCGGCGCGTACATCGGCCTACTCATCGCGTC

GAGCGAACAGTACGGCGGGCTGTTGAGCGACTTCGGGCAGGTCGGCCTCG

ACTGGGGGTTCGTCCCCGCGCTCGTGTTGACGCCGGTGGTCATCTTCGTC

GTCGGCGTCGCGATGGAGCGCTGGATTGCGAAACCGTTCTACGAACGAGA

ACTCCTCGACCAACTGCTCGTCACGTTCGGCATCCTCATCGCCGCCCAAG

AGCTGGTCGCCATCCTCTTCGGCCGAACGGGGACCATCTACCCGCGCCCG

GAGTGGCTCACGGGCGCGATATCCCTTCCCGTCATCGGAACGCCGCCGGG

GATGAGCGCGGCTTCGACGATACGCGTCCTCGTCGTCGCGCTCACGCTGT

TGCTCGTCGCCGGTATCTTCGCGTTCTTCAAGTACACCGATTACGGCCTC

GCCGTCCGGGCGGGGACCGAAGACTCCGAGATGACGCAGATGCTCGGCAT

CCGTATCGGGCGGCCGTTCCTCCTGATATTCGCCGTCGGAGCCGCGTACG

CCGGGCTCGCGGGCGTTCTCGGCGGGTCGCTTTTCAACGTGACCTCCGAA

ATCGGCATGGAGATAATCATCCCGTCGCTGGTCATCGTCATCATGGGCGG

CGTCGGCAGTTTGCGCGGGACCGTCGTCGGCGCGCTCCTCGCCGGGCTCA

CCTTCGCGGTGGCGACGGAACTCGAACCGAGCATGACGCAGGCCAGCATC

TACCTGCTCGCCATCGTGGTGCTGACGATTAGGCCGAGCGGTATCTTCCC

CTCGGCGGAGATCGGACAATGAGCACGGACAACATCCACAAGACCGTAGC

CGAAGCATCGTCGTTGATTAGCTGGGACACCTGGGACCGAATCAAACACA

CCGAGTCGTTCGTCCTCCTGTCGTCGGTGCTTTTCGTCCTCCTGTTCTCG

TACGCATTCGGCCGCGCGCCGGTCGTCTCGGACGTCTTCCAGGGCTATCA

CGGGCTGGCGATTACCATCCTCATCTGGTCGATATTCGCGCTCGGATTCA

ACCTCCTTTTGGGACAGACCGGCCTCCTCTCGTTCGGCCACGCCATGTTC

TTCGGGACCGCGAGCTACGCCGCGGCGCTGTTTGCGATTCACGTGTACAA

CGACCCGCTGGCGGTCATCGTCGTCGGGACGCTGGCCGCGGTCGCCCTCG

GGGCGGTCGCGGCGCTGATTCTCCTGCGCCTCCACACGGTGTACTTCTCC

ATCGTGGCGCTCGCCATCGGGCAGTTCCTCTACTTCCTCGCCCGCGAGCC

GCTCGTGGAAATCACGAAGGGGATAAACGGCCTCGAAGTGCCGCGGTCGG

ACGTTCTCGGGGTCTTCGAGCTGGAACACCAGTACGGCGGCCTCCTCGGG

GAACTCGTCGTGAACAACCTCTATCTCTTCGTCGGCGTCTTCTTCGTCGC

CGTCGTCGCCTCCATCACGCGCATCCGCAAGTCGCCCTACGGGCTCATCT

TCAAGGCGATTCGCGAGAACGAGACGCGGACGGCGTTCGTCGGTCTCGAC

GTCTGGCGCTACAAGTTCGCGGCGTTTCTCCTCTCTGCGGCCATCGTCGG

CCTCGCCGGCGGACTGATGGCCGTCAACACCCAGTTCGCGGGCGTCGAGC

GCCTCTACTGGTCCGTCAGCGGCGACGTGGTCGTCATGACCGTCCTCGGC

GGCCTCGGGACGCTCGCCGGCCCGGTCATCGGGACGTTCGTGTTCTTCTA

CTTCAAGGGCATCGTCAACGGCTTCCCGACGCTCGGCAACTACTGGCTGC

TCCTGCTGTCGCTTTCCTTTACCACGGTCGTGTGGGTCTACCGCGACGGC

ATCTGGGGGATGATTACCGCGCTCACCGGCGCGCTCCGCGACCCCCGAGA

GCTGGTCGCCGCGATCACGGCCCGAGTCACGGGCGGCGACTCGACCGGCG

ACGCCCCGGGAGGTGAGGACTGATGGCGCTGCTCGAAACCCGCGGACTCA

CCAAGGAGTTCGGCGGGCTCACGGCGCTCGACGACGTCGACATCGACGTC

GAAGAAGGCGAACTCGTCTCGCTCATCGGCCCCAACGGAGCGGGTAAATC

CACGCTCATCAACACGATTACCGGACGCCTGCCCCCGACCGAGGGCCAAG

TGTCCTACGCCGGCACCGAGCTCGTCGGGATGAAACCGTTCGAAATCGCA

CAGCTCGGGGTCGGCCGGTCGTTCCAGACCGCGTCGATTCTCCCCGAACT

CACGGTACGCGAGAACGTGCAGGTCGCGTCGTTCGCGGCCGAACACGGCT

CCTTTCGGGTCAACTTCTTCCGCCGACGGGACTCCTTCGACGAGGTCCAA

TCGCGAACGAACGACATCCTCGACACCATCGGCCTCGACTCGAAGGCCCG

GATGGAAGCCGGCTCGCTCCCGTACGGCGACAAGCGGCGCTTGGAGGTCG

CCATCGGGCTGGCGACCGACCCCGACCTGCTGTTCATGGACGAGCCGACC

GCCGGCATGTCCCCGACGGAGACGCAGATGACGGTGAACCTCATCCACGA

CCTGCTGGCCGACTGGGGGATGACCATCTTCCTCGTCGAACACGACATGG

ACATCGTCTTCGACGTGTCCGACCGCATCTTCACGCTCCACCAAGGACGG

CTCATCGCGCAGGGGACTCCCGAGGAGATTCGGGAGAATCCGGCCGTCCG

CGAGGCCTACCTCGGAGGTGGGGAGCAATGAGCCTGCTCGAACTGGACGG

CGTGCACGCCTACTACGGGCCGAGCCACATTCTCCGCGGCGTCTCGTTCG

ATATCGACGAGGGCGAGGTCGTCACGCTCCTCGGCCGCAACGGCGCGGGG

AAGACGACGACGGTCCGCAGCATCGCCGGGACCGAACCGCCGGCGATTCG

GTCAGGGTCGATTCGGTTCGACGGCGACGACATCACGGACTGGCCGGCCG

ACAACATCGCCATGGGCGGCATCGGCGTCGTCCCCGAGGGGCGGCGGCTG

TTCACCGAACTGACGGTCGAGGAGAACCTCGAAATGTCGAAAATCACGCG

CGGCTGGTGGAACACCATCCGCCGGGGCGGCCTCGGCGGCGGCGAGAGCA

CGATGTCGATAGACGAGTTGTACGACCTGTTCCCGCGACTCGACGAGCGG

CGCACCCAACAGGCGGGGACGCTCTCGGGCGGCGAACAGCAGATGCTCTC

CATCGCGCGGACGCTCAGGCTGCCGAACCTGAAACTGCTGTTGCTGGACG

AGCCGACCGAGGGACTGGCCCCGCAAATCGTCAAGGCGGTGGGCGACTCC

ATCACGGAGATTGCCGACCAAGGGCTGACGGTGCTTCTCATCGAGCAGAA

CGTCCGCGAGGCGCTCCGCATCGCGGACCGCGGCTACGTGCTCGATCAGG

GCGACATCGTCTACGGAGGCACCGTGGACGAACTCGAATCCGAAGACCTC

GACGAGTACCTCGTCGTCTGACCGCGCCGGCCTCGCGCTCCTCGGCGCGA

CCGACCGCGCTCCGTTCTATCCCACCTCGATTCCGTTTCGGCCCGTTCTC

GTTCCGACACCTACCTCGGCCTCAGTCTCGAATTTCGAAGTCGGAACCCA

ACCCGCAGCCGCCATCAATCAGTCGACAATATTATTTCGTGACACTCGAA

CACACGGGACATGAAACGGACCAATCGACGACGGTTTCTCGCCGCGTTCT

CCTCGTCGGCGTTCGCGTTCACCGCGGGATGTCAACGCCCGGTGGCGTCC

GCGTCCGAGCGCCACCCCGTGTCCGAGCCCGTCACCTCGTGGCCGACGTT

CCGCGGAGGGCGCTACAACACCGGCTACATCGGCGACGTGTCACCCCTCG

ATTCGGAGCCCTCGGTAGAGTGGACGTTCGAGGCGGGCGACGCGTTCTGG

GGGAGCCCAATCGTCGCCGACGGCACCGTCTACATCGGGAGCGCTGACAG

CGCGCTGTACGCGCTCGACGCCGAGACCGGCGAGGAGCGCTGGTCGTTCG

AGGCCGGACACCGAATCGAGGGGACGCCGGCCTACGCCGACGGCGTCGTC

TACGTCGGGTCGTACGACAAACATCTGTACGCGCTCGACGCCGGGACCGG

CGAGGAGCGCTGGTCCCGTGGGTTCGGCGGCCTGATTCGGGGGAGCCCGA

CGGTCTGGGACGGCACGGTCTACACCGGCGTCGGGTGTCACAACCTCGCG

TGCGCGTGGTACGCCGAGGAGGCCAACGTCTCGGAGACCGGCTGGGTGTA

CGCCCTCGACGCCGAGACGGGGGAGACGGAGTGGCGGTACGAAGTCGGTG

ACGAGGTCGTCAGCACGCCGGCCGTTACGAACAACCGCGTGTACGTCGGC

ACCTCCGACGAGGCGCTGTACGCGCTGTCGCGGTCGACCGGCGAGGTCGA

GTGGACCTACGAGACCCGCGACATGATATGGTCGAGTCCGGCGGTCGCCT

ACGGCTCCGTCTACTTCACCGACTGGAACGGGAACGTCCACGCCGCCGAC

GCGGCGACGGGCGAACAGGAGTGGCTCGCGGACACGGCGGGCCGGTACAT

CTCGGGGTCAGTCGCCGTCGGCGAGGAGGCCGTCTACGTCGGTCACACGC

CGTACAACACGCTCGACGACCCGACGATCAACCACGCGAAGGTGTTCCGC

TTCGACCGCGAGAGCGGCGCTGAGAACTGGAGCTTCGAGACGCCGGCGCT

GGAAGTCGGGAGCAGTCCCGTCCTGACTGAAGACGTGCTCTACGTCGGAA

CGCACCGGCAGTCAGACGGCGACGGCGTCGGCGTCCACGCCATCACGACG

GACGGGCAAGAAGAGTGGTTCATGGAAATCGACGGGCGCGGCGTCGGGTC

GAGTCCGGCGCTCGTCGACGGGCGACTGTACTTCGGCGGGACGGACGCGA

AGGTGTACGCAGTCGAGTAGCTTGGCGGCGCGGGACAGCCCTCACGACGG

CCCGTATTCGGTAGCTGTGGCTTGGAGACCCCTCTAGAACTCGATTTCCT

TGAACTCGCGCGCCTGATTCTCGATGGCTTCCTCGGTGGCGAGGCGCTCG

ATGCTCGACGCGCCGAAGAAGCCGACGACGCCCTCGGTGTTGTTGAGGAC

GTACGCCGCGTCGTCGGGCCACGCGATGGGGCCGCCGTGGCAGATGACGT

GTACGTCGTCGCGGACCGCCTTGGCGGCGTCGTGGTGCGCCTGCACGCGC

TCGGCCGCGTCGTCGAGGTCGAGCGCCGTCTCCGCGCCGATGTCGCCCGA

AGTCGTCAGCCCCATGTGCGAGACGATGACGTCCGCGCCGGCCTCGGTCA

TCGCCGCGGCCTGTTCTTCGGTGAAGACGTACGGGCACGTCAGCATCCCC

TGCTCGCTCGCCTCGCGAATCATCTCGACTTCCTTGTCGTAGCCCATTCC

GGTCTCCTCGAGGTTCTTCCGGTAGCTGCTGTCCTCGTCGATGAGTCCGA

CCGTCGGGAAGTTTTGGACGCCCGAGAAGCCGCGGCGACGCAGGTCTTCG

ATGAACACGCTCATGTCGCGGAAGGGGTCGGTGCCGTTGACGCCGGCGAG

CACCGGCGTGTCCTCGACGACCGGAATGACCTCGTGACCCATCTCGACCA

CGATTTCGTTGGCGTCGCCGTACGGCAGGAGGCCGGCGAGGGAGCCGCGA

CCGTTCATCCGGTAGCGACCCGAGTTGTAGATGATGAGGAGGTCGACGCC

GCCGCGCTCCGCGAACTTGGCGGAGATGCCGGTACCGGCACCGGCACCGA

TGACCGGGTCACCGGCTTCAATCGTGGCTTCGATACGGTCGAGTGATTCA

GCGCGTGTATACTTCATCCGGGTTGACAGGCCATCGCGTGCGACTTTAAT

ATTTCGGAAGAATCGACCGGCAGCCGAATGTCCGGCGAGCGACCCGGCCG

CGACGAAGTTCGGTACGAGGGCGCTCGACCGCGCGAATCAGTTGCCCGGG

AACTCGCCGGGCGGGAAGACCGTCGCGTCGTCGTCCGCGCGGATTTCGGC

TTCCGGGCCGGGCGGGCAGTAGACCGCGAGGAACCGAAGCGGCTCCCACG

AGGTGTTGATAGTGCTGTGTTCGACGCCGCTCGGGATGTGAACCATGTCG

CCCGCGCCGACCGTCCGGGTCTCGTCTTCGATGGTCTGTTCGCCCTCGCC

GCCGAGGAAGTACAGTATCTCCTCGCTGTCGGGATGGGTGTGTCGCTCGT

GGCCCTTCCCGGGTTCGAGCAACACGACGCCGGCGCTGAACCCCTCGGAG

CCGGTGACCTCGGGGGCGTTCATCCACTTCAGCGTGCCCCAGTCTAATCG

AATCGTCTCCACGTCGTCCGGCTGGACGAACCGCTTTTCGGATACCATGA

CGTGCTGAGTCAAACAGTATCAATTATTAAATATTTTGGTGGGCCGAACG

CGCTTGCCGGACCCGCAGAGCGGCCGTGGCCGAGCCGCGGTTTTCGAGAG

GGAAACAGTGGCTCGGAGCAGCAGGTCGTGGATTTTTGCCACTGAACCGA

TGGAAGAGCGGTTCAAACCCCGGTTCGTCCCCAGTCGACCAGTCGGCACT

CGCGTCGGCGGCTGCTCCGTTCTCGTGTTGTAAATTATTAATACAGAGGT

ACATTCGAGGGCGACGCGAGCGCGAATCAGTCGGTGACTGACCGAGAAAC

GCCGCCGAAGCGAGGGTACCGTGCCGACATCGGCGGACTGTCCCGCCTCG

TCGACCCGAACGATTTTCGCCTCGCGCGGCAGATGCGGATTCCGACCGCC

ACGAACGGCGTCGCTCGCGTGTCGAACGCGAGTCGCGTGAGGGGTCGAGG

GCGACTCCTCGAACACGGATTTTCATATCGAAAATGAGGTGAGCAGGCGC

GCGGGTGGCTCGGCGTCGTGTCGGTACTCGCCCGCACCTTCGGTCGGCAT

TACTGACGTACGTTCGTCACACAACCATTACAAGAATATGTCTGTAATTA

CTACACCGTTACCAAGCGTTTACGAGACGAGGAGGGGTCAGTCGTGGTCG

CCAGTCGAGTTCCGTTCTGTGCGTCCGCCCCGCGTGAGCGCTGAGCGATG

TCGTGACGGTGGTACGCCGGCAGTACTCTGAATACCCGCGAGACGTGGCG

ACCGCTTGGGCCCACCCCGTTTACGAGAACACAACCAGCACCGACTCCGC

GTCGGGTGACGAGACGCGTATCCGAGAGTGCACGAGATGCAAGCAGCGCA

CCACAGAGGACGTTTTCGAGACGTGAATCGGCGCGGTCCGACGGCGACGG

CCCGAATACGGCCGTCGCGAGTCGGTTCAGAACAGCGCACCGGCTTGGAG

GACCGCCACGAGGACGGTCAGGACGCCGACGCTGGCGACGGTGGTGGTGA

AGATGGTCGTACTGAGGTACTCGGGTGCAGTGATGCCGTCGACTGTCGTC

TCGTCGGCGTACTCGATGGTGAGCGCCAGCGGGATGACCGCGGCGGGCGT

GGCGCATTCGAGGACGAACACCTTTGCGACTGTCGGGTCACCGAAGCCCA

ACGCGAGCGCGAGGCCGAACCCGACGAGCGGCGCGACGACGAGCTTCAGC

GCCGACGGGGCGACTGACCGCGATATCGCGGACACGTCGGTCTCGGCCAG

TTGGATGCCGAGGATGAGCAACATCACCGGAATCGACGCGTCGCCGACGA

GGCCGACGGTCTCCATGAGCGCCGCGTCGGCCGGCGGGACGACCCCGAGC

GCGCGGGCGACGACGCCGGCGACGATGGCGTACAGCAGCGGGAGACGGAA

TATCTCGGTGACGGCGTCGAGTGCGCCCGCGTCGGAGCCGTTGGACGCGA

TGTAGACGCCGAGCGTGTAGACCACGACGTTCTGGATGGTGAGGTAGATA

ACGGCGGTCGTCCGTCCGACCTCGCCGAAGGCGAATCCGGAAAGCGGGAT

GCCGACGAAGCCCGAGTTCGGGAACGCCGCCGCCAGCATGAGCGCGCCGA

GGAGCGGGCCCGATTCGCCCGCGAGTCGGCCGACGCCCCACGCGATGACG

ATCATGAGAAGGGAGTATCCGACGACGCCGAGGCCGAGTTTGAGGACCTC

CCCGCCGCCGAGGGTCGTCGTGGCGATGCTGTGGAACGCCAACGCCGGGA

TGAGCACGTACAGCCCGAGCGTGTTTATCGGTTCGACATCGATGTCCGTC

GTTCGCGCCAGGAGATAGCCGACGACGGCGACGCCGAGTATCGGGAGAAT

CGCGTTCGTGAACGCCCCCATTAACGACATGGTCGGTCTCCCCGGGAAAG

TCGCTTCGGCGGTCGAACGGGTTCAACTGAGACTCTACGCTGTCGCTGTG

GGCCCATGTCAGTCCAATATCGCTTCTGATATAAATAGTATCGGAGACGG

TTACGTGCGGGTCAAAACCCGTTGCCGACGAGAGTGACCGCCATAGCGAT

GTGACCCGGTCTTTCCCCTTCGAAAACCTCGTGGCCATGCATGTGTTATG

CAACGATACTATCGGAAAATCAAAGGACAGAGAGAAACGACAGCCCACAA

TCCAGATTTTCTCAAAGATTATCCATGAGTGCGTACTGAAAGGAGTATCG

CAGGTCCATAGCCACCACAGAACAACCAGTATTTTAAATTATTCAAATAA

TCCACATTATTCACAATATTTGGATTTCTTCACAGACCAGTGCTATTTCA

GTAGAGTCGCAATATAAAGTACCGAAACCCGTTCGGGAACGACACCGCGA

TTCGCTGAGCGGGGGATGCGACCATGACCATGACAGGACGACTATCGGCT

TCACCTCCGGTCGAGTGGAAGCGTACGCGGCAAACGTCGTCGAGAGCGGA

GTTGTGAGTCGTGAGGCCATTACGGGAACTACGACAGTTCGACGACACGC

TCTCCCGGTTCGTATCCGAAGTTTGACCGACGTCGACCCCGCGGCCTGCG

TAGCCACCTCTCACTCCACGTCGGCTCGCAACCGGGCCACTCCTTCGGGG

GGTACGTCCGGTGGGACGCTCGTCGGACGGTGGCGGACCGGTATGGATAC

CGGAGTATATTCCGAATTAGAGAACAGCTTCGAGAGATAGGACTTTCAGC

CTTCTATGGCCTCTTCAGTACCAACCGAGAGGGCAAAGATGAATTCAATT

ACAACGTTATGGGTGTAATGGAATTGCGCTGAACGGACTTACTGATGGCC

GAGAGGAGAAACACAGCGTGAGTATGGAAACTACTTTCTGAGATTAAGAA

CACTGTGGAGACTGTGCTCATCGGCGTACCGCTCGATGGTCAGGAGTCGA

ACCGACGGAGACAGTCGGCCGTAATACGACCACCGGTCTCGTGCCACCCA

CCGGAGAAAGGGAGTGTGTATAGTCGTTCTTACACTATCTTCGGTTTCGT

GTCTATGCACGTACAGTGTAGCCATCGTTCACCGGAGTAGCCGACGGCTT

CTACTAGTTCGTTGCAGGCGGTCCCGCCGGGTCACCCCGGGCGGAGTCTA

TTCTGTATTTCAGAATGCAGTTGTTCGTCCCAAGACTACGGGGCGGGCAT

GAGCGGTTCAGTCATCCACGGGTTGTATCGGCGGCGATCCGGGAGGCGTC

GATAGCGTCGCCGCACCTCGCCGGAGACGAACCGCAGTGCCGACTACGGC

CTTCACCGACGAATCGAACCACGCGTCGCTCGCGTCTCCCCTCCGATAAA

ACTAGCTCCAAGTATCAGCCGTGGCTCTCCGGAAAACCGGGTTCGTTGCG

TTTCACAGGGCCGCTATCAGGTGTTCTGCCCAGCCGTCACCTCTAACGGT

GGTTCTTCTTGTGCGAGAATGTTGATGCCGTCAGACTGCCGAAAGAGATG

CAACCGCTCCTCGTCGAAGGCGAGCGACAGCTCTTGGTTCGTGTCGGGTT

TGACATCGCCAGGCACGCGGACCCAGCACTCCTCGCCCTCGATGTCGAAG

TGGAGGTAGTTGTCCGACCCCACCGGCTCGCGGACGTCGAGGAACGCTCG

AACCGCGTTGGGCTCGTCGCTGTCTGTGAGGGAGATGTTCTCGGGCCGAA

TGCCGAGCACGAACTCGTCTACGCCATCGGCGTACTGCTTGATATGTTCG

AGAACTCGCTCGGAGAGGTCGTAACTGAAGCCGTCGGCGACCAGCGACGT

GCCCGACAGTCGTACCTCGAACTGGTTCATCGGCGGGTTCCCGACGAAGT

CGCCCACGAACAGGTTCCGAGGTTCGTTGTATATCTCCTCCGGCGTCCCT

ATCTGCTGGAGTTCGCCGCCGTCGACAACAGCGATGCGGTCGGACATCGT

CATCGCCTCCTCTTGGTCGTGAGTGACGTACACCGTCGTCGTCCCGAGCG

TGTCTTGGAGCTGCTGGAGTTCCGTCCGCATGTGCAGCCTGAGCTTGGCG

TCGAGGTTCGACAGCGGCTCGTCGAACAGGAAGACGGACGGCTCGCGCAC

GATGGCGCGGCCCGTGGCGATACGCTGTTGCTGTCCTCCAGAGAGGTTTC

CAGGATGCTTGTCGAGCTGGTCCGTAACACCCATCATCTCCGCCGCGTCC

ATCACCCGCTGGTTTATCTCGTCTTCGGGGAGGTCGGTCGTCAACTGCAA

CCCGTAACTCATGTTCTTGCGCGCGGTCATGTGCGGGTACAACGCGTAGT

TCTGAAACACCATCGCGACGTCCCGGTCCTGCGGCTGGACGCCGTTGACG

ACCCGGTCCCCGATACGTATCGTCCCTTCTGTGATATCTTCCAGACCCGC

AATCATACGGAGCAACGTCGACTTGCCCGACCCCGAGGGACCGACGATAG

TCAGGAACTCAGCGTCCGCTATCTCGAAGCTGATGTCGTCGACGGCGACA

ATATCCGAGTCGCCACCGTAGACTTTTCGCACGTTTTCGATGCTAACAGT

ACTCATTGTTCGTGGGTTGCCAAGAGAACTAATAATAGTTGTGGTTACCT

CCGCTCGAAGTCGCGTCATTCGGGGACGCGAGCGCAGTCTGGACGGCGCT

CGCTCTCCGCGCCGCCACGACAGACAGGCGAGCGAGTAGTGCGTCGGATT

TCATCTACCGGCAGAATTAGTCACGGAAAATAGTTTAATCCATACCCCTA

CATATACACATGACACATGGTTCGAGAAGCTATCACTAGCAGGACCCGTC

GGAAGTTTATCAAATCTGCCGGAGTCGCGGGAACCGTCGCACTCGCGGGC

TGCGCCGGTAACAGCGACAACAGCGGAGATAGCGGAAGTGGCGGCGGTGA

CGGCGACAGCAGTGACGGCGGCAGTTCCGGCAACACGCGGAGCGAGTCCT

ACGAGCTCACGTTCTGGGAGCTGTTCAGCGGCGGCGAAGGGCCGATTATG

GAGAGCATCGTCAACAAGTTCAACGAGGAACAGCCGCTCGACACGGACGC

GGAGGTGACCATCAACCGCCAACGCACCCCGTGGAACGAGTACTACAACA

AGCTGTTCACCGCCCTCTCCGGCGGCGAAGCGCCAGACATGGCGGTCATG

CACGCCGCGTACCTGCGAGCGTGGGACGGCGGTATCGACGCAATCGGGTC

CTACGCCGACACGGCGTCGATGGAGAGCGACTACACCGAAAGCCACTGGG

ACCTCGTCACCGTCAACGACAACGTGAACGCGCTACCGATGGACATGCAT

CCCATCGGCGTGTACTACAACAAGGACCTGTTCGAGCAGGCCGGCTTGGA

CCCCGAGAGCCCGCCGACGAACTGGGAGGAGTTCCGGCAGGCGGGTGACG

CGGTCGTCGAGAACACCGACGCTCACGCGTTTACGCAGTCGCCGTACAAC

GACGGATTCGGGTCGTTCCGGACGTGGAGTACCTGGGTCAAACAGCAGGG

CGGGAGTCTCTACGACGACGAGTGGAACCCGACGTTCGACAGCGAGGCCG

GGATGAACATCGCCCAACTCTTCTCCGACATGACCGGCGACATGGGATGG

GCCCCCGCCACGAGCGAGGACACGTGGGGGAACAACGCCTTCCAGAACGG

AACGTGCGCGATGGCGATGAACGGGACGTGGTACGTCGCCGCCCTGTCCG

GCGTCGACGACCTCAACTGGGGCTTCTTCGAACCGACCGTCGGCCCGAAC

AAACAGCAGGATAAGGTCTGGGCTGACGGCCACTCCATCGTCCTCCCGAA

GAAGGAGGGCCGGAGCGACGCCAAGTCGGAGATTGCCGCCGAAGTCGCCC

ACTGGATGACCACGCAGAACCCCTCGTGGGGTGCCGAAGCCGGTCACCTG

CCCGCCGCGAACAGCATCTACGAGTCCGACACCTTCCAGAACAGCCCGTA

CTACGATAAGACGCTCAACAAGTACGTCGAGATGGCCGAAGACGGCGACT

ACTTCTACCACCCGAAGGTCCCGAACGGCGACCCGAACGCGCAGAACTGG

TACACGTGGCTCGTCGACATCTGGGCGCAGAACTCCGAGCCACAGGCTGC

TATCGATTCCGGAATCCAGACGGTCTCGAACGGCATCTCGGAGTAATCGA

AGATGGGGATAACAGAACGCATCATGGGAGGTGAGACCGCGGCGAACAGC

AAGACCGTTATGGGTCTGCGGAAGGAGACCATCGAGGGCATCGTCTGGTC

GCTTCCGTACCTCGCCGTGTTCACGGTGTTCCTCATCTGGCCGGCCCTGA

AGGGGCTCTACATGAGCCTCCACACGTACCCGAACAAGTTCGACCTCACC

GAGACGGAGTGGGTCGGCCTCCAGAACTACGTGGAACTGTTTCAGGACCC

CGTGTTTTACAACGCGATGGAGGGAACGCTCCTCTTCGTCGCTATCTCGG

TTCCGGCGCTGGTCATCCTCGGCTTGGTAATGGCACTCGGCGTAAACCGG

GACGTGGCCGGGAAACGAACCCTTCGGGCCATCTACTTCAGTCCGTACGT

CCTCACCGTCGCGGTCGTGACGCTGGTGTGGGGACAGGTGTACTCCCAGA

GCTACGGCCTGATAAACTACTACCTCGGGTTCTTCATCGACAACCCGCCG

GGGTGGCTCACGTCGCCGGACCTCGTCATGGCGGCGCTCGCGTTCATGAC

CGTCTGGTGGCTGGTCGGGTTCAACTTCGTCATTTTCCTGGCGGCCCGAC

AGAGCATCCCCGAACGGCTGTACGAGGCCGCTCGGCTCGACGGCGCGACC

GGCTGGCGAGCCTTCAAGGATATCACGCTGCCCCAGATGCGAAACTCGGT

CGTGTTCGTGGTGATGGTGCAGTTCATCCTCCAGTTCCAGGTGTTCGCAC

AACCGTACGTGATGACGCAGGGCGGCCCGAAGGACACCTCCGACACGCTC

GTCTACTATCTCTACCGGAGCGCCTTCTCCCAACAGCAGTTCGGCTACGG

CGCGGCGATGGGGTACGTGCTGGTCGCCATCCTCATCGTCATCGCAATCG

TGAACTTCAAAGTAATCGGTGATTCAAATGACTGAAACGACATCGCAGGA

CTCGATACGAGCACGGCTCGACGGCGACACGGTTCGGAACGTGTTGCTCC

ACGCGTTCCTCTACGGCCTCGCCGTTCTCATGGCGGTCCCGTACCTGTAC

ACGCTCTCTCGGTCCTTCCAGCCGACCGAGCTGTTGCGCGACCCGCGACC

GTACTGGATACCGCCGCTCGCGGGCGAGCCGATAACGCTCGAACACTACC

AGTACCTCCTGAACAACACGCTCGTCGTCGAGTGGACCATCAACACGTTC

ATCATCGCCGCCGGAGCGACGCTGCTCATCGTCGTCATCGACTCGATGAT

CGCCTTTTCGCTCACGCGGCTCGACTGGCCCGGTCAAGGGCTGGTCATGG

GTGTTATCCTCGCGAGCTTCATGGTCCCGTACTACATGAACATCGTCCCG

CTGTACCAGATCGTCTCGGACCTCGGCCTCATCAACACCTACTGGGGTGT

GATACTGCCGGCCGTGGCCAGCCCGCTCGGCGTGTTCCTCCTCTACCAGT

TCTTCAAGGACATCCCGGCCGAGTACGAGGAGGCGGCGAGGCTCGACGGG

TTCACCACCTTCCAGGTGTACACCCGAATCATCCTCCCGCTCGCGAAGCC

GATTCTCTCGGCGCTGGCGCTGTTCATGTTCGTCTACAACTGGAACGCGT

TCCTCTGGCCCCTCCTCGTGCTGTCGAACGAGACGGCGTACACGCTTCCC

ATCGGACTGGTGAACCTCTATCAGGGGAACATCGACACGCCGGGACTCCA

CATGGCGGTCGCGATTCTGGGGTCCTTGCCGCTGTTCATCATCTACCTCA

TCTTCCAGGGACAAATCGTGCGAGCAGTGCAGATGCAGGGCGCGACGGGA

TGAGCGAGTCAGCGGACGGGACCGTCGGCGTCCACCGCGCGCTCGTCGCC

GCGGTCCGAACGGTGTTCCATCACCCCGTTCGGATGGTCGCTATCAGCGT

CGCGTGGGTGCTCGCATCGGTCCCGCTGGTCACAATCGGGCCGGCGACGT

TGGGCGTCTACGGCGCCGTCGCTTCCGTCCGCGAACATGGCCACGTCGAC

CGCGACGCCGTCAGAGAGACGCTTTCGGACCACTGGCTCGACGCGCTCCT

GTTCAGCGGACTCCTGGTCGCGTTCCCGGCGCTCGCCGTCTTCTATCTCG

GCCGGTTCTCCGCCTCCGGGTCGGCGTTCGCGGGCGTCCTCGGGATGGTC

GGGTTCTATCTCGCGTATCACGCGTGGGTCGTCTTCGCGCTGGGCTTCGT

CGCGCTCGCGCAGGGCGACGACGCGTTCGACGCCGTCACCGACGGCTACC

GGTGGTCCGTCGAGCGGCCGGTGGCGACGGTGCTGGTCGGCGTCGTGACC

GCGACGCTGTTCGTCGTATGTCTGGTCTTGACGGTCGCACTCCCGCTCGT

GTTTCCCGCGCTCGCGGCCGCGTTCCACACGGAACTGGCCGCCCAAACGC

GCGACCGAGAAGAGGATGCGACCGACGCGGCCGACACAGCCGACGCGACC

GCGCCGACCGACCGTCCAGTCTCCGGTGAGAGTCCGAAAAACGAGTTCTC

CGCGACGTTTCGACGGTGACGCTACGTCCCGTCGTCGAGGGGTAGCGACT

GGACGCCGTAACTGATTTCACAGAGATTCGGGCCGTCCGCGACCGACGTG

AGTTGCAGTAGTTCGTCCCCGTGGCGCGAGGGCAGCAGCGGTGTCGTCCA

CCCGACGAACGCCCGACCGCCGAGGTCGCCCTCCGTCTCGAACGACAGCG

GGGCGTCGACCGCCTCCCAGTCGCCGCTCCCGTCGAGGTTCGAGTTCGCG

AGCACGACCTCGCCGTTCCCCTCCGCGAGGTCGCGGTTCCCGTCGACGAG

CTGTTTTCCGGACACGAGTATCGTTCCGTCTTTGCCGCCGGCCCGTGTCC

ACGTCACGTACGGGCCGTTGATGAACCGCCGACCGTCGGCCGTCGAGACG

AGGGTCCCCACGTCCGTCGGGTCGCCCCACTCCCGCCCGTCGGGCGAGGT

CTTGACGTGGACCTCGCCGTACAGGTAGTCCGGCCCGATGACCTCGTAGC

TCATCACGTACGTGCCGTTGGGGAGCGGGGTGACGGTGGGCATCCCCGGG

CGTGTCGTCTCGTCCGGGATGGCCGCGACGAACTGCTCGTCGCCCCACGA

CTGGCCGCCGTCCTCCGAGGCCTTGTACGCGACCAACTGATTGTAGTCGT

CGTCGCTCCCCATCCGCTCGTCGGCGAAGTAACAGACGAGGTTCCCGTCG

TCGTCCAGTCCCAGTTCCGGCTCCCAGACGGGGCTGTTCCCGTCGTAAGG

GACGGCTTTGCCGCCCGTGACGACGGTGCTCACGAACTCCCAGGACTCCC

CGCGGTCGGTGCTGGCGTACAGGTCGATGCTCGTCTCACCGAGTTCGCCG

ACCTCACCTTCGGGGACGTCTTCGGGGTCGTCGAGGATCGGAATGGAGTT

CCCGGCGGCGAGGACCGTCCCGGCCGGCCACGGACCGACGGCGTGCGGGA

GTTCAAACAGGGTCGGCTGGTATCGAAGCCCCCAGTCCTTCCCGCTCGTG

TCGTGAATCTCGGAGAACTTCGACCAAGTCTCTCCCCCGTCGGTGCTTCG

GTAGACGGGGAAGTACGGCCTCGACCCGCCGCTCATCGACGGATAGTACT

CGAACGTCGCGAGCAACTGCTTCTCGCTGCCTTTCCCCGACCCGCGTTTC

AGTCGCGTGACGCGGGGATACAGCGACCCGGGAGCGGGCGCGCCCGACGG

GGGCGAGTACAGCGTCCCTTCGACGTCTGTCTGTTCGTCGTCGCGTCCGC

GGCCCGCGATAACGCCGGTTGACCCGACCGCTGCGACCCCGGCAGCGCCG

AGCGCGGCGAGGTACTTCCGGCGGCTATGCTGGCGGCTTGAATTGCGTTC

GCTCATGACGCAGCCGCATGTTAACACGGGACTACAATAACTGTACCCCC

GCCGTCAGCTCAAAATAGAACGGTGGCGGCGCGGTTACTCGGTCAGCGTC

AGACCGACGACGGACGCCGGCGGCGCGTCGAACGTGACCGTGCCGTCGCC

CTCGTCGGTGACGTCCAAGACATCGGCTTCGAACGAGTCCGCGTTCTCCT

TCGTCGAGTACTCTCGGATGTCGTTGCCTTCGAAGAGAACGGTCGCCGAC

GACACCGAGTACGCCGACGACCCGACGTCGACGGTGAGCGTCTCCGGTTC

GTCGTGGCGGCGGTTCGAGGCCGTGACGAACAGTTCACCGTCGCCCTCAG

AGGCCGACGCCGAGACCATCGGTACGTCGTTTTCTTCGCCTTCGATAGTG

CGCTGTTCCGTGTCGACGCTCGTTTCGAGCGCCGTCCGGCCGACGTGGTT

CTCGTACAGGTCGAAGACGCGATACGTCGGCGTCCGCCACGCGTCCTCTT

CGTCGGTCTGGACGAGACACTGCAAGACGTTGACGAGCTGTGCGATGTTC

GCCATCGAGACGACGTCGGCCCGCTCGTGGATGAGGTCGAGGACGCCGGC

GGTCGTCAGCGCATCCCGAACCGTGTTCTCCTGCTCCAGTCCGTTGTCGC

TCCGCGCCTCCGGATGCCACACGCCCCACTCGTCGACGATGATGCCGATT

TCGTCCTCCGGAACGTACTGCGAAATCGTCTCGGCGGCTCGGTCGATGTC

CCCGCCGACCTTCTGTGCGCGCGCGAAAATCTTGTAGTACTGCTCGTCGG

TGAAGTCGGTGTCACCGCCCGCCTGATAGTAGCGGTGGACGGAGAAGTGG

TCCATCAGGTTGAACGGCGACCCCATGCCGAGGAACGAGCCCGGACCGTA

CTCCATGCCGCCGTTGAGGCTGTCGAAGAAGACCTTGTTCCAGTCGTCCG

TGAGGTGTCCGCACGCGATGAACTCCGTCGACTCCTCGTTCATCAGCTTG

TCGAAGCCGTTGAAGTAGTTCGCGAACCGGCGGTATTCGTCGGCGTACTC

GTCGGGTGCGAACCGGCCGCCACAGCCCCAGTTTTCGTTCCCGATGCCCC

AGTACTTCACCCCGTACGGCTCTTCTTGGCCGTTCTCTCGCCGTCTGTTA

GCGAGTTCCGTGTCGCCGCCGTAGTTGCAGTATTCGATCCAGTCGAGCGC

CTCCTGCGGCGTCGACGACCCGACGTTGACCGCGATGTACGGGTCCGTGT

CGAGGAGTTGGCAGAGTCGGAGGAACTCGTCCGTCCCGAACTCGTTGGAC

TCCTCGGGGAGGTTTTCGCGCCCCTGGGTCCACCACAGATTCCGCCGTCG

GGGACGCTCGTCGCGCGGGCCGACGCCGTCTTCCCAGTGATAGTCGTCGG

CGAAACAGCCACCCGGCCAGCGAAGCACCGGCATGTTCAGTCCGCGGAGC

AGCGAGACGGTGTCCATTCGGATGCCGTCTTCGGTCTCGACTCTGTCGTC

GTCACCGACCCAGAGACCGCCGTAGATACAGCGTCCGAGGTGTTCGGCGA

AGTGGCCGTAGATGTTCTCCGAGATACGGTCTATCGGCTCGTGACTGCTA

ACGCGTACTTCACTCTGCATACGTTCTCCCATCAGTACCCGGTTAGATAA

CTATTGTGCCTTGTTCCCTGCTTACACGAGATGCGCTGTGGAACGGTCCG

GATAAGCGATGAACCCGCGTCAAGCGTTGGGAGCGCAAGTCGGTGAGAGA

CGCGTTCGGTGCAACCGCACCAAGGTTCCCTATTTCAGAACTAGCTAACG

TCGTTCCACGCATTACAATTACAAAGGTAAATATGTAATCTCCGTGGCGC

CCAAAGTGGATACTCTCCGAAACCGGTCTTCTCGGCCGGTTTCGAGGAAC

CCGACGTGTTGTTCCAGCATAATGAATAGCGGCAAATAACCGGGAACCAT

CGTCGCGGATGGGTAGTTCGACGACAATCGTCGTTCTCGGAGACATCCAG

CGGACTCCTCGTGAAATCTATCCTCACCGAGCGACGAGCCGACGCGAGAT

ACGAGCCGTTCACGGAACGTCGCGGAGACGTTCAGAGGTTGACGCACCGG

GAATCCGCAGGGACTCGTCGATGTCGATGGCGCATCCGCGCTCGCGAAAA

CCGTATCTCAGTCACGCAGCCGTCTCCAGACTGTAACGAGGATCAGTACT

TCACAAAGCCGTTTAGTTAGAAGGTCTGCTTGCAGAAGGTGTGTCTAAAA

CCCAGCAAGCAGACAGTGAAATCCATGAGGACCAGCTCCTTAACTTCCTC

GTCAACTCCCTTGACGAGGAAGTTGCTCTCACTCTCGGTGAAAACGCTGA

ACTCGATGCTGAAGACATCTACGAGGTCCTCGTCGGCGCGTGCGCCGACG

GGACCTCGGTCTCAACGCTCTGTGAGAGAAGCGAAGATGCACCCCACGAA

AACTCCGTTCTCTACCATCTCCGCACTAAATTCGATCTAGAAACGCTCGA

ACAGGTTGGCAACATGCTCCTCCAGAAAGACGTTCTCGACGTCCTTCCCC

AGCAGGTGGAGGTCTGCGCAGACCTCCACCTGCGGCCCTACTATGGTGAC

GAAGACGACACGGACGGCCTGTATCACTCACAAGCGAAGCGTGGAACCAC

CGCGTTCCACGCGTACGCGACACTGTACGCACGCGTGAAGAACAAACGCT

ACACGCTGGCGGTGCGCCGTCTCGAATACGGCGACACCGCCAGCAGTGTC

CTCGCAGAGTTCCTCGGTATTCTCGACGGCCTTGACCTCGGTGTCAAGGC

CGTCTATCTTGACCGCGAATTCTACGACAGCAAGTGTTTGACGCTGCTTC

AGGCGCACAACCACGCGTACGTCATGCCGATCGTCCGCTGGGGACGGACG

ATCAAGCGAGAACTCTCAGAAGGGTGGAGTCGCGTGATTCAGCACAGTCT

GACAGCGAAACTCGACGGTCACAGCTGGACCGTCGAGTTTCCCGTCTACA

TCGACTGTACCTACCAGAACGGACGGTACGACGAACATGGGGTGGCGCGT

CACGGCTACGCCGCTGACGCGCCGTTCATCAACTCACCACGAGACGCTCG

ATACCACTACGCGAAACGCTTCGGTATCGAGGCAAGCTATCGACTCTCCG

AGCAAAGTATCGCGACGACCTCGACACAGAATCCGGTCGTACGGCTGTTG

TACGTCGTGGTGAGCCTGCTGTTACAGAACGTCTGGCGGTATTTGCACTG

GGAGTACGTGGCGACGCCCCGCCGTGGGGGGCGTCGCCTCTGGGAGTGGT

CGTTCAAGGAGTTCATCAATATGGTCCGTCGAGCAGCGTGGACGGCCCTC

GCGGTGCGTCGGGCCGTCCCCGCGAACCGACCACCAGACGACCGGTTCCA

CCGGTAACTCCCGACCGGGCTAGCCAACGGTGGGAGTGGCGACGCTGTCG

CGTCGGCGGCAGCCGCCGCCGACAGCGACGGCTCTCCGCCGATCCGTCCA

TGATTCTCTCGTCGAAACCGCCAGTCCAACCGCTCCGCCACAGAACTCAG

GCTTCAGAAACAGCTAGCCGAGGATGCTTTGTGAGGTACTGAGGATGGTT

TCTGGAGCGCTGTCAGGCGGTTGCGGTCGGGCTTTTGTCCGTGGAGAGCG

TTTTGACCGACTCAAGGACCCGACACGCGTACGTTTCAATTCACACATCG

GGGAAGGAGCCCCGTGTTTCTGGTATCGAGAACAGCAGACCGGCTGGGGA

AAACGGGACAGAGCCGTCATCGTGAACTCAGGTGAGTCAGGCGACCAACT

GGTCTGCGACCCGGTCGGTTGCAAGCGCGCACAAGGGTTCTACCTCGGTA

TAGCGTCGGTGCTGGAACCGGTACGACGTTCCAAAAAATGATGGCAGCGG

GTGGTGTGCTGAGCTCGACGTGGTAGTGCTGTCAGGTGTTCATGAGCTTC

GGTCGGGACAGACCGACTCTGCGCATATCACCACGTCTGTGACATGACTG

ATACGTTAGTCTCTTGGTCGGTTGCGCTATCGACCATATGGCTTGACGAC

TGAGTTCGACATTAATGTTTTCGTCTCTGCAAATATTAATATCGGGACTG

AACGCAACTGCCCTTCGAGCGATACGACAATCGTCGTGGTAAGGGTTAAG

TGACTATCGAATGGTTACACGAAACGCATGCGTTGCCCAAAGTGCAGTCA

CAGTCTCGCCATCTACGATTCGTTTTACGACATCGCCTTCGTCTGCGACT

CGTGTGGATACGTCCTTCCGCGGGGAGCAGATTGAACAGACAGCCGAACC

GATAACCGACCAACCACACCGCTTAGCTGAATCGCGGCCGCGGACCGCAG

CGCCGGAAGAGGAGTCACAACGATGTGTGAACACAATCTCGGAGGCTGAT

TTTGCTGCGTGACTTATTTGCAGCGTCGGTACGGAAAATCAGCCGCTTAA

TAGAGAAGATGTACGGGTATTCATCTTCTAACGATATCGAATGTCTTGTT

CAAAATCGGAGTGATAACAATAAATCCGATGAAGATTGCAATAATAGAGA

AGATGAACTCGACACCAATCGTGACTGGGTCGGCAGTATCGCCAGTGTAG

GTGTTGTAGATTGTGAATATGGTCGCAGCAACTACGGCGTTTGGAAGGTA

ATCGGAGACCAACTTTCGTAACTCCATATCCGAACTCGTCGGTGGATCCC

CAAATTCGCATCGGTTAGGACCTAGTTGCCAGAGAATAGACACGGGTAGT

ATGCACCCGACACTACTCAGCTAATGCACACCAGATAACGCTGCAAGTAA

GCAACGCATCAAACCGATTCCCCTAATGAGAAGCAGGGGAAGAATCGCTC

AGTTCAAACTAAATCCCGCTACAAACGGTTCGAGAGTCTGATATCCGCTC

GATTTACTCGTGGACCCGCTGTGAAGCCAGTCGGTATCTTCCCTTGAGAG

TGAGGAAGGGAGACCAGAGCAAGAACGAACACCAGAGCTCCCCTAATAGA

GATACAAACGGATGGTCACCTACACGCCTCGGACCGATACGTCCGCTATC

TCGCTTGTATATAGCAGCCTCGCCGTTCAGTTCTGACGTGAGACGCCGGT

TCCAGAACTACCCTCCGAAACTGTGAGCGAAGCGGGGAATAAATATATCG

ATATGCGGTGTATAGACGTTGTCGACCCTCGCTCGCGGGCCGAAACGGAG

TACAGACAGGAGTTCCGTCACAGACACGCTTGCAGGGGCAGTCAGTTCAC

TCCGGAGAGGAGTACAGCGAAACGTAGCGCCCCCGTGGGTCAGTCGTCGC

CGCGTGCGGCGCTCCCAGCGCCGGCGTCCGGGGTCCGCAGCACGTCCGGG

TCGGCGTCCGGGCCGAGCGGTTCCACGCGCACGCTCGACACTTTGAACTC

CGGAATGCCGGCCTGCGGGTCGAAGACTTCCTGCGTGAGTTTGTTGACCG

CACCGGCCGCGAAGTGCATCGGGATGAACAGCGTCCCGTCGCCGACGCGG

TCGGTCACGGTCGCCTTGACGACGATGTCGCCACGCCGGGACTCGACCCT

GACGTACTCGCCGTCCGAAACGTCGAGTTCCGCGGCGGTCGTCGGGTTGA

TTTCGATGAAGCTCTCGCCGACGTGGCTCATGAGGCCCTCGACGCGTCGG

GTAATCTGTCCGGTGTGCCAGTGGTAGAGGACCCGCCCGGACGTGAGCGT

GAGGGGGAACTCCTCGTCGGGGAGTTCGCCGGGGTGGCCGCCGTCCGCCG

GCACGAAGCGGGCCAGCCCGTCGTCGAAGTTGAAGTTCCCCCCCTCGTAG

TCGTAGAGATACGGCGTACCGGGGTGGTCGTCGTCCCAGCACGGCCACTG

GAGTCCGTGCTCGTCGCCCGAATCGAGTCGCTCGTAGCTGACGCCGCCGT

AGATGGGCGTCAACTCGCTAATCTCGTCCATGACTTCCCGCGGGTGGTCG

TAGCCCCAGTCGTAGCCCAGTCTGGCCGCCAGCTCCTGCGTGATTTCCCA

GTCCTGACGCGCCTTTCCCGGCGGCTCCGCGGACGCTCGAACCCGCTGGA

CCCGACGCTCGGTGTTGGTGAACGTGCCGTGTTTCTCCGGCGAGGTCGCC

GCCGGCAGAATCACGTCCGCGTGTTCTGCGGTCTCCGTCATGAAGATGTC

CTGAACGACGAGGAAGTCTAAGCTTTCGAGCGCCTCGCCGGCGTGCTGGA

TGTCTGGCTCAGAGAGCGCGGGGTTCTCCCCGACGATGTACATCCCGCGG

AGATTGCCCTCGTGGGCCTCCGAAAGCATCTCCGGCACTTTGAGGCCGGG

TTCAGCCGGTGGACGCTCGCCCCACACCTCGGCGAATTTCTCGCCGACCT

CGTCGTCGGCCGGGTCCTGGTAGCCGGGGAGGCTCCCGGGCAGGGTCCCC

ATGTCGCCGCCGCCGCCCTGCACGTTGTTCTGGCCGCGGAAGGGCGACAG

TCCGGCGCCCGGTTTCCCGACCTGCCCGAGGGTGAGCGCGAGGTCGGCCA

TCGCGAGGAGATTCTCCGTCCCGTGGCTGGATTGGGTCATCCCCATCGCC

CAGCCGAAGACGACGTTCTCGGCGTCGGCGAGCGTCTCGGCGGCCGACTT

CAGTTCGTCGGGCGAGACGCCGGCCAGCCTCTCGACTTTCTCGGGGGTGA

ACGCCTGAACTTTCCGTTTGACCTCCGCGAACCCTTTCGTATTCCGTTCG

ATAAACTCCTCGTCGTGCAGGTCGTGCTCGATGAGATATCGGATGAGTCC

GTTGAGCCACGCCACGTCGTAGCCGGGCGTCGTTCTCGTGTACTGGTCGG

CGTGCTCGGCGATGCCGATTTTCCGGGGGTCGAACACCACGAGGTCGGCC

CCGTCGCGGACGTTCTGCTTGATGCGAGTCGCCAACACCGGATGCGACTC

GGTCGTGTTCGACCCGCTGATGAGGTAGGCGTCGGCCTCGCCGATGTCCT

CGTTGATGCGGTTCGTCATCGCGCCGTAGCCGAGCGTCTGTTGGAGCGCC

GCCACCGTCGACGAGTGGCAGAGCCGGGCGCAGTTGTCGATGTTCTTCGT

GCCGAGTACCTGCCGCGCGAACTTCTGGACGAGATACGCCTCCTCGTTGC

TCCCCTTCGACGACGCGAGGCAACTGACCGCGTCGACGCCGTGTTCGTCC

TGAATCTCGCGGAGTCGGCCGGCGACGTAGTCGAGCGCCTCGTCCCACGA

GGCCGCCTCGAACTCGCCGTCTTCGTTTCGAATCAGGGGCTTCGTGAGCC

GGTTTTCGCTGTTGGCAAAGTCGTGGCCGAACTTTCCTTTCACGCACGTC

GAGAAGTTGTTCGCCGGGGCGTTGTCCGGGTCGTCGACGGGTTGGACGCC

GATGGCGTTGCCGTCTTTGCCCCACATCTCGAACCGACAGCCGACCGCGC

AGAACCCGCAGGTCGTCTCCGCTTTGTCGAGGTTGCCCAGCCGGTAGTCG

CTGACGAGCGACGCGACATCGAACAGTCGGCCCTCCGGCAGCGTCCGCGC

CGCGAGGTTCTCGGCGGTGTGTTCGCCCGCGAGCATGGCCTTCCGGCCGT

ATGTTTCGAGGAAGTCGGACGCTCGGCGCTTGCTCTGGGCCATGTATCGG

GCGACGCCGCTCCGTTTCTCCCGTCCCGCCCGTCCCGACTCGGCTTCGCC

CCCAGCGGTCCCAGAACTGCCGGGGTCCGGCGAGCGGTTCGGTGCCGTCG

TGTCGTCGAGGGTCTCGACATCCTCGTGTTCGATGACTCGCCCGACGGAG

TTCCGCTGCGTGAACCCCGGAAGCGGGAGCGTCCCCGCGCCACCGATGTC

TTTCTCGGTCAGGGCACCGGTCGGACAGACCGTGGCACAGTGTCCGCAGG

AGACGCAGTCGGAATCCGCCATCGTCTCGGCGTCGGACTGGAAGCCGATG

CGGGTGTCCTCCCCGGAGCCCTCGATACGGAGGACGCCTTCGACCTGCAC

GTCGTTACAGCCCTCGACGCACCGGTTACAGAGGATACACTTGTTGCGGT

CGATTTGGATGAACGAGGAGCTGTCGTCGAGCGGTTCGTACTCGCTTCGG

TCGTCGAAGACGCCGTAGCGCGGGTGGTCGACCCCCTCGCTGACGGCCGT

CTCCTGGAGTTCACACCGACCGTTCCCGTTACAGGTCGTACAGCGTAGAT

TGTGGTTGGAGAGGACGAGGTCGAGGTTGACGCTCCGGGATTCCTCGGCG

GCGGACGTGTCGGTCTCGACCCTCAGCCCGTCGGTGGCCGGGAACGAACA

GGAGGGGACGACCCCGTGTTCGTCGGTCTCGACCATGCAGGTTCGACACT

CGCTCCGAGGGCCGATTTCGTCGCTCGCGTCGCCCCCGCGGTCGTAGTAG

CAGAGCGCCGGCACGTCGGCGTCCTCGTCGAGGCTGTCGGCGCCGGGGTC

CACGCTCACGACCTCGTCGTCGACGGCTTGCATGGCGTCGATGACGGTCG

ACCCCGGCGGGACGGTGACCTGTGTGCCGTCGACGCTGAGTGTCGTCGGG

TCCTCGCCGTCGGTTCCGACGGGCGGGTCGTTCGCGGTCCCGGTCTCGAA

CTCCGCCGTGACCGGCGTCTCGTGCTGCGGGTCGTCTATCTCCGGAACGC

CCGGGAGTGGGTCGTCAGTGCTCATAGTTTGTCAGAACAGGTGCCGCTCG

GACAGCGGCCGTCGGCGTGTGCTTCGAAACTCGATTCGAACTCGTCCATC

GCGGTCAGCACCGGACGCGGTGCATGAGCGCCAAGCAGGCAGTTGCTCGA

CCGGGACATGACCCGACCGAGTTCGCGTATCTTCGCTCGGTCGAACGAGC

CTCGGTAGACCGCTCGCAGCAGTTCGGTGAGTTGGACGGTTCCCTCCCGT

CCCGGTACGCACCGCCCGCTGTTGGCTTCCGAGGCAAAGCGCGCCCGCTC

TCCGGCAGTCGCGACCGCACACCGCTCGTCGTTCAACAGTTCGACGACGC

CTTCCGTTCCGAGGCCGGCCGCGGTCAGTTCGGCCGCGGTCGATTCGACG

TCCAACGTCCGGGTGAGACCGCCGAACACCCCGCCGACGCAGGCCATCTT

GAACGACCCCTCCAGTTCGACGGCGTTCCGAGCCGTCGAGAGGCGGGCGT

CCGAGCCGACCTCTATCGTCGCGGGCGCGGCTACGTCGCCGGTGACGGTC

AGGAGTCGCGTCGTCGATGCGGCGTCTCTATCGGTGCCCTCTGGGTCCGC

GAGCGCGAGCCGTACCCGGGCGAAGGTCCGCGGCGTGTGGATGACCGTCG

GGCGACCGTAGAGCCCGTACTCGGCCGGCGTCGGCGGCTGGAGACGGGGC

TCGGTTCTGTCCACCCCCTCTATCGCTTCCAGCGCCGCCGTCGGTTCGCC

GGCGCGGTACTCGTCGGGACCGGTGACGACGACCGGCACGACCGGGAGTT

CGTCCGCGGCGGCGTCGATAGCCTCCCGAAGATGTGCTTGCAGTTCGGTG

TCGGTCTCGGCGACGTGGATGACCGCCTCGGTCGTGCCGAGGTACTCGGC

GACGGCCGCCACGCCGTCGAGCACCGACATCGGCGACCCGGCGAGGAGCG

TTCGGTCCGCGCGCTGGCGGTCGTCCGCGTCGTTCGCGTTCACGACGACG

ACCGGGTCGCCGTCGGTTTCTCGCGCGCGTCGCCAAGCGTCTGCGACCGG

GTCGTCGGCGGCGGCGTCGCATCTACCTCGACCGACGAGCCCGGCGTCCG

CGACGGCGCTCGCGTCCCTATCGAGCGAGTGAAACTGATACGCTGTCGGG

TCCAGCGGGTCGAGCCACCCACAGGGGCCGAGGACGCGCCGCGTTCCCAC

CGCGAGCGGCCCGTCCGTCGGAACTGGCAGCGTCGTCCGCTCGGCGTCGT

GGTCGACGACTGCCGCAGCACCGTCCGTCGGTAGCTCCCCGTCTTCCATG

GCGGTGACGAGGTCTCGGACTCTCGATGTCTCCGCGGTCGGGAAGAACGC

GGTCCGCCCCGCGTCGGTCGCGAGAACCAACGGGTCGTACTCGCTGATTC

CGGTGGGTCCCGTTCGGAGGACCGTGACGGAGTCGGCGGTGCGACGTGCG

GCGCTCAACACACGCGCTCCGCGCTCCGTCCGCCCGGCTCCCGAGACCCG

AAGTACCGGCGACCGGACTGCGTTTCCTGTGCGACTCATTCTCACAGATG

AAGAGACACGGCGGGGTTAAAAAATCACCCCAACCCACTCGCGTCGGAGT

GCGATTTCGAGGCGTTCGGGAAGCCGAACACGAAGTCGGTGCGAGCGCGA

GTGGGCGGGAGACGGGTGACCGCTCCAGACGGGACAGCGAGCGGAGCAAA

GAGCTAAGGCGGCGACGCGTCGTCTACCGTGATGATGACGAATCCACGGT

TCGACGACGTGCGAGCGAAGGCGGCCGACGCGACTCGCGAAGACGACATC

CAGTCCGTGTACACCGGACTCGTCCACGACGACGGTCGACAGGAGTACTA

CTTCGCCAACGACACCGAAGAGGCGTCGGAGTTACGCGAGACGGCGGCCG

TCCAACTCGGAATGTTGGTTCGCGTCCTCGCGGACCGCTCCGAGAGCGAC

ATCGAGGAGATTACCGACCTCGCGGCCGAGCGAGCCGAGAATATGCGATT

GGAATAGCCGACGATTATAGCACCGACTGCTCGGAAACGCATTCTCGTCG

GGACGTGTCGGCTCGAAAAATACCGGGCGCGTCGAGGTCCGTCGAGCGCC

CGTCAGAGGTCAGCGGCGTGTCGCCTACTCGGTCACCGCGACGGCTTCGA

TTTCTATCGCGGCTCCTTTCGGCACGTTGCCGACTTCGACCGCGCTCCGA

GCCGGCGGGTCTTCGTCGAAGAACTCGCCGTACACCTCGTTGAACTCCTC

GAAGTCGTCGATGTCGTCGAGGAAGACTGTCGTTTTGAGCACGTCGTCCA

AGGACGCATCCTCGGCGGCGAGAATCGCCGCCACGTTTTCGAGACACTGC

TCCGTCTGGTCACCGACCGAGGCGTCGTCGAGTAGCTCACCGTCGGGCGT

CAGCGGGAGCTGTCCCGCGGTGAGAAGTAGTTCGCCGTTCGTCGTCGCTT

GGCTGTACGCGCCAACAGCCGCCGGAGCGTCCGAAGTGCTAATCGTGCGC

TTCATAGCCGGACCTTCCGGGCGGTTCCTATTAAATCCAGTTCGGGGACG

GAAGCCGCCACACGTCGTGTAAGAAACCGGAGCGTTTGTGGAATGTCTCG

GCGCACGCGCGAGTTGACGACGACCGTTCGGCGGCCGGGGAGTGGTGCGT

CACCGGACGGTCCGGCAGGGAGCGCCGAGCGTGGCCGGGACCGGCGACTG

CGATGTGGAACCGGGTCGTTCGGGTGTGTCCTCCAGAGAGTACGACGTGA

GAGAACACAGCTAGTAACCTATCTGGTAGTTTGAGAATGCGCTACGCCAC

TCTACCCAACCGTAACGTGTCAGATTATGTCAAAATAGAGCCCACCCGAG

CTATAAATAGTGACTGTTCGCAGATGTCGAGTGGAGTCCACACAATGTCT

ATGGACGCAGTCGTCTACAAAGGCGAACGAGAGGTCGCAGTCGAAGCGGT

CGAAGAGCCCCAGATTCAACACCCGAACGACGTCGTCATCGACATCACGA

CGACCTGTATCTGCGGGTCCGACCTGCACATGTACGAGGGGCGGACGGCC

GCGGAGCCGGGAATCGTGTTCGGCCACGAGAATATGGGTATCGTCGAAGA

GGTCGGCGATGCCGTCAGCAGTCTCGAAGTGGGCGACCGCGTCGTCGCGC

CGTTCAACGTCGCCTGTGGCTTCTGTGAGAACTGTGAGAACGGCTACACC

GGCTTCTGTACGAACGTGAATCCGGGCTTCGCCGGGGGAGCGTACGGCTA

CGTCGCCATGGGCCCCTATCAGGGAGGACAGGCCGAGAAGCTCCGCATCC

CGTACGCCGACTTCAACGCGCTCAAACTGCCGGACGGACGGGAACACGAG

GACTCGTTCGCGCTGCTCGCGGACATCTTCCCGACGGGCTGGCACGGCAC

GGAACTCGCCAACCTCGAATCCGGTGACTCCGTCGCCATCTACGGGGCGG

GTCCGGTCGGCCTGATGACCGCTTACAGCGCCAAACTCAAGGGCGCGGCC

GAGATTTACGTCGTCGACCGCGTTCCCAGTCGCCTCGCGCTCGCCGAGGA

ACACTGTGACGCCACGCCCATCAACTTCGAGGAGGGCGACCCCGTCGAAC

AGATCAAAGAGATTCACGGCGGCGGCGTCGACAAAGGCGTCGACGCGGTC

GGCTACCAGGCCATCGACCCGGAGAAGGAAGCCGACTCCGCGTACGACCC

CGCCCGGGAGAACCCGGCCGTCGTCATCAACAACCTCATTCGGACGGTCC

GACCGACCGGCGAACTCGGTATCCCCGGTCTCTACGTCCCCGACGACCCC

GGCGCGCCCGACGAGATGGCCGCGCAGGGTCGCCTCGGCATCGACTTCGG

CCTCCTCTTCGAGAAGGGGCAAGCCCTCGGCACCGGCCAGTGTAACGTCA

AGGAGTACAACCGACAGCTCCGCGACATGATAATCGAGGGTCGCGCCGAC

CCGAGTTGGGTCGTCTCCCACCGCGTCGGCCTCGAAGACGCGCCCGAGAT

GTACGAGAAGTTCGACAACCGCGAAGAGGGCGTCACGAAGGTCCTGCTGG

AACCCTAGGACTAGGGGCAGCGGAGTCTCCCGCTCCCAACTCGGTTCCCA

CCGATACTCCTTTGCCGGTTTGCCCGGTATCCCGTTCTATGTGTCAGTAC

TGCAGTTACCGGTACCACGACGGCTGGACGCAGTTGTTGGAGTACGACGA

GGTGTACCAGACGGTCATCGGCGGTGAGTCGGAGTCGACCTACGGCTTCC

ACGAATCGTGGGACGAACTCCGAGACGAGGTCGACCTCGGCGCGGCCTAT

TGACCACCTCCCGCGCCTAAAGTCGTCCCCAGAACGCACAGCGTTCTGGT

GTGCGAACGAGACGCAGAGCGTCTCGTCAACGCGGGAATCCCACCATGGG

ATTTCAGGCCGAGTGCGGCCCTAAGGTTTCAAGACGCATACGTTCCAAGC

GTCTCTTGCTGGGTGTCAGCATCGGCTTGGCTGTCTTGTGGGACGGTCAA

ACGTCCCCCTTCCTCAGCCGAGTCATCGCCATCCGTGTGTTCTCTTGAAT

GACTCTCTCCACTGAGGTAGCGGTCTGCAATATTGAGCGCGGCGTTAACG

TCTGCTTGGTACTCCGAAACCCAACACGCCGAGTTGGAACACTTGAACGT

CGCCTGCTTCGGACGGTAGCCCGCCTCTCCGCAACAGTGGCACGTTTTCG

AGGTGTACGCGGGATTCACTGTCTCCACACGAATCCCTTTCTCAGACGCC

TTGTAGCGAATCTGAGCGTGCATCTTGGCGAAGCCCCATCCGTGAAGCCG

TCGATTCATGAACGCGCCGTAGTCCATGTTCTCACGGATGTGCGTCAAGT

CTTCCAGAACCAACACGGGATTCTCGAATCGGTCGGCGTAGGAGACGACC

TCCGACGTGACCGTGTGAAGGATGTGGTCGATGTGTCGCCACAACTCGTC

GCCGTAGGATTCAGCGATACGTTCGCTTCCGCGTTTTTGAAGGCGTCGAG

TCGCCGTGAAGTACGTCTCACGGAGTTGCCGTACTCGCATGCCTTCGTCG

TTCCAGAGGTTGGGTGCAGTCGGGGAGCCGCGCTCGTCACGGTGACACAC

CGTTAACAAAGAGGCTTCCCCGATGTCTACCCCAATCGGCGTTTCCACCG

AACTGGTCTCTCGTTCCTCCACGTCCCGCGTGGCGACGATGTGAAAGTAC

CACTCGCCGTCACGGTCGAACAACCGACATTCGCCCATCTGTGCGTCTCC

TGCGTGCAACGCTTCCAACCATTCTCGCTGTTCGGGATTCGGTTGCGCGG

GCAACCAGAGGTTGTAGTCGTCGTGGTGCGGGATTTTGACGTACCACTCG

ATAGCGTTCTGTGGCTTGTGGTCGAGCTTCGGGCCTTCGTTCGTGAACCG

AACGGGGTGGTCGTCGTGAAGCTCTTTGGCGTCGTAGCTTCCGCCACAGA

GTTGTGGGACGTACTTCTTGAGCGCGTTCTTGGCGTAGCCGCTCAGGTCG

TAGTTGACTACCACGTCGTTCGCCGCCGACTGTGTGGTGCAGTTAGCGTT

GAACGCGGATTCAAGGGCGTCTTGGTACGCCTGTTCCGTCTCACAGAGTT

TCCGATGCTTGTGGGTGTTCGGTTCCACAAGCTTCAGTTCCAGCGTCTCG

GTGAGTTCGGTCACTGTTCGTCCTCCTCGTGACGCTGGATGTACTTCTCG

ACAGTCTCACTCGAAACGTGTCCTGCTGTCCCCGCGTAGTAGCCTCGCAC

CCACTTAATTTTCTCACTGTCGTGGTCGGCGTGTCGGTGGTTGTACTTCC

GCGAACTGATGCCTTTGAACCAGTTGGCGAGAAGTGACGGAGCGTGCTTC

GGCGGGCTACTGACGAACAGGTGGATGTGGTCGGGTTGGACAGTGAGGTC

GATAATCTCCAACCCCTTCTCGTCAGCGATTTCGTGGAGGATGTCTCGCA

CACGGGTTGCAACGTCACCGACGAGTACCGACCGACGGTACTTCGGTATC

CACACTATGTGGTAGTTGAGGTTGTACGTTGCGTGCCGTGTGGCCTTCAT

CCGTACTACACACTATACGTAGCGTGTGTCTTAAAACCACCTTACACGGT

GGGAAATCCAGCCGTACTACCGTCGGTGGTTGTGTACGCTATTGTCCGTT

TGACCCGCGCCTGAAGACGCGGGTATGCGCTCGCACTATGTATCAGGCGG

ACTCGCGGGGAAACGGGTCGTCCGGATACGCCGCCTCTCGCTCGTCCGTC

GTCGCCAAGCACTCGTCGAGTTCTCGGGTCACTGCGTCCTCGTCGAGGTC

GCGGCCGATGAACACGAGTCGCGTCTGGGGGGTATCGTCTCCCCACCGAC

CGATGGGACCGGCCTGCACCGACGGCCCCGCCTGACTCACGCCGAGAACC

GTCTCGGGACGACTCGCGACCCACGCGAACCCCTTCGCGCGGACGACGTT

CCCCCGCCAGTCGTCGAGCCACGCGTCGAGGCGCTGCGGGTGGAACGGAC

GGTCTCGACGGTAGACGAAGGACTCGACGCCGTGGGCCCTCGCTGCCGAG

ACGCCCTCGTCGTGGATATGGTCGTGGTCGTGGCCGTGGTCCGCCTCGAC

CCCGTCGCTCGCGAGGGCCTGCTTCCAACCCTGATGGCGCTTCGCCGCCT

CGAAATCGAAGCGCCCCGTGTCGAGAACCCGCGTCGGGTCGACGTCGCTG

TACGTCGTCCGGCGGATGCTCGCGCGCGGCTGGAGTTCCCGAATCTCGGC

TTCGACGGCGTCCAACGCGTCGTCGGGGACCATATCGCACCTGTTCAGCA

ACAGCACGTCGCAGAACTCGACTTGCTCGACGAGCACCTCCGTCAACGGG

CGCTCCGGGTCGGGGGCGGCGTCCGGAAGCGATTCCGCCGGGTCGAACGC

CTTCCAAAACCCGTAGGTGTCGATGACCGAGACCGTGGTGTCCAACCGGA

GGTGGTCCGGCAGACCACCCTCGTTCGACTCGCTCGCCAGCGTACGTGCA

ATCGGGAGCGGTTCGCTGATTCCCGAGGCCTCGATGAGCAGATAGTCGAA

CGAGCGGTTCTCCGCTAGCCGCGTCACTTCGGTCACGAGGTCGTCCTGAA

GCCGACAGCAGATACACCCGTTCGAGAGGTCGATGACCCCGTCGTCCGAT

TCGGACGCGACTAACTCCGCGTCGACGTTCAGTTCGCCCATGTCGTTGAC

GACGACGGCTATCTCCCTGCCACCGGGGTCGTTCAGGAGTCGGTTGACCA

GCGTCGTTTTCCCCGCGCCGAGCGGACCGCTGACGACCGTAATCGGTATT

TCCGTTGTGGACATATCGATGCTGTGTGACGCCTCGCGGGTGGTCGTAAT

GAAGACTGGGTCGGTCGACCCACACGGGAGACAGAACCCGCCGTGCTCCG

GACTCCCGCCGTGAAAACACAAAATATATTATATTTGTCATGTATGGTTT

CCCGTCTGTTCGCGTCGGGCACCGCGACCCACCGAGCGAACCGCAGAGTA

CGGCTTTGGCGGAGGCGTTCGCGCCGCCGCACTCGCTCGGTGGTCCGCGA

GAACGGCACATCTTTTTGGTCGCCGGCGCTGACGAACGACCAGATGGCAC

CTCCAATCGCTGACCGACTCCTCCGGGTCGACTTGTCCGCCGGTTCGGTC

GAGAGCACGCCGATTCCGGACGAGTGGCGACGGCGATACGTCGGCGGCAA

AGGGTTGGGTGCCCGCTATCTCTACGACGAACTCGACGCCGGCGTCGACC

CGCTCGGCGAGGAGAACGCGATGCTGTTCGTGCTCGGCCCGCTTTCGGGC

TACCTCCCGGGCGAGTCGAGATACGCCGCCATCACGAAATCCCCGCTGAC

GGGGTGTTTTCTCGACTCGTACGCGGGGGGCGAGTTCCCCGACTCGCTGG

CCGGCGCGCTCGGGTCGCACATGGGCCTACTCGTGACGGGTGTCGCGTCC

GAACCCGTTCGCCTCGTCGTCGAAGACGGGGACGCGACAATCGAACCCGC

CGAGACGTGGGGGGCCGGGACGGTCGACACGGCGCAGGCGTTCCCGAACG

CGGCTGTCGCGTGTATCGGCCCCGCGGGAGAACGACGGGTCGCATACGCC

ACCATCGCCTCCGACGCCGGCGACCACCACGCGGGCCGCGGGGGAGCGGG

AGCGGTGATGGGCTCGAAGCGACTGAAGGCCGTCGTCGCGCGGGGAGCCC

CGCCCGACGCCGACGGGTTGGACGACCTGCGAGACGCCTACGCGGCGAAG

TACCGAGAGACCGACACCGGGCGGTGGCTACGGGCCAGCGGAACCGTCGA

GACGATCGATTTCGCGAACGAGATCGGTGCACTCTCGACGCGAGGCTGGA

GCGACGGACAGTTCGAAGCGGCTGAGGAGTTGGGCATCACGACAGTCGAA

GACCGGGCCGTCGGCCGCGAACGCGCCGACACGGAGACTCCGGGCGGGTA

TCGTGTGGCCACGGAGGACGGCGACCACGTCCCGCGCGGTGCCACCGCGA

TGACGCTCGGAGCCGGACTCGACATCGACGACTTCGACGCCGTCGCAGTG

TTGGGACGGACCTGCGACCGCCTCGGGATGGACCTCATTAGCGCCGGAAG

CGCGGTCGCGTGGACGGTCAAGGCCAACGCCGCCGGCGTGCTCGACCGCT

CGCTCTCGTTCGGTGAGCCCGACGGCGCGCGGGCCCTCTTACAGGAGATT

GCGGAGCGCGAGTCGGAGCTCGGTGACGCGCTCGCGGACGGGGTCGAGGC

GGCGGCGACGCGGTTCGGCGGGCGCGACCTCGTCCCCACGGTGAAGTCGA

TGGAGCTACCGGCATACGACCCGCGCGGCGCGCAGAGCATGGCACTCGCG

TACGCGACCAGCGACCGGGGTGCGTGCCATCGGCGGGCGCGACCCATCGA

ACGCGAGGTGTTCGACGGCGACTGGGGAGCCGACCGGACCGCGGCGGAGG

TCATCCGCGAACAGGACCGGCGGTCGACGCTCTGGAGCCTCATCGCGGAT

GACTTTTTCGGCGACGCGCTCGACGACCTCGGCCGGGAGTGGCTCGAATC

GGTCGGCCTCGACCCCGCCGGCGGACTCGCCACAGTCGGCGAGCGGATTT

GGAACGTGACGCGGCTGTTCAACGTCCGCGAAGGCATCTCGCGCGAGGAC

GATTCCCTCCCGGCCGCGCTCCAAGAGCCGCTCGAATCGGGGCCCCGGGC

CGGTGCGACCGTCGACCGCGACGACTTCGACTCGATGCTCGACGCGTACT

ACCGCCGCCGCGGCTGGACCGTCGACGGCGTGCCGACGGCGCAGACTATC

GACCGCCTCGGACTTGCTGCCCTCACCGATGACTTCGACACCCTCGATGA

CTGAACACCGCACGAACGGCTGGACGACGAACCGCGCGCGACCCGGAGAA

CGAACATGACGCTCGCTGATGCGCTCTGCGCTCGACGCGGCGTGGTCTGC

GTCGTCGGCGCGGGCGGAAAAAGTCCACGCTGTACGCGCTGGCGCGTCGG

CTCGGCCGCGCCGTCGTGACGGCGACGGTCCGCATCCCCATCTTCGACCA

GCACGTCGCCGACGTGGTCGTAACCGACAGGCCGGTCGCCGCGCTCGAAA

AGGCGACTGACTGGCCGATGGGTGTCGTCCCCGACCGTGACCGCGACGAC

CGCTACCGCGGCTACGACCCCGACGTGGTCGACGCCATCGGGGAGTCGGG

CGTCGCCGACGCGGTGCTGGTGAAGGCCGACGGCGCTCGGATGCGCGAGT

TCAAGGCCCCGGGCGACCGCGAACCACAACTCCCGGCGACCGCCGACACC

GTCCTCCCCATCGCGAGCGTCCACGCCGTCGGTGAACCGCTCACCGAAGA

CTGCGTCCACCGACCGGAACGGGTCGCCGCCCTCACCGACCTCGAAGTCG

GTGATACGATTCGTCCGTCGGGTATCGCAACCGTGCTCATCAGCGGGCGC

GGCGGCCGCGCGGACGTTCCCGACGGTGCGACAGTCGTGCCCGTGCTGAA

CAAGGTCGACGATGCGACCCTCGAAGCTGTCGCACGGGAGATTGCATCGG

ACATCCTCGCTCGGTCGAACATTCCACACGTCGTACTGACGCAGTTGACG

GCGTCCGAACCCGTCGTGGCCGTCGTGGAGCGCTAGCGCAACTGTTCCGC

CGCGTGAGCGAACTCGTCGGGGGTGTTGATGTTCTCGAAGGTGTCGAGAC

TCGCGTACTCGCGCACTTCGCTCTCTTCGAGGACGACGTAGTCGAGCGTA

AACAGCGGGTCGATGATTTTGTGGCTCCCCTCGGCCAGCGCGGCTTCGCA

CGCGTCAGCCATCGACGAGGCCCGGTACACCGCGTGCGTCGGCTGGAACC

AGCCGTCACCGACCCGCGGCACGGCGGCGTCGTGGCCGGCCGCCCGCTCG

AACAATCTGTCGATGAGCGCGGGTTCGAGAAAGGGCATGTCGCAGGCGGC

GACGAACGCGTACTCCGTCTCGACGGCCCGCAGACCCGTCATAATTCCCG

CCATCGGGCCCTCGTCGCGCTCCGAATCTTCCGCAATCGTTATCGGGTGG

TCGTAGCCGTCGAGCGCGGTCCGGAGCGCGGCAGTCTGTTCCGGTCGGCA

GTTGACGACGAGGCTGTCGACGACGCCGGCCATCCGGTCGGCGACGCGGC

GGATGAGAGGTGTTCCAAGGAGGGATGCGACTGCTTTGTCCTCCTCACCG

AATCGCGTCGAGTATCCGCCGGCAATAATGACACCAGTTCGCATACCGGC

AGTGTTCACGTTCGTCGCGCAAAGCAGTTGTGCTACGAGTCGTATAGTCC

GTCATCGTTTCGCGTCGGCTCGACTATCGCACCGCGCGGTATCTTCGGTC

TACCGTCGCTCGAATTTCGGGAGGTCGACGCTTTTGAGGGTCAGGGCTTC

GATTTCGGGACGTGAGAGTCGGTGAAAACGCACACATTACACAGCTAGCC

GTGTTATTCAGATTTTCAATGTATTTAAAGCTAACGGGGATTGGAACTGA

CTGGCGGTGATAACTGTTGAATCGTGATTGTGCACTCGATGCTCGCTGCC

GCAGTAGAGGAGTGTTGACCTCTCGTACGACGTTCTTCACTACTCAGTCG

CCGCTCGTGCTATCACGCCGGTTCGTCAGCCGGAGACGAAGCAATAGATA

TTGCGCCATACAGTTTATGAAATTGACGACTGTCGGTTTGCAGTACGAGA

CAAACCCCCCGTCAGCACCGAGTCACCACAGTCGTGAGGGCCGAACCGGT

CCAGTTAGAATAATCTGCTGCGTCACGTTTCGACAGTGAGTTGCAGCACT

CTGGCGGTGTACCGGGAAAGTTCATCGACCTCAGTCCATCTCTCATCCGC

GGGTATTCATGCGGCTCGTACCCTGGAGTCGAAGCACCCAAAGCGGCCCT

CTGCCGACCAATCCGAGACTGAATACACGATTTTCGAGACAATCAGCGGT

AGTGAAATGTATTTGAGGAGAATACGAGTGTCGTTGTCTGCTATGGACAA

TGAGTTTTAGTAAACAGCTGATGGAAGCGCGTACAGGCTAAATGAACTGT

ATATCGCTATATTAAATTTGGAGAGTCGGTCGGAACACGGTTCGACGGAA

CTGTCGTTTTCGCGTTGTTCCCCGGTGGTGGCTCGACCGGGCGCGTTACT

GATGGAGAAAACGGAGTAACGACCGCCAGACCGTCGAATCGAGGATTCGA

TTAGCGCACTGAGCTCACCCGACGGTCGCTGTGCAGCGACGTATCGGTTC

AGTACTTCGCTCCGCTGGCGAGCACCGCTCGGAGACAGCAGTACTTAAAC

ACCCATTGCAGATAGCATAACCGGGAAGTCAGTAAAGAGACTACTAACAA

TTGATGTCAGAAGAATTCGGTTCGGAACTGGACGGAAAGGTCGCAATCGT

CACCGGCGCGTCGTCGGGCATCGGCAGCGCGACGGCCAAGTCCCTCGCCT

CTCGCGGGGCCAGCGTCGTCGTCGCCGCCCGCCGCGAAGGCGAACTCGAA

GAACTCGCCGCAACTATCGAAGACGACGGCGGCGACGCCCTCGTCGTCCC

GACGGACGTCACCGTCGACGACGACATCGACGCCCTCGTCGAGGCGACGC

TCGACGAGCACGGTCGCATCGACATTCTCGTCAACAACGCCGGCCTCATG

CCGCTTGCACACATCGGCGAGGCGGACCGCGAGACGCTCCAGACGACCAT

CGACGTGAACCTTACCGGTCTCATCACGCTCACCCACGCGGTCGTCCCGA

CGATGATGGAACAGGAAAGCGGCCACATCGTCAACCTCTCGTCGGTCGTC

GGCCGGTTCCTCCAGGCGAACAGTTCGCACTACAACGCCGCGAAAGCGGG

CGTCAAGATGTTCAGCGACTCGCTGCGACTCGACGTCGCCGAAGCGGGCA

TCCACGTCTCGTCAATCGAACCCGGCGCGGTCGACACGGAACTGCTCGAC

CACATCCCCGACGAGGAAGTCCAAAAGAACGTCAAGGACTACGTCGGCAC

GATGGACGCGCTCGCGCCCGAAGACATCGCGCGGACGATTACGTTCGTCG

TCACGCAACCGGAGCGCGTCGACATCAACGAAGTCCTCATCCGCCCCCTC

GACCAGGTCCAGCCCTGAGCCGCGTCGAACGACCAACACCTCACCTCTCG

TTCGGCGTCTCGATACCGCGGTGACGTTTCGATACCGCGGTGACGGCGCG

ACGAGACGCGACGGTCCGAACCATGTTATCGGGTGCGTGTAGAGTCGTCG

TTCGGTCCGAAGGCGACGTTCGGACGGCACGCTCGGGATACTGAGCCGCC

GCGTGCGTGAGCGTCGTAGGAGCCGTCCGACGAAGAATACTGTGAGCGTG

TCATAGAATCCATCTCATAGTATCTCACAGCCCGGTTCGTTTCCTCGCTC

GTAGTTGGTGATGGCAATGACGAGCCGGAGACACAGCGCAAGGAACACTT

CTGTTCGTGCGTGGACGCGGCCTCGGGCGCGGACGTGCCCGAGGCCGCAG

TCCTTGACCGCGTCGTTGGTTCGCTCGACGCCTGTCCGGTTGTTGTACGT

CTCCTCCAAAATGGATTGTTTCAGCTGCACGTCCTCGCTGTGTTCGGTGA

TGCGGTTTTCCACCCGGTACTTAATATCGAGTGGATCATCGGTGTTTCGC

GGGTTGTACGGCGCGATTGGCACAACCCCTGCGGCCAGCAGGTGGTCGTG

CCAATCGAGGAGATCGTACGCGCTGTCTCCAAGCATCCAGACCGGTGTCT

TGACGGCGAGCGCGTCACGCGTGACGCGCATCGCCGTCTCTTGATCCGCT

TGTTTGCTCTGTGTGAACTCCGCCGCAATCGGGATCTTTGAACCGGTCGA

GACGATTGTACAGCCGAAGCCGTAGTAGTATTCCTCAGCTGTTGGATCGT

AGTTCCACGAGGCGGCGTCGTTGTACTGGATCGCCTCAACGTGGGTCGAA

TCGATGGAGTACGTCGAGTCGAGCAGGCCTGCTCGACGAGCCTGTCGAAG

ACATCGTCGATAACGTGTTCGAGGTCGGTGAGAAACCGGTCAACTGTGTC

TCTGGATGGCGGTTTGTCGAGGCCACAGTAGTACCAGACAAGGCCGTTGT

GAAGTTCTCGTGTAACCGGCCGCGTGCCGTAGACATCCTCGTAGTAACAG

TGGAGAAAGCCGCCCAAGAGATCTGGAGGCTGGTGAACTCGTGTTCGCCC

CCTCGAAGCGGGGGCGAACACGTCGTACTCCAAGAGAAATTCGAACTCAA

GGTGTTCGAACAGCGGTACTGTCTCAGTAGCCGCCGCATTCAAGAAGTCG

TCTACCGAAGCTACGTCTTGCAGGGTTGTGCTGGTGTTGGACACACTTCT

CGCACCCTGCTGCTTCGTACGTGACGCTTTCTATGACACGCTCTACTGTT

ACTAAAACGGATACCGGGCGGTCACGCGCCGAAGGGTAATGATAGCATTA

CTGGCAATGACAACCGACATGGATTCTGTGACTTCAACCAAGAAAGATTA

CACGGATAAGTCTGTAATTTATTGGTCGGAACGACTCGGGGAAGCTGTTT

CGCGGGCGAGAGCGAATCATCTCGAATCGGCGTAGGACACCGCCCGTGAG

TCGTCTATCGGACGTTGAGTCGGCGTGAATCGAGAGTAAGGAGAGTATTA

CTAACCGGCCACGAACGCAGGTGTGGGACATTCGTTGGTCGGTGTCGTCA

CCGCCGCGCTGGGCGGTCGACCGACACGACACGGAGCCGCTGTTCCCGTT

CGGTCACGGCCTGTCGTATGCGACCATCGAGTACGGCGACGTGACCGTGT

CCGAGCGCGAGACCGGTGACGGCTTCGAGGTCGCCGTCGACCTCCGCAAC

GCGAGCGACCGGACCGGAACGGAGGTCGTACAGGTTTTTCGAATCGGTGG

GAACGCCGGGCCACGACGCTCGAGGCGGAACCAACGCCGAAAAACGCGCC

GCCGATGGCCGGTTCGCACCGGCCGAGACGGTCGTCTTCGGGAGCGTCGC

CCAAACGGGGGTCCGGAGGCACCCCGCCAGGTACGTTCGTTCGTGACGCC

CGAGATGAGCGAAACGCTAACCGGATTCGGAAGTATTTATTATCCAGAAT

CAGAACTCGGTAAAGAGGTCAGGTAGCACACGGCGCAGAACGGACCAGAC

CATGATATTCGAAAACACATCCACAGTCGGTAGTATCGAGTGGCGTTTCA

CCCCGTCTCGGTATCGAGATGCGTCCCACCGGTATGGAGGGTTCAATGAG

TAGCTCAGTGTCCGAATCTGACTCTCCACTCAAGGTCGCAACGTTACTGT

TTGCGACGACCTTGGTTGCGCTCTCGGGCGCAATCGTCAATCCCGTGTTG

CCGGCAATCGAAAGCGCGTTCCCGTCGGTTCCGAACGCGGGGACTTTGGC

GCAGCTCGTCAGCACGCTGACTGGGCTAATCATCGCTATCTTCGCCCCGA

TAATCGGCGTGATAGTCGACCGATACGGTCGGAAAGCGGTGTTGGTCGGG

TCGCTGGCGCTGTACGGAGTCGGACCATCACTGGCGTACGTCGCCGATTC

GCTCTACGTAATTCTCGGGACGCGAGTCCTGCTCGGCATCGCCGTCGCCG

GCATCATGGTGAGCAGTACGACGTTGATCGCTGACTACTACTCGGGCAAG

CGCCGGGAGAAGGTGCTGGGCTGGCAGGGAGCCATCATGCCGTTCGGTGC

AGCGGTCGCGGTGATCGCCGGCGGGGTCATCGCTGACCTCAACTGGCGGA

CTGCGTTCCTCACGTATCTCGTCGCCCTGCTCGTGCTCCCGGCGGTAGTC

CGGTATATCGACGAACCGGACCGAGGAGATAAACAGCAGGCGGGGTCGAT

TCCAACGTGGACGGAGCTCCGAGAAATCCTGTCGACGCTCCCGCTCGCGT

TCCTCGCGGCCCTCTATCTGATCATGTTTGCGGGGATGATCGGCTACAAT

CAGATTAACGTCGAGATTCCCTTCTATCTGCGGACGGTGACATCCGTCGG

CGGACTGATGACCGGGGTCGCGCTCGCGGCGATGATGATTATCTCGGGGG

TCGTCACGATGAACTTCGACCAAATCCGCGAGCGGTTCGACCCGGTCGTG

ATTCTGGTCGGCGTCTTCGTCGCGACGGGCGTCGGGTTCACCGTCACGAG

CCTGACCACCAACTACTGGGCTATCGTCGTCGGAATCGTCATCGCGGGAA

CGGGACTCGGCCTGCTCCTCCCGACGACGAACTACTGGGTGTCCGCGCGG

GTCGACGAGCAGTATCGCGGGCGGGCGCTCAGCGGCGTGACGACGACGCA

GTTCCTCGGGATGTTTATCTCCCCGATAGCCGTCGCGCCGCTCATCGAGA

TGTTCGGAACCGGCCGAACCTTCCTCGTGCTCGGCGCGGTCGGGCTCGTG

CTTGCCGGCGTCTTCGGCGCGATTGCCGTGCGCAACCAGTCTTCGCTGAC

CGAAGCGACGCCGGCCGGCTCGGACGGTTAGTCGCACGAGAGCGACGAGC

GGGCGCGGATTGCTCCCGGCCGGGCGATAGCTGAGCGCAGTTGGCGCGGC

CGCGACGGGACCGACCCGCTTGGACCCGTCCCGACCCGACCCGGCCCGAC

CCGACAGCGGTTCGGTCGGCCCGCTCCCTCGTTGGATGGTGTCCGCACAG

CAACGTCGAGGGCGGACCGCCCGCGGAGAGGCGAGAGCGGTTCGTAGCGG

TCTGCCGGAATGCAACAGAGAAAACCCGCCGCCGCGGAGATTACGGCTGG

GCTGCATCGTTAGCTATTCGGGAGTAATATTGTAAATAAGATTGATTTCT

ATTGGTCGGCCGCGTAGCTGTCAGTCCGCGAACGGTGGAGACGCTCCGCT

CGTGGTTTTCGGGCGTGAAACTGCGCTCGACAGGATTCTATCGATACGAT

CAGCTCGAAAACGGGGAGTAATCGAACGCATGCAGCCGAATGGGTCTGCA

ACTGTCGGAGTTCGGGTTCGCTGAAGACCGCGTGTCTCCGAGAGTCGGAA

AGATGAACCGGCCAGCGTCGTCTCTTGCCGTCGAATCGATTGGCCGTCGA

AGACGAGTTGAACCTAACTAATATTGTCCTTGATAAGCAAGTCACTATGA

TGAGTCGACGAGGGGTTCGTCGACTGTACTCTCTCCGTCGGCTTCGCTTT

CGACGGGGTGTATCAGATGGGATAATTCGACGGTGAAGAACGGAACCGTG

AGAAAAACGCGTTCGGCCGACGAGTACCTGCGGCGACGCGGCCGACGACA

CGGTGAAGATGTACTTGTCCTACAATACCGAGTTCGAGAACGGATACGGT

CGGTGACCGGCGACGACGTGCCGGACCGACCGTGTCAGTCGCCCGAGACC

CTTTCATCGAACTGGTCGATGGGAGTCGGAACTCCCGCCCGACTCAACGC

TCGGGGAGGTAGATAATCTGCCGACACAATGCGCTAACGGGCCAGCATGA

GACGGCCAGTGAGCCAAGAGGATTCCGACCTCGCTGACGACGGCTGCCGG

CAGTGAGGTCAATTATCACCTGCACGCGGAATCCGCGCTCAACCGCCAAC

ATCTGGCCGCGGTAGTGAATCGCCATTCGAACAACACTCCGTGACGCGTC

GGCGCGACGGCGAGCGACGCGGGAGCCGAGACGGACCCGGTGGCTCGGCG

GTCACCGACGAGGGAGAGACAGAAGCGCTGTCCGCTGTCCGAGGCTTTTT

TTCGCCGTGGGGAGATGTTCGGGCATGGATGCAGACCCGCGCGAAGCAGT

CGCAATCGACGCCGCGGAAGTGGGAGCCGACCACGCGTCGGCGCGTTTCA

GAACCGAACTGGACATCGAACAGAAAGGCGACGCAATCGACCTCGTGACC

GAGGTCGACCGCGAGACACAACGGCGCGTCATCGCGGCGATTCGAGAGCG

GTTCCCCGACGACGCGGTCGTCGGCGAGGAGGACGACGAACTGAAGACCG

TCCCCGAGTCGGGCTACGCGTGGATTATCGACCCGATAGACGGGACGCAG

AACTACACGCGGGGGGCCCGCGAGTGGGTGACGAGCGTCGCCGTCGTCGA

GAACCAGACGCCGATTGCGGCGGTGAACGTCTCGCCCGAGACGGGAGACA

CCTACGTCGCGACGGCGGCGCGCGTCGAGCGGAACGGCCGACCCATGACG

GTCAGCGACGAATCGAACACCAGCGCCTTTCTCGTCTCCTCGACGCTCCG

GTATCAGGTGGGCGACCGGCCGGGAATCGAACGGCTCTCGGGCGATATCT

TCGGGACGTTCGGTGAGATGCGAAAGCTCGGGACGACGCAGCTGACGCTG

TCGCGACTCGCCGACGGGTCGATAGACGCGGTCATCGGATTCGACGAACA

CCCGAACGCGTGGGACACGGTCGCGGGGGTCTACCTCGTCGAGCGGGCGG

GCGGAACGGTGACCGACATCCACGGGAACGCCTGGGGACCGGGACAGCCC

GGTCTCGTCGCGTCCAACGGGCACGCACACGACGAGGTGCTCGAAGCGGC

GCAGGCGGCGTTCGAGGAGACCCGATAACGGCGCAAACGGCATCACGCGG

CATCGCGCCGACCCAAACCGAGTCCGTGGCGACGTGGGCGTGACCGCGAC

CTCGACCCTGGGCCCCGACCGACTGAATTACACGGTTACGCCTGTAATGG

AGATGCGGACGAGAGACCGACGACGACCGGGAACCGTCAGGCGACCGGGA

AGGTATATCAACTCAAATATAGTTCTGAGCGCGGGAAGCCGCACAGGGGA

CCGACCTCGGACGGACGGCGCGGCCGCAGCGCGCCGACGGATGAGACGGC

AAACGAGCCGGGTTGTTCACCCGCCGACGGATCTACTTAAACGTTCACGC

GTCCGAAAACCGAGCCGAGATCGGTCGTCTCAGCCGCTCAGTCGCCCGTG

AAACCGACTCTCACGCGTCTCGGAAGACTCGGCGTTCCTAACTTCCGAAT

ACGTATATATGAACGAGCCGGTCACCCCGCAGCGGCCACGATGATTCCGA

CCCGCACAGGTCGTAATAATCATAGATAACCATGTTAACTATTGACAATC

AAAGCGCGAGCACATAAACGTTCGACTCCGCGGAACGCTCGGAGCACCGA

CCAGTCGGGGAGTGTAACGGGTACGCGAGCGTGCAGCGCCGGTGTCGCGG

CCGTCGCGAGGGCGACAGTAGTCGTGACGGAGGTACATCTTTAAGCCCAG

ACCGTAGATTCCCCTCCATGAATAGACACGACCCGTCCGGCGTGCTCAAG

ACGACAGCCGCCTCCTTGGCGCTCGTCGACCACATCCTCGAACTGGAGGG

GGCCACGATGGGCGAACTGGTCGAGGCGACCGACCTCGCGAAGAGCACCG

TCCACGCTCACCTCAAGACGCTCGCGGAGTACGGCTACGTCGTGAACGTC

GACAACGAGTACCACCTCGGGGCGAAGTTCTGCCACCTCGGAGACTACGT

CAGGACGCGCAAGGACTACTACCGCGTCGCCCAAGAGACCGTCTCGTGGC

TCGACAGCGAGTCGTCGATGGACGCGGACTTCGCGGTCGAAGAACACGGT

CGCATCGTCTCGCTGTACGGCGACCTCGAATTCGCCAACACGCCGCGCTT

TCTCATCGACGGCAGCCCGTTTCACGTCCATACGACGTGTTCGGGCAAGG

CGATTATCGCGGAGTACCCGGAGACGCGAGTGCGGGAAATCATCGACCGC

TGGGGGCTGCCGGCGGCGACGGACGACTCCATCACGACCGAAGACGAACT

GTTCGCGGAGTTGGCGACCGTCCGCGAGCAGGGCTACGCCGAAAACAGCG

GCGAGGCGGTCGAGGGGTTTTGGGCCATCGGCAAAGCCGTGAAGTCGCCG

CGAGGCGAGGTGTACGGGTCGCTGAATCTGAGCGGTCCCGCCTACGTCAT

CGACGAGGAGACGCGGGCGACGCAGGTCGAACTGCTCGAACGCGCAGCCG

AGCGGTTCGAACAGGGCGTCGCGGAACTGTATCAGACCCAAGCGAACGAG

CCGAACGAAGAGTGAAAACGAGGGCGAGAGGGTCGGTGAAGCCGTCGGGC

GAAGGGGAATGCAGACGCGTTACGCCGGGACGTTTCCGGACGGGACGACG

CCGTCGTCGGTCCAGCCGCGACGCTCGTAGTACGCGTCGAGCGCCGCCTC

GAACCCGTCTAGCTCGTCGGCGTACGGGAGCGTGTCGTCGCCGCGGTCGA

AGCCGCGCTGGTTGTTGAAGTGGCGTTCGAGCGTGACGATGCGGTCGCCG

ACGGCCAGCAGGTCCTCGAAATCGGCTCCGAACAGCATCTCGTAGCGTTC

GGGCGTCATGAAGTCCCGCGAGAACTTGCAGACGATGCCGCTGTCGTTGA

GCGCCATCTGGTTTTCCTTCTCGATGAGGCGCTTGGGTTTGCCCTCGAAC

CCCTCCGGCGGGTAGGCGTCGTCCTTGCCGACCAGCGGGTACTCCTGCGA

GTAGAACACCGCGTACATGTGGTCCGCGCCGCGGTTGGCGACGGCGTACG

ACAGTCCCTGTCCGTGGAGGACGCGGCCCTCGTGGGCGGCGAAGTCCATC

CCCTTGACCGTCCAGTTCTCCACGCCGAGGTCGTCGTGCACGCGGGCGAT

GCCCTCGGCGAGGTCGTCGCCGACGCCCTCACGGAGCGCGATTTTCTCCA

CGAGGTCGTGGATGAGGTCGGTGTTGCCGAACTCGTCTTCGCTGGCGAGG

TAGGCCGCGACGGTGTTGCCGGCCGAGATGGCGTCGAGACCGTAGCGGTC

GCACAGCTCGTTCGACTTCATCACTTCCACGATGTCGTCGACGCCGGAGT

TCGACCCGAACGCCATGGTGACTTCGAACTCGGGCCCCTCGGTCTCGACG

CCCGCCGCCTCGTCTCTCGTGGGGAGCTTACACGCGAACGCGCAGGCCGA

GCAGGTTCCCTTCTTGTACTTCTTCTGCTCGATGGCGTCGCCGTTGATGC

CCTCCGCGCCCTCGAAGTGACGCTCGGAGAAGTAGTACGACGGGAGGCCG

TCCATCTCGTTGGCGAGGTCCAGCACGGCGACGGTCCCCTGCCGCTTCAT

GATGTGGTCGTCGGTGGCGGCCTCGCGGTGAATCTCCATCTGCGACGCCG

GAATCTCGATGTCGGGCGCGGCATCGCCGCCGAACGTGAGCGCCTTCACG

TTCTTCGACCCGAGGACGGCACCGAGGCCGCCGCGGCCGAACGCGCGCTC

CTCGCTCGTCATGATGGAGGCGAAGCGCACGAGGTTCTCGCCGGCGGGGC

CGACGACCATCGTCCGGTCGGCTTCGATATCGTGTTCGTCGTCGAGGTAC

GCGACCGTCTCGGGGACCGTCGCGCCGGCGAGGTCCGGGACGGCCTCGAA

CTCGACGCCCTCGTCTGTGACGTGGAGGACGACCAGTTCGTCGCTCACGC

CCGCGAGTTCGACCGCCGAGTAGCCCGCGTCAGCGAAGTTCCGCGAGACG

AACCCGCCGGCGTTCGACGAGCAGAGGCCGCCGGTCAAAGGCGAGACGCT

GGTGCAGTTCGTGCGGCCGGTGAAGCTCATGTTCGACGCCTGCATCGGGC

CGGTACTGAAGTACACCCGGTTGTCCGGGCTGAACGGGTCCACGTCGAAG

GGAACGCGCTCGTGGGCGAGTCGAGTGGCGACCCCGCGCCCGCCGACGTA

GGATTCGAGAATCCCGTCGATGTCTTCCGTCCGACTCGTCTCGTCCCCCA

GGTCGACCGTGAGAAGCGGACCTTTTGCGTGTAACATTGGAAAAAGAAGG

AGTTGCCTCGCACAAATCCATTGTGGTTACTGTTGTGTCGCTAACCAAAT

CACCGCGGGAGGTCGACTTCGAAACCGGATACTGTGTACGTTATCAATAG

GAATCGTGGACAAATTATTTATGACTGTTCTGCATGGGTTGAGGCATGGT

CGCAGACCCGCCGATTCACGAGTTGCACTTCGACGACGCGCCGAGCGTGG

ACGACGTTCCGGGGCCGAAGTCGACGCGACTCCTCGAAAAGCAACAGCGA

ATCGACAGCAGCGCCGTCTCGTACCCCGAAGACATCCCCATCGCGTTCGA

CAGCGGGAAGGGCGCGACGGTGCGCGACGCCGACGGCAATACCTTCATCG

ACATGTTCGCCGGTATCGGCGTGCTGAACGTCGGACACTCGAACCCGTAC

GTCCTCGAGGCGGTCCACGAGCAGACGGACAAGTTCGTCCACACCGTCGA

CTTCCCGACCGAGGCGCGGCTCGACCTCATCGAGAAGCTGGACGAAATCG

CGCCCGCGGGACTGCGCGGGAACAACCGCGTCGTCTTCGGCGGTCCGACG

GGGAGCGACGCCATCGAGGCGTCCATCAAACTCGCCAAGTACAACACCGA

AGGGACCGGTCTCGTCGCCTTCCGCGGCGCGTACCACGGCGCGACGAGCG

GCGCGATGAGCCTCACCGGCAACAAGAAGTTCAAGGGCGACTACTCGCCG

CTCCTCCCCGACGTGGTCCACGCGCCCTACCCGAACACGGTCGAGATGGG

TAAAGGCCCGCAGGAGGCGGTCGACCACTGTCTCGAAGAGGTCAAGGCCA

TCTTCGAGGACCCCTACGGCGGCCTCGCCAACCCGGCGGGCATCTTCGTC

GAACCGATTCAGGGCGAAGGCGGCGTCGTCACCCCGCCGAAGGGATTCCT

GAAGGGTCTCCGCGACATCGCCGACGACAACGACGTGCCGCTCGTGTTCG

ACGAGATTCAGAGCGGTCTCGGTCGCTCCGGCAAGTGGTGGGCGAGCGAG

TGGTACGGCGTCACGCCGGACGTGATGACCTCGGCGAAGGCGCTCGGCGG

GACCGGCTTCCCGCTGTCGGCGACCATCTACCACGAGGACCTCGACACGT

GGGGGTCGGGCGACCACGCGGGCACGTACCGCGGCCACGTCGTCGGGATG

CGCGCCGGCACCCGCGCCATCGAGTACATCCAGGAACACGACCTCCTCGC

GCACGCCCGCGACCTCGGCCAGTACATCCGCGGCCGGCTCTCGGAGGTCG

CGGAGGACAACCCCCGAATCGTGGACGTTCGCGGGAAGGGCCTGTTCATC

GGCGCGGAGTTCGTCGACGCCGAGGGCAACCCCGACGGCGAGGCGGCCGA

CGCGCTCCAGCAGTACTGCTTCGAGCGCGGCGTACTGGTGTGGAAGGCCG

GCCGGCACGGCAACATCCTGCGACTGCTCCCGCCGCTCGTGCTCACCCAC

GACCTCGCGGAGACGGCGCTCGACGTCATCACCGACGGCATCGAAGCGGT

CACGGCCGAAACGCAACGAGTCTAACCACGGTCGAACACCACGATGCCAC

CCGAACGAGTCATCACGGACGACGCCCCGCGTACGGATAACCCCTACTCG

CAGGGCGTCGTCGCCGGCGACACGCTCTACGTCTCGGGCTACGGCCCGGT

CGACCCCGAGACGGGTGAGGAGATCGACGGCGACATCGAAGCACAGACCG

ACCGCGTGCTCGACAACATCGCGGCCGTCGTCTCCGAGGCCGGCGGCGAC

GGCCTCGACGACGCGGTGAAACTGACGGTGTACGTCACCGACCTCGACGA

CTACGAACGGGTGAACGAGGCGTACGGCGCGCGGTTCGACGAGGTGCCGC

CGGCCCGCGTCTGCGTCGAGGTGGCGCGACTCCCCGGCGACGTGCGAGTC

GAGATGGACGCGATAGCGTATCTCGGATAGGTCGCGGATGCGGGGACGGG

GCGCGGGGACGCACCCGCGCGACCGCCGAGGTTCCGGGGACCGGCGACCC

GCCGGTCAGTCGTTCAGGTCGTCGACTCGGCGGTCGCGCCGCTCGTTCGC

GGCGTCGATTCGCTCGACGAACTCGGACATCTGCGCTTTCATGTCGGCGC

GCAGGTCGGTCGCAGAGAAGTCGTCGGACTCCGCGAGGAGCCTGACGAAC

GCCTGTAACTCCTCGTCTGTGAGCTGGTCGAACGCGCCGCGTTCGAGCTT

GTCGATGACGGCGTCGACGTCGATTTGGCCGACCGGTTCGACGTTGAAAT

CGACCGTGACCATCCGGTAGTCGGAGCCGGCCAGTTCCTGTTCGGCCTTG

TTGACGCCCTCGGCGAGCATGTCCTTGAACGGGGTGTAGTAGCCCATCGT

CCCGAGGTGGAGGAAGGCGAGCATGTCGGTGATGCCCCGCGTGTACGCCT

CGCGGTCGGCGGCGTCGGGGTTGAACACCGTCTCGCGGTCGCGGTCTTCG

AGCGACTCGAACAGGATGGTGAAATCGAGGATGGCGTTGCGGAGGCGTCG

TCGAATCCGGTTTCGCTTCTGTTTTTTCGAGTGGTCGGTGTAGTCGGTCT

TCCGGCCGAGCAGGAACTCGCGGTCGCTCGGCGTGAGGATGCCGCGGCCG

CGGTCGGCGGCGTAGGCGAGGTCGTCGGTGCCGTCCGTTCCGTCGGCGTC

GTTTGCCATATACAGAACGCGTGTACGAATTCGATTAAGCGTTACGAACG

AACCGGAAATTCGGCCGATACGGGCCGGAAATCCGGGATTCCTCGGATTC

AGTCTACAATTCGTGTGAGAACGCCGGGAGCGCGCGAACGAGGCGGCACC

GTTCGGAGCGAAGCGCCCGGCCACGGCGCGACGACCAACCGAAATCGAAC

CGACCACCGATTTTTCGACCGTTTGTAAACGGATTCCGTTCTCGACGTCA

CCGATAACCACAAATTTTATGGTAATGTTTGCACTTGTCGTGACGTAAGA

CACCAGGTAGCATGGTGTCCGTCGGAGCGATAGCATGACAGACCACATTA

CAGCACACGGGTGCAACGGTCATCGTGGCGCACAGTTCGGGGCGCGGGCC

TCGACCAGTCAGTCGGGAGGTGACGGCTCCGTGCGGACGGAGTCGAGAGG

CGCGGTCACTCACGGGGGGTGTCGGCGGTGAGTTCCTCCAACGGCGCGGT

CAGCGAGTTCCTCGACGAAATCGAGCCGGTGATATTCGCGTTCGGCGCGG

CGATAACGCTGCTTTTCGTCGGCGCGTTCAGCCTCAATCCCGAAGGCTCC

TACGAGTTCGTCCTCGGGATTCGCCGGTGGATTCTCGCGACGTTCAACTG

GTTCTTCCTGATAGCGATGCTCGGGTTCGTCCTGTTCTTGGGCTTCGTCA

TCTTCGGGCCGTGGGGGAACCTGAAGCTCGGTGACGAGGACCCCGAGTAC

GGCTTCCTCTCGTACTTCGCGATGATGTACTCCGCCGGCCTCGCGGCGGG

CATCGTCTTCTGGGGCCCCGCGGAGGCGCTGTTCCACTACTCGACGGTCC

CGCCGCTGTACGGGGCCGAGGCCCAGTCCTCGGCGGCGATGCCGCTTGCG

GTCCAGTACTCTATCTTCCATTGGAGTCTGACCCAGTGGTCGTGTTTCAC

CGTGATGGGTCTCGCCATCGGCTACTTCGTCTACAACTACGACGCGCCGC

TTCGCGTGTCGGCGGTCCTGACGCCGATTCTCGGTGCGGACAACGTCGAC

GGCGCGATAGGGAAAACCGTCGATATCCTCGCCGTGTTCGCGACCCTCGG

CGGCGTCGCAACCTCGCTGGGCTTCATCGGAAGCCAGTTCATCACCGGCC

TGAACTTCCAGTGGGGAATCCAACTTGGCGACGTGGGAACCATCCTCGTC

ATCACGGGCATGGTCGTCATCTTCACCATCTCGTTAGTGCTGGGCGTCGA

CAAGGGGATTCGACGGCTCTCGAACTTCAACATGGTCGTGTTCGGCCTAT

TGATGCTCGCGACGCTGATATTCGGGCCGACCTTCCGCATCCTCGAACTC

GGTACGCAGGCGACCGGCGGCTTCGTCGGTGACTTCTTCCAGATGAGCCT

GTTCACGCAGGCCACCGCGACCAGCGCCAGTAAGTGGGTCAACGCGTGGA

CCGTCTTCTACTGGCTTTGGCCGCTCGCGTGGTCGCCCTTCGCGGGGCTG

TTCATCGCCCGCATCTCGCGCGGTCGGAGCGTCCGCGAAGTCGCGTTCGC

CGGCATCGGCGCGACCTCGCTCGCGACGGTCCCGTGGTTCGCCATCGTCG

GCGGCGCGGGCGTCATCATGCAGCACACGGGCGCGGCGAACGTCCTCGGT

CCGGTCTCGGAGTACGGCGAGGCGGTCTCGGGGTACGTCCTCTTCGGCAA

TCTCCCCATCGCCGGGCCGCTCCTCCTGTTCGCGTTCCTCGTGCTCGTGA

CGACGTTCTTCGTCACCTCGGCCGACTCCTCGACGCTCGCGGTGTCGATG

ATGACCACCGGCGGGAAAGAAGAGCCGTCCGCGCTCAACCGCATCTTCTG

GGCCGTCCTCCAGGGCGCGGTCGCGTCCATCCTGATGGTCGTCGGCGGCG

TGAACGCCTTGCAGTCCGCGGCGATTATCACGGGCGCGCCGTTCGCGATT

ATCTGCGTGATAGCGACGCTCGGACTCATCCGAACCTTCCAACAGGACTA

CGGCAGCCTCCTCCTCCAAGACGAGACGCGCCTCTGGGGGAAGTCGGAGT

CGACCGGCGGGCAAGCGCCGGCCGTGAACGTCTCCAGCCACGACGACGAC

TGACGCCGAGTCCAGCCGACCGCAGTTCCGTCCCCGTCGCCGTCCGTCTC

CGTTCTCTGCGCTCGGTTCCCATCGCGAGCGCCGCCGGCGCGGTCGATAC

GGCCGACGCGAATAGACCAACACGGTCGTCCCTGAGATACCAAGTTTTAA

TGTATGGCATTAACAAATCCTGTGTATGAATCGGGATACCGCAGAGCCGG

ACGCAGCAGCGCTGCCCGGACCCAACGCCGAGAAGTGGGTCGAGTTCCAC

CACGAACACGCCGCGCCCAGCGAGTACTCCCACGAGTTCGTCTGGGACGT

GACCGCCGAGGCCGACGGCCCGTTCGTCACCGACGTGGACGGCAACGTCC

TCTTGGACTTCACCTGTCACATCGGGGCCGCCCCGCTCGGCTACAACAAC

GAGAAGGTGCTGTCGAAACTCCGGGAGTTCGACCTCGTCGAGCCGATGAA

AATCGCCGGGCAGGACATGTACTTCGGAGCCGGTCCGGACCCCGAGACGG

CCGAGTTCCCCGGGGCGAGCCACCTGATGGACAAGCTCACCGACGTGTCC

TCTCACTACGGGATGGACACGGTGTTCCTGTCGAACTCCGGGGCCGAAGC

CGTCGAGAACGCGATGAAGATAACGCACGACCACGAGGCCCCCGCGAAGT

ACGGCTACGCCTTCGAGGGGAGTTTCCACGGCCGGACGCTCGGGACGCTC

TCGCTCACGAAGTCGAAGGAGGTCTACACCCGCCACTATCCGCAGGTCGC

GGGCATCGAGACGGTGCCGTTCTGCGCGGATTCGGGCTGTTCGGGGGACG

ACGACGCCTGCGACTGCGGCTTCTTCGCGGGCGACGGCTCGCGGTTGCGG

AACTCCCTGTCGCCGGAGGGCGGCCACGTCAACCCCGACGAGGTCGCCTT

CGCGATTCTCGAACCGATTCAGGGCGTCGGCGGCTACCGCTTCCCGAGCG

AGGCGTTCATGGCCGAGGTGGGCGACGTCTGCGACACCTACGACATCCCG

CTCGTCGTGGACGAGATTCAGTCGGGCGTCGGCCGCACCGGCGAGATGTG

GGCCGCCGACCACTACCCCATCGAACCCGACGTCATCGCCAGCGCGAAGG

GACTCCGCGTCGGCGCGACCGTCTCGCGGACCGACGTGTTCCCGACCGAG

AAGAACCGCCTCGGGTCGACGTTCGGCGGCGGCGACCTGCTCGCGTCGAT

GCAGGGCGCGCTCACCCTCGACGCCATCGAGGAGTACGACCTGCTCGACA

ACGCGACCGAGCGCGGCCGGCAGGCGAGGGAACTCCTCGCCGACGACGCG

CCCGACCACGTGGTCGACGTGCGCGGCAAGGGCCTGATGCTCGCCGTCGA

GTTCGACACCAAGAAGCGCCGCGACGCCGTCGTCGAGGCGGCGCTCGACC

GCGGCCTGCTCACCCTCGGCTGCGGGAAGAAGACGATTCGCCTCCTGCCC

CCGCTCGACTCCACCGAGCGCGAAATCGAACTGGGCGTGGGTATCTTCTG

CGAGGCGATGGACGCCGTGGCGACCGAAGCGGTCGCGTGAGGGTCGGCGT

CGCCAGCGAACAGCACAGAAGAGATGCAGGCCCGAGAGGGGCCACCCCCG

TTTTTCCGGACGCGTCAGGATGACTCAGTAGACTCGCCGCGCTCGTCCAG

CGAGTGCGAAGCGACTTCGACTCCGTCAAGCGCCGAAAGCGAGTCGAGGA

CGTCCGCGAGGTGGTCCGGGCCGCTCCCGTCGAGTCGGACCTCGACCGGA

ACCCGATTCGGCGCGTCGGACGCGGTTCGGGCGGCGCGCTCGACGCTGTC

GAGTTCGGCCCCCGAGGCAGCGACTGCCTCGGAGAGGTCGCCCAGCGTCG

CCGGCCAGCCGTCGAGTTCGAGGCGGGCTTCGACGGCGCGCCCGAGCGCC

TGCATCCCGACGCGAGTCAGTTCGGCGTGTTCGGAGAGGCCGACGTTGCC

GCCGGAGACGACGGCCGCGACGCGCTCGTCCTCGACATCGACCGCGCCGG

AGAGCAGCGCGGCGACCGGGGCCGCGCCGGCCGGTTCGGTGACCGTCTTC

GCCCGCTGCGCGAGGACCGTCGTGGCGACCGCGAGGTCCGTGTCGTCGAC

CGAGACCACGTCGTCGACGCGGTCGCGGACGACCGCGAAGGTCCGCTCTA

ACAGTCGCGCGTCGGCGATGCCCTCGGCGACGGTATCGACCGCCGAGAGC

ATGCGAATCTCGTCGGCCTCCAGCGAGGGCTTGGCGTGGGCCGCGCCCTC

GGGTTGGACGCCGATAACTCGCACCTCGGGGTCCCGCGCCTGCATCGCGG

TGGCGATGCCCGAGATGAGCCCGCCGCCGCCGATGGAGACGAGAACCGCG

TCCACGCCGGGCGCGTCGGCCGCGATTTCCCGCCCGACGGTTCCCTGCCC

GGCGATGACGGCCGCGTCGTCGAAGGGGTGGACGAACTCCAGTCCCTCGT

CCTCTGCGAGTCGCAGGGCGTGTTCGTACGACTCCTCGTACAGTTCTCCT

TCGACGACGACGTCCGCGCCGTAACCGCGGGTCGCGTCGATTTTCACCGC

CGGCGTGATTTCGGGGACGACGATGGTCGTGTCGATGTCCAATAGGTCGC

CCGCGAGTGCGACGCCCTGCGCGTGGTTGCCGGCGCTGGAGGCGACGACG

CCCCGCTCGCGCACCGACTCGGGGAGTTGCGCCATCGCGTTGTACGCGCC

GCGAATCTTGAACGACCCCGTCCGCTGGACGTTCTCCAGTTTGAGGTCGA

CCCGCTCGGCCCCGCAGCGGTCGGCTATCGTCCGCGAGCTATCGAGCGGC

GTCCGGTGGACCACGCCGTCGAGGCGCTCGTGGGCGTCCGCCACGTCGTC

GGCGGTGACGATTTCGGACGCCGGCGGTGCCGACTCGCCGGTGACGCGCC

CGTCGCTCATCGGTTCGCTCTCGCCCCGACTGTCGGTGCCGACTCGAACC

CCGCGACCGACAGAATCGACCGCGTGAGCACGTCGACGCCGGTTTCGAGG

CTTCGTTCGTCCACGTCGAACGTCGCGGTGTGGTGGCTCGTCGGATGGTC

GGTCCCGACGATAGAGTAACAGGCGAGGCCGCCGTCGCGCTGGACGCGCT

CCATGAGGAACGTCGCGTCCTCGCTCGCGCCGAAGTCGGCGGTCTGGACG

ACGCGGTCGACGCTCTCGACGGTCTCGGCCGCGTCGGCCACAGCCTCCAC

GAGTTCGGGGTCGCTGTCGGCCCGGGGACTCTCGCTGACCACGTCGACCG

TCGCCTCGCAGCCGTGCAGTTCGGCGGCCTTCTCGAACCGCCGGCGGAGT

TCGGACTTCGCGTACTCCATGAGTTCAGTCGTCTCGCCGCGCGCCTCGGC

GACGGCCTCGACCTCGTCGGCGATGACGTTGCTCGCGGTGCCGCCTTCGA

CCCGACCGACGTTGACGCGAGTCATCCCGTCGGAGTGTCGCGGGATGCCG

TAGACGCTCTCGATGGCCGTTCCGAGCGCGTGGATGGCGTTCGCGCCGGC

CTCCGGCGCTTTTCCGGCGTGGGCGGTCGTCCCCTCGATTTCCGCGTCGA

TGTGGCACATGGCGAGCGGTTTCTCGATGCCGGCGACGACTTCGCCCGTC

GGGTGGTCGAGGCCGACGTGGACCGCGAAGAAGTAGTCGATGCCGGCGGC

GTACTCGCTTTTCGCCATGGGCGCGCCGCCCCCCGAAACCTCCTCTGCCG

GCTGGAAGAACACGGCGAGGCGACCCGAAAAGTCGCTCTCCTTGACGGCT

TCGAGCGTCGCCAGCCCCCACGTCATGTGGGTGTCGTGGCCGCAGGCGTG

CATCGTCTCGCCCGTCTCCGAGCGGAAGCCCTCGGCGGCGGGGTCGTGGC

CCGCGTCGTCCGACTCCTCGATGAACAGCCCGTCGATGTCGACCCGGAGA

CCGACCGTCGGTCCCTCGCCGCGGTCGAGGACGGCGACACAGCCGGTCGT

CCCGCCGCGGAGGGGTTCGAGAAGCTCCGGGTCGGTCCCGCGGTCGAGCG

CCCGCTCGTACCACTCGTCGAGCCGGTCGTCGCTCGGGACGGCCATCCGG

TCTGCGGGGTCGTAGGCGTCCGCGCCGACGGCGAGTTCGTCCACGCCGAC

GGCGCGGAGTTCCTCGACGAGCCGCGCGGTCGTCCGGAACTCGCACCAGC

CCGGTTCGGGGTGGCGGTGGAGGTCTCTGCGAAGCTCCGCTAATCGCGCT

CGAACGGCGTCAGCCATGCGTACACGTACCACCCGCCACCCACTTAAGTA

TAAACGGTATCCGTGGACACCGGCTACACAAAAAGACTAAGGGAGCGTCA

CCCAATCGATGTAGCAACCGCGCCCGCGACGCGCGCGACCGATATCATGA

CCGAGACAGACATCGTCGTCCTCCGCGAGGGGACCGAGGGCCTTTCGATG

GAATCGTACGCCGACGCGCTCCGCGAGCGCCTCCCGGACCGGACCGTGAC

GCTCGCCCGGACGCCGAAACAGGAACGCGAACTCGTCGTCGACGCCCGCG

TCGTCACGGGCATCACGATAGACGAGGACCTGCTGGACCGCGCCGACCGC

CTCGAACTGTTCGCCTGCACGTTCGCCGGCACCGACCACGTCCCGATGGA

CGCGCTCGCGGACCACGGCGTCGCCGTCACCAACGCCGGCGGCATCCACG

CGCCGGGCATCGCCGAGCAGGCCATCGGCAACATGCTCGTGTTCGCCCGC

CGACTCCACGAGGGCTGGCGGCGCAAAGAGCGCGCCGAGTGGCGGCACTT

CCAATCGGGCGAGTTCACCGACAGCACGGTGACGATTATCGGCCTCGGCT

CCATCGGGCAGGCCGTCGCCCAGCGCTTGCAGGGCTTCGAAGTGGAGACC

ATCGGCATCCGCTACACCCCGTCGAAGGGCGGGCCGACCGACGAAGTCGC

CGGCTTCGACTCCGACGCGATTCACGACGCGCTCTCCCGGAGCGACTACG

TCGTGGTCGCGTGCCCGCTGAACGACCTGACCCGCAGGCTCATTGGGGAC

GCGGAGTTCGCCACGATGCCGACCGACGCCGTGCTCGTCAACGCCGCCCG

CGGCGGCATCGTCGACACCGACGCGCTCGTGTCGGCGCTGCGGTCGAACA

AGATTCGCGGGGCCGCGCTCGACGTGACCGACCCCGAGCCGCTCCCCGCC

GACCACCCACTGTGGGGACTGGAAAACTGCCTCATCACGCCCCACACGGG

CGGCCACACGCCGAAGCACTGGGACCGCCTCGCCGACATCGTCGCCGGGA

ACCTCGGGCGGCTCGACGAGGGCGAGGAGCTGGAGAACCAAGTGCTGGCC

GCCGAGTCGAACTGACTCCCGATGGCGACGCAGGAACGTCCGGACGACTC

CAGCGACGGCCAGCGGACCGACCCGTCAGCCGACCCCGGCGACGACCCGC

GCGCCGACTACGACTACGTCGGCGGCGACACCGACCGCGAGGCGCTCGTC

CGCGACCTCGAACGCCTCGTCGACGGCGACGTGCGATTCGACGAGTACAC

CCGACAACTGTACGCGACCGACGCCTCAGCGTACGAGGTGCTCCCCGTCG

GCGTCGTCATGCCGACCTCGACCGCCGACGTGGCGGCCGTCGTAGAGTAC

TGCGCCGACCGCGAGATTCCCGTCCTCCCCCGCGGCGGCGGCACGAGCCT

CGCCGGACAGGCCGTCAACGAGGCGGTCGTCCTTGACCTCTCGCGGCACA

TGGACGGGGTCCAGTCGGTGGACTCCGAGGCGGCGACGGCGACCGCGCAG

GCCGGGGTTACGCTCGCGGACCTCAATAACTCCGCGGCCGACCATGGTCT

CACTTTCGGCCCGGACCCCGCCGCGGGCGACCGGAGCGTCCTCGGGGGCG

CTATCGGCAACAACTCCACCGGCGCGCACTCCCTGAAGTACGGCAAGACC

GACTACTACGTCGAGGAGTGCGAGGTCGTCCTCGCCGACGGGAGCGTCCA

CCGCTTCGACGAGATGCGCGTGGCCGAACTCCGGGAGAAGGCCGACCCCG

AGAGCGACGCGTGGGGTTCCGACGGGCCGCAGAGTCCGCTTCTCCCCCGA

ATCTACGCCGAGGTCGTCCGCGTGCTCGACGAGGAACGAGCGGAGATAGA

CGCCCGCTACCCGGACCTCAAGCGCAACGTCTCGGGCTACAACCTCGACG

CGCTGGTCGAGGAGGCCCGCGGGGAGCGCCCGCTGGCCGACGGGACCGGA

ACCGACCCGGACTGCGGGTCGGGGACCGTGAACCTCGCGCGCCTGCTCGC

CGGGAGCGAGGGGAGCCTCGCCGTCGTGACCGAGGCGACCGTCTCGCTCG

AACCGCTCCCGGAGACGAAGGCGGTCGCGCTCCTCACCTACGAGGACGTG

TTCGACGCCGCCGCCGACGTGGCCCCCATCCTCGACCACGACCCCGCGGC

GGTCGAACTCATCGACGACGTGCTCATCGACCTCGCGCTCGACACCGCCG

AGTTCCACGACGTGGCCGCGTCGCTCCCCGACGGGACCCGCGCGGCCCTG

CTGGTCGAGTTCTACGCCGACTCCGACGCGGACGGCCGGCGGAAAGTTGC

CGACCTGCTGACGGCCCGCGCGCCCTCGGTGACGCCGGAGGCGACGCCGT

CTTCGGGCGTCGGCGTCGACGACGACGCCCGCGCGTTCGACGCGCTGGAA

GCCCACGACGAGGCGGCGCGCGCGGGGCTCTGGGAGATGCGCAAGGCCGC

CGCGCCCATCCTGCTCTCGCGGACGACCGACGAGAAGCACATCTCGTTCA

TCGAGGACTGCGCCGTCCCGGCCGACGAACTCCCGGGGTACGTCGAGCGC

TTCCGCGAGGTCATCGACGACCACGACGCCAACACGAGTTTCTACGCCCA

CGCGGGGCCGGGCGTGTTGCACGTCCGCCCGCTCGTCAACACGAAGACGC

TCGACGGCCTCGACTCGTTCGAGACCATCGCCGACGAGGTGACCGACTTC

GTCGTCGAGGCCGGCGGGTCGGTGTCGGGCGAACACGGCGACGGCCGCGC

GCGAACCCAGTGGAACCGCAAACTGTACGGCGACCGCCTCTGGAACGCCT

TCCGCGACCTCAAGACGGCGTTCGACCCCGACTGGATCCTGAATCCGGGG

CAGGTCTGCGGCGACGTGTCGATGACCGAGAACCTCCGGTTCGACCCCGA

CTACGAGTTCGACGCCGGCTTCGACCCCGAGCTCGAGTGGGACACCGAAA

ACGGCTTTCAGGGGATGGCGGAACTCTGCCACGGCTGCGGCGGCTGTCGG

GGCGACCAGTCGACCACCGGCGGCGTGATGTGTCCGACCTACCGCGCCAC

GGAAGAGGAAGGATTGTCGACCCGCGGTCGCGCGAACATGCTCAGACAGG

CGATGAGCGGCGACCTCGACGCCGACGCGACGGACACCGAGTTCATGCGC

GAGGTGCTGGACCTCTGTGTCGGCTGTAAGGGCTGTGCCCGCGACTGTCC

GAGCGAAGTCGACATGGCGAAACTCAAAGCCGAAGTCGAACACGCCCACC

ACCAGCGCGAGGGTGCGAGCCTCCGAGACCACCTGTTTGCCGAGGTGGAC

CGGCTGAACGAACTGGGGTCGCGACTCGCTCCGCTTTCGAACTGGGCGGC

GAAGGTGCCTGGCGCGCGGACGGTCATGGAGAAGACCGTCGGCATCGCCC

GAGAGCGGTCGCTCCCGACGTTCCACCGCGAATCGCTGGAAGACTGGTTC

GAGGCGCGCGGCGGCGCTCGCGTCCCCGAGGCCGAGGCGACCGACAAGGT

GCTTCTGTTCCCGGACAGCTACACCAACTACAACCACCCCGACGCGGGGA

AGGCCGCGGTCCGCGTCCTCGAAGCCGCCGGCGTCCACGTCTCGATTCCC

GACGACGTGACCGCGACCGGCCGCCCGGCCCACTCGAAGGGCTTTCTCGA

CCGGTCGCGGGCGCGCGCCGAGACGAACGTCGACGCGCTCGCGCCCCGCG

TCGAAGCCGGCTGGAGCGTCGTGCTCGTCGAGCCCTCGGACGCGGTCATG

TTCCAGTCGGACTACCTCGACCTACTTTCGAGCAAGGACGCCGAGCGCGT

CGCCGCCGAGACCTACGGCATCATGGAGTACCTCGACGCGAAGCGTCTCG

TGGAGGACCTCGACCGCGACGCCTCGGCGGGCGCGGACGACGAGACGCTG

ACGTACCACGGTCACTGCCACCAGAAGGCGACGCGGAAGGACCACCACGC

TGTCGGTGTCCTCCGGCGCGCCGGCTACGACGTGGACCCGCTCGATTCCG

GCTGTTGCGGGATGGCCGGGAGCTTCGGCTACGAGGCCGAACACCACTCG

CTGTCGACGGCCATCGGCGGCATCCTCGCGGACCAGATTAGCGGGAACCG

CGGCGACACCGTCGTCGCCCCCGGCGCGTCGTGTCGGACCCAACTGGAGG

ACGTGGACGGCGCGGCCGACGACCCGCCGCACCCGATAGAGAAGGTCTCC

GAAGCGGTCGCTGACTGAGACGCGCGCCGAAAAAGAACCGCGAGTCGTGT

CAGCTTTCGCCCGACGCGCAGTCGCTCAGGGCCAGAGGCCCCGCGTCTCC

TTCGCGCCGCCGATGCGAGAGAGCGCGACGACGTACGCCGCGTCGCGCCA

CGTCAGGTCGCGTTCTTCGACGTGTTCGCGGACCGCGTTCCACGCCTTGA

GCATCTCCGATTCGAGTTCCTCGTGGACGCGTTCGAGCGACCACTGGCGG

CGGTTGATGTCCTGTAACCACTCGAAGTACGAGACCGTCACGCCGCCGGC

GTTGGCGAGGATGTCGGGAATGACGGGAATCCCGCGCTCTTCGAGGACGG

CGTCGGCCGCGAACGTCGTCGGCCCGTTCGCGCCCTCGACGACCATCTCC

GCCGAGATGGAGTCGACGTTCTCGGTCGTGATGACGTTCCCGATGGCCGC

CGGGATGAGCACGTCCACGTCGAGTTCGAGCAGTTCCTCGTTCGAGAGCG

ACTCGGGGGCGTCGTAGCCCGACACCATGCCGGGGCGCTCGTCGTGGCCC

TCGACGTCCTGCGTGTCGAGGCCGTCGGGGTCGTAGATGGCTCCGTCCAC

GTCGGAGACGGCGACCACCTTCGCGCCCCACTCGTCGAGGAGGCGGGCGG

CGTTCGCGCCGACCGAACCGAACCCTTGGACCGCGACGGTCGTGTCTTCG

ATATCCCAGTCGTAGAAGTCAACGGCCTCGCGGGTGACGATGGCGACGGA

GCGACCGGGAGCCTCCTCGCGGCCGTATGACCCGCCGATGACCGGCGGCT

TGCCCGTCACGACGCCGGGCGTCGTCTCGCCCTGCTGCATCGAGTAGGCG

TCCATGAACCACGCCATCTCCTGCGGACCGGTTCCCATGTCGGGCGCGGG

AACGTCCTTCTTCGGCCCGACGACGTTGCGAATCTCCTCGGCGAAGCGGC

GCGTGAGTCGCTCGCGCTCCTCGTCGGAGAGCGTCTTCGGGTCGACCGCG

ACGCCGCCCTTGCCGCCGCCGAACGGCAGGTCCATCACGGCGCACTTCCA

GGTCATCCACATCGAGAGGCCGATGCACTCGTCGGCGGTCACTTCCGGGT

GGTAGCGAAGTCCGCCCTTGTACGGCCCGCGCACGTCGTCGTGCTGGGCG

CGGAAGCCGGTGAACACGTCGAGCGAGCCGTCGTCGCGGCGGAGCGGGAC

CGACACCTGCTGGACTCGCGTCGGGTGCTTCAGGCGCGCGACGACGCCGT

CGTCCACGTCGGCGTGACTCGCCGCGCGTTCGAGTTGCCGTCGCGCGGTC

GCCAGCGCCGTCTCGGGTTCGGACCCCGAGCCGCCGTCGGTGACCGCCTC

GCCGTTTCGATTTTTGCCATCTTCGTTCATCATATTAGGGCTGTGACCCG

GAGTCATAATTAAGTTGTGAATAGCCGGCGGGGGAAGTCGGCCAGCGGTT

CCGCCCCGTCGGACGTGACGACGAACGTCTCGCTGAGTTCGACGCCGAAG

TCGTCGAACCAGAGACCGGGGATGGTGTGGAACGTCATGTTCTCTTCGAG

GACGGTCTCGTCGCCGGGGCGGAGGCTCGCCGTGTGTTCGCCCCAGTCCG

GGGGGTAGCCCAGCCCCATCGAGTAGCCGATGCGGTCTTTCTTCTCGATG

CCGTACTGAGCGATGGTGTCGCGCCACGCCTTCTCGACGGCCTCGCAGGT

CACGCCGGGTGCGACCGCGTCGAGGGCGGCTTCCATCCCCTCGACGACGA

TGTCGGCCCGCCGGGACAGCTCTTCGGGCGGGTCGCCGACGAACGTCGTC

CGTGCGAGCGGCGAGTGGTAGCGGTGGCGACAGCCCGAGAGTTCGATGAT

GACCGGGTCGCCGTCCTCGAACTCGCGGTCGGTCCACGTGAGGTGCGGCG

TGCCGGTGTGGTCGCCAGAGGGCATCAGCGGGACGATAGACGGGTAGTCG

CCGCCGTAGTCGTCGGTTCCCTCGATGAGCACCGAGTAGATTGCCTCGGC

GGCCTCGTACTCGGGGACGCCCGCCTCGATGACGTCGAGGCCGGCCTGCA

TCGCGTTCTCGGAGATGCGGGCGGCCTCGCGCATGTAGTCGAGTTCGGCG

TCGGACTTCTTGATGCGCACGCGGTTCACGAGGAGCGTCGTGTCCTCGAA

GCTCGCCTCGGCGAGATTCTTTTGCAGGCGCGTGTACGACTTCGCGGTGA

AGTAGTAGGCGTCCATTTCGAGGCCGACGCGAGCGTCGTCGACGCCGAGG

TCCTCCAGCACGGCCGCGACGAAGTCCATCGGGTGGAGGTCGTGCGGCGA

CTGGACGTGGTCGTCGCTGTACGGCCGGATGCTCTCCTCGCTCAGCGTGG

TCGTCGCGCGCGCGCCGTTGGCGTCCATGTCGCGGCCGACCCACACGGGT

TCGTCGCGGTCCCGCGTGACCACGACGCCCTGATGGACGTAAAACGACCA

GCCGTCGTAGCCGGTGAGATAGTTCATGTTCGCGGGGTCGCTCACGAAGA

GCGCGTCCAGTCCCCGCTCGGCCATCCGCTCTTTGGTCCGGGCGATGCGT

CTGTCGTACTCGGCGCTGTCGAATACGGCTCTTGGCATGGGAACTTCTAT

CGAACCATCGAATAGGGCCCGTAAAAATTTTTTGTGGACCACGGTCACGG

ATACTGTGTACGCTAACGCCGCGGTGGCAGTTAGTATTGTATATTATCCA

CATGACTATATCCGATGCAGAGGGCCACAGGTACCGGTCGTGTCTCATTA

CAGCCACATTAAGCCTCCAGAAATTTAAATTACCTAAACTATGTGACTAA

TATAGATAATCTAGATTATTTCACCAATCTATTGTGTTCCACAGATACAA

TTCTAACTGCCACCGGCTCACCGAGAACAGTACGCATCTGCTGGGAGCCC

AATCGTTATTCCTCGCAGTCTCGGAGCGGGGGTATGGACTTCTCTATCGT

CGACCTCTCGCCGGTTCCAAAAGGTGGGACCGCGACCGACGCGTACGCGA

ACACCGTCGAAACCGCCAAGCAAGCCGAGCGACTGGGTTACTCGCGCTTC

TGGGTCGCCGAGCACCACGGCATGGCGAACACCCTCGCGGGGACGACGCC

CGAGGTGTTGCTCGGCCACCTCGCCGCCGAGACGGACTCGATTCGGCTCG

GTTCCGGCGCGGTGTTGCTCAACCATTACAGCCCGTTCAAGGTCGCAGAA

CAGTTCGGCGCGCTCGACGGACTCGCGCCCGGCCGCATCGACGCGGGACT

CGGGCGGGCGAACGGGTCGCCGGCGGCGGACCAGGCGCTCGGGACGGACC

GGCACGTCGAGGACCCCGACGGCGACCACGCGGAGAAAATCGAGGCCGTC

GTCAACCACCTCTACGACGACTACCCCGACGAACACCCCTACGCCGACCT

CGAAATCCCGCGGGGCGACGGCGACCTGCCGGTTCCGTGGGTGCTCGGGT

CGAGTCCGTCGAGCGCGACCATCGCGGGCGAACTCGGACTCCCCTACTGC

TTCGCGGGCTTCATCAGACCCCAGTTCGCCGTCCACTCCTTCGAGGAGTA

CCGCGAGAGCTTCCAGTCGTCGTCGCTCGACGGCGGCATCGACGAACCAC

ACGGCATGGTCGCGGTGAACGCCGTCTGCGCCGAGACCGACGAGGAGGCC

GCGCGACTCCGCGCGGTGGCCGAGGCGTCGTACAAGCGGATGCGCCGCGG

CGAGGTCGGCACGCGACCGACCGTCGAGGAGGCGATAGACGAACTCGGCG

GCGTCCCCGAGCCCACGCCGGCGACCCTCGGAGCGAAGGAGTGGCCGCGC

GCGATTTCCGGCAGTCCCGACACGATTTCGGGGCTGTTGGACCAGCTCGC

GGAGCGCGTCGGGGCCGACGAGATGATGATTCAACACGTCGTCGGCAATC

ACGAAGACGGCCTCCGCTCGCACGAGCTGCTCGCAGAGGGCGTCGACCTC

GTCTGACGCGCCGGTCTCCGAGACAGTCCGTCTCCCGGTCGATTCATATC

CCAGTCCCCGAAATGAGAGGTGTGTCACGCCTACGTCATCTCCGGCTCGA

AGACACGCCGTTCGAGGCCAGAGAGTTCTCGCTGCTCTGGCTCATCGCGC

TGGCGACCTACGGCGTCGGCGACATCGTCACGACGCTGACGATTCTCTAC

TACGAGCGCCGGTTCGACGAGGCGAACGCCCTCCTCGTGGCGAGCACGGA

CGCTCTCGGACGGTGGGGGCTGGTCCTCCCGAAGCTCGCGGTGTTCCTCC

TCTGTCTCGGTATCTGCGTCTACGGCGCTCGCGCCGGCGACAAGGTGCTG

TACTACCTGCCGCCGGTCACGCTCGCCGTCATCGGGGCGTTCGCCACGGC

GTTCAACCTCCGGCTCTTCGTCGGCTGAGCGGCGAACGACGGAAAAACGA

GGCGCGAACGGCTCAGCCGGTCGCCTGTTCGAGCGCGAAGACGAACTCCA

CGTCGACGCTGTCGGTCTGTATCTCGTTGCCTGCCGCCTCCCGAATCTGC

CACTCGACGACGAGCGACAGGGACTCCCCGCCATCGAGTCGGCCGAGCGA

GACGGATTCGTTCCACACGTCGGCGAAGTCGAGATACGTCGTCTCATTTC

CGAGCACGTAGGAACTCGTCCCGTTGGCCCGCTCGACGCGGAGTCTGACG

GCGAGCGCCTCGTGGAGTTCGCCCTCGTCGGCTCCGCCGGTCGAATCGAC

CGCTAACTCGGGGCCGGAGCGACCGTTTTCGGCGGACTCGAACGGCATCG

ACTCCAGCGTCAACACGCCAGGGTCGCGCCCGCGGTTGGTGAGCGTCCCC

GTCGAGTTGCCCGCGGAGCCGGGTGCGACCGACTGGGTGTCGAGAAGCGA

GACGCCCGCCCCGCCGGAGCCGAGTTCGAGGTCGACGCCCGCCGGGGCGC

TCCCGGCGGTGTTCTCGACGGTCACGTCGAACGACGCCGTCTCCTCGGTT

CGGGCGACCTCGGTCGAGTCGCGGAAGAACACCGCGGTCACGGAGACGTA

GCCCGTCGTGACAGTCGTCTCGCCGTCGTCGGTGGCGGCGAAGCCGGCGG

TTCGGTTCCCCTCGGCGTACGTCACGTCGGAGCCGAGTGCGGTGGCGATG

TCCACGGGACTCGACTCCCGGAGCGTGACGCGCCGCTCTTCCTGCCAGCC

GTCGTCGGGGACCCACGCGTTCACCACGAGCACGACGCTGTCGACCGTGC

CGGTCCACGAGAGCGTCCCGGTCACGGGGCCGCTGAGCGTCCCGACCGTC

CCGTCGACGCTGCGGACCGTCGCCGGGTCCGCGGCCTCGACCGTGAGCGT

CGGGTCGGGCGACACCGACGATTCGGCCGTCGTCGTCGTTTGACTGTCTC

CGGAGCCACCACCTCCACCGCCACCGCCACTGCCACCGCCACCGCTACCG

CCACCGCCGCCTCCAACGCCACCGCCAGTTCCAGCGCGTGCCGTGGTGGT

CGGGGGTGGACTGCTCGTCGGCGCGGGCGTTGGCGTGGGCGACGACGGGG

GCGGGTTCGGCGTTGTGGTCGCGCTCGGCGTCGACGCGGGTGTCGGGGTG

TCCGGGTCTTCGAACTGGTTCGTTTCCTCCGGTGGGGCGTCCGGCGTCGG

CGGCGGCGGGGGAGTCACGGTGACGGTCGGCGGCGACGGCTCGGGGTCGT

CGCCACGGAGGTCCGAGAGCACGCCGTTGATACCGCGGTCGTCGAGGAAG

CGTTCGATGCTTCCGGGAGCGGCAAAGCCGGTTCCGACCAGCACGATGCC

GATGACGACGAAGACCATAAACACCCGGCCGACGACCTGCGAGAACTGCG

GCGAGAGCGTGCCGAAAAGTTTCGCCGGCAAGAGCGAGGCGAGCGACAGC

GGAAGCGGGAACAGGCTCGCACGCGGCGGGCGGTACTCGACCACCAGCGC

CGTCTCGGTCGGCCGGTCGCCGGGTTCGACTTCGTCGCCGACGTCGACCG

GACGCGTCTCGGTTCGCGGCCCGTCACCCTCGTCGGAAGAGGACCGCTCG

CGCTCGCGTTCGACGAGCGTGGCGTCACCGTCGACGCCGACGAACGACGC

CGACGACGGGTCCCAACGCCGGACCGCCCGGACGACGCCGTGGACGGGGA

GGCGCTCGCCGGGGAGACAGAGGACGCGGTCGCCCCGCGAGAGACGGTCG

AAGTCGACCTCCTCGAAGCACTCGGCGTCGTCGTCGCGGTCGGCCGCCGA

CGCGTCGACGAGGCGGCGACACCGCGTCGCCGTTCGCCGCGACCACGTCG

GCGAGGGTGGCCGAGCCGCCGAAGCGGTCGTCGAGCACGTCGAGGCTCCC

GACTTCGAACGTCGTCACGTCGAGGACGCCCGCGAGGTCCGCCACCGAGG

CGTCCGCGTCGCCGTCGTCGCCGGCCGTGGCGTCGCTGTCGTTTTCGGTC

AGCGTGAGGTCGATGTCGAGACTGCCGTCGAGCGACCCGTCGTTTTCGAG

GTCGACTTCGAAAGTGTCGGTCTCGTCGCCGGGCGCGAGACCCGTGGCGC

TGAAACTCGCGGAACTGGCTCCGTCGGCGAGGCTCAGCGTCCCCGCCTGA

AGCGTGTTCTCGGTGCTCGATTCGTGTCGTTAAAGAGGGCGAACGTGCCC

ACGCCGGCGGCGGCGCTTCCCGCACCAATCGTGAGGAGCCCCCCGAGGGC

GCGGCGTCGCGTGAGTTTGAATACTTTGTCTGCCATTGAGTTCTCCGTGC

GGCCCACCGTACACCCACTCCCGTCCGTGATATTCTGGGTGGGGTGACCT

TCAGATATCTCGACTCGATTACGTGGGAATAATGGGACACCACCATTATG

CGGTATCCGGCAAAAACCGAGCGGCGTGATAGCGCTCGGCACGGGAGCGC

GGAGGAACCGACGAACGCGTATCGGAGCGGTCGGTTCACCGACCGCCGCG

CTCCCCGTCGAACAGTTCGGCGAAGACCTTCCGGTGGGCCGCCCGGAGGT

GTTGGGTGAACGTCGACCGCGAGACGCCGATGGCCTCGGCGACTTCGGCG

GCGCTCCGGCGTTTCGGGCGCTCGAAGTAGCCCGTCCGGTACGCGACTTC

GAGCGACTCGCGCTGTCGGTCGGTGAGGCCGGCCAGCACGCTCGCGCCGA

TATCGGACTCGACGGTCCGGTAGCTCCGCGCGGTCGTAGCCGGTGAGCGT

CGTCGCCGCCTCGTTGACGAGGACGACGCCGCCGTCGCGGGCGACGAAGA

TGGCGTCGCGGACGTTCTCGACGAGCGTCCGGTAGCGCGCCTCGCTCTCG

GCAAGCGCGCGCTCGGTCCGGTGCTTGTCGACGGCCGTTCGGACGCGTGT

GGCGAGCGCCGCGGCGCTGTCTCCGAGCTCGTCTTGACGATGTAGTCGTT

CGCGCCGGCCGACAGCGCGCTGCTGGCGAGTCCCTCGTTGCTAGCGCCGG

TAAAGAGGAGAAACGGGATGTCCGGTCGGTGCTCGCGGACCTCGGAAAGC

AGCTCGATGCCCGTCCGACCGGGCATGTAGTAGTCGCTGACGACGCGCTC

CACGTCGTCGGTGAGGCGGTCGAGTGCGCCCTCTGGACTGTTGGCCTCCC

GAACGTCGAAGTCGGGGGCCTCCTCGCGGAGTCGCTGCACGACGAGCGCC

AGCCAGTCGCGGTTATCGTCGACGAGGAGGACCGTGACGGGGTCCGCACT

CGTCTCGTCTGTCGTCTCGGTCACGGCGGTTCGGCCGTCCGTGGCACATC

GACCGGCATAAGCTGTTCGGGTGACGGGTCCGGGCGGCGGCCTGAGAAAC

ATGGAATGTTCGTTTGGTTTGCGCCGACAGCGACGGGAATAATCGCTCGC

AAACAGACACCTCTAAATACCAGAGCGGGCACAGAACACGCACCGATGGA

CGCGAGCAGCGGTCGGGCCCGGTGAGAGATGGACTGGCAGTACACCGTTT

TGGTCCTTCCGACCCTCTTTGCGATGAGTACCGCGGTCCTACTCGGGGCG

TACAGCCTCCGGCACATGCAGGCCCACGGGCAGTCGTCCGAGCTCGTTAC

CTTCTTCGCCGTGAACGTCGGACTCGCCCTCTGGACCGGGTTTGCGGCGC

TCAAACTCCTGCAGACCGACCCGGCGCTGAAGCTGCTTTTCTACCGGTTT

CTCTACTTCGGCGTCGCGCCGCTCGGCGCGCTCGCGTTCCTCTTTACGCT

CCTCCACACCGACCGGATCAGCCGAATCACGCCCGGACTGGTCGCGGCGG

TCATGACCGTGCCGGCCGTCTTTCTGGCGGTCCTGTTTGCGAATCCGAAC

GGCCTCGCCATCGAGGCGACGCGCCTCGTCGAGACGAACGGTCTCGTCGT

CCTGCGGGTCGACGTCGGTCTCGGTCACGTCGTGTTGCAGTTGCTCTACA

ACGCGGTGCTCTCCGTGCTGGCTATCGTGCTCATCCTCTACGAAGCGGCC

CGACTGGGTCGAACGTACATCCCGCAGGCGGTGTTGGTGAGCGTCGGTCT

CGCCGCCCCGTTCGTCTTCGTCGCGTGCAGTTCGCTCGGGATCCCGCCGT

TCAACAGCGACGGCGTGAACCTCGTTCCGACCTCGGCCGCAGTCACCTCC

GCGGCCATCGGCATCGCGCTCTTTCGCTACCGGTTTCTCGACTTGCCGCC

CATCGCCTACACGACCGCGATGGAGGAGTCGCCCGACGGCGTGTTCGTCC

TCGACGCGGACAAGCGAATCGTCCACGTCAACACCCGCGGTGCGGCGCTC

TTAGAGCGCTTCGGCGCGGCCGTCGGCGACCCGGTCACCGCGGTCTCGCC

GGACCTCGACCTGACGACGGAACAAAACGACAGCATCGAAACCACCCGAG

AGGGCGGCGAAGCGGTGTATCTCAGCGCCCGCTCCCAGCGACTCGAACGG

GGCGACCAGACCGTCGGGTGGGTCATCGTCCTCCGCGACGTGACCGAGTT

ACACCGCCAGAAACAGACGATTCTCTCCCAAAACGAGAAGCTCACGCTCC

TCAATCAGATCGTCCGCCACGACATCCGAAACGACATGGCCGTCATCCTC

GGCAACGCGCAACTGGTCGAGGAGTCGGTCGACGACGAGGCGCTGCGCGA

GCGCCTCGACACGATAATCAGGAACGGCGAGCACGCCACCGAACTCACGG

TGAACGTCCGGAACCTCATGGCGACGATGCTCGACGACGAGGAGACGAAC

CGGCCGACGACGCTCGCCGGACCGCTGTGGGCCGAGGCGAGCGCGGTTCG

CGACGGAACGTCCGAGGCCGCGGTCTCGCTCCCCGGCGAGCCGCCGGACG

TGACCGTCGTCGCCGACGACATGCTGGGGACCGTCTTCCGCAACCTCCTC

ACGAACGCGGTGCGGCACAACGACGCCGACCGGGCCGAAATCGACGTGTC

GGTCGAGGTCCGCGACGACGACGCGCTCGTCCGCATCGCCGACAACGGAC

CGGGCATCCCCGACGACCGAAAAGAGGTGGTGTTCGGCCGCGGCGAAAAG

GGGCTCGAAAGCCCCGGCACCGGCCTCGGCCTCTACCTCGTCGACACCAT

CGTCAGCGGCTACGGCGGCGACGTGTGGGTCGAGGACAACGAGCCCCGCG

GCGCGGTCTTCGTCGTCCGCCTGCGGAAGGCCTGAGAGCGGGACGACCGC

GCGCCCAAACACTCAAAAAGTCAGCACGTCGATAGGAAGGCGATGCCCTC

CACCGTTCTGCCCGCCGGCGTCAGCCGGTGGCGAGTGGCCGTGCTCGCGG

CCGTCGCCGCCGTCCTCGTCGGTCTGGCGACGCTCACCGACGAGCCGGTC

GGCCCAGTGTTCGCCGCCATGGGTCTGCTCACGCTCGTCTACATGGCGGC

CGGTGCGGTCGATACCGTCCGTGAACACCCCGCCTTCCCGCTCGCTTCCG

CGGTGTACACGACGTTCCTGTTCGCCGGCGGATACGTCAGCGGCGCGCTG

TCGAATCTCCTGTGGGCCGTTCTCGCGGTTCTTTCGGCATTCGGCGTCGT

GGTCGAGGCGTACAACTACCGGCACGGAACGAGCTATCTCCGGTTGGATT

TCGAATGACGGGACCGAGCGAGACCGCCGTTTCAGGGGCGATAGCCGCCA

CGTCGGTCTTCGTGGGACTTGCCGTTGCCTTCGGTGGCCCGCAGGTCAAC

CTCGCGGCCGGCGTCGTCTGGACCGTTGCGACGGGCATGGTGCTCCTGCA

TCTCCGCGGGAAAGTCCGCGACGCTCGGAGCGACGAGTCGCCCGAACCGG

GACCGCTCCTGCGTCTGGCCGAAATCGATTACGACCCGCGGTACGACCGC

TATCTCGGTGTCCTCCTGTTCGCCCTCGGTATCGCGGCGTTCGCGGCGCT

CCTCGTCGTCGACCCGTCCGGTTGGAACGCGTTGTTCCTCGTCGACGTCG

GGAACTGCTGTCTGATTGCCGCGCTCGGGGCGGTCGCGCTCTCCGATAGA

TAGTGCGAAAATCGGAAGGACCACGTCGCAGTCCGCTGGTTTTCGAGGCG

TCGGCCAAAAAGACTACTTAGGGAGCTGGCAATTTCCACATGTCCGCACG

ACGCTGTCTGGTCGGCGGACAGGTGACATATCTGCACAGACAGGCGGCCC

ATCCCGTGCCAACGGTACTCCGACCCTGCCCCCTCGGACTGCCGCCCGAT

GGTCCGCCCCTACATTTCTACCCGGTCATCGTCGTTCAGCGAGCGACCGG

CCTGCGACTCAGTCGCGTCCCGGAGCCGCGCCCCCGGCGAGACCAGCCAC

GCGGGACCCGAGTTCGTCGTAGACGCGGACGACCGTACCGCGGAGCGACT

CCGTCGCGAGGACGGCGAAGCCGGTGAAGATAGTCGCGAAGCCGACGACG

GTGAGCGTCGAAATCGACTCGCCGAGGAGCGCCGTCCCCCCGAGCGTCGC

GACGATGGGCACGGCGTAGAAGACGAGGTTGCCGCGAATCGGCCCCACGT

CGTCGAGGAAGCCGAAGTAGGTGAGGTAGGCCACCGCGCCGGCGAAGACG

CCGACGTAGGCGAGCGCGACGAGCGCCGTCGGAGACCACGTAGCCGCGGC

GAGCGACTCGCCGGAAGCGACGCTCATGCCGTGGGTGAGCGCCGCGCTGA

CGGGGAGCGCCCACGCCGTGCGGACGGTGCTCGAAAGCGACGTGTCGGCC

CAGCGGATGAGCACGGTCCCGAGCGCGCCGCTGACCGCGCCGAGGAAGAC

GACGCCCTTCCAGAGCGCCTCGCCGTCGAGCAGGTTCGCGGGGTCGACGC

CGACGACGAGGCCGACGCCGAGCAGGCCGATGAGCATCCCGAGCACGCCC

CGGGTGGAGAGGCGCTCATCCGCGAGAAGCGCCATGGCGAACACGGGCGT

CAGAATCGGGTTGAGGCTGAAGATAATCGACCCGACGCCGCTGGAGACGT

GCTCCTGCCCCACGAACAGGAGGGCGTTGGCGAGGCCGATGACGAACACG

CCGGTGGCGAGGATGCCGACCACGTCGCGGACCGACCGCGGGAGCAGTTC

GGCGCGGGAGCGGGTGGCGACGACGTAGCCGACGAGCAGCACCGCCGCGA

TGTCGAACCGGAGCGCGACGAACAAAAGCGGCGGCAGGTAGTCGAGACCC

GCCTTCGCGGCGACGAACGTCCCGCCGAAGAAGAACGCCGTCAGCACGAA

CAGGACGAGCCGCCGGCGCGTCGCCGTCACCGTCCCACACCTCCGAGCGA

GAGACCGAACGCAGAGTGAAGTGAACGCATCTTGGATACACGTACGACTC

CCCCGCGTATAGTTTGATTCAGAAATCAATTCACGGCAAGAAAAGTCCGC

GGCGGTCGAGCATGATTCATGGTGTGAAATGATTTCGAGACGCGAAAGAA

GTTTAATCGAGCGCGCACAACGTCGCCGCATGGAGTCGGCATTAGACGAA

ATCGAATTCCTCGCGCTCTCGCAGAACCGCGTCGACGCGCTCAGATACCT

CGCCGAGCGGCCGCACTCCAGACGCGAACTCGTCGAGCGAACCGGGGCGT

CGCAGCCGACGATGGGGCGGATTCTCAGCGACTTCGAAGACCGGTCGTGG

CTCGTCCGCGAGGACGGCGCGTACCGGGCGACGGTGACGGGCGAACTCGT

CTCGCGGGGCTTCGACGACCTCGTCGACATCCTCGACACCGACGCCAAAC

TCCGGCCGGTCGTCGAGTGGCTCCCGACCGACGCCATCACGTTCGACCTC

GAACACCTGACCGAGGCGACGATTACCACGCCGAGTCAGGTCCGGCCGAA

CGCCCCCGTCAAGCGCGCCGTGTCCCTCCTGAGCGAGGCCGAAGACGTGC

GCATCTTCTCCTACGCGTTCAACGAGCAGAACCTCGACATCGTCAGGGAG

CGGACCGCCGCCGGCGACCAGCGGTTCGAAGGCGTCTTCTCGTCGGACGC

CATCGACGCGCTCGCCCACGACTCCCAACTGCGCGAACAGCTCCGCGACC

TCCTCGACGCCGACGCGGCCGCCGTCCGCATCACCGACGAGCCGATTCCG

ATGGCGGCGACCATCGCAGACTCGACGGTCCACCTGTTCCTCCGCGACGA

CAGCGGCATCCTCCAAGCCTCTATCGACGTGACCGAACCGGAGGTGCTCA

CGTGGGCCGACGAGCTGTTCGAGCGCTACTGGGAGGCGGCGGAACCGCTC

GACCTCGACCGACTCTGAAAGCGGGTATCACGACCGACCGCGTCGTCGCC

CCGTCAACCGACTCACGAGACATATCGGCGGCCTGCGAGATGCCAGCAGA

TGCCCTCGTCCCACCCGTCAGTCGAGTTCGTCGAACGAGTCGACCTCGTC

CCAGCAGGAACACGGCAGGTCGTCCAGAGAGGTGACTTCGTCCGGGAGGC

TGGTGATGGGAATCCCGGACCCGGCCTCGTCGCAGTCCTGTTGATGAACG

CTGAAGTCGTCGCTGGACATCGTGTACATGGTCAGCACGCCCTGTTCGCC

GGCTTCATTGAAACAAGTTGCGAGTGTGGCAGCCGAGCGCGGGGCGTCAG

AGAACCCGGAAGACGCGAACCGTGTCGCGCTTGCCCGGTTCGCAGAGCAG

TTGGGCGAGGCCGAACTCGGAGAACTCGTCCTTCCAGTTGCGGTGATACA

GCGGGAAGTCGTCGTTGACGTAGCTCACCTCGGAGCCGGAGCGCCCGCGC

GTCGGGCCGTTGCCCTCGTTTTCGGCGGTTATCACGAGGTCCGAGGAGAC

GCGGGCGATTTCCTCGAACACCCACGTGTCGTCGGGGTGGACGTGCTGGA

GCGTCTCGACGGAGTAGATGACGTCGAACTTGTCGTCGGGGAAGTCGGGG

AGCAGGTCCTCGATTGCGCCGGTGAGGAACGTGCCGGTATCGACGAGTTC

GGGGAAGTACTCGGCCATCACCTCGAAGGAGTCGTCGTTGATGTCGATGC

CGGTGATGTCCTCGTAGCCGCGGTCGTGGAGGTGCGCGAGGTGGCGACCG

GAGCTACATCCGAGTTCGAGAATCGACGCGTCCTCCGCCGCGTAGTGCTC

GAACACGGTCGCGAGCGTCTCGCTGACCTCGTTCGGGCCGATTTGGGCGT

AGTACTCCGGCGAGAAGTCGCCGGAACGCTCGGCCCATCCGCGGTGGTTG

TCGTCTGCGTCCATACCCCGAGTAGCGAGCGGGCGGGTAAATGTTCCGCG

GAGTGTGCGCCACCGGGACGGAACGCGATTGGGGTCCGAAAAGTCGGTGA

CTGCACGTCAGTCGGGTGCGCTGGAGGGCACAGAGGGTGCGTGCTGGAGG

GCACGTCGGGGAGGTGTGCGTCAAGGGGACCGAACGTGAAGGGTGCGTCA

GGGGGGATTCGGCTGTCCCGCGAAGGGACAGGGAGCGAGTGTGTCGTGGG

GGGCTGGAGGGGTGAGCGCCGCCGCGGGCACGAGCAGTCGGGTGTACCAG

TCGAGCGTGCCGTGCCTCGCTCGCGGCGCGTGCGCCACCGGGTCGTCGAC

CGCGGTCGTTACGCCGAGTCCTCGGTCGTCGTCCCGACACCACCGGTCTC

GGCTGTCGTCTCGGTACCGCCGACCGCGGGTTCGCCGTCCGTGCCGTTCG

TACCGTTCACGGGACCATCACCACCGCCCGTGTCCGTACAGCCGGCGAGT

GCGACGGTCGCTACCGCGACGCCGACACCGAACTGCCGTCGAGTGAGCGT

TTCGCGCATCGCAGTCAGCAGTCTCGCTCGGCGGATGATAATGGGGGAGA

GTCGTCCCGACCGTTCCCCCTTTGAACAACCGTTTCGAGTGACTGTTTCG

ACCGTTTCAGTCGGATTAACGGAACGTTCGCCAGCCAACCGAGGGCCGAT

GAGGGCGGCCCAGTACGGCGATTGTCTCGTCGCTGCGGAGCGACCCGGCG

AGAGGCGACGGCGAGCGGTCGCGGACGGCGACGAACGACATCGGGGAGCG

ATAGACCCTCCTGTGAACCCCGGTGGCGACTGTCGATACCGACGCCCCGG

ACCGGGAGAAAATACAGTATTTGAAAATATTCAGACCAAATTGACTGTCC

GAACAGTTGTAAAAATGTTCAATAAACAGATAAACAAAACATTATACACT

AGCAGAGTCGACAGATAAAGAACAGACACATGCCAGTCAAACGCGTCTCG

GGGGTCGGATGGACGCTCGTCGCGGCGTTACTCGTCGTCGCGGTCGCCGT

CTCGCCGGTCGTCGTCGCCGCGGATGGCGTCGAAGTGCGTGCGGTCGACC

ACGGCGGCCCGGGCGTCGTCCCGACCGAGAACGGCCGGCCGTACGTCGCC

TCGTGGCAACCCTCGACGGTTTCGGTGACCGTCGCGGGAGACGGCAACGG

CACCGAGGTCTGCTTGCAGACCGACCGCGACGACGGCTCGACGATGCTGT

TGGGATGCGAACCGCTCGGAAGCGCGGGAGCGAACCCGACCGACGAACGG

CGCGTCGGCTTCGAGTTCGCAACGTGGCCGGCGAACGCGACCGGCGACCG

CACCGTGACAGCGGTCGTCAGACCCGGCGACGGCGGCGAGCCGGTCGCGC

AGGCGAGTCGCGGTGTCACGGTACTCGCGCCGGCCGGCGACGCCGACGGC

GACAACCTCGGCAACCGGGACGAACTCGACCGCGGCACGGACGTGCTCGT

CACCGACACCGACACCGACACCGACGGCGTACCCGACGGCGAGGAGGTCA

ACCGCTACGAGACCGACCCGACGAGCACGGACACCGACGGCGACGACCTC

AGCGACGGCGTCGAAATCAACGAGCAGGGGAGCAACTCGACCGAGACCGA

CACCGACGGCGACGGCCGAGGCGACGGTGCTGAGGTCGAAGCGGGGACTG

ACCCGAACGCCGCGCCGGGGGCGGTCGTCGGGTCGCTGGAACTCGGCGGC

GAGGGCTGGCTGCTCGTCCTCGCGGTCGCGGCCATCGCGGTCGCCCTGCT

CGTCGTCGGCGTGCGAGTCCGCGACAGCGACGCGCGAGCGAGGCTGTCCG

ACGTTCGCGCCCGCGCGGCCGACCATGTCGACGGCCGAGGCGACGGAGCG

TCGGCGGACGCGGTCCAGACTGGCGGAGGCGACAGTGCGGCGCGTGCGCA

GTCGGCCGCCAACAGTTCCCCCGCCGACGGCTCCCCCGCCGCCGAGGAAC

TCCTCGACGACGAGACGCGCGTCCTCAGGCTCCTCGACGACAACGGCGGC

CAACTCCGGCAGTCGAAAGTCGTCGAGGGAACGGAGTGGTCGAAATCGAA

GGTGAGTCGCGTCCTCTCGCGGATGGCCGACGAGGGAACCGTCGCGAAGA

TAAATCTCGGCCGAGAGAACCTCATCGCCCGCCCGGAGAGCGTTCCCGAA

CACGCCCGGTCGCCGTTCGACGAGTCCTGAGCGGGCGACGCGGCGGCCGG

GACGACCCGCCCCGAGAGTTGGAATCCCGAACCGCGGACGGAGTTGGAAC

ATACCAACCAAGAATATAGGCCTCGGGGAACTAGGGAAGACTCATGCGAG

AATTCGTCTTCACCGTCGAGTACGAGCGGGGTGCCGACGAGGTGATGGAC

CTGTTCATCGAGAACCCGGATTTACACTCCAAGACCATGGCGATACACGC

CACGAGCGAGTCGATGTGGCGACTCGACCGCCTCACCGGGCCGACGGAGG

CGCTCGAAGCGTTCGACGAGGTCATCGAGCAGGCCACCCGGTGCAACGGC

GTCCTCGGGATGTGCGGCGCGCCGGTCGTCGAGTGGGAGTACGAGGTGCT

GTCGGAGACGCCAAACGGGCGCATCGTCTACTCCTGCCGCGAGGAGGGCG

ACGGGATTCAGTCGATTCCCCACGTCGCCGCGAAGCACATCGGCGACGGC

CTCCTGATGCAGGCCGAACGCCGGGGGGCACAGAACCAGTGGCGACTGCT

CATCTCCGACGACGACACGGTGAGCGAGATTTACGAGGAGGTCAAAGACA

GCCTCAGCGACGGCCTCTCGCTGAGCGTCCAGCGCATCAGCGAACCCGAG

TGCTGGCTCGAAGAGAGCGTCTCCGCCGACGGCCTCCCGCCGGAACAGCA

GGCCGCGCTCGAAGCCGCCGTCGAGTTCGGCTACTACGAGACGCCGCGCC

AGCACACCGTCCAAGAAATCTCGGAGGAACTCGACATCCCGAACTCGACG

CTCCAGTATCGCCTGACCCGCGCCGAGGCGTGGCTGGCCCGGCAGTTCGT

CACCGGCACGCTCGGCACCGAAGTCGAAGACCGCGTCGACCCCGAACAGC

TCGAAGCGAGCGCCTGACCGGCGACTCCCGGGCCGGACGCTCGACGAGAG

ACGGCCGCAGAACGGTTGGAACATTCCAACCACACCCTGATACGCCGCGG

ACGCGTACGTAGATGCGTGGGAAAAGACACTTTCAGCGACATCACCGGCC

TGCCGGACGACCTCGGAATCGGCGACGACCTCGCGCGGGCGGACCAGCGC

GCGTCGATTCGCGTCGACACCCGACGGTACGGCAAGCCCGTGACGGTGGT

CGACGGTCTCGACCTCCCGGCCGACGAACTGGACGCCCTCGCGTCGACGT

TGAAGCGACGGCTCGCGGTCGGCGGGACCGTGACCGACGGGCGCATCGAA

CTGCAGGGCGAACACGGCGAGCGCCTCGAAGCCGCGCTCCGCGACGAGGG

ATTCGGCGTCGAGCGCTGAGCGTGTTATCGTGATTCGATAGCTCTCGCGG

CCGAAAAGGGCGGATAACTGACGCGGCAACCGACGTGCGAGCGGTCGATG

TCTCCCGGTCGCGTATTCCGGGAACGAACACAGTTGGTATATTCCAAACG

AGATCCCATTACCTTGGGTCCGGAATACAGAGGTACTCAATGGACACGAA

ACACGTCGACTGGCGTTCGATGCGAGACGAGTCGGCACCGGCGGCGGGAC

CCGAAGCCCGACAGTCCGGCGACGAGCGCGACGAGGGCGAGGCCGAGACC

GAATCGGCCGAGGCGGCGGTCGCGGAGCGGACCGACCTCGTCTACGACGA

GCAGCACGGCGAGTGGGTCGACCCCGAGACGGGCGAAATCCTCCGCGAGG

ACGAAATCGACCGCGGCCCCGAGTGGCGCGCGTTCGACGCCGCGGAGCGC

GACCAGAAATCCCGCGTCGGGTCGCCGACGACGACGATGATGCACGACAA

GGGGCTGTCGACTAACATCGGCTGGCAGAACAAAGACGCCTACGGCAACT

CCCTGTCGACGGGACAGCGCCAGAAGATGCAGCGCCTCCGGACGTGGAAC

GAGCGGTTCCGCACCCGCGACTCCAAAGAGCGGAACCTCAAGCAGGCGCT

CGGTGAGGTCGAGCGGATGGGCTCGGCGCTCGGGCTTCCGGACACCGTCC

GCGAGACCGCCTCGGTCATCTACCGCCGCGCGCTCGACGACGACCTGCTG

CCCGGCCGCTCCATAGAGGGCGTCGCCACCGCCGCAATCTACGCCGCCGC

TCGGCAGGCCGGCGTCCCGCGCTCCCTCGACGAGGTGCGGCGCGTCTCCC

GCGTCGACAAGATGGAACTCACCCGGACGTACCGCTACGTCTCCCGCGAA

CTGGGGCTGGACATGAAGCCGGCCGACCCGGCGCAGTACCTCCCGCGGTT

CGTCTCCGAACTCGACGTGAGCGACGACGTGGAGCGCCGCGCTCGGTCGC

TTCTCGACAACGCGAAGCGGCAGGGCATCCACAGCGGCAAGAGCCCGGTC

GGACTCGCGGCGGCGGCCATCTACGCCGGCGCGCTCCTCGCCGACGAGGA

ACTGACGCAGTCCGAGGTGAGCGACGTGACCGACATCAGCGAAGTGACCA

TCAGAAACCGCTACCGCGAACTCCTCGAAGCGACCCAGAAATCGGGCGAA

AGGGCCGTCGGGTCGACCGCCTAAGCGGACGTTTCGAGCGCGAGAAGACG

GTTCTTTCTTCGATTCGCGTCGTTTTCAGACGCCTCGTTCGGACTGTTCG

GCCCGTTCGACCCATTCAGACTGTGCGGGTTCGACCGCGGGCGACGCGAC

GCCGTTCTCACGCCGTCAGCGGTTGCACGAACACGACGACGACGCGCGAG

TCGAACTGCCGCGACCCGGCAGCGCGTCGCGGACGCGGTCAGCGAGTGCG

CCCGCGGTTTCGCGGACTCTCCCCTCGGCGCTCGATTCGGCGCGTCGTTC

CCGGTAGTCGAAGTAGAGCGCCGTCGGCAGGAGAAGCGAGGCGTACACGC

CGCTGATAGCGACGGCAATCTGCATCGGATGGTCTGCGGGGATGACCGCC

CAGCCGACCGCGATAATCGCCCACAGCGCGATTCCCTGCCGGATGGTCGG

ACACCATCGATTCGTGCGCGCCGACCGCGCCTGCACGTCGGCGGCGTCCA

TGTCGGCGTCATCGCCCGATTCGGACGCCGGGCGGTCCGTTCGATGGGAC

TCGGCTCGCGCATCGTCGGATAGCGTAGAGGCGTGACGGAACGTTCGTTC

GGACATAGAATGGTATCGGCGGCTACGGTTCCGAGCCTAATGTGGTCTCG

TTTGGAATATACCAACCACGAGGGCAGAGCCGGCGTTCGGACCCGCGGGT

CAGACGCTCGCGCCGTCCACCGGTTCCGCGAACGCGACGGTCTGTTTCAC

GTCGGTGACTTCGACCTCGACGCGGTCGCCGGGTTCCGTGCCGGGGACGA

TGATGACGAAGCCGCGTTCGACCTTCGCGATGCCGTCGCCTTGGTCACCG

AGCGTGTCGATGATGACGGACCGAACGTCGCCCTCCGCGACGGGCGGTTG

TCGCGGCTGAGAGTGCGCTTCGCGGGGTCCAGACGCGGTGTCGGCGGCGG

ATTCCCGAGCGGGGTCCGAATCGATGTCGCGGTCGTCAGCGGTCGTCGTC

GCCGTCGTCTCCGTCGTCGTCGCCGCCGCCGATTCGACAGCGGCCTCGGT

CGCCGACGCGGCGTGCGGACTCGAAAGCAGCGCGACGCGGTACAGTTCCT

CCGTCGAAATCGACCCGTTTTCGACGAGTTCGGTCGGAATCGACACCACG

TAACGGTCGCCGTCTCGCTCGACCGACGTCTCGAAAAGGAGCCGAAGAGA

ATCTGAGATATCAGTCACTACCGCTACTTCGACTCAAACAAAATAAAAGG

TGGTGAAAGTGACTACCGTTTCGGTCGCCGAGACACGATAGAATCCCCGG

AAATCCGATTCGACCCGACCGGAATCATTCCCGAAGACACATGTCAACCC

AATGCTAACTATCGGGCATGGCACGCGACACACTCGTTTCGTGGCAGACG

TTCGCGCTGTTCGTCCTGTTTCCGCCGGCGGCGCTCGTCGCCCTCGTGTT

CTTTCCCCTGACGCTGCTCGTCTTCGGGTGGCTGTACTACCGCGGCAAGT

ACGAGTCGATACAGGAGTCCGACGACGAGCAGACGGCCGTGGAGCCGAGC

GACGCCGCTCGGTGAGTGACGCATCCGCGGGCGCGAACGTGGCCGGGCGG

CCCCCCGAAGGGTCGGCCGTCCCACACACGGGCGAGCATTTCTCGTCGGG

AAGACGGATGTCGACGAGACAACGGATTTTTGAGTTCGTCAACTGAGTGA

TTGAGTATGGTTTACGAGACTGGGAACCCGACTGTCGACGACGCGGTGTG

GCGGGTGCTCGACGGCGACGAACTCGACCGGACCGACGGGATCGCGCTCA

TCGCGCAGCCGGTCGAGCCCCTCGCGGCGGGTGCCGACTACGTCCGCTCC

GTCCGCTCCGACGGGACCGTCGACGCCTGTTCCATCGTCAACGCGAAGGC

CGGCGACTGCGCCGAGGACTGCGGCTTCTGCGCGCAGTCGGCGCACTTCG

ACACCGGCATCGAGACGCACGGCTTTCTCGGGGCGGAAGCGGTGTTAGAC

GCCGCGCGGCGCGCCGAACGCGACGGCGCACAGCGGTTCGGCATCGTCGT

CGCCGAGAAGGGCGTCTCGAAGGAGCGCCGACCCGACGAGTGGGCGGACG

TGCTGGAGGCGATTCGACTCGTCCGCGACGAGACGAGCGTCGAAATCGAC

GCCTCGCTCGGCCTGCTCACCGAGGAGGAGGCGCGAATCCTCGCCGACGA

GGGCGTGAACCACTACAACCACAACATCGAGACCTCGCCGCGGTACTTCG

ACGAGGTGGTCGGAACCCACGACTTCGAGGACCGACTCGTGACGCTCCGG

CGGGCGAAAGACGCCGGGATGGACCTCTGTGCCGGTGTCATCCTCGGGAT

GGGCGAGACGCCGGCGGACCGGGTCGACGCCGCTCTCGAACTCCAGAAAA

TCGGCGTCGAGTCGCTCCCGGTGAACGTGCTCAACCCCGTCCCCGGCACG

CCGCTCGGCGACGCCGACCACGCCGACATCACGACGACGGAACTCGTCAA

GACCGTCGCAGTCTACCGACTGCTCCACCCCGACGCGCGCGTCCGTTTGA

CCGGCGGGCGGGAAGTGAATCTCGCGCCCGACGAGCAACACCTGCCGTTC

GAGGCCGGCGCGGACGGCGTCCTCACCGGCGACTACCTGACGACCGACGG

CCAGTCGCCGGCGGCCGACATCGAGGTCGTCGAGCGGGCGGGCCTCGAAC

CGAACCGCGCGGTCAACGACTTCGACGTGGACGCCGTCAAGCGCCGAGAA

GCGAGAGACGGCTGAGGGGCTGACGAACCGCCGAGCCGTCGAACAGCCGA

ACCGCCGAACCACCGAACCGCCGAACCACCGAACCGCCGAACCACCGAAC

CGCCGAACCACCGAACCGCCGCCCGAGACGCGCCACATCACCCGAGCGCG

CTGTGCCGGGACATGAGCGAGATGACCTGCCGGTGGAGCGCCTGATTGGA

CGCGACGACGGTGTCGCCGGTCACGTCGTCTATCGCCTCGACGTGCGTCA

CCTCGCCGCCGGCCTCGCGGACGAGAAGCGGGCCGGTCCTCGTGTCCCAC

ATATCGAGGTTCTGCTCGAAGTACACGTCGAAGCGGCCGGCGGCGACGAG

CGCCAACTCCGCGGCGGCCGACCCCATCCGGCGAACGCCTCGCGTCGACT

CGCAGAGCGCTTCGAGGTACGCGCCGTCGCCGTTCGCTCGGGCCAGCGAG

TCGAACCCGGTCACGACGAGCGCCTCAGACAGCTCGGCGGCGTTCGAGAC

CGAGAGTTCCCGCCCGTTGACGAACGCGCCTTCGCCCTCGATTGCGACGT

ACAACTGGTCCGCCGGGACGTAGTTGACGACGCCGACGTCGGGCCGCGTC

GCACCCTCGTACATGATAGAGACAGAGAAGTGCGGCACGCCGTTGTGGAA

GTTGACCGTGCCGTCGAGCGGGTCGACGACCCAGCGCGGCTTCGGGCGGA

ACTCCGGGGAACCCTCCTCGGAGACGACGTTGTCGTCCGGGAAGGCGGCG

GCTATCACGTCGGTGATTCGCGCTTCGGCGGCTTCGTCCGCCGGGCGGTC

GCGGAACCGCTCGCGCGAACTCGACTCGAACCGGGCCCGTAACTCGTCGC

CGGCGACCTCGGCCGCCCGAATCGCGGTCCACAGGTAGCGGTCGTTCACC

ATGCACCGACGTGGCCGGGGCTCGTATATTGTGACTGCTATGTTGTCTCA

ATAGTAATCTGTTCAGTACGTTGCCATCATAGTACGGTTGATAAGTAGTT

GTCGATGACGTATCGTATGCTCACTATCGGGCATCGAGGCTGTGCGGGCC

AGTATCCAGAGAACACGATTCCCGCAATCGAGGGGTCCGCGCCCCACGTC

GACCTCGTCGAGGTGGACGTGATGCGCTGTGGCTCCGGGGAGCTGGTGGC

GTTTCACGACGACGACCTGTCGCGGCTGACGACGGCCGCGGGTCGACTCG

ACGAAACCGCCGTCGACGACCTCACGGCGCTTCGCGTCGACGGCTCGGAG

GCGACGATTCCGCGGTTCGACCAGGTGGTCGAGGCGTGGCCCGCGGGCAC

CGGCATGAATCTCGACACCCGCGACCCGGGCCTCGTCGAAGACGCCTTAG

CGGCCGTCGAGGGACTCGACGAGCGGGTCGTCTTCTCGTCGCCGTCGCGC

GAAGCGGTCGATATCGCGCTGGAGACGCCGACGCCGGCCGAGGTCGGCTA

CTCGTTCCGCGACGACGTGTCCGCGGAGTTGACGTGGGCGGCAGAACGAG

GCTGTGAGTTCGTCCACGTCGAACACGGCCTGTGTACCTCCTCGGACCTC

GTGGAGCGCGCGCACGACGCCGGCCTGCTCGTCGACGCGTGGACCGTCGA

CGACGAAGCGACCGCGACGACCCTCCGGTCGCTCGGCGTGGACGCCCTCA

CCGTCGACCGATGGGATGTCGTCGCCCCGGAGGCGTGCTCGTGAACCCGA

CTCGACGCCCGAGGCCGTCGACCGCCTTTTTATACCCCAATCATAGCGGT

ACGAGCCACACCAGAGAACAACATATGTCTAATTATACTCCAAATAATAG

GAAAATTTAAATTATGTGTTCGGATATGTCCCAGTACATGCCTGAAAATG

GCCCTATCGGCGAGCGAGCCGAAACGACGAACCGAGCTATCGGACGCACA

ACCAGTCGGCGCGGATTCCTCGCGACGGCGGGGGCCGTCGGCGTCGCGGG

ACTCGCGGGCTGTTCCGGCGGCGGGGGCGACTCACCGACCGGGACCGAGG

GCGGAAGCGGGAGCACCGAACAGTCGGGAACGACGGCGGGGACATCCGGC

GCGCAGGACGTGACCGTCTCGTTTTGGCACATCTTCGGCGGCGAACTCGG

CCAGACGCTGGGAGACATGGCCGCCGAGTTCTCACAGCAGAACGACGGAA

TCACCATCGAGGCGGTCAACAACGGCGGCTACCGGCAGAACCTCAACCAG

TCGTTGCAGGCGTCGCGGGCCGGCGATCCGCCGGGTATCGTCCAGATATT

CGAGGTCGGGACGCGACTCGCGCTCGACAGCGGCTCGTTCACGCCGGTCG

AGGAGATTCTGCCGGAAGAGGAAATCGACTTCGACGACTTCCTGCCGTCA

GTCTTGAACTACTACCGCATGGACGGGACGCTGAACTCGATGCCGTTCAA

CTCCTCGAACACCATCATGCTCTACAACAAGACCGCGTTCGAGGAGGCGG

GCCTCGACCCCGAAACCCCGCCGCGGAGCCTCGCGGAGGTCCGGCAGGCC

GCACAGACCATCGTCGACCAGACCGACATGGAGAGCGGAATCAGCTGGCC

GAACCACACCTGGATGCAGGTCGAACAGCAGTTCGCCAAACAGGACCAAG

TGCTCGTGAACCAAGAGAACGGCCGCGACGGTCGACCCGACCAGACGTTC

TTCAACTCCGAGGCGGGCCGCAGCGTCTACTCCTGGTGGAAGGGGATGGC

CCAAGACGGCCTCTATCTCAACCCCGGCATCGAGGCGTGGGGCGAAGCGC

GACAGGCGTTCCTCACCCAGAAGGTGCCGATGCTGTGGGACTCCACGTCG

AACATCGTCTCGATGCAGTCCGGCGCGAAGGAGAACGGCTTCGAACTCGG

CTCCGGCTACCTGCCGACGCCCGACGGCGCGAACACCGGCGTCGTCATCG

GCGGGGGGTCGCTGTGGGTTCCCGACGCCCTCTCCGACGAGAAAAAGCAG

GCCGCGGGAGAGTTCATCGCCTACGTCACCCAGACCGAGCAGCAGGCGCG

CTGGCACCGCAACAGCGGCTACTTCCCGGTCCGACAGAGCTCCATCGACC

AGCTCACGGACGACGGCTGGTTCGACAACAACCCCAACTTCAGCACGGCG

TTCGACCAGCTACAAGACACCGAAGACACGCCCGCGACTCGCGGCGCGGT

GATGGGCGTGTTCCCCAAGACCCGGTCGATTAACGAGGAGATATCGGTCA

GCATCATCAACGACCAACTCGGCGTCGAGGAGGGGCTGTCGCGGATGGAC

ACGCAGGTCGGCGAGGCGCTCGCCGGCTACAACGGCAACTTCGACGGGAG

CGAGTAGGCTCCCCCGACACGTTATTTACATGTCTACTGCCACAAAACCG

TACGATTCGACGCTTCAGGCGGCGCTGTTGTTGCTCCCGACAGCTGCGGT

ACTCGTCGCGTTTCTGTACTACCCCGCGCTCGAAACGTTCCGCCTCAGTC

TCTACGAGACGCTGTTTCTCGGACAGCGACAGACGTGGACCGGGCTCGGG

AATTTCGTCACCCTGCTCACGTCGGGCACCTACCAGTACAGCTTCTTCAT

CACGGTCGCGTTCGCGGCGGTCGTCGTCTTCGGGACGCTCGGCGTCTCGC

TGCTGATCGGCTACCTGATATTCTCCGTCGACGTTCGGTCGTCCGTCTAT

CTCGTCGCCGCGATTTGGCCGTACGCGCTCCCGCCGGCGGTCGCCGCGAT

TCTCCTGAACTTCATTCTCCACCCGAATCTCGGCATCTTCACCCACTACC

TCGAACTCCTGACGCCGCTCACCTTGGACTGGTTCAACGACGGCCCGCAG

GCGTTCGCCATCCTCGCGGTCGTCGCCGTCTGGAAACAGCTCGGCTACAA

CATCATCTTCATCGTCGCCGCGCTGAACAACATCCCCGAGATACTGACGG

AGAACGCGCGCATCGACGGCGTCGGCCGGTTCAGGATGCTGTACAAGGTG

TACGTGCCGATGATTGCGCCGACGATGACGTTCCTCGTCGTCATGAACAC

GATATACGCGTTCTTCTCGACGTTCCCGCTCGTCGACCTGATGACGAGCG

GCGGGCCGAGCGACGCAACGAACTTCCTGATATTCAAGCTCTACCGCGAC

GCCTTCCAGTTCAACAGCCTCGGGCTGGCGTCCGCGCAGTCCGTGATTCT

GTTCGTCGTCGTGTCGGTTCTGATGTACATCCAGCTTCGGTTCGCGTCGG

GCTACGGGCGGTACGGAGGATAACGAGATGGCCACAGAGACAGACACCAC

GACGTTCGACGCGGGAAGACTCGCGCACCGACTGCCGGACGACGCGCTGC

TGCACGGCGGCATCGTCTTCGCAATCCTCCTGATGGCGTCGCCGCTGCTG

CTCGCGATTATCATGAGCACGCAGTCGACGACGGAGGTGTATCAGGTGAC

GAACCTCGGCCTCGGGTCGAGGGGGCTGTCGAACTACTCGGACGCGCTCG

TGAACTACGACTTCAGCACGTACATGCTGAACTCGTTCGTGATGAGCGTC

GTCGTCGTCGTCGGGAAGGTCACGCTCTCGCTGTTCGCGGCGCTCGCGCT

CGTCTACTACCGCTTCCCGTACGAGCGGGCGGTGTTCATGTTCATCCTGC

TGACGCTGTTGTTGCCGGTGCCGGTTCGAATCGTCCCGCTGTTCCAGCTG

ATGGCCGACCTCGGCTGGACGAACTCCCTGTTGGCGTTGACCGGACCCTA

CATCGCGAGCGCGACGGCCGTGTTCCTCTTTCGCCAGCAGTTCACGGGGA

TTCCGGCCTCGCTCGTCGAGGTCGCGCGCCTCGACGGCGTCGGCCCGCTC

ACCTTCCTGTTCAGGGTGCTCGTCCCGATGTCGAGAGGGATGATTGCCGG

GGTGTGCGTTATCACGTTCATCTACACGTGGAACCAGTTCCTCTGGCCGC

TGGTCGTCGTGACCGACAAGAGCAGTCAGGTCGTCCAAGTGGGGATTCGG

TACCTCCAGGGCTCCGCGCAGGCGGGGCTGACGCAGTGGGGGCTCATCAT

GGCCGGCGCGGTCCTCGCGTTGCTCCCGCCGCTCGTGGTGCTCGTCGTGC

TGCACCGACCGCTCCTGCGAACGCTCACCATCCAACAGAAATAGCGAACC

CACCATGTCAGACATCACGATTCAGAACTTACGGAAGACGTACGACGACG

TGCGCGCCGTGAAGGGTATCGACCTCGAAATCGAAGACGGCGAGTTCCTC

GTCGTCGTCGGCCCCTCGGGGTGCGGGAAATCGACGACGCTCCGGATGCT

CGCCGGCCTCGAATCGGTGACGGACGGCTCCATCGCCATCGGCGACCGCG

TCGTCAACGAGGTGCCGCCGAAGGACCGAAGCATCGCGATGGTGTTTCAG

AACTACGCGCTGTACCCGCACATGACGGCGGCAGAGAACATGAAGTTCGG

CATGAAATCGGCCAGCGAGTTCTCCGCCGACGAAATCGAACGGCGCGTGG

AAGACGCCACCGAGACGCTCGGTATCGCACACCTCCGCGACCGGAAGCCG

AAGGAACTCTCGGGCGGGGAGCGACAGCGGGTCGCCATCGGCCGGGCGCT

CGTCCGCGAACCGGACGTGTTCCTGATGGACGAGCCGCTGTCCAACCTCG

ACGCGAAGCTCCGGGTGCAGATGCGCGCCGAACTGCTCAAACTCCACCGC

GACCTCGATACGACGACCGTCTACGTCACCCACGACCAGACGGAGGCGAT

GACGCTCGGCGACAGGGTCGCGGTGCTCGAAGACGGGAGGCTCCAGCAGG

TCGCACCGCCCCAAGAGCTGTACGACTACCCGACGAACCAGTTCGTCGCC

GGCTTCGTCGGCGAGCCCGCGATGAACTTCGTTCCCGTGGCGGTCCGCCG

GCAGAGGAGCGAACTCGTCGCCGAAGCGCCGGGCCTCTCGCTCACGCTCC

CTGCGGGCTCCGGGCTGGACGACGTGGACGACGACGGCCTCACCCTCGGC

GTCAGACCCGAAGACGTGTCTCTCGTCTCGAACACCGCCGGGGCGTCGGA

GTCGTTCACCGCCGAGGTGACGGTCACCGAACCGCTCGGCGAACTCCTGC

TTCTGCACTGTCGCCTCGGCGACGAGGAGATTCGGGTCAAAGTCGAGCCG

CGAAGCCGAATCACGGCCGGAGACACCGTCGAACTCGCCGTCGATACGGA

CCGCCTGCACCTGTTCGATGCGGACGGCGACGCCGTCTACCACTCGTCGG

TTCCCGAGCGGGCGACCGAGAGCGTCGTAGCCGACTGACGAGGCAGGCGG

CGAGGCGAGCGGCGAAGCGAACGCAGGGCGCGTCGCTACCGCCCGTCGAA

CGCCTCGGGCGACAGGTCGAGTCCCGCGCGCTCCTCGTCGGTGAGGTCTT

CGAGGACGCTCGTGCCGGACCCCGGTTTCGAGTCCCACGACCAGTCCTCG

TGAAGGCGGAGGCGGCCGTCTTCCAGTCGCTCGATGCGGTCCGTCGAGTG

GCCGGTCGCAGTCGTCCCGTCGTCGGTGACGTGAGAGTACCGGAAGTCGA

GCGTGTCGCCGCGGTGGCGGCCGACGAGGTGGCCCAGCCTGACGGTTCCG

CCGCGGTACGCCGCGTGAATCAGGTCGCCGGACTGGTCGAACCAGAAGTG

GGTGTCGCCGCCGACGTCGCCGCTCTCGGCGTTGGCGACCGATTTGAAGA

CGCGCCCGTCGAGGGACGGCGCGTGAGATTCGGACATAGTCCGTGTGCGA

GGCGGACCGTTATGAGTGGTCCGCCGAGAACTGCGAGCGGCCGACTTCCC

CCGGTCGACCGCCCGTCGCGGCCCCTGCACCACCACTACCACCAGCGGTC

AGTAGCTACTCCGATCTCCCGAACCGGAGTCAGAGCCGTTTTTGACCGCG

TCAGTCGCCTTTTCGATGCCTTTCTTGGCTTTCTCCTCGCTCACCTTCGT

CCGCTTGCCGACCTGTTTGGCGACTTCGTCTTCGTGGCCCTCTACCAGTC

CGAGCAGCCGTCGAAGCTTGCTCATCACGCCCATGGCGTCGGCAGTAGCG

ACCCCTCGGAGGAAAAGGTGGTGGCCCCGAACCGCGGAATCGACACCCGA

GAGAGACGGCGGCCGGCTTCGGGTAGACGGTGAATCAACAGTCGAAAGAA

CGGCATATCTATAAATCCAATATATAATAAAATACTGAGAGGTGTGCGAG

CAGTCAATCGTCTTTCTCTACGGTAGGAGAGGAGTGAACGGGTCGGTCGA

TACGGCCCGTTCGAAGGAACCAACAGACGAAAAAAGGAACTAGACGCAGT

CTTCAGGCCCGCGAGGGTCGAGCTTTCGATACGTGCCGTTCGGAGCGACT

CTCGCGTCGTCAGCACGAACGACGGCCTCGCGTGATGTTCGAGTCGGTAG

CGGCAGTACGGTCGCTTTCGTCGGGTACAGTGATTGAGCGTCGGTTGTAC

TCTGGTAGCAAGGTCTCGAAACCGGCGAGTATCCGACGGCGGGTTCCGAG

ACGAGTGGGTCCATCGTCCAGTCCCGAGATTGAATGAAATATGGTATTTT

TTCGATACTCCCGAATTTCTGTTAGTTCGTTCTGGCGTTCCTGTCGGGGC

GACGAGCCATGGTCCCGATCGGCGTCTCAGCTTCCCCGGTAGGTCGTGTA

GCTCCCCGGCGACAGCAACAGGGGAACGTGATAGTGCTCGTTGGCGTCGT

CGACGACGAACCGGACCGGAACCGTCTCCAAGAACGACGATTCCGTGGGG

CCGTCGCGGTAGTACGGGCCGACTTCGAACAGGAGTTGGTAGGTTCCGGT

CTCCATCGCGTCGCCCGACAAAAGCGGTTCGTCGACCCGTCCGTCGTCGT

TGGTCGTGCCGCGGCCGACCGTCTCCGCCTCGCCGGACGAATCCAGTTTT

TGAAGCGTCACGTCGACGCCGGCCGCCGGTCCCTCGCTGCTGGTGTCGAG

GACGTGCGTGGTTACTCCTTCGCTCATCTTACTGCTCGTCGAGGTCGCCG

CGGTCCATCGAGAACTGCTGGAAGCCGGTCGGCGCGGGCGGCTCCCGCAG

GACGCTGGCGTCGCCGTCGAGGTCGTCGCGGACGCTGAGCCAGGTGCGGT

TGTTCGCCTCGAACCGAACGGACGCGAGCTGCGGATAGCGTTCGAGGACG

CGCAGGCCGATTTGGTAGATGAGGTCCTGAATCGAGTTCGAGTCGACCTC

GTCGAAGACGACGTGGGCGATGTCGCGGACCTGCTCGGACGGCACGTACC

GTTCGGGGTCCTCGCCGAGCGCGTCTTCGGGGTCGTCGTAGCTCCAGAAG

ATATCGAGCGAGATGTAGAGCGTTCGGTCCTCGCGCTCCGGGAGCGTCGT

GTACTCGTCTTGGACGTAGCCGGTGAACGAACTCCCCTCGACCTTGACCA

GTTCGAGGCCGGTGACGCCGCTCGTCTGGTCGGTGATGACGGGTGTGCCG

TCGTCTCGCGTCAGCGAGATGGAGCCGTAGCCGGACTCGTTGTCGGAGAC

GCGGAAGACGAGGTCGCTCGCCTCGTACCCGTCGCCGTCGTCCTCTGGGA

CGGGCAGTTCGTCGAATCGAATCTCGTCGGCCGACACCTCGACGGCGCTC

ATCTGCGAGTAGGTGTCCAAGAACCCCGACCCGACGAACTCCAAGAAGCC

TTCGAGGGTGGCCCCGTCGTACTCGCCGGCGTGGTGGAGGATGAAGTTCT

TCATCGAGTCCGTCGCCACGACCTTCGTGTTGTCGCCCTCGCTGAACGAC

GGCAGGAACTCCTCGCCTTCGACCTGCACGCGAACGTCGAGGCCGAACAG

CACGTTGTCCCGCCCGTCGAACGACGACTCCGGAATCGTGCGAACGCCCT

CCAGCGGGGTCGCGTACGTCCGGTAGACGGCGACGTTCTCCTTCCCGTAG

TTCATCGTCCGCTGTTCGCCGCTGTCCGGGGCGTCACCGTCCACCGGTGA

CTGTTGCGCTGTCATAATTCGTCTATGAAAGGTGCATCGTTAAACAAAAA

TCTTTGCGTTCGCGTGACATACGCGACTCGTTTCGGCTTTGATTCAGACA

GACAGTCGGTTCCTATTCTGACGAGAACCGCTCCGCCAACCGGAGTTCCG

CAATCGTGTGGACCTCGTCCAGCGCGGTCCGAAACTCCGTCGACTCCGAG

TGGTCGACCCGGCGTTCCATCGCCGCGGCAATCGCGTCGGGGTTCTCGTC

TTTCACCGCCATGACGAACGGGAAGCCGAACCGCTCGCGGTACGTCTCGT

TCAGTCGTTGGAACGTCTCGTACTGGCTCCGCGAGAGCGAGTCCAGCTCG

GCCGACGCCTGTTCCGCCTCCGAGGCGTCGGTCATCTCGGTCCGCTCTCC

GAGGTCGGGGTGCGCCCGGAGCAACTGGAGTTGCTTCTCGCGCGACGCGT

CCTCGACGGCGCGCTTCATGGCCGACCGGAGTTCGTCCACGGACGAAAAC

GGTCGCGACGACCGCGCCCGCTCGGCGACCCACGGCGAGTGTTCGTATAT

CTCCCCGAACGCGTCCACGAACGAGTCGTCGTCGAGGCGGTTCACCTGCT

GGAGCGTGAGTTCGTGCATCGTTGTTTGGCGTGCGTGACCTCGACACTTC

AACCTACTCGTGAACCTCCCTCCTTCCGCGTCGAATCGGCGGGGGGCCCG

ACTCACGCGCTCCAATCGGGGCGCTCGCGCTCCACGAACCGGCCGTGGCC

GGGGTCGCCGACGACCTCGCCGTCGGCCGCGACGACCTCGCCGCGGACGA

GCGTCTTCGTCACCCGGCCCGTGACTTCCCGGCCCTCGTAAATCGAGTAG

TCCGCCTTCGAGGCGTTCGCGTCGGCGGAAATCGTGTACGTCTCGTCGGG

GTCGAACAGGACGATATCGGCGTCGGTTCCGGGTTCGAGCGTGCCCTTGT

TCGGGAGGCCGAACGTCTCGGCCGGGTTCGAACACATCACGCGGACGAGG

AACGGGTACGAGAGCCCGCGCTCGTTGACCGCCTCGTCGTGGAACACCGG

GAGGCTGACCTGCACGCCGTTCGCGCCGAACGGGCCCTCCCACCACGGCC

GGTCGACCTTGTCGGCGCGCTTCGAGGCGACGTGGTCCGTCGAGACGACG

CTCAGCACGCCGCGGCGGAGGTATTCGAACATCGCCTCCACGTCGTCTTC

CTTGCGGATGGGCGGCGCGATTTTCGGCAGGTTCCCCAGTTCCGCGTAGA

CGGAGTCGTCGAGCGTCGTGTAGTGCGTGCAGGTCTCCGCCCTGACCCGC

GACCCGTCCTCGCGGTGGCGGGCGAGCGCGTCGGCGGCCGCCCGGCACGT

CGTGTGGATGCCGTAGTATTTCGCCCCGGCCTCGGTCGCGAGCGTGACCG

CGTCGGACGCGGCCATCGCCTCGGCGTAGTCGGGGCGAGACTGCGGGTAC

GTCTCGGGGGCGTCCTCGCCAGCGGCCCGGAGTTCGGCGGTGAGCGCCCG

GCAGACCGAGTCGTCTTCGCTGTGGACCACGCCGACCGCGTCGTGTTCGG

CGAGGCGGTCGAACACCCGCTTGAGAAAGCCGTTCGAGACGCCGAACTCG

TAGGCGGTGTACATCTTGAACGAGGTCACGCCGTCGTCGACGAGCACCGG

AATCTCCTCGAAGACCGCGTCGTCCTCCCTGAGAATCCCGCCGTGTAATC

CGAAGTCGACGAGCGACTGTTCCTGTTGGTCGCGTTTGCGTTCGAGGCCC

TCCGAAAGGGTCGCCGGCTCGTCCCACGGACTGTCCGCGCCCTCGTAGGC

CTGCCACGCGAAGTCGATGAACGTGGTGACCCCGCCGAGCGCCGCCGCGC

TGGACACGGTTCGGTAGGTGTCGATGGAGACGTGGTCGTCGACGTGGATG

TGCGGGTCGACGACGCCGGGCATGACGACCTGTCCAGTCGCGTCGATTCG

GTTCCGTCCAGCGGGGAGGGCGTCCTCGTCGCCGACGCCGACGATGCGAC

CCTCGTCGACCGCGACCGCGGCGTCGAGCGTCCGCGAGTCGGTGACGACG

GTCCCGCCGGCGAGTATCGTGTCAACAGTCATCACGACACACTCCTCGGT

GGAGCGAATTATACGTACCGGTAGACCGCGGGTCGAACTGGGGCGAGAAA

GCAGCAGACGGGGCGAACTCAGTCGTCGACCGGCGCGGAGGGCGACGACT

CAGAACTCGACAGTTCGGTCAGGTCTTCCGGGTCGAGGCCGTCGGTGTGC

TCGGTCGGGCCGAGGCCGACGCCGCCGCCGGGGAGCACGACGTTGAGCGC

GAGCGCGGTGAAACCGCCGGTGACGAGCGCGGAGCCGAACAACGTCTGCA

CCTCGGCAGGGAAGTTCTGGAGCACCTCGGGGCGGAACGCCACGCCGAGG

CCGAACGCCATCGACAGCGCGAGAATCGTCGAGTTCCGGTGGTCGAGCGT

GACGTTCTGCGTGATGATGCGCGCCCCCGAGGAGAAGATCATCGCGAAGA

GGATGAGCGCGCCGCCGCCGAGGACGGCGTCGGGCATCACGGAGACGACC

GCGCCGACCTTCGGGACGAAGCCGAGCACGACGAGGACGACCCCGCCGAT

GCCGACGACGTATCGGCTCGCGACGCCGGTGAAGCTCACGAGGCCGACGT

TCTGCGAGAAAGACGTGTTGGGAAGCGCGTTGAACAGCGCGGCGAACGCG

CTCATCACGGCGTCGGCGACGAGGCCGCCGCGGAACTCCTTGCGGGTCGG

GTTCCGGCCGGTCGCCGACACCGTGCCGGAGATGTCGCCGATGGTTTCGA

GGCCGGTGATGACGTAGAGGAACGCGACCGTGAGCACGGCACTGGGCGGG

AATTCGAGGCCGAATCTGAGCGGCGTCGGCACGGTTATCCACCCCGCGGT

CGCGACGGCGGTGAAGTCGACCATCCCGAGCGCGATGGCGGCGACGTAGC

CGACGACGATGCCGACGAAGACGCTGATGACGCGGAGGAAGCCGTCGAAG

AACTGGTTGAGAACGACCGTGACGACCAAGACGAGCCCCGCGAGGCCGAG

GTTCGCAAACGAGCCGTAGCCGGCGGCCGAGGGGCCGGCGGACGCGCCCG

CGGCGTAGTTCATCCCCGTCGGGATGAGCGTCAGCCCGATGAGCATCACG

ACGATGCCCGTGACGAGCGGTGGGAAGTACTTCTGGAAGCGGTCGAGCGT

GACGCCCATCACCAACTCGACGGGCGCGGCGAGAAGTGACGTGCCGAAAA

CCGCGGCGATGCCGAACTGTTGGCCGATACCGATGAGCGGGCCGAGGAAC

GCGAAGCTCGTTCCCATGACGATTGGGAGTCGCGCGCCGACGGGACCGAT

TGGGAAGGCCTGCACGAGCGTCGCCGCGCCGGCGACGATGAGCGCCATCT

GCACGAGAAACGTCGTCTGGCCCGACACCGAACCGACGGCGCCCGCGAGG

ATGAGCGGCGGCGCGACGTTGCCGAGGAACATCGCGAGGACGTGTTGGAT

TCCCAGCGGGACCGCCTCGCCCAGCGGCGGTCTGTCTTCGATATCGTACA

GTACGACCGAGTCGCTCTCCGCATCGGCGGATGTGCTAGTCATCAGCTCA

CACGACACGAGACTGGCTTTTGAAACTACCCCTTATTCACCTAATATATC

TATGAATGGTGTTTTCCTTCGAGTAAGAATACACGTACAGACAGCGCTCG

CTCGTCGAGCGAGACAGTCGTAGAAATCATCGAGGGAACGTATTCAATCG

CGCGGCTCATCGAGGGCGCGCGCTACCGGTGCGACCGCCTGTGGCGTCCC

CGCCTACGGACGGGCCGATACCGACGGTCGGGAGCGCGTTGAACGTCCGC

TCGTCGACGAGGCCGCGCGTCGTCTCGGTGAGGGGCACGCGAGTACGAGG

TAGTCGCTCCGCGCGGCGGCCGAAAGCACGTCGTGAAGGACCGCGATGTC

GGTCACTCCGATACCTCTCGGATGACGACGTCTTGGACGCCCTCGCGGAG

CGTCAGTTCCTCGATTGCCTTCAGCTCCTCCGCCGTCGCGTCGATGGTCG

CGAAGTGGTTCCCGGCGAGTTCACCGGCGGGGCTCTTGAAGCACGTCGGG

CCGCAGGGGACGAGAATCTCCTGTTCGTTCCGGTAGCCGGGGTAGAGAAG

CAGGTCGCCCCGCGACGGGTAGACGGTGTGGTTTTCCCGCGGAATCTCGG

GGAGGTCGATTTCGTCGATGTTTATCCACGTCGCGATGCCGCTCCAGCGG

ACGTGCATGAGCTCCGACTCCAACGGGAGGAACTCTCGAACCGCCTCGAC

GGAGGCGGGCGCGCGGTCTTCGTGCAGTTCTGCGGTGAACGTCCGGTCTC

CGATTTCTAGCTGTAACATGGGTGTAGTGGCTCGTGTGATGGTGGTTAGT

TTCGTTCGGTGTCGTCCGTCTCGGTGTCGTCCGTCGTCGCCAGTCTGGCG

AGCGTCCCCGCGAGCACTTCGGTCGCGGCGGTGCAGTCGGCCCAGTCGGC

CCGCTCTCTCGGTGAGTGCGAGTGGCCGTTCTCGGAGGCGACGAACACGA

GCGCGGCGTCGGTCACGTCGGCGACCTGCATCGTGTCGTGGCCCGCGCCC

GAGTGGAGCGACAGCGTCTCGATGTCGCACGCGCGGGACGCCTCGGTCGC

GACCCGCCGACAGCGCTCCGACAGCGGCGTCGGCGGAACGTCGTAGGTGC

AGTCGAACGAAGTCGTCACGCCGCGGTGGGTCTCGACCGCGTCGAGCGCC

CGTTCGACCGCGTCCAACTGGAGGCGTATCTCCTCGCGGTCGACCGACCG

GAGGTCGAGGCGGAGCGAGGCCGCACCGGGGACGACGTTGACGACGTTCG

GTTCGACGTCGAGTTCGCCGACGGTGCCGACGGCGGTGCCGCTGCCGGTC

GTCGCGATGTCCGCGGCGCGCCGCTCGACCTCCAAGACGAGTTCGCTCGC

GGCCGCGAGCGCATCGTGGCGCGCCGTCATCTCGGTCGTCCCCGAGTGGT

CGGCCTGCCCCTCGATAGACACGTGACACCGCGCGGTTCCGGTGATGTCG

GTGACGATACCGAGAGGGACGCCGGCGTCTCCGAGGCGCGTGTTCTGTTC

GATGTGGAGTTCGACCCACGCGTCCCACGCGCTCGCGTCGAGGCGGCCCG

AGCCGCGGTAGCCGACGCGGTCGAGGGCGTCTTCGAGCGTCACGTCGCCG

TCCGTCAGCGCGAGCGTCTCGTCGACGCCGCGTTTCCCGGCGGCGACCGT

CGACCCGAGGACGCCGTCAGCGAAGCGCGTCCCCTCCTCGCCGGTGAAGC

AGACGACTTCGAGCGGGCGAGCGGGCGTCAGGTCGCTCTCGCGGATACTT

CGGACGGCCTCCACGGCGGCGTAGACCCCGAGCGGTCCGTCGAAGATGCC

GCCGTTCGGAACCGAATCGAGGTGGCTCCCGGCGGCGACCGGGGCCGCAT

CCGGGTCGACGCCGTCCGGGGTCCACCGACCGGCGATGTTGCCGACGGCG

TCGATACGGACGTCGAGACCGACCGCTTCGAGGCGGCTCACGAGGTAATC

GCGGGCCCGCCCGTTCGCCGCGTCGGCGGGGAGCGCGGTCCGACCGCGCC

CGTGTTCGGTGTCTACCGAGCCGAATACCGCCGTTCGCTCGATGTCCTCG

CGGAAAGAGTCGCCGTCGATGTCGAACGAAACGCGACCCGCACTGCCGCG

AGTCGGGGCCGTCACGGTCCCCCGTCACGACGACCGCTGCGCCGTCGATT

CCGCCGCCGGGTGGCACCGTGTCGCGGCGAGGCAGGAACGTCCGCGACCG

AACCGCCGCCCGTCCCCGTCGTCCGGAGCCGGCGGTCGGCGGCTGTCGGC

TTCGGTCTGAAAGGTATCATTGCGTACTTACCCCGAGAGTAACACCACTT

GTTGATAAACATTTCGTGAGATGGCGATTTGGTGGCCAGAATACTCGATG

TTTTTCAAATTTAGTGAATACATACACAGTTCCAACCGGAGGGCCGGATT

AGAATGTTCGACTATTTCAAACGCCGACGAGACAAGTGCGTCCGACAACC

GCCCACACGACCAGCTCGAACCCGATTACTCATCCCGTCCGAGCGAGACA

TCGAGCGCGCTCGACGAAAGTGGGGTGATACCGCTCCGTTAGCCACCGCC

GAGCGACGGTGGTTACCTCGGCTCGTCCAAGATGCTCGAACGCGCGACGG

GGGACAGCGACACCACGGCGTGCGCTCCCGTCCTTGGGTGAACTCCTCGC

GATGGATTACAAATTTACGGTTGTAATTTCCTATTCCAGACGCACGACCC

CCGTGATGACAAACGGTATCGTGTCTCTCGCGAGCCGCGAGCGGTCGCCA

CAGCGCGCTCGAACCTCCCGCGGAACGCGTTCGACTATTTCAAACGGGAG

CGTCGAATCGGGAATCAGGCGGGGTCGGTGCGAGGCGGGGTGAGGCTGGG

GAGACGACGAACGAAACGACCACACCGGCGACATCGAGACCGCTCCCGAA

GACCGCGCTCGTGAACCGGACAATCGAGGGAATGCTGGCACAATTATGCC

CCCTACCAGAATATTTAATTGCGTATAGGAGCGGATATATTCAAGAATGA

TTCAAGATAGCACGTCAACTACCGTAGTCCGAGCGAGCGTGAACGGTGAA

GACGAACAGTTCGCCGTCTCGTCCGGCGAGACGCTCATGGAGACGCTCCG

CGGGGCGGGGTACTACGGCGTCAAAAACGGCTGTGACGAGGGCGTTTGCG

GGGCATGCAACGTCATCCTCGGCGACGAGGGAGTCACCCGGTCGTGTCTC

GTGCCGGCGGCGAGTTGCGACGGAGCGGAGGTGATGACCGTCGAGGGACT

GGCCGACGACGACGGCGGCCTCCACCCCCTTCAGTCGGCGTTCCTCGAAC

ACGGGGCCGCCCAATGTAGCTACTGCATCCCCGGCGTACTGCTCGCCTCG

TACGACCTCCTCCAGCGGAACGACGAGCCGACCGAGAGCGAGGTCGCAGA

CGGGCTGAGCGGCAACATCTGTCGCTGTACGGGCTACGTCCAGCAAATAG

AGGCGGTGCAAGCGGCGGCCGACCGCCTCTCCGGGTCGGACGCGTCGTCC

GAGGGCTCCGGGTAAGATGCCTCGGTCCGACACCGGCGACCCCGAGACGG

CGGCGAAGTCGACCAGCGAGGCGCGCGGAGACGACGCCCCCGTCGGCGAG

AGCGTCGAGAAAGTCGACGGCTGGGGGCTCGTCACCGGCAAGGCACGCTA

CACCGACGACGTGCCGACCGACGACGCCCTCGCCGCGAAGGTGCTTCGGA

GCCCGCACGCGCACGCTCGCGTTCGGTCTATCGACACCGAGGCCGCGGAA

ACGATAGCCGGCGTGAAGGCGGTGCTCACCCACGAGGACGTTCCCGAGGA

CCGCTTCACGCGAACCGGTTTTCCCTACCCGGCCCCGGCCCCGTTCGACG

AGCGCGTCCTGAACGAGACGGTCCGGTTCGTCGGCGAGCCGGTGGCCGCC

GTCGCCGCGCGAACCGCCGAGGCCGCCGCGGCCGCGGTCGACGCCATCGA

GGTCGACTACGAGCCGTACGACCACGTCCTCGACGCGCACGAGGCGATGG

GGGCGGACGCGCCGACGCTCCACCCTGAGCCGTACGAGAACCCTCAAGAG

AACGCGGCTCCCGAGCGGAACGTCGTCTGCGAGACCCGCCACGAGGAGGG

AAACGTCGAACGCGGGTTCGAGGCGGCCGACGAGGTCGTCGAGGGGGAGT

ACGAGACGCAGGCCGTCCACCACCTCCCGATGGAGACGAACACGACCATC

GCGTGGGTGGACGACCGAAGGCGGCTCGTCCTGCGGACGACGACGCAGGT

CTCGCACATCTGCCGCGACAAAATCGCCCGCGTGTTCGGCCTGAACCGCA

CCGACGTTCGAGTCGTCAAGCCCCGCGTCGGCGGCGGCTTCGGCGTCAGA

CAGGACACGCTCCCGAACCAGTTCATCGGCGCGGCACTCGCGCTCGAAAC

CGGCGAGAAGGTCCGGCTGAAAAACGACCGAAAGGAGGACCTCCACGGGG

CGCAGACGCGCCACGCCCAGACCGTCCGCGTCAAGACCGGCGTCCGGGAG

GACGGGACGCTCACGGCGATGCACGTCGACGTGACCTCGAACACGGGCGC

GTACGGCTGTCACGCCTCCGCGGTGCTGATGAACGCCGCCCACGAACCGC

TGTCTGTCTACCCCTGCGAGAACCGGCTGTTCACCGGGCGCGCGGTCTAC

ACCAACGTCACGCCCGGCGGGGCGATGCGCGGGTACGGGGCGGTACAGGG

GACGTTCGGCATCGAGAGCCACCTCGACGAGGTCGCCGCGGCCATCGGGA

TGGACCCGGTCGAGTTGCGGCGGCGAAACGCGGTCGAGGAGGGCGACGAG

AGCTTCGAACCGGAGTACAGCGAGAGCAAGCGAACCCTCGAATCCGTCGG

CGTCAAGGAGTGTCTCGAACGCGCCTGCGAGACGCTCGGCTGGGCGGACG

GCCCCGAGGAACCGGCCGACGACCGCTACCAGCGCGGGTACGGGGTCGCG

CTGGCGATGGCGAAATCCGGCGTCCCGAGCAGCGAGTTTTCCCGGTGTAA

CATCACGCTGGAAGATGACGGGACGCTCACCGTCCGGGTCGGCGTCGGAG

ACACCGGACAGGGCTCTGAGACGGTGATGGGCCAGATTGCGGCGAGCGTG

TTCGGACTCGGCATCGAGTCGGTCCACGTGAAGGCCGACGACACCGACGC

GACCCCGTGGGACAACGGGGCGTACGCGAGCAGTACGACCTACATCAGCG

GGAACGCGACCGAGAAGGCCGCTCGCGACCTCGCGGCGAAGGTTCGCGAC

CTCGCGGCCGAGTGGCGCGGAGTCGCCCCGGACGACGTGGAACTGGCCGA

CGGCGAGGTCCGGTTCCCCGACGGAGAGTCGATGGCCCTCGAACGGTTCG

CGTCGGAGGCGTTTCAGGGGATTCACGGGCCGAAGCGGCGACTCACGGGA

ACCGGGAAGCACCACACTGCGCTCTCGCCCAAGCCGTTCGCGGCGCAGCT

CGCGGCGGTCGAAGTGGACACCGAGACCGGCGAGTTCGAAGTGCGCCGAC

TCGTGAACGCCGTCGACTGCGGACGGGCGATTAATCCCGCGGGCGCTCGC

GGCCAAGTCATCGGCGGCGCGGTCATGGGACTCGGACAGACGGTCTCCGA

GGACCTCCCGCTGGACGACCGGGGCGCGCCGGAGTTCCGCGGGCTCCGCG

ACTACGAGGTGATGCACGCGCCGGAACTCCCCGACATCGACACGGAGTTG

GTCGAAACCGACGAGCCGACGGGGCCGTTCGGCGCGAAGAGCGTCGGCGA

GGTCTCGATACTCGGCCCGCCCGCCGCGATTGCGAACGCGATACACGACG

CCGTCGGCGTCCGGGCGACCGAACTCCCGATTACGCCCGAGACGGTGTGG

ACCGCGCTCCAGGAGGACCGGGACCGATGACGGCGTTCGAGTCGGTCGAG

TACGTCGAACCCGAGACCGTGTCGGAGGCGGTCGAGCGACTCGCAGACGC

CGACGAGGCGCGCGTCATCGCCGGCGGATACAGCCTCGTACCCCTGCTGA

AAGACGGCATCGAGACTCCCGACCGGCTGATAGACGTGAGCGGCCTCAAC

GAGCTGCGCGGGATCACGGAGCGGGGCGACGGCGTCGCTATCGGCGCGCT

CGTGACCCACGACGAGATTGCAACCGACGCGACCGTCGCGGAGCGCGCGC

GGGCGCTGGCCGACGCGACCGATTCGGTCGGCGACTTTCAGGCGAGAAAC

CGCGGGACGATCGCGGGCAACCTCGTGTTTGCCGACCCGAAATACGACGC

GCCGGCGGCGTTTCTCGCACTCGGCGGGAGAGTCGTCACTCTCGGGCCGG

CCGGCCGGCGGGCGATAGCGGCGGACGACTGGTTCCGCGGCCCCGGAACG

ACCGCGCTCGACGGGAACGAGCTCGTCACCAGAGTCGAGGTGCCGAGCGT

CGAACGGAGCGGCTACGTCCGAACCTCGGAGTACTCGGGGTACGCCGTCG

TCGGTGTCGCGGCCGCGTTCGAGACCGACGACGGGGTCGTGCGCTCGGCC

CGAGTCGCGGTGAACGGGGCCAAGCCGTATCCGGTTCGGCTCCCGAACGT

CGAACGCGCGCTCGTCGACGAGGCCGTCGGCAGGGTCGTCGACGGCGACG

GTGCGACCGAGGCCGCGACCGCCGCACTCGACGACGTCGAGACGGCGTCG

CTACTGGCGAACGACGCGGCGACCGAGGAGTACCGGCGACAGCTCGTCCG

GACGTACTGCCGACAGGCAATCGAGCAGGCCGCTTCGGAGCCGTGAACCG

CCGGCCGAGCGGGGCTGTGGGCGTGTTCGTCCGACCGCCCCGTGCGGCGG

GGCCACCGAACCGACCGATAAATCAATACTGCTGACGGGAGTACGGGAAG

GCGACTGAACGATGCGAACGACAGACGACCCGTGGAGCGCGACCGCAGAC

AGCGTTCGGCAGTCGATGCGCGAGCAGCGAGGCTCGGGGTCGGAGGCGGT

CGTCGCGACCGTCGTGAACGTCGAGGAGTCGGGCTACCGCCGACCGGGGG

CGCGTATGGTCGTCGTGCCCGACGGCGAGCGACTCGGCGCGGTCACCGCC

GGCTGTATAACCGAGTCGGTGACAGAGACCGCGCGGCGGGTGAGCGCGAG

CGGGACGCCGCGACTGGAGACGTTCGACCTCCGGGGAGGCGACGCCGACG

CGTGGGGGCTCGGACTCGGCTGCAACGGCGTCATCGACGTGCTCGTCGAG

CCGGTCGACGCGAGTTTCGACCCCGCGTTGAGCGAACTCCGCGAGAAACG

CGCGGCGACGGTGCTCACCGCCGTCGAGACCGACGACCCGGCAATCGGCG

TCGGCGACCGGACGGTGATAACGCGGGGACGGGGCGAACGGCTCTCGAAG

GGCCGTCCCGGCCTTCCCGAGGCGGTCGTCGCCAAGGCAGAATCGCGGCT

CGACGGGGCGGCGTCAGGCGCGGCGGCGACGCTCGACGTGCGGACGAGCG

ACGGCGACGTGCGGGTGTTCGCAAACGACCTCGCACCGACGCCGGAGCTG

CTCGTCTTCGGCGGCCACGGAGACGTCAACCCCGTCGCGTCGCTCGGCGC

GCAGGCCGGCTTCCGCGTCCGCGTCGCGGCCGCGAGAGGGGCGCACGCCG

ACGAGAGTCGGTTCCCGGCGGCCAGCGAAGTGACCGCGGTACACCCCGCC

GAACTCGCGGCCCTCGTCGACGCGCCCGACCACACCTACGTCGTGCTCAT

GTCGCACAACTTCGTCGACGACCGACTCGCGCTGGAGTCGATACTCGGGA

CCGAGGTTCCGTACGTCGGCGTGATGGGCCCGCGGCGGCGGTTCGACCAG

CTCCGCGAGGCGATGGACCGAGAGCTTGACGCGACCGACCGCGAGCGAAT

CGCGGCCCCGTGCGGGCTCGACATCGGCGGCGACGGGCCGTCGACCGTCG

GGCTGAGCGTCGTCAGCGAACTGCTCGCGGTTCACAACGGCCGCAGCGGG

GCACGGCTGACCGACCGCGCGGGCCCGATTCACGGGCGGCCCGAGCTCGG

ATGACCCGGGTGGGCATCCTGCTCGCCGCGGGGGCGGGAACCCGGTTCGA

ACCCGGGAACAAGCTCTGTCAGCCGCTCGACGGCGAGCCAATCGTCCGCC

GGGCGGCCCGGCGGCTGATCGAGTCGCCGGTCGATGAAACCGTGGTCGTC

CTCGGACACGACGCCGAACGGGTCCGGCGGGCGTTAGAACCGCTCGAAAC

TCGGCTGACGGTCGTCCGTAACGAGCGGTACGACGCTGGACAGAGCGCGT

CGGTTCGGCGCGGGGCGGAGGAGGTGCTGTCGCGCGGCGGGTCGGTCGGC

GTGTTCGCGCTCGGCGACATGCCCGCCGTCGGGAGCGCGACCTACGACGA

ACTCCTCGCGGCGATTGACCGCGACGACCGACACGTCGTCGTCCCGGTGT

ACGACGGGCAACGCGGAAACCCCGTCGCGTTCGACGCGGCAGCGCTCGAC

CGGTTCGGGAGGCTCACGGGAGACGCGGGTGCGCGCGCCCTGTTCGAAGC

GATGCCAGTCGCTCGAGTCGCCGTGGACGACCCCGGAATCCACGCGGATA

TCGACACGGTGGCCGACCTCGAAGCGCACCGCTGACGCCGGTCGTCGAGG

CGTCTCCCGACCGCCGCGAGTTCGGCATCGTGGAGGGCTGACCGGGCACG

CGGGACCGCCGGAGCGACGCGGGAGCCACACCACCGCCCTCGGGATGCAG

TAGACAATTATATTCATTATATCCGTGGGGTGTGAGGGAGGAGATACTAA

CAACATTACGACGGTACTACTGTAACGAGTCGCGGGCGCGGCTCGGCGTG

ACCGAACGACGGGCGACACGAGTGAAGGCGGCGTGAGCGGTGAAAAGAGG

GGTGACGGCGCGAGTCAGGGCACGGCCAGTGCCACGGTCGTGGCGTCGAA

CGCCGGGTCAGTCCATCTCGCGGTTGTACGACTGCAACAGCGCGGAGGGG

ACCGCGAGTCGGACGCTCTCGGTCCGGATGACCGCGTACGAGAGGACGGC

AATCGTCGTCAGGTAGGGGTACGTAGACATAATCTGCGGGTTCATGAGCG

TGTTGACGAGCGGGTTGAGGACGCCGGCAAGGGGGGCGTTCGGGTCGAGG

GCCAGCGAGAGCGACTGCGACCGGAGTTGAAGCGCGTCGAGCAGGCCGAA

GAGGTACGCGCCGACGAGCATCCTGCTGGGCCGCCACTGGGCGAAGACGA

CGAGTGCGACCGCAATCCAGCCGCGGCCCACGGTCATGCCGGGAACCCAC

AGTTGGGAGAACGCCAGCGAGAGGTGCGCGCCGGCGGCCCCGGCGAAGCC

GCCGCCGATGATGACCGCGAGATAGCGAAATTTGAACACCGGCACACCCA

TCGTGTCGGCCATCTCGGGGTCTTCGCCGACAGCGATGATTTCGAGACCG

AGGTTCGACCGATACAGGAAAAACCAGACGACCGGCACCGCGAGGAGCGC

GAGGTAGTCGGTTGCGGTACTGCGGAAGAACGCCTCACCGACGAGTGGAA

GGTGGACGAGGTAGCGGCCGACTATCGGGAAAGTCATCTGGGGGAAGCCG

GTGATGGACTCTTGGACCCACCCGGACCCGAAGAACGTCGTCAGGCCGGT

CCCCAACAGCGTCAGCATGACGCCGCTTATGACCTGGTTCGACTTCAGGG

AGATACAGAGGAACGCGTGGACGGCCGCGAGCGCCATCCCGCAGGCGATG

CCGACACCGAAGCCGAGCCAGTAGTTCCCCGTAACGGCGGTGACGACGAA

CCCGCCGAGCGCGCCGACGAGCATCATCCCCTCGACGCCGAGGTTGAGGA

CGCCCGCGCGCTCGCTGATGAGCTCTCCGAGCCCGGCCAACAACAGCACC

GTCGCGGCCTGCACCGTCGCGTTCAGGAGGCCGGCGGCGAAGCTCACCAC

CGCGCATCACCGCCTGTGGCGTCGGCCGGCGCGTCGTTCCCCCGCCGGTC

GAACGCGATGCCGACTCGGTAGCGCTTGAAGAACTCCGAGGTGATGAGAA

AGAGGATGACGAGCGCCTGAATGATTTCGACCAACGCCGCGGGGACGCCG

AAGGCGACCTCGACGCTCGACCCGCCGACGAACAGGACGGCGAAAAACAG

CCCGGCGAGCGTGACCTTGACCGCGCTGTTGCGGCCCAGGAGGGCGATGG

GAATCGCGGTGAACCCGTAGCCGGGGGCGAAGGCGGCGCGGAACCGCCCC

TGCGAGCCGGCGATTTCAGCGATGCCGCCGAGCGCCGCGAACGCGCCGCC

GAGGAGGAAGACGAACAGGTACACCGTGTGCCGGCTCATCCCCGCCTGCC

GGGCGGCCTCGTCGTTCGAGCCGACGAACGTGACCTCGAAGCCGAGGCGC

GTCTTGGTCATGACGACGTACGTGGCGACGACCGCTGCGACGGCGACCAA

CAGGCCGGCGTGAACGTCCGCGAAGAGCGGTATCGACTCGCCGCCGGGCA

CGAGTCCGGCGAGCGGCGGGAGGGTCGCGGCGTCCGAAAAGCGAGCCGAC

TGCGGGAAGTTCCCGGTGCCGCCCTGCATGGGACCGCGGAGCAGGTAGCT

CTGGAGTTCCTGTGCGACGAACGTCAACAGAAGCGAGGTGATAATCTCGT

TGACGTCCCACTTCGCGCGGAGCCACGCGGGGATGCCCGCCCAGAACGCG

CCGGCGACGCAGGCGGCGAGAAACATCAGCGGGAGCAACGCCACGGCGGG

AAGCGAGACGTTTACGGCGACCCACGTGCCGGCGAGCGCGCCGAGGAGGA

GCTGTCCCTCGGCACCGATGTTGAACAGCCCCGCCTTCAGCGGGAGATAC

ACCGCGAGGCCCGCCAGAATCAGGGGGACCGCCCTGACGAGGACCTCCGT

GAGGCCGAACTGGCTGGTGAGCGTCTCGACGAACATCACGCCGTAGGCGT

CCACCGGGTCGACGTTCAGCGCGACGAGCGCGACGCCGCCGACGGCTAAG

GCCGCGAGGACCGTCAGTACCGGCGTCCCGTAGGCTAGCCACGCGGGGAC

GGTTCGACGGGGGTCGAGTTGCAGTTCGACGTTCATGAGCTCCCACCGAG

TCCTCGGACTTGAGCGCCCGCGGTCGCGGTGGCGTCCCCGCCGCCGGTCA

TTTCGAGCCCGATTCGCTCGCGGTCGGCGTCCTCGGGCGTCGTCTCGTAG

ACGACCTCGCCCTCGTAGACGACGAGGATGCGGTCGCTCAGGTCGATAAT

CTCGTCGAGGTCCTCCGAGAGCAGCACCACGCCGGTCCCCTCGGTCCGCT

GGTCGAGAATCGCCTCGCGGATGGACTCTATCGCGCCGACGTCGACGCCG

CGGGTGGGCTGGTTCGCGACGAGGAGGTCGGGGTCACGGGAGATTTCTCG

GGCGAGAATGAGCTTCTGTAAGTTACCGCCCGAGAGGTCGCCGGCCGTCG

CGTCGGTCACGTCGTGGACGCCGCGAACGTCGAACGCCTCGACGAGCGCC

TCGGCGTGCGCCCGGAGTTCGCCGTAGTCCAGCGTCACCCCGCTCCCGAA

GCGGTCCTCGCGGAAATCCTTCATCGCCGCGTTGTGCATCACAGAAAGGT

CCCCCGCGGAGCCGTAGCGGTTTCTATCTTCGGGGACGAACGAGACGCCG

TTGTCGATAAACGTCCGGGGCGAGGCGTTCGTGAGGTCCCTCCCGTCGAC

AGTCAGTTCGCCCGCGGAGACAGTGCGGAGTCCGGCGATGACTTCCGCGA

GTTCCTTCTGCCCGTTACCGCTGACGCCGGCGATGCCGACAACCTCACCG

CGGCGAACCGTGAGGTCGACGTTCGACAGCGCCTCGATGCCGCGGTCGTC

CGTGGCGGAGACGTTCCGCGCCCGGAGGACTGGCTCGCCGGGGGCGACGC

GTTCTCTGTCGACCGTGAACAGCACTTCGCGGCCGACCATCATCTCCGCG

AGGTCGGCTCGAGAAACGTCTGACGTGGAGACGGTGCCGACGTTCTCGCC

GTCGCGGAGGACGGTCACGCGGTCGACGACCGCTTCGACCTCCTCGAGCT

TGTGCGTGATGAAGATAATCGACACGCCCTCGTCGGTGAGGCGTTCGAGC

GAGTCAAAAAGGAGGTCGGCCTCGGTCGGCGTCAACACGGCCGTCGGCTC

GTCGAGGATGAGCAGGTCGACATCGCGGTACAGCGCCTTGAGAATCTCGA

CGCGCTGTTGCTGGCCGACGCCCAGCTCCCAGACTTTCGCGCTCACGTCG

ATGTCGAAGCCGTACCGGTCGGCCAGTTCTTGGATGCGCCGCTCCGGAAC

GTCGAGGCCGAGCGAAAACCGCCCGGCGAGCGACTGGACGAGCGAATTGG

AGCGGACGGCGTCCGGGAGCCATCCGCCCGCGTCCGCGTCCGGGCGAAAC

GGCGTCGCCGGCTCGCGCTCGCCGAGGACGACGTTCTCCGCGACGGTTAG

ACGGGGAATCAACATGAAGTGCTGGTGGACCATCCCGATTCCCGCGTCGA

TAGCGTCCTGCGGGGAGTCCAGCCGAAGCCGCGTTCCGGAGAGGGAGATT

TCGCCGTCGTCCTGCGAGTAGAGACCGTACAGAATCTTCATGAGCGTGCT

CTTTCCAGCGCCGTTCTCGCCGAGCAACCCGTGGATTTCACCGCGCTCGA

CGGTGAGGTCAATGTGGTCGTTGGCCACGACGCCCGGAAACGCTTTCACG

ATGTTCTCCATCCGGAGAAATGGTTCCCTTGTCATGTGGTATATTCACAG

GAGTGTATTGAATCCACCAAGATAACTGTTCTCATGACCGGAATATTTGT

TTTGACGTGCCCATCTGTTCGGGGGTGTTCGGATGACGTGGGGAAATTCA

TAAATCCCGCCTCCGACAAGAGCGCCTATCGATGTGGACACCGACCGCCT

CTCGGATAGCCGAATCGAACGGAGCGCAACGATGAGCGACGAACCGGTTG

ACCTCCTCGTCAGCGGGAACCTCGTCAACGTCGACACGGGGACCGTCTCG

GAGTCGTTCGTCGCCGTGGACGACGGGCGAATCGTCGCGCTCGAAGAGCG

ACCGGCGGCCCGCGAACTCGACGCGGAGTACATCGCGCCCGGTCTCATCG

ACGCGCACATGCACGTCGAGTCGTCGATGGTGACGCTCCCGCAGTACGCC

GACGCGGTGGTGCCGCGGGGCGTCACGAGCGTGGTTCACGACCCGCACGA

GATTGCGAACGTGAGCGGCGTCGCCGGCGTCGAGGCGATGATGGCCGACG

CGACACGGACGCCGCTGAAAGTCCGCTTTACCGTTCCGTCGTCGGTCCCG

GCGACGACGTTACAGGACGGGGGCGCGACGCTCGATGCCGAGGCCGTCGA

CGCCCTGCTCGGGGAAGACGAGGTCGTCGCGCTGGGCGAAGTGATGAACG

TTCCCGGCGTCGTCGCCGGCGACGACTCCGTCCACGAGAAGATTCGGGCC

GCGAGGGACCACGGGCTGACGGTCGACGGACACGCGCCTCGGGTACGCGG

AAGCGACCTCCAGACGGTCGCCCGATACCTCGACAACGACCACGAGAGCA

TCAGTCTCGCGGAAGCCCGAGAGAAAGTCGAGGCGGGACTCCGCGTCTAC

CTCAGGGAGGGTTCCGCGAGCAAGAACCTCGCGGACGTGGTCGGTCTCGT

CGACGAGGTGGGCAGTCGGCGGCTCTCGCTGTGTACCGACGACAGAGACG

TCGTCGACCTCGTCGAGCGGGGCGGCGTCGACTTCGCGGTTCGAAAGGCC

ATCCGGTTGGGTGTCGACCCGGTTGAGGCGGTGCAGATGGCGACCGTCAA

CACCGCCGAGAGCTACGGGCTCCCCTTCGGGCGGATTCGGCCGGGGGCAC

CGGCTGACCTCGTCTTGCTCTCGGACCTGCGAACGTGGGAGGTGGACCAC

GTCGTCGTCGACGGAGCGGTCGACCCGACGAAGGGTGGTGGCGCGCCGCC

GGCGTCGGCCATAGCGACCGGCACCGTGAGGTTCGAACCGGTGTCGGCGG

CGGACCTCGCGGTGCGACACGCGGGGGACTACCCCGTGCGAGTCAGAGTC

ATCGACGCGGTCGGCGGACTCCAGACAGGACGGGCGGAAGCAGACGTTCC

CGTCGCGCCGGTCAACGAGGAACGGGTCCTCACCGCGAACGCGGACGACG

ACGTGCTTCCACTCGCCGTCATCGAACGGCACGGGGAAGCGGGCGGCATC

GGCCGCGGATTCGTCCGCGGGCTCGGGCTGGAGCGGGGCGCTGTGGGATC

GACGGTGGCCCACGACGCGCACAACTGCATCGTCGCCGGCGTCGACCACG

ACTCGATGGCGACCGTGGCGAACCACCTCCGGGATGTCGGCGGCGGCGTC

GCGGCGTACGACCCGGAGGACGACGCCCTGTCGTCGCTGGCGCTTCCGAT

GGGAGGGTTGATGTCGGACGAATCGCTGGGGACCGTAAAGGAGACGTTCG

AATCAGTCGAGGCGACCGCCGACGACCTCGGACTCGACCGCGACGGTGGC

CTGATGGCGCTGTCGTTTCTCGCCCTCGAAGTGATTCCGACGTACCGACT

CACCAACAACGGGCTGGTCGACGTGGAGTCGTTCGAGTACGTGCCGACAG

TTATCGACTGACAGCAGAACGACCGTCGCGGCGCGTGGAGTCGATATTCG

TCCACAGAAACATCGTATTCAGCGACATTCGATTTCAAAACGGGGAGGAA

CTGACTTGTATGACAATTATATCTAGAAGATGATGAAAATATTTAAGTAT

AAATTACAATGACAAGTAAGCGGTTACGCGATGTTAGACGAGGAATCTTC

GATTCAGCGGAGAGACGTACTGAGTGCGCTCGGTGCCGCCGGCGTCACCA

CGCTCGCGGGTTGTACCGGTGGAGACACCGGAGACACCGACGACACGGAG

GCGAGCGAAACGACGGCCAGCGAGGGAACGACCAGTGGAACGACGACCGG

CGACGTCGAGACCACCGACGGCGGGGGGCCAAGCGAGGGAGAGACGGTCA

ACGCCGCGTGGGTGTACATCTCCGAAATCGGTGATCTCGGGTGGTCGTGG

GCGCACGACCAAGCCCGGCAGGCCGTCGACGAGCAGTACGACTGGTTGGA

GACGGAGTACACGGAGGCCGTCGCGCCCTCGGACTCCGAGCGGGTCTTCG

AGCAGTACGCCCAAGGGGACGTGGACGTGATATTCGGAACCACGTTCGGC

TACCAGGACCCGATGTACGCGGTGGCGGAGGACTACCCGGACACCGTGTT

CGAACACGCGACGGGCTACCGGACGCGGGAGAACATGGGTCGCTACATGG

GTCGCATCTACGAGCCGCGGTATCTGGCCGGACAGGCCACCGGAATGGTC

ACCGAGAACAACACCATCGGGTACGTGGCCGCGTTTCCGATTCCGGAGGT

CGTTCGGTCCATCAACGCGATGGCGCTGGGCGCGCGCTCCGTCAACCCCG

AGGCGACGTTCAAGGTTCGCTGGGTCAACGCCTGGTTCGACCCGCCGACG

GCAAGAGAGGCCGCGAACGCGCTCATCGACGAGGGCTGTGACGTCATCGC

CCAAGAGCAGGACTCGCCGGCGGCGGTCAGGGCCGCCAGCGACGCCGGCG

TCTGGACCTCAGGGTACAACGCTCCGATGGGGCAGTTCGGCGGTGAGAAC

TACCTTATCTCACCGATTTGGGACTGGACCGAGTTCTACGGTCCGACCCT

CGAATCGCTCCACGAGGGGTCGTGGGAAGCCGATGCGTTCTGGGGCGGGA

TGGAGACGGGCGTTCCGATGCTCGACGAGTGGGGACCGAACGTCTCACAG

GAGGTCAAAGACCAGGTCGCCGCGACGGAAGAGCAAATCCTGAACGACGA

ACTCGACGTGTGGGCGGGCAGCGCGTTCGAGGGGGAAAGCGACGAGTTCC

TCTTCCAAGAGATGAGCAGTTTCGTCGAGGGCGTCGAAGGCGAAGTGCCG

AGCTAATCCGCGTCTGCCGGGGATGCGCGGCGGAGCACGACGACGGTCGA

CCGGCCGACCGACGGACGAGTATGCCAACCCACAGCGCACTTTTTGCGGC

CGCGAACTACCGCAGACGACGAGGGCCACCGGACCCCCGCCGGTGGTGGC

GACCCGGAGTCACTGCGTTCGGCTATGGTAAACGAATTTCGCGGATTCCG

AGAACGGACAGACCCGCCCTTAGACCGGAATATGAGAATAAGGCCAAATA

TAGTGTATTTACCGTACGTACATATTCATGTAGCATCGTATAGTTAACAT

AGGCGAACGTTAATACGGGAGGAGTGACACAACCACACTATGAAACAGAC

GGGGCAAAAAGGGGGGCCACGGACGCTGAAGACGGTCACGACGGCGTCAC

GGGTTCTCGACGCCGTCAAAGACTGCGATGGAATCGGTGTTTCGGAGCTC

TCCGACTATTTGGACATCTCGAAGAGCACGGCGTACATCCATCTCAGGAC

GCTGGAGGAAAACGGCTTGCTCGTCCAGCGCGGGGACCGCTACCGGTTCG

CGTTCAAATTCACCGTCCTCGGAGAGTACGCGCGGAATCAGAGCCCGCTG

TACCGATACGGTAAGCCGGAGGTCGAAAAACTCGCGGCGGAGACCGACCA

GTACACGCACATCGTCACCGAGGAGAACGGCTACGGGGTCAACCTCTACC

AGGTGAAAGGGGACACGAGCGTCGATGGAGAGTACCAGACCGAGAAGGTA

CAGAGCCAAGACCACCTCCACTACACCGCCTCCGGAAAGGCGATTTTGGC

CGCCCTTCCCGACGAGCGAGTCGAGAAAATCATCGAACAACGCGGACTCC

CTGCGCAGACGAAAGCCACTATCACCGACCGCGAGGCGCTGTTCGACGAA

CTCGAACAGATTCGCGAGCGCGGCTACGCGTACAACGACGAAGAGGAGAT

CAGAGGGTTTCGCGCCATCGGTGCGCCAATCGAAGACCCGAGCGGGCAGG

TGCTCGGTTCGGTGAGCGTGTCGGGCCCGACCAGTCTCCTGCAAGGCGAG

CAGTTCCAAGAGCACGTCCCGAAACTCGTCACGCAGTCCGCGAACGTCAT

CGAGGTCAACATCAACATGAACGCGCAGTCGTAGTCGATGGGACGGGGCG

TTTGTAATGTTCGAACGCGCTTCCAAACGGGAGTCGTATGAGAATATTGT

TAAATCCCCACGATATTCATCTTGCATCGCCTTGGAGAACGCTCAATATC

ACCGACATCAGCACTCTCTGTTGGAGTTGATTCGTCACACCCACACAAGT

GTGTTATCACCGTACAATACACCGTGCGCGATGACACGGATGCGGGAGCG

GCAGATAATTTTACACATCTACGACCGTAATCCGCTGCGACGAAGTGCTG

AAATTGTATCATTAGATAGTCACGATTCGAGAGCATATAAAGTTCGGCAA

AAACGAACGTAACCGTCGGGCGAGCCAGGAGAGCGGGCGAAGACCGCGGG

TTCGGCTCGCTCGAACCCGAGTCACGCGAGCGCGAGTTCCAGTTCGAACT

CGCTGACGATGTCCTTGACCGTGTGTGCGAGTTCGGCCTTCCGGTCCTCG

CCGGTCACCGAGTGGGCCGGGCCCGTGACGCTGAAACCGCCGATAACGTC

GCCCGAACGGCGCGTCGCGGCGACGCCGATGGCGTAGAGTTCGTTGAAGT

TCTCGGCGCGGTTGACGGCGTAGCCCTGCTCGCGGACGCGCTCCAGTTCC

TCGAACAGCGCCTCGCGGTCGGTGGTGGTGTTTTCCGTCTCCTGCGGCAG

GCCCCACCGGTCGAGTATCTGTTCGATGCGCGGTTCGGGGAGTTCCGCGA

GAATCGCCTTCCCGACGGCGGTGTTGTGCAGGAACAACCGATTGCCGAGT

TGCTCGTGGGACCACCCCATCTTGCTCCCCGAGGCGGTGTGCAGGAAGAC

GGCCTTCCCACCCATCTCGACGGTGAAAATCGACCGAAGGCGGGTCTCTT

CGAACAGTCGCTCGGTGAACTGCCGCGCGAGGACGAACGCCGGTTCCCGC

GTCCGGACTTGGTTCCCTAACCGAAGCAGTTCCGGGCCGATGAAGTAGAA

ATCGCCCTCCTTGATGACGAACTGTTCCTGCTTGAGCGTCGCGAGGTGAC

CGTGAATCGTGCTTTTGGGCTTGTCCATCCGCTCGGCGATTTCGGAGACT

CGCGCGCCGTCTATCGCTTCGAGTTGCTTCAGAATCGCTATCGAGGTGGC

GGTCGTCCGGAGCGTCTTTGGTGAGTTATCCGTTGGCATACCTCAATATT

TTCGGCTATGTATTTAAAGTTCAGGATAGTCGAACAGCTGGTCGACCCCG

CGGCGCAGGGTGGCCCGGCGGGCGCGGCGTCGTCGGGCGCGTTCACCCCT

CGACTTCGACCCAGCCGCCGCGCTCGCCGGACTCGTAGGCCGCATCGAGG

ACGCGAAGCAGCTGCACGGCGTCGTCGGCCGTCGCGGGCACGTCGCCGGT

CTCGTAGCCCGCGAAGTAGTCCTCGAAGTAGTCCTGCACGAAGTCGCCCC

ACGCCGGGAACCGGTCGTAGGTGAACTCGAACTCGACGGTCCGCTTGGGC

GCGGCGGTCCAGTCGGGGTGTTCCGAGGTGATGTCGAGCGGGACCGTGGG

TTCGTGCTGCAGCGAGTCGTGGTGCAGCGGCGTCAGCGCCTGCCCCTCGG

TGCCGTAGAGCCCGAGGTGCGTGTCCTTGCCGCGGTCGCTCAGGTAGTAG

CCGGTGTGGTACGTCCCGAGCGTCCCGCTTTCGGTCTCGAACTGGAGGAC

TGCTCCGGCTTCCACGTCAGCCTCGACCGCGTCGTGGAACCGGGCGTTCA

CCCGCGCGATGGGGTCGTCGAGAATCCACGGCATCACGTCGACCCAGTGC

GGGCCAATCCACTGGAGCGCGCCGCCGCGACTCGTCTCCCTGTCGTAGAG

GTAGTGGTCGGTGTCCCGGTAGGAGAGCTGGCTGGCGTTGAAGCGGCCGT

CGAGCGTCCACACGTCGCCGAAGAAGCCCTCCGAAACTCGGTCGCGGAGC

GCCATCGCGACGGGGTTGCGCCGGTAGTACATCGTCGGCGAGACGGTCAC

GCCGAGCTCGTTGGCCCGCTCGGCGATGTCTTCGAGGTCGGCGGCGGTCC

GAGCGATGGGTTTCTCGCTGACGACGTGAACGCCGTTCTCGACGGCGCTC

TCGATGATTTCCGGCGTCTCGTCGCTCCGGTAGGTTATCCACGCCACGTC

GACGTCGGCGTCGGAGACGAGTTCGTGTGGGTCCTCGTAGACCGCCGCGC

CGCCGACGAGGTCCGCCATGTCCTGTCCCTCGGTCGTGATTTCGTCGGGC

CGGTCGTCCATCGCCGCGATGTTCTCCACGTCGACCCGCCGGCCGGGCTC

GCAGACCGCGGTAATCGTCGCGTCCAACTCGCTCGCGACCGCGAAGTAGG

GGTCCCGGTGGTGGTGGTCGATACCGATGTATCCGATATTTGTACCCATA

CACGTAGTTCACCGAAAGTGATTTAAAAATGTTCGGGTATAAGCAAGGTA

ATCGGCGGCGGACGGTGCGGTGGCAGACGGTGCGTCGGCCTCGTGCGGTT

AGCGCTGCGGCGTCTCAACCCGACGAGACCACGACCGGAGCCACGGGCGA

GTCCCGCCGTTGGGGACGTTCAGTTCGTGGCTGTTGACCGGGAGGTCCGG

GTAGCCGCCGACCTGTTCCTGACTGTCGACGAGGTAGCTCTCCCCGCCTT

CGACGTTTTCGAGAATGCGTTCGTCGGTCGCCGTCCGGTCGGCCGGGCGC

GCGCCGACGTTCGCGAGGTTGTGGTCGAACGTCCGGTCCGACGACATCGC

CGCCAGTCCGTCGGGCCACAGCGGCCGTTCATCGACGACAGTCACGTTCT

CGTCGACCATCGCCACGTCGCCGTCGGTGAGGTTGTCTTCGAGGTACGCC

ACGGCGGTGTCCACGTCGTCCTCGGTGAAGACGTTCGCCTTGTCCGAGTT

GGGTCGGAGGTAGGCGTTGCCGACGATGCTCGCCTCGGTGTCGGGGTCAA

GCCAGGTGCCGTCTTCGAAGTCGTACATCACGTTGTTGACGACGACGCTC

TGGGTTCCCTCCTTGAGTCTGGGATGGCGGTCGGTATTGAACGCCCAGAC

GTTCCCCAGCATGGCGACGTTCTTCGCGTCGTTGCCGATGAGCGACCCGT

AGCCGTGTTCGCCCTTCGGGTGGACCGAGTCGTCGAGCGCCTCGGCGACG

AGGCAGTTCGAGACGGTCGTCTCCGCGGTCTCGTAGCCGACCGAGAGGCA

CTCGTCGACGCTCCACGAGGCGGAGACGTGGTCGATGACGTTGTTCGTCG

TCTCGTCGGCCGTGTTGACCGTGTCGAGCGCCCAGTCCTCGGTCGCGTCC

TCGATGCCCGCGTCGCCGAGTCGGACTCGAACGTGCTGGAGGACGCAGTC

GCTCGCCCCGATATTCACGCGACCCTTGACCAGGGTGACGCCCGGCGAGG

GCGCGGTCTGGCCCGCGATGTAGCACTTGTCGTACGGAATCGGGAGGTCG

CGCACGCCGAGGTCGATTGTTCCGCTGGTCTCGAAGACGACCAATCGCTC

GCCGTCGATGGTGACCGCCTTTTCCAGTTGGCGGCGGGTCGGTTCGGTTA

TCACGATGATCGGGGTGTCGTCGTCGAGCCACGGTGCGGCCTCGGCGAAC

CCGTCGCTCGGTCCGAAGTGGGACACGCTGTCCGGGCCGGGGTGGGCCGG

AACCTTGCTGTTCGACGGGTTCGGGGTGTTGAACGTGGAAACCGAACCCC

GAGCGGTGTCGCCGTCGGCCGTGTCCGCCACCGCGCGAACCTCGTAGTAG

CGACGCCGCGTCAGGTCGTCGGCGTCGACGCTGAACGCTCCCGACGAGGT

GAGTTCCGTCGAATCCGTGGTCTTCCACGACTCGGTGGGTACCTCCCGGT

ACTCGAAGTAACACTCTGCGGAGTCGGCACCGCCGAGGTCGCCGAGGTCG

CCGGTGAGCGTGGCGGACCCGGGGCCGATGTCGGTCACGTCGCCGGTCGA

GACGAGCGGAACGCCCGTCGTGTTCTCCACGCTCCCCGCGACTTCGACGT

CGCCGATATCGCGGTTCCGGTCGGGGTCGACCCCCGAGAGACTCTGGAAC

GTCGCCGTCGCGAGCGTCCCCGAGAGGTGGCTGGTGACTGCCAGTCCGAC

GTAGACGCTGTCGCCCAGCGAGATGTCGGCGGCGTCGAGCGTGTTAATCG

ACGTCCACGTCTCGCCGTCCGTGGAGTGGTAGGTCTCTATCGCGTCTCCG

CTTCGCTTGAGACGGAGCCAGTCGGCCGGTGTGCCGCCGACGCTATCCGT

CTCAGCGCCGTCTTCGGGCCGATACTGCATCGACGCCTCGCCGTTGGGCC

GGCGACGGACCATGACGTTCTTCGAGTCGGGGTCGAGCGACTCGCGGACC

ATCGGGCCCGCCTTCGTCCAGCTTTCGACGTTGTCGATGCCGGTGTTCTG

GACGGCGACGTCGAAATCGCCGCTCAGCTCCGTATAGTAGTAGTGAAACG

CGTCGGCGGTGCCCCAGATGTCGTCGCCGCCGCCCTGCATCGAAATCACG

GTCGCAGCCGCGGCGCTGCCACCGAAGAGGCCAGTCGTCGCCGCACCGAT

ACTTCCTGCTCCGAGCACGCGGAGGAACGTCCGTCTGTCGGGTCTCATGA

GTGCATCACTCGAACAGACAACATATTCAACGATTGTTATAATTTATGGC

AGCCTGGACGGAGGCCGACACAGCATATAAAGTCCGAGAGTGTCGAACGA

ATGACGCCGGCGCTGGTTCGAAATGACGAGATGAGACATGAGATATTACA

CCAATATGTTTGTAACACATCGCCTATTCCTTATAAAATTCGGGATATTC

GAATAGAGGCGTTCACTCACACACGTCCTCGATTACGCTCTGTCGGACAT

CGTCGTAATCGACGGTTCGCGGATGCAGGCGACGGTCAGGCGCGCGAATC

GTTCCGGCGGAAACCGGAACCATGCAAAAGGTTATTACACTCCGACCAGC

AGATTGATGCAACGAGTCAGCACATGCAAGAGCTAGGTATCATCATGAAC

GGCGTGACCGGCCGGATGGGAACGAATCAGCATCTCATCCGCTCTATCGT

CGCGCTGCGCGAGGAGGGCGGCGTGGAACTCCCCAGCGGGGAACGAGTCA

TGCCGGACCCGCTTCTCGTCGGCCGCAACGAACGCAAACTCCGCGAACTG

AGCGAGGAACACGGTATCGACCGGTGGACCGTCGACCCCGACCTCGAGAC

GTGTCTCGACGGCGACGACGAGGTGTACTTCGACTCGCAGATAACCCCCC

GCCGCCCGGACAGCGTCATGAAGGCCATCGACGCCGGAAAGCACGTCTAC

TGCGAGAAACCGCTGGCGAGCGACCTCAGCGCCGCGCTCGACGTGGCCGG

GATGGCTGAAGAGAGCGACGTGAAACACGGTATCGTCCAAGACAAACTCT

GGCTCCCGGGGCTGTTGAAGCTCCAGCGACTCATCGAACAGGACTTCTTC

GGCGACATCCTCTCGGTGCGCGTCGAGTTCGGCTACTGGGTGTTCACCGG

CCACGGCCAGGAGGCACAGCGCCCCTCGTGGAACTACCGCGCCGAGGACG

GCGGCGGTATCGTCGACGACATGTTCTCCCATTGGAGCTACGTGCTGGAG

AACCTCTTCGGCGAGGTGGAGTCCGTCCGCTGTCTCCAGAAGACCCACAT

CGACGAGCGAATCGACGAGGACGGCGAGCCCTACGAGGCGACCGCCGACG

ACGCCGCCTACGCCATCATGGAGTTGGAAGACGACATCGTCGCCCAACTC

AACTCCTCGTGGACCGTCCGCGTCAACCGCGACGACCTCCTCGAAATCCA

GGTCGACGGGACCGAGGGAAGCGCGGTCGCCGGCCTGCGCGACTGCAAGA

CGCAGGGCCACGCGAACACGCCGAAACCGGAGTGGAACCCCGACACGCCG

AAGGAACACGACTTCTACGAGGACTGGACGGGCGTCCCGAACAACCGGGT

GTTCGAGAACGCGTTCAAGCTCCAGTGGGAGAAGTTCGTCCGCCACGTCG

TCGCCGACGAGCCGTTCCCGTGGGACTTCACCGCCGGGGCGCGTGGCGTC

CAACTCACCGAGGCGAGCTATCGGTCCTCCGAGGAGGGTCGCCGCGTCGT

CCTCGACGACCTCAGCGTGTAGAAAGGCCCCCCGACGAACAGTTTTATGT

CGAACTCGGCCGTCGAGTTCCGCATGCACACAGATGGCGACGAGAACCCC

GTAGACTGGCTCTCAGTCCCGGAAGACGTTATCCTCGAAGCGTGCGGTAA

CCCCTCGCTCCCCGAGTTAGGTATCATCGAACAAGTCTGGGAGACGAATC

CCATTCCGGCGGCCGACGTTCCGGAGGCGGCCGCGAAGGCCGTCGCCGCG

CTGTCGTTCGCGGCTGTTCCCGAGGGTGGCGAGGTCGCGCTCGGCGTCGG

GAGCCGCGGTATCGCGAACATACCCGACATCGTCGCGGGCGTCGTCGACG

CCGTCTCCGAGGCGGGCTACGAGCCGTTCGTCTTCCCCGCGATGGGGAGC

CACGGCGGTGCGACCGGCGACGGACAGCGGGAGATGCTGAACGAACTCGG

CATCACCGAGGAGCGAATCGGCTGCGAGATTCGGTCCAGTATGGAGGTCG

TGGAGGTCGGGCGCACGCCGGACCGCGACGTGCCCGTCGTCGCCGACGCC

AACGCCGCGGGCGCGGACGCCATCATCCCCATCAACCGCGTCAAGCCCCA

CACAGACTTCGACGGCGAGGTCGAAAGCGGGCTGTCGAAGATGCTCGTCA

TCGGCATGGGCAAACAGCGCGGCGCGCAAATCGCCCACAAGTGGGCCGTC

GACTGGTCGTTCCGACGGATGATTCCCGAGATAACCGAGCAACTGCTGGA

CTCGCTTCCCATCGTGGGCGGCGTCGCCATCGTGGAAGACCAGCACGACG

ACACCACGCTCATCGAGGGCGTCCCCCCGTCGGGCTTCCTCGACCGCGAG

CGCGAACTGCTCGAAACCGCGTACGAACTGATGCCGAAGCTCCCGTTCGA

GGAGCTCGATTTGGTCGTGTTCGACCGACAGGGCAAGGAGATTAGCGGGC

AGGGGATGGACACGAACGTCATCGGCCGCCGGCCGTTTTCCATCAACGAG

CCCGCGCCCGAGAAGCCGAACATCAAGCGCATCTACACCCACGGGCTGAC

CGAGAAGACCCACGGCAACGCGATGGGCGTCGGGTCGGCCGACGTGATTC

ACGAGGACATCGTCGCCGAGTTGGACGCGCAGACGACGCTCATCAACGCG

CTCACCGCGAGCACCATCCGCGGCGTGAAACTCCCGCCCGTCGTCGAGAC

CGACCGCGCGGGCGTGGTCGCCGCGCTGTCGACAATCGGCGTCGTCGAGC

CCGACACGGTCCGGGTCGTCCGCGCGGCCGACACCATGCATCTCCACCGG

CTGTACGCCTCGCCGGCGCTCGTCGAGGAGGCCCGCGAGCGAGACGACCT

GCGCGTGGTCGAAGATCCCACACCGATAGCGTTCGAGGACGGCCAGTTCG

CCGCGCCGTCGCTCCGGGACTGAAGAACGCGAGCGCGTCTTTTTCGGAAA

AACGAGTGCGTCAGGCGTCGCCTACCGCTCGACCGTTTCTGGCTGCACCG

AGACGACGGCCTCGTCCATGTCGCCGTAGTTGCCGGCGAGGAGTACCGAG

GACTCGCCGTCGCTCTCGCCGCGGACGCGGGCGAACGGCTCGCCGTCGTC

GGGGGCGACGCAGCCGCCGATTCGGAGGTTCTCGACGCCGTCTGCCTCGA

CTGCCGGCCCCTCATCCGCCTCGGCCTCGAAACCGTCGAGGACGACGGTC

GAACTCGCTTCCGCGGTCAGCGGGGTCCCGCTCCCGTCGGGGACCGTCAC

GCGAACGTCTTTGAACGAGACGCGACCGAGCGACTTACAGAAGACGCCGT

GTCGCTGGTCGTACCCCTTCGCCATCGCCGGCGAGAGGTCCGAGGCGTCG

AACGGGCGGGTCGCGTCGATGTCCACGTCGGTAAAGGAGATTCCCTCGAA

CCGCTGTTCGGGGAGGCCGGCGAGGAACGCCGCCGACTCGACCTCCTCGG

CGGTGATGTGGTGGAAGTCGACGTTTCGGACGTTCGGCGTCGCCTCGGTG

ACGGGCTTGGGGTCGCTGTCGATGTCCGTCTGGTAGTAGCCGTTGATGAC

GAACGGGCAGGCGACGCGGCGCATGATGATGGTGTCGAACCGGAGGTCCT

CGACCGTCCCGCCGCGGCCGCGCTTGGACTTGATGCGGATGCCGCGGTCG

GTGTCGGTGAAGGTGCAGTTCGTGACGGTGACGTGCCGCACGTCGCCGGC

AGTCTCGCTCCCGATGACGACGCCGCCGTGGCCGTGTTCGACCGTACAGT

TGGTTACGACGACGTTCTCGGTCGGGCGTCCGACCTCGCGGCCCTGCTCG

TCCTTGCCGGACTTCAGGCAGATGGCGTCGTCGCCGGCGTCGATGTGGGT

GTCGCTGACCCTGACAAAGCGCGAGGAGTCGATGTCGATGCCGTCGCCGT

TCGGGGCGTCCGGCGGGTTCTGAATCGAGACGTCGTGAATCGTCACGTCG

TCGGAGTAGACCACGTGGGTGTTCCAGAACGGGGAATTCCGGAGCGTCAC

GCCGGAGACTGTCACGTTCTCACAGCCGTCTATCTGGAGCAGCGGCGGGC

GAACCGTGAACGTGCTCACCTCGTCCTGCTGGTTGCCGCTTCGAATCTCC

TCGAGTCGCGCGGCGAGTTCGGAGGGGTACTGCTCGGGCGGTAACGACAC

GAACACCCACCAGTAGGAGCCGCCGCCGTCGATGACGCCCTCGCCGGTGA

TGGTGACGTTCGAGGCGTCGGCCACGTGGAGACACGGGTGGAAGCCGTCT

TGGTCCCACCCCTCCCAGCGGCTCTCGACCGTCGGGAACTCGGTGAAGTC

CTGGACGAACCGGAGTTCCGCCCCGTTTGCCAGTCTGAAGGTCGTGTCGT

CGCCGACGCGGAGCGGGGCGCTGCGGTACGTCCCGGGCGGGAGATACACC

TCGCCGCCCTCGCCGGCGCAGTCGTCGAGCGCCGCCTGAATCGCCGCCGT

GTCGAGCGAATCGTCGTCTTCGATACCGTAGTCGCGCACGTTCCGCTGTT

CGAGAGTCACGCCTGAAGCATCCGCGAGCGCGTTCATAAACGTTGTGCCG

AGGTACCACGACGCACCGAAATCGGCGACGAGAGGCGAGACGAGAGACGG

AAGCGGGGGCGGAGAGAGCACGCCGACTCAGAGTCGGTCGCGCAGGAAGG

GCCACGCGCCGGCCTCGAAGAAGCGGTGGCCGCCGTCGCCGACGAAGAGG

CTGCACTCTCTCTCGGCGTCCGCAGCGCGGTACCGACCCTGGATGCGGTC

GAACGCCCGTCTCGTCCCGGCTATCGGGAAGATGGGGTCGGATTCGCCCG

CGACGATGCGGAGCGGTCGCGGGGCGACGAGGCCGGCGATGTCCCACGCC

TCGCCGAGGCGTCGGAGGCCGGGAACGTAGTTGCACAGGCAGTGGTCGAT

GGGGACGATGGAGTCCTCGAACGGACAGACCGACGCGCAGGCGACGACGT

GCCCGATTCGGTCGTCGAGCGCGGAGTGCCCCTCGACGAAGTCGACCAGA

CGACGCACGTCCCACACGCGCTCGCCGACGAGCGACCGGCCGAGGAGTTG

GGCGCGCTTCTGCCAGCGCGTACACGCGCTGACGGCGTCGGACGAGTCCG

AGTCGTCGGATGTGTCGGGTGACAGCTCGCCGAAGGCGCGCATGTCGGGA

GCGACGACGGCGAAGCCCCGCCGGGCCGCCTGTCGGGCCATGTCTCGTCG

GTCGTCGGCGATGTGACGGCCGGCCGCCTCGCCCTCGGCGACGCCGGCGG

CGAGGTCTTTGCCGTGTTCGGTGTGGCCGTGAAGCGCGACGGCGACGGGG

TACGGCGGGTCCACCGAGTCGGGGAGAAGCAGGTAGAACGGGACGCGGAA

CCCCCGTTCGGTGCGGACGCTCCACGTCCGTCGCTCGTAACTCCGGTGTC

GAACCGTCGCTTCCGGATGCTCCCGTCGCCTCGGGTCGATGTCCTCGACG

GCGCTGTCGCGAACCGCCGGAAATCCGAGCACGTCGCGCAGTTCGGCGCG

GAACGACTCCTGCCACGCCTCGAATTCCTGTCCTGTCTCACCCGCGTACG

CGGCCGAGCCGTCGGTCGCTCGGAGGACGTCGTCGAACCAGTCGTCGTAG

TCGAAGCCAGTCATTCGGTGTCGTTCCGACGAACGCAACGGGTTCACAAG

TGCGTGTCGACGTCGCGGATCGGAGGGTTGTTCCCCGTAGACGAACAAAC

CTAAATGCGAATGAAATCGGGCGTAAAACCCATCACTCGTCGGTTTTCGG

TCGTTTTCGATGGAGAACGAACCGACCCGAAATCGGGAGAATGAGCATTA

CGGTTGTAGCGTCGTCACGGCGCGTGGCGAACCGAGTCAAGCCCTATTTC

CGCTAGTAGAGGCCACAAATGCCACGATTCTGATGTTCGCGGCGGTGACT

GGGACGCCACTCAGCGAGGACGCCCAACCGAATTCGGATGTTGAGTTCCC

CATACGCGAACAAAAACTAACATATCAATTATATATTAGGAACGAAAATC

GGAAGACAGCGGCGAGATAGCGATGAAAGTGATACTATGATATGAAGAAC

AGCGATGAGCCGAGTCGGGAGAGACGACGGGAGAAAGCAGAAAGCCGAAA

GCGTTCAGTCGAGGGCGTCACTCAGGTAGCCGCACCGCCAGAAGACGACG

CCGACGGTCGCGAGGAGCACGCCGGCGAGGGCGAAGTCCATCATGAGGAC

GACGTACGACGCGGTCCCGCGTTCGATGCCCGTCATCGCGAGGAGGCCGA

GGACGACGATGAGCAACGTAAACGAGAGCACGCTCGTCCACAGCATCTTC

TTTCGGCAAATCCAACATTTGAAGTACGACCGTTCTGAGGTTCCCATTAC

CGGCGATTTCGCAAAACAAGATAATAGTCCTTTGGGGGACCGCCCCGCGC

GTCGCGAGAGTCACGGGCGCGACCGCGTAATCAAATGATGTCAGTAGACA

ATTACCATTATCGATGGGTATATACGGGATAGTCATAATGGGTACTGTGT

GATGGATTCGCAAAGCAGCGCTGAGTCCGACAAGTGGATCAGCGACGACG

AGTGGGAAGACGTATCGCCACAGACGCGCCGTCGTCTGCTTCGAAACGCG

TTCGGTGCCACGGCCGGCGCCGCTGCGCTGGCTGGCTGTAGTCAGGGCGG

GAACGAGGATACGCCGACGCCGCTCGAGAACGAAGGCGGTGGCGGCGAAA

GCACGACGACTGACGGCGATTCGACCAGCCAGTCGTCGGGTCGAACGCTC

GACCACATCATCTCGCTCGCGCCGACGAACGCCCGGTTCAATCCGTACGG

CAACGCCGCGAACTTCTCGTTCCGGTGGTACTGGGCGATGTTCGACCAAC

TGGCCGTCCACGACAAGGTGTCGAACAAGACGAAAGGGATGGTCGTCGAG

GACTGGGAGTACGCCGACAACGGACAGGTCGCGTGGAACATCCGCGACAC

CTACACGTGGCACAACGGCGACGACCTGACCGCGGAGGACGTGGCGACCC

AGCTCAAAATCGGCAAGCTGATGCAGACCGTCCACAAGGGCTACGGCGCG

CAACCGCTGTACGAGAACGTGGAGACGACCGGGAAGTACGAACTCACGTT

CGACCTGGTCGAGCCCGACATCAGCCGGGAGATATTCGAGTTCGGTCACA

TGAAACGCCGCGCGTGGCTCTGGGCGCACCGAGACATCTGGGGGAAGTAC

GCCGAGATGTTCGACGACGCCACCACCGAATCGGAACGGACCTCCGCCCA

GCAGGAGATGATGCAAGCGGTTCAACAGAACGTCTGGGACAACGCGAACG

TCCCGGGTAACTCCGTCTGGGAGTTCGTCAGCGGAGAGGAGAACGTCGCG

CACTTCGAACCCTACGACGACTACGTCAGCCCCTTCAGCGACGCCGAGTG

GTCGAACGGCGAGATTACGGGCGACATGATAGACTACAGCCTCGACTGGC

ACCGCTACCCGAACCAGCAACAGCGGACCAAGGCGATGCAGGAGGGCGTC

ATCGACGTGAGCTACCCGCCGGAGTCGCAGTCCGCTCGTGACCGGCTCAA

GGAGAACGGGTGGGGGCCGGCCGACAGCCTCTCCGAGGACCAGATTACGC

CGCAGATGCGCAGCGGGGCGATGGGCGTCCTGCTCAACTGTCAGAGCGAC

ATCACCGGCGACCCGCGCGTCCGGAAGGCGATTCACCACATCGTCCCCCG

GAAGCCGCTCGTCGAGTGGGTTCCGGACTACGGCACCTACTGGGTCGAAG

ACCGCATTCCGTCCGGCATCGGGCAGGACAAGGAAGTGCCATGGTTCGGC

GGGAACTCGAACTGGCCGAGCGGCGACCTCGCGAAGCTCGAGCGGTACGC

CCACACGGAAGACGACGTGGACGAACAGCGGGCGACCGAACTCCTCGAGG

CCGCCGGCTTCTCGAAGTCGGGCAACCGCTGGAAGGACCCGAACGGGGAG

AACGTCACCCTTCGGTTCTACACGGCGACGTCCGACGAGGAGCCGATGGC

CCTACGGTTCGCACAGATCGCGAAGTCCTACCTGGACGACTTCGGTCTCC

AGACCGAAGTGACCGCACAGGAAGCGACCATCCGCGCCGGCAAGACCATC

GAGTCCGGCGACTGGGAGTTACTGTTCGACAACTGGGGCGGTGCGAAGTC

CGGCCCGCCGTTCCTCGGCTTCTCGAACTCCTTCAGAGTCCAGGAGTCGC

TGTCCGGACAGCCGCTTCCGAGCAACGACAGCTGGGCGATGGACCGCGTC

GTCGAGGTTCCGTACCCCATCGGGGACCCGGCCGGTGACCTCCAGGAAGT

CGACGTCATCGACAAGCTCAACGAGCTCCGGACGCAGATGTCGGACGAAG

AGCGCAGACAGAAGGTCGCGGAAGTCTCGTGGATATTCAACCAGTCGCTG

CCGATGATGCTCATCAACGAGGAGGGCGGCACGGGCGGCTACTGGCTGAA

TCACGACAACTGGGCGACGAAGCCCCCGAACGACAACCTGAACCCGGCGT

ACCGCTACGAGATCGTCGAGATCGGTCAGTACTCCTACTGCCAGTACATG

GCCAAGATGGGAACCGAATACTTCCACCCGGCGGAGGGAGGCCAGTAACC

GCGCCGAGCGCCGCCACCTGAACGAGAAACCGTCGTCTCACCGAGTCAGT

CAGAACGTATCAGCCATACGGTGAACTCCCCGTCCGGAGGCGCAACGCGC

CGCCTCCGGACGCGACGACGGCGAGGTCGGTTCGGTCGAGTGCGGACGAA

AGTAGCCCTATTCATAATGATTAAGTACCCACATGAGAGGTAAGAACTAG

CAAGGATGGTCGAGAAAAATTACTACGTGCGCCGGATAGCGCAGGCCCTG

ATAACGGTCTGGGCGGCGATCACGTTCTCGTTCGTGACGGTTCGGCTCAT

TCCCGGCGGGCCGATGGACTACATCGCCGCGCAGATCAGACAGCGGTCCG

GAGGGGCGGTTTCGTCGACCCGCCTCAACCAGATGGTCGAGATGTACTCG

AACATCAACCCGGACAAGCCGATGCTGCAGGCGTACGTCGAATACATCGT

ATCGGTCGCGCAACTGGACTTCGGACGGTCTATCTTCTACGCGGACCCGG

TCTGGAACGTGCTCGGACCGGCAATCCCGTGGACGATGTTCGTCTCGCTT

TTCGCCCTCGTCATCGGCACCATCATGGTGTTTCTCATCGGCGGCTTCCT

CGCCGTCCACGAGGGCGACACCTGGGACGTGGTCGGGAGTTACTTCGTCA

TCTTCTCCGACGCCGTCCCGTACTACATCGTCGGGCTCGTACTGCTTTTC

ACGCTCGGATTCGGTACCGAGTTCTTCCCCAACGGGGGGCGGTACGACAA

CGCGCTCACGCCCGGGTTCAACATCGAGTTCATGGCCAGCGTGCTGCGAC

ACGCGGCGCTGCCCATCATGTCGTTCGCGCTGACCTCGTGGGGCGGCCTC

GAGTTCCGCGCGCACTGTACCCGCATCATCGGTGAGGACTACGTCGACGT

GGCGGAACTCAGGGGACTGCCCGACAGCCGGATTCAACTGCGGTACATCG

GTTGGAACTCGATGCTGCCGATGTACACCGGCCTCATGGCCGGCCTCGCC

AGCCTGTTCGGCGGGTCGGTCATTATGGAGCAGATATTCCGCTACCGCGG

CATGGGCTTCTACTTCTACGAAGCCACGATGGCGCGGGACTACACGACGG

TCATGGCGGCAGTCATCCTGTTTACCATCGTCACCGTCATCGGCCTGCTC

ATCGCCGACTTCACCTACGGCTTCATCGACCCCCGCGCCGGGCACACCCA

GCGCGAGTCCTACTCGCGGTCGTACCGCGAAACGCTCCAGCGGTTCGTCA

ACCGCCTGCGGGGCGGCGGCGCAGACGAGACCGAAACCATCGACGACCGC

ATCGAAGGGAACCGCATCTCGGCCAGCGGCGCGATGAGCTTCGACGTCGA

TAGCTCCGACTTCTCGCGCGGAGAGTACATCAGAGAGCGAATCGACCAGA

GTCTCCTGACCCCGTTCCGCATCATCTGGACCTCGCCGCGGGCGCGGGTC

GGCGCGATAATCCTCGGTATCTTCGGCTTCCTCGGGCTGTTCGGGCAGTT

CCTCGTCCCCGAGGTCAGCCCGATGCAGGGGCCGATTCTCCTGCAACCGC

TGGAGAACGTGCGCTACCCGCTCGGCACCGACGACATCGGGCGCGACCTC

CTCGCACAGCAGGTGCTCGCGACCCGCCCGATGCTCATCATGATGCTGTC

GGGCGCGGCGGTGACGCTCAGCCTGGCCATCCTTCTCGGCGTCACGGCCG

GCTACCTCGGCGGCGTCATCGACAAGGTGCTCATGACGGTGACGGACGTG

ATGATGAACATCCCCGGCCTGGCGCTGGTCATCGTCCTCGCCGTGATTCT

CGAACCGAAGAGCCCGGTGTTCGTCGGGGTCGTGCTGGCCATCGACAACT

GGCCACGGTTGGCCCGACAGCTCCGCTCGCAGGTGCTCACTATCCGCAGT

GAGTCCCACGTCGAGGCCTCGCGGGTGCTCGGACTGCCAACATCGAGCAT

CCTCTCGAAGAACGTCATGTCCCGGCTCATGCCGATGCTGACGATGGGGA

CTGTCGGCTCCATGCGGAACATCCTGATGGAGTCCGTCGGCCTGTACTTC

ATCGGCGTGCTGCCGTTCACGACGAGCAACTGGGGCGTCGTCCTCAACAT

CGCCCGCAACGGCGGCGCGCTGTCCGGCATGGACCGCTTCCACTGGCTGC

TCGCGCCGACACTGTTCATCGTCACGATGACGTACGGCCTCCAGCTGTTC

GCGCAGGGGACGGACCGACTGTTCAACCCCCGCCTCCGCGCGCGGCACGC

GGCCACCGTCTCGACCGACGACATGGAAGGCGGTCGCGGCGGCTCGGAAG

GCGGCGACGGTGCCGCATCGGCCGCGGACTAACCAACCGGACCAGCCGGT

CCGTTTCGCGTCGCTGCTTTTTTCGATTATCTCGACCCGAGAACGGTCGT

GCCACACTGTCTCAGGGAAAAGGTTAACACACGCGCTATCGACCTACGAA

ACGGACTTCAACCATGAAAGTGGAGAAAGAAGGAAATACGACGCGGTACC

TGCTCGGCCTCGTGATACTCGTAATCCTGCTCATCGGGGTGTACTACGGA

TTCACCATGATGTGATCAGAACAGTCGGTACGCCCAGAGCAGTACCCGGA

ACGCGACGGCGACGAACACCAGCAATCCACCGATTATTCCGAACATTCCC

GCCCAGACGCCGGTCTGAAGGGCGGCACCGAAGAGAATCAACAGCGCGCC

GAGGAAAACCACCGTCGAGGTGACACCGCCGATTTTGGAGCGAACTGTCC

ACAGCACGTTCTGTCGCGTTTGCGCTTGCGCCATGTCAGCACGGACATCG

CGGACGAACTTATAGTTTGCCGGTATCGGCAGCCGAACCGCCCGAAGACT

CCGATATCGACGCGCATGGACTGCGCTGTCGGAAAGTATTAAGTAGTATT

GAACGTGATAGATTCCTACTACGGTCACATGTTGACGATAGGCACGCGTA
[truncated: 55,651 more chars]
