# Supplementary material for: Mutations Affecting HVO_1357 or HVO_2248 Cause Hypermotility in Haloferax volcanii, Suggesting Roles in Motility Regulation
Source: Genes (Basel). 2020 Dec 31;12(1):58. doi: 10.3390/genes12010058 (PMC7824242; doi:10.3390/genes12010058)
Supplement: Supplementary file 1 [file genes-12-00058-s001.zip › genes-12-00058-s001/genes-1028798-supplementary/Collins et al. 2020 Supplementary Figures 2/Table S1.docx]

**Table S1. Primary and secondary mutations in hypermotile *Hfx. volcanii* Tn-isolates**

| **Name^1^** | **Tn-**  **insertion^2^** | **Annotation** | **Tn-insertion position /**  **orientation^3^** | **Position in DS2^T^ strain^4^** | **Details/Comments^5^** |
| --- | --- | --- | --- | --- | --- |
| **MC3** | HVO_2248 | Hypothetical | 2,734,510;– | 2,105,440 | Tn insertion is 230 nt from start of CDS **Also C>G (Pro>Ala) within HVO_1357, nt 1,867,338; 141 nt from end of CDS** |
| **MC50** | HVO_2248 | Hypothetical | 2,734,510;– | 2,105,440 | Tn insertion is 230 nt from start of CDS |
| **SAH4** | HVO_2248 | Hypothetical | 2,734,510;– | 2,105,440 | Tn insertion is 230 nt from start of CDS.  **Also** 559 bp deletion of CRISPR3 spacers 12-20, nt 3,014,879-3,015,437 |
| **MC14** | HVO_2248 | Hypothetical | 2,734,434;+ | 2,105,364 | Tn insertion is 306 nt from start of CDS  **Also G>A (Asp>Asn) within HVO_1357, nt 1,866,735; 1006 nt from start of CDS** |
| **SAH5, MC9, 15, 16, 17, 26** | HVO_2248 | Hypothetical | 2,734,434;+ | 2,105,364 | Tn insertion is 306 nt from start of CDS |
| **MC27** | HVO_2248 | Hypothetical | 2,734,434;+ | 2,105,364 | Tn insertion is 306 nt from start of CDS  **Also** G>T (Ala>Glu) within HVO_1308 (*aroA*), nt 1,823,227; 497 nt from end of CDS |
| **MC49** | HVO_2248 | Hypothetical | 2,734,434;+ | 2,105,364 | Tn insertion is 306 nt from start of CDS  **Also** G>T (Ala>Glu) within HVO_1308 (*aroA*), nt 1,823,227; 497 nt from end of CDS  **Also** 12,527 bp deletion of provirus3, nt 1,924,029-1,936,555 |
| **MC1** | Near  HVO_2248 | Hypothetical | 2,734,755;+ | 2,105,685 | Tn insertion is 16 nt upstream of HVO_2248  **Also** 29,821 bp deletion from HVO_2014 to HVO_2042, nt 2489096-2518916 **Also 2 bp insertion (CG) within HVO_1357, 459 nt from start of CDS (after nt 1,866,188) that causes termination of CDS after 3 codons, truncating the protein** |
| **MC28, 31** | Near  HVO_2248 | Hypothetical | 2,734,755;+ | 2,105,685 | Tn insertion is 16 nt upstream of HVO_2248  **Also 99 bp deletion within HVO_1357, nt 1,866,844-1,866,942; 635 nt from end of CDS, deletes codons 372-404**  **Also** T>C (Lys>Glu) within HVO_2380 (cdc48a), nt 2,875,094 1216 nt from start of CDS |
| **MC8, 23, 30, 35** | Near  HVO_2248 | Hypothetical | 2,734,755;+ | 2,105,685 | Tn insertion is 16 nt upstream of HVO_2248 |
| **MC4** | Near  HVO_2248 | Hypothetical | 2,734,755;+ | 2,105,685 | Tn insertion is 16 nt upstream of HVO_2248  **Also** C>T (Gly>Ser) within HVO_0163 (Tx regulator), nt 149,285; 94 nt from start of CDS |
| **MC6** | Near  HVO_2248 | Hypothetical | 2,734,755;+ | 2,105,685 | Tn insertion is 16 nt upstream of HVO_2248  **Also** T>C, intergenic, nt 3,243,220; 59 nt upstream of HVO_2772 (rhodanese/metallo-hydrolase domain protein) |
| **MC7** | Near  HVO_2248 | Hypothetical | 2,734,755;+ | 2,105,685 | Tn insertion is 16 nt upstream of HVO_2248  **Also** G>T (Pro>Thr) within HVO_A0269 (gpdA2), nt 260,247 (on integrated pHV4), 513 nt from end of CDS |
| **MC36, 45** | HVO_0576 | HxlR family transcription regulator | 1,147,136;– | 513,479 | Tn insertion is 82 nt from end of CDS **Also 12,345 bp deletion of HVO_2234 to HVO_2251 (including HVO_2248), nt 2,726,437-2,738,781**  **Also** 109,198 bp deletion of HVO_A0128 to HVO_A0017 (on integrated pHV4), nt 406,701-515,898 |
| **MC34** | HVO_1926 | DUF541 protein | 2,404,062;– | 1,774,992 | Tn insertion is 390 nt from start of CDS  **Also 12,345 bp deletion of HVO_2234 to HVO_2251 (including HVO_2248), nt 2,726,437-2,738,781**  **Also** 12,527 bp deletion of provirus3, HVO_1422 to HVO_1434, nt 1,924,029-1,936,555 |
| **MC54** | HVO_2229 | amine oxidase family oxidoreductase | 2,723,422;– | 2,094,352 | Tn insertion is 64 nt from start of CDS  **Also 12,345 bp deletion of HVO_2234 to HVO_2251 (including HVO_2248), nt 2,726,437-2,738,781** |
| **MC44** | HVO_2377 | pstA1, P-permease | 2,871,511;+ | 2,242,441 | Tn insertion is 706 nt from end of CDS  **Also 12,345 bp deletion of HVO_2234 to HVO_2251 (including HVO_2248), nt 2,726,437-2,738,781**  **Also** T>C, intergenic, nt 265,337 (on pHV3), 100 bp from end of HVO_B0225 |
| **MC52** | HVO_A0546 | short-chain family oxidoreductase | 621,497;+ | 548,278 (pHV4) | Tn insertion is 105 nt from start of CDS  **Also 31,614 bp deletion of HVO_2234 to HVO_2269 (including HVO_2248), nt 2,726,437-2,758,050**  **Also** 107,469 bp deletion of HVO_A0127 to HVO_A0017 (on integrated pHV4), nt 408,026-515,494  **Also** C>A (Pro>Thr) within HVO_2923 (psmA2), nt 3,385,434, 109 nt from start of CDS |
| **MC11** | HVO_1357 | HTH-10 family transcription regulator | 1,867,146;+ | 1,238,076 | Tn insertion is 333 nt from end of CDS **Also C>A (Ser>Phe) within HVO_2248, nt 2,734,129; 167 nt from end of CDS**  **Also** C>T (Asp>Asn) within HVO_0258, nt 231,609; 72 nt from end of CDS  **Also** G>A (Gly>Asp) within HVO_2377, nt 2,870,763; 176 nt from start of CDS  **Also** T>C, intergenic, nt 265,337 (on pHV3), 100 bp from end of HVO_B0225 |
| **MC19** | HVO_1357 | HTH-10 family transcription regulator | 1,867,146;+ | 1,238,076 | Tn insertion is 333 nt from end of CDS |
| **MC24, 37** | HVO_1357 | HTH-10 family transcription regulator | 1,867,146;+ | 1,238,076 | Tn insertion is 333 nt from end of CDS  **Also** T>C, intergenic, nt 265,337 (on pHV3), 100 bp from end of HVO_B0225 |
| **MC5, 10, 22, 29, 40, 41, 48** | HVO_1357 | HTH-10 family transcription regulator | 1,867,189;+ | 1,238,119 | Tn insertion is 290 nt from end of CDS |
| **MC2** | HVO_1357 | HTH-10 family transcription regulator | 1,867,189;+ | 1,238,119 | Tn insertion is 290 nt from end of CDS **Also** C>T (Arg>His) within HVO_2380 (cdc48a), nt 2,874,457; 380 nt from end of CDS  **Also** 164 bp deletion of CRISPR3 spacers 18-20, nt 3,015,274-3,015,437  **Also** 123,755 bp deletion of HVO_B0234A to HVO_B0348, nt 278,878-402,632 (on pHV3) |
| **MC12** | HVO_1357 | HTH-10 family transcription regulator | 1,867,189;+ | 1,238,119 | Tn insertion is 290 nt from end of CDS  **Also** C>T, intergenic, nt 2,741,558; 215 nt from end of HVO_2253 |
| **MC20** | HVO_1357 | HTH-10 family transcription regulator | 1,867,189;+ | 1,238,119 | Tn insertion is 290 nt from end of CDS  **Also** 9,197 bp deletion of HVO_A0124 to HVO_A0111 (on integrated pHV4), nt 412,541- 421,737 |
| **MC21, 25** | HVO_1357 | HTH-10 family transcription regulator | 1,867,189;+ | 1,238,119 | Tn insertion is 290 nt from end of CDS  **Also** 168 bp deletion of CRISPR3 spacers 15-17, nt 3,015,076-3,015,243 |
| **MC33** | HVO_1357 | HTH-10 family transcription regulator | 1,867,189;+ | 1,238,119 | Tn insertion is 290 nt from end of CDS  **Also** three closely spaced base changes in HVO_A0436 (A>G (silent), nt 724,581; C>G (silent), nt 724,584; G>A (Ala>Thr), nt 724,594) (on integrated pHV4); this makes HVO_A0436 identical to its close paralog HVO_A0011 in the covered region; no reads representing the original sequence of HVO_A0436 were obtained for this region, so that a gene conversion event is likely in this strain |
| **MC42, 46** | HVO_1357 | HTH-10 family transcription regulator | 1,865,880;– | 1,236,810 | Tn insertion is 151 nt from start of CDS |
| **SAH1** | Near  HVO_2176 | Hypothetical | 2,678,278;– | 2,049,208 | Tn insertion is 44 nt upstream of HVO_2176 |
| **SAH2** | HVO_1726 | HTH domain protein | 2,225,641;+ | 1,596,571 | Tn insertion is 299 nt from start of CDS |
| **SAH3** | HVO_0430 | UPF0146 protein | 1,020,227;– | 386,570 | Tn insertion is 52 nt from end of CDS  **Also** 101,420 bp deletion of HVO_A0119 to HVO_A0015 (on integrated pHV4), nt 416032**-**517451  Tn insertion is 52 nt from end of CDS  **Also** 101,420 bp deletion of HVO_A0119 to HVO_A0015 (on integrated pHV4), nt 416,032-517,451  **Also** 131,671 bp deletion of HVO_A0412 to HVO_A0279A (on integrated pHV4), nt 750,510-882,180 |
| **MC47** | HVO_2649 | Hypothetical | 3,126,642;– | 2,497,572 | Tn insertion is 63 nt from start of CDS  **Also** 101,420 bp deletion of HVO_A0119 to HVO_A0015 (on integrated pHV4), nt 416,032-517,451  **Also** 131,670 bp deletion of HVO_A0412 to HVO_A0279A (on integrated pHV4), nt 750,510-882,180  **Also** 5 bp insertion (TCTCC), intergenic, after nt 44,716 of pHV3 (extra repeat motif in a series)  **Also** G>A (silent) within HVO_B0203, nt 235,949 (on pHV3), 1083 nt from start of CDS |

^1^Names starting with SAH indicate isolates from the 1st screen (stabbing individual colonies on hypermotility plates). Names starting with MC indicate isolates from the 2nd screen (central streak of the amplified library, picking strains that had moved farthest). Rows series indicate isolates with a common characteristic, blue for isolates associated with HVO_2248, green for isolates associated with HVO_1357. All shades of green and the three most intense shades of blue indicate an identical Tn-insertion site. The rows colored the lightest blue have distinct primary Tn-insertion sites but all have deleted a region that traverses HVO_2248. Isolates with identical Tn-insertion sites and identical secondary genome alterations are grouped in a single row and only the serial number of additional MC isolates is given.

^2^Tn-insertion names the gene that is affected by transposon insertion. This may occur with the CDS or close to and upstream of the gene (indicated by “Near”).

^3^Tn-insertion position refers to the first change compared to the chromosomal sequence of strain H295mod1 (see Methods), which includes plasmid pHV4 due to its chromosomal integration that occurred in the parent strain H26 ^[^[^47^](https://www.zotero.org/google-docs/?ChKHCP)^]^. The construct integrated by the transposase carries a *trpA* marker, and the orientation is denoted according to this.

^4^Corresponding position in the type strain (DS2^T^) genome (GenBank accessions CP001956 and CP001955 for chromosome and pHV4, respectively).

^5^For all isolates, the relative position of the transposon insertion compared to the start or end of the primary target is reported. In lines starting with “Also,” secondary genome alterations are specified.
